# A genome-wide SNP scan accelerates trait-regulatory genomic loci identification in chickpea

**DOI:** 10.1038/srep11166

**Published:** 2015-06-10

**Authors:** Alice Kujur, Deepak Bajaj, Hari D. Upadhyaya, Shouvik Das, Rajeev Ranjan, Tanima Shree, Maneesha S. Saxena, Saurabh Badoni, Vinod Kumar, Shailesh Tripathi, C.L.L. Gowda, Shivali Sharma, Sube Singh, Akhilesh K. Tyagi, Swarup K. Parida

**Affiliations:** 1 National Institute of Plant Genome Research (NIPGR), Aruna Asaf Ali Marg, New Delhi 110067, India; 2 International Crops Research Institute for the Semi-Arid Tropics (ICRISAT), Patancheru 502324, Andhra Pradesh, India; 3 National Research Centre on Plant Biotechnology (NRCPB), New Delhi 110012, India; 4 Division of Genetics, Indian Agricultural Research Institute (IARI), New Delhi 110012, India

## Abstract

We identified 44844 high-quality SNPs by sequencing 92 diverse chickpea accessions belonging to a seed and pod trait-specific association panel using reference genome- and *de novo*-based GBS (genotyping-by-sequencing) assays. A GWAS (genome-wide association study) in an association panel of 211, including the 92 sequenced accessions, identified 22 major genomic loci showing significant association (explaining 23–47% phenotypic variation) with pod and seed number/plant and 100-seed weight. Eighteen trait-regulatory major genomic loci underlying 13 robust QTLs were validated and mapped on an intra-specific genetic linkage map by QTL mapping. A combinatorial approach of GWAS, QTL mapping and gene haplotype-specific LD mapping and transcript profiling uncovered one superior haplotype and favourable natural allelic variants in the upstream regulatory region of a *CesA*-type cellulose synthase (*Ca_Kabuli_CesA3*) gene regulating high pod and seed number/plant (explaining 47% phenotypic variation) in chickpea. The up-regulation of this superior gene haplotype correlated with increased transcript expression of *Ca_Kabuli_CesA3* gene in the pollen and pod of high pod/seed number accession, resulting in higher cellulose accumulation for normal pollen and pollen tube growth. A rapid combinatorial genome-wide SNP genotyping-based approach has potential to dissect complex quantitative agronomic traits and delineate trait-regulatory genomic loci (candidate genes) for genetic enhancement in crop plants, including chickpea.

Chickpea (*Cicer arietinum* L.), a member of family Fabaceae is an annual, diploid, self-pollinated crop species with a very small genome size of ~740 Mbp. It is the most cultivated food legume/pulse crop world-wide that serves as an important dietary source of protein with essential amino acids for human. The seed and pod characters are major yield contributing traits of chickpea and vary widely across global germplasm collections, landraces and cultivated *desi* and *kabuli* accessions. Considering the agronomic importance of seed and pod traits, multiple efforts have been made for molecular mapping of quantitative trait loci (QTLs) governing major seed and pod yield contributing traits, including seed and pod number, double podding and seed weight in chickpea^1^,^2^,^3^,^4^,^5^,^6^,^7^,^8^,^9^,^10^,^11^,^12^,^13^,^14^,^15^,^16^,^17^,^18^,^19^,^20^,^21^,^22^,^23^,^24^,^25^,^26^,^27^,^28^,^29^,^30^,^31^. Unfortunately, due to lack of requisite intra-specific polymorphism in chickpea, no such robust genes/QTLs associated with seed and pod traits have been identified/fine mapped hitherto, which can be utilized for marker-assisted genetic improvement of chickpea. Therefore, to dissect such complex quantitative agronomic traits in chickpea, the marker-assisted breeding approach encompassing high resolution gene-specific and/or genome-wide LD (linkage disequilibrium)/association mapping and map-based gene/QTL cloning could be an attractive strategy in chickpea^24^,^26^,^32^. The discovery, large-scale validation and high-throughput genotyping of genome-wide SNPs in diverse natural germplasm accessions (core/minicore collections) and advanced generation bi-parental mapping populations of chickpea would essentially propel the above strategies to a great dimension.

Incredible efforts have been made in chickpea towards large-scale discovery of genome-wide SNPs in diverse cultivated and wild accessions using traditional Sanger and high-throughput next-generation sequencing (NGS) approaches^24^,^33^,^34^,^35^,^36^,^37^,^38^,^39^,^40^,^41^,^42^,^43^. Notably, few small-scale SNPs of these, validated by genotyping assays like Illumina GoldenGate/Infinium (Bead Xpress array) and Competitive Allele Specific PCR (KASPar) assays have been specifically exploited for high-resolution genetic linkage map construction and genetic/QTL mapping as well as comparative genome analysis involving chickpea and other legumes^27^,^44^,^45^,^46^,^47^. In present times, the NGS-based genotyping-by-sequencing (GBS) assay is considered the most preferred and convenient approach for large-scale discovery and high-throughput genotyping of genome-wide SNPs simultaneously in diverse crop germplasm accessions (association panel) and mapping populations with optimal use of resources^48^,^49^. The highest barcoded genotype multiplexing capability, and rapid SNP discovery and genotyping potential indeed emphasizes the efficacy of GBS for diverse high-throughput genetic analysis as well as multi-dimensional genomics-assisted breeding applications in crop plants^48^,^49^,^50^,^51^,^52^,^53^,^54^,^55^. Some of these applications include genome map saturation, targeted/fine mapping of trait-influencing genes/QTLs and genome-wide association studies (GWAS) in crop plants^56^,^57^,^58^,^59^,^60^,^61^,^62^,^63^,^64^,^65^,^66^,^67^,^68^.

However, the well-established GBS approach is yet to be utilised for large-scale mining and validating of genome-wide SNPs in diverse natural and mapping populations of chickpea. The necessary information scanned from diverse germplasm lines (association panel) and mapping populations by GBS assay could facilitate construction of ultra-high-density genetic linkage maps, molecular mapping/fine-mapping of genes/QTLs and high-resolution genome-wide/gene-specific trait association analysis in chickpea. It would further expedite the identification of novel functionally relevant molecular tags (markers, genes/QTLs, alleles and haplotypes) controlling qualitative and complex quantitative traits of agricultural importance (specifically seed and pod yield-related traits) for genetic enhancement of chickpea.

In view of the aforesaid possibilities, the present study has utilized GBS approach to discover, validate and genotype SNPs in a constituted seed and pod trait-specific association panel (211, including 92 diverse cultivated *desi* and *kabuli* chickpea accessions) at a genome-wide scale. The large-scale SNP genotyping, diversity and LD-based information of natural and mapping populations was further correlated with their robust field phenotyping data to identify functionally relevant potential genomic loci (gene-associated targets) regulating three seed and pod yield-contributing traits using a combinatorial approach of GWAS, QTL mapping, gene haplotype-specific LD mapping and transcript profiling in chickpea. These integrated analyses, coupled with our preliminary *in vitro* pollen tube growth and cellulose estimation assays, were able to identify one superior haplotype and favourable natural allelic variants in the upstream regulatory region of a candidate cellulose synthase (*Ca*_*Kabuli*_*CesA3*) gene. This candidate gene possibly governs high pod and seed number with its increased transcript expression and higher accumulation of cellulose, specifically in pollen and pod tissues, thereby promoting normal pollen and pollen tube growth in chickpea.

## Results

### Genome-wide mining and genotyping of GBS-based SNPs

**Figure 1:**
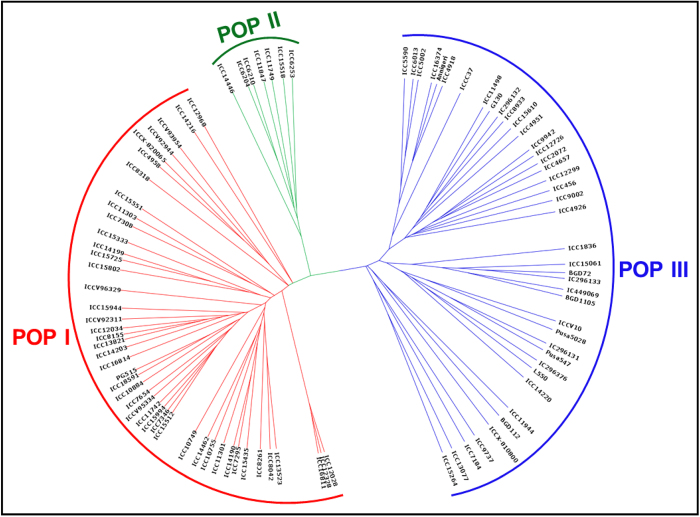
Unrooted phylogenetic tree depicting the genetic relations among 92 *desi* and *kabuli* chickpea accessions belonging to a seed and pod trait-specific association panel based on Nei’s genetic distance using 44844 high-quality GBS-based SNPs (MAF ≥ 0.05). Molecular classification differentiated these accessions into three different clusters/populations (POP I, POP II and POP III) as expected based on their cultivar/species-specific origination.

**Figure 2:**
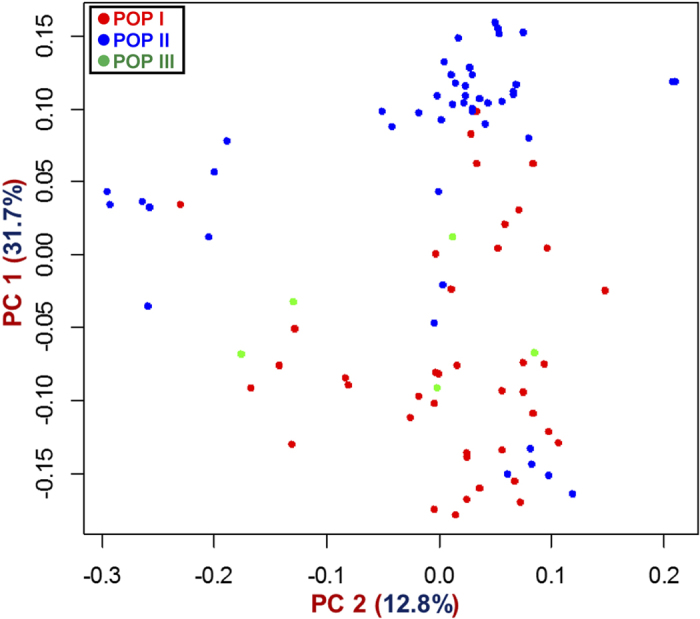
Principal component analysis (PCA) differentiating the 92 ***desi*** and ***kabuli*** chickpea accessions belonging to an association panel into three populations (POP I, POP II and POP III as determined by population genetic structure) for GWAS. The PC 1 and PC 2 explained 31.7% and 12.8% of the total variance, respectively.

**Figure 3:**
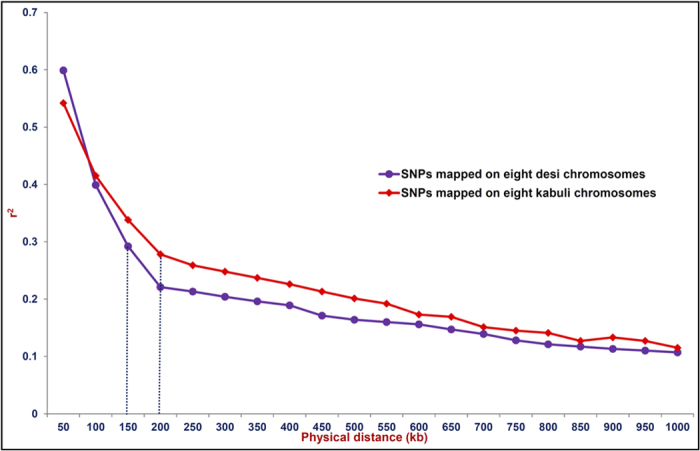
LD decay (mean r^2^) measured in a seed and pod trait-specific association panel (92 chickpea accessions) using 6063 and 14115 SNPs physically mapped on eight ***desi*** and ***kabuli*** chromosomes, respectively. The plotted curved lines illustrate the average r^2^ values among SNP loci spaced with uniform 50 kb physical intervals from 0 to 1000 kb.

The sequencing of 96-plex *Ape*KI GBS libraries generated 207.9 million high-quality reads that are uniformly distributed across 92 diverse chickpea accessions (seed and pod trait-specific association panel, Fig. S1, Table S1). In total, 44844, including 20439 and 24405 high-quality SNPs (with read-depth ≥10, SNP base quality ≥20, <10% missing data and ~2% heterozygosity in each accession) were identified (MAF ≥ 0.05) from *desi* and *kabuli* genomes, respectively using both reference and *de novo*-based GBS approaches in 92 accessions. Of these, 20178 SNPs, including 6063 and 14115 SNPs were physically mapped on eight *desi* and *kabuli* chromosomes with average inter-marker distances of 20.5 and 24.6 kb, respectively. All 44844 genome-wide GBS-based SNPs [submitted to NCBI dbSNP Build (B142) ( http://www.ncbi.nlm.nih.gov/SNP/snp_viewTable.cgi?handle=NIPGR)] showing polymorphism among 92 diverse *desi* and *kabuli* chickpea accessions were selected for GWAS. The neighbour-joining phylogenetic tree analysis, high-resolution population genetic structure and PCA among 92 cultivated *desi* and *kabuli* accessions (association panel) using 44844 genome-wide SNPs differentiated all these accessions from each other and clustered into three distinct populations (POP I, POP II and POP III) (Fig. 1,2). The determination of LD patterns in a constituted association panel using 20178 SNPs (physically mapped on eight chromosomes) revealed a higher LD estimates (0.50–0.62) and faster LD decay (150–200 kb) in three populations and both *desi* and *kabuli* chromosomes (Fig. 3).

### GWAS for three yield-contributing traits

**Figure 4:**
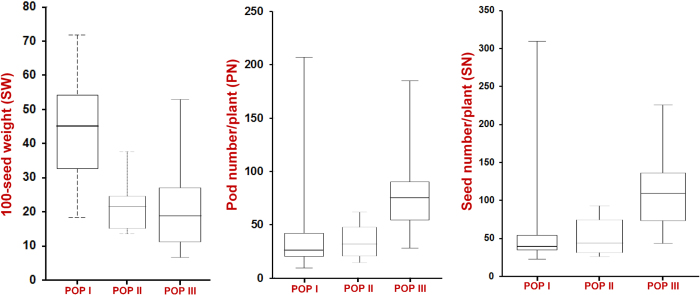
Boxplots showing the differences in three seed and pod yield-contributing traits (SW, PN and SN) among three populations (POP I, POP II and POP III) as defined by population structure. Box edges represent the upper and lower quantile with median value in the middle of the box.

The GWAS was performed by correlating the 20439 *desi* and 24405 *kabuli* SNP genotyping information of 92 accessions (association panel) with their phenotyping (replicated multilocation/years) data of three seed and pod yield-contributing quantitative traits (PN, SN and SW). A wider level of significant phenotypic variation (PN; 21.9–204.5 g with 70% H^2^, SN; 22.3–306.8 with 68% H^2^ and SW; 5.9–70.3 g with 80% H^2^) of these three quantitative traits was observed among 92 accessions belonging to an association panel (three population groups) based on two years of multi-location replicated field data (Fig. S2). The coefficient of variation was maximum (0.33) for PN, followed by SN (0.32) and minimum (0.27) for SW (Table 1). A higher significant (P < 0.0001) positive correlation between PN and SN (r = 0.95) and a negative correlation between PN and SW (−0.30) were observed. POP I showed higher phenotypic diversity for both PN (CV: 0.47) and SN (CV: 0.49) compared to POP II and POP III, whereas for SW, POP III (CV: 0.30) had comparable trait variation with POP I (CV: 0.29) (Table 1, Fig. 4).

**Figure 5:**
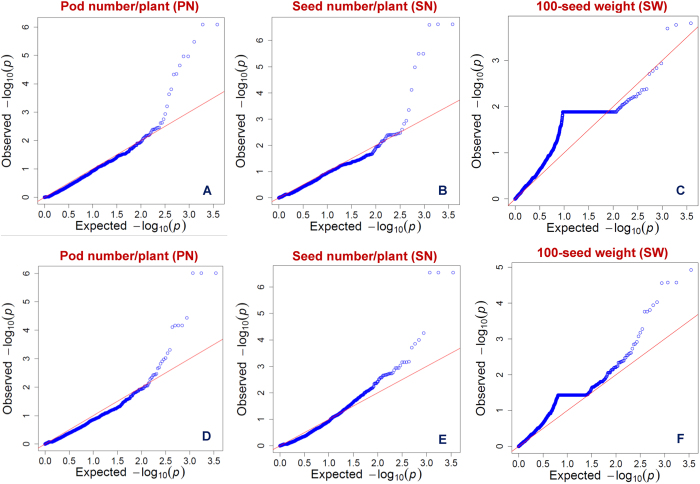
Quantile-quantile plots showing the comparison between expected and observed -log10 P-values with FDR cut-off <0.05 for identifying significant genomic loci associated with three agronomic traits (PN, SN and SW). The plots of each seed and pod yield-contributing traits among 92 chickpea accessions (association panel) were generated individually using 20439 *desi* (**A**, **B** and **C**) and 24405 *kabuli* (**D**, **E** and **F**) SNPs.

**Figure 6:**
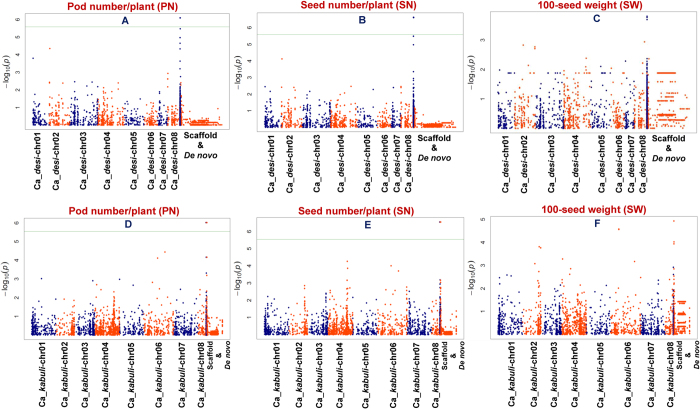
GWAS showing significant P-values (estimated integrating GLM, MLM, EMMA and CMLM) associated with three seed and pod yield-contributing traits (PN, SN and SW) using 20439 ***desi*** (A, B and C) and 24405 ***kabuli*** (D, E and F) SNPs (MAF ≥ 0.05). The x-axis indicates the relative density of identified reference genome- and *de novo*-based SNPs physically mapped on eight chromosomes and scaffolds of *desi* and *kabuli*. The y-axis represents the -log_10_ P-value for significant association with traits. The SNPs with P values ≤ 1x10^−6^ showing strong trait association are demarcated with lines. The SNPs with P-values ≤ 10^−4^ are considered significant for trait association.

For GWAS, the individual outcomes obtained from four model-based approaches of TASSEL (GLM and MLM) and GAPIT (EMMA and CMLM) using *desi* (20439 SNPs) and *kabuli* (24405) SNP genotyping data were analysed and compared with each other. The GWAS by use of *desi* SNP genotyping information identified 37, 36 and 28 genomic loci showing significant association with PN, SN and SW, respectively at a P ≤ 10^−4^. Similarly, *kabuli* SNP genotyping data detected 31, 66 and 53 significant (P ≤ 10^−4^) genomic loci showing association with PN, SN and SW, respectively. The comparison of a quantile-quantile plot (Fig. 5) of the expected and observed -log_10_ P-values with FDR cut-off ≤ 0.05 identified eight and seven genomic loci revealing strong association with PN and SN, respectively using *desi* SNP genotyping data (Fig. 6A,B,C, Table 2). Seven of these genomic SNP loci showed association with both PN and SN, whereas one SNP in the intronic region of AP2/ERF (APETALA2/ethylene responsive factor) transcription factor (two-repeated AP2/ERF functional domain) showed association with only PN (Fig. 6A,B,C, Table 2). One of the eight trait-specific unique genomic loci was physically mapped on *desi* chromosome 2 and the other seven genomic loci were distributed across different scaffold regions of *desi* genome. Five of the eight trait-associated genomic loci were present in the intergenic regions (two gene-associated targets) and the remaining three loci were annotated in different non-coding intronic and URR sequence components of three *desi* genes (Table 2).

The *kabuli* SNP genotyping information identified nine, five and four genomic loci associated with PN, SN and SW, respectively (Fig. 6C,D,E, Table 2). Eight of these genomic loci mapped on the *kabuli* genome showed association with multiple traits, including PN and SN, whereas the remaining 10 SNP loci had association with only a single trait. Eight of 14 trait-associated unique genomic loci identified in the *kabuli* genome were physically mapped on chromosomes 4 (1 SNP), 5 (1), 6 (5) and 8 (1). The remaining six SNPs were represented from different scaffold regions (three SNPs) of the *kabuli* genome and were also derived from *de novo* GBS data (three SNPs) (Fig. 6C,D,E, Table 2). Six of 14 trait-associated genomic SNP loci were present in the intergenic (five gene-associated targets) regions, whereas five SNP loci were annotated in different coding (three synonymous and non-synonymous SNPs), and non-coding intronic and URR sequence components of three *kabuli* genes (Table 2).

The GWAS in each of the three populations (POP I, POP II and POP III) and the entire association panel as a whole revealed no significant differences in terms of marker-trait association potential (identity and physical locations of GWAS-associated genomic loci) between populations I and III (contained a greater percentage of 90.3% accessions) compared with that of whole populations. For large-scale validation of trait-associated genomic loci identified in our study, the GoldenGate assay genotyping information of 96 trait-associated SNPs (Fig. S3) and field phenotyping data (PN, SN and SW traits) of 211 chickpea minicore accessions (Table S3) were utilized in GWAS. It confirmed and validated the trait association potential (identity and physical locations of SNP loci at a significant P value with FDR threshold ≤0.05) of genomic loci similar to that identified using genome-wide GBS-based SNP genotyping and phenotyping data of 92 accessions (Table 2). The significant percentage contributions of eight and fourteen unique genomic loci identified in *desi* and *kabuli* genomes for phenotypic variation (R^2^) varied from 23 to 47% (mean: 33%), respectively (Table 2). The significant SNPs identified in both *desi* and *kabuli* genomes explained an average of 30 (ranging 23–47%), 32 (23–47%) and 37% (35–39%) of the PN, SN and SW phenotypic variations in chickpea accessions, respectively. Incidentally, two SNPs in the genes encoding cellulose synthase (P: 1.1 × 10^−7^ to 2.2 × 10^−7^ with R^2^: 40–47%) and AP2/ERF transcription factor (P: 4.5 × 10^−5^ to 7.7 × 10^−5^ with R^2^: 25–29%) significantly associated with PN and SN were identified commonly between *desi* and *kabuli* genomes (Table 2). Two SNPs in the CDS of a haloacid dehydrogenase gene (mapped on *kabuli* chromosome 6) showing missense non-synonymous substitutions had association (P: 2.7 × 10^−5^ to 2.8 × 10^−5^ with R^2^: 37–39%) with SW (Table 2). One SNP locus (A/G) in the URR of the cellulose synthase gene (mapped on *kabuli* chromosome 5) revealing strong association (P: 1.1 × 10^−7^ to 1.3 × 10^−7^ with R^2^: 45–47%) with PN and SN as compared to other identified SNPs was considered as a potential candidate for further characterisation. Large-scale validation of this potential regulatory SNP in 211 minicore accessions further inferred significant contributions of A and G-alleles with ~40–45% PN and SN phenotypic variation in 152 high and 55 low pod and seed number-containing accessions, respectively (Fig. S4).

### Construction of an intra-specific genetic linkage map and validation of trait-associated genomic loci through QTL mapping

**Figure 7:**
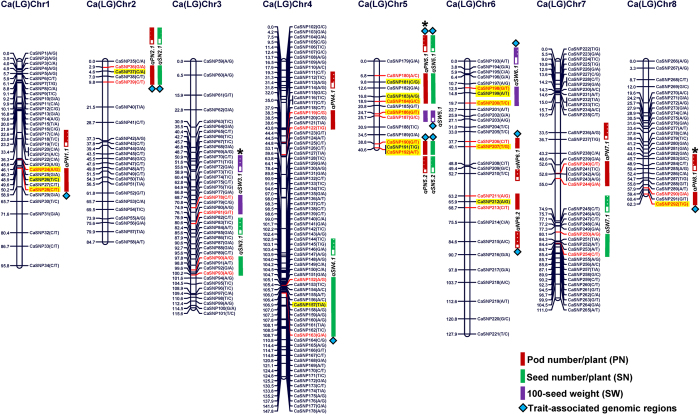
Fifteen major genomic regions harbouring 18 significant QTLs (PVE 13.6-28.7%) associated with three seed and pod yield-contributing traits (PN, SN and SW) identified and mapped on eight LGs (with a significant LOD score of >5.1; p <0.05) using a 283 F_4_ mapping population (ICC 6013 x ICC 7346). The genetic distance (cM) and identity of the marker loci integrated on the LGs are indicated on the left and right side of the chromosomes, respectively. Eighteen major genomic loci underlying 13 robust QTLs showed strong association with PN, SN and SW traits based on GWAS are highlighted with yellow. Brown, green and violet lines indicate the QTLs regulating PN, SN and SW mapped on eight LGs, respectively. The directions of QTLs (additive effects) are designated with filled (ICC 6013-specific alleles) vs. empty (ICC 7346-specific alleles) boxes. *qPN1.1* (QTL for pod number on chromosome 1 number 1), *qSN2.1* (QTL for seed number on chromosome 2 number 1) and *qSW3.1* (QTL for seed weight on chromosome 3 number 1). *qPN5.1, *qPN8.1* and *qSW3.1* correspond to known QTLs (TA103, TS45 and TA53 SSR markers flanking/linked to QTLs) from early reports by Radhika *et al.*^13^; Gowda *et al.*^18^; Varshney *et al.*^30^.

An intra-specific genetic linkage map (ICC 6013 x ICC 7346) was constructed by integrating 292 SNP markers (by MALDI-TOF SNP genotyping assay) on eight chickpea LGs (LG1 to LG8) (Table 3). The genetic map spanned a total map length of 785.6 cM, with an average inter-marker distance of 2.69 cM. The most saturated intra-specific genetic map was LG4 (average inter-marker distance: 1.92 cM), followed by LG8 (2.30 cM) and the least saturated map was LG7 (2.52 cM) (Table 3). Based on ANOVA, a significant difference of three yield-contributing quantitative traits (PN; 12.3–139.7 with 76% H^2^, SN; 19.8–225 with 70% H^2^ and SW; 6–45 g with 83% H^2^) among 283 segregating F_4_ mapping individuals and parental genotypes across two years was evident (Table S4). The normal frequency distribution (bi-directional transgressive segregation, Fig. S5) of these traits among mapping individuals and parental genotypes highlighted the utility of a mapping population (ICC 6013 x ICC 7346) generated for QTL analysis. The QTL mapping by use of genotyping data of 292 genetically mapped SNP markers and field phenotyping data (two geographical locations with two years/seasons) of the segregating individuals along with parental genotypes, identified and mapped 18 major (LOD: 5.1–9.7) QTLs governing PN, SN and SW on eight chickpea LGs (Table S5, Fig. 7). The phenotypic variation explained (PVE) for three agronomic traits by individual QTL (R^2^) varied from 13.6–28.7%. The PVE for all the 18 QTLs was 41.8%. All the identified QTLs showed positive additive gene effects (varied from 2.7–4.9), indicating the effective contribution of ICC 6013 alleles at these loci for increasing target traits (Table S5). Thirteen QTLs (LOD: 5.5–9.7) (R^2^: 17.9–28.7%) harbouring 18 genomic loci (six genes and six gene-associated targets) that were mapped on six chromosomes (spanned with 62 SNPs) had strong PN, SN and SW trait association potential based on our GWAS (Table 2, Fig. 7). Strong association potential of SNP (G/A) in URR of a cellulose synthase gene with PN and SN based on GWAS (R^2^: 45–47%) and QTL mapping (R^2^: 28.7%) than that of other trait-associated genomic loci was evident (Table 2). Henceforth, cellulose synthase gene including 11 other trait-influencing genes/gene-associated targets validated by both GWAS and QTL mapping (Table 2) were selected as target candidates to be further validated through expression profiling in chickpea.

### Validation of the trait-associated genes through differential expression profiling

**Figure 8:**
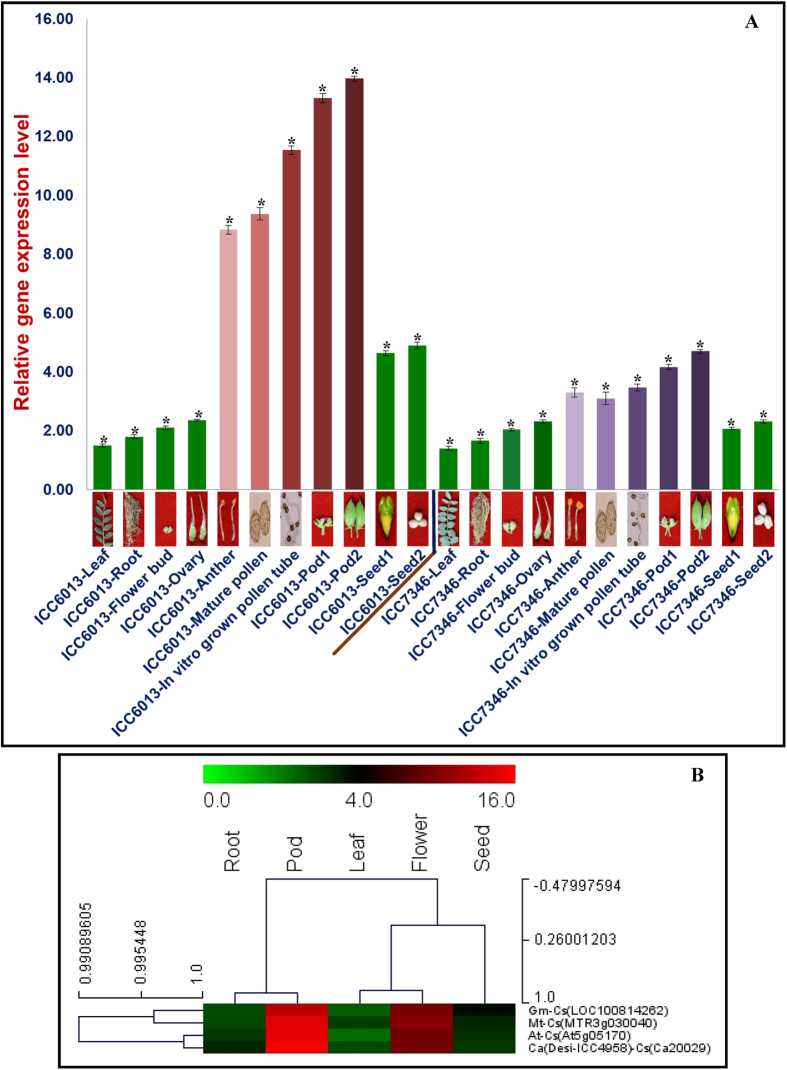
(**A**) Differential expression profiling of a strong PN- and SN-associated cellulose synthase gene in seven different vegetative (leaf and root) and reproductive (flower bud, ovary, anther, mature pollen and *in vitro* grown pollen tube) tissues as well as two pod and seed developmental stages of high (ICC 6013) and low (ICC 7346) pod and seed number-containing chickpea accessions using semi-quantitative and quantitative RT-PCR assays. The elongation factor-1 alpha gene was used as the internal control in the RT-PCR. The bars indicate the standard error. *Significance at p ≤ 0.01. (**B**) Hierarchical cluster display representing the differential expression profile of a strong PN- and SN-associated cellulose synthase gene (validated by GWAS and QTL mapping) determined by comparative global *in silico* digital transcript profiling. For digital transcript profiling, the whole-genome microarray expression and global transcriptome sequencing data available previously for a similar cellulose synthase gene in different vegetative and reproductive tissues of *Arabidopsis*, soybean, *Medicago* and chickpea were used. The colour-scale at the top represents average log signal expression values of genes in various tissues, in which green, black and red signify low, medium and high level of expression, respectively. The tissues and identities of cellulose synthase genes for which the differential expression data available in *Glycine max* (*Gm*), *Medicago truncatula* (*Mt*), *Arabidopsis thaliana* (*At*) and *Cicer arietinum* (*Ca*) are mentioned on the top and right-sides of expression map, respectively. We thankfully acknowledge DB and SB for taking this original photograph.

The differential expression profiling of 12 strong PN, SN and SW trait-influencing genes (validated by QTL mapping and GWAS) were performed in seven different vegetative and reproductive tissues and two pod and seed developmental stages of high (ICC 6013, ICCV 92311, ICC 9942, ICC 11944, ICC 456 and ICC 7184) and low (ICC 7346, Phule G0515, ICC 15994, ICC 14446, ICC 12034 and ICC 18591) pod and seed number-containing chickpea accessions using semi-quantitative and quantitative RT-PCR assays (Fig. S6A). A strong PN- and SN-associated gene (cellulose synthase) revealed preferential expression in the anthers, mature pollens, *in vitro* grown pollen tubes and pods of these accessions compared with their respective vegetative and reproductive tissues, including leaf, root, flower bud, ovary and seed (Fig. 8A, Fig. S6B). This gene showed pronounced differential up-regulation (~5.7-fold, P ≤ 10^−2^) in two pod developmental stages (compared with vegetative tissues) of all 12 high and low pod and seed number-containing chickpea accessions (Fig. 8A, Fig. S6B). Remarkably, the differential up-regulation of cellulose synthase gene in six high pod number-containing accessions during pod development was significantly higher (~3-fold, P ≤ 10^−2^) as compared to the six low pod number accessions (Fig. 8A, Fig. S6B).

To further validate the differential expression pattern of the strong PN and SN-associated cellulose synthase gene based on comparative global *in silico* digital transcript profiling, our experimental expression profiling data of this target gene was compared with its available whole-genome microarray expression and global transcriptome sequencing information in different vegetative and reproductive tissues of *Arabidopsis*, soybean, *Medicago* and chickpea. Interestingly, the cellulose synthase gene had preferential expression in flower and young pod/silique than its vegetative tissues, including root and leaf (Fig. 8B).

### Molecular haplotyping of a strong trait-regulatory gene

To determine the precise genomic constitution of a strong PN and SN trait-associated cellulose synthase gene (validated by GWAS, QTL mapping and differential expression profiling), a comprehensive genome-wide survey of all cellulose synthase protein-coding genes composing cellulose synthase superfamily was performed individually in *desi* and *kabuli* genomes. The structural and functional annotation of genes based on characteristics of their encoded functional domains (following the classifications of Richmond and Somerville^69^) identified 38 non-redundant cellulose synthase A (*CesA*; 20 genes) and cellulose synthase-like (*Csl*; 18) genes from *desi* (22) and *kabuli* (16) genomes (Fig. S7, Table S6). Twenty-four including 14 *CesA* and 10 *Csl* genes were physically mapped on either of the *desi* and *kabuli* chromosomes, whereas 14 (6 *CesA* and 8 *Csl* genes) genes were mapped on the scaffolds of *desi* genome. The genome-wide characterization of cellulose synthase superfamily in chickpea genome enabled to annotate a strong PN and SN trait-regulatory two functional domains (zinc-binding ring/U-box and cellulose synthase domains)-encoding cellulose synthase gene (validated by GWAS, QTL mapping and expression profiling), mapped on *kabuli* chromosome 5 as *CesA*-type cellulose synthase (*Ca*_*Kabuli*_*CesA3*) (Fig. S7).

**Figure 9:**
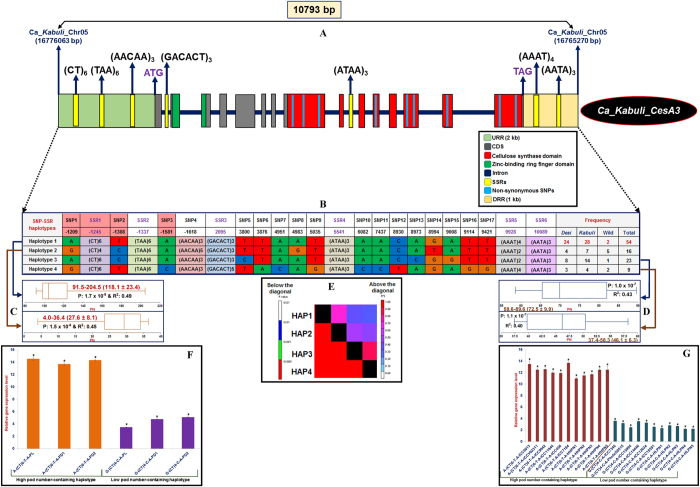
The gene haplotype-specific LD/association mapping and expression profiling in a strong PN- and SN trait-associated ***Ca_Kabuli_CesA3*** gene, validating its potential for regulating pod/seed number in chickpea. The genotyping of 91 SNPs and 7 SSRs in different coding and non-coding sequence component of cellulose synthase gene (**A**) among 102 accessions (92 association panel and 10 wild accessions) constituted four haplotypes (**B**). The two-specific haplotypes, haplotype 1: [A-(CT)_6_-T-A] and haplotype 2: [G-(CT)_4_-C-A], which were determined by three SNPs (−1209, −1308 and −1581 bp) and one SSR (−1245 bp) in the URR of gene showed strong association potential for high and low pod number differentiation, respectively (**C**). The four haplotype marker-based genotyping information produced greater LD estimates (r^2^ > 0.70 with P < 1.4 × 10^−4^) covering the entire 10793 bp sequenced region of the gene (**D**). The differential up-regulated expression of one high pod number-regulating superior haplotype in the URR of a *Ca_Kabuli_CesA3* gene was evident in mature pollen and two pod developmental stages of high pod number-containing accessions (E), homozygous mapping individuals and parental accession (ICC 6013) compared with that of low pod number accessions. HHPN: homozygous high pod number and HLPN: homozygous low pod number.

The sequencing of a 10793 bp cloned amplicon covering the entire 2 kb URR (upstream regulatory region), 15 exons, 1 kb DRR (downstream regulatory region) and 14 intronic regions of *Ca*_*Kabuli*_*CesA3* gene among 102 cultivated and wild chickpea accessions (92 association panel and 10 wild accessions) identified 91 SNPs and seven SSRs (Fig. 9A). Eight SNPs and three SSRs were identified from URR of this gene. Forty SNPs were detected in the intronic regions, whereas seven SNPs and two SSRs were mined from DRR of the gene. Thirty-six SNPs, including 13 missense non-synonymous SNPs (nine non-synonymous SNP loci within cellulose synthase-domain) and two SSRs were identified in the exons (CDS) of the target gene. The gene-based haplotype analysis was performed by integrating the genotyping data of 91 SNPs and seven SSRs among 102 accessions, which led to constitution of four haplotypes at most (PIC: 0.48–0.83; mean: 0.52) in each wild, *desi* and *kabuli* chickpea (Fig. 9B). About 61.5% *desi* and 52.8% *kabuli* accessions were represented by haplotype 1 compared with the other three haplotypes. In contrast, haplotype 2 contained the maximum percentage (4.9%) of wild accessions (Fig. 9C, D). The use of four SNP-SSR marker-based haplotypes in a *Ca*_*Kabuli*_*CesA* gene for haplotype-specific LD mapping and association analysis, depicted strong association potential (P = 2.1 × 10^−8^ with R^2^ = 0.51–0.53) of the gene with PN and SN. A significant higher degree of LD (r^2^ > 0.70 with P < 1.4 × 10^−4^) resolution was evident across the entire 10793 bp sequenced region of this strong PN trait-associated gene (Fig. 9E). Markedly, two specific haplotypes, haplotype 1: [A-(CT)_6_-T-A] and haplotype 2: [G-(CT)_4_-C-A] determined by three SNPs (−1209, −1308 and −1581 bp) and one SSR (−1245 bp) in URR of the gene showed strong association potential for high (54 accessions with PN: 91.5–204.5; mean: 118.1 and association potential P: 1.7 × 10^−8^ with R^2^: 0.49–0.52) and low pod number (16 accessions with PN 4.0–36.4; mean: 27.6 and P: 1.5 × 10^−8^ with R^2^: 0.45–0.47) differentiation, respectively (Fig. 9C,D). The superiority of haplotype 1 [A-(CT)_6_-T-A] identified in the URR of a *Ca*_*Kabuli*_*CesA3* gene was represented most commonly by high-pod/seed number-containing *desi* and *kabuli* accessions compared with the other three haplotypes. The pronounced up-regulated expression (~3.5-fold) of this high PN and SN trait-associated superior gene haplotype was observed in mature pollens and two pod developmental stages of six high (ICC 6013, ICCV 92311, ICC 9942, ICC 11944, ICC 456 and ICC 7184)-pod/seed number-containing chickpea accessions and homozygous mapping individuals compared with low (ICC 7346, Phule G0515, ICC 15994, ICC 14446, ICC 12034 and ICC 18591)-pod/seed number accessions and homozygous mapping individuals (Fig. 9F,G). The Northern-based expression profiling, that used a high pod/seed number-regulating superior haplotype-specific probe in the *Ca*_*Kabuli*_*CesA3* gene, detected strong hybridisation of 1.5 kb transcripts in anther, mature pollen and mature pod of a high pod and seed number-containing accession (ICC 6013). In contrast, weak cross-hybridisation of this transcript was observed in the ovary tissue of ICC 6013 (Fig. S8). The sharing of all four SNP-SSR marker-based haplotypes was observed among 92 cultivated (*desi* and *kabuli*) and 10 wild accessions. However, the low pod/seed number-associated haplotype 2 was primarily represented by low pod/seed number (PN: 4–20)-containing wild accessions from primary and secondary gene pools (*C. reticulatum* and *C. echinospermum*), whereas high pod/seed number (PN: 35–63) accessions of the tertiary gene pool (*C. judaicum*, *C. bijugum* and *C. pinnatifidum*) had haplotypes 1, 3 and 4 (Fig. 9B). Maximum sharing of haplotypes was observed between high pod/seed number-associated haplotype 1 in cultivated *desi* and *kabuli* accessions and low pod/seed number-regulatory haplotype 2 of wild accessions.

### Cellulose content might regulate the *in vitro* pollen tube growth

**Figure 10:**
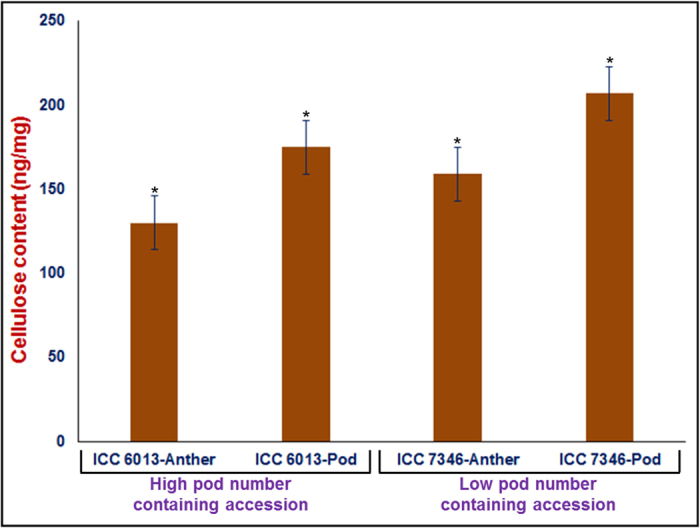
Cellulose content estimated in the anthers with mature pollen and young pods of high (ICC 6013) and low (ICC 7346) pod number-containing chickpea accessions. The bar indicates the standard error. *ANOVA significance at p < 0.001.

The *in vitro* pollen germination and pollen tube growth assays were performed in two chickpea accessions representing high (ICC 6013) and low (ICC 7346) pod/seed number-regulating haplotypes. We observed low (87%) pollen germination and pollen tube growth in the low pod number-containing accession ICC 7346 compared to high pod number accession ICC 6013 (93%) (Fig. S9, Table S7). To infer the possible cause of these differential *in vitro* pollen tube growth characteristics in these two contrasting accessions, the calcofluor white fluorescence pollen dye-staining approach was used to investigate the content and distribution of cellulose in pollen. The low pod/seed number accession, which normally fails to grow pollen tubes, did not display fluorescent blue signals (Fig. S9). In contrast, the *in vitro* normal pollen tube growing high pod/seed number accession displayed homogeneous intense fluorescent blue signals. Moreover, scanning electron microscopy revealed the presence of 2–3% irregularly shaped pollen, particularly in the low pod/seed number-containing accession (Fig. S9). The estimation of cellulose content in anthers (with mature pollen) and young pods of high and low pod/seed number-containing accessions (three biological replicates) detected significantly higher cellulose content in both anthers (150 ng/mg) and pods (180 ng/mg) of ICC 6013 compared with that of ICC 7346 (Fig. 10). Overall, our preliminary observations indicated that a significant reduction of cellulose content in the low pod/seed number-containing chickpea accession might affect its normal pollen germination and pollen tube growth compared with the high pod number accession.

## Discussion

### Efficacy of GWAS in trait-regulatory genomic loci identification

Considering disproportionate draft genome assembly and size (Mb) of chromosomal pseudomolecules between *desi* and *kabuli* genomes, GWAS needs to be performed separately using *desi* and *kabuli* SNPs, which in turn would strengthen the possibility of identifying numerous trait-associated valid genomic loci with wider genome coverage in chickpea. This strategy is also essential to access the precise trait association potential of genomic loci by establishing their correlation with chromosomal LD estimates and LD decay separately for *desi* and *kabuli* genomes. The implications of such chromosomal LD patterns in GWAS for identification of potential trait-associated genomic loci have been demonstrated in many crop plants including chickpea^70^,^71^. Henceforth, GWAS with 20439 *desi* and 24405 *kabuli* SNPs in this study enabled to identify two common validated PN and SN-associated genomic loci between *desi* and *kabuli* along with six and twelve PN, SN and SW-specific unique genomic loci individually from *desi* and *kabuli* genomes, respectively. The trait association potential (P ≤ 10^−4^ with R^2^ 23 to 47%) of these 22 genomic loci (gene-associated targets) for PN, SN and SW has been ascertained by combined use of four model-based algorithms of GLM, MLM, EMMA and CMLM and with a minimised confounding effect of population structure (FDR threshold <0.05). It is further supported by the large-scale validation, association study and strong marker-allele effects of 22 genomic loci on PN, SN and SW-specific phenotypic variation in 211 chickpea minicore accessions. Therefore, the phenotypic variation explained by SNP loci and trait association-related genomic information generated in our study is reliable and robust, thus having utility in establishing rapid marker-trait linkages and identifying genes/QTLs regulating important agronomic traits in chickpea. The significant association of multiple SNP loci in more than one genomic region (genes) with multiple quantitative traits (PN, SN and SW) hints at pleiotropy and/or local LD (closely linked genes), which might account for the complex genetic inheritance pattern of trait regulation. This association was further evident from high chromosomal and population-specific LD estimates with desirable LD decay of trait-associated linked/unlinked multiple SNP loci and the observed genetic heterogeneity of three quantitative traits (PN, SN and SW) across three populations. Despite diverse genetic architecture of PN, SN and SW in the three populations and along entire association panel, no significant differences concerning the marker-trait association potential of SNP loci in different populations was observed. The identification of strong PN, SN and SW trait-associated non-synonymous and regulatory SNP loci specifically in coding (functional domain) and URRs of protein-coding genes (for instance, *Ca_Kabuli_CesA3*, AP2/ERF transcription factor, and haloacid dehydrogenase) suggests their functional significance in rapid trait-regulatory gene identification and characterisation in chickpea. The non-synonymous substitution of SNP loci in the CDS (functional domain) of genes encoding variable amino acid residues could create altered secondary structures of proteins and functional domain regions that may affect the DNA binding and transcriptional activity of target genes during growth and developmental processes. Such possible transcriptional mechanism of trait regulation due to non-synonymous SNP (amino acid) substitutions and regulatory SNPs has already been reported in high seed weight-associated chickpea transcription factor genes^24^,^32^ and rice grain size genes (*GS3*, *GS5* and *qGL3*)^72^,^73^,^74^,^75^.

### A combinatorial approach of GWAS, QTL mapping, gene haplotype-specific LD mapping and transcript profiling accelerates functionally relevant trait-regulatory robust molecular tag identification in chickpea

Eighteen PN, SN and SW-associated genomic loci (six genes and six gene-associated targets) underlying 13 robust QTLs were validated by both SNP marker-based GWAS and traditional genetic/QTL mapping, which could essentially help in characterising these complex quantitative agronomic traits in chickpea. Altogether, 18 robust PN, SN and SW-associated robust QTLs (PVE: 13.6–28.7% and LOD: 5.1–9.7) harbouring 15 major genomic regions mapped on eight chickpea chromosomes were identified. To ascertain the potential and novelty of trait-specific 22 genomic loci and 18 QTLs identified by GWAS and QTL mapping, respectively, the markers linked/flanking the PN, SN and SW-associated known QTLs/genes, already reported in chickpea association and QTL mapping studies^1^,^8^,^13^,^18^,^24^,^30^,^32^,^71^,^76^,^77^, were considered for validation in our association panel and mapping population under study. Having compared the association and QTL mapping outcomes between past studies with our present one, two PN-regulating QTLs (*qPN5.1* and *qPN8.1*) and one SW-associated QTL (*qSW3.1*) showing correspondence with QTLs reported in three different QTL mapping studies^13^,^18^,^30^ based on congruent flanking/linked marker genetic positions (cM) on LGs were identified. Thus, most of the genomic loci and QTLs governing PN, SN and SW identified in our study by using high-resolution SNP marker-based association and genetic mapping are novel and population-specific. All these molecular tags once successfully validated in diverse genetic backgrounds or through fine mapping/map-based positional cloning, can be utilised for marker-assisted trait improvement in chickpea.

The tissue-specific expression of a particular cellulose synthase (*Ca_Kabuli_CesA3*) gene (validated both by GWAS and QTL mapping) in anther, mature pollen, *in vitro* grown pollen tube and pod, along with its higher differential up-regulation (~5.7-fold) in two pod developmental stages of primarily the high pod/seed number-containing chickpea accessions, reemphasises the functional relevance of this gene in possible regulation of pod/seed number in chickpea. The strong PN and SN trait association potential of this target gene was further ascertained by SNP-SSR marker-based haplotyping (four haplotypes), high-resolution gene-specific LD mapping in 102 cultivated and wild chickpea accessions and through haplotype-specific differential transcript profiling (semi-quantitative RT-PCR and Northern assays) in contrasting high and low pod/seed number-containing accessions and homozygous mapping individuals. These analyses confirm the strong association potential of a high pod/seed number-containing novel superior haplotype [A-(CT)6-T-A] identified in URR of a *Ca_Kabuli_CesA3* gene for pod/seed number differentiation and its implication in deciphering the transcriptional regulatory gene function during pod development in chickpea. The SNP-SSR marker-based haplotypes in the URR of this gene have significant role in regulating gene expression and identifying genes/QTLs governing multiple agronomic traits, including starch biosynthesis, seed shattering and seed setting rate in rice and seed weight in chickpea^24^,^32^,^78^,^79^ Henceforth, the strong PN and SN trait-regulatory *Ca_Kabuli_CesA3* gene identified in chickpea by integrating GWAS and QTL mapping with marker-based haplotyping and superior haplotype-specific transcript profiling, can be considered a candidate for marker-assisted genetic enhancement of chickpea to increase pod numbers and yield. The sharing of haplotypes constituted in a *Ca_Kabuli_CesA3* gene among 92 domesticated *desi* and *kabuli* and 10 wild chickpea accessions representing primary, secondary and tertiary gene pools was apparent. Out of these 102 accessions under analysis, 77 (75.5%) with high PN and SN-associated A-SNP allele of a *Ca_Kabuli_CesA3* gene were represented by only two haplotypes suggesting high artificial selection pressure on the trait-specific locus/haplotypes of this gene during chickpea domestication. The influence of positive selection on *Ca_Kabuli_CesA3* gene is more evident from its higher Ka/Ks (0.96) (higher than the average 0.84 Ka/Ks estimated in chickpea genes) and haplotype diversity (mean θπ: 0.61 and θω: 0.57) among cultivated and wild accessions. This clearly reflects the role of novel superior high pod/seed number-associated haplotypes and favourable natural allelic variants of the target cellulose synthase gene in regulating pod/seed number-specific traits and evolution during domestication of chickpea.

### Cellulose synthase: a potential candidate gene regulating pod and seed number in chickpea

The chickpea cellulose synthase (*Ca_Kabuli_CesA3*) gene homolog in *Arabidopsis thaliana* (*AtCesA3*), showing silique-specific expression^80^, is known to interact with the *AtbHLH15* (basic helix-loop-helix) gene^81^,^82^. Furthermore, based on a mutant complementation analysis, this gene act as a component of the catalytic core complex and is known to regulate cellulose synthesis^83^,^84^ in primary cell wall of pollen tubes during pollen development in *Arabidopsis*^80^,^85^. Pollen tube development during reproductive stage is the major factor said to control pod and seed number in legumes^86^. Because the cellulose synthase-encoding gene has significance in controlling pollen development by its proper cell wall growth in diverse crop plants, so, this gene may serve as a potential candidate for pod and seed number regulation in chickpea. Collectively, our results, encompassing GWAS, QTL mapping, transcript profiling and preliminary *in vitro* pollen tube growth and cellulose estimation assays, indicate that the high pod/seed number-specific novel superior haplotype [A-(CT)_6_-T-A] identified in URR of a *Ca_Kabuli_CesA3* gene might regulate a higher accumulation of cellulose synthase transcripts specifically, in mature pollen and pod tissues/developmental stages of high pod/seed number-containing chickpea accession, leading to normal pollen germination and pollen tube growth due to increased cellulose synthesis. As per our observations in the low pod/seed number-containing accession, normal pollen and pollen tube growth was affected possibly due to low cellulose deposition and reduced expression of the superior haplotype in URR of the *Ca_Kabuli_CesA3* gene in its pollens and pods (pod developmental stages). The higher expression of different isoforms of cellulose synthase gene in developing mature pollen and pollen tubes is known to increase cellulose content and enhance normal pollen tube growth in crop plants, including *Arabidopsis* and *Nicotiana*^87^,^88^,^89^. However, large-scale validation and a detailed molecular characterization of *Ca_Kabuli_CesA3* gene in diverse chickpea accessions as well as its mutant complementation analysis in *Arabidopsis* is required to understand its definite role in pod and seed number regulation in chickpea. Interestingly, the cellulose synthase gene has an additional vital role in imparting enhanced abiotic (drought and osmotic) and biotic stress tolerance in plant species, including *Arabidopsis* by root growth and lignification^90^,^91^,^92^. Henceforth, after being functionally well characterised, the strong PN/SN-regulatory cellulose synthase gene identified in our study via a combinatorial approach can be utilised in marker-assisted breeding for improving both yield (pod and seed number) and stress tolerance in chickpea.

Conclusively, our study identified 23798 high-quality genome-wide SNPs employing reference genome- and *de novo*-based GBS assays in a constituted seed and pod trait-specific association panel (92 *desi* and *kabuli* accessions) of chickpea. The use of a combinatorial approach of GWAS, QTL mapping, differential expression profiling, gene haplotype-based LD mapping and transcript profiling delineated one high pod/seed number-regulating superior haplotype and favourable allelic variants in the URR of a CesA-type cellulose synthase (*Ca_Kabuli_CesA3*) gene. The possible regulatory role of such superior gene haplotype with its increased transcript expression, cellulose accumulation in pollen and pod tissue and subsequently, normal pollen and pollen tube growth in high pod/seed number chickpea accession was evident. This novel integrative strategy for quick identification of functionally relevant trait-regulatory molecular tags can essentially dissect complex quantitative agronomic traits for marker-assisted genetic improvement of crop plants, including chickpea.

## Methods

### Plant resources used for genomic DNA extraction

To compose a seed and pod trait-specific association panel, 92 phenotypically and genotypically diverse (>80% diversity of total germplasm lines evaluated) accessions were selected from the available chickpea germplasm collections (16991, including 211 minicore germplasm lines)^93^,^94^,^95^ as per the detailed procedures described in Fig. S1. The constituted association panel (representing diverse eco-geographical regions of 21 countries of the world) with *C. arietinum desi* (39 accessions) and *kabuli* (53) accessions was used (Table S1) for large-scale discovery and high-throughput genotyping of genome-wide SNPs using GBS assay. For molecular mapping of QTLs, a 283 F_4_ mapping population derived from an intra-specific cross (ICC 6013 x ICC 7346) between two parental chickpea accessions (selected from the association panel) was developed. ICC 6013 (Indian origin) is a low 100-seed weight (10 g) and high pod (130) and seed number (210)-containing *desi* accession, whereas ICC 7346 (Mexican origin) is a high 100-seed weight (39 g) and low pod (26) and seed number (42)-containing *kabuli* accession. Genomic DNA was isolated from the leaves of 92 accessions and 283 mapping individuals using a QIAGEN DNeasy 96 Plant Kit (QIAGEN, CA, USA) as per the manufacturer’s instructions.

### Discovery, genotyping and annotation of genome-wide GBS-based SNPs

The 96-plex GBS libraries were made by digesting the genomic DNA of 92 chickpea accessions (association panel) with *Ape*KI and ligating the digested DNA to adapters containing one of 96 unique barcodes. The pooling of libraries and their sequencing (100-bp single end) were performed using Illumina HiSeq2000 with respect to Elshire *et al.*^49^ and Spindel *et al.*^60^. The high-quality FASTQ sequence reads (a *phred* score >10) were de-multiplexed relying their unique barcodes and the individual sequence reads of 92 accessions were mapped to reference drafts of *desi* (ICC 4958^42^) and *kabuli* (CDC Frontier^43^) chickpea genome sequences using Bowtie v2.1.0. The sequence reads remained unaligned with *desi* and *kabuli* reference genomes were further analyzed individually using the *de novo* genotyping approach of STACKS v1.0 ( http://creskolab.uoregon.edu/stacks). The sequence reads aligned and unaligned with each of *desi* and *kabuli* genomes were processed using the reference-based GBS pipeline of STACKS and the *de novo* genotyping approach of STACKS to identify valid and high-quality SNPs (no sequencing errors with minimum sequence read depth: 10 and SNP base quality ≥20) in 92 accessions. The structural and functional annotation of SNPs identified by reference-based GBS approach in various coding (synonymous and non-synonymous SNPs) and non-coding sequence components of genes and genomes (chromosomes/pseudomolecules and scaffolds) were performed using the available *desi* (CGAP v1.0^42^) and *kabuli*^43^ genome annotations.

### Phenotypic evaluation of agronomic traits

Ninety-two chickpea accessions representing an association panel along with two parental accessions and 283 segregating individuals of a F_4_ mapping population (ICC 6013 x ICC 7346) were grown in the field as per a randomised complete block design (RCBD) for two consecutive years (2011 and 2012) at two diverse geographical locations (Hyderabad; latitude: 17.1°N and longitude: 78.9°E, and New Delhi; 28.4°N and 77.1°E) in India during the crop growing season. These natural and mapping populations were phenotyped for three yield-contributing quantitative traits; pod number/plant (PN), seed number/plant (SN) and 100-seed weight (SW). The PN (the count of average number of fully formed pods per plant at maturity), SN (the count of average number of fully formed seeds per plant at maturity) and SW (g) (the average weight of 100-matured seeds at 10% moisture content) from 10–12 representative plants of each accessions and mapping individuals was measured. Diverse statistical parameters, including coefficient of variation (CV), frequency distribution, broad-sense heritability (H^2^), Pearson’s correlation coefficient and analysis of variance (ANOVA) of the phenotyping data were evaluated using SPSSv17.0 ( http://www.spss.com/statistics) and the methods of Saxena *et al.*^26^.

### Genome-wide association study (GWAS)

The genome-wide SNP genotyping information (MAF ≥ 5%), robust field phenotyping data of three seed and pod yield-contributing traits (PN, SN and SW), ancestry coefficient data (Q matrix deived from population structure at optimal population numbers) and relative kinship matrix (K) generated from 92 chickpea accessions (association panel) were analysed^24^,^32^ by use of general linear model (GLM, Q model)- and mixed linear model (MLM, Q + K model)-based approaches of TASSEL. Additionally, the principal component analysis (PCA) integrated with the efficient mixed-model (P + K, K and Q + K) association (EMMA) and P3D/compressed mixed linear model (CMLM) interfaces of GAPIT were utilised for GWAS. The relative distribution of observed -log_10_ P-value for each SNP marker-trait association was compared individually with that of the expected distribution using quantile-quantile plot of GAPIT. The adjusted P-value threshold of significance in each trait was corrected for multiple comparisons basing upon false discovery rate (FDR cut-off ≤ 0.05). Combining the four model-based outputs of TASSEL and GAPIT, the SNP loci in the target genomic (gene) regions (significant LD regions) revealing significant contributions to phenotypic variation of three agronomic traits at highest R^2^ (magnitude of marker trait-association) and lowest FDR adjusted P-values (threshold P < 2 × 10^−4^) were identified. For large-scale validation and verification of the accuracy of identified SNP marker-trait associations, the high-throughput genotyping data (Illumina GoldenGate assay^96^) of 96 SNPs (including strong trait-associated SNPs) in 211 chickpea minicore accessions were correlated with their field phenotyping information of the three agronomic traits under study, following the afore-mentioned GWAS methods.

### Genetic linkage map construction and QTL mapping

For genetic linkage map construction, 384 SNPs (physically mapped across eight chromosomes) showing polymorphism between two parental accessions (ICC 6013 and ICC 7346) were genotyped in 283 F_4_ segregating mapping individuals using a MALDI-TOF SNP genotyping assay with respect to Saxena *et al.*^26^,^97^. The SNP genotyping information was analysed in JoinMap 4.1 ( http://www.kyazma.nl/index.php/mc.JoinMap) at a higher LOD (logarithm of odds) threshold (>4.0) using Kosambi mapping function. The SNPs mapped on eight linkage groups (LGs) of an intra-specific chickpea genetic map were designated (LG1 to LG8) according to their corresponding physical positions (bp) on chromosomes, as determined in our study.

For QTL mapping, the genotyping data of parental polymorphic SNPs genetically mapped on eight LGs and field phenotyping information (PN, SN and SW) of 283 F_4_ mapping individuals and parental genotypes were analysed using the composite interval mapping (CIM) function (LOD > 4.0 with 1000 permutations and p ≤ 0.05) of MapQTL 6.^26^ The percentage of phenotypic variation explained (PVE) by significant QTLs (R^2^) was estimated to identify and map the novel major genomic regions harbouring QTLs associated with three agronomic traits in chickpea.

### Differential expression profiling

To infer differential gene regulatory function, the expression profiling of 12 strong trait (PN, SN and SW)-regulatory genes and gene-associated targets validated by both GWAS and QTL mapping was performed. Seven different vegetative (leaf and root) and reproductive (flower bud, ovary, anther, mature pollen and *in vitro* grown pollen tube after flowering) tissues were collected from 40–60 days old healthy plants of high (ICCV 92311, ICC 6013, ICC 9942, ICC 11944, ICC 456 and ICC 7184) and low (Phule G0515, ICC 7346, ICC 15994, ICC 14446, ICC 12034 and ICC 18591) pod and seed number-containing accessions. Moreover, tissues from two pod (~5 mm beginning pod size at 5–10 days after anthesis/DAA and ~2 cm full pod at 15–20 DAA) and seed developmental stages (early cell division phase during 10–20 days after podding/DAP and late maturation phase during 21–30 DAP) of these 12 contrasting accessions were gathered (adapted from^24^,^32^,^98^,^99^). RNA isolated from all the tissues were amplified by suitable gene-specific primers using semi-quantitative and quantitative RT-PCR assays. The differential expression level of genes observed in diverse tissues/developmental stages of 12 accessions was compared following Kujur *et al.*^24^.

For *in silico* digital expression analysis, the global transcript profiling data (generated from whole-genome microarray and transcriptome sequencing assays) available for a strong PN and SN trait-associated gene (validated by GWAS, QTL mapping and expression profiling) in different vegetative and reproductive tissues (leaf, root, flower, pod/silique and seed) of soybean (SoyBase; http://www.soybase.org), *Medicago* (*Medicago truncatula* Gene Expression Atlas; http://mtgea.noble.org/v3), *Arabidopsis* (AtGenExpress Visualization Tool; http://jsp.weigelworld.org/expviz/expviz.jsp) and chickpea (ICC 4958; Chickpea Transcriptome Database; http://www.nipgr.res.in/ctdb.html) were acquired and correlated with our experimental gene expression profiling results.

### Molecular haplotyping

For marker-based haplotyping, the entire non-coding 2 kb URR, exons, introns and 1 kb DRR of one strong PN- and SN-trait regulatory gene (validated by GWAS, QTL mapping and differential transcript profiling) amplified from 92 chickpea accessions (association panel) and 10 wild accessions (two accessions each from *C. echinospermum*, *C. judaicum*, *C. bijugum, C. pinnatifidum* and *C. reticulatum*) (Table S1) were cloned and sequenced. The high-quality gene sequences generated were aligned among accessions, and SSR and SNP loci were mined. The SSR-SNP marker-based haplotypes were constituted, and haplotype diversity and LD patterns were determined as per Kujur *et al.*^24^,^32^. The marker-based gene haplotype genotyping information was integrated with field phenotyping data (PN, SN and SW) of 102 accessions to evaluate trait-association potential and haplotype-based evolutionary significance of the gene in chickpea. Next to evaluate the potential of high pod/seed number-regulatory superior haplotypes constituted in a gene, differential expression profiling in mature pollen and two pod developmental stages of chickpea accessions representing high (ICCV 92311, ICC 6013, ICC 9942, ICC 11944, ICC 456 and ICC 7184) and low (Phule G0515, ICC 7346, ICC 15994, ICC 14446, ICC 12034 and ICC 18591) pod/seed number-specific haplotype groups was performed using gene haplotype-specific primers. In addition, high and low pod number-containing parental accessions (ICC 6013 and ICC 7346) and five homozygous individuals of a F_4_ mapping population were included for gene haplotype-specific expression study.

### Northern hybridisation

For Northern analysis, RNA (10–20 μg) isolated from four different tissues (ovary, anther, mature pollen and pod) of one chickpea accession (ICC 6013) representing a high pod number-specific superior haplotype was transferred to a Nylon membrane and RNA blots were prepared following Doblin *et al.*^87^. To prepare the radiolabelled probe for Northern hybridisation, the isolated RNA was amplified using high pod/seed number-regulating gene haplotype-specific primers (producing 1.5 kb amplicon) and the amplicon was further labelled with α-P^32^-dCTP using a random primer labelling kit (MBI, Fermentas, USA). The blots were pre-soaked with pre-hybridisation buffer, hybridised with denatured radiolabelled probe (at 65 °C for 16–18 h) and washed using wash solution I (2x SSC + 0.1x SDS at 65 °C/20 min), wash solution II (1x SSC at 65 °C/20 min) and wash solution III (0.25x SSC at 65 °C/5 min). The blots were wrapped with plastic wrap; exposed to a phosphoimaging K-Screen (Bio-Rad, USA) overnight and visualised using PharosFX of a Phosphoimager (Bio-Rad, USA).

### *In vitro* pollen-tube growth assay, scanning electron microscopy and cellulose estimation

Pollen grains were collected from only dehisced anthers of two chickpea accessions representing high (ICC 6013) and low (ICC 7346) pod and seed number-regulating haplotypes. The pollens were placed on glass slides coated with pollen tube growing medium (1 mM CaCl_2_, 1 mM MgSO_4_, 0.01% boric acid and 18% sucrose)^84^. Calcofluor white (Sigma-Aldrich, USA) was used for staining the cellulose. After 4 h of incubation in pollen germination medium at 30 °C, the pollen tube growth was observed. For scanning electron microscopy, the pollens of two accessions were fixed in FAA (3.7% formaldehyde, 50% ethanol and 5% acetic acid) and dehydrated using an ethanol series. The samples were further gold-palladium coated and observed using a Zeiss EVO 40 scanning electron microscope under low vacuum at 20 kV. The cellulose content in the mature pollen-containing anther and young pod tissues of ICC 6013 and ICC 7346 was estimated following Ghosh *et al.*^100^.

## Additional Information

**How to cite this article**: Kujur, A. *et al.* A genome-wide SNP scan accelerates trait-regulatory genomic loci identification in chickpea. *Sci. Rep.* **5**, 11166; doi: 10.1038/srep11166 (2015).

## Supplementary Material

Supplementary Information

## Figures and Tables

**Table 1:** Statistical measures estimated for three seed and pod yield-contributing quantitative traits in three model-based individual population and in the entire populations.

**Populations**	**100-seed weight (SW)**				**Pod number/plant (PN)**				**Seed number/plant (SN)**			
	**Mean ± S.D.**	**Range**	**CV**	**H**^**2**^ **(%)**	**Mean ± S.D.**	**Range**	**CV**	**H**^**2**^ **(%)**	**Mean ± S.D.**	**Range**	**CV**	**H**^**2**^ **(%)**
POP I	38.4 ± 11.2	19.1–70.3	0.29	81	46 ± 21.5	21.9–204.5	0.47	73	50.8 ± 24.7	22.3-306.8	0.49	70
POP II	31.6 ± 7.8	15.6–39.2	0.25	80	45.4 ± 13.2	28.9–67.4	0.29	70	51 ± 18.5	30.8-80.9	0.36	69
POP III	19.7 ± 10.9	5.9–52.9	0.30	82	83.6 ± 25.9	39.7–189	0.31	72	114.7 ± 32.5	42.2-243.9	0.28	72
All populations	29.3 ± 9.8	5.9–70.3	0.27	80	62.4 ± 19.3	21.9–204.5	0.33	70	78.9 ± 25.3	22.3-306.8	0.32	68

S.D: standard deviation, CV: coefficient of variation and H^2^: broad-sense heritability.

**Table 2:** Functional relevance of 22 trait-associated genomic loci (gene-associated targets) identified by integrating GWAS, QTL mapping and differential expression profiling.

SNP IDs*^a^	***Desi*** **and** ***kabuli*** **chromosomes/ scaffolds**	**SNPs physical positions (bp)**	**SNPs (major/minor alleles)**	**Minor allele frequency (MAF)**	**Genes/gene-associated targets**^b^	**Structural annotation**	**Association analysis**			**Genetic/QTL mapping**^c^				
							**P**	**R**^**2**^ **(%)**	**Associated traits**	**LGs/chromosomes**	**QTLs identity**	**QTL intervals with genetic positions (cM)**	**LOD**	**R**^**2**^**(%)**
CaSNP190	*Desi*-Sc-289	150550	(G/T)	0.29	Ca11846^b^ (AP-1 complex subunit sigma-1)	Intergenic	2.5 × 10^−6^-3.4 × 10^−6^	26 & 38	PN & SN	CaLG(Chr5)	*qPN5.2* & *qSN5.2*	38.8-40.6	7.2	18.6
CaSNP52	*Desi*-Sc-330	3079	(G/T)	0.30	Ca12072^b^ (Leucine-rich repeat receptor-like protein kinase)	Intergenic	2.4 × 10^−6^-8.2 × 10^−6^	28 & 37	PN & SN	–	–	–	–	–
CaSNP192	*Desi*-Sc-994	225780	(A/T)	0.30	Ca09657^b^ (Unknown expressed protein)	Intron	2.1 × 10^−6^-8.5 × 10^−6^	35 & 26	PN & SN	CaLG(Chr5)	*qPN5.2* & *qSN5.2*	38.8-40.6	7.2	18.6
CaSNP24	*Desi*-Sc-2103	15896	(A/G)	0.29	—	Intergenic	3.2 × 10^−6^-1.1 × 10^−5^	34 & 23	PN & SN	CaLG(Chr1)	*qPN1.1*	46.1-50.2	8.4	23.6
CaSNP7	*Desi*-Sc-2103	15914	(T/G)	0.29	—	Intergenic	2.6 × 10^−6^-1.3 × 10^−5^	28 & 31	PN & SN	—	—	—	—	—
CaSNP70	*Desi*-Sc-4437	15942	(C/T)	0.30	—	Intergenic	4.7 × 10^−5^	26	PN	—	—	—	—	—
CaSNP182	*Desi*-Sc-8364	45741	(A/G)	0.29	Ca20029^b^ (*Ca_Desi_CesA10*)^d^	URR	1.1 × 10^−7^-2.2 × 10^−7^	42 & 40	PN & SN	—	—	—	—	—
CaSNP37	*Desi*-Chr2	595853	(C/A)	0.24	Ca01065^b^ (AP2/ERF transcription factor)	Intron	4.5 × 10^−5^-7.6 × 10^−5^	29	PN	CaLG(Chr2)	*qPN2.1* & *qSN2.1*	2.9-9.8	7.4	21.7
CaSNP191	*Kabuli*-Sc-193	186515	(T/G)	0.30	Ca25161^b^ (Unknown expressed protein)	Intergenic	6.8 × 10^−5^	24	PN	CaLG(Chr5)	*qPN5.2* & *qSN5.2*	38.8-40.6	7.2	18.6
CaSNP26	*Kabuli*-Sc-193	186523	(T/C)	0.30	Ca25161 (Unknown expressed protein)	Intergenic	6.2 × 10^−5^	26	PN	CaLG(Chr1)	*qPN1.1*	46.1-50.2	8.4	23.6
CaSNP28	*Kabuli*-Sc-1504	189681	(C/T)	0.30	Ca24092^b^ (Unknown expressed protein)	Intergenic	2.5 × 10^−6^- 9.3 × 10^−6^	31 & 36	PN & SN	CaLG(Chr1)	*qPN1.1*	46.1-50.2	8.4	23.6
CaSNP183	*Kabuli*-Chr5	16766270	(A/G)	0.35	Ca17942^b^ (*Ca_Kabuli_CesA3*)^d^	URR	1.1 × 10^−7^- 1.3 × 10^−7^	45 & 47	PN & SN	CaLG(Chr5)	*qPN5.1* & *qSN5.1*	6.8-18.9	9.7	28.7
CaSNP207	*Kabuli*-Chr6	27310325	(G/T)	0.25	Ca14618^b^ (AP2/ERF transcription factor)	Intron	7.7 × 10^−5^	25	PN	CaLG(Chr6)	*qPN6.1*	37.7-40.1	8.0	18.2
CaSNP212	*Kabuli*-Chr6	41708309	(G/A)	0.28	—	Intergenic	3.6 × 10^−5^	27	PN	CaLG(Chr6)	*qPN6.2*	63.2-68.7	7.4	19.0
CaSNP292	*Kabuli*-Chr8	16102414	(G/T)	0.30	Ca15527b(Domain of unknown function DUF292	Intergenic	2.9 × 10^−6^-9.8 × 10^−6^	31 & 35	PN & SN	CaLG(Chr8)	*qPN8.1*	59.4-62.3	7.4	17.9
CaSNP181	*Kabuli*-*De novo*	309293	(C/G)	0.30	—	—	2.4 × 10^−6^-9.0 × 10^−6^	30 & 33	PN & SN	CaLG(Chr5)	*qPN5.1* & *qSN5.1*	6.8-18.9	9.7	28.7
CaSNP184	*Kabuli*-*De novo*	316184	(G/C)	0.30	—	—	5.5 × 10^−5^	25	PN	CaLG(Chr5)	*qPN5.1* & *qSN5.1*	6.8-18.9	9.7	28.7
CaSNP157	*Kabuli*-Chr4	38343633	(A/T)	0.32	Ca13120^b^ (Calcium dependent protein kinase)	Intergenic	5.5 × 10^−5^	28	SN	CaLG(Chr4)	*qSN4.1*	105.1-108.7	7.8	21.8
CaSNP198	*Kabuli*-Chr6	16113521	(G/T)	0.21	Ca19678^b^ (Haloacid dehydrogenase)	CDS (Syn)	2.8 × 10^−5^	38	SW	CaLG(Chr6)	*qSW6.1*	12.5-19.7	8.2	19.5
CaSNP199	*Kabuli*-Chr6	16113522	(A/T)	0.24	Ca19678 (Haloacid dehydrogenase)	CDS (NSyn)	2.7 × 10^−5^	39	SW	CaLG(Chr6)	*qSW6.1*	12.5-19.7	8.2	19.5
CaSNP200	*Kabuli*-Chr6	16113523	(T/C)	0.23	Ca19678 (Haloacid dehydrogenase)	CDS (NSyn)	2.7 × 10^−5^	37	SW	CaLG(Chr6)	*qSW6.1*	12.5-19.7	8.2	19.5
CaSNP186	*Kabuli*-*De novo*	63305	(T/G)	0.30	—	—	1.2 × 10^−5^	35	SW	CaLG(Chr5)	*qSW5.1*	24.7-25.7	6.5	18.7

^*^CaSNP (*Cicer arietinum* SNP).

^a^Details regarding markers are provided in the Table S2.

^b^Validated through differential expression profiling.

^c^Details regarding QTLs are mentioned in the Table S5.

^d^Details provided in the Table S6 and Figure S7.

CDS (coding sequences), Nsyn (non-synonymous), Syn (synonymous) and URR (upstream regulatory region). AP2/ERF transcription factor classified following Magnani *et al.* (2004) Plant Cell 16:2265 and Nakano *et al.* (2006) Plant Physiology 140:411.

**Table 3:** SNPs mapped on the eight LGs/chromosomes of an intra-specific chickpea genetic linkage map.

**LGs/chromosomes**	**Mapped SNP markers**	**Map length covered (cM)**	**Average inter-marker distance (cM)**
CaLG (Chr)1	34	95.8	2.82
CaLG(Chr)2	24	84.7	3.53
CaLG(Chr)3	43	115.6	2.70
CaLG(Chr)4	77	147.8	1.92
CaLG(Chr)5	14	40.6	2.90
CaLG(Chr)6	29	127.9	4.41
CaLG(Chr)7	44	110.9	2.52
CaLG(Chr)8	27	62.3	2.30
**Total**	**292**	**785.6**	**2.69**

CaLG (Chr): *Cicer arietinum* linkage group (chromosome).
