# Supplementary material for: A genome-wide SNP scan accelerates trait-regulatory genomic loci identification in chickpea
Source: Sci Rep. 2015 Jun 10;5:11166. doi: 10.1038/srep11166 (PMC4461920; doi:10.1038/srep11166)
Supplement: Supplementary Information [file srep11166-s1.pdf]

A genome-wide SNP scan accelerates trait-regulatory genomic loci identification in chickpea

Alice Kujur[1a], Deepak Bajaj[1a], Hari D. Upadhyaya[2a], Shouvik Das[1], Rajeev Ranjan[1], Tanima Shree[1], Maneesha S. Saxena[1], Saurabh Badoni[1], Vinod Kumar[3], Shailesh Tripathi[4], C.L.L. Gowda[2], Shivali Sharma[2], Sube Singh[2], Akhilesh K. Tyagi[1], Swarup K. Parida[1]*

[1]National Institute of Plant Genome Research (NIPGR), Aruna Asaf Ali Marg, New Delhi 110067, India

[2]International Crops Research Institute for the Semi-Arid Tropics (ICRISAT), Patancheru 502324, Andhra Pradesh, India

[3]National Research Centre on Plant Biotechnology (NRCPB), New Delhi 110012, India

[4]Division of Genetics, Indian Agricultural Research Institute (IARI), New Delhi 110012, India

[a]These authors contributed equally to this work.

*Corresponding Author

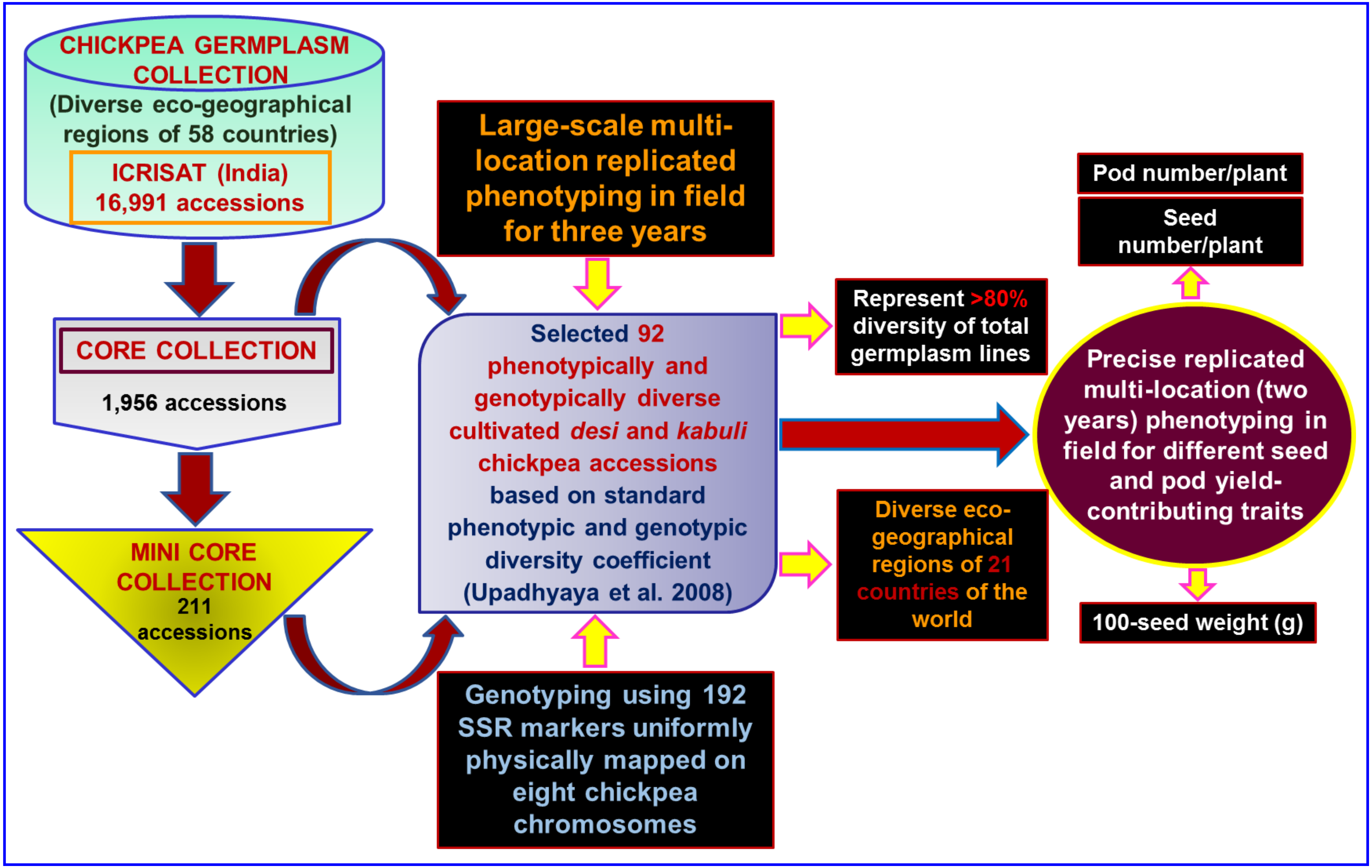

Fig. S1: Major steps/strategies followed for selecting 92 phenotypically and genotypically diverse chickpea accessions belonging to a seed and pod trait-specific association panel from 16991, including 1956 core and 211 mini-core germplasm lines that are available at the International Crops Research Institute for the Semi-Arid Tropics (ICRISAT), India.

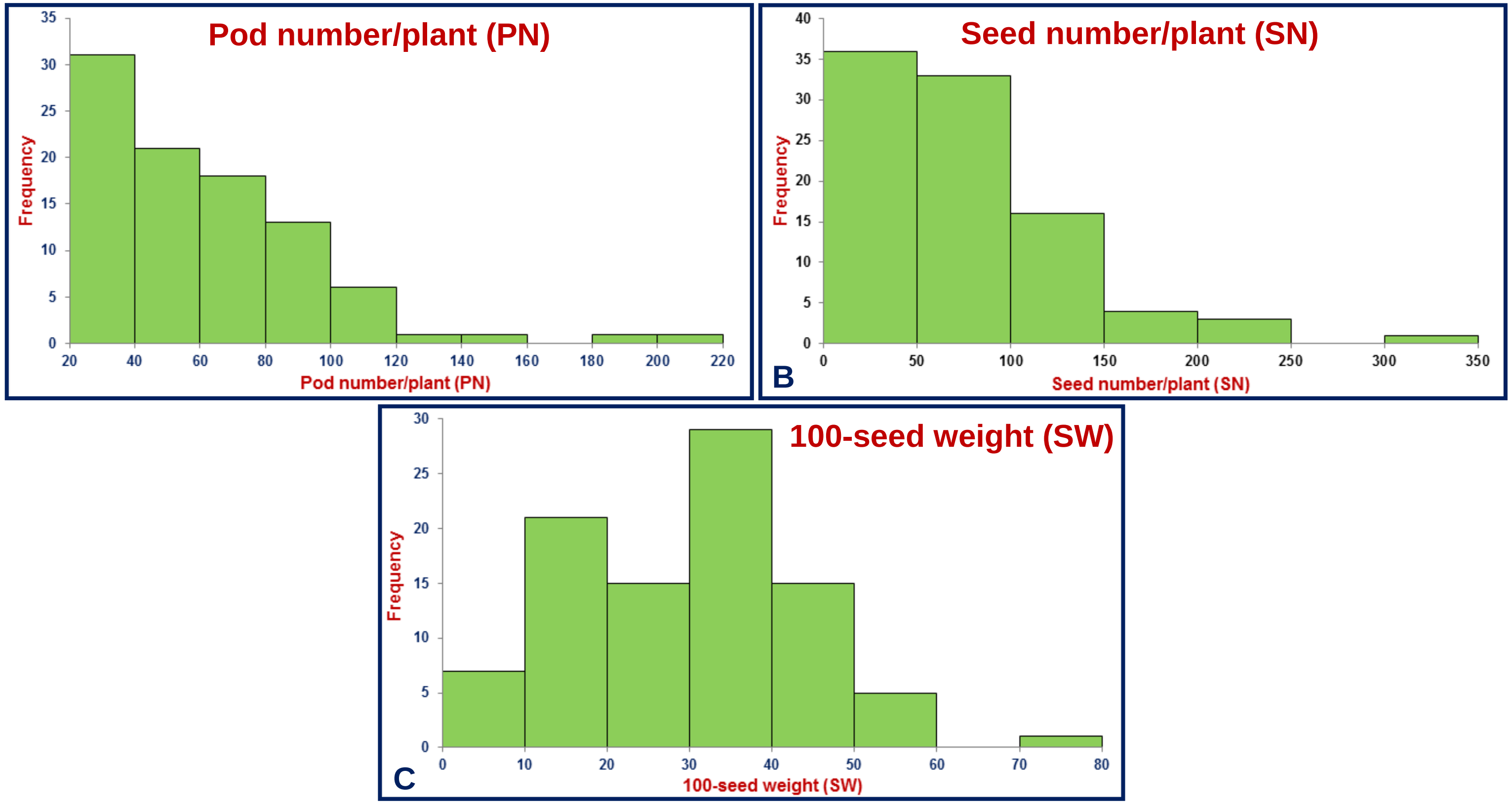

Fig. S2: Frequency distribution (histogram) of three seed and pod yield-contributing quantitative traits (PN, SN and SW) in 92 chickpea accessions belonging to an association panel. Blank lines represent the null distribution.

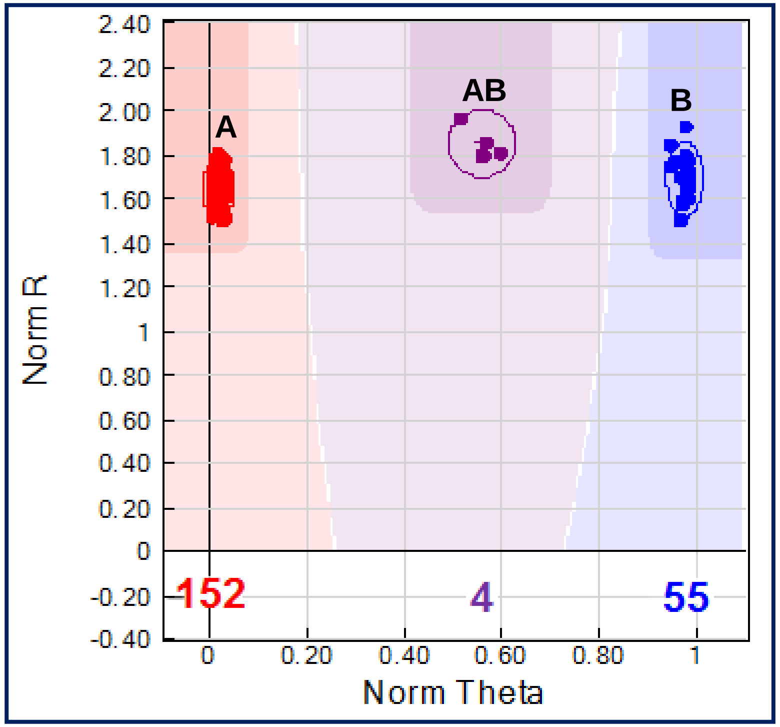

Fig. S3: Example of one SNP (A/G) validated in a cellulose synthase gene by the Illumina GoldenGate genotyping assay showing homozygous and heterozygous cluster separation for 211 chickpea minicore accessions based on plotting of normalised R [sum of intensities of the two channels (Cy3 and Cy5)] on the y-axis vs. normalised theta [$(2/\pi)Tan^{-1}(Cy5/Cy3)$] on the x-axis. A normalised theta value nearest 0 is homozygous for allele A (red), a theta value nearest 0.5 is heterozygote AB (violet) and a theta value nearest 1 is homozygous for allele B (blue).

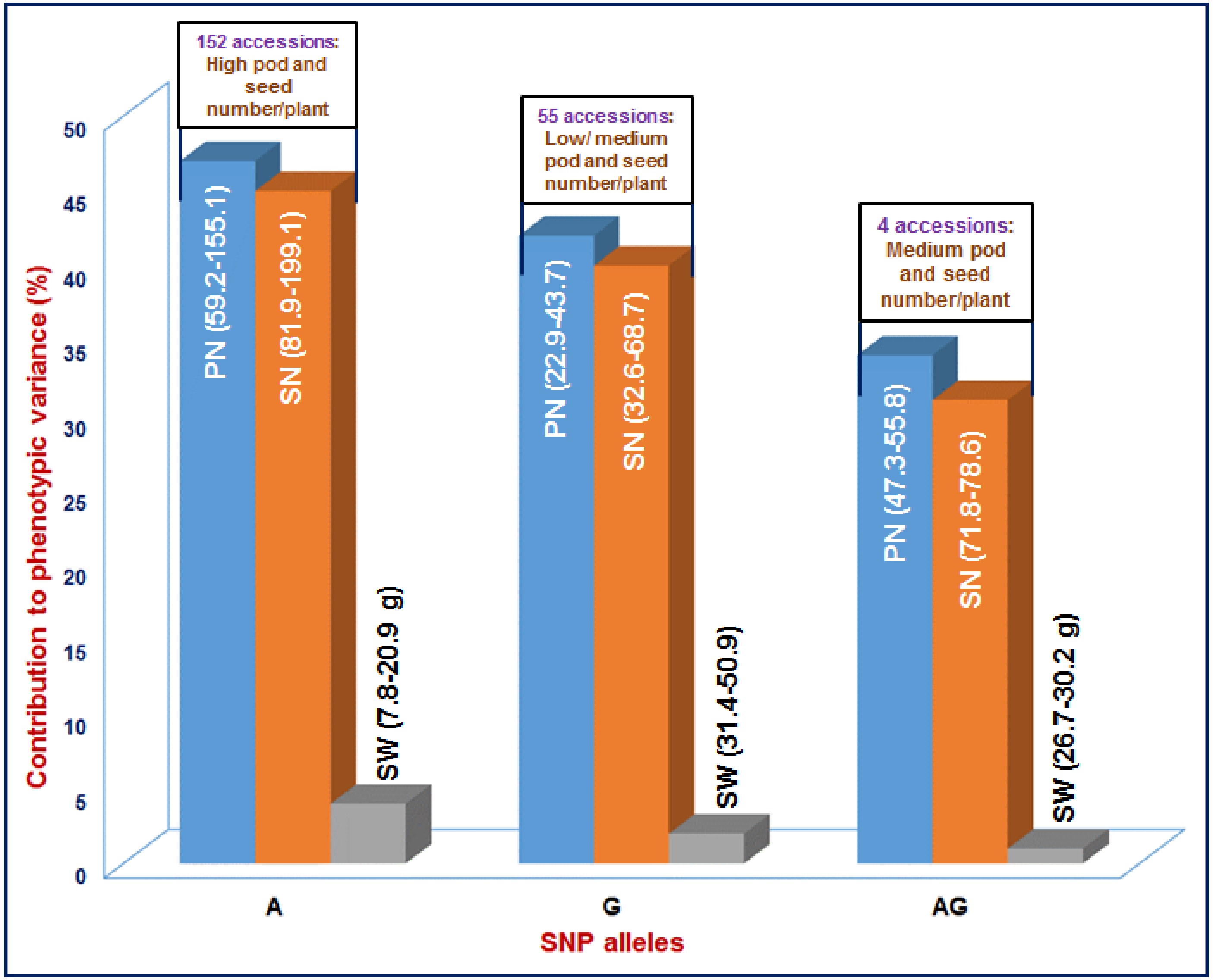

Fig. S4: Large-scale validation and marker allele effects of GWAS-associated SNP (A/G) identified in URR of a cellulose synthase gene for PN, SN and SW trait variation in 211 chickpea minicore accessions. The significant contributions of A- and G-alleles with ~40-45% PN and SN phenotypic variation in 152 high and 55 low pod- and seed number-containing accessions were evident. AG allele had 30-35% PVE for PN and SN in four medium pod- and seed number-containing accessions. Low trait association potential of these gene-based SNP alleles with SW was evident. Range of phenotypic variation observed for PN, SN and SW traits in minicore accessions are mentioned inside the bars.

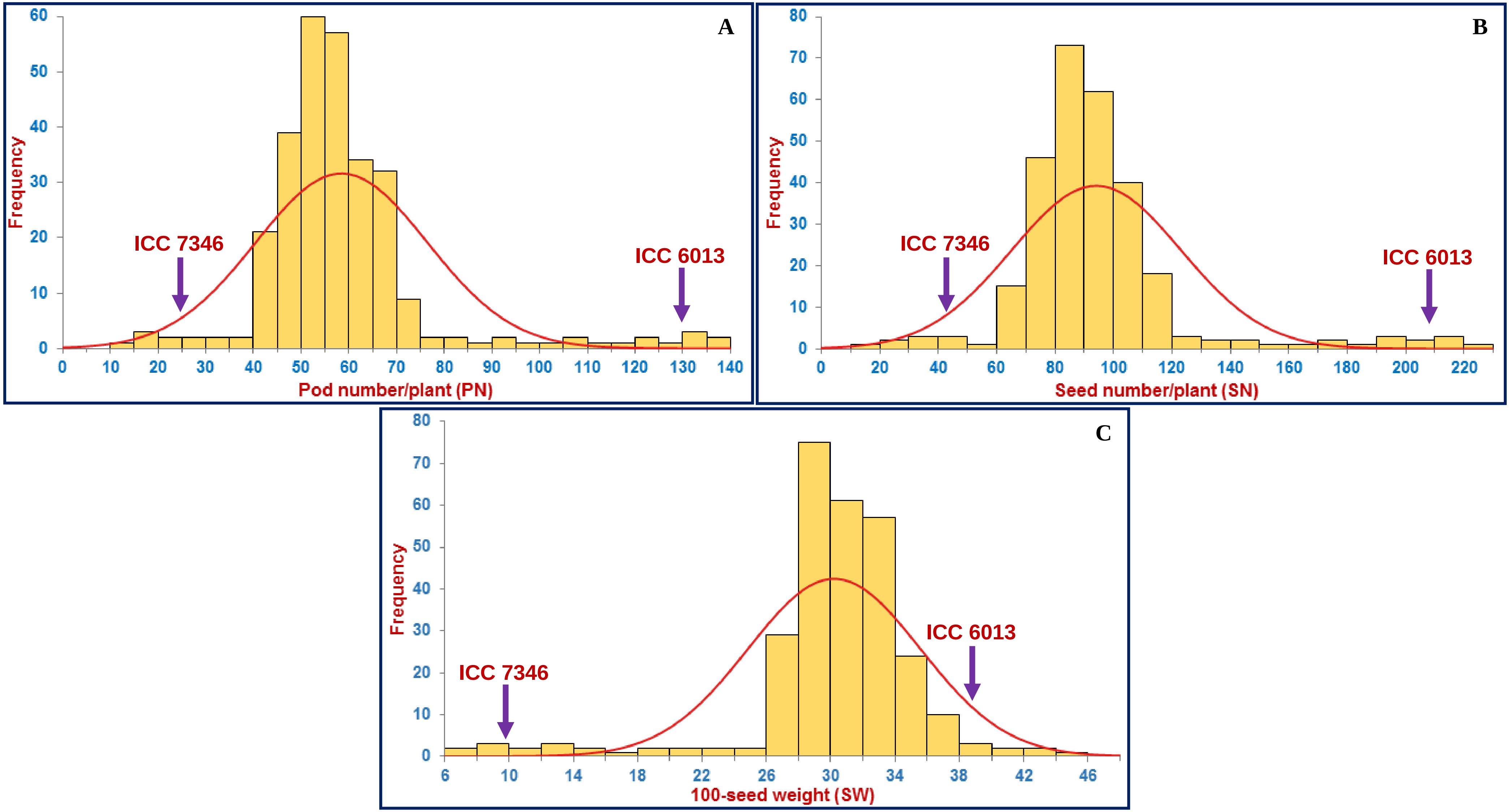

Fig. S5: Frequency distribution of three seed and pod yield-contributing quantitative traits; PN (A), SN (B) and SW (C) in 283 F_4 mapping individuals (ICC 6013 x ICC 7346) and parental accessions, depicted a goodness of fit to the normal distribution.

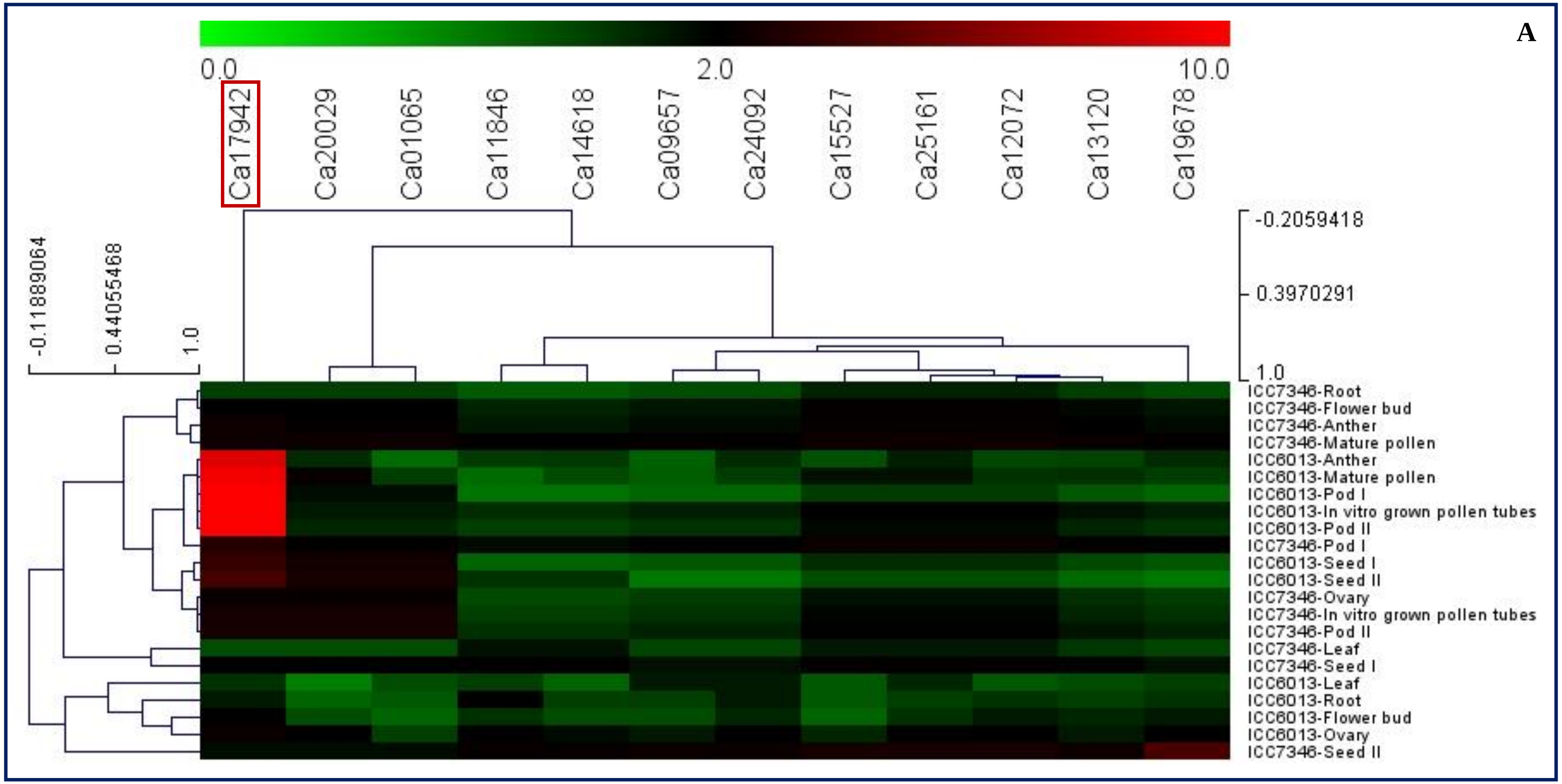
A
0.0
2.0
10.0
Ca17942
Ca20029
Ca01065
Ca11846
Ca14618
Ca09657
Ca24092
Ca15527
Ca25161
Ca12072
Ca13120
Ca19678
-0.2059418
0.3970291
1.0
-0.11889064
0.44055468
1.0
ICC7346-Root
ICC7346-Flower bud
ICC7346-Anther
ICC7346-Mature pollen
ICC6013-Anther
ICC6013-Mature pollen
ICC6013-Pod I
ICC6013-In vitro grown pollen tubes
ICC6013-Pod II
ICC7346-Pod I
ICC6013-Seed I
ICC6013-Seed II
ICC7346-Ovary
ICC7346-In vitro grown pollen tubes
ICC7346-Pod II
ICC7346-Leaf
ICC7346-Seed I
ICC6013-Leaf
ICC6013-Root
ICC6013-Flower bud
ICC6013-Ovary
ICC7346-Seed II

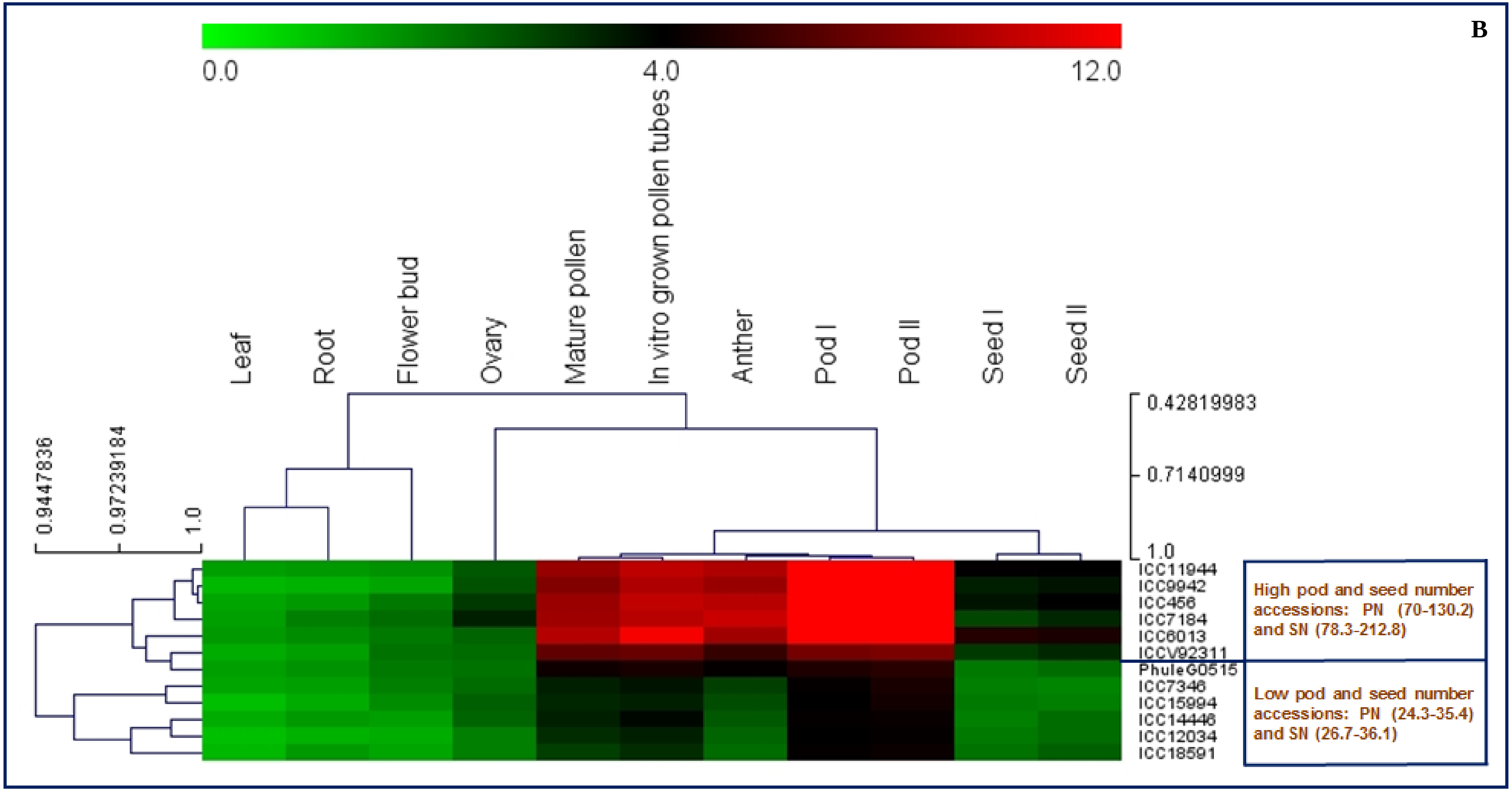

Fig. S6: Hierarchical cluster display represented differential expression profiles of (**A**) 12 genes and gene-associated targets (validated by GWAS and QTL mapping) including a (**B**) cellulose synthase gene (validated by GWAS, QTL mapping and expression profiling) in seven different vegetative and reproductive tissues and two pod developmental stages of high (ICCV 92311, ICC 6013, ICC 9942, ICC 11944, ICC 456 and ICC 7184) and low (Phule G0515, ICC 7346, ICC 15994, ICC 14446, ICC 12034 and ICC 18591) pod and seed number-containing accessions. The colour scale at the top represents average log signal expression values of genes in various tissues and developmental stages; in which green, black and red color signify low, medium and high level of expression, respectively. (**A**) One cellulose synthase *kabuli* gene (Ca17942) showing higher differential up-regulation during pod development of high pod and seed number-containing accession (ICC 6013) is highlighted with red boxes. The details on identities of genes are mentioned in the Table 3. (**A**) The tissues and genes used for expression profiling are mentioned on the right and top side of expression map, respectively. (**B**) The tissues and genotypes used for expression profiling are mentioned on the top and right side of expression map, respectively.

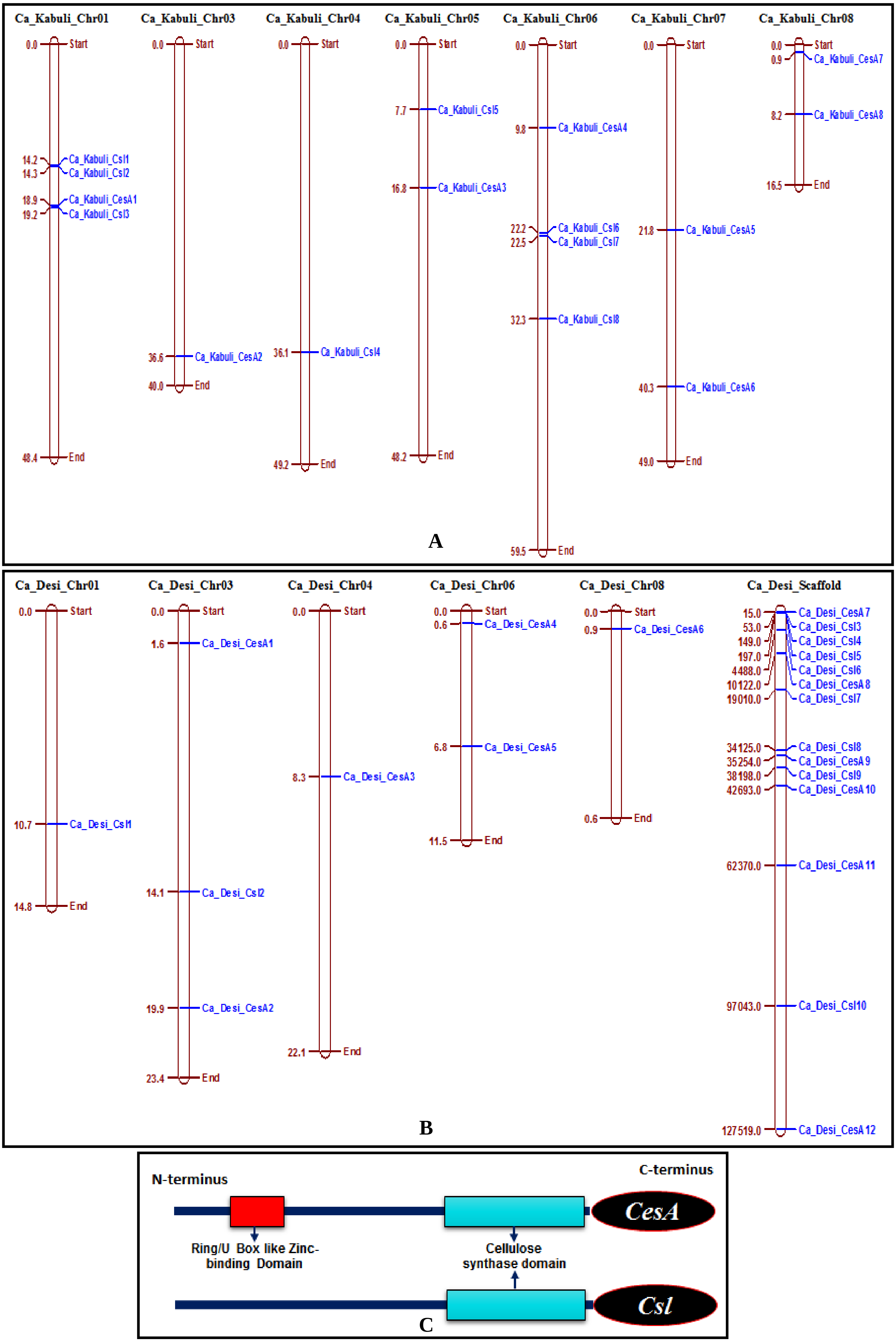

Ca_Kabuli_Chr01
0.0 Start
14.2 Ca_Kabuli_Csl1
14.3 Ca_Kabuli_Csl2
18.9 Ca_Kabuli_CesA1
19.2 Ca_Kabuli_Csl3
48.4 End
Ca_Kabuli_Chr03
0.0 Start
36.6 Ca_Kabuli_CesA2
40.0 End
Ca_Kabuli_Chr04
0.0 Start
36.1 Ca_Kabuli_Csl4
49.2 End
Ca_Kabuli_Chr05
0.0 Start
7.7 Ca_Kabuli_Csl5
16.8 Ca_Kabuli_CesA3
48.2 End
Ca_Kabuli_Chr06
0.0 Start
9.8 Ca_Kabuli_CesA4
22.2 Ca_Kabuli_Csl6
22.5 Ca_Kabuli_Csl7
32.3 Ca_Kabuli_Csl8
59.5 End
Ca_Kabuli_Chr07
0.0 Start
21.8 Ca_Kabuli_CesA5
40.3 Ca_Kabuli_CesA6
49.0 End
Ca_Kabuli_Chr08
0.0 Start
0.9 Ca_Kabuli_CesA7
8.2 Ca_Kabuli_CesA8
16.5 End
A
Ca_Desi_Chr01
0.0 Start
10.7 Ca_Desi_Csl1
14.8 End
Ca_Desi_Chr03
0.0 Start
1.6 Ca_Desi_CesA1
14.1 Ca_Desi_Csl2
19.9 Ca_Desi_CesA2
23.4 End
Ca_Desi_Chr04
0.0 Start
8.3 Ca_Desi_CesA3
22.1 End
Ca_Desi_Chr06
0.0 Start
0.6 Ca_Desi_CesA4
6.8 Ca_Desi_CesA5
11.5 End
Ca_Desi_Chr08
0.0 Start
0.9 Ca_Desi_CesA6
0.6 End
Ca_Desi_Scaffold
15.0 Ca_Desi_CesA7
53.0 Ca_Desi_Csl3
149.0 Ca_Desi_Csl4
197.0 Ca_Desi_Csl5
4488.0 Ca_Desi_Csl6
10122.0 Ca_Desi_CesA8
19010.0 Ca_Desi_Csl7
34125.0 Ca_Desi_Csl8
35254.0 Ca_Desi_CesA9
38198.0 Ca_Desi_Csl9
42693.0 Ca_Desi_CesA10
62370.0 Ca_Desi_CesA11
97043.0 Ca_Desi_Csl10
127519.0 Ca_Desi_CesA12
B
N-terminus
C-terminus
CesA
Ring/U Box like Zinc-binding Domain
Cellulose synthase domain
Csl
C

Fig. S7: A physical map depicting the genomic distribution of 38 non-redundant cellulose synthase protein-encoding genes on chromosomes/scaffolds of *kabuli* (**A**) and *desi* (**B**) genomes. The physical distance (Mb) and identities of the genes integrated on the chromosomes/scaffolds are indicated on the left and right side of the chromosomes, respectively. The size of the chromosomes (Mb) are based on the draft genome sequences of *kabuli* (Varshney et al., 2013) and *desi* (Jain et al., 2013). Details regarding identities of two classes of cellulose synthase genes annotated from *desi* and *kabuli* genomes are mentioned in the Table S6. (**C**) Broad classification of 38 chickpea genes belonging to cellulose synthase superfamily into two groups based on diverse domain organization of representative cellulose synthase-encoding proteins from each of the cellulose synthase A (*CesA*) and cellulose synthase-like (*Csl*) family.

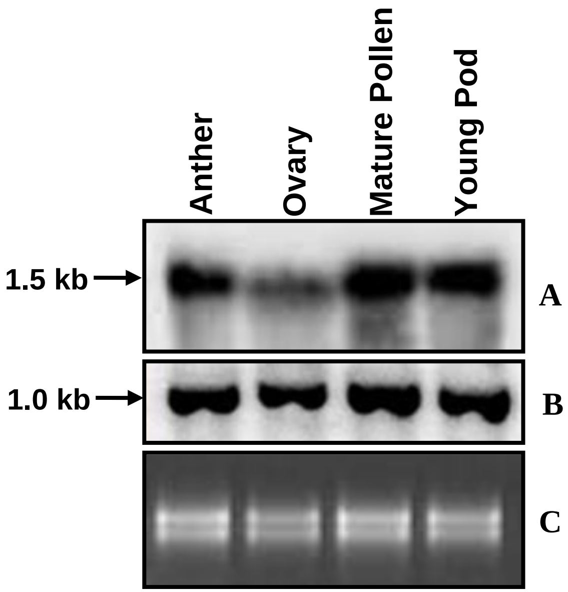

Fig. S8: Autoradiogram showing northern hybridisation pattern of a high pod number-regulating superior cellulose synthase gene-based haplotype (**A**) in the anther, ovary, mature pollen and young pod of a high pod number-containing chickpea accession (ICC 6013). The transcripts sizes (in kb) hybridising with the probes (**A**: cellulose synthase gene-specific haplotype and **B**: elongation factor 1-alpha as the internal control) are indicated by arrows. **C**: Normalised RNA isolated from the anther, ovary and mature pollen and young pod tissues of ICC 6013.

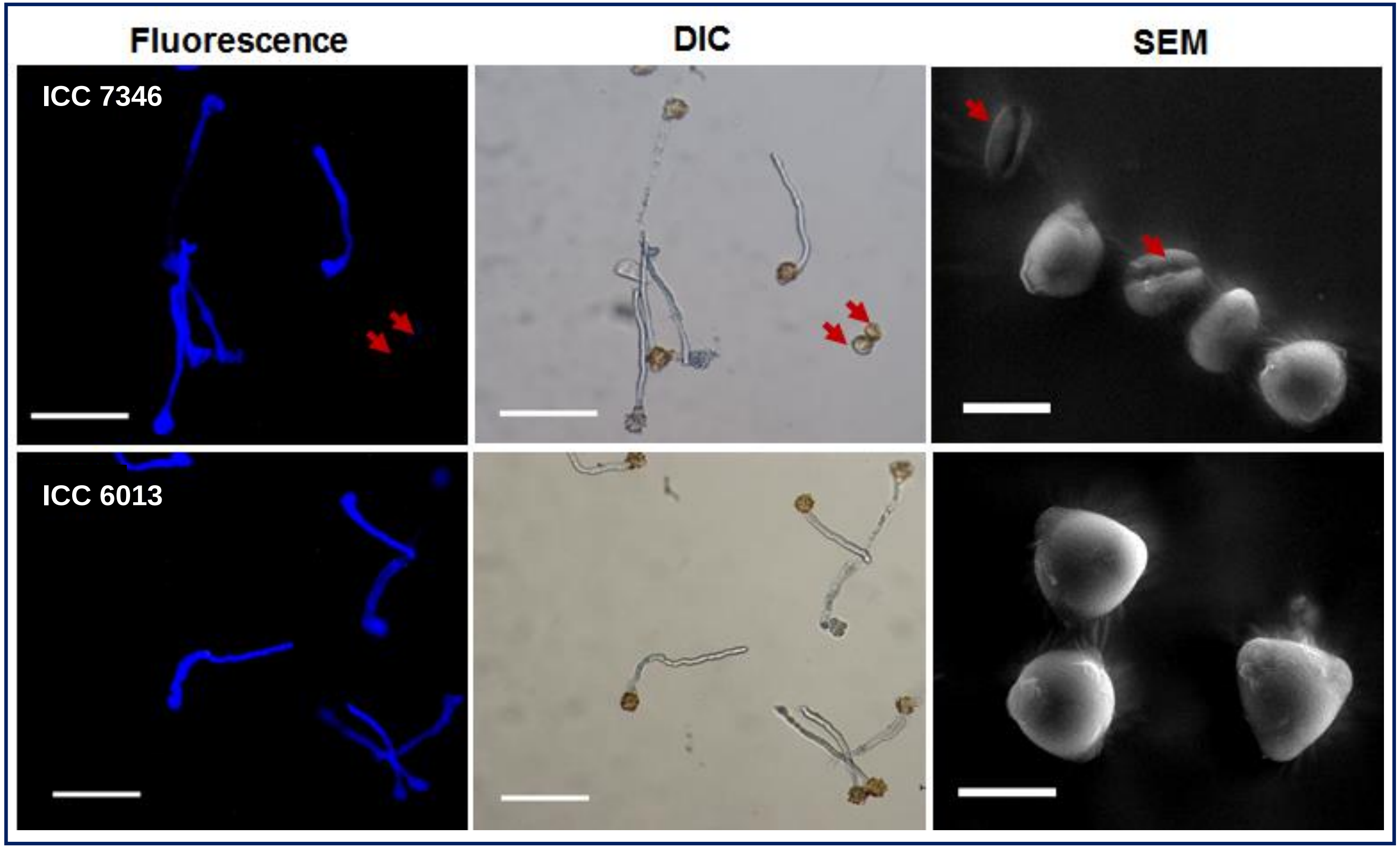

Fig. S9: Observation of pollen and pollen tube growth. Pollens of high (ICC 6013) and low (ICC 7346) pod number-containing accessions were used for *in vitro* germination and observed under differential interference contrast (DIC) and fluorescence channel microscopy after staining with calcofluor white fluorescent dye. Red arrows in the fluorescence and DIC panels indicate the pollen of the Phule G0515 accession that failed to germinate and that did not show fluorescent blue signals possibly due to its low cellulose content. Scanning electron microscope (SEM) images depict the presence of some irregular shaped pollen specifically in the ICC 7346 accession. (Scale bars: fluorescence and DIC, 100 μm, and SEM, 20 μm).

Table S1: Chickpea accessions belonging to a seed and pod trait-specific association panel) selected for GWAS

Sl. No.	Accession Numbers	Cultivar types	Geographical origin	Biological status
1	ICC5590	*Desi*	India	Breeding/Research material
2	ICC6013	*Desi*	India	Breeding/Research material
3	ICC5002	*Desi*	India	Breeding/Research material
4	ICC7184	*Desi*	Turkey	Traditional cultivar/Landrace
5	ICC4926	*Desi*	India	Breeding/Research material
6	ICC4657	*Desi*	India	Traditional cultivar/Landrace
7	ICC12299	*Desi*	Nepal	Traditional cultivar/Landrace
8	ICC456	*Desi*	India	Traditional cultivar/Landrace
9	ICC12726	*Desi*	Ethiopia	Traditional cultivar/Landrace
10	ICC11944	*Desi*	Nepal	Traditional cultivar/Landrace
11	ICC11498	*Desi*	India	Traditional cultivar/Landrace
12	ICC9942	*Desi*	India	Traditional cultivar/Landrace
13	ICC2072	*Desi*	India	Traditional cultivar/Landrace
14	ICC9737	*Desi*	Afghanistan	Traditional cultivar/Landrace
15	ICC9002	*Desi*	Iran	Traditional cultivar/Landrace
16	ICC16374	*Desi*	Malawi	Breeding material
17	ICC12028	*Desi*	Mexico	Traditional cultivar/Landrace
18	ICC8318	*Desi*	India	Traditional cultivar/Landrace
19	ICC15610	*Desi*	India	Traditional cultivar/Landrace
20	ICC4918	*Desi*	India	Advanced cultivar
21	ICC1836	*Desi*	India	Traditional cultivar/Landrace
22	ICC15061	*Desi*	India	Traditional cultivar/Landrace
23	ICC4958	*Desi*	Central India	Traditional cultivar/Landrace/genetic stock
24	ICCV10	*Desi*	India	Released Cultivar
25	ICCX-810800	*Desi*	Northern India	Variety
26	IC296132	*Desi*	Northern India	Variety
27	IC296131	*Desi*	Northern India	Variety
28	BGD112	*Desi*	Northern India	Genetic stock
29	ICC4951	*Desi*	Central India	Landrace
30	ICC8933	*Desi*	Northern India	Genetic stock
31	ICCV93954	*Desi*	Southern India	Variety
32	BGD72	*Desi*	Northern India	Genetic stock
33	IC296133	*Desi*	Northern India	Variety
34	ICCV92944	*Desi*	Central India	Variety
35	ICCC37	*Desi*	Southern India	Variety
36	ICCX-820065	*Desi*	Western India	Variety
37	Pusa5028	*Desi*	Northern India	Variety
38	Pusa547	*Desi*	Northern India	Variety
39	G130	*Desi*	India	Released Cultivar
40	ICC13523	*Kabuli*	Iran	Traditional cultivar/Landrace
41	ICC15802	*Kabuli*	Syria	Traditional cultivar/Landrace
42	ICC13077	*Kabuli*	India	Traditional cultivar/Landrace
43	ICC15264	*Kabuli*	Iran	Traditional cultivar/Landrace
44	ICC7308	*Kabuli*	Peru	Traditional cultivar/Landrace
45	ICC15435	*Kabuli*	Morocco	Traditional cultivar/Landrace
46	ICC7295	*Kabuli*	Tunisia	Traditional cultivar/Landrace
47	ICC12328	*Kabuli*	Cyprus	Traditional cultivar/Landrace
48	ICC14446	*Kabuli*	Italy	Traditional cultivar/Landrace
49	ICC10755	*Kabuli*	Turkey	Traditional cultivar/Landrace
50	ICC14190	*Kabuli*	India	Traditional cultivar/Landrace
51	ICC8042	*Kabuli*	Iran	Traditional cultivar/Landrace

Sl. No.	Accession Numbers	Cultivar types	Geographical origin	Biological status
52	ICC8261	*Kabuli*	Turkey	Traditional cultivar/Landrace
53	ICC15333	*Kabuli*	Iran	Traditional cultivar/Landrace
54	ICC15512	*Kabuli*	Morocco	Traditional cultivar/Landrace
55	ICC10884	*Kabuli*	Ethiopia	Traditional cultivar/Landrace
56	ICC15551	*Kabuli*	Australia	Traditional cultivar/Landrace
57	ICC15725	*Kabuli*	Syrian Arab Republic	Traditional cultivar/Landrace
58	ICC6204	*Kabuli*	Spain	Traditional cultivar/Landrace
59	ICC6210	*Kabuli*	Spain	Traditional cultivar/Landrace
60	ICC7654	*Kabuli*	Turkey	Traditional cultivar/Landrace
61	ICC11847	*Kabuli*	Chile	Traditional cultivar/Landrace
62	ICC11749	*Kabuli*	Chile	Traditional cultivar/Landrace
63	ICC14199	*Kabuli*	Mexico	Breeding material
64	ICC15518	*Kabuli*	Morocco	Traditional cultivar/Landrace
65	ICC16814	*Kabuli*	Portugal	Traditional cultivar/Landrace
66	ICC16811	*Kabuli*	Portugal	Traditional cultivar/Landrace
67	ICC6253	*Kabuli*	Morocco	Traditional cultivar/Landrace
68	ICC11301	*Kabuli*	United States of America	Traditional cultivar/Landrace
69	ICC10749	*Kabuli*	Turkey	Traditional cultivar/Landrace
70	ICC14216	*Kabuli*	Mexico	Breeding/Research material
71	ICC14203	*Kabuli*	Mexico	Traditional cultivar/Landrace
72	ICC11742	*Kabuli*	Chile	Traditional cultivar/Landrace
73	ICC13821	*Kabuli*	Ethiopia	Traditional cultivar/Landrace
74	ICC14462	*Kabuli*	India	Traditional cultivar/Landrace
75	ICC14220	*Kabuli*	Kenya	Traditional cultivar/Landrace
76	ICC15944	*Kabuli*	United States of America	Advanced/Improved cultivar
77	ICC11303	*Kabuli*	Chile	Traditional cultivar/Landrace
78	ICC12034	*Kabuli*	Mexico	Advanced/Improved cultivar
79	ICC7346	*Kabuli*	Mexico	Breeding/Research material
80	ICC15994	*Kabuli*	Spain	Traditional cultivar/Landrace
81	ICC18591	*Kabuli*	Mexico	Traditional cultivar/Landrace
82	ICC8155	*Kabuli*	United States of America	Traditional cultivar/Landrace
83	ICCV92311	*Kabuli*	India	Released Cultivar/Variety
84	ICCV95334	*Kabuli*	Central India	Variety
85	PhuleG0515	*Kabuli*	Central India	Variety
86	ICCV96329	*Kabuli*	Southern India	Variety
87	IC296376	*Kabuli*	Northern India	Variety
88	ICC12968	*Kabuli*	Southern India	Variety
89	IC449069	*Kabuli*	Northern India	Variety
90	BGD1105	*Kabuli*	Northern India	Genetic stock
91	Annigeri	*Kabuli*	India	Released Cultivar
92	L550	*Kabuli*	India	Released Cultivar
93	ICC17160	*C. reticulatum*	Turkey	Wild
94	ILWC21	*C. reticulatum*	Turkey	Wild
95	ICC17159	*C. echinospermum*	Turkey	Wild
96	IG135418	*C. echinospermum*	Syrian Arab Republic	Wild
97	ICC17150	*C. judaicum*	Lebanon	Wild
98	ILWC50	*C. judaicum*	Syrian Arab Republic	Wild
99	IG136792	*C. bijugum*	Syrian Arab Republic	Syrian Arab Republic
100	ILWC240	*C. bijugum*	Turkey	Wild
101	ILWC22	*C.pinnatifidum*	Turkey	Wild
102	ILWC261	*C.pinnatifidum*	Syrian Arab Republic	Wild

**Table S2:** 44844 genome-wide high-quality SNPs identified using reference genome (*desi* and *kabuli*)- and *de novo*-based GBS assay in chickpea					
**S.N.**	**SNP IDs**	**Cultivars**	**Chromosomes/scaffolds**	**Physical positions (bp)**	**SNPs**
1	CakSNP1	*Kabuli*	Ca_Kabuli_Chr01	523	(G/C)
2	CakSNP2	*Kabuli*	Ca_Kabuli_Chr01	462	(G/C)
3	CakSNP3	*Kabuli*	Ca_Kabuli_Chr01	548	(G/C)
4	CakSNP4	*Kabuli*	Ca_Kabuli_Chr01	578	(G/A)
5	CakSNP5	*Kabuli*	Ca_Kabuli_Chr01	579	(T/C)
6	CakSNP6	*Kabuli*	Ca_Kabuli_Chr01	589	(A/C)
7	CakSNP7	*Kabuli*	Ca_Kabuli_Chr01	627	(C/A)
8	CakSNP8	*Kabuli*	Ca_Kabuli_Chr01	839	(A/T)
9	CakSNP9	*Kabuli*	Ca_Kabuli_Chr01	903	(G/T)
10	CakSNP10	*Kabuli*	Ca_Kabuli_Chr01	927	(C/A)
11	CakSNP11	*Kabuli*	Ca_Kabuli_Chr01	98930	(T/C)
12	CakSNP12	*Kabuli*	Ca_Kabuli_Chr01	98992	(G/T)
13	CakSNP13	*Kabuli*	Ca_Kabuli_Chr01	99242	(G/A)
14	CakSNP14	*Kabuli*	Ca_Kabuli_Chr01	108882	(C/A)
15	CakSNP15	*Kabuli*	Ca_Kabuli_Chr01	108984	(G/A)
16	CakSNP16	*Kabuli*	Ca_Kabuli_Chr01	108979	(T/G)
17	CakSNP17	*Kabuli*	Ca_Kabuli_Chr01	129394	(A/G)
18	CakSNP18	*Kabuli*	Ca_Kabuli_Chr01	129401	(A/G)
19	CakSNP19	*Kabuli*	Ca_Kabuli_Chr01	173669	(C/T)
20	CakSNP20	*Kabuli*	Ca_Kabuli_Chr01	202954	(G/T)
21	CakSNP21	*Kabuli*	Ca_Kabuli_Chr01	208244	(G/A)
22	CakSNP22	*Kabuli*	Ca_Kabuli_Chr01	214979	(T/A)
23	CakSNP23	*Kabuli*	Ca_Kabuli_Chr01	215066	(G/T)
24	CakSNP24	*Kabuli*	Ca_Kabuli_Chr01	215058	(T/C)
25	CakSNP25	*Kabuli*	Ca_Kabuli_Chr01	215053	(G/T)
26	CakSNP26	*Kabuli*	Ca_Kabuli_Chr01	215048	(C/T)
27	CakSNP27	*Kabuli*	Ca_Kabuli_Chr01	215029	(C/A)
28	CakSNP28	*Kabuli*	Ca_Kabuli_Chr01	215027	(T/C)
29	CakSNP29	*Kabuli*	Ca_Kabuli_Chr01	215006	(C/T)
30	CakSNP30	*Kabuli*	Ca_Kabuli_Chr01	215239	(C/G)
31	CakSNP31	*Kabuli*	Ca_Kabuli_Chr01	292962	(T/C)
32	CakSNP32	*Kabuli*	Ca_Kabuli_Chr01	292972	(G/A)
33	CakSNP33	*Kabuli*	Ca_Kabuli_Chr01	292998	(T/C)
34	CakSNP34	*Kabuli*	Ca_Kabuli_Chr01	347620	(A/C)
35	CakSNP35	*Kabuli*	Ca_Kabuli_Chr01	372530	(A/G)
36	CakSNP36	*Kabuli*	Ca_Kabuli_Chr01	406570	(A/G)
37	CakSNP37	*Kabuli*	Ca_Kabuli_Chr01	425149	(T/C)

S.N.	SNP IDs	Cultivars	Chromosomes/scaffolds	Physical positions (bp)	SNPs
38	CakSNP38	*Kabuli*	Ca_Kabuli_Chr01	425076	(A/G)
39	CakSNP39	*Kabuli*	Ca_Kabuli_Chr01	435259	(A/G)
40	CakSNP40	*Kabuli*	Ca_Kabuli_Chr01	435274	(G/T)
41	CakSNP41	*Kabuli*	Ca_Kabuli_Chr01	435280	(T/C)
42	CakSNP42	*Kabuli*	Ca_Kabuli_Chr01	435297	(G/A)
43	CakSNP43	*Kabuli*	Ca_Kabuli_Chr01	435304	(G/T)
44	CakSNP44	*Kabuli*	Ca_Kabuli_Chr01	435343	(C/A)
45	CakSNP45	*Kabuli*	Ca_Kabuli_Chr01	435338	(T/C)
46	CakSNP46	*Kabuli*	Ca_Kabuli_Chr01	435264	(G/T)
47	CakSNP47	*Kabuli*	Ca_Kabuli_Chr01	441283	(G/A)
48	CakSNP48	*Kabuli*	Ca_Kabuli_Chr01	441286	(C/T)
49	CakSNP49	*Kabuli*	Ca_Kabuli_Chr01	441288	(T/A)
50	CakSNP50	*Kabuli*	Ca_Kabuli_Chr01	441343	(T/A)
51	CakSNP51	*Kabuli*	Ca_Kabuli_Chr01	446409	(T/C)
52	CakSNP52	*Kabuli*	Ca_Kabuli_Chr01	446443	(A/G)
53	CakSNP53	*Kabuli*	Ca_Kabuli_Chr01	446484	(C/T)
54	CakSNP54	*Kabuli*	Ca_Kabuli_Chr01	446558	(G/T)
55	CakSNP55	*Kabuli*	Ca_Kabuli_Chr01	459968	(T/G)
56	CakSNP56	*Kabuli*	Ca_Kabuli_Chr01	464039	(A/C)
57	CakSNP57	*Kabuli*	Ca_Kabuli_Chr01	464040	(G/C)
58	CakSNP58	*Kabuli*	Ca_Kabuli_Chr01	464043	(C/G)
59	CakSNP59	*Kabuli*	Ca_Kabuli_Chr01	471007	(C/T)
60	CakSNP60	*Kabuli*	Ca_Kabuli_Chr01	471037	(C/T)
61	CakSNP61	*Kabuli*	Ca_Kabuli_Chr01	527271	(T/G)
62	CakSNP62	*Kabuli*	Ca_Kabuli_Chr01	527321	(C/T)
63	CakSNP63	*Kabuli*	Ca_Kabuli_Chr01	554581	(G/T)
64	CakSNP64	*Kabuli*	Ca_Kabuli_Chr01	554677	(G/A)
65	CakSNP65	*Kabuli*	Ca_Kabuli_Chr01	571020	(T/G)
66	CakSNP66	*Kabuli*	Ca_Kabuli_Chr01	591609	(T/C)
67	CakSNP67	*Kabuli*	Ca_Kabuli_Chr01	609709	(A/G)
68	CakSNP68	*Kabuli*	Ca_Kabuli_Chr01	609671	(C/T)
69	CakSNP69	*Kabuli*	Ca_Kabuli_Chr01	609669	(C/G)
70	CakSNP70	*Kabuli*	Ca_Kabuli_Chr01	644318	(C/T)
71	CakSNP71	*Kabuli*	Ca_Kabuli_Chr01	644353	(G/A)
72	CakSNP72	*Kabuli*	Ca_Kabuli_Chr01	644359	(T/A)
73	CakSNP73	*Kabuli*	Ca_Kabuli_Chr01	644440	(T/C)
74	CakSNP74	*Kabuli*	Ca_Kabuli_Chr01	644425	(G/A)
75	CakSNP75	*Kabuli*	Ca_Kabuli_Chr01	661882	(A/G)

S.N.	SNP IDs	Cultivars	Chromosomes/scaffolds	Physical positions (bp)	SNPs
76	CakSNP76	*Kabuli*	Ca_Kabuli_Chr01	662341	(A/C)
77	CakSNP77	*Kabuli*	Ca_Kabuli_Chr01	662489	(T/A)
78	CakSNP78	*Kabuli*	Ca_Kabuli_Chr01	671420	(C/A)
79	CakSNP79	*Kabuli*	Ca_Kabuli_Chr01	671410	(T/C)
80	CakSNP80	*Kabuli*	Ca_Kabuli_Chr01	671391	(T/C)
81	CakSNP81	*Kabuli*	Ca_Kabuli_Chr01	671389	(A/G)
82	CakSNP82	*Kabuli*	Ca_Kabuli_Chr01	671388	(A/T)
83	CakSNP83	*Kabuli*	Ca_Kabuli_Chr01	690156	(C/A)
84	CakSNP84	*Kabuli*	Ca_Kabuli_Chr01	690431	(G/A)
85	CakSNP85	*Kabuli*	Ca_Kabuli_Chr01	691102	(A/T)
86	CakSNP86	*Kabuli*	Ca_Kabuli_Chr01	695962	(A/C)
87	CakSNP87	*Kabuli*	Ca_Kabuli_Chr01	712110	(T/C)
88	CakSNP88	*Kabuli*	Ca_Kabuli_Chr01	712073	(C/T)
89	CakSNP89	*Kabuli*	Ca_Kabuli_Chr01	741514	(G/T)
90	CakSNP90	*Kabuli*	Ca_Kabuli_Chr01	741535	(C/T)
91	CakSNP91	*Kabuli*	Ca_Kabuli_Chr01	741565	(T/C)
92	CakSNP92	*Kabuli*	Ca_Kabuli_Chr01	741549	(C/T)
93	CakSNP93	*Kabuli*	Ca_Kabuli_Chr01	741980	(C/G)
94	CakSNP94	*Kabuli*	Ca_Kabuli_Chr01	742043	(G/A)
95	CakSNP95	*Kabuli*	Ca_Kabuli_Chr01	742026	(T/G)
96	CakSNP96	*Kabuli*	Ca_Kabuli_Chr01	750693	(C/T)
97	CakSNP97	*Kabuli*	Ca_Kabuli_Chr01	777273	(C/T)
98	CakSNP98	*Kabuli*	Ca_Kabuli_Chr01	777240	(G/A)
99	CakSNP99	*Kabuli*	Ca_Kabuli_Chr01	790004	(A/C)
100	CakSNP100	*Kabuli*	Ca_Kabuli_Chr01	790000	(C/T)
101	CakSNP101	*Kabuli*	Ca_Kabuli_Chr01	790102	(G/T)
102	CakSNP102	*Kabuli*	Ca_Kabuli_Chr01	864205	(T/C)
103	CakSNP103	*Kabuli*	Ca_Kabuli_Chr01	864382	(T/C)
104	CakSNP104	*Kabuli*	Ca_Kabuli_Chr01	864381	(G/A)
105	CakSNP105	*Kabuli*	Ca_Kabuli_Chr01	864351	(T/C)
106	CakSNP106	*Kabuli*	Ca_Kabuli_Chr01	866872	(T/C)
107	CakSNP107	*Kabuli*	Ca_Kabuli_Chr01	872443	(G/T)
108	CakSNP108	*Kabuli*	Ca_Kabuli_Chr01	872452	(C/T)
109	CakSNP109	*Kabuli*	Ca_Kabuli_Chr01	876616	(T/C)
110	CakSNP110	*Kabuli*	Ca_Kabuli_Chr01	876633	(T/A)
111	CakSNP111	*Kabuli*	Ca_Kabuli_Chr01	881543	(C/A)
112	CakSNP112	*Kabuli*	Ca_Kabuli_Chr01	881673	(G/A)
113	CakSNP113	*Kabuli*	Ca_Kabuli_Chr01	881703	(G/T)

S.N.	SNP IDs	Cultivars	Chromosomes/scaffolds	Physical positions (bp)	SNPs
114	CakSNP114	*Kabuli*	Ca_Kabuli_Chr01	890949	(G/A)
115	CakSNP115	*Kabuli*	Ca_Kabuli_Chr01	890948	(C/T)
116	CakSNP116	*Kabuli*	Ca_Kabuli_Chr01	910486	(T/A)
117	CakSNP117	*Kabuli*	Ca_Kabuli_Chr01	970315	(A/T)
118	CakSNP118	*Kabuli*	Ca_Kabuli_Chr01	996439	(T/C)
119	CakSNP119	*Kabuli*	Ca_Kabuli_Chr01	1007698	(A/G)
120	CakSNP120	*Kabuli*	Ca_Kabuli_Chr01	1008895	(A/G)
121	CakSNP121	*Kabuli*	Ca_Kabuli_Chr01	1008971	(C/G)
122	CakSNP122	*Kabuli*	Ca_Kabuli_Chr01	1009058	(A/C)
123	CakSNP123	*Kabuli*	Ca_Kabuli_Chr01	1018966	(C/G)
124	CakSNP124	*Kabuli*	Ca_Kabuli_Chr01	1020800	(A/G)
125	CakSNP125	*Kabuli*	Ca_Kabuli_Chr01	1023550	(C/T)
126	CakSNP126	*Kabuli*	Ca_Kabuli_Chr01	1023538	(T/C)
127	CakSNP127	*Kabuli*	Ca_Kabuli_Chr01	1032734	(A/T)
128	CakSNP128	*Kabuli*	Ca_Kabuli_Chr01	1059209	(A/G)
129	CakSNP129	*Kabuli*	Ca_Kabuli_Chr01	1059311	(A/G)
130	CakSNP130	*Kabuli*	Ca_Kabuli_Chr01	1083274	(T/C)
131	CakSNP131	*Kabuli*	Ca_Kabuli_Chr01	1083388	(G/A)
132	CakSNP132	*Kabuli*	Ca_Kabuli_Chr01	1083403	(C/T)
133	CakSNP133	*Kabuli*	Ca_Kabuli_Chr01	1085228	(A/C)
134	CakSNP134	*Kabuli*	Ca_Kabuli_Chr01	1085230	(T/G)
135	CakSNP135	*Kabuli*	Ca_Kabuli_Chr01	1085238	(A/G)
136	CakSNP136	*Kabuli*	Ca_Kabuli_Chr01	1085254	(A/C)
137	CakSNP137	*Kabuli*	Ca_Kabuli_Chr01	1085263	(A/C)
138	CakSNP138	*Kabuli*	Ca_Kabuli_Chr01	1085293	(G/A)
139	CakSNP139	*Kabuli*	Ca_Kabuli_Chr01	1085348	(G/A)
140	CakSNP140	*Kabuli*	Ca_Kabuli_Chr01	1091548	(T/A)
141	CakSNP141	*Kabuli*	Ca_Kabuli_Chr01	1091534	(A/C)
142	CakSNP142	*Kabuli*	Ca_Kabuli_Chr01	1091504	(C/T)
143	CakSNP143	*Kabuli*	Ca_Kabuli_Chr01	1098703	(C/G)
144	CakSNP144	*Kabuli*	Ca_Kabuli_Chr01	1098789	(A/G)
145	CakSNP145	*Kabuli*	Ca_Kabuli_Chr01	1115668	(C/T)
146	CakSNP146	*Kabuli*	Ca_Kabuli_Chr01	1115809	(A/G)
147	CakSNP147	*Kabuli*	Ca_Kabuli_Chr01	1116043	(C/T)
148	CakSNP148	*Kabuli*	Ca_Kabuli_Chr01	1140313	(A/C)
149	CakSNP149	*Kabuli*	Ca_Kabuli_Chr01	1140405	(A/T)
150	CakSNP150	*Kabuli*	Ca_Kabuli_Chr01	1150973	(C/T)
151	CakSNP151	*Kabuli*	Ca_Kabuli_Chr01	1159383	(C/A)

S.N.	SNP IDs	Cultivars	Chromosomes/scaffolds	Physical positions (bp)	SNPs
152	CakSNP152	*Kabuli*	Ca_Kabuli_Chr01	1159411	(T/G)
153	CakSNP153	*Kabuli*	Ca_Kabuli_Chr01	1215024	(T/C)
154	CakSNP154	*Kabuli*	Ca_Kabuli_Chr01	1269212	(C/G)
155	CakSNP155	*Kabuli*	Ca_Kabuli_Chr01	1269180	(C/A)
156	CakSNP156	*Kabuli*	Ca_Kabuli_Chr01	1278884	(G/T)
157	CakSNP157	*Kabuli*	Ca_Kabuli_Chr01	1278997	(T/A)
158	CakSNP158	*Kabuli*	Ca_Kabuli_Chr01	1295527	(T/G)
159	CakSNP159	*Kabuli*	Ca_Kabuli_Chr01	1335518	(A/C)
160	CakSNP160	*Kabuli*	Ca_Kabuli_Chr01	1385553	(C/A)
161	CakSNP161	*Kabuli*	Ca_Kabuli_Chr01	1385554	(G/T)
162	CakSNP162	*Kabuli*	Ca_Kabuli_Chr01	1390073	(A/G)
163	CakSNP163	*Kabuli*	Ca_Kabuli_Chr01	1411348	(C/T)
164	CakSNP164	*Kabuli*	Ca_Kabuli_Chr01	1411351	(A/C)
165	CakSNP165	*Kabuli*	Ca_Kabuli_Chr01	1411501	(C/T)
166	CakSNP166	*Kabuli*	Ca_Kabuli_Chr01	1411498	(T/A)
167	CakSNP167	*Kabuli*	Ca_Kabuli_Chr01	1411495	(A/G)
168	CakSNP168	*Kabuli*	Ca_Kabuli_Chr01	1411490	(T/C)
169	CakSNP169	*Kabuli*	Ca_Kabuli_Chr01	1428222	(C/G)
170	CakSNP170	*Kabuli*	Ca_Kabuli_Chr01	1428251	(T/G)
171	CakSNP171	*Kabuli*	Ca_Kabuli_Chr01	1428264	(T/G)
172	CakSNP172	*Kabuli*	Ca_Kabuli_Chr01	1434121	(A/G)
173	CakSNP173	*Kabuli*	Ca_Kabuli_Chr01	1434883	(A/G)
174	CakSNP174	*Kabuli*	Ca_Kabuli_Chr01	1434875	(A/G)
175	CakSNP175	*Kabuli*	Ca_Kabuli_Chr01	1449848	(G/A)
176	CakSNP176	*Kabuli*	Ca_Kabuli_Chr01	1449913	(T/G)
177	CakSNP177	*Kabuli*	Ca_Kabuli_Chr01	1449949	(G/A)
178	CakSNP178	*Kabuli*	Ca_Kabuli_Chr01	1449980	(A/G)
179	CakSNP179	*Kabuli*	Ca_Kabuli_Chr01	1461574	(T/A)
180	CakSNP180	*Kabuli*	Ca_Kabuli_Chr01	1461600	(T/C)
181	CakSNP181	*Kabuli*	Ca_Kabuli_Chr01	1467056	(A/G)
182	CakSNP182	*Kabuli*	Ca_Kabuli_Chr01	1527513	(A/G)
183	CakSNP183	*Kabuli*	Ca_Kabuli_Chr01	1528325	(G/A)
184	CakSNP184	*Kabuli*	Ca_Kabuli_Chr01	1533723	(G/A)
185	CakSNP185	*Kabuli*	Ca_Kabuli_Chr01	1546679	(G/C)
186	CakSNP186	*Kabuli*	Ca_Kabuli_Chr01	1599821	(A/G)
187	CakSNP187	*Kabuli*	Ca_Kabuli_Chr01	1654823	(C/T)
188	CakSNP188	*Kabuli*	Ca_Kabuli_Chr01	1654855	(A/C)
189	CakSNP189	*Kabuli*	Ca_Kabuli_Chr01	1655018	(G/A)

S.N.	SNP IDs	Cultivars	Chromosomes/scaffolds	Physical positions (bp)	SNPs
190	CakSNP190	*Kabuli*	Ca_Kabuli_Chr01	1655012	(G/T)
191	CakSNP191	*Kabuli*	Ca_Kabuli_Chr01	1693010	(T/A)
192	CakSNP192	*Kabuli*	Ca_Kabuli_Chr01	1693013	(C/T)
193	CakSNP193	*Kabuli*	Ca_Kabuli_Chr01	1693022	(T/C)
194	CakSNP194	*Kabuli*	Ca_Kabuli_Chr01	1693023	(T/C)
195	CakSNP195	*Kabuli*	Ca_Kabuli_Chr01	1693031	(C/A)
196	CakSNP196	*Kabuli*	Ca_Kabuli_Chr01	1738179	(G/C)
197	CakSNP197	*Kabuli*	Ca_Kabuli_Chr01	1772813	(T/C)
198	CakSNP198	*Kabuli*	Ca_Kabuli_Chr01	1782760	(G/A)
199	CakSNP199	*Kabuli*	Ca_Kabuli_Chr01	1782793	(G/T)
200	CakSNP200	*Kabuli*	Ca_Kabuli_Chr01	1782808	(G/C)
201	CakSNP201	*Kabuli*	Ca_Kabuli_Chr01	1782953	(G/A)
202	CakSNP202	*Kabuli*	Ca_Kabuli_Chr01	1782948	(T/C)
203	CakSNP203	*Kabuli*	Ca_Kabuli_Chr01	1782930	(G/A)
204	CakSNP204	*Kabuli*	Ca_Kabuli_Chr01	1782929	(C/T)
205	CakSNP205	*Kabuli*	Ca_Kabuli_Chr01	1783207	(G/A)
206	CakSNP206	*Kabuli*	Ca_Kabuli_Chr01	1783277	(G/A)
207	CakSNP207	*Kabuli*	Ca_Kabuli_Chr01	1783280	(A/C)
208	CakSNP208	*Kabuli*	Ca_Kabuli_Chr01	1783342	(A/T)
209	CakSNP209	*Kabuli*	Ca_Kabuli_Chr01	1783923	(C/A)
210	CakSNP210	*Kabuli*	Ca_Kabuli_Chr01	1784006	(T/C)
211	CakSNP211	*Kabuli*	Ca_Kabuli_Chr01	1788958	(T/A)
212	CakSNP212	*Kabuli*	Ca_Kabuli_Chr01	1789087	(T/C)
213	CakSNP213	*Kabuli*	Ca_Kabuli_Chr01	1802992	(C/G)
214	CakSNP214	*Kabuli*	Ca_Kabuli_Chr01	1858073	(C/T)
215	CakSNP215	*Kabuli*	Ca_Kabuli_Chr01	1858078	(T/C)
216	CakSNP216	*Kabuli*	Ca_Kabuli_Chr01	1858084	(C/T)
217	CakSNP217	*Kabuli*	Ca_Kabuli_Chr01	1858132	(T/A)
218	CakSNP218	*Kabuli*	Ca_Kabuli_Chr01	1858156	(C/T)
219	CakSNP219	*Kabuli*	Ca_Kabuli_Chr01	1858177	(T/C)
220	CakSNP220	*Kabuli*	Ca_Kabuli_Chr01	1858159	(A/G)
221	CakSNP221	*Kabuli*	Ca_Kabuli_Chr01	1858509	(C/T)
222	CakSNP222	*Kabuli*	Ca_Kabuli_Chr01	1858608	(T/A)
223	CakSNP223	*Kabuli*	Ca_Kabuli_Chr01	1858600	(G/A)
224	CakSNP224	*Kabuli*	Ca_Kabuli_Chr01	1859789	(C/T)
225	CakSNP225	*Kabuli*	Ca_Kabuli_Chr01	1859857	(A/G)
226	CakSNP226	*Kabuli*	Ca_Kabuli_Chr01	1859914	(G/A)
227	CakSNP227	*Kabuli*	Ca_Kabuli_Chr01	1859902	(G/A)

S.N.	SNP IDs	Cultivars	Chromosomes/scaffolds	Physical positions (bp)	SNPs
228	CakSNP228	*Kabuli*	Ca_Kabuli_Chr01	1861696	(C/T)
229	CakSNP229	*Kabuli*	Ca_Kabuli_Chr01	1861707	(T/G)
230	CakSNP230	*Kabuli*	Ca_Kabuli_Chr01	1861722	(T/A)
231	CakSNP231	*Kabuli*	Ca_Kabuli_Chr01	1861729	(T/C)
232	CakSNP232	*Kabuli*	Ca_Kabuli_Chr01	1861837	(T/G)
233	CakSNP233	*Kabuli*	Ca_Kabuli_Chr01	1904233	(T/C)
234	CakSNP234	*Kabuli*	Ca_Kabuli_Chr01	1904230	(G/T)
235	CakSNP235	*Kabuli*	Ca_Kabuli_Chr01	1904365	(G/A)
236	CakSNP236	*Kabuli*	Ca_Kabuli_Chr01	1904391	(A/G)
237	CakSNP237	*Kabuli*	Ca_Kabuli_Chr01	1935729	(A/G)
238	CakSNP238	*Kabuli*	Ca_Kabuli_Chr01	1962876	(A/G)
239	CakSNP239	*Kabuli*	Ca_Kabuli_Chr01	1968828	(G/A)
240	CakSNP240	*Kabuli*	Ca_Kabuli_Chr01	2002844	(G/A)
241	CakSNP241	*Kabuli*	Ca_Kabuli_Chr01	2002922	(G/T)
242	CakSNP242	*Kabuli*	Ca_Kabuli_Chr01	2040943	(C/T)
243	CakSNP243	*Kabuli*	Ca_Kabuli_Chr01	2040946	(G/T)
244	CakSNP244	*Kabuli*	Ca_Kabuli_Chr01	2041027	(C/G)
245	CakSNP245	*Kabuli*	Ca_Kabuli_Chr01	2041190	(A/C)
246	CakSNP246	*Kabuli*	Ca_Kabuli_Chr01	2041180	(T/C)
247	CakSNP247	*Kabuli*	Ca_Kabuli_Chr01	2041178	(A/C)
248	CakSNP248	*Kabuli*	Ca_Kabuli_Chr01	2041174	(C/T)
249	CakSNP249	*Kabuli*	Ca_Kabuli_Chr01	2041154	(G/C)
250	CakSNP250	*Kabuli*	Ca_Kabuli_Chr01	2041132	(C/T)
251	CakSNP251	*Kabuli*	Ca_Kabuli_Chr01	2042071	(C/G)
252	CakSNP252	*Kabuli*	Ca_Kabuli_Chr01	2042185	(T/C)
253	CakSNP253	*Kabuli*	Ca_Kabuli_Chr01	2042152	(G/C)
254	CakSNP254	*Kabuli*	Ca_Kabuli_Chr01	2042149	(G/T)
255	CakSNP255	*Kabuli*	Ca_Kabuli_Chr01	2044338	(G/A)
256	CakSNP256	*Kabuli*	Ca_Kabuli_Chr01	2044304	(A/C)
257	CakSNP257	*Kabuli*	Ca_Kabuli_Chr01	2044289	(A/G)
258	CakSNP258	*Kabuli*	Ca_Kabuli_Chr01	2044263	(A/C)
259	CakSNP259	*Kabuli*	Ca_Kabuli_Chr01	2044359	(C/T)
260	CakSNP260	*Kabuli*	Ca_Kabuli_Chr01	2044374	(C/G)
261	CakSNP261	*Kabuli*	Ca_Kabuli_Chr01	2044380	(G/C)
262	CakSNP262	*Kabuli*	Ca_Kabuli_Chr01	2044408	(A/C)
263	CakSNP263	*Kabuli*	Ca_Kabuli_Chr01	2044414	(G/A)
264	CakSNP264	*Kabuli*	Ca_Kabuli_Chr01	2044474	(A/G)
265	CakSNP265	*Kabuli*	Ca_Kabuli_Chr01	2050232	(A/T)

S.N.	SNP IDs	Cultivars	Chromosomes/scaffolds	Physical positions (bp)	SNPs
266	CakSNP266	*Kabuli*	Ca_Kabuli_Chr01	2050187	(C/G)
267	CakSNP267	*Kabuli*	Ca_Kabuli_Chr01	2050479	(A/C)
268	CakSNP268	*Kabuli*	Ca_Kabuli_Chr01	2050469	(T/C)
269	CakSNP269	*Kabuli*	Ca_Kabuli_Chr01	2050467	(A/C)
270	CakSNP270	*Kabuli*	Ca_Kabuli_Chr01	2050465	(A/G)
271	CakSNP271	*Kabuli*	Ca_Kabuli_Chr01	2050463	(C/T)
272	CakSNP272	*Kabuli*	Ca_Kabuli_Chr01	2050443	(G/C)
273	CakSNP273	*Kabuli*	Ca_Kabuli_Chr01	2050421	(C/T)
274	CakSNP274	*Kabuli*	Ca_Kabuli_Chr01	2051360	(A/G)
275	CakSNP275	*Kabuli*	Ca_Kabuli_Chr01	2051375	(G/T)
276	CakSNP276	*Kabuli*	Ca_Kabuli_Chr01	2051379	(C/T)
277	CakSNP277	*Kabuli*	Ca_Kabuli_Chr01	2053732	(C/T)
278	CakSNP278	*Kabuli*	Ca_Kabuli_Chr01	2053744	(C/A)
279	CakSNP279	*Kabuli*	Ca_Kabuli_Chr01	2053749	(T/A)
280	CakSNP280	*Kabuli*	Ca_Kabuli_Chr01	2053756	(G/T)
281	CakSNP281	*Kabuli*	Ca_Kabuli_Chr01	2053757	(C/T)
282	CakSNP282	*Kabuli*	Ca_Kabuli_Chr01	2053758	(A/G)
283	CakSNP283	*Kabuli*	Ca_Kabuli_Chr01	2053784	(C/A)
284	CakSNP284	*Kabuli*	Ca_Kabuli_Chr01	2053788	(A/G)
285	CakSNP285	*Kabuli*	Ca_Kabuli_Chr01	2053790	(G/A)
286	CakSNP286	*Kabuli*	Ca_Kabuli_Chr01	2053808	(G/T)
287	CakSNP287	*Kabuli*	Ca_Kabuli_Chr01	2053856	(T/G)
288	CakSNP288	*Kabuli*	Ca_Kabuli_Chr01	2053843	(T/A)
289	CakSNP289	*Kabuli*	Ca_Kabuli_Chr01	2053841	(C/A)
290	CakSNP290	*Kabuli*	Ca_Kabuli_Chr01	2053840	(A/C)
291	CakSNP291	*Kabuli*	Ca_Kabuli_Chr01	2053833	(T/G)
292	CakSNP292	*Kabuli*	Ca_Kabuli_Chr01	2053830	(A/G)
293	CakSNP293	*Kabuli*	Ca_Kabuli_Chr01	2053819	(C/A)
294	CakSNP294	*Kabuli*	Ca_Kabuli_Chr01	2053818	(G/C)
295	CakSNP295	*Kabuli*	Ca_Kabuli_Chr01	2071456	(T/A)
296	CakSNP296	*Kabuli*	Ca_Kabuli_Chr01	2071448	(A/T)
297	CakSNP297	*Kabuli*	Ca_Kabuli_Chr01	2071424	(T/A)
298	CakSNP298	*Kabuli*	Ca_Kabuli_Chr01	2071708	(C/G)
299	CakSNP299	*Kabuli*	Ca_Kabuli_Chr01	2071703	(A/C)
300	CakSNP300	*Kabuli*	Ca_Kabuli_Chr01	2071699	(T/C)
301	CakSNP301	*Kabuli*	Ca_Kabuli_Chr01	2071693	(T/C)
302	CakSNP302	*Kabuli*	Ca_Kabuli_Chr01	2071691	(A/C)
303	CakSNP303	*Kabuli*	Ca_Kabuli_Chr01	2071689	(A/G)

S.N.	SNP IDs	Cultivars	Chromosomes/scaffolds	Physical positions (bp)	SNPs
304	CakSNP304	*Kabuli*	Ca_Kabuli_Chr01	2071687	(C/T)
305	CakSNP305	*Kabuli*	Ca_Kabuli_Chr01	2071667	(G/C)
306	CakSNP306	*Kabuli*	Ca_Kabuli_Chr01	2071645	(C/T)
307	CakSNP307	*Kabuli*	Ca_Kabuli_Chr01	2072613	(G/A)
308	CakSNP308	*Kabuli*	Ca_Kabuli_Chr01	2072627	(A/T)
309	CakSNP309	*Kabuli*	Ca_Kabuli_Chr01	2074998	(A/C)
310	CakSNP310	*Kabuli*	Ca_Kabuli_Chr01	2075087	(A/T)
311	CakSNP311	*Kabuli*	Ca_Kabuli_Chr01	2075057	(G/T)
312	CakSNP312	*Kabuli*	Ca_Kabuli_Chr01	2075045	(C/A)
313	CakSNP313	*Kabuli*	Ca_Kabuli_Chr01	2075044	(A/G)
314	CakSNP314	*Kabuli*	Ca_Kabuli_Chr01	2075043	(C/A)
315	CakSNP315	*Kabuli*	Ca_Kabuli_Chr01	2075015	(G/A)
316	CakSNP316	*Kabuli*	Ca_Kabuli_Chr01	2075013	(A/G)
317	CakSNP317	*Kabuli*	Ca_Kabuli_Chr01	2075009	(C/A)
318	CakSNP318	*Kabuli*	Ca_Kabuli_Chr01	2082760	(C/G)
319	CakSNP319	*Kabuli*	Ca_Kabuli_Chr01	2082801	(G/A)
320	CakSNP320	*Kabuli*	Ca_Kabuli_Chr01	2083003	(G/C)
321	CakSNP321	*Kabuli*	Ca_Kabuli_Chr01	2083059	(A/T)
322	CakSNP322	*Kabuli*	Ca_Kabuli_Chr01	2126267	(G/A)
323	CakSNP323	*Kabuli*	Ca_Kabuli_Chr01	2148583	(T/G)
324	CakSNP324	*Kabuli*	Ca_Kabuli_Chr01	2170090	(G/C)
325	CakSNP325	*Kabuli*	Ca_Kabuli_Chr01	2170935	(A/C)
326	CakSNP326	*Kabuli*	Ca_Kabuli_Chr01	2172076	(T/C)
327	CakSNP327	*Kabuli*	Ca_Kabuli_Chr01	2172113	(G/A)
328	CakSNP328	*Kabuli*	Ca_Kabuli_Chr01	2172143	(T/G)
329	CakSNP329	*Kabuli*	Ca_Kabuli_Chr01	2172149	(G/A)
330	CakSNP330	*Kabuli*	Ca_Kabuli_Chr01	2183544	(A/G)
331	CakSNP331	*Kabuli*	Ca_Kabuli_Chr01	2188472	(T/C)
332	CakSNP332	*Kabuli*	Ca_Kabuli_Chr01	2232724	(A/G)
333	CakSNP333	*Kabuli*	Ca_Kabuli_Chr01	2232764	(C/T)
334	CakSNP334	*Kabuli*	Ca_Kabuli_Chr01	2232779	(A/G)
335	CakSNP335	*Kabuli*	Ca_Kabuli_Chr01	2252270	(C/T)
336	CakSNP336	*Kabuli*	Ca_Kabuli_Chr01	2252320	(G/T)
337	CakSNP337	*Kabuli*	Ca_Kabuli_Chr01	2252328	(C/A)
338	CakSNP338	*Kabuli*	Ca_Kabuli_Chr01	2252342	(C/T)
339	CakSNP339	*Kabuli*	Ca_Kabuli_Chr01	2252343	(G/T)
340	CakSNP340	*Kabuli*	Ca_Kabuli_Chr01	2252463	(T/C)
341	CakSNP341	*Kabuli*	Ca_Kabuli_Chr01	2252401	(A/G)

S.N.	SNP IDs	Cultivars	Chromosomes/scaffolds	Physical positions (bp)	SNPs
342	CakSNP342	*Kabuli*	Ca_Kabuli_Chr01	2252496	(C/A)
343	CakSNP343	*Kabuli*	Ca_Kabuli_Chr01	2252513	(C/T)
344	CakSNP344	*Kabuli*	Ca_Kabuli_Chr01	2269319	(T/G)
345	CakSNP345	*Kabuli*	Ca_Kabuli_Chr01	2269489	(A/C)
346	CakSNP346	*Kabuli*	Ca_Kabuli_Chr01	2277459	(G/A)
347	CakSNP347	*Kabuli*	Ca_Kabuli_Chr01	2285879	(G/A)
348	CakSNP348	*Kabuli*	Ca_Kabuli_Chr01	2287408	(A/T)
349	CakSNP349	*Kabuli*	Ca_Kabuli_Chr01	2287478	(A/T)
350	CakSNP350	*Kabuli*	Ca_Kabuli_Chr01	2366413	(T/C)
351	CakSNP351	*Kabuli*	Ca_Kabuli_Chr01	2366409	(T/A)
352	CakSNP352	*Kabuli*	Ca_Kabuli_Chr01	2373166	(A/G)
353	CakSNP353	*Kabuli*	Ca_Kabuli_Chr01	2373862	(G/A)
354	CakSNP354	*Kabuli*	Ca_Kabuli_Chr01	2373910	(C/T)
355	CakSNP355	*Kabuli*	Ca_Kabuli_Chr01	2373954	(G/T)
356	CakSNP356	*Kabuli*	Ca_Kabuli_Chr01	2395842	(A/C)
357	CakSNP357	*Kabuli*	Ca_Kabuli_Chr01	2400799	(T/G)
358	CakSNP358	*Kabuli*	Ca_Kabuli_Chr01	2400892	(C/T)
359	CakSNP359	*Kabuli*	Ca_Kabuli_Chr01	2428531	(A/G)
360	CakSNP360	*Kabuli*	Ca_Kabuli_Chr01	2439915	(A/G)
361	CakSNP361	*Kabuli*	Ca_Kabuli_Chr01	2439917	(G/A)
362	CakSNP362	*Kabuli*	Ca_Kabuli_Chr01	2473309	(T/C)
363	CakSNP363	*Kabuli*	Ca_Kabuli_Chr01	2513476	(T/A)
364	CakSNP364	*Kabuli*	Ca_Kabuli_Chr01	2513516	(T/A)
365	CakSNP365	*Kabuli*	Ca_Kabuli_Chr01	2513535	(T/C)
366	CakSNP366	*Kabuli*	Ca_Kabuli_Chr01	2513737	(T/C)
367	CakSNP367	*Kabuli*	Ca_Kabuli_Chr01	2513860	(G/A)
368	CakSNP368	*Kabuli*	Ca_Kabuli_Chr01	2513854	(G/A)
369	CakSNP369	*Kabuli*	Ca_Kabuli_Chr01	2513792	(A/G)
370	CakSNP370	*Kabuli*	Ca_Kabuli_Chr01	2513914	(T/C)
371	CakSNP371	*Kabuli*	Ca_Kabuli_Chr01	2542652	(G/A)
372	CakSNP372	*Kabuli*	Ca_Kabuli_Chr01	2649822	(G/C)
373	CakSNP373	*Kabuli*	Ca_Kabuli_Chr01	2649864	(C/A)
374	CakSNP374	*Kabuli*	Ca_Kabuli_Chr01	2649921	(T/C)
375	CakSNP375	*Kabuli*	Ca_Kabuli_Chr01	2649906	(C/T)
376	CakSNP376	*Kabuli*	Ca_Kabuli_Chr01	2649974	(A/C)
377	CakSNP377	*Kabuli*	Ca_Kabuli_Chr01	2671403	(G/T)
378	CakSNP378	*Kabuli*	Ca_Kabuli_Chr01	2694584	(T/C)
379	CakSNP379	*Kabuli*	Ca_Kabuli_Chr01	2745499	(C/A)

S.N.	SNP IDs	Cultivars	Chromosomes/scaffolds	Physical positions (bp)	SNPs
380	CakSNP380	*Kabuli*	Ca_Kabuli_Chr01	2745514	(A/C)
381	CakSNP381	*Kabuli*	Ca_Kabuli_Chr01	2745519	(G/C)
382	CakSNP382	*Kabuli*	Ca_Kabuli_Chr01	2745486	(C/T)
383	CakSNP383	*Kabuli*	Ca_Kabuli_Chr01	2754998	(C/T)
384	CakSNP384	*Kabuli*	Ca_Kabuli_Chr01	2755012	(C/T)
385	CakSNP385	*Kabuli*	Ca_Kabuli_Chr01	2755013	(T/A)
386	CakSNP386	*Kabuli*	Ca_Kabuli_Chr01	2755015	(T/C)
387	CakSNP387	*Kabuli*	Ca_Kabuli_Chr01	2755443	(T/A)
388	CakSNP388	*Kabuli*	Ca_Kabuli_Chr01	2755455	(G/A)
389	CakSNP389	*Kabuli*	Ca_Kabuli_Chr01	2755457	(C/T)
390	CakSNP390	*Kabuli*	Ca_Kabuli_Chr01	2755459	(T/G)
391	CakSNP391	*Kabuli*	Ca_Kabuli_Chr01	2755465	(T/A)
392	CakSNP392	*Kabuli*	Ca_Kabuli_Chr01	2755466	(C/G)
393	CakSNP393	*Kabuli*	Ca_Kabuli_Chr01	2755470	(A/T)
394	CakSNP394	*Kabuli*	Ca_Kabuli_Chr01	2755471	(C/A)
395	CakSNP395	*Kabuli*	Ca_Kabuli_Chr01	2755489	(G/A)
396	CakSNP396	*Kabuli*	Ca_Kabuli_Chr01	2755494	(T/C)
397	CakSNP397	*Kabuli*	Ca_Kabuli_Chr01	2755496	(A/G)
398	CakSNP398	*Kabuli*	Ca_Kabuli_Chr01	2755613	(G/T)
399	CakSNP399	*Kabuli*	Ca_Kabuli_Chr01	2755604	(A/C)
400	CakSNP400	*Kabuli*	Ca_Kabuli_Chr01	2755596	(C/A)
401	CakSNP401	*Kabuli*	Ca_Kabuli_Chr01	2757711	(T/G)
402	CakSNP402	*Kabuli*	Ca_Kabuli_Chr01	2757696	(A/G)
403	CakSNP403	*Kabuli*	Ca_Kabuli_Chr01	2757693	(C/T)
404	CakSNP404	*Kabuli*	Ca_Kabuli_Chr01	2757678	(A/G)
405	CakSNP405	*Kabuli*	Ca_Kabuli_Chr01	2758781	(G/C)
406	CakSNP406	*Kabuli*	Ca_Kabuli_Chr01	2758759	(G/C)
407	CakSNP407	*Kabuli*	Ca_Kabuli_Chr01	2758750	(A/G)
408	CakSNP408	*Kabuli*	Ca_Kabuli_Chr01	2758746	(G/A)
409	CakSNP409	*Kabuli*	Ca_Kabuli_Chr01	2758739	(A/T)
410	CakSNP410	*Kabuli*	Ca_Kabuli_Chr01	2758738	(T/G)
411	CakSNP411	*Kabuli*	Ca_Kabuli_Chr01	2758859	(A/T)
412	CakSNP412	*Kabuli*	Ca_Kabuli_Chr01	2758835	(T/A)
413	CakSNP413	*Kabuli*	Ca_Kabuli_Chr01	2758827	(A/T)
414	CakSNP414	*Kabuli*	Ca_Kabuli_Chr01	2787006	(C/T)
415	CakSNP415	*Kabuli*	Ca_Kabuli_Chr01	2786994	(A/C)
416	CakSNP416	*Kabuli*	Ca_Kabuli_Chr01	2786989	(T/C)
417	CakSNP417	*Kabuli*	Ca_Kabuli_Chr01	2786976	(A/C)

S.N.	SNP IDs	Cultivars	Chromosomes/scaffolds	Physical positions (bp)	SNPs
418	CakSNP418	*Kabuli*	Ca_Kabuli_Chr01	2786974	(T/C)
419	CakSNP419	*Kabuli*	Ca_Kabuli_Chr01	2786970	(T/A)
420	CakSNP420	*Kabuli*	Ca_Kabuli_Chr01	2807605	(G/C)
421	CakSNP421	*Kabuli*	Ca_Kabuli_Chr01	2807616	(G/A)
422	CakSNP422	*Kabuli*	Ca_Kabuli_Chr01	2808197	(T/C)
423	CakSNP423	*Kabuli*	Ca_Kabuli_Chr01	2808170	(T/G)
424	CakSNP424	*Kabuli*	Ca_Kabuli_Chr01	2832792	(G/A)
425	CakSNP425	*Kabuli*	Ca_Kabuli_Chr01	2832969	(G/A)
426	CakSNP426	*Kabuli*	Ca_Kabuli_Chr01	2832939	(G/T)
427	CakSNP427	*Kabuli*	Ca_Kabuli_Chr01	2833448	(C/A)
428	CakSNP428	*Kabuli*	Ca_Kabuli_Chr01	2855919	(T/G)
429	CakSNP429	*Kabuli*	Ca_Kabuli_Chr01	2855926	(T/A)
430	CakSNP430	*Kabuli*	Ca_Kabuli_Chr01	2855927	(T/A)
431	CakSNP431	*Kabuli*	Ca_Kabuli_Chr01	2855941	(T/G)
432	CakSNP432	*Kabuli*	Ca_Kabuli_Chr01	2855962	(A/G)
433	CakSNP433	*Kabuli*	Ca_Kabuli_Chr01	2855969	(C/T)
434	CakSNP434	*Kabuli*	Ca_Kabuli_Chr01	2856003	(T/C)
435	CakSNP435	*Kabuli*	Ca_Kabuli_Chr01	2856045	(C/T)
436	CakSNP436	*Kabuli*	Ca_Kabuli_Chr01	2856079	(T/G)
437	CakSNP437	*Kabuli*	Ca_Kabuli_Chr01	2856094	(T/G)
438	CakSNP438	*Kabuli*	Ca_Kabuli_Chr01	2856120	(T/G)
439	CakSNP439	*Kabuli*	Ca_Kabuli_Chr01	2856534	(T/A)
440	CakSNP440	*Kabuli*	Ca_Kabuli_Chr01	2858263	(A/G)
441	CakSNP441	*Kabuli*	Ca_Kabuli_Chr01	2858278	(T/A)
442	CakSNP442	*Kabuli*	Ca_Kabuli_Chr01	2858287	(G/T)
443	CakSNP443	*Kabuli*	Ca_Kabuli_Chr01	2858299	(C/A)
444	CakSNP444	*Kabuli*	Ca_Kabuli_Chr01	2868942	(G/A)
445	CakSNP445	*Kabuli*	Ca_Kabuli_Chr01	2881315	(T/C)
446	CakSNP446	*Kabuli*	Ca_Kabuli_Chr01	2881321	(C/A)
447	CakSNP447	*Kabuli*	Ca_Kabuli_Chr01	2881323	(A/T)
448	CakSNP448	*Kabuli*	Ca_Kabuli_Chr01	2881327	(C/T)
449	CakSNP449	*Kabuli*	Ca_Kabuli_Chr01	2881356	(C/A)
450	CakSNP450	*Kabuli*	Ca_Kabuli_Chr01	2881357	(A/T)
451	CakSNP451	*Kabuli*	Ca_Kabuli_Chr01	2881422	(T/C)
452	CakSNP452	*Kabuli*	Ca_Kabuli_Chr01	2881389	(G/C)
453	CakSNP453	*Kabuli*	Ca_Kabuli_Chr01	2881386	(G/T)
454	CakSNP454	*Kabuli*	Ca_Kabuli_Chr01	2883625	(T/A)
455	CakSNP455	*Kabuli*	Ca_Kabuli_Chr01	2883634	(T/A)

S.N.	SNP IDs	Cultivars	Chromosomes/scaffolds	Physical positions (bp)	SNPs
456	CakSNP456	*Kabuli*	Ca_Kabuli_Chr01	2883635	(A/G)
457	CakSNP457	*Kabuli*	Ca_Kabuli_Chr01	2883660	(C/G)
458	CakSNP458	*Kabuli*	Ca_Kabuli_Chr01	2883666	(G/A)
459	CakSNP459	*Kabuli*	Ca_Kabuli_Chr01	2883684	(G/T)
460	CakSNP460	*Kabuli*	Ca_Kabuli_Chr01	2883717	(C/A)
461	CakSNP461	*Kabuli*	Ca_Kabuli_Chr01	2883716	(A/C)
462	CakSNP462	*Kabuli*	Ca_Kabuli_Chr01	2883709	(A/G)
463	CakSNP463	*Kabuli*	Ca_Kabuli_Chr01	2885508	(T/C)
464	CakSNP464	*Kabuli*	Ca_Kabuli_Chr01	2885525	(T/C)
465	CakSNP465	*Kabuli*	Ca_Kabuli_Chr01	2885531	(C/T)
466	CakSNP466	*Kabuli*	Ca_Kabuli_Chr01	2885626	(A/G)
467	CakSNP467	*Kabuli*	Ca_Kabuli_Chr01	2885673	(G/C)
468	CakSNP468	*Kabuli*	Ca_Kabuli_Chr01	2885892	(T/A)
469	CakSNP469	*Kabuli*	Ca_Kabuli_Chr01	2885906	(C/T)
470	CakSNP470	*Kabuli*	Ca_Kabuli_Chr01	2885923	(C/G)
471	CakSNP471	*Kabuli*	Ca_Kabuli_Chr01	2886025	(A/G)
472	CakSNP472	*Kabuli*	Ca_Kabuli_Chr01	2886020	(G/T)
473	CakSNP473	*Kabuli*	Ca_Kabuli_Chr01	2886015	(G/A)
474	CakSNP474	*Kabuli*	Ca_Kabuli_Chr01	2886005	(G/A)
475	CakSNP475	*Kabuli*	Ca_Kabuli_Chr01	2885997	(G/A)
476	CakSNP476	*Kabuli*	Ca_Kabuli_Chr01	2885988	(C/G)
477	CakSNP477	*Kabuli*	Ca_Kabuli_Chr01	2885987	(T/A)
478	CakSNP478	*Kabuli*	Ca_Kabuli_Chr01	2948211	(C/T)
479	CakSNP479	*Kabuli*	Ca_Kabuli_Chr01	3009417	(C/T)
480	CakSNP480	*Kabuli*	Ca_Kabuli_Chr01	3032061	(C/A)
481	CakSNP481	*Kabuli*	Ca_Kabuli_Chr01	3081723	(G/T)
482	CakSNP482	*Kabuli*	Ca_Kabuli_Chr01	3081851	(T/G)
483	CakSNP483	*Kabuli*	Ca_Kabuli_Chr01	3081850	(C/A)
484	CakSNP484	*Kabuli*	Ca_Kabuli_Chr01	3082280	(G/A)
485	CakSNP485	*Kabuli*	Ca_Kabuli_Chr01	3151007	(G/A)
486	CakSNP486	*Kabuli*	Ca_Kabuli_Chr01	3150986	(T/C)
487	CakSNP487	*Kabuli*	Ca_Kabuli_Chr01	3150964	(C/G)
488	CakSNP488	*Kabuli*	Ca_Kabuli_Chr01	3259114	(G/A)
489	CakSNP489	*Kabuli*	Ca_Kabuli_Chr01	3259117	(G/C)
490	CakSNP490	*Kabuli*	Ca_Kabuli_Chr01	3259177	(G/C)
491	CakSNP491	*Kabuli*	Ca_Kabuli_Chr01	3298327	(C/G)
492	CakSNP492	*Kabuli*	Ca_Kabuli_Chr01	3298783	(T/G)
493	CakSNP493	*Kabuli*	Ca_Kabuli_Chr01	3359148	(C/A)

S.N.	SNP IDs	Cultivars	Chromosomes/scaffolds	Physical positions (bp)	SNPs
494	CakSNP494	*Kabuli*	Ca_Kabuli_Chr01	3364952	(A/C)
495	CakSNP495	*Kabuli*	Ca_Kabuli_Chr01	3374375	(G/A)
496	CakSNP496	*Kabuli*	Ca_Kabuli_Chr01	3427555	(A/C)
497	CakSNP497	*Kabuli*	Ca_Kabuli_Chr01	3427649	(A/G)
498	CakSNP498	*Kabuli*	Ca_Kabuli_Chr01	3503059	(C/A)
499	CakSNP499	*Kabuli*	Ca_Kabuli_Chr01	3503153	(G/A)
500	CakSNP500	*Kabuli*	Ca_Kabuli_Chr01	3542263	(A/C)
501	CakSNP501	*Kabuli*	Ca_Kabuli_Chr01	3544948	(A/T)
502	CakSNP502	*Kabuli*	Ca_Kabuli_Chr01	3545011	(G/A)
503	CakSNP503	*Kabuli*	Ca_Kabuli_Chr01	3564342	(G/T)
504	CakSNP504	*Kabuli*	Ca_Kabuli_Chr01	3596241	(C/T)
505	CakSNP505	*Kabuli*	Ca_Kabuli_Chr01	3597842	(G/A)
506	CakSNP506	*Kabuli*	Ca_Kabuli_Chr01	3605582	(G/A)
507	CakSNP507	*Kabuli*	Ca_Kabuli_Chr01	3605583	(C/T)
508	CakSNP508	*Kabuli*	Ca_Kabuli_Chr01	3609580	(T/C)
509	CakSNP509	*Kabuli*	Ca_Kabuli_Chr01	3611231	(C/A)
510	CakSNP510	*Kabuli*	Ca_Kabuli_Chr01	3614966	(A/G)
511	CakSNP511	*Kabuli*	Ca_Kabuli_Chr01	3615037	(G/T)
512	CakSNP512	*Kabuli*	Ca_Kabuli_Chr01	3615064	(T/C)
513	CakSNP513	*Kabuli*	Ca_Kabuli_Chr01	3615052	(C/A)
514	CakSNP514	*Kabuli*	Ca_Kabuli_Chr01	3622757	(C/T)
515	CakSNP515	*Kabuli*	Ca_Kabuli_Chr01	3711498	(T/G)
516	CakSNP516	*Kabuli*	Ca_Kabuli_Chr01	3711525	(T/G)
517	CakSNP517	*Kabuli*	Ca_Kabuli_Chr01	3751865	(G/T)
518	CakSNP518	*Kabuli*	Ca_Kabuli_Chr01	3777223	(C/T)
519	CakSNP519	*Kabuli*	Ca_Kabuli_Chr01	3810931	(T/C)
520	CakSNP520	*Kabuli*	Ca_Kabuli_Chr01	3852035	(A/C)
521	CakSNP521	*Kabuli*	Ca_Kabuli_Chr01	3852476	(A/C)
522	CakSNP522	*Kabuli*	Ca_Kabuli_Chr01	3853408	(T/C)
523	CakSNP523	*Kabuli*	Ca_Kabuli_Chr01	3855229	(G/A)
524	CakSNP524	*Kabuli*	Ca_Kabuli_Chr01	3891319	(T/C)
525	CakSNP525	*Kabuli*	Ca_Kabuli_Chr01	3911547	(G/A)
526	CakSNP526	*Kabuli*	Ca_Kabuli_Chr01	3941250	(C/T)
527	CakSNP527	*Kabuli*	Ca_Kabuli_Chr01	3941399	(T/C)
528	CakSNP528	*Kabuli*	Ca_Kabuli_Chr01	3952819	(G/A)
529	CakSNP529	*Kabuli*	Ca_Kabuli_Chr01	3956031	(T/C)
530	CakSNP530	*Kabuli*	Ca_Kabuli_Chr01	3956028	(C/T)
531	CakSNP531	*Kabuli*	Ca_Kabuli_Chr01	3972671	(G/T)

S.N.	SNP IDs	Cultivars	Chromosomes/scaffolds	Physical positions (bp)	SNPs
532	CakSNP532	*Kabuli*	Ca_Kabuli_Chr01	4048854	(G/A)
533	CakSNP533	*Kabuli*	Ca_Kabuli_Chr01	4048875	(A/T)
534	CakSNP534	*Kabuli*	Ca_Kabuli_Chr01	4073941	(A/C)
535	CakSNP535	*Kabuli*	Ca_Kabuli_Chr01	4112929	(A/T)
536	CakSNP536	*Kabuli*	Ca_Kabuli_Chr01	4189333	(C/T)
537	CakSNP537	*Kabuli*	Ca_Kabuli_Chr01	4189297	(T/C)
538	CakSNP538	*Kabuli*	Ca_Kabuli_Chr01	4245058	(C/G)
539	CakSNP539	*Kabuli*	Ca_Kabuli_Chr01	4245073	(C/G)
540	CakSNP540	*Kabuli*	Ca_Kabuli_Chr01	4245176	(G/T)
541	CakSNP541	*Kabuli*	Ca_Kabuli_Chr01	4292981	(G/C)
542	CakSNP542	*Kabuli*	Ca_Kabuli_Chr01	4302669	(G/C)
543	CakSNP543	*Kabuli*	Ca_Kabuli_Chr01	4302770	(A/C)
544	CakSNP544	*Kabuli*	Ca_Kabuli_Chr01	4302751	(T/C)
545	CakSNP545	*Kabuli*	Ca_Kabuli_Chr01	4334299	(A/T)
546	CakSNP546	*Kabuli*	Ca_Kabuli_Chr01	4334336	(A/G)
547	CakSNP547	*Kabuli*	Ca_Kabuli_Chr01	4372257	(A/G)
548	CakSNP548	*Kabuli*	Ca_Kabuli_Chr01	4375710	(T/C)
549	CakSNP549	*Kabuli*	Ca_Kabuli_Chr01	4385002	(C/T)
550	CakSNP550	*Kabuli*	Ca_Kabuli_Chr01	4386114	(T/G)
551	CakSNP551	*Kabuli*	Ca_Kabuli_Chr01	4386111	(T/C)
552	CakSNP552	*Kabuli*	Ca_Kabuli_Chr01	4392680	(A/G)
553	CakSNP553	*Kabuli*	Ca_Kabuli_Chr01	4414410	(A/T)
554	CakSNP554	*Kabuli*	Ca_Kabuli_Chr01	4423984	(A/G)
555	CakSNP555	*Kabuli*	Ca_Kabuli_Chr01	4429044	(C/T)
556	CakSNP556	*Kabuli*	Ca_Kabuli_Chr01	4432569	(A/G)
557	CakSNP557	*Kabuli*	Ca_Kabuli_Chr01	4432549	(C/T)
558	CakSNP558	*Kabuli*	Ca_Kabuli_Chr01	4432545	(C/A)
559	CakSNP559	*Kabuli*	Ca_Kabuli_Chr01	4432541	(C/A)
560	CakSNP560	*Kabuli*	Ca_Kabuli_Chr01	4456662	(G/A)
561	CakSNP561	*Kabuli*	Ca_Kabuli_Chr01	4456654	(A/C)
562	CakSNP562	*Kabuli*	Ca_Kabuli_Chr01	4456648	(T/C)
563	CakSNP563	*Kabuli*	Ca_Kabuli_Chr01	4457588	(A/G)
564	CakSNP564	*Kabuli*	Ca_Kabuli_Chr01	4460112	(A/T)
565	CakSNP565	*Kabuli*	Ca_Kabuli_Chr01	4494270	(C/T)
566	CakSNP566	*Kabuli*	Ca_Kabuli_Chr01	4494467	(A/G)
567	CakSNP567	*Kabuli*	Ca_Kabuli_Chr01	4512566	(T/C)
568	CakSNP568	*Kabuli*	Ca_Kabuli_Chr01	4528117	(A/G)
569	CakSNP569	*Kabuli*	Ca_Kabuli_Chr01	4528184	(C/T)

S.N.	SNP IDs	Cultivars	Chromosomes/scaffolds	Physical positions (bp)	SNPs
570	CakSNP570	*Kabuli*	Ca_Kabuli_Chr01	4530304	(T/C)
571	CakSNP571	*Kabuli*	Ca_Kabuli_Chr01	4560792	(C/T)
572	CakSNP572	*Kabuli*	Ca_Kabuli_Chr01	4560920	(G/A)
573	CakSNP573	*Kabuli*	Ca_Kabuli_Chr01	4569669	(T/A)
574	CakSNP574	*Kabuli*	Ca_Kabuli_Chr01	4569670	(T/A)
575	CakSNP575	*Kabuli*	Ca_Kabuli_Chr01	4569690	(A/G)
576	CakSNP576	*Kabuli*	Ca_Kabuli_Chr01	4571615	(T/C)
577	CakSNP577	*Kabuli*	Ca_Kabuli_Chr01	4571540	(C/G)
578	CakSNP578	*Kabuli*	Ca_Kabuli_Chr01	4571539	(G/A)
579	CakSNP579	*Kabuli*	Ca_Kabuli_Chr01	4576888	(A/G)
580	CakSNP580	*Kabuli*	Ca_Kabuli_Chr01	4578967	(T/G)
581	CakSNP581	*Kabuli*	Ca_Kabuli_Chr01	4578999	(T/A)
582	CakSNP582	*Kabuli*	Ca_Kabuli_Chr01	4582189	(C/T)
583	CakSNP583	*Kabuli*	Ca_Kabuli_Chr01	4626099	(T/G)
584	CakSNP584	*Kabuli*	Ca_Kabuli_Chr01	4626098	(C/G)
585	CakSNP585	*Kabuli*	Ca_Kabuli_Chr01	4631467	(G/A)
586	CakSNP586	*Kabuli*	Ca_Kabuli_Chr01	4632188	(G/A)
587	CakSNP587	*Kabuli*	Ca_Kabuli_Chr01	4632965	(G/A)
588	CakSNP588	*Kabuli*	Ca_Kabuli_Chr01	4634413	(C/T)
589	CakSNP589	*Kabuli*	Ca_Kabuli_Chr01	4635347	(G/A)
590	CakSNP590	*Kabuli*	Ca_Kabuli_Chr01	4635302	(A/C)
591	CakSNP591	*Kabuli*	Ca_Kabuli_Chr01	4662564	(A/G)
592	CakSNP592	*Kabuli*	Ca_Kabuli_Chr01	4672439	(G/A)
593	CakSNP593	*Kabuli*	Ca_Kabuli_Chr01	4715563	(T/C)
594	CakSNP594	*Kabuli*	Ca_Kabuli_Chr01	4780330	(C/T)
595	CakSNP595	*Kabuli*	Ca_Kabuli_Chr01	4780345	(G/T)
596	CakSNP596	*Kabuli*	Ca_Kabuli_Chr01	4781362	(C/T)
597	CakSNP597	*Kabuli*	Ca_Kabuli_Chr01	4781416	(G/A)
598	CakSNP598	*Kabuli*	Ca_Kabuli_Chr01	4781704	(G/A)
599	CakSNP599	*Kabuli*	Ca_Kabuli_Chr01	4808910	(A/T)
600	CakSNP600	*Kabuli*	Ca_Kabuli_Chr01	4878683	(T/C)
601	CakSNP601	*Kabuli*	Ca_Kabuli_Chr01	4878691	(A/C)
602	CakSNP602	*Kabuli*	Ca_Kabuli_Chr01	4878695	(T/C)
603	CakSNP603	*Kabuli*	Ca_Kabuli_Chr01	4878823	(A/G)
604	CakSNP604	*Kabuli*	Ca_Kabuli_Chr01	4903185	(T/A)
605	CakSNP605	*Kabuli*	Ca_Kabuli_Chr01	4903203	(A/G)
606	CakSNP606	*Kabuli*	Ca_Kabuli_Chr01	4903239	(G/A)
607	CakSNP607	*Kabuli*	Ca_Kabuli_Chr01	4906759	(G/C)

S.N.	SNP IDs	Cultivars	Chromosomes/scaffolds	Physical positions (bp)	SNPs
608	CakSNP608	*Kabuli*	Ca_Kabuli_Chr01	4906775	(A/T)
609	CakSNP609	*Kabuli*	Ca_Kabuli_Chr01	4906777	(G/T)
610	CakSNP610	*Kabuli*	Ca_Kabuli_Chr01	4906778	(T/G)
611	CakSNP611	*Kabuli*	Ca_Kabuli_Chr01	4906800	(T/G)
612	CakSNP612	*Kabuli*	Ca_Kabuli_Chr01	4906816	(A/G)
613	CakSNP613	*Kabuli*	Ca_Kabuli_Chr01	4906868	(G/C)
614	CakSNP614	*Kabuli*	Ca_Kabuli_Chr01	4906859	(T/C)
615	CakSNP615	*Kabuli*	Ca_Kabuli_Chr01	4906854	(A/G)
616	CakSNP616	*Kabuli*	Ca_Kabuli_Chr01	4909155	(T/C)
617	CakSNP617	*Kabuli*	Ca_Kabuli_Chr01	4909286	(T/G)
618	CakSNP618	*Kabuli*	Ca_Kabuli_Chr01	4909280	(T/G)
619	CakSNP619	*Kabuli*	Ca_Kabuli_Chr01	4909271	(A/G)
620	CakSNP620	*Kabuli*	Ca_Kabuli_Chr01	4909268	(C/T)
621	CakSNP621	*Kabuli*	Ca_Kabuli_Chr01	4909261	(G/T)
622	CakSNP622	*Kabuli*	Ca_Kabuli_Chr01	4909255	(G/A)
623	CakSNP623	*Kabuli*	Ca_Kabuli_Chr01	4909253	(A/G)
624	CakSNP624	*Kabuli*	Ca_Kabuli_Chr01	4910497	(A/G)
625	CakSNP625	*Kabuli*	Ca_Kabuli_Chr01	4910494	(C/A)
626	CakSNP626	*Kabuli*	Ca_Kabuli_Chr01	4948740	(A/T)
627	CakSNP627	*Kabuli*	Ca_Kabuli_Chr01	4949578	(A/T)
628	CakSNP628	*Kabuli*	Ca_Kabuli_Chr01	4950457	(G/A)
629	CakSNP629	*Kabuli*	Ca_Kabuli_Chr01	4950726	(G/T)
630	CakSNP630	*Kabuli*	Ca_Kabuli_Chr01	4959562	(A/C)
631	CakSNP631	*Kabuli*	Ca_Kabuli_Chr01	4995240	(T/A)
632	CakSNP632	*Kabuli*	Ca_Kabuli_Chr01	4996404	(A/C)
633	CakSNP633	*Kabuli*	Ca_Kabuli_Chr01	4996459	(T/C)
634	CakSNP634	*Kabuli*	Ca_Kabuli_Chr01	4996476	(G/A)
635	CakSNP635	*Kabuli*	Ca_Kabuli_Chr01	4996536	(C/T)
636	CakSNP636	*Kabuli*	Ca_Kabuli_Chr01	5071210	(A/G)
637	CakSNP637	*Kabuli*	Ca_Kabuli_Chr01	5071195	(T/A)
638	CakSNP638	*Kabuli*	Ca_Kabuli_Chr01	5071177	(A/G)
639	CakSNP639	*Kabuli*	Ca_Kabuli_Chr01	5071158	(G/A)
640	CakSNP640	*Kabuli*	Ca_Kabuli_Chr01	5072022	(C/A)
641	CakSNP641	*Kabuli*	Ca_Kabuli_Chr01	5080342	(A/T)
642	CakSNP642	*Kabuli*	Ca_Kabuli_Chr01	5080358	(G/A)
643	CakSNP643	*Kabuli*	Ca_Kabuli_Chr01	5087428	(T/C)
644	CakSNP644	*Kabuli*	Ca_Kabuli_Chr01	5096676	(A/T)
645	CakSNP645	*Kabuli*	Ca_Kabuli_Chr01	5096650	(C/T)

S.N.	SNP IDs	Cultivars	Chromosomes/scaffolds	Physical positions (bp)	SNPs
646	CakSNP646	*Kabuli*	Ca_Kabuli_Chr01	5122663	(A/G)
647	CakSNP647	*Kabuli*	Ca_Kabuli_Chr01	5129940	(G/A)
648	CakSNP648	*Kabuli*	Ca_Kabuli_Chr01	5138013	(A/C)
649	CakSNP649	*Kabuli*	Ca_Kabuli_Chr01	5140801	(C/A)
650	CakSNP650	*Kabuli*	Ca_Kabuli_Chr01	5140837	(A/T)
651	CakSNP651	*Kabuli*	Ca_Kabuli_Chr01	5141009	(G/C)
652	CakSNP652	*Kabuli*	Ca_Kabuli_Chr01	5140944	(C/G)
653	CakSNP653	*Kabuli*	Ca_Kabuli_Chr01	5146658	(C/A)
654	CakSNP654	*Kabuli*	Ca_Kabuli_Chr01	5146667	(T/G)
655	CakSNP655	*Kabuli*	Ca_Kabuli_Chr01	5146696	(T/A)
656	CakSNP656	*Kabuli*	Ca_Kabuli_Chr01	5164127	(A/G)
657	CakSNP657	*Kabuli*	Ca_Kabuli_Chr01	5164136	(C/T)
658	CakSNP658	*Kabuli*	Ca_Kabuli_Chr01	5164198	(C/A)
659	CakSNP659	*Kabuli*	Ca_Kabuli_Chr01	5197461	(C/T)
660	CakSNP660	*Kabuli*	Ca_Kabuli_Chr01	5197475	(T/A)
661	CakSNP661	*Kabuli*	Ca_Kabuli_Chr01	5200250	(T/G)
662	CakSNP662	*Kabuli*	Ca_Kabuli_Chr01	5200289	(G/T)
663	CakSNP663	*Kabuli*	Ca_Kabuli_Chr01	5249839	(G/A)
664	CakSNP664	*Kabuli*	Ca_Kabuli_Chr01	5249846	(A/C)
665	CakSNP665	*Kabuli*	Ca_Kabuli_Chr01	5249868	(G/A)
666	CakSNP666	*Kabuli*	Ca_Kabuli_Chr01	5288427	(T/C)
667	CakSNP667	*Kabuli*	Ca_Kabuli_Chr01	5288421	(A/C)
668	CakSNP668	*Kabuli*	Ca_Kabuli_Chr01	5304576	(A/C)
669	CakSNP669	*Kabuli*	Ca_Kabuli_Chr01	5321398	(A/G)
670	CakSNP670	*Kabuli*	Ca_Kabuli_Chr01	5430700	(A/C)
671	CakSNP671	*Kabuli*	Ca_Kabuli_Chr01	5430703	(T/G)
672	CakSNP672	*Kabuli*	Ca_Kabuli_Chr01	5430809	(G/A)
673	CakSNP673	*Kabuli*	Ca_Kabuli_Chr01	5444130	(A/C)
674	CakSNP674	*Kabuli*	Ca_Kabuli_Chr01	5444142	(A/T)
675	CakSNP675	*Kabuli*	Ca_Kabuli_Chr01	5474604	(G/A)
676	CakSNP676	*Kabuli*	Ca_Kabuli_Chr01	5477196	(T/C)
677	CakSNP677	*Kabuli*	Ca_Kabuli_Chr01	5487535	(A/G)
678	CakSNP678	*Kabuli*	Ca_Kabuli_Chr01	5489806	(C/G)
679	CakSNP679	*Kabuli*	Ca_Kabuli_Chr01	5577080	(T/C)
680	CakSNP680	*Kabuli*	Ca_Kabuli_Chr01	5629027	(G/A)
681	CakSNP681	*Kabuli*	Ca_Kabuli_Chr01	5637388	(T/A)
682	CakSNP682	*Kabuli*	Ca_Kabuli_Chr01	5637442	(C/T)
683	CakSNP683	*Kabuli*	Ca_Kabuli_Chr01	5643313	(G/C)

S.N.	SNP IDs	Cultivars	Chromosomes/scaffolds	Physical positions (bp)	SNPs
684	CakSNP684	*Kabuli*	Ca_Kabuli_Chr01	5666178	(A/C)
685	CakSNP685	*Kabuli*	Ca_Kabuli_Chr01	5666217	(C/A)
686	CakSNP686	*Kabuli*	Ca_Kabuli_Chr01	5666260	(T/G)
687	CakSNP687	*Kabuli*	Ca_Kabuli_Chr01	5666266	(T/G)
688	CakSNP688	*Kabuli*	Ca_Kabuli_Chr01	5666263	(T/G)
689	CakSNP689	*Kabuli*	Ca_Kabuli_Chr01	5666355	(T/G)
690	CakSNP690	*Kabuli*	Ca_Kabuli_Chr01	5667605	(G/A)
691	CakSNP691	*Kabuli*	Ca_Kabuli_Chr01	5702638	(C/T)
692	CakSNP692	*Kabuli*	Ca_Kabuli_Chr01	5706858	(A/T)
693	CakSNP693	*Kabuli*	Ca_Kabuli_Chr01	5706961	(G/T)
694	CakSNP694	*Kabuli*	Ca_Kabuli_Chr01	5706948	(A/T)
695	CakSNP695	*Kabuli*	Ca_Kabuli_Chr01	5706944	(T/G)
696	CakSNP696	*Kabuli*	Ca_Kabuli_Chr01	5706920	(G/A)
697	CakSNP697	*Kabuli*	Ca_Kabuli_Chr01	5721616	(A/T)
698	CakSNP698	*Kabuli*	Ca_Kabuli_Chr01	5749456	(C/T)
699	CakSNP699	*Kabuli*	Ca_Kabuli_Chr01	5791118	(T/G)
700	CakSNP700	*Kabuli*	Ca_Kabuli_Chr01	5796146	(C/T)
701	CakSNP701	*Kabuli*	Ca_Kabuli_Chr01	5796886	(A/G)
702	CakSNP702	*Kabuli*	Ca_Kabuli_Chr01	5807492	(T/C)
703	CakSNP703	*Kabuli*	Ca_Kabuli_Chr01	5812283	(G/T)
704	CakSNP704	*Kabuli*	Ca_Kabuli_Chr01	5812298	(G/A)
705	CakSNP705	*Kabuli*	Ca_Kabuli_Chr01	5862565	(C/T)
706	CakSNP706	*Kabuli*	Ca_Kabuli_Chr01	5960264	(T/C)
707	CakSNP707	*Kabuli*	Ca_Kabuli_Chr01	5978146	(G/A)
708	CakSNP708	*Kabuli*	Ca_Kabuli_Chr01	5978289	(C/T)
709	CakSNP709	*Kabuli*	Ca_Kabuli_Chr01	5981127	(C/T)
710	CakSNP710	*Kabuli*	Ca_Kabuli_Chr01	5981178	(A/G)
711	CakSNP711	*Kabuli*	Ca_Kabuli_Chr01	6011727	(G/T)
712	CakSNP712	*Kabuli*	Ca_Kabuli_Chr01	6011697	(T/G)
713	CakSNP713	*Kabuli*	Ca_Kabuli_Chr01	6011689	(G/A)
714	CakSNP714	*Kabuli*	Ca_Kabuli_Chr01	6011657	(T/C)
715	CakSNP715	*Kabuli*	Ca_Kabuli_Chr01	6011761	(C/T)
716	CakSNP716	*Kabuli*	Ca_Kabuli_Chr01	6017854	(A/C)
717	CakSNP717	*Kabuli*	Ca_Kabuli_Chr01	6066972	(C/T)
718	CakSNP718	*Kabuli*	Ca_Kabuli_Chr01	6066936	(A/G)
719	CakSNP719	*Kabuli*	Ca_Kabuli_Chr01	6066933	(C/T)
720	CakSNP720	*Kabuli*	Ca_Kabuli_Chr01	6067846	(T/C)
721	CakSNP721	*Kabuli*	Ca_Kabuli_Chr01	6088283	(C/T)

S.N.	SNP IDs	Cultivars	Chromosomes/scaffolds	Physical positions (bp)	SNPs
722	CakSNP722	*Kabuli*	Ca_Kabuli_Chr01	6101205	(C/T)
723	CakSNP723	*Kabuli*	Ca_Kabuli_Chr01	6126761	(T/A)
724	CakSNP724	*Kabuli*	Ca_Kabuli_Chr01	6126772	(G/A)
725	CakSNP725	*Kabuli*	Ca_Kabuli_Chr01	6155341	(A/G)
726	CakSNP726	*Kabuli*	Ca_Kabuli_Chr01	6224010	(C/G)
727	CakSNP727	*Kabuli*	Ca_Kabuli_Chr01	6224015	(C/T)
728	CakSNP728	*Kabuli*	Ca_Kabuli_Chr01	6249062	(T/C)
729	CakSNP729	*Kabuli*	Ca_Kabuli_Chr01	6249035	(T/C)
730	CakSNP730	*Kabuli*	Ca_Kabuli_Chr01	6257653	(C/G)
731	CakSNP731	*Kabuli*	Ca_Kabuli_Chr01	6257712	(A/T)
732	CakSNP732	*Kabuli*	Ca_Kabuli_Chr01	6259458	(T/C)
733	CakSNP733	*Kabuli*	Ca_Kabuli_Chr01	6262461	(A/G)
734	CakSNP734	*Kabuli*	Ca_Kabuli_Chr01	6262529	(T/G)
735	CakSNP735	*Kabuli*	Ca_Kabuli_Chr01	6262577	(A/G)
736	CakSNP736	*Kabuli*	Ca_Kabuli_Chr01	6278572	(T/C)
737	CakSNP737	*Kabuli*	Ca_Kabuli_Chr01	6278625	(T/C)
738	CakSNP738	*Kabuli*	Ca_Kabuli_Chr01	6279644	(G/T)
739	CakSNP739	*Kabuli*	Ca_Kabuli_Chr01	6296858	(G/A)
740	CakSNP740	*Kabuli*	Ca_Kabuli_Chr01	6296986	(C/A)
741	CakSNP741	*Kabuli*	Ca_Kabuli_Chr01	6296929	(C/T)
742	CakSNP742	*Kabuli*	Ca_Kabuli_Chr01	6315337	(G/T)
743	CakSNP743	*Kabuli*	Ca_Kabuli_Chr01	6315402	(C/T)
744	CakSNP744	*Kabuli*	Ca_Kabuli_Chr01	6315399	(T/G)
745	CakSNP745	*Kabuli*	Ca_Kabuli_Chr01	6315397	(C/G)
746	CakSNP746	*Kabuli*	Ca_Kabuli_Chr01	6328801	(T/A)
747	CakSNP747	*Kabuli*	Ca_Kabuli_Chr01	6328957	(C/T)
748	CakSNP748	*Kabuli*	Ca_Kabuli_Chr01	6328963	(A/G)
749	CakSNP749	*Kabuli*	Ca_Kabuli_Chr01	6329805	(A/G)
750	CakSNP750	*Kabuli*	Ca_Kabuli_Chr01	6329814	(T/A)
751	CakSNP751	*Kabuli*	Ca_Kabuli_Chr01	6329844	(C/T)
752	CakSNP752	*Kabuli*	Ca_Kabuli_Chr01	6329847	(A/G)
753	CakSNP753	*Kabuli*	Ca_Kabuli_Chr01	6332229	(T/C)
754	CakSNP754	*Kabuli*	Ca_Kabuli_Chr01	6347626	(C/T)
755	CakSNP755	*Kabuli*	Ca_Kabuli_Chr01	6347720	(G/A)
756	CakSNP756	*Kabuli*	Ca_Kabuli_Chr01	6381114	(T/G)
757	CakSNP757	*Kabuli*	Ca_Kabuli_Chr01	6381191	(T/C)
758	CakSNP758	*Kabuli*	Ca_Kabuli_Chr01	6390541	(T/A)
759	CakSNP759	*Kabuli*	Ca_Kabuli_Chr01	6390849	(G/A)

S.N.	SNP IDs	Cultivars	Chromosomes/scaffolds	Physical positions (bp)	SNPs
760	CakSNP760	*Kabuli*	Ca_Kabuli_Chr01	6391017	(T/C)
761	CakSNP761	*Kabuli*	Ca_Kabuli_Chr01	6433592	(T/G)
762	CakSNP762	*Kabuli*	Ca_Kabuli_Chr01	6433826	(G/A)
763	CakSNP763	*Kabuli*	Ca_Kabuli_Chr01	6433838	(C/T)
764	CakSNP764	*Kabuli*	Ca_Kabuli_Chr01	6433841	(C/T)
765	CakSNP765	*Kabuli*	Ca_Kabuli_Chr01	6433883	(A/G)
766	CakSNP766	*Kabuli*	Ca_Kabuli_Chr01	6433934	(T/A)
767	CakSNP767	*Kabuli*	Ca_Kabuli_Chr01	6433925	(C/T)
768	CakSNP768	*Kabuli*	Ca_Kabuli_Chr01	6478736	(A/G)
769	CakSNP769	*Kabuli*	Ca_Kabuli_Chr01	6478758	(A/G)
770	CakSNP770	*Kabuli*	Ca_Kabuli_Chr01	6486446	(G/C)
771	CakSNP771	*Kabuli*	Ca_Kabuli_Chr01	6490978	(A/T)
772	CakSNP772	*Kabuli*	Ca_Kabuli_Chr01	6491008	(A/T)
773	CakSNP773	*Kabuli*	Ca_Kabuli_Chr01	6491030	(T/G)
774	CakSNP774	*Kabuli*	Ca_Kabuli_Chr01	6491017	(T/C)
775	CakSNP775	*Kabuli*	Ca_Kabuli_Chr01	6521670	(C/T)
776	CakSNP776	*Kabuli*	Ca_Kabuli_Chr01	6521673	(G/A)
777	CakSNP777	*Kabuli*	Ca_Kabuli_Chr01	6521856	(A/G)
778	CakSNP778	*Kabuli*	Ca_Kabuli_Chr01	6521859	(T/A)
779	CakSNP779	*Kabuli*	Ca_Kabuli_Chr01	6521970	(C/T)
780	CakSNP780	*Kabuli*	Ca_Kabuli_Chr01	6540056	(C/T)
781	CakSNP781	*Kabuli*	Ca_Kabuli_Chr01	6540170	(G/A)
782	CakSNP782	*Kabuli*	Ca_Kabuli_Chr01	6553107	(A/C)
783	CakSNP783	*Kabuli*	Ca_Kabuli_Chr01	6564716	(C/T)
784	CakSNP784	*Kabuli*	Ca_Kabuli_Chr01	6564789	(C/T)
785	CakSNP785	*Kabuli*	Ca_Kabuli_Chr01	6591506	(G/C)
786	CakSNP786	*Kabuli*	Ca_Kabuli_Chr01	6591518	(G/T)
787	CakSNP787	*Kabuli*	Ca_Kabuli_Chr01	6599917	(C/A)
788	CakSNP788	*Kabuli*	Ca_Kabuli_Chr01	6634482	(G/C)
789	CakSNP789	*Kabuli*	Ca_Kabuli_Chr01	6635404	(C/T)
790	CakSNP790	*Kabuli*	Ca_Kabuli_Chr01	6635401	(T/C)
791	CakSNP791	*Kabuli*	Ca_Kabuli_Chr01	6635366	(T/C)
792	CakSNP792	*Kabuli*	Ca_Kabuli_Chr01	6679253	(A/G)
793	CakSNP793	*Kabuli*	Ca_Kabuli_Chr01	6679329	(A/G)
794	CakSNP794	*Kabuli*	Ca_Kabuli_Chr01	6803364	(G/A)
795	CakSNP795	*Kabuli*	Ca_Kabuli_Chr01	6804031	(C/T)
796	CakSNP796	*Kabuli*	Ca_Kabuli_Chr01	6804110	(A/T)
797	CakSNP797	*Kabuli*	Ca_Kabuli_Chr01	6849437	(C/T)

S.N.	SNP IDs	Cultivars	Chromosomes/scaffolds	Physical positions (bp)	SNPs
798	CakSNP798	*Kabuli*	Ca_Kabuli_Chr01	6849512	(G/C)
799	CakSNP799	*Kabuli*	Ca_Kabuli_Chr01	6879110	(G/C)
800	CakSNP800	*Kabuli*	Ca_Kabuli_Chr01	6880666	(A/G)
801	CakSNP801	*Kabuli*	Ca_Kabuli_Chr01	6880837	(A/G)
802	CakSNP802	*Kabuli*	Ca_Kabuli_Chr01	6904278	(A/C)
803	CakSNP803	*Kabuli*	Ca_Kabuli_Chr01	6904313	(A/C)
804	CakSNP804	*Kabuli*	Ca_Kabuli_Chr01	6904338	(C/T)
805	CakSNP805	*Kabuli*	Ca_Kabuli_Chr01	6952382	(G/T)
806	CakSNP806	*Kabuli*	Ca_Kabuli_Chr01	7050825	(G/A)
807	CakSNP807	*Kabuli*	Ca_Kabuli_Chr01	7050958	(C/T)
808	CakSNP808	*Kabuli*	Ca_Kabuli_Chr01	7050953	(C/T)
809	CakSNP809	*Kabuli*	Ca_Kabuli_Chr01	7104985	(G/T)
810	CakSNP810	*Kabuli*	Ca_Kabuli_Chr01	7127843	(A/G)
811	CakSNP811	*Kabuli*	Ca_Kabuli_Chr01	7136677	(T/C)
812	CakSNP812	*Kabuli*	Ca_Kabuli_Chr01	7147651	(T/A)
813	CakSNP813	*Kabuli*	Ca_Kabuli_Chr01	7147643	(A/G)
814	CakSNP814	*Kabuli*	Ca_Kabuli_Chr01	7155344	(A/G)
815	CakSNP815	*Kabuli*	Ca_Kabuli_Chr01	7155302	(A/G)
816	CakSNP816	*Kabuli*	Ca_Kabuli_Chr01	7156354	(G/T)
817	CakSNP817	*Kabuli*	Ca_Kabuli_Chr01	7156355	(A/T)
818	CakSNP818	*Kabuli*	Ca_Kabuli_Chr01	7157057	(A/C)
819	CakSNP819	*Kabuli*	Ca_Kabuli_Chr01	7157112	(T/C)
820	CakSNP820	*Kabuli*	Ca_Kabuli_Chr01	7160649	(T/C)
821	CakSNP821	*Kabuli*	Ca_Kabuli_Chr01	7160648	(A/T)
822	CakSNP822	*Kabuli*	Ca_Kabuli_Chr01	7160695	(G/A)
823	CakSNP823	*Kabuli*	Ca_Kabuli_Chr01	7160698	(A/C)
824	CakSNP824	*Kabuli*	Ca_Kabuli_Chr01	7160754	(A/T)
825	CakSNP825	*Kabuli*	Ca_Kabuli_Chr01	7160781	(A/C)
826	CakSNP826	*Kabuli*	Ca_Kabuli_Chr01	7160773	(G/A)
827	CakSNP827	*Kabuli*	Ca_Kabuli_Chr01	7161122	(A/G)
828	CakSNP828	*Kabuli*	Ca_Kabuli_Chr01	7184813	(A/C)
829	CakSNP829	*Kabuli*	Ca_Kabuli_Chr01	7184773	(G/A)
830	CakSNP830	*Kabuli*	Ca_Kabuli_Chr01	7187934	(A/G)
831	CakSNP831	*Kabuli*	Ca_Kabuli_Chr01	7187969	(C/G)
832	CakSNP832	*Kabuli*	Ca_Kabuli_Chr01	7196570	(T/C)
833	CakSNP833	*Kabuli*	Ca_Kabuli_Chr01	7196601	(T/C)
834	CakSNP834	*Kabuli*	Ca_Kabuli_Chr01	7231750	(A/C)
835	CakSNP835	*Kabuli*	Ca_Kabuli_Chr01	7325164	(T/A)

S.N.	SNP IDs	Cultivars	Chromosomes/scaffolds	Physical positions (bp)	SNPs
836	CakSNP836	*Kabuli*	Ca_Kabuli_Chr01	7417717	(G/A)
837	CakSNP837	*Kabuli*	Ca_Kabuli_Chr01	7417699	(G/A)
838	CakSNP838	*Kabuli*	Ca_Kabuli_Chr01	7417788	(G/A)
839	CakSNP839	*Kabuli*	Ca_Kabuli_Chr01	7439421	(C/T)
840	CakSNP840	*Kabuli*	Ca_Kabuli_Chr01	7467280	(A/G)
841	CakSNP841	*Kabuli*	Ca_Kabuli_Chr01	7478022	(G/A)
842	CakSNP842	*Kabuli*	Ca_Kabuli_Chr01	7478046	(T/G)
843	CakSNP843	*Kabuli*	Ca_Kabuli_Chr01	7486102	(C/T)
844	CakSNP844	*Kabuli*	Ca_Kabuli_Chr01	7486273	(T/C)
845	CakSNP845	*Kabuli*	Ca_Kabuli_Chr01	7486282	(G/A)
846	CakSNP846	*Kabuli*	Ca_Kabuli_Chr01	7497334	(C/A)
847	CakSNP847	*Kabuli*	Ca_Kabuli_Chr01	7497362	(T/C)
848	CakSNP848	*Kabuli*	Ca_Kabuli_Chr01	7500494	(T/G)
849	CakSNP849	*Kabuli*	Ca_Kabuli_Chr01	7500524	(T/C)
850	CakSNP850	*Kabuli*	Ca_Kabuli_Chr01	7500665	(C/A)
851	CakSNP851	*Kabuli*	Ca_Kabuli_Chr01	7505779	(A/T)
852	CakSNP852	*Kabuli*	Ca_Kabuli_Chr01	7505792	(A/T)
853	CakSNP853	*Kabuli*	Ca_Kabuli_Chr01	7515002	(T/C)
854	CakSNP854	*Kabuli*	Ca_Kabuli_Chr01	7515605	(T/C)
855	CakSNP855	*Kabuli*	Ca_Kabuli_Chr01	7515578	(A/C)
856	CakSNP856	*Kabuli*	Ca_Kabuli_Chr01	7557751	(T/G)
857	CakSNP857	*Kabuli*	Ca_Kabuli_Chr01	7557811	(A/T)
858	CakSNP858	*Kabuli*	Ca_Kabuli_Chr01	7690613	(T/G)
859	CakSNP859	*Kabuli*	Ca_Kabuli_Chr01	7690620	(T/G)
860	CakSNP860	*Kabuli*	Ca_Kabuli_Chr01	7690625	(A/C)
861	CakSNP861	*Kabuli*	Ca_Kabuli_Chr01	7695844	(T/C)
862	CakSNP862	*Kabuli*	Ca_Kabuli_Chr01	7811388	(C/T)
863	CakSNP863	*Kabuli*	Ca_Kabuli_Chr01	7925666	(A/C)
864	CakSNP864	*Kabuli*	Ca_Kabuli_Chr01	7925801	(C/T)
865	CakSNP865	*Kabuli*	Ca_Kabuli_Chr01	7925774	(C/T)
866	CakSNP866	*Kabuli*	Ca_Kabuli_Chr01	7925738	(C/T)
867	CakSNP867	*Kabuli*	Ca_Kabuli_Chr01	7969202	(A/C)
868	CakSNP868	*Kabuli*	Ca_Kabuli_Chr01	7969694	(A/G)
869	CakSNP869	*Kabuli*	Ca_Kabuli_Chr01	7969647	(G/A)
870	CakSNP870	*Kabuli*	Ca_Kabuli_Chr01	8036666	(C/T)
871	CakSNP871	*Kabuli*	Ca_Kabuli_Chr01	8036706	(A/G)
872	CakSNP872	*Kabuli*	Ca_Kabuli_Chr01	8036917	(T/G)
873	CakSNP873	*Kabuli*	Ca_Kabuli_Chr01	8036903	(A/T)

S.N.	SNP IDs	Cultivars	Chromosomes/scaffolds	Physical positions (bp)	SNPs
874	CakSNP874	*Kabuli*	Ca_Kabuli_Chr01	8075631	(A/T)
875	CakSNP875	*Kabuli*	Ca_Kabuli_Chr01	8103090	(G/T)
876	CakSNP876	*Kabuli*	Ca_Kabuli_Chr01	8103339	(T/C)
877	CakSNP877	*Kabuli*	Ca_Kabuli_Chr01	8103401	(T/A)
878	CakSNP878	*Kabuli*	Ca_Kabuli_Chr01	8107967	(A/C)
879	CakSNP879	*Kabuli*	Ca_Kabuli_Chr01	8108014	(C/T)
880	CakSNP880	*Kabuli*	Ca_Kabuli_Chr01	8111588	(G/A)
881	CakSNP881	*Kabuli*	Ca_Kabuli_Chr01	8112343	(C/T)
882	CakSNP882	*Kabuli*	Ca_Kabuli_Chr01	8113235	(G/T)
883	CakSNP883	*Kabuli*	Ca_Kabuli_Chr01	8113383	(T/G)
884	CakSNP884	*Kabuli*	Ca_Kabuli_Chr01	8113449	(T/A)
885	CakSNP885	*Kabuli*	Ca_Kabuli_Chr01	8113403	(A/T)
886	CakSNP886	*Kabuli*	Ca_Kabuli_Chr01	8113398	(C/G)
887	CakSNP887	*Kabuli*	Ca_Kabuli_Chr01	8123033	(A/G)
888	CakSNP888	*Kabuli*	Ca_Kabuli_Chr01	8123257	(A/G)
889	CakSNP889	*Kabuli*	Ca_Kabuli_Chr01	8123481	(A/G)
890	CakSNP890	*Kabuli*	Ca_Kabuli_Chr01	8134368	(T/C)
891	CakSNP891	*Kabuli*	Ca_Kabuli_Chr01	8178109	(T/C)
892	CakSNP892	*Kabuli*	Ca_Kabuli_Chr01	8178146	(G/A)
893	CakSNP893	*Kabuli*	Ca_Kabuli_Chr01	8192580	(T/C)
894	CakSNP894	*Kabuli*	Ca_Kabuli_Chr01	8220996	(T/A)
895	CakSNP895	*Kabuli*	Ca_Kabuli_Chr01	8220968	(A/G)
896	CakSNP896	*Kabuli*	Ca_Kabuli_Chr01	8343356	(G/A)
897	CakSNP897	*Kabuli*	Ca_Kabuli_Chr01	8389242	(G/A)
898	CakSNP898	*Kabuli*	Ca_Kabuli_Chr01	8464621	(C/G)
899	CakSNP899	*Kabuli*	Ca_Kabuli_Chr01	8582341	(T/C)
900	CakSNP900	*Kabuli*	Ca_Kabuli_Chr01	8607820	(T/C)
901	CakSNP901	*Kabuli*	Ca_Kabuli_Chr01	8607919	(A/C)
902	CakSNP902	*Kabuli*	Ca_Kabuli_Chr01	8633598	(G/A)
903	CakSNP903	*Kabuli*	Ca_Kabuli_Chr01	8665705	(T/G)
904	CakSNP904	*Kabuli*	Ca_Kabuli_Chr01	8697177	(G/T)
905	CakSNP905	*Kabuli*	Ca_Kabuli_Chr01	8698059	(T/C)
906	CakSNP906	*Kabuli*	Ca_Kabuli_Chr01	8735740	(G/T)
907	CakSNP907	*Kabuli*	Ca_Kabuli_Chr01	8735727	(G/C)
908	CakSNP908	*Kabuli*	Ca_Kabuli_Chr01	8736733	(G/A)
909	CakSNP909	*Kabuli*	Ca_Kabuli_Chr01	8736711	(G/T)
910	CakSNP910	*Kabuli*	Ca_Kabuli_Chr01	8737380	(A/G)
911	CakSNP911	*Kabuli*	Ca_Kabuli_Chr01	8738691	(T/G)

S.N.	SNP IDs	Cultivars	Chromosomes/scaffolds	Physical positions (bp)	SNPs
912	CakSNP912	*Kabuli*	Ca_Kabuli_Chr01	8738695	(G/C)
913	CakSNP913	*Kabuli*	Ca_Kabuli_Chr01	8767122	(T/A)
914	CakSNP914	*Kabuli*	Ca_Kabuli_Chr01	8767121	(A/T)
915	CakSNP915	*Kabuli*	Ca_Kabuli_Chr01	8799685	(T/G)
916	CakSNP916	*Kabuli*	Ca_Kabuli_Chr01	8799701	(T/C)
917	CakSNP917	*Kabuli*	Ca_Kabuli_Chr01	8799708	(T/C)
918	CakSNP918	*Kabuli*	Ca_Kabuli_Chr01	8799758	(C/G)
919	CakSNP919	*Kabuli*	Ca_Kabuli_Chr01	8809188	(T/A)
920	CakSNP920	*Kabuli*	Ca_Kabuli_Chr01	8809191	(C/T)
921	CakSNP921	*Kabuli*	Ca_Kabuli_Chr01	8809295	(A/T)
922	CakSNP922	*Kabuli*	Ca_Kabuli_Chr01	8809517	(C/G)
923	CakSNP923	*Kabuli*	Ca_Kabuli_Chr01	8822121	(G/T)
924	CakSNP924	*Kabuli*	Ca_Kabuli_Chr01	8822375	(G/A)
925	CakSNP925	*Kabuli*	Ca_Kabuli_Chr01	8822366	(T/A)
926	CakSNP926	*Kabuli*	Ca_Kabuli_Chr01	8824856	(T/C)
927	CakSNP927	*Kabuli*	Ca_Kabuli_Chr01	8852863	(G/T)
928	CakSNP928	*Kabuli*	Ca_Kabuli_Chr01	8852928	(T/A)
929	CakSNP929	*Kabuli*	Ca_Kabuli_Chr01	8852981	(C/A)
930	CakSNP930	*Kabuli*	Ca_Kabuli_Chr01	8852957	(C/A)
931	CakSNP931	*Kabuli*	Ca_Kabuli_Chr01	8852943	(C/A)
932	CakSNP932	*Kabuli*	Ca_Kabuli_Chr01	8853010	(A/G)
933	CakSNP933	*Kabuli*	Ca_Kabuli_Chr01	8854211	(G/T)
934	CakSNP934	*Kabuli*	Ca_Kabuli_Chr01	8891438	(T/G)
935	CakSNP935	*Kabuli*	Ca_Kabuli_Chr01	8891485	(T/G)
936	CakSNP936	*Kabuli*	Ca_Kabuli_Chr01	8891562	(C/G)
937	CakSNP937	*Kabuli*	Ca_Kabuli_Chr01	8891561	(G/A)
938	CakSNP938	*Kabuli*	Ca_Kabuli_Chr01	8893985	(G/A)
939	CakSNP939	*Kabuli*	Ca_Kabuli_Chr01	8901769	(C/A)
940	CakSNP940	*Kabuli*	Ca_Kabuli_Chr01	8901842	(T/A)
941	CakSNP941	*Kabuli*	Ca_Kabuli_Chr01	8941004	(A/C)
942	CakSNP942	*Kabuli*	Ca_Kabuli_Chr01	8941029	(G/A)
943	CakSNP943	*Kabuli*	Ca_Kabuli_Chr01	8941031	(G/C)
944	CakSNP944	*Kabuli*	Ca_Kabuli_Chr01	8942137	(T/C)
945	CakSNP945	*Kabuli*	Ca_Kabuli_Chr01	8942131	(T/C)
946	CakSNP946	*Kabuli*	Ca_Kabuli_Chr01	8942198	(A/G)
947	CakSNP947	*Kabuli*	Ca_Kabuli_Chr01	8942179	(C/G)
948	CakSNP948	*Kabuli*	Ca_Kabuli_Chr01	8949723	(G/T)
949	CakSNP949	*Kabuli*	Ca_Kabuli_Chr01	8960630	(T/C)

S.N.	SNP IDs	Cultivars	Chromosomes/scaffolds	Physical positions (bp)	SNPs
950	CakSNP950	*Kabuli*	Ca_Kabuli_Chr01	8969321	(C/T)
951	CakSNP951	*Kabuli*	Ca_Kabuli_Chr01	8971718	(C/G)
952	CakSNP952	*Kabuli*	Ca_Kabuli_Chr01	8971776	(A/G)
953	CakSNP953	*Kabuli*	Ca_Kabuli_Chr01	8976169	(A/T)
954	CakSNP954	*Kabuli*	Ca_Kabuli_Chr01	8980112	(C/A)
955	CakSNP955	*Kabuli*	Ca_Kabuli_Chr01	8993300	(T/C)
956	CakSNP956	*Kabuli*	Ca_Kabuli_Chr01	8993329	(A/C)
957	CakSNP957	*Kabuli*	Ca_Kabuli_Chr01	9018113	(A/C)
958	CakSNP958	*Kabuli*	Ca_Kabuli_Chr01	9031417	(G/T)
959	CakSNP959	*Kabuli*	Ca_Kabuli_Chr01	9031825	(G/T)
960	CakSNP960	*Kabuli*	Ca_Kabuli_Chr01	9032126	(G/A)
961	CakSNP961	*Kabuli*	Ca_Kabuli_Chr01	9035744	(G/A)
962	CakSNP962	*Kabuli*	Ca_Kabuli_Chr01	9040419	(T/A)
963	CakSNP963	*Kabuli*	Ca_Kabuli_Chr01	9048675	(A/T)
964	CakSNP964	*Kabuli*	Ca_Kabuli_Chr01	9048682	(T/G)
965	CakSNP965	*Kabuli*	Ca_Kabuli_Chr01	9054490	(G/A)
966	CakSNP966	*Kabuli*	Ca_Kabuli_Chr01	9065933	(T/C)
967	CakSNP967	*Kabuli*	Ca_Kabuli_Chr01	9093812	(C/G)
968	CakSNP968	*Kabuli*	Ca_Kabuli_Chr01	9093964	(T/A)
969	CakSNP969	*Kabuli*	Ca_Kabuli_Chr01	9097885	(A/G)
970	CakSNP970	*Kabuli*	Ca_Kabuli_Chr01	9097881	(C/G)
971	CakSNP971	*Kabuli*	Ca_Kabuli_Chr01	9097879	(C/A)
972	CakSNP972	*Kabuli*	Ca_Kabuli_Chr01	9097878	(A/G)
973	CakSNP973	*Kabuli*	Ca_Kabuli_Chr01	9097877	(C/A)
974	CakSNP974	*Kabuli*	Ca_Kabuli_Chr01	9097869	(G/T)
975	CakSNP975	*Kabuli*	Ca_Kabuli_Chr01	9097947	(C/A)
976	CakSNP976	*Kabuli*	Ca_Kabuli_Chr01	9097949	(A/C)
977	CakSNP977	*Kabuli*	Ca_Kabuli_Chr01	9098003	(C/T)
978	CakSNP978	*Kabuli*	Ca_Kabuli_Chr01	9098051	(A/G)
979	CakSNP979	*Kabuli*	Ca_Kabuli_Chr01	9105432	(C/T)
980	CakSNP980	*Kabuli*	Ca_Kabuli_Chr01	9195312	(G/A)
981	CakSNP981	*Kabuli*	Ca_Kabuli_Chr01	9237766	(T/G)
982	CakSNP982	*Kabuli*	Ca_Kabuli_Chr01	9237813	(G/T)
983	CakSNP983	*Kabuli*	Ca_Kabuli_Chr01	9269888	(T/A)
984	CakSNP984	*Kabuli*	Ca_Kabuli_Chr01	9468041	(C/T)
985	CakSNP985	*Kabuli*	Ca_Kabuli_Chr01	9468895	(C/A)
986	CakSNP986	*Kabuli*	Ca_Kabuli_Chr01	9476207	(A/C)
987	CakSNP987	*Kabuli*	Ca_Kabuli_Chr01	9476388	(A/G)

S.N.	SNP IDs	Cultivars	Chromosomes/scaffolds	Physical positions (bp)	SNPs
988	CakSNP988	*Kabuli*	Ca_Kabuli_Chr01	9476385	(A/G)
989	CakSNP989	*Kabuli*	Ca_Kabuli_Chr01	9535616	(C/T)
990	CakSNP990	*Kabuli*	Ca_Kabuli_Chr01	9547223	(A/G)
991	CakSNP991	*Kabuli*	Ca_Kabuli_Chr01	9547310	(A/G)
992	CakSNP992	*Kabuli*	Ca_Kabuli_Chr01	9547854	(G/A)
993	CakSNP993	*Kabuli*	Ca_Kabuli_Chr01	9547834	(C/T)
994	CakSNP994	*Kabuli*	Ca_Kabuli_Chr01	9547819	(T/C)
995	CakSNP995	*Kabuli*	Ca_Kabuli_Chr01	9556418	(C/G)
996	CakSNP996	*Kabuli*	Ca_Kabuli_Chr01	9556486	(T/G)
997	CakSNP997	*Kabuli*	Ca_Kabuli_Chr01	9556487	(T/A)
998	CakSNP998	*Kabuli*	Ca_Kabuli_Chr01	9556488	(G/A)
999	CakSNP999	*Kabuli*	Ca_Kabuli_Chr01	9556588	(G/T)
1000	CakSNP1000	*Kabuli*	Ca_Kabuli_Chr01	9556604	(G/C)
1001	CakSNP1001	*Kabuli*	Ca_Kabuli_Chr01	9556628	(G/A)
1002	CakSNP1002	*Kabuli*	Ca_Kabuli_Chr01	9556735	(G/A)
1003	CakSNP1003	*Kabuli*	Ca_Kabuli_Chr01	9556662	(C/T)
1004	CakSNP1004	*Kabuli*	Ca_Kabuli_Chr01	9567049	(C/G)
1005	CakSNP1005	*Kabuli*	Ca_Kabuli_Chr01	9603896	(T/A)
1006	CakSNP1006	*Kabuli*	Ca_Kabuli_Chr01	9634823	(C/T)
1007	CakSNP1007	*Kabuli*	Ca_Kabuli_Chr01	9647861	(G/C)
1008	CakSNP1008	*Kabuli*	Ca_Kabuli_Chr01	9659316	(C/T)
1009	CakSNP1009	*Kabuli*	Ca_Kabuli_Chr01	9705162	(A/C)
1010	CakSNP1010	*Kabuli*	Ca_Kabuli_Chr01	9706910	(C/T)
1011	CakSNP1011	*Kabuli*	Ca_Kabuli_Chr01	9737032	(C/T)
1012	CakSNP1012	*Kabuli*	Ca_Kabuli_Chr01	9737067	(T/C)
1013	CakSNP1013	*Kabuli*	Ca_Kabuli_Chr01	9757006	(G/A)
1014	CakSNP1014	*Kabuli*	Ca_Kabuli_Chr01	9779398	(G/A)
1015	CakSNP1015	*Kabuli*	Ca_Kabuli_Chr01	9797342	(G/T)
1016	CakSNP1016	*Kabuli*	Ca_Kabuli_Chr01	9810275	(T/C)
1017	CakSNP1017	*Kabuli*	Ca_Kabuli_Chr01	9815448	(A/T)
1018	CakSNP1018	*Kabuli*	Ca_Kabuli_Chr01	9815508	(G/C)
1019	CakSNP1019	*Kabuli*	Ca_Kabuli_Chr01	9836198	(A/C)
1020	CakSNP1020	*Kabuli*	Ca_Kabuli_Chr01	9896511	(C/T)
1021	CakSNP1021	*Kabuli*	Ca_Kabuli_Chr01	9920951	(G/A)
1022	CakSNP1022	*Kabuli*	Ca_Kabuli_Chr01	9920986	(A/C)
1023	CakSNP1023	*Kabuli*	Ca_Kabuli_Chr01	9932690	(C/G)
1024	CakSNP1024	*Kabuli*	Ca_Kabuli_Chr01	10038486	(T/G)
1025	CakSNP1025	*Kabuli*	Ca_Kabuli_Chr01	10049364	(A/G)

S.N.	SNP IDs	Cultivars	Chromosomes/scaffolds	Physical positions (bp)	SNPs
1026	CakSNP1026	*Kabuli*	Ca_Kabuli_Chr01	10049469	(A/G)
1027	CakSNP1027	*Kabuli*	Ca_Kabuli_Chr01	10142688	(A/G)
1028	CakSNP1028	*Kabuli*	Ca_Kabuli_Chr01	10155496	(T/G)
1029	CakSNP1029	*Kabuli*	Ca_Kabuli_Chr01	10155584	(T/C)
1030	CakSNP1030	*Kabuli*	Ca_Kabuli_Chr01	10191578	(A/C)
1031	CakSNP1031	*Kabuli*	Ca_Kabuli_Chr01	10201074	(C/G)
1032	CakSNP1032	*Kabuli*	Ca_Kabuli_Chr01	10212925	(G/C)
1033	CakSNP1033	*Kabuli*	Ca_Kabuli_Chr01	10212926	(A/C)
1034	CakSNP1034	*Kabuli*	Ca_Kabuli_Chr01	10212948	(T/C)
1035	CakSNP1035	*Kabuli*	Ca_Kabuli_Chr01	10212976	(T/A)
1036	CakSNP1036	*Kabuli*	Ca_Kabuli_Chr01	10213111	(T/C)
1037	CakSNP1037	*Kabuli*	Ca_Kabuli_Chr01	10266774	(C/T)
1038	CakSNP1038	*Kabuli*	Ca_Kabuli_Chr01	10289781	(G/A)
1039	CakSNP1039	*Kabuli*	Ca_Kabuli_Chr01	10340870	(G/C)
1040	CakSNP1040	*Kabuli*	Ca_Kabuli_Chr01	10364986	(C/G)
1041	CakSNP1041	*Kabuli*	Ca_Kabuli_Chr01	10365024	(G/A)
1042	CakSNP1042	*Kabuli*	Ca_Kabuli_Chr01	10365029	(T/A)
1043	CakSNP1043	*Kabuli*	Ca_Kabuli_Chr01	10366383	(G/A)
1044	CakSNP1044	*Kabuli*	Ca_Kabuli_Chr01	10377700	(G/C)
1045	CakSNP1045	*Kabuli*	Ca_Kabuli_Chr01	10377698	(T/C)
1046	CakSNP1046	*Kabuli*	Ca_Kabuli_Chr01	10377691	(T/C)
1047	CakSNP1047	*Kabuli*	Ca_Kabuli_Chr01	10377860	(T/A)
1048	CakSNP1048	*Kabuli*	Ca_Kabuli_Chr01	10408322	(A/G)
1049	CakSNP1049	*Kabuli*	Ca_Kabuli_Chr01	10408349	(G/T)
1050	CakSNP1050	*Kabuli*	Ca_Kabuli_Chr01	10410479	(T/C)
1051	CakSNP1051	*Kabuli*	Ca_Kabuli_Chr01	10439153	(C/G)
1052	CakSNP1052	*Kabuli*	Ca_Kabuli_Chr01	10439177	(T/C)
1053	CakSNP1053	*Kabuli*	Ca_Kabuli_Chr01	10439190	(C/A)
1054	CakSNP1054	*Kabuli*	Ca_Kabuli_Chr01	10440393	(C/T)
1055	CakSNP1055	*Kabuli*	Ca_Kabuli_Chr01	10440430	(G/T)
1056	CakSNP1056	*Kabuli*	Ca_Kabuli_Chr01	10440533	(C/A)
1057	CakSNP1057	*Kabuli*	Ca_Kabuli_Chr01	10440479	(C/T)
1058	CakSNP1058	*Kabuli*	Ca_Kabuli_Chr01	10489197	(A/G)
1059	CakSNP1059	*Kabuli*	Ca_Kabuli_Chr01	10527733	(A/G)
1060	CakSNP1060	*Kabuli*	Ca_Kabuli_Chr01	10551440	(T/C)
1061	CakSNP1061	*Kabuli*	Ca_Kabuli_Chr01	10556610	(T/G)
1062	CakSNP1062	*Kabuli*	Ca_Kabuli_Chr01	10560693	(A/G)
1063	CakSNP1063	*Kabuli*	Ca_Kabuli_Chr01	10560690	(T/G)

S.N.	SNP IDs	Cultivars	Chromosomes/scaffolds	Physical positions (bp)	SNPs
1064	CakSNP1064	*Kabuli*	Ca_Kabuli_Chr01	10567303	(A/C)
1065	CakSNP1065	*Kabuli*	Ca_Kabuli_Chr01	10571949	(G/A)
1066	CakSNP1066	*Kabuli*	Ca_Kabuli_Chr01	10601318	(T/G)
1067	CakSNP1067	*Kabuli*	Ca_Kabuli_Chr01	10605977	(G/A)
1068	CakSNP1068	*Kabuli*	Ca_Kabuli_Chr01	10623359	(T/G)
1069	CakSNP1069	*Kabuli*	Ca_Kabuli_Chr01	10630658	(A/T)
1070	CakSNP1070	*Kabuli*	Ca_Kabuli_Chr01	10649758	(C/T)
1071	CakSNP1071	*Kabuli*	Ca_Kabuli_Chr01	10655633	(G/T)
1072	CakSNP1072	*Kabuli*	Ca_Kabuli_Chr01	10657464	(T/A)
1073	CakSNP1073	*Kabuli*	Ca_Kabuli_Chr01	10697972	(C/G)
1074	CakSNP1074	*Kabuli*	Ca_Kabuli_Chr01	10710672	(T/G)
1075	CakSNP1075	*Kabuli*	Ca_Kabuli_Chr01	10712235	(G/A)
1076	CakSNP1076	*Kabuli*	Ca_Kabuli_Chr01	10713001	(T/A)
1077	CakSNP1077	*Kabuli*	Ca_Kabuli_Chr01	10734511	(G/A)
1078	CakSNP1078	*Kabuli*	Ca_Kabuli_Chr01	10734570	(G/A)
1079	CakSNP1079	*Kabuli*	Ca_Kabuli_Chr01	10739374	(T/A)
1080	CakSNP1080	*Kabuli*	Ca_Kabuli_Chr01	10761609	(G/T)
1081	CakSNP1081	*Kabuli*	Ca_Kabuli_Chr01	10761648	(C/T)
1082	CakSNP1082	*Kabuli*	Ca_Kabuli_Chr01	10814166	(T/C)
1083	CakSNP1083	*Kabuli*	Ca_Kabuli_Chr01	10837203	(C/T)
1084	CakSNP1084	*Kabuli*	Ca_Kabuli_Chr01	10861334	(A/G)
1085	CakSNP1085	*Kabuli*	Ca_Kabuli_Chr01	10861332	(C/G)
1086	CakSNP1086	*Kabuli*	Ca_Kabuli_Chr01	10861326	(T/G)
1087	CakSNP1087	*Kabuli*	Ca_Kabuli_Chr01	10869338	(A/T)
1088	CakSNP1088	*Kabuli*	Ca_Kabuli_Chr01	10869336	(G/A)
1089	CakSNP1089	*Kabuli*	Ca_Kabuli_Chr01	10887927	(C/A)
1090	CakSNP1090	*Kabuli*	Ca_Kabuli_Chr01	10939428	(T/C)
1091	CakSNP1091	*Kabuli*	Ca_Kabuli_Chr01	10973356	(C/T)
1092	CakSNP1092	*Kabuli*	Ca_Kabuli_Chr01	11065411	(T/A)
1093	CakSNP1093	*Kabuli*	Ca_Kabuli_Chr01	11103176	(T/C)
1094	CakSNP1094	*Kabuli*	Ca_Kabuli_Chr01	11121162	(G/A)
1095	CakSNP1095	*Kabuli*	Ca_Kabuli_Chr01	11122665	(G/T)
1096	CakSNP1096	*Kabuli*	Ca_Kabuli_Chr01	11191023	(G/A)
1097	CakSNP1097	*Kabuli*	Ca_Kabuli_Chr01	11193334	(G/C)
1098	CakSNP1098	*Kabuli*	Ca_Kabuli_Chr01	11225828	(T/C)
1099	CakSNP1099	*Kabuli*	Ca_Kabuli_Chr01	11227614	(T/C)
1100	CakSNP1100	*Kabuli*	Ca_Kabuli_Chr01	11230086	(A/G)
1101	CakSNP1101	*Kabuli*	Ca_Kabuli_Chr01	11230109	(T/C)

S.N.	SNP IDs	Cultivars	Chromosomes/scaffolds	Physical positions (bp)	SNPs
1102	CakSNP1102	*Kabuli*	Ca_Kabuli_Chr01	11230115	(G/C)
1103	CakSNP1103	*Kabuli*	Ca_Kabuli_Chr01	11230116	(T/C)
1104	CakSNP1104	*Kabuli*	Ca_Kabuli_Chr01	11230118	(A/T)
1105	CakSNP1105	*Kabuli*	Ca_Kabuli_Chr01	11230125	(A/C)
1106	CakSNP1106	*Kabuli*	Ca_Kabuli_Chr01	11230126	(G/T)
1107	CakSNP1107	*Kabuli*	Ca_Kabuli_Chr01	11230127	(G/T)
1108	CakSNP1108	*Kabuli*	Ca_Kabuli_Chr01	11230128	(T/G)
1109	CakSNP1109	*Kabuli*	Ca_Kabuli_Chr01	11230133	(G/A)
1110	CakSNP1110	*Kabuli*	Ca_Kabuli_Chr01	11230972	(C/T)
1111	CakSNP1111	*Kabuli*	Ca_Kabuli_Chr01	11230998	(A/T)
1112	CakSNP1112	*Kabuli*	Ca_Kabuli_Chr01	11231052	(A/G)
1113	CakSNP1113	*Kabuli*	Ca_Kabuli_Chr01	11231064	(A/G)
1114	CakSNP1114	*Kabuli*	Ca_Kabuli_Chr01	11231077	(T/C)
1115	CakSNP1115	*Kabuli*	Ca_Kabuli_Chr01	11235211	(C/T)
1116	CakSNP1116	*Kabuli*	Ca_Kabuli_Chr01	11265367	(T/C)
1117	CakSNP1117	*Kabuli*	Ca_Kabuli_Chr01	11277703	(T/A)
1118	CakSNP1118	*Kabuli*	Ca_Kabuli_Chr01	11277964	(C/T)
1119	CakSNP1119	*Kabuli*	Ca_Kabuli_Chr01	11284183	(T/C)
1120	CakSNP1120	*Kabuli*	Ca_Kabuli_Chr01	11285192	(G/A)
1121	CakSNP1121	*Kabuli*	Ca_Kabuli_Chr01	11285247	(T/C)
1122	CakSNP1122	*Kabuli*	Ca_Kabuli_Chr01	11285291	(C/A)
1123	CakSNP1123	*Kabuli*	Ca_Kabuli_Chr01	11291516	(G/A)
1124	CakSNP1124	*Kabuli*	Ca_Kabuli_Chr01	11291741	(A/C)
1125	CakSNP1125	*Kabuli*	Ca_Kabuli_Chr01	11291740	(T/A)
1126	CakSNP1126	*Kabuli*	Ca_Kabuli_Chr01	11291737	(C/A)
1127	CakSNP1127	*Kabuli*	Ca_Kabuli_Chr01	11319264	(A/C)
1128	CakSNP1128	*Kabuli*	Ca_Kabuli_Chr01	11321670	(G/C)
1129	CakSNP1129	*Kabuli*	Ca_Kabuli_Chr01	11324018	(C/A)
1130	CakSNP1130	*Kabuli*	Ca_Kabuli_Chr01	11324023	(C/T)
1131	CakSNP1131	*Kabuli*	Ca_Kabuli_Chr01	11324035	(C/T)
1132	CakSNP1132	*Kabuli*	Ca_Kabuli_Chr01	11324050	(G/T)
1133	CakSNP1133	*Kabuli*	Ca_Kabuli_Chr01	11342388	(T/C)
1134	CakSNP1134	*Kabuli*	Ca_Kabuli_Chr01	11361646	(G/A)
1135	CakSNP1135	*Kabuli*	Ca_Kabuli_Chr01	11361588	(T/G)
1136	CakSNP1136	*Kabuli*	Ca_Kabuli_Chr01	11361583	(T/C)
1137	CakSNP1137	*Kabuli*	Ca_Kabuli_Chr01	11374707	(G/A)
1138	CakSNP1138	*Kabuli*	Ca_Kabuli_Chr01	11405015	(A/G)
1139	CakSNP1139	*Kabuli*	Ca_Kabuli_Chr01	11405118	(G/A)

S.N.	SNP IDs	Cultivars	Chromosomes/scaffolds	Physical positions (bp)	SNPs
1140	CakSNP1140	*Kabuli*	Ca_Kabuli_Chr01	11423677	(C/A)
1141	CakSNP1141	*Kabuli*	Ca_Kabuli_Chr01	11487689	(A/G)
1142	CakSNP1142	*Kabuli*	Ca_Kabuli_Chr01	11487630	(C/A)
1143	CakSNP1143	*Kabuli*	Ca_Kabuli_Chr01	11487752	(G/T)
1144	CakSNP1144	*Kabuli*	Ca_Kabuli_Chr01	11502148	(C/A)
1145	CakSNP1145	*Kabuli*	Ca_Kabuli_Chr01	11514888	(T/A)
1146	CakSNP1146	*Kabuli*	Ca_Kabuli_Chr01	11514898	(T/C)
1147	CakSNP1147	*Kabuli*	Ca_Kabuli_Chr01	11514903	(A/G)
1148	CakSNP1148	*Kabuli*	Ca_Kabuli_Chr01	11515055	(G/A)
1149	CakSNP1149	*Kabuli*	Ca_Kabuli_Chr01	11515130	(T/G)
1150	CakSNP1150	*Kabuli*	Ca_Kabuli_Chr01	11522656	(G/T)
1151	CakSNP1151	*Kabuli*	Ca_Kabuli_Chr01	11522743	(T/C)
1152	CakSNP1152	*Kabuli*	Ca_Kabuli_Chr01	11522875	(C/T)
1153	CakSNP1153	*Kabuli*	Ca_Kabuli_Chr01	11601347	(T/C)
1154	CakSNP1154	*Kabuli*	Ca_Kabuli_Chr01	11624419	(C/G)
1155	CakSNP1155	*Kabuli*	Ca_Kabuli_Chr01	11624464	(C/T)
1156	CakSNP1156	*Kabuli*	Ca_Kabuli_Chr01	11669794	(T/G)
1157	CakSNP1157	*Kabuli*	Ca_Kabuli_Chr01	11673478	(A/C)
1158	CakSNP1158	*Kabuli*	Ca_Kabuli_Chr01	11677097	(C/A)
1159	CakSNP1159	*Kabuli*	Ca_Kabuli_Chr01	11685769	(A/G)
1160	CakSNP1160	*Kabuli*	Ca_Kabuli_Chr01	11685790	(T/C)
1161	CakSNP1161	*Kabuli*	Ca_Kabuli_Chr01	11706451	(C/T)
1162	CakSNP1162	*Kabuli*	Ca_Kabuli_Chr01	11706494	(G/C)
1163	CakSNP1163	*Kabuli*	Ca_Kabuli_Chr01	11706583	(A/C)
1164	CakSNP1164	*Kabuli*	Ca_Kabuli_Chr01	11706545	(C/G)
1165	CakSNP1165	*Kabuli*	Ca_Kabuli_Chr01	11741448	(T/A)
1166	CakSNP1166	*Kabuli*	Ca_Kabuli_Chr01	11793443	(C/A)
1167	CakSNP1167	*Kabuli*	Ca_Kabuli_Chr01	11822248	(A/T)
1168	CakSNP1168	*Kabuli*	Ca_Kabuli_Chr01	11822305	(G/T)
1169	CakSNP1169	*Kabuli*	Ca_Kabuli_Chr01	11822309	(C/A)
1170	CakSNP1170	*Kabuli*	Ca_Kabuli_Chr01	11870401	(A/G)
1171	CakSNP1171	*Kabuli*	Ca_Kabuli_Chr01	11935333	(A/T)
1172	CakSNP1172	*Kabuli*	Ca_Kabuli_Chr01	11955896	(G/T)
1173	CakSNP1173	*Kabuli*	Ca_Kabuli_Chr01	11955866	(G/A)
1174	CakSNP1174	*Kabuli*	Ca_Kabuli_Chr01	11955853	(A/T)
1175	CakSNP1175	*Kabuli*	Ca_Kabuli_Chr01	12054809	(C/T)
1176	CakSNP1176	*Kabuli*	Ca_Kabuli_Chr01	12189822	(T/G)
1177	CakSNP1177	*Kabuli*	Ca_Kabuli_Chr01	12189825	(T/G)

**S.N.**	**SNP IDs**	**Cultivars**	**Chromosomes/scaffolds**	**Physical positions (bp)**	**SNPs**
1178	CakSNP1178	*Kabuli*	Ca_Kabuli_Chr01	12193933	(T/G)
1179	CakSNP1179	*Kabuli*	Ca_Kabuli_Chr01	12199691	(C/G)
1180	CakSNP1180	*Kabuli*	Ca_Kabuli_Chr01	12236387	(T/A)
1181	CakSNP1181	*Kabuli*	Ca_Kabuli_Chr01	12264697	(T/G)
1182	CakSNP1182	*Kabuli*	Ca_Kabuli_Chr01	12326230	(A/G)
1183	CakSNP1183	*Kabuli*	Ca_Kabuli_Chr01	12326234	(T/A)
1184	CakSNP1184	*Kabuli*	Ca_Kabuli_Chr01	12326318	(C/G)
1185	CakSNP1185	*Kabuli*	Ca_Kabuli_Chr01	12334259	(C/T)
1186	CakSNP1186	*Kabuli*	Ca_Kabuli_Chr01	12340884	(C/T)
1187	CakSNP1187	*Kabuli*	Ca_Kabuli_Chr01	12390756	(G/T)
1188	CakSNP1188	*Kabuli*	Ca_Kabuli_Chr01	12390750	(T/C)
1189	CakSNP1189	*Kabuli*	Ca_Kabuli_Chr01	12391116	(G/C)
1190	CakSNP1190	*Kabuli*	Ca_Kabuli_Chr01	12391074	(G/A)
1191	CakSNP1191	*Kabuli*	Ca_Kabuli_Chr01	12391067	(C/A)
1192	CakSNP1192	*Kabuli*	Ca_Kabuli_Chr01	12413012	(G/T)
1193	CakSNP1193	*Kabuli*	Ca_Kabuli_Chr01	12413016	(T/A)
1194	CakSNP1194	*Kabuli*	Ca_Kabuli_Chr01	12413025	(G/C)
1195	CakSNP1195	*Kabuli*	Ca_Kabuli_Chr01	12413116	(G/A)
1196	CakSNP1196	*Kabuli*	Ca_Kabuli_Chr01	12414040	(G/A)
1197	CakSNP1197	*Kabuli*	Ca_Kabuli_Chr01	12481879	(C/A)
1198	CakSNP1198	*Kabuli*	Ca_Kabuli_Chr01	12525445	(A/G)
1199	CakSNP1199	*Kabuli*	Ca_Kabuli_Chr01	12525640	(G/C)
1200	CakSNP1200	*Kabuli*	Ca_Kabuli_Chr01	12566565	(C/T)
1201	CakSNP1201	*Kabuli*	Ca_Kabuli_Chr01	12566579	(C/T)
1202	CakSNP1202	*Kabuli*	Ca_Kabuli_Chr01	12566607	(T/C)
1203	CakSNP1203	*Kabuli*	Ca_Kabuli_Chr01	12566615	(C/T)
1204	CakSNP1204	*Kabuli*	Ca_Kabuli_Chr01	12566641	(T/C)
1205	CakSNP1205	*Kabuli*	Ca_Kabuli_Chr01	12588525	(A/G)
1206	CakSNP1206	*Kabuli*	Ca_Kabuli_Chr01	12597939	(A/T)
1207	CakSNP1207	*Kabuli*	Ca_Kabuli_Chr01	12639552	(T/A)
1208	CakSNP1208	*Kabuli*	Ca_Kabuli_Chr01	12639584	(T/C)
1209	CakSNP1209	*Kabuli*	Ca_Kabuli_Chr01	12687506	(C/T)
1210	CakSNP1210	*Kabuli*	Ca_Kabuli_Chr01	12688424	(A/G)
1211	CakSNP1211	*Kabuli*	Ca_Kabuli_Chr01	12688435	(A/C)
1212	CakSNP1212	*Kabuli*	Ca_Kabuli_Chr01	12704406	(G/T)
1213	CakSNP1213	*Kabuli*	Ca_Kabuli_Chr01	12705665	(C/T)
1214	CakSNP1214	*Kabuli*	Ca_Kabuli_Chr01	12705731	(G/A)
1215	CakSNP1215	*Kabuli*	Ca_Kabuli_Chr01	12715427	(C/T)

S.N.	SNP IDs	Cultivars	Chromosomes/scaffolds	Physical positions (bp)	SNPs
1216	CakSNP1216	*Kabuli*	Ca_Kabuli_Chr01	12719734	(T/A)
1217	CakSNP1217	*Kabuli*	Ca_Kabuli_Chr01	12719735	(G/T)
1218	CakSNP1218	*Kabuli*	Ca_Kabuli_Chr01	12719752	(G/C)
1219	CakSNP1219	*Kabuli*	Ca_Kabuli_Chr01	12736298	(A/G)
1220	CakSNP1220	*Kabuli*	Ca_Kabuli_Chr01	12736353	(A/C)
1221	CakSNP1221	*Kabuli*	Ca_Kabuli_Chr01	12736622	(T/G)
1222	CakSNP1222	*Kabuli*	Ca_Kabuli_Chr01	12738564	(C/T)
1223	CakSNP1223	*Kabuli*	Ca_Kabuli_Chr01	12754422	(G/T)
1224	CakSNP1224	*Kabuli*	Ca_Kabuli_Chr01	12794075	(C/A)
1225	CakSNP1225	*Kabuli*	Ca_Kabuli_Chr01	12794147	(G/T)
1226	CakSNP1226	*Kabuli*	Ca_Kabuli_Chr01	12794251	(A/C)
1227	CakSNP1227	*Kabuli*	Ca_Kabuli_Chr01	12800638	(T/C)
1228	CakSNP1228	*Kabuli*	Ca_Kabuli_Chr01	12800610	(A/C)
1229	CakSNP1229	*Kabuli*	Ca_Kabuli_Chr01	12800607	(T/A)
1230	CakSNP1230	*Kabuli*	Ca_Kabuli_Chr01	12800601	(T/A)
1231	CakSNP1231	*Kabuli*	Ca_Kabuli_Chr01	12800597	(G/T)
1232	CakSNP1232	*Kabuli*	Ca_Kabuli_Chr01	12831717	(G/T)
1233	CakSNP1233	*Kabuli*	Ca_Kabuli_Chr01	12845445	(A/C)
1234	CakSNP1234	*Kabuli*	Ca_Kabuli_Chr01	12875506	(G/A)
1235	CakSNP1235	*Kabuli*	Ca_Kabuli_Chr01	12880408	(G/A)
1236	CakSNP1236	*Kabuli*	Ca_Kabuli_Chr01	12912621	(C/T)
1237	CakSNP1237	*Kabuli*	Ca_Kabuli_Chr01	12912625	(T/C)
1238	CakSNP1238	*Kabuli*	Ca_Kabuli_Chr01	12912670	(C/T)
1239	CakSNP1239	*Kabuli*	Ca_Kabuli_Chr01	12947377	(C/T)
1240	CakSNP1240	*Kabuli*	Ca_Kabuli_Chr01	12947376	(C/T)
1241	CakSNP1241	*Kabuli*	Ca_Kabuli_Chr01	12947358	(A/T)
1242	CakSNP1242	*Kabuli*	Ca_Kabuli_Chr01	12947347	(A/G)
1243	CakSNP1243	*Kabuli*	Ca_Kabuli_Chr01	12992445	(A/C)
1244	CakSNP1244	*Kabuli*	Ca_Kabuli_Chr01	12992423	(A/C)
1245	CakSNP1245	*Kabuli*	Ca_Kabuli_Chr01	12992569	(A/G)
1246	CakSNP1246	*Kabuli*	Ca_Kabuli_Chr01	13028543	(T/C)
1247	CakSNP1247	*Kabuli*	Ca_Kabuli_Chr01	13028517	(G/A)
1248	CakSNP1248	*Kabuli*	Ca_Kabuli_Chr01	13028505	(A/G)
1249	CakSNP1249	*Kabuli*	Ca_Kabuli_Chr01	13034708	(T/G)
1250	CakSNP1250	*Kabuli*	Ca_Kabuli_Chr01	13034810	(G/A)
1251	CakSNP1251	*Kabuli*	Ca_Kabuli_Chr01	13040664	(T/C)
1252	CakSNP1252	*Kabuli*	Ca_Kabuli_Chr01	13066954	(C/T)
1253	CakSNP1253	*Kabuli*	Ca_Kabuli_Chr01	13066968	(C/G)

S.N.	SNP IDs	Cultivars	Chromosomes/scaffolds	Physical positions (bp)	SNPs
1254	CakSNP1254	*Kabuli*	Ca_Kabuli_Chr01	13076647	(T/A)
1255	CakSNP1255	*Kabuli*	Ca_Kabuli_Chr01	13076695	(G/A)
1256	CakSNP1256	*Kabuli*	Ca_Kabuli_Chr01	13156473	(A/C)
1257	CakSNP1257	*Kabuli*	Ca_Kabuli_Chr01	13158244	(C/T)
1258	CakSNP1258	*Kabuli*	Ca_Kabuli_Chr01	13158201	(C/T)
1259	CakSNP1259	*Kabuli*	Ca_Kabuli_Chr01	13158192	(T/C)
1260	CakSNP1260	*Kabuli*	Ca_Kabuli_Chr01	13199761	(T/A)
1261	CakSNP1261	*Kabuli*	Ca_Kabuli_Chr01	13202986	(T/G)
1262	CakSNP1262	*Kabuli*	Ca_Kabuli_Chr01	13202991	(T/G)
1263	CakSNP1263	*Kabuli*	Ca_Kabuli_Chr01	13216776	(G/T)
1264	CakSNP1264	*Kabuli*	Ca_Kabuli_Chr01	13216741	(T/A)
1265	CakSNP1265	*Kabuli*	Ca_Kabuli_Chr01	13216710	(G/A)
1266	CakSNP1266	*Kabuli*	Ca_Kabuli_Chr01	13267896	(T/C)
1267	CakSNP1267	*Kabuli*	Ca_Kabuli_Chr01	13282556	(A/G)
1268	CakSNP1268	*Kabuli*	Ca_Kabuli_Chr01	13282552	(T/C)
1269	CakSNP1269	*Kabuli*	Ca_Kabuli_Chr01	13316992	(C/A)
1270	CakSNP1270	*Kabuli*	Ca_Kabuli_Chr01	13331955	(T/A)
1271	CakSNP1271	*Kabuli*	Ca_Kabuli_Chr01	13331941	(C/T)
1272	CakSNP1272	*Kabuli*	Ca_Kabuli_Chr01	13332015	(C/A)
1273	CakSNP1273	*Kabuli*	Ca_Kabuli_Chr01	13332025	(T/G)
1274	CakSNP1274	*Kabuli*	Ca_Kabuli_Chr01	13365594	(T/A)
1275	CakSNP1275	*Kabuli*	Ca_Kabuli_Chr01	13365625	(C/G)
1276	CakSNP1276	*Kabuli*	Ca_Kabuli_Chr01	13368341	(G/A)
1277	CakSNP1277	*Kabuli*	Ca_Kabuli_Chr01	13368556	(T/A)
1278	CakSNP1278	*Kabuli*	Ca_Kabuli_Chr01	13369730	(G/A)
1279	CakSNP1279	*Kabuli*	Ca_Kabuli_Chr01	13369986	(C/A)
1280	CakSNP1280	*Kabuli*	Ca_Kabuli_Chr01	13497572	(G/T)
1281	CakSNP1281	*Kabuli*	Ca_Kabuli_Chr01	13549635	(T/C)
1282	CakSNP1282	*Kabuli*	Ca_Kabuli_Chr01	13550600	(G/A)
1283	CakSNP1283	*Kabuli*	Ca_Kabuli_Chr01	13560364	(T/G)
1284	CakSNP1284	*Kabuli*	Ca_Kabuli_Chr01	13590903	(G/T)
1285	CakSNP1285	*Kabuli*	Ca_Kabuli_Chr01	13591177	(G/A)
1286	CakSNP1286	*Kabuli*	Ca_Kabuli_Chr01	13591203	(C/G)
1287	CakSNP1287	*Kabuli*	Ca_Kabuli_Chr01	13591314	(C/T)
1288	CakSNP1288	*Kabuli*	Ca_Kabuli_Chr01	13593931	(T/A)
1289	CakSNP1289	*Kabuli*	Ca_Kabuli_Chr01	13596411	(G/T)
1290	CakSNP1290	*Kabuli*	Ca_Kabuli_Chr01	13596528	(T/A)
1291	CakSNP1291	*Kabuli*	Ca_Kabuli_Chr01	13596539	(A/G)

S.N.	SNP IDs	Cultivars	Chromosomes/scaffolds	Physical positions (bp)	SNPs
1292	CakSNP1292	*Kabuli*	Ca_Kabuli_Chr01	13596666	(T/A)
1293	CakSNP1293	*Kabuli*	Ca_Kabuli_Chr01	13596659	(T/A)
1294	CakSNP1294	*Kabuli*	Ca_Kabuli_Chr01	13596631	(C/T)
1295	CakSNP1295	*Kabuli*	Ca_Kabuli_Chr01	13596621	(C/T)
1296	CakSNP1296	*Kabuli*	Ca_Kabuli_Chr01	13596741	(C/T)
1297	CakSNP1297	*Kabuli*	Ca_Kabuli_Chr01	13596771	(C/A)
1298	CakSNP1298	*Kabuli*	Ca_Kabuli_Chr01	13607486	(A/C)
1299	CakSNP1299	*Kabuli*	Ca_Kabuli_Chr01	13608414	(T/C)
1300	CakSNP1300	*Kabuli*	Ca_Kabuli_Chr01	13637705	(G/T)
1301	CakSNP1301	*Kabuli*	Ca_Kabuli_Chr01	13637688	(A/G)
1302	CakSNP1302	*Kabuli*	Ca_Kabuli_Chr01	13637687	(C/T)
1303	CakSNP1303	*Kabuli*	Ca_Kabuli_Chr01	13637684	(C/T)
1304	CakSNP1304	*Kabuli*	Ca_Kabuli_Chr01	13639157	(T/C)
1305	CakSNP1305	*Kabuli*	Ca_Kabuli_Chr01	13641517	(A/G)
1306	CakSNP1306	*Kabuli*	Ca_Kabuli_Chr01	13641644	(G/A)
1307	CakSNP1307	*Kabuli*	Ca_Kabuli_Chr01	13641615	(A/G)
1308	CakSNP1308	*Kabuli*	Ca_Kabuli_Chr01	13641608	(G/T)
1309	CakSNP1309	*Kabuli*	Ca_Kabuli_Chr01	13641574	(A/C)
1310	CakSNP1310	*Kabuli*	Ca_Kabuli_Chr01	13641564	(G/A)
1311	CakSNP1311	*Kabuli*	Ca_Kabuli_Chr01	13668759	(T/A)
1312	CakSNP1312	*Kabuli*	Ca_Kabuli_Chr01	13680002	(G/T)
1313	CakSNP1313	*Kabuli*	Ca_Kabuli_Chr01	13680008	(G/C)
1314	CakSNP1314	*Kabuli*	Ca_Kabuli_Chr01	13762410	(C/A)
1315	CakSNP1315	*Kabuli*	Ca_Kabuli_Chr01	13769805	(C/T)
1316	CakSNP1316	*Kabuli*	Ca_Kabuli_Chr01	13769771	(T/C)
1317	CakSNP1317	*Kabuli*	Ca_Kabuli_Chr01	13782049	(C/T)
1318	CakSNP1318	*Kabuli*	Ca_Kabuli_Chr01	13814790	(T/C)
1319	CakSNP1319	*Kabuli*	Ca_Kabuli_Chr01	13828703	(C/T)
1320	CakSNP1320	*Kabuli*	Ca_Kabuli_Chr01	13828659	(G/A)
1321	CakSNP1321	*Kabuli*	Ca_Kabuli_Chr01	13828657	(G/A)
1322	CakSNP1322	*Kabuli*	Ca_Kabuli_Chr01	13828744	(A/T)
1323	CakSNP1323	*Kabuli*	Ca_Kabuli_Chr01	13991356	(T/G)
1324	CakSNP1324	*Kabuli*	Ca_Kabuli_Chr01	14014566	(C/A)
1325	CakSNP1325	*Kabuli*	Ca_Kabuli_Chr01	14015757	(T/C)
1326	CakSNP1326	*Kabuli*	Ca_Kabuli_Chr01	14015781	(G/C)
1327	CakSNP1327	*Kabuli*	Ca_Kabuli_Chr01	14015818	(C/T)
1328	CakSNP1328	*Kabuli*	Ca_Kabuli_Chr01	14022892	(C/A)
1329	CakSNP1329	*Kabuli*	Ca_Kabuli_Chr01	14022890	(A/T)

S.N.	SNP IDs	Cultivars	Chromosomes/scaffolds	Physical positions (bp)	SNPs
1330	CakSNP1330	*Kabuli*	Ca_Kabuli_Chr01	14023097	(A/C)
1331	CakSNP1331	*Kabuli*	Ca_Kabuli_Chr01	14023095	(T/A)
1332	CakSNP1332	*Kabuli*	Ca_Kabuli_Chr01	14023078	(T/A)
1333	CakSNP1333	*Kabuli*	Ca_Kabuli_Chr01	14025015	(G/A)
1334	CakSNP1334	*Kabuli*	Ca_Kabuli_Chr01	14025075	(A/G)
1335	CakSNP1335	*Kabuli*	Ca_Kabuli_Chr01	14026347	(C/G)
1336	CakSNP1336	*Kabuli*	Ca_Kabuli_Chr01	14026391	(T/C)
1337	CakSNP1337	*Kabuli*	Ca_Kabuli_Chr01	14026467	(C/T)
1338	CakSNP1338	*Kabuli*	Ca_Kabuli_Chr01	14026444	(G/T)
1339	CakSNP1339	*Kabuli*	Ca_Kabuli_Chr01	14026437	(C/A)
1340	CakSNP1340	*Kabuli*	Ca_Kabuli_Chr01	14033226	(C/T)
1341	CakSNP1341	*Kabuli*	Ca_Kabuli_Chr01	14033285	(C/T)
1342	CakSNP1342	*Kabuli*	Ca_Kabuli_Chr01	14033277	(G/C)
1343	CakSNP1343	*Kabuli*	Ca_Kabuli_Chr01	14033951	(G/A)
1344	CakSNP1344	*Kabuli*	Ca_Kabuli_Chr01	14034011	(A/G)
1345	CakSNP1345	*Kabuli*	Ca_Kabuli_Chr01	14045110	(T/C)
1346	CakSNP1346	*Kabuli*	Ca_Kabuli_Chr01	14045239	(A/G)
1347	CakSNP1347	*Kabuli*	Ca_Kabuli_Chr01	14076924	(T/C)
1348	CakSNP1348	*Kabuli*	Ca_Kabuli_Chr01	14101608	(C/T)
1349	CakSNP1349	*Kabuli*	Ca_Kabuli_Chr01	14101621	(T/C)
1350	CakSNP1350	*Kabuli*	Ca_Kabuli_Chr01	14101624	(C/T)
1351	CakSNP1351	*Kabuli*	Ca_Kabuli_Chr01	14101644	(A/G)
1352	CakSNP1352	*Kabuli*	Ca_Kabuli_Chr01	14163638	(C/T)
1353	CakSNP1353	*Kabuli*	Ca_Kabuli_Chr01	14163721	(T/C)
1354	CakSNP1354	*Kabuli*	Ca_Kabuli_Chr01	14163836	(A/C)
1355	CakSNP1355	*Kabuli*	Ca_Kabuli_Chr01	14225210	(G/T)
1356	CakSNP1356	*Kabuli*	Ca_Kabuli_Chr01	14237163	(T/G)
1357	CakSNP1357	*Kabuli*	Ca_Kabuli_Chr01	14237164	(T/A)
1358	CakSNP1358	*Kabuli*	Ca_Kabuli_Chr01	14237273	(A/T)
1359	CakSNP1359	*Kabuli*	Ca_Kabuli_Chr01	14240571	(C/T)
1360	CakSNP1360	*Kabuli*	Ca_Kabuli_Chr01	14259435	(G/C)
1361	CakSNP1361	*Kabuli*	Ca_Kabuli_Chr01	14259455	(A/G)
1362	CakSNP1362	*Kabuli*	Ca_Kabuli_Chr01	14281643	(T/C)
1363	CakSNP1363	*Kabuli*	Ca_Kabuli_Chr01	14348207	(C/T)
1364	CakSNP1364	*Kabuli*	Ca_Kabuli_Chr01	14393255	(A/G)
1365	CakSNP1365	*Kabuli*	Ca_Kabuli_Chr01	14393313	(C/A)
1366	CakSNP1366	*Kabuli*	Ca_Kabuli_Chr01	14459908	(T/C)
1367	CakSNP1367	*Kabuli*	Ca_Kabuli_Chr01	14576525	(C/T)

S.N.	SNP IDs	Cultivars	Chromosomes/scaffolds	Physical positions (bp)	SNPs
1368	CakSNP1368	*Kabuli*	Ca_Kabuli_Chr01	14623271	(T/C)
1369	CakSNP1369	*Kabuli*	Ca_Kabuli_Chr01	14698461	(A/T)
1370	CakSNP1370	*Kabuli*	Ca_Kabuli_Chr01	14801406	(A/G)
1371	CakSNP1371	*Kabuli*	Ca_Kabuli_Chr01	14884940	(G/T)
1372	CakSNP1372	*Kabuli*	Ca_Kabuli_Chr01	14916633	(G/C)
1373	CakSNP1373	*Kabuli*	Ca_Kabuli_Chr01	15126244	(T/C)
1374	CakSNP1374	*Kabuli*	Ca_Kabuli_Chr01	15137319	(C/T)
1375	CakSNP1375	*Kabuli*	Ca_Kabuli_Chr01	15146123	(A/G)
1376	CakSNP1376	*Kabuli*	Ca_Kabuli_Chr01	15146149	(G/A)
1377	CakSNP1377	*Kabuli*	Ca_Kabuli_Chr01	15167641	(T/C)
1378	CakSNP1378	*Kabuli*	Ca_Kabuli_Chr01	15167643	(G/A)
1379	CakSNP1379	*Kabuli*	Ca_Kabuli_Chr01	15196154	(T/A)
1380	CakSNP1380	*Kabuli*	Ca_Kabuli_Chr01	15236202	(A/G)
1381	CakSNP1381	*Kabuli*	Ca_Kabuli_Chr01	15253283	(G/A)
1382	CakSNP1382	*Kabuli*	Ca_Kabuli_Chr01	15253285	(A/G)
1383	CakSNP1383	*Kabuli*	Ca_Kabuli_Chr01	15291748	(C/T)
1384	CakSNP1384	*Kabuli*	Ca_Kabuli_Chr01	15319181	(T/C)
1385	CakSNP1385	*Kabuli*	Ca_Kabuli_Chr01	15611784	(T/C)
1386	CakSNP1386	*Kabuli*	Ca_Kabuli_Chr01	15613071	(A/C)
1387	CakSNP1387	*Kabuli*	Ca_Kabuli_Chr01	15683091	(G/A)
1388	CakSNP1388	*Kabuli*	Ca_Kabuli_Chr01	15683101	(A/C)
1389	CakSNP1389	*Kabuli*	Ca_Kabuli_Chr01	15683106	(C/A)
1390	CakSNP1390	*Kabuli*	Ca_Kabuli_Chr01	15683117	(G/T)
1391	CakSNP1391	*Kabuli*	Ca_Kabuli_Chr01	15683138	(A/G)
1392	CakSNP1392	*Kabuli*	Ca_Kabuli_Chr01	15760638	(C/A)
1393	CakSNP1393	*Kabuli*	Ca_Kabuli_Chr01	15784021	(C/T)
1394	CakSNP1394	*Kabuli*	Ca_Kabuli_Chr01	15802393	(G/A)
1395	CakSNP1395	*Kabuli*	Ca_Kabuli_Chr01	15862493	(C/T)
1396	CakSNP1396	*Kabuli*	Ca_Kabuli_Chr01	15960425	(A/C)
1397	CakSNP1397	*Kabuli*	Ca_Kabuli_Chr01	15965278	(G/A)
1398	CakSNP1398	*Kabuli*	Ca_Kabuli_Chr01	16061242	(A/G)
1399	CakSNP1399	*Kabuli*	Ca_Kabuli_Chr01	16061293	(C/G)
1400	CakSNP1400	*Kabuli*	Ca_Kabuli_Chr01	16061294	(T/A)
1401	CakSNP1401	*Kabuli*	Ca_Kabuli_Chr01	16061295	(A/G)
1402	CakSNP1402	*Kabuli*	Ca_Kabuli_Chr01	16061300	(A/G)
1403	CakSNP1403	*Kabuli*	Ca_Kabuli_Chr01	16061385	(A/G)
1404	CakSNP1404	*Kabuli*	Ca_Kabuli_Chr01	16061486	(G/A)
1405	CakSNP1405	*Kabuli*	Ca_Kabuli_Chr01	16061541	(G/A)

S.N.	SNP IDs	Cultivars	Chromosomes/scaffolds	Physical positions (bp)	SNPs
1406	CakSNP1406	*Kabuli*	Ca_Kabuli_Chr01	16061536	(A/C)
1407	CakSNP1407	*Kabuli*	Ca_Kabuli_Chr01	16081438	(T/C)
1408	CakSNP1408	*Kabuli*	Ca_Kabuli_Chr01	16099437	(T/G)
1409	CakSNP1409	*Kabuli*	Ca_Kabuli_Chr01	16099438	(G/A)
1410	CakSNP1410	*Kabuli*	Ca_Kabuli_Chr01	16099468	(G/A)
1411	CakSNP1411	*Kabuli*	Ca_Kabuli_Chr01	16099444	(T/C)
1412	CakSNP1412	*Kabuli*	Ca_Kabuli_Chr01	16099459	(T/C)
1413	CakSNP1413	*Kabuli*	Ca_Kabuli_Chr01	16099457	(T/C)
1414	CakSNP1414	*Kabuli*	Ca_Kabuli_Chr01	16104255	(G/T)
1415	CakSNP1415	*Kabuli*	Ca_Kabuli_Chr01	16104351	(G/A)
1416	CakSNP1416	*Kabuli*	Ca_Kabuli_Chr01	16104334	(T/G)
1417	CakSNP1417	*Kabuli*	Ca_Kabuli_Chr01	16104298	(C/T)
1418	CakSNP1418	*Kabuli*	Ca_Kabuli_Chr01	16114607	(C/A)
1419	CakSNP1419	*Kabuli*	Ca_Kabuli_Chr01	16196930	(A/G)
1420	CakSNP1420	*Kabuli*	Ca_Kabuli_Chr01	16228049	(C/A)
1421	CakSNP1421	*Kabuli*	Ca_Kabuli_Chr01	16228039	(C/A)
1422	CakSNP1422	*Kabuli*	Ca_Kabuli_Chr01	16289397	(G/A)
1423	CakSNP1423	*Kabuli*	Ca_Kabuli_Chr01	16329161	(G/A)
1424	CakSNP1424	*Kabuli*	Ca_Kabuli_Chr01	16376254	(G/T)
1425	CakSNP1425	*Kabuli*	Ca_Kabuli_Chr01	16379239	(A/G)
1426	CakSNP1426	*Kabuli*	Ca_Kabuli_Chr01	16379258	(C/T)
1427	CakSNP1427	*Kabuli*	Ca_Kabuli_Chr01	16410186	(C/T)
1428	CakSNP1428	*Kabuli*	Ca_Kabuli_Chr01	16419469	(C/T)
1429	CakSNP1429	*Kabuli*	Ca_Kabuli_Chr01	16454432	(C/T)
1430	CakSNP1430	*Kabuli*	Ca_Kabuli_Chr01	16454487	(G/C)
1431	CakSNP1431	*Kabuli*	Ca_Kabuli_Chr01	16493894	(A/G)
1432	CakSNP1432	*Kabuli*	Ca_Kabuli_Chr01	16512011	(G/T)
1433	CakSNP1433	*Kabuli*	Ca_Kabuli_Chr01	16512026	(C/T)
1434	CakSNP1434	*Kabuli*	Ca_Kabuli_Chr01	16512171	(G/A)
1435	CakSNP1435	*Kabuli*	Ca_Kabuli_Chr01	16512195	(G/A)
1436	CakSNP1436	*Kabuli*	Ca_Kabuli_Chr01	16515112	(T/C)
1437	CakSNP1437	*Kabuli*	Ca_Kabuli_Chr01	16527347	(T/A)
1438	CakSNP1438	*Kabuli*	Ca_Kabuli_Chr01	16527366	(G/A)
1439	CakSNP1439	*Kabuli*	Ca_Kabuli_Chr01	16575167	(A/G)
1440	CakSNP1440	*Kabuli*	Ca_Kabuli_Chr01	16636587	(G/A)
1441	CakSNP1441	*Kabuli*	Ca_Kabuli_Chr01	16743569	(C/T)
1442	CakSNP1442	*Kabuli*	Ca_Kabuli_Chr01	16799112	(T/C)
1443	CakSNP1443	*Kabuli*	Ca_Kabuli_Chr01	16799142	(C/T)

S.N.	SNP IDs	Cultivars	Chromosomes/scaffolds	Physical positions (bp)	SNPs
1444	CakSNP1444	*Kabuli*	Ca_Kabuli_Chr01	16813600	(T/A)
1445	CakSNP1445	*Kabuli*	Ca_Kabuli_Chr01	16813603	(T/G)
1446	CakSNP1446	*Kabuli*	Ca_Kabuli_Chr01	16813604	(T/C)
1447	CakSNP1447	*Kabuli*	Ca_Kabuli_Chr01	16813657	(A/T)
1448	CakSNP1448	*Kabuli*	Ca_Kabuli_Chr01	16813616	(T/A)
1449	CakSNP1449	*Kabuli*	Ca_Kabuli_Chr01	16873356	(C/T)
1450	CakSNP1450	*Kabuli*	Ca_Kabuli_Chr01	16877812	(C/A)
1451	CakSNP1451	*Kabuli*	Ca_Kabuli_Chr01	16877829	(C/A)
1452	CakSNP1452	*Kabuli*	Ca_Kabuli_Chr01	16883153	(C/T)
1453	CakSNP1453	*Kabuli*	Ca_Kabuli_Chr01	16901798	(C/A)
1454	CakSNP1454	*Kabuli*	Ca_Kabuli_Chr01	16902129	(G/A)
1455	CakSNP1455	*Kabuli*	Ca_Kabuli_Chr01	16908636	(G/A)
1456	CakSNP1456	*Kabuli*	Ca_Kabuli_Chr01	16933423	(T/G)
1457	CakSNP1457	*Kabuli*	Ca_Kabuli_Chr01	16933431	(T/G)
1458	CakSNP1458	*Kabuli*	Ca_Kabuli_Chr01	17006391	(T/C)
1459	CakSNP1459	*Kabuli*	Ca_Kabuli_Chr01	17023111	(G/C)
1460	CakSNP1460	*Kabuli*	Ca_Kabuli_Chr01	17024083	(A/C)
1461	CakSNP1461	*Kabuli*	Ca_Kabuli_Chr01	17068798	(A/C)
1462	CakSNP1462	*Kabuli*	Ca_Kabuli_Chr01	17092204	(C/T)
1463	CakSNP1463	*Kabuli*	Ca_Kabuli_Chr01	17092404	(G/A)
1464	CakSNP1464	*Kabuli*	Ca_Kabuli_Chr01	17092413	(C/T)
1465	CakSNP1465	*Kabuli*	Ca_Kabuli_Chr01	17093640	(T/C)
1466	CakSNP1466	*Kabuli*	Ca_Kabuli_Chr01	17167019	(G/A)
1467	CakSNP1467	*Kabuli*	Ca_Kabuli_Chr01	17170443	(A/T)
1468	CakSNP1468	*Kabuli*	Ca_Kabuli_Chr01	17170727	(T/C)
1469	CakSNP1469	*Kabuli*	Ca_Kabuli_Chr01	17182711	(C/T)
1470	CakSNP1470	*Kabuli*	Ca_Kabuli_Chr01	17182697	(A/T)
1471	CakSNP1471	*Kabuli*	Ca_Kabuli_Chr01	17182867	(G/A)
1472	CakSNP1472	*Kabuli*	Ca_Kabuli_Chr01	17194835	(C/T)
1473	CakSNP1473	*Kabuli*	Ca_Kabuli_Chr01	17272338	(A/C)
1474	CakSNP1474	*Kabuli*	Ca_Kabuli_Chr01	17272401	(C/T)
1475	CakSNP1475	*Kabuli*	Ca_Kabuli_Chr01	17314880	(A/C)
1476	CakSNP1476	*Kabuli*	Ca_Kabuli_Chr01	17314911	(A/G)
1477	CakSNP1477	*Kabuli*	Ca_Kabuli_Chr01	17314945	(T/C)
1478	CakSNP1478	*Kabuli*	Ca_Kabuli_Chr01	17315046	(G/A)
1479	CakSNP1479	*Kabuli*	Ca_Kabuli_Chr01	17328721	(G/A)
1480	CakSNP1480	*Kabuli*	Ca_Kabuli_Chr01	17328808	(G/A)
1481	CakSNP1481	*Kabuli*	Ca_Kabuli_Chr01	17332981	(C/T)

S.N.	SNP IDs	Cultivars	Chromosomes/scaffolds	Physical positions (bp)	SNPs
1482	CakSNP1482	*Kabuli*	Ca_Kabuli_Chr01	17333056	(C/G)
1483	CakSNP1483	*Kabuli*	Ca_Kabuli_Chr01	17333049	(C/T)
1484	CakSNP1484	*Kabuli*	Ca_Kabuli_Chr01	17340321	(T/C)
1485	CakSNP1485	*Kabuli*	Ca_Kabuli_Chr01	17340350	(G/T)
1486	CakSNP1486	*Kabuli*	Ca_Kabuli_Chr01	17340398	(G/T)
1487	CakSNP1487	*Kabuli*	Ca_Kabuli_Chr01	17359712	(A/G)
1488	CakSNP1488	*Kabuli*	Ca_Kabuli_Chr01	17359688	(A/C)
1489	CakSNP1489	*Kabuli*	Ca_Kabuli_Chr01	17359686	(C/T)
1490	CakSNP1490	*Kabuli*	Ca_Kabuli_Chr01	17359682	(A/C)
1491	CakSNP1491	*Kabuli*	Ca_Kabuli_Chr01	17386549	(C/T)
1492	CakSNP1492	*Kabuli*	Ca_Kabuli_Chr01	17386603	(A/T)
1493	CakSNP1493	*Kabuli*	Ca_Kabuli_Chr01	17395543	(C/A)
1494	CakSNP1494	*Kabuli*	Ca_Kabuli_Chr01	17437336	(G/C)
1495	CakSNP1495	*Kabuli*	Ca_Kabuli_Chr01	17558885	(T/C)
1496	CakSNP1496	*Kabuli*	Ca_Kabuli_Chr01	17558940	(A/G)
1497	CakSNP1497	*Kabuli*	Ca_Kabuli_Chr01	17558975	(A/G)
1498	CakSNP1498	*Kabuli*	Ca_Kabuli_Chr01	17576793	(T/G)
1499	CakSNP1499	*Kabuli*	Ca_Kabuli_Chr01	17761244	(T/C)
1500	CakSNP1500	*Kabuli*	Ca_Kabuli_Chr01	17763949	(G/A)
1501	CakSNP1501	*Kabuli*	Ca_Kabuli_Chr01	17819374	(A/C)
1502	CakSNP1502	*Kabuli*	Ca_Kabuli_Chr01	17856892	(A/G)
1503	CakSNP1503	*Kabuli*	Ca_Kabuli_Chr01	17857089	(T/A)
1504	CakSNP1504	*Kabuli*	Ca_Kabuli_Chr01	17880143	(T/C)
1505	CakSNP1505	*Kabuli*	Ca_Kabuli_Chr01	17880185	(T/C)
1506	CakSNP1506	*Kabuli*	Ca_Kabuli_Chr01	17961779	(T/C)
1507	CakSNP1507	*Kabuli*	Ca_Kabuli_Chr01	17983301	(T/G)
1508	CakSNP1508	*Kabuli*	Ca_Kabuli_Chr01	17983310	(T/G)
1509	CakSNP1509	*Kabuli*	Ca_Kabuli_Chr01	18009665	(C/G)
1510	CakSNP1510	*Kabuli*	Ca_Kabuli_Chr01	18050590	(G/T)
1511	CakSNP1511	*Kabuli*	Ca_Kabuli_Chr01	18064930	(C/T)
1512	CakSNP1512	*Kabuli*	Ca_Kabuli_Chr01	18065239	(T/C)
1513	CakSNP1513	*Kabuli*	Ca_Kabuli_Chr01	18092704	(T/G)
1514	CakSNP1514	*Kabuli*	Ca_Kabuli_Chr01	18092761	(C/T)
1515	CakSNP1515	*Kabuli*	Ca_Kabuli_Chr01	18098516	(C/T)
1516	CakSNP1516	*Kabuli*	Ca_Kabuli_Chr01	18099331	(C/T)
1517	CakSNP1517	*Kabuli*	Ca_Kabuli_Chr01	18099334	(T/A)
1518	CakSNP1518	*Kabuli*	Ca_Kabuli_Chr01	18112318	(A/G)
1519	CakSNP1519	*Kabuli*	Ca_Kabuli_Chr01	18112353	(T/C)

S.N.	SNP IDs	Cultivars	Chromosomes/scaffolds	Physical positions (bp)	SNPs
1520	CakSNP1520	*Kabuli*	Ca_Kabuli_Chr01	18131516	(T/G)
1521	CakSNP1521	*Kabuli*	Ca_Kabuli_Chr01	18131545	(A/T)
1522	CakSNP1522	*Kabuli*	Ca_Kabuli_Chr01	18131898	(G/A)
1523	CakSNP1523	*Kabuli*	Ca_Kabuli_Chr01	18141990	(C/A)
1524	CakSNP1524	*Kabuli*	Ca_Kabuli_Chr01	18144107	(C/G)
1525	CakSNP1525	*Kabuli*	Ca_Kabuli_Chr01	18144137	(T/A)
1526	CakSNP1526	*Kabuli*	Ca_Kabuli_Chr01	18147011	(C/G)
1527	CakSNP1527	*Kabuli*	Ca_Kabuli_Chr01	18150151	(G/A)
1528	CakSNP1528	*Kabuli*	Ca_Kabuli_Chr01	18153870	(T/G)
1529	CakSNP1529	*Kabuli*	Ca_Kabuli_Chr01	18154083	(A/G)
1530	CakSNP1530	*Kabuli*	Ca_Kabuli_Chr01	18156634	(A/G)
1531	CakSNP1531	*Kabuli*	Ca_Kabuli_Chr01	18172762	(G/T)
1532	CakSNP1532	*Kabuli*	Ca_Kabuli_Chr01	18198444	(C/T)
1533	CakSNP1533	*Kabuli*	Ca_Kabuli_Chr01	18488172	(G/A)
1534	CakSNP1534	*Kabuli*	Ca_Kabuli_Chr01	18746045	(T/G)
1535	CakSNP1535	*Kabuli*	Ca_Kabuli_Chr01	18763916	(G/A)
1536	CakSNP1536	*Kabuli*	Ca_Kabuli_Chr01	18763911	(C/A)
1537	CakSNP1537	*Kabuli*	Ca_Kabuli_Chr01	18764225	(C/T)
1538	CakSNP1538	*Kabuli*	Ca_Kabuli_Chr01	18768563	(C/A)
1539	CakSNP1539	*Kabuli*	Ca_Kabuli_Chr01	18797330	(T/C)
1540	CakSNP1540	*Kabuli*	Ca_Kabuli_Chr01	18890559	(A/G)
1541	CakSNP1541	*Kabuli*	Ca_Kabuli_Chr01	18890616	(G/A)
1542	CakSNP1542	*Kabuli*	Ca_Kabuli_Chr01	18890833	(G/A)
1543	CakSNP1543	*Kabuli*	Ca_Kabuli_Chr01	18910213	(C/T)
1544	CakSNP1544	*Kabuli*	Ca_Kabuli_Chr01	18910202	(G/A)
1545	CakSNP1545	*Kabuli*	Ca_Kabuli_Chr01	18910173	(T/C)
1546	CakSNP1546	*Kabuli*	Ca_Kabuli_Chr01	19000445	(T/C)
1547	CakSNP1547	*Kabuli*	Ca_Kabuli_Chr01	19055085	(C/T)
1548	CakSNP1548	*Kabuli*	Ca_Kabuli_Chr01	19130894	(T/C)
1549	CakSNP1549	*Kabuli*	Ca_Kabuli_Chr01	19130884	(C/A)
1550	CakSNP1550	*Kabuli*	Ca_Kabuli_Chr01	19130883	(C/A)
1551	CakSNP1551	*Kabuli*	Ca_Kabuli_Chr01	19130872	(G/A)
1552	CakSNP1552	*Kabuli*	Ca_Kabuli_Chr01	19130833	(G/T)
1553	CakSNP1553	*Kabuli*	Ca_Kabuli_Chr01	19130831	(A/G)
1554	CakSNP1554	*Kabuli*	Ca_Kabuli_Chr01	19130848	(C/T)
1555	CakSNP1555	*Kabuli*	Ca_Kabuli_Chr01	19130853	(G/A)
1556	CakSNP1556	*Kabuli*	Ca_Kabuli_Chr01	19164976	(T/C)
1557	CakSNP1557	*Kabuli*	Ca_Kabuli_Chr01	19166597	(A/G)

S.N.	SNP IDs	Cultivars	Chromosomes/scaffolds	Physical positions (bp)	SNPs
1558	CakSNP1558	*Kabuli*	Ca_Kabuli_Chr01	19218067	(G/A)
1559	CakSNP1559	*Kabuli*	Ca_Kabuli_Chr01	19218093	(T/G)
1560	CakSNP1560	*Kabuli*	Ca_Kabuli_Chr01	19252952	(G/T)
1561	CakSNP1561	*Kabuli*	Ca_Kabuli_Chr01	19252938	(G/A)
1562	CakSNP1562	*Kabuli*	Ca_Kabuli_Chr01	19253655	(C/T)
1563	CakSNP1563	*Kabuli*	Ca_Kabuli_Chr01	19254161	(G/A)
1564	CakSNP1564	*Kabuli*	Ca_Kabuli_Chr01	19276489	(A/G)
1565	CakSNP1565	*Kabuli*	Ca_Kabuli_Chr01	19276697	(C/G)
1566	CakSNP1566	*Kabuli*	Ca_Kabuli_Chr01	19359812	(A/C)
1567	CakSNP1567	*Kabuli*	Ca_Kabuli_Chr01	19359844	(C/A)
1568	CakSNP1568	*Kabuli*	Ca_Kabuli_Chr01	19399112	(C/T)
1569	CakSNP1569	*Kabuli*	Ca_Kabuli_Chr01	19526320	(T/C)
1570	CakSNP1570	*Kabuli*	Ca_Kabuli_Chr01	19526531	(C/G)
1571	CakSNP1571	*Kabuli*	Ca_Kabuli_Chr01	19526548	(G/A)
1572	CakSNP1572	*Kabuli*	Ca_Kabuli_Chr01	19526561	(T/C)
1573	CakSNP1573	*Kabuli*	Ca_Kabuli_Chr01	19526592	(T/A)
1574	CakSNP1574	*Kabuli*	Ca_Kabuli_Chr01	19526652	(G/T)
1575	CakSNP1575	*Kabuli*	Ca_Kabuli_Chr01	19526632	(C/T)
1576	CakSNP1576	*Kabuli*	Ca_Kabuli_Chr01	19572408	(C/T)
1577	CakSNP1577	*Kabuli*	Ca_Kabuli_Chr01	19572494	(G/C)
1578	CakSNP1578	*Kabuli*	Ca_Kabuli_Chr01	19591671	(A/G)
1579	CakSNP1579	*Kabuli*	Ca_Kabuli_Chr01	19877654	(A/T)
1580	CakSNP1580	*Kabuli*	Ca_Kabuli_Chr01	19902735	(T/A)
1581	CakSNP1581	*Kabuli*	Ca_Kabuli_Chr01	19903665	(A/G)
1582	CakSNP1582	*Kabuli*	Ca_Kabuli_Chr01	19903683	(G/A)
1583	CakSNP1583	*Kabuli*	Ca_Kabuli_Chr01	19903686	(T/A)
1584	CakSNP1584	*Kabuli*	Ca_Kabuli_Chr01	19903690	(A/G)
1585	CakSNP1585	*Kabuli*	Ca_Kabuli_Chr01	19903693	(G/T)
1586	CakSNP1586	*Kabuli*	Ca_Kabuli_Chr01	19903701	(G/A)
1587	CakSNP1587	*Kabuli*	Ca_Kabuli_Chr01	19903704	(G/A)
1588	CakSNP1588	*Kabuli*	Ca_Kabuli_Chr01	20004655	(A/G)
1589	CakSNP1589	*Kabuli*	Ca_Kabuli_Chr01	20005747	(C/T)
1590	CakSNP1590	*Kabuli*	Ca_Kabuli_Chr01	20016525	(C/T)
1591	CakSNP1591	*Kabuli*	Ca_Kabuli_Chr01	20070045	(A/G)
1592	CakSNP1592	*Kabuli*	Ca_Kabuli_Chr01	20070197	(T/C)
1593	CakSNP1593	*Kabuli*	Ca_Kabuli_Chr01	20091291	(T/G)
1594	CakSNP1594	*Kabuli*	Ca_Kabuli_Chr01	20092571	(C/G)
1595	CakSNP1595	*Kabuli*	Ca_Kabuli_Chr01	20092677	(C/A)

S.N.	SNP IDs	Cultivars	Chromosomes/scaffolds	Physical positions (bp)	SNPs
1596	CakSNP1596	*Kabuli*	Ca_Kabuli_Chr01	21822975	(A/G)
1597	CakSNP1597	*Kabuli*	Ca_Kabuli_Chr01	21881133	(G/A)
1598	CakSNP1598	*Kabuli*	Ca_Kabuli_Chr01	21881112	(C/T)
1599	CakSNP1599	*Kabuli*	Ca_Kabuli_Chr01	21881103	(G/A)
1600	CakSNP1600	*Kabuli*	Ca_Kabuli_Chr01	21881094	(G/A)
1601	CakSNP1601	*Kabuli*	Ca_Kabuli_Chr01	21918747	(G/A)
1602	CakSNP1602	*Kabuli*	Ca_Kabuli_Chr01	22344003	(G/A)
1603	CakSNP1603	*Kabuli*	Ca_Kabuli_Chr01	22437033	(C/T)
1604	CakSNP1604	*Kabuli*	Ca_Kabuli_Chr01	22465555	(C/T)
1605	CakSNP1605	*Kabuli*	Ca_Kabuli_Chr01	22465692	(A/G)
1606	CakSNP1606	*Kabuli*	Ca_Kabuli_Chr01	22719581	(T/G)
1607	CakSNP1607	*Kabuli*	Ca_Kabuli_Chr01	22743361	(T/G)
1608	CakSNP1608	*Kabuli*	Ca_Kabuli_Chr01	23122951	(A/G)
1609	CakSNP1609	*Kabuli*	Ca_Kabuli_Chr01	23128921	(T/A)
1610	CakSNP1610	*Kabuli*	Ca_Kabuli_Chr01	23128979	(G/T)
1611	CakSNP1611	*Kabuli*	Ca_Kabuli_Chr01	23129094	(C/T)
1612	CakSNP1612	*Kabuli*	Ca_Kabuli_Chr01	23131178	(A/C)
1613	CakSNP1613	*Kabuli*	Ca_Kabuli_Chr01	23131914	(T/C)
1614	CakSNP1614	*Kabuli*	Ca_Kabuli_Chr01	23148736	(A/C)
1615	CakSNP1615	*Kabuli*	Ca_Kabuli_Chr01	23149053	(A/G)
1616	CakSNP1616	*Kabuli*	Ca_Kabuli_Chr01	23149096	(A/C)
1617	CakSNP1617	*Kabuli*	Ca_Kabuli_Chr01	23341499	(C/G)
1618	CakSNP1618	*Kabuli*	Ca_Kabuli_Chr01	23382483	(T/A)
1619	CakSNP1619	*Kabuli*	Ca_Kabuli_Chr01	23382454	(G/T)
1620	CakSNP1620	*Kabuli*	Ca_Kabuli_Chr01	23382664	(C/T)
1621	CakSNP1621	*Kabuli*	Ca_Kabuli_Chr01	23382645	(C/G)
1622	CakSNP1622	*Kabuli*	Ca_Kabuli_Chr01	23915127	(C/G)
1623	CakSNP1623	*Kabuli*	Ca_Kabuli_Chr01	23941438	(G/A)
1624	CakSNP1624	*Kabuli*	Ca_Kabuli_Chr01	23941518	(C/T)
1625	CakSNP1625	*Kabuli*	Ca_Kabuli_Chr01	24070908	(C/T)
1626	CakSNP1626	*Kabuli*	Ca_Kabuli_Chr01	24070968	(C/T)
1627	CakSNP1627	*Kabuli*	Ca_Kabuli_Chr01	24071007	(C/A)
1628	CakSNP1628	*Kabuli*	Ca_Kabuli_Chr01	24071014	(T/G)
1629	CakSNP1629	*Kabuli*	Ca_Kabuli_Chr01	24173273	(A/G)
1630	CakSNP1630	*Kabuli*	Ca_Kabuli_Chr01	24450415	(T/C)
1631	CakSNP1631	*Kabuli*	Ca_Kabuli_Chr01	24450511	(A/G)
1632	CakSNP1632	*Kabuli*	Ca_Kabuli_Chr01	24450483	(T/G)
1633	CakSNP1633	*Kabuli*	Ca_Kabuli_Chr01	24693506	(C/T)

S.N.	SNP IDs	Cultivars	Chromosomes/scaffolds	Physical positions (bp)	SNPs
1634	CakSNP1634	*Kabuli*	Ca_Kabuli_Chr01	24693602	(A/G)
1635	CakSNP1635	*Kabuli*	Ca_Kabuli_Chr01	24693601	(C/T)
1636	CakSNP1636	*Kabuli*	Ca_Kabuli_Chr01	24693586	(C/T)
1637	CakSNP1637	*Kabuli*	Ca_Kabuli_Chr01	24693571	(G/A)
1638	CakSNP1638	*Kabuli*	Ca_Kabuli_Chr01	24693550	(G/A)
1639	CakSNP1639	*Kabuli*	Ca_Kabuli_Chr01	24693533	(G/A)
1640	CakSNP1640	*Kabuli*	Ca_Kabuli_Chr01	24693521	(G/A)
1641	CakSNP1641	*Kabuli*	Ca_Kabuli_Chr01	24828169	(C/T)
1642	CakSNP1642	*Kabuli*	Ca_Kabuli_Chr01	24828245	(A/C)
1643	CakSNP1643	*Kabuli*	Ca_Kabuli_Chr01	24845148	(A/C)
1644	CakSNP1644	*Kabuli*	Ca_Kabuli_Chr01	24983908	(A/G)
1645	CakSNP1645	*Kabuli*	Ca_Kabuli_Chr01	25039836	(T/C)
1646	CakSNP1646	*Kabuli*	Ca_Kabuli_Chr01	25083482	(A/C)
1647	CakSNP1647	*Kabuli*	Ca_Kabuli_Chr01	25083534	(A/C)
1648	CakSNP1648	*Kabuli*	Ca_Kabuli_Chr01	25175124	(C/T)
1649	CakSNP1649	*Kabuli*	Ca_Kabuli_Chr01	25176162	(C/A)
1650	CakSNP1650	*Kabuli*	Ca_Kabuli_Chr01	25192363	(T/C)
1651	CakSNP1651	*Kabuli*	Ca_Kabuli_Chr01	25524382	(C/T)
1652	CakSNP1652	*Kabuli*	Ca_Kabuli_Chr01	25524425	(C/T)
1653	CakSNP1653	*Kabuli*	Ca_Kabuli_Chr01	25524441	(T/C)
1654	CakSNP1654	*Kabuli*	Ca_Kabuli_Chr01	25524459	(C/G)
1655	CakSNP1655	*Kabuli*	Ca_Kabuli_Chr01	25524532	(T/C)
1656	CakSNP1656	*Kabuli*	Ca_Kabuli_Chr01	25524519	(C/T)
1657	CakSNP1657	*Kabuli*	Ca_Kabuli_Chr01	25524481	(T/A)
1658	CakSNP1658	*Kabuli*	Ca_Kabuli_Chr01	25524480	(G/A)
1659	CakSNP1659	*Kabuli*	Ca_Kabuli_Chr01	25524473	(C/T)
1660	CakSNP1660	*Kabuli*	Ca_Kabuli_Chr01	25524465	(A/G)
1661	CakSNP1661	*Kabuli*	Ca_Kabuli_Chr01	25524460	(G/A)
1662	CakSNP1662	*Kabuli*	Ca_Kabuli_Chr01	25524458	(G/A)
1663	CakSNP1663	*Kabuli*	Ca_Kabuli_Chr01	25524456	(C/T)
1664	CakSNP1664	*Kabuli*	Ca_Kabuli_Chr01	25524455	(G/A)
1665	CakSNP1665	*Kabuli*	Ca_Kabuli_Chr01	25524453	(C/T)
1666	CakSNP1666	*Kabuli*	Ca_Kabuli_Chr01	25524451	(A/T)
1667	CakSNP1667	*Kabuli*	Ca_Kabuli_Chr01	25524450	(C/T)
1668	CakSNP1668	*Kabuli*	Ca_Kabuli_Chr01	25524464	(G/A)
1669	CakSNP1669	*Kabuli*	Ca_Kabuli_Chr01	25524478	(C/A)
1670	CakSNP1670	*Kabuli*	Ca_Kabuli_Chr01	25524488	(G/T)
1671	CakSNP1671	*Kabuli*	Ca_Kabuli_Chr01	25524498	(G/C)

S.N.	SNP IDs	Cultivars	Chromosomes/scaffolds	Physical positions (bp)	SNPs
1672	CakSNP1672	*Kabuli*	Ca_Kabuli_Chr01	25524505	(A/G)
1673	CakSNP1673	*Kabuli*	Ca_Kabuli_Chr01	25524520	(C/T)
1674	CakSNP1674	*Kabuli*	Ca_Kabuli_Chr01	25524522	(G/A)
1675	CakSNP1675	*Kabuli*	Ca_Kabuli_Chr01	25524524	(C/T)
1676	CakSNP1676	*Kabuli*	Ca_Kabuli_Chr01	25732004	(T/C)
1677	CakSNP1677	*Kabuli*	Ca_Kabuli_Chr01	25887539	(G/A)
1678	CakSNP1678	*Kabuli*	Ca_Kabuli_Chr01	25987201	(A/C)
1679	CakSNP1679	*Kabuli*	Ca_Kabuli_Chr01	26026817	(C/T)
1680	CakSNP1680	*Kabuli*	Ca_Kabuli_Chr01	26190096	(G/A)
1681	CakSNP1681	*Kabuli*	Ca_Kabuli_Chr01	26190050	(G/A)
1682	CakSNP1682	*Kabuli*	Ca_Kabuli_Chr01	26481730	(C/T)
1683	CakSNP1683	*Kabuli*	Ca_Kabuli_Chr01	26481747	(T/G)
1684	CakSNP1684	*Kabuli*	Ca_Kabuli_Chr01	26484868	(G/C)
1685	CakSNP1685	*Kabuli*	Ca_Kabuli_Chr01	26574098	(C/T)
1686	CakSNP1686	*Kabuli*	Ca_Kabuli_Chr01	26574146	(C/T)
1687	CakSNP1687	*Kabuli*	Ca_Kabuli_Chr01	26579621	(A/C)
1688	CakSNP1688	*Kabuli*	Ca_Kabuli_Chr01	26746094	(A/T)
1689	CakSNP1689	*Kabuli*	Ca_Kabuli_Chr01	26767522	(A/G)
1690	CakSNP1690	*Kabuli*	Ca_Kabuli_Chr01	26767514	(A/G)
1691	CakSNP1691	*Kabuli*	Ca_Kabuli_Chr01	26784404	(G/A)
1692	CakSNP1692	*Kabuli*	Ca_Kabuli_Chr01	26784421	(G/T)
1693	CakSNP1693	*Kabuli*	Ca_Kabuli_Chr01	26784422	(C/T)
1694	CakSNP1694	*Kabuli*	Ca_Kabuli_Chr01	26784464	(C/T)
1695	CakSNP1695	*Kabuli*	Ca_Kabuli_Chr01	26784548	(C/A)
1696	CakSNP1696	*Kabuli*	Ca_Kabuli_Chr01	26784570	(A/G)
1697	CakSNP1697	*Kabuli*	Ca_Kabuli_Chr01	26784592	(A/G)
1698	CakSNP1698	*Kabuli*	Ca_Kabuli_Chr01	26784618	(G/A)
1699	CakSNP1699	*Kabuli*	Ca_Kabuli_Chr01	26784732	(C/G)
1700	CakSNP1700	*Kabuli*	Ca_Kabuli_Chr01	26785566	(T/G)
1701	CakSNP1701	*Kabuli*	Ca_Kabuli_Chr01	26807401	(A/G)
1702	CakSNP1702	*Kabuli*	Ca_Kabuli_Chr01	26807395	(T/G)
1703	CakSNP1703	*Kabuli*	Ca_Kabuli_Chr01	26856541	(A/T)
1704	CakSNP1704	*Kabuli*	Ca_Kabuli_Chr01	26856691	(G/A)
1705	CakSNP1705	*Kabuli*	Ca_Kabuli_Chr01	26921471	(C/T)
1706	CakSNP1706	*Kabuli*	Ca_Kabuli_Chr01	26921487	(C/A)
1707	CakSNP1707	*Kabuli*	Ca_Kabuli_Chr01	26922011	(T/G)
1708	CakSNP1708	*Kabuli*	Ca_Kabuli_Chr01	26922631	(C/T)
1709	CakSNP1709	*Kabuli*	Ca_Kabuli_Chr01	26953535	(C/T)

S.N.	SNP IDs	Cultivars	Chromosomes/scaffolds	Physical positions (bp)	SNPs
1710	CakSNP1710	*Kabuli*	Ca_Kabuli_Chr01	27012495	(C/T)
1711	CakSNP1711	*Kabuli*	Ca_Kabuli_Chr01	27012660	(G/C)
1712	CakSNP1712	*Kabuli*	Ca_Kabuli_Chr01	27014895	(T/G)
1713	CakSNP1713	*Kabuli*	Ca_Kabuli_Chr01	27014981	(A/G)
1714	CakSNP1714	*Kabuli*	Ca_Kabuli_Chr01	27014951	(A/C)
1715	CakSNP1715	*Kabuli*	Ca_Kabuli_Chr01	27016101	(G/A)
1716	CakSNP1716	*Kabuli*	Ca_Kabuli_Chr01	27016206	(T/A)
1717	CakSNP1717	*Kabuli*	Ca_Kabuli_Chr01	27017559	(G/A)
1718	CakSNP1718	*Kabuli*	Ca_Kabuli_Chr01	27017560	(G/A)
1719	CakSNP1719	*Kabuli*	Ca_Kabuli_Chr01	27017570	(A/G)
1720	CakSNP1720	*Kabuli*	Ca_Kabuli_Chr01	27017604	(T/G)
1721	CakSNP1721	*Kabuli*	Ca_Kabuli_Chr01	27017606	(C/T)
1722	CakSNP1722	*Kabuli*	Ca_Kabuli_Chr01	27017617	(G/A)
1723	CakSNP1723	*Kabuli*	Ca_Kabuli_Chr01	27017626	(A/C)
1724	CakSNP1724	*Kabuli*	Ca_Kabuli_Chr01	27017628	(C/A)
1725	CakSNP1725	*Kabuli*	Ca_Kabuli_Chr01	27017734	(T/C)
1726	CakSNP1726	*Kabuli*	Ca_Kabuli_Chr01	27017765	(A/T)
1727	CakSNP1727	*Kabuli*	Ca_Kabuli_Chr01	27017771	(G/A)
1728	CakSNP1728	*Kabuli*	Ca_Kabuli_Chr01	27020193	(T/C)
1729	CakSNP1729	*Kabuli*	Ca_Kabuli_Chr01	27020354	(C/A)
1730	CakSNP1730	*Kabuli*	Ca_Kabuli_Chr01	28088501	(T/C)
1731	CakSNP1731	*Kabuli*	Ca_Kabuli_Chr01	28106756	(G/A)
1732	CakSNP1732	*Kabuli*	Ca_Kabuli_Chr01	28133847	(A/G)
1733	CakSNP1733	*Kabuli*	Ca_Kabuli_Chr01	28133830	(A/T)
1734	CakSNP1734	*Kabuli*	Ca_Kabuli_Chr01	28133814	(C/T)
1735	CakSNP1735	*Kabuli*	Ca_Kabuli_Chr01	28143955	(G/A)
1736	CakSNP1736	*Kabuli*	Ca_Kabuli_Chr01	28152260	(A/G)
1737	CakSNP1737	*Kabuli*	Ca_Kabuli_Chr01	28656197	(C/A)
1738	CakSNP1738	*Kabuli*	Ca_Kabuli_Chr01	28832810	(C/A)
1739	CakSNP1739	*Kabuli*	Ca_Kabuli_Chr01	28907617	(G/A)
1740	CakSNP1740	*Kabuli*	Ca_Kabuli_Chr01	28909059	(T/A)
1741	CakSNP1741	*Kabuli*	Ca_Kabuli_Chr01	29020220	(T/G)
1742	CakSNP1742	*Kabuli*	Ca_Kabuli_Chr01	29116430	(T/C)
1743	CakSNP1743	*Kabuli*	Ca_Kabuli_Chr01	29460440	(C/A)
1744	CakSNP1744	*Kabuli*	Ca_Kabuli_Chr01	29634320	(G/T)
1745	CakSNP1745	*Kabuli*	Ca_Kabuli_Chr01	29637509	(A/C)
1746	CakSNP1746	*Kabuli*	Ca_Kabuli_Chr01	29637526	(C/T)
1747	CakSNP1747	*Kabuli*	Ca_Kabuli_Chr01	29637572	(T/G)

S.N.	SNP IDs	Cultivars	Chromosomes/scaffolds	Physical positions (bp)	SNPs
1748	CakSNP1748	*Kabuli*	Ca_Kabuli_Chr01	29637571	(T/G)
1749	CakSNP1749	*Kabuli*	Ca_Kabuli_Chr01	30262124	(A/G)
1750	CakSNP1750	*Kabuli*	Ca_Kabuli_Chr01	31080673	(T/G)
1751	CakSNP1751	*Kabuli*	Ca_Kabuli_Chr01	31080792	(T/C)
1752	CakSNP1752	*Kabuli*	Ca_Kabuli_Chr01	31240370	(A/G)
1753	CakSNP1753	*Kabuli*	Ca_Kabuli_Chr01	31359861	(C/A)
1754	CakSNP1754	*Kabuli*	Ca_Kabuli_Chr01	31369599	(T/C)
1755	CakSNP1755	*Kabuli*	Ca_Kabuli_Chr01	31590263	(C/G)
1756	CakSNP1756	*Kabuli*	Ca_Kabuli_Chr01	31590510	(G/A)
1757	CakSNP1757	*Kabuli*	Ca_Kabuli_Chr01	31590548	(C/T)
1758	CakSNP1758	*Kabuli*	Ca_Kabuli_Chr01	31838239	(G/A)
1759	CakSNP1759	*Kabuli*	Ca_Kabuli_Chr01	32286538	(C/T)
1760	CakSNP1760	*Kabuli*	Ca_Kabuli_Chr01	32286534	(G/C)
1761	CakSNP1761	*Kabuli*	Ca_Kabuli_Chr01	32286518	(C/A)
1762	CakSNP1762	*Kabuli*	Ca_Kabuli_Chr01	32286517	(G/A)
1763	CakSNP1763	*Kabuli*	Ca_Kabuli_Chr01	32286469	(G/A)
1764	CakSNP1764	*Kabuli*	Ca_Kabuli_Chr01	32286466	(A/G)
1765	CakSNP1765	*Kabuli*	Ca_Kabuli_Chr01	32286568	(A/C)
1766	CakSNP1766	*Kabuli*	Ca_Kabuli_Chr01	32286558	(A/G)
1767	CakSNP1767	*Kabuli*	Ca_Kabuli_Chr01	32286550	(G/A)
1768	CakSNP1768	*Kabuli*	Ca_Kabuli_Chr01	32286536	(G/A)
1769	CakSNP1769	*Kabuli*	Ca_Kabuli_Chr01	32286551	(C/T)
1770	CakSNP1770	*Kabuli*	Ca_Kabuli_Chr01	32286567	(C/T)
1771	CakSNP1771	*Kabuli*	Ca_Kabuli_Chr01	32467929	(G/A)
1772	CakSNP1772	*Kabuli*	Ca_Kabuli_Chr01	32467954	(A/C)
1773	CakSNP1773	*Kabuli*	Ca_Kabuli_Chr01	32468053	(A/C)
1774	CakSNP1774	*Kabuli*	Ca_Kabuli_Chr01	32468080	(A/T)
1775	CakSNP1775	*Kabuli*	Ca_Kabuli_Chr01	32468105	(C/G)
1776	CakSNP1776	*Kabuli*	Ca_Kabuli_Chr01	32468116	(A/G)
1777	CakSNP1777	*Kabuli*	Ca_Kabuli_Chr01	32468119	(T/C)
1778	CakSNP1778	*Kabuli*	Ca_Kabuli_Chr01	33058743	(T/C)
1779	CakSNP1779	*Kabuli*	Ca_Kabuli_Chr01	33063207	(C/T)
1780	CakSNP1780	*Kabuli*	Ca_Kabuli_Chr01	33194600	(T/C)
1781	CakSNP1781	*Kabuli*	Ca_Kabuli_Chr01	33804152	(C/T)
1782	CakSNP1782	*Kabuli*	Ca_Kabuli_Chr01	33804153	(C/T)
1783	CakSNP1783	*Kabuli*	Ca_Kabuli_Chr01	33804189	(C/T)
1784	CakSNP1784	*Kabuli*	Ca_Kabuli_Chr01	33804201	(C/T)
1785	CakSNP1785	*Kabuli*	Ca_Kabuli_Chr01	33804232	(T/G)

S.N.	SNP IDs	Cultivars	Chromosomes/scaffolds	Physical positions (bp)	SNPs
1786	CakSNP1786	*Kabuli*	Ca_Kabuli_Chr01	33804247	(A/G)
1787	CakSNP1787	*Kabuli*	Ca_Kabuli_Chr01	33804262	(C/G)
1788	CakSNP1788	*Kabuli*	Ca_Kabuli_Chr01	34161921	(G/A)
1789	CakSNP1789	*Kabuli*	Ca_Kabuli_Chr01	34261166	(C/A)
1790	CakSNP1790	*Kabuli*	Ca_Kabuli_Chr01	34261158	(C/A)
1791	CakSNP1791	*Kabuli*	Ca_Kabuli_Chr01	34311145	(T/G)
1792	CakSNP1792	*Kabuli*	Ca_Kabuli_Chr01	34437114	(C/T)
1793	CakSNP1793	*Kabuli*	Ca_Kabuli_Chr01	34727251	(T/G)
1794	CakSNP1794	*Kabuli*	Ca_Kabuli_Chr01	34772813	(T/A)
1795	CakSNP1795	*Kabuli*	Ca_Kabuli_Chr01	34866709	(C/A)
1796	CakSNP1796	*Kabuli*	Ca_Kabuli_Chr01	34905580	(A/G)
1797	CakSNP1797	*Kabuli*	Ca_Kabuli_Chr01	34911065	(T/A)
1798	CakSNP1798	*Kabuli*	Ca_Kabuli_Chr01	34948934	(A/G)
1799	CakSNP1799	*Kabuli*	Ca_Kabuli_Chr01	35041241	(T/G)
1800	CakSNP1800	*Kabuli*	Ca_Kabuli_Chr01	35041324	(G/T)
1801	CakSNP1801	*Kabuli*	Ca_Kabuli_Chr01	35313277	(A/G)
1802	CakSNP1802	*Kabuli*	Ca_Kabuli_Chr01	35315381	(T/A)
1803	CakSNP1803	*Kabuli*	Ca_Kabuli_Chr01	35315379	(A/T)
1804	CakSNP1804	*Kabuli*	Ca_Kabuli_Chr01	35315347	(C/T)
1805	CakSNP1805	*Kabuli*	Ca_Kabuli_Chr01	35315327	(T/A)
1806	CakSNP1806	*Kabuli*	Ca_Kabuli_Chr01	35315436	(G/A)
1807	CakSNP1807	*Kabuli*	Ca_Kabuli_Chr01	35708841	(C/T)
1808	CakSNP1808	*Kabuli*	Ca_Kabuli_Chr01	36126090	(C/T)
1809	CakSNP1809	*Kabuli*	Ca_Kabuli_Chr01	36794731	(A/G)
1810	CakSNP1810	*Kabuli*	Ca_Kabuli_Chr01	36794797	(G/A)
1811	CakSNP1811	*Kabuli*	Ca_Kabuli_Chr01	36794800	(G/T)
1812	CakSNP1812	*Kabuli*	Ca_Kabuli_Chr01	36794806	(G/T)
1813	CakSNP1813	*Kabuli*	Ca_Kabuli_Chr01	36794813	(C/T)
1814	CakSNP1814	*Kabuli*	Ca_Kabuli_Chr01	36794887	(G/A)
1815	CakSNP1815	*Kabuli*	Ca_Kabuli_Chr01	36794870	(C/T)
1816	CakSNP1816	*Kabuli*	Ca_Kabuli_Chr01	36794900	(C/T)
1817	CakSNP1817	*Kabuli*	Ca_Kabuli_Chr01	36794906	(A/C)
1818	CakSNP1818	*Kabuli*	Ca_Kabuli_Chr01	36794912	(G/T)
1819	CakSNP1819	*Kabuli*	Ca_Kabuli_Chr01	36794913	(A/T)
1820	CakSNP1820	*Kabuli*	Ca_Kabuli_Chr01	36794915	(G/A)
1821	CakSNP1821	*Kabuli*	Ca_Kabuli_Chr01	36892533	(C/T)
1822	CakSNP1822	*Kabuli*	Ca_Kabuli_Chr01	37026302	(C/T)
1823	CakSNP1823	*Kabuli*	Ca_Kabuli_Chr01	37608030	(G/A)

**S.N.**	**SNP IDs**	**Cultivars**	**Chromosomes/scaffolds**	**Physical positions (bp)**	**SNPs**
1824	CakSNP1824	*Kabuli*	Ca_Kabuli_Chr01	37608050	(T/G)
1825	CakSNP1825	*Kabuli*	Ca_Kabuli_Chr01	37642546	(G/A)
1826	CakSNP1826	*Kabuli*	Ca_Kabuli_Chr01	37643213	(T/C)
1827	CakSNP1827	*Kabuli*	Ca_Kabuli_Chr01	37647602	(C/G)
1828	CakSNP1828	*Kabuli*	Ca_Kabuli_Chr01	37651679	(G/A)
1829	CakSNP1829	*Kabuli*	Ca_Kabuli_Chr01	37651695	(G/A)
1830	CakSNP1830	*Kabuli*	Ca_Kabuli_Chr01	37651764	(A/C)
1831	CakSNP1831	*Kabuli*	Ca_Kabuli_Chr01	37651711	(G/T)
1832	CakSNP1832	*Kabuli*	Ca_Kabuli_Chr01	37651800	(T/A)
1833	CakSNP1833	*Kabuli*	Ca_Kabuli_Chr01	37651823	(A/G)
1834	CakSNP1834	*Kabuli*	Ca_Kabuli_Chr01	37651827	(C/T)
1835	CakSNP1835	*Kabuli*	Ca_Kabuli_Chr01	37651843	(T/C)
1836	CakSNP1836	*Kabuli*	Ca_Kabuli_Chr01	37654039	(C/G)
1837	CakSNP1837	*Kabuli*	Ca_Kabuli_Chr01	37654136	(C/G)
1838	CakSNP1838	*Kabuli*	Ca_Kabuli_Chr01	37654926	(C/T)
1839	CakSNP1839	*Kabuli*	Ca_Kabuli_Chr01	37690416	(G/A)
1840	CakSNP1840	*Kabuli*	Ca_Kabuli_Chr01	37690465	(T/C)
1841	CakSNP1841	*Kabuli*	Ca_Kabuli_Chr01	37690498	(C/A)
1842	CakSNP1842	*Kabuli*	Ca_Kabuli_Chr01	37690497	(A/T)
1843	CakSNP1843	*Kabuli*	Ca_Kabuli_Chr01	37699039	(T/G)
1844	CakSNP1844	*Kabuli*	Ca_Kabuli_Chr01	37699036	(T/C)
1845	CakSNP1845	*Kabuli*	Ca_Kabuli_Chr01	37722095	(G/A)
1846	CakSNP1846	*Kabuli*	Ca_Kabuli_Chr01	37722143	(G/C)
1847	CakSNP1847	*Kabuli*	Ca_Kabuli_Chr01	37768179	(T/A)
1848	CakSNP1848	*Kabuli*	Ca_Kabuli_Chr01	37768206	(A/T)
1849	CakSNP1849	*Kabuli*	Ca_Kabuli_Chr01	37768238	(A/G)
1850	CakSNP1850	*Kabuli*	Ca_Kabuli_Chr01	37768333	(C/T)
1851	CakSNP1851	*Kabuli*	Ca_Kabuli_Chr01	37768299	(A/G)
1852	CakSNP1852	*Kabuli*	Ca_Kabuli_Chr01	37768276	(G/C)
1853	CakSNP1853	*Kabuli*	Ca_Kabuli_Chr01	37768264	(A/G)
1854	CakSNP1854	*Kabuli*	Ca_Kabuli_Chr01	37862342	(T/G)
1855	CakSNP1855	*Kabuli*	Ca_Kabuli_Chr01	37880995	(T/C)
1856	CakSNP1856	*Kabuli*	Ca_Kabuli_Chr01	37892088	(A/C)
1857	CakSNP1857	*Kabuli*	Ca_Kabuli_Chr01	37892089	(C/T)
1858	CakSNP1858	*Kabuli*	Ca_Kabuli_Chr01	37892095	(A/G)
1859	CakSNP1859	*Kabuli*	Ca_Kabuli_Chr01	37892139	(A/T)
1860	CakSNP1860	*Kabuli*	Ca_Kabuli_Chr01	38049721	(C/T)
1861	CakSNP1861	*Kabuli*	Ca_Kabuli_Chr01	38678712	(T/C)

S.N.	SNP IDs	Cultivars	Chromosomes/scaffolds	Physical positions (bp)	SNPs
1862	CakSNP1862	*Kabuli*	Ca_Kabuli_Chr01	38877670	(T/C)
1863	CakSNP1863	*Kabuli*	Ca_Kabuli_Chr01	38877692	(G/T)
1864	CakSNP1864	*Kabuli*	Ca_Kabuli_Chr01	39436291	(T/C)
1865	CakSNP1865	*Kabuli*	Ca_Kabuli_Chr01	39601982	(T/G)
1866	CakSNP1866	*Kabuli*	Ca_Kabuli_Chr01	39602029	(G/T)
1867	CakSNP1867	*Kabuli*	Ca_Kabuli_Chr01	39616139	(A/C)
1868	CakSNP1868	*Kabuli*	Ca_Kabuli_Chr01	39663432	(C/G)
1869	CakSNP1869	*Kabuli*	Ca_Kabuli_Chr01	40224835	(T/A)
1870	CakSNP1870	*Kabuli*	Ca_Kabuli_Chr01	40224834	(C/G)
1871	CakSNP1871	*Kabuli*	Ca_Kabuli_Chr01	40224804	(A/G)
1872	CakSNP1872	*Kabuli*	Ca_Kabuli_Chr01	40343216	(G/T)
1873	CakSNP1873	*Kabuli*	Ca_Kabuli_Chr01	40346280	(A/C)
1874	CakSNP1874	*Kabuli*	Ca_Kabuli_Chr01	40358693	(C/T)
1875	CakSNP1875	*Kabuli*	Ca_Kabuli_Chr01	40358747	(T/G)
1876	CakSNP1876	*Kabuli*	Ca_Kabuli_Chr01	40367940	(G/A)
1877	CakSNP1877	*Kabuli*	Ca_Kabuli_Chr01	40368845	(C/T)
1878	CakSNP1878	*Kabuli*	Ca_Kabuli_Chr01	40502077	(T/C)
1879	CakSNP1879	*Kabuli*	Ca_Kabuli_Chr01	40570811	(G/T)
1880	CakSNP1880	*Kabuli*	Ca_Kabuli_Chr01	40585026	(T/C)
1881	CakSNP1881	*Kabuli*	Ca_Kabuli_Chr01	40630215	(C/T)
1882	CakSNP1882	*Kabuli*	Ca_Kabuli_Chr01	40630213	(G/T)
1883	CakSNP1883	*Kabuli*	Ca_Kabuli_Chr01	40630158	(T/A)
1884	CakSNP1884	*Kabuli*	Ca_Kabuli_Chr01	40660069	(T/G)
1885	CakSNP1885	*Kabuli*	Ca_Kabuli_Chr01	40660116	(G/A)
1886	CakSNP1886	*Kabuli*	Ca_Kabuli_Chr01	40745421	(G/T)
1887	CakSNP1887	*Kabuli*	Ca_Kabuli_Chr01	40745452	(T/C)
1888	CakSNP1888	*Kabuli*	Ca_Kabuli_Chr01	40745492	(A/G)
1889	CakSNP1889	*Kabuli*	Ca_Kabuli_Chr01	40745846	(T/C)
1890	CakSNP1890	*Kabuli*	Ca_Kabuli_Chr01	41029732	(C/T)
1891	CakSNP1891	*Kabuli*	Ca_Kabuli_Chr01	41065797	(G/C)
1892	CakSNP1892	*Kabuli*	Ca_Kabuli_Chr01	41125493	(T/C)
1893	CakSNP1893	*Kabuli*	Ca_Kabuli_Chr01	41169218	(G/T)
1894	CakSNP1894	*Kabuli*	Ca_Kabuli_Chr01	41169430	(T/G)
1895	CakSNP1895	*Kabuli*	Ca_Kabuli_Chr01	41169372	(T/A)
1896	CakSNP1896	*Kabuli*	Ca_Kabuli_Chr01	41178168	(G/A)
1897	CakSNP1897	*Kabuli*	Ca_Kabuli_Chr01	41178183	(C/A)
1898	CakSNP1898	*Kabuli*	Ca_Kabuli_Chr01	41179660	(G/T)
1899	CakSNP1899	*Kabuli*	Ca_Kabuli_Chr01	41179650	(A/T)

S.N.	SNP IDs	Cultivars	Chromosomes/scaffolds	Physical positions (bp)	SNPs
1900	CakSNP1900	*Kabuli*	Ca_Kabuli_Chr01	41185512	(T/G)
1901	CakSNP1901	*Kabuli*	Ca_Kabuli_Chr01	41202173	(T/G)
1902	CakSNP1902	*Kabuli*	Ca_Kabuli_Chr01	41202206	(A/G)
1903	CakSNP1903	*Kabuli*	Ca_Kabuli_Chr01	41310401	(T/G)
1904	CakSNP1904	*Kabuli*	Ca_Kabuli_Chr01	41339408	(T/A)
1905	CakSNP1905	*Kabuli*	Ca_Kabuli_Chr01	41339445	(C/A)
1906	CakSNP1906	*Kabuli*	Ca_Kabuli_Chr01	41380061	(G/T)
1907	CakSNP1907	*Kabuli*	Ca_Kabuli_Chr01	41437222	(A/G)
1908	CakSNP1908	*Kabuli*	Ca_Kabuli_Chr01	41437366	(T/C)
1909	CakSNP1909	*Kabuli*	Ca_Kabuli_Chr01	41437453	(T/G)
1910	CakSNP1910	*Kabuli*	Ca_Kabuli_Chr01	41437483	(G/A)
1911	CakSNP1911	*Kabuli*	Ca_Kabuli_Chr01	41439539	(A/G)
1912	CakSNP1912	*Kabuli*	Ca_Kabuli_Chr01	41439593	(A/G)
1913	CakSNP1913	*Kabuli*	Ca_Kabuli_Chr01	41451910	(C/T)
1914	CakSNP1914	*Kabuli*	Ca_Kabuli_Chr01	41451977	(C/T)
1915	CakSNP1915	*Kabuli*	Ca_Kabuli_Chr01	41475442	(T/C)
1916	CakSNP1916	*Kabuli*	Ca_Kabuli_Chr01	41484033	(T/C)
1917	CakSNP1917	*Kabuli*	Ca_Kabuli_Chr01	41484085	(C/T)
1918	CakSNP1918	*Kabuli*	Ca_Kabuli_Chr01	41564809	(T/C)
1919	CakSNP1919	*Kabuli*	Ca_Kabuli_Chr01	41795343	(G/A)
1920	CakSNP1920	*Kabuli*	Ca_Kabuli_Chr01	41795367	(C/T)
1921	CakSNP1921	*Kabuli*	Ca_Kabuli_Chr01	41822419	(C/T)
1922	CakSNP1922	*Kabuli*	Ca_Kabuli_Chr01	41827388	(T/C)
1923	CakSNP1923	*Kabuli*	Ca_Kabuli_Chr01	41867035	(C/T)
1924	CakSNP1924	*Kabuli*	Ca_Kabuli_Chr01	41897216	(C/A)
1925	CakSNP1925	*Kabuli*	Ca_Kabuli_Chr01	41946005	(A/G)
1926	CakSNP1926	*Kabuli*	Ca_Kabuli_Chr01	41981244	(G/A)
1927	CakSNP1927	*Kabuli*	Ca_Kabuli_Chr01	41981259	(G/A)
1928	CakSNP1928	*Kabuli*	Ca_Kabuli_Chr01	42189380	(A/G)
1929	CakSNP1929	*Kabuli*	Ca_Kabuli_Chr01	42452801	(G/A)
1930	CakSNP1930	*Kabuli*	Ca_Kabuli_Chr01	42452939	(G/T)
1931	CakSNP1931	*Kabuli*	Ca_Kabuli_Chr01	42515406	(C/T)
1932	CakSNP1932	*Kabuli*	Ca_Kabuli_Chr01	42551574	(T/G)
1933	CakSNP1933	*Kabuli*	Ca_Kabuli_Chr01	42551585	(C/A)
1934	CakSNP1934	*Kabuli*	Ca_Kabuli_Chr01	42552658	(A/C)
1935	CakSNP1935	*Kabuli*	Ca_Kabuli_Chr01	42605239	(T/G)
1936	CakSNP1936	*Kabuli*	Ca_Kabuli_Chr01	42610115	(A/C)
1937	CakSNP1937	*Kabuli*	Ca_Kabuli_Chr01	42613060	(T/G)

S.N.	SNP IDs	Cultivars	Chromosomes/scaffolds	Physical positions (bp)	SNPs
1938	CakSNP1938	*Kabuli*	Ca_Kabuli_Chr01	42894518	(T/C)
1939	CakSNP1939	*Kabuli*	Ca_Kabuli_Chr01	42894525	(T/C)
1940	CakSNP1940	*Kabuli*	Ca_Kabuli_Chr01	42894536	(G/T)
1941	CakSNP1941	*Kabuli*	Ca_Kabuli_Chr01	43053424	(G/C)
1942	CakSNP1942	*Kabuli*	Ca_Kabuli_Chr01	43273910	(G/T)
1943	CakSNP1943	*Kabuli*	Ca_Kabuli_Chr01	43273922	(T/C)
1944	CakSNP1944	*Kabuli*	Ca_Kabuli_Chr01	43273925	(A/G)
1945	CakSNP1945	*Kabuli*	Ca_Kabuli_Chr01	43273931	(G/A)
1946	CakSNP1946	*Kabuli*	Ca_Kabuli_Chr01	43273941	(C/G)
1947	CakSNP1947	*Kabuli*	Ca_Kabuli_Chr01	43273943	(A/G)
1948	CakSNP1948	*Kabuli*	Ca_Kabuli_Chr01	43273961	(C/T)
1949	CakSNP1949	*Kabuli*	Ca_Kabuli_Chr01	43273962	(A/G)
1950	CakSNP1950	*Kabuli*	Ca_Kabuli_Chr01	43273968	(T/G)
1951	CakSNP1951	*Kabuli*	Ca_Kabuli_Chr01	43273972	(C/T)
1952	CakSNP1952	*Kabuli*	Ca_Kabuli_Chr01	43273981	(G/T)
1953	CakSNP1953	*Kabuli*	Ca_Kabuli_Chr01	43273985	(G/A)
1954	CakSNP1954	*Kabuli*	Ca_Kabuli_Chr01	43304584	(A/C)
1955	CakSNP1955	*Kabuli*	Ca_Kabuli_Chr01	43304573	(C/T)
1956	CakSNP1956	*Kabuli*	Ca_Kabuli_Chr01	43305858	(T/C)
1957	CakSNP1957	*Kabuli*	Ca_Kabuli_Chr01	43305870	(G/A)
1958	CakSNP1958	*Kabuli*	Ca_Kabuli_Chr01	43305886	(T/C)
1959	CakSNP1959	*Kabuli*	Ca_Kabuli_Chr01	43305904	(C/A)
1960	CakSNP1960	*Kabuli*	Ca_Kabuli_Chr01	43306048	(G/A)
1961	CakSNP1961	*Kabuli*	Ca_Kabuli_Chr01	43306045	(C/A)
1962	CakSNP1962	*Kabuli*	Ca_Kabuli_Chr01	43306036	(G/A)
1963	CakSNP1963	*Kabuli*	Ca_Kabuli_Chr01	43306012	(G/A)
1964	CakSNP1964	*Kabuli*	Ca_Kabuli_Chr01	43306221	(A/G)
1965	CakSNP1965	*Kabuli*	Ca_Kabuli_Chr01	43509159	(A/G)
1966	CakSNP1966	*Kabuli*	Ca_Kabuli_Chr01	43509409	(T/C)
1967	CakSNP1967	*Kabuli*	Ca_Kabuli_Chr01	43576059	(A/G)
1968	CakSNP1968	*Kabuli*	Ca_Kabuli_Chr01	43585657	(T/G)
1969	CakSNP1969	*Kabuli*	Ca_Kabuli_Chr01	43590106	(C/T)
1970	CakSNP1970	*Kabuli*	Ca_Kabuli_Chr01	43590258	(T/C)
1971	CakSNP1971	*Kabuli*	Ca_Kabuli_Chr01	43724824	(C/T)
1972	CakSNP1972	*Kabuli*	Ca_Kabuli_Chr01	43724839	(C/T)
1973	CakSNP1973	*Kabuli*	Ca_Kabuli_Chr01	43724963	(C/T)
1974	CakSNP1974	*Kabuli*	Ca_Kabuli_Chr01	43725059	(C/T)
1975	CakSNP1975	*Kabuli*	Ca_Kabuli_Chr01	43725104	(T/C)

S.N.	SNP IDs	Cultivars	Chromosomes/scaffolds	Physical positions (bp)	SNPs
1976	CakSNP1976	*Kabuli*	Ca_Kabuli_Chr01	43727804	(C/T)
1977	CakSNP1977	*Kabuli*	Ca_Kabuli_Chr01	43792565	(G/A)
1978	CakSNP1978	*Kabuli*	Ca_Kabuli_Chr01	43800367	(C/T)
1979	CakSNP1979	*Kabuli*	Ca_Kabuli_Chr01	43930143	(A/T)
1980	CakSNP1980	*Kabuli*	Ca_Kabuli_Chr01	43930138	(T/C)
1981	CakSNP1981	*Kabuli*	Ca_Kabuli_Chr01	43933881	(G/C)
1982	CakSNP1982	*Kabuli*	Ca_Kabuli_Chr01	43933904	(A/G)
1983	CakSNP1983	*Kabuli*	Ca_Kabuli_Chr01	43934024	(G/T)
1984	CakSNP1984	*Kabuli*	Ca_Kabuli_Chr01	43934120	(A/G)
1985	CakSNP1985	*Kabuli*	Ca_Kabuli_Chr01	43934117	(G/T)
1986	CakSNP1986	*Kabuli*	Ca_Kabuli_Chr01	43934081	(C/T)
1987	CakSNP1987	*Kabuli*	Ca_Kabuli_Chr01	43943035	(G/T)
1988	CakSNP1988	*Kabuli*	Ca_Kabuli_Chr01	43954743	(C/G)
1989	CakSNP1989	*Kabuli*	Ca_Kabuli_Chr01	44043281	(G/A)
1990	CakSNP1990	*Kabuli*	Ca_Kabuli_Chr01	44085069	(C/T)
1991	CakSNP1991	*Kabuli*	Ca_Kabuli_Chr01	44348947	(C/T)
1992	CakSNP1992	*Kabuli*	Ca_Kabuli_Chr01	44349004	(T/C)
1993	CakSNP1993	*Kabuli*	Ca_Kabuli_Chr01	44350953	(G/T)
1994	CakSNP1994	*Kabuli*	Ca_Kabuli_Chr01	44635939	(T/C)
1995	CakSNP1995	*Kabuli*	Ca_Kabuli_Chr01	44635926	(A/G)
1996	CakSNP1996	*Kabuli*	Ca_Kabuli_Chr01	44635893	(A/G)
1997	CakSNP1997	*Kabuli*	Ca_Kabuli_Chr01	44976738	(T/A)
1998	CakSNP1998	*Kabuli*	Ca_Kabuli_Chr01	44976735	(C/T)
1999	CakSNP1999	*Kabuli*	Ca_Kabuli_Chr01	44988344	(A/G)
2000	CakSNP2000	*Kabuli*	Ca_Kabuli_Chr01	44988387	(C/A)
2001	CakSNP2001	*Kabuli*	Ca_Kabuli_Chr01	45003190	(G/A)
2002	CakSNP2002	*Kabuli*	Ca_Kabuli_Chr01	45003514	(G/A)
2003	CakSNP2003	*Kabuli*	Ca_Kabuli_Chr01	45003509	(C/A)
2004	CakSNP2004	*Kabuli*	Ca_Kabuli_Chr01	45003505	(A/C)
2005	CakSNP2005	*Kabuli*	Ca_Kabuli_Chr01	45003938	(A/G)
2006	CakSNP2006	*Kabuli*	Ca_Kabuli_Chr01	45003939	(T/A)
2007	CakSNP2007	*Kabuli*	Ca_Kabuli_Chr01	45003940	(T/G)
2008	CakSNP2008	*Kabuli*	Ca_Kabuli_Chr01	45003944	(C/T)
2009	CakSNP2009	*Kabuli*	Ca_Kabuli_Chr01	45003945	(T/A)
2010	CakSNP2010	*Kabuli*	Ca_Kabuli_Chr01	45003946	(T/G)
2011	CakSNP2011	*Kabuli*	Ca_Kabuli_Chr01	45003947	(C/A)
2012	CakSNP2012	*Kabuli*	Ca_Kabuli_Chr01	45003973	(T/C)
2013	CakSNP2013	*Kabuli*	Ca_Kabuli_Chr01	45003985	(G/T)

S.N.	SNP IDs	Cultivars	Chromosomes/scaffolds	Physical positions (bp)	SNPs
2014	CakSNP2014	*Kabuli*	Ca_Kabuli_Chr01	45003998	(G/A)
2015	CakSNP2015	*Kabuli*	Ca_Kabuli_Chr01	45004001	(C/T)
2016	CakSNP2016	*Kabuli*	Ca_Kabuli_Chr01	45007032	(A/C)
2017	CakSNP2017	*Kabuli*	Ca_Kabuli_Chr01	45007227	(T/C)
2018	CakSNP2018	*Kabuli*	Ca_Kabuli_Chr01	45007259	(A/G)
2019	CakSNP2019	*Kabuli*	Ca_Kabuli_Chr01	45007331	(C/T)
2020	CakSNP2020	*Kabuli*	Ca_Kabuli_Chr01	45008076	(G/A)
2021	CakSNP2021	*Kabuli*	Ca_Kabuli_Chr01	45008075	(T/C)
2022	CakSNP2022	*Kabuli*	Ca_Kabuli_Chr01	45008073	(T/G)
2023	CakSNP2023	*Kabuli*	Ca_Kabuli_Chr01	45008071	(G/C)
2024	CakSNP2024	*Kabuli*	Ca_Kabuli_Chr01	45008050	(C/G)
2025	CakSNP2025	*Kabuli*	Ca_Kabuli_Chr01	45008044	(T/C)
2026	CakSNP2026	*Kabuli*	Ca_Kabuli_Chr01	45008041	(C/G)
2027	CakSNP2027	*Kabuli*	Ca_Kabuli_Chr01	45008040	(G/C)
2028	CakSNP2028	*Kabuli*	Ca_Kabuli_Chr01	45008039	(C/T)
2029	CakSNP2029	*Kabuli*	Ca_Kabuli_Chr01	45008016	(T/A)
2030	CakSNP2030	*Kabuli*	Ca_Kabuli_Chr01	45008015	(G/A)
2031	CakSNP2031	*Kabuli*	Ca_Kabuli_Chr01	45008009	(C/T)
2032	CakSNP2032	*Kabuli*	Ca_Kabuli_Chr01	45008006	(C/A)
2033	CakSNP2033	*Kabuli*	Ca_Kabuli_Chr01	45008004	(C/T)
2034	CakSNP2034	*Kabuli*	Ca_Kabuli_Chr01	45008074	(C/T)
2035	CakSNP2035	*Kabuli*	Ca_Kabuli_Chr01	45250874	(G/A)
2036	CakSNP2036	*Kabuli*	Ca_Kabuli_Chr01	45250872	(G/T)
2037	CakSNP2037	*Kabuli*	Ca_Kabuli_Chr01	45250869	(G/A)
2038	CakSNP2038	*Kabuli*	Ca_Kabuli_Chr01	45250863	(C/T)
2039	CakSNP2039	*Kabuli*	Ca_Kabuli_Chr01	45250862	(A/T)
2040	CakSNP2040	*Kabuli*	Ca_Kabuli_Chr01	45250834	(A/G)
2041	CakSNP2041	*Kabuli*	Ca_Kabuli_Chr01	45250828	(G/C)
2042	CakSNP2042	*Kabuli*	Ca_Kabuli_Chr01	45250807	(C/G)
2043	CakSNP2043	*Kabuli*	Ca_Kabuli_Chr01	45250805	(A/C)
2044	CakSNP2044	*Kabuli*	Ca_Kabuli_Chr01	45250803	(A/G)
2045	CakSNP2045	*Kabuli*	Ca_Kabuli_Chr01	45250802	(C/T)
2046	CakSNP2046	*Kabuli*	Ca_Kabuli_Chr01	46437023	(T/G)
2047	CakSNP2047	*Kabuli*	Ca_Kabuli_Chr01	46523221	(C/T)
2048	CakSNP2048	*Kabuli*	Ca_Kabuli_Chr01	46597131	(T/G)
2049	CakSNP2049	*Kabuli*	Ca_Kabuli_Chr01	46745628	(C/A)
2050	CakSNP2050	*Kabuli*	Ca_Kabuli_Chr01	46778814	(A/C)
2051	CakSNP2051	*Kabuli*	Ca_Kabuli_Chr01	46791939	(T/G)

S.N.	SNP IDs	Cultivars	Chromosomes/scaffolds	Physical positions (bp)	SNPs
2052	CakSNP2052	*Kabuli*	Ca_Kabuli_Chr01	46793244	(G/A)
2053	CakSNP2053	*Kabuli*	Ca_Kabuli_Chr01	46793357	(T/C)
2054	CakSNP2054	*Kabuli*	Ca_Kabuli_Chr01	46793331	(C/G)
2055	CakSNP2055	*Kabuli*	Ca_Kabuli_Chr01	46816041	(A/T)
2056	CakSNP2056	*Kabuli*	Ca_Kabuli_Chr01	46825211	(G/A)
2057	CakSNP2057	*Kabuli*	Ca_Kabuli_Chr01	46829662	(C/T)
2058	CakSNP2058	*Kabuli*	Ca_Kabuli_Chr01	46832306	(T/G)
2059	CakSNP2059	*Kabuli*	Ca_Kabuli_Chr01	46832395	(A/G)
2060	CakSNP2060	*Kabuli*	Ca_Kabuli_Chr01	46832383	(T/C)
2061	CakSNP2061	*Kabuli*	Ca_Kabuli_Chr01	46920524	(C/T)
2062	CakSNP2062	*Kabuli*	Ca_Kabuli_Chr01	46920554	(T/C)
2063	CakSNP2063	*Kabuli*	Ca_Kabuli_Chr01	46936196	(C/A)
2064	CakSNP2064	*Kabuli*	Ca_Kabuli_Chr01	46936201	(C/T)
2065	CakSNP2065	*Kabuli*	Ca_Kabuli_Chr01	46936207	(C/A)
2066	CakSNP2066	*Kabuli*	Ca_Kabuli_Chr01	46936209	(C/T)
2067	CakSNP2067	*Kabuli*	Ca_Kabuli_Chr01	46936329	(C/A)
2068	CakSNP2068	*Kabuli*	Ca_Kabuli_Chr01	46936297	(A/G)
2069	CakSNP2069	*Kabuli*	Ca_Kabuli_Chr01	46936428	(T/C)
2070	CakSNP2070	*Kabuli*	Ca_Kabuli_Chr01	46936460	(A/C)
2071	CakSNP2071	*Kabuli*	Ca_Kabuli_Chr01	46936456	(T/C)
2072	CakSNP2072	*Kabuli*	Ca_Kabuli_Chr01	46936448	(A/C)
2073	CakSNP2073	*Kabuli*	Ca_Kabuli_Chr01	46936447	(G/A)
2074	CakSNP2074	*Kabuli*	Ca_Kabuli_Chr01	46941875	(T/C)
2075	CakSNP2075	*Kabuli*	Ca_Kabuli_Chr01	46968740	(T/C)
2076	CakSNP2076	*Kabuli*	Ca_Kabuli_Chr01	46969221	(T/A)
2077	CakSNP2077	*Kabuli*	Ca_Kabuli_Chr01	46976508	(A/G)
2078	CakSNP2078	*Kabuli*	Ca_Kabuli_Chr01	47042798	(T/A)
2079	CakSNP2079	*Kabuli*	Ca_Kabuli_Chr01	47203123	(A/C)
2080	CakSNP2080	*Kabuli*	Ca_Kabuli_Chr01	47263589	(C/T)
2081	CakSNP2081	*Kabuli*	Ca_Kabuli_Chr01	47278505	(T/C)
2082	CakSNP2082	*Kabuli*	Ca_Kabuli_Chr01	47331082	(T/C)
2083	CakSNP2083	*Kabuli*	Ca_Kabuli_Chr01	47331080	(T/C)
2084	CakSNP2084	*Kabuli*	Ca_Kabuli_Chr01	47423715	(A/T)
2085	CakSNP2085	*Kabuli*	Ca_Kabuli_Chr01	47449818	(A/G)
2086	CakSNP2086	*Kabuli*	Ca_Kabuli_Chr01	47449950	(T/C)
2087	CakSNP2087	*Kabuli*	Ca_Kabuli_Chr01	47449891	(T/C)
2088	CakSNP2088	*Kabuli*	Ca_Kabuli_Chr01	47449882	(A/C)
2089	CakSNP2089	*Kabuli*	Ca_Kabuli_Chr01	47801491	(A/G)

S.N.	SNP IDs	Cultivars	Chromosomes/scaffolds	Physical positions (bp)	SNPs
2090	CakSNP2090	*Kabuli*	Ca_Kabuli_Chr01	47890026	(C/T)
2091	CakSNP2091	*Kabuli*	Ca_Kabuli_Chr01	47897914	(C/T)
2092	CakSNP2092	*Kabuli*	Ca_Kabuli_Chr01	47936748	(G/A)
2093	CakSNP2093	*Kabuli*	Ca_Kabuli_Chr01	47936841	(A/G)
2094	CakSNP2094	*Kabuli*	Ca_Kabuli_Chr01	47938884	(T/G)
2095	CakSNP2095	*Kabuli*	Ca_Kabuli_Chr01	47947009	(T/C)
2096	CakSNP2096	*Kabuli*	Ca_Kabuli_Chr01	47947281	(A/C)
2097	CakSNP2097	*Kabuli*	Ca_Kabuli_Chr01	48026664	(G/T)
2098	CakSNP2098	*Kabuli*	Ca_Kabuli_Chr01	48026701	(C/T)
2099	CakSNP2099	*Kabuli*	Ca_Kabuli_Chr01	48026716	(T/G)
2100	CakSNP2100	*Kabuli*	Ca_Kabuli_Chr01	48026727	(A/C)
2101	CakSNP2101	*Kabuli*	Ca_Kabuli_Chr01	48027714	(C/A)
2102	CakSNP2102	*Kabuli*	Ca_Kabuli_Chr01	48027869	(T/C)
2103	CakSNP2103	*Kabuli*	Ca_Kabuli_Chr01	48028094	(G/A)
2104	CakSNP2104	*Kabuli*	Ca_Kabuli_Chr01	48049293	(C/T)
2105	CakSNP2105	*Kabuli*	Ca_Kabuli_Chr01	48132217	(G/A)
2106	CakSNP2106	*Kabuli*	Ca_Kabuli_Chr01	48132521	(A/C)
2107	CakSNP2107	*Kabuli*	Ca_Kabuli_Chr01	48154321	(A/C)
2108	CakSNP2108	*Kabuli*	Ca_Kabuli_Chr01	48191460	(T/A)
2109	CakSNP2109	*Kabuli*	Ca_Kabuli_Chr01	48195174	(T/A)
2110	CakSNP2110	*Kabuli*	Ca_Kabuli_Chr01	48197895	(A/T)
2111	CakSNP2111	*Kabuli*	Ca_Kabuli_Chr01	48197911	(T/G)
2112	CakSNP2112	*Kabuli*	Ca_Kabuli_Chr01	48197979	(A/G)
2113	CakSNP2113	*Kabuli*	Ca_Kabuli_Chr01	48197969	(G/A)
2114	CakSNP2114	*Kabuli*	Ca_Kabuli_Chr01	48198932	(A/T)
2115	CakSNP2115	*Kabuli*	Ca_Kabuli_Chr01	48265590	(T/G)
2116	CakSNP2116	*Kabuli*	Ca_Kabuli_Chr01	48273097	(G/C)
2117	CakSNP2117	*Kabuli*	Ca_Kabuli_Chr01	48273307	(C/T)
2118	CakSNP2118	*Kabuli*	Ca_Kabuli_Chr02	113330	(C/T)
2119	CakSNP2119	*Kabuli*	Ca_Kabuli_Chr02	149250	(C/A)
2120	CakSNP2120	*Kabuli*	Ca_Kabuli_Chr02	149251	(A/T)
2121	CakSNP2121	*Kabuli*	Ca_Kabuli_Chr02	149259	(A/C)
2122	CakSNP2122	*Kabuli*	Ca_Kabuli_Chr02	149269	(C/T)
2123	CakSNP2123	*Kabuli*	Ca_Kabuli_Chr02	149278	(G/T)
2124	CakSNP2124	*Kabuli*	Ca_Kabuli_Chr02	149315	(C/A)
2125	CakSNP2125	*Kabuli*	Ca_Kabuli_Chr02	149322	(C/A)
2126	CakSNP2126	*Kabuli*	Ca_Kabuli_Chr02	149402	(C/T)
2127	CakSNP2127	*Kabuli*	Ca_Kabuli_Chr02	149395	(T/C)

S.N.	SNP IDs	Cultivars	Chromosomes/scaffolds	Physical positions (bp)	SNPs
2128	CakSNP2128	*Kabuli*	Ca_Kabuli_Chr02	189287	(A/G)
2129	CakSNP2129	*Kabuli*	Ca_Kabuli_Chr02	189784	(C/A)
2130	CakSNP2130	*Kabuli*	Ca_Kabuli_Chr02	221903	(A/T)
2131	CakSNP2131	*Kabuli*	Ca_Kabuli_Chr02	221955	(A/G)
2132	CakSNP2132	*Kabuli*	Ca_Kabuli_Chr02	233589	(C/T)
2133	CakSNP2133	*Kabuli*	Ca_Kabuli_Chr02	270139	(G/A)
2134	CakSNP2134	*Kabuli*	Ca_Kabuli_Chr02	354795	(T/C)
2135	CakSNP2135	*Kabuli*	Ca_Kabuli_Chr02	355905	(T/C)
2136	CakSNP2136	*Kabuli*	Ca_Kabuli_Chr02	507857	(G/T)
2137	CakSNP2137	*Kabuli*	Ca_Kabuli_Chr02	522943	(A/G)
2138	CakSNP2138	*Kabuli*	Ca_Kabuli_Chr02	543360	(G/T)
2139	CakSNP2139	*Kabuli*	Ca_Kabuli_Chr02	616251	(C/T)
2140	CakSNP2140	*Kabuli*	Ca_Kabuli_Chr02	616410	(C/G)
2141	CakSNP2141	*Kabuli*	Ca_Kabuli_Chr02	669304	(A/T)
2142	CakSNP2142	*Kabuli*	Ca_Kabuli_Chr02	728957	(T/C)
2143	CakSNP2143	*Kabuli*	Ca_Kabuli_Chr02	737183	(T/G)
2144	CakSNP2144	*Kabuli*	Ca_Kabuli_Chr02	770164	(A/C)
2145	CakSNP2145	*Kabuli*	Ca_Kabuli_Chr02	770183	(C/T)
2146	CakSNP2146	*Kabuli*	Ca_Kabuli_Chr02	770234	(C/T)
2147	CakSNP2147	*Kabuli*	Ca_Kabuli_Chr02	770237	(C/T)
2148	CakSNP2148	*Kabuli*	Ca_Kabuli_Chr02	770225	(C/T)
2149	CakSNP2149	*Kabuli*	Ca_Kabuli_Chr02	770260	(C/T)
2150	CakSNP2150	*Kabuli*	Ca_Kabuli_Chr02	770266	(C/A)
2151	CakSNP2151	*Kabuli*	Ca_Kabuli_Chr02	1231486	(A/G)
2152	CakSNP2152	*Kabuli*	Ca_Kabuli_Chr02	1231500	(A/G)
2153	CakSNP2153	*Kabuli*	Ca_Kabuli_Chr02	1231519	(T/C)
2154	CakSNP2154	*Kabuli*	Ca_Kabuli_Chr02	1231618	(G/A)
2155	CakSNP2155	*Kabuli*	Ca_Kabuli_Chr02	1234932	(C/T)
2156	CakSNP2156	*Kabuli*	Ca_Kabuli_Chr02	1235018	(G/A)
2157	CakSNP2157	*Kabuli*	Ca_Kabuli_Chr02	1234983	(T/C)
2158	CakSNP2158	*Kabuli*	Ca_Kabuli_Chr02	1314367	(G/A)
2159	CakSNP2159	*Kabuli*	Ca_Kabuli_Chr02	1368911	(A/G)
2160	CakSNP2160	*Kabuli*	Ca_Kabuli_Chr02	1389661	(A/G)
2161	CakSNP2161	*Kabuli*	Ca_Kabuli_Chr02	1397779	(C/T)
2162	CakSNP2162	*Kabuli*	Ca_Kabuli_Chr02	1429186	(T/C)
2163	CakSNP2163	*Kabuli*	Ca_Kabuli_Chr02	1429471	(G/C)
2164	CakSNP2164	*Kabuli*	Ca_Kabuli_Chr02	1431368	(A/C)
2165	CakSNP2165	*Kabuli*	Ca_Kabuli_Chr02	1493971	(A/G)

S.N.	SNP IDs	Cultivars	Chromosomes/scaffolds	Physical positions (bp)	SNPs
2166	CakSNP2166	*Kabuli*	Ca_Kabuli_Chr02	1610477	(G/T)
2167	CakSNP2167	*Kabuli*	Ca_Kabuli_Chr02	1668116	(A/G)
2168	CakSNP2168	*Kabuli*	Ca_Kabuli_Chr02	1713343	(T/C)
2169	CakSNP2169	*Kabuli*	Ca_Kabuli_Chr02	1797399	(A/G)
2170	CakSNP2170	*Kabuli*	Ca_Kabuli_Chr02	1801341	(C/T)
2171	CakSNP2171	*Kabuli*	Ca_Kabuli_Chr02	1828318	(T/C)
2172	CakSNP2172	*Kabuli*	Ca_Kabuli_Chr02	1828406	(G/A)
2173	CakSNP2173	*Kabuli*	Ca_Kabuli_Chr02	1829772	(C/T)
2174	CakSNP2174	*Kabuli*	Ca_Kabuli_Chr02	1835055	(G/A)
2175	CakSNP2175	*Kabuli*	Ca_Kabuli_Chr02	1841290	(A/T)
2176	CakSNP2176	*Kabuli*	Ca_Kabuli_Chr02	1841267	(C/T)
2177	CakSNP2177	*Kabuli*	Ca_Kabuli_Chr02	1867041	(C/A)
2178	CakSNP2178	*Kabuli*	Ca_Kabuli_Chr02	1882691	(C/T)
2179	CakSNP2179	*Kabuli*	Ca_Kabuli_Chr02	1884583	(C/T)
2180	CakSNP2180	*Kabuli*	Ca_Kabuli_Chr02	1884704	(A/G)
2181	CakSNP2181	*Kabuli*	Ca_Kabuli_Chr02	1888657	(C/G)
2182	CakSNP2182	*Kabuli*	Ca_Kabuli_Chr02	1953614	(T/C)
2183	CakSNP2183	*Kabuli*	Ca_Kabuli_Chr02	1997914	(C/T)
2184	CakSNP2184	*Kabuli*	Ca_Kabuli_Chr02	1998003	(C/T)
2185	CakSNP2185	*Kabuli*	Ca_Kabuli_Chr02	2086763	(T/G)
2186	CakSNP2186	*Kabuli*	Ca_Kabuli_Chr02	2220871	(C/T)
2187	CakSNP2187	*Kabuli*	Ca_Kabuli_Chr02	2227118	(A/G)
2188	CakSNP2188	*Kabuli*	Ca_Kabuli_Chr02	2227074	(T/C)
2189	CakSNP2189	*Kabuli*	Ca_Kabuli_Chr02	2227465	(G/A)
2190	CakSNP2190	*Kabuli*	Ca_Kabuli_Chr02	2230089	(G/A)
2191	CakSNP2191	*Kabuli*	Ca_Kabuli_Chr02	2230056	(T/C)
2192	CakSNP2192	*Kabuli*	Ca_Kabuli_Chr02	2230984	(T/G)
2193	CakSNP2193	*Kabuli*	Ca_Kabuli_Chr02	2230980	(C/A)
2194	CakSNP2194	*Kabuli*	Ca_Kabuli_Chr02	2230976	(T/G)
2195	CakSNP2195	*Kabuli*	Ca_Kabuli_Chr02	2246678	(T/C)
2196	CakSNP2196	*Kabuli*	Ca_Kabuli_Chr02	2311915	(C/A)
2197	CakSNP2197	*Kabuli*	Ca_Kabuli_Chr02	2311917	(A/C)
2198	CakSNP2198	*Kabuli*	Ca_Kabuli_Chr02	2312104	(C/T)
2199	CakSNP2199	*Kabuli*	Ca_Kabuli_Chr02	2312037	(T/G)
2200	CakSNP2200	*Kabuli*	Ca_Kabuli_Chr02	2344777	(G/A)
2201	CakSNP2201	*Kabuli*	Ca_Kabuli_Chr02	2344778	(A/G)
2202	CakSNP2202	*Kabuli*	Ca_Kabuli_Chr02	2344786	(G/A)
2203	CakSNP2203	*Kabuli*	Ca_Kabuli_Chr02	2344835	(G/T)

S.N.	SNP IDs	Cultivars	Chromosomes/scaffolds	Physical positions (bp)	SNPs
2204	CakSNP2204	*Kabuli*	Ca_Kabuli_Chr02	2414265	(A/G)
2205	CakSNP2205	*Kabuli*	Ca_Kabuli_Chr02	2416422	(C/T)
2206	CakSNP2206	*Kabuli*	Ca_Kabuli_Chr02	2441571	(G/A)
2207	CakSNP2207	*Kabuli*	Ca_Kabuli_Chr02	2602831	(A/C)
2208	CakSNP2208	*Kabuli*	Ca_Kabuli_Chr02	2650375	(T/C)
2209	CakSNP2209	*Kabuli*	Ca_Kabuli_Chr02	2650373	(A/T)
2210	CakSNP2210	*Kabuli*	Ca_Kabuli_Chr02	2652189	(A/T)
2211	CakSNP2211	*Kabuli*	Ca_Kabuli_Chr02	2682235	(T/C)
2212	CakSNP2212	*Kabuli*	Ca_Kabuli_Chr02	2682293	(T/C)
2213	CakSNP2213	*Kabuli*	Ca_Kabuli_Chr02	2682321	(G/A)
2214	CakSNP2214	*Kabuli*	Ca_Kabuli_Chr02	2682375	(A/G)
2215	CakSNP2215	*Kabuli*	Ca_Kabuli_Chr02	2682408	(G/A)
2216	CakSNP2216	*Kabuli*	Ca_Kabuli_Chr02	2682471	(A/G)
2217	CakSNP2217	*Kabuli*	Ca_Kabuli_Chr02	2685299	(T/C)
2218	CakSNP2218	*Kabuli*	Ca_Kabuli_Chr02	2697688	(G/C)
2219	CakSNP2219	*Kabuli*	Ca_Kabuli_Chr02	2708805	(G/C)
2220	CakSNP2220	*Kabuli*	Ca_Kabuli_Chr02	2780283	(C/T)
2221	CakSNP2221	*Kabuli*	Ca_Kabuli_Chr02	2783727	(G/A)
2222	CakSNP2222	*Kabuli*	Ca_Kabuli_Chr02	2846667	(T/C)
2223	CakSNP2223	*Kabuli*	Ca_Kabuli_Chr02	2846774	(C/A)
2224	CakSNP2224	*Kabuli*	Ca_Kabuli_Chr02	2965867	(G/A)
2225	CakSNP2225	*Kabuli*	Ca_Kabuli_Chr02	3099959	(C/A)
2226	CakSNP2226	*Kabuli*	Ca_Kabuli_Chr02	3099997	(A/T)
2227	CakSNP2227	*Kabuli*	Ca_Kabuli_Chr02	3100116	(A/G)
2228	CakSNP2228	*Kabuli*	Ca_Kabuli_Chr02	3113560	(G/A)
2229	CakSNP2229	*Kabuli*	Ca_Kabuli_Chr02	3141128	(A/C)
2230	CakSNP2230	*Kabuli*	Ca_Kabuli_Chr02	3164404	(A/G)
2231	CakSNP2231	*Kabuli*	Ca_Kabuli_Chr02	3164355	(G/A)
2232	CakSNP2232	*Kabuli*	Ca_Kabuli_Chr02	3366023	(C/T)
2233	CakSNP2233	*Kabuli*	Ca_Kabuli_Chr02	3410375	(C/T)
2234	CakSNP2234	*Kabuli*	Ca_Kabuli_Chr02	3450904	(C/A)
2235	CakSNP2235	*Kabuli*	Ca_Kabuli_Chr02	3503763	(T/G)
2236	CakSNP2236	*Kabuli*	Ca_Kabuli_Chr02	3503759	(T/A)
2237	CakSNP2237	*Kabuli*	Ca_Kabuli_Chr02	3503753	(C/G)
2238	CakSNP2238	*Kabuli*	Ca_Kabuli_Chr02	3503750	(C/G)
2239	CakSNP2239	*Kabuli*	Ca_Kabuli_Chr02	3789808	(G/T)
2240	CakSNP2240	*Kabuli*	Ca_Kabuli_Chr02	3843814	(C/T)
2241	CakSNP2241	*Kabuli*	Ca_Kabuli_Chr02	3843826	(T/C)

S.N.	SNP IDs	Cultivars	Chromosomes/scaffolds	Physical positions (bp)	SNPs
2242	CakSNP2242	*Kabuli*	Ca_Kabuli_Chr02	3843836	(G/A)
2243	CakSNP2243	*Kabuli*	Ca_Kabuli_Chr02	3852203	(C/A)
2244	CakSNP2244	*Kabuli*	Ca_Kabuli_Chr02	3894041	(G/T)
2245	CakSNP2245	*Kabuli*	Ca_Kabuli_Chr02	3904757	(G/A)
2246	CakSNP2246	*Kabuli*	Ca_Kabuli_Chr02	3904761	(A/G)
2247	CakSNP2247	*Kabuli*	Ca_Kabuli_Chr02	3908928	(A/G)
2248	CakSNP2248	*Kabuli*	Ca_Kabuli_Chr02	3908955	(G/A)
2249	CakSNP2249	*Kabuli*	Ca_Kabuli_Chr02	3909068	(G/A)
2250	CakSNP2250	*Kabuli*	Ca_Kabuli_Chr02	3909062	(C/T)
2251	CakSNP2251	*Kabuli*	Ca_Kabuli_Chr02	3915549	(A/G)
2252	CakSNP2252	*Kabuli*	Ca_Kabuli_Chr02	3930211	(A/G)
2253	CakSNP2253	*Kabuli*	Ca_Kabuli_Chr02	3930182	(A/G)
2254	CakSNP2254	*Kabuli*	Ca_Kabuli_Chr02	3941397	(A/T)
2255	CakSNP2255	*Kabuli*	Ca_Kabuli_Chr02	3994150	(A/C)
2256	CakSNP2256	*Kabuli*	Ca_Kabuli_Chr02	3998491	(A/C)
2257	CakSNP2257	*Kabuli*	Ca_Kabuli_Chr02	3999615	(C/T)
2258	CakSNP2258	*Kabuli*	Ca_Kabuli_Chr02	4039745	(T/C)
2259	CakSNP2259	*Kabuli*	Ca_Kabuli_Chr02	4039854	(G/A)
2260	CakSNP2260	*Kabuli*	Ca_Kabuli_Chr02	4325658	(A/C)
2261	CakSNP2261	*Kabuli*	Ca_Kabuli_Chr02	4325684	(C/A)
2262	CakSNP2262	*Kabuli*	Ca_Kabuli_Chr02	4404813	(C/T)
2263	CakSNP2263	*Kabuli*	Ca_Kabuli_Chr02	4404797	(G/A)
2264	CakSNP2264	*Kabuli*	Ca_Kabuli_Chr02	4966216	(C/T)
2265	CakSNP2265	*Kabuli*	Ca_Kabuli_Chr02	4966168	(G/C)
2266	CakSNP2266	*Kabuli*	Ca_Kabuli_Chr02	4981626	(A/G)
2267	CakSNP2267	*Kabuli*	Ca_Kabuli_Chr02	4981698	(C/T)
2268	CakSNP2268	*Kabuli*	Ca_Kabuli_Chr02	4981686	(T/C)
2269	CakSNP2269	*Kabuli*	Ca_Kabuli_Chr02	4981666	(G/C)
2270	CakSNP2270	*Kabuli*	Ca_Kabuli_Chr02	5017291	(T/A)
2271	CakSNP2271	*Kabuli*	Ca_Kabuli_Chr02	5017330	(G/C)
2272	CakSNP2272	*Kabuli*	Ca_Kabuli_Chr02	5078323	(T/C)
2273	CakSNP2273	*Kabuli*	Ca_Kabuli_Chr02	5079639	(C/T)
2274	CakSNP2274	*Kabuli*	Ca_Kabuli_Chr02	5085761	(A/C)
2275	CakSNP2275	*Kabuli*	Ca_Kabuli_Chr02	5085836	(G/C)
2276	CakSNP2276	*Kabuli*	Ca_Kabuli_Chr02	5100017	(T/A)
2277	CakSNP2277	*Kabuli*	Ca_Kabuli_Chr02	5099985	(A/G)
2278	CakSNP2278	*Kabuli*	Ca_Kabuli_Chr02	5118370	(A/T)
2279	CakSNP2279	*Kabuli*	Ca_Kabuli_Chr02	5118364	(C/T)

S.N.	SNP IDs	Cultivars	Chromosomes/scaffolds	Physical positions (bp)	SNPs
2280	CakSNP2280	*Kabuli*	Ca_Kabuli_Chr02	5141075	(C/T)
2281	CakSNP2281	*Kabuli*	Ca_Kabuli_Chr02	5141081	(A/T)
2282	CakSNP2282	*Kabuli*	Ca_Kabuli_Chr02	5247305	(T/C)
2283	CakSNP2283	*Kabuli*	Ca_Kabuli_Chr02	5247548	(G/T)
2284	CakSNP2284	*Kabuli*	Ca_Kabuli_Chr02	5247547	(A/G)
2285	CakSNP2285	*Kabuli*	Ca_Kabuli_Chr02	5247540	(T/C)
2286	CakSNP2286	*Kabuli*	Ca_Kabuli_Chr02	5247481	(T/A)
2287	CakSNP2287	*Kabuli*	Ca_Kabuli_Chr02	5247557	(A/C)
2288	CakSNP2288	*Kabuli*	Ca_Kabuli_Chr02	5247558	(G/T)
2289	CakSNP2289	*Kabuli*	Ca_Kabuli_Chr02	5259254	(T/C)
2290	CakSNP2290	*Kabuli*	Ca_Kabuli_Chr02	5276208	(T/C)
2291	CakSNP2291	*Kabuli*	Ca_Kabuli_Chr02	5342864	(C/A)
2292	CakSNP2292	*Kabuli*	Ca_Kabuli_Chr02	5342923	(A/G)
2293	CakSNP2293	*Kabuli*	Ca_Kabuli_Chr02	5359177	(C/A)
2294	CakSNP2294	*Kabuli*	Ca_Kabuli_Chr02	5449886	(C/T)
2295	CakSNP2295	*Kabuli*	Ca_Kabuli_Chr02	5449978	(T/C)
2296	CakSNP2296	*Kabuli*	Ca_Kabuli_Chr02	5449907	(G/A)
2297	CakSNP2297	*Kabuli*	Ca_Kabuli_Chr02	5462902	(A/T)
2298	CakSNP2298	*Kabuli*	Ca_Kabuli_Chr02	5468575	(A/T)
2299	CakSNP2299	*Kabuli*	Ca_Kabuli_Chr02	5475329	(C/A)
2300	CakSNP2300	*Kabuli*	Ca_Kabuli_Chr02	5475344	(A/G)
2301	CakSNP2301	*Kabuli*	Ca_Kabuli_Chr02	5475365	(T/C)
2302	CakSNP2302	*Kabuli*	Ca_Kabuli_Chr02	5475407	(C/T)
2303	CakSNP2303	*Kabuli*	Ca_Kabuli_Chr02	5504300	(T/G)
2304	CakSNP2304	*Kabuli*	Ca_Kabuli_Chr02	5601092	(G/C)
2305	CakSNP2305	*Kabuli*	Ca_Kabuli_Chr02	5787862	(G/A)
2306	CakSNP2306	*Kabuli*	Ca_Kabuli_Chr02	5787887	(A/C)
2307	CakSNP2307	*Kabuli*	Ca_Kabuli_Chr02	5877688	(A/G)
2308	CakSNP2308	*Kabuli*	Ca_Kabuli_Chr02	5881503	(A/G)
2309	CakSNP2309	*Kabuli*	Ca_Kabuli_Chr02	5897314	(T/C)
2310	CakSNP2310	*Kabuli*	Ca_Kabuli_Chr02	5904368	(T/C)
2311	CakSNP2311	*Kabuli*	Ca_Kabuli_Chr02	5936796	(C/A)
2312	CakSNP2312	*Kabuli*	Ca_Kabuli_Chr02	5936902	(A/T)
2313	CakSNP2313	*Kabuli*	Ca_Kabuli_Chr02	6042131	(G/A)
2314	CakSNP2314	*Kabuli*	Ca_Kabuli_Chr02	6042139	(C/A)
2315	CakSNP2315	*Kabuli*	Ca_Kabuli_Chr02	6042210	(C/A)
2316	CakSNP2316	*Kabuli*	Ca_Kabuli_Chr02	6060259	(G/C)
2317	CakSNP2317	*Kabuli*	Ca_Kabuli_Chr02	6130301	(G/C)

S.N.	SNP IDs	Cultivars	Chromosomes/scaffolds	Physical positions (bp)	SNPs
2318	CakSNP2318	*Kabuli*	Ca_Kabuli_Chr02	6130257	(C/A)
2319	CakSNP2319	*Kabuli*	Ca_Kabuli_Chr02	6130247	(G/C)
2320	CakSNP2320	*Kabuli*	Ca_Kabuli_Chr02	6162378	(T/G)
2321	CakSNP2321	*Kabuli*	Ca_Kabuli_Chr02	6162406	(G/C)
2322	CakSNP2322	*Kabuli*	Ca_Kabuli_Chr02	6213582	(T/A)
2323	CakSNP2323	*Kabuli*	Ca_Kabuli_Chr02	6213580	(C/A)
2324	CakSNP2324	*Kabuli*	Ca_Kabuli_Chr02	6213566	(C/T)
2325	CakSNP2325	*Kabuli*	Ca_Kabuli_Chr02	6213530	(C/A)
2326	CakSNP2326	*Kabuli*	Ca_Kabuli_Chr02	6213520	(G/T)
2327	CakSNP2327	*Kabuli*	Ca_Kabuli_Chr02	6349208	(T/G)
2328	CakSNP2328	*Kabuli*	Ca_Kabuli_Chr02	6367649	(A/G)
2329	CakSNP2329	*Kabuli*	Ca_Kabuli_Chr02	6367704	(A/G)
2330	CakSNP2330	*Kabuli*	Ca_Kabuli_Chr02	6391819	(T/C)
2331	CakSNP2331	*Kabuli*	Ca_Kabuli_Chr02	6392158	(A/T)
2332	CakSNP2332	*Kabuli*	Ca_Kabuli_Chr02	6392209	(G/A)
2333	CakSNP2333	*Kabuli*	Ca_Kabuli_Chr02	6392272	(T/C)
2334	CakSNP2334	*Kabuli*	Ca_Kabuli_Chr02	6392254	(T/C)
2335	CakSNP2335	*Kabuli*	Ca_Kabuli_Chr02	6392449	(A/G)
2336	CakSNP2336	*Kabuli*	Ca_Kabuli_Chr02	6397551	(A/G)
2337	CakSNP2337	*Kabuli*	Ca_Kabuli_Chr02	6397597	(A/C)
2338	CakSNP2338	*Kabuli*	Ca_Kabuli_Chr02	6408406	(C/A)
2339	CakSNP2339	*Kabuli*	Ca_Kabuli_Chr02	6534776	(C/A)
2340	CakSNP2340	*Kabuli*	Ca_Kabuli_Chr02	6537296	(G/T)
2341	CakSNP2341	*Kabuli*	Ca_Kabuli_Chr02	6537300	(G/T)
2342	CakSNP2342	*Kabuli*	Ca_Kabuli_Chr02	6727932	(G/C)
2343	CakSNP2343	*Kabuli*	Ca_Kabuli_Chr02	6727905	(G/C)
2344	CakSNP2344	*Kabuli*	Ca_Kabuli_Chr02	6727900	(G/C)
2345	CakSNP2345	*Kabuli*	Ca_Kabuli_Chr02	6728716	(A/T)
2346	CakSNP2346	*Kabuli*	Ca_Kabuli_Chr02	6728719	(C/T)
2347	CakSNP2347	*Kabuli*	Ca_Kabuli_Chr02	6728763	(T/C)
2348	CakSNP2348	*Kabuli*	Ca_Kabuli_Chr02	6989950	(A/G)
2349	CakSNP2349	*Kabuli*	Ca_Kabuli_Chr02	6990083	(G/A)
2350	CakSNP2350	*Kabuli*	Ca_Kabuli_Chr02	7007701	(G/C)
2351	CakSNP2351	*Kabuli*	Ca_Kabuli_Chr02	7027093	(G/A)
2352	CakSNP2352	*Kabuli*	Ca_Kabuli_Chr02	7027146	(T/C)
2353	CakSNP2353	*Kabuli*	Ca_Kabuli_Chr02	7047195	(C/A)
2354	CakSNP2354	*Kabuli*	Ca_Kabuli_Chr02	7047196	(T/C)
2355	CakSNP2355	*Kabuli*	Ca_Kabuli_Chr02	7047197	(G/C)

S.N.	SNP IDs	Cultivars	Chromosomes/scaffolds	Physical positions (bp)	SNPs
2356	CakSNP2356	*Kabuli*	Ca_Kabuli_Chr02	7051451	(G/A)
2357	CakSNP2357	*Kabuli*	Ca_Kabuli_Chr02	7125992	(T/G)
2358	CakSNP2358	*Kabuli*	Ca_Kabuli_Chr02	7126018	(T/G)
2359	CakSNP2359	*Kabuli*	Ca_Kabuli_Chr02	7507259	(C/A)
2360	CakSNP2360	*Kabuli*	Ca_Kabuli_Chr02	7633520	(G/A)
2361	CakSNP2361	*Kabuli*	Ca_Kabuli_Chr02	7633547	(C/T)
2362	CakSNP2362	*Kabuli*	Ca_Kabuli_Chr02	7633564	(G/T)
2363	CakSNP2363	*Kabuli*	Ca_Kabuli_Chr02	7633572	(T/C)
2364	CakSNP2364	*Kabuli*	Ca_Kabuli_Chr02	7633605	(T/A)
2365	CakSNP2365	*Kabuli*	Ca_Kabuli_Chr02	7633592	(G/A)
2366	CakSNP2366	*Kabuli*	Ca_Kabuli_Chr02	7633585	(G/A)
2367	CakSNP2367	*Kabuli*	Ca_Kabuli_Chr02	7633869	(G/A)
2368	CakSNP2368	*Kabuli*	Ca_Kabuli_Chr02	7633826	(G/A)
2369	CakSNP2369	*Kabuli*	Ca_Kabuli_Chr02	7633818	(G/A)
2370	CakSNP2370	*Kabuli*	Ca_Kabuli_Chr02	7633816	(G/A)
2371	CakSNP2371	*Kabuli*	Ca_Kabuli_Chr02	7633813	(G/T)
2372	CakSNP2372	*Kabuli*	Ca_Kabuli_Chr02	7633793	(C/T)
2373	CakSNP2373	*Kabuli*	Ca_Kabuli_Chr02	7634041	(G/A)
2374	CakSNP2374	*Kabuli*	Ca_Kabuli_Chr02	7679627	(A/T)
2375	CakSNP2375	*Kabuli*	Ca_Kabuli_Chr02	7679693	(C/G)
2376	CakSNP2376	*Kabuli*	Ca_Kabuli_Chr02	7701189	(T/G)
2377	CakSNP2377	*Kabuli*	Ca_Kabuli_Chr02	7701199	(T/G)
2378	CakSNP2378	*Kabuli*	Ca_Kabuli_Chr02	7701216	(T/G)
2379	CakSNP2379	*Kabuli*	Ca_Kabuli_Chr02	7701230	(T/G)
2380	CakSNP2380	*Kabuli*	Ca_Kabuli_Chr02	7702468	(A/T)
2381	CakSNP2381	*Kabuli*	Ca_Kabuli_Chr02	7708668	(C/T)
2382	CakSNP2382	*Kabuli*	Ca_Kabuli_Chr02	7708663	(T/C)
2383	CakSNP2383	*Kabuli*	Ca_Kabuli_Chr02	7708660	(G/A)
2384	CakSNP2384	*Kabuli*	Ca_Kabuli_Chr02	7708630	(C/A)
2385	CakSNP2385	*Kabuli*	Ca_Kabuli_Chr02	7708617	(G/T)
2386	CakSNP2386	*Kabuli*	Ca_Kabuli_Chr02	7708603	(C/T)
2387	CakSNP2387	*Kabuli*	Ca_Kabuli_Chr02	7708598	(C/T)
2388	CakSNP2388	*Kabuli*	Ca_Kabuli_Chr02	7708596	(T/C)
2389	CakSNP2389	*Kabuli*	Ca_Kabuli_Chr02	7708590	(A/T)
2390	CakSNP2390	*Kabuli*	Ca_Kabuli_Chr02	7708678	(C/T)
2391	CakSNP2391	*Kabuli*	Ca_Kabuli_Chr02	7712181	(C/T)
2392	CakSNP2392	*Kabuli*	Ca_Kabuli_Chr02	7712155	(G/T)
2393	CakSNP2393	*Kabuli*	Ca_Kabuli_Chr02	7714392	(G/A)

S.N.	SNP IDs	Cultivars	Chromosomes/scaffolds	Physical positions (bp)	SNPs
2394	CakSNP2394	*Kabuli*	Ca_Kabuli_Chr02	7714454	(A/G)
2395	CakSNP2395	*Kabuli*	Ca_Kabuli_Chr02	7714463	(T/C)
2396	CakSNP2396	*Kabuli*	Ca_Kabuli_Chr02	7753419	(G/C)
2397	CakSNP2397	*Kabuli*	Ca_Kabuli_Chr02	7753425	(T/C)
2398	CakSNP2398	*Kabuli*	Ca_Kabuli_Chr02	7753440	(C/T)
2399	CakSNP2399	*Kabuli*	Ca_Kabuli_Chr02	7819281	(T/C)
2400	CakSNP2400	*Kabuli*	Ca_Kabuli_Chr02	7870919	(G/T)
2401	CakSNP2401	*Kabuli*	Ca_Kabuli_Chr02	7870943	(T/G)
2402	CakSNP2402	*Kabuli*	Ca_Kabuli_Chr02	7938890	(T/C)
2403	CakSNP2403	*Kabuli*	Ca_Kabuli_Chr02	8337517	(C/T)
2404	CakSNP2404	*Kabuli*	Ca_Kabuli_Chr02	8484840	(A/T)
2405	CakSNP2405	*Kabuli*	Ca_Kabuli_Chr02	8487331	(T/A)
2406	CakSNP2406	*Kabuli*	Ca_Kabuli_Chr02	8487343	(C/T)
2407	CakSNP2407	*Kabuli*	Ca_Kabuli_Chr02	8489241	(C/A)
2408	CakSNP2408	*Kabuli*	Ca_Kabuli_Chr02	8489237	(G/A)
2409	CakSNP2409	*Kabuli*	Ca_Kabuli_Chr02	8489172	(C/A)
2410	CakSNP2410	*Kabuli*	Ca_Kabuli_Chr02	8600256	(C/G)
2411	CakSNP2411	*Kabuli*	Ca_Kabuli_Chr02	8600298	(A/G)
2412	CakSNP2412	*Kabuli*	Ca_Kabuli_Chr02	8600309	(T/C)
2413	CakSNP2413	*Kabuli*	Ca_Kabuli_Chr02	8600450	(C/T)
2414	CakSNP2414	*Kabuli*	Ca_Kabuli_Chr02	8600666	(T/C)
2415	CakSNP2415	*Kabuli*	Ca_Kabuli_Chr02	8685674	(G/A)
2416	CakSNP2416	*Kabuli*	Ca_Kabuli_Chr02	8719725	(T/A)
2417	CakSNP2417	*Kabuli*	Ca_Kabuli_Chr02	8734136	(G/A)
2418	CakSNP2418	*Kabuli*	Ca_Kabuli_Chr02	8802884	(A/G)
2419	CakSNP2419	*Kabuli*	Ca_Kabuli_Chr02	9458933	(C/T)
2420	CakSNP2420	*Kabuli*	Ca_Kabuli_Chr02	9458985	(A/G)
2421	CakSNP2421	*Kabuli*	Ca_Kabuli_Chr02	9473339	(T/C)
2422	CakSNP2422	*Kabuli*	Ca_Kabuli_Chr02	9473353	(C/A)
2423	CakSNP2423	*Kabuli*	Ca_Kabuli_Chr02	9473362	(G/A)
2424	CakSNP2424	*Kabuli*	Ca_Kabuli_Chr02	9473380	(A/G)
2425	CakSNP2425	*Kabuli*	Ca_Kabuli_Chr02	9473393	(G/A)
2426	CakSNP2426	*Kabuli*	Ca_Kabuli_Chr02	9473474	(T/A)
2427	CakSNP2427	*Kabuli*	Ca_Kabuli_Chr02	9473452	(A/G)
2428	CakSNP2428	*Kabuli*	Ca_Kabuli_Chr02	9473430	(A/G)
2429	CakSNP2429	*Kabuli*	Ca_Kabuli_Chr02	9621953	(A/C)
2430	CakSNP2430	*Kabuli*	Ca_Kabuli_Chr02	9740018	(G/A)
2431	CakSNP2431	*Kabuli*	Ca_Kabuli_Chr02	9740017	(C/T)

S.N.	SNP IDs	Cultivars	Chromosomes/scaffolds	Physical positions (bp)	SNPs
2432	CakSNP2432	*Kabuli*	Ca_Kabuli_Chr02	9740010	(A/G)
2433	CakSNP2433	*Kabuli*	Ca_Kabuli_Chr02	9740007	(A/G)
2434	CakSNP2434	*Kabuli*	Ca_Kabuli_Chr02	9739980	(G/C)
2435	CakSNP2435	*Kabuli*	Ca_Kabuli_Chr02	9739974	(T/C)
2436	CakSNP2436	*Kabuli*	Ca_Kabuli_Chr02	9739959	(T/C)
2437	CakSNP2437	*Kabuli*	Ca_Kabuli_Chr02	9739965	(A/G)
2438	CakSNP2438	*Kabuli*	Ca_Kabuli_Chr02	9756955	(G/A)
2439	CakSNP2439	*Kabuli*	Ca_Kabuli_Chr02	9756946	(C/T)
2440	CakSNP2440	*Kabuli*	Ca_Kabuli_Chr02	9756910	(G/A)
2441	CakSNP2441	*Kabuli*	Ca_Kabuli_Chr02	9756887	(G/T)
2442	CakSNP2442	*Kabuli*	Ca_Kabuli_Chr02	9823224	(G/A)
2443	CakSNP2443	*Kabuli*	Ca_Kabuli_Chr02	9823165	(T/C)
2444	CakSNP2444	*Kabuli*	Ca_Kabuli_Chr02	9825526	(C/G)
2445	CakSNP2445	*Kabuli*	Ca_Kabuli_Chr02	9841625	(A/G)
2446	CakSNP2446	*Kabuli*	Ca_Kabuli_Chr02	9862794	(A/G)
2447	CakSNP2447	*Kabuli*	Ca_Kabuli_Chr02	9906042	(T/G)
2448	CakSNP2448	*Kabuli*	Ca_Kabuli_Chr02	9906077	(T/A)
2449	CakSNP2449	*Kabuli*	Ca_Kabuli_Chr02	9918536	(T/A)
2450	CakSNP2450	*Kabuli*	Ca_Kabuli_Chr02	9943370	(A/G)
2451	CakSNP2451	*Kabuli*	Ca_Kabuli_Chr02	9943374	(C/T)
2452	CakSNP2452	*Kabuli*	Ca_Kabuli_Chr02	9943404	(T/G)
2453	CakSNP2453	*Kabuli*	Ca_Kabuli_Chr02	9986639	(C/T)
2454	CakSNP2454	*Kabuli*	Ca_Kabuli_Chr02	9986683	(C/T)
2455	CakSNP2455	*Kabuli*	Ca_Kabuli_Chr02	10004694	(G/A)
2456	CakSNP2456	*Kabuli*	Ca_Kabuli_Chr02	10025451	(G/A)
2457	CakSNP2457	*Kabuli*	Ca_Kabuli_Chr02	10033991	(T/C)
2458	CakSNP2458	*Kabuli*	Ca_Kabuli_Chr02	10060196	(G/T)
2459	CakSNP2459	*Kabuli*	Ca_Kabuli_Chr02	10060200	(G/T)
2460	CakSNP2460	*Kabuli*	Ca_Kabuli_Chr02	10060207	(C/T)
2461	CakSNP2461	*Kabuli*	Ca_Kabuli_Chr02	10067093	(G/C)
2462	CakSNP2462	*Kabuli*	Ca_Kabuli_Chr02	10067078	(T/G)
2463	CakSNP2463	*Kabuli*	Ca_Kabuli_Chr02	10067061	(T/G)
2464	CakSNP2464	*Kabuli*	Ca_Kabuli_Chr02	10067195	(T/A)
2465	CakSNP2465	*Kabuli*	Ca_Kabuli_Chr02	10067262	(A/C)
2466	CakSNP2466	*Kabuli*	Ca_Kabuli_Chr02	10067278	(G/T)
2467	CakSNP2467	*Kabuli*	Ca_Kabuli_Chr02	10067285	(T/G)
2468	CakSNP2468	*Kabuli*	Ca_Kabuli_Chr02	10067286	(A/T)
2469	CakSNP2469	*Kabuli*	Ca_Kabuli_Chr02	10067451	(A/T)

S.N.	SNP IDs	Cultivars	Chromosomes/scaffolds	Physical positions (bp)	SNPs
2470	CakSNP2470	*Kabuli*	Ca_Kabuli_Chr02	10067516	(G/A)
2471	CakSNP2471	*Kabuli*	Ca_Kabuli_Chr02	10067538	(C/T)
2472	CakSNP2472	*Kabuli*	Ca_Kabuli_Chr02	10068214	(A/G)
2473	CakSNP2473	*Kabuli*	Ca_Kabuli_Chr02	10220396	(T/C)
2474	CakSNP2474	*Kabuli*	Ca_Kabuli_Chr02	10270474	(C/A)
2475	CakSNP2475	*Kabuli*	Ca_Kabuli_Chr02	10284426	(C/G)
2476	CakSNP2476	*Kabuli*	Ca_Kabuli_Chr02	10294698	(A/G)
2477	CakSNP2477	*Kabuli*	Ca_Kabuli_Chr02	10294635	(T/C)
2478	CakSNP2478	*Kabuli*	Ca_Kabuli_Chr02	10322943	(T/C)
2479	CakSNP2479	*Kabuli*	Ca_Kabuli_Chr02	10352705	(T/A)
2480	CakSNP2480	*Kabuli*	Ca_Kabuli_Chr02	10358484	(C/T)
2481	CakSNP2481	*Kabuli*	Ca_Kabuli_Chr02	10358496	(G/T)
2482	CakSNP2482	*Kabuli*	Ca_Kabuli_Chr02	10377695	(G/A)
2483	CakSNP2483	*Kabuli*	Ca_Kabuli_Chr02	10434727	(T/C)
2484	CakSNP2484	*Kabuli*	Ca_Kabuli_Chr02	10434726	(G/C)
2485	CakSNP2485	*Kabuli*	Ca_Kabuli_Chr02	10434714	(C/G)
2486	CakSNP2486	*Kabuli*	Ca_Kabuli_Chr02	10540735	(C/T)
2487	CakSNP2487	*Kabuli*	Ca_Kabuli_Chr02	10637631	(C/A)
2488	CakSNP2488	*Kabuli*	Ca_Kabuli_Chr02	10656518	(A/T)
2489	CakSNP2489	*Kabuli*	Ca_Kabuli_Chr02	10705902	(C/A)
2490	CakSNP2490	*Kabuli*	Ca_Kabuli_Chr02	10705923	(T/A)
2491	CakSNP2491	*Kabuli*	Ca_Kabuli_Chr02	10705979	(C/A)
2492	CakSNP2492	*Kabuli*	Ca_Kabuli_Chr02	10705982	(C/T)
2493	CakSNP2493	*Kabuli*	Ca_Kabuli_Chr02	10760939	(G/A)
2494	CakSNP2494	*Kabuli*	Ca_Kabuli_Chr02	10761036	(C/G)
2495	CakSNP2495	*Kabuli*	Ca_Kabuli_Chr02	11799472	(G/A)
2496	CakSNP2496	*Kabuli*	Ca_Kabuli_Chr02	11799462	(G/A)
2497	CakSNP2497	*Kabuli*	Ca_Kabuli_Chr02	12252285	(T/G)
2498	CakSNP2498	*Kabuli*	Ca_Kabuli_Chr02	12252370	(A/G)
2499	CakSNP2499	*Kabuli*	Ca_Kabuli_Chr02	12278583	(G/A)
2500	CakSNP2500	*Kabuli*	Ca_Kabuli_Chr02	12278584	(T/C)
2501	CakSNP2501	*Kabuli*	Ca_Kabuli_Chr02	12278606	(T/C)
2502	CakSNP2502	*Kabuli*	Ca_Kabuli_Chr02	12281599	(A/C)
2503	CakSNP2503	*Kabuli*	Ca_Kabuli_Chr02	12357732	(A/G)
2504	CakSNP2504	*Kabuli*	Ca_Kabuli_Chr02	12389166	(G/T)
2505	CakSNP2505	*Kabuli*	Ca_Kabuli_Chr02	12389112	(G/A)
2506	CakSNP2506	*Kabuli*	Ca_Kabuli_Chr02	12515595	(A/G)
2507	CakSNP2507	*Kabuli*	Ca_Kabuli_Chr02	12528825	(C/G)

S.N.	SNP IDs	Cultivars	Chromosomes/scaffolds	Physical positions (bp)	SNPs
2508	CakSNP2508	*Kabuli*	Ca_Kabuli_Chr02	12710137	(G/A)
2509	CakSNP2509	*Kabuli*	Ca_Kabuli_Chr02	12737814	(T/C)
2510	CakSNP2510	*Kabuli*	Ca_Kabuli_Chr02	12794438	(G/A)
2511	CakSNP2511	*Kabuli*	Ca_Kabuli_Chr02	12854610	(A/G)
2512	CakSNP2512	*Kabuli*	Ca_Kabuli_Chr02	12854591	(T/A)
2513	CakSNP2513	*Kabuli*	Ca_Kabuli_Chr02	12854586	(G/A)
2514	CakSNP2514	*Kabuli*	Ca_Kabuli_Chr02	12862414	(G/C)
2515	CakSNP2515	*Kabuli*	Ca_Kabuli_Chr02	13079417	(T/G)
2516	CakSNP2516	*Kabuli*	Ca_Kabuli_Chr02	13079445	(T/G)
2517	CakSNP2517	*Kabuli*	Ca_Kabuli_Chr02	13217781	(T/C)
2518	CakSNP2518	*Kabuli*	Ca_Kabuli_Chr02	13355018	(T/G)
2519	CakSNP2519	*Kabuli*	Ca_Kabuli_Chr02	13355022	(G/T)
2520	CakSNP2520	*Kabuli*	Ca_Kabuli_Chr02	13355033	(A/T)
2521	CakSNP2521	*Kabuli*	Ca_Kabuli_Chr02	13355615	(T/G)
2522	CakSNP2522	*Kabuli*	Ca_Kabuli_Chr02	13425568	(T/A)
2523	CakSNP2523	*Kabuli*	Ca_Kabuli_Chr02	13434904	(G/A)
2524	CakSNP2524	*Kabuli*	Ca_Kabuli_Chr02	13434884	(T/A)
2525	CakSNP2525	*Kabuli*	Ca_Kabuli_Chr02	13435043	(C/T)
2526	CakSNP2526	*Kabuli*	Ca_Kabuli_Chr02	13435030	(G/C)
2527	CakSNP2527	*Kabuli*	Ca_Kabuli_Chr02	13434995	(A/C)
2528	CakSNP2528	*Kabuli*	Ca_Kabuli_Chr02	13437317	(A/G)
2529	CakSNP2529	*Kabuli*	Ca_Kabuli_Chr02	13529057	(G/A)
2530	CakSNP2530	*Kabuli*	Ca_Kabuli_Chr02	13552505	(T/A)
2531	CakSNP2531	*Kabuli*	Ca_Kabuli_Chr02	13554038	(T/C)
2532	CakSNP2532	*Kabuli*	Ca_Kabuli_Chr02	13554045	(T/C)
2533	CakSNP2533	*Kabuli*	Ca_Kabuli_Chr02	13554047	(A/T)
2534	CakSNP2534	*Kabuli*	Ca_Kabuli_Chr02	13703083	(G/A)
2535	CakSNP2535	*Kabuli*	Ca_Kabuli_Chr02	13782906	(C/A)
2536	CakSNP2536	*Kabuli*	Ca_Kabuli_Chr02	14101913	(T/A)
2537	CakSNP2537	*Kabuli*	Ca_Kabuli_Chr02	14134413	(A/C)
2538	CakSNP2538	*Kabuli*	Ca_Kabuli_Chr02	14134676	(T/C)
2539	CakSNP2539	*Kabuli*	Ca_Kabuli_Chr02	14136062	(C/A)
2540	CakSNP2540	*Kabuli*	Ca_Kabuli_Chr02	14136019	(A/G)
2541	CakSNP2541	*Kabuli*	Ca_Kabuli_Chr02	14136013	(T/G)
2542	CakSNP2542	*Kabuli*	Ca_Kabuli_Chr02	14136223	(A/C)
2543	CakSNP2543	*Kabuli*	Ca_Kabuli_Chr02	15160301	(T/C)
2544	CakSNP2544	*Kabuli*	Ca_Kabuli_Chr02	15178565	(G/A)
2545	CakSNP2545	*Kabuli*	Ca_Kabuli_Chr02	15178564	(A/G)

S.N.	SNP IDs	Cultivars	Chromosomes/scaffolds	Physical positions (bp)	SNPs
2546	CakSNP2546	*Kabuli*	Ca_Kabuli_Chr02	15178540	(A/G)
2547	CakSNP2547	*Kabuli*	Ca_Kabuli_Chr02	15181635	(G/T)
2548	CakSNP2548	*Kabuli*	Ca_Kabuli_Chr02	15234951	(C/A)
2549	CakSNP2549	*Kabuli*	Ca_Kabuli_Chr02	15389412	(G/A)
2550	CakSNP2550	*Kabuli*	Ca_Kabuli_Chr02	15390673	(A/G)
2551	CakSNP2551	*Kabuli*	Ca_Kabuli_Chr02	15390731	(G/A)
2552	CakSNP2552	*Kabuli*	Ca_Kabuli_Chr02	15390684	(A/G)
2553	CakSNP2553	*Kabuli*	Ca_Kabuli_Chr02	15688657	(G/T)
2554	CakSNP2554	*Kabuli*	Ca_Kabuli_Chr02	15817688	(T/C)
2555	CakSNP2555	*Kabuli*	Ca_Kabuli_Chr02	16460174	(G/A)
2556	CakSNP2556	*Kabuli*	Ca_Kabuli_Chr02	16460142	(A/G)
2557	CakSNP2557	*Kabuli*	Ca_Kabuli_Chr02	16460097	(C/A)
2558	CakSNP2558	*Kabuli*	Ca_Kabuli_Chr02	16460106	(C/T)
2559	CakSNP2559	*Kabuli*	Ca_Kabuli_Chr02	16460175	(C/A)
2560	CakSNP2560	*Kabuli*	Ca_Kabuli_Chr02	16462095	(A/G)
2561	CakSNP2561	*Kabuli*	Ca_Kabuli_Chr02	16462089	(C/T)
2562	CakSNP2562	*Kabuli*	Ca_Kabuli_Chr02	16462056	(A/C)
2563	CakSNP2563	*Kabuli*	Ca_Kabuli_Chr02	16462038	(G/T)
2564	CakSNP2564	*Kabuli*	Ca_Kabuli_Chr02	16555733	(C/T)
2565	CakSNP2565	*Kabuli*	Ca_Kabuli_Chr02	16555790	(C/G)
2566	CakSNP2566	*Kabuli*	Ca_Kabuli_Chr02	16563117	(A/T)
2567	CakSNP2567	*Kabuli*	Ca_Kabuli_Chr02	16563121	(A/T)
2568	CakSNP2568	*Kabuli*	Ca_Kabuli_Chr02	16563123	(C/T)
2569	CakSNP2569	*Kabuli*	Ca_Kabuli_Chr02	16563125	(A/T)
2570	CakSNP2570	*Kabuli*	Ca_Kabuli_Chr02	16563133	(A/T)
2571	CakSNP2571	*Kabuli*	Ca_Kabuli_Chr02	16705119	(C/T)
2572	CakSNP2572	*Kabuli*	Ca_Kabuli_Chr02	17450780	(C/T)
2573	CakSNP2573	*Kabuli*	Ca_Kabuli_Chr02	17578623	(G/A)
2574	CakSNP2574	*Kabuli*	Ca_Kabuli_Chr02	17578633	(C/G)
2575	CakSNP2575	*Kabuli*	Ca_Kabuli_Chr02	17578659	(A/G)
2576	CakSNP2576	*Kabuli*	Ca_Kabuli_Chr02	17578689	(G/A)
2577	CakSNP2577	*Kabuli*	Ca_Kabuli_Chr02	17578663	(C/A)
2578	CakSNP2578	*Kabuli*	Ca_Kabuli_Chr02	17578676	(T/C)
2579	CakSNP2579	*Kabuli*	Ca_Kabuli_Chr02	17578701	(A/C)
2580	CakSNP2580	*Kabuli*	Ca_Kabuli_Chr02	17578706	(A/T)
2581	CakSNP2581	*Kabuli*	Ca_Kabuli_Chr02	17578726	(A/T)
2582	CakSNP2582	*Kabuli*	Ca_Kabuli_Chr02	17578740	(A/G)
2583	CakSNP2583	*Kabuli*	Ca_Kabuli_Chr02	17681915	(C/T)

S.N.	SNP IDs	Cultivars	Chromosomes/scaffolds	Physical positions (bp)	SNPs
2584	CakSNP2584	*Kabuli*	Ca_Kabuli_Chr02	17716022	(A/T)
2585	CakSNP2585	*Kabuli*	Ca_Kabuli_Chr02	17727340	(G/A)
2586	CakSNP2586	*Kabuli*	Ca_Kabuli_Chr02	17727362	(G/C)
2587	CakSNP2587	*Kabuli*	Ca_Kabuli_Chr02	17727403	(C/T)
2588	CakSNP2588	*Kabuli*	Ca_Kabuli_Chr02	17839209	(T/A)
2589	CakSNP2589	*Kabuli*	Ca_Kabuli_Chr02	17839297	(T/C)
2590	CakSNP2590	*Kabuli*	Ca_Kabuli_Chr02	17910111	(C/G)
2591	CakSNP2591	*Kabuli*	Ca_Kabuli_Chr02	17910145	(C/T)
2592	CakSNP2592	*Kabuli*	Ca_Kabuli_Chr02	17910783	(A/T)
2593	CakSNP2593	*Kabuli*	Ca_Kabuli_Chr02	18312200	(T/C)
2594	CakSNP2594	*Kabuli*	Ca_Kabuli_Chr02	18395345	(A/C)
2595	CakSNP2595	*Kabuli*	Ca_Kabuli_Chr02	18395410	(T/C)
2596	CakSNP2596	*Kabuli*	Ca_Kabuli_Chr02	18395378	(A/G)
2597	CakSNP2597	*Kabuli*	Ca_Kabuli_Chr02	18546345	(T/C)
2598	CakSNP2598	*Kabuli*	Ca_Kabuli_Chr02	18546366	(A/T)
2599	CakSNP2599	*Kabuli*	Ca_Kabuli_Chr02	18631367	(T/A)
2600	CakSNP2600	*Kabuli*	Ca_Kabuli_Chr02	18631383	(A/G)
2601	CakSNP2601	*Kabuli*	Ca_Kabuli_Chr02	18631404	(T/G)
2602	CakSNP2602	*Kabuli*	Ca_Kabuli_Chr02	18631412	(T/G)
2603	CakSNP2603	*Kabuli*	Ca_Kabuli_Chr02	18631522	(A/C)
2604	CakSNP2604	*Kabuli*	Ca_Kabuli_Chr02	18671692	(C/A)
2605	CakSNP2605	*Kabuli*	Ca_Kabuli_Chr02	18671672	(T/A)
2606	CakSNP2606	*Kabuli*	Ca_Kabuli_Chr02	18671666	(C/T)
2607	CakSNP2607	*Kabuli*	Ca_Kabuli_Chr02	18671637	(G/A)
2608	CakSNP2608	*Kabuli*	Ca_Kabuli_Chr02	18671618	(T/G)
2609	CakSNP2609	*Kabuli*	Ca_Kabuli_Chr02	18671723	(C/G)
2610	CakSNP2610	*Kabuli*	Ca_Kabuli_Chr02	18671746	(C/T)
2611	CakSNP2611	*Kabuli*	Ca_Kabuli_Chr02	18671756	(T/A)
2612	CakSNP2612	*Kabuli*	Ca_Kabuli_Chr02	18671765	(C/A)
2613	CakSNP2613	*Kabuli*	Ca_Kabuli_Chr02	18671767	(G/C)
2614	CakSNP2614	*Kabuli*	Ca_Kabuli_Chr02	18671785	(G/A)
2615	CakSNP2615	*Kabuli*	Ca_Kabuli_Chr02	18671824	(C/T)
2616	CakSNP2616	*Kabuli*	Ca_Kabuli_Chr02	20392224	(A/G)
2617	CakSNP2617	*Kabuli*	Ca_Kabuli_Chr02	20762187	(A/G)
2618	CakSNP2618	*Kabuli*	Ca_Kabuli_Chr02	20762205	(A/T)
2619	CakSNP2619	*Kabuli*	Ca_Kabuli_Chr02	21000509	(C/T)
2620	CakSNP2620	*Kabuli*	Ca_Kabuli_Chr02	21000466	(C/T)
2621	CakSNP2621	*Kabuli*	Ca_Kabuli_Chr02	21000463	(C/T)

S.N.	SNP IDs	Cultivars	Chromosomes/scaffolds	Physical positions (bp)	SNPs
2622	CakSNP2622	*Kabuli*	Ca_Kabuli_Chr02	21849874	(A/T)
2623	CakSNP2623	*Kabuli*	Ca_Kabuli_Chr02	21906952	(A/G)
2624	CakSNP2624	*Kabuli*	Ca_Kabuli_Chr02	21965365	(T/C)
2625	CakSNP2625	*Kabuli*	Ca_Kabuli_Chr02	22158593	(C/T)
2626	CakSNP2626	*Kabuli*	Ca_Kabuli_Chr02	22158602	(C/T)
2627	CakSNP2627	*Kabuli*	Ca_Kabuli_Chr02	22190700	(C/T)
2628	CakSNP2628	*Kabuli*	Ca_Kabuli_Chr02	22190743	(C/T)
2629	CakSNP2629	*Kabuli*	Ca_Kabuli_Chr02	22190748	(G/T)
2630	CakSNP2630	*Kabuli*	Ca_Kabuli_Chr02	22190751	(C/T)
2631	CakSNP2631	*Kabuli*	Ca_Kabuli_Chr02	22190756	(C/A)
2632	CakSNP2632	*Kabuli*	Ca_Kabuli_Chr02	22190753	(C/T)
2633	CakSNP2633	*Kabuli*	Ca_Kabuli_Chr02	22190790	(G/A)
2634	CakSNP2634	*Kabuli*	Ca_Kabuli_Chr02	22190815	(C/T)
2635	CakSNP2635	*Kabuli*	Ca_Kabuli_Chr02	22205024	(G/C)
2636	CakSNP2636	*Kabuli*	Ca_Kabuli_Chr02	22347405	(C/T)
2637	CakSNP2637	*Kabuli*	Ca_Kabuli_Chr02	22415930	(C/T)
2638	CakSNP2638	*Kabuli*	Ca_Kabuli_Chr02	22416899	(A/C)
2639	CakSNP2639	*Kabuli*	Ca_Kabuli_Chr02	22419501	(C/G)
2640	CakSNP2640	*Kabuli*	Ca_Kabuli_Chr02	22419513	(T/C)
2641	CakSNP2641	*Kabuli*	Ca_Kabuli_Chr02	22419514	(C/T)
2642	CakSNP2642	*Kabuli*	Ca_Kabuli_Chr02	22419529	(T/C)
2643	CakSNP2643	*Kabuli*	Ca_Kabuli_Chr02	22419625	(C/T)
2644	CakSNP2644	*Kabuli*	Ca_Kabuli_Chr02	22419633	(C/T)
2645	CakSNP2645	*Kabuli*	Ca_Kabuli_Chr02	22419634	(G/C)
2646	CakSNP2646	*Kabuli*	Ca_Kabuli_Chr02	22419679	(G/A)
2647	CakSNP2647	*Kabuli*	Ca_Kabuli_Chr02	22457234	(G/A)
2648	CakSNP2648	*Kabuli*	Ca_Kabuli_Chr02	22457221	(T/A)
2649	CakSNP2649	*Kabuli*	Ca_Kabuli_Chr02	22457215	(G/A)
2650	CakSNP2650	*Kabuli*	Ca_Kabuli_Chr02	22457181	(G/T)
2651	CakSNP2651	*Kabuli*	Ca_Kabuli_Chr02	22457233	(C/T)
2652	CakSNP2652	*Kabuli*	Ca_Kabuli_Chr02	22457255	(C/T)
2653	CakSNP2653	*Kabuli*	Ca_Kabuli_Chr02	22457263	(G/A)
2654	CakSNP2654	*Kabuli*	Ca_Kabuli_Chr02	22457257	(G/A)
2655	CakSNP2655	*Kabuli*	Ca_Kabuli_Chr02	22483072	(C/T)
2656	CakSNP2656	*Kabuli*	Ca_Kabuli_Chr02	22483067	(G/A)
2657	CakSNP2657	*Kabuli*	Ca_Kabuli_Chr02	22483052	(C/T)
2658	CakSNP2658	*Kabuli*	Ca_Kabuli_Chr02	22483019	(A/T)
2659	CakSNP2659	*Kabuli*	Ca_Kabuli_Chr02	22703091	(A/C)

S.N.	SNP IDs	Cultivars	Chromosomes/scaffolds	Physical positions (bp)	SNPs
2660	CakSNP2660	*Kabuli*	Ca_Kabuli_Chr02	22820616	(C/G)
2661	CakSNP2661	*Kabuli*	Ca_Kabuli_Chr02	22820588	(A/C)
2662	CakSNP2662	*Kabuli*	Ca_Kabuli_Chr02	22897694	(G/A)
2663	CakSNP2663	*Kabuli*	Ca_Kabuli_Chr02	22897647	(T/C)
2664	CakSNP2664	*Kabuli*	Ca_Kabuli_Chr02	23123734	(C/T)
2665	CakSNP2665	*Kabuli*	Ca_Kabuli_Chr02	23141564	(C/T)
2666	CakSNP2666	*Kabuli*	Ca_Kabuli_Chr02	23181353	(C/T)
2667	CakSNP2667	*Kabuli*	Ca_Kabuli_Chr02	23247801	(C/T)
2668	CakSNP2668	*Kabuli*	Ca_Kabuli_Chr02	23282374	(T/C)
2669	CakSNP2669	*Kabuli*	Ca_Kabuli_Chr02	23297412	(G/A)
2670	CakSNP2670	*Kabuli*	Ca_Kabuli_Chr02	23329325	(G/T)
2671	CakSNP2671	*Kabuli*	Ca_Kabuli_Chr02	23329424	(C/T)
2672	CakSNP2672	*Kabuli*	Ca_Kabuli_Chr02	23329440	(G/A)
2673	CakSNP2673	*Kabuli*	Ca_Kabuli_Chr02	23417518	(T/G)
2674	CakSNP2674	*Kabuli*	Ca_Kabuli_Chr02	23435939	(A/G)
2675	CakSNP2675	*Kabuli*	Ca_Kabuli_Chr02	23439966	(T/C)
2676	CakSNP2676	*Kabuli*	Ca_Kabuli_Chr02	23441740	(A/G)
2677	CakSNP2677	*Kabuli*	Ca_Kabuli_Chr02	23910254	(T/C)
2678	CakSNP2678	*Kabuli*	Ca_Kabuli_Chr02	23961003	(G/A)
2679	CakSNP2679	*Kabuli*	Ca_Kabuli_Chr02	24048087	(A/C)
2680	CakSNP2680	*Kabuli*	Ca_Kabuli_Chr02	24098243	(T/C)
2681	CakSNP2681	*Kabuli*	Ca_Kabuli_Chr02	24101371	(C/T)
2682	CakSNP2682	*Kabuli*	Ca_Kabuli_Chr02	24164335	(A/T)
2683	CakSNP2683	*Kabuli*	Ca_Kabuli_Chr02	24164840	(T/A)
2684	CakSNP2684	*Kabuli*	Ca_Kabuli_Chr02	24164903	(T/C)
2685	CakSNP2685	*Kabuli*	Ca_Kabuli_Chr02	24164900	(A/T)
2686	CakSNP2686	*Kabuli*	Ca_Kabuli_Chr02	24221498	(G/T)
2687	CakSNP2687	*Kabuli*	Ca_Kabuli_Chr02	24291665	(T/C)
2688	CakSNP2688	*Kabuli*	Ca_Kabuli_Chr02	24353303	(A/G)
2689	CakSNP2689	*Kabuli*	Ca_Kabuli_Chr02	24353243	(A/T)
2690	CakSNP2690	*Kabuli*	Ca_Kabuli_Chr02	24511053	(G/A)
2691	CakSNP2691	*Kabuli*	Ca_Kabuli_Chr02	24511114	(G/A)
2692	CakSNP2692	*Kabuli*	Ca_Kabuli_Chr02	24511121	(G/A)
2693	CakSNP2693	*Kabuli*	Ca_Kabuli_Chr02	24511119	(T/G)
2694	CakSNP2694	*Kabuli*	Ca_Kabuli_Chr02	24511116	(C/A)
2695	CakSNP2695	*Kabuli*	Ca_Kabuli_Chr02	24586360	(T/C)
2696	CakSNP2696	*Kabuli*	Ca_Kabuli_Chr02	24709014	(C/G)
2697	CakSNP2697	*Kabuli*	Ca_Kabuli_Chr02	24709103	(G/A)

S.N.	SNP IDs	Cultivars	Chromosomes/scaffolds	Physical positions (bp)	SNPs
2698	CakSNP2698	*Kabuli*	Ca_Kabuli_Chr02	24709139	(A/G)
2699	CakSNP2699	*Kabuli*	Ca_Kabuli_Chr02	24709159	(T/A)
2700	CakSNP2700	*Kabuli*	Ca_Kabuli_Chr02	24709295	(G/A)
2701	CakSNP2701	*Kabuli*	Ca_Kabuli_Chr02	24741195	(C/T)
2702	CakSNP2702	*Kabuli*	Ca_Kabuli_Chr02	24744726	(A/T)
2703	CakSNP2703	*Kabuli*	Ca_Kabuli_Chr02	24817957	(G/A)
2704	CakSNP2704	*Kabuli*	Ca_Kabuli_Chr02	24817945	(C/T)
2705	CakSNP2705	*Kabuli*	Ca_Kabuli_Chr02	24817940	(G/A)
2706	CakSNP2706	*Kabuli*	Ca_Kabuli_Chr02	24817934	(A/G)
2707	CakSNP2707	*Kabuli*	Ca_Kabuli_Chr02	24817921	(G/A)
2708	CakSNP2708	*Kabuli*	Ca_Kabuli_Chr02	24876355	(T/G)
2709	CakSNP2709	*Kabuli*	Ca_Kabuli_Chr02	24911080	(T/C)
2710	CakSNP2710	*Kabuli*	Ca_Kabuli_Chr02	24911154	(G/C)
2711	CakSNP2711	*Kabuli*	Ca_Kabuli_Chr02	25022971	(A/G)
2712	CakSNP2712	*Kabuli*	Ca_Kabuli_Chr02	25104319	(G/T)
2713	CakSNP2713	*Kabuli*	Ca_Kabuli_Chr02	25104291	(A/T)
2714	CakSNP2714	*Kabuli*	Ca_Kabuli_Chr02	25108249	(T/C)
2715	CakSNP2715	*Kabuli*	Ca_Kabuli_Chr02	25126798	(C/T)
2716	CakSNP2716	*Kabuli*	Ca_Kabuli_Chr02	25126804	(A/G)
2717	CakSNP2717	*Kabuli*	Ca_Kabuli_Chr02	25126837	(G/T)
2718	CakSNP2718	*Kabuli*	Ca_Kabuli_Chr02	25126910	(G/C)
2719	CakSNP2719	*Kabuli*	Ca_Kabuli_Chr02	25163062	(C/G)
2720	CakSNP2720	*Kabuli*	Ca_Kabuli_Chr02	25194583	(G/A)
2721	CakSNP2721	*Kabuli*	Ca_Kabuli_Chr02	25317391	(A/G)
2722	CakSNP2722	*Kabuli*	Ca_Kabuli_Chr02	25321993	(C/A)
2723	CakSNP2723	*Kabuli*	Ca_Kabuli_Chr02	25493973	(T/G)
2724	CakSNP2724	*Kabuli*	Ca_Kabuli_Chr02	25507637	(C/G)
2725	CakSNP2725	*Kabuli*	Ca_Kabuli_Chr02	25507681	(A/G)
2726	CakSNP2726	*Kabuli*	Ca_Kabuli_Chr02	25508579	(C/A)
2727	CakSNP2727	*Kabuli*	Ca_Kabuli_Chr02	25508679	(A/G)
2728	CakSNP2728	*Kabuli*	Ca_Kabuli_Chr02	25508660	(G/C)
2729	CakSNP2729	*Kabuli*	Ca_Kabuli_Chr02	25508920	(G/A)
2730	CakSNP2730	*Kabuli*	Ca_Kabuli_Chr02	25508969	(T/C)
2731	CakSNP2731	*Kabuli*	Ca_Kabuli_Chr02	25508964	(A/C)
2732	CakSNP2732	*Kabuli*	Ca_Kabuli_Chr02	25508963	(A/G)
2733	CakSNP2733	*Kabuli*	Ca_Kabuli_Chr02	25508935	(A/T)
2734	CakSNP2734	*Kabuli*	Ca_Kabuli_Chr02	25509087	(C/A)
2735	CakSNP2735	*Kabuli*	Ca_Kabuli_Chr02	25509093	(C/G)

S.N.	SNP IDs	Cultivars	Chromosomes/scaffolds	Physical positions (bp)	SNPs
2736	CakSNP2736	*Kabuli*	Ca_Kabuli_Chr02	25509095	(G/A)
2737	CakSNP2737	*Kabuli*	Ca_Kabuli_Chr02	25509203	(T/C)
2738	CakSNP2738	*Kabuli*	Ca_Kabuli_Chr02	25509202	(T/A)
2739	CakSNP2739	*Kabuli*	Ca_Kabuli_Chr02	25667417	(G/C)
2740	CakSNP2740	*Kabuli*	Ca_Kabuli_Chr02	25667427	(T/C)
2741	CakSNP2741	*Kabuli*	Ca_Kabuli_Chr02	25788124	(T/A)
2742	CakSNP2742	*Kabuli*	Ca_Kabuli_Chr02	25788139	(T/C)
2743	CakSNP2743	*Kabuli*	Ca_Kabuli_Chr02	25880318	(T/C)
2744	CakSNP2744	*Kabuli*	Ca_Kabuli_Chr02	25898931	(T/C)
2745	CakSNP2745	*Kabuli*	Ca_Kabuli_Chr02	25930490	(G/A)
2746	CakSNP2746	*Kabuli*	Ca_Kabuli_Chr02	26051667	(T/G)
2747	CakSNP2747	*Kabuli*	Ca_Kabuli_Chr02	26092881	(C/G)
2748	CakSNP2748	*Kabuli*	Ca_Kabuli_Chr02	26092913	(C/A)
2749	CakSNP2749	*Kabuli*	Ca_Kabuli_Chr02	26092928	(T/G)
2750	CakSNP2750	*Kabuli*	Ca_Kabuli_Chr02	26092991	(T/C)
2751	CakSNP2751	*Kabuli*	Ca_Kabuli_Chr02	26121495	(A/C)
2752	CakSNP2752	*Kabuli*	Ca_Kabuli_Chr02	26121491	(T/G)
2753	CakSNP2753	*Kabuli*	Ca_Kabuli_Chr02	26417311	(T/A)
2754	CakSNP2754	*Kabuli*	Ca_Kabuli_Chr02	26417281	(A/G)
2755	CakSNP2755	*Kabuli*	Ca_Kabuli_Chr02	26417256	(T/C)
2756	CakSNP2756	*Kabuli*	Ca_Kabuli_Chr02	26431061	(T/C)
2757	CakSNP2757	*Kabuli*	Ca_Kabuli_Chr02	26431058	(G/C)
2758	CakSNP2758	*Kabuli*	Ca_Kabuli_Chr02	26431052	(T/C)
2759	CakSNP2759	*Kabuli*	Ca_Kabuli_Chr02	26461867	(C/G)
2760	CakSNP2760	*Kabuli*	Ca_Kabuli_Chr02	26461986	(A/C)
2761	CakSNP2761	*Kabuli*	Ca_Kabuli_Chr02	26461960	(T/A)
2762	CakSNP2762	*Kabuli*	Ca_Kabuli_Chr02	26486049	(G/A)
2763	CakSNP2763	*Kabuli*	Ca_Kabuli_Chr02	26486065	(C/T)
2764	CakSNP2764	*Kabuli*	Ca_Kabuli_Chr02	26486109	(T/C)
2765	CakSNP2765	*Kabuli*	Ca_Kabuli_Chr02	26502153	(T/C)
2766	CakSNP2766	*Kabuli*	Ca_Kabuli_Chr02	26503262	(C/A)
2767	CakSNP2767	*Kabuli*	Ca_Kabuli_Chr02	26567088	(T/C)
2768	CakSNP2768	*Kabuli*	Ca_Kabuli_Chr02	26585842	(T/A)
2769	CakSNP2769	*Kabuli*	Ca_Kabuli_Chr02	26601337	(C/A)
2770	CakSNP2770	*Kabuli*	Ca_Kabuli_Chr02	26601331	(G/C)
2771	CakSNP2771	*Kabuli*	Ca_Kabuli_Chr02	26706849	(A/G)
2772	CakSNP2772	*Kabuli*	Ca_Kabuli_Chr02	27057582	(C/T)
2773	CakSNP2773	*Kabuli*	Ca_Kabuli_Chr02	27063749	(C/A)

S.N.	SNP IDs	Cultivars	Chromosomes/scaffolds	Physical positions (bp)	SNPs
2774	CakSNP2774	*Kabuli*	Ca_Kabuli_Chr02	27134574	(A/G)
2775	CakSNP2775	*Kabuli*	Ca_Kabuli_Chr02	27134560	(G/T)
2776	CakSNP2776	*Kabuli*	Ca_Kabuli_Chr02	27182855	(T/C)
2777	CakSNP2777	*Kabuli*	Ca_Kabuli_Chr02	27317272	(A/T)
2778	CakSNP2778	*Kabuli*	Ca_Kabuli_Chr02	27374064	(A/T)
2779	CakSNP2779	*Kabuli*	Ca_Kabuli_Chr02	27374094	(C/A)
2780	CakSNP2780	*Kabuli*	Ca_Kabuli_Chr02	27377809	(T/G)
2781	CakSNP2781	*Kabuli*	Ca_Kabuli_Chr02	27623258	(A/G)
2782	CakSNP2782	*Kabuli*	Ca_Kabuli_Chr02	27703356	(G/A)
2783	CakSNP2783	*Kabuli*	Ca_Kabuli_Chr02	27724425	(G/T)
2784	CakSNP2784	*Kabuli*	Ca_Kabuli_Chr02	27757490	(T/C)
2785	CakSNP2785	*Kabuli*	Ca_Kabuli_Chr02	27757824	(A/G)
2786	CakSNP2786	*Kabuli*	Ca_Kabuli_Chr02	27757804	(A/G)
2787	CakSNP2787	*Kabuli*	Ca_Kabuli_Chr02	27757795	(T/G)
2788	CakSNP2788	*Kabuli*	Ca_Kabuli_Chr02	27758057	(C/T)
2789	CakSNP2789	*Kabuli*	Ca_Kabuli_Chr02	27758058	(A/G)
2790	CakSNP2790	*Kabuli*	Ca_Kabuli_Chr02	27758065	(C/G)
2791	CakSNP2791	*Kabuli*	Ca_Kabuli_Chr02	27758093	(C/T)
2792	CakSNP2792	*Kabuli*	Ca_Kabuli_Chr02	27766561	(T/A)
2793	CakSNP2793	*Kabuli*	Ca_Kabuli_Chr02	27766623	(T/C)
2794	CakSNP2794	*Kabuli*	Ca_Kabuli_Chr02	27766605	(A/C)
2795	CakSNP2795	*Kabuli*	Ca_Kabuli_Chr02	27766594	(C/A)
2796	CakSNP2796	*Kabuli*	Ca_Kabuli_Chr02	27807994	(A/G)
2797	CakSNP2797	*Kabuli*	Ca_Kabuli_Chr02	27808070	(G/A)
2798	CakSNP2798	*Kabuli*	Ca_Kabuli_Chr02	27824962	(G/A)
2799	CakSNP2799	*Kabuli*	Ca_Kabuli_Chr02	27831340	(A/C)
2800	CakSNP2800	*Kabuli*	Ca_Kabuli_Chr02	27831398	(A/G)
2801	CakSNP2801	*Kabuli*	Ca_Kabuli_Chr02	27835871	(G/A)
2802	CakSNP2802	*Kabuli*	Ca_Kabuli_Chr02	27835850	(G/A)
2803	CakSNP2803	*Kabuli*	Ca_Kabuli_Chr02	27835843	(C/A)
2804	CakSNP2804	*Kabuli*	Ca_Kabuli_Chr02	27835837	(C/T)
2805	CakSNP2805	*Kabuli*	Ca_Kabuli_Chr02	27835832	(C/T)
2806	CakSNP2806	*Kabuli*	Ca_Kabuli_Chr02	27847670	(A/C)
2807	CakSNP2807	*Kabuli*	Ca_Kabuli_Chr02	28017163	(A/G)
2808	CakSNP2808	*Kabuli*	Ca_Kabuli_Chr02	28018158	(T/C)
2809	CakSNP2809	*Kabuli*	Ca_Kabuli_Chr02	28060867	(T/G)
2810	CakSNP2810	*Kabuli*	Ca_Kabuli_Chr02	28060850	(A/G)
2811	CakSNP2811	*Kabuli*	Ca_Kabuli_Chr02	28060879	(C/T)

S.N.	SNP IDs	Cultivars	Chromosomes/scaffolds	Physical positions (bp)	SNPs
2812	CakSNP2812	*Kabuli*	Ca_Kabuli_Chr02	28060896	(T/C)
2813	CakSNP2813	*Kabuli*	Ca_Kabuli_Chr02	28060921	(C/T)
2814	CakSNP2814	*Kabuli*	Ca_Kabuli_Chr02	28060922	(A/G)
2815	CakSNP2815	*Kabuli*	Ca_Kabuli_Chr02	28060928	(C/T)
2816	CakSNP2816	*Kabuli*	Ca_Kabuli_Chr02	28060930	(T/G)
2817	CakSNP2817	*Kabuli*	Ca_Kabuli_Chr02	28060940	(C/T)
2818	CakSNP2818	*Kabuli*	Ca_Kabuli_Chr02	28061049	(G/T)
2819	CakSNP2819	*Kabuli*	Ca_Kabuli_Chr02	28076664	(C/T)
2820	CakSNP2820	*Kabuli*	Ca_Kabuli_Chr02	28076834	(C/T)
2821	CakSNP2821	*Kabuli*	Ca_Kabuli_Chr02	28076851	(G/A)
2822	CakSNP2822	*Kabuli*	Ca_Kabuli_Chr02	28084910	(C/T)
2823	CakSNP2823	*Kabuli*	Ca_Kabuli_Chr02	28084913	(C/A)
2824	CakSNP2824	*Kabuli*	Ca_Kabuli_Chr02	28084953	(C/T)
2825	CakSNP2825	*Kabuli*	Ca_Kabuli_Chr02	28229031	(G/A)
2826	CakSNP2826	*Kabuli*	Ca_Kabuli_Chr02	28229046	(G/A)
2827	CakSNP2827	*Kabuli*	Ca_Kabuli_Chr02	28324577	(T/A)
2828	CakSNP2828	*Kabuli*	Ca_Kabuli_Chr02	28352533	(C/A)
2829	CakSNP2829	*Kabuli*	Ca_Kabuli_Chr02	28407316	(T/C)
2830	CakSNP2830	*Kabuli*	Ca_Kabuli_Chr02	28407324	(C/T)
2831	CakSNP2831	*Kabuli*	Ca_Kabuli_Chr02	28534235	(T/C)
2832	CakSNP2832	*Kabuli*	Ca_Kabuli_Chr02	28699149	(C/T)
2833	CakSNP2833	*Kabuli*	Ca_Kabuli_Chr02	28699178	(T/C)
2834	CakSNP2834	*Kabuli*	Ca_Kabuli_Chr02	28699185	(C/A)
2835	CakSNP2835	*Kabuli*	Ca_Kabuli_Chr02	28699219	(T/G)
2836	CakSNP2836	*Kabuli*	Ca_Kabuli_Chr02	28699229	(C/T)
2837	CakSNP2837	*Kabuli*	Ca_Kabuli_Chr02	28699276	(T/C)
2838	CakSNP2838	*Kabuli*	Ca_Kabuli_Chr02	28699383	(A/T)
2839	CakSNP2839	*Kabuli*	Ca_Kabuli_Chr02	28699403	(C/T)
2840	CakSNP2840	*Kabuli*	Ca_Kabuli_Chr02	28699414	(C/T)
2841	CakSNP2841	*Kabuli*	Ca_Kabuli_Chr02	28699579	(A/C)
2842	CakSNP2842	*Kabuli*	Ca_Kabuli_Chr02	28701724	(A/G)
2843	CakSNP2843	*Kabuli*	Ca_Kabuli_Chr02	28741764	(T/C)
2844	CakSNP2844	*Kabuli*	Ca_Kabuli_Chr02	28931402	(T/C)
2845	CakSNP2845	*Kabuli*	Ca_Kabuli_Chr02	28931340	(T/C)
2846	CakSNP2846	*Kabuli*	Ca_Kabuli_Chr02	28944712	(T/C)
2847	CakSNP2847	*Kabuli*	Ca_Kabuli_Chr02	28944707	(A/C)
2848	CakSNP2848	*Kabuli*	Ca_Kabuli_Chr02	28949900	(C/T)
2849	CakSNP2849	*Kabuli*	Ca_Kabuli_Chr02	28949909	(G/A)

S.N.	SNP IDs	Cultivars	Chromosomes/scaffolds	Physical positions (bp)	SNPs
2850	CakSNP2850	*Kabuli*	Ca_Kabuli_Chr02	28949911	(A/C)
2851	CakSNP2851	*Kabuli*	Ca_Kabuli_Chr02	28949919	(A/T)
2852	CakSNP2852	*Kabuli*	Ca_Kabuli_Chr02	28949954	(T/A)
2853	CakSNP2853	*Kabuli*	Ca_Kabuli_Chr02	28949948	(A/G)
2854	CakSNP2854	*Kabuli*	Ca_Kabuli_Chr02	28970621	(A/G)
2855	CakSNP2855	*Kabuli*	Ca_Kabuli_Chr02	28970618	(A/G)
2856	CakSNP2856	*Kabuli*	Ca_Kabuli_Chr02	29103993	(T/C)
2857	CakSNP2857	*Kabuli*	Ca_Kabuli_Chr02	29103970	(C/T)
2858	CakSNP2858	*Kabuli*	Ca_Kabuli_Chr02	29122581	(G/A)
2859	CakSNP2859	*Kabuli*	Ca_Kabuli_Chr02	29122589	(T/G)
2860	CakSNP2860	*Kabuli*	Ca_Kabuli_Chr02	29592666	(G/A)
2861	CakSNP2861	*Kabuli*	Ca_Kabuli_Chr02	29592694	(T/C)
2862	CakSNP2862	*Kabuli*	Ca_Kabuli_Chr02	29601458	(C/T)
2863	CakSNP2863	*Kabuli*	Ca_Kabuli_Chr02	29601463	(C/T)
2864	CakSNP2864	*Kabuli*	Ca_Kabuli_Chr02	29614370	(C/T)
2865	CakSNP2865	*Kabuli*	Ca_Kabuli_Chr02	29614375	(C/T)
2866	CakSNP2866	*Kabuli*	Ca_Kabuli_Chr02	29693107	(A/G)
2867	CakSNP2867	*Kabuli*	Ca_Kabuli_Chr02	29948356	(G/A)
2868	CakSNP2868	*Kabuli*	Ca_Kabuli_Chr02	29960049	(G/A)
2869	CakSNP2869	*Kabuli*	Ca_Kabuli_Chr02	29960054	(G/A)
2870	CakSNP2870	*Kabuli*	Ca_Kabuli_Chr02	29960130	(C/T)
2871	CakSNP2871	*Kabuli*	Ca_Kabuli_Chr02	30042255	(A/C)
2872	CakSNP2872	*Kabuli*	Ca_Kabuli_Chr02	30042309	(T/G)
2873	CakSNP2873	*Kabuli*	Ca_Kabuli_Chr02	30042302	(A/T)
2874	CakSNP2874	*Kabuli*	Ca_Kabuli_Chr02	30042297	(C/T)
2875	CakSNP2875	*Kabuli*	Ca_Kabuli_Chr02	30042267	(C/T)
2876	CakSNP2876	*Kabuli*	Ca_Kabuli_Chr02	30045338	(C/G)
2877	CakSNP2877	*Kabuli*	Ca_Kabuli_Chr02	30091313	(G/A)
2878	CakSNP2878	*Kabuli*	Ca_Kabuli_Chr02	30091311	(C/T)
2879	CakSNP2879	*Kabuli*	Ca_Kabuli_Chr02	30091308	(A/T)
2880	CakSNP2880	*Kabuli*	Ca_Kabuli_Chr02	30091307	(C/T)
2881	CakSNP2881	*Kabuli*	Ca_Kabuli_Chr02	30091295	(T/A)
2882	CakSNP2882	*Kabuli*	Ca_Kabuli_Chr02	30091290	(T/C)
2883	CakSNP2883	*Kabuli*	Ca_Kabuli_Chr02	30091286	(G/A)
2884	CakSNP2884	*Kabuli*	Ca_Kabuli_Chr02	30091284	(A/C)
2885	CakSNP2885	*Kabuli*	Ca_Kabuli_Chr02	30091280	(C/G)
2886	CakSNP2886	*Kabuli*	Ca_Kabuli_Chr02	30133162	(G/A)
2887	CakSNP2887	*Kabuli*	Ca_Kabuli_Chr02	30133189	(G/A)

S.N.	SNP IDs	Cultivars	Chromosomes/scaffolds	Physical positions (bp)	SNPs
2888	CakSNP2888	*Kabuli*	Ca_Kabuli_Chr02	30133215	(G/T)
2889	CakSNP2889	*Kabuli*	Ca_Kabuli_Chr02	30133221	(A/T)
2890	CakSNP2890	*Kabuli*	Ca_Kabuli_Chr02	30335160	(T/C)
2891	CakSNP2891	*Kabuli*	Ca_Kabuli_Chr02	30335212	(T/A)
2892	CakSNP2892	*Kabuli*	Ca_Kabuli_Chr02	30335194	(A/G)
2893	CakSNP2893	*Kabuli*	Ca_Kabuli_Chr02	30363957	(C/A)
2894	CakSNP2894	*Kabuli*	Ca_Kabuli_Chr02	30364073	(T/A)
2895	CakSNP2895	*Kabuli*	Ca_Kabuli_Chr02	30364042	(A/G)
2896	CakSNP2896	*Kabuli*	Ca_Kabuli_Chr02	30364025	(T/C)
2897	CakSNP2897	*Kabuli*	Ca_Kabuli_Chr02	30370013	(C/A)
2898	CakSNP2898	*Kabuli*	Ca_Kabuli_Chr02	30370003	(A/C)
2899	CakSNP2899	*Kabuli*	Ca_Kabuli_Chr02	30370411	(T/C)
2900	CakSNP2900	*Kabuli*	Ca_Kabuli_Chr02	30467552	(C/T)
2901	CakSNP2901	*Kabuli*	Ca_Kabuli_Chr02	30467551	(A/T)
2902	CakSNP2902	*Kabuli*	Ca_Kabuli_Chr02	30508454	(T/C)
2903	CakSNP2903	*Kabuli*	Ca_Kabuli_Chr02	30530642	(A/T)
2904	CakSNP2904	*Kabuli*	Ca_Kabuli_Chr02	30537667	(A/T)
2905	CakSNP2905	*Kabuli*	Ca_Kabuli_Chr02	30537673	(A/G)
2906	CakSNP2906	*Kabuli*	Ca_Kabuli_Chr02	30537687	(T/C)
2907	CakSNP2907	*Kabuli*	Ca_Kabuli_Chr02	30537701	(T/A)
2908	CakSNP2908	*Kabuli*	Ca_Kabuli_Chr02	30537953	(T/C)
2909	CakSNP2909	*Kabuli*	Ca_Kabuli_Chr02	30614086	(C/T)
2910	CakSNP2910	*Kabuli*	Ca_Kabuli_Chr02	30686920	(C/T)
2911	CakSNP2911	*Kabuli*	Ca_Kabuli_Chr02	30702121	(T/C)
2912	CakSNP2912	*Kabuli*	Ca_Kabuli_Chr02	30702090	(G/A)
2913	CakSNP2913	*Kabuli*	Ca_Kabuli_Chr02	30867547	(G/A)
2914	CakSNP2914	*Kabuli*	Ca_Kabuli_Chr02	30880148	(A/G)
2915	CakSNP2915	*Kabuli*	Ca_Kabuli_Chr02	30880243	(G/C)
2916	CakSNP2916	*Kabuli*	Ca_Kabuli_Chr02	30880246	(G/A)
2917	CakSNP2917	*Kabuli*	Ca_Kabuli_Chr02	30880303	(T/A)
2918	CakSNP2918	*Kabuli*	Ca_Kabuli_Chr02	30880302	(T/C)
2919	CakSNP2919	*Kabuli*	Ca_Kabuli_Chr02	30880282	(A/G)
2920	CakSNP2920	*Kabuli*	Ca_Kabuli_Chr02	30921092	(C/A)
2921	CakSNP2921	*Kabuli*	Ca_Kabuli_Chr02	30921758	(C/T)
2922	CakSNP2922	*Kabuli*	Ca_Kabuli_Chr02	30921888	(T/C)
2923	CakSNP2923	*Kabuli*	Ca_Kabuli_Chr02	30921882	(C/G)
2924	CakSNP2924	*Kabuli*	Ca_Kabuli_Chr02	30921917	(C/A)
2925	CakSNP2925	*Kabuli*	Ca_Kabuli_Chr02	30921923	(A/G)

S.N.	SNP IDs	Cultivars	Chromosomes/scaffolds	Physical positions (bp)	SNPs
2926	CakSNP2926	*Kabuli*	Ca_Kabuli_Chr02	30954317	(G/A)
2927	CakSNP2927	*Kabuli*	Ca_Kabuli_Chr02	30954305	(T/G)
2928	CakSNP2928	*Kabuli*	Ca_Kabuli_Chr02	30977572	(A/G)
2929	CakSNP2929	*Kabuli*	Ca_Kabuli_Chr02	30977528	(C/T)
2930	CakSNP2930	*Kabuli*	Ca_Kabuli_Chr02	30977522	(A/T)
2931	CakSNP2931	*Kabuli*	Ca_Kabuli_Chr02	30977508	(A/T)
2932	CakSNP2932	*Kabuli*	Ca_Kabuli_Chr02	30997769	(T/A)
2933	CakSNP2933	*Kabuli*	Ca_Kabuli_Chr02	30997780	(A/C)
2934	CakSNP2934	*Kabuli*	Ca_Kabuli_Chr02	30997784	(T/A)
2935	CakSNP2935	*Kabuli*	Ca_Kabuli_Chr02	31005847	(T/G)
2936	CakSNP2936	*Kabuli*	Ca_Kabuli_Chr02	31005845	(C/T)
2937	CakSNP2937	*Kabuli*	Ca_Kabuli_Chr02	31014295	(A/T)
2938	CakSNP2938	*Kabuli*	Ca_Kabuli_Chr02	31055083	(C/T)
2939	CakSNP2939	*Kabuli*	Ca_Kabuli_Chr02	31055007	(G/A)
2940	CakSNP2940	*Kabuli*	Ca_Kabuli_Chr02	31088681	(G/T)
2941	CakSNP2941	*Kabuli*	Ca_Kabuli_Chr02	31088676	(G/C)
2942	CakSNP2942	*Kabuli*	Ca_Kabuli_Chr02	31088670	(C/T)
2943	CakSNP2943	*Kabuli*	Ca_Kabuli_Chr02	31088648	(A/G)
2944	CakSNP2944	*Kabuli*	Ca_Kabuli_Chr02	31088645	(A/C)
2945	CakSNP2945	*Kabuli*	Ca_Kabuli_Chr02	31088778	(A/T)
2946	CakSNP2946	*Kabuli*	Ca_Kabuli_Chr02	31088800	(T/C)
2947	CakSNP2947	*Kabuli*	Ca_Kabuli_Chr02	31088882	(A/G)
2948	CakSNP2948	*Kabuli*	Ca_Kabuli_Chr02	31109311	(G/T)
2949	CakSNP2949	*Kabuli*	Ca_Kabuli_Chr02	31109358	(C/T)
2950	CakSNP2950	*Kabuli*	Ca_Kabuli_Chr02	31109481	(G/A)
2951	CakSNP2951	*Kabuli*	Ca_Kabuli_Chr02	31109441	(C/G)
2952	CakSNP2952	*Kabuli*	Ca_Kabuli_Chr02	31109435	(G/A)
2953	CakSNP2953	*Kabuli*	Ca_Kabuli_Chr02	31109427	(C/T)
2954	CakSNP2954	*Kabuli*	Ca_Kabuli_Chr02	31141019	(G/A)
2955	CakSNP2955	*Kabuli*	Ca_Kabuli_Chr02	31167603	(G/A)
2956	CakSNP2956	*Kabuli*	Ca_Kabuli_Chr02	31167594	(A/G)
2957	CakSNP2957	*Kabuli*	Ca_Kabuli_Chr02	31188355	(A/G)
2958	CakSNP2958	*Kabuli*	Ca_Kabuli_Chr02	31201872	(T/C)
2959	CakSNP2959	*Kabuli*	Ca_Kabuli_Chr02	31201859	(T/A)
2960	CakSNP2960	*Kabuli*	Ca_Kabuli_Chr02	31201795	(A/G)
2961	CakSNP2961	*Kabuli*	Ca_Kabuli_Chr02	31203154	(G/A)
2962	CakSNP2962	*Kabuli*	Ca_Kabuli_Chr02	31205531	(C/T)
2963	CakSNP2963	*Kabuli*	Ca_Kabuli_Chr02	31427861	(A/G)

S.N.	SNP IDs	Cultivars	Chromosomes/scaffolds	Physical positions (bp)	SNPs
2964	CakSNP2964	*Kabuli*	Ca_Kabuli_Chr02	31427902	(G/C)
2965	CakSNP2965	*Kabuli*	Ca_Kabuli_Chr02	31427915	(A/G)
2966	CakSNP2966	*Kabuli*	Ca_Kabuli_Chr02	31428024	(T/G)
2967	CakSNP2967	*Kabuli*	Ca_Kabuli_Chr02	31428132	(G/A)
2968	CakSNP2968	*Kabuli*	Ca_Kabuli_Chr02	31428405	(T/C)
2969	CakSNP2969	*Kabuli*	Ca_Kabuli_Chr02	31428429	(T/G)
2970	CakSNP2970	*Kabuli*	Ca_Kabuli_Chr02	31534265	(A/C)
2971	CakSNP2971	*Kabuli*	Ca_Kabuli_Chr02	31534283	(A/G)
2972	CakSNP2972	*Kabuli*	Ca_Kabuli_Chr02	31534455	(T/C)
2973	CakSNP2973	*Kabuli*	Ca_Kabuli_Chr02	31686714	(C/T)
2974	CakSNP2974	*Kabuli*	Ca_Kabuli_Chr02	31686716	(C/T)
2975	CakSNP2975	*Kabuli*	Ca_Kabuli_Chr02	31686742	(A/C)
2976	CakSNP2976	*Kabuli*	Ca_Kabuli_Chr02	31686751	(A/T)
2977	CakSNP2977	*Kabuli*	Ca_Kabuli_Chr02	31686758	(C/A)
2978	CakSNP2978	*Kabuli*	Ca_Kabuli_Chr02	31686761	(A/C)
2979	CakSNP2979	*Kabuli*	Ca_Kabuli_Chr02	31686767	(C/T)
2980	CakSNP2980	*Kabuli*	Ca_Kabuli_Chr02	31686779	(G/A)
2981	CakSNP2981	*Kabuli*	Ca_Kabuli_Chr02	31686820	(T/C)
2982	CakSNP2982	*Kabuli*	Ca_Kabuli_Chr02	31756777	(G/T)
2983	CakSNP2983	*Kabuli*	Ca_Kabuli_Chr02	31847033	(A/C)
2984	CakSNP2984	*Kabuli*	Ca_Kabuli_Chr02	31881918	(G/T)
2985	CakSNP2985	*Kabuli*	Ca_Kabuli_Chr02	31885934	(A/G)
2986	CakSNP2986	*Kabuli*	Ca_Kabuli_Chr02	31908694	(G/A)
2987	CakSNP2987	*Kabuli*	Ca_Kabuli_Chr02	31908742	(G/A)
2988	CakSNP2988	*Kabuli*	Ca_Kabuli_Chr02	31908803	(C/T)
2989	CakSNP2989	*Kabuli*	Ca_Kabuli_Chr02	31908787	(G/A)
2990	CakSNP2990	*Kabuli*	Ca_Kabuli_Chr02	31908845	(T/A)
2991	CakSNP2991	*Kabuli*	Ca_Kabuli_Chr02	31908857	(C/T)
2992	CakSNP2992	*Kabuli*	Ca_Kabuli_Chr02	31941463	(T/C)
2993	CakSNP2993	*Kabuli*	Ca_Kabuli_Chr02	31941554	(A/T)
2994	CakSNP2994	*Kabuli*	Ca_Kabuli_Chr02	31975269	(C/T)
2995	CakSNP2995	*Kabuli*	Ca_Kabuli_Chr02	32003536	(G/T)
2996	CakSNP2996	*Kabuli*	Ca_Kabuli_Chr02	32017361	(G/A)
2997	CakSNP2997	*Kabuli*	Ca_Kabuli_Chr02	32049523	(C/A)
2998	CakSNP2998	*Kabuli*	Ca_Kabuli_Chr02	32049525	(A/C)
2999	CakSNP2999	*Kabuli*	Ca_Kabuli_Chr02	32058591	(T/C)
3000	CakSNP3000	*Kabuli*	Ca_Kabuli_Chr02	32061053	(C/T)
3001	CakSNP3001	*Kabuli*	Ca_Kabuli_Chr02	32061054	(T/C)

S.N.	SNP IDs	Cultivars	Chromosomes/scaffolds	Physical positions (bp)	SNPs
3002	CakSNP3002	*Kabuli*	Ca_Kabuli_Chr02	32062010	(A/C)
3003	CakSNP3003	*Kabuli*	Ca_Kabuli_Chr02	32071107	(C/G)
3004	CakSNP3004	*Kabuli*	Ca_Kabuli_Chr02	32080002	(T/C)
3005	CakSNP3005	*Kabuli*	Ca_Kabuli_Chr02	32080007	(T/C)
3006	CakSNP3006	*Kabuli*	Ca_Kabuli_Chr02	32080010	(G/C)
3007	CakSNP3007	*Kabuli*	Ca_Kabuli_Chr02	32080016	(A/T)
3008	CakSNP3008	*Kabuli*	Ca_Kabuli_Chr02	32080022	(C/A)
3009	CakSNP3009	*Kabuli*	Ca_Kabuli_Chr02	32080037	(A/G)
3010	CakSNP3010	*Kabuli*	Ca_Kabuli_Chr02	32096192	(A/C)
3011	CakSNP3011	*Kabuli*	Ca_Kabuli_Chr02	32096186	(T/A)
3012	CakSNP3012	*Kabuli*	Ca_Kabuli_Chr02	32096180	(C/G)
3013	CakSNP3013	*Kabuli*	Ca_Kabuli_Chr02	32096177	(C/T)
3014	CakSNP3014	*Kabuli*	Ca_Kabuli_Chr02	32096172	(C/T)
3015	CakSNP3015	*Kabuli*	Ca_Kabuli_Chr02	32099757	(C/T)
3016	CakSNP3016	*Kabuli*	Ca_Kabuli_Chr02	32099907	(T/C)
3017	CakSNP3017	*Kabuli*	Ca_Kabuli_Chr02	32109805	(G/A)
3018	CakSNP3018	*Kabuli*	Ca_Kabuli_Chr02	32109811	(G/A)
3019	CakSNP3019	*Kabuli*	Ca_Kabuli_Chr02	32109813	(C/T)
3020	CakSNP3020	*Kabuli*	Ca_Kabuli_Chr02	32109821	(G/A)
3021	CakSNP3021	*Kabuli*	Ca_Kabuli_Chr02	32109827	(T/C)
3022	CakSNP3022	*Kabuli*	Ca_Kabuli_Chr02	32109859	(A/C)
3023	CakSNP3023	*Kabuli*	Ca_Kabuli_Chr02	32109904	(T/A)
3024	CakSNP3024	*Kabuli*	Ca_Kabuli_Chr02	32109895	(A/G)
3025	CakSNP3025	*Kabuli*	Ca_Kabuli_Chr02	32109892	(T/A)
3026	CakSNP3026	*Kabuli*	Ca_Kabuli_Chr02	32119094	(A/T)
3027	CakSNP3027	*Kabuli*	Ca_Kabuli_Chr02	32119268	(T/A)
3028	CakSNP3028	*Kabuli*	Ca_Kabuli_Chr02	32119274	(T/C)
3029	CakSNP3029	*Kabuli*	Ca_Kabuli_Chr02	32119331	(A/T)
3030	CakSNP3030	*Kabuli*	Ca_Kabuli_Chr02	32129272	(T/C)
3031	CakSNP3031	*Kabuli*	Ca_Kabuli_Chr02	32129395	(C/G)
3032	CakSNP3032	*Kabuli*	Ca_Kabuli_Chr02	32140939	(A/T)
3033	CakSNP3033	*Kabuli*	Ca_Kabuli_Chr02	32140948	(A/C)
3034	CakSNP3034	*Kabuli*	Ca_Kabuli_Chr02	32140987	(A/G)
3035	CakSNP3035	*Kabuli*	Ca_Kabuli_Chr02	32141047	(A/G)
3036	CakSNP3036	*Kabuli*	Ca_Kabuli_Chr02	32141038	(T/C)
3037	CakSNP3037	*Kabuli*	Ca_Kabuli_Chr02	32141019	(G/A)
3038	CakSNP3038	*Kabuli*	Ca_Kabuli_Chr02	32141013	(G/C)
3039	CakSNP3039	*Kabuli*	Ca_Kabuli_Chr02	32141002	(A/T)

S.N.	SNP IDs	Cultivars	Chromosomes/scaffolds	Physical positions (bp)	SNPs
3040	CakSNP3040	*Kabuli*	Ca_Kabuli_Chr02	32140998	(G/T)
3041	CakSNP3041	*Kabuli*	Ca_Kabuli_Chr02	32140990	(G/A)
3042	CakSNP3042	*Kabuli*	Ca_Kabuli_Chr02	32203032	(A/G)
3043	CakSNP3043	*Kabuli*	Ca_Kabuli_Chr02	32203036	(A/T)
3044	CakSNP3044	*Kabuli*	Ca_Kabuli_Chr02	32242818	(T/G)
3045	CakSNP3045	*Kabuli*	Ca_Kabuli_Chr02	32348054	(T/A)
3046	CakSNP3046	*Kabuli*	Ca_Kabuli_Chr02	32348087	(A/C)
3047	CakSNP3047	*Kabuli*	Ca_Kabuli_Chr02	32348119	(G/T)
3048	CakSNP3048	*Kabuli*	Ca_Kabuli_Chr02	32348793	(G/A)
3049	CakSNP3049	*Kabuli*	Ca_Kabuli_Chr02	32348870	(T/C)
3050	CakSNP3050	*Kabuli*	Ca_Kabuli_Chr02	32411994	(G/T)
3051	CakSNP3051	*Kabuli*	Ca_Kabuli_Chr02	32412000	(G/T)
3052	CakSNP3052	*Kabuli*	Ca_Kabuli_Chr02	32481969	(C/T)
3053	CakSNP3053	*Kabuli*	Ca_Kabuli_Chr02	32481983	(G/T)
3054	CakSNP3054	*Kabuli*	Ca_Kabuli_Chr02	32484223	(C/T)
3055	CakSNP3055	*Kabuli*	Ca_Kabuli_Chr02	32545320	(A/G)
3056	CakSNP3056	*Kabuli*	Ca_Kabuli_Chr02	32545329	(T/C)
3057	CakSNP3057	*Kabuli*	Ca_Kabuli_Chr02	32545351	(G/A)
3058	CakSNP3058	*Kabuli*	Ca_Kabuli_Chr02	32556992	(T/A)
3059	CakSNP3059	*Kabuli*	Ca_Kabuli_Chr02	32564427	(T/C)
3060	CakSNP3060	*Kabuli*	Ca_Kabuli_Chr02	32564480	(G/A)
3061	CakSNP3061	*Kabuli*	Ca_Kabuli_Chr02	32564770	(G/T)
3062	CakSNP3062	*Kabuli*	Ca_Kabuli_Chr02	32564796	(A/T)
3063	CakSNP3063	*Kabuli*	Ca_Kabuli_Chr02	32570394	(G/A)
3064	CakSNP3064	*Kabuli*	Ca_Kabuli_Chr02	32570479	(T/G)
3065	CakSNP3065	*Kabuli*	Ca_Kabuli_Chr02	32570429	(G/A)
3066	CakSNP3066	*Kabuli*	Ca_Kabuli_Chr02	32570504	(G/C)
3067	CakSNP3067	*Kabuli*	Ca_Kabuli_Chr02	32577274	(T/A)
3068	CakSNP3068	*Kabuli*	Ca_Kabuli_Chr02	32577593	(A/G)
3069	CakSNP3069	*Kabuli*	Ca_Kabuli_Chr02	32579849	(T/C)
3070	CakSNP3070	*Kabuli*	Ca_Kabuli_Chr02	32579900	(C/G)
3071	CakSNP3071	*Kabuli*	Ca_Kabuli_Chr02	32608900	(A/T)
3072	CakSNP3072	*Kabuli*	Ca_Kabuli_Chr02	32611624	(C/T)
3073	CakSNP3073	*Kabuli*	Ca_Kabuli_Chr02	32611609	(A/G)
3074	CakSNP3074	*Kabuli*	Ca_Kabuli_Chr02	32611601	(G/T)
3075	CakSNP3075	*Kabuli*	Ca_Kabuli_Chr02	32627727	(C/G)
3076	CakSNP3076	*Kabuli*	Ca_Kabuli_Chr02	32629763	(A/G)
3077	CakSNP3077	*Kabuli*	Ca_Kabuli_Chr02	32670054	(C/T)

S.N.	SNP IDs	Cultivars	Chromosomes/scaffolds	Physical positions (bp)	SNPs
3078	CakSNP3078	*Kabuli*	Ca_Kabuli_Chr02	32670099	(C/T)
3079	CakSNP3079	*Kabuli*	Ca_Kabuli_Chr02	32670181	(T/G)
3080	CakSNP3080	*Kabuli*	Ca_Kabuli_Chr02	32671354	(T/A)
3081	CakSNP3081	*Kabuli*	Ca_Kabuli_Chr02	32781880	(A/G)
3082	CakSNP3082	*Kabuli*	Ca_Kabuli_Chr02	32844754	(T/G)
3083	CakSNP3083	*Kabuli*	Ca_Kabuli_Chr02	32844842	(C/G)
3084	CakSNP3084	*Kabuli*	Ca_Kabuli_Chr02	32867125	(A/G)
3085	CakSNP3085	*Kabuli*	Ca_Kabuli_Chr02	32934646	(C/T)
3086	CakSNP3086	*Kabuli*	Ca_Kabuli_Chr02	32934633	(G/C)
3087	CakSNP3087	*Kabuli*	Ca_Kabuli_Chr02	32936855	(A/G)
3088	CakSNP3088	*Kabuli*	Ca_Kabuli_Chr02	32936908	(G/A)
3089	CakSNP3089	*Kabuli*	Ca_Kabuli_Chr02	32957515	(A/G)
3090	CakSNP3090	*Kabuli*	Ca_Kabuli_Chr02	32957523	(T/C)
3091	CakSNP3091	*Kabuli*	Ca_Kabuli_Chr02	32957538	(A/C)
3092	CakSNP3092	*Kabuli*	Ca_Kabuli_Chr02	32957572	(A/C)
3093	CakSNP3093	*Kabuli*	Ca_Kabuli_Chr02	32957958	(A/C)
3094	CakSNP3094	*Kabuli*	Ca_Kabuli_Chr02	32958034	(C/G)
3095	CakSNP3095	*Kabuli*	Ca_Kabuli_Chr02	32989134	(G/T)
3096	CakSNP3096	*Kabuli*	Ca_Kabuli_Chr02	32989205	(G/T)
3097	CakSNP3097	*Kabuli*	Ca_Kabuli_Chr02	33083903	(T/A)
3098	CakSNP3098	*Kabuli*	Ca_Kabuli_Chr02	33084095	(T/G)
3099	CakSNP3099	*Kabuli*	Ca_Kabuli_Chr02	33084132	(T/A)
3100	CakSNP3100	*Kabuli*	Ca_Kabuli_Chr02	33103318	(G/A)
3101	CakSNP3101	*Kabuli*	Ca_Kabuli_Chr02	33103466	(G/A)
3102	CakSNP3102	*Kabuli*	Ca_Kabuli_Chr02	33103750	(A/G)
3103	CakSNP3103	*Kabuli*	Ca_Kabuli_Chr02	33103766	(G/A)
3104	CakSNP3104	*Kabuli*	Ca_Kabuli_Chr02	33172936	(C/T)
3105	CakSNP3105	*Kabuli*	Ca_Kabuli_Chr02	33172971	(T/G)
3106	CakSNP3106	*Kabuli*	Ca_Kabuli_Chr02	33178391	(G/A)
3107	CakSNP3107	*Kabuli*	Ca_Kabuli_Chr02	33178407	(C/T)
3108	CakSNP3108	*Kabuli*	Ca_Kabuli_Chr02	33178419	(C/A)
3109	CakSNP3109	*Kabuli*	Ca_Kabuli_Chr02	33178420	(A/C)
3110	CakSNP3110	*Kabuli*	Ca_Kabuli_Chr02	33178506	(T/C)
3111	CakSNP3111	*Kabuli*	Ca_Kabuli_Chr02	33184262	(T/C)
3112	CakSNP3112	*Kabuli*	Ca_Kabuli_Chr02	33194198	(A/G)
3113	CakSNP3113	*Kabuli*	Ca_Kabuli_Chr02	33204253	(C/T)
3114	CakSNP3114	*Kabuli*	Ca_Kabuli_Chr02	33326392	(T/G)
3115	CakSNP3115	*Kabuli*	Ca_Kabuli_Chr02	33326398	(C/T)

S.N.	SNP IDs	Cultivars	Chromosomes/scaffolds	Physical positions (bp)	SNPs
3116	CakSNP3116	*Kabuli*	Ca_Kabuli_Chr02	33326444	(G/A)
3117	CakSNP3117	*Kabuli*	Ca_Kabuli_Chr02	33326522	(G/A)
3118	CakSNP3118	*Kabuli*	Ca_Kabuli_Chr02	33326520	(T/C)
3119	CakSNP3119	*Kabuli*	Ca_Kabuli_Chr02	33326516	(G/A)
3120	CakSNP3120	*Kabuli*	Ca_Kabuli_Chr02	33326510	(C/G)
3121	CakSNP3121	*Kabuli*	Ca_Kabuli_Chr02	33393209	(T/C)
3122	CakSNP3122	*Kabuli*	Ca_Kabuli_Chr02	33398561	(C/T)
3123	CakSNP3123	*Kabuli*	Ca_Kabuli_Chr02	33398550	(G/A)
3124	CakSNP3124	*Kabuli*	Ca_Kabuli_Chr02	33398549	(T/C)
3125	CakSNP3125	*Kabuli*	Ca_Kabuli_Chr02	33398527	(T/G)
3126	CakSNP3126	*Kabuli*	Ca_Kabuli_Chr02	33399010	(C/T)
3127	CakSNP3127	*Kabuli*	Ca_Kabuli_Chr02	33399019	(T/C)
3128	CakSNP3128	*Kabuli*	Ca_Kabuli_Chr02	33399045	(A/C)
3129	CakSNP3129	*Kabuli*	Ca_Kabuli_Chr02	33399098	(G/A)
3130	CakSNP3130	*Kabuli*	Ca_Kabuli_Chr02	33399063	(C/T)
3131	CakSNP3131	*Kabuli*	Ca_Kabuli_Chr02	33399405	(C/T)
3132	CakSNP3132	*Kabuli*	Ca_Kabuli_Chr02	33399404	(C/A)
3133	CakSNP3133	*Kabuli*	Ca_Kabuli_Chr02	33399397	(C/T)
3134	CakSNP3134	*Kabuli*	Ca_Kabuli_Chr02	33399393	(C/A)
3135	CakSNP3135	*Kabuli*	Ca_Kabuli_Chr02	33399381	(C/T)
3136	CakSNP3136	*Kabuli*	Ca_Kabuli_Chr02	33399342	(A/C)
3137	CakSNP3137	*Kabuli*	Ca_Kabuli_Chr02	33399465	(C/G)
3138	CakSNP3138	*Kabuli*	Ca_Kabuli_Chr02	33399608	(C/A)
3139	CakSNP3139	*Kabuli*	Ca_Kabuli_Chr02	33401608	(A/G)
3140	CakSNP3140	*Kabuli*	Ca_Kabuli_Chr02	33490058	(C/A)
3141	CakSNP3141	*Kabuli*	Ca_Kabuli_Chr02	33489989	(T/C)
3142	CakSNP3142	*Kabuli*	Ca_Kabuli_Chr02	33575286	(A/C)
3143	CakSNP3143	*Kabuli*	Ca_Kabuli_Chr02	33582948	(G/A)
3144	CakSNP3144	*Kabuli*	Ca_Kabuli_Chr02	33582900	(G/A)
3145	CakSNP3145	*Kabuli*	Ca_Kabuli_Chr02	33650447	(A/T)
3146	CakSNP3146	*Kabuli*	Ca_Kabuli_Chr02	33651426	(G/A)
3147	CakSNP3147	*Kabuli*	Ca_Kabuli_Chr02	33651408	(A/G)
3148	CakSNP3148	*Kabuli*	Ca_Kabuli_Chr02	33661177	(C/T)
3149	CakSNP3149	*Kabuli*	Ca_Kabuli_Chr02	33661316	(T/C)
3150	CakSNP3150	*Kabuli*	Ca_Kabuli_Chr02	33686794	(G/A)
3151	CakSNP3151	*Kabuli*	Ca_Kabuli_Chr02	33692368	(C/A)
3152	CakSNP3152	*Kabuli*	Ca_Kabuli_Chr02	33713018	(G/A)
3153	CakSNP3153	*Kabuli*	Ca_Kabuli_Chr02	33721515	(T/A)

S.N.	SNP IDs	Cultivars	Chromosomes/scaffolds	Physical positions (bp)	SNPs
3154	CakSNP3154	*Kabuli*	Ca_Kabuli_Chr02	33733893	(C/T)
3155	CakSNP3155	*Kabuli*	Ca_Kabuli_Chr02	33745281	(A/G)
3156	CakSNP3156	*Kabuli*	Ca_Kabuli_Chr02	33764634	(G/A)
3157	CakSNP3157	*Kabuli*	Ca_Kabuli_Chr02	33783871	(C/T)
3158	CakSNP3158	*Kabuli*	Ca_Kabuli_Chr02	33785203	(G/A)
3159	CakSNP3159	*Kabuli*	Ca_Kabuli_Chr02	33786243	(G/A)
3160	CakSNP3160	*Kabuli*	Ca_Kabuli_Chr02	33788913	(A/C)
3161	CakSNP3161	*Kabuli*	Ca_Kabuli_Chr02	33798717	(T/C)
3162	CakSNP3162	*Kabuli*	Ca_Kabuli_Chr02	33798748	(T/C)
3163	CakSNP3163	*Kabuli*	Ca_Kabuli_Chr02	34152352	(T/C)
3164	CakSNP3164	*Kabuli*	Ca_Kabuli_Chr02	34152384	(T/C)
3165	CakSNP3165	*Kabuli*	Ca_Kabuli_Chr02	34152476	(C/T)
3166	CakSNP3166	*Kabuli*	Ca_Kabuli_Chr02	34155090	(A/G)
3167	CakSNP3167	*Kabuli*	Ca_Kabuli_Chr02	34155179	(A/G)
3168	CakSNP3168	*Kabuli*	Ca_Kabuli_Chr02	34178534	(G/A)
3169	CakSNP3169	*Kabuli*	Ca_Kabuli_Chr02	34203162	(T/C)
3170	CakSNP3170	*Kabuli*	Ca_Kabuli_Chr02	34203444	(T/C)
3171	CakSNP3171	*Kabuli*	Ca_Kabuli_Chr02	34222585	(G/A)
3172	CakSNP3172	*Kabuli*	Ca_Kabuli_Chr02	34249987	(C/T)
3173	CakSNP3173	*Kabuli*	Ca_Kabuli_Chr02	34249953	(G/T)
3174	CakSNP3174	*Kabuli*	Ca_Kabuli_Chr02	34250042	(C/T)
3175	CakSNP3175	*Kabuli*	Ca_Kabuli_Chr02	34250266	(T/C)
3176	CakSNP3176	*Kabuli*	Ca_Kabuli_Chr02	34266109	(A/G)
3177	CakSNP3177	*Kabuli*	Ca_Kabuli_Chr02	34288116	(G/C)
3178	CakSNP3178	*Kabuli*	Ca_Kabuli_Chr02	34299096	(G/A)
3179	CakSNP3179	*Kabuli*	Ca_Kabuli_Chr02	34299075	(C/T)
3180	CakSNP3180	*Kabuli*	Ca_Kabuli_Chr02	34299074	(A/C)
3181	CakSNP3181	*Kabuli*	Ca_Kabuli_Chr02	34299063	(G/A)
3182	CakSNP3182	*Kabuli*	Ca_Kabuli_Chr02	34299079	(A/C)
3183	CakSNP3183	*Kabuli*	Ca_Kabuli_Chr02	34299846	(C/T)
3184	CakSNP3184	*Kabuli*	Ca_Kabuli_Chr02	34300059	(A/G)
3185	CakSNP3185	*Kabuli*	Ca_Kabuli_Chr02	34300057	(A/G)
3186	CakSNP3186	*Kabuli*	Ca_Kabuli_Chr02	34343478	(C/G)
3187	CakSNP3187	*Kabuli*	Ca_Kabuli_Chr02	34343705	(G/A)
3188	CakSNP3188	*Kabuli*	Ca_Kabuli_Chr02	34346849	(C/T)
3189	CakSNP3189	*Kabuli*	Ca_Kabuli_Chr02	34346842	(T/C)
3190	CakSNP3190	*Kabuli*	Ca_Kabuli_Chr02	34372503	(T/G)
3191	CakSNP3191	*Kabuli*	Ca_Kabuli_Chr02	34428978	(C/A)

S.N.	SNP IDs	Cultivars	Chromosomes/scaffolds	Physical positions (bp)	SNPs
3192	CakSNP3192	*Kabuli*	Ca_Kabuli_Chr02	34428979	(G/C)
3193	CakSNP3193	*Kabuli*	Ca_Kabuli_Chr02	34429029	(G/T)
3194	CakSNP3194	*Kabuli*	Ca_Kabuli_Chr02	34429030	(A/T)
3195	CakSNP3195	*Kabuli*	Ca_Kabuli_Chr02	34551849	(A/C)
3196	CakSNP3196	*Kabuli*	Ca_Kabuli_Chr02	34554164	(A/C)
3197	CakSNP3197	*Kabuli*	Ca_Kabuli_Chr02	34601444	(C/A)
3198	CakSNP3198	*Kabuli*	Ca_Kabuli_Chr02	34634128	(C/T)
3199	CakSNP3199	*Kabuli*	Ca_Kabuli_Chr02	34638960	(A/C)
3200	CakSNP3200	*Kabuli*	Ca_Kabuli_Chr02	34638955	(T/A)
3201	CakSNP3201	*Kabuli*	Ca_Kabuli_Chr02	34734329	(G/A)
3202	CakSNP3202	*Kabuli*	Ca_Kabuli_Chr02	34734379	(G/A)
3203	CakSNP3203	*Kabuli*	Ca_Kabuli_Chr02	34734439	(G/A)
3204	CakSNP3204	*Kabuli*	Ca_Kabuli_Chr02	34734472	(A/G)
3205	CakSNP3205	*Kabuli*	Ca_Kabuli_Chr02	34736649	(T/C)
3206	CakSNP3206	*Kabuli*	Ca_Kabuli_Chr02	34736658	(A/C)
3207	CakSNP3207	*Kabuli*	Ca_Kabuli_Chr02	34736753	(C/T)
3208	CakSNP3208	*Kabuli*	Ca_Kabuli_Chr02	34754363	(T/C)
3209	CakSNP3209	*Kabuli*	Ca_Kabuli_Chr02	34754507	(C/T)
3210	CakSNP3210	*Kabuli*	Ca_Kabuli_Chr02	34820075	(C/T)
3211	CakSNP3211	*Kabuli*	Ca_Kabuli_Chr02	34830995	(A/G)
3212	CakSNP3212	*Kabuli*	Ca_Kabuli_Chr02	34847681	(C/A)
3213	CakSNP3213	*Kabuli*	Ca_Kabuli_Chr02	34848053	(G/A)
3214	CakSNP3214	*Kabuli*	Ca_Kabuli_Chr02	34848480	(T/C)
3215	CakSNP3215	*Kabuli*	Ca_Kabuli_Chr02	34862129	(G/T)
3216	CakSNP3216	*Kabuli*	Ca_Kabuli_Chr02	34862135	(G/A)
3217	CakSNP3217	*Kabuli*	Ca_Kabuli_Chr02	34862139	(A/G)
3218	CakSNP3218	*Kabuli*	Ca_Kabuli_Chr02	34862150	(C/T)
3219	CakSNP3219	*Kabuli*	Ca_Kabuli_Chr02	34862276	(C/G)
3220	CakSNP3220	*Kabuli*	Ca_Kabuli_Chr02	34888414	(T/C)
3221	CakSNP3221	*Kabuli*	Ca_Kabuli_Chr02	35013088	(G/C)
3222	CakSNP3222	*Kabuli*	Ca_Kabuli_Chr02	35013084	(T/C)
3223	CakSNP3223	*Kabuli*	Ca_Kabuli_Chr02	35052930	(C/A)
3224	CakSNP3224	*Kabuli*	Ca_Kabuli_Chr02	35054244	(C/A)
3225	CakSNP3225	*Kabuli*	Ca_Kabuli_Chr02	35059446	(A/T)
3226	CakSNP3226	*Kabuli*	Ca_Kabuli_Chr02	35059434	(T/C)
3227	CakSNP3227	*Kabuli*	Ca_Kabuli_Chr02	35059440	(A/C)
3228	CakSNP3228	*Kabuli*	Ca_Kabuli_Chr02	35059451	(C/A)
3229	CakSNP3229	*Kabuli*	Ca_Kabuli_Chr02	35060171	(A/G)

S.N.	SNP IDs	Cultivars	Chromosomes/scaffolds	Physical positions (bp)	SNPs
3230	CakSNP3230	*Kabuli*	Ca_Kabuli_Chr02	35060178	(A/T)
3231	CakSNP3231	*Kabuli*	Ca_Kabuli_Chr02	35060228	(T/C)
3232	CakSNP3232	*Kabuli*	Ca_Kabuli_Chr02	35060273	(G/A)
3233	CakSNP3233	*Kabuli*	Ca_Kabuli_Chr02	35060264	(A/T)
3234	CakSNP3234	*Kabuli*	Ca_Kabuli_Chr02	35066138	(G/A)
3235	CakSNP3235	*Kabuli*	Ca_Kabuli_Chr02	35095010	(A/G)
3236	CakSNP3236	*Kabuli*	Ca_Kabuli_Chr02	35096648	(A/T)
3237	CakSNP3237	*Kabuli*	Ca_Kabuli_Chr02	35096649	(A/T)
3238	CakSNP3238	*Kabuli*	Ca_Kabuli_Chr02	35120761	(G/A)
3239	CakSNP3239	*Kabuli*	Ca_Kabuli_Chr02	35122109	(C/G)
3240	CakSNP3240	*Kabuli*	Ca_Kabuli_Chr02	35154629	(C/A)
3241	CakSNP3241	*Kabuli*	Ca_Kabuli_Chr02	35154603	(G/A)
3242	CakSNP3242	*Kabuli*	Ca_Kabuli_Chr02	35171882	(T/G)
3243	CakSNP3243	*Kabuli*	Ca_Kabuli_Chr02	35189471	(C/T)
3244	CakSNP3244	*Kabuli*	Ca_Kabuli_Chr02	35195303	(T/C)
3245	CakSNP3245	*Kabuli*	Ca_Kabuli_Chr02	35198352	(T/C)
3246	CakSNP3246	*Kabuli*	Ca_Kabuli_Chr02	35209940	(C/T)
3247	CakSNP3247	*Kabuli*	Ca_Kabuli_Chr02	35362587	(A/G)
3248	CakSNP3248	*Kabuli*	Ca_Kabuli_Chr02	35362642	(A/G)
3249	CakSNP3249	*Kabuli*	Ca_Kabuli_Chr02	35362657	(C/A)
3250	CakSNP3250	*Kabuli*	Ca_Kabuli_Chr02	35362729	(A/G)
3251	CakSNP3251	*Kabuli*	Ca_Kabuli_Chr02	35363488	(T/C)
3252	CakSNP3252	*Kabuli*	Ca_Kabuli_Chr02	35363501	(T/C)
3253	CakSNP3253	*Kabuli*	Ca_Kabuli_Chr02	35365029	(C/T)
3254	CakSNP3254	*Kabuli*	Ca_Kabuli_Chr02	35367161	(G/A)
3255	CakSNP3255	*Kabuli*	Ca_Kabuli_Chr02	35413400	(T/A)
3256	CakSNP3256	*Kabuli*	Ca_Kabuli_Chr02	35441184	(C/T)
3257	CakSNP3257	*Kabuli*	Ca_Kabuli_Chr02	35441180	(A/G)
3258	CakSNP3258	*Kabuli*	Ca_Kabuli_Chr02	35441174	(T/G)
3259	CakSNP3259	*Kabuli*	Ca_Kabuli_Chr02	35447976	(A/G)
3260	CakSNP3260	*Kabuli*	Ca_Kabuli_Chr02	35448018	(C/G)
3261	CakSNP3261	*Kabuli*	Ca_Kabuli_Chr02	35448126	(T/A)
3262	CakSNP3262	*Kabuli*	Ca_Kabuli_Chr02	35449653	(G/A)
3263	CakSNP3263	*Kabuli*	Ca_Kabuli_Chr02	35473250	(C/T)
3264	CakSNP3264	*Kabuli*	Ca_Kabuli_Chr02	35505791	(C/G)
3265	CakSNP3265	*Kabuli*	Ca_Kabuli_Chr02	35505874	(T/G)
3266	CakSNP3266	*Kabuli*	Ca_Kabuli_Chr02	35533246	(G/A)
3267	CakSNP3267	*Kabuli*	Ca_Kabuli_Chr02	35593439	(T/C)

S.N.	SNP IDs	Cultivars	Chromosomes/scaffolds	Physical positions (bp)	SNPs
3268	CakSNP3268	*Kabuli*	Ca_Kabuli_Chr02	35593797	(G/T)
3269	CakSNP3269	*Kabuli*	Ca_Kabuli_Chr02	35593824	(A/C)
3270	CakSNP3270	*Kabuli*	Ca_Kabuli_Chr02	35593827	(A/G)
3271	CakSNP3271	*Kabuli*	Ca_Kabuli_Chr02	35594018	(A/T)
3272	CakSNP3272	*Kabuli*	Ca_Kabuli_Chr02	35594013	(C/T)
3273	CakSNP3273	*Kabuli*	Ca_Kabuli_Chr02	35593985	(A/G)
3274	CakSNP3274	*Kabuli*	Ca_Kabuli_Chr02	35593965	(C/T)
3275	CakSNP3275	*Kabuli*	Ca_Kabuli_Chr02	35593956	(A/G)
3276	CakSNP3276	*Kabuli*	Ca_Kabuli_Chr02	35601566	(T/C)
3277	CakSNP3277	*Kabuli*	Ca_Kabuli_Chr02	35601571	(A/C)
3278	CakSNP3278	*Kabuli*	Ca_Kabuli_Chr02	35601578	(C/T)
3279	CakSNP3279	*Kabuli*	Ca_Kabuli_Chr02	35601605	(G/A)
3280	CakSNP3280	*Kabuli*	Ca_Kabuli_Chr02	35601691	(A/G)
3281	CakSNP3281	*Kabuli*	Ca_Kabuli_Chr02	35601657	(T/C)
3282	CakSNP3282	*Kabuli*	Ca_Kabuli_Chr02	35601632	(G/T)
3283	CakSNP3283	*Kabuli*	Ca_Kabuli_Chr02	35612587	(C/A)
3284	CakSNP3284	*Kabuli*	Ca_Kabuli_Chr02	35612590	(G/A)
3285	CakSNP3285	*Kabuli*	Ca_Kabuli_Chr02	35612644	(G/A)
3286	CakSNP3286	*Kabuli*	Ca_Kabuli_Chr02	35612788	(G/A)
3287	CakSNP3287	*Kabuli*	Ca_Kabuli_Chr02	35612895	(T/C)
3288	CakSNP3288	*Kabuli*	Ca_Kabuli_Chr02	35612900	(C/T)
3289	CakSNP3289	*Kabuli*	Ca_Kabuli_Chr02	35613016	(G/A)
3290	CakSNP3290	*Kabuli*	Ca_Kabuli_Chr02	35613135	(T/C)
3291	CakSNP3291	*Kabuli*	Ca_Kabuli_Chr02	35658426	(C/T)
3292	CakSNP3292	*Kabuli*	Ca_Kabuli_Chr02	35658537	(T/C)
3293	CakSNP3293	*Kabuli*	Ca_Kabuli_Chr02	35661053	(C/T)
3294	CakSNP3294	*Kabuli*	Ca_Kabuli_Chr02	35670478	(A/G)
3295	CakSNP3295	*Kabuli*	Ca_Kabuli_Chr02	35704018	(A/G)
3296	CakSNP3296	*Kabuli*	Ca_Kabuli_Chr02	35704069	(G/A)
3297	CakSNP3297	*Kabuli*	Ca_Kabuli_Chr02	35704228	(C/A)
3298	CakSNP3298	*Kabuli*	Ca_Kabuli_Chr02	35704213	(A/G)
3299	CakSNP3299	*Kabuli*	Ca_Kabuli_Chr02	35704204	(T/C)
3300	CakSNP3300	*Kabuli*	Ca_Kabuli_Chr02	35704200	(T/C)
3301	CakSNP3301	*Kabuli*	Ca_Kabuli_Chr02	35704180	(A/G)
3302	CakSNP3302	*Kabuli*	Ca_Kabuli_Chr02	35704178	(G/A)
3303	CakSNP3303	*Kabuli*	Ca_Kabuli_Chr02	35728285	(T/A)
3304	CakSNP3304	*Kabuli*	Ca_Kabuli_Chr02	35754617	(G/T)
3305	CakSNP3305	*Kabuli*	Ca_Kabuli_Chr02	35754557	(C/T)

S.N.	SNP IDs	Cultivars	Chromosomes/scaffolds	Physical positions (bp)	SNPs
3306	CakSNP3306	*Kabuli*	Ca_Kabuli_Chr02	35754680	(C/A)
3307	CakSNP3307	*Kabuli*	Ca_Kabuli_Chr02	35754682	(A/G)
3308	CakSNP3308	*Kabuli*	Ca_Kabuli_Chr02	35754806	(G/C)
3309	CakSNP3309	*Kabuli*	Ca_Kabuli_Chr02	35754757	(G/C)
3310	CakSNP3310	*Kabuli*	Ca_Kabuli_Chr02	35759932	(A/T)
3311	CakSNP3311	*Kabuli*	Ca_Kabuli_Chr02	35759940	(T/C)
3312	CakSNP3312	*Kabuli*	Ca_Kabuli_Chr02	35759981	(G/A)
3313	CakSNP3313	*Kabuli*	Ca_Kabuli_Chr02	35760062	(G/C)
3314	CakSNP3314	*Kabuli*	Ca_Kabuli_Chr02	35760073	(A/T)
3315	CakSNP3315	*Kabuli*	Ca_Kabuli_Chr02	35760170	(A/G)
3316	CakSNP3316	*Kabuli*	Ca_Kabuli_Chr02	35760155	(G/A)
3317	CakSNP3317	*Kabuli*	Ca_Kabuli_Chr02	35770569	(A/G)
3318	CakSNP3318	*Kabuli*	Ca_Kabuli_Chr02	35795446	(G/A)
3319	CakSNP3319	*Kabuli*	Ca_Kabuli_Chr02	35795395	(A/G)
3320	CakSNP3320	*Kabuli*	Ca_Kabuli_Chr02	35795494	(G/A)
3321	CakSNP3321	*Kabuli*	Ca_Kabuli_Chr02	35795496	(G/T)
3322	CakSNP3322	*Kabuli*	Ca_Kabuli_Chr02	35795497	(G/T)
3323	CakSNP3323	*Kabuli*	Ca_Kabuli_Chr02	35795521	(T/C)
3324	CakSNP3324	*Kabuli*	Ca_Kabuli_Chr02	35828553	(G/A)
3325	CakSNP3325	*Kabuli*	Ca_Kabuli_Chr02	35829782	(A/G)
3326	CakSNP3326	*Kabuli*	Ca_Kabuli_Chr02	35844790	(A/G)
3327	CakSNP3327	*Kabuli*	Ca_Kabuli_Chr02	35844762	(A/G)
3328	CakSNP3328	*Kabuli*	Ca_Kabuli_Chr02	35862852	(C/T)
3329	CakSNP3329	*Kabuli*	Ca_Kabuli_Chr02	35862878	(T/G)
3330	CakSNP3330	*Kabuli*	Ca_Kabuli_Chr02	35875666	(A/G)
3331	CakSNP3331	*Kabuli*	Ca_Kabuli_Chr02	35875653	(C/T)
3332	CakSNP3332	*Kabuli*	Ca_Kabuli_Chr02	35930699	(T/G)
3333	CakSNP3333	*Kabuli*	Ca_Kabuli_Chr02	35993062	(C/T)
3334	CakSNP3334	*Kabuli*	Ca_Kabuli_Chr02	35993082	(T/G)
3335	CakSNP3335	*Kabuli*	Ca_Kabuli_Chr02	35993089	(C/T)
3336	CakSNP3336	*Kabuli*	Ca_Kabuli_Chr02	35993092	(A/T)
3337	CakSNP3337	*Kabuli*	Ca_Kabuli_Chr02	35993178	(G/A)
3338	CakSNP3338	*Kabuli*	Ca_Kabuli_Chr02	36013809	(A/G)
3339	CakSNP3339	*Kabuli*	Ca_Kabuli_Chr02	36039428	(C/T)
3340	CakSNP3340	*Kabuli*	Ca_Kabuli_Chr02	36039377	(C/T)
3341	CakSNP3341	*Kabuli*	Ca_Kabuli_Chr02	36039364	(G/T)
3342	CakSNP3342	*Kabuli*	Ca_Kabuli_Chr02	36046593	(G/T)
3343	CakSNP3343	*Kabuli*	Ca_Kabuli_Chr02	36046595	(T/G)

S.N.	SNP IDs	Cultivars	Chromosomes/scaffolds	Physical positions (bp)	SNPs
3344	CakSNP3344	*Kabuli*	Ca_Kabuli_Chr02	36046741	(G/A)
3345	CakSNP3345	*Kabuli*	Ca_Kabuli_Chr02	36046720	(T/A)
3346	CakSNP3346	*Kabuli*	Ca_Kabuli_Chr02	36046767	(T/A)
3347	CakSNP3347	*Kabuli*	Ca_Kabuli_Chr02	36046799	(C/A)
3348	CakSNP3348	*Kabuli*	Ca_Kabuli_Chr02	36046820	(T/A)
3349	CakSNP3349	*Kabuli*	Ca_Kabuli_Chr02	36046844	(T/G)
3350	CakSNP3350	*Kabuli*	Ca_Kabuli_Chr02	36069340	(T/C)
3351	CakSNP3351	*Kabuli*	Ca_Kabuli_Chr02	36072247	(C/A)
3352	CakSNP3352	*Kabuli*	Ca_Kabuli_Chr02	36074147	(T/C)
3353	CakSNP3353	*Kabuli*	Ca_Kabuli_Chr02	36088577	(A/T)
3354	CakSNP3354	*Kabuli*	Ca_Kabuli_Chr02	36088623	(C/T)
3355	CakSNP3355	*Kabuli*	Ca_Kabuli_Chr02	36088676	(T/C)
3356	CakSNP3356	*Kabuli*	Ca_Kabuli_Chr02	36088853	(G/A)
3357	CakSNP3357	*Kabuli*	Ca_Kabuli_Chr02	36101358	(A/G)
3358	CakSNP3358	*Kabuli*	Ca_Kabuli_Chr02	36101379	(A/G)
3359	CakSNP3359	*Kabuli*	Ca_Kabuli_Chr02	36101421	(C/T)
3360	CakSNP3360	*Kabuli*	Ca_Kabuli_Chr02	36117695	(T/A)
3361	CakSNP3361	*Kabuli*	Ca_Kabuli_Chr02	36117647	(G/A)
3362	CakSNP3362	*Kabuli*	Ca_Kabuli_Chr02	36253581	(C/T)
3363	CakSNP3363	*Kabuli*	Ca_Kabuli_Chr02	36276791	(G/A)
3364	CakSNP3364	*Kabuli*	Ca_Kabuli_Chr02	36323575	(G/T)
3365	CakSNP3365	*Kabuli*	Ca_Kabuli_Chr02	36326361	(A/C)
3366	CakSNP3366	*Kabuli*	Ca_Kabuli_Chr02	36335252	(A/C)
3367	CakSNP3367	*Kabuli*	Ca_Kabuli_Chr02	36343900	(C/T)
3368	CakSNP3368	*Kabuli*	Ca_Kabuli_Chr02	36343933	(T/C)
3369	CakSNP3369	*Kabuli*	Ca_Kabuli_Chr02	36346336	(A/G)
3370	CakSNP3370	*Kabuli*	Ca_Kabuli_Chr02	36375224	(A/G)
3371	CakSNP3371	*Kabuli*	Ca_Kabuli_Chr02	36375638	(A/G)
3372	CakSNP3372	*Kabuli*	Ca_Kabuli_Chr02	36387189	(G/C)
3373	CakSNP3373	*Kabuli*	Ca_Kabuli_Chr02	36387245	(T/C)
3374	CakSNP3374	*Kabuli*	Ca_Kabuli_Chr02	36389041	(G/A)
3375	CakSNP3375	*Kabuli*	Ca_Kabuli_Chr02	36389096	(C/T)
3376	CakSNP3376	*Kabuli*	Ca_Kabuli_Chr02	36403788	(A/G)
3377	CakSNP3377	*Kabuli*	Ca_Kabuli_Chr02	36427841	(C/T)
3378	CakSNP3378	*Kabuli*	Ca_Kabuli_Chr02	36429512	(T/C)
3379	CakSNP3379	*Kabuli*	Ca_Kabuli_Chr02	36438939	(T/G)
3380	CakSNP3380	*Kabuli*	Ca_Kabuli_Chr02	36453691	(A/G)
3381	CakSNP3381	*Kabuli*	Ca_Kabuli_Chr02	36453685	(G/A)

S.N.	SNP IDs	Cultivars	Chromosomes/scaffolds	Physical positions (bp)	SNPs
3382	CakSNP3382	*Kabuli*	Ca_Kabuli_Chr02	36466837	(C/T)
3383	CakSNP3383	*Kabuli*	Ca_Kabuli_Chr02	36466998	(A/G)
3384	CakSNP3384	*Kabuli*	Ca_Kabuli_Chr02	36467100	(A/C)
3385	CakSNP3385	*Kabuli*	Ca_Kabuli_Chr02	36468390	(A/G)
3386	CakSNP3386	*Kabuli*	Ca_Kabuli_Chr02	36468505	(C/A)
3387	CakSNP3387	*Kabuli*	Ca_Kabuli_Chr02	36469921	(A/G)
3388	CakSNP3388	*Kabuli*	Ca_Kabuli_Chr02	36505316	(C/A)
3389	CakSNP3389	*Kabuli*	Ca_Kabuli_Chr02	36521200	(T/C)
3390	CakSNP3390	*Kabuli*	Ca_Kabuli_Chr02	36533821	(A/C)
3391	CakSNP3391	*Kabuli*	Ca_Kabuli_Chr02	36533910	(G/A)
3392	CakSNP3392	*Kabuli*	Ca_Kabuli_Chr02	36554430	(G/A)
3393	CakSNP3393	*Kabuli*	Ca_Kabuli_Chr02	36609730	(T/A)
3394	CakSNP3394	*Kabuli*	Ca_Kabuli_Chr02	36609743	(C/T)
3395	CakSNP3395	*Kabuli*	Ca_Kabuli_Chr02	36609744	(G/C)
3396	CakSNP3396	*Kabuli*	Ca_Kabuli_Chr02	36609858	(C/G)
3397	CakSNP3397	*Kabuli*	Ca_Kabuli_Chr02	36609950	(C/T)
3398	CakSNP3398	*Kabuli*	Ca_Kabuli_Chr02	36609968	(C/T)
3399	CakSNP3399	*Kabuli*	Ca_Kabuli_Chr02	36634234	(T/C)
3400	CakSNP3400	*Kabuli*	Ca_Kabuli_Chr02	36634297	(C/G)
3401	CakSNP3401	*Kabuli*	Ca_Kabuli_Chr03	171409	(A/T)
3402	CakSNP3402	*Kabuli*	Ca_Kabuli_Chr03	171468	(A/G)
3403	CakSNP3403	*Kabuli*	Ca_Kabuli_Chr03	171574	(C/A)
3404	CakSNP3404	*Kabuli*	Ca_Kabuli_Chr03	171576	(G/T)
3405	CakSNP3405	*Kabuli*	Ca_Kabuli_Chr03	171579	(C/T)
3406	CakSNP3406	*Kabuli*	Ca_Kabuli_Chr03	171675	(T/A)
3407	CakSNP3407	*Kabuli*	Ca_Kabuli_Chr03	208233	(C/A)
3408	CakSNP3408	*Kabuli*	Ca_Kabuli_Chr03	220564	(A/G)
3409	CakSNP3409	*Kabuli*	Ca_Kabuli_Chr03	220568	(A/T)
3410	CakSNP3410	*Kabuli*	Ca_Kabuli_Chr03	220572	(T/C)
3411	CakSNP3411	*Kabuli*	Ca_Kabuli_Chr03	220573	(G/T)
3412	CakSNP3412	*Kabuli*	Ca_Kabuli_Chr03	223943	(G/A)
3413	CakSNP3413	*Kabuli*	Ca_Kabuli_Chr03	223976	(G/C)
3414	CakSNP3414	*Kabuli*	Ca_Kabuli_Chr03	286425	(G/T)
3415	CakSNP3415	*Kabuli*	Ca_Kabuli_Chr03	419715	(T/G)
3416	CakSNP3416	*Kabuli*	Ca_Kabuli_Chr03	988477	(G/A)
3417	CakSNP3417	*Kabuli*	Ca_Kabuli_Chr03	988471	(T/C)
3418	CakSNP3418	*Kabuli*	Ca_Kabuli_Chr03	1499038	(G/A)
3419	CakSNP3419	*Kabuli*	Ca_Kabuli_Chr03	1499006	(A/G)

S.N.	SNP IDs	Cultivars	Chromosomes/scaffolds	Physical positions (bp)	SNPs
3420	CakSNP3420	*Kabuli*	Ca_Kabuli_Chr03	1746388	(C/A)
3421	CakSNP3421	*Kabuli*	Ca_Kabuli_Chr03	1746389	(A/G)
3422	CakSNP3422	*Kabuli*	Ca_Kabuli_Chr03	1829412	(T/G)
3423	CakSNP3423	*Kabuli*	Ca_Kabuli_Chr03	1829358	(T/C)
3424	CakSNP3424	*Kabuli*	Ca_Kabuli_Chr03	1829352	(C/T)
3425	CakSNP3425	*Kabuli*	Ca_Kabuli_Chr03	1831920	(T/G)
3426	CakSNP3426	*Kabuli*	Ca_Kabuli_Chr03	1831866	(T/C)
3427	CakSNP3427	*Kabuli*	Ca_Kabuli_Chr03	1831860	(C/T)
3428	CakSNP3428	*Kabuli*	Ca_Kabuli_Chr03	1831931	(A/C)
3429	CakSNP3429	*Kabuli*	Ca_Kabuli_Chr03	2097376	(T/G)
3430	CakSNP3430	*Kabuli*	Ca_Kabuli_Chr03	2097413	(A/G)
3431	CakSNP3431	*Kabuli*	Ca_Kabuli_Chr03	2391515	(T/G)
3432	CakSNP3432	*Kabuli*	Ca_Kabuli_Chr03	2423760	(T/C)
3433	CakSNP3433	*Kabuli*	Ca_Kabuli_Chr03	2708703	(C/T)
3434	CakSNP3434	*Kabuli*	Ca_Kabuli_Chr03	2825546	(G/T)
3435	CakSNP3435	*Kabuli*	Ca_Kabuli_Chr03	2927148	(T/A)
3436	CakSNP3436	*Kabuli*	Ca_Kabuli_Chr03	2927150	(A/T)
3437	CakSNP3437	*Kabuli*	Ca_Kabuli_Chr03	2931027	(G/C)
3438	CakSNP3438	*Kabuli*	Ca_Kabuli_Chr03	2931046	(C/T)
3439	CakSNP3439	*Kabuli*	Ca_Kabuli_Chr03	2975179	(T/C)
3440	CakSNP3440	*Kabuli*	Ca_Kabuli_Chr03	3280478	(C/T)
3441	CakSNP3441	*Kabuli*	Ca_Kabuli_Chr03	3280500	(C/T)
3442	CakSNP3442	*Kabuli*	Ca_Kabuli_Chr03	3280530	(G/A)
3443	CakSNP3443	*Kabuli*	Ca_Kabuli_Chr03	3280595	(G/A)
3444	CakSNP3444	*Kabuli*	Ca_Kabuli_Chr03	3617555	(G/A)
3445	CakSNP3445	*Kabuli*	Ca_Kabuli_Chr03	3701394	(A/T)
3446	CakSNP3446	*Kabuli*	Ca_Kabuli_Chr03	3701396	(C/T)
3447	CakSNP3447	*Kabuli*	Ca_Kabuli_Chr03	3701398	(A/T)
3448	CakSNP3448	*Kabuli*	Ca_Kabuli_Chr03	3701401	(A/T)
3449	CakSNP3449	*Kabuli*	Ca_Kabuli_Chr03	3701403	(A/T)
3450	CakSNP3450	*Kabuli*	Ca_Kabuli_Chr03	3701405	(G/T)
3451	CakSNP3451	*Kabuli*	Ca_Kabuli_Chr03	3701415	(C/T)
3452	CakSNP3452	*Kabuli*	Ca_Kabuli_Chr03	3701422	(C/T)
3453	CakSNP3453	*Kabuli*	Ca_Kabuli_Chr03	3701426	(T/C)
3454	CakSNP3454	*Kabuli*	Ca_Kabuli_Chr03	3701435	(A/T)
3455	CakSNP3455	*Kabuli*	Ca_Kabuli_Chr03	3701436	(C/T)
3456	CakSNP3456	*Kabuli*	Ca_Kabuli_Chr03	3701437	(A/T)
3457	CakSNP3457	*Kabuli*	Ca_Kabuli_Chr03	3701440	(G/T)

S.N.	SNP IDs	Cultivars	Chromosomes/scaffolds	Physical positions (bp)	SNPs
3458	CakSNP3458	*Kabuli*	Ca_Kabuli_Chr03	3701450	(C/T)
3459	CakSNP3459	*Kabuli*	Ca_Kabuli_Chr03	3701375	(G/A)
3460	CakSNP3460	*Kabuli*	Ca_Kabuli_Chr03	3701370	(G/A)
3461	CakSNP3461	*Kabuli*	Ca_Kabuli_Chr03	3744908	(G/A)
3462	CakSNP3462	*Kabuli*	Ca_Kabuli_Chr03	3764420	(T/A)
3463	CakSNP3463	*Kabuli*	Ca_Kabuli_Chr03	3764508	(T/C)
3464	CakSNP3464	*Kabuli*	Ca_Kabuli_Chr03	3764545	(C/T)
3465	CakSNP3465	*Kabuli*	Ca_Kabuli_Chr03	4145743	(G/A)
3466	CakSNP3466	*Kabuli*	Ca_Kabuli_Chr03	4178154	(C/T)
3467	CakSNP3467	*Kabuli*	Ca_Kabuli_Chr03	4266739	(G/A)
3468	CakSNP3468	*Kabuli*	Ca_Kabuli_Chr03	4266700	(T/G)
3469	CakSNP3469	*Kabuli*	Ca_Kabuli_Chr03	4266691	(C/T)
3470	CakSNP3470	*Kabuli*	Ca_Kabuli_Chr03	4266686	(C/T)
3471	CakSNP3471	*Kabuli*	Ca_Kabuli_Chr03	4266668	(G/A)
3472	CakSNP3472	*Kabuli*	Ca_Kabuli_Chr03	4555124	(G/A)
3473	CakSNP3473	*Kabuli*	Ca_Kabuli_Chr03	4555142	(T/A)
3474	CakSNP3474	*Kabuli*	Ca_Kabuli_Chr03	4555167	(C/G)
3475	CakSNP3475	*Kabuli*	Ca_Kabuli_Chr03	4555177	(C/T)
3476	CakSNP3476	*Kabuli*	Ca_Kabuli_Chr03	4596815	(C/T)
3477	CakSNP3477	*Kabuli*	Ca_Kabuli_Chr03	4596782	(A/G)
3478	CakSNP3478	*Kabuli*	Ca_Kabuli_Chr03	4604649	(A/G)
3479	CakSNP3479	*Kabuli*	Ca_Kabuli_Chr03	4606129	(T/A)
3480	CakSNP3480	*Kabuli*	Ca_Kabuli_Chr03	4606128	(A/G)
3481	CakSNP3481	*Kabuli*	Ca_Kabuli_Chr03	4629742	(A/T)
3482	CakSNP3482	*Kabuli*	Ca_Kabuli_Chr03	4629747	(A/C)
3483	CakSNP3483	*Kabuli*	Ca_Kabuli_Chr03	4654906	(C/T)
3484	CakSNP3484	*Kabuli*	Ca_Kabuli_Chr03	4654898	(G/T)
3485	CakSNP3485	*Kabuli*	Ca_Kabuli_Chr03	4654896	(T/C)
3486	CakSNP3486	*Kabuli*	Ca_Kabuli_Chr03	4823828	(T/C)
3487	CakSNP3487	*Kabuli*	Ca_Kabuli_Chr03	5224781	(A/G)
3488	CakSNP3488	*Kabuli*	Ca_Kabuli_Chr03	5225052	(A/G)
3489	CakSNP3489	*Kabuli*	Ca_Kabuli_Chr03	5225063	(G/T)
3490	CakSNP3490	*Kabuli*	Ca_Kabuli_Chr03	5225108	(T/G)
3491	CakSNP3491	*Kabuli*	Ca_Kabuli_Chr03	5225079	(G/A)
3492	CakSNP3492	*Kabuli*	Ca_Kabuli_Chr03	5415782	(G/A)
3493	CakSNP3493	*Kabuli*	Ca_Kabuli_Chr03	5436267	(A/G)
3494	CakSNP3494	*Kabuli*	Ca_Kabuli_Chr03	5436272	(T/C)
3495	CakSNP3495	*Kabuli*	Ca_Kabuli_Chr03	5436277	(C/T)

S.N.	SNP IDs	Cultivars	Chromosomes/scaffolds	Physical positions (bp)	SNPs
3496	CakSNP3496	*Kabuli*	Ca_Kabuli_Chr03	5436288	(G/T)
3497	CakSNP3497	*Kabuli*	Ca_Kabuli_Chr03	5436295	(G/A)
3498	CakSNP3498	*Kabuli*	Ca_Kabuli_Chr03	5436296	(C/T)
3499	CakSNP3499	*Kabuli*	Ca_Kabuli_Chr03	5436299	(A/C)
3500	CakSNP3500	*Kabuli*	Ca_Kabuli_Chr03	5436331	(C/A)
3501	CakSNP3501	*Kabuli*	Ca_Kabuli_Chr03	5514176	(A/G)
3502	CakSNP3502	*Kabuli*	Ca_Kabuli_Chr03	5514287	(C/A)
3503	CakSNP3503	*Kabuli*	Ca_Kabuli_Chr03	6169456	(G/T)
3504	CakSNP3504	*Kabuli*	Ca_Kabuli_Chr03	6169539	(T/C)
3505	CakSNP3505	*Kabuli*	Ca_Kabuli_Chr03	6169510	(A/C)
3506	CakSNP3506	*Kabuli*	Ca_Kabuli_Chr03	6278104	(G/A)
3507	CakSNP3507	*Kabuli*	Ca_Kabuli_Chr03	6467014	(G/A)
3508	CakSNP3508	*Kabuli*	Ca_Kabuli_Chr03	6683646	(A/G)
3509	CakSNP3509	*Kabuli*	Ca_Kabuli_Chr03	6683587	(T/A)
3510	CakSNP3510	*Kabuli*	Ca_Kabuli_Chr03	6828181	(A/T)
3511	CakSNP3511	*Kabuli*	Ca_Kabuli_Chr03	6840644	(C/T)
3512	CakSNP3512	*Kabuli*	Ca_Kabuli_Chr03	6894987	(C/A)
3513	CakSNP3513	*Kabuli*	Ca_Kabuli_Chr03	6895085	(T/C)
3514	CakSNP3514	*Kabuli*	Ca_Kabuli_Chr03	6899667	(T/C)
3515	CakSNP3515	*Kabuli*	Ca_Kabuli_Chr03	7013492	(G/A)
3516	CakSNP3516	*Kabuli*	Ca_Kabuli_Chr03	7015213	(T/G)
3517	CakSNP3517	*Kabuli*	Ca_Kabuli_Chr03	7024620	(G/A)
3518	CakSNP3518	*Kabuli*	Ca_Kabuli_Chr03	7204053	(G/A)
3519	CakSNP3519	*Kabuli*	Ca_Kabuli_Chr03	7204071	(T/C)
3520	CakSNP3520	*Kabuli*	Ca_Kabuli_Chr03	7204087	(C/T)
3521	CakSNP3521	*Kabuli*	Ca_Kabuli_Chr03	7204088	(G/C)
3522	CakSNP3522	*Kabuli*	Ca_Kabuli_Chr03	7204098	(G/A)
3523	CakSNP3523	*Kabuli*	Ca_Kabuli_Chr03	7204099	(C/T)
3524	CakSNP3524	*Kabuli*	Ca_Kabuli_Chr03	7204111	(G/A)
3525	CakSNP3525	*Kabuli*	Ca_Kabuli_Chr03	7204112	(G/A)
3526	CakSNP3526	*Kabuli*	Ca_Kabuli_Chr03	7204133	(G/T)
3527	CakSNP3527	*Kabuli*	Ca_Kabuli_Chr03	7226689	(T/C)
3528	CakSNP3528	*Kabuli*	Ca_Kabuli_Chr03	7226661	(G/T)
3529	CakSNP3529	*Kabuli*	Ca_Kabuli_Chr03	7226632	(G/T)
3530	CakSNP3530	*Kabuli*	Ca_Kabuli_Chr03	7246049	(T/G)
3531	CakSNP3531	*Kabuli*	Ca_Kabuli_Chr03	7420705	(G/A)
3532	CakSNP3532	*Kabuli*	Ca_Kabuli_Chr03	7433747	(C/A)
3533	CakSNP3533	*Kabuli*	Ca_Kabuli_Chr03	7461833	(A/G)

S.N.	SNP IDs	Cultivars	Chromosomes/scaffolds	Physical positions (bp)	SNPs
3534	CakSNP3534	*Kabuli*	Ca_Kabuli_Chr03	7482871	(C/T)
3535	CakSNP3535	*Kabuli*	Ca_Kabuli_Chr03	7606843	(A/G)
3536	CakSNP3536	*Kabuli*	Ca_Kabuli_Chr03	7606901	(T/C)
3537	CakSNP3537	*Kabuli*	Ca_Kabuli_Chr03	7606905	(C/G)
3538	CakSNP3538	*Kabuli*	Ca_Kabuli_Chr03	7608100	(A/G)
3539	CakSNP3539	*Kabuli*	Ca_Kabuli_Chr03	7665123	(G/A)
3540	CakSNP3540	*Kabuli*	Ca_Kabuli_Chr03	7665112	(C/T)
3541	CakSNP3541	*Kabuli*	Ca_Kabuli_Chr03	7665100	(G/T)
3542	CakSNP3542	*Kabuli*	Ca_Kabuli_Chr03	7665098	(C/T)
3543	CakSNP3543	*Kabuli*	Ca_Kabuli_Chr03	7665090	(C/T)
3544	CakSNP3544	*Kabuli*	Ca_Kabuli_Chr03	7665078	(G/A)
3545	CakSNP3545	*Kabuli*	Ca_Kabuli_Chr03	7665075	(G/A)
3546	CakSNP3546	*Kabuli*	Ca_Kabuli_Chr03	7665156	(G/A)
3547	CakSNP3547	*Kabuli*	Ca_Kabuli_Chr03	7665149	(T/G)
3548	CakSNP3548	*Kabuli*	Ca_Kabuli_Chr03	7665142	(G/A)
3549	CakSNP3549	*Kabuli*	Ca_Kabuli_Chr03	7665140	(G/T)
3550	CakSNP3550	*Kabuli*	Ca_Kabuli_Chr03	7665157	(C/T)
3551	CakSNP3551	*Kabuli*	Ca_Kabuli_Chr03	7694198	(C/T)
3552	CakSNP3552	*Kabuli*	Ca_Kabuli_Chr03	7694297	(T/C)
3553	CakSNP3553	*Kabuli*	Ca_Kabuli_Chr03	8536213	(T/C)
3554	CakSNP3554	*Kabuli*	Ca_Kabuli_Chr03	8536275	(C/T)
3555	CakSNP3555	*Kabuli*	Ca_Kabuli_Chr03	8536295	(A/T)
3556	CakSNP3556	*Kabuli*	Ca_Kabuli_Chr03	8536301	(G/T)
3557	CakSNP3557	*Kabuli*	Ca_Kabuli_Chr03	8536425	(C/A)
3558	CakSNP3558	*Kabuli*	Ca_Kabuli_Chr03	8536498	(T/A)
3559	CakSNP3559	*Kabuli*	Ca_Kabuli_Chr03	8536559	(T/C)
3560	CakSNP3560	*Kabuli*	Ca_Kabuli_Chr03	8668628	(A/C)
3561	CakSNP3561	*Kabuli*	Ca_Kabuli_Chr03	8669004	(A/T)
3562	CakSNP3562	*Kabuli*	Ca_Kabuli_Chr03	8669132	(T/A)
3563	CakSNP3563	*Kabuli*	Ca_Kabuli_Chr03	8697186	(A/G)
3564	CakSNP3564	*Kabuli*	Ca_Kabuli_Chr03	8712381	(A/C)
3565	CakSNP3565	*Kabuli*	Ca_Kabuli_Chr03	8933037	(T/C)
3566	CakSNP3566	*Kabuli*	Ca_Kabuli_Chr03	8933084	(G/A)
3567	CakSNP3567	*Kabuli*	Ca_Kabuli_Chr03	8933086	(C/A)
3568	CakSNP3568	*Kabuli*	Ca_Kabuli_Chr03	8933221	(T/A)
3569	CakSNP3569	*Kabuli*	Ca_Kabuli_Chr03	8933183	(A/G)
3570	CakSNP3570	*Kabuli*	Ca_Kabuli_Chr03	8933255	(A/T)
3571	CakSNP3571	*Kabuli*	Ca_Kabuli_Chr03	8933266	(G/T)

S.N.	SNP IDs	Cultivars	Chromosomes/scaffolds	Physical positions (bp)	SNPs
3572	CakSNP3572	*Kabuli*	Ca_Kabuli_Chr03	8933409	(A/C)
3573	CakSNP3573	*Kabuli*	Ca_Kabuli_Chr03	8933370	(A/G)
3574	CakSNP3574	*Kabuli*	Ca_Kabuli_Chr03	8933355	(A/T)
3575	CakSNP3575	*Kabuli*	Ca_Kabuli_Chr03	9221403	(G/A)
3576	CakSNP3576	*Kabuli*	Ca_Kabuli_Chr03	9221528	(C/T)
3577	CakSNP3577	*Kabuli*	Ca_Kabuli_Chr03	9221579	(G/C)
3578	CakSNP3578	*Kabuli*	Ca_Kabuli_Chr03	9221580	(T/G)
3579	CakSNP3579	*Kabuli*	Ca_Kabuli_Chr03	9223444	(G/C)
3580	CakSNP3580	*Kabuli*	Ca_Kabuli_Chr03	9223875	(G/A)
3581	CakSNP3581	*Kabuli*	Ca_Kabuli_Chr03	9224033	(C/A)
3582	CakSNP3582	*Kabuli*	Ca_Kabuli_Chr03	9224011	(G/A)
3583	CakSNP3583	*Kabuli*	Ca_Kabuli_Chr03	9296469	(T/G)
3584	CakSNP3584	*Kabuli*	Ca_Kabuli_Chr03	9448525	(T/C)
3585	CakSNP3585	*Kabuli*	Ca_Kabuli_Chr03	9577402	(G/A)
3586	CakSNP3586	*Kabuli*	Ca_Kabuli_Chr03	9579805	(A/T)
3587	CakSNP3587	*Kabuli*	Ca_Kabuli_Chr03	9579808	(C/T)
3588	CakSNP3588	*Kabuli*	Ca_Kabuli_Chr03	9579811	(G/C)
3589	CakSNP3589	*Kabuli*	Ca_Kabuli_Chr03	9579817	(G/A)
3590	CakSNP3590	*Kabuli*	Ca_Kabuli_Chr03	9579818	(G/T)
3591	CakSNP3591	*Kabuli*	Ca_Kabuli_Chr03	9579824	(A/G)
3592	CakSNP3592	*Kabuli*	Ca_Kabuli_Chr03	9579830	(T/A)
3593	CakSNP3593	*Kabuli*	Ca_Kabuli_Chr03	9579831	(C/G)
3594	CakSNP3594	*Kabuli*	Ca_Kabuli_Chr03	9579839	(G/A)
3595	CakSNP3595	*Kabuli*	Ca_Kabuli_Chr03	9894316	(C/T)
3596	CakSNP3596	*Kabuli*	Ca_Kabuli_Chr03	9894310	(C/A)
3597	CakSNP3597	*Kabuli*	Ca_Kabuli_Chr03	10039539	(T/A)
3598	CakSNP3598	*Kabuli*	Ca_Kabuli_Chr03	10105733	(A/G)
3599	CakSNP3599	*Kabuli*	Ca_Kabuli_Chr03	10142152	(C/A)
3600	CakSNP3600	*Kabuli*	Ca_Kabuli_Chr03	10159836	(A/C)
3601	CakSNP3601	*Kabuli*	Ca_Kabuli_Chr03	10159859	(G/A)
3602	CakSNP3602	*Kabuli*	Ca_Kabuli_Chr03	10159941	(C/A)
3603	CakSNP3603	*Kabuli*	Ca_Kabuli_Chr03	10159943	(G/A)
3604	CakSNP3604	*Kabuli*	Ca_Kabuli_Chr03	10159944	(T/C)
3605	CakSNP3605	*Kabuli*	Ca_Kabuli_Chr03	10186893	(G/C)
3606	CakSNP3606	*Kabuli*	Ca_Kabuli_Chr03	10186861	(G/T)
3607	CakSNP3607	*Kabuli*	Ca_Kabuli_Chr03	10186857	(A/G)
3608	CakSNP3608	*Kabuli*	Ca_Kabuli_Chr03	10186880	(A/G)
3609	CakSNP3609	*Kabuli*	Ca_Kabuli_Chr03	10186889	(G/A)

S.N.	SNP IDs	Cultivars	Chromosomes/scaffolds	Physical positions (bp)	SNPs
3610	CakSNP3610	*Kabuli*	Ca_Kabuli_Chr03	10186891	(C/G)
3611	CakSNP3611	*Kabuli*	Ca_Kabuli_Chr03	10201361	(G/T)
3612	CakSNP3612	*Kabuli*	Ca_Kabuli_Chr03	10722780	(G/T)
3613	CakSNP3613	*Kabuli*	Ca_Kabuli_Chr03	10722849	(G/C)
3614	CakSNP3614	*Kabuli*	Ca_Kabuli_Chr03	10722859	(G/A)
3615	CakSNP3615	*Kabuli*	Ca_Kabuli_Chr03	11143751	(G/A)
3616	CakSNP3616	*Kabuli*	Ca_Kabuli_Chr03	11143818	(G/A)
3617	CakSNP3617	*Kabuli*	Ca_Kabuli_Chr03	11143816	(C/T)
3618	CakSNP3618	*Kabuli*	Ca_Kabuli_Chr03	11376752	(C/A)
3619	CakSNP3619	*Kabuli*	Ca_Kabuli_Chr03	11378141	(C/T)
3620	CakSNP3620	*Kabuli*	Ca_Kabuli_Chr03	11378425	(G/T)
3621	CakSNP3621	*Kabuli*	Ca_Kabuli_Chr03	11590767	(C/T)
3622	CakSNP3622	*Kabuli*	Ca_Kabuli_Chr03	11590793	(C/T)
3623	CakSNP3623	*Kabuli*	Ca_Kabuli_Chr03	11590817	(C/G)
3624	CakSNP3624	*Kabuli*	Ca_Kabuli_Chr03	11590874	(A/G)
3625	CakSNP3625	*Kabuli*	Ca_Kabuli_Chr03	11590860	(G/A)
3626	CakSNP3626	*Kabuli*	Ca_Kabuli_Chr03	11590832	(G/A)
3627	CakSNP3627	*Kabuli*	Ca_Kabuli_Chr03	11590814	(T/C)
3628	CakSNP3628	*Kabuli*	Ca_Kabuli_Chr03	11610500	(A/G)
3629	CakSNP3629	*Kabuli*	Ca_Kabuli_Chr03	11610494	(C/T)
3630	CakSNP3630	*Kabuli*	Ca_Kabuli_Chr03	11610460	(A/G)
3631	CakSNP3631	*Kabuli*	Ca_Kabuli_Chr03	11610441	(G/T)
3632	CakSNP3632	*Kabuli*	Ca_Kabuli_Chr03	11610481	(C/T)
3633	CakSNP3633	*Kabuli*	Ca_Kabuli_Chr03	11610451	(C/T)
3634	CakSNP3634	*Kabuli*	Ca_Kabuli_Chr03	11678625	(G/A)
3635	CakSNP3635	*Kabuli*	Ca_Kabuli_Chr03	11684552	(G/A)
3636	CakSNP3636	*Kabuli*	Ca_Kabuli_Chr03	11709777	(G/T)
3637	CakSNP3637	*Kabuli*	Ca_Kabuli_Chr03	11811685	(A/G)
3638	CakSNP3638	*Kabuli*	Ca_Kabuli_Chr03	12051826	(A/C)
3639	CakSNP3639	*Kabuli*	Ca_Kabuli_Chr03	12058576	(T/C)
3640	CakSNP3640	*Kabuli*	Ca_Kabuli_Chr03	12058756	(A/G)
3641	CakSNP3641	*Kabuli*	Ca_Kabuli_Chr03	12058748	(A/T)
3642	CakSNP3642	*Kabuli*	Ca_Kabuli_Chr03	12067458	(A/C)
3643	CakSNP3643	*Kabuli*	Ca_Kabuli_Chr03	12069092	(T/G)
3644	CakSNP3644	*Kabuli*	Ca_Kabuli_Chr03	12095571	(T/G)
3645	CakSNP3645	*Kabuli*	Ca_Kabuli_Chr03	12124099	(G/A)
3646	CakSNP3646	*Kabuli*	Ca_Kabuli_Chr03	12394742	(G/A)
3647	CakSNP3647	*Kabuli*	Ca_Kabuli_Chr03	12599496	(A/C)

S.N.	SNP IDs	Cultivars	Chromosomes/scaffolds	Physical positions (bp)	SNPs
3648	CakSNP3648	*Kabuli*	Ca_Kabuli_Chr03	12622503	(A/G)
3649	CakSNP3649	*Kabuli*	Ca_Kabuli_Chr03	12622504	(G/T)
3650	CakSNP3650	*Kabuli*	Ca_Kabuli_Chr03	12622518	(T/G)
3651	CakSNP3651	*Kabuli*	Ca_Kabuli_Chr03	12622534	(C/T)
3652	CakSNP3652	*Kabuli*	Ca_Kabuli_Chr03	12622550	(A/T)
3653	CakSNP3653	*Kabuli*	Ca_Kabuli_Chr03	12622578	(G/A)
3654	CakSNP3654	*Kabuli*	Ca_Kabuli_Chr03	12622567	(A/C)
3655	CakSNP3655	*Kabuli*	Ca_Kabuli_Chr03	12622543	(G/A)
3656	CakSNP3656	*Kabuli*	Ca_Kabuli_Chr03	12627120	(C/T)
3657	CakSNP3657	*Kabuli*	Ca_Kabuli_Chr03	12627125	(T/C)
3658	CakSNP3658	*Kabuli*	Ca_Kabuli_Chr03	12627131	(G/A)
3659	CakSNP3659	*Kabuli*	Ca_Kabuli_Chr03	12627144	(G/A)
3660	CakSNP3660	*Kabuli*	Ca_Kabuli_Chr03	12627165	(T/C)
3661	CakSNP3661	*Kabuli*	Ca_Kabuli_Chr03	12627143	(T/C)
3662	CakSNP3662	*Kabuli*	Ca_Kabuli_Chr03	12627164	(G/A)
3663	CakSNP3663	*Kabuli*	Ca_Kabuli_Chr03	12627170	(C/G)
3664	CakSNP3664	*Kabuli*	Ca_Kabuli_Chr03	12809390	(A/G)
3665	CakSNP3665	*Kabuli*	Ca_Kabuli_Chr03	12809794	(T/C)
3666	CakSNP3666	*Kabuli*	Ca_Kabuli_Chr03	12814631	(C/T)
3667	CakSNP3667	*Kabuli*	Ca_Kabuli_Chr03	12814603	(T/C)
3668	CakSNP3668	*Kabuli*	Ca_Kabuli_Chr03	12814601	(A/C)
3669	CakSNP3669	*Kabuli*	Ca_Kabuli_Chr03	12814600	(A/G)
3670	CakSNP3670	*Kabuli*	Ca_Kabuli_Chr03	12814599	(A/T)
3671	CakSNP3671	*Kabuli*	Ca_Kabuli_Chr03	12814597	(T/A)
3672	CakSNP3672	*Kabuli*	Ca_Kabuli_Chr03	12814571	(C/A)
3673	CakSNP3673	*Kabuli*	Ca_Kabuli_Chr03	12814558	(A/G)
3674	CakSNP3674	*Kabuli*	Ca_Kabuli_Chr03	12897820	(C/T)
3675	CakSNP3675	*Kabuli*	Ca_Kabuli_Chr03	12897831	(C/A)
3676	CakSNP3676	*Kabuli*	Ca_Kabuli_Chr03	12897839	(G/A)
3677	CakSNP3677	*Kabuli*	Ca_Kabuli_Chr03	12897841	(C/A)
3678	CakSNP3678	*Kabuli*	Ca_Kabuli_Chr03	12897876	(C/T)
3679	CakSNP3679	*Kabuli*	Ca_Kabuli_Chr03	12897881	(C/T)
3680	CakSNP3680	*Kabuli*	Ca_Kabuli_Chr03	12897882	(T/G)
3681	CakSNP3681	*Kabuli*	Ca_Kabuli_Chr03	12897926	(A/C)
3682	CakSNP3682	*Kabuli*	Ca_Kabuli_Chr03	12897906	(C/A)
3683	CakSNP3683	*Kabuli*	Ca_Kabuli_Chr03	12897887	(T/C)
3684	CakSNP3684	*Kabuli*	Ca_Kabuli_Chr03	12897884	(C/T)
3685	CakSNP3685	*Kabuli*	Ca_Kabuli_Chr03	12897883	(G/T)

S.N.	SNP IDs	Cultivars	Chromosomes/scaffolds	Physical positions (bp)	SNPs
3686	CakSNP3686	*Kabuli*	Ca_Kabuli_Chr03	12897886	(G/A)
3687	CakSNP3687	*Kabuli*	Ca_Kabuli_Chr03	13185541	(G/A)
3688	CakSNP3688	*Kabuli*	Ca_Kabuli_Chr03	13185530	(C/T)
3689	CakSNP3689	*Kabuli*	Ca_Kabuli_Chr03	13185496	(G/A)
3690	CakSNP3690	*Kabuli*	Ca_Kabuli_Chr03	13185490	(G/A)
3691	CakSNP3691	*Kabuli*	Ca_Kabuli_Chr03	13185493	(G/A)
3692	CakSNP3692	*Kabuli*	Ca_Kabuli_Chr03	13185508	(C/T)
3693	CakSNP3693	*Kabuli*	Ca_Kabuli_Chr03	13185516	(C/T)
3694	CakSNP3694	*Kabuli*	Ca_Kabuli_Chr03	13185583	(T/C)
3695	CakSNP3695	*Kabuli*	Ca_Kabuli_Chr03	13185581	(T/A)
3696	CakSNP3696	*Kabuli*	Ca_Kabuli_Chr03	13185574	(G/A)
3697	CakSNP3697	*Kabuli*	Ca_Kabuli_Chr03	13185560	(G/A)
3698	CakSNP3698	*Kabuli*	Ca_Kabuli_Chr03	13185575	(C/T)
3699	CakSNP3699	*Kabuli*	Ca_Kabuli_Chr03	13185582	(A/G)
3700	CakSNP3700	*Kabuli*	Ca_Kabuli_Chr03	13185590	(C/A)
3701	CakSNP3701	*Kabuli*	Ca_Kabuli_Chr03	13185591	(C/T)
3702	CakSNP3702	*Kabuli*	Ca_Kabuli_Chr03	13185592	(A/C)
3703	CakSNP3703	*Kabuli*	Ca_Kabuli_Chr03	13202989	(C/T)
3704	CakSNP3704	*Kabuli*	Ca_Kabuli_Chr03	13396925	(A/T)
3705	CakSNP3705	*Kabuli*	Ca_Kabuli_Chr03	13397207	(A/C)
3706	CakSNP3706	*Kabuli*	Ca_Kabuli_Chr03	13420776	(T/C)
3707	CakSNP3707	*Kabuli*	Ca_Kabuli_Chr03	13420758	(A/G)
3708	CakSNP3708	*Kabuli*	Ca_Kabuli_Chr03	13420756	(A/G)
3709	CakSNP3709	*Kabuli*	Ca_Kabuli_Chr03	13617948	(A/C)
3710	CakSNP3710	*Kabuli*	Ca_Kabuli_Chr03	14200381	(A/C)
3711	CakSNP3711	*Kabuli*	Ca_Kabuli_Chr03	14465236	(T/G)
3712	CakSNP3712	*Kabuli*	Ca_Kabuli_Chr03	14497374	(C/A)
3713	CakSNP3713	*Kabuli*	Ca_Kabuli_Chr03	14653195	(G/T)
3714	CakSNP3714	*Kabuli*	Ca_Kabuli_Chr03	14653194	(C/T)
3715	CakSNP3715	*Kabuli*	Ca_Kabuli_Chr03	14653193	(T/G)
3716	CakSNP3716	*Kabuli*	Ca_Kabuli_Chr03	14653270	(C/A)
3717	CakSNP3717	*Kabuli*	Ca_Kabuli_Chr03	14653271	(G/A)
3718	CakSNP3718	*Kabuli*	Ca_Kabuli_Chr03	14658175	(A/C)
3719	CakSNP3719	*Kabuli*	Ca_Kabuli_Chr03	14812960	(A/C)
3720	CakSNP3720	*Kabuli*	Ca_Kabuli_Chr03	14982667	(C/T)
3721	CakSNP3721	*Kabuli*	Ca_Kabuli_Chr03	14982739	(A/T)
3722	CakSNP3722	*Kabuli*	Ca_Kabuli_Chr03	15744906	(T/A)
3723	CakSNP3723	*Kabuli*	Ca_Kabuli_Chr03	15745010	(C/T)

S.N.	SNP IDs	Cultivars	Chromosomes/scaffolds	Physical positions (bp)	SNPs
3724	CakSNP3724	*Kabuli*	Ca_Kabuli_Chr03	15745017	(A/G)
3725	CakSNP3725	*Kabuli*	Ca_Kabuli_Chr03	15745264	(G/C)
3726	CakSNP3726	*Kabuli*	Ca_Kabuli_Chr03	15745311	(G/A)
3727	CakSNP3727	*Kabuli*	Ca_Kabuli_Chr03	15757804	(T/C)
3728	CakSNP3728	*Kabuli*	Ca_Kabuli_Chr03	15757829	(C/T)
3729	CakSNP3729	*Kabuli*	Ca_Kabuli_Chr03	15757852	(A/C)
3730	CakSNP3730	*Kabuli*	Ca_Kabuli_Chr03	16071411	(G/C)
3731	CakSNP3731	*Kabuli*	Ca_Kabuli_Chr03	16396662	(C/G)
3732	CakSNP3732	*Kabuli*	Ca_Kabuli_Chr03	16396673	(A/C)
3733	CakSNP3733	*Kabuli*	Ca_Kabuli_Chr03	16436286	(T/A)
3734	CakSNP3734	*Kabuli*	Ca_Kabuli_Chr03	16458123	(G/T)
3735	CakSNP3735	*Kabuli*	Ca_Kabuli_Chr03	16458413	(T/A)
3736	CakSNP3736	*Kabuli*	Ca_Kabuli_Chr03	16540328	(T/G)
3737	CakSNP3737	*Kabuli*	Ca_Kabuli_Chr03	16568567	(A/G)
3738	CakSNP3738	*Kabuli*	Ca_Kabuli_Chr03	16621498	(G/A)
3739	CakSNP3739	*Kabuli*	Ca_Kabuli_Chr03	16621624	(G/T)
3740	CakSNP3740	*Kabuli*	Ca_Kabuli_Chr03	16768867	(A/G)
3741	CakSNP3741	*Kabuli*	Ca_Kabuli_Chr03	16768936	(A/G)
3742	CakSNP3742	*Kabuli*	Ca_Kabuli_Chr03	16768917	(C/T)
3743	CakSNP3743	*Kabuli*	Ca_Kabuli_Chr03	16948510	(G/T)
3744	CakSNP3744	*Kabuli*	Ca_Kabuli_Chr03	16948854	(A/T)
3745	CakSNP3745	*Kabuli*	Ca_Kabuli_Chr03	16995243	(C/T)
3746	CakSNP3746	*Kabuli*	Ca_Kabuli_Chr03	16997328	(G/A)
3747	CakSNP3747	*Kabuli*	Ca_Kabuli_Chr03	16997331	(C/T)
3748	CakSNP3748	*Kabuli*	Ca_Kabuli_Chr03	16997400	(T/C)
3749	CakSNP3749	*Kabuli*	Ca_Kabuli_Chr03	17136071	(G/C)
3750	CakSNP3750	*Kabuli*	Ca_Kabuli_Chr03	17136097	(T/C)
3751	CakSNP3751	*Kabuli*	Ca_Kabuli_Chr03	17145639	(A/T)
3752	CakSNP3752	*Kabuli*	Ca_Kabuli_Chr03	17145643	(G/T)
3753	CakSNP3753	*Kabuli*	Ca_Kabuli_Chr03	17181213	(A/G)
3754	CakSNP3754	*Kabuli*	Ca_Kabuli_Chr03	17185682	(A/G)
3755	CakSNP3755	*Kabuli*	Ca_Kabuli_Chr03	17334924	(G/T)
3756	CakSNP3756	*Kabuli*	Ca_Kabuli_Chr03	17335851	(A/C)
3757	CakSNP3757	*Kabuli*	Ca_Kabuli_Chr03	17351553	(A/T)
3758	CakSNP3758	*Kabuli*	Ca_Kabuli_Chr03	17351526	(A/C)
3759	CakSNP3759	*Kabuli*	Ca_Kabuli_Chr03	17459986	(T/A)
3760	CakSNP3760	*Kabuli*	Ca_Kabuli_Chr03	17460002	(G/A)
3761	CakSNP3761	*Kabuli*	Ca_Kabuli_Chr03	17460012	(C/A)

S.N.	SNP IDs	Cultivars	Chromosomes/scaffolds	Physical positions (bp)	SNPs
3762	CakSNP3762	*Kabuli*	Ca_Kabuli_Chr03	17574247	(C/T)
3763	CakSNP3763	*Kabuli*	Ca_Kabuli_Chr03	17635591	(T/A)
3764	CakSNP3764	*Kabuli*	Ca_Kabuli_Chr03	17635531	(T/C)
3765	CakSNP3765	*Kabuli*	Ca_Kabuli_Chr03	17679409	(C/T)
3766	CakSNP3766	*Kabuli*	Ca_Kabuli_Chr03	17679404	(C/T)
3767	CakSNP3767	*Kabuli*	Ca_Kabuli_Chr03	17914677	(T/G)
3768	CakSNP3768	*Kabuli*	Ca_Kabuli_Chr03	17936321	(T/C)
3769	CakSNP3769	*Kabuli*	Ca_Kabuli_Chr03	17936729	(G/A)
3770	CakSNP3770	*Kabuli*	Ca_Kabuli_Chr03	17936842	(A/G)
3771	CakSNP3771	*Kabuli*	Ca_Kabuli_Chr03	17984978	(T/G)
3772	CakSNP3772	*Kabuli*	Ca_Kabuli_Chr03	17986521	(G/C)
3773	CakSNP3773	*Kabuli*	Ca_Kabuli_Chr03	17986525	(G/T)
3774	CakSNP3774	*Kabuli*	Ca_Kabuli_Chr03	17986740	(T/C)
3775	CakSNP3775	*Kabuli*	Ca_Kabuli_Chr03	18215321	(G/C)
3776	CakSNP3776	*Kabuli*	Ca_Kabuli_Chr03	18215332	(T/C)
3777	CakSNP3777	*Kabuli*	Ca_Kabuli_Chr03	18355427	(T/C)
3778	CakSNP3778	*Kabuli*	Ca_Kabuli_Chr03	18355445	(T/C)
3779	CakSNP3779	*Kabuli*	Ca_Kabuli_Chr03	18375547	(T/C)
3780	CakSNP3780	*Kabuli*	Ca_Kabuli_Chr03	18375581	(G/C)
3781	CakSNP3781	*Kabuli*	Ca_Kabuli_Chr03	18382404	(T/C)
3782	CakSNP3782	*Kabuli*	Ca_Kabuli_Chr03	18382438	(G/C)
3783	CakSNP3783	*Kabuli*	Ca_Kabuli_Chr03	18383884	(T/C)
3784	CakSNP3784	*Kabuli*	Ca_Kabuli_Chr03	18476006	(A/G)
3785	CakSNP3785	*Kabuli*	Ca_Kabuli_Chr03	18476065	(C/A)
3786	CakSNP3786	*Kabuli*	Ca_Kabuli_Chr03	18497633	(T/C)
3787	CakSNP3787	*Kabuli*	Ca_Kabuli_Chr03	18573701	(T/A)
3788	CakSNP3788	*Kabuli*	Ca_Kabuli_Chr03	18645114	(T/C)
3789	CakSNP3789	*Kabuli*	Ca_Kabuli_Chr03	18687994	(T/A)
3790	CakSNP3790	*Kabuli*	Ca_Kabuli_Chr03	18709398	(G/A)
3791	CakSNP3791	*Kabuli*	Ca_Kabuli_Chr03	18731098	(T/C)
3792	CakSNP3792	*Kabuli*	Ca_Kabuli_Chr03	18825708	(T/C)
3793	CakSNP3793	*Kabuli*	Ca_Kabuli_Chr03	18825687	(T/C)
3794	CakSNP3794	*Kabuli*	Ca_Kabuli_Chr03	18939425	(C/G)
3795	CakSNP3795	*Kabuli*	Ca_Kabuli_Chr03	19372722	(G/T)
3796	CakSNP3796	*Kabuli*	Ca_Kabuli_Chr03	19372739	(C/T)
3797	CakSNP3797	*Kabuli*	Ca_Kabuli_Chr03	19400104	(T/C)
3798	CakSNP3798	*Kabuli*	Ca_Kabuli_Chr03	19400121	(G/T)
3799	CakSNP3799	*Kabuli*	Ca_Kabuli_Chr03	19400276	(C/T)

S.N.	SNP IDs	Cultivars	Chromosomes/scaffolds	Physical positions (bp)	SNPs
3800	CakSNP3800	*Kabuli*	Ca_Kabuli_Chr03	19442839	(G/A)
3801	CakSNP3801	*Kabuli*	Ca_Kabuli_Chr03	19442884	(G/C)
3802	CakSNP3802	*Kabuli*	Ca_Kabuli_Chr03	19442941	(G/A)
3803	CakSNP3803	*Kabuli*	Ca_Kabuli_Chr03	19445925	(A/G)
3804	CakSNP3804	*Kabuli*	Ca_Kabuli_Chr03	19445920	(T/A)
3805	CakSNP3805	*Kabuli*	Ca_Kabuli_Chr03	19448140	(A/G)
3806	CakSNP3806	*Kabuli*	Ca_Kabuli_Chr03	19475095	(G/T)
3807	CakSNP3807	*Kabuli*	Ca_Kabuli_Chr03	19571396	(C/A)
3808	CakSNP3808	*Kabuli*	Ca_Kabuli_Chr03	19571382	(G/T)
3809	CakSNP3809	*Kabuli*	Ca_Kabuli_Chr03	19571371	(A/G)
3810	CakSNP3810	*Kabuli*	Ca_Kabuli_Chr03	19742600	(G/A)
3811	CakSNP3811	*Kabuli*	Ca_Kabuli_Chr03	19742530	(C/T)
3812	CakSNP3812	*Kabuli*	Ca_Kabuli_Chr03	19788512	(G/A)
3813	CakSNP3813	*Kabuli*	Ca_Kabuli_Chr03	19802763	(C/T)
3814	CakSNP3814	*Kabuli*	Ca_Kabuli_Chr03	19804985	(T/C)
3815	CakSNP3815	*Kabuli*	Ca_Kabuli_Chr03	19805063	(A/C)
3816	CakSNP3816	*Kabuli*	Ca_Kabuli_Chr03	19829510	(G/T)
3817	CakSNP3817	*Kabuli*	Ca_Kabuli_Chr03	19850532	(T/C)
3818	CakSNP3818	*Kabuli*	Ca_Kabuli_Chr03	19850589	(C/T)
3819	CakSNP3819	*Kabuli*	Ca_Kabuli_Chr03	19855217	(T/C)
3820	CakSNP3820	*Kabuli*	Ca_Kabuli_Chr03	19857613	(T/A)
3821	CakSNP3821	*Kabuli*	Ca_Kabuli_Chr03	19883898	(T/C)
3822	CakSNP3822	*Kabuli*	Ca_Kabuli_Chr03	19883927	(A/G)
3823	CakSNP3823	*Kabuli*	Ca_Kabuli_Chr03	19883936	(T/G)
3824	CakSNP3824	*Kabuli*	Ca_Kabuli_Chr03	19896800	(C/T)
3825	CakSNP3825	*Kabuli*	Ca_Kabuli_Chr03	19896849	(G/A)
3826	CakSNP3826	*Kabuli*	Ca_Kabuli_Chr03	19907774	(T/C)
3827	CakSNP3827	*Kabuli*	Ca_Kabuli_Chr03	19956343	(C/T)
3828	CakSNP3828	*Kabuli*	Ca_Kabuli_Chr03	19956391	(C/T)
3829	CakSNP3829	*Kabuli*	Ca_Kabuli_Chr03	19978343	(A/C)
3830	CakSNP3830	*Kabuli*	Ca_Kabuli_Chr03	19978334	(A/C)
3831	CakSNP3831	*Kabuli*	Ca_Kabuli_Chr03	19978407	(C/T)
3832	CakSNP3832	*Kabuli*	Ca_Kabuli_Chr03	19979288	(A/T)
3833	CakSNP3833	*Kabuli*	Ca_Kabuli_Chr03	19979318	(C/A)
3834	CakSNP3834	*Kabuli*	Ca_Kabuli_Chr03	19979390	(A/T)
3835	CakSNP3835	*Kabuli*	Ca_Kabuli_Chr03	20000967	(C/G)
3836	CakSNP3836	*Kabuli*	Ca_Kabuli_Chr03	20030262	(T/C)
3837	CakSNP3837	*Kabuli*	Ca_Kabuli_Chr03	20030298	(G/A)

S.N.	SNP IDs	Cultivars	Chromosomes/scaffolds	Physical positions (bp)	SNPs
3838	CakSNP3838	*Kabuli*	Ca_Kabuli_Chr03	20030484	(A/G)
3839	CakSNP3839	*Kabuli*	Ca_Kabuli_Chr03	20103039	(G/A)
3840	CakSNP3840	*Kabuli*	Ca_Kabuli_Chr03	20103060	(C/T)
3841	CakSNP3841	*Kabuli*	Ca_Kabuli_Chr03	20103126	(A/C)
3842	CakSNP3842	*Kabuli*	Ca_Kabuli_Chr03	20172492	(C/T)
3843	CakSNP3843	*Kabuli*	Ca_Kabuli_Chr03	20240118	(T/G)
3844	CakSNP3844	*Kabuli*	Ca_Kabuli_Chr03	20242663	(C/T)
3845	CakSNP3845	*Kabuli*	Ca_Kabuli_Chr03	20331659	(G/A)
3846	CakSNP3846	*Kabuli*	Ca_Kabuli_Chr03	20365442	(A/C)
3847	CakSNP3847	*Kabuli*	Ca_Kabuli_Chr03	20387399	(T/A)
3848	CakSNP3848	*Kabuli*	Ca_Kabuli_Chr03	20387471	(A/G)
3849	CakSNP3849	*Kabuli*	Ca_Kabuli_Chr03	20415660	(C/G)
3850	CakSNP3850	*Kabuli*	Ca_Kabuli_Chr03	20415615	(A/G)
3851	CakSNP3851	*Kabuli*	Ca_Kabuli_Chr03	20462715	(T/A)
3852	CakSNP3852	*Kabuli*	Ca_Kabuli_Chr03	20490488	(T/C)
3853	CakSNP3853	*Kabuli*	Ca_Kabuli_Chr03	20495810	(G/A)
3854	CakSNP3854	*Kabuli*	Ca_Kabuli_Chr03	20503939	(G/T)
3855	CakSNP3855	*Kabuli*	Ca_Kabuli_Chr03	20506663	(C/G)
3856	CakSNP3856	*Kabuli*	Ca_Kabuli_Chr03	20552836	(T/G)
3857	CakSNP3857	*Kabuli*	Ca_Kabuli_Chr03	20555942	(G/A)
3858	CakSNP3858	*Kabuli*	Ca_Kabuli_Chr03	20690207	(T/G)
3859	CakSNP3859	*Kabuli*	Ca_Kabuli_Chr03	20690185	(A/T)
3860	CakSNP3860	*Kabuli*	Ca_Kabuli_Chr03	20794236	(A/T)
3861	CakSNP3861	*Kabuli*	Ca_Kabuli_Chr03	20794206	(A/T)
3862	CakSNP3862	*Kabuli*	Ca_Kabuli_Chr03	20794984	(C/T)
3863	CakSNP3863	*Kabuli*	Ca_Kabuli_Chr03	20795019	(C/T)
3864	CakSNP3864	*Kabuli*	Ca_Kabuli_Chr03	20795014	(C/T)
3865	CakSNP3865	*Kabuli*	Ca_Kabuli_Chr03	20794976	(A/C)
3866	CakSNP3866	*Kabuli*	Ca_Kabuli_Chr03	20794993	(A/G)
3867	CakSNP3867	*Kabuli*	Ca_Kabuli_Chr03	20794990	(G/A)
3868	CakSNP3868	*Kabuli*	Ca_Kabuli_Chr03	20799767	(A/G)
3869	CakSNP3869	*Kabuli*	Ca_Kabuli_Chr03	20799778	(C/T)
3870	CakSNP3870	*Kabuli*	Ca_Kabuli_Chr03	20799811	(C/T)
3871	CakSNP3871	*Kabuli*	Ca_Kabuli_Chr03	20799860	(A/G)
3872	CakSNP3872	*Kabuli*	Ca_Kabuli_Chr03	20799841	(C/T)
3873	CakSNP3873	*Kabuli*	Ca_Kabuli_Chr03	20799819	(C/T)
3874	CakSNP3874	*Kabuli*	Ca_Kabuli_Chr03	20799820	(A/G)
3875	CakSNP3875	*Kabuli*	Ca_Kabuli_Chr03	20822676	(T/C)

S.N.	SNP IDs	Cultivars	Chromosomes/scaffolds	Physical positions (bp)	SNPs
3876	CakSNP3876	*Kabuli*	Ca_Kabuli_Chr03	20823146	(C/T)
3877	CakSNP3877	*Kabuli*	Ca_Kabuli_Chr03	20828272	(G/T)
3878	CakSNP3878	*Kabuli*	Ca_Kabuli_Chr03	20829399	(A/G)
3879	CakSNP3879	*Kabuli*	Ca_Kabuli_Chr03	20829407	(T/G)
3880	CakSNP3880	*Kabuli*	Ca_Kabuli_Chr03	20841298	(T/G)
3881	CakSNP3881	*Kabuli*	Ca_Kabuli_Chr03	20841352	(C/T)
3882	CakSNP3882	*Kabuli*	Ca_Kabuli_Chr03	20880294	(T/A)
3883	CakSNP3883	*Kabuli*	Ca_Kabuli_Chr03	21020182	(A/G)
3884	CakSNP3884	*Kabuli*	Ca_Kabuli_Chr03	21020191	(C/T)
3885	CakSNP3885	*Kabuli*	Ca_Kabuli_Chr03	21069672	(A/G)
3886	CakSNP3886	*Kabuli*	Ca_Kabuli_Chr03	21069714	(G/A)
3887	CakSNP3887	*Kabuli*	Ca_Kabuli_Chr03	21069916	(C/T)
3888	CakSNP3888	*Kabuli*	Ca_Kabuli_Chr03	21069917	(G/A)
3889	CakSNP3889	*Kabuli*	Ca_Kabuli_Chr03	21069938	(C/A)
3890	CakSNP3890	*Kabuli*	Ca_Kabuli_Chr03	21069959	(G/A)
3891	CakSNP3891	*Kabuli*	Ca_Kabuli_Chr03	21069986	(A/C)
3892	CakSNP3892	*Kabuli*	Ca_Kabuli_Chr03	21127162	(C/G)
3893	CakSNP3893	*Kabuli*	Ca_Kabuli_Chr03	21127156	(T/G)
3894	CakSNP3894	*Kabuli*	Ca_Kabuli_Chr03	21198607	(A/T)
3895	CakSNP3895	*Kabuli*	Ca_Kabuli_Chr03	21210002	(T/A)
3896	CakSNP3896	*Kabuli*	Ca_Kabuli_Chr03	21210005	(C/A)
3897	CakSNP3897	*Kabuli*	Ca_Kabuli_Chr03	21210008	(T/C)
3898	CakSNP3898	*Kabuli*	Ca_Kabuli_Chr03	21210049	(A/G)
3899	CakSNP3899	*Kabuli*	Ca_Kabuli_Chr03	21273368	(A/G)
3900	CakSNP3900	*Kabuli*	Ca_Kabuli_Chr03	21288626	(C/A)
3901	CakSNP3901	*Kabuli*	Ca_Kabuli_Chr03	21288652	(G/C)
3902	CakSNP3902	*Kabuli*	Ca_Kabuli_Chr03	21288695	(G/A)
3903	CakSNP3903	*Kabuli*	Ca_Kabuli_Chr03	21335892	(C/T)
3904	CakSNP3904	*Kabuli*	Ca_Kabuli_Chr03	21382790	(G/A)
3905	CakSNP3905	*Kabuli*	Ca_Kabuli_Chr03	21421082	(A/G)
3906	CakSNP3906	*Kabuli*	Ca_Kabuli_Chr03	21421085	(G/A)
3907	CakSNP3907	*Kabuli*	Ca_Kabuli_Chr03	21421146	(C/A)
3908	CakSNP3908	*Kabuli*	Ca_Kabuli_Chr03	21421162	(G/A)
3909	CakSNP3909	*Kabuli*	Ca_Kabuli_Chr03	21421358	(G/A)
3910	CakSNP3910	*Kabuli*	Ca_Kabuli_Chr03	21421362	(T/A)
3911	CakSNP3911	*Kabuli*	Ca_Kabuli_Chr03	21421369	(G/A)
3912	CakSNP3912	*Kabuli*	Ca_Kabuli_Chr03	21421424	(G/A)
3913	CakSNP3913	*Kabuli*	Ca_Kabuli_Chr03	21421440	(C/T)

S.N.	SNP IDs	Cultivars	Chromosomes/scaffolds	Physical positions (bp)	SNPs
3914	CakSNP3914	*Kabuli*	Ca_Kabuli_Chr03	21450650	(A/G)
3915	CakSNP3915	*Kabuli*	Ca_Kabuli_Chr03	21452017	(G/A)
3916	CakSNP3916	*Kabuli*	Ca_Kabuli_Chr03	21468634	(G/A)
3917	CakSNP3917	*Kabuli*	Ca_Kabuli_Chr03	21468692	(A/C)
3918	CakSNP3918	*Kabuli*	Ca_Kabuli_Chr03	21470784	(C/T)
3919	CakSNP3919	*Kabuli*	Ca_Kabuli_Chr03	21500178	(G/A)
3920	CakSNP3920	*Kabuli*	Ca_Kabuli_Chr03	21500179	(A/T)
3921	CakSNP3921	*Kabuli*	Ca_Kabuli_Chr03	21507422	(T/C)
3922	CakSNP3922	*Kabuli*	Ca_Kabuli_Chr03	21524084	(T/C)
3923	CakSNP3923	*Kabuli*	Ca_Kabuli_Chr03	21524192	(T/C)
3924	CakSNP3924	*Kabuli*	Ca_Kabuli_Chr03	21524296	(G/T)
3925	CakSNP3925	*Kabuli*	Ca_Kabuli_Chr03	21524379	(A/G)
3926	CakSNP3926	*Kabuli*	Ca_Kabuli_Chr03	21525184	(C/T)
3927	CakSNP3927	*Kabuli*	Ca_Kabuli_Chr03	21525177	(G/A)
3928	CakSNP3928	*Kabuli*	Ca_Kabuli_Chr03	21558388	(A/G)
3929	CakSNP3929	*Kabuli*	Ca_Kabuli_Chr03	21558357	(T/G)
3930	CakSNP3930	*Kabuli*	Ca_Kabuli_Chr03	21627610	(T/C)
3931	CakSNP3931	*Kabuli*	Ca_Kabuli_Chr03	21634275	(A/C)
3932	CakSNP3932	*Kabuli*	Ca_Kabuli_Chr03	21634271	(A/G)
3933	CakSNP3933	*Kabuli*	Ca_Kabuli_Chr03	21646863	(C/T)
3934	CakSNP3934	*Kabuli*	Ca_Kabuli_Chr03	21646872	(T/C)
3935	CakSNP3935	*Kabuli*	Ca_Kabuli_Chr03	21646893	(C/A)
3936	CakSNP3936	*Kabuli*	Ca_Kabuli_Chr03	21646965	(C/T)
3937	CakSNP3937	*Kabuli*	Ca_Kabuli_Chr03	21646962	(A/G)
3938	CakSNP3938	*Kabuli*	Ca_Kabuli_Chr03	21654592	(T/A)
3939	CakSNP3939	*Kabuli*	Ca_Kabuli_Chr03	21686470	(C/T)
3940	CakSNP3940	*Kabuli*	Ca_Kabuli_Chr03	21687439	(G/T)
3941	CakSNP3941	*Kabuli*	Ca_Kabuli_Chr03	21687966	(C/A)
3942	CakSNP3942	*Kabuli*	Ca_Kabuli_Chr03	21687961	(C/A)
3943	CakSNP3943	*Kabuli*	Ca_Kabuli_Chr03	21687960	(G/A)
3944	CakSNP3944	*Kabuli*	Ca_Kabuli_Chr03	21687959	(T/C)
3945	CakSNP3945	*Kabuli*	Ca_Kabuli_Chr03	21687956	(C/T)
3946	CakSNP3946	*Kabuli*	Ca_Kabuli_Chr03	21687953	(C/T)
3947	CakSNP3947	*Kabuli*	Ca_Kabuli_Chr03	21741914	(C/A)
3948	CakSNP3948	*Kabuli*	Ca_Kabuli_Chr03	21809395	(G/A)
3949	CakSNP3949	*Kabuli*	Ca_Kabuli_Chr03	21809456	(T/C)
3950	CakSNP3950	*Kabuli*	Ca_Kabuli_Chr03	21809457	(G/A)
3951	CakSNP3951	*Kabuli*	Ca_Kabuli_Chr03	21809546	(T/A)

S.N.	SNP IDs	Cultivars	Chromosomes/scaffolds	Physical positions (bp)	SNPs
3952	CakSNP3952	*Kabuli*	Ca_Kabuli_Chr03	21809505	(A/G)
3953	CakSNP3953	*Kabuli*	Ca_Kabuli_Chr03	21809503	(T/C)
3954	CakSNP3954	*Kabuli*	Ca_Kabuli_Chr03	21950053	(C/G)
3955	CakSNP3955	*Kabuli*	Ca_Kabuli_Chr03	21950186	(A/G)
3956	CakSNP3956	*Kabuli*	Ca_Kabuli_Chr03	21950164	(A/G)
3957	CakSNP3957	*Kabuli*	Ca_Kabuli_Chr03	22008087	(G/A)
3958	CakSNP3958	*Kabuli*	Ca_Kabuli_Chr03	22026111	(A/G)
3959	CakSNP3959	*Kabuli*	Ca_Kabuli_Chr03	22026647	(A/G)
3960	CakSNP3960	*Kabuli*	Ca_Kabuli_Chr03	22060199	(G/T)
3961	CakSNP3961	*Kabuli*	Ca_Kabuli_Chr03	22061236	(A/G)
3962	CakSNP3962	*Kabuli*	Ca_Kabuli_Chr03	22062029	(G/T)
3963	CakSNP3963	*Kabuli*	Ca_Kabuli_Chr03	22062958	(T/C)
3964	CakSNP3964	*Kabuli*	Ca_Kabuli_Chr03	22062965	(C/T)
3965	CakSNP3965	*Kabuli*	Ca_Kabuli_Chr03	22063826	(G/A)
3966	CakSNP3966	*Kabuli*	Ca_Kabuli_Chr03	22063829	(G/A)
3967	CakSNP3967	*Kabuli*	Ca_Kabuli_Chr03	22063835	(C/T)
3968	CakSNP3968	*Kabuli*	Ca_Kabuli_Chr03	22073381	(C/T)
3969	CakSNP3969	*Kabuli*	Ca_Kabuli_Chr03	22073447	(T/A)
3970	CakSNP3970	*Kabuli*	Ca_Kabuli_Chr03	22075779	(C/G)
3971	CakSNP3971	*Kabuli*	Ca_Kabuli_Chr03	22075990	(T/C)
3972	CakSNP3972	*Kabuli*	Ca_Kabuli_Chr03	22169816	(T/C)
3973	CakSNP3973	*Kabuli*	Ca_Kabuli_Chr03	22188124	(T/C)
3974	CakSNP3974	*Kabuli*	Ca_Kabuli_Chr03	22188169	(T/A)
3975	CakSNP3975	*Kabuli*	Ca_Kabuli_Chr03	22200927	(T/C)
3976	CakSNP3976	*Kabuli*	Ca_Kabuli_Chr03	22201045	(T/A)
3977	CakSNP3977	*Kabuli*	Ca_Kabuli_Chr03	22202317	(C/T)
3978	CakSNP3978	*Kabuli*	Ca_Kabuli_Chr03	22202819	(T/C)
3979	CakSNP3979	*Kabuli*	Ca_Kabuli_Chr03	22203460	(A/G)
3980	CakSNP3980	*Kabuli*	Ca_Kabuli_Chr03	22225186	(C/A)
3981	CakSNP3981	*Kabuli*	Ca_Kabuli_Chr03	22225154	(C/G)
3982	CakSNP3982	*Kabuli*	Ca_Kabuli_Chr03	22225129	(C/G)
3983	CakSNP3983	*Kabuli*	Ca_Kabuli_Chr03	22233428	(G/A)
3984	CakSNP3984	*Kabuli*	Ca_Kabuli_Chr03	22233409	(T/C)
3985	CakSNP3985	*Kabuli*	Ca_Kabuli_Chr03	22235801	(G/A)
3986	CakSNP3986	*Kabuli*	Ca_Kabuli_Chr03	22253842	(T/C)
3987	CakSNP3987	*Kabuli*	Ca_Kabuli_Chr03	22331057	(C/T)
3988	CakSNP3988	*Kabuli*	Ca_Kabuli_Chr03	22346294	(T/C)
3989	CakSNP3989	*Kabuli*	Ca_Kabuli_Chr03	22404660	(G/T)

S.N.	SNP IDs	Cultivars	Chromosomes/scaffolds	Physical positions (bp)	SNPs
3990	CakSNP3990	*Kabuli*	Ca_Kabuli_Chr03	22527529	(A/C)
3991	CakSNP3991	*Kabuli*	Ca_Kabuli_Chr03	22539384	(G/A)
3992	CakSNP3992	*Kabuli*	Ca_Kabuli_Chr03	22539702	(C/T)
3993	CakSNP3993	*Kabuli*	Ca_Kabuli_Chr03	22539885	(C/T)
3994	CakSNP3994	*Kabuli*	Ca_Kabuli_Chr03	22539952	(G/A)
3995	CakSNP3995	*Kabuli*	Ca_Kabuli_Chr03	22636201	(T/C)
3996	CakSNP3996	*Kabuli*	Ca_Kabuli_Chr03	22646897	(T/C)
3997	CakSNP3997	*Kabuli*	Ca_Kabuli_Chr03	22659924	(G/A)
3998	CakSNP3998	*Kabuli*	Ca_Kabuli_Chr03	22681658	(G/A)
3999	CakSNP3999	*Kabuli*	Ca_Kabuli_Chr03	22719470	(T/C)
4000	CakSNP4000	*Kabuli*	Ca_Kabuli_Chr03	22719487	(T/C)
4001	CakSNP4001	*Kabuli*	Ca_Kabuli_Chr03	22719494	(C/T)
4002	CakSNP4002	*Kabuli*	Ca_Kabuli_Chr03	22719519	(G/A)
4003	CakSNP4003	*Kabuli*	Ca_Kabuli_Chr03	22727393	(G/A)
4004	CakSNP4004	*Kabuli*	Ca_Kabuli_Chr03	22727439	(A/C)
4005	CakSNP4005	*Kabuli*	Ca_Kabuli_Chr03	22765806	(G/A)
4006	CakSNP4006	*Kabuli*	Ca_Kabuli_Chr03	22765862	(T/C)
4007	CakSNP4007	*Kabuli*	Ca_Kabuli_Chr03	22765987	(G/T)
4008	CakSNP4008	*Kabuli*	Ca_Kabuli_Chr03	22798281	(T/G)
4009	CakSNP4009	*Kabuli*	Ca_Kabuli_Chr03	22812717	(A/T)
4010	CakSNP4010	*Kabuli*	Ca_Kabuli_Chr03	22846051	(G/A)
4011	CakSNP4011	*Kabuli*	Ca_Kabuli_Chr03	22847885	(G/T)
4012	CakSNP4012	*Kabuli*	Ca_Kabuli_Chr03	22847984	(A/G)
4013	CakSNP4013	*Kabuli*	Ca_Kabuli_Chr03	22990523	(C/T)
4014	CakSNP4014	*Kabuli*	Ca_Kabuli_Chr03	23028855	(A/G)
4015	CakSNP4015	*Kabuli*	Ca_Kabuli_Chr03	23033373	(A/C)
4016	CakSNP4016	*Kabuli*	Ca_Kabuli_Chr03	23042249	(A/C)
4017	CakSNP4017	*Kabuli*	Ca_Kabuli_Chr03	23053008	(C/G)
4018	CakSNP4018	*Kabuli*	Ca_Kabuli_Chr03	23053005	(G/C)
4019	CakSNP4019	*Kabuli*	Ca_Kabuli_Chr03	23053066	(A/C)
4020	CakSNP4020	*Kabuli*	Ca_Kabuli_Chr03	23095498	(C/T)
4021	CakSNP4021	*Kabuli*	Ca_Kabuli_Chr03	23273057	(T/A)
4022	CakSNP4022	*Kabuli*	Ca_Kabuli_Chr03	23273069	(C/G)
4023	CakSNP4023	*Kabuli*	Ca_Kabuli_Chr03	23327648	(C/T)
4024	CakSNP4024	*Kabuli*	Ca_Kabuli_Chr03	23327700	(G/C)
4025	CakSNP4025	*Kabuli*	Ca_Kabuli_Chr03	23331530	(T/G)
4026	CakSNP4026	*Kabuli*	Ca_Kabuli_Chr03	23335788	(A/T)
4027	CakSNP4027	*Kabuli*	Ca_Kabuli_Chr03	23335789	(T/G)

S.N.	SNP IDs	Cultivars	Chromosomes/scaffolds	Physical positions (bp)	SNPs
4028	CakSNP4028	*Kabuli*	Ca_Kabuli_Chr03	23340171	(A/G)
4029	CakSNP4029	*Kabuli*	Ca_Kabuli_Chr03	23340134	(G/A)
4030	CakSNP4030	*Kabuli*	Ca_Kabuli_Chr03	23340133	(T/C)
4031	CakSNP4031	*Kabuli*	Ca_Kabuli_Chr03	23341384	(T/G)
4032	CakSNP4032	*Kabuli*	Ca_Kabuli_Chr03	23342297	(T/C)
4033	CakSNP4033	*Kabuli*	Ca_Kabuli_Chr03	23342305	(T/C)
4034	CakSNP4034	*Kabuli*	Ca_Kabuli_Chr03	23342338	(G/T)
4035	CakSNP4035	*Kabuli*	Ca_Kabuli_Chr03	23342347	(G/A)
4036	CakSNP4036	*Kabuli*	Ca_Kabuli_Chr03	23342350	(A/G)
4037	CakSNP4037	*Kabuli*	Ca_Kabuli_Chr03	23342351	(T/C)
4038	CakSNP4038	*Kabuli*	Ca_Kabuli_Chr03	23360857	(C/T)
4039	CakSNP4039	*Kabuli*	Ca_Kabuli_Chr03	23400864	(A/C)
4040	CakSNP4040	*Kabuli*	Ca_Kabuli_Chr03	23455008	(A/G)
4041	CakSNP4041	*Kabuli*	Ca_Kabuli_Chr03	23455128	(A/G)
4042	CakSNP4042	*Kabuli*	Ca_Kabuli_Chr03	23461370	(C/A)
4043	CakSNP4043	*Kabuli*	Ca_Kabuli_Chr03	23555586	(C/A)
4044	CakSNP4044	*Kabuli*	Ca_Kabuli_Chr03	23593288	(G/A)
4045	CakSNP4045	*Kabuli*	Ca_Kabuli_Chr03	23593294	(T/C)
4046	CakSNP4046	*Kabuli*	Ca_Kabuli_Chr03	23593309	(T/C)
4047	CakSNP4047	*Kabuli*	Ca_Kabuli_Chr03	23593269	(T/C)
4048	CakSNP4048	*Kabuli*	Ca_Kabuli_Chr03	23714158	(T/A)
4049	CakSNP4049	*Kabuli*	Ca_Kabuli_Chr03	23714129	(C/A)
4050	CakSNP4050	*Kabuli*	Ca_Kabuli_Chr03	23745324	(C/T)
4051	CakSNP4051	*Kabuli*	Ca_Kabuli_Chr03	23792975	(A/T)
4052	CakSNP4052	*Kabuli*	Ca_Kabuli_Chr03	23833683	(G/A)
4053	CakSNP4053	*Kabuli*	Ca_Kabuli_Chr03	23833680	(G/C)
4054	CakSNP4054	*Kabuli*	Ca_Kabuli_Chr03	23833668	(G/A)
4055	CakSNP4055	*Kabuli*	Ca_Kabuli_Chr03	23886582	(A/G)
4056	CakSNP4056	*Kabuli*	Ca_Kabuli_Chr03	23918105	(T/C)
4057	CakSNP4057	*Kabuli*	Ca_Kabuli_Chr03	23966110	(G/A)
4058	CakSNP4058	*Kabuli*	Ca_Kabuli_Chr03	23997287	(A/T)
4059	CakSNP4059	*Kabuli*	Ca_Kabuli_Chr03	24000847	(G/T)
4060	CakSNP4060	*Kabuli*	Ca_Kabuli_Chr03	24000818	(T/C)
4061	CakSNP4061	*Kabuli*	Ca_Kabuli_Chr03	24024560	(T/C)
4062	CakSNP4062	*Kabuli*	Ca_Kabuli_Chr03	24024513	(A/C)
4063	CakSNP4063	*Kabuli*	Ca_Kabuli_Chr03	24025900	(G/C)
4064	CakSNP4064	*Kabuli*	Ca_Kabuli_Chr03	24025924	(T/G)
4065	CakSNP4065	*Kabuli*	Ca_Kabuli_Chr03	24026428	(C/T)

S.N.	SNP IDs	Cultivars	Chromosomes/scaffolds	Physical positions (bp)	SNPs
4066	CakSNP4066	*Kabuli*	Ca_Kabuli_Chr03	24041806	(C/T)
4067	CakSNP4067	*Kabuli*	Ca_Kabuli_Chr03	24095268	(T/C)
4068	CakSNP4068	*Kabuli*	Ca_Kabuli_Chr03	24099191	(C/G)
4069	CakSNP4069	*Kabuli*	Ca_Kabuli_Chr03	24127222	(C/T)
4070	CakSNP4070	*Kabuli*	Ca_Kabuli_Chr03	24127201	(A/C)
4071	CakSNP4071	*Kabuli*	Ca_Kabuli_Chr03	24162153	(C/T)
4072	CakSNP4072	*Kabuli*	Ca_Kabuli_Chr03	24194574	(C/T)
4073	CakSNP4073	*Kabuli*	Ca_Kabuli_Chr03	24247161	(G/C)
4074	CakSNP4074	*Kabuli*	Ca_Kabuli_Chr03	24297842	(T/G)
4075	CakSNP4075	*Kabuli*	Ca_Kabuli_Chr03	24297894	(C/A)
4076	CakSNP4076	*Kabuli*	Ca_Kabuli_Chr03	24417477	(T/G)
4077	CakSNP4077	*Kabuli*	Ca_Kabuli_Chr03	24417507	(A/T)
4078	CakSNP4078	*Kabuli*	Ca_Kabuli_Chr03	24418063	(G/T)
4079	CakSNP4079	*Kabuli*	Ca_Kabuli_Chr03	24418111	(A/G)
4080	CakSNP4080	*Kabuli*	Ca_Kabuli_Chr03	24418130	(T/A)
4081	CakSNP4081	*Kabuli*	Ca_Kabuli_Chr03	24459669	(G/A)
4082	CakSNP4082	*Kabuli*	Ca_Kabuli_Chr03	24515489	(T/G)
4083	CakSNP4083	*Kabuli*	Ca_Kabuli_Chr03	24538726	(A/C)
4084	CakSNP4084	*Kabuli*	Ca_Kabuli_Chr03	24540199	(A/G)
4085	CakSNP4085	*Kabuli*	Ca_Kabuli_Chr03	24540304	(T/C)
4086	CakSNP4086	*Kabuli*	Ca_Kabuli_Chr03	24637834	(G/T)
4087	CakSNP4087	*Kabuli*	Ca_Kabuli_Chr03	24637837	(A/C)
4088	CakSNP4088	*Kabuli*	Ca_Kabuli_Chr03	24664582	(G/T)
4089	CakSNP4089	*Kabuli*	Ca_Kabuli_Chr03	24664550	(T/G)
4090	CakSNP4090	*Kabuli*	Ca_Kabuli_Chr03	24664651	(G/T)
4091	CakSNP4091	*Kabuli*	Ca_Kabuli_Chr03	24666594	(T/C)
4092	CakSNP4092	*Kabuli*	Ca_Kabuli_Chr03	24763730	(A/G)
4093	CakSNP4093	*Kabuli*	Ca_Kabuli_Chr03	24933940	(T/C)
4094	CakSNP4094	*Kabuli*	Ca_Kabuli_Chr03	24933901	(C/A)
4095	CakSNP4095	*Kabuli*	Ca_Kabuli_Chr03	24933899	(G/T)
4096	CakSNP4096	*Kabuli*	Ca_Kabuli_Chr03	24998316	(C/G)
4097	CakSNP4097	*Kabuli*	Ca_Kabuli_Chr03	24998300	(C/T)
4098	CakSNP4098	*Kabuli*	Ca_Kabuli_Chr03	25037746	(G/A)
4099	CakSNP4099	*Kabuli*	Ca_Kabuli_Chr03	25046423	(C/G)
4100	CakSNP4100	*Kabuli*	Ca_Kabuli_Chr03	25046421	(T/C)
4101	CakSNP4101	*Kabuli*	Ca_Kabuli_Chr03	25055049	(C/T)
4102	CakSNP4102	*Kabuli*	Ca_Kabuli_Chr03	25062250	(C/A)
4103	CakSNP4103	*Kabuli*	Ca_Kabuli_Chr03	25110037	(T/G)

S.N.	SNP IDs	Cultivars	Chromosomes/scaffolds	Physical positions (bp)	SNPs
4104	CakSNP4104	*Kabuli*	Ca_Kabuli_Chr03	25137192	(T/A)
4105	CakSNP4105	*Kabuli*	Ca_Kabuli_Chr03	25147096	(G/A)
4106	CakSNP4106	*Kabuli*	Ca_Kabuli_Chr03	25205548	(C/A)
4107	CakSNP4107	*Kabuli*	Ca_Kabuli_Chr03	25241115	(G/A)
4108	CakSNP4108	*Kabuli*	Ca_Kabuli_Chr03	25244194	(A/C)
4109	CakSNP4109	*Kabuli*	Ca_Kabuli_Chr03	25270592	(C/G)
4110	CakSNP4110	*Kabuli*	Ca_Kabuli_Chr03	25399911	(C/G)
4111	CakSNP4111	*Kabuli*	Ca_Kabuli_Chr03	25457474	(T/A)
4112	CakSNP4112	*Kabuli*	Ca_Kabuli_Chr03	25457494	(A/T)
4113	CakSNP4113	*Kabuli*	Ca_Kabuli_Chr03	25457514	(C/A)
4114	CakSNP4114	*Kabuli*	Ca_Kabuli_Chr03	25462227	(A/C)
4115	CakSNP4115	*Kabuli*	Ca_Kabuli_Chr03	25504708	(A/G)
4116	CakSNP4116	*Kabuli*	Ca_Kabuli_Chr03	25505438	(C/T)
4117	CakSNP4117	*Kabuli*	Ca_Kabuli_Chr03	25505420	(G/T)
4118	CakSNP4118	*Kabuli*	Ca_Kabuli_Chr03	25534044	(C/T)
4119	CakSNP4119	*Kabuli*	Ca_Kabuli_Chr03	25538867	(T/C)
4120	CakSNP4120	*Kabuli*	Ca_Kabuli_Chr03	25580072	(A/T)
4121	CakSNP4121	*Kabuli*	Ca_Kabuli_Chr03	25614515	(T/G)
4122	CakSNP4122	*Kabuli*	Ca_Kabuli_Chr03	25641434	(C/A)
4123	CakSNP4123	*Kabuli*	Ca_Kabuli_Chr03	25641433	(G/A)
4124	CakSNP4124	*Kabuli*	Ca_Kabuli_Chr03	25688341	(G/A)
4125	CakSNP4125	*Kabuli*	Ca_Kabuli_Chr03	25702238	(G/A)
4126	CakSNP4126	*Kabuli*	Ca_Kabuli_Chr03	25718829	(C/A)
4127	CakSNP4127	*Kabuli*	Ca_Kabuli_Chr03	25718827	(T/A)
4128	CakSNP4128	*Kabuli*	Ca_Kabuli_Chr03	25757693	(C/T)
4129	CakSNP4129	*Kabuli*	Ca_Kabuli_Chr03	25987920	(T/G)
4130	CakSNP4130	*Kabuli*	Ca_Kabuli_Chr03	26119425	(T/G)
4131	CakSNP4131	*Kabuli*	Ca_Kabuli_Chr03	26119461	(C/T)
4132	CakSNP4132	*Kabuli*	Ca_Kabuli_Chr03	26163449	(G/A)
4133	CakSNP4133	*Kabuli*	Ca_Kabuli_Chr03	26178592	(G/A)
4134	CakSNP4134	*Kabuli*	Ca_Kabuli_Chr03	26198368	(G/A)
4135	CakSNP4135	*Kabuli*	Ca_Kabuli_Chr03	26198372	(G/A)
4136	CakSNP4136	*Kabuli*	Ca_Kabuli_Chr03	26293846	(A/G)
4137	CakSNP4137	*Kabuli*	Ca_Kabuli_Chr03	26335889	(T/A)
4138	CakSNP4138	*Kabuli*	Ca_Kabuli_Chr03	26339286	(T/C)
4139	CakSNP4139	*Kabuli*	Ca_Kabuli_Chr03	26339449	(G/T)
4140	CakSNP4140	*Kabuli*	Ca_Kabuli_Chr03	26562004	(A/G)
4141	CakSNP4141	*Kabuli*	Ca_Kabuli_Chr03	26573902	(A/G)

S.N.	SNP IDs	Cultivars	Chromosomes/scaffolds	Physical positions (bp)	SNPs
4142	CakSNP4142	*Kabuli*	Ca_Kabuli_Chr03	26574056	(C/T)
4143	CakSNP4143	*Kabuli*	Ca_Kabuli_Chr03	26574068	(T/G)
4144	CakSNP4144	*Kabuli*	Ca_Kabuli_Chr03	26574831	(A/G)
4145	CakSNP4145	*Kabuli*	Ca_Kabuli_Chr03	26589678	(T/A)
4146	CakSNP4146	*Kabuli*	Ca_Kabuli_Chr03	26627150	(T/G)
4147	CakSNP4147	*Kabuli*	Ca_Kabuli_Chr03	26636553	(A/G)
4148	CakSNP4148	*Kabuli*	Ca_Kabuli_Chr03	26646152	(C/T)
4149	CakSNP4149	*Kabuli*	Ca_Kabuli_Chr03	26678343	(T/C)
4150	CakSNP4150	*Kabuli*	Ca_Kabuli_Chr03	26678433	(T/G)
4151	CakSNP4151	*Kabuli*	Ca_Kabuli_Chr03	26726897	(C/A)
4152	CakSNP4152	*Kabuli*	Ca_Kabuli_Chr03	26758452	(C/A)
4153	CakSNP4153	*Kabuli*	Ca_Kabuli_Chr03	26848870	(G/C)
4154	CakSNP4154	*Kabuli*	Ca_Kabuli_Chr03	26849288	(T/A)
4155	CakSNP4155	*Kabuli*	Ca_Kabuli_Chr03	26878028	(A/T)
4156	CakSNP4156	*Kabuli*	Ca_Kabuli_Chr03	26900271	(T/A)
4157	CakSNP4157	*Kabuli*	Ca_Kabuli_Chr03	26911443	(A/T)
4158	CakSNP4158	*Kabuli*	Ca_Kabuli_Chr03	26911514	(A/G)
4159	CakSNP4159	*Kabuli*	Ca_Kabuli_Chr03	26911723	(T/A)
4160	CakSNP4160	*Kabuli*	Ca_Kabuli_Chr03	26911792	(A/G)
4161	CakSNP4161	*Kabuli*	Ca_Kabuli_Chr03	26976249	(A/G)
4162	CakSNP4162	*Kabuli*	Ca_Kabuli_Chr03	26976323	(G/A)
4163	CakSNP4163	*Kabuli*	Ca_Kabuli_Chr03	27024215	(T/C)
4164	CakSNP4164	*Kabuli*	Ca_Kabuli_Chr03	27047840	(A/G)
4165	CakSNP4165	*Kabuli*	Ca_Kabuli_Chr03	27047847	(A/C)
4166	CakSNP4166	*Kabuli*	Ca_Kabuli_Chr03	27063150	(A/C)
4167	CakSNP4167	*Kabuli*	Ca_Kabuli_Chr03	27074842	(C/A)
4168	CakSNP4168	*Kabuli*	Ca_Kabuli_Chr03	27074896	(A/T)
4169	CakSNP4169	*Kabuli*	Ca_Kabuli_Chr03	27074931	(A/T)
4170	CakSNP4170	*Kabuli*	Ca_Kabuli_Chr03	27074930	(A/C)
4171	CakSNP4171	*Kabuli*	Ca_Kabuli_Chr03	27076716	(T/C)
4172	CakSNP4172	*Kabuli*	Ca_Kabuli_Chr03	27076735	(T/G)
4173	CakSNP4173	*Kabuli*	Ca_Kabuli_Chr03	27076744	(C/A)
4174	CakSNP4174	*Kabuli*	Ca_Kabuli_Chr03	27105637	(C/T)
4175	CakSNP4175	*Kabuli*	Ca_Kabuli_Chr03	27157840	(T/A)
4176	CakSNP4176	*Kabuli*	Ca_Kabuli_Chr03	27157848	(C/T)
4177	CakSNP4177	*Kabuli*	Ca_Kabuli_Chr03	27161813	(T/G)
4178	CakSNP4178	*Kabuli*	Ca_Kabuli_Chr03	27161801	(A/G)
4179	CakSNP4179	*Kabuli*	Ca_Kabuli_Chr03	27194033	(C/T)

S.N.	SNP IDs	Cultivars	Chromosomes/scaffolds	Physical positions (bp)	SNPs
4180	CakSNP4180	*Kabuli*	Ca_Kabuli_Chr03	27194069	(T/C)
4181	CakSNP4181	*Kabuli*	Ca_Kabuli_Chr03	27338521	(T/C)
4182	CakSNP4182	*Kabuli*	Ca_Kabuli_Chr03	27342919	(G/A)
4183	CakSNP4183	*Kabuli*	Ca_Kabuli_Chr03	27391199	(G/A)
4184	CakSNP4184	*Kabuli*	Ca_Kabuli_Chr03	27391354	(G/A)
4185	CakSNP4185	*Kabuli*	Ca_Kabuli_Chr03	27391321	(A/G)
4186	CakSNP4186	*Kabuli*	Ca_Kabuli_Chr03	27417561	(A/T)
4187	CakSNP4187	*Kabuli*	Ca_Kabuli_Chr03	27420276	(T/C)
4188	CakSNP4188	*Kabuli*	Ca_Kabuli_Chr03	27420330	(C/T)
4189	CakSNP4189	*Kabuli*	Ca_Kabuli_Chr03	27435210	(T/C)
4190	CakSNP4190	*Kabuli*	Ca_Kabuli_Chr03	27435209	(G/C)
4191	CakSNP4191	*Kabuli*	Ca_Kabuli_Chr03	27435198	(T/A)
4192	CakSNP4192	*Kabuli*	Ca_Kabuli_Chr03	27447685	(C/A)
4193	CakSNP4193	*Kabuli*	Ca_Kabuli_Chr03	27470443	(A/C)
4194	CakSNP4194	*Kabuli*	Ca_Kabuli_Chr03	27470439	(G/T)
4195	CakSNP4195	*Kabuli*	Ca_Kabuli_Chr03	27597331	(A/C)
4196	CakSNP4196	*Kabuli*	Ca_Kabuli_Chr03	27597409	(A/G)
4197	CakSNP4197	*Kabuli*	Ca_Kabuli_Chr03	27632100	(G/A)
4198	CakSNP4198	*Kabuli*	Ca_Kabuli_Chr03	27632169	(T/C)
4199	CakSNP4199	*Kabuli*	Ca_Kabuli_Chr03	27654798	(A/C)
4200	CakSNP4200	*Kabuli*	Ca_Kabuli_Chr03	27673727	(C/A)
4201	CakSNP4201	*Kabuli*	Ca_Kabuli_Chr03	27681551	(T/C)
4202	CakSNP4202	*Kabuli*	Ca_Kabuli_Chr03	27716396	(C/T)
4203	CakSNP4203	*Kabuli*	Ca_Kabuli_Chr03	27763236	(C/T)
4204	CakSNP4204	*Kabuli*	Ca_Kabuli_Chr03	27812571	(A/T)
4205	CakSNP4205	*Kabuli*	Ca_Kabuli_Chr03	27876405	(A/G)
4206	CakSNP4206	*Kabuli*	Ca_Kabuli_Chr03	27877442	(A/T)
4207	CakSNP4207	*Kabuli*	Ca_Kabuli_Chr03	27877479	(C/T)
4208	CakSNP4208	*Kabuli*	Ca_Kabuli_Chr03	27877480	(G/A)
4209	CakSNP4209	*Kabuli*	Ca_Kabuli_Chr03	27877510	(G/A)
4210	CakSNP4210	*Kabuli*	Ca_Kabuli_Chr03	27888817	(G/C)
4211	CakSNP4211	*Kabuli*	Ca_Kabuli_Chr03	27889107	(A/G)
4212	CakSNP4212	*Kabuli*	Ca_Kabuli_Chr03	27890849	(C/T)
4213	CakSNP4213	*Kabuli*	Ca_Kabuli_Chr03	27905944	(T/C)
4214	CakSNP4214	*Kabuli*	Ca_Kabuli_Chr03	27905989	(G/A)
4215	CakSNP4215	*Kabuli*	Ca_Kabuli_Chr03	27905995	(C/T)
4216	CakSNP4216	*Kabuli*	Ca_Kabuli_Chr03	27906069	(C/A)
4217	CakSNP4217	*Kabuli*	Ca_Kabuli_Chr03	27914409	(C/T)

S.N.	SNP IDs	Cultivars	Chromosomes/scaffolds	Physical positions (bp)	SNPs
4218	CakSNP4218	*Kabuli*	Ca_Kabuli_Chr03	27968979	(T/G)
4219	CakSNP4219	*Kabuli*	Ca_Kabuli_Chr03	27969014	(T/C)
4220	CakSNP4220	*Kabuli*	Ca_Kabuli_Chr03	27969005	(A/G)
4221	CakSNP4221	*Kabuli*	Ca_Kabuli_Chr03	27969207	(C/T)
4222	CakSNP4222	*Kabuli*	Ca_Kabuli_Chr03	28044035	(A/T)
4223	CakSNP4223	*Kabuli*	Ca_Kabuli_Chr03	28176380	(A/C)
4224	CakSNP4224	*Kabuli*	Ca_Kabuli_Chr03	28176333	(C/A)
4225	CakSNP4225	*Kabuli*	Ca_Kabuli_Chr03	28277991	(C/G)
4226	CakSNP4226	*Kabuli*	Ca_Kabuli_Chr03	28278018	(C/G)
4227	CakSNP4227	*Kabuli*	Ca_Kabuli_Chr03	28374024	(C/T)
4228	CakSNP4228	*Kabuli*	Ca_Kabuli_Chr03	28375082	(G/A)
4229	CakSNP4229	*Kabuli*	Ca_Kabuli_Chr03	28409001	(G/A)
4230	CakSNP4230	*Kabuli*	Ca_Kabuli_Chr03	28420948	(C/T)
4231	CakSNP4231	*Kabuli*	Ca_Kabuli_Chr03	28478044	(C/T)
4232	CakSNP4232	*Kabuli*	Ca_Kabuli_Chr03	28478539	(C/G)
4233	CakSNP4233	*Kabuli*	Ca_Kabuli_Chr03	28478569	(C/A)
4234	CakSNP4234	*Kabuli*	Ca_Kabuli_Chr03	28602055	(C/A)
4235	CakSNP4235	*Kabuli*	Ca_Kabuli_Chr03	28603892	(T/C)
4236	CakSNP4236	*Kabuli*	Ca_Kabuli_Chr03	28613067	(A/G)
4237	CakSNP4237	*Kabuli*	Ca_Kabuli_Chr03	28614784	(A/G)
4238	CakSNP4238	*Kabuli*	Ca_Kabuli_Chr03	28614919	(A/T)
4239	CakSNP4239	*Kabuli*	Ca_Kabuli_Chr03	28680858	(C/T)
4240	CakSNP4240	*Kabuli*	Ca_Kabuli_Chr03	28706106	(T/A)
4241	CakSNP4241	*Kabuli*	Ca_Kabuli_Chr03	28706292	(G/C)
4242	CakSNP4242	*Kabuli*	Ca_Kabuli_Chr03	28712993	(T/C)
4243	CakSNP4243	*Kabuli*	Ca_Kabuli_Chr03	28716003	(C/T)
4244	CakSNP4244	*Kabuli*	Ca_Kabuli_Chr03	28716027	(A/T)
4245	CakSNP4245	*Kabuli*	Ca_Kabuli_Chr03	28716028	(A/T)
4246	CakSNP4246	*Kabuli*	Ca_Kabuli_Chr03	28716029	(A/G)
4247	CakSNP4247	*Kabuli*	Ca_Kabuli_Chr03	28716038	(T/G)
4248	CakSNP4248	*Kabuli*	Ca_Kabuli_Chr03	28716047	(G/A)
4249	CakSNP4249	*Kabuli*	Ca_Kabuli_Chr03	28755111	(A/G)
4250	CakSNP4250	*Kabuli*	Ca_Kabuli_Chr03	28755149	(G/A)
4251	CakSNP4251	*Kabuli*	Ca_Kabuli_Chr03	28845958	(A/C)
4252	CakSNP4252	*Kabuli*	Ca_Kabuli_Chr03	28926094	(T/A)
4253	CakSNP4253	*Kabuli*	Ca_Kabuli_Chr03	28926290	(T/C)
4254	CakSNP4254	*Kabuli*	Ca_Kabuli_Chr03	28934196	(C/T)
4255	CakSNP4255	*Kabuli*	Ca_Kabuli_Chr03	28943956	(C/T)

S.N.	SNP IDs	Cultivars	Chromosomes/scaffolds	Physical positions (bp)	SNPs
4256	CakSNP4256	*Kabuli*	Ca_Kabuli_Chr03	28943989	(G/T)
4257	CakSNP4257	*Kabuli*	Ca_Kabuli_Chr03	29056451	(T/C)
4258	CakSNP4258	*Kabuli*	Ca_Kabuli_Chr03	29056528	(G/T)
4259	CakSNP4259	*Kabuli*	Ca_Kabuli_Chr03	29056768	(T/C)
4260	CakSNP4260	*Kabuli*	Ca_Kabuli_Chr03	29107776	(A/G)
4261	CakSNP4261	*Kabuli*	Ca_Kabuli_Chr03	29107947	(C/A)
4262	CakSNP4262	*Kabuli*	Ca_Kabuli_Chr03	29155741	(C/G)
4263	CakSNP4263	*Kabuli*	Ca_Kabuli_Chr03	29177885	(A/C)
4264	CakSNP4264	*Kabuli*	Ca_Kabuli_Chr03	29188300	(T/G)
4265	CakSNP4265	*Kabuli*	Ca_Kabuli_Chr03	29188308	(G/A)
4266	CakSNP4266	*Kabuli*	Ca_Kabuli_Chr03	29207338	(G/A)
4267	CakSNP4267	*Kabuli*	Ca_Kabuli_Chr03	29293770	(G/T)
4268	CakSNP4268	*Kabuli*	Ca_Kabuli_Chr03	29293783	(G/A)
4269	CakSNP4269	*Kabuli*	Ca_Kabuli_Chr03	29293840	(C/A)
4270	CakSNP4270	*Kabuli*	Ca_Kabuli_Chr03	29302251	(C/T)
4271	CakSNP4271	*Kabuli*	Ca_Kabuli_Chr03	29302448	(A/C)
4272	CakSNP4272	*Kabuli*	Ca_Kabuli_Chr03	29303144	(A/C)
4273	CakSNP4273	*Kabuli*	Ca_Kabuli_Chr03	29304116	(A/G)
4274	CakSNP4274	*Kabuli*	Ca_Kabuli_Chr03	29304161	(C/T)
4275	CakSNP4275	*Kabuli*	Ca_Kabuli_Chr03	29311672	(C/A)
4276	CakSNP4276	*Kabuli*	Ca_Kabuli_Chr03	29348267	(C/T)
4277	CakSNP4277	*Kabuli*	Ca_Kabuli_Chr03	29355268	(T/A)
4278	CakSNP4278	*Kabuli*	Ca_Kabuli_Chr03	29376025	(A/G)
4279	CakSNP4279	*Kabuli*	Ca_Kabuli_Chr03	29376008	(G/A)
4280	CakSNP4280	*Kabuli*	Ca_Kabuli_Chr03	29439505	(A/G)
4281	CakSNP4281	*Kabuli*	Ca_Kabuli_Chr03	29485590	(T/C)
4282	CakSNP4282	*Kabuli*	Ca_Kabuli_Chr03	29485566	(T/A)
4283	CakSNP4283	*Kabuli*	Ca_Kabuli_Chr03	29508831	(C/T)
4284	CakSNP4284	*Kabuli*	Ca_Kabuli_Chr03	29509323	(G/A)
4285	CakSNP4285	*Kabuli*	Ca_Kabuli_Chr03	29509310	(G/T)
4286	CakSNP4286	*Kabuli*	Ca_Kabuli_Chr03	29547528	(G/A)
4287	CakSNP4287	*Kabuli*	Ca_Kabuli_Chr03	29588940	(C/T)
4288	CakSNP4288	*Kabuli*	Ca_Kabuli_Chr03	29588934	(T/C)
4289	CakSNP4289	*Kabuli*	Ca_Kabuli_Chr03	29588930	(T/C)
4290	CakSNP4290	*Kabuli*	Ca_Kabuli_Chr03	29591575	(T/G)
4291	CakSNP4291	*Kabuli*	Ca_Kabuli_Chr03	29591613	(G/T)
4292	CakSNP4292	*Kabuli*	Ca_Kabuli_Chr03	29603720	(T/C)
4293	CakSNP4293	*Kabuli*	Ca_Kabuli_Chr03	29640075	(A/C)

S.N.	SNP IDs	Cultivars	Chromosomes/scaffolds	Physical positions (bp)	SNPs
4294	CakSNP4294	*Kabuli*	Ca_Kabuli_Chr03	29640071	(T/C)
4295	CakSNP4295	*Kabuli*	Ca_Kabuli_Chr03	29666633	(C/T)
4296	CakSNP4296	*Kabuli*	Ca_Kabuli_Chr03	29690732	(C/A)
4297	CakSNP4297	*Kabuli*	Ca_Kabuli_Chr03	29698166	(G/T)
4298	CakSNP4298	*Kabuli*	Ca_Kabuli_Chr03	29698245	(T/A)
4299	CakSNP4299	*Kabuli*	Ca_Kabuli_Chr03	29698289	(G/A)
4300	CakSNP4300	*Kabuli*	Ca_Kabuli_Chr03	29698273	(A/C)
4301	CakSNP4301	*Kabuli*	Ca_Kabuli_Chr03	29730019	(G/T)
4302	CakSNP4302	*Kabuli*	Ca_Kabuli_Chr03	29744878	(T/G)
4303	CakSNP4303	*Kabuli*	Ca_Kabuli_Chr03	29744890	(A/C)
4304	CakSNP4304	*Kabuli*	Ca_Kabuli_Chr03	29803866	(C/T)
4305	CakSNP4305	*Kabuli*	Ca_Kabuli_Chr03	29803962	(A/G)
4306	CakSNP4306	*Kabuli*	Ca_Kabuli_Chr03	29807091	(C/G)
4307	CakSNP4307	*Kabuli*	Ca_Kabuli_Chr03	29807248	(A/G)
4308	CakSNP4308	*Kabuli*	Ca_Kabuli_Chr03	29836337	(C/A)
4309	CakSNP4309	*Kabuli*	Ca_Kabuli_Chr03	29840029	(C/G)
4310	CakSNP4310	*Kabuli*	Ca_Kabuli_Chr03	29840028	(T/C)
4311	CakSNP4311	*Kabuli*	Ca_Kabuli_Chr03	29840022	(C/A)
4312	CakSNP4312	*Kabuli*	Ca_Kabuli_Chr03	29840012	(A/T)
4313	CakSNP4313	*Kabuli*	Ca_Kabuli_Chr03	29844509	(T/A)
4314	CakSNP4314	*Kabuli*	Ca_Kabuli_Chr03	29869190	(G/T)
4315	CakSNP4315	*Kabuli*	Ca_Kabuli_Chr03	29869199	(T/C)
4316	CakSNP4316	*Kabuli*	Ca_Kabuli_Chr03	29869428	(G/T)
4317	CakSNP4317	*Kabuli*	Ca_Kabuli_Chr03	29869381	(G/A)
4318	CakSNP4318	*Kabuli*	Ca_Kabuli_Chr03	29869380	(G/A)
4319	CakSNP4319	*Kabuli*	Ca_Kabuli_Chr03	29872643	(A/G)
4320	CakSNP4320	*Kabuli*	Ca_Kabuli_Chr03	29872682	(G/A)
4321	CakSNP4321	*Kabuli*	Ca_Kabuli_Chr03	29898396	(A/G)
4322	CakSNP4322	*Kabuli*	Ca_Kabuli_Chr03	29912488	(A/G)
4323	CakSNP4323	*Kabuli*	Ca_Kabuli_Chr03	29915510	(G/A)
4324	CakSNP4324	*Kabuli*	Ca_Kabuli_Chr03	29932378	(C/G)
4325	CakSNP4325	*Kabuli*	Ca_Kabuli_Chr03	29932504	(T/A)
4326	CakSNP4326	*Kabuli*	Ca_Kabuli_Chr03	29932502	(G/C)
4327	CakSNP4327	*Kabuli*	Ca_Kabuli_Chr03	29954498	(G/A)
4328	CakSNP4328	*Kabuli*	Ca_Kabuli_Chr03	29954533	(A/G)
4329	CakSNP4329	*Kabuli*	Ca_Kabuli_Chr03	29954648	(A/T)
4330	CakSNP4330	*Kabuli*	Ca_Kabuli_Chr03	29954612	(T/G)
4331	CakSNP4331	*Kabuli*	Ca_Kabuli_Chr03	29974532	(A/G)

S.N.	SNP IDs	Cultivars	Chromosomes/scaffolds	Physical positions (bp)	SNPs
4332	CakSNP4332	*Kabuli*	Ca_Kabuli_Chr03	29974535	(A/G)
4333	CakSNP4333	*Kabuli*	Ca_Kabuli_Chr03	29974595	(C/T)
4334	CakSNP4334	*Kabuli*	Ca_Kabuli_Chr03	29982638	(A/G)
4335	CakSNP4335	*Kabuli*	Ca_Kabuli_Chr03	29982644	(T/C)
4336	CakSNP4336	*Kabuli*	Ca_Kabuli_Chr03	29982718	(C/A)
4337	CakSNP4337	*Kabuli*	Ca_Kabuli_Chr03	30004352	(T/A)
4338	CakSNP4338	*Kabuli*	Ca_Kabuli_Chr03	30004311	(T/G)
4339	CakSNP4339	*Kabuli*	Ca_Kabuli_Chr03	30004295	(A/G)
4340	CakSNP4340	*Kabuli*	Ca_Kabuli_Chr03	30004279	(G/T)
4341	CakSNP4341	*Kabuli*	Ca_Kabuli_Chr03	30009698	(C/T)
4342	CakSNP4342	*Kabuli*	Ca_Kabuli_Chr03	30009751	(A/C)
4343	CakSNP4343	*Kabuli*	Ca_Kabuli_Chr03	30011584	(T/C)
4344	CakSNP4344	*Kabuli*	Ca_Kabuli_Chr03	30011669	(A/G)
4345	CakSNP4345	*Kabuli*	Ca_Kabuli_Chr03	30011642	(T/A)
4346	CakSNP4346	*Kabuli*	Ca_Kabuli_Chr03	30011621	(C/T)
4347	CakSNP4347	*Kabuli*	Ca_Kabuli_Chr03	30029874	(C/T)
4348	CakSNP4348	*Kabuli*	Ca_Kabuli_Chr03	30029858	(T/A)
4349	CakSNP4349	*Kabuli*	Ca_Kabuli_Chr03	30029842	(G/C)
4350	CakSNP4350	*Kabuli*	Ca_Kabuli_Chr03	30029826	(T/C)
4351	CakSNP4351	*Kabuli*	Ca_Kabuli_Chr03	30029817	(T/G)
4352	CakSNP4352	*Kabuli*	Ca_Kabuli_Chr03	30084154	(T/C)
4353	CakSNP4353	*Kabuli*	Ca_Kabuli_Chr03	30101412	(T/C)
4354	CakSNP4354	*Kabuli*	Ca_Kabuli_Chr03	30101476	(A/C)
4355	CakSNP4355	*Kabuli*	Ca_Kabuli_Chr03	30111154	(A/C)
4356	CakSNP4356	*Kabuli*	Ca_Kabuli_Chr03	30111146	(G/C)
4357	CakSNP4357	*Kabuli*	Ca_Kabuli_Chr03	30148907	(T/C)
4358	CakSNP4358	*Kabuli*	Ca_Kabuli_Chr03	30194810	(C/T)
4359	CakSNP4359	*Kabuli*	Ca_Kabuli_Chr03	30206121	(T/A)
4360	CakSNP4360	*Kabuli*	Ca_Kabuli_Chr03	30206266	(G/A)
4361	CakSNP4361	*Kabuli*	Ca_Kabuli_Chr03	30206281	(A/T)
4362	CakSNP4362	*Kabuli*	Ca_Kabuli_Chr03	30206436	(G/A)
4363	CakSNP4363	*Kabuli*	Ca_Kabuli_Chr03	30267761	(T/G)
4364	CakSNP4364	*Kabuli*	Ca_Kabuli_Chr03	30271022	(G/T)
4365	CakSNP4365	*Kabuli*	Ca_Kabuli_Chr03	30271028	(G/A)
4366	CakSNP4366	*Kabuli*	Ca_Kabuli_Chr03	30271034	(G/A)
4367	CakSNP4367	*Kabuli*	Ca_Kabuli_Chr03	30309625	(G/C)
4368	CakSNP4368	*Kabuli*	Ca_Kabuli_Chr03	30309624	(A/T)
4369	CakSNP4369	*Kabuli*	Ca_Kabuli_Chr03	30321177	(C/G)

S.N.	SNP IDs	Cultivars	Chromosomes/scaffolds	Physical positions (bp)	SNPs
4370	CakSNP4370	*Kabuli*	Ca_Kabuli_Chr03	30321245	(C/T)
4371	CakSNP4371	*Kabuli*	Ca_Kabuli_Chr03	30321272	(C/A)
4372	CakSNP4372	*Kabuli*	Ca_Kabuli_Chr03	30323585	(G/A)
4373	CakSNP4373	*Kabuli*	Ca_Kabuli_Chr03	30376319	(G/A)
4374	CakSNP4374	*Kabuli*	Ca_Kabuli_Chr03	30385144	(G/A)
4375	CakSNP4375	*Kabuli*	Ca_Kabuli_Chr03	30385153	(T/A)
4376	CakSNP4376	*Kabuli*	Ca_Kabuli_Chr03	30390663	(C/T)
4377	CakSNP4377	*Kabuli*	Ca_Kabuli_Chr03	30397262	(A/T)
4378	CakSNP4378	*Kabuli*	Ca_Kabuli_Chr03	30425496	(G/T)
4379	CakSNP4379	*Kabuli*	Ca_Kabuli_Chr03	30510392	(T/A)
4380	CakSNP4380	*Kabuli*	Ca_Kabuli_Chr03	30510333	(C/T)
4381	CakSNP4381	*Kabuli*	Ca_Kabuli_Chr03	30522442	(A/C)
4382	CakSNP4382	*Kabuli*	Ca_Kabuli_Chr03	30522485	(C/T)
4383	CakSNP4383	*Kabuli*	Ca_Kabuli_Chr03	30522498	(T/C)
4384	CakSNP4384	*Kabuli*	Ca_Kabuli_Chr03	30650598	(C/A)
4385	CakSNP4385	*Kabuli*	Ca_Kabuli_Chr03	30650634	(T/C)
4386	CakSNP4386	*Kabuli*	Ca_Kabuli_Chr03	30650659	(C/T)
4387	CakSNP4387	*Kabuli*	Ca_Kabuli_Chr03	30659438	(T/A)
4388	CakSNP4388	*Kabuli*	Ca_Kabuli_Chr03	30675468	(T/G)
4389	CakSNP4389	*Kabuli*	Ca_Kabuli_Chr03	30681031	(A/G)
4390	CakSNP4390	*Kabuli*	Ca_Kabuli_Chr03	30681032	(A/G)
4391	CakSNP4391	*Kabuli*	Ca_Kabuli_Chr03	30688336	(T/G)
4392	CakSNP4392	*Kabuli*	Ca_Kabuli_Chr03	30688354	(G/A)
4393	CakSNP4393	*Kabuli*	Ca_Kabuli_Chr03	30688369	(T/C)
4394	CakSNP4394	*Kabuli*	Ca_Kabuli_Chr03	30688418	(A/C)
4395	CakSNP4395	*Kabuli*	Ca_Kabuli_Chr03	30688511	(G/C)
4396	CakSNP4396	*Kabuli*	Ca_Kabuli_Chr03	30688485	(C/A)
4397	CakSNP4397	*Kabuli*	Ca_Kabuli_Chr03	30688484	(A/C)
4398	CakSNP4398	*Kabuli*	Ca_Kabuli_Chr03	30688454	(A/G)
4399	CakSNP4399	*Kabuli*	Ca_Kabuli_Chr03	30688451	(T/C)
4400	CakSNP4400	*Kabuli*	Ca_Kabuli_Chr03	30718334	(G/T)
4401	CakSNP4401	*Kabuli*	Ca_Kabuli_Chr03	30751893	(A/C)
4402	CakSNP4402	*Kabuli*	Ca_Kabuli_Chr03	30751949	(A/T)
4403	CakSNP4403	*Kabuli*	Ca_Kabuli_Chr03	30791169	(A/T)
4404	CakSNP4404	*Kabuli*	Ca_Kabuli_Chr03	30791229	(G/A)
4405	CakSNP4405	*Kabuli*	Ca_Kabuli_Chr03	30791693	(C/T)
4406	CakSNP4406	*Kabuli*	Ca_Kabuli_Chr03	30794040	(C/A)
4407	CakSNP4407	*Kabuli*	Ca_Kabuli_Chr03	30794046	(A/C)

S.N.	SNP IDs	Cultivars	Chromosomes/scaffolds	Physical positions (bp)	SNPs
4408	CakSNP4408	*Kabuli*	Ca_Kabuli_Chr03	30837147	(A/G)
4409	CakSNP4409	*Kabuli*	Ca_Kabuli_Chr03	30837284	(C/T)
4410	CakSNP4410	*Kabuli*	Ca_Kabuli_Chr03	30837265	(T/C)
4411	CakSNP4411	*Kabuli*	Ca_Kabuli_Chr03	30880452	(T/C)
4412	CakSNP4412	*Kabuli*	Ca_Kabuli_Chr03	30880610	(A/G)
4413	CakSNP4413	*Kabuli*	Ca_Kabuli_Chr03	31020357	(C/A)
4414	CakSNP4414	*Kabuli*	Ca_Kabuli_Chr03	31020358	(C/G)
4415	CakSNP4415	*Kabuli*	Ca_Kabuli_Chr03	31073396	(T/C)
4416	CakSNP4416	*Kabuli*	Ca_Kabuli_Chr03	31073425	(G/C)
4417	CakSNP4417	*Kabuli*	Ca_Kabuli_Chr03	31073451	(A/G)
4418	CakSNP4418	*Kabuli*	Ca_Kabuli_Chr03	31091875	(T/G)
4419	CakSNP4419	*Kabuli*	Ca_Kabuli_Chr03	31267639	(T/A)
4420	CakSNP4420	*Kabuli*	Ca_Kabuli_Chr03	31267675	(A/G)
4421	CakSNP4421	*Kabuli*	Ca_Kabuli_Chr03	31267681	(G/A)
4422	CakSNP4422	*Kabuli*	Ca_Kabuli_Chr03	31267828	(A/G)
4423	CakSNP4423	*Kabuli*	Ca_Kabuli_Chr03	31267927	(T/G)
4424	CakSNP4424	*Kabuli*	Ca_Kabuli_Chr03	31268143	(A/G)
4425	CakSNP4425	*Kabuli*	Ca_Kabuli_Chr03	31279502	(C/T)
4426	CakSNP4426	*Kabuli*	Ca_Kabuli_Chr03	31279541	(A/G)
4427	CakSNP4427	*Kabuli*	Ca_Kabuli_Chr03	31279639	(C/T)
4428	CakSNP4428	*Kabuli*	Ca_Kabuli_Chr03	31279629	(C/G)
4429	CakSNP4429	*Kabuli*	Ca_Kabuli_Chr03	31279628	(T/C)
4430	CakSNP4430	*Kabuli*	Ca_Kabuli_Chr03	31279616	(T/A)
4431	CakSNP4431	*Kabuli*	Ca_Kabuli_Chr03	31279606	(G/A)
4432	CakSNP4432	*Kabuli*	Ca_Kabuli_Chr03	31279605	(G/A)
4433	CakSNP4433	*Kabuli*	Ca_Kabuli_Chr03	31279604	(C/G)
4434	CakSNP4434	*Kabuli*	Ca_Kabuli_Chr03	31279600	(T/C)
4435	CakSNP4435	*Kabuli*	Ca_Kabuli_Chr03	31279586	(A/G)
4436	CakSNP4436	*Kabuli*	Ca_Kabuli_Chr03	31287618	(C/A)
4437	CakSNP4437	*Kabuli*	Ca_Kabuli_Chr03	31287909	(A/G)
4438	CakSNP4438	*Kabuli*	Ca_Kabuli_Chr03	31288143	(T/C)
4439	CakSNP4439	*Kabuli*	Ca_Kabuli_Chr03	31301723	(G/A)
4440	CakSNP4440	*Kabuli*	Ca_Kabuli_Chr03	31301783	(A/G)
4441	CakSNP4441	*Kabuli*	Ca_Kabuli_Chr03	31302060	(T/C)
4442	CakSNP4442	*Kabuli*	Ca_Kabuli_Chr03	31302117	(C/T)
4443	CakSNP4443	*Kabuli*	Ca_Kabuli_Chr03	31313021	(G/T)
4444	CakSNP4444	*Kabuli*	Ca_Kabuli_Chr03	31317521	(T/G)
4445	CakSNP4445	*Kabuli*	Ca_Kabuli_Chr03	31321725	(A/G)

S.N.	SNP IDs	Cultivars	Chromosomes/scaffolds	Physical positions (bp)	SNPs
4446	CakSNP4446	*Kabuli*	Ca_Kabuli_Chr03	31321778	(G/A)
4447	CakSNP4447	*Kabuli*	Ca_Kabuli_Chr03	31321850	(A/G)
4448	CakSNP4448	*Kabuli*	Ca_Kabuli_Chr03	31341335	(C/T)
4449	CakSNP4449	*Kabuli*	Ca_Kabuli_Chr03	31341323	(T/C)
4450	CakSNP4450	*Kabuli*	Ca_Kabuli_Chr03	31342119	(A/T)
4451	CakSNP4451	*Kabuli*	Ca_Kabuli_Chr03	31354988	(G/C)
4452	CakSNP4452	*Kabuli*	Ca_Kabuli_Chr03	31355001	(C/T)
4453	CakSNP4453	*Kabuli*	Ca_Kabuli_Chr03	31355026	(T/C)
4454	CakSNP4454	*Kabuli*	Ca_Kabuli_Chr03	31355066	(A/T)
4455	CakSNP4455	*Kabuli*	Ca_Kabuli_Chr03	31357787	(A/T)
4456	CakSNP4456	*Kabuli*	Ca_Kabuli_Chr03	31431216	(C/T)
4457	CakSNP4457	*Kabuli*	Ca_Kabuli_Chr03	31478804	(T/C)
4458	CakSNP4458	*Kabuli*	Ca_Kabuli_Chr03	31478882	(C/T)
4459	CakSNP4459	*Kabuli*	Ca_Kabuli_Chr03	31480603	(T/C)
4460	CakSNP4460	*Kabuli*	Ca_Kabuli_Chr03	31516662	(G/T)
4461	CakSNP4461	*Kabuli*	Ca_Kabuli_Chr03	31516766	(T/C)
4462	CakSNP4462	*Kabuli*	Ca_Kabuli_Chr03	31520119	(C/T)
4463	CakSNP4463	*Kabuli*	Ca_Kabuli_Chr03	31520225	(A/T)
4464	CakSNP4464	*Kabuli*	Ca_Kabuli_Chr03	31520198	(G/A)
4465	CakSNP4465	*Kabuli*	Ca_Kabuli_Chr03	31520190	(T/C)
4466	CakSNP4466	*Kabuli*	Ca_Kabuli_Chr03	31520832	(G/A)
4467	CakSNP4467	*Kabuli*	Ca_Kabuli_Chr03	31521142	(T/C)
4468	CakSNP4468	*Kabuli*	Ca_Kabuli_Chr03	31521155	(T/A)
4469	CakSNP4469	*Kabuli*	Ca_Kabuli_Chr03	31589355	(A/G)
4470	CakSNP4470	*Kabuli*	Ca_Kabuli_Chr03	31589329	(C/T)
4471	CakSNP4471	*Kabuli*	Ca_Kabuli_Chr03	31591065	(G/A)
4472	CakSNP4472	*Kabuli*	Ca_Kabuli_Chr03	31606359	(C/T)
4473	CakSNP4473	*Kabuli*	Ca_Kabuli_Chr03	31630175	(T/A)
4474	CakSNP4474	*Kabuli*	Ca_Kabuli_Chr03	31630178	(G/T)
4475	CakSNP4475	*Kabuli*	Ca_Kabuli_Chr03	31630195	(T/C)
4476	CakSNP4476	*Kabuli*	Ca_Kabuli_Chr03	31631451	(A/C)
4477	CakSNP4477	*Kabuli*	Ca_Kabuli_Chr03	31631444	(T/G)
4478	CakSNP4478	*Kabuli*	Ca_Kabuli_Chr03	31631561	(G/A)
4479	CakSNP4479	*Kabuli*	Ca_Kabuli_Chr03	31631534	(A/G)
4480	CakSNP4480	*Kabuli*	Ca_Kabuli_Chr03	31631622	(G/A)
4481	CakSNP4481	*Kabuli*	Ca_Kabuli_Chr03	31631644	(A/C)
4482	CakSNP4482	*Kabuli*	Ca_Kabuli_Chr03	31646735	(C/T)
4483	CakSNP4483	*Kabuli*	Ca_Kabuli_Chr03	31646817	(C/T)

S.N.	SNP IDs	Cultivars	Chromosomes/scaffolds	Physical positions (bp)	SNPs
4484	CakSNP4484	*Kabuli*	Ca_Kabuli_Chr03	31646802	(G/A)
4485	CakSNP4485	*Kabuli*	Ca_Kabuli_Chr03	31683906	(A/G)
4486	CakSNP4486	*Kabuli*	Ca_Kabuli_Chr03	31689128	(C/T)
4487	CakSNP4487	*Kabuli*	Ca_Kabuli_Chr03	31698493	(A/C)
4488	CakSNP4488	*Kabuli*	Ca_Kabuli_Chr03	31766363	(G/A)
4489	CakSNP4489	*Kabuli*	Ca_Kabuli_Chr03	31770365	(A/C)
4490	CakSNP4490	*Kabuli*	Ca_Kabuli_Chr03	31771314	(C/A)
4491	CakSNP4491	*Kabuli*	Ca_Kabuli_Chr03	31790189	(C/T)
4492	CakSNP4492	*Kabuli*	Ca_Kabuli_Chr03	31790216	(G/A)
4493	CakSNP4493	*Kabuli*	Ca_Kabuli_Chr03	31790239	(G/T)
4494	CakSNP4494	*Kabuli*	Ca_Kabuli_Chr03	31790352	(G/A)
4495	CakSNP4495	*Kabuli*	Ca_Kabuli_Chr03	31814300	(A/G)
4496	CakSNP4496	*Kabuli*	Ca_Kabuli_Chr03	31852191	(T/C)
4497	CakSNP4497	*Kabuli*	Ca_Kabuli_Chr03	31857877	(C/T)
4498	CakSNP4498	*Kabuli*	Ca_Kabuli_Chr03	31859152	(C/G)
4499	CakSNP4499	*Kabuli*	Ca_Kabuli_Chr03	31859251	(T/C)
4500	CakSNP4500	*Kabuli*	Ca_Kabuli_Chr03	31926694	(C/G)
4501	CakSNP4501	*Kabuli*	Ca_Kabuli_Chr03	31931014	(A/C)
4502	CakSNP4502	*Kabuli*	Ca_Kabuli_Chr03	31945504	(C/T)
4503	CakSNP4503	*Kabuli*	Ca_Kabuli_Chr03	31945634	(A/G)
4504	CakSNP4504	*Kabuli*	Ca_Kabuli_Chr03	31951994	(G/A)
4505	CakSNP4505	*Kabuli*	Ca_Kabuli_Chr03	31953466	(G/A)
4506	CakSNP4506	*Kabuli*	Ca_Kabuli_Chr03	31953549	(C/T)
4507	CakSNP4507	*Kabuli*	Ca_Kabuli_Chr03	32351052	(C/T)
4508	CakSNP4508	*Kabuli*	Ca_Kabuli_Chr03	32351031	(G/C)
4509	CakSNP4509	*Kabuli*	Ca_Kabuli_Chr03	32357599	(C/T)
4510	CakSNP4510	*Kabuli*	Ca_Kabuli_Chr03	32357578	(G/C)
4511	CakSNP4511	*Kabuli*	Ca_Kabuli_Chr03	32448959	(T/C)
4512	CakSNP4512	*Kabuli*	Ca_Kabuli_Chr03	32449654	(T/G)
4513	CakSNP4513	*Kabuli*	Ca_Kabuli_Chr03	32449700	(C/T)
4514	CakSNP4514	*Kabuli*	Ca_Kabuli_Chr03	32510033	(T/G)
4515	CakSNP4515	*Kabuli*	Ca_Kabuli_Chr03	32510090	(A/G)
4516	CakSNP4516	*Kabuli*	Ca_Kabuli_Chr03	32510130	(A/G)
4517	CakSNP4517	*Kabuli*	Ca_Kabuli_Chr03	32510124	(C/T)
4518	CakSNP4518	*Kabuli*	Ca_Kabuli_Chr03	32510111	(C/T)
4519	CakSNP4519	*Kabuli*	Ca_Kabuli_Chr03	32510071	(G/T)
4520	CakSNP4520	*Kabuli*	Ca_Kabuli_Chr03	32510081	(C/T)
4521	CakSNP4521	*Kabuli*	Ca_Kabuli_Chr03	32514908	(A/G)

S.N.	SNP IDs	Cultivars	Chromosomes/scaffolds	Physical positions (bp)	SNPs
4522	CakSNP4522	*Kabuli*	Ca_Kabuli_Chr03	32514890	(A/G)
4523	CakSNP4523	*Kabuli*	Ca_Kabuli_Chr03	32514889	(C/T)
4524	CakSNP4524	*Kabuli*	Ca_Kabuli_Chr03	32514869	(C/T)
4525	CakSNP4525	*Kabuli*	Ca_Kabuli_Chr03	32514868	(A/G)
4526	CakSNP4526	*Kabuli*	Ca_Kabuli_Chr03	32514867	(C/T)
4527	CakSNP4527	*Kabuli*	Ca_Kabuli_Chr03	32514863	(G/C)
4528	CakSNP4528	*Kabuli*	Ca_Kabuli_Chr03	32514859	(C/T)
4529	CakSNP4529	*Kabuli*	Ca_Kabuli_Chr03	32514916	(G/C)
4530	CakSNP4530	*Kabuli*	Ca_Kabuli_Chr03	32525128	(G/T)
4531	CakSNP4531	*Kabuli*	Ca_Kabuli_Chr03	32557850	(C/T)
4532	CakSNP4532	*Kabuli*	Ca_Kabuli_Chr03	32571237	(G/A)
4533	CakSNP4533	*Kabuli*	Ca_Kabuli_Chr03	32571291	(A/G)
4534	CakSNP4534	*Kabuli*	Ca_Kabuli_Chr03	32571275	(T/C)
4535	CakSNP4535	*Kabuli*	Ca_Kabuli_Chr03	32600003	(A/G)
4536	CakSNP4536	*Kabuli*	Ca_Kabuli_Chr03	32601732	(T/C)
4537	CakSNP4537	*Kabuli*	Ca_Kabuli_Chr03	32608947	(A/G)
4538	CakSNP4538	*Kabuli*	Ca_Kabuli_Chr03	32662861	(A/G)
4539	CakSNP4539	*Kabuli*	Ca_Kabuli_Chr03	32662858	(A/G)
4540	CakSNP4540	*Kabuli*	Ca_Kabuli_Chr03	32663015	(C/T)
4541	CakSNP4541	*Kabuli*	Ca_Kabuli_Chr03	32663059	(A/C)
4542	CakSNP4542	*Kabuli*	Ca_Kabuli_Chr03	32694509	(T/C)
4543	CakSNP4543	*Kabuli*	Ca_Kabuli_Chr03	32694454	(T/C)
4544	CakSNP4544	*Kabuli*	Ca_Kabuli_Chr03	32704734	(G/A)
4545	CakSNP4545	*Kabuli*	Ca_Kabuli_Chr03	32704868	(G/T)
4546	CakSNP4546	*Kabuli*	Ca_Kabuli_Chr03	32704953	(G/A)
4547	CakSNP4547	*Kabuli*	Ca_Kabuli_Chr03	32706200	(C/T)
4548	CakSNP4548	*Kabuli*	Ca_Kabuli_Chr03	32725039	(C/G)
4549	CakSNP4549	*Kabuli*	Ca_Kabuli_Chr03	32725096	(A/T)
4550	CakSNP4550	*Kabuli*	Ca_Kabuli_Chr03	32758259	(C/A)
4551	CakSNP4551	*Kabuli*	Ca_Kabuli_Chr03	32758293	(T/C)
4552	CakSNP4552	*Kabuli*	Ca_Kabuli_Chr03	32785228	(A/G)
4553	CakSNP4553	*Kabuli*	Ca_Kabuli_Chr03	32827067	(C/G)
4554	CakSNP4554	*Kabuli*	Ca_Kabuli_Chr03	32827065	(T/G)
4555	CakSNP4555	*Kabuli*	Ca_Kabuli_Chr03	32845361	(A/G)
4556	CakSNP4556	*Kabuli*	Ca_Kabuli_Chr03	32845395	(C/A)
4557	CakSNP4557	*Kabuli*	Ca_Kabuli_Chr03	32847120	(T/A)
4558	CakSNP4558	*Kabuli*	Ca_Kabuli_Chr03	32847216	(A/G)
4559	CakSNP4559	*Kabuli*	Ca_Kabuli_Chr03	32865549	(C/T)

S.N.	SNP IDs	Cultivars	Chromosomes/scaffolds	Physical positions (bp)	SNPs
4560	CakSNP4560	*Kabuli*	Ca_Kabuli_Chr03	32865530	(C/G)
4561	CakSNP4561	*Kabuli*	Ca_Kabuli_Chr03	32954428	(T/C)
4562	CakSNP4562	*Kabuli*	Ca_Kabuli_Chr03	33033245	(T/C)
4563	CakSNP4563	*Kabuli*	Ca_Kabuli_Chr03	33091737	(A/C)
4564	CakSNP4564	*Kabuli*	Ca_Kabuli_Chr03	33120619	(G/A)
4565	CakSNP4565	*Kabuli*	Ca_Kabuli_Chr03	33120825	(G/A)
4566	CakSNP4566	*Kabuli*	Ca_Kabuli_Chr03	33520615	(A/C)
4567	CakSNP4567	*Kabuli*	Ca_Kabuli_Chr03	33520887	(G/A)
4568	CakSNP4568	*Kabuli*	Ca_Kabuli_Chr03	33522974	(G/A)
4569	CakSNP4569	*Kabuli*	Ca_Kabuli_Chr03	33533273	(G/A)
4570	CakSNP4570	*Kabuli*	Ca_Kabuli_Chr03	33627826	(A/C)
4571	CakSNP4571	*Kabuli*	Ca_Kabuli_Chr03	33715215	(G/T)
4572	CakSNP4572	*Kabuli*	Ca_Kabuli_Chr03	33898250	(C/T)
4573	CakSNP4573	*Kabuli*	Ca_Kabuli_Chr03	33900522	(A/G)
4574	CakSNP4574	*Kabuli*	Ca_Kabuli_Chr03	33900533	(A/G)
4575	CakSNP4575	*Kabuli*	Ca_Kabuli_Chr03	33904394	(T/A)
4576	CakSNP4576	*Kabuli*	Ca_Kabuli_Chr03	34247736	(T/G)
4577	CakSNP4577	*Kabuli*	Ca_Kabuli_Chr03	34247831	(G/A)
4578	CakSNP4578	*Kabuli*	Ca_Kabuli_Chr03	34247828	(T/C)
4579	CakSNP4579	*Kabuli*	Ca_Kabuli_Chr03	34247893	(A/G)
4580	CakSNP4580	*Kabuli*	Ca_Kabuli_Chr03	34268356	(A/T)
4581	CakSNP4581	*Kabuli*	Ca_Kabuli_Chr03	34282419	(C/T)
4582	CakSNP4582	*Kabuli*	Ca_Kabuli_Chr03	34290129	(T/C)
4583	CakSNP4583	*Kabuli*	Ca_Kabuli_Chr03	34290135	(G/A)
4584	CakSNP4584	*Kabuli*	Ca_Kabuli_Chr03	34290173	(C/T)
4585	CakSNP4585	*Kabuli*	Ca_Kabuli_Chr03	34352385	(A/C)
4586	CakSNP4586	*Kabuli*	Ca_Kabuli_Chr03	34356844	(A/C)
4587	CakSNP4587	*Kabuli*	Ca_Kabuli_Chr03	34357171	(T/G)
4588	CakSNP4588	*Kabuli*	Ca_Kabuli_Chr03	34357296	(C/G)
4589	CakSNP4589	*Kabuli*	Ca_Kabuli_Chr03	34496982	(G/C)
4590	CakSNP4590	*Kabuli*	Ca_Kabuli_Chr03	34524656	(C/A)
4591	CakSNP4591	*Kabuli*	Ca_Kabuli_Chr03	34542046	(T/G)
4592	CakSNP4592	*Kabuli*	Ca_Kabuli_Chr03	34543039	(C/T)
4593	CakSNP4593	*Kabuli*	Ca_Kabuli_Chr03	34543111	(A/G)
4594	CakSNP4594	*Kabuli*	Ca_Kabuli_Chr03	34547052	(A/T)
4595	CakSNP4595	*Kabuli*	Ca_Kabuli_Chr03	34547069	(G/T)
4596	CakSNP4596	*Kabuli*	Ca_Kabuli_Chr03	34547141	(A/G)
4597	CakSNP4597	*Kabuli*	Ca_Kabuli_Chr03	34576769	(G/A)

S.N.	SNP IDs	Cultivars	Chromosomes/scaffolds	Physical positions (bp)	SNPs
4598	CakSNP4598	*Kabuli*	Ca_Kabuli_Chr03	34595487	(A/G)
4599	CakSNP4599	*Kabuli*	Ca_Kabuli_Chr03	34601038	(T/C)
4600	CakSNP4600	*Kabuli*	Ca_Kabuli_Chr03	34603669	(T/A)
4601	CakSNP4601	*Kabuli*	Ca_Kabuli_Chr03	34650308	(G/A)
4602	CakSNP4602	*Kabuli*	Ca_Kabuli_Chr03	34650245	(G/A)
4603	CakSNP4603	*Kabuli*	Ca_Kabuli_Chr03	34662736	(T/G)
4604	CakSNP4604	*Kabuli*	Ca_Kabuli_Chr03	34699114	(C/A)
4605	CakSNP4605	*Kabuli*	Ca_Kabuli_Chr03	34699076	(T/C)
4606	CakSNP4606	*Kabuli*	Ca_Kabuli_Chr03	34722508	(A/T)
4607	CakSNP4607	*Kabuli*	Ca_Kabuli_Chr03	34722931	(A/G)
4608	CakSNP4608	*Kabuli*	Ca_Kabuli_Chr03	34722979	(T/C)
4609	CakSNP4609	*Kabuli*	Ca_Kabuli_Chr03	34722992	(T/G)
4610	CakSNP4610	*Kabuli*	Ca_Kabuli_Chr03	34730463	(T/C)
4611	CakSNP4611	*Kabuli*	Ca_Kabuli_Chr03	34730954	(G/A)
4612	CakSNP4612	*Kabuli*	Ca_Kabuli_Chr03	34800277	(G/A)
4613	CakSNP4613	*Kabuli*	Ca_Kabuli_Chr03	34820472	(T/A)
4614	CakSNP4614	*Kabuli*	Ca_Kabuli_Chr03	34820532	(T/A)
4615	CakSNP4615	*Kabuli*	Ca_Kabuli_Chr03	34852030	(G/A)
4616	CakSNP4616	*Kabuli*	Ca_Kabuli_Chr03	34852070	(T/G)
4617	CakSNP4617	*Kabuli*	Ca_Kabuli_Chr03	34852047	(T/A)
4618	CakSNP4618	*Kabuli*	Ca_Kabuli_Chr03	34889316	(C/G)
4619	CakSNP4619	*Kabuli*	Ca_Kabuli_Chr03	34889361	(T/C)
4620	CakSNP4620	*Kabuli*	Ca_Kabuli_Chr03	34911621	(G/A)
4621	CakSNP4621	*Kabuli*	Ca_Kabuli_Chr03	35071821	(A/G)
4622	CakSNP4622	*Kabuli*	Ca_Kabuli_Chr03	35071852	(T/G)
4623	CakSNP4623	*Kabuli*	Ca_Kabuli_Chr03	35085559	(T/A)
4624	CakSNP4624	*Kabuli*	Ca_Kabuli_Chr03	35085658	(C/A)
4625	CakSNP4625	*Kabuli*	Ca_Kabuli_Chr03	35126649	(C/G)
4626	CakSNP4626	*Kabuli*	Ca_Kabuli_Chr03	35126766	(G/A)
4627	CakSNP4627	*Kabuli*	Ca_Kabuli_Chr03	35126760	(G/A)
4628	CakSNP4628	*Kabuli*	Ca_Kabuli_Chr03	35167312	(T/G)
4629	CakSNP4629	*Kabuli*	Ca_Kabuli_Chr03	35167359	(T/G)
4630	CakSNP4630	*Kabuli*	Ca_Kabuli_Chr03	35185001	(G/T)
4631	CakSNP4631	*Kabuli*	Ca_Kabuli_Chr03	35274992	(A/G)
4632	CakSNP4632	*Kabuli*	Ca_Kabuli_Chr03	35275199	(T/C)
4633	CakSNP4633	*Kabuli*	Ca_Kabuli_Chr03	35332088	(T/A)
4634	CakSNP4634	*Kabuli*	Ca_Kabuli_Chr03	35332091	(T/C)
4635	CakSNP4635	*Kabuli*	Ca_Kabuli_Chr03	35383318	(C/T)

S.N.	SNP IDs	Cultivars	Chromosomes/scaffolds	Physical positions (bp)	SNPs
4636	CakSNP4636	*Kabuli*	Ca_Kabuli_Chr03	35394684	(G/T)
4637	CakSNP4637	*Kabuli*	Ca_Kabuli_Chr03	35403074	(A/G)
4638	CakSNP4638	*Kabuli*	Ca_Kabuli_Chr03	35420061	(A/G)
4639	CakSNP4639	*Kabuli*	Ca_Kabuli_Chr03	35420108	(T/A)
4640	CakSNP4640	*Kabuli*	Ca_Kabuli_Chr03	35424994	(T/C)
4641	CakSNP4641	*Kabuli*	Ca_Kabuli_Chr03	35483932	(T/C)
4642	CakSNP4642	*Kabuli*	Ca_Kabuli_Chr03	35483960	(T/C)
4643	CakSNP4643	*Kabuli*	Ca_Kabuli_Chr03	35483995	(T/C)
4644	CakSNP4644	*Kabuli*	Ca_Kabuli_Chr03	35483999	(C/T)
4645	CakSNP4645	*Kabuli*	Ca_Kabuli_Chr03	35484034	(G/C)
4646	CakSNP4646	*Kabuli*	Ca_Kabuli_Chr03	35487736	(T/C)
4647	CakSNP4647	*Kabuli*	Ca_Kabuli_Chr03	35488503	(A/C)
4648	CakSNP4648	*Kabuli*	Ca_Kabuli_Chr03	35488597	(T/C)
4649	CakSNP4649	*Kabuli*	Ca_Kabuli_Chr03	35488570	(G/T)
4650	CakSNP4650	*Kabuli*	Ca_Kabuli_Chr03	35490991	(A/G)
4651	CakSNP4651	*Kabuli*	Ca_Kabuli_Chr03	35490951	(C/T)
4652	CakSNP4652	*Kabuli*	Ca_Kabuli_Chr03	35543614	(C/T)
4653	CakSNP4653	*Kabuli*	Ca_Kabuli_Chr03	35631679	(C/G)
4654	CakSNP4654	*Kabuli*	Ca_Kabuli_Chr03	35631689	(C/A)
4655	CakSNP4655	*Kabuli*	Ca_Kabuli_Chr03	35631723	(A/G)
4656	CakSNP4656	*Kabuli*	Ca_Kabuli_Chr03	35647986	(C/G)
4657	CakSNP4657	*Kabuli*	Ca_Kabuli_Chr03	35747394	(C/G)
4658	CakSNP4658	*Kabuli*	Ca_Kabuli_Chr03	35807222	(A/G)
4659	CakSNP4659	*Kabuli*	Ca_Kabuli_Chr03	35807265	(T/C)
4660	CakSNP4660	*Kabuli*	Ca_Kabuli_Chr03	35807407	(A/T)
4661	CakSNP4661	*Kabuli*	Ca_Kabuli_Chr03	35829834	(A/G)
4662	CakSNP4662	*Kabuli*	Ca_Kabuli_Chr03	35898320	(G/C)
4663	CakSNP4663	*Kabuli*	Ca_Kabuli_Chr03	35898374	(G/A)
4664	CakSNP4664	*Kabuli*	Ca_Kabuli_Chr03	35898581	(C/T)
4665	CakSNP4665	*Kabuli*	Ca_Kabuli_Chr03	35920684	(A/C)
4666	CakSNP4666	*Kabuli*	Ca_Kabuli_Chr03	35920860	(C/A)
4667	CakSNP4667	*Kabuli*	Ca_Kabuli_Chr03	35948487	(G/A)
4668	CakSNP4668	*Kabuli*	Ca_Kabuli_Chr03	35979412	(G/T)
4669	CakSNP4669	*Kabuli*	Ca_Kabuli_Chr03	36003699	(G/A)
4670	CakSNP4670	*Kabuli*	Ca_Kabuli_Chr03	36003682	(G/T)
4671	CakSNP4671	*Kabuli*	Ca_Kabuli_Chr03	36004638	(C/T)
4672	CakSNP4672	*Kabuli*	Ca_Kabuli_Chr03	36004663	(T/C)
4673	CakSNP4673	*Kabuli*	Ca_Kabuli_Chr03	36004668	(A/G)

S.N.	SNP IDs	Cultivars	Chromosomes/scaffolds	Physical positions (bp)	SNPs
4674	CakSNP4674	*Kabuli*	Ca_Kabuli_Chr03	36046759	(G/A)
4675	CakSNP4675	*Kabuli*	Ca_Kabuli_Chr03	36046938	(C/T)
4676	CakSNP4676	*Kabuli*	Ca_Kabuli_Chr03	36046956	(A/C)
4677	CakSNP4677	*Kabuli*	Ca_Kabuli_Chr03	36050259	(T/G)
4678	CakSNP4678	*Kabuli*	Ca_Kabuli_Chr03	36050319	(T/C)
4679	CakSNP4679	*Kabuli*	Ca_Kabuli_Chr03	36071033	(A/G)
4680	CakSNP4680	*Kabuli*	Ca_Kabuli_Chr03	36085966	(G/A)
4681	CakSNP4681	*Kabuli*	Ca_Kabuli_Chr03	36117521	(A/G)
4682	CakSNP4682	*Kabuli*	Ca_Kabuli_Chr03	36118120	(T/C)
4683	CakSNP4683	*Kabuli*	Ca_Kabuli_Chr03	36118156	(C/T)
4684	CakSNP4684	*Kabuli*	Ca_Kabuli_Chr03	36119592	(T/C)
4685	CakSNP4685	*Kabuli*	Ca_Kabuli_Chr03	36119602	(C/A)
4686	CakSNP4686	*Kabuli*	Ca_Kabuli_Chr03	36130561	(T/A)
4687	CakSNP4687	*Kabuli*	Ca_Kabuli_Chr03	36130610	(C/A)
4688	CakSNP4688	*Kabuli*	Ca_Kabuli_Chr03	36132440	(T/C)
4689	CakSNP4689	*Kabuli*	Ca_Kabuli_Chr03	36132437	(C/T)
4690	CakSNP4690	*Kabuli*	Ca_Kabuli_Chr03	36132401	(C/T)
4691	CakSNP4691	*Kabuli*	Ca_Kabuli_Chr03	36132480	(C/T)
4692	CakSNP4692	*Kabuli*	Ca_Kabuli_Chr03	36132539	(C/T)
4693	CakSNP4693	*Kabuli*	Ca_Kabuli_Chr03	36132586	(T/G)
4694	CakSNP4694	*Kabuli*	Ca_Kabuli_Chr03	36132570	(G/A)
4695	CakSNP4695	*Kabuli*	Ca_Kabuli_Chr03	36132554	(A/T)
4696	CakSNP4696	*Kabuli*	Ca_Kabuli_Chr03	36150722	(G/A)
4697	CakSNP4697	*Kabuli*	Ca_Kabuli_Chr03	36150717	(T/C)
4698	CakSNP4698	*Kabuli*	Ca_Kabuli_Chr03	36164863	(T/G)
4699	CakSNP4699	*Kabuli*	Ca_Kabuli_Chr03	36164866	(T/C)
4700	CakSNP4700	*Kabuli*	Ca_Kabuli_Chr03	36165997	(C/G)
4701	CakSNP4701	*Kabuli*	Ca_Kabuli_Chr03	36167136	(G/T)
4702	CakSNP4702	*Kabuli*	Ca_Kabuli_Chr03	36175164	(G/A)
4703	CakSNP4703	*Kabuli*	Ca_Kabuli_Chr03	36177201	(T/C)
4704	CakSNP4704	*Kabuli*	Ca_Kabuli_Chr03	36205806	(T/G)
4705	CakSNP4705	*Kabuli*	Ca_Kabuli_Chr03	36205819	(A/G)
4706	CakSNP4706	*Kabuli*	Ca_Kabuli_Chr03	36205845	(A/G)
4707	CakSNP4707	*Kabuli*	Ca_Kabuli_Chr03	36206615	(A/C)
4708	CakSNP4708	*Kabuli*	Ca_Kabuli_Chr03	36212799	(A/T)
4709	CakSNP4709	*Kabuli*	Ca_Kabuli_Chr03	36212809	(G/A)
4710	CakSNP4710	*Kabuli*	Ca_Kabuli_Chr03	36212843	(T/A)
4711	CakSNP4711	*Kabuli*	Ca_Kabuli_Chr03	36217686	(G/A)

S.N.	SNP IDs	Cultivars	Chromosomes/scaffolds	Physical positions (bp)	SNPs
4712	CakSNP4712	*Kabuli*	Ca_Kabuli_Chr03	36227565	(A/T)
4713	CakSNP4713	*Kabuli*	Ca_Kabuli_Chr03	36227566	(A/T)
4714	CakSNP4714	*Kabuli*	Ca_Kabuli_Chr03	36227948	(A/G)
4715	CakSNP4715	*Kabuli*	Ca_Kabuli_Chr03	36227949	(C/T)
4716	CakSNP4716	*Kabuli*	Ca_Kabuli_Chr03	36228078	(G/A)
4717	CakSNP4717	*Kabuli*	Ca_Kabuli_Chr03	36228032	(G/T)
4718	CakSNP4718	*Kabuli*	Ca_Kabuli_Chr03	36228121	(T/A)
4719	CakSNP4719	*Kabuli*	Ca_Kabuli_Chr03	36228126	(T/A)
4720	CakSNP4720	*Kabuli*	Ca_Kabuli_Chr03	36242506	(C/T)
4721	CakSNP4721	*Kabuli*	Ca_Kabuli_Chr03	36242504	(C/T)
4722	CakSNP4722	*Kabuli*	Ca_Kabuli_Chr03	36242490	(G/T)
4723	CakSNP4723	*Kabuli*	Ca_Kabuli_Chr03	36242480	(G/C)
4724	CakSNP4724	*Kabuli*	Ca_Kabuli_Chr03	36247792	(T/C)
4725	CakSNP4725	*Kabuli*	Ca_Kabuli_Chr03	36260865	(C/A)
4726	CakSNP4726	*Kabuli*	Ca_Kabuli_Chr03	36282091	(T/G)
4727	CakSNP4727	*Kabuli*	Ca_Kabuli_Chr03	36293589	(A/G)
4728	CakSNP4728	*Kabuli*	Ca_Kabuli_Chr03	36293625	(C/T)
4729	CakSNP4729	*Kabuli*	Ca_Kabuli_Chr03	36293637	(C/T)
4730	CakSNP4730	*Kabuli*	Ca_Kabuli_Chr03	36330750	(A/G)
4731	CakSNP4731	*Kabuli*	Ca_Kabuli_Chr03	36330815	(T/C)
4732	CakSNP4732	*Kabuli*	Ca_Kabuli_Chr03	36345158	(T/C)
4733	CakSNP4733	*Kabuli*	Ca_Kabuli_Chr03	36347203	(T/A)
4734	CakSNP4734	*Kabuli*	Ca_Kabuli_Chr03	36361339	(C/T)
4735	CakSNP4735	*Kabuli*	Ca_Kabuli_Chr03	36378294	(G/T)
4736	CakSNP4736	*Kabuli*	Ca_Kabuli_Chr03	36378299	(C/G)
4737	CakSNP4737	*Kabuli*	Ca_Kabuli_Chr03	36378358	(G/A)
4738	CakSNP4738	*Kabuli*	Ca_Kabuli_Chr03	36387615	(A/T)
4739	CakSNP4739	*Kabuli*	Ca_Kabuli_Chr03	36387732	(G/A)
4740	CakSNP4740	*Kabuli*	Ca_Kabuli_Chr03	36387718	(C/T)
4741	CakSNP4741	*Kabuli*	Ca_Kabuli_Chr03	36396559	(C/A)
4742	CakSNP4742	*Kabuli*	Ca_Kabuli_Chr03	36420902	(T/C)
4743	CakSNP4743	*Kabuli*	Ca_Kabuli_Chr03	36432389	(A/G)
4744	CakSNP4744	*Kabuli*	Ca_Kabuli_Chr03	36447274	(G/A)
4745	CakSNP4745	*Kabuli*	Ca_Kabuli_Chr03	36447364	(T/A)
4746	CakSNP4746	*Kabuli*	Ca_Kabuli_Chr03	36447520	(T/A)
4747	CakSNP4747	*Kabuli*	Ca_Kabuli_Chr03	36447525	(C/T)
4748	CakSNP4748	*Kabuli*	Ca_Kabuli_Chr03	36488722	(T/A)
4749	CakSNP4749	*Kabuli*	Ca_Kabuli_Chr03	36488736	(T/C)

S.N.	SNP IDs	Cultivars	Chromosomes/scaffolds	Physical positions (bp)	SNPs
4750	CakSNP4750	*Kabuli*	Ca_Kabuli_Chr03	36488853	(A/G)
4751	CakSNP4751	*Kabuli*	Ca_Kabuli_Chr03	36488890	(A/G)
4752	CakSNP4752	*Kabuli*	Ca_Kabuli_Chr03	36503906	(C/T)
4753	CakSNP4753	*Kabuli*	Ca_Kabuli_Chr03	36503912	(T/C)
4754	CakSNP4754	*Kabuli*	Ca_Kabuli_Chr03	36524598	(G/C)
4755	CakSNP4755	*Kabuli*	Ca_Kabuli_Chr03	36524635	(A/G)
4756	CakSNP4756	*Kabuli*	Ca_Kabuli_Chr03	36524659	(T/C)
4757	CakSNP4757	*Kabuli*	Ca_Kabuli_Chr03	36531151	(C/A)
4758	CakSNP4758	*Kabuli*	Ca_Kabuli_Chr03	36588856	(C/T)
4759	CakSNP4759	*Kabuli*	Ca_Kabuli_Chr03	36588896	(C/G)
4760	CakSNP4760	*Kabuli*	Ca_Kabuli_Chr03	36606527	(G/A)
4761	CakSNP4761	*Kabuli*	Ca_Kabuli_Chr03	36609043	(G/A)
4762	CakSNP4762	*Kabuli*	Ca_Kabuli_Chr03	36616855	(T/C)
4763	CakSNP4763	*Kabuli*	Ca_Kabuli_Chr03	36644117	(A/T)
4764	CakSNP4764	*Kabuli*	Ca_Kabuli_Chr03	36733097	(G/A)
4765	CakSNP4765	*Kabuli*	Ca_Kabuli_Chr03	36733157	(A/G)
4766	CakSNP4766	*Kabuli*	Ca_Kabuli_Chr03	36733211	(A/G)
4767	CakSNP4767	*Kabuli*	Ca_Kabuli_Chr03	36749775	(T/A)
4768	CakSNP4768	*Kabuli*	Ca_Kabuli_Chr03	36749826	(G/A)
4769	CakSNP4769	*Kabuli*	Ca_Kabuli_Chr03	36749829	(T/C)
4770	CakSNP4770	*Kabuli*	Ca_Kabuli_Chr03	36749831	(T/C)
4771	CakSNP4771	*Kabuli*	Ca_Kabuli_Chr03	36749871	(T/C)
4772	CakSNP4772	*Kabuli*	Ca_Kabuli_Chr03	36749904	(C/T)
4773	CakSNP4773	*Kabuli*	Ca_Kabuli_Chr03	36749916	(C/A)
4774	CakSNP4774	*Kabuli*	Ca_Kabuli_Chr03	36752689	(T/G)
4775	CakSNP4775	*Kabuli*	Ca_Kabuli_Chr03	36769775	(G/T)
4776	CakSNP4776	*Kabuli*	Ca_Kabuli_Chr03	36783494	(T/A)
4777	CakSNP4777	*Kabuli*	Ca_Kabuli_Chr03	36825463	(T/C)
4778	CakSNP4778	*Kabuli*	Ca_Kabuli_Chr03	36860134	(A/G)
4779	CakSNP4779	*Kabuli*	Ca_Kabuli_Chr03	36860246	(T/C)
4780	CakSNP4780	*Kabuli*	Ca_Kabuli_Chr03	36873720	(T/C)
4781	CakSNP4781	*Kabuli*	Ca_Kabuli_Chr03	36883268	(T/C)
4782	CakSNP4782	*Kabuli*	Ca_Kabuli_Chr03	36883333	(A/T)
4783	CakSNP4783	*Kabuli*	Ca_Kabuli_Chr03	36895362	(T/C)
4784	CakSNP4784	*Kabuli*	Ca_Kabuli_Chr03	36938148	(A/G)
4785	CakSNP4785	*Kabuli*	Ca_Kabuli_Chr03	36938271	(G/C)
4786	CakSNP4786	*Kabuli*	Ca_Kabuli_Chr03	36943522	(A/G)
4787	CakSNP4787	*Kabuli*	Ca_Kabuli_Chr03	36991718	(C/A)

S.N.	SNP IDs	Cultivars	Chromosomes/scaffolds	Physical positions (bp)	SNPs
4788	CakSNP4788	*Kabuli*	Ca_Kabuli_Chr03	37003976	(C/T)
4789	CakSNP4789	*Kabuli*	Ca_Kabuli_Chr03	37004026	(A/G)
4790	CakSNP4790	*Kabuli*	Ca_Kabuli_Chr03	37022354	(A/G)
4791	CakSNP4791	*Kabuli*	Ca_Kabuli_Chr03	37034618	(G/A)
4792	CakSNP4792	*Kabuli*	Ca_Kabuli_Chr03	37067095	(T/G)
4793	CakSNP4793	*Kabuli*	Ca_Kabuli_Chr03	37072289	(A/G)
4794	CakSNP4794	*Kabuli*	Ca_Kabuli_Chr03	37072276	(T/G)
4795	CakSNP4795	*Kabuli*	Ca_Kabuli_Chr03	37072312	(T/C)
4796	CakSNP4796	*Kabuli*	Ca_Kabuli_Chr03	37077087	(G/T)
4797	CakSNP4797	*Kabuli*	Ca_Kabuli_Chr03	37090968	(T/C)
4798	CakSNP4798	*Kabuli*	Ca_Kabuli_Chr03	37091004	(A/G)
4799	CakSNP4799	*Kabuli*	Ca_Kabuli_Chr03	37094437	(T/C)
4800	CakSNP4800	*Kabuli*	Ca_Kabuli_Chr03	37100897	(G/T)
4801	CakSNP4801	*Kabuli*	Ca_Kabuli_Chr03	37101339	(A/T)
4802	CakSNP4802	*Kabuli*	Ca_Kabuli_Chr03	37101433	(T/G)
4803	CakSNP4803	*Kabuli*	Ca_Kabuli_Chr03	37101409	(A/G)
4804	CakSNP4804	*Kabuli*	Ca_Kabuli_Chr03	37108762	(A/G)
4805	CakSNP4805	*Kabuli*	Ca_Kabuli_Chr03	37108908	(A/G)
4806	CakSNP4806	*Kabuli*	Ca_Kabuli_Chr03	37108904	(A/T)
4807	CakSNP4807	*Kabuli*	Ca_Kabuli_Chr03	37108876	(C/A)
4808	CakSNP4808	*Kabuli*	Ca_Kabuli_Chr03	37110070	(A/G)
4809	CakSNP4809	*Kabuli*	Ca_Kabuli_Chr03	37110124	(A/T)
4810	CakSNP4810	*Kabuli*	Ca_Kabuli_Chr03	37146766	(C/G)
4811	CakSNP4811	*Kabuli*	Ca_Kabuli_Chr03	37146840	(A/G)
4812	CakSNP4812	*Kabuli*	Ca_Kabuli_Chr03	37154761	(T/G)
4813	CakSNP4813	*Kabuli*	Ca_Kabuli_Chr03	37154857	(T/C)
4814	CakSNP4814	*Kabuli*	Ca_Kabuli_Chr03	37154938	(A/C)
4815	CakSNP4815	*Kabuli*	Ca_Kabuli_Chr03	37154957	(C/A)
4816	CakSNP4816	*Kabuli*	Ca_Kabuli_Chr03	37155925	(G/A)
4817	CakSNP4817	*Kabuli*	Ca_Kabuli_Chr03	37156951	(A/G)
4818	CakSNP4818	*Kabuli*	Ca_Kabuli_Chr03	37160822	(G/T)
4819	CakSNP4819	*Kabuli*	Ca_Kabuli_Chr03	37160939	(A/G)
4820	CakSNP4820	*Kabuli*	Ca_Kabuli_Chr03	37164633	(T/C)
4821	CakSNP4821	*Kabuli*	Ca_Kabuli_Chr03	37170249	(C/T)
4822	CakSNP4822	*Kabuli*	Ca_Kabuli_Chr03	37271869	(T/C)
4823	CakSNP4823	*Kabuli*	Ca_Kabuli_Chr03	37300206	(T/G)
4824	CakSNP4824	*Kabuli*	Ca_Kabuli_Chr03	37303478	(G/A)
4825	CakSNP4825	*Kabuli*	Ca_Kabuli_Chr03	37308614	(T/C)

S.N.	SNP IDs	Cultivars	Chromosomes/scaffolds	Physical positions (bp)	SNPs
4826	CakSNP4826	*Kabuli*	Ca_Kabuli_Chr03	37309420	(A/C)
4827	CakSNP4827	*Kabuli*	Ca_Kabuli_Chr03	37316736	(T/C)
4828	CakSNP4828	*Kabuli*	Ca_Kabuli_Chr03	37316737	(C/T)
4829	CakSNP4829	*Kabuli*	Ca_Kabuli_Chr03	37316782	(G/A)
4830	CakSNP4830	*Kabuli*	Ca_Kabuli_Chr03	37355013	(A/G)
4831	CakSNP4831	*Kabuli*	Ca_Kabuli_Chr03	37444451	(C/A)
4832	CakSNP4832	*Kabuli*	Ca_Kabuli_Chr03	37566557	(C/A)
4833	CakSNP4833	*Kabuli*	Ca_Kabuli_Chr03	37599142	(T/C)
4834	CakSNP4834	*Kabuli*	Ca_Kabuli_Chr03	37604672	(T/A)
4835	CakSNP4835	*Kabuli*	Ca_Kabuli_Chr03	37630502	(T/C)
4836	CakSNP4836	*Kabuli*	Ca_Kabuli_Chr03	37635909	(G/A)
4837	CakSNP4837	*Kabuli*	Ca_Kabuli_Chr03	37635961	(C/T)
4838	CakSNP4838	*Kabuli*	Ca_Kabuli_Chr03	37639300	(A/G)
4839	CakSNP4839	*Kabuli*	Ca_Kabuli_Chr03	37682816	(T/C)
4840	CakSNP4840	*Kabuli*	Ca_Kabuli_Chr03	37682824	(G/A)
4841	CakSNP4841	*Kabuli*	Ca_Kabuli_Chr03	37723959	(C/T)
4842	CakSNP4842	*Kabuli*	Ca_Kabuli_Chr03	37759329	(G/A)
4843	CakSNP4843	*Kabuli*	Ca_Kabuli_Chr03	37765767	(A/C)
4844	CakSNP4844	*Kabuli*	Ca_Kabuli_Chr03	37806690	(G/A)
4845	CakSNP4845	*Kabuli*	Ca_Kabuli_Chr03	37814955	(A/T)
4846	CakSNP4846	*Kabuli*	Ca_Kabuli_Chr03	37814954	(T/A)
4847	CakSNP4847	*Kabuli*	Ca_Kabuli_Chr03	37816895	(T/A)
4848	CakSNP4848	*Kabuli*	Ca_Kabuli_Chr03	37859943	(T/C)
4849	CakSNP4849	*Kabuli*	Ca_Kabuli_Chr03	37864390	(C/T)
4850	CakSNP4850	*Kabuli*	Ca_Kabuli_Chr03	37866258	(A/G)
4851	CakSNP4851	*Kabuli*	Ca_Kabuli_Chr03	37886983	(G/T)
4852	CakSNP4852	*Kabuli*	Ca_Kabuli_Chr03	37886971	(A/G)
4853	CakSNP4853	*Kabuli*	Ca_Kabuli_Chr03	37886942	(A/C)
4854	CakSNP4854	*Kabuli*	Ca_Kabuli_Chr03	37908317	(T/C)
4855	CakSNP4855	*Kabuli*	Ca_Kabuli_Chr03	37908492	(A/C)
4856	CakSNP4856	*Kabuli*	Ca_Kabuli_Chr03	37910881	(A/G)
4857	CakSNP4857	*Kabuli*	Ca_Kabuli_Chr03	37910899	(T/C)
4858	CakSNP4858	*Kabuli*	Ca_Kabuli_Chr03	37910911	(C/T)
4859	CakSNP4859	*Kabuli*	Ca_Kabuli_Chr03	37913266	(C/T)
4860	CakSNP4860	*Kabuli*	Ca_Kabuli_Chr03	37919880	(C/T)
4861	CakSNP4861	*Kabuli*	Ca_Kabuli_Chr03	37942807	(A/G)
4862	CakSNP4862	*Kabuli*	Ca_Kabuli_Chr03	37942962	(T/C)
4863	CakSNP4863	*Kabuli*	Ca_Kabuli_Chr03	37985612	(A/C)

S.N.	SNP IDs	Cultivars	Chromosomes/scaffolds	Physical positions (bp)	SNPs
4864	CakSNP4864	*Kabuli*	Ca_Kabuli_Chr03	37985652	(C/T)
4865	CakSNP4865	*Kabuli*	Ca_Kabuli_Chr03	37985776	(T/A)
4866	CakSNP4866	*Kabuli*	Ca_Kabuli_Chr03	37985977	(A/G)
4867	CakSNP4867	*Kabuli*	Ca_Kabuli_Chr03	37985947	(A/T)
4868	CakSNP4868	*Kabuli*	Ca_Kabuli_Chr03	38026470	(G/C)
4869	CakSNP4869	*Kabuli*	Ca_Kabuli_Chr03	38026539	(A/G)
4870	CakSNP4870	*Kabuli*	Ca_Kabuli_Chr03	38026734	(G/C)
4871	CakSNP4871	*Kabuli*	Ca_Kabuli_Chr03	38026725	(G/A)
4872	CakSNP4872	*Kabuli*	Ca_Kabuli_Chr03	38029095	(G/C)
4873	CakSNP4873	*Kabuli*	Ca_Kabuli_Chr03	38033965	(G/A)
4874	CakSNP4874	*Kabuli*	Ca_Kabuli_Chr03	38033969	(A/G)
4875	CakSNP4875	*Kabuli*	Ca_Kabuli_Chr03	38046510	(T/C)
4876	CakSNP4876	*Kabuli*	Ca_Kabuli_Chr03	38046575	(A/C)
4877	CakSNP4877	*Kabuli*	Ca_Kabuli_Chr03	38049996	(A/G)
4878	CakSNP4878	*Kabuli*	Ca_Kabuli_Chr03	38059303	(T/C)
4879	CakSNP4879	*Kabuli*	Ca_Kabuli_Chr03	38059311	(T/A)
4880	CakSNP4880	*Kabuli*	Ca_Kabuli_Chr03	38060814	(A/G)
4881	CakSNP4881	*Kabuli*	Ca_Kabuli_Chr03	38060755	(T/C)
4882	CakSNP4882	*Kabuli*	Ca_Kabuli_Chr03	38060742	(C/T)
4883	CakSNP4883	*Kabuli*	Ca_Kabuli_Chr03	38074259	(C/A)
4884	CakSNP4884	*Kabuli*	Ca_Kabuli_Chr03	38103375	(T/C)
4885	CakSNP4885	*Kabuli*	Ca_Kabuli_Chr03	38103378	(C/T)
4886	CakSNP4886	*Kabuli*	Ca_Kabuli_Chr03	38103381	(C/T)
4887	CakSNP4887	*Kabuli*	Ca_Kabuli_Chr03	38103385	(C/T)
4888	CakSNP4888	*Kabuli*	Ca_Kabuli_Chr03	38103524	(C/T)
4889	CakSNP4889	*Kabuli*	Ca_Kabuli_Chr03	38112801	(C/T)
4890	CakSNP4890	*Kabuli*	Ca_Kabuli_Chr03	38112802	(T/C)
4891	CakSNP4891	*Kabuli*	Ca_Kabuli_Chr03	38112971	(A/G)
4892	CakSNP4892	*Kabuli*	Ca_Kabuli_Chr03	38117169	(T/C)
4893	CakSNP4893	*Kabuli*	Ca_Kabuli_Chr03	38130415	(T/C)
4894	CakSNP4894	*Kabuli*	Ca_Kabuli_Chr03	38130410	(A/G)
4895	CakSNP4895	*Kabuli*	Ca_Kabuli_Chr03	38145115	(C/T)
4896	CakSNP4896	*Kabuli*	Ca_Kabuli_Chr03	38157519	(T/C)
4897	CakSNP4897	*Kabuli*	Ca_Kabuli_Chr03	38166074	(A/G)
4898	CakSNP4898	*Kabuli*	Ca_Kabuli_Chr03	38166881	(C/T)
4899	CakSNP4899	*Kabuli*	Ca_Kabuli_Chr03	38202223	(C/T)
4900	CakSNP4900	*Kabuli*	Ca_Kabuli_Chr03	38220910	(T/C)
4901	CakSNP4901	*Kabuli*	Ca_Kabuli_Chr03	38220949	(A/T)

S.N.	SNP IDs	Cultivars	Chromosomes/scaffolds	Physical positions (bp)	SNPs
4902	CakSNP4902	*Kabuli*	Ca_Kabuli_Chr03	38220958	(A/G)
4903	CakSNP4903	*Kabuli*	Ca_Kabuli_Chr03	38222750	(A/C)
4904	CakSNP4904	*Kabuli*	Ca_Kabuli_Chr03	38222747	(G/A)
4905	CakSNP4905	*Kabuli*	Ca_Kabuli_Chr03	38222733	(C/G)
4906	CakSNP4906	*Kabuli*	Ca_Kabuli_Chr03	38228118	(G/T)
4907	CakSNP4907	*Kabuli*	Ca_Kabuli_Chr03	38237246	(T/G)
4908	CakSNP4908	*Kabuli*	Ca_Kabuli_Chr03	38251660	(C/A)
4909	CakSNP4909	*Kabuli*	Ca_Kabuli_Chr03	38253654	(T/C)
4910	CakSNP4910	*Kabuli*	Ca_Kabuli_Chr03	38258645	(C/A)
4911	CakSNP4911	*Kabuli*	Ca_Kabuli_Chr03	38264250	(C/T)
4912	CakSNP4912	*Kabuli*	Ca_Kabuli_Chr03	38294286	(C/A)
4913	CakSNP4913	*Kabuli*	Ca_Kabuli_Chr03	38296573	(A/G)
4914	CakSNP4914	*Kabuli*	Ca_Kabuli_Chr03	38315560	(G/T)
4915	CakSNP4915	*Kabuli*	Ca_Kabuli_Chr03	38321159	(T/C)
4916	CakSNP4916	*Kabuli*	Ca_Kabuli_Chr03	38321153	(G/T)
4917	CakSNP4917	*Kabuli*	Ca_Kabuli_Chr03	38386392	(T/C)
4918	CakSNP4918	*Kabuli*	Ca_Kabuli_Chr03	38424238	(A/T)
4919	CakSNP4919	*Kabuli*	Ca_Kabuli_Chr03	38452060	(A/C)
4920	CakSNP4920	*Kabuli*	Ca_Kabuli_Chr03	38465366	(G/A)
4921	CakSNP4921	*Kabuli*	Ca_Kabuli_Chr03	38465335	(G/A)
4922	CakSNP4922	*Kabuli*	Ca_Kabuli_Chr03	38465440	(G/A)
4923	CakSNP4923	*Kabuli*	Ca_Kabuli_Chr03	38496236	(A/G)
4924	CakSNP4924	*Kabuli*	Ca_Kabuli_Chr03	38501705	(T/C)
4925	CakSNP4925	*Kabuli*	Ca_Kabuli_Chr03	38539721	(T/G)
4926	CakSNP4926	*Kabuli*	Ca_Kabuli_Chr03	38551142	(C/T)
4927	CakSNP4927	*Kabuli*	Ca_Kabuli_Chr03	38555941	(G/A)
4928	CakSNP4928	*Kabuli*	Ca_Kabuli_Chr03	38555944	(A/G)
4929	CakSNP4929	*Kabuli*	Ca_Kabuli_Chr03	38592077	(G/A)
4930	CakSNP4930	*Kabuli*	Ca_Kabuli_Chr03	38624786	(C/T)
4931	CakSNP4931	*Kabuli*	Ca_Kabuli_Chr03	38624934	(T/C)
4932	CakSNP4932	*Kabuli*	Ca_Kabuli_Chr03	38672863	(C/A)
4933	CakSNP4933	*Kabuli*	Ca_Kabuli_Chr03	38704356	(C/T)
4934	CakSNP4934	*Kabuli*	Ca_Kabuli_Chr03	38728528	(C/T)
4935	CakSNP4935	*Kabuli*	Ca_Kabuli_Chr03	38728679	(T/C)
4936	CakSNP4936	*Kabuli*	Ca_Kabuli_Chr03	38731315	(C/T)
4937	CakSNP4937	*Kabuli*	Ca_Kabuli_Chr03	38731466	(T/G)
4938	CakSNP4938	*Kabuli*	Ca_Kabuli_Chr03	38764372	(G/A)
4939	CakSNP4939	*Kabuli*	Ca_Kabuli_Chr03	38764390	(A/C)

S.N.	SNP IDs	Cultivars	Chromosomes/scaffolds	Physical positions (bp)	SNPs
4940	CakSNP4940	*Kabuli*	Ca_Kabuli_Chr03	38766156	(T/A)
4941	CakSNP4941	*Kabuli*	Ca_Kabuli_Chr03	38766174	(A/G)
4942	CakSNP4942	*Kabuli*	Ca_Kabuli_Chr03	38766311	(A/C)
4943	CakSNP4943	*Kabuli*	Ca_Kabuli_Chr03	38771817	(A/G)
4944	CakSNP4944	*Kabuli*	Ca_Kabuli_Chr03	38776366	(T/G)
4945	CakSNP4945	*Kabuli*	Ca_Kabuli_Chr03	38785843	(C/G)
4946	CakSNP4946	*Kabuli*	Ca_Kabuli_Chr03	38785845	(C/T)
4947	CakSNP4947	*Kabuli*	Ca_Kabuli_Chr03	38793194	(T/C)
4948	CakSNP4948	*Kabuli*	Ca_Kabuli_Chr03	38805822	(T/A)
4949	CakSNP4949	*Kabuli*	Ca_Kabuli_Chr03	38805831	(A/C)
4950	CakSNP4950	*Kabuli*	Ca_Kabuli_Chr03	38809132	(G/A)
4951	CakSNP4951	*Kabuli*	Ca_Kabuli_Chr03	38810765	(A/T)
4952	CakSNP4952	*Kabuli*	Ca_Kabuli_Chr03	38818340	(A/G)
4953	CakSNP4953	*Kabuli*	Ca_Kabuli_Chr03	38821990	(A/G)
4954	CakSNP4954	*Kabuli*	Ca_Kabuli_Chr03	38831533	(T/C)
4955	CakSNP4955	*Kabuli*	Ca_Kabuli_Chr03	38848358	(C/T)
4956	CakSNP4956	*Kabuli*	Ca_Kabuli_Chr03	38848412	(C/G)
4957	CakSNP4957	*Kabuli*	Ca_Kabuli_Chr03	38878577	(C/G)
4958	CakSNP4958	*Kabuli*	Ca_Kabuli_Chr03	38878664	(G/C)
4959	CakSNP4959	*Kabuli*	Ca_Kabuli_Chr03	38878649	(T/C)
4960	CakSNP4960	*Kabuli*	Ca_Kabuli_Chr03	38878736	(G/C)
4961	CakSNP4961	*Kabuli*	Ca_Kabuli_Chr03	38887640	(C/T)
4962	CakSNP4962	*Kabuli*	Ca_Kabuli_Chr03	38891567	(T/C)
4963	CakSNP4963	*Kabuli*	Ca_Kabuli_Chr03	38896013	(A/G)
4964	CakSNP4964	*Kabuli*	Ca_Kabuli_Chr03	38913449	(T/C)
4965	CakSNP4965	*Kabuli*	Ca_Kabuli_Chr03	38914528	(A/G)
4966	CakSNP4966	*Kabuli*	Ca_Kabuli_Chr03	38923321	(C/T)
4967	CakSNP4967	*Kabuli*	Ca_Kabuli_Chr03	38951428	(G/A)
4968	CakSNP4968	*Kabuli*	Ca_Kabuli_Chr03	38951698	(A/C)
4969	CakSNP4969	*Kabuli*	Ca_Kabuli_Chr03	38965610	(T/A)
4970	CakSNP4970	*Kabuli*	Ca_Kabuli_Chr03	38982481	(A/G)
4971	CakSNP4971	*Kabuli*	Ca_Kabuli_Chr03	38987810	(G/A)
4972	CakSNP4972	*Kabuli*	Ca_Kabuli_Chr03	38987916	(A/C)
4973	CakSNP4973	*Kabuli*	Ca_Kabuli_Chr03	39003771	(G/A)
4974	CakSNP4974	*Kabuli*	Ca_Kabuli_Chr03	39013988	(T/G)
4975	CakSNP4975	*Kabuli*	Ca_Kabuli_Chr03	39029251	(A/C)
4976	CakSNP4976	*Kabuli*	Ca_Kabuli_Chr03	39034246	(C/T)
4977	CakSNP4977	*Kabuli*	Ca_Kabuli_Chr03	39034433	(A/G)

S.N.	SNP IDs	Cultivars	Chromosomes/scaffolds	Physical positions (bp)	SNPs
4978	CakSNP4978	*Kabuli*	Ca_Kabuli_Chr03	39036130	(T/C)
4979	CakSNP4979	*Kabuli*	Ca_Kabuli_Chr03	39052024	(T/C)
4980	CakSNP4980	*Kabuli*	Ca_Kabuli_Chr03	39055529	(C/T)
4981	CakSNP4981	*Kabuli*	Ca_Kabuli_Chr03	39055455	(T/C)
4982	CakSNP4982	*Kabuli*	Ca_Kabuli_Chr03	39081040	(A/G)
4983	CakSNP4983	*Kabuli*	Ca_Kabuli_Chr03	39084916	(T/A)
4984	CakSNP4984	*Kabuli*	Ca_Kabuli_Chr03	39084979	(A/C)
4985	CakSNP4985	*Kabuli*	Ca_Kabuli_Chr03	39093242	(G/A)
4986	CakSNP4986	*Kabuli*	Ca_Kabuli_Chr03	39093243	(C/A)
4987	CakSNP4987	*Kabuli*	Ca_Kabuli_Chr03	39093245	(T/A)
4988	CakSNP4988	*Kabuli*	Ca_Kabuli_Chr03	39093215	(G/A)
4989	CakSNP4989	*Kabuli*	Ca_Kabuli_Chr03	39115274	(A/C)
4990	CakSNP4990	*Kabuli*	Ca_Kabuli_Chr03	39115986	(C/G)
4991	CakSNP4991	*Kabuli*	Ca_Kabuli_Chr03	39115979	(A/G)
4992	CakSNP4992	*Kabuli*	Ca_Kabuli_Chr03	39115972	(T/A)
4993	CakSNP4993	*Kabuli*	Ca_Kabuli_Chr03	39115970	(T/G)
4994	CakSNP4994	*Kabuli*	Ca_Kabuli_Chr03	39118510	(C/T)
4995	CakSNP4995	*Kabuli*	Ca_Kabuli_Chr03	39200823	(C/A)
4996	CakSNP4996	*Kabuli*	Ca_Kabuli_Chr03	39200847	(A/C)
4997	CakSNP4997	*Kabuli*	Ca_Kabuli_Chr03	39202479	(G/C)
4998	CakSNP4998	*Kabuli*	Ca_Kabuli_Chr03	39202481	(A/T)
4999	CakSNP4999	*Kabuli*	Ca_Kabuli_Chr03	39207112	(A/C)
5000	CakSNP5000	*Kabuli*	Ca_Kabuli_Chr03	39215327	(C/T)
5001	CakSNP5001	*Kabuli*	Ca_Kabuli_Chr03	39215476	(G/A)
5002	CakSNP5002	*Kabuli*	Ca_Kabuli_Chr03	39215558	(C/T)
5003	CakSNP5003	*Kabuli*	Ca_Kabuli_Chr03	39233774	(A/T)
5004	CakSNP5004	*Kabuli*	Ca_Kabuli_Chr03	39233852	(A/C)
5005	CakSNP5005	*Kabuli*	Ca_Kabuli_Chr03	39233885	(A/T)
5006	CakSNP5006	*Kabuli*	Ca_Kabuli_Chr03	39234161	(A/G)
5007	CakSNP5007	*Kabuli*	Ca_Kabuli_Chr03	39254527	(T/G)
5008	CakSNP5008	*Kabuli*	Ca_Kabuli_Chr03	39254532	(C/G)
5009	CakSNP5009	*Kabuli*	Ca_Kabuli_Chr03	39255721	(A/C)
5010	CakSNP5010	*Kabuli*	Ca_Kabuli_Chr03	39255715	(G/A)
5011	CakSNP5011	*Kabuli*	Ca_Kabuli_Chr03	39255713	(C/A)
5012	CakSNP5012	*Kabuli*	Ca_Kabuli_Chr03	39255709	(A/T)
5013	CakSNP5013	*Kabuli*	Ca_Kabuli_Chr03	39255729	(C/T)
5014	CakSNP5014	*Kabuli*	Ca_Kabuli_Chr03	39255792	(C/T)
5015	CakSNP5015	*Kabuli*	Ca_Kabuli_Chr03	39257226	(A/G)

S.N.	SNP IDs	Cultivars	Chromosomes/scaffolds	Physical positions (bp)	SNPs
5016	CakSNP5016	*Kabuli*	Ca_Kabuli_Chr03	39258671	(A/C)
5017	CakSNP5017	*Kabuli*	Ca_Kabuli_Chr03	39265412	(A/C)
5018	CakSNP5018	*Kabuli*	Ca_Kabuli_Chr03	39289840	(T/A)
5019	CakSNP5019	*Kabuli*	Ca_Kabuli_Chr03	39303386	(C/G)
5020	CakSNP5020	*Kabuli*	Ca_Kabuli_Chr03	39303416	(G/T)
5021	CakSNP5021	*Kabuli*	Ca_Kabuli_Chr03	39303407	(C/A)
5022	CakSNP5022	*Kabuli*	Ca_Kabuli_Chr03	39323779	(A/G)
5023	CakSNP5023	*Kabuli*	Ca_Kabuli_Chr03	39326387	(C/T)
5024	CakSNP5024	*Kabuli*	Ca_Kabuli_Chr03	39405252	(C/A)
5025	CakSNP5025	*Kabuli*	Ca_Kabuli_Chr03	39405873	(C/G)
5026	CakSNP5026	*Kabuli*	Ca_Kabuli_Chr03	39472291	(G/C)
5027	CakSNP5027	*Kabuli*	Ca_Kabuli_Chr03	39481371	(A/C)
5028	CakSNP5028	*Kabuli*	Ca_Kabuli_Chr03	39485182	(G/A)
5029	CakSNP5029	*Kabuli*	Ca_Kabuli_Chr03	39504242	(A/G)
5030	CakSNP5030	*Kabuli*	Ca_Kabuli_Chr03	39515531	(A/G)
5031	CakSNP5031	*Kabuli*	Ca_Kabuli_Chr03	39515710	(T/A)
5032	CakSNP5032	*Kabuli*	Ca_Kabuli_Chr03	39519640	(A/G)
5033	CakSNP5033	*Kabuli*	Ca_Kabuli_Chr03	39556535	(T/A)
5034	CakSNP5034	*Kabuli*	Ca_Kabuli_Chr03	39587620	(T/G)
5035	CakSNP5035	*Kabuli*	Ca_Kabuli_Chr03	39592340	(C/T)
5036	CakSNP5036	*Kabuli*	Ca_Kabuli_Chr03	39612466	(T/C)
5037	CakSNP5037	*Kabuli*	Ca_Kabuli_Chr03	39612458	(C/A)
5038	CakSNP5038	*Kabuli*	Ca_Kabuli_Chr03	39612447	(C/A)
5039	CakSNP5039	*Kabuli*	Ca_Kabuli_Chr03	39612568	(A/T)
5040	CakSNP5040	*Kabuli*	Ca_Kabuli_Chr03	39628916	(G/A)
5041	CakSNP5041	*Kabuli*	Ca_Kabuli_Chr03	39698060	(T/C)
5042	CakSNP5042	*Kabuli*	Ca_Kabuli_Chr03	39701532	(G/A)
5043	CakSNP5043	*Kabuli*	Ca_Kabuli_Chr03	39722314	(C/T)
5044	CakSNP5044	*Kabuli*	Ca_Kabuli_Chr03	39731437	(A/C)
5045	CakSNP5045	*Kabuli*	Ca_Kabuli_Chr03	39787513	(A/C)
5046	CakSNP5046	*Kabuli*	Ca_Kabuli_Chr03	39787615	(C/T)
5047	CakSNP5047	*Kabuli*	Ca_Kabuli_Chr03	39787716	(A/C)
5048	CakSNP5048	*Kabuli*	Ca_Kabuli_Chr03	39791717	(C/T)
5049	CakSNP5049	*Kabuli*	Ca_Kabuli_Chr03	39793539	(G/A)
5050	CakSNP5050	*Kabuli*	Ca_Kabuli_Chr03	39794672	(C/T)
5051	CakSNP5051	*Kabuli*	Ca_Kabuli_Chr03	39794639	(C/G)
5052	CakSNP5052	*Kabuli*	Ca_Kabuli_Chr03	39801213	(A/G)
5053	CakSNP5053	*Kabuli*	Ca_Kabuli_Chr03	39801242	(C/T)

S.N.	SNP IDs	Cultivars	Chromosomes/scaffolds	Physical positions (bp)	SNPs
5054	CakSNP5054	*Kabuli*	Ca_Kabuli_Chr03	39817122	(G/A)
5055	CakSNP5055	*Kabuli*	Ca_Kabuli_Chr03	39839763	(T/G)
5056	CakSNP5056	*Kabuli*	Ca_Kabuli_Chr03	39891075	(G/A)
5057	CakSNP5057	*Kabuli*	Ca_Kabuli_Chr03	39890991	(A/T)
5058	CakSNP5058	*Kabuli*	Ca_Kabuli_Chr03	39896395	(A/G)
5059	CakSNP5059	*Kabuli*	Ca_Kabuli_Chr03	39908983	(C/G)
5060	CakSNP5060	*Kabuli*	Ca_Kabuli_Chr03	39909824	(C/G)
5061	CakSNP5061	*Kabuli*	Ca_Kabuli_Chr03	39909839	(T/C)
5062	CakSNP5062	*Kabuli*	Ca_Kabuli_Chr03	39914344	(T/G)
5063	CakSNP5063	*Kabuli*	Ca_Kabuli_Chr03	39941101	(T/G)
5064	CakSNP5064	*Kabuli*	Ca_Kabuli_Chr03	39941090	(G/A)
5065	CakSNP5065	*Kabuli*	Ca_Kabuli_Chr04	20297	(A/G)
5066	CakSNP5066	*Kabuli*	Ca_Kabuli_Chr04	20495	(G/C)
5067	CakSNP5067	*Kabuli*	Ca_Kabuli_Chr04	167229	(T/G)
5068	CakSNP5068	*Kabuli*	Ca_Kabuli_Chr04	228864	(G/A)
5069	CakSNP5069	*Kabuli*	Ca_Kabuli_Chr04	305558	(C/T)
5070	CakSNP5070	*Kabuli*	Ca_Kabuli_Chr04	335941	(C/T)
5071	CakSNP5071	*Kabuli*	Ca_Kabuli_Chr04	336055	(G/A)
5072	CakSNP5072	*Kabuli*	Ca_Kabuli_Chr04	336050	(T/C)
5073	CakSNP5073	*Kabuli*	Ca_Kabuli_Chr04	336029	(G/A)
5074	CakSNP5074	*Kabuli*	Ca_Kabuli_Chr04	338641	(A/G)
5075	CakSNP5075	*Kabuli*	Ca_Kabuli_Chr04	338721	(C/T)
5076	CakSNP5076	*Kabuli*	Ca_Kabuli_Chr04	338707	(G/T)
5077	CakSNP5077	*Kabuli*	Ca_Kabuli_Chr04	349309	(C/T)
5078	CakSNP5078	*Kabuli*	Ca_Kabuli_Chr04	353069	(C/A)
5079	CakSNP5079	*Kabuli*	Ca_Kabuli_Chr04	353079	(C/T)
5080	CakSNP5080	*Kabuli*	Ca_Kabuli_Chr04	353119	(G/A)
5081	CakSNP5081	*Kabuli*	Ca_Kabuli_Chr04	353120	(C/G)
5082	CakSNP5082	*Kabuli*	Ca_Kabuli_Chr04	353124	(A/G)
5083	CakSNP5083	*Kabuli*	Ca_Kabuli_Chr04	353126	(T/G)
5084	CakSNP5084	*Kabuli*	Ca_Kabuli_Chr04	369258	(C/T)
5085	CakSNP5085	*Kabuli*	Ca_Kabuli_Chr04	394661	(C/G)
5086	CakSNP5086	*Kabuli*	Ca_Kabuli_Chr04	411208	(A/C)
5087	CakSNP5087	*Kabuli*	Ca_Kabuli_Chr04	412017	(C/A)
5088	CakSNP5088	*Kabuli*	Ca_Kabuli_Chr04	455361	(A/G)
5089	CakSNP5089	*Kabuli*	Ca_Kabuli_Chr04	459360	(T/C)
5090	CakSNP5090	*Kabuli*	Ca_Kabuli_Chr04	480138	(C/A)
5091	CakSNP5091	*Kabuli*	Ca_Kabuli_Chr04	481556	(A/G)

S.N.	SNP IDs	Cultivars	Chromosomes/scaffolds	Physical positions (bp)	SNPs
5092	CakSNP5092	*Kabuli*	Ca_Kabuli_Chr04	481687	(T/G)
5093	CakSNP5093	*Kabuli*	Ca_Kabuli_Chr04	504928	(C/A)
5094	CakSNP5094	*Kabuli*	Ca_Kabuli_Chr04	586273	(A/G)
5095	CakSNP5095	*Kabuli*	Ca_Kabuli_Chr04	586703	(T/C)
5096	CakSNP5096	*Kabuli*	Ca_Kabuli_Chr04	589140	(T/C)
5097	CakSNP5097	*Kabuli*	Ca_Kabuli_Chr04	589191	(A/G)
5098	CakSNP5098	*Kabuli*	Ca_Kabuli_Chr04	589204	(G/A)
5099	CakSNP5099	*Kabuli*	Ca_Kabuli_Chr04	589220	(A/C)
5100	CakSNP5100	*Kabuli*	Ca_Kabuli_Chr04	589224	(T/C)
5101	CakSNP5101	*Kabuli*	Ca_Kabuli_Chr04	595982	(C/G)
5102	CakSNP5102	*Kabuli*	Ca_Kabuli_Chr04	613150	(G/A)
5103	CakSNP5103	*Kabuli*	Ca_Kabuli_Chr04	613166	(A/G)
5104	CakSNP5104	*Kabuli*	Ca_Kabuli_Chr04	645103	(T/A)
5105	CakSNP5105	*Kabuli*	Ca_Kabuli_Chr04	645075	(T/A)
5106	CakSNP5106	*Kabuli*	Ca_Kabuli_Chr04	646567	(C/T)
5107	CakSNP5107	*Kabuli*	Ca_Kabuli_Chr04	646576	(A/G)
5108	CakSNP5108	*Kabuli*	Ca_Kabuli_Chr04	651040	(C/T)
5109	CakSNP5109	*Kabuli*	Ca_Kabuli_Chr04	660034	(A/C)
5110	CakSNP5110	*Kabuli*	Ca_Kabuli_Chr04	660029	(T/G)
5111	CakSNP5111	*Kabuli*	Ca_Kabuli_Chr04	703978	(A/T)
5112	CakSNP5112	*Kabuli*	Ca_Kabuli_Chr04	703975	(G/T)
5113	CakSNP5113	*Kabuli*	Ca_Kabuli_Chr04	704009	(G/A)
5114	CakSNP5114	*Kabuli*	Ca_Kabuli_Chr04	705336	(T/G)
5115	CakSNP5115	*Kabuli*	Ca_Kabuli_Chr04	783302	(C/T)
5116	CakSNP5116	*Kabuli*	Ca_Kabuli_Chr04	783382	(G/T)
5117	CakSNP5117	*Kabuli*	Ca_Kabuli_Chr04	827630	(T/C)
5118	CakSNP5118	*Kabuli*	Ca_Kabuli_Chr04	848192	(G/A)
5119	CakSNP5119	*Kabuli*	Ca_Kabuli_Chr04	913689	(A/G)
5120	CakSNP5120	*Kabuli*	Ca_Kabuli_Chr04	951389	(T/G)
5121	CakSNP5121	*Kabuli*	Ca_Kabuli_Chr04	951390	(G/A)
5122	CakSNP5122	*Kabuli*	Ca_Kabuli_Chr04	1035328	(G/C)
5123	CakSNP5123	*Kabuli*	Ca_Kabuli_Chr04	1047639	(G/T)
5124	CakSNP5124	*Kabuli*	Ca_Kabuli_Chr04	1065107	(C/T)
5125	CakSNP5125	*Kabuli*	Ca_Kabuli_Chr04	1065245	(A/G)
5126	CakSNP5126	*Kabuli*	Ca_Kabuli_Chr04	1189997	(C/T)
5127	CakSNP5127	*Kabuli*	Ca_Kabuli_Chr04	1190007	(C/G)
5128	CakSNP5128	*Kabuli*	Ca_Kabuli_Chr04	1190038	(G/T)
5129	CakSNP5129	*Kabuli*	Ca_Kabuli_Chr04	1190208	(T/C)

S.N.	SNP IDs	Cultivars	Chromosomes/scaffolds	Physical positions (bp)	SNPs
5130	CakSNP5130	*Kabuli*	Ca_Kabuli_Chr04	1190184	(A/C)
5131	CakSNP5131	*Kabuli*	Ca_Kabuli_Chr04	1190158	(T/G)
5132	CakSNP5132	*Kabuli*	Ca_Kabuli_Chr04	1259917	(G/T)
5133	CakSNP5133	*Kabuli*	Ca_Kabuli_Chr04	1259915	(T/G)
5134	CakSNP5134	*Kabuli*	Ca_Kabuli_Chr04	1264361	(T/C)
5135	CakSNP5135	*Kabuli*	Ca_Kabuli_Chr04	1266175	(A/G)
5136	CakSNP5136	*Kabuli*	Ca_Kabuli_Chr04	1266174	(C/T)
5137	CakSNP5137	*Kabuli*	Ca_Kabuli_Chr04	1283369	(A/G)
5138	CakSNP5138	*Kabuli*	Ca_Kabuli_Chr04	1344151	(T/C)
5139	CakSNP5139	*Kabuli*	Ca_Kabuli_Chr04	1396281	(G/C)
5140	CakSNP5140	*Kabuli*	Ca_Kabuli_Chr04	1396425	(C/A)
5141	CakSNP5141	*Kabuli*	Ca_Kabuli_Chr04	1474771	(T/C)
5142	CakSNP5142	*Kabuli*	Ca_Kabuli_Chr04	1474898	(A/G)
5143	CakSNP5143	*Kabuli*	Ca_Kabuli_Chr04	1474922	(G/T)
5144	CakSNP5144	*Kabuli*	Ca_Kabuli_Chr04	1493927	(T/C)
5145	CakSNP5145	*Kabuli*	Ca_Kabuli_Chr04	1513664	(T/A)
5146	CakSNP5146	*Kabuli*	Ca_Kabuli_Chr04	1526270	(T/C)
5147	CakSNP5147	*Kabuli*	Ca_Kabuli_Chr04	1526731	(T/G)
5148	CakSNP5148	*Kabuli*	Ca_Kabuli_Chr04	1613153	(G/A)
5149	CakSNP5149	*Kabuli*	Ca_Kabuli_Chr04	1613193	(G/A)
5150	CakSNP5150	*Kabuli*	Ca_Kabuli_Chr04	1613303	(G/A)
5151	CakSNP5151	*Kabuli*	Ca_Kabuli_Chr04	1687346	(A/G)
5152	CakSNP5152	*Kabuli*	Ca_Kabuli_Chr04	1691555	(T/G)
5153	CakSNP5153	*Kabuli*	Ca_Kabuli_Chr04	1708370	(C/T)
5154	CakSNP5154	*Kabuli*	Ca_Kabuli_Chr04	1711864	(A/C)
5155	CakSNP5155	*Kabuli*	Ca_Kabuli_Chr04	1711865	(G/A)
5156	CakSNP5156	*Kabuli*	Ca_Kabuli_Chr04	1720544	(A/C)
5157	CakSNP5157	*Kabuli*	Ca_Kabuli_Chr04	1720658	(A/G)
5158	CakSNP5158	*Kabuli*	Ca_Kabuli_Chr04	1720642	(A/G)
5159	CakSNP5159	*Kabuli*	Ca_Kabuli_Chr04	1720619	(A/C)
5160	CakSNP5160	*Kabuli*	Ca_Kabuli_Chr04	1742468	(C/T)
5161	CakSNP5161	*Kabuli*	Ca_Kabuli_Chr04	1747181	(A/T)
5162	CakSNP5162	*Kabuli*	Ca_Kabuli_Chr04	1754555	(C/T)
5163	CakSNP5163	*Kabuli*	Ca_Kabuli_Chr04	1755066	(G/C)
5164	CakSNP5164	*Kabuli*	Ca_Kabuli_Chr04	1773886	(C/G)
5165	CakSNP5165	*Kabuli*	Ca_Kabuli_Chr04	1773939	(C/T)
5166	CakSNP5166	*Kabuli*	Ca_Kabuli_Chr04	1773951	(C/G)
5167	CakSNP5167	*Kabuli*	Ca_Kabuli_Chr04	1785705	(G/C)

S.N.	SNP IDs	Cultivars	Chromosomes/scaffolds	Physical positions (bp)	SNPs
5168	CakSNP5168	*Kabuli*	Ca_Kabuli_Chr04	1785680	(C/T)
5169	CakSNP5169	*Kabuli*	Ca_Kabuli_Chr04	1807484	(G/A)
5170	CakSNP5170	*Kabuli*	Ca_Kabuli_Chr04	1817779	(G/C)
5171	CakSNP5171	*Kabuli*	Ca_Kabuli_Chr04	1833570	(T/C)
5172	CakSNP5172	*Kabuli*	Ca_Kabuli_Chr04	1833678	(A/G)
5173	CakSNP5173	*Kabuli*	Ca_Kabuli_Chr04	1833725	(C/T)
5174	CakSNP5174	*Kabuli*	Ca_Kabuli_Chr04	1845301	(G/A)
5175	CakSNP5175	*Kabuli*	Ca_Kabuli_Chr04	1867921	(G/A)
5176	CakSNP5176	*Kabuli*	Ca_Kabuli_Chr04	1869577	(C/T)
5177	CakSNP5177	*Kabuli*	Ca_Kabuli_Chr04	1870333	(A/G)
5178	CakSNP5178	*Kabuli*	Ca_Kabuli_Chr04	1870317	(T/C)
5179	CakSNP5179	*Kabuli*	Ca_Kabuli_Chr04	1870666	(G/C)
5180	CakSNP5180	*Kabuli*	Ca_Kabuli_Chr04	1870806	(C/A)
5181	CakSNP5181	*Kabuli*	Ca_Kabuli_Chr04	1870802	(C/T)
5182	CakSNP5182	*Kabuli*	Ca_Kabuli_Chr04	1873733	(T/C)
5183	CakSNP5183	*Kabuli*	Ca_Kabuli_Chr04	1890220	(G/C)
5184	CakSNP5184	*Kabuli*	Ca_Kabuli_Chr04	1941001	(T/G)
5185	CakSNP5185	*Kabuli*	Ca_Kabuli_Chr04	1941089	(C/T)
5186	CakSNP5186	*Kabuli*	Ca_Kabuli_Chr04	1970471	(A/C)
5187	CakSNP5187	*Kabuli*	Ca_Kabuli_Chr04	1973375	(A/C)
5188	CakSNP5188	*Kabuli*	Ca_Kabuli_Chr04	2009707	(A/G)
5189	CakSNP5189	*Kabuli*	Ca_Kabuli_Chr04	2025214	(A/C)
5190	CakSNP5190	*Kabuli*	Ca_Kabuli_Chr04	2027063	(G/A)
5191	CakSNP5191	*Kabuli*	Ca_Kabuli_Chr04	2027101	(T/A)
5192	CakSNP5192	*Kabuli*	Ca_Kabuli_Chr04	2034429	(C/A)
5193	CakSNP5193	*Kabuli*	Ca_Kabuli_Chr04	2034398	(C/T)
5194	CakSNP5194	*Kabuli*	Ca_Kabuli_Chr04	2034396	(A/G)
5195	CakSNP5195	*Kabuli*	Ca_Kabuli_Chr04	2034739	(A/C)
5196	CakSNP5196	*Kabuli*	Ca_Kabuli_Chr04	2035797	(G/A)
5197	CakSNP5197	*Kabuli*	Ca_Kabuli_Chr04	2035801	(G/A)
5198	CakSNP5198	*Kabuli*	Ca_Kabuli_Chr04	2035841	(C/T)
5199	CakSNP5199	*Kabuli*	Ca_Kabuli_Chr04	2035894	(C/A)
5200	CakSNP5200	*Kabuli*	Ca_Kabuli_Chr04	2051684	(T/C)
5201	CakSNP5201	*Kabuli*	Ca_Kabuli_Chr04	2051642	(G/C)
5202	CakSNP5202	*Kabuli*	Ca_Kabuli_Chr04	2060244	(G/A)
5203	CakSNP5203	*Kabuli*	Ca_Kabuli_Chr04	2060262	(C/T)
5204	CakSNP5204	*Kabuli*	Ca_Kabuli_Chr04	2113564	(A/G)
5205	CakSNP5205	*Kabuli*	Ca_Kabuli_Chr04	2125774	(G/A)

S.N.	SNP IDs	Cultivars	Chromosomes/scaffolds	Physical positions (bp)	SNPs
5206	CakSNP5206	*Kabuli*	Ca_Kabuli_Chr04	2143155	(A/G)
5207	CakSNP5207	*Kabuli*	Ca_Kabuli_Chr04	2213710	(G/A)
5208	CakSNP5208	*Kabuli*	Ca_Kabuli_Chr04	2238480	(A/T)
5209	CakSNP5209	*Kabuli*	Ca_Kabuli_Chr04	2270360	(T/C)
5210	CakSNP5210	*Kabuli*	Ca_Kabuli_Chr04	2277421	(A/T)
5211	CakSNP5211	*Kabuli*	Ca_Kabuli_Chr04	2311977	(A/G)
5212	CakSNP5212	*Kabuli*	Ca_Kabuli_Chr04	2313969	(G/A)
5213	CakSNP5213	*Kabuli*	Ca_Kabuli_Chr04	2361088	(T/A)
5214	CakSNP5214	*Kabuli*	Ca_Kabuli_Chr04	2380206	(A/G)
5215	CakSNP5215	*Kabuli*	Ca_Kabuli_Chr04	2393033	(T/G)
5216	CakSNP5216	*Kabuli*	Ca_Kabuli_Chr04	2396883	(A/G)
5217	CakSNP5217	*Kabuli*	Ca_Kabuli_Chr04	2411925	(G/T)
5218	CakSNP5218	*Kabuli*	Ca_Kabuli_Chr04	2422087	(A/G)
5219	CakSNP5219	*Kabuli*	Ca_Kabuli_Chr04	2422092	(G/A)
5220	CakSNP5220	*Kabuli*	Ca_Kabuli_Chr04	2422949	(C/T)
5221	CakSNP5221	*Kabuli*	Ca_Kabuli_Chr04	2463064	(A/G)
5222	CakSNP5222	*Kabuli*	Ca_Kabuli_Chr04	2463038	(T/C)
5223	CakSNP5223	*Kabuli*	Ca_Kabuli_Chr04	2492726	(A/G)
5224	CakSNP5224	*Kabuli*	Ca_Kabuli_Chr04	2493195	(T/C)
5225	CakSNP5225	*Kabuli*	Ca_Kabuli_Chr04	2494101	(C/A)
5226	CakSNP5226	*Kabuli*	Ca_Kabuli_Chr04	2494140	(C/A)
5227	CakSNP5227	*Kabuli*	Ca_Kabuli_Chr04	2499257	(A/C)
5228	CakSNP5228	*Kabuli*	Ca_Kabuli_Chr04	2562541	(G/C)
5229	CakSNP5229	*Kabuli*	Ca_Kabuli_Chr04	2564500	(T/A)
5230	CakSNP5230	*Kabuli*	Ca_Kabuli_Chr04	2609945	(C/T)
5231	CakSNP5231	*Kabuli*	Ca_Kabuli_Chr04	2676174	(C/T)
5232	CakSNP5232	*Kabuli*	Ca_Kabuli_Chr04	2740041	(T/G)
5233	CakSNP5233	*Kabuli*	Ca_Kabuli_Chr04	2740068	(A/T)
5234	CakSNP5234	*Kabuli*	Ca_Kabuli_Chr04	2740065	(A/G)
5235	CakSNP5235	*Kabuli*	Ca_Kabuli_Chr04	2752538	(G/A)
5236	CakSNP5236	*Kabuli*	Ca_Kabuli_Chr04	2752568	(C/T)
5237	CakSNP5237	*Kabuli*	Ca_Kabuli_Chr04	2886775	(T/G)
5238	CakSNP5238	*Kabuli*	Ca_Kabuli_Chr04	2886863	(C/T)
5239	CakSNP5239	*Kabuli*	Ca_Kabuli_Chr04	2978875	(T/C)
5240	CakSNP5240	*Kabuli*	Ca_Kabuli_Chr04	2978876	(T/G)
5241	CakSNP5241	*Kabuli*	Ca_Kabuli_Chr04	2978882	(G/T)
5242	CakSNP5242	*Kabuli*	Ca_Kabuli_Chr04	2978932	(A/C)
5243	CakSNP5243	*Kabuli*	Ca_Kabuli_Chr04	2978931	(A/T)

S.N.	SNP IDs	Cultivars	Chromosomes/scaffolds	Physical positions (bp)	SNPs
5244	CakSNP5244	*Kabuli*	Ca_Kabuli_Chr04	3002755	(A/G)
5245	CakSNP5245	*Kabuli*	Ca_Kabuli_Chr04	3002810	(T/C)
5246	CakSNP5246	*Kabuli*	Ca_Kabuli_Chr04	3037899	(A/C)
5247	CakSNP5247	*Kabuli*	Ca_Kabuli_Chr04	3037986	(T/A)
5248	CakSNP5248	*Kabuli*	Ca_Kabuli_Chr04	3037929	(T/G)
5249	CakSNP5249	*Kabuli*	Ca_Kabuli_Chr04	3038022	(C/T)
5250	CakSNP5250	*Kabuli*	Ca_Kabuli_Chr04	3046920	(A/G)
5251	CakSNP5251	*Kabuli*	Ca_Kabuli_Chr04	3144454	(T/C)
5252	CakSNP5252	*Kabuli*	Ca_Kabuli_Chr04	3158321	(A/G)
5253	CakSNP5253	*Kabuli*	Ca_Kabuli_Chr04	3158323	(T/G)
5254	CakSNP5254	*Kabuli*	Ca_Kabuli_Chr04	3158328	(G/T)
5255	CakSNP5255	*Kabuli*	Ca_Kabuli_Chr04	3158331	(A/T)
5256	CakSNP5256	*Kabuli*	Ca_Kabuli_Chr04	3158426	(G/A)
5257	CakSNP5257	*Kabuli*	Ca_Kabuli_Chr04	3158504	(A/T)
5258	CakSNP5258	*Kabuli*	Ca_Kabuli_Chr04	3158506	(T/C)
5259	CakSNP5259	*Kabuli*	Ca_Kabuli_Chr04	3158549	(A/G)
5260	CakSNP5260	*Kabuli*	Ca_Kabuli_Chr04	3164115	(G/A)
5261	CakSNP5261	*Kabuli*	Ca_Kabuli_Chr04	3164083	(G/A)
5262	CakSNP5262	*Kabuli*	Ca_Kabuli_Chr04	3166657	(C/T)
5263	CakSNP5263	*Kabuli*	Ca_Kabuli_Chr04	3166663	(C/T)
5264	CakSNP5264	*Kabuli*	Ca_Kabuli_Chr04	3166674	(G/T)
5265	CakSNP5265	*Kabuli*	Ca_Kabuli_Chr04	3171162	(G/A)
5266	CakSNP5266	*Kabuli*	Ca_Kabuli_Chr04	3171384	(T/G)
5267	CakSNP5267	*Kabuli*	Ca_Kabuli_Chr04	3171385	(A/G)
5268	CakSNP5268	*Kabuli*	Ca_Kabuli_Chr04	3171428	(T/C)
5269	CakSNP5269	*Kabuli*	Ca_Kabuli_Chr04	3171551	(C/T)
5270	CakSNP5270	*Kabuli*	Ca_Kabuli_Chr04	3264351	(T/C)
5271	CakSNP5271	*Kabuli*	Ca_Kabuli_Chr04	3280147	(G/A)
5272	CakSNP5272	*Kabuli*	Ca_Kabuli_Chr04	3280217	(A/G)
5273	CakSNP5273	*Kabuli*	Ca_Kabuli_Chr04	3281050	(T/G)
5274	CakSNP5274	*Kabuli*	Ca_Kabuli_Chr04	3290298	(T/G)
5275	CakSNP5275	*Kabuli*	Ca_Kabuli_Chr04	3353106	(T/C)
5276	CakSNP5276	*Kabuli*	Ca_Kabuli_Chr04	3356252	(C/G)
5277	CakSNP5277	*Kabuli*	Ca_Kabuli_Chr04	3356242	(C/G)
5278	CakSNP5278	*Kabuli*	Ca_Kabuli_Chr04	3400828	(G/C)
5279	CakSNP5279	*Kabuli*	Ca_Kabuli_Chr04	3427396	(A/G)
5280	CakSNP5280	*Kabuli*	Ca_Kabuli_Chr04	3429035	(G/A)
5281	CakSNP5281	*Kabuli*	Ca_Kabuli_Chr04	3429059	(C/A)

S.N.	SNP IDs	Cultivars	Chromosomes/scaffolds	Physical positions (bp)	SNPs
5282	CakSNP5282	*Kabuli*	Ca_Kabuli_Chr04	3455029	(T/C)
5283	CakSNP5283	*Kabuli*	Ca_Kabuli_Chr04	3455047	(G/A)
5284	CakSNP5284	*Kabuli*	Ca_Kabuli_Chr04	3456450	(G/T)
5285	CakSNP5285	*Kabuli*	Ca_Kabuli_Chr04	3456501	(C/T)
5286	CakSNP5286	*Kabuli*	Ca_Kabuli_Chr04	3483687	(A/C)
5287	CakSNP5287	*Kabuli*	Ca_Kabuli_Chr04	3627810	(G/T)
5288	CakSNP5288	*Kabuli*	Ca_Kabuli_Chr04	3642051	(A/T)
5289	CakSNP5289	*Kabuli*	Ca_Kabuli_Chr04	3642070	(T/C)
5290	CakSNP5290	*Kabuli*	Ca_Kabuli_Chr04	3642116	(A/T)
5291	CakSNP5291	*Kabuli*	Ca_Kabuli_Chr04	3689840	(T/C)
5292	CakSNP5292	*Kabuli*	Ca_Kabuli_Chr04	3689871	(G/A)
5293	CakSNP5293	*Kabuli*	Ca_Kabuli_Chr04	3739386	(C/A)
5294	CakSNP5294	*Kabuli*	Ca_Kabuli_Chr04	3741522	(T/G)
5295	CakSNP5295	*Kabuli*	Ca_Kabuli_Chr04	3741539	(T/G)
5296	CakSNP5296	*Kabuli*	Ca_Kabuli_Chr04	3752141	(C/G)
5297	CakSNP5297	*Kabuli*	Ca_Kabuli_Chr04	3762559	(T/C)
5298	CakSNP5298	*Kabuli*	Ca_Kabuli_Chr04	3762561	(C/A)
5299	CakSNP5299	*Kabuli*	Ca_Kabuli_Chr04	3762585	(G/T)
5300	CakSNP5300	*Kabuli*	Ca_Kabuli_Chr04	3794764	(G/A)
5301	CakSNP5301	*Kabuli*	Ca_Kabuli_Chr04	3794885	(C/A)
5302	CakSNP5302	*Kabuli*	Ca_Kabuli_Chr04	3885444	(G/C)
5303	CakSNP5303	*Kabuli*	Ca_Kabuli_Chr04	3888010	(G/C)
5304	CakSNP5304	*Kabuli*	Ca_Kabuli_Chr04	3888053	(C/A)
5305	CakSNP5305	*Kabuli*	Ca_Kabuli_Chr04	3888164	(T/A)
5306	CakSNP5306	*Kabuli*	Ca_Kabuli_Chr04	3888185	(A/G)
5307	CakSNP5307	*Kabuli*	Ca_Kabuli_Chr04	3888236	(A/G)
5308	CakSNP5308	*Kabuli*	Ca_Kabuli_Chr04	3888328	(C/T)
5309	CakSNP5309	*Kabuli*	Ca_Kabuli_Chr04	3888462	(T/C)
5310	CakSNP5310	*Kabuli*	Ca_Kabuli_Chr04	3888510	(G/A)
5311	CakSNP5311	*Kabuli*	Ca_Kabuli_Chr04	3888514	(G/A)
5312	CakSNP5312	*Kabuli*	Ca_Kabuli_Chr04	3888529	(T/G)
5313	CakSNP5313	*Kabuli*	Ca_Kabuli_Chr04	3890556	(A/G)
5314	CakSNP5314	*Kabuli*	Ca_Kabuli_Chr04	3890709	(A/G)
5315	CakSNP5315	*Kabuli*	Ca_Kabuli_Chr04	3990810	(C/G)
5316	CakSNP5316	*Kabuli*	Ca_Kabuli_Chr04	3990809	(G/A)
5317	CakSNP5317	*Kabuli*	Ca_Kabuli_Chr04	4002917	(T/C)
5318	CakSNP5318	*Kabuli*	Ca_Kabuli_Chr04	4006213	(A/C)
5319	CakSNP5319	*Kabuli*	Ca_Kabuli_Chr04	4027829	(A/T)

S.N.	SNP IDs	Cultivars	Chromosomes/scaffolds	Physical positions (bp)	SNPs
5320	CakSNP5320	*Kabuli*	Ca_Kabuli_Chr04	4027872	(A/C)
5321	CakSNP5321	*Kabuli*	Ca_Kabuli_Chr04	4097989	(G/A)
5322	CakSNP5322	*Kabuli*	Ca_Kabuli_Chr04	4097935	(T/C)
5323	CakSNP5323	*Kabuli*	Ca_Kabuli_Chr04	4116386	(G/C)
5324	CakSNP5324	*Kabuli*	Ca_Kabuli_Chr04	4148061	(T/C)
5325	CakSNP5325	*Kabuli*	Ca_Kabuli_Chr04	4148250	(A/G)
5326	CakSNP5326	*Kabuli*	Ca_Kabuli_Chr04	4154417	(A/C)
5327	CakSNP5327	*Kabuli*	Ca_Kabuli_Chr04	4154507	(G/T)
5328	CakSNP5328	*Kabuli*	Ca_Kabuli_Chr04	4229295	(T/A)
5329	CakSNP5329	*Kabuli*	Ca_Kabuli_Chr04	4229345	(C/T)
5330	CakSNP5330	*Kabuli*	Ca_Kabuli_Chr04	4229381	(T/C)
5331	CakSNP5331	*Kabuli*	Ca_Kabuli_Chr04	4229371	(C/T)
5332	CakSNP5332	*Kabuli*	Ca_Kabuli_Chr04	4255863	(T/G)
5333	CakSNP5333	*Kabuli*	Ca_Kabuli_Chr04	4256023	(C/T)
5334	CakSNP5334	*Kabuli*	Ca_Kabuli_Chr04	4258002	(A/G)
5335	CakSNP5335	*Kabuli*	Ca_Kabuli_Chr04	4258149	(A/G)
5336	CakSNP5336	*Kabuli*	Ca_Kabuli_Chr04	4305891	(A/C)
5337	CakSNP5337	*Kabuli*	Ca_Kabuli_Chr04	4320615	(C/T)
5338	CakSNP5338	*Kabuli*	Ca_Kabuli_Chr04	4320743	(A/G)
5339	CakSNP5339	*Kabuli*	Ca_Kabuli_Chr04	4327268	(A/G)
5340	CakSNP5340	*Kabuli*	Ca_Kabuli_Chr04	4334307	(T/G)
5341	CakSNP5341	*Kabuli*	Ca_Kabuli_Chr04	4343586	(C/T)
5342	CakSNP5342	*Kabuli*	Ca_Kabuli_Chr04	4343540	(C/A)
5343	CakSNP5343	*Kabuli*	Ca_Kabuli_Chr04	4410351	(T/C)
5344	CakSNP5344	*Kabuli*	Ca_Kabuli_Chr04	4426659	(C/T)
5345	CakSNP5345	*Kabuli*	Ca_Kabuli_Chr04	4426760	(T/C)
5346	CakSNP5346	*Kabuli*	Ca_Kabuli_Chr04	4435621	(T/C)
5347	CakSNP5347	*Kabuli*	Ca_Kabuli_Chr04	4451928	(T/C)
5348	CakSNP5348	*Kabuli*	Ca_Kabuli_Chr04	4452018	(T/A)
5349	CakSNP5349	*Kabuli*	Ca_Kabuli_Chr04	4452380	(C/G)
5350	CakSNP5350	*Kabuli*	Ca_Kabuli_Chr04	4454305	(A/C)
5351	CakSNP5351	*Kabuli*	Ca_Kabuli_Chr04	4454464	(A/G)
5352	CakSNP5352	*Kabuli*	Ca_Kabuli_Chr04	4461562	(G/C)
5353	CakSNP5353	*Kabuli*	Ca_Kabuli_Chr04	4468394	(C/T)
5354	CakSNP5354	*Kabuli*	Ca_Kabuli_Chr04	4536310	(A/G)
5355	CakSNP5355	*Kabuli*	Ca_Kabuli_Chr04	4536286	(G/C)
5356	CakSNP5356	*Kabuli*	Ca_Kabuli_Chr04	4578836	(C/T)
5357	CakSNP5357	*Kabuli*	Ca_Kabuli_Chr04	4579274	(C/T)

S.N.	SNP IDs	Cultivars	Chromosomes/scaffolds	Physical positions (bp)	SNPs
5358	CakSNP5358	*Kabuli*	Ca_Kabuli_Chr04	4605340	(A/C)
5359	CakSNP5359	*Kabuli*	Ca_Kabuli_Chr04	4622932	(T/G)
5360	CakSNP5360	*Kabuli*	Ca_Kabuli_Chr04	4622913	(C/T)
5361	CakSNP5361	*Kabuli*	Ca_Kabuli_Chr04	4628221	(T/G)
5362	CakSNP5362	*Kabuli*	Ca_Kabuli_Chr04	4628232	(G/A)
5363	CakSNP5363	*Kabuli*	Ca_Kabuli_Chr04	4628240	(C/T)
5364	CakSNP5364	*Kabuli*	Ca_Kabuli_Chr04	4628243	(G/A)
5365	CakSNP5365	*Kabuli*	Ca_Kabuli_Chr04	4628309	(C/T)
5366	CakSNP5366	*Kabuli*	Ca_Kabuli_Chr04	4630407	(G/A)
5367	CakSNP5367	*Kabuli*	Ca_Kabuli_Chr04	4630419	(A/C)
5368	CakSNP5368	*Kabuli*	Ca_Kabuli_Chr04	4630514	(T/C)
5369	CakSNP5369	*Kabuli*	Ca_Kabuli_Chr04	4633913	(T/G)
5370	CakSNP5370	*Kabuli*	Ca_Kabuli_Chr04	4634542	(A/T)
5371	CakSNP5371	*Kabuli*	Ca_Kabuli_Chr04	4660220	(A/G)
5372	CakSNP5372	*Kabuli*	Ca_Kabuli_Chr04	4670913	(A/G)
5373	CakSNP5373	*Kabuli*	Ca_Kabuli_Chr04	4670975	(C/A)
5374	CakSNP5374	*Kabuli*	Ca_Kabuli_Chr04	4675182	(T/G)
5375	CakSNP5375	*Kabuli*	Ca_Kabuli_Chr04	4675177	(T/A)
5376	CakSNP5376	*Kabuli*	Ca_Kabuli_Chr04	4675176	(T/G)
5377	CakSNP5377	*Kabuli*	Ca_Kabuli_Chr04	4675172	(T/A)
5378	CakSNP5378	*Kabuli*	Ca_Kabuli_Chr04	4675171	(T/A)
5379	CakSNP5379	*Kabuli*	Ca_Kabuli_Chr04	4675166	(A/T)
5380	CakSNP5380	*Kabuli*	Ca_Kabuli_Chr04	4679236	(A/G)
5381	CakSNP5381	*Kabuli*	Ca_Kabuli_Chr04	4707682	(A/G)
5382	CakSNP5382	*Kabuli*	Ca_Kabuli_Chr04	4722348	(G/A)
5383	CakSNP5383	*Kabuli*	Ca_Kabuli_Chr04	4757357	(T/C)
5384	CakSNP5384	*Kabuli*	Ca_Kabuli_Chr04	4757416	(G/A)
5385	CakSNP5385	*Kabuli*	Ca_Kabuli_Chr04	4757498	(C/G)
5386	CakSNP5386	*Kabuli*	Ca_Kabuli_Chr04	4794122	(G/A)
5387	CakSNP5387	*Kabuli*	Ca_Kabuli_Chr04	4794192	(G/C)
5388	CakSNP5388	*Kabuli*	Ca_Kabuli_Chr04	4799122	(C/T)
5389	CakSNP5389	*Kabuli*	Ca_Kabuli_Chr04	4799116	(C/T)
5390	CakSNP5390	*Kabuli*	Ca_Kabuli_Chr04	4803168	(T/A)
5391	CakSNP5391	*Kabuli*	Ca_Kabuli_Chr04	4803213	(C/T)
5392	CakSNP5392	*Kabuli*	Ca_Kabuli_Chr04	4874780	(A/T)
5393	CakSNP5393	*Kabuli*	Ca_Kabuli_Chr04	4874782	(C/A)
5394	CakSNP5394	*Kabuli*	Ca_Kabuli_Chr04	4874836	(C/T)
5395	CakSNP5395	*Kabuli*	Ca_Kabuli_Chr04	4882813	(C/G)

S.N.	SNP IDs	Cultivars	Chromosomes/scaffolds	Physical positions (bp)	SNPs
5396	CakSNP5396	*Kabuli*	Ca_Kabuli_Chr04	4897934	(C/G)
5397	CakSNP5397	*Kabuli*	Ca_Kabuli_Chr04	4907987	(G/A)
5398	CakSNP5398	*Kabuli*	Ca_Kabuli_Chr04	4911083	(T/C)
5399	CakSNP5399	*Kabuli*	Ca_Kabuli_Chr04	4911130	(T/C)
5400	CakSNP5400	*Kabuli*	Ca_Kabuli_Chr04	4911209	(A/G)
5401	CakSNP5401	*Kabuli*	Ca_Kabuli_Chr04	4911217	(A/C)
5402	CakSNP5402	*Kabuli*	Ca_Kabuli_Chr04	4920706	(T/C)
5403	CakSNP5403	*Kabuli*	Ca_Kabuli_Chr04	4920712	(A/T)
5404	CakSNP5404	*Kabuli*	Ca_Kabuli_Chr04	4920719	(A/G)
5405	CakSNP5405	*Kabuli*	Ca_Kabuli_Chr04	4922290	(A/C)
5406	CakSNP5406	*Kabuli*	Ca_Kabuli_Chr04	4928764	(T/C)
5407	CakSNP5407	*Kabuli*	Ca_Kabuli_Chr04	4928890	(A/G)
5408	CakSNP5408	*Kabuli*	Ca_Kabuli_Chr04	4929387	(T/C)
5409	CakSNP5409	*Kabuli*	Ca_Kabuli_Chr04	4953629	(C/A)
5410	CakSNP5410	*Kabuli*	Ca_Kabuli_Chr04	4967336	(T/C)
5411	CakSNP5411	*Kabuli*	Ca_Kabuli_Chr04	4967342	(G/A)
5412	CakSNP5412	*Kabuli*	Ca_Kabuli_Chr04	4967347	(A/G)
5413	CakSNP5413	*Kabuli*	Ca_Kabuli_Chr04	5018591	(G/A)
5414	CakSNP5414	*Kabuli*	Ca_Kabuli_Chr04	5038300	(G/C)
5415	CakSNP5415	*Kabuli*	Ca_Kabuli_Chr04	5051617	(A/G)
5416	CakSNP5416	*Kabuli*	Ca_Kabuli_Chr04	5051574	(T/G)
5417	CakSNP5417	*Kabuli*	Ca_Kabuli_Chr04	5051573	(A/C)
5418	CakSNP5418	*Kabuli*	Ca_Kabuli_Chr04	5086705	(T/C)
5419	CakSNP5419	*Kabuli*	Ca_Kabuli_Chr04	5120207	(C/T)
5420	CakSNP5420	*Kabuli*	Ca_Kabuli_Chr04	5186401	(A/G)
5421	CakSNP5421	*Kabuli*	Ca_Kabuli_Chr04	5187402	(G/C)
5422	CakSNP5422	*Kabuli*	Ca_Kabuli_Chr04	5187344	(C/G)
5423	CakSNP5423	*Kabuli*	Ca_Kabuli_Chr04	5188084	(G/A)
5424	CakSNP5424	*Kabuli*	Ca_Kabuli_Chr04	5188040	(A/G)
5425	CakSNP5425	*Kabuli*	Ca_Kabuli_Chr04	5197824	(T/C)
5426	CakSNP5426	*Kabuli*	Ca_Kabuli_Chr04	5199907	(C/T)
5427	CakSNP5427	*Kabuli*	Ca_Kabuli_Chr04	5222669	(A/C)
5428	CakSNP5428	*Kabuli*	Ca_Kabuli_Chr04	5222805	(G/C)
5429	CakSNP5429	*Kabuli*	Ca_Kabuli_Chr04	5222851	(C/T)
5430	CakSNP5430	*Kabuli*	Ca_Kabuli_Chr04	5223100	(G/A)
5431	CakSNP5431	*Kabuli*	Ca_Kabuli_Chr04	5223206	(A/T)
5432	CakSNP5432	*Kabuli*	Ca_Kabuli_Chr04	5228950	(G/T)
5433	CakSNP5433	*Kabuli*	Ca_Kabuli_Chr04	5232364	(T/C)

S.N.	SNP IDs	Cultivars	Chromosomes/scaffolds	Physical positions (bp)	SNPs
5434	CakSNP5434	*Kabuli*	Ca_Kabuli_Chr04	5263199	(A/G)
5435	CakSNP5435	*Kabuli*	Ca_Kabuli_Chr04	5268995	(A/C)
5436	CakSNP5436	*Kabuli*	Ca_Kabuli_Chr04	5282131	(T/C)
5437	CakSNP5437	*Kabuli*	Ca_Kabuli_Chr04	5310873	(A/G)
5438	CakSNP5438	*Kabuli*	Ca_Kabuli_Chr04	5345520	(T/G)
5439	CakSNP5439	*Kabuli*	Ca_Kabuli_Chr04	5377067	(A/C)
5440	CakSNP5440	*Kabuli*	Ca_Kabuli_Chr04	5377400	(C/T)
5441	CakSNP5441	*Kabuli*	Ca_Kabuli_Chr04	5390236	(C/A)
5442	CakSNP5442	*Kabuli*	Ca_Kabuli_Chr04	5390317	(G/A)
5443	CakSNP5443	*Kabuli*	Ca_Kabuli_Chr04	5453008	(A/C)
5444	CakSNP5444	*Kabuli*	Ca_Kabuli_Chr04	5473174	(C/T)
5445	CakSNP5445	*Kabuli*	Ca_Kabuli_Chr04	5484259	(G/T)
5446	CakSNP5446	*Kabuli*	Ca_Kabuli_Chr04	5484257	(G/C)
5447	CakSNP5447	*Kabuli*	Ca_Kabuli_Chr04	5484238	(G/A)
5448	CakSNP5448	*Kabuli*	Ca_Kabuli_Chr04	5484236	(C/T)
5449	CakSNP5449	*Kabuli*	Ca_Kabuli_Chr04	5484220	(A/G)
5450	CakSNP5450	*Kabuli*	Ca_Kabuli_Chr04	5485052	(G/T)
5451	CakSNP5451	*Kabuli*	Ca_Kabuli_Chr04	5485050	(G/C)
5452	CakSNP5452	*Kabuli*	Ca_Kabuli_Chr04	5485031	(G/A)
5453	CakSNP5453	*Kabuli*	Ca_Kabuli_Chr04	5485029	(C/T)
5454	CakSNP5454	*Kabuli*	Ca_Kabuli_Chr04	5485013	(A/G)
5455	CakSNP5455	*Kabuli*	Ca_Kabuli_Chr04	5528421	(T/G)
5456	CakSNP5456	*Kabuli*	Ca_Kabuli_Chr04	5530612	(A/G)
5457	CakSNP5457	*Kabuli*	Ca_Kabuli_Chr04	5535661	(A/G)
5458	CakSNP5458	*Kabuli*	Ca_Kabuli_Chr04	5575846	(G/C)
5459	CakSNP5459	*Kabuli*	Ca_Kabuli_Chr04	5595079	(T/C)
5460	CakSNP5460	*Kabuli*	Ca_Kabuli_Chr04	5595077	(T/A)
5461	CakSNP5461	*Kabuli*	Ca_Kabuli_Chr04	5623571	(A/G)
5462	CakSNP5462	*Kabuli*	Ca_Kabuli_Chr04	5627096	(C/T)
5463	CakSNP5463	*Kabuli*	Ca_Kabuli_Chr04	5701873	(A/C)
5464	CakSNP5464	*Kabuli*	Ca_Kabuli_Chr04	5701900	(G/T)
5465	CakSNP5465	*Kabuli*	Ca_Kabuli_Chr04	5701902	(C/T)
5466	CakSNP5466	*Kabuli*	Ca_Kabuli_Chr04	5701907	(G/T)
5467	CakSNP5467	*Kabuli*	Ca_Kabuli_Chr04	5779698	(A/G)
5468	CakSNP5468	*Kabuli*	Ca_Kabuli_Chr04	5779765	(A/T)
5469	CakSNP5469	*Kabuli*	Ca_Kabuli_Chr04	5787727	(G/A)
5470	CakSNP5470	*Kabuli*	Ca_Kabuli_Chr04	5787722	(G/A)
5471	CakSNP5471	*Kabuli*	Ca_Kabuli_Chr04	5841444	(C/G)

S.N.	SNP IDs	Cultivars	Chromosomes/scaffolds	Physical positions (bp)	SNPs
5472	CakSNP5472	*Kabuli*	Ca_Kabuli_Chr04	5841462	(A/C)
5473	CakSNP5473	*Kabuli*	Ca_Kabuli_Chr04	5853789	(C/T)
5474	CakSNP5474	*Kabuli*	Ca_Kabuli_Chr04	5853798	(T/G)
5475	CakSNP5475	*Kabuli*	Ca_Kabuli_Chr04	5900194	(C/A)
5476	CakSNP5476	*Kabuli*	Ca_Kabuli_Chr04	5900290	(T/C)
5477	CakSNP5477	*Kabuli*	Ca_Kabuli_Chr04	5905252	(C/T)
5478	CakSNP5478	*Kabuli*	Ca_Kabuli_Chr04	5905276	(C/T)
5479	CakSNP5479	*Kabuli*	Ca_Kabuli_Chr04	5905303	(A/C)
5480	CakSNP5480	*Kabuli*	Ca_Kabuli_Chr04	5905385	(G/A)
5481	CakSNP5481	*Kabuli*	Ca_Kabuli_Chr04	5907409	(T/G)
5482	CakSNP5482	*Kabuli*	Ca_Kabuli_Chr04	5907421	(T/G)
5483	CakSNP5483	*Kabuli*	Ca_Kabuli_Chr04	5907437	(C/A)
5484	CakSNP5484	*Kabuli*	Ca_Kabuli_Chr04	5907465	(C/A)
5485	CakSNP5485	*Kabuli*	Ca_Kabuli_Chr04	5940176	(C/T)
5486	CakSNP5486	*Kabuli*	Ca_Kabuli_Chr04	5999964	(T/C)
5487	CakSNP5487	*Kabuli*	Ca_Kabuli_Chr04	6000010	(T/A)
5488	CakSNP5488	*Kabuli*	Ca_Kabuli_Chr04	6000013	(A/C)
5489	CakSNP5489	*Kabuli*	Ca_Kabuli_Chr04	6000037	(T/G)
5490	CakSNP5490	*Kabuli*	Ca_Kabuli_Chr04	6006849	(T/G)
5491	CakSNP5491	*Kabuli*	Ca_Kabuli_Chr04	6030948	(T/G)
5492	CakSNP5492	*Kabuli*	Ca_Kabuli_Chr04	6030966	(A/G)
5493	CakSNP5493	*Kabuli*	Ca_Kabuli_Chr04	6041134	(A/C)
5494	CakSNP5494	*Kabuli*	Ca_Kabuli_Chr04	6041130	(C/A)
5495	CakSNP5495	*Kabuli*	Ca_Kabuli_Chr04	6041126	(A/G)
5496	CakSNP5496	*Kabuli*	Ca_Kabuli_Chr04	6041120	(G/T)
5497	CakSNP5497	*Kabuli*	Ca_Kabuli_Chr04	6041117	(C/T)
5498	CakSNP5498	*Kabuli*	Ca_Kabuli_Chr04	6076530	(T/C)
5499	CakSNP5499	*Kabuli*	Ca_Kabuli_Chr04	6076594	(G/C)
5500	CakSNP5500	*Kabuli*	Ca_Kabuli_Chr04	6076614	(A/G)
5501	CakSNP5501	*Kabuli*	Ca_Kabuli_Chr04	6077103	(G/A)
5502	CakSNP5502	*Kabuli*	Ca_Kabuli_Chr04	6077113	(G/T)
5503	CakSNP5503	*Kabuli*	Ca_Kabuli_Chr04	6077156	(G/T)
5504	CakSNP5504	*Kabuli*	Ca_Kabuli_Chr04	6077168	(T/C)
5505	CakSNP5505	*Kabuli*	Ca_Kabuli_Chr04	6157625	(T/C)
5506	CakSNP5506	*Kabuli*	Ca_Kabuli_Chr04	6201137	(T/C)
5507	CakSNP5507	*Kabuli*	Ca_Kabuli_Chr04	6238354	(G/A)
5508	CakSNP5508	*Kabuli*	Ca_Kabuli_Chr04	6238367	(T/A)
5509	CakSNP5509	*Kabuli*	Ca_Kabuli_Chr04	6238376	(A/G)

S.N.	SNP IDs	Cultivars	Chromosomes/scaffolds	Physical positions (bp)	SNPs
5510	CakSNP5510	*Kabuli*	Ca_Kabuli_Chr04	6238414	(T/A)
5511	CakSNP5511	*Kabuli*	Ca_Kabuli_Chr04	6277398	(C/T)
5512	CakSNP5512	*Kabuli*	Ca_Kabuli_Chr04	6281228	(A/C)
5513	CakSNP5513	*Kabuli*	Ca_Kabuli_Chr04	6296089	(G/T)
5514	CakSNP5514	*Kabuli*	Ca_Kabuli_Chr04	6387938	(T/G)
5515	CakSNP5515	*Kabuli*	Ca_Kabuli_Chr04	6403910	(A/C)
5516	CakSNP5516	*Kabuli*	Ca_Kabuli_Chr04	6408543	(T/G)
5517	CakSNP5517	*Kabuli*	Ca_Kabuli_Chr04	6415058	(A/T)
5518	CakSNP5518	*Kabuli*	Ca_Kabuli_Chr04	6462228	(C/T)
5519	CakSNP5519	*Kabuli*	Ca_Kabuli_Chr04	6507540	(G/A)
5520	CakSNP5520	*Kabuli*	Ca_Kabuli_Chr04	6509701	(T/A)
5521	CakSNP5521	*Kabuli*	Ca_Kabuli_Chr04	6509761	(C/A)
5522	CakSNP5522	*Kabuli*	Ca_Kabuli_Chr04	6511977	(C/T)
5523	CakSNP5523	*Kabuli*	Ca_Kabuli_Chr04	6512060	(A/T)
5524	CakSNP5524	*Kabuli*	Ca_Kabuli_Chr04	6513321	(A/T)
5525	CakSNP5525	*Kabuli*	Ca_Kabuli_Chr04	6513493	(C/T)
5526	CakSNP5526	*Kabuli*	Ca_Kabuli_Chr04	6513576	(A/T)
5527	CakSNP5527	*Kabuli*	Ca_Kabuli_Chr04	6544385	(G/A)
5528	CakSNP5528	*Kabuli*	Ca_Kabuli_Chr04	6544342	(A/C)
5529	CakSNP5529	*Kabuli*	Ca_Kabuli_Chr04	6544337	(A/C)
5530	CakSNP5530	*Kabuli*	Ca_Kabuli_Chr04	6552776	(A/G)
5531	CakSNP5531	*Kabuli*	Ca_Kabuli_Chr04	6562373	(T/A)
5532	CakSNP5532	*Kabuli*	Ca_Kabuli_Chr04	6562391	(A/C)
5533	CakSNP5533	*Kabuli*	Ca_Kabuli_Chr04	6562402	(G/C)
5534	CakSNP5534	*Kabuli*	Ca_Kabuli_Chr04	6611820	(G/A)
5535	CakSNP5535	*Kabuli*	Ca_Kabuli_Chr04	6616016	(C/T)
5536	CakSNP5536	*Kabuli*	Ca_Kabuli_Chr04	6616071	(C/T)
5537	CakSNP5537	*Kabuli*	Ca_Kabuli_Chr04	6688218	(G/A)
5538	CakSNP5538	*Kabuli*	Ca_Kabuli_Chr04	6715326	(C/T)
5539	CakSNP5539	*Kabuli*	Ca_Kabuli_Chr04	6723103	(G/T)
5540	CakSNP5540	*Kabuli*	Ca_Kabuli_Chr04	6749334	(A/G)
5541	CakSNP5541	*Kabuli*	Ca_Kabuli_Chr04	6749481	(C/T)
5542	CakSNP5542	*Kabuli*	Ca_Kabuli_Chr04	6749550	(G/A)
5543	CakSNP5543	*Kabuli*	Ca_Kabuli_Chr04	6749522	(T/C)
5544	CakSNP5544	*Kabuli*	Ca_Kabuli_Chr04	6751549	(A/T)
5545	CakSNP5545	*Kabuli*	Ca_Kabuli_Chr04	6761778	(C/A)
5546	CakSNP5546	*Kabuli*	Ca_Kabuli_Chr04	6765922	(T/G)
5547	CakSNP5547	*Kabuli*	Ca_Kabuli_Chr04	6765884	(T/C)

S.N.	SNP IDs	Cultivars	Chromosomes/scaffolds	Physical positions (bp)	SNPs
5548	CakSNP5548	*Kabuli*	Ca_Kabuli_Chr04	6792858	(T/G)
5549	CakSNP5549	*Kabuli*	Ca_Kabuli_Chr04	6801632	(C/A)
5550	CakSNP5550	*Kabuli*	Ca_Kabuli_Chr04	6841854	(G/A)
5551	CakSNP5551	*Kabuli*	Ca_Kabuli_Chr04	6841856	(G/A)
5552	CakSNP5552	*Kabuli*	Ca_Kabuli_Chr04	6841858	(G/A)
5553	CakSNP5553	*Kabuli*	Ca_Kabuli_Chr04	6841859	(A/C)
5554	CakSNP5554	*Kabuli*	Ca_Kabuli_Chr04	6841860	(G/A)
5555	CakSNP5555	*Kabuli*	Ca_Kabuli_Chr04	6841861	(A/C)
5556	CakSNP5556	*Kabuli*	Ca_Kabuli_Chr04	6843659	(T/C)
5557	CakSNP5557	*Kabuli*	Ca_Kabuli_Chr04	6852908	(T/C)
5558	CakSNP5558	*Kabuli*	Ca_Kabuli_Chr04	6889964	(T/G)
5559	CakSNP5559	*Kabuli*	Ca_Kabuli_Chr04	6889974	(T/G)
5560	CakSNP5560	*Kabuli*	Ca_Kabuli_Chr04	6890000	(A/G)
5561	CakSNP5561	*Kabuli*	Ca_Kabuli_Chr04	6890005	(C/T)
5562	CakSNP5562	*Kabuli*	Ca_Kabuli_Chr04	6890014	(G/T)
5563	CakSNP5563	*Kabuli*	Ca_Kabuli_Chr04	6889994	(T/G)
5564	CakSNP5564	*Kabuli*	Ca_Kabuli_Chr04	6895804	(G/A)
5565	CakSNP5565	*Kabuli*	Ca_Kabuli_Chr04	6919896	(C/T)
5566	CakSNP5566	*Kabuli*	Ca_Kabuli_Chr04	6920068	(G/A)
5567	CakSNP5567	*Kabuli*	Ca_Kabuli_Chr04	6931668	(T/A)
5568	CakSNP5568	*Kabuli*	Ca_Kabuli_Chr04	6960336	(G/A)
5569	CakSNP5569	*Kabuli*	Ca_Kabuli_Chr04	6969771	(G/T)
5570	CakSNP5570	*Kabuli*	Ca_Kabuli_Chr04	6969799	(T/C)
5571	CakSNP5571	*Kabuli*	Ca_Kabuli_Chr04	6970808	(A/G)
5572	CakSNP5572	*Kabuli*	Ca_Kabuli_Chr04	6970800	(A/G)
5573	CakSNP5573	*Kabuli*	Ca_Kabuli_Chr04	6970792	(T/A)
5574	CakSNP5574	*Kabuli*	Ca_Kabuli_Chr04	6970781	(G/A)
5575	CakSNP5575	*Kabuli*	Ca_Kabuli_Chr04	6970856	(T/C)
5576	CakSNP5576	*Kabuli*	Ca_Kabuli_Chr04	7013498	(A/G)
5577	CakSNP5577	*Kabuli*	Ca_Kabuli_Chr04	7040229	(A/T)
5578	CakSNP5578	*Kabuli*	Ca_Kabuli_Chr04	7142498	(G/A)
5579	CakSNP5579	*Kabuli*	Ca_Kabuli_Chr04	7143207	(G/T)
5580	CakSNP5580	*Kabuli*	Ca_Kabuli_Chr04	7143214	(T/C)
5581	CakSNP5581	*Kabuli*	Ca_Kabuli_Chr04	7143238	(C/T)
5582	CakSNP5582	*Kabuli*	Ca_Kabuli_Chr04	7155826	(G/A)
5583	CakSNP5583	*Kabuli*	Ca_Kabuli_Chr04	7185974	(G/A)
5584	CakSNP5584	*Kabuli*	Ca_Kabuli_Chr04	7231299	(C/T)
5585	CakSNP5585	*Kabuli*	Ca_Kabuli_Chr04	7239917	(T/G)

S.N.	SNP IDs	Cultivars	Chromosomes/scaffolds	Physical positions (bp)	SNPs
5586	CakSNP5586	*Kabuli*	Ca_Kabuli_Chr04	7244301	(G/A)
5587	CakSNP5587	*Kabuli*	Ca_Kabuli_Chr04	7335587	(C/T)
5588	CakSNP5588	*Kabuli*	Ca_Kabuli_Chr04	7349958	(T/C)
5589	CakSNP5589	*Kabuli*	Ca_Kabuli_Chr04	7349948	(T/C)
5590	CakSNP5590	*Kabuli*	Ca_Kabuli_Chr04	7349900	(G/A)
5591	CakSNP5591	*Kabuli*	Ca_Kabuli_Chr04	7349885	(G/A)
5592	CakSNP5592	*Kabuli*	Ca_Kabuli_Chr04	7350192	(G/T)
5593	CakSNP5593	*Kabuli*	Ca_Kabuli_Chr04	7371667	(G/A)
5594	CakSNP5594	*Kabuli*	Ca_Kabuli_Chr04	7371708	(T/C)
5595	CakSNP5595	*Kabuli*	Ca_Kabuli_Chr04	7416949	(C/T)
5596	CakSNP5596	*Kabuli*	Ca_Kabuli_Chr04	7496298	(A/G)
5597	CakSNP5597	*Kabuli*	Ca_Kabuli_Chr04	7520182	(A/T)
5598	CakSNP5598	*Kabuli*	Ca_Kabuli_Chr04	7520184	(A/C)
5599	CakSNP5599	*Kabuli*	Ca_Kabuli_Chr04	7522047	(C/T)
5600	CakSNP5600	*Kabuli*	Ca_Kabuli_Chr04	7522033	(A/G)
5601	CakSNP5601	*Kabuli*	Ca_Kabuli_Chr04	7587317	(A/G)
5602	CakSNP5602	*Kabuli*	Ca_Kabuli_Chr04	7587516	(T/C)
5603	CakSNP5603	*Kabuli*	Ca_Kabuli_Chr04	7598474	(G/C)
5604	CakSNP5604	*Kabuli*	Ca_Kabuli_Chr04	7699429	(C/T)
5605	CakSNP5605	*Kabuli*	Ca_Kabuli_Chr04	7717770	(A/G)
5606	CakSNP5606	*Kabuli*	Ca_Kabuli_Chr04	7717935	(T/G)
5607	CakSNP5607	*Kabuli*	Ca_Kabuli_Chr04	7717978	(G/A)
5608	CakSNP5608	*Kabuli*	Ca_Kabuli_Chr04	7718270	(T/C)
5609	CakSNP5609	*Kabuli*	Ca_Kabuli_Chr04	7718277	(G/A)
5610	CakSNP5610	*Kabuli*	Ca_Kabuli_Chr04	7739202	(C/T)
5611	CakSNP5611	*Kabuli*	Ca_Kabuli_Chr04	7746230	(A/C)
5612	CakSNP5612	*Kabuli*	Ca_Kabuli_Chr04	7746242	(C/G)
5613	CakSNP5613	*Kabuli*	Ca_Kabuli_Chr04	7812468	(C/T)
5614	CakSNP5614	*Kabuli*	Ca_Kabuli_Chr04	7812491	(T/A)
5615	CakSNP5615	*Kabuli*	Ca_Kabuli_Chr04	7812521	(A/G)
5616	CakSNP5616	*Kabuli*	Ca_Kabuli_Chr04	7819865	(T/C)
5617	CakSNP5617	*Kabuli*	Ca_Kabuli_Chr04	7821269	(G/A)
5618	CakSNP5618	*Kabuli*	Ca_Kabuli_Chr04	7868480	(A/T)
5619	CakSNP5619	*Kabuli*	Ca_Kabuli_Chr04	7883707	(T/A)
5620	CakSNP5620	*Kabuli*	Ca_Kabuli_Chr04	7892596	(A/C)
5621	CakSNP5621	*Kabuli*	Ca_Kabuli_Chr04	7975577	(C/G)
5622	CakSNP5622	*Kabuli*	Ca_Kabuli_Chr04	7975586	(T/G)
5623	CakSNP5623	*Kabuli*	Ca_Kabuli_Chr04	7988367	(A/G)

S.N.	SNP IDs	Cultivars	Chromosomes/scaffolds	Physical positions (bp)	SNPs
5624	CakSNP5624	*Kabuli*	Ca_Kabuli_Chr04	7988396	(C/T)
5625	CakSNP5625	*Kabuli*	Ca_Kabuli_Chr04	7988399	(G/A)
5626	CakSNP5626	*Kabuli*	Ca_Kabuli_Chr04	7988422	(T/G)
5627	CakSNP5627	*Kabuli*	Ca_Kabuli_Chr04	7988444	(T/G)
5628	CakSNP5628	*Kabuli*	Ca_Kabuli_Chr04	8025363	(T/C)
5629	CakSNP5629	*Kabuli*	Ca_Kabuli_Chr04	8025348	(A/T)
5630	CakSNP5630	*Kabuli*	Ca_Kabuli_Chr04	8025530	(A/G)
5631	CakSNP5631	*Kabuli*	Ca_Kabuli_Chr04	8050770	(T/C)
5632	CakSNP5632	*Kabuli*	Ca_Kabuli_Chr04	8051306	(T/C)
5633	CakSNP5633	*Kabuli*	Ca_Kabuli_Chr04	8117401	(G/A)
5634	CakSNP5634	*Kabuli*	Ca_Kabuli_Chr04	8178043	(G/A)
5635	CakSNP5635	*Kabuli*	Ca_Kabuli_Chr04	8198702	(T/G)
5636	CakSNP5636	*Kabuli*	Ca_Kabuli_Chr04	8203586	(C/T)
5637	CakSNP5637	*Kabuli*	Ca_Kabuli_Chr04	8236449	(C/A)
5638	CakSNP5638	*Kabuli*	Ca_Kabuli_Chr04	8237145	(C/T)
5639	CakSNP5639	*Kabuli*	Ca_Kabuli_Chr04	8269099	(T/G)
5640	CakSNP5640	*Kabuli*	Ca_Kabuli_Chr04	8269152	(C/T)
5641	CakSNP5641	*Kabuli*	Ca_Kabuli_Chr04	8269347	(C/G)
5642	CakSNP5642	*Kabuli*	Ca_Kabuli_Chr04	8302198	(A/G)
5643	CakSNP5643	*Kabuli*	Ca_Kabuli_Chr04	8383890	(G/A)
5644	CakSNP5644	*Kabuli*	Ca_Kabuli_Chr04	8396861	(T/A)
5645	CakSNP5645	*Kabuli*	Ca_Kabuli_Chr04	8396837	(T/G)
5646	CakSNP5646	*Kabuli*	Ca_Kabuli_Chr04	8401109	(A/G)
5647	CakSNP5647	*Kabuli*	Ca_Kabuli_Chr04	8401126	(G/T)
5648	CakSNP5648	*Kabuli*	Ca_Kabuli_Chr04	8401143	(A/T)
5649	CakSNP5649	*Kabuli*	Ca_Kabuli_Chr04	8401248	(T/A)
5650	CakSNP5650	*Kabuli*	Ca_Kabuli_Chr04	8401230	(A/G)
5651	CakSNP5651	*Kabuli*	Ca_Kabuli_Chr04	8404108	(T/C)
5652	CakSNP5652	*Kabuli*	Ca_Kabuli_Chr04	8404165	(C/A)
5653	CakSNP5653	*Kabuli*	Ca_Kabuli_Chr04	8404244	(C/G)
5654	CakSNP5654	*Kabuli*	Ca_Kabuli_Chr04	8471029	(G/A)
5655	CakSNP5655	*Kabuli*	Ca_Kabuli_Chr04	8473120	(G/A)
5656	CakSNP5656	*Kabuli*	Ca_Kabuli_Chr04	8473127	(G/A)
5657	CakSNP5657	*Kabuli*	Ca_Kabuli_Chr04	8473294	(G/A)
5658	CakSNP5658	*Kabuli*	Ca_Kabuli_Chr04	8473288	(A/G)
5659	CakSNP5659	*Kabuli*	Ca_Kabuli_Chr04	8473246	(T/C)
5660	CakSNP5660	*Kabuli*	Ca_Kabuli_Chr04	8474442	(C/T)
5661	CakSNP5661	*Kabuli*	Ca_Kabuli_Chr04	8474441	(G/A)

S.N.	SNP IDs	Cultivars	Chromosomes/scaffolds	Physical positions (bp)	SNPs
5662	CakSNP5662	*Kabuli*	Ca_Kabuli_Chr04	8474401	(T/A)
5663	CakSNP5663	*Kabuli*	Ca_Kabuli_Chr04	8475634	(A/C)
5664	CakSNP5664	*Kabuli*	Ca_Kabuli_Chr04	8483482	(T/C)
5665	CakSNP5665	*Kabuli*	Ca_Kabuli_Chr04	8483494	(T/C)
5666	CakSNP5666	*Kabuli*	Ca_Kabuli_Chr04	8484976	(G/A)
5667	CakSNP5667	*Kabuli*	Ca_Kabuli_Chr04	8484978	(A/G)
5668	CakSNP5668	*Kabuli*	Ca_Kabuli_Chr04	8496641	(C/A)
5669	CakSNP5669	*Kabuli*	Ca_Kabuli_Chr04	8510011	(A/C)
5670	CakSNP5670	*Kabuli*	Ca_Kabuli_Chr04	8512095	(G/A)
5671	CakSNP5671	*Kabuli*	Ca_Kabuli_Chr04	8512280	(C/T)
5672	CakSNP5672	*Kabuli*	Ca_Kabuli_Chr04	8518979	(G/A)
5673	CakSNP5673	*Kabuli*	Ca_Kabuli_Chr04	8534120	(C/T)
5674	CakSNP5674	*Kabuli*	Ca_Kabuli_Chr04	8567380	(A/T)
5675	CakSNP5675	*Kabuli*	Ca_Kabuli_Chr04	8567431	(C/T)
5676	CakSNP5676	*Kabuli*	Ca_Kabuli_Chr04	8567490	(C/A)
5677	CakSNP5677	*Kabuli*	Ca_Kabuli_Chr04	8567478	(A/C)
5678	CakSNP5678	*Kabuli*	Ca_Kabuli_Chr04	8567477	(T/C)
5679	CakSNP5679	*Kabuli*	Ca_Kabuli_Chr04	8567465	(C/T)
5680	CakSNP5680	*Kabuli*	Ca_Kabuli_Chr04	8570458	(T/G)
5681	CakSNP5681	*Kabuli*	Ca_Kabuli_Chr04	8570501	(G/C)
5682	CakSNP5682	*Kabuli*	Ca_Kabuli_Chr04	8595427	(C/T)
5683	CakSNP5683	*Kabuli*	Ca_Kabuli_Chr04	8601364	(T/G)
5684	CakSNP5684	*Kabuli*	Ca_Kabuli_Chr04	8659774	(G/A)
5685	CakSNP5685	*Kabuli*	Ca_Kabuli_Chr04	8659935	(T/A)
5686	CakSNP5686	*Kabuli*	Ca_Kabuli_Chr04	8659946	(C/T)
5687	CakSNP5687	*Kabuli*	Ca_Kabuli_Chr04	8659985	(T/C)
5688	CakSNP5688	*Kabuli*	Ca_Kabuli_Chr04	8660115	(A/T)
5689	CakSNP5689	*Kabuli*	Ca_Kabuli_Chr04	8660136	(G/A)
5690	CakSNP5690	*Kabuli*	Ca_Kabuli_Chr04	8660608	(G/T)
5691	CakSNP5691	*Kabuli*	Ca_Kabuli_Chr04	8660599	(A/G)
5692	CakSNP5692	*Kabuli*	Ca_Kabuli_Chr04	8668648	(G/A)
5693	CakSNP5693	*Kabuli*	Ca_Kabuli_Chr04	8668753	(A/G)
5694	CakSNP5694	*Kabuli*	Ca_Kabuli_Chr04	8668945	(T/C)
5695	CakSNP5695	*Kabuli*	Ca_Kabuli_Chr04	8669014	(C/G)
5696	CakSNP5696	*Kabuli*	Ca_Kabuli_Chr04	8669104	(T/C)
5697	CakSNP5697	*Kabuli*	Ca_Kabuli_Chr04	8669515	(C/T)
5698	CakSNP5698	*Kabuli*	Ca_Kabuli_Chr04	8669513	(G/A)
5699	CakSNP5699	*Kabuli*	Ca_Kabuli_Chr04	8669505	(G/T)

S.N.	SNP IDs	Cultivars	Chromosomes/scaffolds	Physical positions (bp)	SNPs
5700	CakSNP5700	*Kabuli*	Ca_Kabuli_Chr04	8681949	(G/A)
5701	CakSNP5701	*Kabuli*	Ca_Kabuli_Chr04	8695562	(C/T)
5702	CakSNP5702	*Kabuli*	Ca_Kabuli_Chr04	8751638	(A/C)
5703	CakSNP5703	*Kabuli*	Ca_Kabuli_Chr04	8751629	(T/C)
5704	CakSNP5704	*Kabuli*	Ca_Kabuli_Chr04	8751618	(A/C)
5705	CakSNP5705	*Kabuli*	Ca_Kabuli_Chr04	8751591	(A/C)
5706	CakSNP5706	*Kabuli*	Ca_Kabuli_Chr04	8772215	(A/C)
5707	CakSNP5707	*Kabuli*	Ca_Kabuli_Chr04	8782879	(G/C)
5708	CakSNP5708	*Kabuli*	Ca_Kabuli_Chr04	8791654	(A/G)
5709	CakSNP5709	*Kabuli*	Ca_Kabuli_Chr04	8805504	(A/G)
5710	CakSNP5710	*Kabuli*	Ca_Kabuli_Chr04	8805578	(A/C)
5711	CakSNP5711	*Kabuli*	Ca_Kabuli_Chr04	8805667	(C/T)
5712	CakSNP5712	*Kabuli*	Ca_Kabuli_Chr04	8805867	(A/C)
5713	CakSNP5713	*Kabuli*	Ca_Kabuli_Chr04	8805839	(G/A)
5714	CakSNP5714	*Kabuli*	Ca_Kabuli_Chr04	8809032	(C/T)
5715	CakSNP5715	*Kabuli*	Ca_Kabuli_Chr04	8809064	(T/C)
5716	CakSNP5716	*Kabuli*	Ca_Kabuli_Chr04	8809010	(A/G)
5717	CakSNP5717	*Kabuli*	Ca_Kabuli_Chr04	8809456	(A/C)
5718	CakSNP5718	*Kabuli*	Ca_Kabuli_Chr04	8844846	(A/G)
5719	CakSNP5719	*Kabuli*	Ca_Kabuli_Chr04	8848654	(C/T)
5720	CakSNP5720	*Kabuli*	Ca_Kabuli_Chr04	8848653	(T/C)
5721	CakSNP5721	*Kabuli*	Ca_Kabuli_Chr04	8852334	(T/A)
5722	CakSNP5722	*Kabuli*	Ca_Kabuli_Chr04	8852420	(T/C)
5723	CakSNP5723	*Kabuli*	Ca_Kabuli_Chr04	8873494	(G/T)
5724	CakSNP5724	*Kabuli*	Ca_Kabuli_Chr04	8873509	(G/C)
5725	CakSNP5725	*Kabuli*	Ca_Kabuli_Chr04	8919264	(C/T)
5726	CakSNP5726	*Kabuli*	Ca_Kabuli_Chr04	8919300	(C/T)
5727	CakSNP5727	*Kabuli*	Ca_Kabuli_Chr04	8919395	(A/G)
5728	CakSNP5728	*Kabuli*	Ca_Kabuli_Chr04	8952236	(A/G)
5729	CakSNP5729	*Kabuli*	Ca_Kabuli_Chr04	8985949	(C/T)
5730	CakSNP5730	*Kabuli*	Ca_Kabuli_Chr04	8985961	(C/T)
5731	CakSNP5731	*Kabuli*	Ca_Kabuli_Chr04	8986031	(T/A)
5732	CakSNP5732	*Kabuli*	Ca_Kabuli_Chr04	8986026	(T/A)
5733	CakSNP5733	*Kabuli*	Ca_Kabuli_Chr04	8986024	(T/A)
5734	CakSNP5734	*Kabuli*	Ca_Kabuli_Chr04	8986018	(G/A)
5735	CakSNP5735	*Kabuli*	Ca_Kabuli_Chr04	8986008	(T/A)
5736	CakSNP5736	*Kabuli*	Ca_Kabuli_Chr04	8986002	(T/A)
5737	CakSNP5737	*Kabuli*	Ca_Kabuli_Chr04	8985998	(T/A)

S.N.	SNP IDs	Cultivars	Chromosomes/scaffolds	Physical positions (bp)	SNPs
5738	CakSNP5738	*Kabuli*	Ca_Kabuli_Chr04	8985994	(C/A)
5739	CakSNP5739	*Kabuli*	Ca_Kabuli_Chr04	8986207	(G/C)
5740	CakSNP5740	*Kabuli*	Ca_Kabuli_Chr04	8986217	(T/A)
5741	CakSNP5741	*Kabuli*	Ca_Kabuli_Chr04	9075398	(G/A)
5742	CakSNP5742	*Kabuli*	Ca_Kabuli_Chr04	9075395	(A/G)
5743	CakSNP5743	*Kabuli*	Ca_Kabuli_Chr04	9075459	(C/T)
5744	CakSNP5744	*Kabuli*	Ca_Kabuli_Chr04	9075520	(C/T)
5745	CakSNP5745	*Kabuli*	Ca_Kabuli_Chr04	9075565	(A/G)
5746	CakSNP5746	*Kabuli*	Ca_Kabuli_Chr04	9075543	(T/C)
5747	CakSNP5747	*Kabuli*	Ca_Kabuli_Chr04	9075671	(A/G)
5748	CakSNP5748	*Kabuli*	Ca_Kabuli_Chr04	9075677	(T/C)
5749	CakSNP5749	*Kabuli*	Ca_Kabuli_Chr04	9075682	(G/A)
5750	CakSNP5750	*Kabuli*	Ca_Kabuli_Chr04	9075737	(G/A)
5751	CakSNP5751	*Kabuli*	Ca_Kabuli_Chr04	9075744	(T/G)
5752	CakSNP5752	*Kabuli*	Ca_Kabuli_Chr04	9187887	(G/C)
5753	CakSNP5753	*Kabuli*	Ca_Kabuli_Chr04	9187973	(T/G)
5754	CakSNP5754	*Kabuli*	Ca_Kabuli_Chr04	9189976	(T/A)
5755	CakSNP5755	*Kabuli*	Ca_Kabuli_Chr04	9211570	(A/G)
5756	CakSNP5756	*Kabuli*	Ca_Kabuli_Chr04	9257131	(G/A)
5757	CakSNP5757	*Kabuli*	Ca_Kabuli_Chr04	9331949	(C/T)
5758	CakSNP5758	*Kabuli*	Ca_Kabuli_Chr04	9392373	(A/T)
5759	CakSNP5759	*Kabuli*	Ca_Kabuli_Chr04	9392455	(A/G)
5760	CakSNP5760	*Kabuli*	Ca_Kabuli_Chr04	9392427	(G/A)
5761	CakSNP5761	*Kabuli*	Ca_Kabuli_Chr04	9427890	(T/G)
5762	CakSNP5762	*Kabuli*	Ca_Kabuli_Chr04	9427892	(G/T)
5763	CakSNP5763	*Kabuli*	Ca_Kabuli_Chr04	9427894	(A/C)
5764	CakSNP5764	*Kabuli*	Ca_Kabuli_Chr04	9427964	(C/A)
5765	CakSNP5765	*Kabuli*	Ca_Kabuli_Chr04	9451895	(T/A)
5766	CakSNP5766	*Kabuli*	Ca_Kabuli_Chr04	9451850	(T/G)
5767	CakSNP5767	*Kabuli*	Ca_Kabuli_Chr04	9491741	(A/T)
5768	CakSNP5768	*Kabuli*	Ca_Kabuli_Chr04	9533658	(A/G)
5769	CakSNP5769	*Kabuli*	Ca_Kabuli_Chr04	9533688	(T/G)
5770	CakSNP5770	*Kabuli*	Ca_Kabuli_Chr04	9579383	(C/A)
5771	CakSNP5771	*Kabuli*	Ca_Kabuli_Chr04	9579393	(G/A)
5772	CakSNP5772	*Kabuli*	Ca_Kabuli_Chr04	9579401	(G/T)
5773	CakSNP5773	*Kabuli*	Ca_Kabuli_Chr04	9579519	(A/T)
5774	CakSNP5774	*Kabuli*	Ca_Kabuli_Chr04	9579517	(T/C)
5775	CakSNP5775	*Kabuli*	Ca_Kabuli_Chr04	9580341	(T/A)

S.N.	SNP IDs	Cultivars	Chromosomes/scaffolds	Physical positions (bp)	SNPs
5776	CakSNP5776	*Kabuli*	Ca_Kabuli_Chr04	9580339	(T/G)
5777	CakSNP5777	*Kabuli*	Ca_Kabuli_Chr04	9614897	(G/A)
5778	CakSNP5778	*Kabuli*	Ca_Kabuli_Chr04	9614905	(C/A)
5779	CakSNP5779	*Kabuli*	Ca_Kabuli_Chr04	9661685	(G/A)
5780	CakSNP5780	*Kabuli*	Ca_Kabuli_Chr04	9670905	(C/T)
5781	CakSNP5781	*Kabuli*	Ca_Kabuli_Chr04	9670996	(G/T)
5782	CakSNP5782	*Kabuli*	Ca_Kabuli_Chr04	9671007	(G/T)
5783	CakSNP5783	*Kabuli*	Ca_Kabuli_Chr04	9671089	(T/G)
5784	CakSNP5784	*Kabuli*	Ca_Kabuli_Chr04	9706453	(T/G)
5785	CakSNP5785	*Kabuli*	Ca_Kabuli_Chr04	9706480	(T/A)
5786	CakSNP5786	*Kabuli*	Ca_Kabuli_Chr04	9706481	(A/C)
5787	CakSNP5787	*Kabuli*	Ca_Kabuli_Chr04	9740099	(A/G)
5788	CakSNP5788	*Kabuli*	Ca_Kabuli_Chr04	9800865	(C/T)
5789	CakSNP5789	*Kabuli*	Ca_Kabuli_Chr04	9802603	(T/C)
5790	CakSNP5790	*Kabuli*	Ca_Kabuli_Chr04	9836559	(G/T)
5791	CakSNP5791	*Kabuli*	Ca_Kabuli_Chr04	9840763	(C/A)
5792	CakSNP5792	*Kabuli*	Ca_Kabuli_Chr04	9840906	(A/C)
5793	CakSNP5793	*Kabuli*	Ca_Kabuli_Chr04	9846321	(T/A)
5794	CakSNP5794	*Kabuli*	Ca_Kabuli_Chr04	9947077	(A/G)
5795	CakSNP5795	*Kabuli*	Ca_Kabuli_Chr04	9973728	(C/G)
5796	CakSNP5796	*Kabuli*	Ca_Kabuli_Chr04	9976039	(C/T)
5797	CakSNP5797	*Kabuli*	Ca_Kabuli_Chr04	9999907	(A/T)
5798	CakSNP5798	*Kabuli*	Ca_Kabuli_Chr04	10007069	(A/G)
5799	CakSNP5799	*Kabuli*	Ca_Kabuli_Chr04	10007151	(C/T)
5800	CakSNP5800	*Kabuli*	Ca_Kabuli_Chr04	10007193	(T/A)
5801	CakSNP5801	*Kabuli*	Ca_Kabuli_Chr04	10007332	(T/C)
5802	CakSNP5802	*Kabuli*	Ca_Kabuli_Chr04	10007286	(C/T)
5803	CakSNP5803	*Kabuli*	Ca_Kabuli_Chr04	10007372	(C/A)
5804	CakSNP5804	*Kabuli*	Ca_Kabuli_Chr04	10009839	(G/A)
5805	CakSNP5805	*Kabuli*	Ca_Kabuli_Chr04	10013120	(G/A)
5806	CakSNP5806	*Kabuli*	Ca_Kabuli_Chr04	10013148	(G/T)
5807	CakSNP5807	*Kabuli*	Ca_Kabuli_Chr04	10017467	(A/C)
5808	CakSNP5808	*Kabuli*	Ca_Kabuli_Chr04	10024226	(G/A)
5809	CakSNP5809	*Kabuli*	Ca_Kabuli_Chr04	10030134	(A/G)
5810	CakSNP5810	*Kabuli*	Ca_Kabuli_Chr04	10116516	(T/G)
5811	CakSNP5811	*Kabuli*	Ca_Kabuli_Chr04	10151711	(A/C)
5812	CakSNP5812	*Kabuli*	Ca_Kabuli_Chr04	10151943	(C/T)
5813	CakSNP5813	*Kabuli*	Ca_Kabuli_Chr04	10184428	(G/T)

S.N.	SNP IDs	Cultivars	Chromosomes/scaffolds	Physical positions (bp)	SNPs
5814	CakSNP5814	*Kabuli*	Ca_Kabuli_Chr04	10184427	(C/A)
5815	CakSNP5815	*Kabuli*	Ca_Kabuli_Chr04	10185602	(T/G)
5816	CakSNP5816	*Kabuli*	Ca_Kabuli_Chr04	10185625	(A/G)
5817	CakSNP5817	*Kabuli*	Ca_Kabuli_Chr04	10205246	(C/T)
5818	CakSNP5818	*Kabuli*	Ca_Kabuli_Chr04	10290706	(G/T)
5819	CakSNP5819	*Kabuli*	Ca_Kabuli_Chr04	10293203	(T/C)
5820	CakSNP5820	*Kabuli*	Ca_Kabuli_Chr04	10293241	(C/G)
5821	CakSNP5821	*Kabuli*	Ca_Kabuli_Chr04	10294380	(A/G)
5822	CakSNP5822	*Kabuli*	Ca_Kabuli_Chr04	10305276	(G/A)
5823	CakSNP5823	*Kabuli*	Ca_Kabuli_Chr04	10305235	(A/G)
5824	CakSNP5824	*Kabuli*	Ca_Kabuli_Chr04	10305285	(C/A)
5825	CakSNP5825	*Kabuli*	Ca_Kabuli_Chr04	10411809	(G/A)
5826	CakSNP5826	*Kabuli*	Ca_Kabuli_Chr04	10422142	(A/T)
5827	CakSNP5827	*Kabuli*	Ca_Kabuli_Chr04	10422469	(C/A)
5828	CakSNP5828	*Kabuli*	Ca_Kabuli_Chr04	10430622	(A/C)
5829	CakSNP5829	*Kabuli*	Ca_Kabuli_Chr04	10430617	(A/C)
5830	CakSNP5830	*Kabuli*	Ca_Kabuli_Chr04	10430613	(C/A)
5831	CakSNP5831	*Kabuli*	Ca_Kabuli_Chr04	10430603	(G/A)
5832	CakSNP5832	*Kabuli*	Ca_Kabuli_Chr04	10430595	(A/C)
5833	CakSNP5833	*Kabuli*	Ca_Kabuli_Chr04	10430588	(T/C)
5834	CakSNP5834	*Kabuli*	Ca_Kabuli_Chr04	10430574	(A/T)
5835	CakSNP5835	*Kabuli*	Ca_Kabuli_Chr04	10464927	(T/G)
5836	CakSNP5836	*Kabuli*	Ca_Kabuli_Chr04	10465328	(T/C)
5837	CakSNP5837	*Kabuli*	Ca_Kabuli_Chr04	10547575	(G/A)
5838	CakSNP5838	*Kabuli*	Ca_Kabuli_Chr04	10547561	(T/C)
5839	CakSNP5839	*Kabuli*	Ca_Kabuli_Chr04	10642002	(A/G)
5840	CakSNP5840	*Kabuli*	Ca_Kabuli_Chr04	10642043	(T/G)
5841	CakSNP5841	*Kabuli*	Ca_Kabuli_Chr04	10642044	(A/T)
5842	CakSNP5842	*Kabuli*	Ca_Kabuli_Chr04	10643339	(T/A)
5843	CakSNP5843	*Kabuli*	Ca_Kabuli_Chr04	10643331	(T/A)
5844	CakSNP5844	*Kabuli*	Ca_Kabuli_Chr04	10644245	(G/A)
5845	CakSNP5845	*Kabuli*	Ca_Kabuli_Chr04	10670664	(C/A)
5846	CakSNP5846	*Kabuli*	Ca_Kabuli_Chr04	10699624	(A/T)
5847	CakSNP5847	*Kabuli*	Ca_Kabuli_Chr04	10780990	(A/G)
5848	CakSNP5848	*Kabuli*	Ca_Kabuli_Chr04	10807993	(T/C)
5849	CakSNP5849	*Kabuli*	Ca_Kabuli_Chr04	10827485	(G/A)
5850	CakSNP5850	*Kabuli*	Ca_Kabuli_Chr04	10827515	(C/G)
5851	CakSNP5851	*Kabuli*	Ca_Kabuli_Chr04	10901282	(T/C)

S.N.	SNP IDs	Cultivars	Chromosomes/scaffolds	Physical positions (bp)	SNPs
5852	CakSNP5852	*Kabuli*	Ca_Kabuli_Chr04	10917723	(T/C)
5853	CakSNP5853	*Kabuli*	Ca_Kabuli_Chr04	10917717	(C/G)
5854	CakSNP5854	*Kabuli*	Ca_Kabuli_Chr04	10925490	(G/A)
5855	CakSNP5855	*Kabuli*	Ca_Kabuli_Chr04	10928923	(T/C)
5856	CakSNP5856	*Kabuli*	Ca_Kabuli_Chr04	10950823	(C/T)
5857	CakSNP5857	*Kabuli*	Ca_Kabuli_Chr04	10971039	(C/A)
5858	CakSNP5858	*Kabuli*	Ca_Kabuli_Chr04	10990000	(T/C)
5859	CakSNP5859	*Kabuli*	Ca_Kabuli_Chr04	10990008	(A/T)
5860	CakSNP5860	*Kabuli*	Ca_Kabuli_Chr04	10990049	(G/T)
5861	CakSNP5861	*Kabuli*	Ca_Kabuli_Chr04	10990058	(T/C)
5862	CakSNP5862	*Kabuli*	Ca_Kabuli_Chr04	10990265	(T/A)
5863	CakSNP5863	*Kabuli*	Ca_Kabuli_Chr04	10990261	(A/G)
5864	CakSNP5864	*Kabuli*	Ca_Kabuli_Chr04	10991842	(G/A)
5865	CakSNP5865	*Kabuli*	Ca_Kabuli_Chr04	10991910	(C/T)
5866	CakSNP5866	*Kabuli*	Ca_Kabuli_Chr04	11040108	(C/T)
5867	CakSNP5867	*Kabuli*	Ca_Kabuli_Chr04	11051335	(C/T)
5868	CakSNP5868	*Kabuli*	Ca_Kabuli_Chr04	11059403	(T/G)
5869	CakSNP5869	*Kabuli*	Ca_Kabuli_Chr04	11059404	(T/G)
5870	CakSNP5870	*Kabuli*	Ca_Kabuli_Chr04	11059414	(T/G)
5871	CakSNP5871	*Kabuli*	Ca_Kabuli_Chr04	11059572	(G/A)
5872	CakSNP5872	*Kabuli*	Ca_Kabuli_Chr04	11059540	(T/C)
5873	CakSNP5873	*Kabuli*	Ca_Kabuli_Chr04	11096124	(A/G)
5874	CakSNP5874	*Kabuli*	Ca_Kabuli_Chr04	11096225	(A/G)
5875	CakSNP5875	*Kabuli*	Ca_Kabuli_Chr04	11096290	(G/A)
5876	CakSNP5876	*Kabuli*	Ca_Kabuli_Chr04	11113277	(A/C)
5877	CakSNP5877	*Kabuli*	Ca_Kabuli_Chr04	11114238	(T/C)
5878	CakSNP5878	*Kabuli*	Ca_Kabuli_Chr04	11142202	(A/G)
5879	CakSNP5879	*Kabuli*	Ca_Kabuli_Chr04	11142227	(C/T)
5880	CakSNP5880	*Kabuli*	Ca_Kabuli_Chr04	11196803	(T/G)
5881	CakSNP5881	*Kabuli*	Ca_Kabuli_Chr04	11230403	(G/T)
5882	CakSNP5882	*Kabuli*	Ca_Kabuli_Chr04	11230400	(T/A)
5883	CakSNP5883	*Kabuli*	Ca_Kabuli_Chr04	11230397	(G/C)
5884	CakSNP5884	*Kabuli*	Ca_Kabuli_Chr04	11231142	(A/G)
5885	CakSNP5885	*Kabuli*	Ca_Kabuli_Chr04	11231137	(A/G)
5886	CakSNP5886	*Kabuli*	Ca_Kabuli_Chr04	11231135	(C/T)
5887	CakSNP5887	*Kabuli*	Ca_Kabuli_Chr04	11244334	(T/G)
5888	CakSNP5888	*Kabuli*	Ca_Kabuli_Chr04	11244395	(G/T)
5889	CakSNP5889	*Kabuli*	Ca_Kabuli_Chr04	11246093	(G/A)

S.N.	SNP IDs	Cultivars	Chromosomes/scaffolds	Physical positions (bp)	SNPs
5890	CakSNP5890	*Kabuli*	Ca_Kabuli_Chr04	11246173	(C/T)
5891	CakSNP5891	*Kabuli*	Ca_Kabuli_Chr04	11246164	(G/A)
5892	CakSNP5892	*Kabuli*	Ca_Kabuli_Chr04	11273328	(T/C)
5893	CakSNP5893	*Kabuli*	Ca_Kabuli_Chr04	11273405	(A/T)
5894	CakSNP5894	*Kabuli*	Ca_Kabuli_Chr04	11274281	(T/C)
5895	CakSNP5895	*Kabuli*	Ca_Kabuli_Chr04	11275171	(T/C)
5896	CakSNP5896	*Kabuli*	Ca_Kabuli_Chr04	11276413	(C/G)
5897	CakSNP5897	*Kabuli*	Ca_Kabuli_Chr04	11276484	(T/C)
5898	CakSNP5898	*Kabuli*	Ca_Kabuli_Chr04	11277138	(A/G)
5899	CakSNP5899	*Kabuli*	Ca_Kabuli_Chr04	11277297	(G/A)
5900	CakSNP5900	*Kabuli*	Ca_Kabuli_Chr04	11277574	(G/C)
5901	CakSNP5901	*Kabuli*	Ca_Kabuli_Chr04	11334350	(A/G)
5902	CakSNP5902	*Kabuli*	Ca_Kabuli_Chr04	11334343	(A/T)
5903	CakSNP5903	*Kabuli*	Ca_Kabuli_Chr04	11343257	(T/C)
5904	CakSNP5904	*Kabuli*	Ca_Kabuli_Chr04	11343230	(G/C)
5905	CakSNP5905	*Kabuli*	Ca_Kabuli_Chr04	11379077	(T/C)
5906	CakSNP5906	*Kabuli*	Ca_Kabuli_Chr04	11398699	(A/G)
5907	CakSNP5907	*Kabuli*	Ca_Kabuli_Chr04	11398682	(G/A)
5908	CakSNP5908	*Kabuli*	Ca_Kabuli_Chr04	11398889	(A/G)
5909	CakSNP5909	*Kabuli*	Ca_Kabuli_Chr04	11414506	(C/T)
5910	CakSNP5910	*Kabuli*	Ca_Kabuli_Chr04	11435651	(A/G)
5911	CakSNP5911	*Kabuli*	Ca_Kabuli_Chr04	11441604	(T/A)
5912	CakSNP5912	*Kabuli*	Ca_Kabuli_Chr04	11441615	(T/C)
5913	CakSNP5913	*Kabuli*	Ca_Kabuli_Chr04	11441619	(G/A)
5914	CakSNP5914	*Kabuli*	Ca_Kabuli_Chr04	11441655	(T/A)
5915	CakSNP5915	*Kabuli*	Ca_Kabuli_Chr04	11441707	(A/C)
5916	CakSNP5916	*Kabuli*	Ca_Kabuli_Chr04	11465057	(C/G)
5917	CakSNP5917	*Kabuli*	Ca_Kabuli_Chr04	11465075	(T/C)
5918	CakSNP5918	*Kabuli*	Ca_Kabuli_Chr04	11465113	(C/T)
5919	CakSNP5919	*Kabuli*	Ca_Kabuli_Chr04	11490100	(C/T)
5920	CakSNP5920	*Kabuli*	Ca_Kabuli_Chr04	11490154	(A/T)
5921	CakSNP5921	*Kabuli*	Ca_Kabuli_Chr04	11490125	(G/T)
5922	CakSNP5922	*Kabuli*	Ca_Kabuli_Chr04	11490496	(G/A)
5923	CakSNP5923	*Kabuli*	Ca_Kabuli_Chr04	11517582	(C/T)
5924	CakSNP5924	*Kabuli*	Ca_Kabuli_Chr04	11517517	(C/A)
5925	CakSNP5925	*Kabuli*	Ca_Kabuli_Chr04	11572051	(C/T)
5926	CakSNP5926	*Kabuli*	Ca_Kabuli_Chr04	11645738	(A/G)
5927	CakSNP5927	*Kabuli*	Ca_Kabuli_Chr04	11646347	(C/G)

S.N.	SNP IDs	Cultivars	Chromosomes/scaffolds	Physical positions (bp)	SNPs
5928	CakSNP5928	*Kabuli*	Ca_Kabuli_Chr04	11646453	(G/A)
5929	CakSNP5929	*Kabuli*	Ca_Kabuli_Chr04	11658323	(A/G)
5930	CakSNP5930	*Kabuli*	Ca_Kabuli_Chr04	11658314	(A/G)
5931	CakSNP5931	*Kabuli*	Ca_Kabuli_Chr04	11689611	(C/T)
5932	CakSNP5932	*Kabuli*	Ca_Kabuli_Chr04	11689647	(T/C)
5933	CakSNP5933	*Kabuli*	Ca_Kabuli_Chr04	11752862	(A/G)
5934	CakSNP5934	*Kabuli*	Ca_Kabuli_Chr04	11752855	(A/G)
5935	CakSNP5935	*Kabuli*	Ca_Kabuli_Chr04	11772023	(G/T)
5936	CakSNP5936	*Kabuli*	Ca_Kabuli_Chr04	11838005	(C/T)
5937	CakSNP5937	*Kabuli*	Ca_Kabuli_Chr04	11852291	(A/G)
5938	CakSNP5938	*Kabuli*	Ca_Kabuli_Chr04	11926352	(G/C)
5939	CakSNP5939	*Kabuli*	Ca_Kabuli_Chr04	11985628	(G/A)
5940	CakSNP5940	*Kabuli*	Ca_Kabuli_Chr04	11985604	(C/T)
5941	CakSNP5941	*Kabuli*	Ca_Kabuli_Chr04	12004082	(A/T)
5942	CakSNP5942	*Kabuli*	Ca_Kabuli_Chr04	12004209	(C/T)
5943	CakSNP5943	*Kabuli*	Ca_Kabuli_Chr04	12004205	(A/C)
5944	CakSNP5944	*Kabuli*	Ca_Kabuli_Chr04	12023137	(A/T)
5945	CakSNP5945	*Kabuli*	Ca_Kabuli_Chr04	12023215	(G/A)
5946	CakSNP5946	*Kabuli*	Ca_Kabuli_Chr04	12045899	(T/A)
5947	CakSNP5947	*Kabuli*	Ca_Kabuli_Chr04	12045903	(C/A)
5948	CakSNP5948	*Kabuli*	Ca_Kabuli_Chr04	12045906	(C/A)
5949	CakSNP5949	*Kabuli*	Ca_Kabuli_Chr04	12045910	(G/A)
5950	CakSNP5950	*Kabuli*	Ca_Kabuli_Chr04	12070990	(T/A)
5951	CakSNP5951	*Kabuli*	Ca_Kabuli_Chr04	12070994	(C/A)
5952	CakSNP5952	*Kabuli*	Ca_Kabuli_Chr04	12070997	(C/A)
5953	CakSNP5953	*Kabuli*	Ca_Kabuli_Chr04	12071001	(G/A)
5954	CakSNP5954	*Kabuli*	Ca_Kabuli_Chr04	12167328	(A/G)
5955	CakSNP5955	*Kabuli*	Ca_Kabuli_Chr04	12167568	(G/A)
5956	CakSNP5956	*Kabuli*	Ca_Kabuli_Chr04	12212917	(G/A)
5957	CakSNP5957	*Kabuli*	Ca_Kabuli_Chr04	12213071	(C/A)
5958	CakSNP5958	*Kabuli*	Ca_Kabuli_Chr04	12213046	(T/C)
5959	CakSNP5959	*Kabuli*	Ca_Kabuli_Chr04	12234358	(C/A)
5960	CakSNP5960	*Kabuli*	Ca_Kabuli_Chr04	12249494	(T/C)
5961	CakSNP5961	*Kabuli*	Ca_Kabuli_Chr04	12251184	(G/A)
5962	CakSNP5962	*Kabuli*	Ca_Kabuli_Chr04	12274290	(A/T)
5963	CakSNP5963	*Kabuli*	Ca_Kabuli_Chr04	12316847	(T/C)
5964	CakSNP5964	*Kabuli*	Ca_Kabuli_Chr04	12431895	(C/T)
5965	CakSNP5965	*Kabuli*	Ca_Kabuli_Chr04	12431915	(A/G)

S.N.	SNP IDs	Cultivars	Chromosomes/scaffolds	Physical positions (bp)	SNPs
5966	CakSNP5966	*Kabuli*	Ca_Kabuli_Chr04	12484911	(A/T)
5967	CakSNP5967	*Kabuli*	Ca_Kabuli_Chr04	12506440	(A/G)
5968	CakSNP5968	*Kabuli*	Ca_Kabuli_Chr04	12506450	(T/A)
5969	CakSNP5969	*Kabuli*	Ca_Kabuli_Chr04	12514728	(G/A)
5970	CakSNP5970	*Kabuli*	Ca_Kabuli_Chr04	12514726	(C/T)
5971	CakSNP5971	*Kabuli*	Ca_Kabuli_Chr04	12514724	(T/C)
5972	CakSNP5972	*Kabuli*	Ca_Kabuli_Chr04	12514706	(G/A)
5973	CakSNP5973	*Kabuli*	Ca_Kabuli_Chr04	12525328	(A/G)
5974	CakSNP5974	*Kabuli*	Ca_Kabuli_Chr04	12525358	(C/G)
5975	CakSNP5975	*Kabuli*	Ca_Kabuli_Chr04	12525639	(T/A)
5976	CakSNP5976	*Kabuli*	Ca_Kabuli_Chr04	12534847	(T/C)
5977	CakSNP5977	*Kabuli*	Ca_Kabuli_Chr04	12558541	(T/C)
5978	CakSNP5978	*Kabuli*	Ca_Kabuli_Chr04	12558680	(C/T)
5979	CakSNP5979	*Kabuli*	Ca_Kabuli_Chr04	12568707	(G/A)
5980	CakSNP5980	*Kabuli*	Ca_Kabuli_Chr04	12568845	(G/T)
5981	CakSNP5981	*Kabuli*	Ca_Kabuli_Chr04	12568905	(G/A)
5982	CakSNP5982	*Kabuli*	Ca_Kabuli_Chr04	12568944	(A/G)
5983	CakSNP5983	*Kabuli*	Ca_Kabuli_Chr04	12568995	(G/A)
5984	CakSNP5984	*Kabuli*	Ca_Kabuli_Chr04	12569133	(G/A)
5985	CakSNP5985	*Kabuli*	Ca_Kabuli_Chr04	12574650	(G/A)
5986	CakSNP5986	*Kabuli*	Ca_Kabuli_Chr04	12578259	(A/G)
5987	CakSNP5987	*Kabuli*	Ca_Kabuli_Chr04	12578350	(A/G)
5988	CakSNP5988	*Kabuli*	Ca_Kabuli_Chr04	12581371	(G/T)
5989	CakSNP5989	*Kabuli*	Ca_Kabuli_Chr04	12692925	(C/T)
5990	CakSNP5990	*Kabuli*	Ca_Kabuli_Chr04	12692958	(G/C)
5991	CakSNP5991	*Kabuli*	Ca_Kabuli_Chr04	12693000	(T/C)
5992	CakSNP5992	*Kabuli*	Ca_Kabuli_Chr04	12740330	(A/G)
5993	CakSNP5993	*Kabuli*	Ca_Kabuli_Chr04	12740351	(T/G)
5994	CakSNP5994	*Kabuli*	Ca_Kabuli_Chr04	12779146	(A/C)
5995	CakSNP5995	*Kabuli*	Ca_Kabuli_Chr04	12780274	(C/T)
5996	CakSNP5996	*Kabuli*	Ca_Kabuli_Chr04	12795036	(G/A)
5997	CakSNP5997	*Kabuli*	Ca_Kabuli_Chr04	12795037	(G/A)
5998	CakSNP5998	*Kabuli*	Ca_Kabuli_Chr04	12869023	(C/T)
5999	CakSNP5999	*Kabuli*	Ca_Kabuli_Chr04	12869049	(C/T)
6000	CakSNP6000	*Kabuli*	Ca_Kabuli_Chr04	12907910	(C/A)
6001	CakSNP6001	*Kabuli*	Ca_Kabuli_Chr04	12908080	(C/T)
6002	CakSNP6002	*Kabuli*	Ca_Kabuli_Chr04	12911860	(A/G)
6003	CakSNP6003	*Kabuli*	Ca_Kabuli_Chr04	12911874	(T/C)

S.N.	SNP IDs	Cultivars	Chromosomes/scaffolds	Physical positions (bp)	SNPs
6004	CakSNP6004	*Kabuli*	Ca_Kabuli_Chr04	12911888	(G/C)
6005	CakSNP6005	*Kabuli*	Ca_Kabuli_Chr04	12912015	(G/A)
6006	CakSNP6006	*Kabuli*	Ca_Kabuli_Chr04	12955130	(G/A)
6007	CakSNP6007	*Kabuli*	Ca_Kabuli_Chr04	12973657	(C/A)
6008	CakSNP6008	*Kabuli*	Ca_Kabuli_Chr04	12982420	(G/A)
6009	CakSNP6009	*Kabuli*	Ca_Kabuli_Chr04	13018788	(C/G)
6010	CakSNP6010	*Kabuli*	Ca_Kabuli_Chr04	13020891	(A/G)
6011	CakSNP6011	*Kabuli*	Ca_Kabuli_Chr04	13050470	(T/C)
6012	CakSNP6012	*Kabuli*	Ca_Kabuli_Chr04	13050516	(A/G)
6013	CakSNP6013	*Kabuli*	Ca_Kabuli_Chr04	13050527	(C/T)
6014	CakSNP6014	*Kabuli*	Ca_Kabuli_Chr04	13050585	(C/T)
6015	CakSNP6015	*Kabuli*	Ca_Kabuli_Chr04	13062744	(T/A)
6016	CakSNP6016	*Kabuli*	Ca_Kabuli_Chr04	13062761	(A/T)
6017	CakSNP6017	*Kabuli*	Ca_Kabuli_Chr04	13062805	(C/G)
6018	CakSNP6018	*Kabuli*	Ca_Kabuli_Chr04	13062972	(A/T)
6019	CakSNP6019	*Kabuli*	Ca_Kabuli_Chr04	13063689	(T/A)
6020	CakSNP6020	*Kabuli*	Ca_Kabuli_Chr04	13063699	(G/T)
6021	CakSNP6021	*Kabuli*	Ca_Kabuli_Chr04	13069574	(T/C)
6022	CakSNP6022	*Kabuli*	Ca_Kabuli_Chr04	13071169	(T/A)
6023	CakSNP6023	*Kabuli*	Ca_Kabuli_Chr04	13071199	(A/T)
6024	CakSNP6024	*Kabuli*	Ca_Kabuli_Chr04	13071230	(T/G)
6025	CakSNP6025	*Kabuli*	Ca_Kabuli_Chr04	13072114	(T/A)
6026	CakSNP6026	*Kabuli*	Ca_Kabuli_Chr04	13072118	(T/G)
6027	CakSNP6027	*Kabuli*	Ca_Kabuli_Chr04	13072208	(C/A)
6028	CakSNP6028	*Kabuli*	Ca_Kabuli_Chr04	13082999	(A/G)
6029	CakSNP6029	*Kabuli*	Ca_Kabuli_Chr04	13102381	(G/A)
6030	CakSNP6030	*Kabuli*	Ca_Kabuli_Chr04	13102348	(G/T)
6031	CakSNP6031	*Kabuli*	Ca_Kabuli_Chr04	13147948	(A/G)
6032	CakSNP6032	*Kabuli*	Ca_Kabuli_Chr04	13147951	(C/T)
6033	CakSNP6033	*Kabuli*	Ca_Kabuli_Chr04	13147960	(T/C)
6034	CakSNP6034	*Kabuli*	Ca_Kabuli_Chr04	13147965	(A/G)
6035	CakSNP6035	*Kabuli*	Ca_Kabuli_Chr04	13148016	(G/A)
6036	CakSNP6036	*Kabuli*	Ca_Kabuli_Chr04	13179829	(T/C)
6037	CakSNP6037	*Kabuli*	Ca_Kabuli_Chr04	13203008	(A/T)
6038	CakSNP6038	*Kabuli*	Ca_Kabuli_Chr04	13229035	(C/T)
6039	CakSNP6039	*Kabuli*	Ca_Kabuli_Chr04	13252015	(A/T)
6040	CakSNP6040	*Kabuli*	Ca_Kabuli_Chr04	13252077	(C/G)
6041	CakSNP6041	*Kabuli*	Ca_Kabuli_Chr04	13252126	(T/C)

S.N.	SNP IDs	Cultivars	Chromosomes/scaffolds	Physical positions (bp)	SNPs
6042	CakSNP6042	*Kabuli*	Ca_Kabuli_Chr04	13252212	(G/A)
6043	CakSNP6043	*Kabuli*	Ca_Kabuli_Chr04	13272282	(T/C)
6044	CakSNP6044	*Kabuli*	Ca_Kabuli_Chr04	13282984	(A/C)
6045	CakSNP6045	*Kabuli*	Ca_Kabuli_Chr04	13296221	(A/T)
6046	CakSNP6046	*Kabuli*	Ca_Kabuli_Chr04	13296217	(G/T)
6047	CakSNP6047	*Kabuli*	Ca_Kabuli_Chr04	13310700	(T/C)
6048	CakSNP6048	*Kabuli*	Ca_Kabuli_Chr04	13318811	(C/T)
6049	CakSNP6049	*Kabuli*	Ca_Kabuli_Chr04	13331280	(A/G)
6050	CakSNP6050	*Kabuli*	Ca_Kabuli_Chr04	13332384	(G/A)
6051	CakSNP6051	*Kabuli*	Ca_Kabuli_Chr04	13332452	(T/C)
6052	CakSNP6052	*Kabuli*	Ca_Kabuli_Chr04	13376456	(A/T)
6053	CakSNP6053	*Kabuli*	Ca_Kabuli_Chr04	13463481	(C/T)
6054	CakSNP6054	*Kabuli*	Ca_Kabuli_Chr04	13500679	(A/C)
6055	CakSNP6055	*Kabuli*	Ca_Kabuli_Chr04	13500693	(G/A)
6056	CakSNP6056	*Kabuli*	Ca_Kabuli_Chr04	13588956	(C/T)
6057	CakSNP6057	*Kabuli*	Ca_Kabuli_Chr04	13588928	(T/C)
6058	CakSNP6058	*Kabuli*	Ca_Kabuli_Chr04	13646571	(T/C)
6059	CakSNP6059	*Kabuli*	Ca_Kabuli_Chr04	13646628	(C/T)
6060	CakSNP6060	*Kabuli*	Ca_Kabuli_Chr04	13687456	(G/A)
6061	CakSNP6061	*Kabuli*	Ca_Kabuli_Chr04	13713380	(G/T)
6062	CakSNP6062	*Kabuli*	Ca_Kabuli_Chr04	13713367	(G/C)
6063	CakSNP6063	*Kabuli*	Ca_Kabuli_Chr04	13724666	(G/C)
6064	CakSNP6064	*Kabuli*	Ca_Kabuli_Chr04	13787448	(A/C)
6065	CakSNP6065	*Kabuli*	Ca_Kabuli_Chr04	13787649	(T/C)
6066	CakSNP6066	*Kabuli*	Ca_Kabuli_Chr04	13787720	(G/C)
6067	CakSNP6067	*Kabuli*	Ca_Kabuli_Chr04	13823667	(T/A)
6068	CakSNP6068	*Kabuli*	Ca_Kabuli_Chr04	13823672	(G/T)
6069	CakSNP6069	*Kabuli*	Ca_Kabuli_Chr04	13823695	(T/C)
6070	CakSNP6070	*Kabuli*	Ca_Kabuli_Chr04	13838241	(G/T)
6071	CakSNP6071	*Kabuli*	Ca_Kabuli_Chr04	13838796	(A/G)
6072	CakSNP6072	*Kabuli*	Ca_Kabuli_Chr04	13839294	(A/G)
6073	CakSNP6073	*Kabuli*	Ca_Kabuli_Chr04	13839288	(G/A)
6074	CakSNP6074	*Kabuli*	Ca_Kabuli_Chr04	13840191	(T/A)
6075	CakSNP6075	*Kabuli*	Ca_Kabuli_Chr04	13840227	(C/A)
6076	CakSNP6076	*Kabuli*	Ca_Kabuli_Chr04	13840251	(A/G)
6077	CakSNP6077	*Kabuli*	Ca_Kabuli_Chr04	13840484	(T/C)
6078	CakSNP6078	*Kabuli*	Ca_Kabuli_Chr04	13841340	(G/T)
6079	CakSNP6079	*Kabuli*	Ca_Kabuli_Chr04	13841401	(A/G)

S.N.	SNP IDs	Cultivars	Chromosomes/scaffolds	Physical positions (bp)	SNPs
6080	CakSNP6080	*Kabuli*	Ca_Kabuli_Chr04	13845581	(A/T)
6081	CakSNP6081	*Kabuli*	Ca_Kabuli_Chr04	13845639	(A/C)
6082	CakSNP6082	*Kabuli*	Ca_Kabuli_Chr04	13845675	(T/A)
6083	CakSNP6083	*Kabuli*	Ca_Kabuli_Chr04	13900013	(A/G)
6084	CakSNP6084	*Kabuli*	Ca_Kabuli_Chr04	13923366	(T/C)
6085	CakSNP6085	*Kabuli*	Ca_Kabuli_Chr04	13949513	(A/C)
6086	CakSNP6086	*Kabuli*	Ca_Kabuli_Chr04	13949580	(C/G)
6087	CakSNP6087	*Kabuli*	Ca_Kabuli_Chr04	13954832	(G/A)
6088	CakSNP6088	*Kabuli*	Ca_Kabuli_Chr04	13968291	(C/T)
6089	CakSNP6089	*Kabuli*	Ca_Kabuli_Chr04	13968397	(A/G)
6090	CakSNP6090	*Kabuli*	Ca_Kabuli_Chr04	13968354	(A/G)
6091	CakSNP6091	*Kabuli*	Ca_Kabuli_Chr04	13982970	(G/A)
6092	CakSNP6092	*Kabuli*	Ca_Kabuli_Chr04	14031548	(A/G)
6093	CakSNP6093	*Kabuli*	Ca_Kabuli_Chr04	14056908	(A/G)
6094	CakSNP6094	*Kabuli*	Ca_Kabuli_Chr04	14073059	(C/T)
6095	CakSNP6095	*Kabuli*	Ca_Kabuli_Chr04	14073056	(C/T)
6096	CakSNP6096	*Kabuli*	Ca_Kabuli_Chr04	14073045	(G/A)
6097	CakSNP6097	*Kabuli*	Ca_Kabuli_Chr04	14073044	(C/T)
6098	CakSNP6098	*Kabuli*	Ca_Kabuli_Chr04	14073004	(C/A)
6099	CakSNP6099	*Kabuli*	Ca_Kabuli_Chr04	14082157	(C/A)
6100	CakSNP6100	*Kabuli*	Ca_Kabuli_Chr04	14082158	(T/G)
6101	CakSNP6101	*Kabuli*	Ca_Kabuli_Chr04	14082186	(G/A)
6102	CakSNP6102	*Kabuli*	Ca_Kabuli_Chr04	14082210	(T/C)
6103	CakSNP6103	*Kabuli*	Ca_Kabuli_Chr04	14082285	(C/G)
6104	CakSNP6104	*Kabuli*	Ca_Kabuli_Chr04	14093027	(A/G)
6105	CakSNP6105	*Kabuli*	Ca_Kabuli_Chr04	14104096	(A/C)
6106	CakSNP6106	*Kabuli*	Ca_Kabuli_Chr04	14112911	(C/A)
6107	CakSNP6107	*Kabuli*	Ca_Kabuli_Chr04	14132659	(A/C)
6108	CakSNP6108	*Kabuli*	Ca_Kabuli_Chr04	14149589	(G/A)
6109	CakSNP6109	*Kabuli*	Ca_Kabuli_Chr04	14149588	(A/G)
6110	CakSNP6110	*Kabuli*	Ca_Kabuli_Chr04	14155978	(A/G)
6111	CakSNP6111	*Kabuli*	Ca_Kabuli_Chr04	14156014	(C/T)
6112	CakSNP6112	*Kabuli*	Ca_Kabuli_Chr04	14163060	(C/T)
6113	CakSNP6113	*Kabuli*	Ca_Kabuli_Chr04	14181527	(A/G)
6114	CakSNP6114	*Kabuli*	Ca_Kabuli_Chr04	14189356	(A/C)
6115	CakSNP6115	*Kabuli*	Ca_Kabuli_Chr04	14199191	(G/C)
6116	CakSNP6116	*Kabuli*	Ca_Kabuli_Chr04	14225284	(A/G)
6117	CakSNP6117	*Kabuli*	Ca_Kabuli_Chr04	14270811	(G/A)

S.N.	SNP IDs	Cultivars	Chromosomes/scaffolds	Physical positions (bp)	SNPs
6118	CakSNP6118	*Kabuli*	Ca_Kabuli_Chr04	14270763	(G/A)
6119	CakSNP6119	*Kabuli*	Ca_Kabuli_Chr04	14453942	(T/C)
6120	CakSNP6120	*Kabuli*	Ca_Kabuli_Chr04	14515543	(T/A)
6121	CakSNP6121	*Kabuli*	Ca_Kabuli_Chr04	14515531	(T/A)
6122	CakSNP6122	*Kabuli*	Ca_Kabuli_Chr04	14517424	(C/T)
6123	CakSNP6123	*Kabuli*	Ca_Kabuli_Chr04	14529007	(A/G)
6124	CakSNP6124	*Kabuli*	Ca_Kabuli_Chr04	14565948	(A/C)
6125	CakSNP6125	*Kabuli*	Ca_Kabuli_Chr04	14566064	(G/A)
6126	CakSNP6126	*Kabuli*	Ca_Kabuli_Chr04	14566074	(T/A)
6127	CakSNP6127	*Kabuli*	Ca_Kabuli_Chr04	14566081	(A/G)
6128	CakSNP6128	*Kabuli*	Ca_Kabuli_Chr04	14611177	(A/G)
6129	CakSNP6129	*Kabuli*	Ca_Kabuli_Chr04	14657743	(C/T)
6130	CakSNP6130	*Kabuli*	Ca_Kabuli_Chr04	14696775	(T/C)
6131	CakSNP6131	*Kabuli*	Ca_Kabuli_Chr04	14698825	(A/T)
6132	CakSNP6132	*Kabuli*	Ca_Kabuli_Chr04	14699270	(T/C)
6133	CakSNP6133	*Kabuli*	Ca_Kabuli_Chr04	14748051	(A/C)
6134	CakSNP6134	*Kabuli*	Ca_Kabuli_Chr04	14770484	(C/T)
6135	CakSNP6135	*Kabuli*	Ca_Kabuli_Chr04	14770524	(G/C)
6136	CakSNP6136	*Kabuli*	Ca_Kabuli_Chr04	14770525	(G/A)
6137	CakSNP6137	*Kabuli*	Ca_Kabuli_Chr04	14770532	(C/T)
6138	CakSNP6138	*Kabuli*	Ca_Kabuli_Chr04	14785530	(G/A)
6139	CakSNP6139	*Kabuli*	Ca_Kabuli_Chr04	14785557	(A/C)
6140	CakSNP6140	*Kabuli*	Ca_Kabuli_Chr04	14786093	(C/G)
6141	CakSNP6141	*Kabuli*	Ca_Kabuli_Chr04	14831204	(A/T)
6142	CakSNP6142	*Kabuli*	Ca_Kabuli_Chr04	14831298	(A/T)
6143	CakSNP6143	*Kabuli*	Ca_Kabuli_Chr04	14855406	(T/C)
6144	CakSNP6144	*Kabuli*	Ca_Kabuli_Chr04	14855417	(T/A)
6145	CakSNP6145	*Kabuli*	Ca_Kabuli_Chr04	14870658	(T/G)
6146	CakSNP6146	*Kabuli*	Ca_Kabuli_Chr04	14870641	(G/A)
6147	CakSNP6147	*Kabuli*	Ca_Kabuli_Chr04	14989557	(C/T)
6148	CakSNP6148	*Kabuli*	Ca_Kabuli_Chr04	14989544	(C/T)
6149	CakSNP6149	*Kabuli*	Ca_Kabuli_Chr04	14989526	(G/A)
6150	CakSNP6150	*Kabuli*	Ca_Kabuli_Chr04	15025765	(T/A)
6151	CakSNP6151	*Kabuli*	Ca_Kabuli_Chr04	15025785	(T/G)
6152	CakSNP6152	*Kabuli*	Ca_Kabuli_Chr04	15026058	(C/T)
6153	CakSNP6153	*Kabuli*	Ca_Kabuli_Chr04	15036362	(A/G)
6154	CakSNP6154	*Kabuli*	Ca_Kabuli_Chr04	15039897	(C/G)
6155	CakSNP6155	*Kabuli*	Ca_Kabuli_Chr04	15039905	(T/C)

S.N.	SNP IDs	Cultivars	Chromosomes/scaffolds	Physical positions (bp)	SNPs
6156	CakSNP6156	*Kabuli*	Ca_Kabuli_Chr04	15039948	(G/C)
6157	CakSNP6157	*Kabuli*	Ca_Kabuli_Chr04	15039996	(C/T)
6158	CakSNP6158	*Kabuli*	Ca_Kabuli_Chr04	15040072	(A/C)
6159	CakSNP6159	*Kabuli*	Ca_Kabuli_Chr04	15053643	(T/C)
6160	CakSNP6160	*Kabuli*	Ca_Kabuli_Chr04	15075489	(T/G)
6161	CakSNP6161	*Kabuli*	Ca_Kabuli_Chr04	15075502	(G/T)
6162	CakSNP6162	*Kabuli*	Ca_Kabuli_Chr04	15159143	(A/G)
6163	CakSNP6163	*Kabuli*	Ca_Kabuli_Chr04	15190387	(T/G)
6164	CakSNP6164	*Kabuli*	Ca_Kabuli_Chr04	15190423	(T/A)
6165	CakSNP6165	*Kabuli*	Ca_Kabuli_Chr04	15212787	(G/A)
6166	CakSNP6166	*Kabuli*	Ca_Kabuli_Chr04	15240675	(G/A)
6167	CakSNP6167	*Kabuli*	Ca_Kabuli_Chr04	15240678	(T/C)
6168	CakSNP6168	*Kabuli*	Ca_Kabuli_Chr04	15299232	(G/A)
6169	CakSNP6169	*Kabuli*	Ca_Kabuli_Chr04	15320777	(G/A)
6170	CakSNP6170	*Kabuli*	Ca_Kabuli_Chr04	15357651	(C/T)
6171	CakSNP6171	*Kabuli*	Ca_Kabuli_Chr04	15357695	(A/G)
6172	CakSNP6172	*Kabuli*	Ca_Kabuli_Chr04	15394470	(T/C)
6173	CakSNP6173	*Kabuli*	Ca_Kabuli_Chr04	15448826	(G/T)
6174	CakSNP6174	*Kabuli*	Ca_Kabuli_Chr04	15449843	(A/G)
6175	CakSNP6175	*Kabuli*	Ca_Kabuli_Chr04	15542543	(T/C)
6176	CakSNP6176	*Kabuli*	Ca_Kabuli_Chr04	15542597	(A/G)
6177	CakSNP6177	*Kabuli*	Ca_Kabuli_Chr04	15552690	(C/T)
6178	CakSNP6178	*Kabuli*	Ca_Kabuli_Chr04	15552683	(C/T)
6179	CakSNP6179	*Kabuli*	Ca_Kabuli_Chr04	15697128	(A/G)
6180	CakSNP6180	*Kabuli*	Ca_Kabuli_Chr04	15697303	(G/T)
6181	CakSNP6181	*Kabuli*	Ca_Kabuli_Chr04	15698212	(G/T)
6182	CakSNP6182	*Kabuli*	Ca_Kabuli_Chr04	15698179	(G/A)
6183	CakSNP6183	*Kabuli*	Ca_Kabuli_Chr04	15698233	(A/C)
6184	CakSNP6184	*Kabuli*	Ca_Kabuli_Chr04	15698305	(T/C)
6185	CakSNP6185	*Kabuli*	Ca_Kabuli_Chr04	15698405	(G/A)
6186	CakSNP6186	*Kabuli*	Ca_Kabuli_Chr04	15698486	(T/C)
6187	CakSNP6187	*Kabuli*	Ca_Kabuli_Chr04	15698456	(T/C)
6188	CakSNP6188	*Kabuli*	Ca_Kabuli_Chr04	15698444	(C/T)
6189	CakSNP6189	*Kabuli*	Ca_Kabuli_Chr04	15698420	(G/C)
6190	CakSNP6190	*Kabuli*	Ca_Kabuli_Chr04	15700603	(A/T)
6191	CakSNP6191	*Kabuli*	Ca_Kabuli_Chr04	15700630	(G/A)
6192	CakSNP6192	*Kabuli*	Ca_Kabuli_Chr04	15713955	(G/A)
6193	CakSNP6193	*Kabuli*	Ca_Kabuli_Chr04	15717281	(T/C)

S.N.	SNP IDs	Cultivars	Chromosomes/scaffolds	Physical positions (bp)	SNPs
6194	CakSNP6194	*Kabuli*	Ca_Kabuli_Chr04	15735993	(T/C)
6195	CakSNP6195	*Kabuli*	Ca_Kabuli_Chr04	15736015	(C/T)
6196	CakSNP6196	*Kabuli*	Ca_Kabuli_Chr04	15736562	(A/T)
6197	CakSNP6197	*Kabuli*	Ca_Kabuli_Chr04	15736619	(T/G)
6198	CakSNP6198	*Kabuli*	Ca_Kabuli_Chr04	15736627	(T/C)
6199	CakSNP6199	*Kabuli*	Ca_Kabuli_Chr04	15744703	(T/G)
6200	CakSNP6200	*Kabuli*	Ca_Kabuli_Chr04	15752567	(G/C)
6201	CakSNP6201	*Kabuli*	Ca_Kabuli_Chr04	15766341	(A/G)
6202	CakSNP6202	*Kabuli*	Ca_Kabuli_Chr04	15766360	(G/A)
6203	CakSNP6203	*Kabuli*	Ca_Kabuli_Chr04	15772235	(C/A)
6204	CakSNP6204	*Kabuli*	Ca_Kabuli_Chr04	15772275	(A/G)
6205	CakSNP6205	*Kabuli*	Ca_Kabuli_Chr04	15821282	(G/A)
6206	CakSNP6206	*Kabuli*	Ca_Kabuli_Chr04	15821311	(T/A)
6207	CakSNP6207	*Kabuli*	Ca_Kabuli_Chr04	15825621	(A/T)
6208	CakSNP6208	*Kabuli*	Ca_Kabuli_Chr04	15860388	(A/G)
6209	CakSNP6209	*Kabuli*	Ca_Kabuli_Chr04	15860359	(C/A)
6210	CakSNP6210	*Kabuli*	Ca_Kabuli_Chr04	15900602	(G/A)
6211	CakSNP6211	*Kabuli*	Ca_Kabuli_Chr04	15925936	(T/G)
6212	CakSNP6212	*Kabuli*	Ca_Kabuli_Chr04	15926160	(A/G)
6213	CakSNP6213	*Kabuli*	Ca_Kabuli_Chr04	15934607	(A/T)
6214	CakSNP6214	*Kabuli*	Ca_Kabuli_Chr04	15934901	(T/G)
6215	CakSNP6215	*Kabuli*	Ca_Kabuli_Chr04	15957070	(T/A)
6216	CakSNP6216	*Kabuli*	Ca_Kabuli_Chr04	16027272	(G/A)
6217	CakSNP6217	*Kabuli*	Ca_Kabuli_Chr04	16027292	(A/C)
6218	CakSNP6218	*Kabuli*	Ca_Kabuli_Chr04	16027368	(G/T)
6219	CakSNP6219	*Kabuli*	Ca_Kabuli_Chr04	16027299	(T/A)
6220	CakSNP6220	*Kabuli*	Ca_Kabuli_Chr04	16045694	(T/G)
6221	CakSNP6221	*Kabuli*	Ca_Kabuli_Chr04	16045870	(G/A)
6222	CakSNP6222	*Kabuli*	Ca_Kabuli_Chr04	16046928	(T/C)
6223	CakSNP6223	*Kabuli*	Ca_Kabuli_Chr04	16278600	(C/T)
6224	CakSNP6224	*Kabuli*	Ca_Kabuli_Chr04	16278671	(T/A)
6225	CakSNP6225	*Kabuli*	Ca_Kabuli_Chr04	16446305	(T/C)
6226	CakSNP6226	*Kabuli*	Ca_Kabuli_Chr04	16446341	(T/G)
6227	CakSNP6227	*Kabuli*	Ca_Kabuli_Chr04	16446336	(C/G)
6228	CakSNP6228	*Kabuli*	Ca_Kabuli_Chr04	16446956	(T/C)
6229	CakSNP6229	*Kabuli*	Ca_Kabuli_Chr04	16457940	(G/A)
6230	CakSNP6230	*Kabuli*	Ca_Kabuli_Chr04	16501291	(T/A)
6231	CakSNP6231	*Kabuli*	Ca_Kabuli_Chr04	16501306	(T/A)

S.N.	SNP IDs	Cultivars	Chromosomes/scaffolds	Physical positions (bp)	SNPs
6232	CakSNP6232	*Kabuli*	Ca_Kabuli_Chr04	16501354	(C/T)
6233	CakSNP6233	*Kabuli*	Ca_Kabuli_Chr04	16501782	(G/A)
6234	CakSNP6234	*Kabuli*	Ca_Kabuli_Chr04	16501781	(C/A)
6235	CakSNP6235	*Kabuli*	Ca_Kabuli_Chr04	16501769	(C/T)
6236	CakSNP6236	*Kabuli*	Ca_Kabuli_Chr04	16501738	(G/A)
6237	CakSNP6237	*Kabuli*	Ca_Kabuli_Chr04	16501716	(T/C)
6238	CakSNP6238	*Kabuli*	Ca_Kabuli_Chr04	16505159	(A/G)
6239	CakSNP6239	*Kabuli*	Ca_Kabuli_Chr04	16536754	(C/T)
6240	CakSNP6240	*Kabuli*	Ca_Kabuli_Chr04	16571288	(T/C)
6241	CakSNP6241	*Kabuli*	Ca_Kabuli_Chr04	16586863	(G/C)
6242	CakSNP6242	*Kabuli*	Ca_Kabuli_Chr04	16586913	(G/C)
6243	CakSNP6243	*Kabuli*	Ca_Kabuli_Chr04	16586929	(G/C)
6244	CakSNP6244	*Kabuli*	Ca_Kabuli_Chr04	16619312	(T/C)
6245	CakSNP6245	*Kabuli*	Ca_Kabuli_Chr04	16622514	(C/A)
6246	CakSNP6246	*Kabuli*	Ca_Kabuli_Chr04	16625148	(T/G)
6247	CakSNP6247	*Kabuli*	Ca_Kabuli_Chr04	16625270	(T/G)
6248	CakSNP6248	*Kabuli*	Ca_Kabuli_Chr04	16625221	(G/A)
6249	CakSNP6249	*Kabuli*	Ca_Kabuli_Chr04	16628559	(T/G)
6250	CakSNP6250	*Kabuli*	Ca_Kabuli_Chr04	16628591	(T/G)
6251	CakSNP6251	*Kabuli*	Ca_Kabuli_Chr04	16628594	(T/G)
6252	CakSNP6252	*Kabuli*	Ca_Kabuli_Chr04	16628542	(G/A)
6253	CakSNP6253	*Kabuli*	Ca_Kabuli_Chr04	16628540	(G/A)
6254	CakSNP6254	*Kabuli*	Ca_Kabuli_Chr04	16628536	(T/A)
6255	CakSNP6255	*Kabuli*	Ca_Kabuli_Chr04	16628529	(C/A)
6256	CakSNP6256	*Kabuli*	Ca_Kabuli_Chr04	16628527	(G/A)
6257	CakSNP6257	*Kabuli*	Ca_Kabuli_Chr04	16628523	(T/C)
6258	CakSNP6258	*Kabuli*	Ca_Kabuli_Chr04	16629560	(G/T)
6259	CakSNP6259	*Kabuli*	Ca_Kabuli_Chr04	16629559	(T/G)
6260	CakSNP6260	*Kabuli*	Ca_Kabuli_Chr04	16629638	(C/A)
6261	CakSNP6261	*Kabuli*	Ca_Kabuli_Chr04	16630385	(G/T)
6262	CakSNP6262	*Kabuli*	Ca_Kabuli_Chr04	16669916	(T/C)
6263	CakSNP6263	*Kabuli*	Ca_Kabuli_Chr04	16749740	(A/G)
6264	CakSNP6264	*Kabuli*	Ca_Kabuli_Chr04	16750416	(A/G)
6265	CakSNP6265	*Kabuli*	Ca_Kabuli_Chr04	16751205	(T/C)
6266	CakSNP6266	*Kabuli*	Ca_Kabuli_Chr04	16789476	(G/A)
6267	CakSNP6267	*Kabuli*	Ca_Kabuli_Chr04	16789532	(A/C)
6268	CakSNP6268	*Kabuli*	Ca_Kabuli_Chr04	16820726	(C/A)
6269	CakSNP6269	*Kabuli*	Ca_Kabuli_Chr04	16837336	(T/A)

S.N.	SNP IDs	Cultivars	Chromosomes/scaffolds	Physical positions (bp)	SNPs
6270	CakSNP6270	*Kabuli*	Ca_Kabuli_Chr04	16839081	(T/A)
6271	CakSNP6271	*Kabuli*	Ca_Kabuli_Chr04	16845299	(G/A)
6272	CakSNP6272	*Kabuli*	Ca_Kabuli_Chr04	16889650	(A/T)
6273	CakSNP6273	*Kabuli*	Ca_Kabuli_Chr04	16926545	(T/C)
6274	CakSNP6274	*Kabuli*	Ca_Kabuli_Chr04	16926527	(T/A)
6275	CakSNP6275	*Kabuli*	Ca_Kabuli_Chr04	16926614	(A/T)
6276	CakSNP6276	*Kabuli*	Ca_Kabuli_Chr04	17028303	(A/G)
6277	CakSNP6277	*Kabuli*	Ca_Kabuli_Chr04	17028368	(G/A)
6278	CakSNP6278	*Kabuli*	Ca_Kabuli_Chr04	17028523	(C/T)
6279	CakSNP6279	*Kabuli*	Ca_Kabuli_Chr04	17028495	(T/A)
6280	CakSNP6280	*Kabuli*	Ca_Kabuli_Chr04	17032676	(T/C)
6281	CakSNP6281	*Kabuli*	Ca_Kabuli_Chr04	17032673	(C/T)
6282	CakSNP6282	*Kabuli*	Ca_Kabuli_Chr04	17059830	(G/A)
6283	CakSNP6283	*Kabuli*	Ca_Kabuli_Chr04	17059833	(A/T)
6284	CakSNP6284	*Kabuli*	Ca_Kabuli_Chr04	17059852	(T/C)
6285	CakSNP6285	*Kabuli*	Ca_Kabuli_Chr04	17059866	(G/A)
6286	CakSNP6286	*Kabuli*	Ca_Kabuli_Chr04	17059872	(A/G)
6287	CakSNP6287	*Kabuli*	Ca_Kabuli_Chr04	17059887	(G/A)
6288	CakSNP6288	*Kabuli*	Ca_Kabuli_Chr04	17093090	(C/G)
6289	CakSNP6289	*Kabuli*	Ca_Kabuli_Chr04	17147875	(C/T)
6290	CakSNP6290	*Kabuli*	Ca_Kabuli_Chr04	17147926	(T/C)
6291	CakSNP6291	*Kabuli*	Ca_Kabuli_Chr04	17187560	(C/G)
6292	CakSNP6292	*Kabuli*	Ca_Kabuli_Chr04	17191562	(T/A)
6293	CakSNP6293	*Kabuli*	Ca_Kabuli_Chr04	17199316	(T/C)
6294	CakSNP6294	*Kabuli*	Ca_Kabuli_Chr04	17199477	(G/A)
6295	CakSNP6295	*Kabuli*	Ca_Kabuli_Chr04	17199432	(G/A)
6296	CakSNP6296	*Kabuli*	Ca_Kabuli_Chr04	17202770	(A/G)
6297	CakSNP6297	*Kabuli*	Ca_Kabuli_Chr04	17225334	(G/A)
6298	CakSNP6298	*Kabuli*	Ca_Kabuli_Chr04	17264248	(C/A)
6299	CakSNP6299	*Kabuli*	Ca_Kabuli_Chr04	17264592	(G/A)
6300	CakSNP6300	*Kabuli*	Ca_Kabuli_Chr04	17264621	(C/G)
6301	CakSNP6301	*Kabuli*	Ca_Kabuli_Chr04	17264637	(C/T)
6302	CakSNP6302	*Kabuli*	Ca_Kabuli_Chr04	17282119	(T/C)
6303	CakSNP6303	*Kabuli*	Ca_Kabuli_Chr04	17282139	(G/C)
6304	CakSNP6304	*Kabuli*	Ca_Kabuli_Chr04	17282148	(G/C)
6305	CakSNP6305	*Kabuli*	Ca_Kabuli_Chr04	17282336	(G/T)
6306	CakSNP6306	*Kabuli*	Ca_Kabuli_Chr04	17282345	(G/C)
6307	CakSNP6307	*Kabuli*	Ca_Kabuli_Chr04	17289594	(G/C)

S.N.	SNP IDs	Cultivars	Chromosomes/scaffolds	Physical positions (bp)	SNPs
6308	CakSNP6308	*Kabuli*	Ca_Kabuli_Chr04	17297498	(G/T)
6309	CakSNP6309	*Kabuli*	Ca_Kabuli_Chr04	17297567	(G/A)
6310	CakSNP6310	*Kabuli*	Ca_Kabuli_Chr04	17297563	(A/G)
6311	CakSNP6311	*Kabuli*	Ca_Kabuli_Chr04	17309656	(A/C)
6312	CakSNP6312	*Kabuli*	Ca_Kabuli_Chr04	17345431	(T/C)
6313	CakSNP6313	*Kabuli*	Ca_Kabuli_Chr04	17345436	(G/T)
6314	CakSNP6314	*Kabuli*	Ca_Kabuli_Chr04	17345439	(G/A)
6315	CakSNP6315	*Kabuli*	Ca_Kabuli_Chr04	17345448	(T/G)
6316	CakSNP6316	*Kabuli*	Ca_Kabuli_Chr04	17345500	(G/A)
6317	CakSNP6317	*Kabuli*	Ca_Kabuli_Chr04	17345483	(C/A)
6318	CakSNP6318	*Kabuli*	Ca_Kabuli_Chr04	17345454	(A/G)
6319	CakSNP6319	*Kabuli*	Ca_Kabuli_Chr04	17358736	(C/T)
6320	CakSNP6320	*Kabuli*	Ca_Kabuli_Chr04	17361143	(T/A)
6321	CakSNP6321	*Kabuli*	Ca_Kabuli_Chr04	17396325	(A/G)
6322	CakSNP6322	*Kabuli*	Ca_Kabuli_Chr04	17396349	(T/G)
6323	CakSNP6323	*Kabuli*	Ca_Kabuli_Chr04	17396352	(T/G)
6324	CakSNP6324	*Kabuli*	Ca_Kabuli_Chr04	17429456	(A/T)
6325	CakSNP6325	*Kabuli*	Ca_Kabuli_Chr04	17429457	(A/T)
6326	CakSNP6326	*Kabuli*	Ca_Kabuli_Chr04	17429468	(T/C)
6327	CakSNP6327	*Kabuli*	Ca_Kabuli_Chr04	17429747	(G/A)
6328	CakSNP6328	*Kabuli*	Ca_Kabuli_Chr04	17429742	(A/G)
6329	CakSNP6329	*Kabuli*	Ca_Kabuli_Chr04	17429735	(A/G)
6330	CakSNP6330	*Kabuli*	Ca_Kabuli_Chr04	17429699	(G/C)
6331	CakSNP6331	*Kabuli*	Ca_Kabuli_Chr04	17433782	(G/A)
6332	CakSNP6332	*Kabuli*	Ca_Kabuli_Chr04	17433914	(G/A)
6333	CakSNP6333	*Kabuli*	Ca_Kabuli_Chr04	17444578	(A/C)
6334	CakSNP6334	*Kabuli*	Ca_Kabuli_Chr04	17472129	(T/A)
6335	CakSNP6335	*Kabuli*	Ca_Kabuli_Chr04	17472992	(T/G)
6336	CakSNP6336	*Kabuli*	Ca_Kabuli_Chr04	17473174	(C/T)
6337	CakSNP6337	*Kabuli*	Ca_Kabuli_Chr04	17473237	(T/C)
6338	CakSNP6338	*Kabuli*	Ca_Kabuli_Chr04	17473234	(C/A)
6339	CakSNP6339	*Kabuli*	Ca_Kabuli_Chr04	17603613	(T/C)
6340	CakSNP6340	*Kabuli*	Ca_Kabuli_Chr04	17603677	(G/C)
6341	CakSNP6341	*Kabuli*	Ca_Kabuli_Chr04	17603689	(T/C)
6342	CakSNP6342	*Kabuli*	Ca_Kabuli_Chr04	17603725	(T/C)
6343	CakSNP6343	*Kabuli*	Ca_Kabuli_Chr04	17609554	(A/T)
6344	CakSNP6344	*Kabuli*	Ca_Kabuli_Chr04	17621035	(G/T)
6345	CakSNP6345	*Kabuli*	Ca_Kabuli_Chr04	17621609	(G/A)

S.N.	SNP IDs	Cultivars	Chromosomes/scaffolds	Physical positions (bp)	SNPs
6346	CakSNP6346	*Kabuli*	Ca_Kabuli_Chr04	17647558	(G/A)
6347	CakSNP6347	*Kabuli*	Ca_Kabuli_Chr04	17652735	(T/C)
6348	CakSNP6348	*Kabuli*	Ca_Kabuli_Chr04	17685058	(T/G)
6349	CakSNP6349	*Kabuli*	Ca_Kabuli_Chr04	17685113	(C/A)
6350	CakSNP6350	*Kabuli*	Ca_Kabuli_Chr04	17718540	(C/T)
6351	CakSNP6351	*Kabuli*	Ca_Kabuli_Chr04	17718536	(C/A)
6352	CakSNP6352	*Kabuli*	Ca_Kabuli_Chr04	17718894	(G/A)
6353	CakSNP6353	*Kabuli*	Ca_Kabuli_Chr04	17718922	(T/G)
6354	CakSNP6354	*Kabuli*	Ca_Kabuli_Chr04	17718997	(C/A)
6355	CakSNP6355	*Kabuli*	Ca_Kabuli_Chr04	17729988	(A/C)
6356	CakSNP6356	*Kabuli*	Ca_Kabuli_Chr04	17748171	(A/T)
6357	CakSNP6357	*Kabuli*	Ca_Kabuli_Chr04	17752013	(C/T)
6358	CakSNP6358	*Kabuli*	Ca_Kabuli_Chr04	17760841	(A/G)
6359	CakSNP6359	*Kabuli*	Ca_Kabuli_Chr04	17760853	(T/G)
6360	CakSNP6360	*Kabuli*	Ca_Kabuli_Chr04	17768507	(T/C)
6361	CakSNP6361	*Kabuli*	Ca_Kabuli_Chr04	17771708	(C/G)
6362	CakSNP6362	*Kabuli*	Ca_Kabuli_Chr04	17784792	(T/A)
6363	CakSNP6363	*Kabuli*	Ca_Kabuli_Chr04	17826472	(G/A)
6364	CakSNP6364	*Kabuli*	Ca_Kabuli_Chr04	17826533	(A/C)
6365	CakSNP6365	*Kabuli*	Ca_Kabuli_Chr04	17826601	(T/C)
6366	CakSNP6366	*Kabuli*	Ca_Kabuli_Chr04	17826580	(C/G)
6367	CakSNP6367	*Kabuli*	Ca_Kabuli_Chr04	17826543	(T/C)
6368	CakSNP6368	*Kabuli*	Ca_Kabuli_Chr04	17843480	(G/T)
6369	CakSNP6369	*Kabuli*	Ca_Kabuli_Chr04	17847737	(G/A)
6370	CakSNP6370	*Kabuli*	Ca_Kabuli_Chr04	17847854	(T/C)
6371	CakSNP6371	*Kabuli*	Ca_Kabuli_Chr04	17856307	(T/C)
6372	CakSNP6372	*Kabuli*	Ca_Kabuli_Chr04	17856311	(T/G)
6373	CakSNP6373	*Kabuli*	Ca_Kabuli_Chr04	17856334	(A/G)
6374	CakSNP6374	*Kabuli*	Ca_Kabuli_Chr04	17857753	(C/T)
6375	CakSNP6375	*Kabuli*	Ca_Kabuli_Chr04	17858122	(T/G)
6376	CakSNP6376	*Kabuli*	Ca_Kabuli_Chr04	17858133	(C/T)
6377	CakSNP6377	*Kabuli*	Ca_Kabuli_Chr04	17858135	(G/A)
6378	CakSNP6378	*Kabuli*	Ca_Kabuli_Chr04	17858140	(A/C)
6379	CakSNP6379	*Kabuli*	Ca_Kabuli_Chr04	17858143	(A/T)
6380	CakSNP6380	*Kabuli*	Ca_Kabuli_Chr04	17858144	(G/C)
6381	CakSNP6381	*Kabuli*	Ca_Kabuli_Chr04	17858152	(A/T)
6382	CakSNP6382	*Kabuli*	Ca_Kabuli_Chr04	17858159	(C/A)
6383	CakSNP6383	*Kabuli*	Ca_Kabuli_Chr04	17858226	(C/T)

S.N.	SNP IDs	Cultivars	Chromosomes/scaffolds	Physical positions (bp)	SNPs
6384	CakSNP6384	*Kabuli*	Ca_Kabuli_Chr04	17871769	(C/A)
6385	CakSNP6385	*Kabuli*	Ca_Kabuli_Chr04	17871763	(T/C)
6386	CakSNP6386	*Kabuli*	Ca_Kabuli_Chr04	17871804	(T/C)
6387	CakSNP6387	*Kabuli*	Ca_Kabuli_Chr04	17871828	(A/C)
6388	CakSNP6388	*Kabuli*	Ca_Kabuli_Chr04	17871919	(A/G)
6389	CakSNP6389	*Kabuli*	Ca_Kabuli_Chr04	17871915	(T/A)
6390	CakSNP6390	*Kabuli*	Ca_Kabuli_Chr04	17913731	(A/C)
6391	CakSNP6391	*Kabuli*	Ca_Kabuli_Chr04	17913726	(G/A)
6392	CakSNP6392	*Kabuli*	Ca_Kabuli_Chr04	17913719	(G/C)
6393	CakSNP6393	*Kabuli*	Ca_Kabuli_Chr04	17913698	(C/T)
6394	CakSNP6394	*Kabuli*	Ca_Kabuli_Chr04	17913682	(C/A)
6395	CakSNP6395	*Kabuli*	Ca_Kabuli_Chr04	17920616	(C/G)
6396	CakSNP6396	*Kabuli*	Ca_Kabuli_Chr04	17931955	(G/A)
6397	CakSNP6397	*Kabuli*	Ca_Kabuli_Chr04	17970218	(T/G)
6398	CakSNP6398	*Kabuli*	Ca_Kabuli_Chr04	17970221	(C/T)
6399	CakSNP6399	*Kabuli*	Ca_Kabuli_Chr04	17970225	(T/C)
6400	CakSNP6400	*Kabuli*	Ca_Kabuli_Chr04	17970232	(A/G)
6401	CakSNP6401	*Kabuli*	Ca_Kabuli_Chr04	17970280	(T/A)
6402	CakSNP6402	*Kabuli*	Ca_Kabuli_Chr04	18053756	(A/G)
6403	CakSNP6403	*Kabuli*	Ca_Kabuli_Chr04	18070293	(G/T)
6404	CakSNP6404	*Kabuli*	Ca_Kabuli_Chr04	18071114	(C/A)
6405	CakSNP6405	*Kabuli*	Ca_Kabuli_Chr04	18071324	(G/A)
6406	CakSNP6406	*Kabuli*	Ca_Kabuli_Chr04	18089423	(G/C)
6407	CakSNP6407	*Kabuli*	Ca_Kabuli_Chr04	18089422	(T/C)
6408	CakSNP6408	*Kabuli*	Ca_Kabuli_Chr04	18117833	(T/C)
6409	CakSNP6409	*Kabuli*	Ca_Kabuli_Chr04	18222762	(T/C)
6410	CakSNP6410	*Kabuli*	Ca_Kabuli_Chr04	18231443	(T/C)
6411	CakSNP6411	*Kabuli*	Ca_Kabuli_Chr04	18231463	(T/C)
6412	CakSNP6412	*Kabuli*	Ca_Kabuli_Chr04	18231466	(T/C)
6413	CakSNP6413	*Kabuli*	Ca_Kabuli_Chr04	18242020	(C/G)
6414	CakSNP6414	*Kabuli*	Ca_Kabuli_Chr04	18241995	(C/G)
6415	CakSNP6415	*Kabuli*	Ca_Kabuli_Chr04	18427385	(T/G)
6416	CakSNP6416	*Kabuli*	Ca_Kabuli_Chr04	18427384	(C/T)
6417	CakSNP6417	*Kabuli*	Ca_Kabuli_Chr04	18429046	(G/T)
6418	CakSNP6418	*Kabuli*	Ca_Kabuli_Chr04	18429139	(C/T)
6419	CakSNP6419	*Kabuli*	Ca_Kabuli_Chr04	18485782	(T/C)
6420	CakSNP6420	*Kabuli*	Ca_Kabuli_Chr04	18489456	(G/C)
6421	CakSNP6421	*Kabuli*	Ca_Kabuli_Chr04	18495186	(A/G)

S.N.	SNP IDs	Cultivars	Chromosomes/scaffolds	Physical positions (bp)	SNPs
6422	CakSNP6422	*Kabuli*	Ca_Kabuli_Chr04	18545489	(A/T)
6423	CakSNP6423	*Kabuli*	Ca_Kabuli_Chr04	18546209	(T/G)
6424	CakSNP6424	*Kabuli*	Ca_Kabuli_Chr04	18625387	(G/A)
6425	CakSNP6425	*Kabuli*	Ca_Kabuli_Chr04	18628582	(A/G)
6426	CakSNP6426	*Kabuli*	Ca_Kabuli_Chr04	18710941	(T/G)
6427	CakSNP6427	*Kabuli*	Ca_Kabuli_Chr04	18719417	(A/G)
6428	CakSNP6428	*Kabuli*	Ca_Kabuli_Chr04	18721801	(G/A)
6429	CakSNP6429	*Kabuli*	Ca_Kabuli_Chr04	18779396	(A/C)
6430	CakSNP6430	*Kabuli*	Ca_Kabuli_Chr04	18812610	(T/C)
6431	CakSNP6431	*Kabuli*	Ca_Kabuli_Chr04	18880757	(A/G)
6432	CakSNP6432	*Kabuli*	Ca_Kabuli_Chr04	18904060	(G/A)
6433	CakSNP6433	*Kabuli*	Ca_Kabuli_Chr04	18912038	(G/C)
6434	CakSNP6434	*Kabuli*	Ca_Kabuli_Chr04	18912137	(T/C)
6435	CakSNP6435	*Kabuli*	Ca_Kabuli_Chr04	18912124	(C/A)
6436	CakSNP6436	*Kabuli*	Ca_Kabuli_Chr04	18912142	(A/G)
6437	CakSNP6437	*Kabuli*	Ca_Kabuli_Chr04	18923641	(G/T)
6438	CakSNP6438	*Kabuli*	Ca_Kabuli_Chr04	18953354	(C/A)
6439	CakSNP6439	*Kabuli*	Ca_Kabuli_Chr04	18953423	(A/C)
6440	CakSNP6440	*Kabuli*	Ca_Kabuli_Chr04	18953511	(C/G)
6441	CakSNP6441	*Kabuli*	Ca_Kabuli_Chr04	18998727	(C/T)
6442	CakSNP6442	*Kabuli*	Ca_Kabuli_Chr04	19005285	(T/G)
6443	CakSNP6443	*Kabuli*	Ca_Kabuli_Chr04	19005416	(T/G)
6444	CakSNP6444	*Kabuli*	Ca_Kabuli_Chr04	19006493	(C/T)
6445	CakSNP6445	*Kabuli*	Ca_Kabuli_Chr04	19024709	(C/T)
6446	CakSNP6446	*Kabuli*	Ca_Kabuli_Chr04	19024699	(T/C)
6447	CakSNP6447	*Kabuli*	Ca_Kabuli_Chr04	19206563	(A/G)
6448	CakSNP6448	*Kabuli*	Ca_Kabuli_Chr04	19206581	(C/T)
6449	CakSNP6449	*Kabuli*	Ca_Kabuli_Chr04	19206611	(T/C)
6450	CakSNP6450	*Kabuli*	Ca_Kabuli_Chr04	19206613	(T/G)
6451	CakSNP6451	*Kabuli*	Ca_Kabuli_Chr04	19206678	(A/T)
6452	CakSNP6452	*Kabuli*	Ca_Kabuli_Chr04	19207121	(A/T)
6453	CakSNP6453	*Kabuli*	Ca_Kabuli_Chr04	19227691	(G/C)
6454	CakSNP6454	*Kabuli*	Ca_Kabuli_Chr04	19263964	(G/C)
6455	CakSNP6455	*Kabuli*	Ca_Kabuli_Chr04	19263967	(T/G)
6456	CakSNP6456	*Kabuli*	Ca_Kabuli_Chr04	19267581	(A/G)
6457	CakSNP6457	*Kabuli*	Ca_Kabuli_Chr04	19481258	(A/G)
6458	CakSNP6458	*Kabuli*	Ca_Kabuli_Chr04	19540632	(A/C)
6459	CakSNP6459	*Kabuli*	Ca_Kabuli_Chr04	19540646	(T/G)

S.N.	SNP IDs	Cultivars	Chromosomes/scaffolds	Physical positions (bp)	SNPs
6460	CakSNP6460	*Kabuli*	Ca_Kabuli_Chr04	19579390	(G/T)
6461	CakSNP6461	*Kabuli*	Ca_Kabuli_Chr04	19579401	(C/A)
6462	CakSNP6462	*Kabuli*	Ca_Kabuli_Chr04	19579423	(A/G)
6463	CakSNP6463	*Kabuli*	Ca_Kabuli_Chr04	19579433	(G/A)
6464	CakSNP6464	*Kabuli*	Ca_Kabuli_Chr04	19579435	(A/G)
6465	CakSNP6465	*Kabuli*	Ca_Kabuli_Chr04	19579436	(A/T)
6466	CakSNP6466	*Kabuli*	Ca_Kabuli_Chr04	19579442	(C/A)
6467	CakSNP6467	*Kabuli*	Ca_Kabuli_Chr04	19649499	(A/G)
6468	CakSNP6468	*Kabuli*	Ca_Kabuli_Chr04	19668499	(T/C)
6469	CakSNP6469	*Kabuli*	Ca_Kabuli_Chr04	19668502	(T/G)
6470	CakSNP6470	*Kabuli*	Ca_Kabuli_Chr04	19668595	(T/C)
6471	CakSNP6471	*Kabuli*	Ca_Kabuli_Chr04	19688016	(A/G)
6472	CakSNP6472	*Kabuli*	Ca_Kabuli_Chr04	19712406	(C/T)
6473	CakSNP6473	*Kabuli*	Ca_Kabuli_Chr04	19713311	(T/G)
6474	CakSNP6474	*Kabuli*	Ca_Kabuli_Chr04	19734940	(G/A)
6475	CakSNP6475	*Kabuli*	Ca_Kabuli_Chr04	19844376	(A/G)
6476	CakSNP6476	*Kabuli*	Ca_Kabuli_Chr04	19869729	(G/T)
6477	CakSNP6477	*Kabuli*	Ca_Kabuli_Chr04	19869722	(T/G)
6478	CakSNP6478	*Kabuli*	Ca_Kabuli_Chr04	20218430	(T/G)
6479	CakSNP6479	*Kabuli*	Ca_Kabuli_Chr04	20308992	(C/A)
6480	CakSNP6480	*Kabuli*	Ca_Kabuli_Chr04	20309151	(C/T)
6481	CakSNP6481	*Kabuli*	Ca_Kabuli_Chr04	20398548	(T/G)
6482	CakSNP6482	*Kabuli*	Ca_Kabuli_Chr04	20398685	(A/C)
6483	CakSNP6483	*Kabuli*	Ca_Kabuli_Chr04	20402361	(A/G)
6484	CakSNP6484	*Kabuli*	Ca_Kabuli_Chr04	20402391	(A/G)
6485	CakSNP6485	*Kabuli*	Ca_Kabuli_Chr04	20412329	(A/T)
6486	CakSNP6486	*Kabuli*	Ca_Kabuli_Chr04	20436136	(G/A)
6487	CakSNP6487	*Kabuli*	Ca_Kabuli_Chr04	20444953	(T/C)
6488	CakSNP6488	*Kabuli*	Ca_Kabuli_Chr04	20599050	(A/G)
6489	CakSNP6489	*Kabuli*	Ca_Kabuli_Chr04	20599058	(T/C)
6490	CakSNP6490	*Kabuli*	Ca_Kabuli_Chr04	20599081	(C/T)
6491	CakSNP6491	*Kabuli*	Ca_Kabuli_Chr04	20599091	(T/G)
6492	CakSNP6492	*Kabuli*	Ca_Kabuli_Chr04	20656170	(T/G)
6493	CakSNP6493	*Kabuli*	Ca_Kabuli_Chr04	20669174	(T/C)
6494	CakSNP6494	*Kabuli*	Ca_Kabuli_Chr04	20669442	(G/A)
6495	CakSNP6495	*Kabuli*	Ca_Kabuli_Chr04	20696913	(A/G)
6496	CakSNP6496	*Kabuli*	Ca_Kabuli_Chr04	20696952	(G/T)
6497	CakSNP6497	*Kabuli*	Ca_Kabuli_Chr04	20696979	(C/T)

S.N.	SNP IDs	Cultivars	Chromosomes/scaffolds	Physical positions (bp)	SNPs
6498	CakSNP6498	*Kabuli*	Ca_Kabuli_Chr04	20697338	(T/A)
6499	CakSNP6499	*Kabuli*	Ca_Kabuli_Chr04	20697408	(A/G)
6500	CakSNP6500	*Kabuli*	Ca_Kabuli_Chr04	20774180	(C/T)
6501	CakSNP6501	*Kabuli*	Ca_Kabuli_Chr04	20905105	(G/A)
6502	CakSNP6502	*Kabuli*	Ca_Kabuli_Chr04	21096178	(G/A)
6503	CakSNP6503	*Kabuli*	Ca_Kabuli_Chr04	21385308	(A/T)
6504	CakSNP6504	*Kabuli*	Ca_Kabuli_Chr04	21830534	(C/T)
6505	CakSNP6505	*Kabuli*	Ca_Kabuli_Chr04	21835459	(T/A)
6506	CakSNP6506	*Kabuli*	Ca_Kabuli_Chr04	21835457	(T/A)
6507	CakSNP6507	*Kabuli*	Ca_Kabuli_Chr04	21835454	(C/A)
6508	CakSNP6508	*Kabuli*	Ca_Kabuli_Chr04	21835446	(T/A)
6509	CakSNP6509	*Kabuli*	Ca_Kabuli_Chr04	21931559	(A/G)
6510	CakSNP6510	*Kabuli*	Ca_Kabuli_Chr04	21931558	(G/A)
6511	CakSNP6511	*Kabuli*	Ca_Kabuli_Chr04	21931541	(C/T)
6512	CakSNP6512	*Kabuli*	Ca_Kabuli_Chr04	22079566	(G/A)
6513	CakSNP6513	*Kabuli*	Ca_Kabuli_Chr04	22237989	(A/T)
6514	CakSNP6514	*Kabuli*	Ca_Kabuli_Chr04	22237971	(C/T)
6515	CakSNP6515	*Kabuli*	Ca_Kabuli_Chr04	22288433	(T/C)
6516	CakSNP6516	*Kabuli*	Ca_Kabuli_Chr04	22293495	(T/C)
6517	CakSNP6517	*Kabuli*	Ca_Kabuli_Chr04	22850596	(C/G)
6518	CakSNP6518	*Kabuli*	Ca_Kabuli_Chr04	22859422	(G/A)
6519	CakSNP6519	*Kabuli*	Ca_Kabuli_Chr04	22860281	(A/C)
6520	CakSNP6520	*Kabuli*	Ca_Kabuli_Chr04	22865436	(A/T)
6521	CakSNP6521	*Kabuli*	Ca_Kabuli_Chr04	22865390	(G/A)
6522	CakSNP6522	*Kabuli*	Ca_Kabuli_Chr04	22865389	(C/T)
6523	CakSNP6523	*Kabuli*	Ca_Kabuli_Chr04	22908360	(A/G)
6524	CakSNP6524	*Kabuli*	Ca_Kabuli_Chr04	22971660	(G/A)
6525	CakSNP6525	*Kabuli*	Ca_Kabuli_Chr04	22971766	(A/G)
6526	CakSNP6526	*Kabuli*	Ca_Kabuli_Chr04	22971746	(G/A)
6527	CakSNP6527	*Kabuli*	Ca_Kabuli_Chr04	22971744	(A/C)
6528	CakSNP6528	*Kabuli*	Ca_Kabuli_Chr04	22971847	(C/A)
6529	CakSNP6529	*Kabuli*	Ca_Kabuli_Chr04	22971853	(C/A)
6530	CakSNP6530	*Kabuli*	Ca_Kabuli_Chr04	22971884	(G/A)
6531	CakSNP6531	*Kabuli*	Ca_Kabuli_Chr04	23157656	(C/G)
6532	CakSNP6532	*Kabuli*	Ca_Kabuli_Chr04	23267842	(T/C)
6533	CakSNP6533	*Kabuli*	Ca_Kabuli_Chr04	23267856	(C/T)
6534	CakSNP6534	*Kabuli*	Ca_Kabuli_Chr04	23267858	(C/T)
6535	CakSNP6535	*Kabuli*	Ca_Kabuli_Chr04	23267859	(T/C)

**S.N.**	**SNP IDs**	**Cultivars**	**Chromosomes/scaffolds**	**Physical positions (bp)**	**SNPs**
6536	CakSNP6536	*Kabuli*	Ca_Kabuli_Chr04	23314624	(C/T)
6537	CakSNP6537	*Kabuli*	Ca_Kabuli_Chr04	23314665	(A/C)
6538	CakSNP6538	*Kabuli*	Ca_Kabuli_Chr04	23318875	(T/C)
6539	CakSNP6539	*Kabuli*	Ca_Kabuli_Chr04	23319309	(G/C)
6540	CakSNP6540	*Kabuli*	Ca_Kabuli_Chr04	23333610	(C/T)
6541	CakSNP6541	*Kabuli*	Ca_Kabuli_Chr04	23333554	(T/C)
6542	CakSNP6542	*Kabuli*	Ca_Kabuli_Chr04	23337341	(T/C)
6543	CakSNP6543	*Kabuli*	Ca_Kabuli_Chr04	23337312	(T/G)
6544	CakSNP6544	*Kabuli*	Ca_Kabuli_Chr04	23481919	(G/A)
6545	CakSNP6545	*Kabuli*	Ca_Kabuli_Chr04	23481927	(G/T)
6546	CakSNP6546	*Kabuli*	Ca_Kabuli_Chr04	23481962	(G/A)
6547	CakSNP6547	*Kabuli*	Ca_Kabuli_Chr04	23482033	(A/G)
6548	CakSNP6548	*Kabuli*	Ca_Kabuli_Chr04	23481998	(T/C)
6549	CakSNP6549	*Kabuli*	Ca_Kabuli_Chr04	23486812	(A/G)
6550	CakSNP6550	*Kabuli*	Ca_Kabuli_Chr04	23486859	(A/G)
6551	CakSNP6551	*Kabuli*	Ca_Kabuli_Chr04	23486824	(T/C)
6552	CakSNP6552	*Kabuli*	Ca_Kabuli_Chr04	23671441	(T/G)
6553	CakSNP6553	*Kabuli*	Ca_Kabuli_Chr04	23671443	(A/T)
6554	CakSNP6554	*Kabuli*	Ca_Kabuli_Chr04	23731572	(C/T)
6555	CakSNP6555	*Kabuli*	Ca_Kabuli_Chr04	23732099	(A/T)
6556	CakSNP6556	*Kabuli*	Ca_Kabuli_Chr04	24050459	(G/A)
6557	CakSNP6557	*Kabuli*	Ca_Kabuli_Chr04	24050464	(G/A)
6558	CakSNP6558	*Kabuli*	Ca_Kabuli_Chr04	24120866	(A/G)
6559	CakSNP6559	*Kabuli*	Ca_Kabuli_Chr04	24544345	(G/A)
6560	CakSNP6560	*Kabuli*	Ca_Kabuli_Chr04	24614582	(G/T)
6561	CakSNP6561	*Kabuli*	Ca_Kabuli_Chr04	24729302	(T/C)
6562	CakSNP6562	*Kabuli*	Ca_Kabuli_Chr04	24732991	(G/T)
6563	CakSNP6563	*Kabuli*	Ca_Kabuli_Chr04	24732990	(T/C)
6564	CakSNP6564	*Kabuli*	Ca_Kabuli_Chr04	24771737	(T/A)
6565	CakSNP6565	*Kabuli*	Ca_Kabuli_Chr04	24782714	(C/A)
6566	CakSNP6566	*Kabuli*	Ca_Kabuli_Chr04	24782715	(A/G)
6567	CakSNP6567	*Kabuli*	Ca_Kabuli_Chr04	24856115	(A/C)
6568	CakSNP6568	*Kabuli*	Ca_Kabuli_Chr04	24857501	(A/G)
6569	CakSNP6569	*Kabuli*	Ca_Kabuli_Chr04	25401714	(T/C)
6570	CakSNP6570	*Kabuli*	Ca_Kabuli_Chr04	25446677	(G/C)
6571	CakSNP6571	*Kabuli*	Ca_Kabuli_Chr04	25446684	(T/C)
6572	CakSNP6572	*Kabuli*	Ca_Kabuli_Chr04	25449897	(T/G)
6573	CakSNP6573	*Kabuli*	Ca_Kabuli_Chr04	25455156	(G/A)

S.N.	SNP IDs	Cultivars	Chromosomes/scaffolds	Physical positions (bp)	SNPs
6574	CakSNP6574	*Kabuli*	Ca_Kabuli_Chr04	25455204	(A/G)
6575	CakSNP6575	*Kabuli*	Ca_Kabuli_Chr04	25455225	(G/A)
6576	CakSNP6576	*Kabuli*	Ca_Kabuli_Chr04	25592561	(T/C)
6577	CakSNP6577	*Kabuli*	Ca_Kabuli_Chr04	25592548	(A/G)
6578	CakSNP6578	*Kabuli*	Ca_Kabuli_Chr04	25592528	(A/C)
6579	CakSNP6579	*Kabuli*	Ca_Kabuli_Chr04	25592525	(C/A)
6580	CakSNP6580	*Kabuli*	Ca_Kabuli_Chr04	25592492	(T/C)
6581	CakSNP6581	*Kabuli*	Ca_Kabuli_Chr04	25592487	(A/G)
6582	CakSNP6582	*Kabuli*	Ca_Kabuli_Chr04	25592484	(A/C)
6583	CakSNP6583	*Kabuli*	Ca_Kabuli_Chr04	25592481	(T/C)
6584	CakSNP6584	*Kabuli*	Ca_Kabuli_Chr04	25729905	(C/A)
6585	CakSNP6585	*Kabuli*	Ca_Kabuli_Chr04	25729914	(C/A)
6586	CakSNP6586	*Kabuli*	Ca_Kabuli_Chr04	25729974	(G/C)
6587	CakSNP6587	*Kabuli*	Ca_Kabuli_Chr04	25729977	(C/T)
6588	CakSNP6588	*Kabuli*	Ca_Kabuli_Chr04	25747256	(T/C)
6589	CakSNP6589	*Kabuli*	Ca_Kabuli_Chr04	25747262	(G/A)
6590	CakSNP6590	*Kabuli*	Ca_Kabuli_Chr04	25747274	(T/G)
6591	CakSNP6591	*Kabuli*	Ca_Kabuli_Chr04	25747275	(G/A)
6592	CakSNP6592	*Kabuli*	Ca_Kabuli_Chr04	25747281	(T/C)
6593	CakSNP6593	*Kabuli*	Ca_Kabuli_Chr04	25747295	(G/A)
6594	CakSNP6594	*Kabuli*	Ca_Kabuli_Chr04	25747296	(T/C)
6595	CakSNP6595	*Kabuli*	Ca_Kabuli_Chr04	25747364	(T/A)
6596	CakSNP6596	*Kabuli*	Ca_Kabuli_Chr04	25747297	(G/A)
6597	CakSNP6597	*Kabuli*	Ca_Kabuli_Chr04	25809665	(T/C)
6598	CakSNP6598	*Kabuli*	Ca_Kabuli_Chr04	25921165	(T/C)
6599	CakSNP6599	*Kabuli*	Ca_Kabuli_Chr04	26000532	(C/T)
6600	CakSNP6600	*Kabuli*	Ca_Kabuli_Chr04	26000483	(T/A)
6601	CakSNP6601	*Kabuli*	Ca_Kabuli_Chr04	26002991	(C/A)
6602	CakSNP6602	*Kabuli*	Ca_Kabuli_Chr04	26008744	(G/A)
6603	CakSNP6603	*Kabuli*	Ca_Kabuli_Chr04	26008745	(G/A)
6604	CakSNP6604	*Kabuli*	Ca_Kabuli_Chr04	26008748	(C/T)
6605	CakSNP6605	*Kabuli*	Ca_Kabuli_Chr04	26008768	(A/G)
6606	CakSNP6606	*Kabuli*	Ca_Kabuli_Chr04	26008816	(C/A)
6607	CakSNP6607	*Kabuli*	Ca_Kabuli_Chr04	26009438	(G/T)
6608	CakSNP6608	*Kabuli*	Ca_Kabuli_Chr04	26009439	(G/T)
6609	CakSNP6609	*Kabuli*	Ca_Kabuli_Chr04	26222180	(A/G)
6610	CakSNP6610	*Kabuli*	Ca_Kabuli_Chr04	26282236	(A/C)
6611	CakSNP6611	*Kabuli*	Ca_Kabuli_Chr04	26324460	(T/C)

S.N.	SNP IDs	Cultivars	Chromosomes/scaffolds	Physical positions (bp)	SNPs
6612	CakSNP6612	*Kabuli*	Ca_Kabuli_Chr04	26529606	(C/T)
6613	CakSNP6613	*Kabuli*	Ca_Kabuli_Chr04	26532763	(T/G)
6614	CakSNP6614	*Kabuli*	Ca_Kabuli_Chr04	26536919	(C/G)
6615	CakSNP6615	*Kabuli*	Ca_Kabuli_Chr04	26537053	(C/T)
6616	CakSNP6616	*Kabuli*	Ca_Kabuli_Chr04	26537258	(A/C)
6617	CakSNP6617	*Kabuli*	Ca_Kabuli_Chr04	26869756	(A/G)
6618	CakSNP6618	*Kabuli*	Ca_Kabuli_Chr04	26915602	(G/A)
6619	CakSNP6619	*Kabuli*	Ca_Kabuli_Chr04	26915615	(A/G)
6620	CakSNP6620	*Kabuli*	Ca_Kabuli_Chr04	26915621	(G/A)
6621	CakSNP6621	*Kabuli*	Ca_Kabuli_Chr04	26915626	(C/T)
6622	CakSNP6622	*Kabuli*	Ca_Kabuli_Chr04	26989085	(G/A)
6623	CakSNP6623	*Kabuli*	Ca_Kabuli_Chr04	26989090	(G/T)
6624	CakSNP6624	*Kabuli*	Ca_Kabuli_Chr04	26989204	(A/C)
6625	CakSNP6625	*Kabuli*	Ca_Kabuli_Chr04	27102807	(G/A)
6626	CakSNP6626	*Kabuli*	Ca_Kabuli_Chr04	27102817	(A/C)
6627	CakSNP6627	*Kabuli*	Ca_Kabuli_Chr04	27102833	(G/T)
6628	CakSNP6628	*Kabuli*	Ca_Kabuli_Chr04	27262241	(C/T)
6629	CakSNP6629	*Kabuli*	Ca_Kabuli_Chr04	27262893	(G/A)
6630	CakSNP6630	*Kabuli*	Ca_Kabuli_Chr04	27262870	(C/T)
6631	CakSNP6631	*Kabuli*	Ca_Kabuli_Chr04	27262830	(T/A)
6632	CakSNP6632	*Kabuli*	Ca_Kabuli_Chr04	27268301	(G/A)
6633	CakSNP6633	*Kabuli*	Ca_Kabuli_Chr04	27268278	(C/T)
6634	CakSNP6634	*Kabuli*	Ca_Kabuli_Chr04	27493010	(G/T)
6635	CakSNP6635	*Kabuli*	Ca_Kabuli_Chr04	27613450	(C/T)
6636	CakSNP6636	*Kabuli*	Ca_Kabuli_Chr04	27617803	(A/G)
6637	CakSNP6637	*Kabuli*	Ca_Kabuli_Chr04	27626559	(G/A)
6638	CakSNP6638	*Kabuli*	Ca_Kabuli_Chr04	27626999	(C/T)
6639	CakSNP6639	*Kabuli*	Ca_Kabuli_Chr04	27661241	(T/G)
6640	CakSNP6640	*Kabuli*	Ca_Kabuli_Chr04	27669829	(C/G)
6641	CakSNP6641	*Kabuli*	Ca_Kabuli_Chr04	27777968	(A/C)
6642	CakSNP6642	*Kabuli*	Ca_Kabuli_Chr04	27786162	(G/C)
6643	CakSNP6643	*Kabuli*	Ca_Kabuli_Chr04	27970989	(T/G)
6644	CakSNP6644	*Kabuli*	Ca_Kabuli_Chr04	28061002	(A/C)
6645	CakSNP6645	*Kabuli*	Ca_Kabuli_Chr04	28092609	(G/A)
6646	CakSNP6646	*Kabuli*	Ca_Kabuli_Chr04	28092727	(T/A)
6647	CakSNP6647	*Kabuli*	Ca_Kabuli_Chr04	28092725	(T/G)
6648	CakSNP6648	*Kabuli*	Ca_Kabuli_Chr04	28092724	(T/A)
6649	CakSNP6649	*Kabuli*	Ca_Kabuli_Chr04	28268788	(G/A)

S.N.	SNP IDs	Cultivars	Chromosomes/scaffolds	Physical positions (bp)	SNPs
6650	CakSNP6650	*Kabuli*	Ca_Kabuli_Chr04	28289900	(C/A)
6651	CakSNP6651	*Kabuli*	Ca_Kabuli_Chr04	28299238	(G/A)
6652	CakSNP6652	*Kabuli*	Ca_Kabuli_Chr04	28301853	(T/C)
6653	CakSNP6653	*Kabuli*	Ca_Kabuli_Chr04	28423079	(T/C)
6654	CakSNP6654	*Kabuli*	Ca_Kabuli_Chr04	28423140	(A/G)
6655	CakSNP6655	*Kabuli*	Ca_Kabuli_Chr04	28431706	(G/A)
6656	CakSNP6656	*Kabuli*	Ca_Kabuli_Chr04	28790085	(A/G)
6657	CakSNP6657	*Kabuli*	Ca_Kabuli_Chr04	29582296	(C/T)
6658	CakSNP6658	*Kabuli*	Ca_Kabuli_Chr04	29582289	(G/A)
6659	CakSNP6659	*Kabuli*	Ca_Kabuli_Chr04	29582285	(C/A)
6660	CakSNP6660	*Kabuli*	Ca_Kabuli_Chr04	29582282	(C/A)
6661	CakSNP6661	*Kabuli*	Ca_Kabuli_Chr04	29582273	(G/A)
6662	CakSNP6662	*Kabuli*	Ca_Kabuli_Chr04	29582268	(C/G)
6663	CakSNP6663	*Kabuli*	Ca_Kabuli_Chr04	29582257	(G/A)
6664	CakSNP6664	*Kabuli*	Ca_Kabuli_Chr04	29582255	(C/T)
6665	CakSNP6665	*Kabuli*	Ca_Kabuli_Chr04	29582252	(G/A)
6666	CakSNP6666	*Kabuli*	Ca_Kabuli_Chr04	29582249	(C/T)
6667	CakSNP6667	*Kabuli*	Ca_Kabuli_Chr04	29582235	(T/A)
6668	CakSNP6668	*Kabuli*	Ca_Kabuli_Chr04	29582232	(C/T)
6669	CakSNP6669	*Kabuli*	Ca_Kabuli_Chr04	29582227	(G/A)
6670	CakSNP6670	*Kabuli*	Ca_Kabuli_Chr04	29582219	(A/C)
6671	CakSNP6671	*Kabuli*	Ca_Kabuli_Chr04	29582271	(C/T)
6672	CakSNP6672	*Kabuli*	Ca_Kabuli_Chr04	29582286	(T/A)
6673	CakSNP6673	*Kabuli*	Ca_Kabuli_Chr04	29582294	(G/A)
6674	CakSNP6674	*Kabuli*	Ca_Kabuli_Chr04	29582301	(G/T)
6675	CakSNP6675	*Kabuli*	Ca_Kabuli_Chr04	29671563	(G/A)
6676	CakSNP6676	*Kabuli*	Ca_Kabuli_Chr04	29671606	(A/G)
6677	CakSNP6677	*Kabuli*	Ca_Kabuli_Chr04	29683718	(A/T)
6678	CakSNP6678	*Kabuli*	Ca_Kabuli_Chr04	29923380	(G/A)
6679	CakSNP6679	*Kabuli*	Ca_Kabuli_Chr04	29958122	(T/G)
6680	CakSNP6680	*Kabuli*	Ca_Kabuli_Chr04	29958113	(G/A)
6681	CakSNP6681	*Kabuli*	Ca_Kabuli_Chr04	29972268	(T/G)
6682	CakSNP6682	*Kabuli*	Ca_Kabuli_Chr04	30066903	(G/A)
6683	CakSNP6683	*Kabuli*	Ca_Kabuli_Chr04	30153390	(G/A)
6684	CakSNP6684	*Kabuli*	Ca_Kabuli_Chr04	30153448	(T/C)
6685	CakSNP6685	*Kabuli*	Ca_Kabuli_Chr04	30153505	(C/G)
6686	CakSNP6686	*Kabuli*	Ca_Kabuli_Chr04	30258044	(A/T)
6687	CakSNP6687	*Kabuli*	Ca_Kabuli_Chr04	30258045	(A/T)

S.N.	SNP IDs	Cultivars	Chromosomes/scaffolds	Physical positions (bp)	SNPs
6688	CakSNP6688	*Kabuli*	Ca_Kabuli_Chr04	30258056	(A/G)
6689	CakSNP6689	*Kabuli*	Ca_Kabuli_Chr04	30258484	(G/C)
6690	CakSNP6690	*Kabuli*	Ca_Kabuli_Chr04	30258613	(C/A)
6691	CakSNP6691	*Kabuli*	Ca_Kabuli_Chr04	30258607	(C/A)
6692	CakSNP6692	*Kabuli*	Ca_Kabuli_Chr04	30258604	(C/A)
6693	CakSNP6693	*Kabuli*	Ca_Kabuli_Chr04	30258596	(G/C)
6694	CakSNP6694	*Kabuli*	Ca_Kabuli_Chr04	30258567	(C/A)
6695	CakSNP6695	*Kabuli*	Ca_Kabuli_Chr04	30260485	(C/A)
6696	CakSNP6696	*Kabuli*	Ca_Kabuli_Chr04	30281991	(A/G)
6697	CakSNP6697	*Kabuli*	Ca_Kabuli_Chr04	30301002	(T/G)
6698	CakSNP6698	*Kabuli*	Ca_Kabuli_Chr04	30308719	(A/C)
6699	CakSNP6699	*Kabuli*	Ca_Kabuli_Chr04	30308812	(G/A)
6700	CakSNP6700	*Kabuli*	Ca_Kabuli_Chr04	30308788	(T/C)
6701	CakSNP6701	*Kabuli*	Ca_Kabuli_Chr04	30463063	(T/C)
6702	CakSNP6702	*Kabuli*	Ca_Kabuli_Chr04	30463117	(C/A)
6703	CakSNP6703	*Kabuli*	Ca_Kabuli_Chr04	30599096	(C/A)
6704	CakSNP6704	*Kabuli*	Ca_Kabuli_Chr04	30599188	(C/T)
6705	CakSNP6705	*Kabuli*	Ca_Kabuli_Chr04	30667916	(G/A)
6706	CakSNP6706	*Kabuli*	Ca_Kabuli_Chr04	30668893	(A/C)
6707	CakSNP6707	*Kabuli*	Ca_Kabuli_Chr04	30851027	(A/T)
6708	CakSNP6708	*Kabuli*	Ca_Kabuli_Chr04	30851411	(T/G)
6709	CakSNP6709	*Kabuli*	Ca_Kabuli_Chr04	30851840	(A/T)
6710	CakSNP6710	*Kabuli*	Ca_Kabuli_Chr04	30931586	(G/T)
6711	CakSNP6711	*Kabuli*	Ca_Kabuli_Chr04	31218647	(T/C)
6712	CakSNP6712	*Kabuli*	Ca_Kabuli_Chr04	31221264	(T/G)
6713	CakSNP6713	*Kabuli*	Ca_Kabuli_Chr04	31221253	(G/A)
6714	CakSNP6714	*Kabuli*	Ca_Kabuli_Chr04	31221226	(T/A)
6715	CakSNP6715	*Kabuli*	Ca_Kabuli_Chr04	31224001	(G/A)
6716	CakSNP6716	*Kabuli*	Ca_Kabuli_Chr04	31262596	(T/A)
6717	CakSNP6717	*Kabuli*	Ca_Kabuli_Chr04	31263205	(T/A)
6718	CakSNP6718	*Kabuli*	Ca_Kabuli_Chr04	31594126	(A/C)
6719	CakSNP6719	*Kabuli*	Ca_Kabuli_Chr04	31618949	(T/A)
6720	CakSNP6720	*Kabuli*	Ca_Kabuli_Chr04	31875713	(A/G)
6721	CakSNP6721	*Kabuli*	Ca_Kabuli_Chr04	31987890	(A/C)
6722	CakSNP6722	*Kabuli*	Ca_Kabuli_Chr04	32042488	(T/G)
6723	CakSNP6723	*Kabuli*	Ca_Kabuli_Chr04	32042527	(C/T)
6724	CakSNP6724	*Kabuli*	Ca_Kabuli_Chr04	32068961	(A/G)
6725	CakSNP6725	*Kabuli*	Ca_Kabuli_Chr04	32073552	(T/C)

S.N.	SNP IDs	Cultivars	Chromosomes/scaffolds	Physical positions (bp)	SNPs
6726	CakSNP6726	*Kabuli*	Ca_Kabuli_Chr04	32073550	(C/G)
6727	CakSNP6727	*Kabuli*	Ca_Kabuli_Chr04	32073549	(G/C)
6728	CakSNP6728	*Kabuli*	Ca_Kabuli_Chr04	32166559	(C/G)
6729	CakSNP6729	*Kabuli*	Ca_Kabuli_Chr04	32166557	(T/A)
6730	CakSNP6730	*Kabuli*	Ca_Kabuli_Chr04	32183307	(C/G)
6731	CakSNP6731	*Kabuli*	Ca_Kabuli_Chr04	32184628	(A/G)
6732	CakSNP6732	*Kabuli*	Ca_Kabuli_Chr04	32212899	(G/A)
6733	CakSNP6733	*Kabuli*	Ca_Kabuli_Chr04	32297780	(C/A)
6734	CakSNP6734	*Kabuli*	Ca_Kabuli_Chr04	32297758	(G/A)
6735	CakSNP6735	*Kabuli*	Ca_Kabuli_Chr04	32297734	(A/G)
6736	CakSNP6736	*Kabuli*	Ca_Kabuli_Chr04	32303394	(C/T)
6737	CakSNP6737	*Kabuli*	Ca_Kabuli_Chr04	32429228	(A/T)
6738	CakSNP6738	*Kabuli*	Ca_Kabuli_Chr04	32429222	(A/C)
6739	CakSNP6739	*Kabuli*	Ca_Kabuli_Chr04	32521346	(T/C)
6740	CakSNP6740	*Kabuli*	Ca_Kabuli_Chr04	32602658	(C/T)
6741	CakSNP6741	*Kabuli*	Ca_Kabuli_Chr04	32604261	(G/A)
6742	CakSNP6742	*Kabuli*	Ca_Kabuli_Chr04	32604538	(T/C)
6743	CakSNP6743	*Kabuli*	Ca_Kabuli_Chr04	32604510	(A/C)
6744	CakSNP6744	*Kabuli*	Ca_Kabuli_Chr04	32636315	(T/C)
6745	CakSNP6745	*Kabuli*	Ca_Kabuli_Chr04	32636276	(T/C)
6746	CakSNP6746	*Kabuli*	Ca_Kabuli_Chr04	32636428	(C/A)
6747	CakSNP6747	*Kabuli*	Ca_Kabuli_Chr04	32636459	(A/G)
6748	CakSNP6748	*Kabuli*	Ca_Kabuli_Chr04	32636690	(C/T)
6749	CakSNP6749	*Kabuli*	Ca_Kabuli_Chr04	32636953	(A/G)
6750	CakSNP6750	*Kabuli*	Ca_Kabuli_Chr04	32639121	(C/T)
6751	CakSNP6751	*Kabuli*	Ca_Kabuli_Chr04	32639188	(C/G)
6752	CakSNP6752	*Kabuli*	Ca_Kabuli_Chr04	32639118	(A/C)
6753	CakSNP6753	*Kabuli*	Ca_Kabuli_Chr04	32639262	(A/G)
6754	CakSNP6754	*Kabuli*	Ca_Kabuli_Chr04	32639315	(A/G)
6755	CakSNP6755	*Kabuli*	Ca_Kabuli_Chr04	32639654	(T/C)
6756	CakSNP6756	*Kabuli*	Ca_Kabuli_Chr04	32639681	(C/T)
6757	CakSNP6757	*Kabuli*	Ca_Kabuli_Chr04	32639770	(C/G)
6758	CakSNP6758	*Kabuli*	Ca_Kabuli_Chr04	32640911	(A/G)
6759	CakSNP6759	*Kabuli*	Ca_Kabuli_Chr04	32853834	(T/C)
6760	CakSNP6760	*Kabuli*	Ca_Kabuli_Chr04	32853838	(G/T)
6761	CakSNP6761	*Kabuli*	Ca_Kabuli_Chr04	33068276	(G/A)
6762	CakSNP6762	*Kabuli*	Ca_Kabuli_Chr04	33221361	(T/C)
6763	CakSNP6763	*Kabuli*	Ca_Kabuli_Chr04	33221371	(T/C)

S.N.	SNP IDs	Cultivars	Chromosomes/scaffolds	Physical positions (bp)	SNPs
6764	CakSNP6764	*Kabuli*	Ca_Kabuli_Chr04	33221397	(T/G)
6765	CakSNP6765	*Kabuli*	Ca_Kabuli_Chr04	33509759	(A/C)
6766	CakSNP6766	*Kabuli*	Ca_Kabuli_Chr04	33509762	(G/A)
6767	CakSNP6767	*Kabuli*	Ca_Kabuli_Chr04	33509804	(A/G)
6768	CakSNP6768	*Kabuli*	Ca_Kabuli_Chr04	33641757	(C/T)
6769	CakSNP6769	*Kabuli*	Ca_Kabuli_Chr04	33820657	(A/C)
6770	CakSNP6770	*Kabuli*	Ca_Kabuli_Chr04	33874346	(T/C)
6771	CakSNP6771	*Kabuli*	Ca_Kabuli_Chr04	33874327	(T/C)
6772	CakSNP6772	*Kabuli*	Ca_Kabuli_Chr04	33874313	(G/A)
6773	CakSNP6773	*Kabuli*	Ca_Kabuli_Chr04	33929598	(A/C)
6774	CakSNP6774	*Kabuli*	Ca_Kabuli_Chr04	33929777	(C/T)
6775	CakSNP6775	*Kabuli*	Ca_Kabuli_Chr04	34032176	(C/T)
6776	CakSNP6776	*Kabuli*	Ca_Kabuli_Chr04	34155135	(A/C)
6777	CakSNP6777	*Kabuli*	Ca_Kabuli_Chr04	34155131	(T/A)
6778	CakSNP6778	*Kabuli*	Ca_Kabuli_Chr04	34155128	(C/T)
6779	CakSNP6779	*Kabuli*	Ca_Kabuli_Chr04	34155127	(G/A)
6780	CakSNP6780	*Kabuli*	Ca_Kabuli_Chr04	34155124	(G/A)
6781	CakSNP6781	*Kabuli*	Ca_Kabuli_Chr04	34155084	(C/G)
6782	CakSNP6782	*Kabuli*	Ca_Kabuli_Chr04	34155083	(C/T)
6783	CakSNP6783	*Kabuli*	Ca_Kabuli_Chr04	34222792	(G/C)
6784	CakSNP6784	*Kabuli*	Ca_Kabuli_Chr04	34354519	(G/T)
6785	CakSNP6785	*Kabuli*	Ca_Kabuli_Chr04	34487064	(T/G)
6786	CakSNP6786	*Kabuli*	Ca_Kabuli_Chr04	34744344	(T/G)
6787	CakSNP6787	*Kabuli*	Ca_Kabuli_Chr04	34744398	(T/A)
6788	CakSNP6788	*Kabuli*	Ca_Kabuli_Chr04	34784285	(G/A)
6789	CakSNP6789	*Kabuli*	Ca_Kabuli_Chr04	34798597	(C/A)
6790	CakSNP6790	*Kabuli*	Ca_Kabuli_Chr04	34916032	(C/T)
6791	CakSNP6791	*Kabuli*	Ca_Kabuli_Chr04	34924575	(C/T)
6792	CakSNP6792	*Kabuli*	Ca_Kabuli_Chr04	34955953	(A/G)
6793	CakSNP6793	*Kabuli*	Ca_Kabuli_Chr04	34955971	(A/T)
6794	CakSNP6794	*Kabuli*	Ca_Kabuli_Chr04	34956006	(C/T)
6795	CakSNP6795	*Kabuli*	Ca_Kabuli_Chr04	34956019	(G/A)
6796	CakSNP6796	*Kabuli*	Ca_Kabuli_Chr04	34956035	(C/T)
6797	CakSNP6797	*Kabuli*	Ca_Kabuli_Chr04	34956111	(G/T)
6798	CakSNP6798	*Kabuli*	Ca_Kabuli_Chr04	34956031	(G/A)
6799	CakSNP6799	*Kabuli*	Ca_Kabuli_Chr04	35141281	(A/T)
6800	CakSNP6800	*Kabuli*	Ca_Kabuli_Chr04	35141309	(A/C)
6801	CakSNP6801	*Kabuli*	Ca_Kabuli_Chr04	35141340	(G/A)

S.N.	SNP IDs	Cultivars	Chromosomes/scaffolds	Physical positions (bp)	SNPs
6802	CakSNP6802	*Kabuli*	Ca_Kabuli_Chr04	35141353	(C/G)
6803	CakSNP6803	*Kabuli*	Ca_Kabuli_Chr04	35141359	(C/G)
6804	CakSNP6804	*Kabuli*	Ca_Kabuli_Chr04	35141375	(A/G)
6805	CakSNP6805	*Kabuli*	Ca_Kabuli_Chr04	35141403	(C/T)
6806	CakSNP6806	*Kabuli*	Ca_Kabuli_Chr04	35141425	(A/T)
6807	CakSNP6807	*Kabuli*	Ca_Kabuli_Chr04	35141421	(T/A)
6808	CakSNP6808	*Kabuli*	Ca_Kabuli_Chr04	35141408	(A/G)
6809	CakSNP6809	*Kabuli*	Ca_Kabuli_Chr04	35168915	(C/T)
6810	CakSNP6810	*Kabuli*	Ca_Kabuli_Chr04	35168939	(C/T)
6811	CakSNP6811	*Kabuli*	Ca_Kabuli_Chr04	35229966	(C/T)
6812	CakSNP6812	*Kabuli*	Ca_Kabuli_Chr04	35230144	(A/G)
6813	CakSNP6813	*Kabuli*	Ca_Kabuli_Chr04	35231891	(C/T)
6814	CakSNP6814	*Kabuli*	Ca_Kabuli_Chr04	35304102	(C/T)
6815	CakSNP6815	*Kabuli*	Ca_Kabuli_Chr04	35304108	(C/A)
6816	CakSNP6816	*Kabuli*	Ca_Kabuli_Chr04	35309709	(A/G)
6817	CakSNP6817	*Kabuli*	Ca_Kabuli_Chr04	35311815	(A/C)
6818	CakSNP6818	*Kabuli*	Ca_Kabuli_Chr04	35337282	(A/G)
6819	CakSNP6819	*Kabuli*	Ca_Kabuli_Chr04	35344356	(A/G)
6820	CakSNP6820	*Kabuli*	Ca_Kabuli_Chr04	35344353	(T/C)
6821	CakSNP6821	*Kabuli*	Ca_Kabuli_Chr04	35344323	(C/T)
6822	CakSNP6822	*Kabuli*	Ca_Kabuli_Chr04	35346415	(G/A)
6823	CakSNP6823	*Kabuli*	Ca_Kabuli_Chr04	35346417	(C/T)
6824	CakSNP6824	*Kabuli*	Ca_Kabuli_Chr04	35346495	(C/T)
6825	CakSNP6825	*Kabuli*	Ca_Kabuli_Chr04	35346465	(G/T)
6826	CakSNP6826	*Kabuli*	Ca_Kabuli_Chr04	35346558	(G/T)
6827	CakSNP6827	*Kabuli*	Ca_Kabuli_Chr04	35346563	(G/T)
6828	CakSNP6828	*Kabuli*	Ca_Kabuli_Chr04	35346573	(A/G)
6829	CakSNP6829	*Kabuli*	Ca_Kabuli_Chr04	35346576	(C/G)
6830	CakSNP6830	*Kabuli*	Ca_Kabuli_Chr04	35346624	(G/A)
6831	CakSNP6831	*Kabuli*	Ca_Kabuli_Chr04	35393505	(G/T)
6832	CakSNP6832	*Kabuli*	Ca_Kabuli_Chr04	35393643	(G/A)
6833	CakSNP6833	*Kabuli*	Ca_Kabuli_Chr04	35455095	(A/C)
6834	CakSNP6834	*Kabuli*	Ca_Kabuli_Chr04	35455062	(A/T)
6835	CakSNP6835	*Kabuli*	Ca_Kabuli_Chr04	35455708	(G/A)
6836	CakSNP6836	*Kabuli*	Ca_Kabuli_Chr04	35469308	(T/G)
6837	CakSNP6837	*Kabuli*	Ca_Kabuli_Chr04	35469304	(G/A)
6838	CakSNP6838	*Kabuli*	Ca_Kabuli_Chr04	35487504	(G/C)
6839	CakSNP6839	*Kabuli*	Ca_Kabuli_Chr04	35487496	(C/A)

S.N.	SNP IDs	Cultivars	Chromosomes/scaffolds	Physical positions (bp)	SNPs
6840	CakSNP6840	*Kabuli*	Ca_Kabuli_Chr04	35487471	(A/C)
6841	CakSNP6841	*Kabuli*	Ca_Kabuli_Chr04	35528758	(C/A)
6842	CakSNP6842	*Kabuli*	Ca_Kabuli_Chr04	35534777	(G/C)
6843	CakSNP6843	*Kabuli*	Ca_Kabuli_Chr04	35534877	(T/C)
6844	CakSNP6844	*Kabuli*	Ca_Kabuli_Chr04	35534891	(C/T)
6845	CakSNP6845	*Kabuli*	Ca_Kabuli_Chr04	35574379	(C/A)
6846	CakSNP6846	*Kabuli*	Ca_Kabuli_Chr04	35634712	(A/G)
6847	CakSNP6847	*Kabuli*	Ca_Kabuli_Chr04	35634751	(A/G)
6848	CakSNP6848	*Kabuli*	Ca_Kabuli_Chr04	35634873	(G/T)
6849	CakSNP6849	*Kabuli*	Ca_Kabuli_Chr04	35961593	(A/C)
6850	CakSNP6850	*Kabuli*	Ca_Kabuli_Chr04	35977422	(G/A)
6851	CakSNP6851	*Kabuli*	Ca_Kabuli_Chr04	36005847	(T/G)
6852	CakSNP6852	*Kabuli*	Ca_Kabuli_Chr04	36010756	(T/G)
6853	CakSNP6853	*Kabuli*	Ca_Kabuli_Chr04	36153818	(A/G)
6854	CakSNP6854	*Kabuli*	Ca_Kabuli_Chr04	36154771	(T/C)
6855	CakSNP6855	*Kabuli*	Ca_Kabuli_Chr04	36154820	(G/A)
6856	CakSNP6856	*Kabuli*	Ca_Kabuli_Chr04	36154822	(T/G)
6857	CakSNP6857	*Kabuli*	Ca_Kabuli_Chr04	36184969	(A/G)
6858	CakSNP6858	*Kabuli*	Ca_Kabuli_Chr04	36277383	(C/T)
6859	CakSNP6859	*Kabuli*	Ca_Kabuli_Chr04	36368713	(T/C)
6860	CakSNP6860	*Kabuli*	Ca_Kabuli_Chr04	36445345	(G/A)
6861	CakSNP6861	*Kabuli*	Ca_Kabuli_Chr04	36445367	(A/G)
6862	CakSNP6862	*Kabuli*	Ca_Kabuli_Chr04	36449101	(C/T)
6863	CakSNP6863	*Kabuli*	Ca_Kabuli_Chr04	36461836	(A/G)
6864	CakSNP6864	*Kabuli*	Ca_Kabuli_Chr04	36467928	(C/T)
6865	CakSNP6865	*Kabuli*	Ca_Kabuli_Chr04	36467943	(A/G)
6866	CakSNP6866	*Kabuli*	Ca_Kabuli_Chr04	36467964	(G/A)
6867	CakSNP6867	*Kabuli*	Ca_Kabuli_Chr04	36467967	(T/C)
6868	CakSNP6868	*Kabuli*	Ca_Kabuli_Chr04	36467980	(C/A)
6869	CakSNP6869	*Kabuli*	Ca_Kabuli_Chr04	36467988	(C/T)
6870	CakSNP6870	*Kabuli*	Ca_Kabuli_Chr04	36467990	(T/G)
6871	CakSNP6871	*Kabuli*	Ca_Kabuli_Chr04	36468003	(T/A)
6872	CakSNP6872	*Kabuli*	Ca_Kabuli_Chr04	36468053	(A/G)
6873	CakSNP6873	*Kabuli*	Ca_Kabuli_Chr04	36488966	(A/G)
6874	CakSNP6874	*Kabuli*	Ca_Kabuli_Chr04	36537118	(C/T)
6875	CakSNP6875	*Kabuli*	Ca_Kabuli_Chr04	36537124	(C/T)
6876	CakSNP6876	*Kabuli*	Ca_Kabuli_Chr04	36537170	(C/T)
6877	CakSNP6877	*Kabuli*	Ca_Kabuli_Chr04	36541088	(G/T)

S.N.	SNP IDs	Cultivars	Chromosomes/scaffolds	Physical positions (bp)	SNPs
6878	CakSNP6878	*Kabuli*	Ca_Kabuli_Chr04	36541087	(T/C)
6879	CakSNP6879	*Kabuli*	Ca_Kabuli_Chr04	36541083	(A/C)
6880	CakSNP6880	*Kabuli*	Ca_Kabuli_Chr04	36639333	(A/T)
6881	CakSNP6881	*Kabuli*	Ca_Kabuli_Chr04	36639382	(T/C)
6882	CakSNP6882	*Kabuli*	Ca_Kabuli_Chr04	36639474	(G/C)
6883	CakSNP6883	*Kabuli*	Ca_Kabuli_Chr04	36673677	(C/A)
6884	CakSNP6884	*Kabuli*	Ca_Kabuli_Chr04	36682215	(G/A)
6885	CakSNP6885	*Kabuli*	Ca_Kabuli_Chr04	36682213	(C/T)
6886	CakSNP6886	*Kabuli*	Ca_Kabuli_Chr04	36682211	(G/A)
6887	CakSNP6887	*Kabuli*	Ca_Kabuli_Chr04	36682253	(T/G)
6888	CakSNP6888	*Kabuli*	Ca_Kabuli_Chr04	36682231	(G/T)
6889	CakSNP6889	*Kabuli*	Ca_Kabuli_Chr04	36709731	(G/A)
6890	CakSNP6890	*Kabuli*	Ca_Kabuli_Chr04	36754121	(T/C)
6891	CakSNP6891	*Kabuli*	Ca_Kabuli_Chr04	36754149	(G/A)
6892	CakSNP6892	*Kabuli*	Ca_Kabuli_Chr04	36754290	(T/A)
6893	CakSNP6893	*Kabuli*	Ca_Kabuli_Chr04	36754378	(C/T)
6894	CakSNP6894	*Kabuli*	Ca_Kabuli_Chr04	36754377	(T/A)
6895	CakSNP6895	*Kabuli*	Ca_Kabuli_Chr04	36754348	(C/T)
6896	CakSNP6896	*Kabuli*	Ca_Kabuli_Chr04	36754343	(A/C)
6897	CakSNP6897	*Kabuli*	Ca_Kabuli_Chr04	36754327	(G/A)
6898	CakSNP6898	*Kabuli*	Ca_Kabuli_Chr04	36754468	(G/A)
6899	CakSNP6899	*Kabuli*	Ca_Kabuli_Chr04	36754453	(A/G)
6900	CakSNP6900	*Kabuli*	Ca_Kabuli_Chr04	36799551	(C/T)
6901	CakSNP6901	*Kabuli*	Ca_Kabuli_Chr04	36831035	(A/G)
6902	CakSNP6902	*Kabuli*	Ca_Kabuli_Chr04	36831199	(G/A)
6903	CakSNP6903	*Kabuli*	Ca_Kabuli_Chr04	36831267	(A/C)
6904	CakSNP6904	*Kabuli*	Ca_Kabuli_Chr04	36955706	(G/T)
6905	CakSNP6905	*Kabuli*	Ca_Kabuli_Chr04	36977192	(C/T)
6906	CakSNP6906	*Kabuli*	Ca_Kabuli_Chr04	36977194	(G/A)
6907	CakSNP6907	*Kabuli*	Ca_Kabuli_Chr04	36979866	(T/G)
6908	CakSNP6908	*Kabuli*	Ca_Kabuli_Chr04	36979926	(G/T)
6909	CakSNP6909	*Kabuli*	Ca_Kabuli_Chr04	36979879	(C/T)
6910	CakSNP6910	*Kabuli*	Ca_Kabuli_Chr04	36982955	(A/G)
6911	CakSNP6911	*Kabuli*	Ca_Kabuli_Chr04	36984727	(C/G)
6912	CakSNP6912	*Kabuli*	Ca_Kabuli_Chr04	36984829	(C/T)
6913	CakSNP6913	*Kabuli*	Ca_Kabuli_Chr04	36984795	(T/A)
6914	CakSNP6914	*Kabuli*	Ca_Kabuli_Chr04	36998201	(A/C)
6915	CakSNP6915	*Kabuli*	Ca_Kabuli_Chr04	36998209	(C/T)

S.N.	SNP IDs	Cultivars	Chromosomes/scaffolds	Physical positions (bp)	SNPs
6916	CakSNP6916	*Kabuli*	Ca_Kabuli_Chr04	37064588	(A/G)
6917	CakSNP6917	*Kabuli*	Ca_Kabuli_Chr04	37064630	(G/A)
6918	CakSNP6918	*Kabuli*	Ca_Kabuli_Chr04	37185576	(T/A)
6919	CakSNP6919	*Kabuli*	Ca_Kabuli_Chr04	37185577	(T/A)
6920	CakSNP6920	*Kabuli*	Ca_Kabuli_Chr04	37185578	(T/A)
6921	CakSNP6921	*Kabuli*	Ca_Kabuli_Chr04	37237211	(G/A)
6922	CakSNP6922	*Kabuli*	Ca_Kabuli_Chr04	37237276	(T/C)
6923	CakSNP6923	*Kabuli*	Ca_Kabuli_Chr04	37241235	(A/G)
6924	CakSNP6924	*Kabuli*	Ca_Kabuli_Chr04	37241262	(G/A)
6925	CakSNP6925	*Kabuli*	Ca_Kabuli_Chr04	37260697	(T/C)
6926	CakSNP6926	*Kabuli*	Ca_Kabuli_Chr04	37260873	(A/G)
6927	CakSNP6927	*Kabuli*	Ca_Kabuli_Chr04	37260841	(T/A)
6928	CakSNP6928	*Kabuli*	Ca_Kabuli_Chr04	37264628	(G/A)
6929	CakSNP6929	*Kabuli*	Ca_Kabuli_Chr04	37286591	(T/A)
6930	CakSNP6930	*Kabuli*	Ca_Kabuli_Chr04	37286625	(C/A)
6931	CakSNP6931	*Kabuli*	Ca_Kabuli_Chr04	37286632	(G/A)
6932	CakSNP6932	*Kabuli*	Ca_Kabuli_Chr04	37286663	(A/G)
6933	CakSNP6933	*Kabuli*	Ca_Kabuli_Chr04	37293752	(G/A)
6934	CakSNP6934	*Kabuli*	Ca_Kabuli_Chr04	37293780	(T/A)
6935	CakSNP6935	*Kabuli*	Ca_Kabuli_Chr04	37293773	(A/T)
6936	CakSNP6936	*Kabuli*	Ca_Kabuli_Chr04	37293769	(C/A)
6937	CakSNP6937	*Kabuli*	Ca_Kabuli_Chr04	37294720	(A/G)
6938	CakSNP6938	*Kabuli*	Ca_Kabuli_Chr04	37316574	(T/A)
6939	CakSNP6939	*Kabuli*	Ca_Kabuli_Chr04	37316748	(T/A)
6940	CakSNP6940	*Kabuli*	Ca_Kabuli_Chr04	37316738	(C/T)
6941	CakSNP6941	*Kabuli*	Ca_Kabuli_Chr04	37316713	(A/T)
6942	CakSNP6942	*Kabuli*	Ca_Kabuli_Chr04	37316700	(C/A)
6943	CakSNP6943	*Kabuli*	Ca_Kabuli_Chr04	37349071	(T/C)
6944	CakSNP6944	*Kabuli*	Ca_Kabuli_Chr04	37349212	(A/C)
6945	CakSNP6945	*Kabuli*	Ca_Kabuli_Chr04	37349239	(C/G)
6946	CakSNP6946	*Kabuli*	Ca_Kabuli_Chr04	37349304	(T/C)
6947	CakSNP6947	*Kabuli*	Ca_Kabuli_Chr04	37349331	(C/T)
6948	CakSNP6948	*Kabuli*	Ca_Kabuli_Chr04	37349321	(T/C)
6949	CakSNP6949	*Kabuli*	Ca_Kabuli_Chr04	37356865	(C/A)
6950	CakSNP6950	*Kabuli*	Ca_Kabuli_Chr04	37356889	(C/T)
6951	CakSNP6951	*Kabuli*	Ca_Kabuli_Chr04	37356892	(C/T)
6952	CakSNP6952	*Kabuli*	Ca_Kabuli_Chr04	37356919	(A/T)
6953	CakSNP6953	*Kabuli*	Ca_Kabuli_Chr04	37356951	(C/T)

S.N.	SNP IDs	Cultivars	Chromosomes/scaffolds	Physical positions (bp)	SNPs
6954	CakSNP6954	*Kabuli*	Ca_Kabuli_Chr04	37364980	(G/A)
6955	CakSNP6955	*Kabuli*	Ca_Kabuli_Chr04	37365020	(C/T)
6956	CakSNP6956	*Kabuli*	Ca_Kabuli_Chr04	37371262	(G/A)
6957	CakSNP6957	*Kabuli*	Ca_Kabuli_Chr04	37371263	(G/A)
6958	CakSNP6958	*Kabuli*	Ca_Kabuli_Chr04	37433459	(G/T)
6959	CakSNP6959	*Kabuli*	Ca_Kabuli_Chr04	37450206	(A/C)
6960	CakSNP6960	*Kabuli*	Ca_Kabuli_Chr04	37450326	(A/T)
6961	CakSNP6961	*Kabuli*	Ca_Kabuli_Chr04	37450308	(C/T)
6962	CakSNP6962	*Kabuli*	Ca_Kabuli_Chr04	37450294	(T/C)
6963	CakSNP6963	*Kabuli*	Ca_Kabuli_Chr04	37453497	(C/G)
6964	CakSNP6964	*Kabuli*	Ca_Kabuli_Chr04	37453449	(A/G)
6965	CakSNP6965	*Kabuli*	Ca_Kabuli_Chr04	37453590	(C/A)
6966	CakSNP6966	*Kabuli*	Ca_Kabuli_Chr04	37453586	(C/A)
6967	CakSNP6967	*Kabuli*	Ca_Kabuli_Chr04	37454296	(A/G)
6968	CakSNP6968	*Kabuli*	Ca_Kabuli_Chr04	37492422	(C/A)
6969	CakSNP6969	*Kabuli*	Ca_Kabuli_Chr04	37549883	(T/C)
6970	CakSNP6970	*Kabuli*	Ca_Kabuli_Chr04	37549871	(A/T)
6971	CakSNP6971	*Kabuli*	Ca_Kabuli_Chr04	37550122	(G/A)
6972	CakSNP6972	*Kabuli*	Ca_Kabuli_Chr04	37552973	(C/T)
6973	CakSNP6973	*Kabuli*	Ca_Kabuli_Chr04	37558880	(C/T)
6974	CakSNP6974	*Kabuli*	Ca_Kabuli_Chr04	37558881	(G/T)
6975	CakSNP6975	*Kabuli*	Ca_Kabuli_Chr04	37575636	(T/C)
6976	CakSNP6976	*Kabuli*	Ca_Kabuli_Chr04	37575670	(C/T)
6977	CakSNP6977	*Kabuli*	Ca_Kabuli_Chr04	37575709	(C/T)
6978	CakSNP6978	*Kabuli*	Ca_Kabuli_Chr04	37612154	(A/G)
6979	CakSNP6979	*Kabuli*	Ca_Kabuli_Chr04	37630404	(A/G)
6980	CakSNP6980	*Kabuli*	Ca_Kabuli_Chr04	37630445	(A/G)
6981	CakSNP6981	*Kabuli*	Ca_Kabuli_Chr04	37630527	(G/A)
6982	CakSNP6982	*Kabuli*	Ca_Kabuli_Chr04	37630520	(G/C)
6983	CakSNP6983	*Kabuli*	Ca_Kabuli_Chr04	37634624	(C/T)
6984	CakSNP6984	*Kabuli*	Ca_Kabuli_Chr04	37635300	(T/A)
6985	CakSNP6985	*Kabuli*	Ca_Kabuli_Chr04	37685824	(T/C)
6986	CakSNP6986	*Kabuli*	Ca_Kabuli_Chr04	37685842	(T/G)
6987	CakSNP6987	*Kabuli*	Ca_Kabuli_Chr04	37699176	(G/A)
6988	CakSNP6988	*Kabuli*	Ca_Kabuli_Chr04	37699141	(T/G)
6989	CakSNP6989	*Kabuli*	Ca_Kabuli_Chr04	37699140	(C/T)
6990	CakSNP6990	*Kabuli*	Ca_Kabuli_Chr04	37703656	(G/T)
6991	CakSNP6991	*Kabuli*	Ca_Kabuli_Chr04	37703653	(A/C)

S.N.	SNP IDs	Cultivars	Chromosomes/scaffolds	Physical positions (bp)	SNPs
6992	CakSNP6992	*Kabuli*	Ca_Kabuli_Chr04	37703651	(T/C)
6993	CakSNP6993	*Kabuli*	Ca_Kabuli_Chr04	37703649	(A/C)
6994	CakSNP6994	*Kabuli*	Ca_Kabuli_Chr04	37703648	(C/T)
6995	CakSNP6995	*Kabuli*	Ca_Kabuli_Chr04	37707115	(G/A)
6996	CakSNP6996	*Kabuli*	Ca_Kabuli_Chr04	37708260	(A/G)
6997	CakSNP6997	*Kabuli*	Ca_Kabuli_Chr04	37708348	(T/G)
6998	CakSNP6998	*Kabuli*	Ca_Kabuli_Chr04	37708334	(T/A)
6999	CakSNP6999	*Kabuli*	Ca_Kabuli_Chr04	37753479	(C/T)
7000	CakSNP7000	*Kabuli*	Ca_Kabuli_Chr04	37806689	(T/C)
7001	CakSNP7001	*Kabuli*	Ca_Kabuli_Chr04	37812917	(G/C)
7002	CakSNP7002	*Kabuli*	Ca_Kabuli_Chr04	37859069	(T/G)
7003	CakSNP7003	*Kabuli*	Ca_Kabuli_Chr04	37897261	(G/T)
7004	CakSNP7004	*Kabuli*	Ca_Kabuli_Chr04	37897266	(A/T)
7005	CakSNP7005	*Kabuli*	Ca_Kabuli_Chr04	37897351	(C/A)
7006	CakSNP7006	*Kabuli*	Ca_Kabuli_Chr04	37898404	(A/G)
7007	CakSNP7007	*Kabuli*	Ca_Kabuli_Chr04	37899597	(C/T)
7008	CakSNP7008	*Kabuli*	Ca_Kabuli_Chr04	37901584	(C/T)
7009	CakSNP7009	*Kabuli*	Ca_Kabuli_Chr04	37901575	(G/A)
7010	CakSNP7010	*Kabuli*	Ca_Kabuli_Chr04	38008062	(T/A)
7011	CakSNP7011	*Kabuli*	Ca_Kabuli_Chr04	38008082	(T/C)
7012	CakSNP7012	*Kabuli*	Ca_Kabuli_Chr04	38008243	(C/T)
7013	CakSNP7013	*Kabuli*	Ca_Kabuli_Chr04	38008227	(G/T)
7014	CakSNP7014	*Kabuli*	Ca_Kabuli_Chr04	38008213	(C/T)
7015	CakSNP7015	*Kabuli*	Ca_Kabuli_Chr04	38008205	(A/G)
7016	CakSNP7016	*Kabuli*	Ca_Kabuli_Chr04	38008204	(G/A)
7017	CakSNP7017	*Kabuli*	Ca_Kabuli_Chr04	38008186	(A/C)
7018	CakSNP7018	*Kabuli*	Ca_Kabuli_Chr04	38008174	(A/G)
7019	CakSNP7019	*Kabuli*	Ca_Kabuli_Chr04	38027560	(G/A)
7020	CakSNP7020	*Kabuli*	Ca_Kabuli_Chr04	38029012	(T/C)
7021	CakSNP7021	*Kabuli*	Ca_Kabuli_Chr04	38029050	(G/A)
7022	CakSNP7022	*Kabuli*	Ca_Kabuli_Chr04	38029136	(G/T)
7023	CakSNP7023	*Kabuli*	Ca_Kabuli_Chr04	38038865	(C/T)
7024	CakSNP7024	*Kabuli*	Ca_Kabuli_Chr04	38038959	(A/G)
7025	CakSNP7025	*Kabuli*	Ca_Kabuli_Chr04	38039037	(T/C)
7026	CakSNP7026	*Kabuli*	Ca_Kabuli_Chr04	38039046	(C/T)
7027	CakSNP7027	*Kabuli*	Ca_Kabuli_Chr04	38039076	(A/G)
7028	CakSNP7028	*Kabuli*	Ca_Kabuli_Chr04	38039088	(T/C)
7029	CakSNP7029	*Kabuli*	Ca_Kabuli_Chr04	38039251	(G/C)

S.N.	SNP IDs	Cultivars	Chromosomes/scaffolds	Physical positions (bp)	SNPs
7030	CakSNP7030	*Kabuli*	Ca_Kabuli_Chr04	38039289	(G/A)
7031	CakSNP7031	*Kabuli*	Ca_Kabuli_Chr04	38039297	(C/T)
7032	CakSNP7032	*Kabuli*	Ca_Kabuli_Chr04	38039313	(G/A)
7033	CakSNP7033	*Kabuli*	Ca_Kabuli_Chr04	38039371	(T/C)
7034	CakSNP7034	*Kabuli*	Ca_Kabuli_Chr04	38153183	(C/A)
7035	CakSNP7035	*Kabuli*	Ca_Kabuli_Chr04	38153195	(T/C)
7036	CakSNP7036	*Kabuli*	Ca_Kabuli_Chr04	38153216	(T/C)
7037	CakSNP7037	*Kabuli*	Ca_Kabuli_Chr04	38153277	(A/G)
7038	CakSNP7038	*Kabuli*	Ca_Kabuli_Chr04	38153275	(C/A)
7039	CakSNP7039	*Kabuli*	Ca_Kabuli_Chr04	38153269	(A/C)
7040	CakSNP7040	*Kabuli*	Ca_Kabuli_Chr04	38179583	(A/C)
7041	CakSNP7041	*Kabuli*	Ca_Kabuli_Chr04	38179614	(G/C)
7042	CakSNP7042	*Kabuli*	Ca_Kabuli_Chr04	38181292	(G/A)
7043	CakSNP7043	*Kabuli*	Ca_Kabuli_Chr04	38195411	(T/A)
7044	CakSNP7044	*Kabuli*	Ca_Kabuli_Chr04	38195438	(C/T)
7045	CakSNP7045	*Kabuli*	Ca_Kabuli_Chr04	38215267	(G/A)
7046	CakSNP7046	*Kabuli*	Ca_Kabuli_Chr04	38221009	(A/G)
7047	CakSNP7047	*Kabuli*	Ca_Kabuli_Chr04	38288823	(G/T)
7048	CakSNP7048	*Kabuli*	Ca_Kabuli_Chr04	38288877	(C/G)
7049	CakSNP7049	*Kabuli*	Ca_Kabuli_Chr04	38289066	(G/A)
7050	CakSNP7050	*Kabuli*	Ca_Kabuli_Chr04	38289052	(G/A)
7051	CakSNP7051	*Kabuli*	Ca_Kabuli_Chr04	38288997	(T/A)
7052	CakSNP7052	*Kabuli*	Ca_Kabuli_Chr04	38343633	(A/T)
7053	CakSNP7053	*Kabuli*	Ca_Kabuli_Chr04	38343750	(C/T)
7054	CakSNP7054	*Kabuli*	Ca_Kabuli_Chr04	38343687	(C/T)
7055	CakSNP7055	*Kabuli*	Ca_Kabuli_Chr04	38358067	(A/G)
7056	CakSNP7056	*Kabuli*	Ca_Kabuli_Chr04	38358398	(A/G)
7057	CakSNP7057	*Kabuli*	Ca_Kabuli_Chr04	38358677	(G/A)
7058	CakSNP7058	*Kabuli*	Ca_Kabuli_Chr04	38358887	(G/A)
7059	CakSNP7059	*Kabuli*	Ca_Kabuli_Chr04	38358869	(G/A)
7060	CakSNP7060	*Kabuli*	Ca_Kabuli_Chr04	38362132	(T/A)
7061	CakSNP7061	*Kabuli*	Ca_Kabuli_Chr04	38362139	(T/A)
7062	CakSNP7062	*Kabuli*	Ca_Kabuli_Chr04	38364369	(C/T)
7063	CakSNP7063	*Kabuli*	Ca_Kabuli_Chr04	38370848	(A/G)
7064	CakSNP7064	*Kabuli*	Ca_Kabuli_Chr04	38370864	(G/A)
7065	CakSNP7065	*Kabuli*	Ca_Kabuli_Chr04	38370941	(A/G)
7066	CakSNP7066	*Kabuli*	Ca_Kabuli_Chr04	38370930	(C/T)
7067	CakSNP7067	*Kabuli*	Ca_Kabuli_Chr04	38370926	(T/C)

**S.N.**	**SNP IDs**	**Cultivars**	**Chromosomes/scaffolds**	**Physical positions (bp)**	**SNPs**
7068	CakSNP7068	*Kabuli*	Ca_Kabuli_Chr04	38370925	(G/A)
7069	CakSNP7069	*Kabuli*	Ca_Kabuli_Chr04	38370922	(G/A)
7070	CakSNP7070	*Kabuli*	Ca_Kabuli_Chr04	38370921	(T/G)
7071	CakSNP7071	*Kabuli*	Ca_Kabuli_Chr04	38382809	(T/C)
7072	CakSNP7072	*Kabuli*	Ca_Kabuli_Chr04	38382890	(C/G)
7073	CakSNP7073	*Kabuli*	Ca_Kabuli_Chr04	38421798	(A/C)
7074	CakSNP7074	*Kabuli*	Ca_Kabuli_Chr04	38422037	(C/T)
7075	CakSNP7075	*Kabuli*	Ca_Kabuli_Chr04	38421984	(C/T)
7076	CakSNP7076	*Kabuli*	Ca_Kabuli_Chr04	38425675	(A/G)
7077	CakSNP7077	*Kabuli*	Ca_Kabuli_Chr04	38425720	(T/A)
7078	CakSNP7078	*Kabuli*	Ca_Kabuli_Chr04	38473540	(A/G)
7079	CakSNP7079	*Kabuli*	Ca_Kabuli_Chr04	38473653	(T/C)
7080	CakSNP7080	*Kabuli*	Ca_Kabuli_Chr04	38474123	(A/T)
7081	CakSNP7081	*Kabuli*	Ca_Kabuli_Chr04	38474108	(T/C)
7082	CakSNP7082	*Kabuli*	Ca_Kabuli_Chr04	38474192	(T/C)
7083	CakSNP7083	*Kabuli*	Ca_Kabuli_Chr04	38475310	(A/C)
7084	CakSNP7084	*Kabuli*	Ca_Kabuli_Chr04	38475425	(A/G)
7085	CakSNP7085	*Kabuli*	Ca_Kabuli_Chr04	38475449	(T/A)
7086	CakSNP7086	*Kabuli*	Ca_Kabuli_Chr04	38475442	(A/G)
7087	CakSNP7087	*Kabuli*	Ca_Kabuli_Chr04	38475424	(C/G)
7088	CakSNP7088	*Kabuli*	Ca_Kabuli_Chr04	38475547	(C/T)
7089	CakSNP7089	*Kabuli*	Ca_Kabuli_Chr04	38540201	(C/T)
7090	CakSNP7090	*Kabuli*	Ca_Kabuli_Chr04	38540236	(C/T)
7091	CakSNP7091	*Kabuli*	Ca_Kabuli_Chr04	38540247	(G/A)
7092	CakSNP7092	*Kabuli*	Ca_Kabuli_Chr04	38620579	(C/T)
7093	CakSNP7093	*Kabuli*	Ca_Kabuli_Chr04	38620620	(A/T)
7094	CakSNP7094	*Kabuli*	Ca_Kabuli_Chr04	38659377	(T/C)
7095	CakSNP7095	*Kabuli*	Ca_Kabuli_Chr04	38659398	(G/A)
7096	CakSNP7096	*Kabuli*	Ca_Kabuli_Chr04	38659534	(T/A)
7097	CakSNP7097	*Kabuli*	Ca_Kabuli_Chr04	38679987	(A/G)
7098	CakSNP7098	*Kabuli*	Ca_Kabuli_Chr04	38721154	(A/G)
7099	CakSNP7099	*Kabuli*	Ca_Kabuli_Chr04	38721160	(A/G)
7100	CakSNP7100	*Kabuli*	Ca_Kabuli_Chr04	38721207	(A/C)
7101	CakSNP7101	*Kabuli*	Ca_Kabuli_Chr04	38756507	(T/C)
7102	CakSNP7102	*Kabuli*	Ca_Kabuli_Chr04	38756510	(C/G)
7103	CakSNP7103	*Kabuli*	Ca_Kabuli_Chr04	38759640	(A/C)
7104	CakSNP7104	*Kabuli*	Ca_Kabuli_Chr04	38770348	(T/A)
7105	CakSNP7105	*Kabuli*	Ca_Kabuli_Chr04	38770370	(G/C)

S.N.	SNP IDs	Cultivars	Chromosomes/scaffolds	Physical positions (bp)	SNPs
7106	CakSNP7106	*Kabuli*	Ca_Kabuli_Chr04	38770450	(C/G)
7107	CakSNP7107	*Kabuli*	Ca_Kabuli_Chr04	38770510	(G/A)
7108	CakSNP7108	*Kabuli*	Ca_Kabuli_Chr04	38770606	(G/A)
7109	CakSNP7109	*Kabuli*	Ca_Kabuli_Chr04	38770582	(G/A)
7110	CakSNP7110	*Kabuli*	Ca_Kabuli_Chr04	38785535	(G/A)
7111	CakSNP7111	*Kabuli*	Ca_Kabuli_Chr04	38829959	(T/G)
7112	CakSNP7112	*Kabuli*	Ca_Kabuli_Chr04	38833304	(C/A)
7113	CakSNP7113	*Kabuli*	Ca_Kabuli_Chr04	38833280	(C/T)
7114	CakSNP7114	*Kabuli*	Ca_Kabuli_Chr04	38833333	(A/C)
7115	CakSNP7115	*Kabuli*	Ca_Kabuli_Chr04	38833414	(A/C)
7116	CakSNP7116	*Kabuli*	Ca_Kabuli_Chr04	38834383	(G/A)
7117	CakSNP7117	*Kabuli*	Ca_Kabuli_Chr04	38834405	(G/A)
7118	CakSNP7118	*Kabuli*	Ca_Kabuli_Chr04	38834423	(G/C)
7119	CakSNP7119	*Kabuli*	Ca_Kabuli_Chr04	38834431	(G/A)
7120	CakSNP7120	*Kabuli*	Ca_Kabuli_Chr04	38834448	(A/T)
7121	CakSNP7121	*Kabuli*	Ca_Kabuli_Chr04	38834477	(A/G)
7122	CakSNP7122	*Kabuli*	Ca_Kabuli_Chr04	38834474	(G/A)
7123	CakSNP7123	*Kabuli*	Ca_Kabuli_Chr04	38834453	(C/T)
7124	CakSNP7124	*Kabuli*	Ca_Kabuli_Chr04	38888779	(C/T)
7125	CakSNP7125	*Kabuli*	Ca_Kabuli_Chr04	38888791	(G/C)
7126	CakSNP7126	*Kabuli*	Ca_Kabuli_Chr04	38889023	(C/T)
7127	CakSNP7127	*Kabuli*	Ca_Kabuli_Chr04	38918394	(A/T)
7128	CakSNP7128	*Kabuli*	Ca_Kabuli_Chr04	38918424	(G/A)
7129	CakSNP7129	*Kabuli*	Ca_Kabuli_Chr04	38918430	(C/A)
7130	CakSNP7130	*Kabuli*	Ca_Kabuli_Chr04	38918628	(C/T)
7131	CakSNP7131	*Kabuli*	Ca_Kabuli_Chr04	38918547	(T/C)
7132	CakSNP7132	*Kabuli*	Ca_Kabuli_Chr04	38918649	(G/C)
7133	CakSNP7133	*Kabuli*	Ca_Kabuli_Chr04	38929681	(A/G)
7134	CakSNP7134	*Kabuli*	Ca_Kabuli_Chr04	38948414	(C/A)
7135	CakSNP7135	*Kabuli*	Ca_Kabuli_Chr04	38961521	(G/A)
7136	CakSNP7136	*Kabuli*	Ca_Kabuli_Chr04	38978462	(A/G)
7137	CakSNP7137	*Kabuli*	Ca_Kabuli_Chr04	39031801	(C/T)
7138	CakSNP7138	*Kabuli*	Ca_Kabuli_Chr04	39031823	(G/A)
7139	CakSNP7139	*Kabuli*	Ca_Kabuli_Chr04	39042938	(T/G)
7140	CakSNP7140	*Kabuli*	Ca_Kabuli_Chr04	39042921	(T/C)
7141	CakSNP7141	*Kabuli*	Ca_Kabuli_Chr04	39057991	(T/C)
7142	CakSNP7142	*Kabuli*	Ca_Kabuli_Chr04	39058011	(G/C)
7143	CakSNP7143	*Kabuli*	Ca_Kabuli_Chr04	39081951	(A/G)

S.N.	SNP IDs	Cultivars	Chromosomes/scaffolds	Physical positions (bp)	SNPs
7144	CakSNP7144	*Kabuli*	Ca_Kabuli_Chr04	39110408	(T/C)
7145	CakSNP7145	*Kabuli*	Ca_Kabuli_Chr04	39110448	(A/G)
7146	CakSNP7146	*Kabuli*	Ca_Kabuli_Chr04	39110735	(A/G)
7147	CakSNP7147	*Kabuli*	Ca_Kabuli_Chr04	39113309	(T/C)
7148	CakSNP7148	*Kabuli*	Ca_Kabuli_Chr04	39113327	(G/A)
7149	CakSNP7149	*Kabuli*	Ca_Kabuli_Chr04	39113330	(G/A)
7150	CakSNP7150	*Kabuli*	Ca_Kabuli_Chr04	39113401	(A/T)
7151	CakSNP7151	*Kabuli*	Ca_Kabuli_Chr04	39114000	(A/C)
7152	CakSNP7152	*Kabuli*	Ca_Kabuli_Chr04	39136826	(C/T)
7153	CakSNP7153	*Kabuli*	Ca_Kabuli_Chr04	39189012	(A/G)
7154	CakSNP7154	*Kabuli*	Ca_Kabuli_Chr04	39189016	(G/A)
7155	CakSNP7155	*Kabuli*	Ca_Kabuli_Chr04	39268505	(T/C)
7156	CakSNP7156	*Kabuli*	Ca_Kabuli_Chr04	39306900	(G/A)
7157	CakSNP7157	*Kabuli*	Ca_Kabuli_Chr04	39635746	(T/A)
7158	CakSNP7158	*Kabuli*	Ca_Kabuli_Chr04	39635721	(A/G)
7159	CakSNP7159	*Kabuli*	Ca_Kabuli_Chr04	39635720	(G/A)
7160	CakSNP7160	*Kabuli*	Ca_Kabuli_Chr04	39635718	(C/T)
7161	CakSNP7161	*Kabuli*	Ca_Kabuli_Chr04	39650872	(T/C)
7162	CakSNP7162	*Kabuli*	Ca_Kabuli_Chr04	39659757	(C/T)
7163	CakSNP7163	*Kabuli*	Ca_Kabuli_Chr04	39659750	(G/A)
7164	CakSNP7164	*Kabuli*	Ca_Kabuli_Chr04	39659832	(C/T)
7165	CakSNP7165	*Kabuli*	Ca_Kabuli_Chr04	39715087	(A/G)
7166	CakSNP7166	*Kabuli*	Ca_Kabuli_Chr04	39719606	(C/G)
7167	CakSNP7167	*Kabuli*	Ca_Kabuli_Chr04	39719747	(G/T)
7168	CakSNP7168	*Kabuli*	Ca_Kabuli_Chr04	39779796	(G/A)
7169	CakSNP7169	*Kabuli*	Ca_Kabuli_Chr04	39803305	(T/C)
7170	CakSNP7170	*Kabuli*	Ca_Kabuli_Chr04	39849612	(G/T)
7171	CakSNP7171	*Kabuli*	Ca_Kabuli_Chr04	39849657	(G/A)
7172	CakSNP7172	*Kabuli*	Ca_Kabuli_Chr04	39964930	(T/C)
7173	CakSNP7173	*Kabuli*	Ca_Kabuli_Chr04	40010119	(A/C)
7174	CakSNP7174	*Kabuli*	Ca_Kabuli_Chr04	40010201	(A/G)
7175	CakSNP7175	*Kabuli*	Ca_Kabuli_Chr04	40010177	(G/A)
7176	CakSNP7176	*Kabuli*	Ca_Kabuli_Chr04	40010165	(A/C)
7177	CakSNP7177	*Kabuli*	Ca_Kabuli_Chr04	40059880	(C/T)
7178	CakSNP7178	*Kabuli*	Ca_Kabuli_Chr04	40060022	(C/T)
7179	CakSNP7179	*Kabuli*	Ca_Kabuli_Chr04	40060063	(T/G)
7180	CakSNP7180	*Kabuli*	Ca_Kabuli_Chr04	40061195	(T/C)
7181	CakSNP7181	*Kabuli*	Ca_Kabuli_Chr04	40061262	(A/C)

S.N.	SNP IDs	Cultivars	Chromosomes/scaffolds	Physical positions (bp)	SNPs
7182	CakSNP7182	*Kabuli*	Ca_Kabuli_Chr04	40065505	(T/A)
7183	CakSNP7183	*Kabuli*	Ca_Kabuli_Chr04	40134460	(A/G)
7184	CakSNP7184	*Kabuli*	Ca_Kabuli_Chr04	40134518	(T/C)
7185	CakSNP7185	*Kabuli*	Ca_Kabuli_Chr04	40149064	(C/T)
7186	CakSNP7186	*Kabuli*	Ca_Kabuli_Chr04	40149132	(G/C)
7187	CakSNP7187	*Kabuli*	Ca_Kabuli_Chr04	40149324	(G/C)
7188	CakSNP7188	*Kabuli*	Ca_Kabuli_Chr04	40169176	(T/G)
7189	CakSNP7189	*Kabuli*	Ca_Kabuli_Chr04	40185606	(A/G)
7190	CakSNP7190	*Kabuli*	Ca_Kabuli_Chr04	40199692	(A/C)
7191	CakSNP7191	*Kabuli*	Ca_Kabuli_Chr04	40218375	(G/T)
7192	CakSNP7192	*Kabuli*	Ca_Kabuli_Chr04	40218410	(G/A)
7193	CakSNP7193	*Kabuli*	Ca_Kabuli_Chr04	40267713	(G/T)
7194	CakSNP7194	*Kabuli*	Ca_Kabuli_Chr04	40267719	(G/A)
7195	CakSNP7195	*Kabuli*	Ca_Kabuli_Chr04	40279513	(G/T)
7196	CakSNP7196	*Kabuli*	Ca_Kabuli_Chr04	40313721	(A/G)
7197	CakSNP7197	*Kabuli*	Ca_Kabuli_Chr04	40349170	(C/T)
7198	CakSNP7198	*Kabuli*	Ca_Kabuli_Chr04	40368028	(C/T)
7199	CakSNP7199	*Kabuli*	Ca_Kabuli_Chr04	40368278	(T/C)
7200	CakSNP7200	*Kabuli*	Ca_Kabuli_Chr04	40368765	(G/A)
7201	CakSNP7201	*Kabuli*	Ca_Kabuli_Chr04	40375386	(C/T)
7202	CakSNP7202	*Kabuli*	Ca_Kabuli_Chr04	40375361	(A/G)
7203	CakSNP7203	*Kabuli*	Ca_Kabuli_Chr04	40429387	(G/A)
7204	CakSNP7204	*Kabuli*	Ca_Kabuli_Chr04	40471412	(G/C)
7205	CakSNP7205	*Kabuli*	Ca_Kabuli_Chr04	40472097	(A/G)
7206	CakSNP7206	*Kabuli*	Ca_Kabuli_Chr04	40513733	(C/T)
7207	CakSNP7207	*Kabuli*	Ca_Kabuli_Chr04	40583205	(G/T)
7208	CakSNP7208	*Kabuli*	Ca_Kabuli_Chr04	40583206	(A/T)
7209	CakSNP7209	*Kabuli*	Ca_Kabuli_Chr04	40583211	(G/T)
7210	CakSNP7210	*Kabuli*	Ca_Kabuli_Chr04	40583214	(G/A)
7211	CakSNP7211	*Kabuli*	Ca_Kabuli_Chr04	40583216	(C/A)
7212	CakSNP7212	*Kabuli*	Ca_Kabuli_Chr04	40583217	(G/A)
7213	CakSNP7213	*Kabuli*	Ca_Kabuli_Chr04	40583219	(G/A)
7214	CakSNP7214	*Kabuli*	Ca_Kabuli_Chr04	40597192	(G/A)
7215	CakSNP7215	*Kabuli*	Ca_Kabuli_Chr04	40597534	(A/G)
7216	CakSNP7216	*Kabuli*	Ca_Kabuli_Chr04	40597464	(G/A)
7217	CakSNP7217	*Kabuli*	Ca_Kabuli_Chr04	40723208	(C/G)
7218	CakSNP7218	*Kabuli*	Ca_Kabuli_Chr04	40723212	(G/A)
7219	CakSNP7219	*Kabuli*	Ca_Kabuli_Chr04	40734572	(T/G)

S.N.	SNP IDs	Cultivars	Chromosomes/scaffolds	Physical positions (bp)	SNPs
7220	CakSNP7220	*Kabuli*	Ca_Kabuli_Chr04	40734765	(G/A)
7221	CakSNP7221	*Kabuli*	Ca_Kabuli_Chr04	40738927	(C/T)
7222	CakSNP7222	*Kabuli*	Ca_Kabuli_Chr04	40795060	(G/A)
7223	CakSNP7223	*Kabuli*	Ca_Kabuli_Chr04	40795085	(C/G)
7224	CakSNP7224	*Kabuli*	Ca_Kabuli_Chr04	40795137	(T/G)
7225	CakSNP7225	*Kabuli*	Ca_Kabuli_Chr04	40801783	(T/C)
7226	CakSNP7226	*Kabuli*	Ca_Kabuli_Chr04	40801791	(C/G)
7227	CakSNP7227	*Kabuli*	Ca_Kabuli_Chr04	40805740	(C/A)
7228	CakSNP7228	*Kabuli*	Ca_Kabuli_Chr04	40806993	(A/G)
7229	CakSNP7229	*Kabuli*	Ca_Kabuli_Chr04	40839967	(G/A)
7230	CakSNP7230	*Kabuli*	Ca_Kabuli_Chr04	40839949	(G/A)
7231	CakSNP7231	*Kabuli*	Ca_Kabuli_Chr04	40852381	(A/C)
7232	CakSNP7232	*Kabuli*	Ca_Kabuli_Chr04	40852475	(T/C)
7233	CakSNP7233	*Kabuli*	Ca_Kabuli_Chr04	40912677	(A/G)
7234	CakSNP7234	*Kabuli*	Ca_Kabuli_Chr04	40912681	(A/G)
7235	CakSNP7235	*Kabuli*	Ca_Kabuli_Chr04	40912714	(C/G)
7236	CakSNP7236	*Kabuli*	Ca_Kabuli_Chr04	40912731	(C/T)
7237	CakSNP7237	*Kabuli*	Ca_Kabuli_Chr04	40917776	(C/A)
7238	CakSNP7238	*Kabuli*	Ca_Kabuli_Chr04	40917777	(T/A)
7239	CakSNP7239	*Kabuli*	Ca_Kabuli_Chr04	40917807	(C/T)
7240	CakSNP7240	*Kabuli*	Ca_Kabuli_Chr04	40941283	(A/G)
7241	CakSNP7241	*Kabuli*	Ca_Kabuli_Chr04	41032564	(A/G)
7242	CakSNP7242	*Kabuli*	Ca_Kabuli_Chr04	41032590	(C/A)
7243	CakSNP7243	*Kabuli*	Ca_Kabuli_Chr04	41032599	(T/C)
7244	CakSNP7244	*Kabuli*	Ca_Kabuli_Chr04	41032608	(T/C)
7245	CakSNP7245	*Kabuli*	Ca_Kabuli_Chr04	41070048	(C/G)
7246	CakSNP7246	*Kabuli*	Ca_Kabuli_Chr04	41070017	(T/A)
7247	CakSNP7247	*Kabuli*	Ca_Kabuli_Chr04	41094082	(T/C)
7248	CakSNP7248	*Kabuli*	Ca_Kabuli_Chr04	41094554	(A/C)
7249	CakSNP7249	*Kabuli*	Ca_Kabuli_Chr04	41094685	(G/C)
7250	CakSNP7250	*Kabuli*	Ca_Kabuli_Chr04	41117822	(C/A)
7251	CakSNP7251	*Kabuli*	Ca_Kabuli_Chr04	41141086	(G/T)
7252	CakSNP7252	*Kabuli*	Ca_Kabuli_Chr04	41142299	(T/C)
7253	CakSNP7253	*Kabuli*	Ca_Kabuli_Chr04	41142290	(A/G)
7254	CakSNP7254	*Kabuli*	Ca_Kabuli_Chr04	41142288	(T/C)
7255	CakSNP7255	*Kabuli*	Ca_Kabuli_Chr04	41144277	(T/A)
7256	CakSNP7256	*Kabuli*	Ca_Kabuli_Chr04	41145767	(C/T)
7257	CakSNP7257	*Kabuli*	Ca_Kabuli_Chr04	41145875	(G/A)

S.N.	SNP IDs	Cultivars	Chromosomes/scaffolds	Physical positions (bp)	SNPs
7258	CakSNP7258	*Kabuli*	Ca_Kabuli_Chr04	41207328	(C/T)
7259	CakSNP7259	*Kabuli*	Ca_Kabuli_Chr04	41207356	(C/T)
7260	CakSNP7260	*Kabuli*	Ca_Kabuli_Chr04	41207392	(T/G)
7261	CakSNP7261	*Kabuli*	Ca_Kabuli_Chr04	41207354	(A/C)
7262	CakSNP7262	*Kabuli*	Ca_Kabuli_Chr04	41209521	(T/G)
7263	CakSNP7263	*Kabuli*	Ca_Kabuli_Chr04	41209711	(C/A)
7264	CakSNP7264	*Kabuli*	Ca_Kabuli_Chr04	41215850	(A/C)
7265	CakSNP7265	*Kabuli*	Ca_Kabuli_Chr04	41228918	(T/C)
7266	CakSNP7266	*Kabuli*	Ca_Kabuli_Chr04	41229605	(C/T)
7267	CakSNP7267	*Kabuli*	Ca_Kabuli_Chr04	41277786	(G/A)
7268	CakSNP7268	*Kabuli*	Ca_Kabuli_Chr04	41277799	(T/G)
7269	CakSNP7269	*Kabuli*	Ca_Kabuli_Chr04	41292152	(T/C)
7270	CakSNP7270	*Kabuli*	Ca_Kabuli_Chr04	41319598	(T/C)
7271	CakSNP7271	*Kabuli*	Ca_Kabuli_Chr04	41319610	(G/A)
7272	CakSNP7272	*Kabuli*	Ca_Kabuli_Chr04	41344739	(A/T)
7273	CakSNP7273	*Kabuli*	Ca_Kabuli_Chr04	41427081	(A/T)
7274	CakSNP7274	*Kabuli*	Ca_Kabuli_Chr04	41427086	(C/A)
7275	CakSNP7275	*Kabuli*	Ca_Kabuli_Chr04	41427093	(T/G)
7276	CakSNP7276	*Kabuli*	Ca_Kabuli_Chr04	41445171	(T/C)
7277	CakSNP7277	*Kabuli*	Ca_Kabuli_Chr04	41445212	(A/G)
7278	CakSNP7278	*Kabuli*	Ca_Kabuli_Chr04	41445225	(A/T)
7279	CakSNP7279	*Kabuli*	Ca_Kabuli_Chr04	41445242	(C/T)
7280	CakSNP7280	*Kabuli*	Ca_Kabuli_Chr04	41458426	(A/G)
7281	CakSNP7281	*Kabuli*	Ca_Kabuli_Chr04	41500284	(A/C)
7282	CakSNP7282	*Kabuli*	Ca_Kabuli_Chr04	41500286	(A/G)
7283	CakSNP7283	*Kabuli*	Ca_Kabuli_Chr04	41512840	(G/A)
7284	CakSNP7284	*Kabuli*	Ca_Kabuli_Chr04	41568682	(A/C)
7285	CakSNP7285	*Kabuli*	Ca_Kabuli_Chr04	41629852	(T/A)
7286	CakSNP7286	*Kabuli*	Ca_Kabuli_Chr04	41648927	(C/T)
7287	CakSNP7287	*Kabuli*	Ca_Kabuli_Chr04	41648998	(A/G)
7288	CakSNP7288	*Kabuli*	Ca_Kabuli_Chr04	41675299	(A/T)
7289	CakSNP7289	*Kabuli*	Ca_Kabuli_Chr04	41698291	(A/G)
7290	CakSNP7290	*Kabuli*	Ca_Kabuli_Chr04	41698386	(A/G)
7291	CakSNP7291	*Kabuli*	Ca_Kabuli_Chr04	41700712	(C/A)
7292	CakSNP7292	*Kabuli*	Ca_Kabuli_Chr04	41721289	(G/A)
7293	CakSNP7293	*Kabuli*	Ca_Kabuli_Chr04	41772690	(C/T)
7294	CakSNP7294	*Kabuli*	Ca_Kabuli_Chr04	41772823	(G/A)
7295	CakSNP7295	*Kabuli*	Ca_Kabuli_Chr04	41772796	(T/A)

S.N.	SNP IDs	Cultivars	Chromosomes/scaffolds	Physical positions (bp)	SNPs
7296	CakSNP7296	*Kabuli*	Ca_Kabuli_Chr04	41772794	(T/C)
7297	CakSNP7297	*Kabuli*	Ca_Kabuli_Chr04	41864826	(G/A)
7298	CakSNP7298	*Kabuli*	Ca_Kabuli_Chr04	41864846	(C/G)
7299	CakSNP7299	*Kabuli*	Ca_Kabuli_Chr04	41938784	(A/G)
7300	CakSNP7300	*Kabuli*	Ca_Kabuli_Chr04	41938812	(T/G)
7301	CakSNP7301	*Kabuli*	Ca_Kabuli_Chr04	41956050	(C/G)
7302	CakSNP7302	*Kabuli*	Ca_Kabuli_Chr04	41956085	(T/C)
7303	CakSNP7303	*Kabuli*	Ca_Kabuli_Chr04	41987193	(A/G)
7304	CakSNP7304	*Kabuli*	Ca_Kabuli_Chr04	42056479	(G/A)
7305	CakSNP7305	*Kabuli*	Ca_Kabuli_Chr04	42092379	(T/G)
7306	CakSNP7306	*Kabuli*	Ca_Kabuli_Chr04	42092381	(A/G)
7307	CakSNP7307	*Kabuli*	Ca_Kabuli_Chr04	42092385	(G/A)
7308	CakSNP7308	*Kabuli*	Ca_Kabuli_Chr04	42105737	(C/T)
7309	CakSNP7309	*Kabuli*	Ca_Kabuli_Chr04	42105701	(A/T)
7310	CakSNP7310	*Kabuli*	Ca_Kabuli_Chr04	42108980	(C/A)
7311	CakSNP7311	*Kabuli*	Ca_Kabuli_Chr04	42246499	(C/T)
7312	CakSNP7312	*Kabuli*	Ca_Kabuli_Chr04	42246527	(C/A)
7313	CakSNP7313	*Kabuli*	Ca_Kabuli_Chr04	42246565	(T/G)
7314	CakSNP7314	*Kabuli*	Ca_Kabuli_Chr04	42246566	(T/G)
7315	CakSNP7315	*Kabuli*	Ca_Kabuli_Chr04	42246572	(T/G)
7316	CakSNP7316	*Kabuli*	Ca_Kabuli_Chr04	42246653	(C/G)
7317	CakSNP7317	*Kabuli*	Ca_Kabuli_Chr04	42273252	(G/A)
7318	CakSNP7318	*Kabuli*	Ca_Kabuli_Chr04	42353949	(C/T)
7319	CakSNP7319	*Kabuli*	Ca_Kabuli_Chr04	42499699	(A/T)
7320	CakSNP7320	*Kabuli*	Ca_Kabuli_Chr04	42603642	(T/C)
7321	CakSNP7321	*Kabuli*	Ca_Kabuli_Chr04	42768142	(T/G)
7322	CakSNP7322	*Kabuli*	Ca_Kabuli_Chr04	42807483	(G/A)
7323	CakSNP7323	*Kabuli*	Ca_Kabuli_Chr04	42807518	(A/G)
7324	CakSNP7324	*Kabuli*	Ca_Kabuli_Chr04	42807509	(C/T)
7325	CakSNP7325	*Kabuli*	Ca_Kabuli_Chr04	43319846	(A/G)
7326	CakSNP7326	*Kabuli*	Ca_Kabuli_Chr04	43324547	(G/C)
7327	CakSNP7327	*Kabuli*	Ca_Kabuli_Chr04	43431770	(C/T)
7328	CakSNP7328	*Kabuli*	Ca_Kabuli_Chr04	43431772	(T/A)
7329	CakSNP7329	*Kabuli*	Ca_Kabuli_Chr04	43431815	(G/T)
7330	CakSNP7330	*Kabuli*	Ca_Kabuli_Chr04	43446753	(T/A)
7331	CakSNP7331	*Kabuli*	Ca_Kabuli_Chr04	43447262	(A/T)
7332	CakSNP7332	*Kabuli*	Ca_Kabuli_Chr04	43476193	(T/C)
7333	CakSNP7333	*Kabuli*	Ca_Kabuli_Chr04	43653225	(A/G)

S.N.	SNP IDs	Cultivars	Chromosomes/scaffolds	Physical positions (bp)	SNPs
7334	CakSNP7334	*Kabuli*	Ca_Kabuli_Chr04	43653226	(C/T)
7335	CakSNP7335	*Kabuli*	Ca_Kabuli_Chr04	43662224	(A/C)
7336	CakSNP7336	*Kabuli*	Ca_Kabuli_Chr04	43677006	(T/G)
7337	CakSNP7337	*Kabuli*	Ca_Kabuli_Chr04	43677222	(C/T)
7338	CakSNP7338	*Kabuli*	Ca_Kabuli_Chr04	43722728	(T/C)
7339	CakSNP7339	*Kabuli*	Ca_Kabuli_Chr04	43722727	(A/G)
7340	CakSNP7340	*Kabuli*	Ca_Kabuli_Chr04	43722704	(C/G)
7341	CakSNP7341	*Kabuli*	Ca_Kabuli_Chr04	43723219	(T/G)
7342	CakSNP7342	*Kabuli*	Ca_Kabuli_Chr04	43723224	(C/A)
7343	CakSNP7343	*Kabuli*	Ca_Kabuli_Chr04	43723248	(T/C)
7344	CakSNP7344	*Kabuli*	Ca_Kabuli_Chr04	43726406	(C/T)
7345	CakSNP7345	*Kabuli*	Ca_Kabuli_Chr04	43729063	(G/T)
7346	CakSNP7346	*Kabuli*	Ca_Kabuli_Chr04	43729261	(C/G)
7347	CakSNP7347	*Kabuli*	Ca_Kabuli_Chr04	43729266	(A/G)
7348	CakSNP7348	*Kabuli*	Ca_Kabuli_Chr04	43807399	(C/T)
7349	CakSNP7349	*Kabuli*	Ca_Kabuli_Chr04	43807460	(C/T)
7350	CakSNP7350	*Kabuli*	Ca_Kabuli_Chr04	43840666	(C/G)
7351	CakSNP7351	*Kabuli*	Ca_Kabuli_Chr04	43840650	(C/T)
7352	CakSNP7352	*Kabuli*	Ca_Kabuli_Chr04	43979065	(A/G)
7353	CakSNP7353	*Kabuli*	Ca_Kabuli_Chr04	44008835	(T/C)
7354	CakSNP7354	*Kabuli*	Ca_Kabuli_Chr04	44059060	(C/T)
7355	CakSNP7355	*Kabuli*	Ca_Kabuli_Chr04	44059061	(G/A)
7356	CakSNP7356	*Kabuli*	Ca_Kabuli_Chr04	44067780	(G/A)
7357	CakSNP7357	*Kabuli*	Ca_Kabuli_Chr04	44090929	(T/G)
7358	CakSNP7358	*Kabuli*	Ca_Kabuli_Chr04	44186434	(C/G)
7359	CakSNP7359	*Kabuli*	Ca_Kabuli_Chr04	44239783	(A/C)
7360	CakSNP7360	*Kabuli*	Ca_Kabuli_Chr04	44239883	(A/T)
7361	CakSNP7361	*Kabuli*	Ca_Kabuli_Chr04	44250159	(C/T)
7362	CakSNP7362	*Kabuli*	Ca_Kabuli_Chr04	44261184	(A/C)
7363	CakSNP7363	*Kabuli*	Ca_Kabuli_Chr04	44261909	(T/A)
7364	CakSNP7364	*Kabuli*	Ca_Kabuli_Chr04	44266692	(T/A)
7365	CakSNP7365	*Kabuli*	Ca_Kabuli_Chr04	44266695	(G/A)
7366	CakSNP7366	*Kabuli*	Ca_Kabuli_Chr04	44266758	(C/T)
7367	CakSNP7367	*Kabuli*	Ca_Kabuli_Chr04	44266819	(G/A)
7368	CakSNP7368	*Kabuli*	Ca_Kabuli_Chr04	44353354	(C/T)
7369	CakSNP7369	*Kabuli*	Ca_Kabuli_Chr04	44353343	(G/A)
7370	CakSNP7370	*Kabuli*	Ca_Kabuli_Chr04	44375761	(T/C)
7371	CakSNP7371	*Kabuli*	Ca_Kabuli_Chr04	44375831	(T/C)

S.N.	SNP IDs	Cultivars	Chromosomes/scaffolds	Physical positions (bp)	SNPs
7372	CakSNP7372	*Kabuli*	Ca_Kabuli_Chr04	44382515	(A/G)
7373	CakSNP7373	*Kabuli*	Ca_Kabuli_Chr04	44382677	(C/T)
7374	CakSNP7374	*Kabuli*	Ca_Kabuli_Chr04	44382671	(C/G)
7375	CakSNP7375	*Kabuli*	Ca_Kabuli_Chr04	44382614	(C/A)
7376	CakSNP7376	*Kabuli*	Ca_Kabuli_Chr04	44382602	(A/G)
7377	CakSNP7377	*Kabuli*	Ca_Kabuli_Chr04	44386579	(A/G)
7378	CakSNP7378	*Kabuli*	Ca_Kabuli_Chr04	44431319	(A/C)
7379	CakSNP7379	*Kabuli*	Ca_Kabuli_Chr04	44431353	(G/A)
7380	CakSNP7380	*Kabuli*	Ca_Kabuli_Chr04	44439372	(C/T)
7381	CakSNP7381	*Kabuli*	Ca_Kabuli_Chr04	44439503	(C/A)
7382	CakSNP7382	*Kabuli*	Ca_Kabuli_Chr04	44439495	(G/A)
7383	CakSNP7383	*Kabuli*	Ca_Kabuli_Chr04	44439493	(A/G)
7384	CakSNP7384	*Kabuli*	Ca_Kabuli_Chr04	44439492	(G/T)
7385	CakSNP7385	*Kabuli*	Ca_Kabuli_Chr04	44439448	(T/C)
7386	CakSNP7386	*Kabuli*	Ca_Kabuli_Chr04	44446468	(G/A)
7387	CakSNP7387	*Kabuli*	Ca_Kabuli_Chr04	44446575	(G/A)
7388	CakSNP7388	*Kabuli*	Ca_Kabuli_Chr04	44446506	(C/T)
7389	CakSNP7389	*Kabuli*	Ca_Kabuli_Chr04	44446503	(T/G)
7390	CakSNP7390	*Kabuli*	Ca_Kabuli_Chr04	44446497	(C/T)
7391	CakSNP7391	*Kabuli*	Ca_Kabuli_Chr04	44472282	(G/A)
7392	CakSNP7392	*Kabuli*	Ca_Kabuli_Chr04	44472278	(A/G)
7393	CakSNP7393	*Kabuli*	Ca_Kabuli_Chr04	44472237	(T/A)
7394	CakSNP7394	*Kabuli*	Ca_Kabuli_Chr04	44590652	(T/C)
7395	CakSNP7395	*Kabuli*	Ca_Kabuli_Chr04	44590678	(G/A)
7396	CakSNP7396	*Kabuli*	Ca_Kabuli_Chr04	44590697	(A/C)
7397	CakSNP7397	*Kabuli*	Ca_Kabuli_Chr04	44591405	(G/T)
7398	CakSNP7398	*Kabuli*	Ca_Kabuli_Chr04	44754355	(C/T)
7399	CakSNP7399	*Kabuli*	Ca_Kabuli_Chr04	44802374	(G/A)
7400	CakSNP7400	*Kabuli*	Ca_Kabuli_Chr04	44802364	(A/C)
7401	CakSNP7401	*Kabuli*	Ca_Kabuli_Chr04	44803694	(T/A)
7402	CakSNP7402	*Kabuli*	Ca_Kabuli_Chr04	44811519	(G/A)
7403	CakSNP7403	*Kabuli*	Ca_Kabuli_Chr04	44814857	(A/G)
7404	CakSNP7404	*Kabuli*	Ca_Kabuli_Chr04	44814981	(T/C)
7405	CakSNP7405	*Kabuli*	Ca_Kabuli_Chr04	44814982	(A/G)
7406	CakSNP7406	*Kabuli*	Ca_Kabuli_Chr04	44824103	(G/T)
7407	CakSNP7407	*Kabuli*	Ca_Kabuli_Chr04	44824105	(T/G)
7408	CakSNP7408	*Kabuli*	Ca_Kabuli_Chr04	44824178	(C/G)
7409	CakSNP7409	*Kabuli*	Ca_Kabuli_Chr04	44824258	(A/G)

S.N.	SNP IDs	Cultivars	Chromosomes/scaffolds	Physical positions (bp)	SNPs
7410	CakSNP7410	*Kabuli*	Ca_Kabuli_Chr04	44832121	(G/C)
7411	CakSNP7411	*Kabuli*	Ca_Kabuli_Chr04	44832144	(T/C)
7412	CakSNP7412	*Kabuli*	Ca_Kabuli_Chr04	44906128	(C/A)
7413	CakSNP7413	*Kabuli*	Ca_Kabuli_Chr04	44906255	(A/G)
7414	CakSNP7414	*Kabuli*	Ca_Kabuli_Chr04	44979108	(G/A)
7415	CakSNP7415	*Kabuli*	Ca_Kabuli_Chr04	45019764	(T/C)
7416	CakSNP7416	*Kabuli*	Ca_Kabuli_Chr04	45019778	(T/C)
7417	CakSNP7417	*Kabuli*	Ca_Kabuli_Chr04	45019787	(G/C)
7418	CakSNP7418	*Kabuli*	Ca_Kabuli_Chr04	45019805	(C/A)
7419	CakSNP7419	*Kabuli*	Ca_Kabuli_Chr04	45019807	(C/T)
7420	CakSNP7420	*Kabuli*	Ca_Kabuli_Chr04	45019814	(C/A)
7421	CakSNP7421	*Kabuli*	Ca_Kabuli_Chr04	45019820	(C/T)
7422	CakSNP7422	*Kabuli*	Ca_Kabuli_Chr04	45019824	(A/C)
7423	CakSNP7423	*Kabuli*	Ca_Kabuli_Chr04	45019867	(C/G)
7424	CakSNP7424	*Kabuli*	Ca_Kabuli_Chr04	45019858	(G/T)
7425	CakSNP7425	*Kabuli*	Ca_Kabuli_Chr04	45019848	(C/T)
7426	CakSNP7426	*Kabuli*	Ca_Kabuli_Chr04	45035589	(G/T)
7427	CakSNP7427	*Kabuli*	Ca_Kabuli_Chr04	45035629	(A/C)
7428	CakSNP7428	*Kabuli*	Ca_Kabuli_Chr04	45035745	(A/G)
7429	CakSNP7429	*Kabuli*	Ca_Kabuli_Chr04	45067534	(C/T)
7430	CakSNP7430	*Kabuli*	Ca_Kabuli_Chr04	45104767	(G/A)
7431	CakSNP7431	*Kabuli*	Ca_Kabuli_Chr04	45104779	(C/A)
7432	CakSNP7432	*Kabuli*	Ca_Kabuli_Chr04	45104805	(A/G)
7433	CakSNP7433	*Kabuli*	Ca_Kabuli_Chr04	45141976	(C/A)
7434	CakSNP7434	*Kabuli*	Ca_Kabuli_Chr04	45141990	(A/G)
7435	CakSNP7435	*Kabuli*	Ca_Kabuli_Chr04	45142020	(T/C)
7436	CakSNP7436	*Kabuli*	Ca_Kabuli_Chr04	45142009	(C/T)
7437	CakSNP7437	*Kabuli*	Ca_Kabuli_Chr04	45142003	(A/G)
7438	CakSNP7438	*Kabuli*	Ca_Kabuli_Chr04	45142077	(C/A)
7439	CakSNP7439	*Kabuli*	Ca_Kabuli_Chr04	45142087	(T/A)
7440	CakSNP7440	*Kabuli*	Ca_Kabuli_Chr04	45142104	(T/C)
7441	CakSNP7441	*Kabuli*	Ca_Kabuli_Chr04	45165191	(T/C)
7442	CakSNP7442	*Kabuli*	Ca_Kabuli_Chr04	45165246	(C/G)
7443	CakSNP7443	*Kabuli*	Ca_Kabuli_Chr04	45165240	(T/C)
7444	CakSNP7444	*Kabuli*	Ca_Kabuli_Chr04	45165231	(A/C)
7445	CakSNP7445	*Kabuli*	Ca_Kabuli_Chr04	45206203	(G/T)
7446	CakSNP7446	*Kabuli*	Ca_Kabuli_Chr04	45206209	(G/C)
7447	CakSNP7447	*Kabuli*	Ca_Kabuli_Chr04	45220084	(G/T)

S.N.	SNP IDs	Cultivars	Chromosomes/scaffolds	Physical positions (bp)	SNPs
7448	CakSNP7448	*Kabuli*	Ca_Kabuli_Chr04	45220642	(C/T)
7449	CakSNP7449	*Kabuli*	Ca_Kabuli_Chr04	45220916	(T/C)
7450	CakSNP7450	*Kabuli*	Ca_Kabuli_Chr04	45225063	(T/C)
7451	CakSNP7451	*Kabuli*	Ca_Kabuli_Chr04	45225059	(G/A)
7452	CakSNP7452	*Kabuli*	Ca_Kabuli_Chr04	45224997	(G/C)
7453	CakSNP7453	*Kabuli*	Ca_Kabuli_Chr04	45225077	(A/T)
7454	CakSNP7454	*Kabuli*	Ca_Kabuli_Chr04	45225093	(G/A)
7455	CakSNP7455	*Kabuli*	Ca_Kabuli_Chr04	45225145	(T/A)
7456	CakSNP7456	*Kabuli*	Ca_Kabuli_Chr04	45225154	(T/A)
7457	CakSNP7457	*Kabuli*	Ca_Kabuli_Chr04	45225155	(C/A)
7458	CakSNP7458	*Kabuli*	Ca_Kabuli_Chr04	45225210	(G/T)
7459	CakSNP7459	*Kabuli*	Ca_Kabuli_Chr04	45225193	(A/G)
7460	CakSNP7460	*Kabuli*	Ca_Kabuli_Chr04	45225165	(T/C)
7461	CakSNP7461	*Kabuli*	Ca_Kabuli_Chr04	45231839	(T/C)
7462	CakSNP7462	*Kabuli*	Ca_Kabuli_Chr04	45231867	(A/T)
7463	CakSNP7463	*Kabuli*	Ca_Kabuli_Chr04	45231868	(C/A)
7464	CakSNP7464	*Kabuli*	Ca_Kabuli_Chr04	45231893	(G/T)
7465	CakSNP7465	*Kabuli*	Ca_Kabuli_Chr04	45292852	(G/T)
7466	CakSNP7466	*Kabuli*	Ca_Kabuli_Chr04	45292918	(T/G)
7467	CakSNP7467	*Kabuli*	Ca_Kabuli_Chr04	45292981	(G/T)
7468	CakSNP7468	*Kabuli*	Ca_Kabuli_Chr04	45292969	(T/A)
7469	CakSNP7469	*Kabuli*	Ca_Kabuli_Chr04	45292960	(A/T)
7470	CakSNP7470	*Kabuli*	Ca_Kabuli_Chr04	45552675	(C/A)
7471	CakSNP7471	*Kabuli*	Ca_Kabuli_Chr04	45595233	(G/A)
7472	CakSNP7472	*Kabuli*	Ca_Kabuli_Chr04	45612178	(T/G)
7473	CakSNP7473	*Kabuli*	Ca_Kabuli_Chr04	45615465	(C/T)
7474	CakSNP7474	*Kabuli*	Ca_Kabuli_Chr04	45615457	(C/T)
7475	CakSNP7475	*Kabuli*	Ca_Kabuli_Chr04	45615452	(A/C)
7476	CakSNP7476	*Kabuli*	Ca_Kabuli_Chr04	45615401	(T/G)
7477	CakSNP7477	*Kabuli*	Ca_Kabuli_Chr04	45646003	(A/G)
7478	CakSNP7478	*Kabuli*	Ca_Kabuli_Chr04	45645996	(A/G)
7479	CakSNP7479	*Kabuli*	Ca_Kabuli_Chr04	45645957	(A/C)
7480	CakSNP7480	*Kabuli*	Ca_Kabuli_Chr04	45708895	(C/A)
7481	CakSNP7481	*Kabuli*	Ca_Kabuli_Chr04	45735754	(G/C)
7482	CakSNP7482	*Kabuli*	Ca_Kabuli_Chr04	45735819	(T/C)
7483	CakSNP7483	*Kabuli*	Ca_Kabuli_Chr04	45751623	(G/A)
7484	CakSNP7484	*Kabuli*	Ca_Kabuli_Chr04	45751618	(C/T)
7485	CakSNP7485	*Kabuli*	Ca_Kabuli_Chr04	45752844	(G/A)

S.N.	SNP IDs	Cultivars	Chromosomes/scaffolds	Physical positions (bp)	SNPs
7486	CakSNP7486	*Kabuli*	Ca_Kabuli_Chr04	45752869	(G/T)
7487	CakSNP7487	*Kabuli*	Ca_Kabuli_Chr04	45752874	(A/T)
7488	CakSNP7488	*Kabuli*	Ca_Kabuli_Chr04	45782453	(A/T)
7489	CakSNP7489	*Kabuli*	Ca_Kabuli_Chr04	45870087	(T/C)
7490	CakSNP7490	*Kabuli*	Ca_Kabuli_Chr04	45870042	(C/T)
7491	CakSNP7491	*Kabuli*	Ca_Kabuli_Chr04	45918351	(A/C)
7492	CakSNP7492	*Kabuli*	Ca_Kabuli_Chr04	45935594	(A/C)
7493	CakSNP7493	*Kabuli*	Ca_Kabuli_Chr04	45942252	(G/A)
7494	CakSNP7494	*Kabuli*	Ca_Kabuli_Chr04	45942341	(T/C)
7495	CakSNP7495	*Kabuli*	Ca_Kabuli_Chr04	45942729	(G/A)
7496	CakSNP7496	*Kabuli*	Ca_Kabuli_Chr04	46134302	(C/G)
7497	CakSNP7497	*Kabuli*	Ca_Kabuli_Chr04	46140729	(A/T)
7498	CakSNP7498	*Kabuli*	Ca_Kabuli_Chr04	46148985	(A/T)
7499	CakSNP7499	*Kabuli*	Ca_Kabuli_Chr04	46149013	(A/G)
7500	CakSNP7500	*Kabuli*	Ca_Kabuli_Chr04	46150670	(T/C)
7501	CakSNP7501	*Kabuli*	Ca_Kabuli_Chr04	46202862	(A/G)
7502	CakSNP7502	*Kabuli*	Ca_Kabuli_Chr04	46344790	(G/A)
7503	CakSNP7503	*Kabuli*	Ca_Kabuli_Chr04	46344826	(A/G)
7504	CakSNP7504	*Kabuli*	Ca_Kabuli_Chr04	46345042	(A/G)
7505	CakSNP7505	*Kabuli*	Ca_Kabuli_Chr04	46402035	(G/A)
7506	CakSNP7506	*Kabuli*	Ca_Kabuli_Chr04	46430326	(C/T)
7507	CakSNP7507	*Kabuli*	Ca_Kabuli_Chr04	46430311	(C/A)
7508	CakSNP7508	*Kabuli*	Ca_Kabuli_Chr04	46430288	(G/C)
7509	CakSNP7509	*Kabuli*	Ca_Kabuli_Chr04	46605167	(A/G)
7510	CakSNP7510	*Kabuli*	Ca_Kabuli_Chr04	46605131	(T/A)
7511	CakSNP7511	*Kabuli*	Ca_Kabuli_Chr04	46612175	(G/A)
7512	CakSNP7512	*Kabuli*	Ca_Kabuli_Chr04	46612197	(G/A)
7513	CakSNP7513	*Kabuli*	Ca_Kabuli_Chr04	46612244	(C/T)
7514	CakSNP7514	*Kabuli*	Ca_Kabuli_Chr04	46613068	(T/G)
7515	CakSNP7515	*Kabuli*	Ca_Kabuli_Chr04	46694919	(T/A)
7516	CakSNP7516	*Kabuli*	Ca_Kabuli_Chr04	46695043	(A/C)
7517	CakSNP7517	*Kabuli*	Ca_Kabuli_Chr04	46695008	(T/C)
7518	CakSNP7518	*Kabuli*	Ca_Kabuli_Chr04	46705181	(G/T)
7519	CakSNP7519	*Kabuli*	Ca_Kabuli_Chr04	46705178	(A/T)
7520	CakSNP7520	*Kabuli*	Ca_Kabuli_Chr04	46705225	(A/C)
7521	CakSNP7521	*Kabuli*	Ca_Kabuli_Chr04	46713972	(C/T)
7522	CakSNP7522	*Kabuli*	Ca_Kabuli_Chr04	46715350	(T/C)
7523	CakSNP7523	*Kabuli*	Ca_Kabuli_Chr04	46715518	(G/C)

S.N.	SNP IDs	Cultivars	Chromosomes/scaffolds	Physical positions (bp)	SNPs
7524	CakSNP7524	*Kabuli*	Ca_Kabuli_Chr04	46717155	(T/C)
7525	CakSNP7525	*Kabuli*	Ca_Kabuli_Chr04	46717153	(C/T)
7526	CakSNP7526	*Kabuli*	Ca_Kabuli_Chr04	46717200	(C/T)
7527	CakSNP7527	*Kabuli*	Ca_Kabuli_Chr04	46717508	(G/A)
7528	CakSNP7528	*Kabuli*	Ca_Kabuli_Chr04	46763540	(C/T)
7529	CakSNP7529	*Kabuli*	Ca_Kabuli_Chr04	46763555	(C/T)
7530	CakSNP7530	*Kabuli*	Ca_Kabuli_Chr04	46763868	(T/C)
7531	CakSNP7531	*Kabuli*	Ca_Kabuli_Chr04	46763841	(A/C)
7532	CakSNP7532	*Kabuli*	Ca_Kabuli_Chr04	47223016	(T/C)
7533	CakSNP7533	*Kabuli*	Ca_Kabuli_Chr04	47223047	(A/G)
7534	CakSNP7534	*Kabuli*	Ca_Kabuli_Chr04	47223635	(G/A)
7535	CakSNP7535	*Kabuli*	Ca_Kabuli_Chr04	47223668	(T/G)
7536	CakSNP7536	*Kabuli*	Ca_Kabuli_Chr04	47296400	(C/T)
7537	CakSNP7537	*Kabuli*	Ca_Kabuli_Chr04	47379992	(C/T)
7538	CakSNP7538	*Kabuli*	Ca_Kabuli_Chr04	47405685	(T/A)
7539	CakSNP7539	*Kabuli*	Ca_Kabuli_Chr04	47419514	(A/G)
7540	CakSNP7540	*Kabuli*	Ca_Kabuli_Chr04	47419517	(C/T)
7541	CakSNP7541	*Kabuli*	Ca_Kabuli_Chr04	47428580	(C/T)
7542	CakSNP7542	*Kabuli*	Ca_Kabuli_Chr04	47428599	(C/T)
7543	CakSNP7543	*Kabuli*	Ca_Kabuli_Chr04	47428631	(A/G)
7544	CakSNP7544	*Kabuli*	Ca_Kabuli_Chr04	47428733	(A/G)
7545	CakSNP7545	*Kabuli*	Ca_Kabuli_Chr04	47440207	(G/A)
7546	CakSNP7546	*Kabuli*	Ca_Kabuli_Chr04	47450409	(C/T)
7547	CakSNP7547	*Kabuli*	Ca_Kabuli_Chr04	47450479	(C/G)
7548	CakSNP7548	*Kabuli*	Ca_Kabuli_Chr04	47562991	(A/T)
7549	CakSNP7549	*Kabuli*	Ca_Kabuli_Chr04	47572228	(G/A)
7550	CakSNP7550	*Kabuli*	Ca_Kabuli_Chr04	47572276	(C/T)
7551	CakSNP7551	*Kabuli*	Ca_Kabuli_Chr04	47578508	(C/T)
7552	CakSNP7552	*Kabuli*	Ca_Kabuli_Chr04	47578556	(T/G)
7553	CakSNP7553	*Kabuli*	Ca_Kabuli_Chr04	47578655	(C/T)
7554	CakSNP7554	*Kabuli*	Ca_Kabuli_Chr04	47578596	(C/T)
7555	CakSNP7555	*Kabuli*	Ca_Kabuli_Chr04	47588760	(G/A)
7556	CakSNP7556	*Kabuli*	Ca_Kabuli_Chr04	47612395	(C/A)
7557	CakSNP7557	*Kabuli*	Ca_Kabuli_Chr04	47612396	(G/T)
7558	CakSNP7558	*Kabuli*	Ca_Kabuli_Chr04	47615800	(G/C)
7559	CakSNP7559	*Kabuli*	Ca_Kabuli_Chr04	47616120	(C/T)
7560	CakSNP7560	*Kabuli*	Ca_Kabuli_Chr04	47616216	(A/G)
7561	CakSNP7561	*Kabuli*	Ca_Kabuli_Chr04	47622112	(G/T)

S.N.	SNP IDs	Cultivars	Chromosomes/scaffolds	Physical positions (bp)	SNPs
7562	CakSNP7562	*Kabuli*	Ca_Kabuli_Chr04	47675446	(A/G)
7563	CakSNP7563	*Kabuli*	Ca_Kabuli_Chr04	47675758	(A/T)
7564	CakSNP7564	*Kabuli*	Ca_Kabuli_Chr04	47675860	(G/A)
7565	CakSNP7565	*Kabuli*	Ca_Kabuli_Chr04	47697239	(C/T)
7566	CakSNP7566	*Kabuli*	Ca_Kabuli_Chr04	47798580	(A/G)
7567	CakSNP7567	*Kabuli*	Ca_Kabuli_Chr04	47798622	(G/A)
7568	CakSNP7568	*Kabuli*	Ca_Kabuli_Chr04	47819943	(T/C)
7569	CakSNP7569	*Kabuli*	Ca_Kabuli_Chr04	48008987	(T/C)
7570	CakSNP7570	*Kabuli*	Ca_Kabuli_Chr04	48009201	(C/T)
7571	CakSNP7571	*Kabuli*	Ca_Kabuli_Chr04	48011446	(A/G)
7572	CakSNP7572	*Kabuli*	Ca_Kabuli_Chr04	48098570	(G/A)
7573	CakSNP7573	*Kabuli*	Ca_Kabuli_Chr04	48098562	(G/A)
7574	CakSNP7574	*Kabuli*	Ca_Kabuli_Chr04	48103202	(G/T)
7575	CakSNP7575	*Kabuli*	Ca_Kabuli_Chr04	48103196	(G/A)
7576	CakSNP7576	*Kabuli*	Ca_Kabuli_Chr04	48103159	(G/A)
7577	CakSNP7577	*Kabuli*	Ca_Kabuli_Chr04	48103153	(A/C)
7578	CakSNP7578	*Kabuli*	Ca_Kabuli_Chr04	48150277	(C/T)
7579	CakSNP7579	*Kabuli*	Ca_Kabuli_Chr04	48150481	(T/C)
7580	CakSNP7580	*Kabuli*	Ca_Kabuli_Chr04	48150480	(C/A)
7581	CakSNP7581	*Kabuli*	Ca_Kabuli_Chr04	48150553	(A/G)
7582	CakSNP7582	*Kabuli*	Ca_Kabuli_Chr04	48150641	(G/T)
7583	CakSNP7583	*Kabuli*	Ca_Kabuli_Chr04	48238919	(C/T)
7584	CakSNP7584	*Kabuli*	Ca_Kabuli_Chr04	48239048	(A/G)
7585	CakSNP7585	*Kabuli*	Ca_Kabuli_Chr04	48263603	(G/A)
7586	CakSNP7586	*Kabuli*	Ca_Kabuli_Chr04	48269138	(G/C)
7587	CakSNP7587	*Kabuli*	Ca_Kabuli_Chr04	48275016	(C/T)
7588	CakSNP7588	*Kabuli*	Ca_Kabuli_Chr04	48322502	(C/T)
7589	CakSNP7589	*Kabuli*	Ca_Kabuli_Chr04	48322507	(C/T)
7590	CakSNP7590	*Kabuli*	Ca_Kabuli_Chr04	48322633	(T/C)
7591	CakSNP7591	*Kabuli*	Ca_Kabuli_Chr04	48331069	(C/A)
7592	CakSNP7592	*Kabuli*	Ca_Kabuli_Chr04	48345604	(A/G)
7593	CakSNP7593	*Kabuli*	Ca_Kabuli_Chr04	48345608	(G/T)
7594	CakSNP7594	*Kabuli*	Ca_Kabuli_Chr04	48345655	(A/C)
7595	CakSNP7595	*Kabuli*	Ca_Kabuli_Chr04	48345697	(C/G)
7596	CakSNP7596	*Kabuli*	Ca_Kabuli_Chr04	48355358	(A/T)
7597	CakSNP7597	*Kabuli*	Ca_Kabuli_Chr04	48355468	(G/T)
7598	CakSNP7598	*Kabuli*	Ca_Kabuli_Chr04	48364170	(G/A)
7599	CakSNP7599	*Kabuli*	Ca_Kabuli_Chr04	48365208	(C/A)

**S.N.**	**SNP IDs**	**Cultivars**	**Chromosomes/scaffolds**	**Physical positions (bp)**	**SNPs**
7600	CakSNP7600	*Kabuli*	Ca_Kabuli_Chr04	48365388	(G/A)
7601	CakSNP7601	*Kabuli*	Ca_Kabuli_Chr04	48382666	(G/C)
7602	CakSNP7602	*Kabuli*	Ca_Kabuli_Chr04	48464493	(T/C)
7603	CakSNP7603	*Kabuli*	Ca_Kabuli_Chr04	48464434	(G/C)
7604	CakSNP7604	*Kabuli*	Ca_Kabuli_Chr04	48470477	(A/G)
7605	CakSNP7605	*Kabuli*	Ca_Kabuli_Chr04	48475447	(T/C)
7606	CakSNP7606	*Kabuli*	Ca_Kabuli_Chr04	48475461	(G/A)
7607	CakSNP7607	*Kabuli*	Ca_Kabuli_Chr04	48478018	(A/G)
7608	CakSNP7608	*Kabuli*	Ca_Kabuli_Chr04	48478058	(T/C)
7609	CakSNP7609	*Kabuli*	Ca_Kabuli_Chr04	48478303	(G/A)
7610	CakSNP7610	*Kabuli*	Ca_Kabuli_Chr04	48478524	(C/T)
7611	CakSNP7611	*Kabuli*	Ca_Kabuli_Chr04	48484453	(A/T)
7612	CakSNP7612	*Kabuli*	Ca_Kabuli_Chr04	48497731	(A/C)
7613	CakSNP7613	*Kabuli*	Ca_Kabuli_Chr04	48497725	(A/C)
7614	CakSNP7614	*Kabuli*	Ca_Kabuli_Chr04	48497756	(A/G)
7615	CakSNP7615	*Kabuli*	Ca_Kabuli_Chr04	48497765	(G/A)
7616	CakSNP7616	*Kabuli*	Ca_Kabuli_Chr04	48497839	(G/A)
7617	CakSNP7617	*Kabuli*	Ca_Kabuli_Chr04	48498133	(C/T)
7618	CakSNP7618	*Kabuli*	Ca_Kabuli_Chr04	48498181	(G/C)
7619	CakSNP7619	*Kabuli*	Ca_Kabuli_Chr04	48498166	(A/G)
7620	CakSNP7620	*Kabuli*	Ca_Kabuli_Chr04	48498368	(C/T)
7621	CakSNP7621	*Kabuli*	Ca_Kabuli_Chr04	48498334	(T/G)
7622	CakSNP7622	*Kabuli*	Ca_Kabuli_Chr04	48498331	(T/A)
7623	CakSNP7623	*Kabuli*	Ca_Kabuli_Chr04	48498312	(T/C)
7624	CakSNP7624	*Kabuli*	Ca_Kabuli_Chr04	48501567	(G/T)
7625	CakSNP7625	*Kabuli*	Ca_Kabuli_Chr04	48501618	(C/A)
7626	CakSNP7626	*Kabuli*	Ca_Kabuli_Chr04	48501662	(A/T)
7627	CakSNP7627	*Kabuli*	Ca_Kabuli_Chr04	48502554	(G/T)
7628	CakSNP7628	*Kabuli*	Ca_Kabuli_Chr04	48502718	(G/C)
7629	CakSNP7629	*Kabuli*	Ca_Kabuli_Chr04	48502714	(T/C)
7630	CakSNP7630	*Kabuli*	Ca_Kabuli_Chr04	48502687	(G/A)
7631	CakSNP7631	*Kabuli*	Ca_Kabuli_Chr04	48591482	(T/G)
7632	CakSNP7632	*Kabuli*	Ca_Kabuli_Chr04	48591539	(T/C)
7633	CakSNP7633	*Kabuli*	Ca_Kabuli_Chr04	48591578	(T/C)
7634	CakSNP7634	*Kabuli*	Ca_Kabuli_Chr04	48591625	(G/T)
7635	CakSNP7635	*Kabuli*	Ca_Kabuli_Chr04	48678278	(T/C)
7636	CakSNP7636	*Kabuli*	Ca_Kabuli_Chr04	48678692	(T/C)
7637	CakSNP7637	*Kabuli*	Ca_Kabuli_Chr04	48688766	(A/G)

S.N.	SNP IDs	Cultivars	Chromosomes/scaffolds	Physical positions (bp)	SNPs
7638	CakSNP7638	*Kabuli*	Ca_Kabuli_Chr04	48705815	(C/T)
7639	CakSNP7639	*Kabuli*	Ca_Kabuli_Chr04	48705856	(A/T)
7640	CakSNP7640	*Kabuli*	Ca_Kabuli_Chr04	48705957	(C/T)
7641	CakSNP7641	*Kabuli*	Ca_Kabuli_Chr04	48714912	(G/A)
7642	CakSNP7642	*Kabuli*	Ca_Kabuli_Chr04	48715028	(A/G)
7643	CakSNP7643	*Kabuli*	Ca_Kabuli_Chr04	48720330	(C/T)
7644	CakSNP7644	*Kabuli*	Ca_Kabuli_Chr04	48720266	(C/T)
7645	CakSNP7645	*Kabuli*	Ca_Kabuli_Chr04	48784151	(T/G)
7646	CakSNP7646	*Kabuli*	Ca_Kabuli_Chr04	48784176	(T/C)
7647	CakSNP7647	*Kabuli*	Ca_Kabuli_Chr04	48784178	(C/G)
7648	CakSNP7648	*Kabuli*	Ca_Kabuli_Chr04	48784179	(G/A)
7649	CakSNP7649	*Kabuli*	Ca_Kabuli_Chr04	48784189	(A/T)
7650	CakSNP7650	*Kabuli*	Ca_Kabuli_Chr04	48784202	(A/C)
7651	CakSNP7651	*Kabuli*	Ca_Kabuli_Chr04	48784204	(G/C)
7652	CakSNP7652	*Kabuli*	Ca_Kabuli_Chr04	48931112	(T/G)
7653	CakSNP7653	*Kabuli*	Ca_Kabuli_Chr04	48931109	(A/G)
7654	CakSNP7654	*Kabuli*	Ca_Kabuli_Chr04	48936643	(G/A)
7655	CakSNP7655	*Kabuli*	Ca_Kabuli_Chr04	48936669	(G/A)
7656	CakSNP7656	*Kabuli*	Ca_Kabuli_Chr04	48936710	(A/G)
7657	CakSNP7657	*Kabuli*	Ca_Kabuli_Chr04	48936819	(C/T)
7658	CakSNP7658	*Kabuli*	Ca_Kabuli_Chr04	48964200	(T/C)
7659	CakSNP7659	*Kabuli*	Ca_Kabuli_Chr04	48964260	(G/C)
7660	CakSNP7660	*Kabuli*	Ca_Kabuli_Chr04	48964248	(C/T)
7661	CakSNP7661	*Kabuli*	Ca_Kabuli_Chr04	49012733	(T/A)
7662	CakSNP7662	*Kabuli*	Ca_Kabuli_Chr04	49012756	(C/T)
7663	CakSNP7663	*Kabuli*	Ca_Kabuli_Chr05	95087	(G/A)
7664	CakSNP7664	*Kabuli*	Ca_Kabuli_Chr05	95253	(A/G)
7665	CakSNP7665	*Kabuli*	Ca_Kabuli_Chr05	131558	(A/C)
7666	CakSNP7666	*Kabuli*	Ca_Kabuli_Chr05	154084	(T/G)
7667	CakSNP7667	*Kabuli*	Ca_Kabuli_Chr05	154095	(C/G)
7668	CakSNP7668	*Kabuli*	Ca_Kabuli_Chr05	154097	(A/T)
7669	CakSNP7669	*Kabuli*	Ca_Kabuli_Chr05	154162	(C/T)
7670	CakSNP7670	*Kabuli*	Ca_Kabuli_Chr05	154128	(G/T)
7671	CakSNP7671	*Kabuli*	Ca_Kabuli_Chr05	166222	(T/G)
7672	CakSNP7672	*Kabuli*	Ca_Kabuli_Chr05	166217	(G/C)
7673	CakSNP7673	*Kabuli*	Ca_Kabuli_Chr05	166216	(T/G)
7674	CakSNP7674	*Kabuli*	Ca_Kabuli_Chr05	166341	(C/A)
7675	CakSNP7675	*Kabuli*	Ca_Kabuli_Chr05	166381	(A/G)

S.N.	SNP IDs	Cultivars	Chromosomes/scaffolds	Physical positions (bp)	SNPs
7676	CakSNP7676	*Kabuli*	Ca_Kabuli_Chr05	210625	(T/C)
7677	CakSNP7677	*Kabuli*	Ca_Kabuli_Chr05	210649	(C/A)
7678	CakSNP7678	*Kabuli*	Ca_Kabuli_Chr05	222651	(A/C)
7679	CakSNP7679	*Kabuli*	Ca_Kabuli_Chr05	222630	(A/G)
7680	CakSNP7680	*Kabuli*	Ca_Kabuli_Chr05	222641	(C/T)
7681	CakSNP7681	*Kabuli*	Ca_Kabuli_Chr05	222653	(A/C)
7682	CakSNP7682	*Kabuli*	Ca_Kabuli_Chr05	222657	(G/A)
7683	CakSNP7683	*Kabuli*	Ca_Kabuli_Chr05	223384	(C/G)
7684	CakSNP7684	*Kabuli*	Ca_Kabuli_Chr05	234247	(T/C)
7685	CakSNP7685	*Kabuli*	Ca_Kabuli_Chr05	234319	(T/A)
7686	CakSNP7686	*Kabuli*	Ca_Kabuli_Chr05	234311	(T/A)
7687	CakSNP7687	*Kabuli*	Ca_Kabuli_Chr05	234310	(G/A)
7688	CakSNP7688	*Kabuli*	Ca_Kabuli_Chr05	234305	(T/A)
7689	CakSNP7689	*Kabuli*	Ca_Kabuli_Chr05	234303	(C/A)
7690	CakSNP7690	*Kabuli*	Ca_Kabuli_Chr05	234410	(A/C)
7691	CakSNP7691	*Kabuli*	Ca_Kabuli_Chr05	245850	(T/G)
7692	CakSNP7692	*Kabuli*	Ca_Kabuli_Chr05	245863	(A/T)
7693	CakSNP7693	*Kabuli*	Ca_Kabuli_Chr05	245896	(A/T)
7694	CakSNP7694	*Kabuli*	Ca_Kabuli_Chr05	246466	(A/T)
7695	CakSNP7695	*Kabuli*	Ca_Kabuli_Chr05	246654	(A/G)
7696	CakSNP7696	*Kabuli*	Ca_Kabuli_Chr05	256745	(G/A)
7697	CakSNP7697	*Kabuli*	Ca_Kabuli_Chr05	272972	(T/A)
7698	CakSNP7698	*Kabuli*	Ca_Kabuli_Chr05	277040	(G/A)
7699	CakSNP7699	*Kabuli*	Ca_Kabuli_Chr05	277316	(T/A)
7700	CakSNP7700	*Kabuli*	Ca_Kabuli_Chr05	343517	(C/T)
7701	CakSNP7701	*Kabuli*	Ca_Kabuli_Chr05	353792	(T/A)
7702	CakSNP7702	*Kabuli*	Ca_Kabuli_Chr05	438448	(C/A)
7703	CakSNP7703	*Kabuli*	Ca_Kabuli_Chr05	438445	(C/A)
7704	CakSNP7704	*Kabuli*	Ca_Kabuli_Chr05	438442	(T/C)
7705	CakSNP7705	*Kabuli*	Ca_Kabuli_Chr05	443309	(A/T)
7706	CakSNP7706	*Kabuli*	Ca_Kabuli_Chr05	469397	(G/A)
7707	CakSNP7707	*Kabuli*	Ca_Kabuli_Chr05	805859	(C/T)
7708	CakSNP7708	*Kabuli*	Ca_Kabuli_Chr05	805882	(A/T)
7709	CakSNP7709	*Kabuli*	Ca_Kabuli_Chr05	805883	(G/T)
7710	CakSNP7710	*Kabuli*	Ca_Kabuli_Chr05	805910	(G/A)
7711	CakSNP7711	*Kabuli*	Ca_Kabuli_Chr05	870867	(A/G)
7712	CakSNP7712	*Kabuli*	Ca_Kabuli_Chr05	870909	(G/A)
7713	CakSNP7713	*Kabuli*	Ca_Kabuli_Chr05	925554	(T/C)

S.N.	SNP IDs	Cultivars	Chromosomes/scaffolds	Physical positions (bp)	SNPs
7714	CakSNP7714	*Kabuli*	Ca_Kabuli_Chr05	925493	(T/C)
7715	CakSNP7715	*Kabuli*	Ca_Kabuli_Chr05	1141755	(C/T)
7716	CakSNP7716	*Kabuli*	Ca_Kabuli_Chr05	1220867	(A/G)
7717	CakSNP7717	*Kabuli*	Ca_Kabuli_Chr05	1252394	(G/A)
7718	CakSNP7718	*Kabuli*	Ca_Kabuli_Chr05	1252388	(A/T)
7719	CakSNP7719	*Kabuli*	Ca_Kabuli_Chr05	1270830	(T/C)
7720	CakSNP7720	*Kabuli*	Ca_Kabuli_Chr05	1272581	(T/C)
7721	CakSNP7721	*Kabuli*	Ca_Kabuli_Chr05	1280977	(T/G)
7722	CakSNP7722	*Kabuli*	Ca_Kabuli_Chr05	1281006	(A/C)
7723	CakSNP7723	*Kabuli*	Ca_Kabuli_Chr05	1281017	(G/A)
7724	CakSNP7724	*Kabuli*	Ca_Kabuli_Chr05	1281021	(C/T)
7725	CakSNP7725	*Kabuli*	Ca_Kabuli_Chr05	1534904	(C/T)
7726	CakSNP7726	*Kabuli*	Ca_Kabuli_Chr05	1534901	(C/T)
7727	CakSNP7727	*Kabuli*	Ca_Kabuli_Chr05	1534888	(T/C)
7728	CakSNP7728	*Kabuli*	Ca_Kabuli_Chr05	2166586	(A/G)
7729	CakSNP7729	*Kabuli*	Ca_Kabuli_Chr05	2519977	(C/T)
7730	CakSNP7730	*Kabuli*	Ca_Kabuli_Chr05	2519949	(C/T)
7731	CakSNP7731	*Kabuli*	Ca_Kabuli_Chr05	2519961	(G/A)
7732	CakSNP7732	*Kabuli*	Ca_Kabuli_Chr05	2519976	(G/A)
7733	CakSNP7733	*Kabuli*	Ca_Kabuli_Chr05	2520035	(G/A)
7734	CakSNP7734	*Kabuli*	Ca_Kabuli_Chr05	2520058	(C/T)
7735	CakSNP7735	*Kabuli*	Ca_Kabuli_Chr05	2520015	(C/G)
7736	CakSNP7736	*Kabuli*	Ca_Kabuli_Chr05	2520049	(C/T)
7737	CakSNP7737	*Kabuli*	Ca_Kabuli_Chr05	2520050	(G/C)
7738	CakSNP7738	*Kabuli*	Ca_Kabuli_Chr05	2520051	(G/T)
7739	CakSNP7739	*Kabuli*	Ca_Kabuli_Chr05	2520055	(C/T)
7740	CakSNP7740	*Kabuli*	Ca_Kabuli_Chr05	2520068	(T/A)
7741	CakSNP7741	*Kabuli*	Ca_Kabuli_Chr05	2520069	(C/T)
7742	CakSNP7742	*Kabuli*	Ca_Kabuli_Chr05	2520094	(G/A)
7743	CakSNP7743	*Kabuli*	Ca_Kabuli_Chr05	2520061	(C/T)
7744	CakSNP7744	*Kabuli*	Ca_Kabuli_Chr05	2520093	(G/A)
7745	CakSNP7745	*Kabuli*	Ca_Kabuli_Chr05	2520123	(A/G)
7746	CakSNP7746	*Kabuli*	Ca_Kabuli_Chr05	2520114	(G/A)
7747	CakSNP7747	*Kabuli*	Ca_Kabuli_Chr05	2520109	(G/A)
7748	CakSNP7748	*Kabuli*	Ca_Kabuli_Chr05	2520101	(C/T)
7749	CakSNP7749	*Kabuli*	Ca_Kabuli_Chr05	2613423	(T/G)
7750	CakSNP7750	*Kabuli*	Ca_Kabuli_Chr05	2948530	(G/T)
7751	CakSNP7751	*Kabuli*	Ca_Kabuli_Chr05	2948531	(T/C)

S.N.	SNP IDs	Cultivars	Chromosomes/scaffolds	Physical positions (bp)	SNPs
7752	CakSNP7752	*Kabuli*	Ca_Kabuli_Chr05	2948549	(C/A)
7753	CakSNP7753	*Kabuli*	Ca_Kabuli_Chr05	2948592	(T/C)
7754	CakSNP7754	*Kabuli*	Ca_Kabuli_Chr05	2948596	(C/T)
7755	CakSNP7755	*Kabuli*	Ca_Kabuli_Chr05	2948616	(G/A)
7756	CakSNP7756	*Kabuli*	Ca_Kabuli_Chr05	2948698	(G/A)
7757	CakSNP7757	*Kabuli*	Ca_Kabuli_Chr05	2948977	(G/A)
7758	CakSNP7758	*Kabuli*	Ca_Kabuli_Chr05	3007213	(A/G)
7759	CakSNP7759	*Kabuli*	Ca_Kabuli_Chr05	3007316	(T/C)
7760	CakSNP7760	*Kabuli*	Ca_Kabuli_Chr05	3040264	(G/T)
7761	CakSNP7761	*Kabuli*	Ca_Kabuli_Chr05	3040948	(G/C)
7762	CakSNP7762	*Kabuli*	Ca_Kabuli_Chr05	3159061	(G/A)
7763	CakSNP7763	*Kabuli*	Ca_Kabuli_Chr05	3348291	(G/A)
7764	CakSNP7764	*Kabuli*	Ca_Kabuli_Chr05	3348310	(A/G)
7765	CakSNP7765	*Kabuli*	Ca_Kabuli_Chr05	3348366	(G/C)
7766	CakSNP7766	*Kabuli*	Ca_Kabuli_Chr05	3353052	(A/G)
7767	CakSNP7767	*Kabuli*	Ca_Kabuli_Chr05	4122395	(T/C)
7768	CakSNP7768	*Kabuli*	Ca_Kabuli_Chr05	4122342	(T/C)
7769	CakSNP7769	*Kabuli*	Ca_Kabuli_Chr05	5133644	(A/G)
7770	CakSNP7770	*Kabuli*	Ca_Kabuli_Chr05	5205261	(T/G)
7771	CakSNP7771	*Kabuli*	Ca_Kabuli_Chr05	5404261	(G/A)
7772	CakSNP7772	*Kabuli*	Ca_Kabuli_Chr05	6190494	(T/C)
7773	CakSNP7773	*Kabuli*	Ca_Kabuli_Chr05	6225426	(T/A)
7774	CakSNP7774	*Kabuli*	Ca_Kabuli_Chr05	6225415	(G/C)
7775	CakSNP7775	*Kabuli*	Ca_Kabuli_Chr05	6225410	(T/A)
7776	CakSNP7776	*Kabuli*	Ca_Kabuli_Chr05	6290453	(G/A)
7777	CakSNP7777	*Kabuli*	Ca_Kabuli_Chr05	6300337	(C/A)
7778	CakSNP7778	*Kabuli*	Ca_Kabuli_Chr05	6300360	(C/A)
7779	CakSNP7779	*Kabuli*	Ca_Kabuli_Chr05	6300502	(C/A)
7780	CakSNP7780	*Kabuli*	Ca_Kabuli_Chr05	6316787	(C/T)
7781	CakSNP7781	*Kabuli*	Ca_Kabuli_Chr05	6317014	(C/T)
7782	CakSNP7782	*Kabuli*	Ca_Kabuli_Chr05	6931865	(A/G)
7783	CakSNP7783	*Kabuli*	Ca_Kabuli_Chr05	7082295	(G/A)
7784	CakSNP7784	*Kabuli*	Ca_Kabuli_Chr05	7082296	(T/G)
7785	CakSNP7785	*Kabuli*	Ca_Kabuli_Chr05	7082301	(G/T)
7786	CakSNP7786	*Kabuli*	Ca_Kabuli_Chr05	7278953	(C/T)
7787	CakSNP7787	*Kabuli*	Ca_Kabuli_Chr05	7398246	(A/C)
7788	CakSNP7788	*Kabuli*	Ca_Kabuli_Chr05	7425116	(C/T)
7789	CakSNP7789	*Kabuli*	Ca_Kabuli_Chr05	7617914	(T/A)

S.N.	SNP IDs	Cultivars	Chromosomes/scaffolds	Physical positions (bp)	SNPs
7790	CakSNP7790	*Kabuli*	Ca_Kabuli_Chr05	7628097	(C/A)
7791	CakSNP7791	*Kabuli*	Ca_Kabuli_Chr05	7641993	(T/C)
7792	CakSNP7792	*Kabuli*	Ca_Kabuli_Chr05	7641925	(G/T)
7793	CakSNP7793	*Kabuli*	Ca_Kabuli_Chr05	7641923	(A/G)
7794	CakSNP7794	*Kabuli*	Ca_Kabuli_Chr05	7664421	(T/C)
7795	CakSNP7795	*Kabuli*	Ca_Kabuli_Chr05	7775286	(C/A)
7796	CakSNP7796	*Kabuli*	Ca_Kabuli_Chr05	7775290	(A/T)
7797	CakSNP7797	*Kabuli*	Ca_Kabuli_Chr05	7775298	(A/T)
7798	CakSNP7798	*Kabuli*	Ca_Kabuli_Chr05	7775329	(A/G)
7799	CakSNP7799	*Kabuli*	Ca_Kabuli_Chr05	7775516	(A/G)
7800	CakSNP7800	*Kabuli*	Ca_Kabuli_Chr05	7775497	(G/C)
7801	CakSNP7801	*Kabuli*	Ca_Kabuli_Chr05	7775472	(G/A)
7802	CakSNP7802	*Kabuli*	Ca_Kabuli_Chr05	7775456	(T/C)
7803	CakSNP7803	*Kabuli*	Ca_Kabuli_Chr05	7775918	(C/T)
7804	CakSNP7804	*Kabuli*	Ca_Kabuli_Chr05	7789021	(G/T)
7805	CakSNP7805	*Kabuli*	Ca_Kabuli_Chr05	7789065	(A/G)
7806	CakSNP7806	*Kabuli*	Ca_Kabuli_Chr05	7826336	(G/T)
7807	CakSNP7807	*Kabuli*	Ca_Kabuli_Chr05	7826338	(T/G)
7808	CakSNP7808	*Kabuli*	Ca_Kabuli_Chr05	7826362	(C/T)
7809	CakSNP7809	*Kabuli*	Ca_Kabuli_Chr05	7826366	(A/G)
7810	CakSNP7810	*Kabuli*	Ca_Kabuli_Chr05	7925356	(A/C)
7811	CakSNP7811	*Kabuli*	Ca_Kabuli_Chr05	8263483	(A/G)
7812	CakSNP7812	*Kabuli*	Ca_Kabuli_Chr05	8653283	(G/A)
7813	CakSNP7813	*Kabuli*	Ca_Kabuli_Chr05	8676750	(T/G)
7814	CakSNP7814	*Kabuli*	Ca_Kabuli_Chr05	8676807	(G/A)
7815	CakSNP7815	*Kabuli*	Ca_Kabuli_Chr05	8676789	(T/C)
7816	CakSNP7816	*Kabuli*	Ca_Kabuli_Chr05	8676771	(G/A)
7817	CakSNP7817	*Kabuli*	Ca_Kabuli_Chr05	8946218	(G/A)
7818	CakSNP7818	*Kabuli*	Ca_Kabuli_Chr05	9034100	(C/G)
7819	CakSNP7819	*Kabuli*	Ca_Kabuli_Chr05	9076709	(C/T)
7820	CakSNP7820	*Kabuli*	Ca_Kabuli_Chr05	9076712	(G/A)
7821	CakSNP7821	*Kabuli*	Ca_Kabuli_Chr05	9076737	(C/A)
7822	CakSNP7822	*Kabuli*	Ca_Kabuli_Chr05	9076738	(G/C)
7823	CakSNP7823	*Kabuli*	Ca_Kabuli_Chr05	9076767	(C/A)
7824	CakSNP7824	*Kabuli*	Ca_Kabuli_Chr05	9076824	(G/A)
7825	CakSNP7825	*Kabuli*	Ca_Kabuli_Chr05	9076821	(G/T)
7826	CakSNP7826	*Kabuli*	Ca_Kabuli_Chr05	9076809	(T/A)
7827	CakSNP7827	*Kabuli*	Ca_Kabuli_Chr05	9077489	(T/C)

S.N.	SNP IDs	Cultivars	Chromosomes/scaffolds	Physical positions (bp)	SNPs
7828	CakSNP7828	*Kabuli*	Ca_Kabuli_Chr05	9519382	(C/A)
7829	CakSNP7829	*Kabuli*	Ca_Kabuli_Chr05	9519390	(G/A)
7830	CakSNP7830	*Kabuli*	Ca_Kabuli_Chr05	9609055	(T/C)
7831	CakSNP7831	*Kabuli*	Ca_Kabuli_Chr05	9620998	(A/C)
7832	CakSNP7832	*Kabuli*	Ca_Kabuli_Chr05	9728498	(A/T)
7833	CakSNP7833	*Kabuli*	Ca_Kabuli_Chr05	9728609	(T/A)
7834	CakSNP7834	*Kabuli*	Ca_Kabuli_Chr05	9728591	(A/G)
7835	CakSNP7835	*Kabuli*	Ca_Kabuli_Chr05	9786213	(C/A)
7836	CakSNP7836	*Kabuli*	Ca_Kabuli_Chr05	9868796	(T/C)
7837	CakSNP7837	*Kabuli*	Ca_Kabuli_Chr05	9869005	(T/G)
7838	CakSNP7838	*Kabuli*	Ca_Kabuli_Chr05	9961273	(T/C)
7839	CakSNP7839	*Kabuli*	Ca_Kabuli_Chr05	9961396	(T/C)
7840	CakSNP7840	*Kabuli*	Ca_Kabuli_Chr05	9961380	(C/T)
7841	CakSNP7841	*Kabuli*	Ca_Kabuli_Chr05	9965880	(G/C)
7842	CakSNP7842	*Kabuli*	Ca_Kabuli_Chr05	9965890	(T/C)
7843	CakSNP7843	*Kabuli*	Ca_Kabuli_Chr05	9965913	(A/C)
7844	CakSNP7844	*Kabuli*	Ca_Kabuli_Chr05	9965848	(T/C)
7845	CakSNP7845	*Kabuli*	Ca_Kabuli_Chr05	9965846	(A/C)
7846	CakSNP7846	*Kabuli*	Ca_Kabuli_Chr05	10004386	(G/T)
7847	CakSNP7847	*Kabuli*	Ca_Kabuli_Chr05	10004427	(T/G)
7848	CakSNP7848	*Kabuli*	Ca_Kabuli_Chr05	10004430	(T/G)
7849	CakSNP7849	*Kabuli*	Ca_Kabuli_Chr05	10004451	(T/G)
7850	CakSNP7850	*Kabuli*	Ca_Kabuli_Chr05	10054140	(G/C)
7851	CakSNP7851	*Kabuli*	Ca_Kabuli_Chr05	10054146	(A/G)
7852	CakSNP7852	*Kabuli*	Ca_Kabuli_Chr05	10054266	(T/C)
7853	CakSNP7853	*Kabuli*	Ca_Kabuli_Chr05	10054213	(A/C)
7854	CakSNP7854	*Kabuli*	Ca_Kabuli_Chr05	10072358	(G/A)
7855	CakSNP7855	*Kabuli*	Ca_Kabuli_Chr05	10072403	(G/C)
7856	CakSNP7856	*Kabuli*	Ca_Kabuli_Chr05	10154016	(A/G)
7857	CakSNP7857	*Kabuli*	Ca_Kabuli_Chr05	10247223	(T/C)
7858	CakSNP7858	*Kabuli*	Ca_Kabuli_Chr05	10247224	(T/A)
7859	CakSNP7859	*Kabuli*	Ca_Kabuli_Chr05	10247233	(C/T)
7860	CakSNP7860	*Kabuli*	Ca_Kabuli_Chr05	10247244	(C/T)
7861	CakSNP7861	*Kabuli*	Ca_Kabuli_Chr05	10390882	(C/T)
7862	CakSNP7862	*Kabuli*	Ca_Kabuli_Chr05	10406562	(C/A)
7863	CakSNP7863	*Kabuli*	Ca_Kabuli_Chr05	10406602	(C/T)
7864	CakSNP7864	*Kabuli*	Ca_Kabuli_Chr05	10406613	(C/T)
7865	CakSNP7865	*Kabuli*	Ca_Kabuli_Chr05	10406636	(A/G)

S.N.	SNP IDs	Cultivars	Chromosomes/scaffolds	Physical positions (bp)	SNPs
7866	CakSNP7866	*Kabuli*	Ca_Kabuli_Chr05	10406649	(A/C)
7867	CakSNP7867	*Kabuli*	Ca_Kabuli_Chr05	10406650	(A/G)
7868	CakSNP7868	*Kabuli*	Ca_Kabuli_Chr05	10406754	(G/T)
7869	CakSNP7869	*Kabuli*	Ca_Kabuli_Chr05	10510166	(G/A)
7870	CakSNP7870	*Kabuli*	Ca_Kabuli_Chr05	10544823	(A/G)
7871	CakSNP7871	*Kabuli*	Ca_Kabuli_Chr05	10546944	(A/T)
7872	CakSNP7872	*Kabuli*	Ca_Kabuli_Chr05	10675252	(A/G)
7873	CakSNP7873	*Kabuli*	Ca_Kabuli_Chr05	10675279	(C/G)
7874	CakSNP7874	*Kabuli*	Ca_Kabuli_Chr05	10675294	(A/G)
7875	CakSNP7875	*Kabuli*	Ca_Kabuli_Chr05	10769765	(C/T)
7876	CakSNP7876	*Kabuli*	Ca_Kabuli_Chr05	10832275	(T/C)
7877	CakSNP7877	*Kabuli*	Ca_Kabuli_Chr05	10858490	(C/T)
7878	CakSNP7878	*Kabuli*	Ca_Kabuli_Chr05	10918368	(A/G)
7879	CakSNP7879	*Kabuli*	Ca_Kabuli_Chr05	10919246	(C/G)
7880	CakSNP7880	*Kabuli*	Ca_Kabuli_Chr05	11230617	(C/G)
7881	CakSNP7881	*Kabuli*	Ca_Kabuli_Chr05	11230627	(A/G)
7882	CakSNP7882	*Kabuli*	Ca_Kabuli_Chr05	11230648	(G/A)
7883	CakSNP7883	*Kabuli*	Ca_Kabuli_Chr05	11230671	(T/C)
7884	CakSNP7884	*Kabuli*	Ca_Kabuli_Chr05	11263571	(C/G)
7885	CakSNP7885	*Kabuli*	Ca_Kabuli_Chr05	11263574	(C/A)
7886	CakSNP7886	*Kabuli*	Ca_Kabuli_Chr05	11317487	(C/T)
7887	CakSNP7887	*Kabuli*	Ca_Kabuli_Chr05	11317502	(C/A)
7888	CakSNP7888	*Kabuli*	Ca_Kabuli_Chr05	11317534	(C/A)
7889	CakSNP7889	*Kabuli*	Ca_Kabuli_Chr05	11385780	(A/C)
7890	CakSNP7890	*Kabuli*	Ca_Kabuli_Chr05	11385874	(A/G)
7891	CakSNP7891	*Kabuli*	Ca_Kabuli_Chr05	11765841	(C/T)
7892	CakSNP7892	*Kabuli*	Ca_Kabuli_Chr05	11777112	(C/T)
7893	CakSNP7893	*Kabuli*	Ca_Kabuli_Chr05	11807822	(G/T)
7894	CakSNP7894	*Kabuli*	Ca_Kabuli_Chr05	11807842	(T/C)
7895	CakSNP7895	*Kabuli*	Ca_Kabuli_Chr05	11845515	(C/T)
7896	CakSNP7896	*Kabuli*	Ca_Kabuli_Chr05	11853090	(C/T)
7897	CakSNP7897	*Kabuli*	Ca_Kabuli_Chr05	11864959	(T/C)
7898	CakSNP7898	*Kabuli*	Ca_Kabuli_Chr05	12044714	(A/G)
7899	CakSNP7899	*Kabuli*	Ca_Kabuli_Chr05	12157678	(A/G)
7900	CakSNP7900	*Kabuli*	Ca_Kabuli_Chr05	12157683	(T/C)
7901	CakSNP7901	*Kabuli*	Ca_Kabuli_Chr05	12157688	(C/T)
7902	CakSNP7902	*Kabuli*	Ca_Kabuli_Chr05	12157706	(G/A)
7903	CakSNP7903	*Kabuli*	Ca_Kabuli_Chr05	12157707	(C/T)

S.N.	SNP IDs	Cultivars	Chromosomes/scaffolds	Physical positions (bp)	SNPs
7904	CakSNP7904	*Kabuli*	Ca_Kabuli_Chr05	12157710	(A/C)
7905	CakSNP7905	*Kabuli*	Ca_Kabuli_Chr05	12157742	(C/A)
7906	CakSNP7906	*Kabuli*	Ca_Kabuli_Chr05	12157699	(G/T)
7907	CakSNP7907	*Kabuli*	Ca_Kabuli_Chr05	12162108	(T/G)
7908	CakSNP7908	*Kabuli*	Ca_Kabuli_Chr05	12162156	(T/C)
7909	CakSNP7909	*Kabuli*	Ca_Kabuli_Chr05	12162160	(G/T)
7910	CakSNP7910	*Kabuli*	Ca_Kabuli_Chr05	12162164	(C/G)
7911	CakSNP7911	*Kabuli*	Ca_Kabuli_Chr05	12198014	(G/A)
7912	CakSNP7912	*Kabuli*	Ca_Kabuli_Chr05	12204521	(T/A)
7913	CakSNP7913	*Kabuli*	Ca_Kabuli_Chr05	12204588	(A/G)
7914	CakSNP7914	*Kabuli*	Ca_Kabuli_Chr05	12427931	(C/T)
7915	CakSNP7915	*Kabuli*	Ca_Kabuli_Chr05	12427940	(T/A)
7916	CakSNP7916	*Kabuli*	Ca_Kabuli_Chr05	12427970	(T/C)
7917	CakSNP7917	*Kabuli*	Ca_Kabuli_Chr05	12427969	(T/C)
7918	CakSNP7918	*Kabuli*	Ca_Kabuli_Chr05	12427966	(T/C)
7919	CakSNP7919	*Kabuli*	Ca_Kabuli_Chr05	12428027	(G/A)
7920	CakSNP7920	*Kabuli*	Ca_Kabuli_Chr05	12428121	(A/T)
7921	CakSNP7921	*Kabuli*	Ca_Kabuli_Chr05	12428120	(G/A)
7922	CakSNP7922	*Kabuli*	Ca_Kabuli_Chr05	12428102	(A/G)
7923	CakSNP7923	*Kabuli*	Ca_Kabuli_Chr05	12480948	(T/G)
7924	CakSNP7924	*Kabuli*	Ca_Kabuli_Chr05	12553469	(A/C)
7925	CakSNP7925	*Kabuli*	Ca_Kabuli_Chr05	12553470	(G/C)
7926	CakSNP7926	*Kabuli*	Ca_Kabuli_Chr05	12590330	(A/G)
7927	CakSNP7927	*Kabuli*	Ca_Kabuli_Chr05	12590361	(T/C)
7928	CakSNP7928	*Kabuli*	Ca_Kabuli_Chr05	12590478	(T/C)
7929	CakSNP7929	*Kabuli*	Ca_Kabuli_Chr05	12662138	(G/T)
7930	CakSNP7930	*Kabuli*	Ca_Kabuli_Chr05	13982000	(T/G)
7931	CakSNP7931	*Kabuli*	Ca_Kabuli_Chr05	13982041	(C/T)
7932	CakSNP7932	*Kabuli*	Ca_Kabuli_Chr05	15401230	(C/T)
7933	CakSNP7933	*Kabuli*	Ca_Kabuli_Chr05	15500782	(G/C)
7934	CakSNP7934	*Kabuli*	Ca_Kabuli_Chr05	15548719	(G/A)
7935	CakSNP7935	*Kabuli*	Ca_Kabuli_Chr05	15952395	(C/T)
7936	CakSNP7936	*Kabuli*	Ca_Kabuli_Chr05	16227319	(G/T)
7937	CakSNP7937	*Kabuli*	Ca_Kabuli_Chr05	16227321	(C/T)
7938	CakSNP7938	*Kabuli*	Ca_Kabuli_Chr05	16227353	(G/A)
7939	CakSNP7939	*Kabuli*	Ca_Kabuli_Chr05	16227395	(G/A)
7940	CakSNP7940	*Kabuli*	Ca_Kabuli_Chr05	16227401	(G/A)
7941	CakSNP7941	*Kabuli*	Ca_Kabuli_Chr05	16227408	(C/T)

S.N.	SNP IDs	Cultivars	Chromosomes/scaffolds	Physical positions (bp)	SNPs
7942	CakSNP7942	*Kabuli*	Ca_Kabuli_Chr05	16426578	(G/A)
7943	CakSNP7943	*Kabuli*	Ca_Kabuli_Chr05	16426575	(G/A)
7944	CakSNP7944	*Kabuli*	Ca_Kabuli_Chr05	16426549	(G/A)
7945	CakSNP7945	*Kabuli*	Ca_Kabuli_Chr05	16426662	(C/T)
7946	CakSNP7946	*Kabuli*	Ca_Kabuli_Chr05	16426657	(C/T)
7947	CakSNP7947	*Kabuli*	Ca_Kabuli_Chr05	16426656	(A/G)
7948	CakSNP7948	*Kabuli*	Ca_Kabuli_Chr05	16426642	(A/G)
7949	CakSNP7949	*Kabuli*	Ca_Kabuli_Chr05	16593880	(A/G)
7950	CakSNP7950	*Kabuli*	Ca_Kabuli_Chr05	16751536	(C/T)
7951	CakSNP7951	*Kabuli*	Ca_Kabuli_Chr05	16767134	(C/T)
7952	CakSNP7952	*Kabuli*	Ca_Kabuli_Chr05	16821406	(A/G)
7953	CakSNP7953	*Kabuli*	Ca_Kabuli_Chr05	16929903	(C/G)
7954	CakSNP7954	*Kabuli*	Ca_Kabuli_Chr05	16929897	(A/G)
7955	CakSNP7955	*Kabuli*	Ca_Kabuli_Chr05	17037918	(G/T)
7956	CakSNP7956	*Kabuli*	Ca_Kabuli_Chr05	17044340	(G/A)
7957	CakSNP7957	*Kabuli*	Ca_Kabuli_Chr05	17044338	(G/A)
7958	CakSNP7958	*Kabuli*	Ca_Kabuli_Chr05	17044332	(A/G)
7959	CakSNP7959	*Kabuli*	Ca_Kabuli_Chr05	17045235	(T/C)
7960	CakSNP7960	*Kabuli*	Ca_Kabuli_Chr05	17045244	(A/G)
7961	CakSNP7961	*Kabuli*	Ca_Kabuli_Chr05	17045485	(T/G)
7962	CakSNP7962	*Kabuli*	Ca_Kabuli_Chr05	17239612	(T/C)
7963	CakSNP7963	*Kabuli*	Ca_Kabuli_Chr05	17494714	(A/G)
7964	CakSNP7964	*Kabuli*	Ca_Kabuli_Chr05	17494702	(G/A)
7965	CakSNP7965	*Kabuli*	Ca_Kabuli_Chr05	17494700	(T/C)
7966	CakSNP7966	*Kabuli*	Ca_Kabuli_Chr05	17494697	(C/T)
7967	CakSNP7967	*Kabuli*	Ca_Kabuli_Chr05	17494672	(C/T)
7968	CakSNP7968	*Kabuli*	Ca_Kabuli_Chr05	17494699	(C/A)
7969	CakSNP7969	*Kabuli*	Ca_Kabuli_Chr05	17494705	(A/T)
7970	CakSNP7970	*Kabuli*	Ca_Kabuli_Chr05	17494710	(C/T)
7971	CakSNP7971	*Kabuli*	Ca_Kabuli_Chr05	17494731	(A/T)
7972	CakSNP7972	*Kabuli*	Ca_Kabuli_Chr05	18044808	(C/G)
7973	CakSNP7973	*Kabuli*	Ca_Kabuli_Chr05	18664706	(C/T)
7974	CakSNP7974	*Kabuli*	Ca_Kabuli_Chr05	18667782	(T/C)
7975	CakSNP7975	*Kabuli*	Ca_Kabuli_Chr05	18667775	(T/C)
7976	CakSNP7976	*Kabuli*	Ca_Kabuli_Chr05	18682788	(A/C)
7977	CakSNP7977	*Kabuli*	Ca_Kabuli_Chr05	18682792	(T/C)
7978	CakSNP7978	*Kabuli*	Ca_Kabuli_Chr05	18682793	(A/G)
7979	CakSNP7979	*Kabuli*	Ca_Kabuli_Chr05	18682794	(A/G)

S.N.	SNP IDs	Cultivars	Chromosomes/scaffolds	Physical positions (bp)	SNPs
7980	CakSNP7980	*Kabuli*	Ca_Kabuli_Chr05	18682812	(A/G)
7981	CakSNP7981	*Kabuli*	Ca_Kabuli_Chr05	18682814	(T/A)
7982	CakSNP7982	*Kabuli*	Ca_Kabuli_Chr05	18682822	(A/G)
7983	CakSNP7983	*Kabuli*	Ca_Kabuli_Chr05	18682827	(C/G)
7984	CakSNP7984	*Kabuli*	Ca_Kabuli_Chr05	18682830	(G/A)
7985	CakSNP7985	*Kabuli*	Ca_Kabuli_Chr05	18684334	(C/T)
7986	CakSNP7986	*Kabuli*	Ca_Kabuli_Chr05	18971085	(G/A)
7987	CakSNP7987	*Kabuli*	Ca_Kabuli_Chr05	18971088	(C/A)
7988	CakSNP7988	*Kabuli*	Ca_Kabuli_Chr05	19421820	(C/T)
7989	CakSNP7989	*Kabuli*	Ca_Kabuli_Chr05	19421868	(C/T)
7990	CakSNP7990	*Kabuli*	Ca_Kabuli_Chr05	19423387	(G/A)
7991	CakSNP7991	*Kabuli*	Ca_Kabuli_Chr05	19423357	(C/T)
7992	CakSNP7992	*Kabuli*	Ca_Kabuli_Chr05	19828124	(G/A)
7993	CakSNP7993	*Kabuli*	Ca_Kabuli_Chr05	19828151	(C/A)
7994	CakSNP7994	*Kabuli*	Ca_Kabuli_Chr05	19828163	(C/T)
7995	CakSNP7995	*Kabuli*	Ca_Kabuli_Chr05	19828166	(C/T)
7996	CakSNP7996	*Kabuli*	Ca_Kabuli_Chr05	19828167	(A/C)
7997	CakSNP7997	*Kabuli*	Ca_Kabuli_Chr05	19828226	(G/A)
7998	CakSNP7998	*Kabuli*	Ca_Kabuli_Chr05	19828190	(T/C)
7999	CakSNP7999	*Kabuli*	Ca_Kabuli_Chr05	19828184	(C/A)
8000	CakSNP8000	*Kabuli*	Ca_Kabuli_Chr05	19828172	(T/G)
8001	CakSNP8001	*Kabuli*	Ca_Kabuli_Chr05	19828144	(A/G)
8002	CakSNP8002	*Kabuli*	Ca_Kabuli_Chr05	19884132	(C/T)
8003	CakSNP8003	*Kabuli*	Ca_Kabuli_Chr05	19884124	(A/G)
8004	CakSNP8004	*Kabuli*	Ca_Kabuli_Chr05	20008545	(C/T)
8005	CakSNP8005	*Kabuli*	Ca_Kabuli_Chr05	20031711	(A/G)
8006	CakSNP8006	*Kabuli*	Ca_Kabuli_Chr05	20354352	(T/C)
8007	CakSNP8007	*Kabuli*	Ca_Kabuli_Chr05	20359674	(G/A)
8008	CakSNP8008	*Kabuli*	Ca_Kabuli_Chr05	20460857	(G/A)
8009	CakSNP8009	*Kabuli*	Ca_Kabuli_Chr05	20460859	(T/G)
8010	CakSNP8010	*Kabuli*	Ca_Kabuli_Chr05	20546402	(T/C)
8011	CakSNP8011	*Kabuli*	Ca_Kabuli_Chr05	20546385	(T/G)
8012	CakSNP8012	*Kabuli*	Ca_Kabuli_Chr05	20644984	(C/A)
8013	CakSNP8013	*Kabuli*	Ca_Kabuli_Chr05	20834519	(A/G)
8014	CakSNP8014	*Kabuli*	Ca_Kabuli_Chr05	21163371	(G/A)
8015	CakSNP8015	*Kabuli*	Ca_Kabuli_Chr05	21385948	(T/C)
8016	CakSNP8016	*Kabuli*	Ca_Kabuli_Chr05	21495583	(C/A)
8017	CakSNP8017	*Kabuli*	Ca_Kabuli_Chr05	21495631	(C/T)

S.N.	SNP IDs	Cultivars	Chromosomes/scaffolds	Physical positions (bp)	SNPs
8018	CakSNP8018	*Kabuli*	Ca_Kabuli_Chr05	21495672	(G/A)
8019	CakSNP8019	*Kabuli*	Ca_Kabuli_Chr05	21495707	(A/G)
8020	CakSNP8020	*Kabuli*	Ca_Kabuli_Chr05	21495679	(G/A)
8021	CakSNP8021	*Kabuli*	Ca_Kabuli_Chr05	21495674	(G/A)
8022	CakSNP8022	*Kabuli*	Ca_Kabuli_Chr05	21769327	(G/A)
8023	CakSNP8023	*Kabuli*	Ca_Kabuli_Chr05	21769419	(C/G)
8024	CakSNP8024	*Kabuli*	Ca_Kabuli_Chr05	21769794	(A/C)
8025	CakSNP8025	*Kabuli*	Ca_Kabuli_Chr05	22027215	(T/C)
8026	CakSNP8026	*Kabuli*	Ca_Kabuli_Chr05	22027257	(G/T)
8027	CakSNP8027	*Kabuli*	Ca_Kabuli_Chr05	22027265	(A/T)
8028	CakSNP8028	*Kabuli*	Ca_Kabuli_Chr05	22029684	(C/A)
8029	CakSNP8029	*Kabuli*	Ca_Kabuli_Chr05	22029686	(G/A)
8030	CakSNP8030	*Kabuli*	Ca_Kabuli_Chr05	22029761	(C/T)
8031	CakSNP8031	*Kabuli*	Ca_Kabuli_Chr05	22065518	(C/T)
8032	CakSNP8032	*Kabuli*	Ca_Kabuli_Chr05	22065859	(G/A)
8033	CakSNP8033	*Kabuli*	Ca_Kabuli_Chr05	22066034	(A/T)
8034	CakSNP8034	*Kabuli*	Ca_Kabuli_Chr05	22116961	(T/G)
8035	CakSNP8035	*Kabuli*	Ca_Kabuli_Chr05	22188341	(C/T)
8036	CakSNP8036	*Kabuli*	Ca_Kabuli_Chr05	22188318	(C/T)
8037	CakSNP8037	*Kabuli*	Ca_Kabuli_Chr05	22296731	(G/A)
8038	CakSNP8038	*Kabuli*	Ca_Kabuli_Chr05	22296728	(G/T)
8039	CakSNP8039	*Kabuli*	Ca_Kabuli_Chr05	22302350	(A/C)
8040	CakSNP8040	*Kabuli*	Ca_Kabuli_Chr05	22344868	(A/G)
8041	CakSNP8041	*Kabuli*	Ca_Kabuli_Chr05	22445954	(T/C)
8042	CakSNP8042	*Kabuli*	Ca_Kabuli_Chr05	22679380	(C/A)
8043	CakSNP8043	*Kabuli*	Ca_Kabuli_Chr05	23039312	(C/T)
8044	CakSNP8044	*Kabuli*	Ca_Kabuli_Chr05	23049683	(C/A)
8045	CakSNP8045	*Kabuli*	Ca_Kabuli_Chr05	23157899	(A/G)
8046	CakSNP8046	*Kabuli*	Ca_Kabuli_Chr05	23161492	(A/T)
8047	CakSNP8047	*Kabuli*	Ca_Kabuli_Chr05	23261964	(A/G)
8048	CakSNP8048	*Kabuli*	Ca_Kabuli_Chr05	23262114	(C/A)
8049	CakSNP8049	*Kabuli*	Ca_Kabuli_Chr05	23262264	(C/A)
8050	CakSNP8050	*Kabuli*	Ca_Kabuli_Chr05	23323538	(G/A)
8051	CakSNP8051	*Kabuli*	Ca_Kabuli_Chr05	23323520	(C/A)
8052	CakSNP8052	*Kabuli*	Ca_Kabuli_Chr05	23323514	(C/A)
8053	CakSNP8053	*Kabuli*	Ca_Kabuli_Chr05	23323479	(A/T)
8054	CakSNP8054	*Kabuli*	Ca_Kabuli_Chr05	23323478	(A/T)
8055	CakSNP8055	*Kabuli*	Ca_Kabuli_Chr05	23440293	(C/T)

S.N.	SNP IDs	Cultivars	Chromosomes/scaffolds	Physical positions (bp)	SNPs
8056	CakSNP8056	*Kabuli*	Ca_Kabuli_Chr05	23449048	(T/C)
8057	CakSNP8057	*Kabuli*	Ca_Kabuli_Chr05	23449134	(A/C)
8058	CakSNP8058	*Kabuli*	Ca_Kabuli_Chr05	23449127	(C/A)
8059	CakSNP8059	*Kabuli*	Ca_Kabuli_Chr05	23562595	(G/A)
8060	CakSNP8060	*Kabuli*	Ca_Kabuli_Chr05	23562827	(G/A)
8061	CakSNP8061	*Kabuli*	Ca_Kabuli_Chr05	23674814	(C/G)
8062	CakSNP8062	*Kabuli*	Ca_Kabuli_Chr05	23674815	(C/T)
8063	CakSNP8063	*Kabuli*	Ca_Kabuli_Chr05	23674845	(G/C)
8064	CakSNP8064	*Kabuli*	Ca_Kabuli_Chr05	23783140	(T/C)
8065	CakSNP8065	*Kabuli*	Ca_Kabuli_Chr05	23783156	(C/A)
8066	CakSNP8066	*Kabuli*	Ca_Kabuli_Chr05	23783157	(G/C)
8067	CakSNP8067	*Kabuli*	Ca_Kabuli_Chr05	23783188	(G/A)
8068	CakSNP8068	*Kabuli*	Ca_Kabuli_Chr05	23783195	(C/G)
8069	CakSNP8069	*Kabuli*	Ca_Kabuli_Chr05	23783254	(A/G)
8070	CakSNP8070	*Kabuli*	Ca_Kabuli_Chr05	23783252	(T/A)
8071	CakSNP8071	*Kabuli*	Ca_Kabuli_Chr05	23884909	(C/T)
8072	CakSNP8072	*Kabuli*	Ca_Kabuli_Chr05	23884936	(G/A)
8073	CakSNP8073	*Kabuli*	Ca_Kabuli_Chr05	23930933	(C/T)
8074	CakSNP8074	*Kabuli*	Ca_Kabuli_Chr05	23930988	(G/A)
8075	CakSNP8075	*Kabuli*	Ca_Kabuli_Chr05	23932354	(C/T)
8076	CakSNP8076	*Kabuli*	Ca_Kabuli_Chr05	23934198	(G/T)
8077	CakSNP8077	*Kabuli*	Ca_Kabuli_Chr05	24059182	(C/G)
8078	CakSNP8078	*Kabuli*	Ca_Kabuli_Chr05	24090533	(C/T)
8079	CakSNP8079	*Kabuli*	Ca_Kabuli_Chr05	24090523	(A/C)
8080	CakSNP8080	*Kabuli*	Ca_Kabuli_Chr05	24090515	(A/C)
8081	CakSNP8081	*Kabuli*	Ca_Kabuli_Chr05	24090749	(A/C)
8082	CakSNP8082	*Kabuli*	Ca_Kabuli_Chr05	24097823	(T/C)
8083	CakSNP8083	*Kabuli*	Ca_Kabuli_Chr05	24153061	(G/C)
8084	CakSNP8084	*Kabuli*	Ca_Kabuli_Chr05	24153205	(T/C)
8085	CakSNP8085	*Kabuli*	Ca_Kabuli_Chr05	24153273	(T/C)
8086	CakSNP8086	*Kabuli*	Ca_Kabuli_Chr05	24153202	(A/C)
8087	CakSNP8087	*Kabuli*	Ca_Kabuli_Chr05	24153199	(C/T)
8088	CakSNP8088	*Kabuli*	Ca_Kabuli_Chr05	24166103	(C/T)
8089	CakSNP8089	*Kabuli*	Ca_Kabuli_Chr05	24166095	(G/A)
8090	CakSNP8090	*Kabuli*	Ca_Kabuli_Chr05	24166093	(A/G)
8091	CakSNP8091	*Kabuli*	Ca_Kabuli_Chr05	24166046	(G/A)
8092	CakSNP8092	*Kabuli*	Ca_Kabuli_Chr05	24166047	(C/T)
8093	CakSNP8093	*Kabuli*	Ca_Kabuli_Chr05	24166082	(G/A)

S.N.	SNP IDs	Cultivars	Chromosomes/scaffolds	Physical positions (bp)	SNPs
8094	CakSNP8094	*Kabuli*	Ca_Kabuli_Chr05	24181232	(C/A)
8095	CakSNP8095	*Kabuli*	Ca_Kabuli_Chr05	24181228	(C/A)
8096	CakSNP8096	*Kabuli*	Ca_Kabuli_Chr05	24245312	(C/A)
8097	CakSNP8097	*Kabuli*	Ca_Kabuli_Chr05	24245308	(C/A)
8098	CakSNP8098	*Kabuli*	Ca_Kabuli_Chr05	24346350	(A/T)
8099	CakSNP8099	*Kabuli*	Ca_Kabuli_Chr05	24576960	(C/T)
8100	CakSNP8100	*Kabuli*	Ca_Kabuli_Chr05	24576970	(A/G)
8101	CakSNP8101	*Kabuli*	Ca_Kabuli_Chr05	24576993	(T/G)
8102	CakSNP8102	*Kabuli*	Ca_Kabuli_Chr05	24596227	(T/C)
8103	CakSNP8103	*Kabuli*	Ca_Kabuli_Chr05	24596257	(C/T)
8104	CakSNP8104	*Kabuli*	Ca_Kabuli_Chr05	24596269	(G/A)
8105	CakSNP8105	*Kabuli*	Ca_Kabuli_Chr05	24605438	(A/C)
8106	CakSNP8106	*Kabuli*	Ca_Kabuli_Chr05	24843015	(G/T)
8107	CakSNP8107	*Kabuli*	Ca_Kabuli_Chr05	25003171	(C/T)
8108	CakSNP8108	*Kabuli*	Ca_Kabuli_Chr05	25006110	(A/C)
8109	CakSNP8109	*Kabuli*	Ca_Kabuli_Chr05	25006123	(C/T)
8110	CakSNP8110	*Kabuli*	Ca_Kabuli_Chr05	25184089	(T/C)
8111	CakSNP8111	*Kabuli*	Ca_Kabuli_Chr05	25184133	(G/A)
8112	CakSNP8112	*Kabuli*	Ca_Kabuli_Chr05	25399115	(T/G)
8113	CakSNP8113	*Kabuli*	Ca_Kabuli_Chr05	25400604	(A/G)
8114	CakSNP8114	*Kabuli*	Ca_Kabuli_Chr05	25474110	(A/C)
8115	CakSNP8115	*Kabuli*	Ca_Kabuli_Chr05	25474105	(C/T)
8116	CakSNP8116	*Kabuli*	Ca_Kabuli_Chr05	25474093	(G/C)
8117	CakSNP8117	*Kabuli*	Ca_Kabuli_Chr05	25474089	(C/A)
8118	CakSNP8118	*Kabuli*	Ca_Kabuli_Chr05	25597077	(A/G)
8119	CakSNP8119	*Kabuli*	Ca_Kabuli_Chr05	25678975	(G/T)
8120	CakSNP8120	*Kabuli*	Ca_Kabuli_Chr05	25697487	(C/G)
8121	CakSNP8121	*Kabuli*	Ca_Kabuli_Chr05	25805020	(A/G)
8122	CakSNP8122	*Kabuli*	Ca_Kabuli_Chr05	26025243	(C/T)
8123	CakSNP8123	*Kabuli*	Ca_Kabuli_Chr05	26025282	(A/G)
8124	CakSNP8124	*Kabuli*	Ca_Kabuli_Chr05	26025356	(A/T)
8125	CakSNP8125	*Kabuli*	Ca_Kabuli_Chr05	26027313	(C/A)
8126	CakSNP8126	*Kabuli*	Ca_Kabuli_Chr05	26095084	(T/G)
8127	CakSNP8127	*Kabuli*	Ca_Kabuli_Chr05	26124240	(T/C)
8128	CakSNP8128	*Kabuli*	Ca_Kabuli_Chr05	26191896	(T/C)
8129	CakSNP8129	*Kabuli*	Ca_Kabuli_Chr05	26194579	(A/G)
8130	CakSNP8130	*Kabuli*	Ca_Kabuli_Chr05	26238764	(T/G)
8131	CakSNP8131	*Kabuli*	Ca_Kabuli_Chr05	26238752	(A/T)

S.N.	SNP IDs	Cultivars	Chromosomes/scaffolds	Physical positions (bp)	SNPs
8132	CakSNP8132	*Kabuli*	Ca_Kabuli_Chr05	26238748	(A/T)
8133	CakSNP8133	*Kabuli*	Ca_Kabuli_Chr05	26238741	(G/T)
8134	CakSNP8134	*Kabuli*	Ca_Kabuli_Chr05	26238739	(C/T)
8135	CakSNP8135	*Kabuli*	Ca_Kabuli_Chr05	26384636	(C/G)
8136	CakSNP8136	*Kabuli*	Ca_Kabuli_Chr05	26467869	(A/C)
8137	CakSNP8137	*Kabuli*	Ca_Kabuli_Chr05	26474910	(A/C)
8138	CakSNP8138	*Kabuli*	Ca_Kabuli_Chr05	26475067	(T/C)
8139	CakSNP8139	*Kabuli*	Ca_Kabuli_Chr05	26477751	(A/T)
8140	CakSNP8140	*Kabuli*	Ca_Kabuli_Chr05	26494631	(A/G)
8141	CakSNP8141	*Kabuli*	Ca_Kabuli_Chr05	26494778	(T/C)
8142	CakSNP8142	*Kabuli*	Ca_Kabuli_Chr05	26494769	(T/C)
8143	CakSNP8143	*Kabuli*	Ca_Kabuli_Chr05	26496197	(A/G)
8144	CakSNP8144	*Kabuli*	Ca_Kabuli_Chr05	26558406	(A/G)
8145	CakSNP8145	*Kabuli*	Ca_Kabuli_Chr05	26558366	(A/G)
8146	CakSNP8146	*Kabuli*	Ca_Kabuli_Chr05	26558363	(C/G)
8147	CakSNP8147	*Kabuli*	Ca_Kabuli_Chr05	26558357	(G/A)
8148	CakSNP8148	*Kabuli*	Ca_Kabuli_Chr05	26558356	(G/T)
8149	CakSNP8149	*Kabuli*	Ca_Kabuli_Chr05	26569400	(G/C)
8150	CakSNP8150	*Kabuli*	Ca_Kabuli_Chr05	26609014	(T/C)
8151	CakSNP8151	*Kabuli*	Ca_Kabuli_Chr05	26608993	(A/G)
8152	CakSNP8152	*Kabuli*	Ca_Kabuli_Chr05	26621093	(C/G)
8153	CakSNP8153	*Kabuli*	Ca_Kabuli_Chr05	26626861	(A/G)
8154	CakSNP8154	*Kabuli*	Ca_Kabuli_Chr05	26626855	(T/G)
8155	CakSNP8155	*Kabuli*	Ca_Kabuli_Chr05	26638041	(G/A)
8156	CakSNP8156	*Kabuli*	Ca_Kabuli_Chr05	26638042	(C/T)
8157	CakSNP8157	*Kabuli*	Ca_Kabuli_Chr05	26638080	(A/G)
8158	CakSNP8158	*Kabuli*	Ca_Kabuli_Chr05	26638126	(C/T)
8159	CakSNP8159	*Kabuli*	Ca_Kabuli_Chr05	26638118	(C/T)
8160	CakSNP8160	*Kabuli*	Ca_Kabuli_Chr05	26679836	(C/T)
8161	CakSNP8161	*Kabuli*	Ca_Kabuli_Chr05	26696390	(G/A)
8162	CakSNP8162	*Kabuli*	Ca_Kabuli_Chr05	26696412	(C/A)
8163	CakSNP8163	*Kabuli*	Ca_Kabuli_Chr05	26696455	(G/A)
8164	CakSNP8164	*Kabuli*	Ca_Kabuli_Chr05	26696547	(C/T)
8165	CakSNP8165	*Kabuli*	Ca_Kabuli_Chr05	26696533	(C/T)
8166	CakSNP8166	*Kabuli*	Ca_Kabuli_Chr05	26696494	(G/A)
8167	CakSNP8167	*Kabuli*	Ca_Kabuli_Chr05	26696578	(G/A)
8168	CakSNP8168	*Kabuli*	Ca_Kabuli_Chr05	26696602	(C/T)
8169	CakSNP8169	*Kabuli*	Ca_Kabuli_Chr05	26696620	(C/T)

S.N.	SNP IDs	Cultivars	Chromosomes/scaffolds	Physical positions (bp)	SNPs
8170	CakSNP8170	*Kabuli*	Ca_Kabuli_Chr05	26696624	(G/A)
8171	CakSNP8171	*Kabuli*	Ca_Kabuli_Chr05	26785140	(G/A)
8172	CakSNP8172	*Kabuli*	Ca_Kabuli_Chr05	26785226	(C/A)
8173	CakSNP8173	*Kabuli*	Ca_Kabuli_Chr05	26997335	(T/G)
8174	CakSNP8174	*Kabuli*	Ca_Kabuli_Chr05	26999793	(C/T)
8175	CakSNP8175	*Kabuli*	Ca_Kabuli_Chr05	27000127	(C/G)
8176	CakSNP8176	*Kabuli*	Ca_Kabuli_Chr05	27000104	(C/A)
8177	CakSNP8177	*Kabuli*	Ca_Kabuli_Chr05	27000278	(T/A)
8178	CakSNP8178	*Kabuli*	Ca_Kabuli_Chr05	27000280	(T/G)
8179	CakSNP8179	*Kabuli*	Ca_Kabuli_Chr05	27025766	(A/G)
8180	CakSNP8180	*Kabuli*	Ca_Kabuli_Chr05	27025752	(A/C)
8181	CakSNP8181	*Kabuli*	Ca_Kabuli_Chr05	27042679	(G/A)
8182	CakSNP8182	*Kabuli*	Ca_Kabuli_Chr05	27117953	(A/G)
8183	CakSNP8183	*Kabuli*	Ca_Kabuli_Chr05	27150550	(A/G)
8184	CakSNP8184	*Kabuli*	Ca_Kabuli_Chr05	27230515	(T/A)
8185	CakSNP8185	*Kabuli*	Ca_Kabuli_Chr05	27230514	(A/G)
8186	CakSNP8186	*Kabuli*	Ca_Kabuli_Chr05	27231394	(T/C)
8187	CakSNP8187	*Kabuli*	Ca_Kabuli_Chr05	27231641	(T/A)
8188	CakSNP8188	*Kabuli*	Ca_Kabuli_Chr05	27232304	(T/A)
8189	CakSNP8189	*Kabuli*	Ca_Kabuli_Chr05	27234806	(G/A)
8190	CakSNP8190	*Kabuli*	Ca_Kabuli_Chr05	27235002	(T/G)
8191	CakSNP8191	*Kabuli*	Ca_Kabuli_Chr05	27328552	(G/A)
8192	CakSNP8192	*Kabuli*	Ca_Kabuli_Chr05	27328737	(G/T)
8193	CakSNP8193	*Kabuli*	Ca_Kabuli_Chr05	27334056	(A/C)
8194	CakSNP8194	*Kabuli*	Ca_Kabuli_Chr05	27334057	(G/C)
8195	CakSNP8195	*Kabuli*	Ca_Kabuli_Chr05	27334061	(G/C)
8196	CakSNP8196	*Kabuli*	Ca_Kabuli_Chr05	27349210	(T/A)
8197	CakSNP8197	*Kabuli*	Ca_Kabuli_Chr05	27349237	(C/T)
8198	CakSNP8198	*Kabuli*	Ca_Kabuli_Chr05	27361515	(C/T)
8199	CakSNP8199	*Kabuli*	Ca_Kabuli_Chr05	27361579	(T/G)
8200	CakSNP8200	*Kabuli*	Ca_Kabuli_Chr05	27362517	(C/G)
8201	CakSNP8201	*Kabuli*	Ca_Kabuli_Chr05	27362570	(G/T)
8202	CakSNP8202	*Kabuli*	Ca_Kabuli_Chr05	27362573	(A/G)
8203	CakSNP8203	*Kabuli*	Ca_Kabuli_Chr05	27362584	(T/G)
8204	CakSNP8204	*Kabuli*	Ca_Kabuli_Chr05	27452484	(C/G)
8205	CakSNP8205	*Kabuli*	Ca_Kabuli_Chr05	27516054	(T/A)
8206	CakSNP8206	*Kabuli*	Ca_Kabuli_Chr05	27552106	(A/C)
8207	CakSNP8207	*Kabuli*	Ca_Kabuli_Chr05	27555637	(T/G)

S.N.	SNP IDs	Cultivars	Chromosomes/scaffolds	Physical positions (bp)	SNPs
8208	CakSNP8208	*Kabuli*	Ca_Kabuli_Chr05	27568101	(T/G)
8209	CakSNP8209	*Kabuli*	Ca_Kabuli_Chr05	27568095	(C/A)
8210	CakSNP8210	*Kabuli*	Ca_Kabuli_Chr05	27568064	(A/C)
8211	CakSNP8211	*Kabuli*	Ca_Kabuli_Chr05	27568059	(C/T)
8212	CakSNP8212	*Kabuli*	Ca_Kabuli_Chr05	27568028	(A/T)
8213	CakSNP8213	*Kabuli*	Ca_Kabuli_Chr05	27568033	(G/A)
8214	CakSNP8214	*Kabuli*	Ca_Kabuli_Chr05	27568082	(G/A)
8215	CakSNP8215	*Kabuli*	Ca_Kabuli_Chr05	27568097	(G/A)
8216	CakSNP8216	*Kabuli*	Ca_Kabuli_Chr05	27568098	(C/T)
8217	CakSNP8217	*Kabuli*	Ca_Kabuli_Chr05	27568118	(A/G)
8218	CakSNP8218	*Kabuli*	Ca_Kabuli_Chr05	27568133	(G/T)
8219	CakSNP8219	*Kabuli*	Ca_Kabuli_Chr05	27568081	(G/C)
8220	CakSNP8220	*Kabuli*	Ca_Kabuli_Chr05	27568107	(C/G)
8221	CakSNP8221	*Kabuli*	Ca_Kabuli_Chr05	27568156	(C/G)
8222	CakSNP8222	*Kabuli*	Ca_Kabuli_Chr05	27568141	(C/T)
8223	CakSNP8223	*Kabuli*	Ca_Kabuli_Chr05	27568180	(C/T)
8224	CakSNP8224	*Kabuli*	Ca_Kabuli_Chr05	27568176	(T/C)
8225	CakSNP8225	*Kabuli*	Ca_Kabuli_Chr05	27579347	(C/T)
8226	CakSNP8226	*Kabuli*	Ca_Kabuli_Chr05	27605037	(A/T)
8227	CakSNP8227	*Kabuli*	Ca_Kabuli_Chr05	27605063	(T/A)
8228	CakSNP8228	*Kabuli*	Ca_Kabuli_Chr05	27605081	(C/T)
8229	CakSNP8229	*Kabuli*	Ca_Kabuli_Chr05	27605095	(G/C)
8230	CakSNP8230	*Kabuli*	Ca_Kabuli_Chr05	27692816	(C/T)
8231	CakSNP8231	*Kabuli*	Ca_Kabuli_Chr05	27692818	(C/T)
8232	CakSNP8232	*Kabuli*	Ca_Kabuli_Chr05	27692822	(C/T)
8233	CakSNP8233	*Kabuli*	Ca_Kabuli_Chr05	27692823	(A/T)
8234	CakSNP8234	*Kabuli*	Ca_Kabuli_Chr05	27692831	(A/T)
8235	CakSNP8235	*Kabuli*	Ca_Kabuli_Chr05	27694205	(C/A)
8236	CakSNP8236	*Kabuli*	Ca_Kabuli_Chr05	27750155	(C/T)
8237	CakSNP8237	*Kabuli*	Ca_Kabuli_Chr05	27786025	(T/C)
8238	CakSNP8238	*Kabuli*	Ca_Kabuli_Chr05	27838003	(T/C)
8239	CakSNP8239	*Kabuli*	Ca_Kabuli_Chr05	27850760	(T/C)
8240	CakSNP8240	*Kabuli*	Ca_Kabuli_Chr05	27850735	(C/A)
8241	CakSNP8241	*Kabuli*	Ca_Kabuli_Chr05	27853444	(C/T)
8242	CakSNP8242	*Kabuli*	Ca_Kabuli_Chr05	27872968	(C/T)
8243	CakSNP8243	*Kabuli*	Ca_Kabuli_Chr05	27873001	(T/C)
8244	CakSNP8244	*Kabuli*	Ca_Kabuli_Chr05	27874414	(C/A)
8245	CakSNP8245	*Kabuli*	Ca_Kabuli_Chr05	27936521	(A/G)

S.N.	SNP IDs	Cultivars	Chromosomes/scaffolds	Physical positions (bp)	SNPs
8246	CakSNP8246	*Kabuli*	Ca_Kabuli_Chr05	27936524	(C/T)
8247	CakSNP8247	*Kabuli*	Ca_Kabuli_Chr05	27939162	(G/A)
8248	CakSNP8248	*Kabuli*	Ca_Kabuli_Chr05	27939135	(G/C)
8249	CakSNP8249	*Kabuli*	Ca_Kabuli_Chr05	27939134	(A/T)
8250	CakSNP8250	*Kabuli*	Ca_Kabuli_Chr05	27939128	(A/G)
8251	CakSNP8251	*Kabuli*	Ca_Kabuli_Chr05	27974691	(G/T)
8252	CakSNP8252	*Kabuli*	Ca_Kabuli_Chr05	28016628	(C/T)
8253	CakSNP8253	*Kabuli*	Ca_Kabuli_Chr05	28016669	(A/G)
8254	CakSNP8254	*Kabuli*	Ca_Kabuli_Chr05	28071467	(A/G)
8255	CakSNP8255	*Kabuli*	Ca_Kabuli_Chr05	28116818	(A/G)
8256	CakSNP8256	*Kabuli*	Ca_Kabuli_Chr05	28116988	(A/T)
8257	CakSNP8257	*Kabuli*	Ca_Kabuli_Chr05	28117011	(C/T)
8258	CakSNP8258	*Kabuli*	Ca_Kabuli_Chr05	28164985	(G/C)
8259	CakSNP8259	*Kabuli*	Ca_Kabuli_Chr05	28168910	(A/G)
8260	CakSNP8260	*Kabuli*	Ca_Kabuli_Chr05	28169475	(T/C)
8261	CakSNP8261	*Kabuli*	Ca_Kabuli_Chr05	28175039	(A/G)
8262	CakSNP8262	*Kabuli*	Ca_Kabuli_Chr05	28266046	(T/G)
8263	CakSNP8263	*Kabuli*	Ca_Kabuli_Chr05	28271638	(G/C)
8264	CakSNP8264	*Kabuli*	Ca_Kabuli_Chr05	28271732	(C/T)
8265	CakSNP8265	*Kabuli*	Ca_Kabuli_Chr05	28271733	(G/A)
8266	CakSNP8266	*Kabuli*	Ca_Kabuli_Chr05	28271742	(G/A)
8267	CakSNP8267	*Kabuli*	Ca_Kabuli_Chr05	28271767	(G/C)
8268	CakSNP8268	*Kabuli*	Ca_Kabuli_Chr05	28284640	(T/C)
8269	CakSNP8269	*Kabuli*	Ca_Kabuli_Chr05	28327832	(T/G)
8270	CakSNP8270	*Kabuli*	Ca_Kabuli_Chr05	28327883	(G/A)
8271	CakSNP8271	*Kabuli*	Ca_Kabuli_Chr05	28327870	(T/C)
8272	CakSNP8272	*Kabuli*	Ca_Kabuli_Chr05	28327868	(G/A)
8273	CakSNP8273	*Kabuli*	Ca_Kabuli_Chr05	28327856	(T/C)
8274	CakSNP8274	*Kabuli*	Ca_Kabuli_Chr05	28335455	(A/C)
8275	CakSNP8275	*Kabuli*	Ca_Kabuli_Chr05	28346497	(C/T)
8276	CakSNP8276	*Kabuli*	Ca_Kabuli_Chr05	28360495	(C/T)
8277	CakSNP8277	*Kabuli*	Ca_Kabuli_Chr05	28360492	(C/T)
8278	CakSNP8278	*Kabuli*	Ca_Kabuli_Chr05	28360585	(A/G)
8279	CakSNP8279	*Kabuli*	Ca_Kabuli_Chr05	28383374	(G/T)
8280	CakSNP8280	*Kabuli*	Ca_Kabuli_Chr05	28491113	(G/A)
8281	CakSNP8281	*Kabuli*	Ca_Kabuli_Chr05	28491131	(G/A)
8282	CakSNP8282	*Kabuli*	Ca_Kabuli_Chr05	28509048	(G/A)
8283	CakSNP8283	*Kabuli*	Ca_Kabuli_Chr05	28509066	(G/A)

S.N.	SNP IDs	Cultivars	Chromosomes/scaffolds	Physical positions (bp)	SNPs
8284	CakSNP8284	*Kabuli*	Ca_Kabuli_Chr05	28562734	(G/A)
8285	CakSNP8285	*Kabuli*	Ca_Kabuli_Chr05	28581814	(A/G)
8286	CakSNP8286	*Kabuli*	Ca_Kabuli_Chr05	28581815	(C/G)
8287	CakSNP8287	*Kabuli*	Ca_Kabuli_Chr05	28581827	(T/C)
8288	CakSNP8288	*Kabuli*	Ca_Kabuli_Chr05	28581866	(T/C)
8289	CakSNP8289	*Kabuli*	Ca_Kabuli_Chr05	28581926	(C/A)
8290	CakSNP8290	*Kabuli*	Ca_Kabuli_Chr05	28582525	(C/T)
8291	CakSNP8291	*Kabuli*	Ca_Kabuli_Chr05	28582806	(T/C)
8292	CakSNP8292	*Kabuli*	Ca_Kabuli_Chr05	28582763	(A/G)
8293	CakSNP8293	*Kabuli*	Ca_Kabuli_Chr05	28583402	(G/A)
8294	CakSNP8294	*Kabuli*	Ca_Kabuli_Chr05	28583375	(C/G)
8295	CakSNP8295	*Kabuli*	Ca_Kabuli_Chr05	28682973	(A/C)
8296	CakSNP8296	*Kabuli*	Ca_Kabuli_Chr05	28682995	(G/A)
8297	CakSNP8297	*Kabuli*	Ca_Kabuli_Chr05	28683084	(A/C)
8298	CakSNP8298	*Kabuli*	Ca_Kabuli_Chr05	28683070	(T/A)
8299	CakSNP8299	*Kabuli*	Ca_Kabuli_Chr05	28683069	(A/T)
8300	CakSNP8300	*Kabuli*	Ca_Kabuli_Chr05	28714658	(C/T)
8301	CakSNP8301	*Kabuli*	Ca_Kabuli_Chr05	28717362	(C/T)
8302	CakSNP8302	*Kabuli*	Ca_Kabuli_Chr05	28717384	(C/T)
8303	CakSNP8303	*Kabuli*	Ca_Kabuli_Chr05	28721236	(C/T)
8304	CakSNP8304	*Kabuli*	Ca_Kabuli_Chr05	28721237	(A/G)
8305	CakSNP8305	*Kabuli*	Ca_Kabuli_Chr05	28721386	(T/C)
8306	CakSNP8306	*Kabuli*	Ca_Kabuli_Chr05	28721383	(A/C)
8307	CakSNP8307	*Kabuli*	Ca_Kabuli_Chr05	28746139	(A/G)
8308	CakSNP8308	*Kabuli*	Ca_Kabuli_Chr05	28757467	(A/T)
8309	CakSNP8309	*Kabuli*	Ca_Kabuli_Chr05	28757474	(T/C)
8310	CakSNP8310	*Kabuli*	Ca_Kabuli_Chr05	28757479	(T/C)
8311	CakSNP8311	*Kabuli*	Ca_Kabuli_Chr05	28757485	(A/C)
8312	CakSNP8312	*Kabuli*	Ca_Kabuli_Chr05	28757507	(A/T)
8313	CakSNP8313	*Kabuli*	Ca_Kabuli_Chr05	28758723	(A/G)
8314	CakSNP8314	*Kabuli*	Ca_Kabuli_Chr05	28817832	(A/G)
8315	CakSNP8315	*Kabuli*	Ca_Kabuli_Chr05	28827467	(A/G)
8316	CakSNP8316	*Kabuli*	Ca_Kabuli_Chr05	28843854	(G/A)
8317	CakSNP8317	*Kabuli*	Ca_Kabuli_Chr05	28843801	(A/G)
8318	CakSNP8318	*Kabuli*	Ca_Kabuli_Chr05	28843795	(A/T)
8319	CakSNP8319	*Kabuli*	Ca_Kabuli_Chr05	28843782	(C/G)
8320	CakSNP8320	*Kabuli*	Ca_Kabuli_Chr05	28843775	(C/T)
8321	CakSNP8321	*Kabuli*	Ca_Kabuli_Chr05	28854486	(C/T)

S.N.	SNP IDs	Cultivars	Chromosomes/scaffolds	Physical positions (bp)	SNPs
8322	CakSNP8322	*Kabuli*	Ca_Kabuli_Chr05	28905203	(A/C)
8323	CakSNP8323	*Kabuli*	Ca_Kabuli_Chr05	29007895	(T/C)
8324	CakSNP8324	*Kabuli*	Ca_Kabuli_Chr05	29007886	(C/A)
8325	CakSNP8325	*Kabuli*	Ca_Kabuli_Chr05	29007884	(A/C)
8326	CakSNP8326	*Kabuli*	Ca_Kabuli_Chr05	29007882	(A/C)
8327	CakSNP8327	*Kabuli*	Ca_Kabuli_Chr05	29007879	(T/C)
8328	CakSNP8328	*Kabuli*	Ca_Kabuli_Chr05	29007847	(A/C)
8329	CakSNP8329	*Kabuli*	Ca_Kabuli_Chr05	29007844	(T/C)
8330	CakSNP8330	*Kabuli*	Ca_Kabuli_Chr05	29061056	(T/A)
8331	CakSNP8331	*Kabuli*	Ca_Kabuli_Chr05	29061025	(A/C)
8332	CakSNP8332	*Kabuli*	Ca_Kabuli_Chr05	29060992	(C/T)
8333	CakSNP8333	*Kabuli*	Ca_Kabuli_Chr05	29062696	(G/A)
8334	CakSNP8334	*Kabuli*	Ca_Kabuli_Chr05	29062738	(A/G)
8335	CakSNP8335	*Kabuli*	Ca_Kabuli_Chr05	29079931	(G/A)
8336	CakSNP8336	*Kabuli*	Ca_Kabuli_Chr05	29079962	(A/G)
8337	CakSNP8337	*Kabuli*	Ca_Kabuli_Chr05	29080066	(G/C)
8338	CakSNP8338	*Kabuli*	Ca_Kabuli_Chr05	29080060	(C/T)
8339	CakSNP8339	*Kabuli*	Ca_Kabuli_Chr05	29111292	(T/G)
8340	CakSNP8340	*Kabuli*	Ca_Kabuli_Chr05	29111293	(T/C)
8341	CakSNP8341	*Kabuli*	Ca_Kabuli_Chr05	29186179	(T/G)
8342	CakSNP8342	*Kabuli*	Ca_Kabuli_Chr05	29220980	(T/A)
8343	CakSNP8343	*Kabuli*	Ca_Kabuli_Chr05	29221003	(T/C)
8344	CakSNP8344	*Kabuli*	Ca_Kabuli_Chr05	29221919	(T/A)
8345	CakSNP8345	*Kabuli*	Ca_Kabuli_Chr05	29272117	(C/G)
8346	CakSNP8346	*Kabuli*	Ca_Kabuli_Chr05	29272125	(C/T)
8347	CakSNP8347	*Kabuli*	Ca_Kabuli_Chr05	29354269	(A/T)
8348	CakSNP8348	*Kabuli*	Ca_Kabuli_Chr05	29422029	(T/A)
8349	CakSNP8349	*Kabuli*	Ca_Kabuli_Chr05	29422066	(A/T)
8350	CakSNP8350	*Kabuli*	Ca_Kabuli_Chr05	29422091	(C/T)
8351	CakSNP8351	*Kabuli*	Ca_Kabuli_Chr05	29437139	(C/T)
8352	CakSNP8352	*Kabuli*	Ca_Kabuli_Chr05	29437146	(C/A)
8353	CakSNP8353	*Kabuli*	Ca_Kabuli_Chr05	29437159	(C/T)
8354	CakSNP8354	*Kabuli*	Ca_Kabuli_Chr05	29437162	(A/C)
8355	CakSNP8355	*Kabuli*	Ca_Kabuli_Chr05	29437171	(A/T)
8356	CakSNP8356	*Kabuli*	Ca_Kabuli_Chr05	29437181	(A/C)
8357	CakSNP8357	*Kabuli*	Ca_Kabuli_Chr05	29437189	(C/T)
8358	CakSNP8358	*Kabuli*	Ca_Kabuli_Chr05	29437193	(A/C)
8359	CakSNP8359	*Kabuli*	Ca_Kabuli_Chr05	29437182	(C/A)

S.N.	SNP IDs	Cultivars	Chromosomes/scaffolds	Physical positions (bp)	SNPs
8360	CakSNP8360	*Kabuli*	Ca_Kabuli_Chr05	29528859	(A/T)
8361	CakSNP8361	*Kabuli*	Ca_Kabuli_Chr05	29528890	(T/A)
8362	CakSNP8362	*Kabuli*	Ca_Kabuli_Chr05	29528904	(T/G)
8363	CakSNP8363	*Kabuli*	Ca_Kabuli_Chr05	29530996	(G/A)
8364	CakSNP8364	*Kabuli*	Ca_Kabuli_Chr05	29531122	(A/T)
8365	CakSNP8365	*Kabuli*	Ca_Kabuli_Chr05	29531113	(G/A)
8366	CakSNP8366	*Kabuli*	Ca_Kabuli_Chr05	29531218	(A/T)
8367	CakSNP8367	*Kabuli*	Ca_Kabuli_Chr05	29531416	(A/G)
8368	CakSNP8368	*Kabuli*	Ca_Kabuli_Chr05	29567676	(T/C)
8369	CakSNP8369	*Kabuli*	Ca_Kabuli_Chr05	29567820	(T/C)
8370	CakSNP8370	*Kabuli*	Ca_Kabuli_Chr05	29576690	(A/T)
8371	CakSNP8371	*Kabuli*	Ca_Kabuli_Chr05	29576689	(G/A)
8372	CakSNP8372	*Kabuli*	Ca_Kabuli_Chr05	29576716	(A/C)
8373	CakSNP8373	*Kabuli*	Ca_Kabuli_Chr05	29608657	(T/C)
8374	CakSNP8374	*Kabuli*	Ca_Kabuli_Chr05	29641659	(T/C)
8375	CakSNP8375	*Kabuli*	Ca_Kabuli_Chr05	29658258	(G/C)
8376	CakSNP8376	*Kabuli*	Ca_Kabuli_Chr05	29658280	(A/C)
8377	CakSNP8377	*Kabuli*	Ca_Kabuli_Chr05	29667787	(C/G)
8378	CakSNP8378	*Kabuli*	Ca_Kabuli_Chr05	29667783	(C/T)
8379	CakSNP8379	*Kabuli*	Ca_Kabuli_Chr05	29667774	(G/C)
8380	CakSNP8380	*Kabuli*	Ca_Kabuli_Chr05	29809862	(A/C)
8381	CakSNP8381	*Kabuli*	Ca_Kabuli_Chr05	29816799	(T/C)
8382	CakSNP8382	*Kabuli*	Ca_Kabuli_Chr05	29816800	(T/A)
8383	CakSNP8383	*Kabuli*	Ca_Kabuli_Chr05	29824218	(T/G)
8384	CakSNP8384	*Kabuli*	Ca_Kabuli_Chr05	29852130	(T/C)
8385	CakSNP8385	*Kabuli*	Ca_Kabuli_Chr05	29852105	(T/G)
8386	CakSNP8386	*Kabuli*	Ca_Kabuli_Chr05	29886086	(T/A)
8387	CakSNP8387	*Kabuli*	Ca_Kabuli_Chr05	29981826	(A/G)
8388	CakSNP8388	*Kabuli*	Ca_Kabuli_Chr05	29999414	(C/T)
8389	CakSNP8389	*Kabuli*	Ca_Kabuli_Chr05	29999427	(G/A)
8390	CakSNP8390	*Kabuli*	Ca_Kabuli_Chr05	29999433	(C/A)
8391	CakSNP8391	*Kabuli*	Ca_Kabuli_Chr05	29999465	(T/G)
8392	CakSNP8392	*Kabuli*	Ca_Kabuli_Chr05	29999476	(G/A)
8393	CakSNP8393	*Kabuli*	Ca_Kabuli_Chr05	29999523	(A/T)
8394	CakSNP8394	*Kabuli*	Ca_Kabuli_Chr05	30035763	(A/T)
8395	CakSNP8395	*Kabuli*	Ca_Kabuli_Chr05	30049265	(G/A)
8396	CakSNP8396	*Kabuli*	Ca_Kabuli_Chr05	30053540	(C/G)
8397	CakSNP8397	*Kabuli*	Ca_Kabuli_Chr05	30060160	(C/T)

S.N.	SNP IDs	Cultivars	Chromosomes/scaffolds	Physical positions (bp)	SNPs
8398	CakSNP8398	*Kabuli*	Ca_Kabuli_Chr05	30086073	(A/G)
8399	CakSNP8399	*Kabuli*	Ca_Kabuli_Chr05	30086232	(A/C)
8400	CakSNP8400	*Kabuli*	Ca_Kabuli_Chr05	30090906	(A/G)
8401	CakSNP8401	*Kabuli*	Ca_Kabuli_Chr05	30091063	(G/A)
8402	CakSNP8402	*Kabuli*	Ca_Kabuli_Chr05	30116978	(C/T)
8403	CakSNP8403	*Kabuli*	Ca_Kabuli_Chr05	30116992	(T/G)
8404	CakSNP8404	*Kabuli*	Ca_Kabuli_Chr05	30159226	(C/T)
8405	CakSNP8405	*Kabuli*	Ca_Kabuli_Chr05	30159173	(G/A)
8406	CakSNP8406	*Kabuli*	Ca_Kabuli_Chr05	30159161	(G/A)
8407	CakSNP8407	*Kabuli*	Ca_Kabuli_Chr05	30191389	(A/C)
8408	CakSNP8408	*Kabuli*	Ca_Kabuli_Chr05	30248447	(C/A)
8409	CakSNP8409	*Kabuli*	Ca_Kabuli_Chr05	30258178	(T/G)
8410	CakSNP8410	*Kabuli*	Ca_Kabuli_Chr05	30287853	(C/T)
8411	CakSNP8411	*Kabuli*	Ca_Kabuli_Chr05	30304076	(A/T)
8412	CakSNP8412	*Kabuli*	Ca_Kabuli_Chr05	30319316	(A/G)
8413	CakSNP8413	*Kabuli*	Ca_Kabuli_Chr05	30359047	(T/C)
8414	CakSNP8414	*Kabuli*	Ca_Kabuli_Chr05	30359022	(G/A)
8415	CakSNP8415	*Kabuli*	Ca_Kabuli_Chr05	30360185	(T/C)
8416	CakSNP8416	*Kabuli*	Ca_Kabuli_Chr05	30360348	(T/C)
8417	CakSNP8417	*Kabuli*	Ca_Kabuli_Chr05	30367349	(G/T)
8418	CakSNP8418	*Kabuli*	Ca_Kabuli_Chr05	30373542	(A/G)
8419	CakSNP8419	*Kabuli*	Ca_Kabuli_Chr05	30373783	(T/A)
8420	CakSNP8420	*Kabuli*	Ca_Kabuli_Chr05	30376661	(C/G)
8421	CakSNP8421	*Kabuli*	Ca_Kabuli_Chr05	30376722	(T/C)
8422	CakSNP8422	*Kabuli*	Ca_Kabuli_Chr05	30376723	(T/C)
8423	CakSNP8423	*Kabuli*	Ca_Kabuli_Chr05	30378114	(A/G)
8424	CakSNP8424	*Kabuli*	Ca_Kabuli_Chr05	30378184	(A/G)
8425	CakSNP8425	*Kabuli*	Ca_Kabuli_Chr05	30407725	(C/G)
8426	CakSNP8426	*Kabuli*	Ca_Kabuli_Chr05	30408006	(C/T)
8427	CakSNP8427	*Kabuli*	Ca_Kabuli_Chr05	30438913	(G/A)
8428	CakSNP8428	*Kabuli*	Ca_Kabuli_Chr05	30443858	(A/T)
8429	CakSNP8429	*Kabuli*	Ca_Kabuli_Chr05	30562430	(C/G)
8430	CakSNP8430	*Kabuli*	Ca_Kabuli_Chr05	30562524	(G/T)
8431	CakSNP8431	*Kabuli*	Ca_Kabuli_Chr05	30601932	(T/C)
8432	CakSNP8432	*Kabuli*	Ca_Kabuli_Chr05	30601926	(T/C)
8433	CakSNP8433	*Kabuli*	Ca_Kabuli_Chr05	30601920	(T/C)
8434	CakSNP8434	*Kabuli*	Ca_Kabuli_Chr05	30627937	(G/T)
8435	CakSNP8435	*Kabuli*	Ca_Kabuli_Chr05	30632697	(A/G)

S.N.	SNP IDs	Cultivars	Chromosomes/scaffolds	Physical positions (bp)	SNPs
8436	CakSNP8436	*Kabuli*	Ca_Kabuli_Chr05	30655941	(T/C)
8437	CakSNP8437	*Kabuli*	Ca_Kabuli_Chr05	30656058	(A/T)
8438	CakSNP8438	*Kabuli*	Ca_Kabuli_Chr05	30690891	(T/G)
8439	CakSNP8439	*Kabuli*	Ca_Kabuli_Chr05	30690852	(G/A)
8440	CakSNP8440	*Kabuli*	Ca_Kabuli_Chr05	30690885	(A/G)
8441	CakSNP8441	*Kabuli*	Ca_Kabuli_Chr05	30691510	(A/G)
8442	CakSNP8442	*Kabuli*	Ca_Kabuli_Chr05	30691515	(G/A)
8443	CakSNP8443	*Kabuli*	Ca_Kabuli_Chr05	30691543	(A/G)
8444	CakSNP8444	*Kabuli*	Ca_Kabuli_Chr05	30697511	(G/A)
8445	CakSNP8445	*Kabuli*	Ca_Kabuli_Chr05	30745571	(G/T)
8446	CakSNP8446	*Kabuli*	Ca_Kabuli_Chr05	30749553	(A/T)
8447	CakSNP8447	*Kabuli*	Ca_Kabuli_Chr05	30749696	(A/T)
8448	CakSNP8448	*Kabuli*	Ca_Kabuli_Chr05	30755644	(A/G)
8449	CakSNP8449	*Kabuli*	Ca_Kabuli_Chr05	30755584	(G/A)
8450	CakSNP8450	*Kabuli*	Ca_Kabuli_Chr05	30811250	(A/T)
8451	CakSNP8451	*Kabuli*	Ca_Kabuli_Chr05	30864010	(T/C)
8452	CakSNP8452	*Kabuli*	Ca_Kabuli_Chr05	30868770	(G/C)
8453	CakSNP8453	*Kabuli*	Ca_Kabuli_Chr05	30868695	(G/C)
8454	CakSNP8454	*Kabuli*	Ca_Kabuli_Chr05	30878047	(C/T)
8455	CakSNP8455	*Kabuli*	Ca_Kabuli_Chr05	30878092	(G/T)
8456	CakSNP8456	*Kabuli*	Ca_Kabuli_Chr05	30878100	(T/A)
8457	CakSNP8457	*Kabuli*	Ca_Kabuli_Chr05	30890480	(G/C)
8458	CakSNP8458	*Kabuli*	Ca_Kabuli_Chr05	30962640	(A/T)
8459	CakSNP8459	*Kabuli*	Ca_Kabuli_Chr05	30962641	(C/A)
8460	CakSNP8460	*Kabuli*	Ca_Kabuli_Chr05	30962650	(G/A)
8461	CakSNP8461	*Kabuli*	Ca_Kabuli_Chr05	30964628	(A/G)
8462	CakSNP8462	*Kabuli*	Ca_Kabuli_Chr05	31024408	(T/C)
8463	CakSNP8463	*Kabuli*	Ca_Kabuli_Chr05	31025764	(G/T)
8464	CakSNP8464	*Kabuli*	Ca_Kabuli_Chr05	31034500	(T/C)
8465	CakSNP8465	*Kabuli*	Ca_Kabuli_Chr05	31034898	(A/G)
8466	CakSNP8466	*Kabuli*	Ca_Kabuli_Chr05	31064173	(C/G)
8467	CakSNP8467	*Kabuli*	Ca_Kabuli_Chr05	31072213	(T/C)
8468	CakSNP8468	*Kabuli*	Ca_Kabuli_Chr05	31072276	(G/A)
8469	CakSNP8469	*Kabuli*	Ca_Kabuli_Chr05	31084839	(A/C)
8470	CakSNP8470	*Kabuli*	Ca_Kabuli_Chr05	31088698	(T/G)
8471	CakSNP8471	*Kabuli*	Ca_Kabuli_Chr05	31126125	(C/T)
8472	CakSNP8472	*Kabuli*	Ca_Kabuli_Chr05	31131830	(T/C)
8473	CakSNP8473	*Kabuli*	Ca_Kabuli_Chr05	31132568	(A/T)

S.N.	SNP IDs	Cultivars	Chromosomes/scaffolds	Physical positions (bp)	SNPs
8474	CakSNP8474	*Kabuli*	Ca_Kabuli_Chr05	31138650	(G/A)
8475	CakSNP8475	*Kabuli*	Ca_Kabuli_Chr05	31140531	(T/C)
8476	CakSNP8476	*Kabuli*	Ca_Kabuli_Chr05	31162533	(G/A)
8477	CakSNP8477	*Kabuli*	Ca_Kabuli_Chr05	31164387	(G/C)
8478	CakSNP8478	*Kabuli*	Ca_Kabuli_Chr05	31164539	(T/C)
8479	CakSNP8479	*Kabuli*	Ca_Kabuli_Chr05	31165048	(T/C)
8480	CakSNP8480	*Kabuli*	Ca_Kabuli_Chr05	31165039	(T/C)
8481	CakSNP8481	*Kabuli*	Ca_Kabuli_Chr05	31165035	(T/C)
8482	CakSNP8482	*Kabuli*	Ca_Kabuli_Chr05	31165002	(A/C)
8483	CakSNP8483	*Kabuli*	Ca_Kabuli_Chr05	31171555	(G/A)
8484	CakSNP8484	*Kabuli*	Ca_Kabuli_Chr05	31176722	(T/G)
8485	CakSNP8485	*Kabuli*	Ca_Kabuli_Chr05	31176810	(C/T)
8486	CakSNP8486	*Kabuli*	Ca_Kabuli_Chr05	31176844	(G/A)
8487	CakSNP8487	*Kabuli*	Ca_Kabuli_Chr05	31192415	(C/T)
8488	CakSNP8488	*Kabuli*	Ca_Kabuli_Chr05	31192413	(G/A)
8489	CakSNP8489	*Kabuli*	Ca_Kabuli_Chr05	31194845	(G/T)
8490	CakSNP8490	*Kabuli*	Ca_Kabuli_Chr05	31194974	(T/C)
8491	CakSNP8491	*Kabuli*	Ca_Kabuli_Chr05	31194988	(G/T)
8492	CakSNP8492	*Kabuli*	Ca_Kabuli_Chr05	31194989	(A/C)
8493	CakSNP8493	*Kabuli*	Ca_Kabuli_Chr05	31215109	(C/T)
8494	CakSNP8494	*Kabuli*	Ca_Kabuli_Chr05	31310698	(C/T)
8495	CakSNP8495	*Kabuli*	Ca_Kabuli_Chr05	31310702	(C/G)
8496	CakSNP8496	*Kabuli*	Ca_Kabuli_Chr05	31310703	(A/G)
8497	CakSNP8497	*Kabuli*	Ca_Kabuli_Chr05	31310741	(A/G)
8498	CakSNP8498	*Kabuli*	Ca_Kabuli_Chr05	31314012	(A/C)
8499	CakSNP8499	*Kabuli*	Ca_Kabuli_Chr05	31314056	(A/C)
8500	CakSNP8500	*Kabuli*	Ca_Kabuli_Chr05	31344725	(G/A)
8501	CakSNP8501	*Kabuli*	Ca_Kabuli_Chr05	31377103	(C/T)
8502	CakSNP8502	*Kabuli*	Ca_Kabuli_Chr05	31377122	(C/T)
8503	CakSNP8503	*Kabuli*	Ca_Kabuli_Chr05	31377134	(A/T)
8504	CakSNP8504	*Kabuli*	Ca_Kabuli_Chr05	31390221	(A/G)
8505	CakSNP8505	*Kabuli*	Ca_Kabuli_Chr05	31390319	(C/T)
8506	CakSNP8506	*Kabuli*	Ca_Kabuli_Chr05	31522389	(T/G)
8507	CakSNP8507	*Kabuli*	Ca_Kabuli_Chr05	31548555	(G/A)
8508	CakSNP8508	*Kabuli*	Ca_Kabuli_Chr05	31651335	(G/T)
8509	CakSNP8509	*Kabuli*	Ca_Kabuli_Chr05	31651336	(T/G)
8510	CakSNP8510	*Kabuli*	Ca_Kabuli_Chr05	31653418	(T/C)
8511	CakSNP8511	*Kabuli*	Ca_Kabuli_Chr05	31653423	(T/C)

S.N.	SNP IDs	Cultivars	Chromosomes/scaffolds	Physical positions (bp)	SNPs
8512	CakSNP8512	*Kabuli*	Ca_Kabuli_Chr05	31653432	(A/G)
8513	CakSNP8513	*Kabuli*	Ca_Kabuli_Chr05	31653437	(C/T)
8514	CakSNP8514	*Kabuli*	Ca_Kabuli_Chr05	31653452	(C/A)
8515	CakSNP8515	*Kabuli*	Ca_Kabuli_Chr05	31687881	(C/T)
8516	CakSNP8516	*Kabuli*	Ca_Kabuli_Chr05	31687891	(A/C)
8517	CakSNP8517	*Kabuli*	Ca_Kabuli_Chr05	31696236	(T/C)
8518	CakSNP8518	*Kabuli*	Ca_Kabuli_Chr05	31710670	(G/A)
8519	CakSNP8519	*Kabuli*	Ca_Kabuli_Chr05	31724217	(G/A)
8520	CakSNP8520	*Kabuli*	Ca_Kabuli_Chr05	31724272	(C/T)
8521	CakSNP8521	*Kabuli*	Ca_Kabuli_Chr05	31724332	(G/A)
8522	CakSNP8522	*Kabuli*	Ca_Kabuli_Chr05	31750150	(T/G)
8523	CakSNP8523	*Kabuli*	Ca_Kabuli_Chr05	31750242	(A/G)
8524	CakSNP8524	*Kabuli*	Ca_Kabuli_Chr05	31758166	(T/C)
8525	CakSNP8525	*Kabuli*	Ca_Kabuli_Chr05	31802684	(G/T)
8526	CakSNP8526	*Kabuli*	Ca_Kabuli_Chr05	31802727	(G/A)
8527	CakSNP8527	*Kabuli*	Ca_Kabuli_Chr05	31816202	(A/C)
8528	CakSNP8528	*Kabuli*	Ca_Kabuli_Chr05	31816228	(A/G)
8529	CakSNP8529	*Kabuli*	Ca_Kabuli_Chr05	31935674	(T/C)
8530	CakSNP8530	*Kabuli*	Ca_Kabuli_Chr05	31951925	(G/A)
8531	CakSNP8531	*Kabuli*	Ca_Kabuli_Chr05	31951944	(C/T)
8532	CakSNP8532	*Kabuli*	Ca_Kabuli_Chr05	31985390	(G/C)
8533	CakSNP8533	*Kabuli*	Ca_Kabuli_Chr05	32011781	(G/T)
8534	CakSNP8534	*Kabuli*	Ca_Kabuli_Chr05	32011792	(T/G)
8535	CakSNP8535	*Kabuli*	Ca_Kabuli_Chr05	32015967	(T/C)
8536	CakSNP8536	*Kabuli*	Ca_Kabuli_Chr05	32019864	(G/A)
8537	CakSNP8537	*Kabuli*	Ca_Kabuli_Chr05	32023925	(T/C)
8538	CakSNP8538	*Kabuli*	Ca_Kabuli_Chr05	32090295	(C/A)
8539	CakSNP8539	*Kabuli*	Ca_Kabuli_Chr05	32144081	(G/C)
8540	CakSNP8540	*Kabuli*	Ca_Kabuli_Chr05	32144108	(A/C)
8541	CakSNP8541	*Kabuli*	Ca_Kabuli_Chr05	32144120	(C/T)
8542	CakSNP8542	*Kabuli*	Ca_Kabuli_Chr05	32147522	(C/T)
8543	CakSNP8543	*Kabuli*	Ca_Kabuli_Chr05	32171632	(G/A)
8544	CakSNP8544	*Kabuli*	Ca_Kabuli_Chr05	32171652	(T/A)
8545	CakSNP8545	*Kabuli*	Ca_Kabuli_Chr05	32209547	(C/G)
8546	CakSNP8546	*Kabuli*	Ca_Kabuli_Chr05	32209750	(T/C)
8547	CakSNP8547	*Kabuli*	Ca_Kabuli_Chr05	32229485	(A/T)
8548	CakSNP8548	*Kabuli*	Ca_Kabuli_Chr05	32291158	(C/T)
8549	CakSNP8549	*Kabuli*	Ca_Kabuli_Chr05	32291118	(G/A)

S.N.	SNP IDs	Cultivars	Chromosomes/scaffolds	Physical positions (bp)	SNPs
8550	CakSNP8550	*Kabuli*	Ca_Kabuli_Chr05	32328110	(T/C)
8551	CakSNP8551	*Kabuli*	Ca_Kabuli_Chr05	32346655	(A/C)
8552	CakSNP8552	*Kabuli*	Ca_Kabuli_Chr05	32357511	(G/A)
8553	CakSNP8553	*Kabuli*	Ca_Kabuli_Chr05	32368485	(G/C)
8554	CakSNP8554	*Kabuli*	Ca_Kabuli_Chr05	32369197	(C/A)
8555	CakSNP8555	*Kabuli*	Ca_Kabuli_Chr05	32399391	(T/A)
8556	CakSNP8556	*Kabuli*	Ca_Kabuli_Chr05	32476432	(A/G)
8557	CakSNP8557	*Kabuli*	Ca_Kabuli_Chr05	32476423	(A/G)
8558	CakSNP8558	*Kabuli*	Ca_Kabuli_Chr05	32516773	(T/A)
8559	CakSNP8559	*Kabuli*	Ca_Kabuli_Chr05	32524646	(T/C)
8560	CakSNP8560	*Kabuli*	Ca_Kabuli_Chr05	32566131	(C/G)
8561	CakSNP8561	*Kabuli*	Ca_Kabuli_Chr05	32598221	(C/A)
8562	CakSNP8562	*Kabuli*	Ca_Kabuli_Chr05	32623507	(T/G)
8563	CakSNP8563	*Kabuli*	Ca_Kabuli_Chr05	32631930	(C/A)
8564	CakSNP8564	*Kabuli*	Ca_Kabuli_Chr05	32643759	(A/G)
8565	CakSNP8565	*Kabuli*	Ca_Kabuli_Chr05	32732862	(A/T)
8566	CakSNP8566	*Kabuli*	Ca_Kabuli_Chr05	32732913	(A/T)
8567	CakSNP8567	*Kabuli*	Ca_Kabuli_Chr05	32759420	(T/A)
8568	CakSNP8568	*Kabuli*	Ca_Kabuli_Chr05	32761420	(G/A)
8569	CakSNP8569	*Kabuli*	Ca_Kabuli_Chr05	32794288	(G/A)
8570	CakSNP8570	*Kabuli*	Ca_Kabuli_Chr05	32816626	(G/T)
8571	CakSNP8571	*Kabuli*	Ca_Kabuli_Chr05	32839347	(C/A)
8572	CakSNP8572	*Kabuli*	Ca_Kabuli_Chr05	32840512	(A/T)
8573	CakSNP8573	*Kabuli*	Ca_Kabuli_Chr05	32840522	(T/C)
8574	CakSNP8574	*Kabuli*	Ca_Kabuli_Chr05	32840541	(T/G)
8575	CakSNP8575	*Kabuli*	Ca_Kabuli_Chr05	32859483	(T/A)
8576	CakSNP8576	*Kabuli*	Ca_Kabuli_Chr05	32859504	(T/G)
8577	CakSNP8577	*Kabuli*	Ca_Kabuli_Chr05	32859515	(T/G)
8578	CakSNP8578	*Kabuli*	Ca_Kabuli_Chr05	32860087	(C/T)
8579	CakSNP8579	*Kabuli*	Ca_Kabuli_Chr05	32978975	(A/T)
8580	CakSNP8580	*Kabuli*	Ca_Kabuli_Chr05	33052329	(C/G)
8581	CakSNP8581	*Kabuli*	Ca_Kabuli_Chr05	33143038	(A/T)
8582	CakSNP8582	*Kabuli*	Ca_Kabuli_Chr05	33189733	(A/G)
8583	CakSNP8583	*Kabuli*	Ca_Kabuli_Chr05	33189791	(G/A)
8584	CakSNP8584	*Kabuli*	Ca_Kabuli_Chr05	33189808	(A/G)
8585	CakSNP8585	*Kabuli*	Ca_Kabuli_Chr05	33255823	(T/G)
8586	CakSNP8586	*Kabuli*	Ca_Kabuli_Chr05	33255971	(G/T)
8587	CakSNP8587	*Kabuli*	Ca_Kabuli_Chr05	33256053	(C/G)

S.N.	SNP IDs	Cultivars	Chromosomes/scaffolds	Physical positions (bp)	SNPs
8588	CakSNP8588	*Kabuli*	Ca_Kabuli_Chr05	33256089	(T/C)
8589	CakSNP8589	*Kabuli*	Ca_Kabuli_Chr05	33256245	(T/C)
8590	CakSNP8590	*Kabuli*	Ca_Kabuli_Chr05	33256412	(A/T)
8591	CakSNP8591	*Kabuli*	Ca_Kabuli_Chr05	33256388	(T/A)
8592	CakSNP8592	*Kabuli*	Ca_Kabuli_Chr05	33336127	(C/G)
8593	CakSNP8593	*Kabuli*	Ca_Kabuli_Chr05	33336135	(G/T)
8594	CakSNP8594	*Kabuli*	Ca_Kabuli_Chr05	33349598	(C/T)
8595	CakSNP8595	*Kabuli*	Ca_Kabuli_Chr05	33349700	(A/G)
8596	CakSNP8596	*Kabuli*	Ca_Kabuli_Chr05	33379904	(A/G)
8597	CakSNP8597	*Kabuli*	Ca_Kabuli_Chr05	33379873	(C/T)
8598	CakSNP8598	*Kabuli*	Ca_Kabuli_Chr05	33381774	(C/T)
8599	CakSNP8599	*Kabuli*	Ca_Kabuli_Chr05	33422813	(C/T)
8600	CakSNP8600	*Kabuli*	Ca_Kabuli_Chr05	33422780	(A/G)
8601	CakSNP8601	*Kabuli*	Ca_Kabuli_Chr05	33422779	(C/T)
8602	CakSNP8602	*Kabuli*	Ca_Kabuli_Chr05	33422767	(T/C)
8603	CakSNP8603	*Kabuli*	Ca_Kabuli_Chr05	33431897	(T/C)
8604	CakSNP8604	*Kabuli*	Ca_Kabuli_Chr05	33431996	(T/C)
8605	CakSNP8605	*Kabuli*	Ca_Kabuli_Chr05	33523676	(T/C)
8606	CakSNP8606	*Kabuli*	Ca_Kabuli_Chr05	33523686	(G/A)
8607	CakSNP8607	*Kabuli*	Ca_Kabuli_Chr05	33523691	(T/C)
8608	CakSNP8608	*Kabuli*	Ca_Kabuli_Chr05	33532807	(A/G)
8609	CakSNP8609	*Kabuli*	Ca_Kabuli_Chr05	33532804	(A/T)
8610	CakSNP8610	*Kabuli*	Ca_Kabuli_Chr05	33532798	(A/T)
8611	CakSNP8611	*Kabuli*	Ca_Kabuli_Chr05	33574726	(T/C)
8612	CakSNP8612	*Kabuli*	Ca_Kabuli_Chr05	33578061	(T/A)
8613	CakSNP8613	*Kabuli*	Ca_Kabuli_Chr05	33578167	(A/T)
8614	CakSNP8614	*Kabuli*	Ca_Kabuli_Chr05	33578438	(T/C)
8615	CakSNP8615	*Kabuli*	Ca_Kabuli_Chr05	33578477	(A/G)
8616	CakSNP8616	*Kabuli*	Ca_Kabuli_Chr05	33581040	(A/G)
8617	CakSNP8617	*Kabuli*	Ca_Kabuli_Chr05	33626841	(C/T)
8618	CakSNP8618	*Kabuli*	Ca_Kabuli_Chr05	33627132	(T/C)
8619	CakSNP8619	*Kabuli*	Ca_Kabuli_Chr05	33631108	(A/G)
8620	CakSNP8620	*Kabuli*	Ca_Kabuli_Chr05	33631266	(T/A)
8621	CakSNP8621	*Kabuli*	Ca_Kabuli_Chr05	33656560	(C/G)
8622	CakSNP8622	*Kabuli*	Ca_Kabuli_Chr05	33673894	(T/C)
8623	CakSNP8623	*Kabuli*	Ca_Kabuli_Chr05	33700181	(G/C)
8624	CakSNP8624	*Kabuli*	Ca_Kabuli_Chr05	33700196	(A/T)
8625	CakSNP8625	*Kabuli*	Ca_Kabuli_Chr05	33721487	(C/T)

S.N.	SNP IDs	Cultivars	Chromosomes/scaffolds	Physical positions (bp)	SNPs
8626	CakSNP8626	*Kabuli*	Ca_Kabuli_Chr05	33721544	(A/G)
8627	CakSNP8627	*Kabuli*	Ca_Kabuli_Chr05	33771359	(T/C)
8628	CakSNP8628	*Kabuli*	Ca_Kabuli_Chr05	33812108	(T/C)
8629	CakSNP8629	*Kabuli*	Ca_Kabuli_Chr05	33812213	(A/C)
8630	CakSNP8630	*Kabuli*	Ca_Kabuli_Chr05	33812363	(T/A)
8631	CakSNP8631	*Kabuli*	Ca_Kabuli_Chr05	33818742	(T/A)
8632	CakSNP8632	*Kabuli*	Ca_Kabuli_Chr05	33818789	(C/G)
8633	CakSNP8633	*Kabuli*	Ca_Kabuli_Chr05	33818823	(A/G)
8634	CakSNP8634	*Kabuli*	Ca_Kabuli_Chr05	33824516	(G/T)
8635	CakSNP8635	*Kabuli*	Ca_Kabuli_Chr05	33824513	(G/A)
8636	CakSNP8636	*Kabuli*	Ca_Kabuli_Chr05	33824503	(G/A)
8637	CakSNP8637	*Kabuli*	Ca_Kabuli_Chr05	33835816	(T/C)
8638	CakSNP8638	*Kabuli*	Ca_Kabuli_Chr05	33841544	(T/G)
8639	CakSNP8639	*Kabuli*	Ca_Kabuli_Chr05	33844056	(A/T)
8640	CakSNP8640	*Kabuli*	Ca_Kabuli_Chr05	33844059	(G/C)
8641	CakSNP8641	*Kabuli*	Ca_Kabuli_Chr05	33844222	(T/C)
8642	CakSNP8642	*Kabuli*	Ca_Kabuli_Chr05	33844436	(T/A)
8643	CakSNP8643	*Kabuli*	Ca_Kabuli_Chr05	33892596	(A/C)
8644	CakSNP8644	*Kabuli*	Ca_Kabuli_Chr05	33937307	(G/A)
8645	CakSNP8645	*Kabuli*	Ca_Kabuli_Chr05	33937316	(G/A)
8646	CakSNP8646	*Kabuli*	Ca_Kabuli_Chr05	33963525	(G/C)
8647	CakSNP8647	*Kabuli*	Ca_Kabuli_Chr05	33985819	(A/G)
8648	CakSNP8648	*Kabuli*	Ca_Kabuli_Chr05	33986234	(C/T)
8649	CakSNP8649	*Kabuli*	Ca_Kabuli_Chr05	33986253	(A/C)
8650	CakSNP8650	*Kabuli*	Ca_Kabuli_Chr05	34022362	(G/T)
8651	CakSNP8651	*Kabuli*	Ca_Kabuli_Chr05	34040479	(A/G)
8652	CakSNP8652	*Kabuli*	Ca_Kabuli_Chr05	34040508	(A/C)
8653	CakSNP8653	*Kabuli*	Ca_Kabuli_Chr05	34040535	(G/A)
8654	CakSNP8654	*Kabuli*	Ca_Kabuli_Chr05	34041705	(G/T)
8655	CakSNP8655	*Kabuli*	Ca_Kabuli_Chr05	34073378	(A/T)
8656	CakSNP8656	*Kabuli*	Ca_Kabuli_Chr05	34073395	(G/A)
8657	CakSNP8657	*Kabuli*	Ca_Kabuli_Chr05	34073445	(A/T)
8658	CakSNP8658	*Kabuli*	Ca_Kabuli_Chr05	34201989	(C/G)
8659	CakSNP8659	*Kabuli*	Ca_Kabuli_Chr05	34202000	(T/C)
8660	CakSNP8660	*Kabuli*	Ca_Kabuli_Chr05	34202075	(T/G)
8661	CakSNP8661	*Kabuli*	Ca_Kabuli_Chr05	34202222	(G/A)
8662	CakSNP8662	*Kabuli*	Ca_Kabuli_Chr05	34241269	(C/G)
8663	CakSNP8663	*Kabuli*	Ca_Kabuli_Chr05	34244499	(C/T)

S.N.	SNP IDs	Cultivars	Chromosomes/scaffolds	Physical positions (bp)	SNPs
8664	CakSNP8664	*Kabuli*	Ca_Kabuli_Chr05	34244681	(G/A)
8665	CakSNP8665	*Kabuli*	Ca_Kabuli_Chr05	34244670	(A/G)
8666	CakSNP8666	*Kabuli*	Ca_Kabuli_Chr05	34244662	(C/G)
8667	CakSNP8667	*Kabuli*	Ca_Kabuli_Chr05	34244754	(T/A)
8668	CakSNP8668	*Kabuli*	Ca_Kabuli_Chr05	34313779	(A/G)
8669	CakSNP8669	*Kabuli*	Ca_Kabuli_Chr05	34313758	(T/G)
8670	CakSNP8670	*Kabuli*	Ca_Kabuli_Chr05	34334980	(A/G)
8671	CakSNP8671	*Kabuli*	Ca_Kabuli_Chr05	34334993	(A/C)
8672	CakSNP8672	*Kabuli*	Ca_Kabuli_Chr05	34335252	(T/A)
8673	CakSNP8673	*Kabuli*	Ca_Kabuli_Chr05	34418798	(G/C)
8674	CakSNP8674	*Kabuli*	Ca_Kabuli_Chr05	34441581	(A/C)
8675	CakSNP8675	*Kabuli*	Ca_Kabuli_Chr05	34461159	(T/C)
8676	CakSNP8676	*Kabuli*	Ca_Kabuli_Chr05	34482245	(G/T)
8677	CakSNP8677	*Kabuli*	Ca_Kabuli_Chr05	34482217	(G/T)
8678	CakSNP8678	*Kabuli*	Ca_Kabuli_Chr05	34511669	(G/T)
8679	CakSNP8679	*Kabuli*	Ca_Kabuli_Chr05	34511678	(T/C)
8680	CakSNP8680	*Kabuli*	Ca_Kabuli_Chr05	34511693	(C/T)
8681	CakSNP8681	*Kabuli*	Ca_Kabuli_Chr05	34511705	(G/T)
8682	CakSNP8682	*Kabuli*	Ca_Kabuli_Chr05	34521655	(T/C)
8683	CakSNP8683	*Kabuli*	Ca_Kabuli_Chr05	34531222	(A/G)
8684	CakSNP8684	*Kabuli*	Ca_Kabuli_Chr05	34535758	(T/C)
8685	CakSNP8685	*Kabuli*	Ca_Kabuli_Chr05	34535893	(G/T)
8686	CakSNP8686	*Kabuli*	Ca_Kabuli_Chr05	34535878	(G/A)
8687	CakSNP8687	*Kabuli*	Ca_Kabuli_Chr05	34629028	(A/G)
8688	CakSNP8688	*Kabuli*	Ca_Kabuli_Chr05	34673784	(T/A)
8689	CakSNP8689	*Kabuli*	Ca_Kabuli_Chr05	34691234	(A/C)
8690	CakSNP8690	*Kabuli*	Ca_Kabuli_Chr05	34691302	(T/C)
8691	CakSNP8691	*Kabuli*	Ca_Kabuli_Chr05	34691453	(G/A)
8692	CakSNP8692	*Kabuli*	Ca_Kabuli_Chr05	34807071	(C/T)
8693	CakSNP8693	*Kabuli*	Ca_Kabuli_Chr05	34814216	(T/C)
8694	CakSNP8694	*Kabuli*	Ca_Kabuli_Chr05	34824647	(C/G)
8695	CakSNP8695	*Kabuli*	Ca_Kabuli_Chr05	34824749	(A/T)
8696	CakSNP8696	*Kabuli*	Ca_Kabuli_Chr05	34845398	(T/G)
8697	CakSNP8697	*Kabuli*	Ca_Kabuli_Chr05	34879731	(A/G)
8698	CakSNP8698	*Kabuli*	Ca_Kabuli_Chr05	34974615	(T/A)
8699	CakSNP8699	*Kabuli*	Ca_Kabuli_Chr05	34974703	(C/T)
8700	CakSNP8700	*Kabuli*	Ca_Kabuli_Chr05	34974795	(A/G)
8701	CakSNP8701	*Kabuli*	Ca_Kabuli_Chr05	34975712	(T/A)

S.N.	SNP IDs	Cultivars	Chromosomes/scaffolds	Physical positions (bp)	SNPs
8702	CakSNP8702	*Kabuli*	Ca_Kabuli_Chr05	34998725	(G/A)
8703	CakSNP8703	*Kabuli*	Ca_Kabuli_Chr05	35126544	(A/C)
8704	CakSNP8704	*Kabuli*	Ca_Kabuli_Chr05	35164295	(C/T)
8705	CakSNP8705	*Kabuli*	Ca_Kabuli_Chr05	35191371	(C/G)
8706	CakSNP8706	*Kabuli*	Ca_Kabuli_Chr05	35209511	(T/C)
8707	CakSNP8707	*Kabuli*	Ca_Kabuli_Chr05	35213601	(C/T)
8708	CakSNP8708	*Kabuli*	Ca_Kabuli_Chr05	35226049	(G/C)
8709	CakSNP8709	*Kabuli*	Ca_Kabuli_Chr05	35290452	(A/G)
8710	CakSNP8710	*Kabuli*	Ca_Kabuli_Chr05	35296135	(A/C)
8711	CakSNP8711	*Kabuli*	Ca_Kabuli_Chr05	35330946	(G/A)
8712	CakSNP8712	*Kabuli*	Ca_Kabuli_Chr05	35330995	(A/C)
8713	CakSNP8713	*Kabuli*	Ca_Kabuli_Chr05	35340068	(A/G)
8714	CakSNP8714	*Kabuli*	Ca_Kabuli_Chr05	35340065	(G/A)
8715	CakSNP8715	*Kabuli*	Ca_Kabuli_Chr05	35374184	(G/A)
8716	CakSNP8716	*Kabuli*	Ca_Kabuli_Chr05	35438327	(G/T)
8717	CakSNP8717	*Kabuli*	Ca_Kabuli_Chr05	35495900	(T/A)
8718	CakSNP8718	*Kabuli*	Ca_Kabuli_Chr05	35500751	(T/C)
8719	CakSNP8719	*Kabuli*	Ca_Kabuli_Chr05	35534923	(T/A)
8720	CakSNP8720	*Kabuli*	Ca_Kabuli_Chr05	35534897	(G/A)
8721	CakSNP8721	*Kabuli*	Ca_Kabuli_Chr05	35540372	(T/C)
8722	CakSNP8722	*Kabuli*	Ca_Kabuli_Chr05	35543648	(T/C)
8723	CakSNP8723	*Kabuli*	Ca_Kabuli_Chr05	35554534	(C/A)
8724	CakSNP8724	*Kabuli*	Ca_Kabuli_Chr05	35554593	(C/T)
8725	CakSNP8725	*Kabuli*	Ca_Kabuli_Chr05	35555184	(A/G)
8726	CakSNP8726	*Kabuli*	Ca_Kabuli_Chr05	35576495	(A/G)
8727	CakSNP8727	*Kabuli*	Ca_Kabuli_Chr05	35617798	(A/C)
8728	CakSNP8728	*Kabuli*	Ca_Kabuli_Chr05	35688558	(T/C)
8729	CakSNP8729	*Kabuli*	Ca_Kabuli_Chr05	35690996	(G/A)
8730	CakSNP8730	*Kabuli*	Ca_Kabuli_Chr05	35691581	(T/C)
8731	CakSNP8731	*Kabuli*	Ca_Kabuli_Chr05	35715495	(C/T)
8732	CakSNP8732	*Kabuli*	Ca_Kabuli_Chr05	35715550	(G/C)
8733	CakSNP8733	*Kabuli*	Ca_Kabuli_Chr05	35715571	(A/C)
8734	CakSNP8734	*Kabuli*	Ca_Kabuli_Chr05	35715563	(G/A)
8735	CakSNP8735	*Kabuli*	Ca_Kabuli_Chr05	35715548	(T/C)
8736	CakSNP8736	*Kabuli*	Ca_Kabuli_Chr05	35773040	(C/A)
8737	CakSNP8737	*Kabuli*	Ca_Kabuli_Chr05	35773044	(A/G)
8738	CakSNP8738	*Kabuli*	Ca_Kabuli_Chr05	35775200	(T/C)
8739	CakSNP8739	*Kabuli*	Ca_Kabuli_Chr05	35775191	(T/G)

S.N.	SNP IDs	Cultivars	Chromosomes/scaffolds	Physical positions (bp)	SNPs
8740	CakSNP8740	*Kabuli*	Ca_Kabuli_Chr05	35775266	(G/A)
8741	CakSNP8741	*Kabuli*	Ca_Kabuli_Chr05	35789373	(T/A)
8742	CakSNP8742	*Kabuli*	Ca_Kabuli_Chr05	35789775	(A/G)
8743	CakSNP8743	*Kabuli*	Ca_Kabuli_Chr05	35839230	(T/C)
8744	CakSNP8744	*Kabuli*	Ca_Kabuli_Chr05	35839233	(G/A)
8745	CakSNP8745	*Kabuli*	Ca_Kabuli_Chr05	35844295	(G/T)
8746	CakSNP8746	*Kabuli*	Ca_Kabuli_Chr05	35845094	(G/A)
8747	CakSNP8747	*Kabuli*	Ca_Kabuli_Chr05	35845780	(T/C)
8748	CakSNP8748	*Kabuli*	Ca_Kabuli_Chr05	35845801	(T/C)
8749	CakSNP8749	*Kabuli*	Ca_Kabuli_Chr05	35846404	(A/G)
8750	CakSNP8750	*Kabuli*	Ca_Kabuli_Chr05	35868865	(G/A)
8751	CakSNP8751	*Kabuli*	Ca_Kabuli_Chr05	35877505	(T/C)
8752	CakSNP8752	*Kabuli*	Ca_Kabuli_Chr05	35926105	(T/C)
8753	CakSNP8753	*Kabuli*	Ca_Kabuli_Chr05	35937904	(C/T)
8754	CakSNP8754	*Kabuli*	Ca_Kabuli_Chr05	35937842	(G/T)
8755	CakSNP8755	*Kabuli*	Ca_Kabuli_Chr05	35947574	(G/A)
8756	CakSNP8756	*Kabuli*	Ca_Kabuli_Chr05	35954656	(T/C)
8757	CakSNP8757	*Kabuli*	Ca_Kabuli_Chr05	35956534	(C/T)
8758	CakSNP8758	*Kabuli*	Ca_Kabuli_Chr05	36012698	(C/G)
8759	CakSNP8759	*Kabuli*	Ca_Kabuli_Chr05	36039365	(A/C)
8760	CakSNP8760	*Kabuli*	Ca_Kabuli_Chr05	36128655	(G/T)
8761	CakSNP8761	*Kabuli*	Ca_Kabuli_Chr05	36131156	(A/C)
8762	CakSNP8762	*Kabuli*	Ca_Kabuli_Chr05	36217803	(C/T)
8763	CakSNP8763	*Kabuli*	Ca_Kabuli_Chr05	36238656	(G/A)
8764	CakSNP8764	*Kabuli*	Ca_Kabuli_Chr05	36238655	(G/C)
8765	CakSNP8765	*Kabuli*	Ca_Kabuli_Chr05	36248568	(C/T)
8766	CakSNP8766	*Kabuli*	Ca_Kabuli_Chr05	36292611	(C/G)
8767	CakSNP8767	*Kabuli*	Ca_Kabuli_Chr05	36334569	(C/A)
8768	CakSNP8768	*Kabuli*	Ca_Kabuli_Chr05	36334592	(C/T)
8769	CakSNP8769	*Kabuli*	Ca_Kabuli_Chr05	36334625	(A/G)
8770	CakSNP8770	*Kabuli*	Ca_Kabuli_Chr05	36338598	(A/C)
8771	CakSNP8771	*Kabuli*	Ca_Kabuli_Chr05	36338658	(A/G)
8772	CakSNP8772	*Kabuli*	Ca_Kabuli_Chr05	36397489	(A/C)
8773	CakSNP8773	*Kabuli*	Ca_Kabuli_Chr05	36400943	(C/T)
8774	CakSNP8774	*Kabuli*	Ca_Kabuli_Chr05	36408701	(G/A)
8775	CakSNP8775	*Kabuli*	Ca_Kabuli_Chr05	36408719	(G/A)
8776	CakSNP8776	*Kabuli*	Ca_Kabuli_Chr05	36408724	(T/C)
8777	CakSNP8777	*Kabuli*	Ca_Kabuli_Chr05	36408736	(C/A)

S.N.	SNP IDs	Cultivars	Chromosomes/scaffolds	Physical positions (bp)	SNPs
8778	CakSNP8778	*Kabuli*	Ca_Kabuli_Chr05	36430677	(A/C)
8779	CakSNP8779	*Kabuli*	Ca_Kabuli_Chr05	36441063	(C/T)
8780	CakSNP8780	*Kabuli*	Ca_Kabuli_Chr05	36533996	(T/A)
8781	CakSNP8781	*Kabuli*	Ca_Kabuli_Chr05	36534056	(G/C)
8782	CakSNP8782	*Kabuli*	Ca_Kabuli_Chr05	36534057	(T/G)
8783	CakSNP8783	*Kabuli*	Ca_Kabuli_Chr05	36534060	(G/A)
8784	CakSNP8784	*Kabuli*	Ca_Kabuli_Chr05	36534063	(C/T)
8785	CakSNP8785	*Kabuli*	Ca_Kabuli_Chr05	36534126	(A/G)
8786	CakSNP8786	*Kabuli*	Ca_Kabuli_Chr05	36534110	(T/C)
8787	CakSNP8787	*Kabuli*	Ca_Kabuli_Chr05	36566175	(G/A)
8788	CakSNP8788	*Kabuli*	Ca_Kabuli_Chr05	36613610	(T/C)
8789	CakSNP8789	*Kabuli*	Ca_Kabuli_Chr05	36619837	(A/G)
8790	CakSNP8790	*Kabuli*	Ca_Kabuli_Chr05	36621872	(A/T)
8791	CakSNP8791	*Kabuli*	Ca_Kabuli_Chr05	36672147	(C/T)
8792	CakSNP8792	*Kabuli*	Ca_Kabuli_Chr05	36672164	(A/G)
8793	CakSNP8793	*Kabuli*	Ca_Kabuli_Chr05	36673950	(C/A)
8794	CakSNP8794	*Kabuli*	Ca_Kabuli_Chr05	36731961	(T/C)
8795	CakSNP8795	*Kabuli*	Ca_Kabuli_Chr05	36735459	(A/T)
8796	CakSNP8796	*Kabuli*	Ca_Kabuli_Chr05	36751625	(C/T)
8797	CakSNP8797	*Kabuli*	Ca_Kabuli_Chr05	36751851	(T/A)
8798	CakSNP8798	*Kabuli*	Ca_Kabuli_Chr05	36757408	(G/A)
8799	CakSNP8799	*Kabuli*	Ca_Kabuli_Chr05	36797173	(A/G)
8800	CakSNP8800	*Kabuli*	Ca_Kabuli_Chr05	36797187	(G/T)
8801	CakSNP8801	*Kabuli*	Ca_Kabuli_Chr05	36800497	(G/T)
8802	CakSNP8802	*Kabuli*	Ca_Kabuli_Chr05	36842784	(G/T)
8803	CakSNP8803	*Kabuli*	Ca_Kabuli_Chr05	36855028	(G/A)
8804	CakSNP8804	*Kabuli*	Ca_Kabuli_Chr05	36916560	(T/C)
8805	CakSNP8805	*Kabuli*	Ca_Kabuli_Chr05	36999074	(G/C)
8806	CakSNP8806	*Kabuli*	Ca_Kabuli_Chr05	37016704	(C/G)
8807	CakSNP8807	*Kabuli*	Ca_Kabuli_Chr05	37039246	(A/T)
8808	CakSNP8808	*Kabuli*	Ca_Kabuli_Chr05	37039300	(A/G)
8809	CakSNP8809	*Kabuli*	Ca_Kabuli_Chr05	37040045	(G/A)
8810	CakSNP8810	*Kabuli*	Ca_Kabuli_Chr05	37067293	(A/G)
8811	CakSNP8811	*Kabuli*	Ca_Kabuli_Chr05	37067371	(T/C)
8812	CakSNP8812	*Kabuli*	Ca_Kabuli_Chr05	37067384	(G/C)
8813	CakSNP8813	*Kabuli*	Ca_Kabuli_Chr05	37083276	(T/G)
8814	CakSNP8814	*Kabuli*	Ca_Kabuli_Chr05	37083275	(G/T)
8815	CakSNP8815	*Kabuli*	Ca_Kabuli_Chr05	37114574	(A/G)

S.N.	SNP IDs	Cultivars	Chromosomes/scaffolds	Physical positions (bp)	SNPs
8816	CakSNP8816	*Kabuli*	Ca_Kabuli_Chr05	37126371	(A/G)
8817	CakSNP8817	*Kabuli*	Ca_Kabuli_Chr05	37126354	(T/C)
8818	CakSNP8818	*Kabuli*	Ca_Kabuli_Chr05	37147572	(C/T)
8819	CakSNP8819	*Kabuli*	Ca_Kabuli_Chr05	37167061	(A/G)
8820	CakSNP8820	*Kabuli*	Ca_Kabuli_Chr05	37167114	(A/T)
8821	CakSNP8821	*Kabuli*	Ca_Kabuli_Chr05	37167108	(A/T)
8822	CakSNP8822	*Kabuli*	Ca_Kabuli_Chr05	37194939	(G/A)
8823	CakSNP8823	*Kabuli*	Ca_Kabuli_Chr05	37194917	(G/A)
8824	CakSNP8824	*Kabuli*	Ca_Kabuli_Chr05	37197321	(C/A)
8825	CakSNP8825	*Kabuli*	Ca_Kabuli_Chr05	37207821	(A/T)
8826	CakSNP8826	*Kabuli*	Ca_Kabuli_Chr05	37207837	(G/T)
8827	CakSNP8827	*Kabuli*	Ca_Kabuli_Chr05	37211645	(T/G)
8828	CakSNP8828	*Kabuli*	Ca_Kabuli_Chr05	37213853	(C/T)
8829	CakSNP8829	*Kabuli*	Ca_Kabuli_Chr05	37232307	(T/C)
8830	CakSNP8830	*Kabuli*	Ca_Kabuli_Chr05	37254596	(A/G)
8831	CakSNP8831	*Kabuli*	Ca_Kabuli_Chr05	37260601	(T/C)
8832	CakSNP8832	*Kabuli*	Ca_Kabuli_Chr05	37274572	(C/T)
8833	CakSNP8833	*Kabuli*	Ca_Kabuli_Chr05	37274583	(C/T)
8834	CakSNP8834	*Kabuli*	Ca_Kabuli_Chr05	37274616	(C/G)
8835	CakSNP8835	*Kabuli*	Ca_Kabuli_Chr05	37309917	(T/G)
8836	CakSNP8836	*Kabuli*	Ca_Kabuli_Chr05	37309935	(T/G)
8837	CakSNP8837	*Kabuli*	Ca_Kabuli_Chr05	37332101	(G/T)
8838	CakSNP8838	*Kabuli*	Ca_Kabuli_Chr05	37332198	(G/A)
8839	CakSNP8839	*Kabuli*	Ca_Kabuli_Chr05	37332304	(G/A)
8840	CakSNP8840	*Kabuli*	Ca_Kabuli_Chr05	37332303	(A/C)
8841	CakSNP8841	*Kabuli*	Ca_Kabuli_Chr05	37332370	(T/C)
8842	CakSNP8842	*Kabuli*	Ca_Kabuli_Chr05	37332441	(T/C)
8843	CakSNP8843	*Kabuli*	Ca_Kabuli_Chr05	37332501	(C/T)
8844	CakSNP8844	*Kabuli*	Ca_Kabuli_Chr05	37339022	(T/G)
8845	CakSNP8845	*Kabuli*	Ca_Kabuli_Chr05	37339358	(T/G)
8846	CakSNP8846	*Kabuli*	Ca_Kabuli_Chr05	37339327	(A/G)
8847	CakSNP8847	*Kabuli*	Ca_Kabuli_Chr05	37339300	(A/G)
8848	CakSNP8848	*Kabuli*	Ca_Kabuli_Chr05	37342358	(G/T)
8849	CakSNP8849	*Kabuli*	Ca_Kabuli_Chr05	37363626	(C/T)
8850	CakSNP8850	*Kabuli*	Ca_Kabuli_Chr05	37375575	(C/G)
8851	CakSNP8851	*Kabuli*	Ca_Kabuli_Chr05	37375620	(C/T)
8852	CakSNP8852	*Kabuli*	Ca_Kabuli_Chr05	37445226	(A/G)
8853	CakSNP8853	*Kabuli*	Ca_Kabuli_Chr05	37460980	(A/G)

S.N.	SNP IDs	Cultivars	Chromosomes/scaffolds	Physical positions (bp)	SNPs
8854	CakSNP8854	*Kabuli*	Ca_Kabuli_Chr05	37468727	(T/C)
8855	CakSNP8855	*Kabuli*	Ca_Kabuli_Chr05	37468755	(G/A)
8856	CakSNP8856	*Kabuli*	Ca_Kabuli_Chr05	37555816	(T/A)
8857	CakSNP8857	*Kabuli*	Ca_Kabuli_Chr05	37555846	(T/A)
8858	CakSNP8858	*Kabuli*	Ca_Kabuli_Chr05	37578999	(T/G)
8859	CakSNP8859	*Kabuli*	Ca_Kabuli_Chr05	37591782	(A/T)
8860	CakSNP8860	*Kabuli*	Ca_Kabuli_Chr05	37593444	(A/T)
8861	CakSNP8861	*Kabuli*	Ca_Kabuli_Chr05	37594347	(T/C)
8862	CakSNP8862	*Kabuli*	Ca_Kabuli_Chr05	37604030	(T/C)
8863	CakSNP8863	*Kabuli*	Ca_Kabuli_Chr05	37604090	(G/T)
8864	CakSNP8864	*Kabuli*	Ca_Kabuli_Chr05	37604780	(C/T)
8865	CakSNP8865	*Kabuli*	Ca_Kabuli_Chr05	37796337	(T/G)
8866	CakSNP8866	*Kabuli*	Ca_Kabuli_Chr05	37798313	(T/G)
8867	CakSNP8867	*Kabuli*	Ca_Kabuli_Chr05	37868250	(C/T)
8868	CakSNP8868	*Kabuli*	Ca_Kabuli_Chr05	37868256	(T/A)
8869	CakSNP8869	*Kabuli*	Ca_Kabuli_Chr05	37869744	(T/C)
8870	CakSNP8870	*Kabuli*	Ca_Kabuli_Chr05	37872223	(C/G)
8871	CakSNP8871	*Kabuli*	Ca_Kabuli_Chr05	37874564	(T/A)
8872	CakSNP8872	*Kabuli*	Ca_Kabuli_Chr05	37874725	(A/C)
8873	CakSNP8873	*Kabuli*	Ca_Kabuli_Chr05	37874694	(C/T)
8874	CakSNP8874	*Kabuli*	Ca_Kabuli_Chr05	37886417	(T/G)
8875	CakSNP8875	*Kabuli*	Ca_Kabuli_Chr05	37899098	(T/C)
8876	CakSNP8876	*Kabuli*	Ca_Kabuli_Chr05	37899238	(T/C)
8877	CakSNP8877	*Kabuli*	Ca_Kabuli_Chr05	37908818	(A/C)
8878	CakSNP8878	*Kabuli*	Ca_Kabuli_Chr05	37908807	(A/T)
8879	CakSNP8879	*Kabuli*	Ca_Kabuli_Chr05	37911813	(A/C)
8880	CakSNP8880	*Kabuli*	Ca_Kabuli_Chr05	38018552	(A/T)
8881	CakSNP8881	*Kabuli*	Ca_Kabuli_Chr05	38018558	(G/C)
8882	CakSNP8882	*Kabuli*	Ca_Kabuli_Chr05	38057582	(C/T)
8883	CakSNP8883	*Kabuli*	Ca_Kabuli_Chr05	38080226	(A/G)
8884	CakSNP8884	*Kabuli*	Ca_Kabuli_Chr05	38080349	(C/T)
8885	CakSNP8885	*Kabuli*	Ca_Kabuli_Chr05	38092606	(G/A)
8886	CakSNP8886	*Kabuli*	Ca_Kabuli_Chr05	38092619	(T/C)
8887	CakSNP8887	*Kabuli*	Ca_Kabuli_Chr05	38092732	(C/T)
8888	CakSNP8888	*Kabuli*	Ca_Kabuli_Chr05	38092735	(C/T)
8889	CakSNP8889	*Kabuli*	Ca_Kabuli_Chr05	38092793	(C/T)
8890	CakSNP8890	*Kabuli*	Ca_Kabuli_Chr05	38102941	(T/G)
8891	CakSNP8891	*Kabuli*	Ca_Kabuli_Chr05	38108396	(G/A)

S.N.	SNP IDs	Cultivars	Chromosomes/scaffolds	Physical positions (bp)	SNPs
8892	CakSNP8892	*Kabuli*	Ca_Kabuli_Chr05	38110219	(T/G)
8893	CakSNP8893	*Kabuli*	Ca_Kabuli_Chr05	38220843	(G/A)
8894	CakSNP8894	*Kabuli*	Ca_Kabuli_Chr05	38220908	(G/T)
8895	CakSNP8895	*Kabuli*	Ca_Kabuli_Chr05	38248895	(A/C)
8896	CakSNP8896	*Kabuli*	Ca_Kabuli_Chr05	38249134	(T/C)
8897	CakSNP8897	*Kabuli*	Ca_Kabuli_Chr05	38249132	(A/C)
8898	CakSNP8898	*Kabuli*	Ca_Kabuli_Chr05	38266153	(G/A)
8899	CakSNP8899	*Kabuli*	Ca_Kabuli_Chr05	38307036	(G/A)
8900	CakSNP8900	*Kabuli*	Ca_Kabuli_Chr05	38333718	(C/T)
8901	CakSNP8901	*Kabuli*	Ca_Kabuli_Chr05	38333746	(G/A)
8902	CakSNP8902	*Kabuli*	Ca_Kabuli_Chr05	38333774	(A/G)
8903	CakSNP8903	*Kabuli*	Ca_Kabuli_Chr05	38347201	(A/C)
8904	CakSNP8904	*Kabuli*	Ca_Kabuli_Chr05	38455563	(A/C)
8905	CakSNP8905	*Kabuli*	Ca_Kabuli_Chr05	38509657	(A/C)
8906	CakSNP8906	*Kabuli*	Ca_Kabuli_Chr05	38538904	(A/G)
8907	CakSNP8907	*Kabuli*	Ca_Kabuli_Chr05	38551438	(C/T)
8908	CakSNP8908	*Kabuli*	Ca_Kabuli_Chr05	38551381	(G/A)
8909	CakSNP8909	*Kabuli*	Ca_Kabuli_Chr05	38647040	(A/C)
8910	CakSNP8910	*Kabuli*	Ca_Kabuli_Chr05	38656599	(C/T)
8911	CakSNP8911	*Kabuli*	Ca_Kabuli_Chr05	38783604	(C/A)
8912	CakSNP8912	*Kabuli*	Ca_Kabuli_Chr05	38784078	(G/A)
8913	CakSNP8913	*Kabuli*	Ca_Kabuli_Chr05	38784074	(A/G)
8914	CakSNP8914	*Kabuli*	Ca_Kabuli_Chr05	38784150	(T/A)
8915	CakSNP8915	*Kabuli*	Ca_Kabuli_Chr05	38784129	(G/C)
8916	CakSNP8916	*Kabuli*	Ca_Kabuli_Chr05	38857904	(T/C)
8917	CakSNP8917	*Kabuli*	Ca_Kabuli_Chr05	38872143	(A/G)
8918	CakSNP8918	*Kabuli*	Ca_Kabuli_Chr05	38892604	(A/C)
8919	CakSNP8919	*Kabuli*	Ca_Kabuli_Chr05	38898971	(G/A)
8920	CakSNP8920	*Kabuli*	Ca_Kabuli_Chr05	38909701	(C/T)
8921	CakSNP8921	*Kabuli*	Ca_Kabuli_Chr05	38937304	(C/G)
8922	CakSNP8922	*Kabuli*	Ca_Kabuli_Chr05	38937398	(C/A)
8923	CakSNP8923	*Kabuli*	Ca_Kabuli_Chr05	38939578	(C/A)
8924	CakSNP8924	*Kabuli*	Ca_Kabuli_Chr05	38984762	(A/G)
8925	CakSNP8925	*Kabuli*	Ca_Kabuli_Chr05	38984769	(A/C)
8926	CakSNP8926	*Kabuli*	Ca_Kabuli_Chr05	38984776	(A/G)
8927	CakSNP8927	*Kabuli*	Ca_Kabuli_Chr05	39008980	(G/A)
8928	CakSNP8928	*Kabuli*	Ca_Kabuli_Chr05	39137170	(A/G)
8929	CakSNP8929	*Kabuli*	Ca_Kabuli_Chr05	39175007	(A/T)

S.N.	SNP IDs	Cultivars	Chromosomes/scaffolds	Physical positions (bp)	SNPs
8930	CakSNP8930	*Kabuli*	Ca_Kabuli_Chr05	39176391	(T/A)
8931	CakSNP8931	*Kabuli*	Ca_Kabuli_Chr05	39266333	(T/C)
8932	CakSNP8932	*Kabuli*	Ca_Kabuli_Chr05	39277581	(T/C)
8933	CakSNP8933	*Kabuli*	Ca_Kabuli_Chr05	39283856	(G/A)
8934	CakSNP8934	*Kabuli*	Ca_Kabuli_Chr05	39326933	(G/T)
8935	CakSNP8935	*Kabuli*	Ca_Kabuli_Chr05	39353755	(T/G)
8936	CakSNP8936	*Kabuli*	Ca_Kabuli_Chr05	39364522	(T/C)
8937	CakSNP8937	*Kabuli*	Ca_Kabuli_Chr05	39364569	(G/A)
8938	CakSNP8938	*Kabuli*	Ca_Kabuli_Chr05	39364571	(C/A)
8939	CakSNP8939	*Kabuli*	Ca_Kabuli_Chr05	39364720	(A/T)
8940	CakSNP8940	*Kabuli*	Ca_Kabuli_Chr05	39364700	(C/A)
8941	CakSNP8941	*Kabuli*	Ca_Kabuli_Chr05	39364695	(A/C)
8942	CakSNP8942	*Kabuli*	Ca_Kabuli_Chr05	39364667	(T/A)
8943	CakSNP8943	*Kabuli*	Ca_Kabuli_Chr05	39364741	(A/T)
8944	CakSNP8944	*Kabuli*	Ca_Kabuli_Chr05	39364752	(G/T)
8945	CakSNP8945	*Kabuli*	Ca_Kabuli_Chr05	39364895	(A/C)
8946	CakSNP8946	*Kabuli*	Ca_Kabuli_Chr05	39364856	(A/G)
8947	CakSNP8947	*Kabuli*	Ca_Kabuli_Chr05	39364848	(C/T)
8948	CakSNP8948	*Kabuli*	Ca_Kabuli_Chr05	39364841	(A/T)
8949	CakSNP8949	*Kabuli*	Ca_Kabuli_Chr05	39366353	(C/G)
8950	CakSNP8950	*Kabuli*	Ca_Kabuli_Chr05	39385603	(T/G)
8951	CakSNP8951	*Kabuli*	Ca_Kabuli_Chr05	39385630	(C/T)
8952	CakSNP8952	*Kabuli*	Ca_Kabuli_Chr05	39410377	(T/G)
8953	CakSNP8953	*Kabuli*	Ca_Kabuli_Chr05	39410329	(T/G)
8954	CakSNP8954	*Kabuli*	Ca_Kabuli_Chr05	39460567	(A/C)
8955	CakSNP8955	*Kabuli*	Ca_Kabuli_Chr05	39475960	(T/C)
8956	CakSNP8956	*Kabuli*	Ca_Kabuli_Chr05	39476040	(T/G)
8957	CakSNP8957	*Kabuli*	Ca_Kabuli_Chr05	39498665	(G/A)
8958	CakSNP8958	*Kabuli*	Ca_Kabuli_Chr05	39513563	(C/A)
8959	CakSNP8959	*Kabuli*	Ca_Kabuli_Chr05	39543929	(C/T)
8960	CakSNP8960	*Kabuli*	Ca_Kabuli_Chr05	39576823	(C/T)
8961	CakSNP8961	*Kabuli*	Ca_Kabuli_Chr05	39576836	(T/A)
8962	CakSNP8962	*Kabuli*	Ca_Kabuli_Chr05	39576839	(C/T)
8963	CakSNP8963	*Kabuli*	Ca_Kabuli_Chr05	39576864	(C/A)
8964	CakSNP8964	*Kabuli*	Ca_Kabuli_Chr05	39576870	(C/T)
8965	CakSNP8965	*Kabuli*	Ca_Kabuli_Chr05	39576874	(G/T)
8966	CakSNP8966	*Kabuli*	Ca_Kabuli_Chr05	39576841	(G/A)
8967	CakSNP8967	*Kabuli*	Ca_Kabuli_Chr05	39592439	(A/G)

S.N.	SNP IDs	Cultivars	Chromosomes/scaffolds	Physical positions (bp)	SNPs
8968	CakSNP8968	*Kabuli*	Ca_Kabuli_Chr05	39597485	(C/T)
8969	CakSNP8969	*Kabuli*	Ca_Kabuli_Chr05	39597459	(T/C)
8970	CakSNP8970	*Kabuli*	Ca_Kabuli_Chr05	39608204	(A/G)
8971	CakSNP8971	*Kabuli*	Ca_Kabuli_Chr05	39608209	(G/T)
8972	CakSNP8972	*Kabuli*	Ca_Kabuli_Chr05	39684480	(A/G)
8973	CakSNP8973	*Kabuli*	Ca_Kabuli_Chr05	39684592	(A/T)
8974	CakSNP8974	*Kabuli*	Ca_Kabuli_Chr05	39713021	(G/A)
8975	CakSNP8975	*Kabuli*	Ca_Kabuli_Chr05	39776794	(G/A)
8976	CakSNP8976	*Kabuli*	Ca_Kabuli_Chr05	39776795	(G/A)
8977	CakSNP8977	*Kabuli*	Ca_Kabuli_Chr05	39776818	(C/T)
8978	CakSNP8978	*Kabuli*	Ca_Kabuli_Chr05	39776850	(G/A)
8979	CakSNP8979	*Kabuli*	Ca_Kabuli_Chr05	39776869	(C/T)
8980	CakSNP8980	*Kabuli*	Ca_Kabuli_Chr05	39794718	(C/T)
8981	CakSNP8981	*Kabuli*	Ca_Kabuli_Chr05	39851570	(T/G)
8982	CakSNP8982	*Kabuli*	Ca_Kabuli_Chr05	39869398	(C/T)
8983	CakSNP8983	*Kabuli*	Ca_Kabuli_Chr05	39943298	(C/T)
8984	CakSNP8984	*Kabuli*	Ca_Kabuli_Chr05	39985042	(G/A)
8985	CakSNP8985	*Kabuli*	Ca_Kabuli_Chr05	39985332	(A/G)
8986	CakSNP8986	*Kabuli*	Ca_Kabuli_Chr05	40018264	(A/C)
8987	CakSNP8987	*Kabuli*	Ca_Kabuli_Chr05	40020062	(C/T)
8988	CakSNP8988	*Kabuli*	Ca_Kabuli_Chr05	40020073	(C/G)
8989	CakSNP8989	*Kabuli*	Ca_Kabuli_Chr05	40025250	(G/A)
8990	CakSNP8990	*Kabuli*	Ca_Kabuli_Chr05	40025319	(T/C)
8991	CakSNP8991	*Kabuli*	Ca_Kabuli_Chr05	40025326	(C/T)
8992	CakSNP8992	*Kabuli*	Ca_Kabuli_Chr05	40025376	(A/G)
8993	CakSNP8993	*Kabuli*	Ca_Kabuli_Chr05	40025377	(T/A)
8994	CakSNP8994	*Kabuli*	Ca_Kabuli_Chr05	40027707	(A/G)
8995	CakSNP8995	*Kabuli*	Ca_Kabuli_Chr05	40078085	(C/G)
8996	CakSNP8996	*Kabuli*	Ca_Kabuli_Chr05	40078077	(A/G)
8997	CakSNP8997	*Kabuli*	Ca_Kabuli_Chr05	40159260	(C/A)
8998	CakSNP8998	*Kabuli*	Ca_Kabuli_Chr05	40159348	(G/A)
8999	CakSNP8999	*Kabuli*	Ca_Kabuli_Chr05	40162755	(T/C)
9000	CakSNP9000	*Kabuli*	Ca_Kabuli_Chr05	40162995	(C/T)
9001	CakSNP9001	*Kabuli*	Ca_Kabuli_Chr05	40164176	(C/G)
9002	CakSNP9002	*Kabuli*	Ca_Kabuli_Chr05	40239397	(G/C)
9003	CakSNP9003	*Kabuli*	Ca_Kabuli_Chr05	40290082	(G/A)
9004	CakSNP9004	*Kabuli*	Ca_Kabuli_Chr05	40290066	(A/G)
9005	CakSNP9005	*Kabuli*	Ca_Kabuli_Chr05	40404136	(C/T)

S.N.	SNP IDs	Cultivars	Chromosomes/scaffolds	Physical positions (bp)	SNPs
9006	CakSNP9006	*Kabuli*	Ca_Kabuli_Chr05	40404105	(G/C)
9007	CakSNP9007	*Kabuli*	Ca_Kabuli_Chr05	40410891	(C/A)
9008	CakSNP9008	*Kabuli*	Ca_Kabuli_Chr05	40410895	(G/T)
9009	CakSNP9009	*Kabuli*	Ca_Kabuli_Chr05	40411325	(G/C)
9010	CakSNP9010	*Kabuli*	Ca_Kabuli_Chr05	40464608	(A/G)
9011	CakSNP9011	*Kabuli*	Ca_Kabuli_Chr05	40505498	(T/G)
9012	CakSNP9012	*Kabuli*	Ca_Kabuli_Chr05	40507018	(T/A)
9013	CakSNP9013	*Kabuli*	Ca_Kabuli_Chr05	40507107	(A/G)
9014	CakSNP9014	*Kabuli*	Ca_Kabuli_Chr05	40507116	(A/G)
9015	CakSNP9015	*Kabuli*	Ca_Kabuli_Chr05	40507158	(A/G)
9016	CakSNP9016	*Kabuli*	Ca_Kabuli_Chr05	40507653	(G/C)
9017	CakSNP9017	*Kabuli*	Ca_Kabuli_Chr05	40507662	(C/T)
9018	CakSNP9018	*Kabuli*	Ca_Kabuli_Chr05	40507683	(G/T)
9019	CakSNP9019	*Kabuli*	Ca_Kabuli_Chr05	40532435	(A/G)
9020	CakSNP9020	*Kabuli*	Ca_Kabuli_Chr05	40543989	(G/T)
9021	CakSNP9021	*Kabuli*	Ca_Kabuli_Chr05	40553279	(C/G)
9022	CakSNP9022	*Kabuli*	Ca_Kabuli_Chr05	40631069	(G/C)
9023	CakSNP9023	*Kabuli*	Ca_Kabuli_Chr05	40666160	(T/A)
9024	CakSNP9024	*Kabuli*	Ca_Kabuli_Chr05	40666171	(T/C)
9025	CakSNP9025	*Kabuli*	Ca_Kabuli_Chr05	40666174	(C/T)
9026	CakSNP9026	*Kabuli*	Ca_Kabuli_Chr05	40673241	(A/G)
9027	CakSNP9027	*Kabuli*	Ca_Kabuli_Chr05	40700278	(C/G)
9028	CakSNP9028	*Kabuli*	Ca_Kabuli_Chr05	40700260	(T/C)
9029	CakSNP9029	*Kabuli*	Ca_Kabuli_Chr05	40700318	(C/A)
9030	CakSNP9030	*Kabuli*	Ca_Kabuli_Chr05	40700772	(C/T)
9031	CakSNP9031	*Kabuli*	Ca_Kabuli_Chr05	40736291	(C/G)
9032	CakSNP9032	*Kabuli*	Ca_Kabuli_Chr05	40828566	(A/C)
9033	CakSNP9033	*Kabuli*	Ca_Kabuli_Chr05	40828567	(G/C)
9034	CakSNP9034	*Kabuli*	Ca_Kabuli_Chr05	40841690	(C/T)
9035	CakSNP9035	*Kabuli*	Ca_Kabuli_Chr05	40845320	(C/T)
9036	CakSNP9036	*Kabuli*	Ca_Kabuli_Chr05	40883438	(C/A)
9037	CakSNP9037	*Kabuli*	Ca_Kabuli_Chr05	40955055	(A/T)
9038	CakSNP9038	*Kabuli*	Ca_Kabuli_Chr05	40966058	(G/A)
9039	CakSNP9039	*Kabuli*	Ca_Kabuli_Chr05	41024079	(A/G)
9040	CakSNP9040	*Kabuli*	Ca_Kabuli_Chr05	41024135	(C/G)
9041	CakSNP9041	*Kabuli*	Ca_Kabuli_Chr05	41024201	(G/A)
9042	CakSNP9042	*Kabuli*	Ca_Kabuli_Chr05	41054578	(A/G)
9043	CakSNP9043	*Kabuli*	Ca_Kabuli_Chr05	41055111	(T/A)

S.N.	SNP IDs	Cultivars	Chromosomes/scaffolds	Physical positions (bp)	SNPs
9044	CakSNP9044	*Kabuli*	Ca_Kabuli_Chr05	41063657	(C/T)
9045	CakSNP9045	*Kabuli*	Ca_Kabuli_Chr05	41066644	(C/G)
9046	CakSNP9046	*Kabuli*	Ca_Kabuli_Chr05	41066664	(T/C)
9047	CakSNP9047	*Kabuli*	Ca_Kabuli_Chr05	41066667	(C/A)
9048	CakSNP9048	*Kabuli*	Ca_Kabuli_Chr05	41080035	(C/T)
9049	CakSNP9049	*Kabuli*	Ca_Kabuli_Chr05	41191566	(A/T)
9050	CakSNP9050	*Kabuli*	Ca_Kabuli_Chr05	41191676	(G/C)
9051	CakSNP9051	*Kabuli*	Ca_Kabuli_Chr05	41197909	(A/T)
9052	CakSNP9052	*Kabuli*	Ca_Kabuli_Chr05	41203594	(C/T)
9053	CakSNP9053	*Kabuli*	Ca_Kabuli_Chr05	41253742	(A/G)
9054	CakSNP9054	*Kabuli*	Ca_Kabuli_Chr05	41283253	(T/C)
9055	CakSNP9055	*Kabuli*	Ca_Kabuli_Chr05	41283453	(A/C)
9056	CakSNP9056	*Kabuli*	Ca_Kabuli_Chr05	41285223	(A/C)
9057	CakSNP9057	*Kabuli*	Ca_Kabuli_Chr05	41302544	(C/G)
9058	CakSNP9058	*Kabuli*	Ca_Kabuli_Chr05	41302601	(G/A)
9059	CakSNP9059	*Kabuli*	Ca_Kabuli_Chr05	41302733	(T/C)
9060	CakSNP9060	*Kabuli*	Ca_Kabuli_Chr05	41302804	(A/G)
9061	CakSNP9061	*Kabuli*	Ca_Kabuli_Chr05	41302859	(C/A)
9062	CakSNP9062	*Kabuli*	Ca_Kabuli_Chr05	41302901	(C/A)
9063	CakSNP9063	*Kabuli*	Ca_Kabuli_Chr05	41302900	(T/A)
9064	CakSNP9064	*Kabuli*	Ca_Kabuli_Chr05	41302895	(G/A)
9065	CakSNP9065	*Kabuli*	Ca_Kabuli_Chr05	41304103	(G/A)
9066	CakSNP9066	*Kabuli*	Ca_Kabuli_Chr05	41399241	(C/T)
9067	CakSNP9067	*Kabuli*	Ca_Kabuli_Chr05	41407077	(A/C)
9068	CakSNP9068	*Kabuli*	Ca_Kabuli_Chr05	41407253	(T/C)
9069	CakSNP9069	*Kabuli*	Ca_Kabuli_Chr05	41519141	(C/T)
9070	CakSNP9070	*Kabuli*	Ca_Kabuli_Chr05	41521854	(T/G)
9071	CakSNP9071	*Kabuli*	Ca_Kabuli_Chr05	41526026	(T/G)
9072	CakSNP9072	*Kabuli*	Ca_Kabuli_Chr05	41635262	(C/T)
9073	CakSNP9073	*Kabuli*	Ca_Kabuli_Chr05	41678144	(C/T)
9074	CakSNP9074	*Kabuli*	Ca_Kabuli_Chr05	41696426	(T/C)
9075	CakSNP9075	*Kabuli*	Ca_Kabuli_Chr05	41696496	(G/C)
9076	CakSNP9076	*Kabuli*	Ca_Kabuli_Chr05	41701479	(T/C)
9077	CakSNP9077	*Kabuli*	Ca_Kabuli_Chr05	41772759	(G/A)
9078	CakSNP9078	*Kabuli*	Ca_Kabuli_Chr05	41887214	(A/T)
9079	CakSNP9079	*Kabuli*	Ca_Kabuli_Chr05	41902419	(A/C)
9080	CakSNP9080	*Kabuli*	Ca_Kabuli_Chr05	41945316	(A/G)
9081	CakSNP9081	*Kabuli*	Ca_Kabuli_Chr05	41945373	(C/T)

S.N.	SNP IDs	Cultivars	Chromosomes/scaffolds	Physical positions (bp)	SNPs
9082	CakSNP9082	*Kabuli*	Ca_Kabuli_Chr05	41945308	(G/A)
9083	CakSNP9083	*Kabuli*	Ca_Kabuli_Chr05	41974690	(T/C)
9084	CakSNP9084	*Kabuli*	Ca_Kabuli_Chr05	42004600	(T/G)
9085	CakSNP9085	*Kabuli*	Ca_Kabuli_Chr05	42004602	(A/G)
9086	CakSNP9086	*Kabuli*	Ca_Kabuli_Chr05	42004734	(G/A)
9087	CakSNP9087	*Kabuli*	Ca_Kabuli_Chr05	42004742	(G/A)
9088	CakSNP9088	*Kabuli*	Ca_Kabuli_Chr05	42004751	(G/A)
9089	CakSNP9089	*Kabuli*	Ca_Kabuli_Chr05	42004753	(C/A)
9090	CakSNP9090	*Kabuli*	Ca_Kabuli_Chr05	42008626	(A/C)
9091	CakSNP9091	*Kabuli*	Ca_Kabuli_Chr05	42012972	(C/A)
9092	CakSNP9092	*Kabuli*	Ca_Kabuli_Chr05	42012983	(G/A)
9093	CakSNP9093	*Kabuli*	Ca_Kabuli_Chr05	42012998	(C/T)
9094	CakSNP9094	*Kabuli*	Ca_Kabuli_Chr05	42020435	(A/G)
9095	CakSNP9095	*Kabuli*	Ca_Kabuli_Chr05	42100070	(A/G)
9096	CakSNP9096	*Kabuli*	Ca_Kabuli_Chr05	42117516	(C/T)
9097	CakSNP9097	*Kabuli*	Ca_Kabuli_Chr05	42120698	(T/C)
9098	CakSNP9098	*Kabuli*	Ca_Kabuli_Chr05	42120735	(G/C)
9099	CakSNP9099	*Kabuli*	Ca_Kabuli_Chr05	42121472	(C/A)
9100	CakSNP9100	*Kabuli*	Ca_Kabuli_Chr05	42225634	(G/A)
9101	CakSNP9101	*Kabuli*	Ca_Kabuli_Chr05	42275523	(G/A)
9102	CakSNP9102	*Kabuli*	Ca_Kabuli_Chr05	42282297	(G/A)
9103	CakSNP9103	*Kabuli*	Ca_Kabuli_Chr05	42413430	(A/C)
9104	CakSNP9104	*Kabuli*	Ca_Kabuli_Chr05	42422867	(C/A)
9105	CakSNP9105	*Kabuli*	Ca_Kabuli_Chr05	42422845	(T/C)
9106	CakSNP9106	*Kabuli*	Ca_Kabuli_Chr05	42422837	(G/A)
9107	CakSNP9107	*Kabuli*	Ca_Kabuli_Chr05	42422808	(A/G)
9108	CakSNP9108	*Kabuli*	Ca_Kabuli_Chr05	42422932	(C/A)
9109	CakSNP9109	*Kabuli*	Ca_Kabuli_Chr05	42435263	(C/T)
9110	CakSNP9110	*Kabuli*	Ca_Kabuli_Chr05	42436972	(A/G)
9111	CakSNP9111	*Kabuli*	Ca_Kabuli_Chr05	42436976	(A/T)
9112	CakSNP9112	*Kabuli*	Ca_Kabuli_Chr05	42443786	(A/G)
9113	CakSNP9113	*Kabuli*	Ca_Kabuli_Chr05	42477712	(G/A)
9114	CakSNP9114	*Kabuli*	Ca_Kabuli_Chr05	42485130	(T/C)
9115	CakSNP9115	*Kabuli*	Ca_Kabuli_Chr05	42528744	(T/G)
9116	CakSNP9116	*Kabuli*	Ca_Kabuli_Chr05	42528934	(A/G)
9117	CakSNP9117	*Kabuli*	Ca_Kabuli_Chr05	42540675	(A/T)
9118	CakSNP9118	*Kabuli*	Ca_Kabuli_Chr05	42582262	(T/A)
9119	CakSNP9119	*Kabuli*	Ca_Kabuli_Chr05	42655824	(A/G)

S.N.	SNP IDs	Cultivars	Chromosomes/scaffolds	Physical positions (bp)	SNPs
9120	CakSNP9120	*Kabuli*	Ca_Kabuli_Chr05	42749499	(C/T)
9121	CakSNP9121	*Kabuli*	Ca_Kabuli_Chr05	42792635	(G/A)
9122	CakSNP9122	*Kabuli*	Ca_Kabuli_Chr05	42814621	(C/T)
9123	CakSNP9123	*Kabuli*	Ca_Kabuli_Chr05	42814623	(A/G)
9124	CakSNP9124	*Kabuli*	Ca_Kabuli_Chr05	42857977	(G/C)
9125	CakSNP9125	*Kabuli*	Ca_Kabuli_Chr05	42863366	(C/T)
9126	CakSNP9126	*Kabuli*	Ca_Kabuli_Chr05	42872074	(A/C)
9127	CakSNP9127	*Kabuli*	Ca_Kabuli_Chr05	42872091	(A/G)
9128	CakSNP9128	*Kabuli*	Ca_Kabuli_Chr05	42881185	(C/T)
9129	CakSNP9129	*Kabuli*	Ca_Kabuli_Chr05	42882945	(C/T)
9130	CakSNP9130	*Kabuli*	Ca_Kabuli_Chr05	42882965	(C/T)
9131	CakSNP9131	*Kabuli*	Ca_Kabuli_Chr05	42883163	(T/C)
9132	CakSNP9132	*Kabuli*	Ca_Kabuli_Chr05	42910653	(A/G)
9133	CakSNP9133	*Kabuli*	Ca_Kabuli_Chr05	42910661	(A/G)
9134	CakSNP9134	*Kabuli*	Ca_Kabuli_Chr05	42910732	(A/T)
9135	CakSNP9135	*Kabuli*	Ca_Kabuli_Chr05	42924113	(C/T)
9136	CakSNP9136	*Kabuli*	Ca_Kabuli_Chr05	42928986	(T/G)
9137	CakSNP9137	*Kabuli*	Ca_Kabuli_Chr05	42929087	(A/C)
9138	CakSNP9138	*Kabuli*	Ca_Kabuli_Chr05	42946580	(T/C)
9139	CakSNP9139	*Kabuli*	Ca_Kabuli_Chr05	42946716	(A/T)
9140	CakSNP9140	*Kabuli*	Ca_Kabuli_Chr05	42999704	(C/T)
9141	CakSNP9141	*Kabuli*	Ca_Kabuli_Chr05	43000210	(A/G)
9142	CakSNP9142	*Kabuli*	Ca_Kabuli_Chr05	43034618	(T/A)
9143	CakSNP9143	*Kabuli*	Ca_Kabuli_Chr05	43037863	(G/A)
9144	CakSNP9144	*Kabuli*	Ca_Kabuli_Chr05	43060091	(C/T)
9145	CakSNP9145	*Kabuli*	Ca_Kabuli_Chr05	43067894	(T/C)
9146	CakSNP9146	*Kabuli*	Ca_Kabuli_Chr05	43067891	(G/C)
9147	CakSNP9147	*Kabuli*	Ca_Kabuli_Chr05	43067905	(A/C)
9148	CakSNP9148	*Kabuli*	Ca_Kabuli_Chr05	43080493	(G/A)
9149	CakSNP9149	*Kabuli*	Ca_Kabuli_Chr05	43081783	(G/A)
9150	CakSNP9150	*Kabuli*	Ca_Kabuli_Chr05	43110660	(G/A)
9151	CakSNP9151	*Kabuli*	Ca_Kabuli_Chr05	43110669	(A/T)
9152	CakSNP9152	*Kabuli*	Ca_Kabuli_Chr05	43110726	(T/G)
9153	CakSNP9153	*Kabuli*	Ca_Kabuli_Chr05	43110737	(A/G)
9154	CakSNP9154	*Kabuli*	Ca_Kabuli_Chr05	43175781	(G/A)
9155	CakSNP9155	*Kabuli*	Ca_Kabuli_Chr05	43188824	(T/G)
9156	CakSNP9156	*Kabuli*	Ca_Kabuli_Chr05	43188853	(C/A)
9157	CakSNP9157	*Kabuli*	Ca_Kabuli_Chr05	43210793	(T/C)

S.N.	SNP IDs	Cultivars	Chromosomes/scaffolds	Physical positions (bp)	SNPs
9158	CakSNP9158	*Kabuli*	Ca_Kabuli_Chr05	43212083	(G/A)
9159	CakSNP9159	*Kabuli*	Ca_Kabuli_Chr05	43212066	(C/T)
9160	CakSNP9160	*Kabuli*	Ca_Kabuli_Chr05	43221125	(T/C)
9161	CakSNP9161	*Kabuli*	Ca_Kabuli_Chr05	43231220	(T/C)
9162	CakSNP9162	*Kabuli*	Ca_Kabuli_Chr05	43231265	(C/T)
9163	CakSNP9163	*Kabuli*	Ca_Kabuli_Chr05	43253586	(C/A)
9164	CakSNP9164	*Kabuli*	Ca_Kabuli_Chr05	43275472	(T/C)
9165	CakSNP9165	*Kabuli*	Ca_Kabuli_Chr05	43334903	(T/C)
9166	CakSNP9166	*Kabuli*	Ca_Kabuli_Chr05	43341646	(A/C)
9167	CakSNP9167	*Kabuli*	Ca_Kabuli_Chr05	43402230	(C/T)
9168	CakSNP9168	*Kabuli*	Ca_Kabuli_Chr05	43410326	(T/G)
9169	CakSNP9169	*Kabuli*	Ca_Kabuli_Chr05	43423593	(G/C)
9170	CakSNP9170	*Kabuli*	Ca_Kabuli_Chr05	43521774	(A/T)
9171	CakSNP9171	*Kabuli*	Ca_Kabuli_Chr05	43521789	(G/A)
9172	CakSNP9172	*Kabuli*	Ca_Kabuli_Chr05	43521830	(T/C)
9173	CakSNP9173	*Kabuli*	Ca_Kabuli_Chr05	43521851	(G/A)
9174	CakSNP9174	*Kabuli*	Ca_Kabuli_Chr05	43523560	(A/G)
9175	CakSNP9175	*Kabuli*	Ca_Kabuli_Chr05	43530567	(C/T)
9176	CakSNP9176	*Kabuli*	Ca_Kabuli_Chr05	43565712	(T/C)
9177	CakSNP9177	*Kabuli*	Ca_Kabuli_Chr05	43632249	(C/A)
9178	CakSNP9178	*Kabuli*	Ca_Kabuli_Chr05	43632281	(C/G)
9179	CakSNP9179	*Kabuli*	Ca_Kabuli_Chr05	43632349	(T/A)
9180	CakSNP9180	*Kabuli*	Ca_Kabuli_Chr05	43632400	(A/G)
9181	CakSNP9181	*Kabuli*	Ca_Kabuli_Chr05	43632377	(C/T)
9182	CakSNP9182	*Kabuli*	Ca_Kabuli_Chr05	43632481	(G/T)
9183	CakSNP9183	*Kabuli*	Ca_Kabuli_Chr05	43632508	(A/G)
9184	CakSNP9184	*Kabuli*	Ca_Kabuli_Chr05	43710359	(T/G)
9185	CakSNP9185	*Kabuli*	Ca_Kabuli_Chr05	43710362	(A/T)
9186	CakSNP9186	*Kabuli*	Ca_Kabuli_Chr05	43710366	(G/T)
9187	CakSNP9187	*Kabuli*	Ca_Kabuli_Chr05	43710391	(A/G)
9188	CakSNP9188	*Kabuli*	Ca_Kabuli_Chr05	43742448	(C/T)
9189	CakSNP9189	*Kabuli*	Ca_Kabuli_Chr05	43742456	(G/A)
9190	CakSNP9190	*Kabuli*	Ca_Kabuli_Chr05	43755074	(A/G)
9191	CakSNP9191	*Kabuli*	Ca_Kabuli_Chr05	43755756	(A/G)
9192	CakSNP9192	*Kabuli*	Ca_Kabuli_Chr05	43759059	(A/C)
9193	CakSNP9193	*Kabuli*	Ca_Kabuli_Chr05	43786883	(A/G)
9194	CakSNP9194	*Kabuli*	Ca_Kabuli_Chr05	43789875	(C/G)
9195	CakSNP9195	*Kabuli*	Ca_Kabuli_Chr05	43790108	(G/A)

S.N.	SNP IDs	Cultivars	Chromosomes/scaffolds	Physical positions (bp)	SNPs
9196	CakSNP9196	*Kabuli*	Ca_Kabuli_Chr05	43812119	(G/A)
9197	CakSNP9197	*Kabuli*	Ca_Kabuli_Chr05	43812213	(C/T)
9198	CakSNP9198	*Kabuli*	Ca_Kabuli_Chr05	43859301	(G/A)
9199	CakSNP9199	*Kabuli*	Ca_Kabuli_Chr05	43904489	(T/A)
9200	CakSNP9200	*Kabuli*	Ca_Kabuli_Chr05	43919009	(C/T)
9201	CakSNP9201	*Kabuli*	Ca_Kabuli_Chr05	43919017	(T/G)
9202	CakSNP9202	*Kabuli*	Ca_Kabuli_Chr05	43919022	(G/C)
9203	CakSNP9203	*Kabuli*	Ca_Kabuli_Chr05	43919028	(A/G)
9204	CakSNP9204	*Kabuli*	Ca_Kabuli_Chr05	43982994	(C/T)
9205	CakSNP9205	*Kabuli*	Ca_Kabuli_Chr05	43995843	(T/C)
9206	CakSNP9206	*Kabuli*	Ca_Kabuli_Chr05	44002824	(T/G)
9207	CakSNP9207	*Kabuli*	Ca_Kabuli_Chr05	44002862	(C/A)
9208	CakSNP9208	*Kabuli*	Ca_Kabuli_Chr05	44038229	(T/C)
9209	CakSNP9209	*Kabuli*	Ca_Kabuli_Chr05	44064495	(G/A)
9210	CakSNP9210	*Kabuli*	Ca_Kabuli_Chr05	44080450	(G/C)
9211	CakSNP9211	*Kabuli*	Ca_Kabuli_Chr05	44109260	(G/T)
9212	CakSNP9212	*Kabuli*	Ca_Kabuli_Chr05	44154671	(A/G)
9213	CakSNP9213	*Kabuli*	Ca_Kabuli_Chr05	44159237	(C/G)
9214	CakSNP9214	*Kabuli*	Ca_Kabuli_Chr05	44289612	(T/C)
9215	CakSNP9215	*Kabuli*	Ca_Kabuli_Chr05	44334286	(C/G)
9216	CakSNP9216	*Kabuli*	Ca_Kabuli_Chr05	44334318	(C/T)
9217	CakSNP9217	*Kabuli*	Ca_Kabuli_Chr05	44364134	(C/T)
9218	CakSNP9218	*Kabuli*	Ca_Kabuli_Chr05	44373968	(G/T)
9219	CakSNP9219	*Kabuli*	Ca_Kabuli_Chr05	44378178	(C/A)
9220	CakSNP9220	*Kabuli*	Ca_Kabuli_Chr05	44428965	(G/A)
9221	CakSNP9221	*Kabuli*	Ca_Kabuli_Chr05	44461250	(A/G)
9222	CakSNP9222	*Kabuli*	Ca_Kabuli_Chr05	44491489	(G/T)
9223	CakSNP9223	*Kabuli*	Ca_Kabuli_Chr05	44555718	(A/C)
9224	CakSNP9224	*Kabuli*	Ca_Kabuli_Chr05	44561095	(T/C)
9225	CakSNP9225	*Kabuli*	Ca_Kabuli_Chr05	44656921	(G/A)
9226	CakSNP9226	*Kabuli*	Ca_Kabuli_Chr05	44667285	(C/T)
9227	CakSNP9227	*Kabuli*	Ca_Kabuli_Chr05	44667297	(C/T)
9228	CakSNP9228	*Kabuli*	Ca_Kabuli_Chr05	44667359	(C/A)
9229	CakSNP9229	*Kabuli*	Ca_Kabuli_Chr05	44760403	(A/G)
9230	CakSNP9230	*Kabuli*	Ca_Kabuli_Chr05	44760347	(G/T)
9231	CakSNP9231	*Kabuli*	Ca_Kabuli_Chr05	44760469	(C/T)
9232	CakSNP9232	*Kabuli*	Ca_Kabuli_Chr05	44760490	(C/A)
9233	CakSNP9233	*Kabuli*	Ca_Kabuli_Chr05	44772326	(G/A)

S.N.	SNP IDs	Cultivars	Chromosomes/scaffolds	Physical positions (bp)	SNPs
9234	CakSNP9234	*Kabuli*	Ca_Kabuli_Chr05	44884779	(G/C)
9235	CakSNP9235	*Kabuli*	Ca_Kabuli_Chr05	44887105	(T/G)
9236	CakSNP9236	*Kabuli*	Ca_Kabuli_Chr05	44887241	(T/C)
9237	CakSNP9237	*Kabuli*	Ca_Kabuli_Chr05	44887234	(A/C)
9238	CakSNP9238	*Kabuli*	Ca_Kabuli_Chr05	44891426	(G/C)
9239	CakSNP9239	*Kabuli*	Ca_Kabuli_Chr05	44911842	(C/T)
9240	CakSNP9240	*Kabuli*	Ca_Kabuli_Chr05	44911868	(C/G)
9241	CakSNP9241	*Kabuli*	Ca_Kabuli_Chr05	44911881	(G/A)
9242	CakSNP9242	*Kabuli*	Ca_Kabuli_Chr05	44911906	(C/T)
9243	CakSNP9243	*Kabuli*	Ca_Kabuli_Chr05	44918947	(C/A)
9244	CakSNP9244	*Kabuli*	Ca_Kabuli_Chr05	44938814	(A/T)
9245	CakSNP9245	*Kabuli*	Ca_Kabuli_Chr05	44938832	(A/G)
9246	CakSNP9246	*Kabuli*	Ca_Kabuli_Chr05	44939058	(C/G)
9247	CakSNP9247	*Kabuli*	Ca_Kabuli_Chr05	44939033	(G/A)
9248	CakSNP9248	*Kabuli*	Ca_Kabuli_Chr05	45087170	(G/A)
9249	CakSNP9249	*Kabuli*	Ca_Kabuli_Chr05	45087156	(A/G)
9250	CakSNP9250	*Kabuli*	Ca_Kabuli_Chr05	45087146	(C/G)
9251	CakSNP9251	*Kabuli*	Ca_Kabuli_Chr05	45087140	(C/A)
9252	CakSNP9252	*Kabuli*	Ca_Kabuli_Chr05	45087202	(G/C)
9253	CakSNP9253	*Kabuli*	Ca_Kabuli_Chr05	45087267	(A/G)
9254	CakSNP9254	*Kabuli*	Ca_Kabuli_Chr05	45087228	(A/C)
9255	CakSNP9255	*Kabuli*	Ca_Kabuli_Chr05	45087215	(G/A)
9256	CakSNP9256	*Kabuli*	Ca_Kabuli_Chr05	45215617	(G/A)
9257	CakSNP9257	*Kabuli*	Ca_Kabuli_Chr05	45219125	(C/T)
9258	CakSNP9258	*Kabuli*	Ca_Kabuli_Chr05	45219258	(G/T)
9259	CakSNP9259	*Kabuli*	Ca_Kabuli_Chr05	45219355	(T/G)
9260	CakSNP9260	*Kabuli*	Ca_Kabuli_Chr05	45254102	(C/T)
9261	CakSNP9261	*Kabuli*	Ca_Kabuli_Chr05	45254112	(T/G)
9262	CakSNP9262	*Kabuli*	Ca_Kabuli_Chr05	45301772	(T/G)
9263	CakSNP9263	*Kabuli*	Ca_Kabuli_Chr05	45337274	(C/G)
9264	CakSNP9264	*Kabuli*	Ca_Kabuli_Chr05	45337307	(C/T)
9265	CakSNP9265	*Kabuli*	Ca_Kabuli_Chr05	45406853	(C/T)
9266	CakSNP9266	*Kabuli*	Ca_Kabuli_Chr05	45407063	(G/A)
9267	CakSNP9267	*Kabuli*	Ca_Kabuli_Chr05	45407044	(A/C)
9268	CakSNP9268	*Kabuli*	Ca_Kabuli_Chr05	45414089	(C/G)
9269	CakSNP9269	*Kabuli*	Ca_Kabuli_Chr05	45428229	(A/G)
9270	CakSNP9270	*Kabuli*	Ca_Kabuli_Chr05	45429513	(A/G)
9271	CakSNP9271	*Kabuli*	Ca_Kabuli_Chr05	45429518	(T/G)

S.N.	SNP IDs	Cultivars	Chromosomes/scaffolds	Physical positions (bp)	SNPs
9272	CakSNP9272	*Kabuli*	Ca_Kabuli_Chr05	45441223	(C/T)
9273	CakSNP9273	*Kabuli*	Ca_Kabuli_Chr05	45444675	(G/T)
9274	CakSNP9274	*Kabuli*	Ca_Kabuli_Chr05	45444924	(G/T)
9275	CakSNP9275	*Kabuli*	Ca_Kabuli_Chr05	45480175	(T/G)
9276	CakSNP9276	*Kabuli*	Ca_Kabuli_Chr05	45501379	(C/T)
9277	CakSNP9277	*Kabuli*	Ca_Kabuli_Chr05	45540473	(G/A)
9278	CakSNP9278	*Kabuli*	Ca_Kabuli_Chr05	45559874	(C/A)
9279	CakSNP9279	*Kabuli*	Ca_Kabuli_Chr05	45601196	(A/G)
9280	CakSNP9280	*Kabuli*	Ca_Kabuli_Chr05	45601313	(G/A)
9281	CakSNP9281	*Kabuli*	Ca_Kabuli_Chr05	45642344	(C/A)
9282	CakSNP9282	*Kabuli*	Ca_Kabuli_Chr05	45646235	(A/G)
9283	CakSNP9283	*Kabuli*	Ca_Kabuli_Chr05	45649180	(G/A)
9284	CakSNP9284	*Kabuli*	Ca_Kabuli_Chr05	45649189	(G/A)
9285	CakSNP9285	*Kabuli*	Ca_Kabuli_Chr05	45661109	(T/C)
9286	CakSNP9286	*Kabuli*	Ca_Kabuli_Chr05	45694453	(T/G)
9287	CakSNP9287	*Kabuli*	Ca_Kabuli_Chr05	45710509	(G/A)
9288	CakSNP9288	*Kabuli*	Ca_Kabuli_Chr05	45710476	(A/G)
9289	CakSNP9289	*Kabuli*	Ca_Kabuli_Chr05	45726539	(T/A)
9290	CakSNP9290	*Kabuli*	Ca_Kabuli_Chr05	45726995	(C/T)
9291	CakSNP9291	*Kabuli*	Ca_Kabuli_Chr05	45727022	(T/C)
9292	CakSNP9292	*Kabuli*	Ca_Kabuli_Chr05	45750121	(A/T)
9293	CakSNP9293	*Kabuli*	Ca_Kabuli_Chr05	45798850	(C/A)
9294	CakSNP9294	*Kabuli*	Ca_Kabuli_Chr05	45798966	(T/G)
9295	CakSNP9295	*Kabuli*	Ca_Kabuli_Chr05	45798950	(C/T)
9296	CakSNP9296	*Kabuli*	Ca_Kabuli_Chr05	45798919	(C/A)
9297	CakSNP9297	*Kabuli*	Ca_Kabuli_Chr05	45808905	(G/A)
9298	CakSNP9298	*Kabuli*	Ca_Kabuli_Chr05	45832009	(T/G)
9299	CakSNP9299	*Kabuli*	Ca_Kabuli_Chr05	45832048	(C/G)
9300	CakSNP9300	*Kabuli*	Ca_Kabuli_Chr05	45832070	(A/T)
9301	CakSNP9301	*Kabuli*	Ca_Kabuli_Chr05	45842438	(A/C)
9302	CakSNP9302	*Kabuli*	Ca_Kabuli_Chr05	45845081	(A/C)
9303	CakSNP9303	*Kabuli*	Ca_Kabuli_Chr05	45848030	(A/G)
9304	CakSNP9304	*Kabuli*	Ca_Kabuli_Chr05	45848087	(T/G)
9305	CakSNP9305	*Kabuli*	Ca_Kabuli_Chr05	45952107	(A/G)
9306	CakSNP9306	*Kabuli*	Ca_Kabuli_Chr05	45952842	(A/C)
9307	CakSNP9307	*Kabuli*	Ca_Kabuli_Chr05	45971560	(G/A)
9308	CakSNP9308	*Kabuli*	Ca_Kabuli_Chr05	45978758	(A/G)
9309	CakSNP9309	*Kabuli*	Ca_Kabuli_Chr05	46002514	(C/T)

S.N.	SNP IDs	Cultivars	Chromosomes/scaffolds	Physical positions (bp)	SNPs
9310	CakSNP9310	*Kabuli*	Ca_Kabuli_Chr05	46002497	(G/A)
9311	CakSNP9311	*Kabuli*	Ca_Kabuli_Chr05	46018939	(C/T)
9312	CakSNP9312	*Kabuli*	Ca_Kabuli_Chr05	46019052	(T/A)
9313	CakSNP9313	*Kabuli*	Ca_Kabuli_Chr05	46022566	(C/T)
9314	CakSNP9314	*Kabuli*	Ca_Kabuli_Chr05	46022598	(T/C)
9315	CakSNP9315	*Kabuli*	Ca_Kabuli_Chr05	46022551	(G/A)
9316	CakSNP9316	*Kabuli*	Ca_Kabuli_Chr05	46022549	(G/A)
9317	CakSNP9317	*Kabuli*	Ca_Kabuli_Chr05	46022547	(G/A)
9318	CakSNP9318	*Kabuli*	Ca_Kabuli_Chr05	46022544	(A/G)
9319	CakSNP9319	*Kabuli*	Ca_Kabuli_Chr05	46022531	(T/A)
9320	CakSNP9320	*Kabuli*	Ca_Kabuli_Chr05	46049587	(A/G)
9321	CakSNP9321	*Kabuli*	Ca_Kabuli_Chr05	46050168	(A/T)
9322	CakSNP9322	*Kabuli*	Ca_Kabuli_Chr05	46077190	(T/C)
9323	CakSNP9323	*Kabuli*	Ca_Kabuli_Chr05	46077302	(G/A)
9324	CakSNP9324	*Kabuli*	Ca_Kabuli_Chr05	46083283	(G/A)
9325	CakSNP9325	*Kabuli*	Ca_Kabuli_Chr05	46083343	(T/G)
9326	CakSNP9326	*Kabuli*	Ca_Kabuli_Chr05	46083414	(T/G)
9327	CakSNP9327	*Kabuli*	Ca_Kabuli_Chr05	46119122	(G/A)
9328	CakSNP9328	*Kabuli*	Ca_Kabuli_Chr05	46119182	(A/G)
9329	CakSNP9329	*Kabuli*	Ca_Kabuli_Chr05	46130289	(A/C)
9330	CakSNP9330	*Kabuli*	Ca_Kabuli_Chr05	46155262	(C/T)
9331	CakSNP9331	*Kabuli*	Ca_Kabuli_Chr05	46176366	(T/C)
9332	CakSNP9332	*Kabuli*	Ca_Kabuli_Chr05	46176443	(G/A)
9333	CakSNP9333	*Kabuli*	Ca_Kabuli_Chr05	46214229	(G/T)
9334	CakSNP9334	*Kabuli*	Ca_Kabuli_Chr05	46214233	(C/T)
9335	CakSNP9335	*Kabuli*	Ca_Kabuli_Chr05	46230010	(G/C)
9336	CakSNP9336	*Kabuli*	Ca_Kabuli_Chr05	46230011	(G/A)
9337	CakSNP9337	*Kabuli*	Ca_Kabuli_Chr05	46230109	(T/G)
9338	CakSNP9338	*Kabuli*	Ca_Kabuli_Chr05	46246419	(G/C)
9339	CakSNP9339	*Kabuli*	Ca_Kabuli_Chr05	46252617	(C/T)
9340	CakSNP9340	*Kabuli*	Ca_Kabuli_Chr05	46252643	(T/A)
9341	CakSNP9341	*Kabuli*	Ca_Kabuli_Chr05	46252605	(C/A)
9342	CakSNP9342	*Kabuli*	Ca_Kabuli_Chr05	46260822	(C/T)
9343	CakSNP9343	*Kabuli*	Ca_Kabuli_Chr05	46294000	(G/A)
9344	CakSNP9344	*Kabuli*	Ca_Kabuli_Chr05	46300789	(G/A)
9345	CakSNP9345	*Kabuli*	Ca_Kabuli_Chr05	46301639	(A/G)
9346	CakSNP9346	*Kabuli*	Ca_Kabuli_Chr05	46321992	(C/T)
9347	CakSNP9347	*Kabuli*	Ca_Kabuli_Chr05	46328589	(C/A)

S.N.	SNP IDs	Cultivars	Chromosomes/scaffolds	Physical positions (bp)	SNPs
9348	CakSNP9348	*Kabuli*	Ca_Kabuli_Chr05	46328627	(A/G)
9349	CakSNP9349	*Kabuli*	Ca_Kabuli_Chr05	46328895	(T/C)
9350	CakSNP9350	*Kabuli*	Ca_Kabuli_Chr05	46328902	(C/T)
9351	CakSNP9351	*Kabuli*	Ca_Kabuli_Chr05	46344109	(G/T)
9352	CakSNP9352	*Kabuli*	Ca_Kabuli_Chr05	46344121	(G/A)
9353	CakSNP9353	*Kabuli*	Ca_Kabuli_Chr05	46356764	(G/A)
9354	CakSNP9354	*Kabuli*	Ca_Kabuli_Chr05	46366598	(G/T)
9355	CakSNP9355	*Kabuli*	Ca_Kabuli_Chr05	46368606	(G/T)
9356	CakSNP9356	*Kabuli*	Ca_Kabuli_Chr05	46376496	(G/T)
9357	CakSNP9357	*Kabuli*	Ca_Kabuli_Chr05	46376532	(A/C)
9358	CakSNP9358	*Kabuli*	Ca_Kabuli_Chr05	46403922	(G/T)
9359	CakSNP9359	*Kabuli*	Ca_Kabuli_Chr05	46422788	(C/G)
9360	CakSNP9360	*Kabuli*	Ca_Kabuli_Chr05	46422869	(A/T)
9361	CakSNP9361	*Kabuli*	Ca_Kabuli_Chr05	46445611	(A/T)
9362	CakSNP9362	*Kabuli*	Ca_Kabuli_Chr05	46445651	(G/T)
9363	CakSNP9363	*Kabuli*	Ca_Kabuli_Chr05	46491761	(C/G)
9364	CakSNP9364	*Kabuli*	Ca_Kabuli_Chr05	46526963	(A/C)
9365	CakSNP9365	*Kabuli*	Ca_Kabuli_Chr05	46621351	(G/A)
9366	CakSNP9366	*Kabuli*	Ca_Kabuli_Chr05	46621455	(C/T)
9367	CakSNP9367	*Kabuli*	Ca_Kabuli_Chr05	46668462	(T/C)
9368	CakSNP9368	*Kabuli*	Ca_Kabuli_Chr05	46792131	(C/T)
9369	CakSNP9369	*Kabuli*	Ca_Kabuli_Chr05	46945916	(G/A)
9370	CakSNP9370	*Kabuli*	Ca_Kabuli_Chr05	46956474	(T/C)
9371	CakSNP9371	*Kabuli*	Ca_Kabuli_Chr05	47061531	(C/T)
9372	CakSNP9372	*Kabuli*	Ca_Kabuli_Chr05	47073011	(A/T)
9373	CakSNP9373	*Kabuli*	Ca_Kabuli_Chr05	47073016	(G/C)
9374	CakSNP9374	*Kabuli*	Ca_Kabuli_Chr05	47091499	(C/T)
9375	CakSNP9375	*Kabuli*	Ca_Kabuli_Chr05	47151660	(C/G)
9376	CakSNP9376	*Kabuli*	Ca_Kabuli_Chr05	47151840	(T/G)
9377	CakSNP9377	*Kabuli*	Ca_Kabuli_Chr05	47165299	(A/C)
9378	CakSNP9378	*Kabuli*	Ca_Kabuli_Chr05	47256002	(G/T)
9379	CakSNP9379	*Kabuli*	Ca_Kabuli_Chr05	47256038	(A/T)
9380	CakSNP9380	*Kabuli*	Ca_Kabuli_Chr05	47289004	(A/C)
9381	CakSNP9381	*Kabuli*	Ca_Kabuli_Chr05	47298345	(A/G)
9382	CakSNP9382	*Kabuli*	Ca_Kabuli_Chr05	47329979	(T/G)
9383	CakSNP9383	*Kabuli*	Ca_Kabuli_Chr05	47362666	(T/G)
9384	CakSNP9384	*Kabuli*	Ca_Kabuli_Chr05	47362814	(C/T)
9385	CakSNP9385	*Kabuli*	Ca_Kabuli_Chr05	47375789	(C/T)

S.N.	SNP IDs	Cultivars	Chromosomes/scaffolds	Physical positions (bp)	SNPs
9386	CakSNP9386	*Kabuli*	Ca_Kabuli_Chr05	47422536	(G/A)
9387	CakSNP9387	*Kabuli*	Ca_Kabuli_Chr05	47518533	(G/A)
9388	CakSNP9388	*Kabuli*	Ca_Kabuli_Chr05	47539345	(T/C)
9389	CakSNP9389	*Kabuli*	Ca_Kabuli_Chr05	47539505	(G/T)
9390	CakSNP9390	*Kabuli*	Ca_Kabuli_Chr05	47570939	(T/C)
9391	CakSNP9391	*Kabuli*	Ca_Kabuli_Chr05	47591327	(G/T)
9392	CakSNP9392	*Kabuli*	Ca_Kabuli_Chr05	47594471	(T/C)
9393	CakSNP9393	*Kabuli*	Ca_Kabuli_Chr05	47641224	(A/C)
9394	CakSNP9394	*Kabuli*	Ca_Kabuli_Chr05	47641472	(T/C)
9395	CakSNP9395	*Kabuli*	Ca_Kabuli_Chr05	47641628	(T/C)
9396	CakSNP9396	*Kabuli*	Ca_Kabuli_Chr05	47641567	(A/G)
9397	CakSNP9397	*Kabuli*	Ca_Kabuli_Chr05	47643992	(G/T)
9398	CakSNP9398	*Kabuli*	Ca_Kabuli_Chr05	47669124	(A/G)
9399	CakSNP9399	*Kabuli*	Ca_Kabuli_Chr05	47670649	(G/A)
9400	CakSNP9400	*Kabuli*	Ca_Kabuli_Chr05	47706780	(G/A)
9401	CakSNP9401	*Kabuli*	Ca_Kabuli_Chr05	47718830	(A/G)
9402	CakSNP9402	*Kabuli*	Ca_Kabuli_Chr05	47718839	(A/G)
9403	CakSNP9403	*Kabuli*	Ca_Kabuli_Chr05	47733645	(A/G)
9404	CakSNP9404	*Kabuli*	Ca_Kabuli_Chr05	47759169	(T/G)
9405	CakSNP9405	*Kabuli*	Ca_Kabuli_Chr05	47759745	(G/A)
9406	CakSNP9406	*Kabuli*	Ca_Kabuli_Chr05	47761348	(A/G)
9407	CakSNP9407	*Kabuli*	Ca_Kabuli_Chr05	47761322	(C/A)
9408	CakSNP9408	*Kabuli*	Ca_Kabuli_Chr05	47770909	(T/G)
9409	CakSNP9409	*Kabuli*	Ca_Kabuli_Chr05	47774516	(A/G)
9410	CakSNP9410	*Kabuli*	Ca_Kabuli_Chr05	47774559	(C/T)
9411	CakSNP9411	*Kabuli*	Ca_Kabuli_Chr05	47774718	(C/G)
9412	CakSNP9412	*Kabuli*	Ca_Kabuli_Chr05	47775175	(C/T)
9413	CakSNP9413	*Kabuli*	Ca_Kabuli_Chr05	47782605	(G/A)
9414	CakSNP9414	*Kabuli*	Ca_Kabuli_Chr05	47782606	(C/T)
9415	CakSNP9415	*Kabuli*	Ca_Kabuli_Chr05	47782697	(T/C)
9416	CakSNP9416	*Kabuli*	Ca_Kabuli_Chr05	47782843	(T/C)
9417	CakSNP9417	*Kabuli*	Ca_Kabuli_Chr05	47803636	(C/A)
9418	CakSNP9418	*Kabuli*	Ca_Kabuli_Chr05	47812041	(C/T)
9419	CakSNP9419	*Kabuli*	Ca_Kabuli_Chr05	47813712	(T/C)
9420	CakSNP9420	*Kabuli*	Ca_Kabuli_Chr05	47813767	(A/G)
9421	CakSNP9421	*Kabuli*	Ca_Kabuli_Chr05	47829261	(T/C)
9422	CakSNP9422	*Kabuli*	Ca_Kabuli_Chr05	47829331	(T/A)
9423	CakSNP9423	*Kabuli*	Ca_Kabuli_Chr05	47871094	(C/T)

S.N.	SNP IDs	Cultivars	Chromosomes/scaffolds	Physical positions (bp)	SNPs
9424	CakSNP9424	*Kabuli*	Ca_Kabuli_Chr05	47871137	(G/A)
9425	CakSNP9425	*Kabuli*	Ca_Kabuli_Chr05	47871248	(G/T)
9426	CakSNP9426	*Kabuli*	Ca_Kabuli_Chr05	47871234	(A/G)
9427	CakSNP9427	*Kabuli*	Ca_Kabuli_Chr05	47871194	(T/C)
9428	CakSNP9428	*Kabuli*	Ca_Kabuli_Chr05	47878997	(C/T)
9429	CakSNP9429	*Kabuli*	Ca_Kabuli_Chr05	47901017	(G/T)
9430	CakSNP9430	*Kabuli*	Ca_Kabuli_Chr05	47931566	(T/C)
9431	CakSNP9431	*Kabuli*	Ca_Kabuli_Chr05	47941783	(C/T)
9432	CakSNP9432	*Kabuli*	Ca_Kabuli_Chr05	47943743	(G/A)
9433	CakSNP9433	*Kabuli*	Ca_Kabuli_Chr05	47965279	(G/A)
9434	CakSNP9434	*Kabuli*	Ca_Kabuli_Chr05	47994780	(A/C)
9435	CakSNP9435	*Kabuli*	Ca_Kabuli_Chr05	47997751	(A/T)
9436	CakSNP9436	*Kabuli*	Ca_Kabuli_Chr05	48005123	(A/C)
9437	CakSNP9437	*Kabuli*	Ca_Kabuli_Chr05	48005073	(G/A)
9438	CakSNP9438	*Kabuli*	Ca_Kabuli_Chr05	48042132	(A/C)
9439	CakSNP9439	*Kabuli*	Ca_Kabuli_Chr05	48063028	(A/T)
9440	CakSNP9440	*Kabuli*	Ca_Kabuli_Chr05	48069744	(G/A)
9441	CakSNP9441	*Kabuli*	Ca_Kabuli_Chr05	48069811	(G/C)
9442	CakSNP9442	*Kabuli*	Ca_Kabuli_Chr05	48069859	(A/T)
9443	CakSNP9443	*Kabuli*	Ca_Kabuli_Chr05	48069844	(T/C)
9444	CakSNP9444	*Kabuli*	Ca_Kabuli_Chr05	48069833	(G/A)
9445	CakSNP9445	*Kabuli*	Ca_Kabuli_Chr06	1916	(T/C)
9446	CakSNP9446	*Kabuli*	Ca_Kabuli_Chr06	1847	(G/T)
9447	CakSNP9447	*Kabuli*	Ca_Kabuli_Chr06	140189	(A/G)
9448	CakSNP9448	*Kabuli*	Ca_Kabuli_Chr06	140279	(T/C)
9449	CakSNP9449	*Kabuli*	Ca_Kabuli_Chr06	140320	(G/A)
9450	CakSNP9450	*Kabuli*	Ca_Kabuli_Chr06	152513	(C/T)
9451	CakSNP9451	*Kabuli*	Ca_Kabuli_Chr06	152556	(T/C)
9452	CakSNP9452	*Kabuli*	Ca_Kabuli_Chr06	166823	(G/A)
9453	CakSNP9453	*Kabuli*	Ca_Kabuli_Chr06	166806	(C/T)
9454	CakSNP9454	*Kabuli*	Ca_Kabuli_Chr06	166959	(G/A)
9455	CakSNP9455	*Kabuli*	Ca_Kabuli_Chr06	166943	(G/A)
9456	CakSNP9456	*Kabuli*	Ca_Kabuli_Chr06	166938	(G/T)
9457	CakSNP9457	*Kabuli*	Ca_Kabuli_Chr06	166934	(G/A)
9458	CakSNP9458	*Kabuli*	Ca_Kabuli_Chr06	166932	(G/A)
9459	CakSNP9459	*Kabuli*	Ca_Kabuli_Chr06	166911	(A/G)
9460	CakSNP9460	*Kabuli*	Ca_Kabuli_Chr06	204368	(G/A)
9461	CakSNP9461	*Kabuli*	Ca_Kabuli_Chr06	204412	(G/C)

S.N.	SNP IDs	Cultivars	Chromosomes/scaffolds	Physical positions (bp)	SNPs
9462	CakSNP9462	*Kabuli*	Ca_Kabuli_Chr06	204438	(T/C)
9463	CakSNP9463	*Kabuli*	Ca_Kabuli_Chr06	267151	(A/G)
9464	CakSNP9464	*Kabuli*	Ca_Kabuli_Chr06	269269	(T/G)
9465	CakSNP9465	*Kabuli*	Ca_Kabuli_Chr06	324301	(C/T)
9466	CakSNP9466	*Kabuli*	Ca_Kabuli_Chr06	348283	(C/T)
9467	CakSNP9467	*Kabuli*	Ca_Kabuli_Chr06	348295	(G/C)
9468	CakSNP9468	*Kabuli*	Ca_Kabuli_Chr06	375684	(T/G)
9469	CakSNP9469	*Kabuli*	Ca_Kabuli_Chr06	386250	(A/G)
9470	CakSNP9470	*Kabuli*	Ca_Kabuli_Chr06	474489	(C/A)
9471	CakSNP9471	*Kabuli*	Ca_Kabuli_Chr06	505875	(T/C)
9472	CakSNP9472	*Kabuli*	Ca_Kabuli_Chr06	515221	(T/G)
9473	CakSNP9473	*Kabuli*	Ca_Kabuli_Chr06	548416	(A/T)
9474	CakSNP9474	*Kabuli*	Ca_Kabuli_Chr06	551130	(T/G)
9475	CakSNP9475	*Kabuli*	Ca_Kabuli_Chr06	561562	(A/G)
9476	CakSNP9476	*Kabuli*	Ca_Kabuli_Chr06	580616	(G/C)
9477	CakSNP9477	*Kabuli*	Ca_Kabuli_Chr06	588592	(C/A)
9478	CakSNP9478	*Kabuli*	Ca_Kabuli_Chr06	638983	(T/A)
9479	CakSNP9479	*Kabuli*	Ca_Kabuli_Chr06	645755	(G/T)
9480	CakSNP9480	*Kabuli*	Ca_Kabuli_Chr06	645746	(G/T)
9481	CakSNP9481	*Kabuli*	Ca_Kabuli_Chr06	647322	(A/C)
9482	CakSNP9482	*Kabuli*	Ca_Kabuli_Chr06	697187	(A/G)
9483	CakSNP9483	*Kabuli*	Ca_Kabuli_Chr06	697169	(A/G)
9484	CakSNP9484	*Kabuli*	Ca_Kabuli_Chr06	697168	(C/T)
9485	CakSNP9485	*Kabuli*	Ca_Kabuli_Chr06	697162	(A/G)
9486	CakSNP9486	*Kabuli*	Ca_Kabuli_Chr06	697158	(G/A)
9487	CakSNP9487	*Kabuli*	Ca_Kabuli_Chr06	697148	(C/T)
9488	CakSNP9488	*Kabuli*	Ca_Kabuli_Chr06	697147	(A/G)
9489	CakSNP9489	*Kabuli*	Ca_Kabuli_Chr06	697142	(G/C)
9490	CakSNP9490	*Kabuli*	Ca_Kabuli_Chr06	894921	(A/G)
9491	CakSNP9491	*Kabuli*	Ca_Kabuli_Chr06	902403	(G/A)
9492	CakSNP9492	*Kabuli*	Ca_Kabuli_Chr06	902402	(T/C)
9493	CakSNP9493	*Kabuli*	Ca_Kabuli_Chr06	949263	(C/A)
9494	CakSNP9494	*Kabuli*	Ca_Kabuli_Chr06	961652	(C/T)
9495	CakSNP9495	*Kabuli*	Ca_Kabuli_Chr06	961805	(C/T)
9496	CakSNP9496	*Kabuli*	Ca_Kabuli_Chr06	961733	(T/A)
9497	CakSNP9497	*Kabuli*	Ca_Kabuli_Chr06	997997	(T/A)
9498	CakSNP9498	*Kabuli*	Ca_Kabuli_Chr06	1130293	(C/T)
9499	CakSNP9499	*Kabuli*	Ca_Kabuli_Chr06	1212889	(G/A)

S.N.	SNP IDs	Cultivars	Chromosomes/scaffolds	Physical positions (bp)	SNPs
9500	CakSNP9500	*Kabuli*	Ca_Kabuli_Chr06	1220425	(C/T)
9501	CakSNP9501	*Kabuli*	Ca_Kabuli_Chr06	1220460	(G/C)
9502	CakSNP9502	*Kabuli*	Ca_Kabuli_Chr06	1220465	(G/A)
9503	CakSNP9503	*Kabuli*	Ca_Kabuli_Chr06	1220510	(A/T)
9504	CakSNP9504	*Kabuli*	Ca_Kabuli_Chr06	1220499	(C/G)
9505	CakSNP9505	*Kabuli*	Ca_Kabuli_Chr06	1287168	(C/T)
9506	CakSNP9506	*Kabuli*	Ca_Kabuli_Chr06	1290413	(C/G)
9507	CakSNP9507	*Kabuli*	Ca_Kabuli_Chr06	1290538	(T/G)
9508	CakSNP9508	*Kabuli*	Ca_Kabuli_Chr06	1295748	(T/C)
9509	CakSNP9509	*Kabuli*	Ca_Kabuli_Chr06	1312881	(T/C)
9510	CakSNP9510	*Kabuli*	Ca_Kabuli_Chr06	1312932	(G/A)
9511	CakSNP9511	*Kabuli*	Ca_Kabuli_Chr06	1312982	(C/A)
9512	CakSNP9512	*Kabuli*	Ca_Kabuli_Chr06	1315583	(G/C)
9513	CakSNP9513	*Kabuli*	Ca_Kabuli_Chr06	1626723	(T/C)
9514	CakSNP9514	*Kabuli*	Ca_Kabuli_Chr06	1626712	(C/T)
9515	CakSNP9515	*Kabuli*	Ca_Kabuli_Chr06	1817599	(C/T)
9516	CakSNP9516	*Kabuli*	Ca_Kabuli_Chr06	1817602	(A/T)
9517	CakSNP9517	*Kabuli*	Ca_Kabuli_Chr06	1817607	(T/C)
9518	CakSNP9518	*Kabuli*	Ca_Kabuli_Chr06	1817628	(A/T)
9519	CakSNP9519	*Kabuli*	Ca_Kabuli_Chr06	1932114	(C/G)
9520	CakSNP9520	*Kabuli*	Ca_Kabuli_Chr06	1932175	(G/A)
9521	CakSNP9521	*Kabuli*	Ca_Kabuli_Chr06	1966137	(T/C)
9522	CakSNP9522	*Kabuli*	Ca_Kabuli_Chr06	1978781	(C/A)
9523	CakSNP9523	*Kabuli*	Ca_Kabuli_Chr06	1978864	(A/C)
9524	CakSNP9524	*Kabuli*	Ca_Kabuli_Chr06	1978867	(T/A)
9525	CakSNP9525	*Kabuli*	Ca_Kabuli_Chr06	2037575	(G/T)
9526	CakSNP9526	*Kabuli*	Ca_Kabuli_Chr06	2037599	(A/G)
9527	CakSNP9527	*Kabuli*	Ca_Kabuli_Chr06	2037620	(T/G)
9528	CakSNP9528	*Kabuli*	Ca_Kabuli_Chr06	2037623	(T/G)
9529	CakSNP9529	*Kabuli*	Ca_Kabuli_Chr06	2037626	(C/T)
9530	CakSNP9530	*Kabuli*	Ca_Kabuli_Chr06	2050907	(A/G)
9531	CakSNP9531	*Kabuli*	Ca_Kabuli_Chr06	2061459	(A/G)
9532	CakSNP9532	*Kabuli*	Ca_Kabuli_Chr06	2078416	(T/A)
9533	CakSNP9533	*Kabuli*	Ca_Kabuli_Chr06	2079137	(T/G)
9534	CakSNP9534	*Kabuli*	Ca_Kabuli_Chr06	2079186	(G/T)
9535	CakSNP9535	*Kabuli*	Ca_Kabuli_Chr06	2083784	(A/T)
9536	CakSNP9536	*Kabuli*	Ca_Kabuli_Chr06	2083805	(G/A)
9537	CakSNP9537	*Kabuli*	Ca_Kabuli_Chr06	2083813	(T/C)

S.N.	SNP IDs	Cultivars	Chromosomes/scaffolds	Physical positions (bp)	SNPs
9538	CakSNP9538	*Kabuli*	Ca_Kabuli_Chr06	2126667	(C/T)
9539	CakSNP9539	*Kabuli*	Ca_Kabuli_Chr06	2126655	(G/A)
9540	CakSNP9540	*Kabuli*	Ca_Kabuli_Chr06	2140189	(G/T)
9541	CakSNP9541	*Kabuli*	Ca_Kabuli_Chr06	2140406	(G/A)
9542	CakSNP9542	*Kabuli*	Ca_Kabuli_Chr06	2193748	(T/A)
9543	CakSNP9543	*Kabuli*	Ca_Kabuli_Chr06	2193763	(A/C)
9544	CakSNP9544	*Kabuli*	Ca_Kabuli_Chr06	2193803	(G/A)
9545	CakSNP9545	*Kabuli*	Ca_Kabuli_Chr06	2214131	(T/A)
9546	CakSNP9546	*Kabuli*	Ca_Kabuli_Chr06	2214178	(T/C)
9547	CakSNP9547	*Kabuli*	Ca_Kabuli_Chr06	2214247	(A/G)
9548	CakSNP9548	*Kabuli*	Ca_Kabuli_Chr06	2223544	(A/T)
9549	CakSNP9549	*Kabuli*	Ca_Kabuli_Chr06	2223578	(T/G)
9550	CakSNP9550	*Kabuli*	Ca_Kabuli_Chr06	2223664	(A/C)
9551	CakSNP9551	*Kabuli*	Ca_Kabuli_Chr06	2244901	(A/C)
9552	CakSNP9552	*Kabuli*	Ca_Kabuli_Chr06	2275505	(A/G)
9553	CakSNP9553	*Kabuli*	Ca_Kabuli_Chr06	2275524	(G/A)
9554	CakSNP9554	*Kabuli*	Ca_Kabuli_Chr06	2275553	(A/G)
9555	CakSNP9555	*Kabuli*	Ca_Kabuli_Chr06	2280734	(T/A)
9556	CakSNP9556	*Kabuli*	Ca_Kabuli_Chr06	2280752	(A/G)
9557	CakSNP9557	*Kabuli*	Ca_Kabuli_Chr06	2286205	(A/G)
9558	CakSNP9558	*Kabuli*	Ca_Kabuli_Chr06	2286237	(C/T)
9559	CakSNP9559	*Kabuli*	Ca_Kabuli_Chr06	2294911	(T/G)
9560	CakSNP9560	*Kabuli*	Ca_Kabuli_Chr06	2303123	(T/G)
9561	CakSNP9561	*Kabuli*	Ca_Kabuli_Chr06	2313683	(A/C)
9562	CakSNP9562	*Kabuli*	Ca_Kabuli_Chr06	2313828	(G/A)
9563	CakSNP9563	*Kabuli*	Ca_Kabuli_Chr06	2314029	(T/C)
9564	CakSNP9564	*Kabuli*	Ca_Kabuli_Chr06	2327842	(A/G)
9565	CakSNP9565	*Kabuli*	Ca_Kabuli_Chr06	2462481	(C/T)
9566	CakSNP9566	*Kabuli*	Ca_Kabuli_Chr06	2502485	(A/G)
9567	CakSNP9567	*Kabuli*	Ca_Kabuli_Chr06	2527768	(A/G)
9568	CakSNP9568	*Kabuli*	Ca_Kabuli_Chr06	2542942	(A/T)
9569	CakSNP9569	*Kabuli*	Ca_Kabuli_Chr06	2542981	(T/C)
9570	CakSNP9570	*Kabuli*	Ca_Kabuli_Chr06	2543096	(C/T)
9571	CakSNP9571	*Kabuli*	Ca_Kabuli_Chr06	2543062	(A/G)
9572	CakSNP9572	*Kabuli*	Ca_Kabuli_Chr06	2548412	(A/G)
9573	CakSNP9573	*Kabuli*	Ca_Kabuli_Chr06	2549997	(C/T)
9574	CakSNP9574	*Kabuli*	Ca_Kabuli_Chr06	2549991	(T/C)
9575	CakSNP9575	*Kabuli*	Ca_Kabuli_Chr06	2550154	(A/C)

S.N.	SNP IDs	Cultivars	Chromosomes/scaffolds	Physical positions (bp)	SNPs
9576	CakSNP9576	*Kabuli*	Ca_Kabuli_Chr06	2623370	(T/C)
9577	CakSNP9577	*Kabuli*	Ca_Kabuli_Chr06	2625793	(C/G)
9578	CakSNP9578	*Kabuli*	Ca_Kabuli_Chr06	2633941	(A/T)
9579	CakSNP9579	*Kabuli*	Ca_Kabuli_Chr06	2639558	(T/A)
9580	CakSNP9580	*Kabuli*	Ca_Kabuli_Chr06	2639606	(A/G)
9581	CakSNP9581	*Kabuli*	Ca_Kabuli_Chr06	2639607	(T/C)
9582	CakSNP9582	*Kabuli*	Ca_Kabuli_Chr06	2676594	(C/T)
9583	CakSNP9583	*Kabuli*	Ca_Kabuli_Chr06	2714894	(T/C)
9584	CakSNP9584	*Kabuli*	Ca_Kabuli_Chr06	2714897	(C/A)
9585	CakSNP9585	*Kabuli*	Ca_Kabuli_Chr06	2714918	(T/G)
9586	CakSNP9586	*Kabuli*	Ca_Kabuli_Chr06	2717150	(G/A)
9587	CakSNP9587	*Kabuli*	Ca_Kabuli_Chr06	2740630	(A/G)
9588	CakSNP9588	*Kabuli*	Ca_Kabuli_Chr06	2740765	(A/G)
9589	CakSNP9589	*Kabuli*	Ca_Kabuli_Chr06	2740784	(T/C)
9590	CakSNP9590	*Kabuli*	Ca_Kabuli_Chr06	2741185	(G/A)
9591	CakSNP9591	*Kabuli*	Ca_Kabuli_Chr06	2777609	(A/G)
9592	CakSNP9592	*Kabuli*	Ca_Kabuli_Chr06	2802942	(C/A)
9593	CakSNP9593	*Kabuli*	Ca_Kabuli_Chr06	2811345	(A/G)
9594	CakSNP9594	*Kabuli*	Ca_Kabuli_Chr06	2812794	(A/C)
9595	CakSNP9595	*Kabuli*	Ca_Kabuli_Chr06	2812719	(G/A)
9596	CakSNP9596	*Kabuli*	Ca_Kabuli_Chr06	2813244	(G/A)
9597	CakSNP9597	*Kabuli*	Ca_Kabuli_Chr06	2821836	(G/T)
9598	CakSNP9598	*Kabuli*	Ca_Kabuli_Chr06	2846051	(C/T)
9599	CakSNP9599	*Kabuli*	Ca_Kabuli_Chr06	2846018	(T/C)
9600	CakSNP9600	*Kabuli*	Ca_Kabuli_Chr06	2855691	(C/G)
9601	CakSNP9601	*Kabuli*	Ca_Kabuli_Chr06	2864713	(G/A)
9602	CakSNP9602	*Kabuli*	Ca_Kabuli_Chr06	2919355	(A/C)
9603	CakSNP9603	*Kabuli*	Ca_Kabuli_Chr06	2925908	(A/G)
9604	CakSNP9604	*Kabuli*	Ca_Kabuli_Chr06	2925962	(G/C)
9605	CakSNP9605	*Kabuli*	Ca_Kabuli_Chr06	2926006	(T/C)
9606	CakSNP9606	*Kabuli*	Ca_Kabuli_Chr06	2927224	(G/A)
9607	CakSNP9607	*Kabuli*	Ca_Kabuli_Chr06	2927255	(C/A)
9608	CakSNP9608	*Kabuli*	Ca_Kabuli_Chr06	2927271	(G/A)
9609	CakSNP9609	*Kabuli*	Ca_Kabuli_Chr06	2927386	(C/G)
9610	CakSNP9610	*Kabuli*	Ca_Kabuli_Chr06	2927387	(T/C)
9611	CakSNP9611	*Kabuli*	Ca_Kabuli_Chr06	2929355	(C/T)
9612	CakSNP9612	*Kabuli*	Ca_Kabuli_Chr06	2930453	(T/C)
9613	CakSNP9613	*Kabuli*	Ca_Kabuli_Chr06	2930459	(C/T)

S.N.	SNP IDs	Cultivars	Chromosomes/scaffolds	Physical positions (bp)	SNPs
9614	CakSNP9614	*Kabuli*	Ca_Kabuli_Chr06	2930504	(T/C)
9615	CakSNP9615	*Kabuli*	Ca_Kabuli_Chr06	2932029	(T/C)
9616	CakSNP9616	*Kabuli*	Ca_Kabuli_Chr06	2932179	(T/G)
9617	CakSNP9617	*Kabuli*	Ca_Kabuli_Chr06	2932168	(A/C)
9618	CakSNP9618	*Kabuli*	Ca_Kabuli_Chr06	2932143	(C/A)
9619	CakSNP9619	*Kabuli*	Ca_Kabuli_Chr06	2932130	(T/G)
9620	CakSNP9620	*Kabuli*	Ca_Kabuli_Chr06	2932777	(A/G)
9621	CakSNP9621	*Kabuli*	Ca_Kabuli_Chr06	2972319	(C/T)
9622	CakSNP9622	*Kabuli*	Ca_Kabuli_Chr06	2972301	(T/C)
9623	CakSNP9623	*Kabuli*	Ca_Kabuli_Chr06	2973741	(A/G)
9624	CakSNP9624	*Kabuli*	Ca_Kabuli_Chr06	2973756	(A/C)
9625	CakSNP9625	*Kabuli*	Ca_Kabuli_Chr06	3060508	(G/A)
9626	CakSNP9626	*Kabuli*	Ca_Kabuli_Chr06	3060502	(G/A)
9627	CakSNP9627	*Kabuli*	Ca_Kabuli_Chr06	3288481	(C/A)
9628	CakSNP9628	*Kabuli*	Ca_Kabuli_Chr06	3288444	(G/A)
9629	CakSNP9629	*Kabuli*	Ca_Kabuli_Chr06	3288441	(A/G)
9630	CakSNP9630	*Kabuli*	Ca_Kabuli_Chr06	3288505	(G/T)
9631	CakSNP9631	*Kabuli*	Ca_Kabuli_Chr06	3297086	(G/A)
9632	CakSNP9632	*Kabuli*	Ca_Kabuli_Chr06	3298952	(A/T)
9633	CakSNP9633	*Kabuli*	Ca_Kabuli_Chr06	3395024	(A/G)
9634	CakSNP9634	*Kabuli*	Ca_Kabuli_Chr06	3395253	(G/A)
9635	CakSNP9635	*Kabuli*	Ca_Kabuli_Chr06	3395370	(C/T)
9636	CakSNP9636	*Kabuli*	Ca_Kabuli_Chr06	3397650	(A/G)
9637	CakSNP9637	*Kabuli*	Ca_Kabuli_Chr06	3401260	(A/G)
9638	CakSNP9638	*Kabuli*	Ca_Kabuli_Chr06	3401272	(G/A)
9639	CakSNP9639	*Kabuli*	Ca_Kabuli_Chr06	3401277	(C/T)
9640	CakSNP9640	*Kabuli*	Ca_Kabuli_Chr06	3482411	(A/G)
9641	CakSNP9641	*Kabuli*	Ca_Kabuli_Chr06	3505662	(C/A)
9642	CakSNP9642	*Kabuli*	Ca_Kabuli_Chr06	3530094	(T/C)
9643	CakSNP9643	*Kabuli*	Ca_Kabuli_Chr06	3530137	(A/G)
9644	CakSNP9644	*Kabuli*	Ca_Kabuli_Chr06	3548121	(C/A)
9645	CakSNP9645	*Kabuli*	Ca_Kabuli_Chr06	3548114	(A/C)
9646	CakSNP9646	*Kabuli*	Ca_Kabuli_Chr06	3786843	(A/G)
9647	CakSNP9647	*Kabuli*	Ca_Kabuli_Chr06	3787239	(C/A)
9648	CakSNP9648	*Kabuli*	Ca_Kabuli_Chr06	3829070	(C/A)
9649	CakSNP9649	*Kabuli*	Ca_Kabuli_Chr06	3962516	(A/C)
9650	CakSNP9650	*Kabuli*	Ca_Kabuli_Chr06	3985519	(G/A)
9651	CakSNP9651	*Kabuli*	Ca_Kabuli_Chr06	3992419	(C/T)

S.N.	SNP IDs	Cultivars	Chromosomes/scaffolds	Physical positions (bp)	SNPs
9652	CakSNP9652	*Kabuli*	Ca_Kabuli_Chr06	4112085	(T/C)
9653	CakSNP9653	*Kabuli*	Ca_Kabuli_Chr06	4226957	(G/A)
9654	CakSNP9654	*Kabuli*	Ca_Kabuli_Chr06	4226947	(A/T)
9655	CakSNP9655	*Kabuli*	Ca_Kabuli_Chr06	4226924	(G/T)
9656	CakSNP9656	*Kabuli*	Ca_Kabuli_Chr06	4226951	(C/A)
9657	CakSNP9657	*Kabuli*	Ca_Kabuli_Chr06	4228728	(T/C)
9658	CakSNP9658	*Kabuli*	Ca_Kabuli_Chr06	4228759	(C/G)
9659	CakSNP9659	*Kabuli*	Ca_Kabuli_Chr06	4228884	(T/C)
9660	CakSNP9660	*Kabuli*	Ca_Kabuli_Chr06	4344604	(T/C)
9661	CakSNP9661	*Kabuli*	Ca_Kabuli_Chr06	4350822	(A/T)
9662	CakSNP9662	*Kabuli*	Ca_Kabuli_Chr06	4350867	(A/C)
9663	CakSNP9663	*Kabuli*	Ca_Kabuli_Chr06	4350889	(A/C)
9664	CakSNP9664	*Kabuli*	Ca_Kabuli_Chr06	4440034	(T/G)
9665	CakSNP9665	*Kabuli*	Ca_Kabuli_Chr06	4562263	(C/A)
9666	CakSNP9666	*Kabuli*	Ca_Kabuli_Chr06	4562264	(C/G)
9667	CakSNP9667	*Kabuli*	Ca_Kabuli_Chr06	4601303	(A/C)
9668	CakSNP9668	*Kabuli*	Ca_Kabuli_Chr06	4678523	(A/C)
9669	CakSNP9669	*Kabuli*	Ca_Kabuli_Chr06	4681686	(C/A)
9670	CakSNP9670	*Kabuli*	Ca_Kabuli_Chr06	4743563	(C/G)
9671	CakSNP9671	*Kabuli*	Ca_Kabuli_Chr06	4743608	(C/T)
9672	CakSNP9672	*Kabuli*	Ca_Kabuli_Chr06	4807908	(G/C)
9673	CakSNP9673	*Kabuli*	Ca_Kabuli_Chr06	4858109	(T/C)
9674	CakSNP9674	*Kabuli*	Ca_Kabuli_Chr06	4861184	(T/G)
9675	CakSNP9675	*Kabuli*	Ca_Kabuli_Chr06	4981078	(G/T)
9676	CakSNP9676	*Kabuli*	Ca_Kabuli_Chr06	5185380	(A/G)
9677	CakSNP9677	*Kabuli*	Ca_Kabuli_Chr06	5309445	(G/A)
9678	CakSNP9678	*Kabuli*	Ca_Kabuli_Chr06	5338761	(T/G)
9679	CakSNP9679	*Kabuli*	Ca_Kabuli_Chr06	5341510	(C/A)
9680	CakSNP9680	*Kabuli*	Ca_Kabuli_Chr06	5366094	(A/G)
9681	CakSNP9681	*Kabuli*	Ca_Kabuli_Chr06	5368455	(T/G)
9682	CakSNP9682	*Kabuli*	Ca_Kabuli_Chr06	5392161	(G/A)
9683	CakSNP9683	*Kabuli*	Ca_Kabuli_Chr06	5392185	(T/C)
9684	CakSNP9684	*Kabuli*	Ca_Kabuli_Chr06	5396888	(T/G)
9685	CakSNP9685	*Kabuli*	Ca_Kabuli_Chr06	5396890	(T/G)
9686	CakSNP9686	*Kabuli*	Ca_Kabuli_Chr06	5396897	(T/G)
9687	CakSNP9687	*Kabuli*	Ca_Kabuli_Chr06	5396913	(C/T)
9688	CakSNP9688	*Kabuli*	Ca_Kabuli_Chr06	5396916	(C/T)
9689	CakSNP9689	*Kabuli*	Ca_Kabuli_Chr06	5401307	(T/C)

S.N.	SNP IDs	Cultivars	Chromosomes/scaffolds	Physical positions (bp)	SNPs
9690	CakSNP9690	*Kabuli*	Ca_Kabuli_Chr06	5453512	(A/G)
9691	CakSNP9691	*Kabuli*	Ca_Kabuli_Chr06	5498898	(C/T)
9692	CakSNP9692	*Kabuli*	Ca_Kabuli_Chr06	5500791	(C/T)
9693	CakSNP9693	*Kabuli*	Ca_Kabuli_Chr06	5501363	(T/C)
9694	CakSNP9694	*Kabuli*	Ca_Kabuli_Chr06	5541264	(A/G)
9695	CakSNP9695	*Kabuli*	Ca_Kabuli_Chr06	5567967	(T/C)
9696	CakSNP9696	*Kabuli*	Ca_Kabuli_Chr06	5589373	(T/C)
9697	CakSNP9697	*Kabuli*	Ca_Kabuli_Chr06	5616372	(C/T)
9698	CakSNP9698	*Kabuli*	Ca_Kabuli_Chr06	5616482	(A/G)
9699	CakSNP9699	*Kabuli*	Ca_Kabuli_Chr06	5616454	(T/A)
9700	CakSNP9700	*Kabuli*	Ca_Kabuli_Chr06	5628948	(C/A)
9701	CakSNP9701	*Kabuli*	Ca_Kabuli_Chr06	5628956	(T/G)
9702	CakSNP9702	*Kabuli*	Ca_Kabuli_Chr06	5692120	(C/A)
9703	CakSNP9703	*Kabuli*	Ca_Kabuli_Chr06	5694397	(G/T)
9704	CakSNP9704	*Kabuli*	Ca_Kabuli_Chr06	5701004	(A/G)
9705	CakSNP9705	*Kabuli*	Ca_Kabuli_Chr06	5704845	(T/C)
9706	CakSNP9706	*Kabuli*	Ca_Kabuli_Chr06	5704809	(T/A)
9707	CakSNP9707	*Kabuli*	Ca_Kabuli_Chr06	5706649	(C/T)
9708	CakSNP9708	*Kabuli*	Ca_Kabuli_Chr06	5841443	(G/C)
9709	CakSNP9709	*Kabuli*	Ca_Kabuli_Chr06	5871537	(G/A)
9710	CakSNP9710	*Kabuli*	Ca_Kabuli_Chr06	5886393	(A/G)
9711	CakSNP9711	*Kabuli*	Ca_Kabuli_Chr06	5888423	(A/G)
9712	CakSNP9712	*Kabuli*	Ca_Kabuli_Chr06	5924967	(G/T)
9713	CakSNP9713	*Kabuli*	Ca_Kabuli_Chr06	5925028	(G/T)
9714	CakSNP9714	*Kabuli*	Ca_Kabuli_Chr06	6110805	(T/C)
9715	CakSNP9715	*Kabuli*	Ca_Kabuli_Chr06	6119644	(C/A)
9716	CakSNP9716	*Kabuli*	Ca_Kabuli_Chr06	6131564	(G/A)
9717	CakSNP9717	*Kabuli*	Ca_Kabuli_Chr06	6131518	(G/T)
9718	CakSNP9718	*Kabuli*	Ca_Kabuli_Chr06	6131514	(G/T)
9719	CakSNP9719	*Kabuli*	Ca_Kabuli_Chr06	6169291	(A/G)
9720	CakSNP9720	*Kabuli*	Ca_Kabuli_Chr06	6192895	(A/T)
9721	CakSNP9721	*Kabuli*	Ca_Kabuli_Chr06	6193105	(G/C)
9722	CakSNP9722	*Kabuli*	Ca_Kabuli_Chr06	6216766	(T/G)
9723	CakSNP9723	*Kabuli*	Ca_Kabuli_Chr06	6216746	(G/C)
9724	CakSNP9724	*Kabuli*	Ca_Kabuli_Chr06	6227887	(A/G)
9725	CakSNP9725	*Kabuli*	Ca_Kabuli_Chr06	6248260	(T/C)
9726	CakSNP9726	*Kabuli*	Ca_Kabuli_Chr06	6248203	(A/G)
9727	CakSNP9727	*Kabuli*	Ca_Kabuli_Chr06	6415818	(A/G)

S.N.	SNP IDs	Cultivars	Chromosomes/scaffolds	Physical positions (bp)	SNPs
9728	CakSNP9728	*Kabuli*	Ca_Kabuli_Chr06	6443828	(C/G)
9729	CakSNP9729	*Kabuli*	Ca_Kabuli_Chr06	6444169	(A/G)
9730	CakSNP9730	*Kabuli*	Ca_Kabuli_Chr06	6444226	(T/G)
9731	CakSNP9731	*Kabuli*	Ca_Kabuli_Chr06	6444243	(G/A)
9732	CakSNP9732	*Kabuli*	Ca_Kabuli_Chr06	6444220	(G/A)
9733	CakSNP9733	*Kabuli*	Ca_Kabuli_Chr06	6517785	(T/C)
9734	CakSNP9734	*Kabuli*	Ca_Kabuli_Chr06	6517849	(C/T)
9735	CakSNP9735	*Kabuli*	Ca_Kabuli_Chr06	6538236	(C/T)
9736	CakSNP9736	*Kabuli*	Ca_Kabuli_Chr06	6604713	(G/A)
9737	CakSNP9737	*Kabuli*	Ca_Kabuli_Chr06	6604766	(C/A)
9738	CakSNP9738	*Kabuli*	Ca_Kabuli_Chr06	6607773	(A/G)
9739	CakSNP9739	*Kabuli*	Ca_Kabuli_Chr06	6631216	(C/T)
9740	CakSNP9740	*Kabuli*	Ca_Kabuli_Chr06	6654917	(T/C)
9741	CakSNP9741	*Kabuli*	Ca_Kabuli_Chr06	6764292	(G/A)
9742	CakSNP9742	*Kabuli*	Ca_Kabuli_Chr06	6779147	(G/C)
9743	CakSNP9743	*Kabuli*	Ca_Kabuli_Chr06	6779982	(C/T)
9744	CakSNP9744	*Kabuli*	Ca_Kabuli_Chr06	6792012	(C/T)
9745	CakSNP9745	*Kabuli*	Ca_Kabuli_Chr06	6800935	(A/T)
9746	CakSNP9746	*Kabuli*	Ca_Kabuli_Chr06	6800927	(G/T)
9747	CakSNP9747	*Kabuli*	Ca_Kabuli_Chr06	6800877	(T/A)
9748	CakSNP9748	*Kabuli*	Ca_Kabuli_Chr06	6802259	(C/T)
9749	CakSNP9749	*Kabuli*	Ca_Kabuli_Chr06	6802651	(A/G)
9750	CakSNP9750	*Kabuli*	Ca_Kabuli_Chr06	6803433	(A/G)
9751	CakSNP9751	*Kabuli*	Ca_Kabuli_Chr06	6815594	(G/A)
9752	CakSNP9752	*Kabuli*	Ca_Kabuli_Chr06	6840803	(T/C)
9753	CakSNP9753	*Kabuli*	Ca_Kabuli_Chr06	6853983	(G/A)
9754	CakSNP9754	*Kabuli*	Ca_Kabuli_Chr06	6871801	(A/G)
9755	CakSNP9755	*Kabuli*	Ca_Kabuli_Chr06	6877020	(C/G)
9756	CakSNP9756	*Kabuli*	Ca_Kabuli_Chr06	6877088	(C/A)
9757	CakSNP9757	*Kabuli*	Ca_Kabuli_Chr06	6877163	(T/C)
9758	CakSNP9758	*Kabuli*	Ca_Kabuli_Chr06	6877246	(C/A)
9759	CakSNP9759	*Kabuli*	Ca_Kabuli_Chr06	6877284	(C/T)
9760	CakSNP9760	*Kabuli*	Ca_Kabuli_Chr06	6899977	(G/A)
9761	CakSNP9761	*Kabuli*	Ca_Kabuli_Chr06	6899949	(T/G)
9762	CakSNP9762	*Kabuli*	Ca_Kabuli_Chr06	6913639	(T/C)
9763	CakSNP9763	*Kabuli*	Ca_Kabuli_Chr06	6913622	(A/C)
9764	CakSNP9764	*Kabuli*	Ca_Kabuli_Chr06	6913695	(G/A)
9765	CakSNP9765	*Kabuli*	Ca_Kabuli_Chr06	6913825	(G/A)

S.N.	SNP IDs	Cultivars	Chromosomes/scaffolds	Physical positions (bp)	SNPs
9766	CakSNP9766	*Kabuli*	Ca_Kabuli_Chr06	6913821	(A/T)
9767	CakSNP9767	*Kabuli*	Ca_Kabuli_Chr06	6954468	(A/G)
9768	CakSNP9768	*Kabuli*	Ca_Kabuli_Chr06	6954480	(A/G)
9769	CakSNP9769	*Kabuli*	Ca_Kabuli_Chr06	7026495	(G/A)
9770	CakSNP9770	*Kabuli*	Ca_Kabuli_Chr06	7026481	(G/C)
9771	CakSNP9771	*Kabuli*	Ca_Kabuli_Chr06	7039578	(C/T)
9772	CakSNP9772	*Kabuli*	Ca_Kabuli_Chr06	7040231	(A/C)
9773	CakSNP9773	*Kabuli*	Ca_Kabuli_Chr06	7138381	(G/A)
9774	CakSNP9774	*Kabuli*	Ca_Kabuli_Chr06	7259533	(T/A)
9775	CakSNP9775	*Kabuli*	Ca_Kabuli_Chr06	7271634	(C/T)
9776	CakSNP9776	*Kabuli*	Ca_Kabuli_Chr06	7332579	(T/C)
9777	CakSNP9777	*Kabuli*	Ca_Kabuli_Chr06	7332585	(T/C)
9778	CakSNP9778	*Kabuli*	Ca_Kabuli_Chr06	7332762	(C/T)
9779	CakSNP9779	*Kabuli*	Ca_Kabuli_Chr06	7349517	(A/C)
9780	CakSNP9780	*Kabuli*	Ca_Kabuli_Chr06	7377229	(G/A)
9781	CakSNP9781	*Kabuli*	Ca_Kabuli_Chr06	7377276	(G/A)
9782	CakSNP9782	*Kabuli*	Ca_Kabuli_Chr06	7406219	(A/G)
9783	CakSNP9783	*Kabuli*	Ca_Kabuli_Chr06	7406233	(G/A)
9784	CakSNP9784	*Kabuli*	Ca_Kabuli_Chr06	7406301	(T/C)
9785	CakSNP9785	*Kabuli*	Ca_Kabuli_Chr06	7531353	(G/A)
9786	CakSNP9786	*Kabuli*	Ca_Kabuli_Chr06	7531445	(G/A)
9787	CakSNP9787	*Kabuli*	Ca_Kabuli_Chr06	7531456	(T/A)
9788	CakSNP9788	*Kabuli*	Ca_Kabuli_Chr06	7534360	(G/T)
9789	CakSNP9789	*Kabuli*	Ca_Kabuli_Chr06	7543580	(C/T)
9790	CakSNP9790	*Kabuli*	Ca_Kabuli_Chr06	7554094	(T/A)
9791	CakSNP9791	*Kabuli*	Ca_Kabuli_Chr06	7571950	(T/C)
9792	CakSNP9792	*Kabuli*	Ca_Kabuli_Chr06	7582726	(G/T)
9793	CakSNP9793	*Kabuli*	Ca_Kabuli_Chr06	7600817	(G/A)
9794	CakSNP9794	*Kabuli*	Ca_Kabuli_Chr06	7603195	(G/A)
9795	CakSNP9795	*Kabuli*	Ca_Kabuli_Chr06	7603178	(A/C)
9796	CakSNP9796	*Kabuli*	Ca_Kabuli_Chr06	7609814	(C/T)
9797	CakSNP9797	*Kabuli*	Ca_Kabuli_Chr06	7647907	(T/G)
9798	CakSNP9798	*Kabuli*	Ca_Kabuli_Chr06	7647931	(G/A)
9799	CakSNP9799	*Kabuli*	Ca_Kabuli_Chr06	7661930	(T/G)
9800	CakSNP9800	*Kabuli*	Ca_Kabuli_Chr06	7661924	(T/C)
9801	CakSNP9801	*Kabuli*	Ca_Kabuli_Chr06	7667301	(T/C)
9802	CakSNP9802	*Kabuli*	Ca_Kabuli_Chr06	7667328	(A/T)
9803	CakSNP9803	*Kabuli*	Ca_Kabuli_Chr06	7673403	(C/T)

S.N.	SNP IDs	Cultivars	Chromosomes/scaffolds	Physical positions (bp)	SNPs
9804	CakSNP9804	*Kabuli*	Ca_Kabuli_Chr06	7694609	(A/T)
9805	CakSNP9805	*Kabuli*	Ca_Kabuli_Chr06	7694625	(G/T)
9806	CakSNP9806	*Kabuli*	Ca_Kabuli_Chr06	7694696	(T/C)
9807	CakSNP9807	*Kabuli*	Ca_Kabuli_Chr06	7744730	(A/G)
9808	CakSNP9808	*Kabuli*	Ca_Kabuli_Chr06	7744752	(T/C)
9809	CakSNP9809	*Kabuli*	Ca_Kabuli_Chr06	7745641	(A/G)
9810	CakSNP9810	*Kabuli*	Ca_Kabuli_Chr06	7747254	(G/A)
9811	CakSNP9811	*Kabuli*	Ca_Kabuli_Chr06	7764968	(A/G)
9812	CakSNP9812	*Kabuli*	Ca_Kabuli_Chr06	7770357	(C/T)
9813	CakSNP9813	*Kabuli*	Ca_Kabuli_Chr06	7805886	(G/T)
9814	CakSNP9814	*Kabuli*	Ca_Kabuli_Chr06	7821850	(A/G)
9815	CakSNP9815	*Kabuli*	Ca_Kabuli_Chr06	7822666	(C/A)
9816	CakSNP9816	*Kabuli*	Ca_Kabuli_Chr06	7829485	(G/A)
9817	CakSNP9817	*Kabuli*	Ca_Kabuli_Chr06	7829627	(T/G)
9818	CakSNP9818	*Kabuli*	Ca_Kabuli_Chr06	7831214	(C/T)
9819	CakSNP9819	*Kabuli*	Ca_Kabuli_Chr06	7831409	(C/T)
9820	CakSNP9820	*Kabuli*	Ca_Kabuli_Chr06	7831585	(G/A)
9821	CakSNP9821	*Kabuli*	Ca_Kabuli_Chr06	7831874	(A/G)
9822	CakSNP9822	*Kabuli*	Ca_Kabuli_Chr06	7831898	(G/A)
9823	CakSNP9823	*Kabuli*	Ca_Kabuli_Chr06	7831899	(G/A)
9824	CakSNP9824	*Kabuli*	Ca_Kabuli_Chr06	7832029	(C/T)
9825	CakSNP9825	*Kabuli*	Ca_Kabuli_Chr06	7832023	(C/T)
9826	CakSNP9826	*Kabuli*	Ca_Kabuli_Chr06	7834595	(C/T)
9827	CakSNP9827	*Kabuli*	Ca_Kabuli_Chr06	7834922	(G/A)
9828	CakSNP9828	*Kabuli*	Ca_Kabuli_Chr06	7834895	(G/T)
9829	CakSNP9829	*Kabuli*	Ca_Kabuli_Chr06	7834976	(G/A)
9830	CakSNP9830	*Kabuli*	Ca_Kabuli_Chr06	7835427	(G/A)
9831	CakSNP9831	*Kabuli*	Ca_Kabuli_Chr06	7835738	(C/T)
9832	CakSNP9832	*Kabuli*	Ca_Kabuli_Chr06	7835984	(T/A)
9833	CakSNP9833	*Kabuli*	Ca_Kabuli_Chr06	7851000	(G/C)
9834	CakSNP9834	*Kabuli*	Ca_Kabuli_Chr06	7929263	(C/T)
9835	CakSNP9835	*Kabuli*	Ca_Kabuli_Chr06	7929338	(T/C)
9836	CakSNP9836	*Kabuli*	Ca_Kabuli_Chr06	7929339	(G/A)
9837	CakSNP9837	*Kabuli*	Ca_Kabuli_Chr06	7929348	(C/T)
9838	CakSNP9838	*Kabuli*	Ca_Kabuli_Chr06	7929384	(T/C)
9839	CakSNP9839	*Kabuli*	Ca_Kabuli_Chr06	7929493	(A/G)
9840	CakSNP9840	*Kabuli*	Ca_Kabuli_Chr06	7929629	(G/A)
9841	CakSNP9841	*Kabuli*	Ca_Kabuli_Chr06	7929628	(C/T)

S.N.	SNP IDs	Cultivars	Chromosomes/scaffolds	Physical positions (bp)	SNPs
9842	CakSNP9842	*Kabuli*	Ca_Kabuli_Chr06	7929607	(A/C)
9843	CakSNP9843	*Kabuli*	Ca_Kabuli_Chr06	7939277	(T/A)
9844	CakSNP9844	*Kabuli*	Ca_Kabuli_Chr06	7939281	(T/G)
9845	CakSNP9845	*Kabuli*	Ca_Kabuli_Chr06	7939465	(A/G)
9846	CakSNP9846	*Kabuli*	Ca_Kabuli_Chr06	7943546	(C/G)
9847	CakSNP9847	*Kabuli*	Ca_Kabuli_Chr06	7994732	(G/T)
9848	CakSNP9848	*Kabuli*	Ca_Kabuli_Chr06	8010016	(T/C)
9849	CakSNP9849	*Kabuli*	Ca_Kabuli_Chr06	8011685	(G/T)
9850	CakSNP9850	*Kabuli*	Ca_Kabuli_Chr06	8029226	(C/T)
9851	CakSNP9851	*Kabuli*	Ca_Kabuli_Chr06	8041582	(A/G)
9852	CakSNP9852	*Kabuli*	Ca_Kabuli_Chr06	8052987	(G/C)
9853	CakSNP9853	*Kabuli*	Ca_Kabuli_Chr06	8076394	(T/C)
9854	CakSNP9854	*Kabuli*	Ca_Kabuli_Chr06	8076446	(G/A)
9855	CakSNP9855	*Kabuli*	Ca_Kabuli_Chr06	8081800	(C/G)
9856	CakSNP9856	*Kabuli*	Ca_Kabuli_Chr06	8084950	(T/A)
9857	CakSNP9857	*Kabuli*	Ca_Kabuli_Chr06	8090739	(T/A)
9858	CakSNP9858	*Kabuli*	Ca_Kabuli_Chr06	8090980	(A/G)
9859	CakSNP9859	*Kabuli*	Ca_Kabuli_Chr06	8091050	(A/G)
9860	CakSNP9860	*Kabuli*	Ca_Kabuli_Chr06	8139434	(G/A)
9861	CakSNP9861	*Kabuli*	Ca_Kabuli_Chr06	8139446	(C/A)
9862	CakSNP9862	*Kabuli*	Ca_Kabuli_Chr06	8166317	(T/C)
9863	CakSNP9863	*Kabuli*	Ca_Kabuli_Chr06	8166339	(T/C)
9864	CakSNP9864	*Kabuli*	Ca_Kabuli_Chr06	8166435	(A/G)
9865	CakSNP9865	*Kabuli*	Ca_Kabuli_Chr06	8166462	(A/T)
9866	CakSNP9866	*Kabuli*	Ca_Kabuli_Chr06	8170583	(C/T)
9867	CakSNP9867	*Kabuli*	Ca_Kabuli_Chr06	8170633	(A/G)
9868	CakSNP9868	*Kabuli*	Ca_Kabuli_Chr06	8179815	(T/C)
9869	CakSNP9869	*Kabuli*	Ca_Kabuli_Chr06	8179797	(C/T)
9870	CakSNP9870	*Kabuli*	Ca_Kabuli_Chr06	8221204	(C/A)
9871	CakSNP9871	*Kabuli*	Ca_Kabuli_Chr06	8222666	(T/C)
9872	CakSNP9872	*Kabuli*	Ca_Kabuli_Chr06	8222685	(A/T)
9873	CakSNP9873	*Kabuli*	Ca_Kabuli_Chr06	8223986	(C/G)
9874	CakSNP9874	*Kabuli*	Ca_Kabuli_Chr06	8237286	(A/G)
9875	CakSNP9875	*Kabuli*	Ca_Kabuli_Chr06	8243936	(A/T)
9876	CakSNP9876	*Kabuli*	Ca_Kabuli_Chr06	8243947	(T/C)
9877	CakSNP9877	*Kabuli*	Ca_Kabuli_Chr06	8244152	(C/T)
9878	CakSNP9878	*Kabuli*	Ca_Kabuli_Chr06	8244249	(C/T)
9879	CakSNP9879	*Kabuli*	Ca_Kabuli_Chr06	8246037	(T/C)

S.N.	SNP IDs	Cultivars	Chromosomes/scaffolds	Physical positions (bp)	SNPs
9880	CakSNP9880	*Kabuli*	Ca_Kabuli_Chr06	8246187	(A/C)
9881	CakSNP9881	*Kabuli*	Ca_Kabuli_Chr06	8248202	(A/C)
9882	CakSNP9882	*Kabuli*	Ca_Kabuli_Chr06	8251365	(T/C)
9883	CakSNP9883	*Kabuli*	Ca_Kabuli_Chr06	8269326	(A/C)
9884	CakSNP9884	*Kabuli*	Ca_Kabuli_Chr06	8285170	(G/A)
9885	CakSNP9885	*Kabuli*	Ca_Kabuli_Chr06	8285147	(A/G)
9886	CakSNP9886	*Kabuli*	Ca_Kabuli_Chr06	8633231	(C/A)
9887	CakSNP9887	*Kabuli*	Ca_Kabuli_Chr06	8633215	(C/A)
9888	CakSNP9888	*Kabuli*	Ca_Kabuli_Chr06	8633187	(A/C)
9889	CakSNP9889	*Kabuli*	Ca_Kabuli_Chr06	8634513	(G/A)
9890	CakSNP9890	*Kabuli*	Ca_Kabuli_Chr06	8634494	(T/C)
9891	CakSNP9891	*Kabuli*	Ca_Kabuli_Chr06	8643286	(T/C)
9892	CakSNP9892	*Kabuli*	Ca_Kabuli_Chr06	8645079	(A/G)
9893	CakSNP9893	*Kabuli*	Ca_Kabuli_Chr06	8710064	(A/T)
9894	CakSNP9894	*Kabuli*	Ca_Kabuli_Chr06	8787556	(C/T)
9895	CakSNP9895	*Kabuli*	Ca_Kabuli_Chr06	8787557	(T/C)
9896	CakSNP9896	*Kabuli*	Ca_Kabuli_Chr06	8787562	(C/T)
9897	CakSNP9897	*Kabuli*	Ca_Kabuli_Chr06	8787564	(G/T)
9898	CakSNP9898	*Kabuli*	Ca_Kabuli_Chr06	8787568	(G/T)
9899	CakSNP9899	*Kabuli*	Ca_Kabuli_Chr06	8787572	(T/A)
9900	CakSNP9900	*Kabuli*	Ca_Kabuli_Chr06	8787573	(G/A)
9901	CakSNP9901	*Kabuli*	Ca_Kabuli_Chr06	8794430	(A/G)
9902	CakSNP9902	*Kabuli*	Ca_Kabuli_Chr06	8794552	(C/G)
9903	CakSNP9903	*Kabuli*	Ca_Kabuli_Chr06	8890548	(A/G)
9904	CakSNP9904	*Kabuli*	Ca_Kabuli_Chr06	8890552	(C/G)
9905	CakSNP9905	*Kabuli*	Ca_Kabuli_Chr06	8893216	(A/G)
9906	CakSNP9906	*Kabuli*	Ca_Kabuli_Chr06	8968068	(G/A)
9907	CakSNP9907	*Kabuli*	Ca_Kabuli_Chr06	8969711	(C/T)
9908	CakSNP9908	*Kabuli*	Ca_Kabuli_Chr06	8969725	(C/A)
9909	CakSNP9909	*Kabuli*	Ca_Kabuli_Chr06	9053279	(A/G)
9910	CakSNP9910	*Kabuli*	Ca_Kabuli_Chr06	9054596	(T/C)
9911	CakSNP9911	*Kabuli*	Ca_Kabuli_Chr06	9088121	(T/C)
9912	CakSNP9912	*Kabuli*	Ca_Kabuli_Chr06	9088151	(G/A)
9913	CakSNP9913	*Kabuli*	Ca_Kabuli_Chr06	9088163	(A/G)
9914	CakSNP9914	*Kabuli*	Ca_Kabuli_Chr06	9088184	(G/A)
9915	CakSNP9915	*Kabuli*	Ca_Kabuli_Chr06	9088234	(A/C)
9916	CakSNP9916	*Kabuli*	Ca_Kabuli_Chr06	9190620	(G/T)
9917	CakSNP9917	*Kabuli*	Ca_Kabuli_Chr06	9221201	(A/G)

S.N.	SNP IDs	Cultivars	Chromosomes/scaffolds	Physical positions (bp)	SNPs
9918	CakSNP9918	*Kabuli*	Ca_Kabuli_Chr06	9227767	(C/A)
9919	CakSNP9919	*Kabuli*	Ca_Kabuli_Chr06	9228004	(C/A)
9920	CakSNP9920	*Kabuli*	Ca_Kabuli_Chr06	9323775	(A/G)
9921	CakSNP9921	*Kabuli*	Ca_Kabuli_Chr06	9324122	(A/C)
9922	CakSNP9922	*Kabuli*	Ca_Kabuli_Chr06	9373240	(C/A)
9923	CakSNP9923	*Kabuli*	Ca_Kabuli_Chr06	9373383	(G/C)
9924	CakSNP9924	*Kabuli*	Ca_Kabuli_Chr06	9378508	(A/T)
9925	CakSNP9925	*Kabuli*	Ca_Kabuli_Chr06	9378557	(G/C)
9926	CakSNP9926	*Kabuli*	Ca_Kabuli_Chr06	9382091	(C/T)
9927	CakSNP9927	*Kabuli*	Ca_Kabuli_Chr06	9423222	(T/C)
9928	CakSNP9928	*Kabuli*	Ca_Kabuli_Chr06	9424317	(T/C)
9929	CakSNP9929	*Kabuli*	Ca_Kabuli_Chr06	9428522	(C/T)
9930	CakSNP9930	*Kabuli*	Ca_Kabuli_Chr06	9428517	(G/A)
9931	CakSNP9931	*Kabuli*	Ca_Kabuli_Chr06	9523228	(T/C)
9932	CakSNP9932	*Kabuli*	Ca_Kabuli_Chr06	9544233	(T/C)
9933	CakSNP9933	*Kabuli*	Ca_Kabuli_Chr06	9565932	(A/G)
9934	CakSNP9934	*Kabuli*	Ca_Kabuli_Chr06	9568243	(A/C)
9935	CakSNP9935	*Kabuli*	Ca_Kabuli_Chr06	9568247	(G/A)
9936	CakSNP9936	*Kabuli*	Ca_Kabuli_Chr06	9568289	(A/C)
9937	CakSNP9937	*Kabuli*	Ca_Kabuli_Chr06	9569170	(A/G)
9938	CakSNP9938	*Kabuli*	Ca_Kabuli_Chr06	9570753	(C/T)
9939	CakSNP9939	*Kabuli*	Ca_Kabuli_Chr06	9617218	(A/G)
9940	CakSNP9940	*Kabuli*	Ca_Kabuli_Chr06	9617233	(A/G)
9941	CakSNP9941	*Kabuli*	Ca_Kabuli_Chr06	9714240	(T/C)
9942	CakSNP9942	*Kabuli*	Ca_Kabuli_Chr06	9728195	(G/T)
9943	CakSNP9943	*Kabuli*	Ca_Kabuli_Chr06	9728181	(A/G)
9944	CakSNP9944	*Kabuli*	Ca_Kabuli_Chr06	9814378	(G/T)
9945	CakSNP9945	*Kabuli*	Ca_Kabuli_Chr06	9834199	(T/C)
9946	CakSNP9946	*Kabuli*	Ca_Kabuli_Chr06	9834198	(C/A)
9947	CakSNP9947	*Kabuli*	Ca_Kabuli_Chr06	9834187	(A/T)
9948	CakSNP9948	*Kabuli*	Ca_Kabuli_Chr06	9834184	(A/T)
9949	CakSNP9949	*Kabuli*	Ca_Kabuli_Chr06	9834183	(C/T)
9950	CakSNP9950	*Kabuli*	Ca_Kabuli_Chr06	9841836	(G/A)
9951	CakSNP9951	*Kabuli*	Ca_Kabuli_Chr06	9841890	(G/A)
9952	CakSNP9952	*Kabuli*	Ca_Kabuli_Chr06	9845070	(C/T)
9953	CakSNP9953	*Kabuli*	Ca_Kabuli_Chr06	9845076	(T/C)
9954	CakSNP9954	*Kabuli*	Ca_Kabuli_Chr06	9872557	(G/A)
9955	CakSNP9955	*Kabuli*	Ca_Kabuli_Chr06	9872657	(C/T)

S.N.	SNP IDs	Cultivars	Chromosomes/scaffolds	Physical positions (bp)	SNPs
9956	CakSNP9956	*Kabuli*	Ca_Kabuli_Chr06	9872988	(A/T)
9957	CakSNP9957	*Kabuli*	Ca_Kabuli_Chr06	9888237	(G/A)
9958	CakSNP9958	*Kabuli*	Ca_Kabuli_Chr06	9938833	(T/C)
9959	CakSNP9959	*Kabuli*	Ca_Kabuli_Chr06	9939017	(G/A)
9960	CakSNP9960	*Kabuli*	Ca_Kabuli_Chr06	9959686	(T/C)
9961	CakSNP9961	*Kabuli*	Ca_Kabuli_Chr06	10020177	(C/G)
9962	CakSNP9962	*Kabuli*	Ca_Kabuli_Chr06	10020187	(A/G)
9963	CakSNP9963	*Kabuli*	Ca_Kabuli_Chr06	10025115	(A/C)
9964	CakSNP9964	*Kabuli*	Ca_Kabuli_Chr06	10025113	(C/G)
9965	CakSNP9965	*Kabuli*	Ca_Kabuli_Chr06	10027019	(G/A)
9966	CakSNP9966	*Kabuli*	Ca_Kabuli_Chr06	10027015	(G/C)
9967	CakSNP9967	*Kabuli*	Ca_Kabuli_Chr06	10028504	(A/G)
9968	CakSNP9968	*Kabuli*	Ca_Kabuli_Chr06	10028508	(G/C)
9969	CakSNP9969	*Kabuli*	Ca_Kabuli_Chr06	10028515	(A/G)
9970	CakSNP9970	*Kabuli*	Ca_Kabuli_Chr06	10045644	(A/C)
9971	CakSNP9971	*Kabuli*	Ca_Kabuli_Chr06	10045844	(A/G)
9972	CakSNP9972	*Kabuli*	Ca_Kabuli_Chr06	10088963	(G/T)
9973	CakSNP9973	*Kabuli*	Ca_Kabuli_Chr06	10088984	(A/G)
9974	CakSNP9974	*Kabuli*	Ca_Kabuli_Chr06	10100863	(C/G)
9975	CakSNP9975	*Kabuli*	Ca_Kabuli_Chr06	10111488	(C/G)
9976	CakSNP9976	*Kabuli*	Ca_Kabuli_Chr06	10111520	(G/C)
9977	CakSNP9977	*Kabuli*	Ca_Kabuli_Chr06	10111870	(A/G)
9978	CakSNP9978	*Kabuli*	Ca_Kabuli_Chr06	10111938	(G/T)
9979	CakSNP9979	*Kabuli*	Ca_Kabuli_Chr06	10116403	(A/T)
9980	CakSNP9980	*Kabuli*	Ca_Kabuli_Chr06	10116475	(C/A)
9981	CakSNP9981	*Kabuli*	Ca_Kabuli_Chr06	10138984	(C/T)
9982	CakSNP9982	*Kabuli*	Ca_Kabuli_Chr06	10151973	(A/G)
9983	CakSNP9983	*Kabuli*	Ca_Kabuli_Chr06	10151974	(A/G)
9984	CakSNP9984	*Kabuli*	Ca_Kabuli_Chr06	10158850	(T/C)
9985	CakSNP9985	*Kabuli*	Ca_Kabuli_Chr06	10188400	(G/T)
9986	CakSNP9986	*Kabuli*	Ca_Kabuli_Chr06	10191941	(A/G)
9987	CakSNP9987	*Kabuli*	Ca_Kabuli_Chr06	10215262	(T/G)
9988	CakSNP9988	*Kabuli*	Ca_Kabuli_Chr06	10230617	(A/G)
9989	CakSNP9989	*Kabuli*	Ca_Kabuli_Chr06	10230657	(A/G)
9990	CakSNP9990	*Kabuli*	Ca_Kabuli_Chr06	10231092	(T/G)
9991	CakSNP9991	*Kabuli*	Ca_Kabuli_Chr06	10231095	(G/T)
9992	CakSNP9992	*Kabuli*	Ca_Kabuli_Chr06	10234241	(A/G)
9993	CakSNP9993	*Kabuli*	Ca_Kabuli_Chr06	10259614	(C/T)

**S.N.**	**SNP IDs**	**Cultivars**	**Chromosomes/scaffolds**	**Physical positions (bp)**	**SNPs**
9994	CakSNP9994	*Kabuli*	Ca_Kabuli_Chr06	10260274	(C/T)
9995	CakSNP9995	*Kabuli*	Ca_Kabuli_Chr06	10314993	(C/G)
9996	CakSNP9996	*Kabuli*	Ca_Kabuli_Chr06	10395411	(G/A)
9997	CakSNP9997	*Kabuli*	Ca_Kabuli_Chr06	10395379	(T/C)
9998	CakSNP9998	*Kabuli*	Ca_Kabuli_Chr06	10395356	(T/A)
9999	CakSNP9999	*Kabuli*	Ca_Kabuli_Chr06	10441736	(A/C)
10000	CakSNP10000	*Kabuli*	Ca_Kabuli_Chr06	10441705	(T/C)
10001	CakSNP10001	*Kabuli*	Ca_Kabuli_Chr06	10442148	(A/C)
10002	CakSNP10002	*Kabuli*	Ca_Kabuli_Chr06	10445185	(C/T)
10003	CakSNP10003	*Kabuli*	Ca_Kabuli_Chr06	10445274	(G/A)
10004	CakSNP10004	*Kabuli*	Ca_Kabuli_Chr06	10456774	(A/G)
10005	CakSNP10005	*Kabuli*	Ca_Kabuli_Chr06	10456854	(G/T)
10006	CakSNP10006	*Kabuli*	Ca_Kabuli_Chr06	10494134	(C/T)
10007	CakSNP10007	*Kabuli*	Ca_Kabuli_Chr06	10494057	(C/A)
10008	CakSNP10008	*Kabuli*	Ca_Kabuli_Chr06	10494055	(T/A)
10009	CakSNP10009	*Kabuli*	Ca_Kabuli_Chr06	10495747	(G/T)
10010	CakSNP10010	*Kabuli*	Ca_Kabuli_Chr06	10495804	(C/T)
10011	CakSNP10011	*Kabuli*	Ca_Kabuli_Chr06	10502381	(A/G)
10012	CakSNP10012	*Kabuli*	Ca_Kabuli_Chr06	10502387	(C/A)
10013	CakSNP10013	*Kabuli*	Ca_Kabuli_Chr06	10510996	(T/C)
10014	CakSNP10014	*Kabuli*	Ca_Kabuli_Chr06	10520958	(C/T)
10015	CakSNP10015	*Kabuli*	Ca_Kabuli_Chr06	10521741	(A/T)
10016	CakSNP10016	*Kabuli*	Ca_Kabuli_Chr06	10531311	(A/G)
10017	CakSNP10017	*Kabuli*	Ca_Kabuli_Chr06	10531399	(C/T)
10018	CakSNP10018	*Kabuli*	Ca_Kabuli_Chr06	10532182	(A/T)
10019	CakSNP10019	*Kabuli*	Ca_Kabuli_Chr06	10532739	(A/G)
10020	CakSNP10020	*Kabuli*	Ca_Kabuli_Chr06	10539918	(A/C)
10021	CakSNP10021	*Kabuli*	Ca_Kabuli_Chr06	10539977	(C/T)
10022	CakSNP10022	*Kabuli*	Ca_Kabuli_Chr06	10539994	(A/C)
10023	CakSNP10023	*Kabuli*	Ca_Kabuli_Chr06	10567518	(A/G)
10024	CakSNP10024	*Kabuli*	Ca_Kabuli_Chr06	10588030	(C/T)
10025	CakSNP10025	*Kabuli*	Ca_Kabuli_Chr06	10613546	(A/G)
10026	CakSNP10026	*Kabuli*	Ca_Kabuli_Chr06	10613612	(A/G)
10027	CakSNP10027	*Kabuli*	Ca_Kabuli_Chr06	10652739	(C/T)
10028	CakSNP10028	*Kabuli*	Ca_Kabuli_Chr06	10667504	(A/T)
10029	CakSNP10029	*Kabuli*	Ca_Kabuli_Chr06	10667559	(T/C)
10030	CakSNP10030	*Kabuli*	Ca_Kabuli_Chr06	10667640	(T/G)
10031	CakSNP10031	*Kabuli*	Ca_Kabuli_Chr06	10670343	(G/A)

S.N.	SNP IDs	Cultivars	Chromosomes/scaffolds	Physical positions (bp)	SNPs
10032	CakSNP10032	*Kabuli*	Ca_Kabuli_Chr06	10670368	(G/A)
10033	CakSNP10033	*Kabuli*	Ca_Kabuli_Chr06	10670409	(G/A)
10034	CakSNP10034	*Kabuli*	Ca_Kabuli_Chr06	10670412	(A/C)
10035	CakSNP10035	*Kabuli*	Ca_Kabuli_Chr06	10670482	(C/T)
10036	CakSNP10036	*Kabuli*	Ca_Kabuli_Chr06	10670582	(G/A)
10037	CakSNP10037	*Kabuli*	Ca_Kabuli_Chr06	10670675	(C/T)
10038	CakSNP10038	*Kabuli*	Ca_Kabuli_Chr06	10670694	(G/A)
10039	CakSNP10039	*Kabuli*	Ca_Kabuli_Chr06	10670773	(A/C)
10040	CakSNP10040	*Kabuli*	Ca_Kabuli_Chr06	10670959	(T/C)
10041	CakSNP10041	*Kabuli*	Ca_Kabuli_Chr06	10671458	(T/C)
10042	CakSNP10042	*Kabuli*	Ca_Kabuli_Chr06	10671444	(A/C)
10043	CakSNP10043	*Kabuli*	Ca_Kabuli_Chr06	10672468	(C/T)
10044	CakSNP10044	*Kabuli*	Ca_Kabuli_Chr06	10677469	(A/G)
10045	CakSNP10045	*Kabuli*	Ca_Kabuli_Chr06	10677501	(C/T)
10046	CakSNP10046	*Kabuli*	Ca_Kabuli_Chr06	10682221	(G/T)
10047	CakSNP10047	*Kabuli*	Ca_Kabuli_Chr06	10682201	(C/G)
10048	CakSNP10048	*Kabuli*	Ca_Kabuli_Chr06	10683193	(C/T)
10049	CakSNP10049	*Kabuli*	Ca_Kabuli_Chr06	10696986	(A/T)
10050	CakSNP10050	*Kabuli*	Ca_Kabuli_Chr06	10696990	(T/C)
10051	CakSNP10051	*Kabuli*	Ca_Kabuli_Chr06	10697064	(T/A)
10052	CakSNP10052	*Kabuli*	Ca_Kabuli_Chr06	10716986	(G/A)
10053	CakSNP10053	*Kabuli*	Ca_Kabuli_Chr06	10716968	(G/A)
10054	CakSNP10054	*Kabuli*	Ca_Kabuli_Chr06	10744029	(C/A)
10055	CakSNP10055	*Kabuli*	Ca_Kabuli_Chr06	10743996	(A/T)
10056	CakSNP10056	*Kabuli*	Ca_Kabuli_Chr06	10748965	(G/T)
10057	CakSNP10057	*Kabuli*	Ca_Kabuli_Chr06	10779368	(G/C)
10058	CakSNP10058	*Kabuli*	Ca_Kabuli_Chr06	10779465	(A/T)
10059	CakSNP10059	*Kabuli*	Ca_Kabuli_Chr06	10779547	(T/G)
10060	CakSNP10060	*Kabuli*	Ca_Kabuli_Chr06	10779636	(C/A)
10061	CakSNP10061	*Kabuli*	Ca_Kabuli_Chr06	10785480	(T/G)
10062	CakSNP10062	*Kabuli*	Ca_Kabuli_Chr06	10870187	(T/G)
10063	CakSNP10063	*Kabuli*	Ca_Kabuli_Chr06	10870534	(A/G)
10064	CakSNP10064	*Kabuli*	Ca_Kabuli_Chr06	10880791	(C/T)
10065	CakSNP10065	*Kabuli*	Ca_Kabuli_Chr06	10880819	(T/G)
10066	CakSNP10066	*Kabuli*	Ca_Kabuli_Chr06	10881002	(A/G)
10067	CakSNP10067	*Kabuli*	Ca_Kabuli_Chr06	10880985	(C/T)
10068	CakSNP10068	*Kabuli*	Ca_Kabuli_Chr06	10880962	(G/T)
10069	CakSNP10069	*Kabuli*	Ca_Kabuli_Chr06	10991652	(C/A)

S.N.	SNP IDs	Cultivars	Chromosomes/scaffolds	Physical positions (bp)	SNPs
10070	CakSNP10070	*Kabuli*	Ca_Kabuli_Chr06	10991717	(T/A)
10071	CakSNP10071	*Kabuli*	Ca_Kabuli_Chr06	10991737	(A/C)
10072	CakSNP10072	*Kabuli*	Ca_Kabuli_Chr06	11011847	(C/G)
10073	CakSNP10073	*Kabuli*	Ca_Kabuli_Chr06	11128158	(A/G)
10074	CakSNP10074	*Kabuli*	Ca_Kabuli_Chr06	11128170	(T/C)
10075	CakSNP10075	*Kabuli*	Ca_Kabuli_Chr06	11128204	(G/A)
10076	CakSNP10076	*Kabuli*	Ca_Kabuli_Chr06	11131010	(T/A)
10077	CakSNP10077	*Kabuli*	Ca_Kabuli_Chr06	11131464	(G/A)
10078	CakSNP10078	*Kabuli*	Ca_Kabuli_Chr06	11131462	(G/A)
10079	CakSNP10079	*Kabuli*	Ca_Kabuli_Chr06	11131436	(T/G)
10080	CakSNP10080	*Kabuli*	Ca_Kabuli_Chr06	11163819	(T/G)
10081	CakSNP10081	*Kabuli*	Ca_Kabuli_Chr06	11163822	(C/T)
10082	CakSNP10082	*Kabuli*	Ca_Kabuli_Chr06	11163836	(C/T)
10083	CakSNP10083	*Kabuli*	Ca_Kabuli_Chr06	11188803	(G/A)
10084	CakSNP10084	*Kabuli*	Ca_Kabuli_Chr06	11189051	(A/G)
10085	CakSNP10085	*Kabuli*	Ca_Kabuli_Chr06	11229102	(C/T)
10086	CakSNP10086	*Kabuli*	Ca_Kabuli_Chr06	11229143	(A/C)
10087	CakSNP10087	*Kabuli*	Ca_Kabuli_Chr06	11229302	(A/G)
10088	CakSNP10088	*Kabuli*	Ca_Kabuli_Chr06	11229388	(C/T)
10089	CakSNP10089	*Kabuli*	Ca_Kabuli_Chr06	11285884	(G/A)
10090	CakSNP10090	*Kabuli*	Ca_Kabuli_Chr06	11300597	(C/T)
10091	CakSNP10091	*Kabuli*	Ca_Kabuli_Chr06	11300549	(A/C)
10092	CakSNP10092	*Kabuli*	Ca_Kabuli_Chr06	11302037	(A/C)
10093	CakSNP10093	*Kabuli*	Ca_Kabuli_Chr06	11302033	(T/C)
10094	CakSNP10094	*Kabuli*	Ca_Kabuli_Chr06	11314581	(T/C)
10095	CakSNP10095	*Kabuli*	Ca_Kabuli_Chr06	11394963	(A/G)
10096	CakSNP10096	*Kabuli*	Ca_Kabuli_Chr06	11395330	(C/T)
10097	CakSNP10097	*Kabuli*	Ca_Kabuli_Chr06	11508828	(C/T)
10098	CakSNP10098	*Kabuli*	Ca_Kabuli_Chr06	11508824	(T/G)
10099	CakSNP10099	*Kabuli*	Ca_Kabuli_Chr06	11508817	(G/A)
10100	CakSNP10100	*Kabuli*	Ca_Kabuli_Chr06	11508816	(A/C)
10101	CakSNP10101	*Kabuli*	Ca_Kabuli_Chr06	11514926	(T/C)
10102	CakSNP10102	*Kabuli*	Ca_Kabuli_Chr06	11514883	(C/T)
10103	CakSNP10103	*Kabuli*	Ca_Kabuli_Chr06	11514935	(G/A)
10104	CakSNP10104	*Kabuli*	Ca_Kabuli_Chr06	11543615	(G/C)
10105	CakSNP10105	*Kabuli*	Ca_Kabuli_Chr06	11543618	(T/G)
10106	CakSNP10106	*Kabuli*	Ca_Kabuli_Chr06	11665233	(G/A)
10107	CakSNP10107	*Kabuli*	Ca_Kabuli_Chr06	11665338	(T/G)

S.N.	SNP IDs	Cultivars	Chromosomes/scaffolds	Physical positions (bp)	SNPs
10108	CakSNP10108	*Kabuli*	Ca_Kabuli_Chr06	12408955	(A/G)
10109	CakSNP10109	*Kabuli*	Ca_Kabuli_Chr06	12408964	(G/C)
10110	CakSNP10110	*Kabuli*	Ca_Kabuli_Chr06	12434183	(G/T)
10111	CakSNP10111	*Kabuli*	Ca_Kabuli_Chr06	12437275	(A/T)
10112	CakSNP10112	*Kabuli*	Ca_Kabuli_Chr06	12437285	(A/G)
10113	CakSNP10113	*Kabuli*	Ca_Kabuli_Chr06	12437288	(G/A)
10114	CakSNP10114	*Kabuli*	Ca_Kabuli_Chr06	12437319	(C/T)
10115	CakSNP10115	*Kabuli*	Ca_Kabuli_Chr06	12494824	(G/T)
10116	CakSNP10116	*Kabuli*	Ca_Kabuli_Chr06	12494972	(A/G)
10117	CakSNP10117	*Kabuli*	Ca_Kabuli_Chr06	12532947	(T/A)
10118	CakSNP10118	*Kabuli*	Ca_Kabuli_Chr06	12535733	(C/G)
10119	CakSNP10119	*Kabuli*	Ca_Kabuli_Chr06	12619757	(C/T)
10120	CakSNP10120	*Kabuli*	Ca_Kabuli_Chr06	12619786	(C/A)
10121	CakSNP10121	*Kabuli*	Ca_Kabuli_Chr06	12619829	(A/T)
10122	CakSNP10122	*Kabuli*	Ca_Kabuli_Chr06	12647539	(G/A)
10123	CakSNP10123	*Kabuli*	Ca_Kabuli_Chr06	12799890	(T/G)
10124	CakSNP10124	*Kabuli*	Ca_Kabuli_Chr06	12844524	(G/A)
10125	CakSNP10125	*Kabuli*	Ca_Kabuli_Chr06	12893335	(C/A)
10126	CakSNP10126	*Kabuli*	Ca_Kabuli_Chr06	12893424	(C/T)
10127	CakSNP10127	*Kabuli*	Ca_Kabuli_Chr06	12906599	(G/A)
10128	CakSNP10128	*Kabuli*	Ca_Kabuli_Chr06	12906680	(T/C)
10129	CakSNP10129	*Kabuli*	Ca_Kabuli_Chr06	12906669	(G/A)
10130	CakSNP10130	*Kabuli*	Ca_Kabuli_Chr06	12973765	(A/G)
10131	CakSNP10131	*Kabuli*	Ca_Kabuli_Chr06	12976279	(T/A)
10132	CakSNP10132	*Kabuli*	Ca_Kabuli_Chr06	12977129	(A/G)
10133	CakSNP10133	*Kabuli*	Ca_Kabuli_Chr06	12977196	(A/G)
10134	CakSNP10134	*Kabuli*	Ca_Kabuli_Chr06	12977204	(T/G)
10135	CakSNP10135	*Kabuli*	Ca_Kabuli_Chr06	12985896	(A/C)
10136	CakSNP10136	*Kabuli*	Ca_Kabuli_Chr06	12985908	(C/G)
10137	CakSNP10137	*Kabuli*	Ca_Kabuli_Chr06	12985910	(C/T)
10138	CakSNP10138	*Kabuli*	Ca_Kabuli_Chr06	12986776	(A/G)
10139	CakSNP10139	*Kabuli*	Ca_Kabuli_Chr06	12986833	(A/C)
10140	CakSNP10140	*Kabuli*	Ca_Kabuli_Chr06	13008333	(A/T)
10141	CakSNP10141	*Kabuli*	Ca_Kabuli_Chr06	13008360	(C/T)
10142	CakSNP10142	*Kabuli*	Ca_Kabuli_Chr06	13008465	(C/T)
10143	CakSNP10143	*Kabuli*	Ca_Kabuli_Chr06	13099658	(G/T)
10144	CakSNP10144	*Kabuli*	Ca_Kabuli_Chr06	13099656	(G/T)
10145	CakSNP10145	*Kabuli*	Ca_Kabuli_Chr06	13107887	(C/T)

S.N.	SNP IDs	Cultivars	Chromosomes/scaffolds	Physical positions (bp)	SNPs
10146	CakSNP10146	*Kabuli*	Ca_Kabuli_Chr06	13117820	(A/T)
10147	CakSNP10147	*Kabuli*	Ca_Kabuli_Chr06	13131682	(C/G)
10148	CakSNP10148	*Kabuli*	Ca_Kabuli_Chr06	13136852	(C/T)
10149	CakSNP10149	*Kabuli*	Ca_Kabuli_Chr06	13136835	(C/T)
10150	CakSNP10150	*Kabuli*	Ca_Kabuli_Chr06	13136976	(A/G)
10151	CakSNP10151	*Kabuli*	Ca_Kabuli_Chr06	13136955	(G/A)
10152	CakSNP10152	*Kabuli*	Ca_Kabuli_Chr06	13187706	(A/G)
10153	CakSNP10153	*Kabuli*	Ca_Kabuli_Chr06	13187709	(T/C)
10154	CakSNP10154	*Kabuli*	Ca_Kabuli_Chr06	13188124	(G/C)
10155	CakSNP10155	*Kabuli*	Ca_Kabuli_Chr06	13188119	(A/C)
10156	CakSNP10156	*Kabuli*	Ca_Kabuli_Chr06	13188480	(T/C)
10157	CakSNP10157	*Kabuli*	Ca_Kabuli_Chr06	13192177	(G/A)
10158	CakSNP10158	*Kabuli*	Ca_Kabuli_Chr06	13243282	(A/G)
10159	CakSNP10159	*Kabuli*	Ca_Kabuli_Chr06	13252152	(C/T)
10160	CakSNP10160	*Kabuli*	Ca_Kabuli_Chr06	13296128	(G/A)
10161	CakSNP10161	*Kabuli*	Ca_Kabuli_Chr06	13326969	(C/G)
10162	CakSNP10162	*Kabuli*	Ca_Kabuli_Chr06	13340012	(A/G)
10163	CakSNP10163	*Kabuli*	Ca_Kabuli_Chr06	13340757	(T/C)
10164	CakSNP10164	*Kabuli*	Ca_Kabuli_Chr06	13340762	(G/A)
10165	CakSNP10165	*Kabuli*	Ca_Kabuli_Chr06	13340782	(G/A)
10166	CakSNP10166	*Kabuli*	Ca_Kabuli_Chr06	13413848	(C/G)
10167	CakSNP10167	*Kabuli*	Ca_Kabuli_Chr06	13483841	(T/G)
10168	CakSNP10168	*Kabuli*	Ca_Kabuli_Chr06	13565854	(T/C)
10169	CakSNP10169	*Kabuli*	Ca_Kabuli_Chr06	13565990	(C/T)
10170	CakSNP10170	*Kabuli*	Ca_Kabuli_Chr06	13566007	(C/T)
10171	CakSNP10171	*Kabuli*	Ca_Kabuli_Chr06	13566012	(T/G)
10172	CakSNP10172	*Kabuli*	Ca_Kabuli_Chr06	13566041	(C/T)
10173	CakSNP10173	*Kabuli*	Ca_Kabuli_Chr06	13566110	(A/G)
10174	CakSNP10174	*Kabuli*	Ca_Kabuli_Chr06	13566136	(A/C)
10175	CakSNP10175	*Kabuli*	Ca_Kabuli_Chr06	13566130	(G/T)
10176	CakSNP10176	*Kabuli*	Ca_Kabuli_Chr06	13572236	(A/T)
10177	CakSNP10177	*Kabuli*	Ca_Kabuli_Chr06	13572221	(A/C)
10178	CakSNP10178	*Kabuli*	Ca_Kabuli_Chr06	13576963	(T/A)
10179	CakSNP10179	*Kabuli*	Ca_Kabuli_Chr06	13591590	(T/G)
10180	CakSNP10180	*Kabuli*	Ca_Kabuli_Chr06	13593544	(A/G)
10181	CakSNP10181	*Kabuli*	Ca_Kabuli_Chr06	13593564	(C/A)
10182	CakSNP10182	*Kabuli*	Ca_Kabuli_Chr06	13593600	(T/C)
10183	CakSNP10183	*Kabuli*	Ca_Kabuli_Chr06	13593685	(G/A)

**S.N.**	**SNP IDs**	**Cultivars**	**Chromosomes/scaffolds**	**Physical positions (bp)**	**SNPs**
10184	CakSNP10184	*Kabuli*	Ca_Kabuli_Chr06	13593668	(A/G)
10185	CakSNP10185	*Kabuli*	Ca_Kabuli_Chr06	13593661	(T/C)
10186	CakSNP10186	*Kabuli*	Ca_Kabuli_Chr06	13593654	(T/C)
10187	CakSNP10187	*Kabuli*	Ca_Kabuli_Chr06	13619538	(C/T)
10188	CakSNP10188	*Kabuli*	Ca_Kabuli_Chr06	13619555	(C/T)
10189	CakSNP10189	*Kabuli*	Ca_Kabuli_Chr06	13619560	(T/G)
10190	CakSNP10190	*Kabuli*	Ca_Kabuli_Chr06	13619589	(C/T)
10191	CakSNP10191	*Kabuli*	Ca_Kabuli_Chr06	13619658	(A/G)
10192	CakSNP10192	*Kabuli*	Ca_Kabuli_Chr06	13619684	(A/C)
10193	CakSNP10193	*Kabuli*	Ca_Kabuli_Chr06	13622292	(C/T)
10194	CakSNP10194	*Kabuli*	Ca_Kabuli_Chr06	13625388	(A/G)
10195	CakSNP10195	*Kabuli*	Ca_Kabuli_Chr06	13637387	(T/G)
10196	CakSNP10196	*Kabuli*	Ca_Kabuli_Chr06	13637347	(C/T)
10197	CakSNP10197	*Kabuli*	Ca_Kabuli_Chr06	13668916	(T/A)
10198	CakSNP10198	*Kabuli*	Ca_Kabuli_Chr06	13668995	(A/G)
10199	CakSNP10199	*Kabuli*	Ca_Kabuli_Chr06	13669052	(G/C)
10200	CakSNP10200	*Kabuli*	Ca_Kabuli_Chr06	13669049	(C/T)
10201	CakSNP10201	*Kabuli*	Ca_Kabuli_Chr06	13669048	(T/A)
10202	CakSNP10202	*Kabuli*	Ca_Kabuli_Chr06	13669042	(A/G)
10203	CakSNP10203	*Kabuli*	Ca_Kabuli_Chr06	13669018	(G/A)
10204	CakSNP10204	*Kabuli*	Ca_Kabuli_Chr06	13669006	(G/C)
10205	CakSNP10205	*Kabuli*	Ca_Kabuli_Chr06	13714897	(A/T)
10206	CakSNP10206	*Kabuli*	Ca_Kabuli_Chr06	13764980	(T/A)
10207	CakSNP10207	*Kabuli*	Ca_Kabuli_Chr06	13827484	(C/T)
10208	CakSNP10208	*Kabuli*	Ca_Kabuli_Chr06	13827452	(C/G)
10209	CakSNP10209	*Kabuli*	Ca_Kabuli_Chr06	13827444	(G/A)
10210	CakSNP10210	*Kabuli*	Ca_Kabuli_Chr06	13913539	(A/G)
10211	CakSNP10211	*Kabuli*	Ca_Kabuli_Chr06	13917076	(T/C)
10212	CakSNP10212	*Kabuli*	Ca_Kabuli_Chr06	13917135	(T/A)
10213	CakSNP10213	*Kabuli*	Ca_Kabuli_Chr06	13921954	(A/T)
10214	CakSNP10214	*Kabuli*	Ca_Kabuli_Chr06	13941701	(A/T)
10215	CakSNP10215	*Kabuli*	Ca_Kabuli_Chr06	13942961	(A/G)
10216	CakSNP10216	*Kabuli*	Ca_Kabuli_Chr06	13948732	(G/T)
10217	CakSNP10217	*Kabuli*	Ca_Kabuli_Chr06	13948751	(T/G)
10218	CakSNP10218	*Kabuli*	Ca_Kabuli_Chr06	13948778	(A/C)
10219	CakSNP10219	*Kabuli*	Ca_Kabuli_Chr06	13986559	(A/G)
10220	CakSNP10220	*Kabuli*	Ca_Kabuli_Chr06	14042859	(A/T)
10221	CakSNP10221	*Kabuli*	Ca_Kabuli_Chr06	14067312	(A/C)

S.N.	SNP IDs	Cultivars	Chromosomes/scaffolds	Physical positions (bp)	SNPs
10222	CakSNP10222	*Kabuli*	Ca_Kabuli_Chr06	14067326	(C/T)
10223	CakSNP10223	*Kabuli*	Ca_Kabuli_Chr06	14112542	(A/G)
10224	CakSNP10224	*Kabuli*	Ca_Kabuli_Chr06	14148771	(A/G)
10225	CakSNP10225	*Kabuli*	Ca_Kabuli_Chr06	14155543	(C/T)
10226	CakSNP10226	*Kabuli*	Ca_Kabuli_Chr06	14155662	(T/C)
10227	CakSNP10227	*Kabuli*	Ca_Kabuli_Chr06	14155643	(A/C)
10228	CakSNP10228	*Kabuli*	Ca_Kabuli_Chr06	14161084	(G/C)
10229	CakSNP10229	*Kabuli*	Ca_Kabuli_Chr06	14201001	(T/C)
10230	CakSNP10230	*Kabuli*	Ca_Kabuli_Chr06	14200997	(G/A)
10231	CakSNP10231	*Kabuli*	Ca_Kabuli_Chr06	14204400	(T/C)
10232	CakSNP10232	*Kabuli*	Ca_Kabuli_Chr06	14204469	(G/A)
10233	CakSNP10233	*Kabuli*	Ca_Kabuli_Chr06	14254394	(A/G)
10234	CakSNP10234	*Kabuli*	Ca_Kabuli_Chr06	14309042	(C/T)
10235	CakSNP10235	*Kabuli*	Ca_Kabuli_Chr06	14330533	(T/C)
10236	CakSNP10236	*Kabuli*	Ca_Kabuli_Chr06	14332018	(A/C)
10237	CakSNP10237	*Kabuli*	Ca_Kabuli_Chr06	14332404	(A/G)
10238	CakSNP10238	*Kabuli*	Ca_Kabuli_Chr06	14353598	(C/T)
10239	CakSNP10239	*Kabuli*	Ca_Kabuli_Chr06	14353624	(G/A)
10240	CakSNP10240	*Kabuli*	Ca_Kabuli_Chr06	14371493	(C/T)
10241	CakSNP10241	*Kabuli*	Ca_Kabuli_Chr06	14371530	(C/T)
10242	CakSNP10242	*Kabuli*	Ca_Kabuli_Chr06	14375135	(G/A)
10243	CakSNP10243	*Kabuli*	Ca_Kabuli_Chr06	14375141	(G/C)
10244	CakSNP10244	*Kabuli*	Ca_Kabuli_Chr06	14375172	(A/G)
10245	CakSNP10245	*Kabuli*	Ca_Kabuli_Chr06	14375196	(G/A)
10246	CakSNP10246	*Kabuli*	Ca_Kabuli_Chr06	14375207	(C/T)
10247	CakSNP10247	*Kabuli*	Ca_Kabuli_Chr06	14401990	(G/A)
10248	CakSNP10248	*Kabuli*	Ca_Kabuli_Chr06	14416997	(A/G)
10249	CakSNP10249	*Kabuli*	Ca_Kabuli_Chr06	14416981	(A/G)
10250	CakSNP10250	*Kabuli*	Ca_Kabuli_Chr06	14419832	(C/T)
10251	CakSNP10251	*Kabuli*	Ca_Kabuli_Chr06	14526540	(A/T)
10252	CakSNP10252	*Kabuli*	Ca_Kabuli_Chr06	14589478	(T/G)
10253	CakSNP10253	*Kabuli*	Ca_Kabuli_Chr06	14589480	(T/C)
10254	CakSNP10254	*Kabuli*	Ca_Kabuli_Chr06	14589520	(G/C)
10255	CakSNP10255	*Kabuli*	Ca_Kabuli_Chr06	14589744	(C/T)
10256	CakSNP10256	*Kabuli*	Ca_Kabuli_Chr06	14589807	(T/G)
10257	CakSNP10257	*Kabuli*	Ca_Kabuli_Chr06	14589873	(T/C)
10258	CakSNP10258	*Kabuli*	Ca_Kabuli_Chr06	14606892	(A/C)
10259	CakSNP10259	*Kabuli*	Ca_Kabuli_Chr06	14628213	(A/T)

S.N.	SNP IDs	Cultivars	Chromosomes/scaffolds	Physical positions (bp)	SNPs
10260	CakSNP10260	*Kabuli*	Ca_Kabuli_Chr06	14628215	(T/C)
10261	CakSNP10261	*Kabuli*	Ca_Kabuli_Chr06	14628216	(A/C)
10262	CakSNP10262	*Kabuli*	Ca_Kabuli_Chr06	14628284	(G/A)
10263	CakSNP10263	*Kabuli*	Ca_Kabuli_Chr06	14719793	(T/C)
10264	CakSNP10264	*Kabuli*	Ca_Kabuli_Chr06	14719840	(G/T)
10265	CakSNP10265	*Kabuli*	Ca_Kabuli_Chr06	14747608	(T/C)
10266	CakSNP10266	*Kabuli*	Ca_Kabuli_Chr06	14753191	(C/T)
10267	CakSNP10267	*Kabuli*	Ca_Kabuli_Chr06	14753190	(G/A)
10268	CakSNP10268	*Kabuli*	Ca_Kabuli_Chr06	14753158	(A/G)
10269	CakSNP10269	*Kabuli*	Ca_Kabuli_Chr06	14753113	(C/A)
10270	CakSNP10270	*Kabuli*	Ca_Kabuli_Chr06	14753145	(C/A)
10271	CakSNP10271	*Kabuli*	Ca_Kabuli_Chr06	14753150	(G/A)
10272	CakSNP10272	*Kabuli*	Ca_Kabuli_Chr06	14762493	(A/G)
10273	CakSNP10273	*Kabuli*	Ca_Kabuli_Chr06	14771564	(G/T)
10274	CakSNP10274	*Kabuli*	Ca_Kabuli_Chr06	14771709	(G/A)
10275	CakSNP10275	*Kabuli*	Ca_Kabuli_Chr06	14786015	(T/G)
10276	CakSNP10276	*Kabuli*	Ca_Kabuli_Chr06	14789964	(A/C)
10277	CakSNP10277	*Kabuli*	Ca_Kabuli_Chr06	14846794	(C/T)
10278	CakSNP10278	*Kabuli*	Ca_Kabuli_Chr06	14857722	(C/A)
10279	CakSNP10279	*Kabuli*	Ca_Kabuli_Chr06	14857709	(A/G)
10280	CakSNP10280	*Kabuli*	Ca_Kabuli_Chr06	14857680	(G/A)
10281	CakSNP10281	*Kabuli*	Ca_Kabuli_Chr06	14861487	(T/A)
10282	CakSNP10282	*Kabuli*	Ca_Kabuli_Chr06	14884604	(T/G)
10283	CakSNP10283	*Kabuli*	Ca_Kabuli_Chr06	14884606	(T/C)
10284	CakSNP10284	*Kabuli*	Ca_Kabuli_Chr06	14891588	(T/C)
10285	CakSNP10285	*Kabuli*	Ca_Kabuli_Chr06	14891533	(T/C)
10286	CakSNP10286	*Kabuli*	Ca_Kabuli_Chr06	14891597	(C/T)
10287	CakSNP10287	*Kabuli*	Ca_Kabuli_Chr06	14891626	(C/A)
10288	CakSNP10288	*Kabuli*	Ca_Kabuli_Chr06	14941348	(C/T)
10289	CakSNP10289	*Kabuli*	Ca_Kabuli_Chr06	14951877	(C/T)
10290	CakSNP10290	*Kabuli*	Ca_Kabuli_Chr06	14951860	(G/A)
10291	CakSNP10291	*Kabuli*	Ca_Kabuli_Chr06	14982998	(T/C)
10292	CakSNP10292	*Kabuli*	Ca_Kabuli_Chr06	14983391	(C/T)
10293	CakSNP10293	*Kabuli*	Ca_Kabuli_Chr06	15024904	(C/A)
10294	CakSNP10294	*Kabuli*	Ca_Kabuli_Chr06	15024881	(C/A)
10295	CakSNP10295	*Kabuli*	Ca_Kabuli_Chr06	15024979	(T/G)
10296	CakSNP10296	*Kabuli*	Ca_Kabuli_Chr06	15024999	(T/A)
10297	CakSNP10297	*Kabuli*	Ca_Kabuli_Chr06	15025010	(T/C)

S.N.	SNP IDs	Cultivars	Chromosomes/scaffolds	Physical positions (bp)	SNPs
10298	CakSNP10298	*Kabuli*	Ca_Kabuli_Chr06	15025012	(A/C)
10299	CakSNP10299	*Kabuli*	Ca_Kabuli_Chr06	15051094	(T/C)
10300	CakSNP10300	*Kabuli*	Ca_Kabuli_Chr06	15061717	(G/A)
10301	CakSNP10301	*Kabuli*	Ca_Kabuli_Chr06	15123739	(C/T)
10302	CakSNP10302	*Kabuli*	Ca_Kabuli_Chr06	15123771	(T/C)
10303	CakSNP10303	*Kabuli*	Ca_Kabuli_Chr06	15123827	(T/G)
10304	CakSNP10304	*Kabuli*	Ca_Kabuli_Chr06	15123947	(G/A)
10305	CakSNP10305	*Kabuli*	Ca_Kabuli_Chr06	15173607	(G/A)
10306	CakSNP10306	*Kabuli*	Ca_Kabuli_Chr06	15185723	(T/C)
10307	CakSNP10307	*Kabuli*	Ca_Kabuli_Chr06	15204957	(G/C)
10308	CakSNP10308	*Kabuli*	Ca_Kabuli_Chr06	15235575	(A/G)
10309	CakSNP10309	*Kabuli*	Ca_Kabuli_Chr06	15235576	(C/T)
10310	CakSNP10310	*Kabuli*	Ca_Kabuli_Chr06	15235615	(C/T)
10311	CakSNP10311	*Kabuli*	Ca_Kabuli_Chr06	15243538	(A/T)
10312	CakSNP10312	*Kabuli*	Ca_Kabuli_Chr06	15243590	(A/G)
10313	CakSNP10313	*Kabuli*	Ca_Kabuli_Chr06	15243630	(C/T)
10314	CakSNP10314	*Kabuli*	Ca_Kabuli_Chr06	15243674	(T/A)
10315	CakSNP10315	*Kabuli*	Ca_Kabuli_Chr06	15268491	(T/G)
10316	CakSNP10316	*Kabuli*	Ca_Kabuli_Chr06	15294332	(T/G)
10317	CakSNP10317	*Kabuli*	Ca_Kabuli_Chr06	15294331	(C/T)
10318	CakSNP10318	*Kabuli*	Ca_Kabuli_Chr06	15294329	(A/C)
10319	CakSNP10319	*Kabuli*	Ca_Kabuli_Chr06	15301137	(C/T)
10320	CakSNP10320	*Kabuli*	Ca_Kabuli_Chr06	15313655	(G/T)
10321	CakSNP10321	*Kabuli*	Ca_Kabuli_Chr06	15328759	(C/A)
10322	CakSNP10322	*Kabuli*	Ca_Kabuli_Chr06	15328805	(A/T)
10323	CakSNP10323	*Kabuli*	Ca_Kabuli_Chr06	15328948	(A/C)
10324	CakSNP10324	*Kabuli*	Ca_Kabuli_Chr06	15347707	(C/T)
10325	CakSNP10325	*Kabuli*	Ca_Kabuli_Chr06	15393321	(A/T)
10326	CakSNP10326	*Kabuli*	Ca_Kabuli_Chr06	15393349	(T/C)
10327	CakSNP10327	*Kabuli*	Ca_Kabuli_Chr06	15463326	(C/G)
10328	CakSNP10328	*Kabuli*	Ca_Kabuli_Chr06	15475948	(A/G)
10329	CakSNP10329	*Kabuli*	Ca_Kabuli_Chr06	15488265	(C/T)
10330	CakSNP10330	*Kabuli*	Ca_Kabuli_Chr06	15488314	(A/T)
10331	CakSNP10331	*Kabuli*	Ca_Kabuli_Chr06	15488309	(G/A)
10332	CakSNP10332	*Kabuli*	Ca_Kabuli_Chr06	15510433	(A/T)
10333	CakSNP10333	*Kabuli*	Ca_Kabuli_Chr06	15510397	(G/A)
10334	CakSNP10334	*Kabuli*	Ca_Kabuli_Chr06	15537908	(A/C)
10335	CakSNP10335	*Kabuli*	Ca_Kabuli_Chr06	15543295	(C/G)

**S.N.**	**SNP IDs**	**Cultivars**	**Chromosomes/scaffolds**	**Physical positions (bp)**	**SNPs**
10336	CakSNP10336	*Kabuli*	Ca_Kabuli_Chr06	15543293	(T/G)
10337	CakSNP10337	*Kabuli*	Ca_Kabuli_Chr06	15543286	(G/A)
10338	CakSNP10338	*Kabuli*	Ca_Kabuli_Chr06	15550044	(C/T)
10339	CakSNP10339	*Kabuli*	Ca_Kabuli_Chr06	15589539	(A/T)
10340	CakSNP10340	*Kabuli*	Ca_Kabuli_Chr06	15589885	(G/T)
10341	CakSNP10341	*Kabuli*	Ca_Kabuli_Chr06	15635126	(A/T)
10342	CakSNP10342	*Kabuli*	Ca_Kabuli_Chr06	15644412	(A/T)
10343	CakSNP10343	*Kabuli*	Ca_Kabuli_Chr06	15644796	(A/T)
10344	CakSNP10344	*Kabuli*	Ca_Kabuli_Chr06	15857728	(T/C)
10345	CakSNP10345	*Kabuli*	Ca_Kabuli_Chr06	15861278	(G/A)
10346	CakSNP10346	*Kabuli*	Ca_Kabuli_Chr06	16113523	(T/C)
10347	CakSNP10347	*Kabuli*	Ca_Kabuli_Chr06	16113522	(A/T)
10348	CakSNP10348	*Kabuli*	Ca_Kabuli_Chr06	16113521	(G/T)
10349	CakSNP10349	*Kabuli*	Ca_Kabuli_Chr06	16115933	(T/C)
10350	CakSNP10350	*Kabuli*	Ca_Kabuli_Chr06	16147469	(A/G)
10351	CakSNP10351	*Kabuli*	Ca_Kabuli_Chr06	16148277	(A/C)
10352	CakSNP10352	*Kabuli*	Ca_Kabuli_Chr06	16189692	(C/A)
10353	CakSNP10353	*Kabuli*	Ca_Kabuli_Chr06	16189693	(G/T)
10354	CakSNP10354	*Kabuli*	Ca_Kabuli_Chr06	16195748	(T/C)
10355	CakSNP10355	*Kabuli*	Ca_Kabuli_Chr06	16196582	(G/C)
10356	CakSNP10356	*Kabuli*	Ca_Kabuli_Chr06	16315552	(T/A)
10357	CakSNP10357	*Kabuli*	Ca_Kabuli_Chr06	16332377	(G/A)
10358	CakSNP10358	*Kabuli*	Ca_Kabuli_Chr06	16353698	(G/A)
10359	CakSNP10359	*Kabuli*	Ca_Kabuli_Chr06	16450107	(C/T)
10360	CakSNP10360	*Kabuli*	Ca_Kabuli_Chr06	16460495	(A/C)
10361	CakSNP10361	*Kabuli*	Ca_Kabuli_Chr06	16561334	(C/T)
10362	CakSNP10362	*Kabuli*	Ca_Kabuli_Chr06	16635061	(C/T)
10363	CakSNP10363	*Kabuli*	Ca_Kabuli_Chr06	16646769	(A/T)
10364	CakSNP10364	*Kabuli*	Ca_Kabuli_Chr06	16646713	(T/G)
10365	CakSNP10365	*Kabuli*	Ca_Kabuli_Chr06	16665385	(G/C)
10366	CakSNP10366	*Kabuli*	Ca_Kabuli_Chr06	16665477	(T/C)
10367	CakSNP10367	*Kabuli*	Ca_Kabuli_Chr06	16665829	(G/C)
10368	CakSNP10368	*Kabuli*	Ca_Kabuli_Chr06	16677320	(C/A)
10369	CakSNP10369	*Kabuli*	Ca_Kabuli_Chr06	16717984	(G/A)
10370	CakSNP10370	*Kabuli*	Ca_Kabuli_Chr06	16718201	(G/A)
10371	CakSNP10371	*Kabuli*	Ca_Kabuli_Chr06	16719918	(C/A)
10372	CakSNP10372	*Kabuli*	Ca_Kabuli_Chr06	16719919	(T/G)
10373	CakSNP10373	*Kabuli*	Ca_Kabuli_Chr06	16741672	(C/T)

S.N.	SNP IDs	Cultivars	Chromosomes/scaffolds	Physical positions (bp)	SNPs
10374	CakSNP10374	*Kabuli*	Ca_Kabuli_Chr06	16748675	(A/C)
10375	CakSNP10375	*Kabuli*	Ca_Kabuli_Chr06	16768782	(G/A)
10376	CakSNP10376	*Kabuli*	Ca_Kabuli_Chr06	16823954	(A/G)
10377	CakSNP10377	*Kabuli*	Ca_Kabuli_Chr06	16932014	(C/G)
10378	CakSNP10378	*Kabuli*	Ca_Kabuli_Chr06	17048493	(A/C)
10379	CakSNP10379	*Kabuli*	Ca_Kabuli_Chr06	17096214	(C/T)
10380	CakSNP10380	*Kabuli*	Ca_Kabuli_Chr06	17096215	(G/A)
10381	CakSNP10381	*Kabuli*	Ca_Kabuli_Chr06	17096224	(C/T)
10382	CakSNP10382	*Kabuli*	Ca_Kabuli_Chr06	17129574	(A/G)
10383	CakSNP10383	*Kabuli*	Ca_Kabuli_Chr06	17148605	(G/A)
10384	CakSNP10384	*Kabuli*	Ca_Kabuli_Chr06	17148773	(A/G)
10385	CakSNP10385	*Kabuli*	Ca_Kabuli_Chr06	17175334	(C/A)
10386	CakSNP10386	*Kabuli*	Ca_Kabuli_Chr06	17175446	(G/A)
10387	CakSNP10387	*Kabuli*	Ca_Kabuli_Chr06	17258827	(C/A)
10388	CakSNP10388	*Kabuli*	Ca_Kabuli_Chr06	17258886	(T/G)
10389	CakSNP10389	*Kabuli*	Ca_Kabuli_Chr06	17262283	(C/T)
10390	CakSNP10390	*Kabuli*	Ca_Kabuli_Chr06	17263126	(G/A)
10391	CakSNP10391	*Kabuli*	Ca_Kabuli_Chr06	17369613	(A/G)
10392	CakSNP10392	*Kabuli*	Ca_Kabuli_Chr06	17445945	(T/G)
10393	CakSNP10393	*Kabuli*	Ca_Kabuli_Chr06	17477741	(A/G)
10394	CakSNP10394	*Kabuli*	Ca_Kabuli_Chr06	17478220	(G/A)
10395	CakSNP10395	*Kabuli*	Ca_Kabuli_Chr06	17480486	(G/C)
10396	CakSNP10396	*Kabuli*	Ca_Kabuli_Chr06	17481288	(T/C)
10397	CakSNP10397	*Kabuli*	Ca_Kabuli_Chr06	17481307	(C/A)
10398	CakSNP10398	*Kabuli*	Ca_Kabuli_Chr06	17481403	(T/G)
10399	CakSNP10399	*Kabuli*	Ca_Kabuli_Chr06	17481392	(A/G)
10400	CakSNP10400	*Kabuli*	Ca_Kabuli_Chr06	17482626	(A/G)
10401	CakSNP10401	*Kabuli*	Ca_Kabuli_Chr06	17482640	(C/T)
10402	CakSNP10402	*Kabuli*	Ca_Kabuli_Chr06	17482649	(A/G)
10403	CakSNP10403	*Kabuli*	Ca_Kabuli_Chr06	17482718	(G/A)
10404	CakSNP10404	*Kabuli*	Ca_Kabuli_Chr06	17482697	(A/G)
10405	CakSNP10405	*Kabuli*	Ca_Kabuli_Chr06	17573309	(A/G)
10406	CakSNP10406	*Kabuli*	Ca_Kabuli_Chr06	17659067	(A/C)
10407	CakSNP10407	*Kabuli*	Ca_Kabuli_Chr06	17741078	(G/A)
10408	CakSNP10408	*Kabuli*	Ca_Kabuli_Chr06	18027391	(G/A)
10409	CakSNP10409	*Kabuli*	Ca_Kabuli_Chr06	18094691	(C/A)
10410	CakSNP10410	*Kabuli*	Ca_Kabuli_Chr06	18125613	(C/A)
10411	CakSNP10411	*Kabuli*	Ca_Kabuli_Chr06	18232696	(G/A)

S.N.	SNP IDs	Cultivars	Chromosomes/scaffolds	Physical positions (bp)	SNPs
10412	CakSNP10412	*Kabuli*	Ca_Kabuli_Chr06	18268960	(G/A)
10413	CakSNP10413	*Kabuli*	Ca_Kabuli_Chr06	18268982	(A/G)
10414	CakSNP10414	*Kabuli*	Ca_Kabuli_Chr06	18389704	(T/G)
10415	CakSNP10415	*Kabuli*	Ca_Kabuli_Chr06	18389661	(A/C)
10416	CakSNP10416	*Kabuli*	Ca_Kabuli_Chr06	18444725	(T/C)
10417	CakSNP10417	*Kabuli*	Ca_Kabuli_Chr06	18549073	(T/C)
10418	CakSNP10418	*Kabuli*	Ca_Kabuli_Chr06	18562366	(C/A)
10419	CakSNP10419	*Kabuli*	Ca_Kabuli_Chr06	18664114	(A/T)
10420	CakSNP10420	*Kabuli*	Ca_Kabuli_Chr06	18747207	(G/A)
10421	CakSNP10421	*Kabuli*	Ca_Kabuli_Chr06	18752554	(G/T)
10422	CakSNP10422	*Kabuli*	Ca_Kabuli_Chr06	18767357	(G/A)
10423	CakSNP10423	*Kabuli*	Ca_Kabuli_Chr06	18771093	(T/A)
10424	CakSNP10424	*Kabuli*	Ca_Kabuli_Chr06	18798747	(A/C)
10425	CakSNP10425	*Kabuli*	Ca_Kabuli_Chr06	18852806	(T/C)
10426	CakSNP10426	*Kabuli*	Ca_Kabuli_Chr06	18852854	(T/C)
10427	CakSNP10427	*Kabuli*	Ca_Kabuli_Chr06	18876221	(T/C)
10428	CakSNP10428	*Kabuli*	Ca_Kabuli_Chr06	18876213	(A/G)
10429	CakSNP10429	*Kabuli*	Ca_Kabuli_Chr06	18899989	(C/A)
10430	CakSNP10430	*Kabuli*	Ca_Kabuli_Chr06	18900031	(C/T)
10431	CakSNP10431	*Kabuli*	Ca_Kabuli_Chr06	18900034	(G/A)
10432	CakSNP10432	*Kabuli*	Ca_Kabuli_Chr06	18916626	(G/T)
10433	CakSNP10433	*Kabuli*	Ca_Kabuli_Chr06	18916619	(C/T)
10434	CakSNP10434	*Kabuli*	Ca_Kabuli_Chr06	18925002	(A/G)
10435	CakSNP10435	*Kabuli*	Ca_Kabuli_Chr06	18938479	(C/T)
10436	CakSNP10436	*Kabuli*	Ca_Kabuli_Chr06	18938512	(G/A)
10437	CakSNP10437	*Kabuli*	Ca_Kabuli_Chr06	18956194	(G/A)
10438	CakSNP10438	*Kabuli*	Ca_Kabuli_Chr06	18987220	(T/G)
10439	CakSNP10439	*Kabuli*	Ca_Kabuli_Chr06	18999175	(C/T)
10440	CakSNP10440	*Kabuli*	Ca_Kabuli_Chr06	19033068	(A/C)
10441	CakSNP10441	*Kabuli*	Ca_Kabuli_Chr06	19033309	(G/A)
10442	CakSNP10442	*Kabuli*	Ca_Kabuli_Chr06	19034129	(A/G)
10443	CakSNP10443	*Kabuli*	Ca_Kabuli_Chr06	19125254	(G/A)
10444	CakSNP10444	*Kabuli*	Ca_Kabuli_Chr06	19125222	(T/C)
10445	CakSNP10445	*Kabuli*	Ca_Kabuli_Chr06	19129651	(T/C)
10446	CakSNP10446	*Kabuli*	Ca_Kabuli_Chr06	19169687	(T/C)
10447	CakSNP10447	*Kabuli*	Ca_Kabuli_Chr06	19195656	(A/T)
10448	CakSNP10448	*Kabuli*	Ca_Kabuli_Chr06	19195659	(A/C)
10449	CakSNP10449	*Kabuli*	Ca_Kabuli_Chr06	19198386	(G/A)

S.N.	SNP IDs	Cultivars	Chromosomes/scaffolds	Physical positions (bp)	SNPs
10450	CakSNP10450	*Kabuli*	Ca_Kabuli_Chr06	19217820	(G/A)
10451	CakSNP10451	*Kabuli*	Ca_Kabuli_Chr06	19304834	(A/G)
10452	CakSNP10452	*Kabuli*	Ca_Kabuli_Chr06	19440988	(T/C)
10453	CakSNP10453	*Kabuli*	Ca_Kabuli_Chr06	19441372	(T/C)
10454	CakSNP10454	*Kabuli*	Ca_Kabuli_Chr06	19450845	(T/A)
10455	CakSNP10455	*Kabuli*	Ca_Kabuli_Chr06	19458843	(C/T)
10456	CakSNP10456	*Kabuli*	Ca_Kabuli_Chr06	19458898	(T/A)
10457	CakSNP10457	*Kabuli*	Ca_Kabuli_Chr06	19458908	(A/T)
10458	CakSNP10458	*Kabuli*	Ca_Kabuli_Chr06	19476915	(T/C)
10459	CakSNP10459	*Kabuli*	Ca_Kabuli_Chr06	19476916	(A/G)
10460	CakSNP10460	*Kabuli*	Ca_Kabuli_Chr06	19477006	(T/C)
10461	CakSNP10461	*Kabuli*	Ca_Kabuli_Chr06	19478493	(T/A)
10462	CakSNP10462	*Kabuli*	Ca_Kabuli_Chr06	19494588	(G/T)
10463	CakSNP10463	*Kabuli*	Ca_Kabuli_Chr06	19494584	(C/A)
10464	CakSNP10464	*Kabuli*	Ca_Kabuli_Chr06	19551459	(G/A)
10465	CakSNP10465	*Kabuli*	Ca_Kabuli_Chr06	19551504	(G/C)
10466	CakSNP10466	*Kabuli*	Ca_Kabuli_Chr06	19556904	(G/A)
10467	CakSNP10467	*Kabuli*	Ca_Kabuli_Chr06	19557045	(C/T)
10468	CakSNP10468	*Kabuli*	Ca_Kabuli_Chr06	19578972	(T/C)
10469	CakSNP10469	*Kabuli*	Ca_Kabuli_Chr06	19725007	(T/A)
10470	CakSNP10470	*Kabuli*	Ca_Kabuli_Chr06	20505641	(A/T)
10471	CakSNP10471	*Kabuli*	Ca_Kabuli_Chr06	20558142	(C/T)
10472	CakSNP10472	*Kabuli*	Ca_Kabuli_Chr06	20558138	(G/A)
10473	CakSNP10473	*Kabuli*	Ca_Kabuli_Chr06	20660779	(T/C)
10474	CakSNP10474	*Kabuli*	Ca_Kabuli_Chr06	20660782	(T/C)
10475	CakSNP10475	*Kabuli*	Ca_Kabuli_Chr06	20660819	(T/C)
10476	CakSNP10476	*Kabuli*	Ca_Kabuli_Chr06	20660820	(T/A)
10477	CakSNP10477	*Kabuli*	Ca_Kabuli_Chr06	20660841	(A/G)
10478	CakSNP10478	*Kabuli*	Ca_Kabuli_Chr06	20660870	(G/C)
10479	CakSNP10479	*Kabuli*	Ca_Kabuli_Chr06	20671043	(T/C)
10480	CakSNP10480	*Kabuli*	Ca_Kabuli_Chr06	20739258	(A/T)
10481	CakSNP10481	*Kabuli*	Ca_Kabuli_Chr06	20739340	(A/G)
10482	CakSNP10482	*Kabuli*	Ca_Kabuli_Chr06	20766342	(T/A)
10483	CakSNP10483	*Kabuli*	Ca_Kabuli_Chr06	20766377	(C/A)
10484	CakSNP10484	*Kabuli*	Ca_Kabuli_Chr06	20766397	(G/T)
10485	CakSNP10485	*Kabuli*	Ca_Kabuli_Chr06	20770728	(C/T)
10486	CakSNP10486	*Kabuli*	Ca_Kabuli_Chr06	20776473	(A/G)
10487	CakSNP10487	*Kabuli*	Ca_Kabuli_Chr06	20786079	(T/A)

S.N.	SNP IDs	Cultivars	Chromosomes/scaffolds	Physical positions (bp)	SNPs
10488	CakSNP10488	*Kabuli*	Ca_Kabuli_Chr06	20802747	(G/C)
10489	CakSNP10489	*Kabuli*	Ca_Kabuli_Chr06	20830353	(T/G)
10490	CakSNP10490	*Kabuli*	Ca_Kabuli_Chr06	20830634	(T/A)
10491	CakSNP10491	*Kabuli*	Ca_Kabuli_Chr06	20890714	(A/G)
10492	CakSNP10492	*Kabuli*	Ca_Kabuli_Chr06	20917713	(A/C)
10493	CakSNP10493	*Kabuli*	Ca_Kabuli_Chr06	20917694	(A/T)
10494	CakSNP10494	*Kabuli*	Ca_Kabuli_Chr06	21025495	(T/A)
10495	CakSNP10495	*Kabuli*	Ca_Kabuli_Chr06	21025494	(C/T)
10496	CakSNP10496	*Kabuli*	Ca_Kabuli_Chr06	21025540	(C/T)
10497	CakSNP10497	*Kabuli*	Ca_Kabuli_Chr06	21025677	(T/A)
10498	CakSNP10498	*Kabuli*	Ca_Kabuli_Chr06	21025742	(T/G)
10499	CakSNP10499	*Kabuli*	Ca_Kabuli_Chr06	21025706	(C/T)
10500	CakSNP10500	*Kabuli*	Ca_Kabuli_Chr06	21025673	(C/T)
10501	CakSNP10501	*Kabuli*	Ca_Kabuli_Chr06	21025680	(C/T)
10502	CakSNP10502	*Kabuli*	Ca_Kabuli_Chr06	21025682	(C/T)
10503	CakSNP10503	*Kabuli*	Ca_Kabuli_Chr06	21025712	(C/T)
10504	CakSNP10504	*Kabuli*	Ca_Kabuli_Chr06	21025718	(C/A)
10505	CakSNP10505	*Kabuli*	Ca_Kabuli_Chr06	21025894	(G/T)
10506	CakSNP10506	*Kabuli*	Ca_Kabuli_Chr06	21054758	(T/C)
10507	CakSNP10507	*Kabuli*	Ca_Kabuli_Chr06	21058286	(G/T)
10508	CakSNP10508	*Kabuli*	Ca_Kabuli_Chr06	21135764	(C/T)
10509	CakSNP10509	*Kabuli*	Ca_Kabuli_Chr06	21135996	(C/T)
10510	CakSNP10510	*Kabuli*	Ca_Kabuli_Chr06	21139038	(C/G)
10511	CakSNP10511	*Kabuli*	Ca_Kabuli_Chr06	21165963	(A/C)
10512	CakSNP10512	*Kabuli*	Ca_Kabuli_Chr06	21165999	(G/T)
10513	CakSNP10513	*Kabuli*	Ca_Kabuli_Chr06	21170382	(G/A)
10514	CakSNP10514	*Kabuli*	Ca_Kabuli_Chr06	21170401	(C/A)
10515	CakSNP10515	*Kabuli*	Ca_Kabuli_Chr06	21177330	(C/A)
10516	CakSNP10516	*Kabuli*	Ca_Kabuli_Chr06	21238178	(A/G)
10517	CakSNP10517	*Kabuli*	Ca_Kabuli_Chr06	21238711	(C/T)
10518	CakSNP10518	*Kabuli*	Ca_Kabuli_Chr06	21238707	(G/T)
10519	CakSNP10519	*Kabuli*	Ca_Kabuli_Chr06	21276479	(G/T)
10520	CakSNP10520	*Kabuli*	Ca_Kabuli_Chr06	21276551	(C/G)
10521	CakSNP10521	*Kabuli*	Ca_Kabuli_Chr06	21323438	(T/C)
10522	CakSNP10522	*Kabuli*	Ca_Kabuli_Chr06	21323390	(C/G)
10523	CakSNP10523	*Kabuli*	Ca_Kabuli_Chr06	21367869	(A/G)
10524	CakSNP10524	*Kabuli*	Ca_Kabuli_Chr06	21367830	(T/A)
10525	CakSNP10525	*Kabuli*	Ca_Kabuli_Chr06	21368163	(C/T)

S.N.	SNP IDs	Cultivars	Chromosomes/scaffolds	Physical positions (bp)	SNPs
10526	CakSNP10526	*Kabuli*	Ca_Kabuli_Chr06	21385915	(C/A)
10527	CakSNP10527	*Kabuli*	Ca_Kabuli_Chr06	21478820	(G/T)
10528	CakSNP10528	*Kabuli*	Ca_Kabuli_Chr06	21478816	(C/T)
10529	CakSNP10529	*Kabuli*	Ca_Kabuli_Chr06	21483425	(T/G)
10530	CakSNP10530	*Kabuli*	Ca_Kabuli_Chr06	21483429	(C/T)
10531	CakSNP10531	*Kabuli*	Ca_Kabuli_Chr06	21483494	(T/G)
10532	CakSNP10532	*Kabuli*	Ca_Kabuli_Chr06	21489498	(A/G)
10533	CakSNP10533	*Kabuli*	Ca_Kabuli_Chr06	21506224	(A/C)
10534	CakSNP10534	*Kabuli*	Ca_Kabuli_Chr06	21540338	(A/G)
10535	CakSNP10535	*Kabuli*	Ca_Kabuli_Chr06	21540382	(T/G)
10536	CakSNP10536	*Kabuli*	Ca_Kabuli_Chr06	21576745	(G/A)
10537	CakSNP10537	*Kabuli*	Ca_Kabuli_Chr06	21602874	(C/T)
10538	CakSNP10538	*Kabuli*	Ca_Kabuli_Chr06	21603102	(G/C)
10539	CakSNP10539	*Kabuli*	Ca_Kabuli_Chr06	21675734	(T/G)
10540	CakSNP10540	*Kabuli*	Ca_Kabuli_Chr06	21675757	(A/G)
10541	CakSNP10541	*Kabuli*	Ca_Kabuli_Chr06	21675762	(G/T)
10542	CakSNP10542	*Kabuli*	Ca_Kabuli_Chr06	21675816	(C/G)
10543	CakSNP10543	*Kabuli*	Ca_Kabuli_Chr06	21718674	(G/T)
10544	CakSNP10544	*Kabuli*	Ca_Kabuli_Chr06	21756666	(A/T)
10545	CakSNP10545	*Kabuli*	Ca_Kabuli_Chr06	21756706	(C/T)
10546	CakSNP10546	*Kabuli*	Ca_Kabuli_Chr06	21763468	(G/A)
10547	CakSNP10547	*Kabuli*	Ca_Kabuli_Chr06	21763611	(C/T)
10548	CakSNP10548	*Kabuli*	Ca_Kabuli_Chr06	21763582	(C/T)
10549	CakSNP10549	*Kabuli*	Ca_Kabuli_Chr06	21804333	(T/A)
10550	CakSNP10550	*Kabuli*	Ca_Kabuli_Chr06	21804337	(G/C)
10551	CakSNP10551	*Kabuli*	Ca_Kabuli_Chr06	21834111	(C/G)
10552	CakSNP10552	*Kabuli*	Ca_Kabuli_Chr06	21861700	(T/A)
10553	CakSNP10553	*Kabuli*	Ca_Kabuli_Chr06	21914387	(T/C)
10554	CakSNP10554	*Kabuli*	Ca_Kabuli_Chr06	21914380	(C/T)
10555	CakSNP10555	*Kabuli*	Ca_Kabuli_Chr06	21916465	(G/A)
10556	CakSNP10556	*Kabuli*	Ca_Kabuli_Chr06	21916476	(T/C)
10557	CakSNP10557	*Kabuli*	Ca_Kabuli_Chr06	21940793	(T/C)
10558	CakSNP10558	*Kabuli*	Ca_Kabuli_Chr06	21979846	(G/A)
10559	CakSNP10559	*Kabuli*	Ca_Kabuli_Chr06	21979860	(C/T)
10560	CakSNP10560	*Kabuli*	Ca_Kabuli_Chr06	21979873	(A/G)
10561	CakSNP10561	*Kabuli*	Ca_Kabuli_Chr06	21979887	(G/A)
10562	CakSNP10562	*Kabuli*	Ca_Kabuli_Chr06	21979889	(A/G)
10563	CakSNP10563	*Kabuli*	Ca_Kabuli_Chr06	21979893	(C/T)

S.N.	SNP IDs	Cultivars	Chromosomes/scaffolds	Physical positions (bp)	SNPs
10564	CakSNP10564	*Kabuli*	Ca_Kabuli_Chr06	21979903	(T/C)
10565	CakSNP10565	*Kabuli*	Ca_Kabuli_Chr06	22015915	(G/T)
10566	CakSNP10566	*Kabuli*	Ca_Kabuli_Chr06	22016086	(A/G)
10567	CakSNP10567	*Kabuli*	Ca_Kabuli_Chr06	22016767	(C/T)
10568	CakSNP10568	*Kabuli*	Ca_Kabuli_Chr06	22016839	(A/G)
10569	CakSNP10569	*Kabuli*	Ca_Kabuli_Chr06	22038857	(A/G)
10570	CakSNP10570	*Kabuli*	Ca_Kabuli_Chr06	22038906	(A/T)
10571	CakSNP10571	*Kabuli*	Ca_Kabuli_Chr06	22074809	(T/C)
10572	CakSNP10572	*Kabuli*	Ca_Kabuli_Chr06	22075447	(C/G)
10573	CakSNP10573	*Kabuli*	Ca_Kabuli_Chr06	22079148	(A/G)
10574	CakSNP10574	*Kabuli*	Ca_Kabuli_Chr06	22137477	(A/T)
10575	CakSNP10575	*Kabuli*	Ca_Kabuli_Chr06	22137464	(G/A)
10576	CakSNP10576	*Kabuli*	Ca_Kabuli_Chr06	22137569	(G/T)
10577	CakSNP10577	*Kabuli*	Ca_Kabuli_Chr06	22175547	(A/G)
10578	CakSNP10578	*Kabuli*	Ca_Kabuli_Chr06	22192503	(G/A)
10579	CakSNP10579	*Kabuli*	Ca_Kabuli_Chr06	22192498	(A/C)
10580	CakSNP10580	*Kabuli*	Ca_Kabuli_Chr06	22194387	(C/A)
10581	CakSNP10581	*Kabuli*	Ca_Kabuli_Chr06	22201688	(C/T)
10582	CakSNP10582	*Kabuli*	Ca_Kabuli_Chr06	22206341	(G/T)
10583	CakSNP10583	*Kabuli*	Ca_Kabuli_Chr06	22208130	(G/C)
10584	CakSNP10584	*Kabuli*	Ca_Kabuli_Chr06	22208125	(G/A)
10585	CakSNP10585	*Kabuli*	Ca_Kabuli_Chr06	22208093	(G/A)
10586	CakSNP10586	*Kabuli*	Ca_Kabuli_Chr06	22211721	(C/T)
10587	CakSNP10587	*Kabuli*	Ca_Kabuli_Chr06	22211823	(T/C)
10588	CakSNP10588	*Kabuli*	Ca_Kabuli_Chr06	22217158	(A/G)
10589	CakSNP10589	*Kabuli*	Ca_Kabuli_Chr06	22217157	(T/G)
10590	CakSNP10590	*Kabuli*	Ca_Kabuli_Chr06	22217107	(G/A)
10591	CakSNP10591	*Kabuli*	Ca_Kabuli_Chr06	22225288	(T/G)
10592	CakSNP10592	*Kabuli*	Ca_Kabuli_Chr06	22235449	(G/A)
10593	CakSNP10593	*Kabuli*	Ca_Kabuli_Chr06	22235421	(T/A)
10594	CakSNP10594	*Kabuli*	Ca_Kabuli_Chr06	22236475	(T/A)
10595	CakSNP10595	*Kabuli*	Ca_Kabuli_Chr06	22252149	(G/A)
10596	CakSNP10596	*Kabuli*	Ca_Kabuli_Chr06	22262999	(A/C)
10597	CakSNP10597	*Kabuli*	Ca_Kabuli_Chr06	22263011	(T/C)
10598	CakSNP10598	*Kabuli*	Ca_Kabuli_Chr06	22263388	(A/G)
10599	CakSNP10599	*Kabuli*	Ca_Kabuli_Chr06	22263452	(A/T)
10600	CakSNP10600	*Kabuli*	Ca_Kabuli_Chr06	22279906	(T/G)
10601	CakSNP10601	*Kabuli*	Ca_Kabuli_Chr06	22287922	(C/T)

S.N.	SNP IDs	Cultivars	Chromosomes/scaffolds	Physical positions (bp)	SNPs
10602	CakSNP10602	*Kabuli*	Ca_Kabuli_Chr06	22334733	(C/T)
10603	CakSNP10603	*Kabuli*	Ca_Kabuli_Chr06	22352976	(T/C)
10604	CakSNP10604	*Kabuli*	Ca_Kabuli_Chr06	22370242	(C/T)
10605	CakSNP10605	*Kabuli*	Ca_Kabuli_Chr06	22371908	(A/C)
10606	CakSNP10606	*Kabuli*	Ca_Kabuli_Chr06	22446629	(G/A)
10607	CakSNP10607	*Kabuli*	Ca_Kabuli_Chr06	22446621	(A/C)
10608	CakSNP10608	*Kabuli*	Ca_Kabuli_Chr06	22446610	(G/A)
10609	CakSNP10609	*Kabuli*	Ca_Kabuli_Chr06	22446570	(C/T)
10610	CakSNP10610	*Kabuli*	Ca_Kabuli_Chr06	22446649	(C/T)
10611	CakSNP10611	*Kabuli*	Ca_Kabuli_Chr06	22446644	(C/T)
10612	CakSNP10612	*Kabuli*	Ca_Kabuli_Chr06	22482366	(A/C)
10613	CakSNP10613	*Kabuli*	Ca_Kabuli_Chr06	22498553	(T/C)
10614	CakSNP10614	*Kabuli*	Ca_Kabuli_Chr06	22498867	(T/C)
10615	CakSNP10615	*Kabuli*	Ca_Kabuli_Chr06	22518747	(G/T)
10616	CakSNP10616	*Kabuli*	Ca_Kabuli_Chr06	22879982	(C/T)
10617	CakSNP10617	*Kabuli*	Ca_Kabuli_Chr06	22881176	(A/T)
10618	CakSNP10618	*Kabuli*	Ca_Kabuli_Chr06	22933938	(T/A)
10619	CakSNP10619	*Kabuli*	Ca_Kabuli_Chr06	22952185	(G/A)
10620	CakSNP10620	*Kabuli*	Ca_Kabuli_Chr06	22952253	(C/T)
10621	CakSNP10621	*Kabuli*	Ca_Kabuli_Chr06	22966001	(C/A)
10622	CakSNP10622	*Kabuli*	Ca_Kabuli_Chr06	22986891	(T/C)
10623	CakSNP10623	*Kabuli*	Ca_Kabuli_Chr06	22986869	(G/C)
10624	CakSNP10624	*Kabuli*	Ca_Kabuli_Chr06	23014724	(T/A)
10625	CakSNP10625	*Kabuli*	Ca_Kabuli_Chr06	23039165	(T/C)
10626	CakSNP10626	*Kabuli*	Ca_Kabuli_Chr06	23039147	(G/A)
10627	CakSNP10627	*Kabuli*	Ca_Kabuli_Chr06	23039145	(A/G)
10628	CakSNP10628	*Kabuli*	Ca_Kabuli_Chr06	23039125	(G/A)
10629	CakSNP10629	*Kabuli*	Ca_Kabuli_Chr06	23039097	(C/T)
10630	CakSNP10630	*Kabuli*	Ca_Kabuli_Chr06	23046076	(C/T)
10631	CakSNP10631	*Kabuli*	Ca_Kabuli_Chr06	23113706	(A/G)
10632	CakSNP10632	*Kabuli*	Ca_Kabuli_Chr06	23129516	(A/G)
10633	CakSNP10633	*Kabuli*	Ca_Kabuli_Chr06	23129504	(C/G)
10634	CakSNP10634	*Kabuli*	Ca_Kabuli_Chr06	23145532	(T/A)
10635	CakSNP10635	*Kabuli*	Ca_Kabuli_Chr06	23166355	(A/C)
10636	CakSNP10636	*Kabuli*	Ca_Kabuli_Chr06	23253687	(G/A)
10637	CakSNP10637	*Kabuli*	Ca_Kabuli_Chr06	23253973	(G/C)
10638	CakSNP10638	*Kabuli*	Ca_Kabuli_Chr06	23254642	(A/G)
10639	CakSNP10639	*Kabuli*	Ca_Kabuli_Chr06	23256848	(C/G)

S.N.	SNP IDs	Cultivars	Chromosomes/scaffolds	Physical positions (bp)	SNPs
10640	CakSNP10640	*Kabuli*	Ca_Kabuli_Chr06	23256891	(T/G)
10641	CakSNP10641	*Kabuli*	Ca_Kabuli_Chr06	23380746	(G/A)
10642	CakSNP10642	*Kabuli*	Ca_Kabuli_Chr06	23393426	(T/C)
10643	CakSNP10643	*Kabuli*	Ca_Kabuli_Chr06	23393446	(A/G)
10644	CakSNP10644	*Kabuli*	Ca_Kabuli_Chr06	23393469	(T/C)
10645	CakSNP10645	*Kabuli*	Ca_Kabuli_Chr06	23393491	(T/G)
10646	CakSNP10646	*Kabuli*	Ca_Kabuli_Chr06	23399103	(A/G)
10647	CakSNP10647	*Kabuli*	Ca_Kabuli_Chr06	23424061	(T/G)
10648	CakSNP10648	*Kabuli*	Ca_Kabuli_Chr06	23549220	(A/T)
10649	CakSNP10649	*Kabuli*	Ca_Kabuli_Chr06	23638882	(A/G)
10650	CakSNP10650	*Kabuli*	Ca_Kabuli_Chr06	23663204	(C/A)
10651	CakSNP10651	*Kabuli*	Ca_Kabuli_Chr06	23663201	(T/C)
10652	CakSNP10652	*Kabuli*	Ca_Kabuli_Chr06	23663180	(G/T)
10653	CakSNP10653	*Kabuli*	Ca_Kabuli_Chr06	23688740	(C/T)
10654	CakSNP10654	*Kabuli*	Ca_Kabuli_Chr06	23688723	(A/G)
10655	CakSNP10655	*Kabuli*	Ca_Kabuli_Chr06	23691440	(A/C)
10656	CakSNP10656	*Kabuli*	Ca_Kabuli_Chr06	23766943	(C/T)
10657	CakSNP10657	*Kabuli*	Ca_Kabuli_Chr06	23768142	(A/T)
10658	CakSNP10658	*Kabuli*	Ca_Kabuli_Chr06	23809120	(G/A)
10659	CakSNP10659	*Kabuli*	Ca_Kabuli_Chr06	23890836	(A/G)
10660	CakSNP10660	*Kabuli*	Ca_Kabuli_Chr06	23891065	(G/A)
10661	CakSNP10661	*Kabuli*	Ca_Kabuli_Chr06	23916260	(A/G)
10662	CakSNP10662	*Kabuli*	Ca_Kabuli_Chr06	23963812	(T/C)
10663	CakSNP10663	*Kabuli*	Ca_Kabuli_Chr06	23973410	(G/A)
10664	CakSNP10664	*Kabuli*	Ca_Kabuli_Chr06	24007185	(C/T)
10665	CakSNP10665	*Kabuli*	Ca_Kabuli_Chr06	24007863	(T/C)
10666	CakSNP10666	*Kabuli*	Ca_Kabuli_Chr06	24061578	(G/T)
10667	CakSNP10667	*Kabuli*	Ca_Kabuli_Chr06	24085747	(G/T)
10668	CakSNP10668	*Kabuli*	Ca_Kabuli_Chr06	24085795	(C/T)
10669	CakSNP10669	*Kabuli*	Ca_Kabuli_Chr06	24085807	(A/T)
10670	CakSNP10670	*Kabuli*	Ca_Kabuli_Chr06	24138357	(A/G)
10671	CakSNP10671	*Kabuli*	Ca_Kabuli_Chr06	24138388	(T/C)
10672	CakSNP10672	*Kabuli*	Ca_Kabuli_Chr06	24174940	(C/T)
10673	CakSNP10673	*Kabuli*	Ca_Kabuli_Chr06	24260527	(T/C)
10674	CakSNP10674	*Kabuli*	Ca_Kabuli_Chr06	24301508	(T/A)
10675	CakSNP10675	*Kabuli*	Ca_Kabuli_Chr06	24302205	(A/G)
10676	CakSNP10676	*Kabuli*	Ca_Kabuli_Chr06	24381949	(A/G)
10677	CakSNP10677	*Kabuli*	Ca_Kabuli_Chr06	24381937	(G/A)

S.N.	SNP IDs	Cultivars	Chromosomes/scaffolds	Physical positions (bp)	SNPs
10678	CakSNP10678	*Kabuli*	Ca_Kabuli_Chr06	24403316	(G/T)
10679	CakSNP10679	*Kabuli*	Ca_Kabuli_Chr06	24413675	(T/C)
10680	CakSNP10680	*Kabuli*	Ca_Kabuli_Chr06	24413714	(G/A)
10681	CakSNP10681	*Kabuli*	Ca_Kabuli_Chr06	24414512	(G/C)
10682	CakSNP10682	*Kabuli*	Ca_Kabuli_Chr06	24454583	(C/T)
10683	CakSNP10683	*Kabuli*	Ca_Kabuli_Chr06	24454557	(T/C)
10684	CakSNP10684	*Kabuli*	Ca_Kabuli_Chr06	24516523	(A/T)
10685	CakSNP10685	*Kabuli*	Ca_Kabuli_Chr06	24516528	(A/T)
10686	CakSNP10686	*Kabuli*	Ca_Kabuli_Chr06	24516532	(C/T)
10687	CakSNP10687	*Kabuli*	Ca_Kabuli_Chr06	24547086	(T/G)
10688	CakSNP10688	*Kabuli*	Ca_Kabuli_Chr06	24644924	(T/G)
10689	CakSNP10689	*Kabuli*	Ca_Kabuli_Chr06	24661895	(G/A)
10690	CakSNP10690	*Kabuli*	Ca_Kabuli_Chr06	24661916	(C/T)
10691	CakSNP10691	*Kabuli*	Ca_Kabuli_Chr06	24661937	(C/T)
10692	CakSNP10692	*Kabuli*	Ca_Kabuli_Chr06	24661945	(T/A)
10693	CakSNP10693	*Kabuli*	Ca_Kabuli_Chr06	24661986	(G/A)
10694	CakSNP10694	*Kabuli*	Ca_Kabuli_Chr06	25575097	(T/C)
10695	CakSNP10695	*Kabuli*	Ca_Kabuli_Chr06	25575078	(C/T)
10696	CakSNP10696	*Kabuli*	Ca_Kabuli_Chr06	25575062	(G/A)
10697	CakSNP10697	*Kabuli*	Ca_Kabuli_Chr06	25575045	(C/A)
10698	CakSNP10698	*Kabuli*	Ca_Kabuli_Chr06	25604942	(C/A)
10699	CakSNP10699	*Kabuli*	Ca_Kabuli_Chr06	25604936	(C/T)
10700	CakSNP10700	*Kabuli*	Ca_Kabuli_Chr06	25604931	(C/A)
10701	CakSNP10701	*Kabuli*	Ca_Kabuli_Chr06	25604922	(A/G)
10702	CakSNP10702	*Kabuli*	Ca_Kabuli_Chr06	25604954	(C/T)
10703	CakSNP10703	*Kabuli*	Ca_Kabuli_Chr06	25604962	(G/A)
10704	CakSNP10704	*Kabuli*	Ca_Kabuli_Chr06	25604994	(T/C)
10705	CakSNP10705	*Kabuli*	Ca_Kabuli_Chr06	25605006	(C/A)
10706	CakSNP10706	*Kabuli*	Ca_Kabuli_Chr06	25605023	(C/A)
10707	CakSNP10707	*Kabuli*	Ca_Kabuli_Chr06	25605022	(T/C)
10708	CakSNP10708	*Kabuli*	Ca_Kabuli_Chr06	25850717	(C/T)
10709	CakSNP10709	*Kabuli*	Ca_Kabuli_Chr06	25850725	(T/G)
10710	CakSNP10710	*Kabuli*	Ca_Kabuli_Chr06	25850736	(C/T)
10711	CakSNP10711	*Kabuli*	Ca_Kabuli_Chr06	25850760	(T/A)
10712	CakSNP10712	*Kabuli*	Ca_Kabuli_Chr06	25850759	(C/A)
10713	CakSNP10713	*Kabuli*	Ca_Kabuli_Chr06	25850783	(G/A)
10714	CakSNP10714	*Kabuli*	Ca_Kabuli_Chr06	25850816	(C/A)
10715	CakSNP10715	*Kabuli*	Ca_Kabuli_Chr06	25850813	(C/T)

S.N.	SNP IDs	Cultivars	Chromosomes/scaffolds	Physical positions (bp)	SNPs
10716	CakSNP10716	*Kabuli*	Ca_Kabuli_Chr06	25850866	(C/G)
10717	CakSNP10717	*Kabuli*	Ca_Kabuli_Chr06	25850827	(C/T)
10718	CakSNP10718	*Kabuli*	Ca_Kabuli_Chr06	26349363	(C/A)
10719	CakSNP10719	*Kabuli*	Ca_Kabuli_Chr06	26349321	(C/T)
10720	CakSNP10720	*Kabuli*	Ca_Kabuli_Chr06	26349434	(G/A)
10721	CakSNP10721	*Kabuli*	Ca_Kabuli_Chr06	26349451	(T/C)
10722	CakSNP10722	*Kabuli*	Ca_Kabuli_Chr06	26353512	(C/T)
10723	CakSNP10723	*Kabuli*	Ca_Kabuli_Chr06	26353740	(G/C)
10724	CakSNP10724	*Kabuli*	Ca_Kabuli_Chr06	26357149	(C/G)
10725	CakSNP10725	*Kabuli*	Ca_Kabuli_Chr06	26360367	(C/A)
10726	CakSNP10726	*Kabuli*	Ca_Kabuli_Chr06	26385448	(A/G)
10727	CakSNP10727	*Kabuli*	Ca_Kabuli_Chr06	26385444	(C/T)
10728	CakSNP10728	*Kabuli*	Ca_Kabuli_Chr06	26385432	(T/C)
10729	CakSNP10729	*Kabuli*	Ca_Kabuli_Chr06	26426239	(C/A)
10730	CakSNP10730	*Kabuli*	Ca_Kabuli_Chr06	26426252	(A/G)
10731	CakSNP10731	*Kabuli*	Ca_Kabuli_Chr06	26426283	(G/A)
10732	CakSNP10732	*Kabuli*	Ca_Kabuli_Chr06	26527086	(T/C)
10733	CakSNP10733	*Kabuli*	Ca_Kabuli_Chr06	26527111	(G/T)
10734	CakSNP10734	*Kabuli*	Ca_Kabuli_Chr06	26527161	(C/G)
10735	CakSNP10735	*Kabuli*	Ca_Kabuli_Chr06	26527416	(C/T)
10736	CakSNP10736	*Kabuli*	Ca_Kabuli_Chr06	26549168	(T/G)
10737	CakSNP10737	*Kabuli*	Ca_Kabuli_Chr06	26549181	(C/T)
10738	CakSNP10738	*Kabuli*	Ca_Kabuli_Chr06	26551390	(C/T)
10739	CakSNP10739	*Kabuli*	Ca_Kabuli_Chr06	26551703	(T/G)
10740	CakSNP10740	*Kabuli*	Ca_Kabuli_Chr06	26567360	(A/G)
10741	CakSNP10741	*Kabuli*	Ca_Kabuli_Chr06	26656016	(T/C)
10742	CakSNP10742	*Kabuli*	Ca_Kabuli_Chr06	26698265	(A/C)
10743	CakSNP10743	*Kabuli*	Ca_Kabuli_Chr06	26703130	(A/G)
10744	CakSNP10744	*Kabuli*	Ca_Kabuli_Chr06	26703132	(T/C)
10745	CakSNP10745	*Kabuli*	Ca_Kabuli_Chr06	26767389	(T/C)
10746	CakSNP10746	*Kabuli*	Ca_Kabuli_Chr06	26769087	(C/A)
10747	CakSNP10747	*Kabuli*	Ca_Kabuli_Chr06	26928255	(T/C)
10748	CakSNP10748	*Kabuli*	Ca_Kabuli_Chr06	27053217	(A/G)
10749	CakSNP10749	*Kabuli*	Ca_Kabuli_Chr06	27053288	(G/C)
10750	CakSNP10750	*Kabuli*	Ca_Kabuli_Chr06	27139791	(T/C)
10751	CakSNP10751	*Kabuli*	Ca_Kabuli_Chr06	27139816	(T/C)
10752	CakSNP10752	*Kabuli*	Ca_Kabuli_Chr06	27190671	(G/A)
10753	CakSNP10753	*Kabuli*	Ca_Kabuli_Chr06	27192557	(C/T)

S.N.	SNP IDs	Cultivars	Chromosomes/scaffolds	Physical positions (bp)	SNPs
10754	CakSNP10754	*Kabuli*	Ca_Kabuli_Chr06	27310342	(A/T)
10755	CakSNP10755	*Kabuli*	Ca_Kabuli_Chr06	27310341	(G/T)
10756	CakSNP10756	*Kabuli*	Ca_Kabuli_Chr06	27310336	(A/T)
10757	CakSNP10757	*Kabuli*	Ca_Kabuli_Chr06	27310333	(C/T)
10758	CakSNP10758	*Kabuli*	Ca_Kabuli_Chr06	27310328	(T/A)
10759	CakSNP10759	*Kabuli*	Ca_Kabuli_Chr06	27310327	(A/T)
10760	CakSNP10760	*Kabuli*	Ca_Kabuli_Chr06	27310325	(G/T)
10761	CakSNP10761	*Kabuli*	Ca_Kabuli_Chr06	27310315	(C/T)
10762	CakSNP10762	*Kabuli*	Ca_Kabuli_Chr06	27326420	(G/A)
10763	CakSNP10763	*Kabuli*	Ca_Kabuli_Chr06	27326978	(C/T)
10764	CakSNP10764	*Kabuli*	Ca_Kabuli_Chr06	27330059	(T/C)
10765	CakSNP10765	*Kabuli*	Ca_Kabuli_Chr06	27330159	(C/T)
10766	CakSNP10766	*Kabuli*	Ca_Kabuli_Chr06	27331165	(T/C)
10767	CakSNP10767	*Kabuli*	Ca_Kabuli_Chr06	27331318	(A/C)
10768	CakSNP10768	*Kabuli*	Ca_Kabuli_Chr06	27364381	(A/G)
10769	CakSNP10769	*Kabuli*	Ca_Kabuli_Chr06	27364395	(C/T)
10770	CakSNP10770	*Kabuli*	Ca_Kabuli_Chr06	27364402	(T/A)
10771	CakSNP10771	*Kabuli*	Ca_Kabuli_Chr06	27364428	(G/T)
10772	CakSNP10772	*Kabuli*	Ca_Kabuli_Chr06	27364452	(A/T)
10773	CakSNP10773	*Kabuli*	Ca_Kabuli_Chr06	27383746	(A/T)
10774	CakSNP10774	*Kabuli*	Ca_Kabuli_Chr06	27383745	(G/T)
10775	CakSNP10775	*Kabuli*	Ca_Kabuli_Chr06	27383744	(G/T)
10776	CakSNP10776	*Kabuli*	Ca_Kabuli_Chr06	27383718	(C/T)
10777	CakSNP10777	*Kabuli*	Ca_Kabuli_Chr06	27515184	(C/T)
10778	CakSNP10778	*Kabuli*	Ca_Kabuli_Chr06	27515293	(G/A)
10779	CakSNP10779	*Kabuli*	Ca_Kabuli_Chr06	27537433	(A/G)
10780	CakSNP10780	*Kabuli*	Ca_Kabuli_Chr06	27558154	(A/G)
10781	CakSNP10781	*Kabuli*	Ca_Kabuli_Chr06	27578968	(A/G)
10782	CakSNP10782	*Kabuli*	Ca_Kabuli_Chr06	27592117	(G/T)
10783	CakSNP10783	*Kabuli*	Ca_Kabuli_Chr06	27592070	(A/G)
10784	CakSNP10784	*Kabuli*	Ca_Kabuli_Chr06	27606193	(A/C)
10785	CakSNP10785	*Kabuli*	Ca_Kabuli_Chr06	27606210	(C/T)
10786	CakSNP10786	*Kabuli*	Ca_Kabuli_Chr06	27622037	(A/C)
10787	CakSNP10787	*Kabuli*	Ca_Kabuli_Chr06	27622045	(A/G)
10788	CakSNP10788	*Kabuli*	Ca_Kabuli_Chr06	27622053	(A/G)
10789	CakSNP10789	*Kabuli*	Ca_Kabuli_Chr06	27636256	(A/G)
10790	CakSNP10790	*Kabuli*	Ca_Kabuli_Chr06	27655605	(A/G)
10791	CakSNP10791	*Kabuli*	Ca_Kabuli_Chr06	27660980	(C/T)

S.N.	SNP IDs	Cultivars	Chromosomes/scaffolds	Physical positions (bp)	SNPs
10792	CakSNP10792	*Kabuli*	Ca_Kabuli_Chr06	27660971	(T/C)
10793	CakSNP10793	*Kabuli*	Ca_Kabuli_Chr06	27661951	(C/T)
10794	CakSNP10794	*Kabuli*	Ca_Kabuli_Chr06	27662026	(C/T)
10795	CakSNP10795	*Kabuli*	Ca_Kabuli_Chr06	27710531	(T/G)
10796	CakSNP10796	*Kabuli*	Ca_Kabuli_Chr06	27710547	(A/T)
10797	CakSNP10797	*Kabuli*	Ca_Kabuli_Chr06	27842297	(G/A)
10798	CakSNP10798	*Kabuli*	Ca_Kabuli_Chr06	27865048	(C/T)
10799	CakSNP10799	*Kabuli*	Ca_Kabuli_Chr06	27865046	(G/A)
10800	CakSNP10800	*Kabuli*	Ca_Kabuli_Chr06	27864997	(C/A)
10801	CakSNP10801	*Kabuli*	Ca_Kabuli_Chr06	27986719	(A/G)
10802	CakSNP10802	*Kabuli*	Ca_Kabuli_Chr06	28021899	(C/T)
10803	CakSNP10803	*Kabuli*	Ca_Kabuli_Chr06	28044830	(C/A)
10804	CakSNP10804	*Kabuli*	Ca_Kabuli_Chr06	28052463	(T/G)
10805	CakSNP10805	*Kabuli*	Ca_Kabuli_Chr06	28074393	(T/C)
10806	CakSNP10806	*Kabuli*	Ca_Kabuli_Chr06	28076323	(T/A)
10807	CakSNP10807	*Kabuli*	Ca_Kabuli_Chr06	28076364	(T/G)
10808	CakSNP10808	*Kabuli*	Ca_Kabuli_Chr06	28076436	(A/T)
10809	CakSNP10809	*Kabuli*	Ca_Kabuli_Chr06	28177397	(A/C)
10810	CakSNP10810	*Kabuli*	Ca_Kabuli_Chr06	28325392	(C/T)
10811	CakSNP10811	*Kabuli*	Ca_Kabuli_Chr06	28372130	(C/T)
10812	CakSNP10812	*Kabuli*	Ca_Kabuli_Chr06	28574699	(T/C)
10813	CakSNP10813	*Kabuli*	Ca_Kabuli_Chr06	28575509	(T/C)
10814	CakSNP10814	*Kabuli*	Ca_Kabuli_Chr06	28575491	(G/A)
10815	CakSNP10815	*Kabuli*	Ca_Kabuli_Chr06	28615404	(G/T)
10816	CakSNP10816	*Kabuli*	Ca_Kabuli_Chr06	28615475	(A/G)
10817	CakSNP10817	*Kabuli*	Ca_Kabuli_Chr06	28650056	(A/G)
10818	CakSNP10818	*Kabuli*	Ca_Kabuli_Chr06	28727478	(T/C)
10819	CakSNP10819	*Kabuli*	Ca_Kabuli_Chr06	28728293	(A/C)
10820	CakSNP10820	*Kabuli*	Ca_Kabuli_Chr06	28755298	(T/A)
10821	CakSNP10821	*Kabuli*	Ca_Kabuli_Chr06	28757208	(C/A)
10822	CakSNP10822	*Kabuli*	Ca_Kabuli_Chr06	28757204	(G/A)
10823	CakSNP10823	*Kabuli*	Ca_Kabuli_Chr06	28757139	(C/A)
10824	CakSNP10824	*Kabuli*	Ca_Kabuli_Chr06	28887176	(G/A)
10825	CakSNP10825	*Kabuli*	Ca_Kabuli_Chr06	28887188	(G/T)
10826	CakSNP10826	*Kabuli*	Ca_Kabuli_Chr06	28887201	(A/G)
10827	CakSNP10827	*Kabuli*	Ca_Kabuli_Chr06	28887212	(C/T)
10828	CakSNP10828	*Kabuli*	Ca_Kabuli_Chr06	28892743	(G/C)
10829	CakSNP10829	*Kabuli*	Ca_Kabuli_Chr06	28893411	(C/T)

S.N.	SNP IDs	Cultivars	Chromosomes/scaffolds	Physical positions (bp)	SNPs
10830	CakSNP10830	*Kabuli*	Ca_Kabuli_Chr06	28917705	(T/A)
10831	CakSNP10831	*Kabuli*	Ca_Kabuli_Chr06	28947529	(A/C)
10832	CakSNP10832	*Kabuli*	Ca_Kabuli_Chr06	28990518	(A/G)
10833	CakSNP10833	*Kabuli*	Ca_Kabuli_Chr06	29085558	(T/C)
10834	CakSNP10834	*Kabuli*	Ca_Kabuli_Chr06	29163667	(C/A)
10835	CakSNP10835	*Kabuli*	Ca_Kabuli_Chr06	29163714	(G/C)
10836	CakSNP10836	*Kabuli*	Ca_Kabuli_Chr06	29163699	(T/A)
10837	CakSNP10837	*Kabuli*	Ca_Kabuli_Chr06	29163642	(G/A)
10838	CakSNP10838	*Kabuli*	Ca_Kabuli_Chr06	29163640	(A/C)
10839	CakSNP10839	*Kabuli*	Ca_Kabuli_Chr06	29203129	(C/G)
10840	CakSNP10840	*Kabuli*	Ca_Kabuli_Chr06	29203201	(G/C)
10841	CakSNP10841	*Kabuli*	Ca_Kabuli_Chr06	29203210	(T/C)
10842	CakSNP10842	*Kabuli*	Ca_Kabuli_Chr06	29205260	(A/G)
10843	CakSNP10843	*Kabuli*	Ca_Kabuli_Chr06	29205940	(A/G)
10844	CakSNP10844	*Kabuli*	Ca_Kabuli_Chr06	29206008	(A/G)
10845	CakSNP10845	*Kabuli*	Ca_Kabuli_Chr06	29307254	(A/G)
10846	CakSNP10846	*Kabuli*	Ca_Kabuli_Chr06	29495856	(C/T)
10847	CakSNP10847	*Kabuli*	Ca_Kabuli_Chr06	29934610	(A/T)
10848	CakSNP10848	*Kabuli*	Ca_Kabuli_Chr06	30127756	(T/G)
10849	CakSNP10849	*Kabuli*	Ca_Kabuli_Chr06	30257217	(C/T)
10850	CakSNP10850	*Kabuli*	Ca_Kabuli_Chr06	30257284	(A/C)
10851	CakSNP10851	*Kabuli*	Ca_Kabuli_Chr06	30567073	(G/A)
10852	CakSNP10852	*Kabuli*	Ca_Kabuli_Chr06	30676031	(G/T)
10853	CakSNP10853	*Kabuli*	Ca_Kabuli_Chr06	30676320	(T/C)
10854	CakSNP10854	*Kabuli*	Ca_Kabuli_Chr06	30676248	(C/T)
10855	CakSNP10855	*Kabuli*	Ca_Kabuli_Chr06	30761620	(C/T)
10856	CakSNP10856	*Kabuli*	Ca_Kabuli_Chr06	30761641	(G/T)
10857	CakSNP10857	*Kabuli*	Ca_Kabuli_Chr06	30913932	(T/C)
10858	CakSNP10858	*Kabuli*	Ca_Kabuli_Chr06	30926084	(G/A)
10859	CakSNP10859	*Kabuli*	Ca_Kabuli_Chr06	30927221	(C/G)
10860	CakSNP10860	*Kabuli*	Ca_Kabuli_Chr06	31011743	(A/G)
10861	CakSNP10861	*Kabuli*	Ca_Kabuli_Chr06	31024728	(A/T)
10862	CakSNP10862	*Kabuli*	Ca_Kabuli_Chr06	31025560	(C/G)
10863	CakSNP10863	*Kabuli*	Ca_Kabuli_Chr06	31038808	(C/T)
10864	CakSNP10864	*Kabuli*	Ca_Kabuli_Chr06	31048949	(T/C)
10865	CakSNP10865	*Kabuli*	Ca_Kabuli_Chr06	31057806	(C/T)
10866	CakSNP10866	*Kabuli*	Ca_Kabuli_Chr06	31068678	(G/A)
10867	CakSNP10867	*Kabuli*	Ca_Kabuli_Chr06	31324962	(A/C)

S.N.	SNP IDs	Cultivars	Chromosomes/scaffolds	Physical positions (bp)	SNPs
10868	CakSNP10868	*Kabuli*	Ca_Kabuli_Chr06	31324976	(A/G)
10869	CakSNP10869	*Kabuli*	Ca_Kabuli_Chr06	31408895	(G/T)
10870	CakSNP10870	*Kabuli*	Ca_Kabuli_Chr06	31408969	(C/T)
10871	CakSNP10871	*Kabuli*	Ca_Kabuli_Chr06	31495897	(C/A)
10872	CakSNP10872	*Kabuli*	Ca_Kabuli_Chr06	31495903	(A/G)
10873	CakSNP10873	*Kabuli*	Ca_Kabuli_Chr06	31592707	(A/C)
10874	CakSNP10874	*Kabuli*	Ca_Kabuli_Chr06	31595142	(A/C)
10875	CakSNP10875	*Kabuli*	Ca_Kabuli_Chr06	31595136	(A/T)
10876	CakSNP10876	*Kabuli*	Ca_Kabuli_Chr06	31895214	(G/C)
10877	CakSNP10877	*Kabuli*	Ca_Kabuli_Chr06	31895264	(C/T)
10878	CakSNP10878	*Kabuli*	Ca_Kabuli_Chr06	31900781	(T/G)
10879	CakSNP10879	*Kabuli*	Ca_Kabuli_Chr06	31900785	(T/G)
10880	CakSNP10880	*Kabuli*	Ca_Kabuli_Chr06	31900790	(A/C)
10881	CakSNP10881	*Kabuli*	Ca_Kabuli_Chr06	31966982	(C/T)
10882	CakSNP10882	*Kabuli*	Ca_Kabuli_Chr06	31967089	(T/G)
10883	CakSNP10883	*Kabuli*	Ca_Kabuli_Chr06	31967106	(G/A)
10884	CakSNP10884	*Kabuli*	Ca_Kabuli_Chr06	31967109	(T/C)
10885	CakSNP10885	*Kabuli*	Ca_Kabuli_Chr06	31967144	(T/C)
10886	CakSNP10886	*Kabuli*	Ca_Kabuli_Chr06	31967201	(C/T)
10887	CakSNP10887	*Kabuli*	Ca_Kabuli_Chr06	31967195	(G/C)
10888	CakSNP10888	*Kabuli*	Ca_Kabuli_Chr06	31992907	(A/G)
10889	CakSNP10889	*Kabuli*	Ca_Kabuli_Chr06	32035882	(C/T)
10890	CakSNP10890	*Kabuli*	Ca_Kabuli_Chr06	32036033	(G/A)
10891	CakSNP10891	*Kabuli*	Ca_Kabuli_Chr06	32035976	(T/C)
10892	CakSNP10892	*Kabuli*	Ca_Kabuli_Chr06	32140428	(C/A)
10893	CakSNP10893	*Kabuli*	Ca_Kabuli_Chr06	32140429	(G/T)
10894	CakSNP10894	*Kabuli*	Ca_Kabuli_Chr06	32262807	(T/C)
10895	CakSNP10895	*Kabuli*	Ca_Kabuli_Chr06	32262855	(C/T)
10896	CakSNP10896	*Kabuli*	Ca_Kabuli_Chr06	32328468	(T/C)
10897	CakSNP10897	*Kabuli*	Ca_Kabuli_Chr06	32717450	(G/A)
10898	CakSNP10898	*Kabuli*	Ca_Kabuli_Chr06	32717665	(C/A)
10899	CakSNP10899	*Kabuli*	Ca_Kabuli_Chr06	32825567	(C/A)
10900	CakSNP10900	*Kabuli*	Ca_Kabuli_Chr06	32825561	(C/T)
10901	CakSNP10901	*Kabuli*	Ca_Kabuli_Chr06	32825547	(A/G)
10902	CakSNP10902	*Kabuli*	Ca_Kabuli_Chr06	32825579	(C/T)
10903	CakSNP10903	*Kabuli*	Ca_Kabuli_Chr06	32825587	(G/A)
10904	CakSNP10904	*Kabuli*	Ca_Kabuli_Chr06	32825612	(G/A)
10905	CakSNP10905	*Kabuli*	Ca_Kabuli_Chr06	32825619	(T/C)

S.N.	SNP IDs	Cultivars	Chromosomes/scaffolds	Physical positions (bp)	SNPs
10906	CakSNP10906	*Kabuli*	Ca_Kabuli_Chr06	32825631	(C/A)
10907	CakSNP10907	*Kabuli*	Ca_Kabuli_Chr06	32914927	(C/G)
10908	CakSNP10908	*Kabuli*	Ca_Kabuli_Chr06	33502994	(C/A)
10909	CakSNP10909	*Kabuli*	Ca_Kabuli_Chr06	33502997	(T/A)
10910	CakSNP10910	*Kabuli*	Ca_Kabuli_Chr06	33503007	(T/C)
10911	CakSNP10911	*Kabuli*	Ca_Kabuli_Chr06	33503014	(G/T)
10912	CakSNP10912	*Kabuli*	Ca_Kabuli_Chr06	33503020	(G/A)
10913	CakSNP10913	*Kabuli*	Ca_Kabuli_Chr06	33503032	(A/C)
10914	CakSNP10914	*Kabuli*	Ca_Kabuli_Chr06	33503037	(A/T)
10915	CakSNP10915	*Kabuli*	Ca_Kabuli_Chr06	33503051	(G/C)
10916	CakSNP10916	*Kabuli*	Ca_Kabuli_Chr06	33503063	(C/G)
10917	CakSNP10917	*Kabuli*	Ca_Kabuli_Chr06	33503065	(C/A)
10918	CakSNP10918	*Kabuli*	Ca_Kabuli_Chr06	33503071	(A/G)
10919	CakSNP10919	*Kabuli*	Ca_Kabuli_Chr06	33598926	(G/A)
10920	CakSNP10920	*Kabuli*	Ca_Kabuli_Chr06	33704792	(C/T)
10921	CakSNP10921	*Kabuli*	Ca_Kabuli_Chr06	33704807	(C/A)
10922	CakSNP10922	*Kabuli*	Ca_Kabuli_Chr06	33704839	(C/T)
10923	CakSNP10923	*Kabuli*	Ca_Kabuli_Chr06	33704782	(C/T)
10924	CakSNP10924	*Kabuli*	Ca_Kabuli_Chr06	33733259	(A/G)
10925	CakSNP10925	*Kabuli*	Ca_Kabuli_Chr06	34235650	(T/C)
10926	CakSNP10926	*Kabuli*	Ca_Kabuli_Chr06	34407309	(T/C)
10927	CakSNP10927	*Kabuli*	Ca_Kabuli_Chr06	34481266	(A/G)
10928	CakSNP10928	*Kabuli*	Ca_Kabuli_Chr06	34481256	(C/T)
10929	CakSNP10929	*Kabuli*	Ca_Kabuli_Chr06	34535695	(T/C)
10930	CakSNP10930	*Kabuli*	Ca_Kabuli_Chr06	34535983	(T/C)
10931	CakSNP10931	*Kabuli*	Ca_Kabuli_Chr06	34596792	(A/G)
10932	CakSNP10932	*Kabuli*	Ca_Kabuli_Chr06	34634028	(T/A)
10933	CakSNP10933	*Kabuli*	Ca_Kabuli_Chr06	34744433	(A/G)
10934	CakSNP10934	*Kabuli*	Ca_Kabuli_Chr06	34744438	(T/G)
10935	CakSNP10935	*Kabuli*	Ca_Kabuli_Chr06	34744681	(T/G)
10936	CakSNP10936	*Kabuli*	Ca_Kabuli_Chr06	34744665	(G/A)
10937	CakSNP10937	*Kabuli*	Ca_Kabuli_Chr06	34773476	(T/A)
10938	CakSNP10938	*Kabuli*	Ca_Kabuli_Chr06	34793312	(T/G)
10939	CakSNP10939	*Kabuli*	Ca_Kabuli_Chr06	34974503	(C/G)
10940	CakSNP10940	*Kabuli*	Ca_Kabuli_Chr06	35126227	(C/T)
10941	CakSNP10941	*Kabuli*	Ca_Kabuli_Chr06	35126273	(T/C)
10942	CakSNP10942	*Kabuli*	Ca_Kabuli_Chr06	35126389	(G/T)
10943	CakSNP10943	*Kabuli*	Ca_Kabuli_Chr06	35129588	(G/A)

S.N.	SNP IDs	Cultivars	Chromosomes/scaffolds	Physical positions (bp)	SNPs
10944	CakSNP10944	*Kabuli*	Ca_Kabuli_Chr06	35596037	(C/A)
10945	CakSNP10945	*Kabuli*	Ca_Kabuli_Chr06	35596048	(G/A)
10946	CakSNP10946	*Kabuli*	Ca_Kabuli_Chr06	35596064	(T/C)
10947	CakSNP10947	*Kabuli*	Ca_Kabuli_Chr06	35596070	(G/A)
10948	CakSNP10948	*Kabuli*	Ca_Kabuli_Chr06	35596161	(C/T)
10949	CakSNP10949	*Kabuli*	Ca_Kabuli_Chr06	35596174	(G/A)
10950	CakSNP10950	*Kabuli*	Ca_Kabuli_Chr06	35596184	(A/G)
10951	CakSNP10951	*Kabuli*	Ca_Kabuli_Chr06	35596195	(G/C)
10952	CakSNP10952	*Kabuli*	Ca_Kabuli_Chr06	35596197	(G/A)
10953	CakSNP10953	*Kabuli*	Ca_Kabuli_Chr06	35596207	(G/T)
10954	CakSNP10954	*Kabuli*	Ca_Kabuli_Chr06	35596218	(T/C)
10955	CakSNP10955	*Kabuli*	Ca_Kabuli_Chr06	35596220	(G/A)
10956	CakSNP10956	*Kabuli*	Ca_Kabuli_Chr06	35596230	(T/A)
10957	CakSNP10957	*Kabuli*	Ca_Kabuli_Chr06	35596300	(G/A)
10958	CakSNP10958	*Kabuli*	Ca_Kabuli_Chr06	35628131	(T/C)
10959	CakSNP10959	*Kabuli*	Ca_Kabuli_Chr06	35719930	(A/C)
10960	CakSNP10960	*Kabuli*	Ca_Kabuli_Chr06	35743792	(A/G)
10961	CakSNP10961	*Kabuli*	Ca_Kabuli_Chr06	35962309	(C/T)
10962	CakSNP10962	*Kabuli*	Ca_Kabuli_Chr06	35962320	(T/C)
10963	CakSNP10963	*Kabuli*	Ca_Kabuli_Chr06	35962360	(T/C)
10964	CakSNP10964	*Kabuli*	Ca_Kabuli_Chr06	35962364	(T/G)
10965	CakSNP10965	*Kabuli*	Ca_Kabuli_Chr06	35962365	(T/C)
10966	CakSNP10966	*Kabuli*	Ca_Kabuli_Chr06	35962366	(G/A)
10967	CakSNP10967	*Kabuli*	Ca_Kabuli_Chr06	36094855	(T/G)
10968	CakSNP10968	*Kabuli*	Ca_Kabuli_Chr06	36371775	(A/G)
10969	CakSNP10969	*Kabuli*	Ca_Kabuli_Chr06	36374561	(C/T)
10970	CakSNP10970	*Kabuli*	Ca_Kabuli_Chr06	36574836	(A/G)
10971	CakSNP10971	*Kabuli*	Ca_Kabuli_Chr06	36790467	(G/A)
10972	CakSNP10972	*Kabuli*	Ca_Kabuli_Chr06	36790458	(G/A)
10973	CakSNP10973	*Kabuli*	Ca_Kabuli_Chr06	36790428	(G/A)
10974	CakSNP10974	*Kabuli*	Ca_Kabuli_Chr06	36790455	(C/T)
10975	CakSNP10975	*Kabuli*	Ca_Kabuli_Chr06	36790460	(A/G)
10976	CakSNP10976	*Kabuli*	Ca_Kabuli_Chr06	37139425	(C/T)
10977	CakSNP10977	*Kabuli*	Ca_Kabuli_Chr06	37139466	(G/C)
10978	CakSNP10978	*Kabuli*	Ca_Kabuli_Chr06	37139484	(T/C)
10979	CakSNP10979	*Kabuli*	Ca_Kabuli_Chr06	37139486	(G/A)
10980	CakSNP10980	*Kabuli*	Ca_Kabuli_Chr06	37139533	(T/A)
10981	CakSNP10981	*Kabuli*	Ca_Kabuli_Chr06	37139514	(C/T)

S.N.	SNP IDs	Cultivars	Chromosomes/scaffolds	Physical positions (bp)	SNPs
10982	CakSNP10982	*Kabuli*	Ca_Kabuli_Chr06	37139499	(C/G)
10983	CakSNP10983	*Kabuli*	Ca_Kabuli_Chr06	37139610	(T/G)
10984	CakSNP10984	*Kabuli*	Ca_Kabuli_Chr06	37139604	(G/T)
10985	CakSNP10985	*Kabuli*	Ca_Kabuli_Chr06	37321783	(A/T)
10986	CakSNP10986	*Kabuli*	Ca_Kabuli_Chr06	37476426	(C/T)
10987	CakSNP10987	*Kabuli*	Ca_Kabuli_Chr06	37476437	(A/C)
10988	CakSNP10988	*Kabuli*	Ca_Kabuli_Chr06	37476516	(G/T)
10989	CakSNP10989	*Kabuli*	Ca_Kabuli_Chr06	37476465	(G/A)
10990	CakSNP10990	*Kabuli*	Ca_Kabuli_Chr06	37484467	(C/T)
10991	CakSNP10991	*Kabuli*	Ca_Kabuli_Chr06	37484468	(G/T)
10992	CakSNP10992	*Kabuli*	Ca_Kabuli_Chr06	37484470	(C/A)
10993	CakSNP10993	*Kabuli*	Ca_Kabuli_Chr06	37484489	(C/G)
10994	CakSNP10994	*Kabuli*	Ca_Kabuli_Chr06	37484513	(T/C)
10995	CakSNP10995	*Kabuli*	Ca_Kabuli_Chr06	38816345	(C/T)
10996	CakSNP10996	*Kabuli*	Ca_Kabuli_Chr06	38838320	(A/G)
10997	CakSNP10997	*Kabuli*	Ca_Kabuli_Chr06	38840279	(G/A)
10998	CakSNP10998	*Kabuli*	Ca_Kabuli_Chr06	38840315	(G/A)
10999	CakSNP10999	*Kabuli*	Ca_Kabuli_Chr06	38840273	(G/A)
11000	CakSNP11000	*Kabuli*	Ca_Kabuli_Chr06	38881909	(C/A)
11001	CakSNP11001	*Kabuli*	Ca_Kabuli_Chr06	38886735	(C/A)
11002	CakSNP11002	*Kabuli*	Ca_Kabuli_Chr06	38994879	(A/G)
11003	CakSNP11003	*Kabuli*	Ca_Kabuli_Chr06	38994925	(G/T)
11004	CakSNP11004	*Kabuli*	Ca_Kabuli_Chr06	38994954	(A/G)
11005	CakSNP11005	*Kabuli*	Ca_Kabuli_Chr06	38994962	(G/A)
11006	CakSNP11006	*Kabuli*	Ca_Kabuli_Chr06	39028564	(T/G)
11007	CakSNP11007	*Kabuli*	Ca_Kabuli_Chr06	39107187	(C/T)
11008	CakSNP11008	*Kabuli*	Ca_Kabuli_Chr06	39107149	(A/C)
11009	CakSNP11009	*Kabuli*	Ca_Kabuli_Chr06	39107188	(A/C)
11010	CakSNP11010	*Kabuli*	Ca_Kabuli_Chr06	39317016	(T/G)
11011	CakSNP11011	*Kabuli*	Ca_Kabuli_Chr06	39380625	(A/C)
11012	CakSNP11012	*Kabuli*	Ca_Kabuli_Chr06	39908314	(T/G)
11013	CakSNP11013	*Kabuli*	Ca_Kabuli_Chr06	40047823	(A/G)
11014	CakSNP11014	*Kabuli*	Ca_Kabuli_Chr06	40128644	(T/C)
11015	CakSNP11015	*Kabuli*	Ca_Kabuli_Chr06	40468356	(T/A)
11016	CakSNP11016	*Kabuli*	Ca_Kabuli_Chr06	40477285	(G/A)
11017	CakSNP11017	*Kabuli*	Ca_Kabuli_Chr06	40657695	(A/C)
11018	CakSNP11018	*Kabuli*	Ca_Kabuli_Chr06	40735186	(C/A)
11019	CakSNP11019	*Kabuli*	Ca_Kabuli_Chr06	40767566	(A/G)

S.N.	SNP IDs	Cultivars	Chromosomes/scaffolds	Physical positions (bp)	SNPs
11020	CakSNP11020	*Kabuli*	Ca_Kabuli_Chr06	40821354	(C/T)
11021	CakSNP11021	*Kabuli*	Ca_Kabuli_Chr06	40935441	(A/T)
11022	CakSNP11022	*Kabuli*	Ca_Kabuli_Chr06	41121337	(C/T)
11023	CakSNP11023	*Kabuli*	Ca_Kabuli_Chr06	41121330	(G/A)
11024	CakSNP11024	*Kabuli*	Ca_Kabuli_Chr06	41363546	(G/A)
11025	CakSNP11025	*Kabuli*	Ca_Kabuli_Chr06	41420918	(T/A)
11026	CakSNP11026	*Kabuli*	Ca_Kabuli_Chr06	41420917	(A/G)
11027	CakSNP11027	*Kabuli*	Ca_Kabuli_Chr06	41449028	(C/T)
11028	CakSNP11028	*Kabuli*	Ca_Kabuli_Chr06	41682983	(T/C)
11029	CakSNP11029	*Kabuli*	Ca_Kabuli_Chr06	41682966	(C/T)
11030	CakSNP11030	*Kabuli*	Ca_Kabuli_Chr06	41682943	(T/C)
11031	CakSNP11031	*Kabuli*	Ca_Kabuli_Chr06	41697812	(T/G)
11032	CakSNP11032	*Kabuli*	Ca_Kabuli_Chr06	41708309	(G/A)
11033	CakSNP11033	*Kabuli*	Ca_Kabuli_Chr06	41708306	(G/A)
11034	CakSNP11034	*Kabuli*	Ca_Kabuli_Chr06	41708523	(T/G)
11035	CakSNP11035	*Kabuli*	Ca_Kabuli_Chr06	41708474	(G/A)
11036	CakSNP11036	*Kabuli*	Ca_Kabuli_Chr06	41708530	(C/A)
11037	CakSNP11037	*Kabuli*	Ca_Kabuli_Chr06	41907459	(C/G)
11038	CakSNP11038	*Kabuli*	Ca_Kabuli_Chr06	42004851	(A/G)
11039	CakSNP11039	*Kabuli*	Ca_Kabuli_Chr06	42004829	(T/G)
11040	CakSNP11040	*Kabuli*	Ca_Kabuli_Chr06	42004818	(C/G)
11041	CakSNP11041	*Kabuli*	Ca_Kabuli_Chr06	42004813	(C/T)
11042	CakSNP11042	*Kabuli*	Ca_Kabuli_Chr06	42161851	(A/G)
11043	CakSNP11043	*Kabuli*	Ca_Kabuli_Chr06	42161857	(C/G)
11044	CakSNP11044	*Kabuli*	Ca_Kabuli_Chr06	42161866	(T/G)
11045	CakSNP11045	*Kabuli*	Ca_Kabuli_Chr06	42521162	(G/T)
11046	CakSNP11046	*Kabuli*	Ca_Kabuli_Chr06	42855091	(T/G)
11047	CakSNP11047	*Kabuli*	Ca_Kabuli_Chr06	42904998	(C/G)
11048	CakSNP11048	*Kabuli*	Ca_Kabuli_Chr06	42905012	(G/A)
11049	CakSNP11049	*Kabuli*	Ca_Kabuli_Chr06	42905039	(C/T)
11050	CakSNP11050	*Kabuli*	Ca_Kabuli_Chr06	42905035	(T/A)
11051	CakSNP11051	*Kabuli*	Ca_Kabuli_Chr06	42905111	(T/C)
11052	CakSNP11052	*Kabuli*	Ca_Kabuli_Chr06	42905094	(C/T)
11053	CakSNP11053	*Kabuli*	Ca_Kabuli_Chr06	42905090	(G/A)
11054	CakSNP11054	*Kabuli*	Ca_Kabuli_Chr06	42905086	(T/C)
11055	CakSNP11055	*Kabuli*	Ca_Kabuli_Chr06	42905058	(C/G)
11056	CakSNP11056	*Kabuli*	Ca_Kabuli_Chr06	42905059	(A/G)
11057	CakSNP11057	*Kabuli*	Ca_Kabuli_Chr06	42905072	(T/C)

S.N.	SNP IDs	Cultivars	Chromosomes/scaffolds	Physical positions (bp)	SNPs
11058	CakSNP11058	*Kabuli*	Ca_Kabuli_Chr06	42905115	(G/T)
11059	CakSNP11059	*Kabuli*	Ca_Kabuli_Chr06	42905116	(A/T)
11060	CakSNP11060	*Kabuli*	Ca_Kabuli_Chr06	42919899	(G/A)
11061	CakSNP11061	*Kabuli*	Ca_Kabuli_Chr06	42923969	(A/C)
11062	CakSNP11062	*Kabuli*	Ca_Kabuli_Chr06	42925974	(C/T)
11063	CakSNP11063	*Kabuli*	Ca_Kabuli_Chr06	43148829	(C/T)
11064	CakSNP11064	*Kabuli*	Ca_Kabuli_Chr06	43148824	(C/A)
11065	CakSNP11065	*Kabuli*	Ca_Kabuli_Chr06	43148784	(C/G)
11066	CakSNP11066	*Kabuli*	Ca_Kabuli_Chr06	43905509	(A/T)
11067	CakSNP11067	*Kabuli*	Ca_Kabuli_Chr06	43907445	(T/C)
11068	CakSNP11068	*Kabuli*	Ca_Kabuli_Chr06	44530888	(C/A)
11069	CakSNP11069	*Kabuli*	Ca_Kabuli_Chr06	44530889	(A/G)
11070	CakSNP11070	*Kabuli*	Ca_Kabuli_Chr06	44707313	(T/A)
11071	CakSNP11071	*Kabuli*	Ca_Kabuli_Chr06	44711166	(A/G)
11072	CakSNP11072	*Kabuli*	Ca_Kabuli_Chr06	44792535	(C/T)
11073	CakSNP11073	*Kabuli*	Ca_Kabuli_Chr06	44792529	(G/C)
11074	CakSNP11074	*Kabuli*	Ca_Kabuli_Chr06	44792493	(C/T)
11075	CakSNP11075	*Kabuli*	Ca_Kabuli_Chr06	44792486	(G/A)
11076	CakSNP11076	*Kabuli*	Ca_Kabuli_Chr06	44937666	(C/T)
11077	CakSNP11077	*Kabuli*	Ca_Kabuli_Chr06	44944917	(G/A)
11078	CakSNP11078	*Kabuli*	Ca_Kabuli_Chr06	45053093	(A/G)
11079	CakSNP11079	*Kabuli*	Ca_Kabuli_Chr06	45053214	(C/T)
11080	CakSNP11080	*Kabuli*	Ca_Kabuli_Chr06	45131295	(C/T)
11081	CakSNP11081	*Kabuli*	Ca_Kabuli_Chr06	45132545	(A/G)
11082	CakSNP11082	*Kabuli*	Ca_Kabuli_Chr06	45178060	(G/A)
11083	CakSNP11083	*Kabuli*	Ca_Kabuli_Chr06	45178056	(A/G)
11084	CakSNP11084	*Kabuli*	Ca_Kabuli_Chr06	45178045	(G/T)
11085	CakSNP11085	*Kabuli*	Ca_Kabuli_Chr06	45178044	(G/A)
11086	CakSNP11086	*Kabuli*	Ca_Kabuli_Chr06	45178033	(C/A)
11087	CakSNP11087	*Kabuli*	Ca_Kabuli_Chr06	45178061	(C/T)
11088	CakSNP11088	*Kabuli*	Ca_Kabuli_Chr06	45201783	(C/T)
11089	CakSNP11089	*Kabuli*	Ca_Kabuli_Chr06	45293271	(T/C)
11090	CakSNP11090	*Kabuli*	Ca_Kabuli_Chr06	45500961	(T/A)
11091	CakSNP11091	*Kabuli*	Ca_Kabuli_Chr06	45501099	(C/A)
11092	CakSNP11092	*Kabuli*	Ca_Kabuli_Chr06	45505942	(A/G)
11093	CakSNP11093	*Kabuli*	Ca_Kabuli_Chr06	45550031	(C/A)
11094	CakSNP11094	*Kabuli*	Ca_Kabuli_Chr06	45554040	(G/A)
11095	CakSNP11095	*Kabuli*	Ca_Kabuli_Chr06	45557656	(T/C)

S.N.	SNP IDs	Cultivars	Chromosomes/scaffolds	Physical positions (bp)	SNPs
11096	CakSNP11096	*Kabuli*	Ca_Kabuli_Chr06	45614017	(T/C)
11097	CakSNP11097	*Kabuli*	Ca_Kabuli_Chr06	45637271	(T/G)
11098	CakSNP11098	*Kabuli*	Ca_Kabuli_Chr06	45637292	(C/A)
11099	CakSNP11099	*Kabuli*	Ca_Kabuli_Chr06	45637293	(T/C)
11100	CakSNP11100	*Kabuli*	Ca_Kabuli_Chr06	45637322	(C/A)
11101	CakSNP11101	*Kabuli*	Ca_Kabuli_Chr06	45637309	(G/A)
11102	CakSNP11102	*Kabuli*	Ca_Kabuli_Chr06	45637308	(G/T)
11103	CakSNP11103	*Kabuli*	Ca_Kabuli_Chr06	45693275	(G/A)
11104	CakSNP11104	*Kabuli*	Ca_Kabuli_Chr06	45695051	(T/G)
11105	CakSNP11105	*Kabuli*	Ca_Kabuli_Chr06	45695043	(C/T)
11106	CakSNP11106	*Kabuli*	Ca_Kabuli_Chr06	45695035	(G/A)
11107	CakSNP11107	*Kabuli*	Ca_Kabuli_Chr06	45709318	(T/C)
11108	CakSNP11108	*Kabuli*	Ca_Kabuli_Chr06	45773318	(C/T)
11109	CakSNP11109	*Kabuli*	Ca_Kabuli_Chr06	45773309	(A/G)
11110	CakSNP11110	*Kabuli*	Ca_Kabuli_Chr06	45773298	(C/G)
11111	CakSNP11111	*Kabuli*	Ca_Kabuli_Chr06	45773409	(T/A)
11112	CakSNP11112	*Kabuli*	Ca_Kabuli_Chr06	45773408	(T/A)
11113	CakSNP11113	*Kabuli*	Ca_Kabuli_Chr06	45773373	(G/T)
11114	CakSNP11114	*Kabuli*	Ca_Kabuli_Chr06	45773367	(G/T)
11115	CakSNP11115	*Kabuli*	Ca_Kabuli_Chr06	45773349	(C/T)
11116	CakSNP11116	*Kabuli*	Ca_Kabuli_Chr06	45812152	(G/A)
11117	CakSNP11117	*Kabuli*	Ca_Kabuli_Chr06	45976962	(G/A)
11118	CakSNP11118	*Kabuli*	Ca_Kabuli_Chr06	45978206	(T/C)
11119	CakSNP11119	*Kabuli*	Ca_Kabuli_Chr06	45978263	(G/A)
11120	CakSNP11120	*Kabuli*	Ca_Kabuli_Chr06	46158211	(C/T)
11121	CakSNP11121	*Kabuli*	Ca_Kabuli_Chr06	46734706	(C/G)
11122	CakSNP11122	*Kabuli*	Ca_Kabuli_Chr06	46734725	(G/A)
11123	CakSNP11123	*Kabuli*	Ca_Kabuli_Chr06	46734726	(T/C)
11124	CakSNP11124	*Kabuli*	Ca_Kabuli_Chr06	46765659	(G/A)
11125	CakSNP11125	*Kabuli*	Ca_Kabuli_Chr06	46765661	(A/T)
11126	CakSNP11126	*Kabuli*	Ca_Kabuli_Chr06	46765673	(G/A)
11127	CakSNP11127	*Kabuli*	Ca_Kabuli_Chr06	46765688	(A/C)
11128	CakSNP11128	*Kabuli*	Ca_Kabuli_Chr06	46765725	(C/T)
11129	CakSNP11129	*Kabuli*	Ca_Kabuli_Chr06	46765778	(C/A)
11130	CakSNP11130	*Kabuli*	Ca_Kabuli_Chr06	46765696	(C/T)
11131	CakSNP11131	*Kabuli*	Ca_Kabuli_Chr06	46830497	(A/T)
11132	CakSNP11132	*Kabuli*	Ca_Kabuli_Chr06	46896903	(C/T)
11133	CakSNP11133	*Kabuli*	Ca_Kabuli_Chr06	46896910	(A/C)

S.N.	SNP IDs	Cultivars	Chromosomes/scaffolds	Physical positions (bp)	SNPs
11134	CakSNP11134	*Kabuli*	Ca_Kabuli_Chr06	46898225	(A/C)
11135	CakSNP11135	*Kabuli*	Ca_Kabuli_Chr06	46898305	(T/G)
11136	CakSNP11136	*Kabuli*	Ca_Kabuli_Chr06	46898316	(T/G)
11137	CakSNP11137	*Kabuli*	Ca_Kabuli_Chr06	46898317	(C/T)
11138	CakSNP11138	*Kabuli*	Ca_Kabuli_Chr06	46898363	(C/T)
11139	CakSNP11139	*Kabuli*	Ca_Kabuli_Chr06	46898329	(T/A)
11140	CakSNP11140	*Kabuli*	Ca_Kabuli_Chr06	46898747	(G/A)
11141	CakSNP11141	*Kabuli*	Ca_Kabuli_Chr06	46898743	(A/G)
11142	CakSNP11142	*Kabuli*	Ca_Kabuli_Chr06	46899229	(G/A)
11143	CakSNP11143	*Kabuli*	Ca_Kabuli_Chr06	46979240	(C/T)
11144	CakSNP11144	*Kabuli*	Ca_Kabuli_Chr06	46979267	(C/A)
11145	CakSNP11145	*Kabuli*	Ca_Kabuli_Chr06	46998948	(G/A)
11146	CakSNP11146	*Kabuli*	Ca_Kabuli_Chr06	46999012	(C/T)
11147	CakSNP11147	*Kabuli*	Ca_Kabuli_Chr06	47104478	(T/G)
11148	CakSNP11148	*Kabuli*	Ca_Kabuli_Chr06	47104505	(C/T)
11149	CakSNP11149	*Kabuli*	Ca_Kabuli_Chr06	47106174	(A/G)
11150	CakSNP11150	*Kabuli*	Ca_Kabuli_Chr06	47106238	(C/A)
11151	CakSNP11151	*Kabuli*	Ca_Kabuli_Chr06	47232630	(C/T)
11152	CakSNP11152	*Kabuli*	Ca_Kabuli_Chr06	47232631	(C/G)
11153	CakSNP11153	*Kabuli*	Ca_Kabuli_Chr06	47232685	(T/A)
11154	CakSNP11154	*Kabuli*	Ca_Kabuli_Chr06	47440468	(A/T)
11155	CakSNP11155	*Kabuli*	Ca_Kabuli_Chr06	47575655	(G/T)
11156	CakSNP11156	*Kabuli*	Ca_Kabuli_Chr06	47575659	(C/G)
11157	CakSNP11157	*Kabuli*	Ca_Kabuli_Chr06	47906683	(G/A)
11158	CakSNP11158	*Kabuli*	Ca_Kabuli_Chr06	47906680	(C/T)
11159	CakSNP11159	*Kabuli*	Ca_Kabuli_Chr06	47906629	(T/G)
11160	CakSNP11160	*Kabuli*	Ca_Kabuli_Chr06	48088550	(G/A)
11161	CakSNP11161	*Kabuli*	Ca_Kabuli_Chr06	48088592	(C/T)
11162	CakSNP11162	*Kabuli*	Ca_Kabuli_Chr06	48255392	(G/A)
11163	CakSNP11163	*Kabuli*	Ca_Kabuli_Chr06	48255538	(A/G)
11164	CakSNP11164	*Kabuli*	Ca_Kabuli_Chr06	48255558	(C/T)
11165	CakSNP11165	*Kabuli*	Ca_Kabuli_Chr06	48255559	(A/G)
11166	CakSNP11166	*Kabuli*	Ca_Kabuli_Chr06	48255568	(A/G)
11167	CakSNP11167	*Kabuli*	Ca_Kabuli_Chr06	48255599	(C/A)
11168	CakSNP11168	*Kabuli*	Ca_Kabuli_Chr06	48314052	(T/A)
11169	CakSNP11169	*Kabuli*	Ca_Kabuli_Chr06	48314788	(T/C)
11170	CakSNP11170	*Kabuli*	Ca_Kabuli_Chr06	48315194	(C/A)
11171	CakSNP11171	*Kabuli*	Ca_Kabuli_Chr06	48315340	(C/A)

S.N.	SNP IDs	Cultivars	Chromosomes/scaffolds	Physical positions (bp)	SNPs
11172	CakSNP11172	*Kabuli*	Ca_Kabuli_Chr06	48315343	(G/T)
11173	CakSNP11173	*Kabuli*	Ca_Kabuli_Chr06	48810611	(A/T)
11174	CakSNP11174	*Kabuli*	Ca_Kabuli_Chr06	48810608	(C/T)
11175	CakSNP11175	*Kabuli*	Ca_Kabuli_Chr06	48864622	(T/C)
11176	CakSNP11176	*Kabuli*	Ca_Kabuli_Chr06	48864625	(G/A)
11177	CakSNP11177	*Kabuli*	Ca_Kabuli_Chr06	48864639	(A/C)
11178	CakSNP11178	*Kabuli*	Ca_Kabuli_Chr06	48864699	(T/C)
11179	CakSNP11179	*Kabuli*	Ca_Kabuli_Chr06	48869462	(C/T)
11180	CakSNP11180	*Kabuli*	Ca_Kabuli_Chr06	48869473	(C/A)
11181	CakSNP11181	*Kabuli*	Ca_Kabuli_Chr06	48869484	(G/A)
11182	CakSNP11182	*Kabuli*	Ca_Kabuli_Chr06	48869577	(C/T)
11183	CakSNP11183	*Kabuli*	Ca_Kabuli_Chr06	48869539	(A/G)
11184	CakSNP11184	*Kabuli*	Ca_Kabuli_Chr06	48869529	(T/C)
11185	CakSNP11185	*Kabuli*	Ca_Kabuli_Chr06	48869512	(G/C)
11186	CakSNP11186	*Kabuli*	Ca_Kabuli_Chr06	48940386	(A/C)
11187	CakSNP11187	*Kabuli*	Ca_Kabuli_Chr06	49044772	(A/C)
11188	CakSNP11188	*Kabuli*	Ca_Kabuli_Chr06	49044785	(C/T)
11189	CakSNP11189	*Kabuli*	Ca_Kabuli_Chr06	49044794	(T/A)
11190	CakSNP11190	*Kabuli*	Ca_Kabuli_Chr06	49044802	(T/C)
11191	CakSNP11191	*Kabuli*	Ca_Kabuli_Chr06	49044805	(A/G)
11192	CakSNP11192	*Kabuli*	Ca_Kabuli_Chr06	49044814	(G/T)
11193	CakSNP11193	*Kabuli*	Ca_Kabuli_Chr06	49044824	(C/T)
11194	CakSNP11194	*Kabuli*	Ca_Kabuli_Chr06	49044839	(T/A)
11195	CakSNP11195	*Kabuli*	Ca_Kabuli_Chr06	49044850	(A/C)
11196	CakSNP11196	*Kabuli*	Ca_Kabuli_Chr06	49044847	(G/A)
11197	CakSNP11197	*Kabuli*	Ca_Kabuli_Chr06	49044842	(G/A)
11198	CakSNP11198	*Kabuli*	Ca_Kabuli_Chr06	49044838	(C/A)
11199	CakSNP11199	*Kabuli*	Ca_Kabuli_Chr06	49044835	(C/A)
11200	CakSNP11200	*Kabuli*	Ca_Kabuli_Chr06	49044826	(G/A)
11201	CakSNP11201	*Kabuli*	Ca_Kabuli_Chr06	49044821	(C/G)
11202	CakSNP11202	*Kabuli*	Ca_Kabuli_Chr06	49044810	(G/A)
11203	CakSNP11203	*Kabuli*	Ca_Kabuli_Chr06	49044808	(C/T)
11204	CakSNP11204	*Kabuli*	Ca_Kabuli_Chr06	49044788	(T/A)
11205	CakSNP11205	*Kabuli*	Ca_Kabuli_Chr06	49044780	(G/A)
11206	CakSNP11206	*Kabuli*	Ca_Kabuli_Chr06	49044915	(A/G)
11207	CakSNP11207	*Kabuli*	Ca_Kabuli_Chr06	49044928	(C/A)
11208	CakSNP11208	*Kabuli*	Ca_Kabuli_Chr06	49044936	(C/T)
11209	CakSNP11209	*Kabuli*	Ca_Kabuli_Chr06	49044937	(A/G)

S.N.	SNP IDs	Cultivars	Chromosomes/scaffolds	Physical positions (bp)	SNPs
11210	CakSNP11210	*Kabuli*	Ca_Kabuli_Chr06	49044944	(A/G)
11211	CakSNP11211	*Kabuli*	Ca_Kabuli_Chr06	49101829	(G/C)
11212	CakSNP11212	*Kabuli*	Ca_Kabuli_Chr06	49167341	(T/G)
11213	CakSNP11213	*Kabuli*	Ca_Kabuli_Chr06	49222291	(G/A)
11214	CakSNP11214	*Kabuli*	Ca_Kabuli_Chr06	49222290	(C/T)
11215	CakSNP11215	*Kabuli*	Ca_Kabuli_Chr06	49222275	(A/C)
11216	CakSNP11216	*Kabuli*	Ca_Kabuli_Chr06	49222268	(T/A)
11217	CakSNP11217	*Kabuli*	Ca_Kabuli_Chr06	49222251	(G/A)
11218	CakSNP11218	*Kabuli*	Ca_Kabuli_Chr06	49222235	(G/C)
11219	CakSNP11219	*Kabuli*	Ca_Kabuli_Chr06	49377275	(G/A)
11220	CakSNP11220	*Kabuli*	Ca_Kabuli_Chr06	49403430	(G/C)
11221	CakSNP11221	*Kabuli*	Ca_Kabuli_Chr06	49406604	(G/A)
11222	CakSNP11222	*Kabuli*	Ca_Kabuli_Chr06	49406599	(C/A)
11223	CakSNP11223	*Kabuli*	Ca_Kabuli_Chr06	49466066	(A/G)
11224	CakSNP11224	*Kabuli*	Ca_Kabuli_Chr06	49475716	(G/A)
11225	CakSNP11225	*Kabuli*	Ca_Kabuli_Chr06	49475715	(A/G)
11226	CakSNP11226	*Kabuli*	Ca_Kabuli_Chr06	49524179	(A/T)
11227	CakSNP11227	*Kabuli*	Ca_Kabuli_Chr06	49565934	(C/T)
11228	CakSNP11228	*Kabuli*	Ca_Kabuli_Chr06	49664132	(C/G)
11229	CakSNP11229	*Kabuli*	Ca_Kabuli_Chr06	49724448	(C/T)
11230	CakSNP11230	*Kabuli*	Ca_Kabuli_Chr06	49724515	(C/T)
11231	CakSNP11231	*Kabuli*	Ca_Kabuli_Chr06	49726734	(C/G)
11232	CakSNP11232	*Kabuli*	Ca_Kabuli_Chr06	49726732	(T/C)
11233	CakSNP11233	*Kabuli*	Ca_Kabuli_Chr06	49726729	(G/A)
11234	CakSNP11234	*Kabuli*	Ca_Kabuli_Chr06	49726711	(C/T)
11235	CakSNP11235	*Kabuli*	Ca_Kabuli_Chr06	49726723	(G/A)
11236	CakSNP11236	*Kabuli*	Ca_Kabuli_Chr06	49830299	(A/G)
11237	CakSNP11237	*Kabuli*	Ca_Kabuli_Chr06	49830384	(G/A)
11238	CakSNP11238	*Kabuli*	Ca_Kabuli_Chr06	49833934	(C/T)
11239	CakSNP11239	*Kabuli*	Ca_Kabuli_Chr06	49843667	(G/A)
11240	CakSNP11240	*Kabuli*	Ca_Kabuli_Chr06	49846257	(A/T)
11241	CakSNP11241	*Kabuli*	Ca_Kabuli_Chr06	49846303	(T/G)
11242	CakSNP11242	*Kabuli*	Ca_Kabuli_Chr06	49931821	(C/T)
11243	CakSNP11243	*Kabuli*	Ca_Kabuli_Chr06	49931879	(T/C)
11244	CakSNP11244	*Kabuli*	Ca_Kabuli_Chr06	49942397	(A/G)
11245	CakSNP11245	*Kabuli*	Ca_Kabuli_Chr06	50086781	(A/G)
11246	CakSNP11246	*Kabuli*	Ca_Kabuli_Chr06	50152053	(C/T)
11247	CakSNP11247	*Kabuli*	Ca_Kabuli_Chr06	50152129	(C/T)

S.N.	SNP IDs	Cultivars	Chromosomes/scaffolds	Physical positions (bp)	SNPs
11248	CakSNP11248	*Kabuli*	Ca_Kabuli_Chr06	50152124	(C/T)
11249	CakSNP11249	*Kabuli*	Ca_Kabuli_Chr06	50152119	(T/C)
11250	CakSNP11250	*Kabuli*	Ca_Kabuli_Chr06	50152083	(C/T)
11251	CakSNP11251	*Kabuli*	Ca_Kabuli_Chr06	50217734	(G/A)
11252	CakSNP11252	*Kabuli*	Ca_Kabuli_Chr06	50356239	(A/G)
11253	CakSNP11253	*Kabuli*	Ca_Kabuli_Chr06	50356229	(C/T)
11254	CakSNP11254	*Kabuli*	Ca_Kabuli_Chr06	50356222	(C/A)
11255	CakSNP11255	*Kabuli*	Ca_Kabuli_Chr06	50356217	(T/C)
11256	CakSNP11256	*Kabuli*	Ca_Kabuli_Chr06	50356206	(G/T)
11257	CakSNP11257	*Kabuli*	Ca_Kabuli_Chr06	50356201	(T/C)
11258	CakSNP11258	*Kabuli*	Ca_Kabuli_Chr06	50382452	(C/T)
11259	CakSNP11259	*Kabuli*	Ca_Kabuli_Chr06	50384101	(A/C)
11260	CakSNP11260	*Kabuli*	Ca_Kabuli_Chr06	50390752	(A/G)
11261	CakSNP11261	*Kabuli*	Ca_Kabuli_Chr06	50476721	(T/C)
11262	CakSNP11262	*Kabuli*	Ca_Kabuli_Chr06	50476775	(G/A)
11263	CakSNP11263	*Kabuli*	Ca_Kabuli_Chr06	50477672	(A/C)
11264	CakSNP11264	*Kabuli*	Ca_Kabuli_Chr06	50581295	(T/G)
11265	CakSNP11265	*Kabuli*	Ca_Kabuli_Chr06	50617926	(G/C)
11266	CakSNP11266	*Kabuli*	Ca_Kabuli_Chr06	50617932	(G/A)
11267	CakSNP11267	*Kabuli*	Ca_Kabuli_Chr06	50723595	(T/C)
11268	CakSNP11268	*Kabuli*	Ca_Kabuli_Chr06	50723630	(C/T)
11269	CakSNP11269	*Kabuli*	Ca_Kabuli_Chr06	50723916	(T/C)
11270	CakSNP11270	*Kabuli*	Ca_Kabuli_Chr06	50723950	(A/G)
11271	CakSNP11271	*Kabuli*	Ca_Kabuli_Chr06	50723980	(A/G)
11272	CakSNP11272	*Kabuli*	Ca_Kabuli_Chr06	50723988	(C/A)
11273	CakSNP11273	*Kabuli*	Ca_Kabuli_Chr06	51157961	(C/T)
11274	CakSNP11274	*Kabuli*	Ca_Kabuli_Chr06	51157939	(A/C)
11275	CakSNP11275	*Kabuli*	Ca_Kabuli_Chr06	51162065	(T/C)
11276	CakSNP11276	*Kabuli*	Ca_Kabuli_Chr06	51281235	(G/A)
11277	CakSNP11277	*Kabuli*	Ca_Kabuli_Chr06	51281178	(C/G)
11278	CakSNP11278	*Kabuli*	Ca_Kabuli_Chr06	51345460	(G/C)
11279	CakSNP11279	*Kabuli*	Ca_Kabuli_Chr06	51392609	(T/C)
11280	CakSNP11280	*Kabuli*	Ca_Kabuli_Chr06	51471573	(T/C)
11281	CakSNP11281	*Kabuli*	Ca_Kabuli_Chr06	52007211	(T/C)
11282	CakSNP11282	*Kabuli*	Ca_Kabuli_Chr06	52007217	(G/A)
11283	CakSNP11283	*Kabuli*	Ca_Kabuli_Chr06	52007471	(C/T)
11284	CakSNP11284	*Kabuli*	Ca_Kabuli_Chr06	52007464	(C/G)
11285	CakSNP11285	*Kabuli*	Ca_Kabuli_Chr06	52007440	(C/T)

S.N.	SNP IDs	Cultivars	Chromosomes/scaffolds	Physical positions (bp)	SNPs
11286	CakSNP11286	*Kabuli*	Ca_Kabuli_Chr06	52033279	(G/A)
11287	CakSNP11287	*Kabuli*	Ca_Kabuli_Chr06	52033244	(A/T)
11288	CakSNP11288	*Kabuli*	Ca_Kabuli_Chr06	52059750	(A/G)
11289	CakSNP11289	*Kabuli*	Ca_Kabuli_Chr06	52065393	(C/A)
11290	CakSNP11290	*Kabuli*	Ca_Kabuli_Chr06	52065527	(T/A)
11291	CakSNP11291	*Kabuli*	Ca_Kabuli_Chr06	52164571	(C/A)
11292	CakSNP11292	*Kabuli*	Ca_Kabuli_Chr06	52227277	(T/C)
11293	CakSNP11293	*Kabuli*	Ca_Kabuli_Chr06	52304094	(C/T)
11294	CakSNP11294	*Kabuli*	Ca_Kabuli_Chr06	52304112	(A/G)
11295	CakSNP11295	*Kabuli*	Ca_Kabuli_Chr06	52363121	(C/T)
11296	CakSNP11296	*Kabuli*	Ca_Kabuli_Chr06	52416366	(T/A)
11297	CakSNP11297	*Kabuli*	Ca_Kabuli_Chr06	52417684	(C/T)
11298	CakSNP11298	*Kabuli*	Ca_Kabuli_Chr06	52417717	(T/G)
11299	CakSNP11299	*Kabuli*	Ca_Kabuli_Chr06	52417720	(A/T)
11300	CakSNP11300	*Kabuli*	Ca_Kabuli_Chr06	52417736	(T/A)
11301	CakSNP11301	*Kabuli*	Ca_Kabuli_Chr06	52417747	(G/T)
11302	CakSNP11302	*Kabuli*	Ca_Kabuli_Chr06	52567474	(A/C)
11303	CakSNP11303	*Kabuli*	Ca_Kabuli_Chr06	52596602	(T/A)
11304	CakSNP11304	*Kabuli*	Ca_Kabuli_Chr06	52596601	(A/T)
11305	CakSNP11305	*Kabuli*	Ca_Kabuli_Chr06	52858359	(A/G)
11306	CakSNP11306	*Kabuli*	Ca_Kabuli_Chr06	52993231	(A/G)
11307	CakSNP11307	*Kabuli*	Ca_Kabuli_Chr06	53068176	(C/T)
11308	CakSNP11308	*Kabuli*	Ca_Kabuli_Chr06	53068201	(G/T)
11309	CakSNP11309	*Kabuli*	Ca_Kabuli_Chr06	53097833	(G/C)
11310	CakSNP11310	*Kabuli*	Ca_Kabuli_Chr06	53097803	(A/G)
11311	CakSNP11311	*Kabuli*	Ca_Kabuli_Chr06	53097766	(G/A)
11312	CakSNP11312	*Kabuli*	Ca_Kabuli_Chr06	53124182	(G/T)
11313	CakSNP11313	*Kabuli*	Ca_Kabuli_Chr06	53168715	(T/G)
11314	CakSNP11314	*Kabuli*	Ca_Kabuli_Chr06	53348993	(C/A)
11315	CakSNP11315	*Kabuli*	Ca_Kabuli_Chr06	53349034	(G/A)
11316	CakSNP11316	*Kabuli*	Ca_Kabuli_Chr06	53390065	(A/G)
11317	CakSNP11317	*Kabuli*	Ca_Kabuli_Chr06	53461929	(C/A)
11318	CakSNP11318	*Kabuli*	Ca_Kabuli_Chr06	53461883	(A/G)
11319	CakSNP11319	*Kabuli*	Ca_Kabuli_Chr06	53541031	(G/A)
11320	CakSNP11320	*Kabuli*	Ca_Kabuli_Chr06	53541094	(C/G)
11321	CakSNP11321	*Kabuli*	Ca_Kabuli_Chr06	53541111	(T/G)
11322	CakSNP11322	*Kabuli*	Ca_Kabuli_Chr06	53551878	(C/A)
11323	CakSNP11323	*Kabuli*	Ca_Kabuli_Chr06	53558430	(A/C)

S.N.	SNP IDs	Cultivars	Chromosomes/scaffolds	Physical positions (bp)	SNPs
11324	CakSNP11324	*Kabuli*	Ca_Kabuli_Chr06	53566496	(G/A)
11325	CakSNP11325	*Kabuli*	Ca_Kabuli_Chr06	53566506	(C/T)
11326	CakSNP11326	*Kabuli*	Ca_Kabuli_Chr06	53625264	(A/C)
11327	CakSNP11327	*Kabuli*	Ca_Kabuli_Chr06	53683753	(T/C)
11328	CakSNP11328	*Kabuli*	Ca_Kabuli_Chr06	53683891	(C/A)
11329	CakSNP11329	*Kabuli*	Ca_Kabuli_Chr06	53683885	(C/T)
11330	CakSNP11330	*Kabuli*	Ca_Kabuli_Chr06	53683879	(C/T)
11331	CakSNP11331	*Kabuli*	Ca_Kabuli_Chr06	53683876	(C/T)
11332	CakSNP11332	*Kabuli*	Ca_Kabuli_Chr06	53692784	(G/A)
11333	CakSNP11333	*Kabuli*	Ca_Kabuli_Chr06	53769044	(A/C)
11334	CakSNP11334	*Kabuli*	Ca_Kabuli_Chr06	53769160	(C/T)
11335	CakSNP11335	*Kabuli*	Ca_Kabuli_Chr06	53769240	(T/A)
11336	CakSNP11336	*Kabuli*	Ca_Kabuli_Chr06	53769241	(A/T)
11337	CakSNP11337	*Kabuli*	Ca_Kabuli_Chr06	54091997	(A/C)
11338	CakSNP11338	*Kabuli*	Ca_Kabuli_Chr06	54151902	(T/G)
11339	CakSNP11339	*Kabuli*	Ca_Kabuli_Chr06	54151966	(G/C)
11340	CakSNP11340	*Kabuli*	Ca_Kabuli_Chr06	54151969	(T/G)
11341	CakSNP11341	*Kabuli*	Ca_Kabuli_Chr06	54203356	(G/A)
11342	CakSNP11342	*Kabuli*	Ca_Kabuli_Chr06	54203400	(C/T)
11343	CakSNP11343	*Kabuli*	Ca_Kabuli_Chr06	54226451	(A/T)
11344	CakSNP11344	*Kabuli*	Ca_Kabuli_Chr06	54226432	(A/C)
11345	CakSNP11345	*Kabuli*	Ca_Kabuli_Chr06	54229449	(G/T)
11346	CakSNP11346	*Kabuli*	Ca_Kabuli_Chr06	54237764	(A/C)
11347	CakSNP11347	*Kabuli*	Ca_Kabuli_Chr06	54237815	(C/T)
11348	CakSNP11348	*Kabuli*	Ca_Kabuli_Chr06	54250702	(C/T)
11349	CakSNP11349	*Kabuli*	Ca_Kabuli_Chr06	54368267	(G/A)
11350	CakSNP11350	*Kabuli*	Ca_Kabuli_Chr06	54869636	(C/T)
11351	CakSNP11351	*Kabuli*	Ca_Kabuli_Chr06	54869666	(G/A)
11352	CakSNP11352	*Kabuli*	Ca_Kabuli_Chr06	54997337	(A/T)
11353	CakSNP11353	*Kabuli*	Ca_Kabuli_Chr06	55024071	(C/T)
11354	CakSNP11354	*Kabuli*	Ca_Kabuli_Chr06	55024091	(G/A)
11355	CakSNP11355	*Kabuli*	Ca_Kabuli_Chr06	55024146	(C/T)
11356	CakSNP11356	*Kabuli*	Ca_Kabuli_Chr06	55024140	(G/A)
11357	CakSNP11357	*Kabuli*	Ca_Kabuli_Chr06	55024130	(T/A)
11358	CakSNP11358	*Kabuli*	Ca_Kabuli_Chr06	55024112	(C/G)
11359	CakSNP11359	*Kabuli*	Ca_Kabuli_Chr06	55024093	(G/C)
11360	CakSNP11360	*Kabuli*	Ca_Kabuli_Chr06	55024108	(C/T)
11361	CakSNP11361	*Kabuli*	Ca_Kabuli_Chr06	55024136	(C/T)

S.N.	SNP IDs	Cultivars	Chromosomes/scaffolds	Physical positions (bp)	SNPs
11362	CakSNP11362	*Kabuli*	Ca_Kabuli_Chr06	55024159	(T/A)
11363	CakSNP11363	*Kabuli*	Ca_Kabuli_Chr06	55024198	(T/G)
11364	CakSNP11364	*Kabuli*	Ca_Kabuli_Chr06	55028980	(G/A)
11365	CakSNP11365	*Kabuli*	Ca_Kabuli_Chr06	55074979	(T/A)
11366	CakSNP11366	*Kabuli*	Ca_Kabuli_Chr06	55083311	(T/A)
11367	CakSNP11367	*Kabuli*	Ca_Kabuli_Chr06	55083721	(T/A)
11368	CakSNP11368	*Kabuli*	Ca_Kabuli_Chr06	55180211	(A/G)
11369	CakSNP11369	*Kabuli*	Ca_Kabuli_Chr06	55332515	(G/A)
11370	CakSNP11370	*Kabuli*	Ca_Kabuli_Chr06	55914455	(T/C)
11371	CakSNP11371	*Kabuli*	Ca_Kabuli_Chr06	55914451	(A/G)
11372	CakSNP11372	*Kabuli*	Ca_Kabuli_Chr06	55914443	(T/C)
11373	CakSNP11373	*Kabuli*	Ca_Kabuli_Chr06	55914434	(C/T)
11374	CakSNP11374	*Kabuli*	Ca_Kabuli_Chr06	55914431	(G/A)
11375	CakSNP11375	*Kabuli*	Ca_Kabuli_Chr06	55922843	(T/A)
11376	CakSNP11376	*Kabuli*	Ca_Kabuli_Chr06	55922850	(C/T)
11377	CakSNP11377	*Kabuli*	Ca_Kabuli_Chr06	55942105	(A/G)
11378	CakSNP11378	*Kabuli*	Ca_Kabuli_Chr06	55953320	(T/A)
11379	CakSNP11379	*Kabuli*	Ca_Kabuli_Chr06	55953327	(C/T)
11380	CakSNP11380	*Kabuli*	Ca_Kabuli_Chr06	56025902	(A/G)
11381	CakSNP11381	*Kabuli*	Ca_Kabuli_Chr06	56025888	(G/A)
11382	CakSNP11382	*Kabuli*	Ca_Kabuli_Chr06	56025884	(A/G)
11383	CakSNP11383	*Kabuli*	Ca_Kabuli_Chr06	56029491	(T/A)
11384	CakSNP11384	*Kabuli*	Ca_Kabuli_Chr06	56029544	(A/T)
11385	CakSNP11385	*Kabuli*	Ca_Kabuli_Chr06	56029549	(C/T)
11386	CakSNP11386	*Kabuli*	Ca_Kabuli_Chr06	56137878	(T/C)
11387	CakSNP11387	*Kabuli*	Ca_Kabuli_Chr06	56553930	(G/A)
11388	CakSNP11388	*Kabuli*	Ca_Kabuli_Chr06	56564249	(G/C)
11389	CakSNP11389	*Kabuli*	Ca_Kabuli_Chr06	56587094	(C/G)
11390	CakSNP11390	*Kabuli*	Ca_Kabuli_Chr06	56593148	(A/G)
11391	CakSNP11391	*Kabuli*	Ca_Kabuli_Chr06	56593121	(T/G)
11392	CakSNP11392	*Kabuli*	Ca_Kabuli_Chr06	56624316	(A/T)
11393	CakSNP11393	*Kabuli*	Ca_Kabuli_Chr06	56624674	(T/C)
11394	CakSNP11394	*Kabuli*	Ca_Kabuli_Chr06	56624718	(C/A)
11395	CakSNP11395	*Kabuli*	Ca_Kabuli_Chr06	56694898	(T/C)
11396	CakSNP11396	*Kabuli*	Ca_Kabuli_Chr06	56765524	(C/A)
11397	CakSNP11397	*Kabuli*	Ca_Kabuli_Chr06	56793077	(G/A)
11398	CakSNP11398	*Kabuli*	Ca_Kabuli_Chr06	56793037	(A/T)
11399	CakSNP11399	*Kabuli*	Ca_Kabuli_Chr06	56793021	(G/T)

S.N.	SNP IDs	Cultivars	Chromosomes/scaffolds	Physical positions (bp)	SNPs
11400	CakSNP11400	*Kabuli*	Ca_Kabuli_Chr06	56793122	(C/T)
11401	CakSNP11401	*Kabuli*	Ca_Kabuli_Chr06	56822084	(G/T)
11402	CakSNP11402	*Kabuli*	Ca_Kabuli_Chr06	56822060	(G/A)
11403	CakSNP11403	*Kabuli*	Ca_Kabuli_Chr06	56840030	(A/C)
11404	CakSNP11404	*Kabuli*	Ca_Kabuli_Chr06	56840084	(A/T)
11405	CakSNP11405	*Kabuli*	Ca_Kabuli_Chr06	56840063	(T/A)
11406	CakSNP11406	*Kabuli*	Ca_Kabuli_Chr06	56840038	(A/T)
11407	CakSNP11407	*Kabuli*	Ca_Kabuli_Chr06	56854753	(T/C)
11408	CakSNP11408	*Kabuli*	Ca_Kabuli_Chr06	56946415	(A/G)
11409	CakSNP11409	*Kabuli*	Ca_Kabuli_Chr06	56946431	(T/C)
11410	CakSNP11410	*Kabuli*	Ca_Kabuli_Chr06	56950487	(G/T)
11411	CakSNP11411	*Kabuli*	Ca_Kabuli_Chr06	56980313	(T/C)
11412	CakSNP11412	*Kabuli*	Ca_Kabuli_Chr06	56980286	(T/C)
11413	CakSNP11413	*Kabuli*	Ca_Kabuli_Chr06	56980321	(T/C)
11414	CakSNP11414	*Kabuli*	Ca_Kabuli_Chr06	57147008	(A/G)
11415	CakSNP11415	*Kabuli*	Ca_Kabuli_Chr06	57198837	(G/A)
11416	CakSNP11416	*Kabuli*	Ca_Kabuli_Chr06	57259982	(C/A)
11417	CakSNP11417	*Kabuli*	Ca_Kabuli_Chr06	57260020	(A/G)
11418	CakSNP11418	*Kabuli*	Ca_Kabuli_Chr06	57260022	(T/C)
11419	CakSNP11419	*Kabuli*	Ca_Kabuli_Chr06	57272604	(A/G)
11420	CakSNP11420	*Kabuli*	Ca_Kabuli_Chr06	57272607	(G/A)
11421	CakSNP11421	*Kabuli*	Ca_Kabuli_Chr06	57272608	(C/A)
11422	CakSNP11422	*Kabuli*	Ca_Kabuli_Chr06	57277430	(A/G)
11423	CakSNP11423	*Kabuli*	Ca_Kabuli_Chr06	57277440	(C/T)
11424	CakSNP11424	*Kabuli*	Ca_Kabuli_Chr06	57344963	(G/T)
11425	CakSNP11425	*Kabuli*	Ca_Kabuli_Chr06	57382220	(G/A)
11426	CakSNP11426	*Kabuli*	Ca_Kabuli_Chr06	57433674	(T/A)
11427	CakSNP11427	*Kabuli*	Ca_Kabuli_Chr06	57436112	(A/T)
11428	CakSNP11428	*Kabuli*	Ca_Kabuli_Chr06	57436119	(T/C)
11429	CakSNP11429	*Kabuli*	Ca_Kabuli_Chr06	57436149	(T/A)
11430	CakSNP11430	*Kabuli*	Ca_Kabuli_Chr06	57436320	(T/G)
11431	CakSNP11431	*Kabuli*	Ca_Kabuli_Chr06	57437904	(A/G)
11432	CakSNP11432	*Kabuli*	Ca_Kabuli_Chr06	57449892	(A/G)
11433	CakSNP11433	*Kabuli*	Ca_Kabuli_Chr06	57475853	(G/T)
11434	CakSNP11434	*Kabuli*	Ca_Kabuli_Chr06	57475892	(A/G)
11435	CakSNP11435	*Kabuli*	Ca_Kabuli_Chr06	57475982	(A/G)
11436	CakSNP11436	*Kabuli*	Ca_Kabuli_Chr06	57533087	(G/A)
11437	CakSNP11437	*Kabuli*	Ca_Kabuli_Chr06	57579770	(G/T)

S.N.	SNP IDs	Cultivars	Chromosomes/scaffolds	Physical positions (bp)	SNPs
11438	CakSNP11438	*Kabuli*	Ca_Kabuli_Chr06	57579768	(C/T)
11439	CakSNP11439	*Kabuli*	Ca_Kabuli_Chr06	57580569	(C/T)
11440	CakSNP11440	*Kabuli*	Ca_Kabuli_Chr06	57603257	(T/A)
11441	CakSNP11441	*Kabuli*	Ca_Kabuli_Chr06	57625134	(G/A)
11442	CakSNP11442	*Kabuli*	Ca_Kabuli_Chr06	57625320	(G/A)
11443	CakSNP11443	*Kabuli*	Ca_Kabuli_Chr06	57632486	(C/T)
11444	CakSNP11444	*Kabuli*	Ca_Kabuli_Chr06	57634387	(G/T)
11445	CakSNP11445	*Kabuli*	Ca_Kabuli_Chr06	57634677	(G/A)
11446	CakSNP11446	*Kabuli*	Ca_Kabuli_Chr06	57637091	(G/A)
11447	CakSNP11447	*Kabuli*	Ca_Kabuli_Chr06	57637083	(T/G)
11448	CakSNP11448	*Kabuli*	Ca_Kabuli_Chr06	57637075	(C/T)
11449	CakSNP11449	*Kabuli*	Ca_Kabuli_Chr06	57662858	(T/A)
11450	CakSNP11450	*Kabuli*	Ca_Kabuli_Chr06	57667515	(A/T)
11451	CakSNP11451	*Kabuli*	Ca_Kabuli_Chr06	57688937	(C/T)
11452	CakSNP11452	*Kabuli*	Ca_Kabuli_Chr06	57704057	(C/T)
11453	CakSNP11453	*Kabuli*	Ca_Kabuli_Chr06	57704802	(T/C)
11454	CakSNP11454	*Kabuli*	Ca_Kabuli_Chr06	57705011	(G/A)
11455	CakSNP11455	*Kabuli*	Ca_Kabuli_Chr06	57707716	(C/T)
11456	CakSNP11456	*Kabuli*	Ca_Kabuli_Chr06	57707724	(A/C)
11457	CakSNP11457	*Kabuli*	Ca_Kabuli_Chr06	57707727	(T/G)
11458	CakSNP11458	*Kabuli*	Ca_Kabuli_Chr06	57707848	(A/G)
11459	CakSNP11459	*Kabuli*	Ca_Kabuli_Chr06	57707846	(G/A)
11460	CakSNP11460	*Kabuli*	Ca_Kabuli_Chr06	57707912	(G/C)
11461	CakSNP11461	*Kabuli*	Ca_Kabuli_Chr06	57708019	(A/G)
11462	CakSNP11462	*Kabuli*	Ca_Kabuli_Chr06	57708130	(T/C)
11463	CakSNP11463	*Kabuli*	Ca_Kabuli_Chr06	57708101	(T/G)
11464	CakSNP11464	*Kabuli*	Ca_Kabuli_Chr06	57708198	(C/T)
11465	CakSNP11465	*Kabuli*	Ca_Kabuli_Chr06	57710133	(C/G)
11466	CakSNP11466	*Kabuli*	Ca_Kabuli_Chr06	57720446	(T/C)
11467	CakSNP11467	*Kabuli*	Ca_Kabuli_Chr06	57723992	(A/C)
11468	CakSNP11468	*Kabuli*	Ca_Kabuli_Chr06	57725372	(A/C)
11469	CakSNP11469	*Kabuli*	Ca_Kabuli_Chr06	57746989	(C/T)
11470	CakSNP11470	*Kabuli*	Ca_Kabuli_Chr06	57747101	(C/T)
11471	CakSNP11471	*Kabuli*	Ca_Kabuli_Chr06	57748565	(A/G)
11472	CakSNP11472	*Kabuli*	Ca_Kabuli_Chr06	57748602	(A/T)
11473	CakSNP11473	*Kabuli*	Ca_Kabuli_Chr06	57753076	(T/C)
11474	CakSNP11474	*Kabuli*	Ca_Kabuli_Chr06	57756699	(G/A)
11475	CakSNP11475	*Kabuli*	Ca_Kabuli_Chr06	57760109	(T/G)

S.N.	SNP IDs	Cultivars	Chromosomes/scaffolds	Physical positions (bp)	SNPs
11476	CakSNP11476	*Kabuli*	Ca_Kabuli_Chr06	57760176	(C/T)
11477	CakSNP11477	*Kabuli*	Ca_Kabuli_Chr06	57760227	(C/T)
11478	CakSNP11478	*Kabuli*	Ca_Kabuli_Chr06	57760290	(A/T)
11479	CakSNP11479	*Kabuli*	Ca_Kabuli_Chr06	57760291	(G/T)
11480	CakSNP11480	*Kabuli*	Ca_Kabuli_Chr06	57760360	(A/G)
11481	CakSNP11481	*Kabuli*	Ca_Kabuli_Chr06	57761758	(G/A)
11482	CakSNP11482	*Kabuli*	Ca_Kabuli_Chr06	57761722	(T/C)
11483	CakSNP11483	*Kabuli*	Ca_Kabuli_Chr06	57766444	(C/T)
11484	CakSNP11484	*Kabuli*	Ca_Kabuli_Chr06	57766484	(T/A)
11485	CakSNP11485	*Kabuli*	Ca_Kabuli_Chr06	57766483	(C/A)
11486	CakSNP11486	*Kabuli*	Ca_Kabuli_Chr06	57766570	(C/T)
11487	CakSNP11487	*Kabuli*	Ca_Kabuli_Chr06	57766588	(G/A)
11488	CakSNP11488	*Kabuli*	Ca_Kabuli_Chr06	57766598	(G/A)
11489	CakSNP11489	*Kabuli*	Ca_Kabuli_Chr06	57768314	(T/C)
11490	CakSNP11490	*Kabuli*	Ca_Kabuli_Chr06	57801985	(A/C)
11491	CakSNP11491	*Kabuli*	Ca_Kabuli_Chr06	57831050	(G/T)
11492	CakSNP11492	*Kabuli*	Ca_Kabuli_Chr06	57831049	(C/T)
11493	CakSNP11493	*Kabuli*	Ca_Kabuli_Chr06	57831040	(T/C)
11494	CakSNP11494	*Kabuli*	Ca_Kabuli_Chr06	57835093	(T/A)
11495	CakSNP11495	*Kabuli*	Ca_Kabuli_Chr06	57835120	(G/A)
11496	CakSNP11496	*Kabuli*	Ca_Kabuli_Chr06	57835917	(G/C)
11497	CakSNP11497	*Kabuli*	Ca_Kabuli_Chr06	57835899	(C/T)
11498	CakSNP11498	*Kabuli*	Ca_Kabuli_Chr06	57836024	(G/A)
11499	CakSNP11499	*Kabuli*	Ca_Kabuli_Chr06	57836237	(A/G)
11500	CakSNP11500	*Kabuli*	Ca_Kabuli_Chr06	57836284	(G/A)
11501	CakSNP11501	*Kabuli*	Ca_Kabuli_Chr06	57836314	(T/A)
11502	CakSNP11502	*Kabuli*	Ca_Kabuli_Chr06	57836316	(G/A)
11503	CakSNP11503	*Kabuli*	Ca_Kabuli_Chr06	57870893	(C/A)
11504	CakSNP11504	*Kabuli*	Ca_Kabuli_Chr06	57877446	(C/A)
11505	CakSNP11505	*Kabuli*	Ca_Kabuli_Chr06	57882364	(C/T)
11506	CakSNP11506	*Kabuli*	Ca_Kabuli_Chr06	57894377	(A/G)
11507	CakSNP11507	*Kabuli*	Ca_Kabuli_Chr06	57894495	(C/G)
11508	CakSNP11508	*Kabuli*	Ca_Kabuli_Chr06	57894590	(G/C)
11509	CakSNP11509	*Kabuli*	Ca_Kabuli_Chr06	57894585	(T/G)
11510	CakSNP11510	*Kabuli*	Ca_Kabuli_Chr06	57904599	(T/C)
11511	CakSNP11511	*Kabuli*	Ca_Kabuli_Chr06	57921373	(C/T)
11512	CakSNP11512	*Kabuli*	Ca_Kabuli_Chr06	57943585	(A/T)
11513	CakSNP11513	*Kabuli*	Ca_Kabuli_Chr06	57943626	(G/A)

S.N.	SNP IDs	Cultivars	Chromosomes/scaffolds	Physical positions (bp)	SNPs
11514	CakSNP11514	*Kabuli*	Ca_Kabuli_Chr06	57943628	(A/C)
11515	CakSNP11515	*Kabuli*	Ca_Kabuli_Chr06	57944439	(C/T)
11516	CakSNP11516	*Kabuli*	Ca_Kabuli_Chr06	57968924	(A/T)
11517	CakSNP11517	*Kabuli*	Ca_Kabuli_Chr06	58036471	(C/T)
11518	CakSNP11518	*Kabuli*	Ca_Kabuli_Chr06	58174702	(T/C)
11519	CakSNP11519	*Kabuli*	Ca_Kabuli_Chr06	58187822	(T/G)
11520	CakSNP11520	*Kabuli*	Ca_Kabuli_Chr06	58187859	(T/A)
11521	CakSNP11521	*Kabuli*	Ca_Kabuli_Chr06	58187957	(A/G)
11522	CakSNP11522	*Kabuli*	Ca_Kabuli_Chr06	58191050	(T/C)
11523	CakSNP11523	*Kabuli*	Ca_Kabuli_Chr06	58224023	(G/T)
11524	CakSNP11524	*Kabuli*	Ca_Kabuli_Chr06	58226908	(T/G)
11525	CakSNP11525	*Kabuli*	Ca_Kabuli_Chr06	58227006	(T/C)
11526	CakSNP11526	*Kabuli*	Ca_Kabuli_Chr06	58237503	(A/T)
11527	CakSNP11527	*Kabuli*	Ca_Kabuli_Chr06	58238371	(T/C)
11528	CakSNP11528	*Kabuli*	Ca_Kabuli_Chr06	58238397	(A/G)
11529	CakSNP11529	*Kabuli*	Ca_Kabuli_Chr06	58243505	(G/A)
11530	CakSNP11530	*Kabuli*	Ca_Kabuli_Chr06	58326344	(A/T)
11531	CakSNP11531	*Kabuli*	Ca_Kabuli_Chr06	58380367	(T/C)
11532	CakSNP11532	*Kabuli*	Ca_Kabuli_Chr06	58380394	(G/A)
11533	CakSNP11533	*Kabuli*	Ca_Kabuli_Chr06	58380563	(G/A)
11534	CakSNP11534	*Kabuli*	Ca_Kabuli_Chr06	58381203	(G/C)
11535	CakSNP11535	*Kabuli*	Ca_Kabuli_Chr06	58381259	(A/G)
11536	CakSNP11536	*Kabuli*	Ca_Kabuli_Chr06	58434217	(C/G)
11537	CakSNP11537	*Kabuli*	Ca_Kabuli_Chr06	58452843	(A/C)
11538	CakSNP11538	*Kabuli*	Ca_Kabuli_Chr06	58569576	(G/T)
11539	CakSNP11539	*Kabuli*	Ca_Kabuli_Chr06	58569569	(T/C)
11540	CakSNP11540	*Kabuli*	Ca_Kabuli_Chr06	58571660	(T/C)
11541	CakSNP11541	*Kabuli*	Ca_Kabuli_Chr06	58596821	(T/G)
11542	CakSNP11542	*Kabuli*	Ca_Kabuli_Chr06	58596878	(C/A)
11543	CakSNP11543	*Kabuli*	Ca_Kabuli_Chr06	58596968	(T/C)
11544	CakSNP11544	*Kabuli*	Ca_Kabuli_Chr06	58596918	(A/G)
11545	CakSNP11545	*Kabuli*	Ca_Kabuli_Chr06	58600930	(C/A)
11546	CakSNP11546	*Kabuli*	Ca_Kabuli_Chr06	58600952	(A/G)
11547	CakSNP11547	*Kabuli*	Ca_Kabuli_Chr06	58600974	(A/G)
11548	CakSNP11548	*Kabuli*	Ca_Kabuli_Chr06	58623095	(A/G)
11549	CakSNP11549	*Kabuli*	Ca_Kabuli_Chr06	58623096	(A/T)
11550	CakSNP11550	*Kabuli*	Ca_Kabuli_Chr06	58623220	(T/C)
11551	CakSNP11551	*Kabuli*	Ca_Kabuli_Chr06	58624183	(G/A)

S.N.	SNP IDs	Cultivars	Chromosomes/scaffolds	Physical positions (bp)	SNPs
11552	CakSNP11552	*Kabuli*	Ca_Kabuli_Chr06	58624233	(C/A)
11553	CakSNP11553	*Kabuli*	Ca_Kabuli_Chr06	58624299	(A/G)
11554	CakSNP11554	*Kabuli*	Ca_Kabuli_Chr06	58624281	(A/G)
11555	CakSNP11555	*Kabuli*	Ca_Kabuli_Chr06	58624251	(C/T)
11556	CakSNP11556	*Kabuli*	Ca_Kabuli_Chr06	58650354	(A/G)
11557	CakSNP11557	*Kabuli*	Ca_Kabuli_Chr06	58650330	(G/A)
11558	CakSNP11558	*Kabuli*	Ca_Kabuli_Chr06	58650318	(A/C)
11559	CakSNP11559	*Kabuli*	Ca_Kabuli_Chr06	58650459	(A/T)
11560	CakSNP11560	*Kabuli*	Ca_Kabuli_Chr06	58650491	(A/G)
11561	CakSNP11561	*Kabuli*	Ca_Kabuli_Chr06	58652881	(A/G)
11562	CakSNP11562	*Kabuli*	Ca_Kabuli_Chr06	58672317	(A/G)
11563	CakSNP11563	*Kabuli*	Ca_Kabuli_Chr06	58675788	(T/C)
11564	CakSNP11564	*Kabuli*	Ca_Kabuli_Chr06	58675749	(T/C)
11565	CakSNP11565	*Kabuli*	Ca_Kabuli_Chr06	58676507	(A/G)
11566	CakSNP11566	*Kabuli*	Ca_Kabuli_Chr06	58676517	(A/C)
11567	CakSNP11567	*Kabuli*	Ca_Kabuli_Chr06	58682427	(G/T)
11568	CakSNP11568	*Kabuli*	Ca_Kabuli_Chr06	58682397	(G/A)
11569	CakSNP11569	*Kabuli*	Ca_Kabuli_Chr06	58682387	(T/A)
11570	CakSNP11570	*Kabuli*	Ca_Kabuli_Chr06	58730925	(T/C)
11571	CakSNP11571	*Kabuli*	Ca_Kabuli_Chr06	58730969	(C/A)
11572	CakSNP11572	*Kabuli*	Ca_Kabuli_Chr06	58730967	(T/G)
11573	CakSNP11573	*Kabuli*	Ca_Kabuli_Chr06	58732424	(G/T)
11574	CakSNP11574	*Kabuli*	Ca_Kabuli_Chr06	58732423	(C/T)
11575	CakSNP11575	*Kabuli*	Ca_Kabuli_Chr06	58732514	(G/A)
11576	CakSNP11576	*Kabuli*	Ca_Kabuli_Chr06	58732451	(T/C)
11577	CakSNP11577	*Kabuli*	Ca_Kabuli_Chr06	58732475	(G/A)
11578	CakSNP11578	*Kabuli*	Ca_Kabuli_Chr06	58745356	(A/G)
11579	CakSNP11579	*Kabuli*	Ca_Kabuli_Chr06	58745473	(A/G)
11580	CakSNP11580	*Kabuli*	Ca_Kabuli_Chr06	58745426	(T/C)
11581	CakSNP11581	*Kabuli*	Ca_Kabuli_Chr06	58745583	(C/T)
11582	CakSNP11582	*Kabuli*	Ca_Kabuli_Chr06	58761879	(G/A)
11583	CakSNP11583	*Kabuli*	Ca_Kabuli_Chr06	58779344	(A/G)
11584	CakSNP11584	*Kabuli*	Ca_Kabuli_Chr06	58779389	(T/G)
11585	CakSNP11585	*Kabuli*	Ca_Kabuli_Chr06	58820632	(A/C)
11586	CakSNP11586	*Kabuli*	Ca_Kabuli_Chr06	58844717	(T/C)
11587	CakSNP11587	*Kabuli*	Ca_Kabuli_Chr06	58844744	(A/T)
11588	CakSNP11588	*Kabuli*	Ca_Kabuli_Chr06	58844776	(C/G)
11589	CakSNP11589	*Kabuli*	Ca_Kabuli_Chr06	58861614	(G/A)

S.N.	SNP IDs	Cultivars	Chromosomes/scaffolds	Physical positions (bp)	SNPs
11590	CakSNP11590	*Kabuli*	Ca_Kabuli_Chr06	58878604	(C/G)
11591	CakSNP11591	*Kabuli*	Ca_Kabuli_Chr06	58879570	(T/C)
11592	CakSNP11592	*Kabuli*	Ca_Kabuli_Chr06	58903346	(G/A)
11593	CakSNP11593	*Kabuli*	Ca_Kabuli_Chr06	58903381	(G/A)
11594	CakSNP11594	*Kabuli*	Ca_Kabuli_Chr06	58903395	(A/G)
11595	CakSNP11595	*Kabuli*	Ca_Kabuli_Chr06	58903416	(T/G)
11596	CakSNP11596	*Kabuli*	Ca_Kabuli_Chr06	58903497	(G/C)
11597	CakSNP11597	*Kabuli*	Ca_Kabuli_Chr06	58903538	(G/A)
11598	CakSNP11598	*Kabuli*	Ca_Kabuli_Chr06	58908024	(G/A)
11599	CakSNP11599	*Kabuli*	Ca_Kabuli_Chr06	58908045	(A/G)
11600	CakSNP11600	*Kabuli*	Ca_Kabuli_Chr06	58908069	(G/A)
11601	CakSNP11601	*Kabuli*	Ca_Kabuli_Chr06	58911548	(T/C)
11602	CakSNP11602	*Kabuli*	Ca_Kabuli_Chr06	58951079	(T/C)
11603	CakSNP11603	*Kabuli*	Ca_Kabuli_Chr06	58951298	(A/G)
11604	CakSNP11604	*Kabuli*	Ca_Kabuli_Chr06	58951583	(C/A)
11605	CakSNP11605	*Kabuli*	Ca_Kabuli_Chr06	58953219	(A/G)
11606	CakSNP11606	*Kabuli*	Ca_Kabuli_Chr06	58994014	(C/T)
11607	CakSNP11607	*Kabuli*	Ca_Kabuli_Chr06	58994083	(A/G)
11608	CakSNP11608	*Kabuli*	Ca_Kabuli_Chr06	58994281	(G/A)
11609	CakSNP11609	*Kabuli*	Ca_Kabuli_Chr06	59001443	(C/T)
11610	CakSNP11610	*Kabuli*	Ca_Kabuli_Chr06	59003792	(A/C)
11611	CakSNP11611	*Kabuli*	Ca_Kabuli_Chr06	59064798	(G/T)
11612	CakSNP11612	*Kabuli*	Ca_Kabuli_Chr06	59064815	(C/T)
11613	CakSNP11613	*Kabuli*	Ca_Kabuli_Chr06	59081169	(C/T)
11614	CakSNP11614	*Kabuli*	Ca_Kabuli_Chr06	59107027	(A/T)
11615	CakSNP11615	*Kabuli*	Ca_Kabuli_Chr06	59107007	(G/T)
11616	CakSNP11616	*Kabuli*	Ca_Kabuli_Chr06	59135944	(A/C)
11617	CakSNP11617	*Kabuli*	Ca_Kabuli_Chr06	59194015	(C/G)
11618	CakSNP11618	*Kabuli*	Ca_Kabuli_Chr06	59193994	(G/T)
11619	CakSNP11619	*Kabuli*	Ca_Kabuli_Chr06	59194081	(T/A)
11620	CakSNP11620	*Kabuli*	Ca_Kabuli_Chr06	59202825	(T/G)
11621	CakSNP11621	*Kabuli*	Ca_Kabuli_Chr06	59219099	(T/C)
11622	CakSNP11622	*Kabuli*	Ca_Kabuli_Chr06	59222848	(C/T)
11623	CakSNP11623	*Kabuli*	Ca_Kabuli_Chr06	59296368	(C/T)
11624	CakSNP11624	*Kabuli*	Ca_Kabuli_Chr06	59296460	(G/A)
11625	CakSNP11625	*Kabuli*	Ca_Kabuli_Chr06	59407761	(G/A)
11626	CakSNP11626	*Kabuli*	Ca_Kabuli_Chr06	59417984	(G/A)
11627	CakSNP11627	*Kabuli*	Ca_Kabuli_Chr07	16760	(T/G)

S.N.	SNP IDs	Cultivars	Chromosomes/scaffolds	Physical positions (bp)	SNPs
11628	CakSNP11628	*Kabuli*	Ca_Kabuli_Chr07	16850	(C/T)
11629	CakSNP11629	*Kabuli*	Ca_Kabuli_Chr07	16880	(G/A)
11630	CakSNP11630	*Kabuli*	Ca_Kabuli_Chr07	16881	(A/G)
11631	CakSNP11631	*Kabuli*	Ca_Kabuli_Chr07	41841	(G/A)
11632	CakSNP11632	*Kabuli*	Ca_Kabuli_Chr07	55131	(T/C)
11633	CakSNP11633	*Kabuli*	Ca_Kabuli_Chr07	208824	(A/G)
11634	CakSNP11634	*Kabuli*	Ca_Kabuli_Chr07	218346	(C/A)
11635	CakSNP11635	*Kabuli*	Ca_Kabuli_Chr07	218341	(C/G)
11636	CakSNP11636	*Kabuli*	Ca_Kabuli_Chr07	419301	(C/T)
11637	CakSNP11637	*Kabuli*	Ca_Kabuli_Chr07	419316	(A/G)
11638	CakSNP11638	*Kabuli*	Ca_Kabuli_Chr07	419334	(A/C)
11639	CakSNP11639	*Kabuli*	Ca_Kabuli_Chr07	419385	(T/G)
11640	CakSNP11640	*Kabuli*	Ca_Kabuli_Chr07	419328	(C/T)
11641	CakSNP11641	*Kabuli*	Ca_Kabuli_Chr07	419679	(C/T)
11642	CakSNP11642	*Kabuli*	Ca_Kabuli_Chr07	419638	(C/T)
11643	CakSNP11643	*Kabuli*	Ca_Kabuli_Chr07	419628	(A/C)
11644	CakSNP11644	*Kabuli*	Ca_Kabuli_Chr07	528521	(G/A)
11645	CakSNP11645	*Kabuli*	Ca_Kabuli_Chr07	601563	(T/A)
11646	CakSNP11646	*Kabuli*	Ca_Kabuli_Chr07	601551	(T/C)
11647	CakSNP11647	*Kabuli*	Ca_Kabuli_Chr07	601545	(C/T)
11648	CakSNP11648	*Kabuli*	Ca_Kabuli_Chr07	601538	(C/A)
11649	CakSNP11649	*Kabuli*	Ca_Kabuli_Chr07	601521	(G/A)
11650	CakSNP11650	*Kabuli*	Ca_Kabuli_Chr07	601516	(G/A)
11651	CakSNP11651	*Kabuli*	Ca_Kabuli_Chr07	601513	(C/G)
11652	CakSNP11652	*Kabuli*	Ca_Kabuli_Chr07	601502	(G/T)
11653	CakSNP11653	*Kabuli*	Ca_Kabuli_Chr07	601496	(C/T)
11654	CakSNP11654	*Kabuli*	Ca_Kabuli_Chr07	601586	(C/T)
11655	CakSNP11655	*Kabuli*	Ca_Kabuli_Chr07	601601	(C/T)
11656	CakSNP11656	*Kabuli*	Ca_Kabuli_Chr07	601612	(A/G)
11657	CakSNP11657	*Kabuli*	Ca_Kabuli_Chr07	601643	(G/T)
11658	CakSNP11658	*Kabuli*	Ca_Kabuli_Chr07	601622	(G/C)
11659	CakSNP11659	*Kabuli*	Ca_Kabuli_Chr07	601657	(C/T)
11660	CakSNP11660	*Kabuli*	Ca_Kabuli_Chr07	601719	(G/T)
11661	CakSNP11661	*Kabuli*	Ca_Kabuli_Chr07	601718	(G/C)
11662	CakSNP11662	*Kabuli*	Ca_Kabuli_Chr07	601714	(A/T)
11663	CakSNP11663	*Kabuli*	Ca_Kabuli_Chr07	601709	(A/C)
11664	CakSNP11664	*Kabuli*	Ca_Kabuli_Chr07	601683	(G/A)
11665	CakSNP11665	*Kabuli*	Ca_Kabuli_Chr07	601647	(C/T)

S.N.	SNP IDs	Cultivars	Chromosomes/scaffolds	Physical positions (bp)	SNPs
11666	CakSNP11666	*Kabuli*	Ca_Kabuli_Chr07	610737	(C/A)
11667	CakSNP11667	*Kabuli*	Ca_Kabuli_Chr07	610723	(G/T)
11668	CakSNP11668	*Kabuli*	Ca_Kabuli_Chr07	667597	(C/T)
11669	CakSNP11669	*Kabuli*	Ca_Kabuli_Chr07	674791	(A/T)
11670	CakSNP11670	*Kabuli*	Ca_Kabuli_Chr07	676785	(G/T)
11671	CakSNP11671	*Kabuli*	Ca_Kabuli_Chr07	676786	(G/A)
11672	CakSNP11672	*Kabuli*	Ca_Kabuli_Chr07	676790	(T/C)
11673	CakSNP11673	*Kabuli*	Ca_Kabuli_Chr07	683245	(T/C)
11674	CakSNP11674	*Kabuli*	Ca_Kabuli_Chr07	761710	(A/G)
11675	CakSNP11675	*Kabuli*	Ca_Kabuli_Chr07	767708	(G/A)
11676	CakSNP11676	*Kabuli*	Ca_Kabuli_Chr07	781377	(G/T)
11677	CakSNP11677	*Kabuli*	Ca_Kabuli_Chr07	781409	(A/G)
11678	CakSNP11678	*Kabuli*	Ca_Kabuli_Chr07	807095	(A/T)
11679	CakSNP11679	*Kabuli*	Ca_Kabuli_Chr07	807110	(T/C)
11680	CakSNP11680	*Kabuli*	Ca_Kabuli_Chr07	807133	(T/A)
11681	CakSNP11681	*Kabuli*	Ca_Kabuli_Chr07	815477	(A/T)
11682	CakSNP11682	*Kabuli*	Ca_Kabuli_Chr07	815526	(C/A)
11683	CakSNP11683	*Kabuli*	Ca_Kabuli_Chr07	838581	(C/A)
11684	CakSNP11684	*Kabuli*	Ca_Kabuli_Chr07	838651	(A/C)
11685	CakSNP11685	*Kabuli*	Ca_Kabuli_Chr07	841988	(T/C)
11686	CakSNP11686	*Kabuli*	Ca_Kabuli_Chr07	844248	(G/A)
11687	CakSNP11687	*Kabuli*	Ca_Kabuli_Chr07	845488	(A/G)
11688	CakSNP11688	*Kabuli*	Ca_Kabuli_Chr07	865007	(C/T)
11689	CakSNP11689	*Kabuli*	Ca_Kabuli_Chr07	865170	(C/T)
11690	CakSNP11690	*Kabuli*	Ca_Kabuli_Chr07	873207	(A/G)
11691	CakSNP11691	*Kabuli*	Ca_Kabuli_Chr07	888900	(G/A)
11692	CakSNP11692	*Kabuli*	Ca_Kabuli_Chr07	888987	(A/G)
11693	CakSNP11693	*Kabuli*	Ca_Kabuli_Chr07	975236	(T/A)
11694	CakSNP11694	*Kabuli*	Ca_Kabuli_Chr07	993031	(G/T)
11695	CakSNP11695	*Kabuli*	Ca_Kabuli_Chr07	994976	(G/A)
11696	CakSNP11696	*Kabuli*	Ca_Kabuli_Chr07	995691	(C/T)
11697	CakSNP11697	*Kabuli*	Ca_Kabuli_Chr07	995674	(T/C)
11698	CakSNP11698	*Kabuli*	Ca_Kabuli_Chr07	1022946	(C/T)
11699	CakSNP11699	*Kabuli*	Ca_Kabuli_Chr07	1045113	(A/C)
11700	CakSNP11700	*Kabuli*	Ca_Kabuli_Chr07	1045127	(T/A)
11701	CakSNP11701	*Kabuli*	Ca_Kabuli_Chr07	1096379	(C/T)
11702	CakSNP11702	*Kabuli*	Ca_Kabuli_Chr07	1153267	(A/C)
11703	CakSNP11703	*Kabuli*	Ca_Kabuli_Chr07	1174188	(C/T)

S.N.	SNP IDs	Cultivars	Chromosomes/scaffolds	Physical positions (bp)	SNPs
11704	CakSNP11704	*Kabuli*	Ca_Kabuli_Chr07	1293601	(C/G)
11705	CakSNP11705	*Kabuli*	Ca_Kabuli_Chr07	1350605	(C/T)
11706	CakSNP11706	*Kabuli*	Ca_Kabuli_Chr07	1350856	(G/T)
11707	CakSNP11707	*Kabuli*	Ca_Kabuli_Chr07	1350846	(A/T)
11708	CakSNP11708	*Kabuli*	Ca_Kabuli_Chr07	1350947	(T/G)
11709	CakSNP11709	*Kabuli*	Ca_Kabuli_Chr07	1414206	(C/A)
11710	CakSNP11710	*Kabuli*	Ca_Kabuli_Chr07	1505646	(G/A)
11711	CakSNP11711	*Kabuli*	Ca_Kabuli_Chr07	1509156	(G/A)
11712	CakSNP11712	*Kabuli*	Ca_Kabuli_Chr07	1511252	(C/T)
11713	CakSNP11713	*Kabuli*	Ca_Kabuli_Chr07	1585622	(T/C)
11714	CakSNP11714	*Kabuli*	Ca_Kabuli_Chr07	1635519	(G/A)
11715	CakSNP11715	*Kabuli*	Ca_Kabuli_Chr07	1635471	(G/T)
11716	CakSNP11716	*Kabuli*	Ca_Kabuli_Chr07	1702033	(G/T)
11717	CakSNP11717	*Kabuli*	Ca_Kabuli_Chr07	1702063	(A/G)
11718	CakSNP11718	*Kabuli*	Ca_Kabuli_Chr07	1702077	(G/C)
11719	CakSNP11719	*Kabuli*	Ca_Kabuli_Chr07	1704780	(T/G)
11720	CakSNP11720	*Kabuli*	Ca_Kabuli_Chr07	1704802	(A/G)
11721	CakSNP11721	*Kabuli*	Ca_Kabuli_Chr07	1704892	(A/G)
11722	CakSNP11722	*Kabuli*	Ca_Kabuli_Chr07	1704947	(C/T)
11723	CakSNP11723	*Kabuli*	Ca_Kabuli_Chr07	1705197	(A/C)
11724	CakSNP11724	*Kabuli*	Ca_Kabuli_Chr07	1707283	(G/T)
11725	CakSNP11725	*Kabuli*	Ca_Kabuli_Chr07	1707375	(C/G)
11726	CakSNP11726	*Kabuli*	Ca_Kabuli_Chr07	1739369	(C/A)
11727	CakSNP11727	*Kabuli*	Ca_Kabuli_Chr07	1787308	(T/A)
11728	CakSNP11728	*Kabuli*	Ca_Kabuli_Chr07	1787417	(G/A)
11729	CakSNP11729	*Kabuli*	Ca_Kabuli_Chr07	1946020	(G/A)
11730	CakSNP11730	*Kabuli*	Ca_Kabuli_Chr07	1963659	(T/A)
11731	CakSNP11731	*Kabuli*	Ca_Kabuli_Chr07	1963757	(A/G)
11732	CakSNP11732	*Kabuli*	Ca_Kabuli_Chr07	1991642	(G/T)
11733	CakSNP11733	*Kabuli*	Ca_Kabuli_Chr07	2012854	(T/A)
11734	CakSNP11734	*Kabuli*	Ca_Kabuli_Chr07	2103626	(C/T)
11735	CakSNP11735	*Kabuli*	Ca_Kabuli_Chr07	2106876	(C/A)
11736	CakSNP11736	*Kabuli*	Ca_Kabuli_Chr07	2106917	(A/T)
11737	CakSNP11737	*Kabuli*	Ca_Kabuli_Chr07	2146554	(G/A)
11738	CakSNP11738	*Kabuli*	Ca_Kabuli_Chr07	2167517	(A/C)
11739	CakSNP11739	*Kabuli*	Ca_Kabuli_Chr07	2167540	(G/C)
11740	CakSNP11740	*Kabuli*	Ca_Kabuli_Chr07	2211897	(G/A)
11741	CakSNP11741	*Kabuli*	Ca_Kabuli_Chr07	2219764	(C/T)

S.N.	SNP IDs	Cultivars	Chromosomes/scaffolds	Physical positions (bp)	SNPs
11742	CakSNP11742	*Kabuli*	Ca_Kabuli_Chr07	2230434	(A/G)
11743	CakSNP11743	*Kabuli*	Ca_Kabuli_Chr07	2262749	(C/G)
11744	CakSNP11744	*Kabuli*	Ca_Kabuli_Chr07	2262865	(A/G)
11745	CakSNP11745	*Kabuli*	Ca_Kabuli_Chr07	2262843	(T/A)
11746	CakSNP11746	*Kabuli*	Ca_Kabuli_Chr07	2361064	(T/G)
11747	CakSNP11747	*Kabuli*	Ca_Kabuli_Chr07	2387109	(C/T)
11748	CakSNP11748	*Kabuli*	Ca_Kabuli_Chr07	2391392	(G/A)
11749	CakSNP11749	*Kabuli*	Ca_Kabuli_Chr07	2401235	(T/C)
11750	CakSNP11750	*Kabuli*	Ca_Kabuli_Chr07	2493033	(A/G)
11751	CakSNP11751	*Kabuli*	Ca_Kabuli_Chr07	2538167	(C/G)
11752	CakSNP11752	*Kabuli*	Ca_Kabuli_Chr07	2541501	(A/G)
11753	CakSNP11753	*Kabuli*	Ca_Kabuli_Chr07	2541551	(G/A)
11754	CakSNP11754	*Kabuli*	Ca_Kabuli_Chr07	2541653	(C/G)
11755	CakSNP11755	*Kabuli*	Ca_Kabuli_Chr07	2545613	(T/C)
11756	CakSNP11756	*Kabuli*	Ca_Kabuli_Chr07	2552454	(G/T)
11757	CakSNP11757	*Kabuli*	Ca_Kabuli_Chr07	2603595	(C/T)
11758	CakSNP11758	*Kabuli*	Ca_Kabuli_Chr07	2603633	(C/T)
11759	CakSNP11759	*Kabuli*	Ca_Kabuli_Chr07	2603649	(T/C)
11760	CakSNP11760	*Kabuli*	Ca_Kabuli_Chr07	2609831	(C/A)
11761	CakSNP11761	*Kabuli*	Ca_Kabuli_Chr07	2618478	(C/T)
11762	CakSNP11762	*Kabuli*	Ca_Kabuli_Chr07	2654442	(T/G)
11763	CakSNP11763	*Kabuli*	Ca_Kabuli_Chr07	2654478	(C/T)
11764	CakSNP11764	*Kabuli*	Ca_Kabuli_Chr07	2684213	(A/G)
11765	CakSNP11765	*Kabuli*	Ca_Kabuli_Chr07	2734680	(A/G)
11766	CakSNP11766	*Kabuli*	Ca_Kabuli_Chr07	2734625	(C/T)
11767	CakSNP11767	*Kabuli*	Ca_Kabuli_Chr07	2745665	(A/C)
11768	CakSNP11768	*Kabuli*	Ca_Kabuli_Chr07	2745656	(A/C)
11769	CakSNP11769	*Kabuli*	Ca_Kabuli_Chr07	2757260	(C/T)
11770	CakSNP11770	*Kabuli*	Ca_Kabuli_Chr07	2757255	(G/C)
11771	CakSNP11771	*Kabuli*	Ca_Kabuli_Chr07	2757253	(T/G)
11772	CakSNP11772	*Kabuli*	Ca_Kabuli_Chr07	2762396	(G/A)
11773	CakSNP11773	*Kabuli*	Ca_Kabuli_Chr07	2762406	(C/T)
11774	CakSNP11774	*Kabuli*	Ca_Kabuli_Chr07	2762423	(G/A)
11775	CakSNP11775	*Kabuli*	Ca_Kabuli_Chr07	2832369	(C/T)
11776	CakSNP11776	*Kabuli*	Ca_Kabuli_Chr07	2857630	(T/G)
11777	CakSNP11777	*Kabuli*	Ca_Kabuli_Chr07	2857818	(T/A)
11778	CakSNP11778	*Kabuli*	Ca_Kabuli_Chr07	2857844	(C/T)
11779	CakSNP11779	*Kabuli*	Ca_Kabuli_Chr07	2909641	(A/G)

S.N.	SNP IDs	Cultivars	Chromosomes/scaffolds	Physical positions (bp)	SNPs
11780	CakSNP11780	*Kabuli*	Ca_Kabuli_Chr07	2915375	(C/G)
11781	CakSNP11781	*Kabuli*	Ca_Kabuli_Chr07	2932309	(C/G)
11782	CakSNP11782	*Kabuli*	Ca_Kabuli_Chr07	2932301	(C/A)
11783	CakSNP11783	*Kabuli*	Ca_Kabuli_Chr07	2932377	(C/T)
11784	CakSNP11784	*Kabuli*	Ca_Kabuli_Chr07	2956665	(C/T)
11785	CakSNP11785	*Kabuli*	Ca_Kabuli_Chr07	2956767	(A/G)
11786	CakSNP11786	*Kabuli*	Ca_Kabuli_Chr07	2969653	(A/G)
11787	CakSNP11787	*Kabuli*	Ca_Kabuli_Chr07	2997261	(A/G)
11788	CakSNP11788	*Kabuli*	Ca_Kabuli_Chr07	3023182	(T/C)
11789	CakSNP11789	*Kabuli*	Ca_Kabuli_Chr07	3023154	(T/C)
11790	CakSNP11790	*Kabuli*	Ca_Kabuli_Chr07	3070531	(A/C)
11791	CakSNP11791	*Kabuli*	Ca_Kabuli_Chr07	3090898	(G/A)
11792	CakSNP11792	*Kabuli*	Ca_Kabuli_Chr07	3093884	(T/A)
11793	CakSNP11793	*Kabuli*	Ca_Kabuli_Chr07	3093953	(T/G)
11794	CakSNP11794	*Kabuli*	Ca_Kabuli_Chr07	3094090	(G/A)
11795	CakSNP11795	*Kabuli*	Ca_Kabuli_Chr07	3094087	(A/G)
11796	CakSNP11796	*Kabuli*	Ca_Kabuli_Chr07	3107197	(C/T)
11797	CakSNP11797	*Kabuli*	Ca_Kabuli_Chr07	3107239	(T/A)
11798	CakSNP11798	*Kabuli*	Ca_Kabuli_Chr07	3109250	(T/C)
11799	CakSNP11799	*Kabuli*	Ca_Kabuli_Chr07	3109352	(T/C)
11800	CakSNP11800	*Kabuli*	Ca_Kabuli_Chr07	3110316	(A/G)
11801	CakSNP11801	*Kabuli*	Ca_Kabuli_Chr07	3130070	(A/G)
11802	CakSNP11802	*Kabuli*	Ca_Kabuli_Chr07	3130069	(A/G)
11803	CakSNP11803	*Kabuli*	Ca_Kabuli_Chr07	3143409	(C/T)
11804	CakSNP11804	*Kabuli*	Ca_Kabuli_Chr07	3186341	(C/T)
11805	CakSNP11805	*Kabuli*	Ca_Kabuli_Chr07	3186262	(T/G)
11806	CakSNP11806	*Kabuli*	Ca_Kabuli_Chr07	3198197	(C/A)
11807	CakSNP11807	*Kabuli*	Ca_Kabuli_Chr07	3198263	(T/C)
11808	CakSNP11808	*Kabuli*	Ca_Kabuli_Chr07	3217916	(T/G)
11809	CakSNP11809	*Kabuli*	Ca_Kabuli_Chr07	3236150	(A/C)
11810	CakSNP11810	*Kabuli*	Ca_Kabuli_Chr07	3265823	(A/G)
11811	CakSNP11811	*Kabuli*	Ca_Kabuli_Chr07	3297560	(G/A)
11812	CakSNP11812	*Kabuli*	Ca_Kabuli_Chr07	3297677	(G/T)
11813	CakSNP11813	*Kabuli*	Ca_Kabuli_Chr07	3315157	(A/G)
11814	CakSNP11814	*Kabuli*	Ca_Kabuli_Chr07	3351037	(G/T)
11815	CakSNP11815	*Kabuli*	Ca_Kabuli_Chr07	3353095	(C/T)
11816	CakSNP11816	*Kabuli*	Ca_Kabuli_Chr07	3355627	(A/G)
11817	CakSNP11817	*Kabuli*	Ca_Kabuli_Chr07	3355763	(A/G)

S.N.	SNP IDs	Cultivars	Chromosomes/scaffolds	Physical positions (bp)	SNPs
11818	CakSNP11818	*Kabuli*	Ca_Kabuli_Chr07	3356399	(G/A)
11819	CakSNP11819	*Kabuli*	Ca_Kabuli_Chr07	3357452	(G/A)
11820	CakSNP11820	*Kabuli*	Ca_Kabuli_Chr07	3430492	(A/T)
11821	CakSNP11821	*Kabuli*	Ca_Kabuli_Chr07	3430479	(G/A)
11822	CakSNP11822	*Kabuli*	Ca_Kabuli_Chr07	3445031	(T/C)
11823	CakSNP11823	*Kabuli*	Ca_Kabuli_Chr07	3476546	(T/C)
11824	CakSNP11824	*Kabuli*	Ca_Kabuli_Chr07	3504900	(G/A)
11825	CakSNP11825	*Kabuli*	Ca_Kabuli_Chr07	3505012	(A/C)
11826	CakSNP11826	*Kabuli*	Ca_Kabuli_Chr07	3518048	(G/A)
11827	CakSNP11827	*Kabuli*	Ca_Kabuli_Chr07	3519510	(A/G)
11828	CakSNP11828	*Kabuli*	Ca_Kabuli_Chr07	3519701	(A/C)
11829	CakSNP11829	*Kabuli*	Ca_Kabuli_Chr07	3521150	(A/G)
11830	CakSNP11830	*Kabuli*	Ca_Kabuli_Chr07	3521257	(G/A)
11831	CakSNP11831	*Kabuli*	Ca_Kabuli_Chr07	3521192	(C/A)
11832	CakSNP11832	*Kabuli*	Ca_Kabuli_Chr07	3536194	(C/T)
11833	CakSNP11833	*Kabuli*	Ca_Kabuli_Chr07	3536233	(T/C)
11834	CakSNP11834	*Kabuli*	Ca_Kabuli_Chr07	3543765	(A/G)
11835	CakSNP11835	*Kabuli*	Ca_Kabuli_Chr07	3543768	(T/C)
11836	CakSNP11836	*Kabuli*	Ca_Kabuli_Chr07	3557570	(C/T)
11837	CakSNP11837	*Kabuli*	Ca_Kabuli_Chr07	3560182	(G/A)
11838	CakSNP11838	*Kabuli*	Ca_Kabuli_Chr07	3560250	(C/T)
11839	CakSNP11839	*Kabuli*	Ca_Kabuli_Chr07	3572963	(T/C)
11840	CakSNP11840	*Kabuli*	Ca_Kabuli_Chr07	3581123	(G/A)
11841	CakSNP11841	*Kabuli*	Ca_Kabuli_Chr07	3600826	(C/T)
11842	CakSNP11842	*Kabuli*	Ca_Kabuli_Chr07	3600833	(T/G)
11843	CakSNP11843	*Kabuli*	Ca_Kabuli_Chr07	3600866	(T/C)
11844	CakSNP11844	*Kabuli*	Ca_Kabuli_Chr07	3600867	(C/T)
11845	CakSNP11845	*Kabuli*	Ca_Kabuli_Chr07	3600987	(A/C)
11846	CakSNP11846	*Kabuli*	Ca_Kabuli_Chr07	3628086	(G/A)
11847	CakSNP11847	*Kabuli*	Ca_Kabuli_Chr07	3635307	(G/A)
11848	CakSNP11848	*Kabuli*	Ca_Kabuli_Chr07	3635309	(C/T)
11849	CakSNP11849	*Kabuli*	Ca_Kabuli_Chr07	3635318	(T/A)
11850	CakSNP11850	*Kabuli*	Ca_Kabuli_Chr07	3635325	(T/C)
11851	CakSNP11851	*Kabuli*	Ca_Kabuli_Chr07	3635369	(G/T)
11852	CakSNP11852	*Kabuli*	Ca_Kabuli_Chr07	3651051	(T/C)
11853	CakSNP11853	*Kabuli*	Ca_Kabuli_Chr07	3655451	(G/T)
11854	CakSNP11854	*Kabuli*	Ca_Kabuli_Chr07	3665651	(G/C)
11855	CakSNP11855	*Kabuli*	Ca_Kabuli_Chr07	3672192	(T/C)

S.N.	SNP IDs	Cultivars	Chromosomes/scaffolds	Physical positions (bp)	SNPs
11856	CakSNP11856	*Kabuli*	Ca_Kabuli_Chr07	3672236	(G/A)
11857	CakSNP11857	*Kabuli*	Ca_Kabuli_Chr07	3734030	(T/A)
11858	CakSNP11858	*Kabuli*	Ca_Kabuli_Chr07	3738769	(T/C)
11859	CakSNP11859	*Kabuli*	Ca_Kabuli_Chr07	3754778	(G/A)
11860	CakSNP11860	*Kabuli*	Ca_Kabuli_Chr07	3754743	(G/C)
11861	CakSNP11861	*Kabuli*	Ca_Kabuli_Chr07	3795825	(T/C)
11862	CakSNP11862	*Kabuli*	Ca_Kabuli_Chr07	3795798	(G/A)
11863	CakSNP11863	*Kabuli*	Ca_Kabuli_Chr07	3795776	(C/A)
11864	CakSNP11864	*Kabuli*	Ca_Kabuli_Chr07	3796826	(A/G)
11865	CakSNP11865	*Kabuli*	Ca_Kabuli_Chr07	3796894	(A/G)
11866	CakSNP11866	*Kabuli*	Ca_Kabuli_Chr07	3802188	(T/A)
11867	CakSNP11867	*Kabuli*	Ca_Kabuli_Chr07	3814791	(A/G)
11868	CakSNP11868	*Kabuli*	Ca_Kabuli_Chr07	3817087	(C/T)
11869	CakSNP11869	*Kabuli*	Ca_Kabuli_Chr07	3817733	(T/G)
11870	CakSNP11870	*Kabuli*	Ca_Kabuli_Chr07	3817740	(G/T)
11871	CakSNP11871	*Kabuli*	Ca_Kabuli_Chr07	3829115	(C/T)
11872	CakSNP11872	*Kabuli*	Ca_Kabuli_Chr07	3843168	(A/T)
11873	CakSNP11873	*Kabuli*	Ca_Kabuli_Chr07	3845953	(A/C)
11874	CakSNP11874	*Kabuli*	Ca_Kabuli_Chr07	3845958	(G/A)
11875	CakSNP11875	*Kabuli*	Ca_Kabuli_Chr07	3845996	(G/A)
11876	CakSNP11876	*Kabuli*	Ca_Kabuli_Chr07	3856308	(C/T)
11877	CakSNP11877	*Kabuli*	Ca_Kabuli_Chr07	3859451	(G/A)
11878	CakSNP11878	*Kabuli*	Ca_Kabuli_Chr07	3916849	(A/C)
11879	CakSNP11879	*Kabuli*	Ca_Kabuli_Chr07	3924920	(T/C)
11880	CakSNP11880	*Kabuli*	Ca_Kabuli_Chr07	3946712	(G/A)
11881	CakSNP11881	*Kabuli*	Ca_Kabuli_Chr07	3982635	(T/G)
11882	CakSNP11882	*Kabuli*	Ca_Kabuli_Chr07	3982622	(G/A)
11883	CakSNP11883	*Kabuli*	Ca_Kabuli_Chr07	4017780	(C/T)
11884	CakSNP11884	*Kabuli*	Ca_Kabuli_Chr07	4017851	(T/C)
11885	CakSNP11885	*Kabuli*	Ca_Kabuli_Chr07	4039347	(C/A)
11886	CakSNP11886	*Kabuli*	Ca_Kabuli_Chr07	4039300	(T/C)
11887	CakSNP11887	*Kabuli*	Ca_Kabuli_Chr07	4039290	(A/G)
11888	CakSNP11888	*Kabuli*	Ca_Kabuli_Chr07	4042123	(T/G)
11889	CakSNP11889	*Kabuli*	Ca_Kabuli_Chr07	4095293	(A/G)
11890	CakSNP11890	*Kabuli*	Ca_Kabuli_Chr07	4168933	(G/T)
11891	CakSNP11891	*Kabuli*	Ca_Kabuli_Chr07	4194809	(T/A)
11892	CakSNP11892	*Kabuli*	Ca_Kabuli_Chr07	4194890	(C/A)
11893	CakSNP11893	*Kabuli*	Ca_Kabuli_Chr07	4194891	(C/G)

S.N.	SNP IDs	Cultivars	Chromosomes/scaffolds	Physical positions (bp)	SNPs
11894	CakSNP11894	*Kabuli*	Ca_Kabuli_Chr07	4198812	(A/C)
11895	CakSNP11895	*Kabuli*	Ca_Kabuli_Chr07	4209425	(C/T)
11896	CakSNP11896	*Kabuli*	Ca_Kabuli_Chr07	4209465	(A/C)
11897	CakSNP11897	*Kabuli*	Ca_Kabuli_Chr07	4225314	(T/C)
11898	CakSNP11898	*Kabuli*	Ca_Kabuli_Chr07	4256529	(G/A)
11899	CakSNP11899	*Kabuli*	Ca_Kabuli_Chr07	4256703	(C/G)
11900	CakSNP11900	*Kabuli*	Ca_Kabuli_Chr07	4257726	(A/T)
11901	CakSNP11901	*Kabuli*	Ca_Kabuli_Chr07	4257727	(T/A)
11902	CakSNP11902	*Kabuli*	Ca_Kabuli_Chr07	4257838	(C/T)
11903	CakSNP11903	*Kabuli*	Ca_Kabuli_Chr07	4257830	(G/T)
11904	CakSNP11904	*Kabuli*	Ca_Kabuli_Chr07	4257799	(G/A)
11905	CakSNP11905	*Kabuli*	Ca_Kabuli_Chr07	4289975	(G/A)
11906	CakSNP11906	*Kabuli*	Ca_Kabuli_Chr07	4300525	(C/T)
11907	CakSNP11907	*Kabuli*	Ca_Kabuli_Chr07	4300650	(T/C)
11908	CakSNP11908	*Kabuli*	Ca_Kabuli_Chr07	4300645	(C/T)
11909	CakSNP11909	*Kabuli*	Ca_Kabuli_Chr07	4300592	(G/A)
11910	CakSNP11910	*Kabuli*	Ca_Kabuli_Chr07	4300584	(C/T)
11911	CakSNP11911	*Kabuli*	Ca_Kabuli_Chr07	4305424	(G/T)
11912	CakSNP11912	*Kabuli*	Ca_Kabuli_Chr07	4431138	(A/G)
11913	CakSNP11913	*Kabuli*	Ca_Kabuli_Chr07	4431253	(T/C)
11914	CakSNP11914	*Kabuli*	Ca_Kabuli_Chr07	4438929	(A/G)
11915	CakSNP11915	*Kabuli*	Ca_Kabuli_Chr07	4455045	(T/C)
11916	CakSNP11916	*Kabuli*	Ca_Kabuli_Chr07	4461990	(A/C)
11917	CakSNP11917	*Kabuli*	Ca_Kabuli_Chr07	4470285	(C/G)
11918	CakSNP11918	*Kabuli*	Ca_Kabuli_Chr07	4490514	(G/A)
11919	CakSNP11919	*Kabuli*	Ca_Kabuli_Chr07	4490964	(G/T)
11920	CakSNP11920	*Kabuli*	Ca_Kabuli_Chr07	4490966	(A/G)
11921	CakSNP11921	*Kabuli*	Ca_Kabuli_Chr07	4497784	(C/T)
11922	CakSNP11922	*Kabuli*	Ca_Kabuli_Chr07	4520606	(T/C)
11923	CakSNP11923	*Kabuli*	Ca_Kabuli_Chr07	4525645	(A/G)
11924	CakSNP11924	*Kabuli*	Ca_Kabuli_Chr07	4552790	(T/C)
11925	CakSNP11925	*Kabuli*	Ca_Kabuli_Chr07	4573236	(G/A)
11926	CakSNP11926	*Kabuli*	Ca_Kabuli_Chr07	4625723	(C/T)
11927	CakSNP11927	*Kabuli*	Ca_Kabuli_Chr07	4648569	(A/C)
11928	CakSNP11928	*Kabuli*	Ca_Kabuli_Chr07	4648697	(T/A)
11929	CakSNP11929	*Kabuli*	Ca_Kabuli_Chr07	4648677	(A/T)
11930	CakSNP11930	*Kabuli*	Ca_Kabuli_Chr07	4648662	(G/A)
11931	CakSNP11931	*Kabuli*	Ca_Kabuli_Chr07	4649287	(A/G)

S.N.	SNP IDs	Cultivars	Chromosomes/scaffolds	Physical positions (bp)	SNPs
11932	CakSNP11932	*Kabuli*	Ca_Kabuli_Chr07	4651931	(T/C)
11933	CakSNP11933	*Kabuli*	Ca_Kabuli_Chr07	4652083	(G/A)
11934	CakSNP11934	*Kabuli*	Ca_Kabuli_Chr07	4653235	(A/C)
11935	CakSNP11935	*Kabuli*	Ca_Kabuli_Chr07	4700570	(C/A)
11936	CakSNP11936	*Kabuli*	Ca_Kabuli_Chr07	4749212	(T/G)
11937	CakSNP11937	*Kabuli*	Ca_Kabuli_Chr07	4752954	(G/C)
11938	CakSNP11938	*Kabuli*	Ca_Kabuli_Chr07	4752887	(T/G)
11939	CakSNP11939	*Kabuli*	Ca_Kabuli_Chr07	4756338	(G/A)
11940	CakSNP11940	*Kabuli*	Ca_Kabuli_Chr07	4832993	(A/G)
11941	CakSNP11941	*Kabuli*	Ca_Kabuli_Chr07	4834177	(G/T)
11942	CakSNP11942	*Kabuli*	Ca_Kabuli_Chr07	4849553	(T/G)
11943	CakSNP11943	*Kabuli*	Ca_Kabuli_Chr07	4849573	(T/C)
11944	CakSNP11944	*Kabuli*	Ca_Kabuli_Chr07	4907315	(C/T)
11945	CakSNP11945	*Kabuli*	Ca_Kabuli_Chr07	4926759	(A/T)
11946	CakSNP11946	*Kabuli*	Ca_Kabuli_Chr07	4942847	(A/G)
11947	CakSNP11947	*Kabuli*	Ca_Kabuli_Chr07	4942902	(G/C)
11948	CakSNP11948	*Kabuli*	Ca_Kabuli_Chr07	4945935	(C/G)
11949	CakSNP11949	*Kabuli*	Ca_Kabuli_Chr07	4981846	(T/C)
11950	CakSNP11950	*Kabuli*	Ca_Kabuli_Chr07	5122536	(T/G)
11951	CakSNP11951	*Kabuli*	Ca_Kabuli_Chr07	5251897	(G/C)
11952	CakSNP11952	*Kabuli*	Ca_Kabuli_Chr07	5361558	(T/C)
11953	CakSNP11953	*Kabuli*	Ca_Kabuli_Chr07	5383564	(G/C)
11954	CakSNP11954	*Kabuli*	Ca_Kabuli_Chr07	5383566	(A/T)
11955	CakSNP11955	*Kabuli*	Ca_Kabuli_Chr07	5383671	(G/T)
11956	CakSNP11956	*Kabuli*	Ca_Kabuli_Chr07	5383695	(T/G)
11957	CakSNP11957	*Kabuli*	Ca_Kabuli_Chr07	5383767	(G/A)
11958	CakSNP11958	*Kabuli*	Ca_Kabuli_Chr07	5384447	(G/C)
11959	CakSNP11959	*Kabuli*	Ca_Kabuli_Chr07	5384467	(C/T)
11960	CakSNP11960	*Kabuli*	Ca_Kabuli_Chr07	5406524	(T/G)
11961	CakSNP11961	*Kabuli*	Ca_Kabuli_Chr07	5416862	(C/G)
11962	CakSNP11962	*Kabuli*	Ca_Kabuli_Chr07	5416865	(A/G)
11963	CakSNP11963	*Kabuli*	Ca_Kabuli_Chr07	5416874	(C/T)
11964	CakSNP11964	*Kabuli*	Ca_Kabuli_Chr07	5464941	(G/A)
11965	CakSNP11965	*Kabuli*	Ca_Kabuli_Chr07	5464942	(A/G)
11966	CakSNP11966	*Kabuli*	Ca_Kabuli_Chr07	5467744	(G/C)
11967	CakSNP11967	*Kabuli*	Ca_Kabuli_Chr07	5500318	(C/A)
11968	CakSNP11968	*Kabuli*	Ca_Kabuli_Chr07	5508162	(A/C)
11969	CakSNP11969	*Kabuli*	Ca_Kabuli_Chr07	5520720	(T/G)

S.N.	SNP IDs	Cultivars	Chromosomes/scaffolds	Physical positions (bp)	SNPs
11970	CakSNP11970	*Kabuli*	Ca_Kabuli_Chr07	5520934	(T/C)
11971	CakSNP11971	*Kabuli*	Ca_Kabuli_Chr07	5521596	(T/C)
11972	CakSNP11972	*Kabuli*	Ca_Kabuli_Chr07	5521659	(G/T)
11973	CakSNP11973	*Kabuli*	Ca_Kabuli_Chr07	5529577	(T/G)
11974	CakSNP11974	*Kabuli*	Ca_Kabuli_Chr07	5529729	(A/G)
11975	CakSNP11975	*Kabuli*	Ca_Kabuli_Chr07	5529888	(A/G)
11976	CakSNP11976	*Kabuli*	Ca_Kabuli_Chr07	5529844	(C/A)
11977	CakSNP11977	*Kabuli*	Ca_Kabuli_Chr07	5537851	(G/C)
11978	CakSNP11978	*Kabuli*	Ca_Kabuli_Chr07	5537880	(G/T)
11979	CakSNP11979	*Kabuli*	Ca_Kabuli_Chr07	5561607	(G/T)
11980	CakSNP11980	*Kabuli*	Ca_Kabuli_Chr07	5568114	(C/A)
11981	CakSNP11981	*Kabuli*	Ca_Kabuli_Chr07	5577467	(G/T)
11982	CakSNP11982	*Kabuli*	Ca_Kabuli_Chr07	5577439	(A/G)
11983	CakSNP11983	*Kabuli*	Ca_Kabuli_Chr07	5581968	(G/C)
11984	CakSNP11984	*Kabuli*	Ca_Kabuli_Chr07	5602106	(T/C)
11985	CakSNP11985	*Kabuli*	Ca_Kabuli_Chr07	5602144	(G/T)
11986	CakSNP11986	*Kabuli*	Ca_Kabuli_Chr07	5605118	(G/A)
11987	CakSNP11987	*Kabuli*	Ca_Kabuli_Chr07	5605106	(G/A)
11988	CakSNP11988	*Kabuli*	Ca_Kabuli_Chr07	5658445	(G/T)
11989	CakSNP11989	*Kabuli*	Ca_Kabuli_Chr07	5671902	(G/T)
11990	CakSNP11990	*Kabuli*	Ca_Kabuli_Chr07	5672009	(G/C)
11991	CakSNP11991	*Kabuli*	Ca_Kabuli_Chr07	5672156	(T/C)
11992	CakSNP11992	*Kabuli*	Ca_Kabuli_Chr07	5683412	(T/A)
11993	CakSNP11993	*Kabuli*	Ca_Kabuli_Chr07	5713977	(T/C)
11994	CakSNP11994	*Kabuli*	Ca_Kabuli_Chr07	5735293	(T/A)
11995	CakSNP11995	*Kabuli*	Ca_Kabuli_Chr07	5735448	(A/T)
11996	CakSNP11996	*Kabuli*	Ca_Kabuli_Chr07	5737341	(C/T)
11997	CakSNP11997	*Kabuli*	Ca_Kabuli_Chr07	5737305	(G/A)
11998	CakSNP11998	*Kabuli*	Ca_Kabuli_Chr07	5756269	(C/T)
11999	CakSNP11999	*Kabuli*	Ca_Kabuli_Chr07	5756832	(A/C)
12000	CakSNP12000	*Kabuli*	Ca_Kabuli_Chr07	5774218	(T/A)
12001	CakSNP12001	*Kabuli*	Ca_Kabuli_Chr07	5774219	(T/A)
12002	CakSNP12002	*Kabuli*	Ca_Kabuli_Chr07	5774220	(A/T)
12003	CakSNP12003	*Kabuli*	Ca_Kabuli_Chr07	5774221	(A/T)
12004	CakSNP12004	*Kabuli*	Ca_Kabuli_Chr07	5774230	(G/A)
12005	CakSNP12005	*Kabuli*	Ca_Kabuli_Chr07	5774257	(C/T)
12006	CakSNP12006	*Kabuli*	Ca_Kabuli_Chr07	5774310	(A/G)
12007	CakSNP12007	*Kabuli*	Ca_Kabuli_Chr07	5811097	(G/A)

S.N.	SNP IDs	Cultivars	Chromosomes/scaffolds	Physical positions (bp)	SNPs
12008	CakSNP12008	*Kabuli*	Ca_Kabuli_Chr07	5811060	(T/C)
12009	CakSNP12009	*Kabuli*	Ca_Kabuli_Chr07	5811044	(T/A)
12010	CakSNP12010	*Kabuli*	Ca_Kabuli_Chr07	5836421	(A/T)
12011	CakSNP12011	*Kabuli*	Ca_Kabuli_Chr07	5846477	(A/G)
12012	CakSNP12012	*Kabuli*	Ca_Kabuli_Chr07	5849936	(C/T)
12013	CakSNP12013	*Kabuli*	Ca_Kabuli_Chr07	5849904	(C/A)
12014	CakSNP12014	*Kabuli*	Ca_Kabuli_Chr07	5850058	(T/C)
12015	CakSNP12015	*Kabuli*	Ca_Kabuli_Chr07	5854598	(C/T)
12016	CakSNP12016	*Kabuli*	Ca_Kabuli_Chr07	5929009	(C/G)
12017	CakSNP12017	*Kabuli*	Ca_Kabuli_Chr07	5946113	(G/A)
12018	CakSNP12018	*Kabuli*	Ca_Kabuli_Chr07	5950352	(T/A)
12019	CakSNP12019	*Kabuli*	Ca_Kabuli_Chr07	5950492	(T/C)
12020	CakSNP12020	*Kabuli*	Ca_Kabuli_Chr07	5957696	(T/C)
12021	CakSNP12021	*Kabuli*	Ca_Kabuli_Chr07	5963611	(C/G)
12022	CakSNP12022	*Kabuli*	Ca_Kabuli_Chr07	5963609	(C/A)
12023	CakSNP12023	*Kabuli*	Ca_Kabuli_Chr07	6012561	(A/C)
12024	CakSNP12024	*Kabuli*	Ca_Kabuli_Chr07	6012611	(A/C)
12025	CakSNP12025	*Kabuli*	Ca_Kabuli_Chr07	6029991	(G/A)
12026	CakSNP12026	*Kabuli*	Ca_Kabuli_Chr07	6029990	(C/G)
12027	CakSNP12027	*Kabuli*	Ca_Kabuli_Chr07	6066322	(G/A)
12028	CakSNP12028	*Kabuli*	Ca_Kabuli_Chr07	6074408	(T/A)
12029	CakSNP12029	*Kabuli*	Ca_Kabuli_Chr07	6075124	(T/G)
12030	CakSNP12030	*Kabuli*	Ca_Kabuli_Chr07	6075218	(C/G)
12031	CakSNP12031	*Kabuli*	Ca_Kabuli_Chr07	6096238	(C/A)
12032	CakSNP12032	*Kabuli*	Ca_Kabuli_Chr07	6096237	(C/G)
12033	CakSNP12033	*Kabuli*	Ca_Kabuli_Chr07	6110019	(T/C)
12034	CakSNP12034	*Kabuli*	Ca_Kabuli_Chr07	6132021	(G/A)
12035	CakSNP12035	*Kabuli*	Ca_Kabuli_Chr07	6232777	(T/C)
12036	CakSNP12036	*Kabuli*	Ca_Kabuli_Chr07	6282979	(C/A)
12037	CakSNP12037	*Kabuli*	Ca_Kabuli_Chr07	6282974	(T/A)
12038	CakSNP12038	*Kabuli*	Ca_Kabuli_Chr07	6282949	(T/A)
12039	CakSNP12039	*Kabuli*	Ca_Kabuli_Chr07	6299248	(A/G)
12040	CakSNP12040	*Kabuli*	Ca_Kabuli_Chr07	6299249	(C/G)
12041	CakSNP12041	*Kabuli*	Ca_Kabuli_Chr07	6330237	(A/T)
12042	CakSNP12042	*Kabuli*	Ca_Kabuli_Chr07	6340051	(A/G)
12043	CakSNP12043	*Kabuli*	Ca_Kabuli_Chr07	6340064	(C/G)
12044	CakSNP12044	*Kabuli*	Ca_Kabuli_Chr07	6340111	(C/G)
12045	CakSNP12045	*Kabuli*	Ca_Kabuli_Chr07	6340113	(G/A)

S.N.	SNP IDs	Cultivars	Chromosomes/scaffolds	Physical positions (bp)	SNPs
12046	CakSNP12046	*Kabuli*	Ca_Kabuli_Chr07	6340167	(T/A)
12047	CakSNP12047	*Kabuli*	Ca_Kabuli_Chr07	6340216	(T/C)
12048	CakSNP12048	*Kabuli*	Ca_Kabuli_Chr07	6372037	(A/G)
12049	CakSNP12049	*Kabuli*	Ca_Kabuli_Chr07	6393366	(G/A)
12050	CakSNP12050	*Kabuli*	Ca_Kabuli_Chr07	6430396	(T/A)
12051	CakSNP12051	*Kabuli*	Ca_Kabuli_Chr07	6433480	(C/G)
12052	CakSNP12052	*Kabuli*	Ca_Kabuli_Chr07	6467903	(C/T)
12053	CakSNP12053	*Kabuli*	Ca_Kabuli_Chr07	6540624	(A/G)
12054	CakSNP12054	*Kabuli*	Ca_Kabuli_Chr07	6540602	(G/A)
12055	CakSNP12055	*Kabuli*	Ca_Kabuli_Chr07	6540601	(G/C)
12056	CakSNP12056	*Kabuli*	Ca_Kabuli_Chr07	6566322	(T/C)
12057	CakSNP12057	*Kabuli*	Ca_Kabuli_Chr07	6654718	(C/T)
12058	CakSNP12058	*Kabuli*	Ca_Kabuli_Chr07	6681050	(C/T)
12059	CakSNP12059	*Kabuli*	Ca_Kabuli_Chr07	6682456	(T/A)
12060	CakSNP12060	*Kabuli*	Ca_Kabuli_Chr07	6682454	(A/G)
12061	CakSNP12061	*Kabuli*	Ca_Kabuli_Chr07	6682453	(G/T)
12062	CakSNP12062	*Kabuli*	Ca_Kabuli_Chr07	6682448	(T/C)
12063	CakSNP12063	*Kabuli*	Ca_Kabuli_Chr07	6682443	(C/T)
12064	CakSNP12064	*Kabuli*	Ca_Kabuli_Chr07	6682439	(T/A)
12065	CakSNP12065	*Kabuli*	Ca_Kabuli_Chr07	6682455	(G/T)
12066	CakSNP12066	*Kabuli*	Ca_Kabuli_Chr07	6703759	(C/T)
12067	CakSNP12067	*Kabuli*	Ca_Kabuli_Chr07	6703849	(G/T)
12068	CakSNP12068	*Kabuli*	Ca_Kabuli_Chr07	6767204	(G/T)
12069	CakSNP12069	*Kabuli*	Ca_Kabuli_Chr07	6769694	(T/G)
12070	CakSNP12070	*Kabuli*	Ca_Kabuli_Chr07	6792352	(T/C)
12071	CakSNP12071	*Kabuli*	Ca_Kabuli_Chr07	6792606	(T/G)
12072	CakSNP12072	*Kabuli*	Ca_Kabuli_Chr07	6807817	(A/C)
12073	CakSNP12073	*Kabuli*	Ca_Kabuli_Chr07	6814954	(G/T)
12074	CakSNP12074	*Kabuli*	Ca_Kabuli_Chr07	6815151	(G/T)
12075	CakSNP12075	*Kabuli*	Ca_Kabuli_Chr07	6815113	(T/C)
12076	CakSNP12076	*Kabuli*	Ca_Kabuli_Chr07	6859511	(C/G)
12077	CakSNP12077	*Kabuli*	Ca_Kabuli_Chr07	6904686	(G/C)
12078	CakSNP12078	*Kabuli*	Ca_Kabuli_Chr07	7026448	(C/T)
12079	CakSNP12079	*Kabuli*	Ca_Kabuli_Chr07	7026516	(T/C)
12080	CakSNP12080	*Kabuli*	Ca_Kabuli_Chr07	7026500	(T/A)
12081	CakSNP12081	*Kabuli*	Ca_Kabuli_Chr07	7072853	(G/A)
12082	CakSNP12082	*Kabuli*	Ca_Kabuli_Chr07	7126974	(T/C)
12083	CakSNP12083	*Kabuli*	Ca_Kabuli_Chr07	7126979	(C/G)

S.N.	SNP IDs	Cultivars	Chromosomes/scaffolds	Physical positions (bp)	SNPs
12084	CakSNP12084	*Kabuli*	Ca_Kabuli_Chr07	7127030	(G/A)
12085	CakSNP12085	*Kabuli*	Ca_Kabuli_Chr07	7146159	(C/G)
12086	CakSNP12086	*Kabuli*	Ca_Kabuli_Chr07	7147557	(A/G)
12087	CakSNP12087	*Kabuli*	Ca_Kabuli_Chr07	7234422	(T/G)
12088	CakSNP12088	*Kabuli*	Ca_Kabuli_Chr07	7234507	(T/C)
12089	CakSNP12089	*Kabuli*	Ca_Kabuli_Chr07	7234484	(G/A)
12090	CakSNP12090	*Kabuli*	Ca_Kabuli_Chr07	7234430	(C/T)
12091	CakSNP12091	*Kabuli*	Ca_Kabuli_Chr07	7261971	(C/T)
12092	CakSNP12092	*Kabuli*	Ca_Kabuli_Chr07	7261941	(T/C)
12093	CakSNP12093	*Kabuli*	Ca_Kabuli_Chr07	7265386	(T/A)
12094	CakSNP12094	*Kabuli*	Ca_Kabuli_Chr07	7277353	(G/A)
12095	CakSNP12095	*Kabuli*	Ca_Kabuli_Chr07	7304614	(T/G)
12096	CakSNP12096	*Kabuli*	Ca_Kabuli_Chr07	7304617	(T/G)
12097	CakSNP12097	*Kabuli*	Ca_Kabuli_Chr07	7423419	(G/A)
12098	CakSNP12098	*Kabuli*	Ca_Kabuli_Chr07	7433827	(A/T)
12099	CakSNP12099	*Kabuli*	Ca_Kabuli_Chr07	7449489	(T/C)
12100	CakSNP12100	*Kabuli*	Ca_Kabuli_Chr07	7451931	(G/C)
12101	CakSNP12101	*Kabuli*	Ca_Kabuli_Chr07	7491960	(G/A)
12102	CakSNP12102	*Kabuli*	Ca_Kabuli_Chr07	7498598	(C/T)
12103	CakSNP12103	*Kabuli*	Ca_Kabuli_Chr07	7544142	(A/G)
12104	CakSNP12104	*Kabuli*	Ca_Kabuli_Chr07	7573889	(C/G)
12105	CakSNP12105	*Kabuli*	Ca_Kabuli_Chr07	7576668	(A/C)
12106	CakSNP12106	*Kabuli*	Ca_Kabuli_Chr07	7580158	(T/C)
12107	CakSNP12107	*Kabuli*	Ca_Kabuli_Chr07	7580147	(C/A)
12108	CakSNP12108	*Kabuli*	Ca_Kabuli_Chr07	7580118	(G/A)
12109	CakSNP12109	*Kabuli*	Ca_Kabuli_Chr07	7612046	(A/T)
12110	CakSNP12110	*Kabuli*	Ca_Kabuli_Chr07	7624697	(G/A)
12111	CakSNP12111	*Kabuli*	Ca_Kabuli_Chr07	7625226	(G/T)
12112	CakSNP12112	*Kabuli*	Ca_Kabuli_Chr07	7625277	(A/T)
12113	CakSNP12113	*Kabuli*	Ca_Kabuli_Chr07	7625901	(A/C)
12114	CakSNP12114	*Kabuli*	Ca_Kabuli_Chr07	7625943	(C/G)
12115	CakSNP12115	*Kabuli*	Ca_Kabuli_Chr07	7663564	(A/G)
12116	CakSNP12116	*Kabuli*	Ca_Kabuli_Chr07	7706816	(A/G)
12117	CakSNP12117	*Kabuli*	Ca_Kabuli_Chr07	7756455	(C/T)
12118	CakSNP12118	*Kabuli*	Ca_Kabuli_Chr07	7756482	(G/A)
12119	CakSNP12119	*Kabuli*	Ca_Kabuli_Chr07	7756566	(G/A)
12120	CakSNP12120	*Kabuli*	Ca_Kabuli_Chr07	7809179	(T/G)
12121	CakSNP12121	*Kabuli*	Ca_Kabuli_Chr07	7809192	(A/C)

S.N.	SNP IDs	Cultivars	Chromosomes/scaffolds	Physical positions (bp)	SNPs
12122	CakSNP12122	*Kabuli*	Ca_Kabuli_Chr07	7875189	(C/A)
12123	CakSNP12123	*Kabuli*	Ca_Kabuli_Chr07	7876554	(G/A)
12124	CakSNP12124	*Kabuli*	Ca_Kabuli_Chr07	7876561	(C/T)
12125	CakSNP12125	*Kabuli*	Ca_Kabuli_Chr07	7876570	(C/A)
12126	CakSNP12126	*Kabuli*	Ca_Kabuli_Chr07	7928398	(C/T)
12127	CakSNP12127	*Kabuli*	Ca_Kabuli_Chr07	7982650	(G/T)
12128	CakSNP12128	*Kabuli*	Ca_Kabuli_Chr07	7982871	(T/C)
12129	CakSNP12129	*Kabuli*	Ca_Kabuli_Chr07	7982890	(T/A)
12130	CakSNP12130	*Kabuli*	Ca_Kabuli_Chr07	7983519	(C/T)
12131	CakSNP12131	*Kabuli*	Ca_Kabuli_Chr07	8041465	(T/G)
12132	CakSNP12132	*Kabuli*	Ca_Kabuli_Chr07	8054489	(G/A)
12133	CakSNP12133	*Kabuli*	Ca_Kabuli_Chr07	8061808	(A/G)
12134	CakSNP12134	*Kabuli*	Ca_Kabuli_Chr07	8111735	(T/C)
12135	CakSNP12135	*Kabuli*	Ca_Kabuli_Chr07	8111754	(A/C)
12136	CakSNP12136	*Kabuli*	Ca_Kabuli_Chr07	8111759	(G/T)
12137	CakSNP12137	*Kabuli*	Ca_Kabuli_Chr07	8169857	(A/G)
12138	CakSNP12138	*Kabuli*	Ca_Kabuli_Chr07	8169866	(A/G)
12139	CakSNP12139	*Kabuli*	Ca_Kabuli_Chr07	8169960	(A/T)
12140	CakSNP12140	*Kabuli*	Ca_Kabuli_Chr07	8169947	(T/C)
12141	CakSNP12141	*Kabuli*	Ca_Kabuli_Chr07	8187181	(G/A)
12142	CakSNP12142	*Kabuli*	Ca_Kabuli_Chr07	8188004	(C/G)
12143	CakSNP12143	*Kabuli*	Ca_Kabuli_Chr07	8210963	(G/A)
12144	CakSNP12144	*Kabuli*	Ca_Kabuli_Chr07	8261062	(C/A)
12145	CakSNP12145	*Kabuli*	Ca_Kabuli_Chr07	8286115	(A/T)
12146	CakSNP12146	*Kabuli*	Ca_Kabuli_Chr07	8515092	(C/T)
12147	CakSNP12147	*Kabuli*	Ca_Kabuli_Chr07	8535189	(C/T)
12148	CakSNP12148	*Kabuli*	Ca_Kabuli_Chr07	8553114	(C/T)
12149	CakSNP12149	*Kabuli*	Ca_Kabuli_Chr07	8569632	(A/G)
12150	CakSNP12150	*Kabuli*	Ca_Kabuli_Chr07	8583890	(T/C)
12151	CakSNP12151	*Kabuli*	Ca_Kabuli_Chr07	8609831	(A/T)
12152	CakSNP12152	*Kabuli*	Ca_Kabuli_Chr07	8626840	(A/C)
12153	CakSNP12153	*Kabuli*	Ca_Kabuli_Chr07	8626875	(A/T)
12154	CakSNP12154	*Kabuli*	Ca_Kabuli_Chr07	8626916	(T/C)
12155	CakSNP12155	*Kabuli*	Ca_Kabuli_Chr07	8741098	(T/C)
12156	CakSNP12156	*Kabuli*	Ca_Kabuli_Chr07	8777670	(A/G)
12157	CakSNP12157	*Kabuli*	Ca_Kabuli_Chr07	8777673	(A/G)
12158	CakSNP12158	*Kabuli*	Ca_Kabuli_Chr07	8777690	(G/A)
12159	CakSNP12159	*Kabuli*	Ca_Kabuli_Chr07	8824586	(A/G)

S.N.	SNP IDs	Cultivars	Chromosomes/scaffolds	Physical positions (bp)	SNPs
12160	CakSNP12160	*Kabuli*	Ca_Kabuli_Chr07	8824591	(C/T)
12161	CakSNP12161	*Kabuli*	Ca_Kabuli_Chr07	8830772	(A/C)
12162	CakSNP12162	*Kabuli*	Ca_Kabuli_Chr07	8830985	(A/G)
12163	CakSNP12163	*Kabuli*	Ca_Kabuli_Chr07	8831024	(C/T)
12164	CakSNP12164	*Kabuli*	Ca_Kabuli_Chr07	8831096	(G/A)
12165	CakSNP12165	*Kabuli*	Ca_Kabuli_Chr07	8835601	(G/A)
12166	CakSNP12166	*Kabuli*	Ca_Kabuli_Chr07	8835602	(A/G)
12167	CakSNP12167	*Kabuli*	Ca_Kabuli_Chr07	8835610	(G/A)
12168	CakSNP12168	*Kabuli*	Ca_Kabuli_Chr07	8835659	(G/T)
12169	CakSNP12169	*Kabuli*	Ca_Kabuli_Chr07	8835757	(T/C)
12170	CakSNP12170	*Kabuli*	Ca_Kabuli_Chr07	8835718	(T/G)
12171	CakSNP12171	*Kabuli*	Ca_Kabuli_Chr07	8835705	(T/A)
12172	CakSNP12172	*Kabuli*	Ca_Kabuli_Chr07	8844902	(C/T)
12173	CakSNP12173	*Kabuli*	Ca_Kabuli_Chr07	8894362	(A/T)
12174	CakSNP12174	*Kabuli*	Ca_Kabuli_Chr07	8895087	(T/C)
12175	CakSNP12175	*Kabuli*	Ca_Kabuli_Chr07	8913092	(C/G)
12176	CakSNP12176	*Kabuli*	Ca_Kabuli_Chr07	8937391	(A/G)
12177	CakSNP12177	*Kabuli*	Ca_Kabuli_Chr07	8970387	(A/G)
12178	CakSNP12178	*Kabuli*	Ca_Kabuli_Chr07	9128044	(A/G)
12179	CakSNP12179	*Kabuli*	Ca_Kabuli_Chr07	9128050	(C/A)
12180	CakSNP12180	*Kabuli*	Ca_Kabuli_Chr07	9147367	(A/T)
12181	CakSNP12181	*Kabuli*	Ca_Kabuli_Chr07	9147366	(T/G)
12182	CakSNP12182	*Kabuli*	Ca_Kabuli_Chr07	9147502	(C/T)
12183	CakSNP12183	*Kabuli*	Ca_Kabuli_Chr07	9151709	(T/G)
12184	CakSNP12184	*Kabuli*	Ca_Kabuli_Chr07	9151755	(C/T)
12185	CakSNP12185	*Kabuli*	Ca_Kabuli_Chr07	9153104	(C/A)
12186	CakSNP12186	*Kabuli*	Ca_Kabuli_Chr07	9153062	(G/A)
12187	CakSNP12187	*Kabuli*	Ca_Kabuli_Chr07	9214457	(T/A)
12188	CakSNP12188	*Kabuli*	Ca_Kabuli_Chr07	9214422	(C/T)
12189	CakSNP12189	*Kabuli*	Ca_Kabuli_Chr07	9301825	(G/C)
12190	CakSNP12190	*Kabuli*	Ca_Kabuli_Chr07	9301881	(C/T)
12191	CakSNP12191	*Kabuli*	Ca_Kabuli_Chr07	9327709	(C/T)
12192	CakSNP12192	*Kabuli*	Ca_Kabuli_Chr07	9327687	(G/A)
12193	CakSNP12193	*Kabuli*	Ca_Kabuli_Chr07	9348281	(T/C)
12194	CakSNP12194	*Kabuli*	Ca_Kabuli_Chr07	9350506	(T/G)
12195	CakSNP12195	*Kabuli*	Ca_Kabuli_Chr07	9350535	(A/C)
12196	CakSNP12196	*Kabuli*	Ca_Kabuli_Chr07	9351783	(C/G)
12197	CakSNP12197	*Kabuli*	Ca_Kabuli_Chr07	9351863	(G/A)

S.N.	SNP IDs	Cultivars	Chromosomes/scaffolds	Physical positions (bp)	SNPs
12198	CakSNP12198	*Kabuli*	Ca_Kabuli_Chr07	9422842	(T/C)
12199	CakSNP12199	*Kabuli*	Ca_Kabuli_Chr07	9422859	(T/C)
12200	CakSNP12200	*Kabuli*	Ca_Kabuli_Chr07	9422876	(T/C)
12201	CakSNP12201	*Kabuli*	Ca_Kabuli_Chr07	9422946	(G/C)
12202	CakSNP12202	*Kabuli*	Ca_Kabuli_Chr07	9442836	(A/C)
12203	CakSNP12203	*Kabuli*	Ca_Kabuli_Chr07	9475275	(T/G)
12204	CakSNP12204	*Kabuli*	Ca_Kabuli_Chr07	9475817	(C/A)
12205	CakSNP12205	*Kabuli*	Ca_Kabuli_Chr07	9475922	(A/G)
12206	CakSNP12206	*Kabuli*	Ca_Kabuli_Chr07	9475874	(C/T)
12207	CakSNP12207	*Kabuli*	Ca_Kabuli_Chr07	9523902	(C/T)
12208	CakSNP12208	*Kabuli*	Ca_Kabuli_Chr07	9528324	(A/G)
12209	CakSNP12209	*Kabuli*	Ca_Kabuli_Chr07	9536012	(C/T)
12210	CakSNP12210	*Kabuli*	Ca_Kabuli_Chr07	9550284	(G/C)
12211	CakSNP12211	*Kabuli*	Ca_Kabuli_Chr07	9550230	(C/T)
12212	CakSNP12212	*Kabuli*	Ca_Kabuli_Chr07	9555319	(C/G)
12213	CakSNP12213	*Kabuli*	Ca_Kabuli_Chr07	9577441	(G/A)
12214	CakSNP12214	*Kabuli*	Ca_Kabuli_Chr07	9577424	(G/C)
12215	CakSNP12215	*Kabuli*	Ca_Kabuli_Chr07	9593677	(C/T)
12216	CakSNP12216	*Kabuli*	Ca_Kabuli_Chr07	9612558	(A/C)
12217	CakSNP12217	*Kabuli*	Ca_Kabuli_Chr07	9616335	(G/A)
12218	CakSNP12218	*Kabuli*	Ca_Kabuli_Chr07	9616347	(C/G)
12219	CakSNP12219	*Kabuli*	Ca_Kabuli_Chr07	9616357	(C/T)
12220	CakSNP12220	*Kabuli*	Ca_Kabuli_Chr07	9616363	(A/C)
12221	CakSNP12221	*Kabuli*	Ca_Kabuli_Chr07	9616367	(C/G)
12222	CakSNP12222	*Kabuli*	Ca_Kabuli_Chr07	9616378	(G/A)
12223	CakSNP12223	*Kabuli*	Ca_Kabuli_Chr07	9637456	(C/T)
12224	CakSNP12224	*Kabuli*	Ca_Kabuli_Chr07	9639993	(C/A)
12225	CakSNP12225	*Kabuli*	Ca_Kabuli_Chr07	9661050	(A/C)
12226	CakSNP12226	*Kabuli*	Ca_Kabuli_Chr07	9671210	(T/C)
12227	CakSNP12227	*Kabuli*	Ca_Kabuli_Chr07	9696620	(A/G)
12228	CakSNP12228	*Kabuli*	Ca_Kabuli_Chr07	9738801	(T/C)
12229	CakSNP12229	*Kabuli*	Ca_Kabuli_Chr07	9744436	(G/T)
12230	CakSNP12230	*Kabuli*	Ca_Kabuli_Chr07	9890779	(A/C)
12231	CakSNP12231	*Kabuli*	Ca_Kabuli_Chr07	9890777	(A/G)
12232	CakSNP12232	*Kabuli*	Ca_Kabuli_Chr07	9893688	(A/G)
12233	CakSNP12233	*Kabuli*	Ca_Kabuli_Chr07	9897698	(T/G)
12234	CakSNP12234	*Kabuli*	Ca_Kabuli_Chr07	9898718	(A/T)
12235	CakSNP12235	*Kabuli*	Ca_Kabuli_Chr07	9899348	(C/T)

S.N.	SNP IDs	Cultivars	Chromosomes/scaffolds	Physical positions (bp)	SNPs
12236	CakSNP12236	*Kabuli*	Ca_Kabuli_Chr07	9914751	(G/A)
12237	CakSNP12237	*Kabuli*	Ca_Kabuli_Chr07	9914764	(G/A)
12238	CakSNP12238	*Kabuli*	Ca_Kabuli_Chr07	9914770	(T/C)
12239	CakSNP12239	*Kabuli*	Ca_Kabuli_Chr07	9914785	(T/C)
12240	CakSNP12240	*Kabuli*	Ca_Kabuli_Chr07	9914794	(G/A)
12241	CakSNP12241	*Kabuli*	Ca_Kabuli_Chr07	9914737	(C/G)
12242	CakSNP12242	*Kabuli*	Ca_Kabuli_Chr07	9914745	(T/C)
12243	CakSNP12243	*Kabuli*	Ca_Kabuli_Chr07	9914856	(G/A)
12244	CakSNP12244	*Kabuli*	Ca_Kabuli_Chr07	9914845	(A/C)
12245	CakSNP12245	*Kabuli*	Ca_Kabuli_Chr07	9914832	(C/T)
12246	CakSNP12246	*Kabuli*	Ca_Kabuli_Chr07	9914802	(T/G)
12247	CakSNP12247	*Kabuli*	Ca_Kabuli_Chr07	9932369	(A/G)
12248	CakSNP12248	*Kabuli*	Ca_Kabuli_Chr07	9936661	(C/T)
12249	CakSNP12249	*Kabuli*	Ca_Kabuli_Chr07	9936854	(A/G)
12250	CakSNP12250	*Kabuli*	Ca_Kabuli_Chr07	9938937	(T/C)
12251	CakSNP12251	*Kabuli*	Ca_Kabuli_Chr07	10050020	(A/C)
12252	CakSNP12252	*Kabuli*	Ca_Kabuli_Chr07	10049998	(T/A)
12253	CakSNP12253	*Kabuli*	Ca_Kabuli_Chr07	10049996	(A/G)
12254	CakSNP12254	*Kabuli*	Ca_Kabuli_Chr07	10089428	(T/C)
12255	CakSNP12255	*Kabuli*	Ca_Kabuli_Chr07	10089539	(T/A)
12256	CakSNP12256	*Kabuli*	Ca_Kabuli_Chr07	10106084	(C/G)
12257	CakSNP12257	*Kabuli*	Ca_Kabuli_Chr07	10106065	(A/G)
12258	CakSNP12258	*Kabuli*	Ca_Kabuli_Chr07	10106054	(G/C)
12259	CakSNP12259	*Kabuli*	Ca_Kabuli_Chr07	10137842	(A/G)
12260	CakSNP12260	*Kabuli*	Ca_Kabuli_Chr07	10137890	(A/G)
12261	CakSNP12261	*Kabuli*	Ca_Kabuli_Chr07	10181630	(G/T)
12262	CakSNP12262	*Kabuli*	Ca_Kabuli_Chr07	10208029	(T/C)
12263	CakSNP12263	*Kabuli*	Ca_Kabuli_Chr07	10216813	(G/A)
12264	CakSNP12264	*Kabuli*	Ca_Kabuli_Chr07	10223046	(C/T)
12265	CakSNP12265	*Kabuli*	Ca_Kabuli_Chr07	10223068	(G/T)
12266	CakSNP12266	*Kabuli*	Ca_Kabuli_Chr07	10223069	(G/T)
12267	CakSNP12267	*Kabuli*	Ca_Kabuli_Chr07	10223071	(T/A)
12268	CakSNP12268	*Kabuli*	Ca_Kabuli_Chr07	10223074	(G/T)
12269	CakSNP12269	*Kabuli*	Ca_Kabuli_Chr07	10223075	(C/T)
12270	CakSNP12270	*Kabuli*	Ca_Kabuli_Chr07	10322726	(T/C)
12271	CakSNP12271	*Kabuli*	Ca_Kabuli_Chr07	10340199	(T/C)
12272	CakSNP12272	*Kabuli*	Ca_Kabuli_Chr07	10435983	(C/A)
12273	CakSNP12273	*Kabuli*	Ca_Kabuli_Chr07	10465140	(G/A)

S.N.	SNP IDs	Cultivars	Chromosomes/scaffolds	Physical positions (bp)	SNPs
12274	CakSNP12274	*Kabuli*	Ca_Kabuli_Chr07	10480401	(A/C)
12275	CakSNP12275	*Kabuli*	Ca_Kabuli_Chr07	10480462	(A/G)
12276	CakSNP12276	*Kabuli*	Ca_Kabuli_Chr07	10484200	(G/A)
12277	CakSNP12277	*Kabuli*	Ca_Kabuli_Chr07	10484225	(C/A)
12278	CakSNP12278	*Kabuli*	Ca_Kabuli_Chr07	10484196	(C/T)
12279	CakSNP12279	*Kabuli*	Ca_Kabuli_Chr07	10485414	(C/T)
12280	CakSNP12280	*Kabuli*	Ca_Kabuli_Chr07	10521423	(G/T)
12281	CakSNP12281	*Kabuli*	Ca_Kabuli_Chr07	10521449	(C/A)
12282	CakSNP12282	*Kabuli*	Ca_Kabuli_Chr07	10614159	(G/A)
12283	CakSNP12283	*Kabuli*	Ca_Kabuli_Chr07	10658367	(A/T)
12284	CakSNP12284	*Kabuli*	Ca_Kabuli_Chr07	10658446	(G/T)
12285	CakSNP12285	*Kabuli*	Ca_Kabuli_Chr07	10688189	(T/G)
12286	CakSNP12286	*Kabuli*	Ca_Kabuli_Chr07	10688401	(A/G)
12287	CakSNP12287	*Kabuli*	Ca_Kabuli_Chr07	10688464	(G/A)
12288	CakSNP12288	*Kabuli*	Ca_Kabuli_Chr07	10688434	(T/C)
12289	CakSNP12289	*Kabuli*	Ca_Kabuli_Chr07	10688499	(T/C)
12290	CakSNP12290	*Kabuli*	Ca_Kabuli_Chr07	10703569	(A/T)
12291	CakSNP12291	*Kabuli*	Ca_Kabuli_Chr07	10703612	(A/C)
12292	CakSNP12292	*Kabuli*	Ca_Kabuli_Chr07	10703704	(G/A)
12293	CakSNP12293	*Kabuli*	Ca_Kabuli_Chr07	10703679	(T/C)
12294	CakSNP12294	*Kabuli*	Ca_Kabuli_Chr07	10712202	(T/C)
12295	CakSNP12295	*Kabuli*	Ca_Kabuli_Chr07	10712231	(G/A)
12296	CakSNP12296	*Kabuli*	Ca_Kabuli_Chr07	10758973	(A/T)
12297	CakSNP12297	*Kabuli*	Ca_Kabuli_Chr07	10759142	(T/G)
12298	CakSNP12298	*Kabuli*	Ca_Kabuli_Chr07	10775881	(C/T)
12299	CakSNP12299	*Kabuli*	Ca_Kabuli_Chr07	10775875	(A/G)
12300	CakSNP12300	*Kabuli*	Ca_Kabuli_Chr07	10775851	(C/T)
12301	CakSNP12301	*Kabuli*	Ca_Kabuli_Chr07	10775860	(A/G)
12302	CakSNP12302	*Kabuli*	Ca_Kabuli_Chr07	10780680	(C/G)
12303	CakSNP12303	*Kabuli*	Ca_Kabuli_Chr07	10780682	(G/A)
12304	CakSNP12304	*Kabuli*	Ca_Kabuli_Chr07	10780686	(T/C)
12305	CakSNP12305	*Kabuli*	Ca_Kabuli_Chr07	10797204	(T/C)
12306	CakSNP12306	*Kabuli*	Ca_Kabuli_Chr07	10812617	(G/T)
12307	CakSNP12307	*Kabuli*	Ca_Kabuli_Chr07	10816523	(T/G)
12308	CakSNP12308	*Kabuli*	Ca_Kabuli_Chr07	10816674	(C/T)
12309	CakSNP12309	*Kabuli*	Ca_Kabuli_Chr07	10816662	(T/A)
12310	CakSNP12310	*Kabuli*	Ca_Kabuli_Chr07	10859907	(G/A)
12311	CakSNP12311	*Kabuli*	Ca_Kabuli_Chr07	10859842	(G/T)

S.N.	SNP IDs	Cultivars	Chromosomes/scaffolds	Physical positions (bp)	SNPs
12312	CakSNP12312	*Kabuli*	Ca_Kabuli_Chr07	10900910	(T/C)
12313	CakSNP12313	*Kabuli*	Ca_Kabuli_Chr07	11086047	(G/A)
12314	CakSNP12314	*Kabuli*	Ca_Kabuli_Chr07	11086071	(A/T)
12315	CakSNP12315	*Kabuli*	Ca_Kabuli_Chr07	11105847	(C/G)
12316	CakSNP12316	*Kabuli*	Ca_Kabuli_Chr07	11105961	(A/C)
12317	CakSNP12317	*Kabuli*	Ca_Kabuli_Chr07	11192667	(G/A)
12318	CakSNP12318	*Kabuli*	Ca_Kabuli_Chr07	11192712	(C/T)
12319	CakSNP12319	*Kabuli*	Ca_Kabuli_Chr07	11192703	(A/C)
12320	CakSNP12320	*Kabuli*	Ca_Kabuli_Chr07	11261924	(G/A)
12321	CakSNP12321	*Kabuli*	Ca_Kabuli_Chr07	11339511	(C/T)
12322	CakSNP12322	*Kabuli*	Ca_Kabuli_Chr07	11393795	(A/G)
12323	CakSNP12323	*Kabuli*	Ca_Kabuli_Chr07	11393986	(A/G)
12324	CakSNP12324	*Kabuli*	Ca_Kabuli_Chr07	11473766	(A/G)
12325	CakSNP12325	*Kabuli*	Ca_Kabuli_Chr07	11521694	(G/A)
12326	CakSNP12326	*Kabuli*	Ca_Kabuli_Chr07	11527428	(G/A)
12327	CakSNP12327	*Kabuli*	Ca_Kabuli_Chr07	11545846	(A/G)
12328	CakSNP12328	*Kabuli*	Ca_Kabuli_Chr07	11568963	(T/A)
12329	CakSNP12329	*Kabuli*	Ca_Kabuli_Chr07	11568945	(G/T)
12330	CakSNP12330	*Kabuli*	Ca_Kabuli_Chr07	11590987	(A/C)
12331	CakSNP12331	*Kabuli*	Ca_Kabuli_Chr07	11630509	(A/G)
12332	CakSNP12332	*Kabuli*	Ca_Kabuli_Chr07	11630511	(C/T)
12333	CakSNP12333	*Kabuli*	Ca_Kabuli_Chr07	11638885	(C/A)
12334	CakSNP12334	*Kabuli*	Ca_Kabuli_Chr07	11639117	(C/G)
12335	CakSNP12335	*Kabuli*	Ca_Kabuli_Chr07	11685818	(G/T)
12336	CakSNP12336	*Kabuli*	Ca_Kabuli_Chr07	11685899	(A/G)
12337	CakSNP12337	*Kabuli*	Ca_Kabuli_Chr07	11686238	(C/T)
12338	CakSNP12338	*Kabuli*	Ca_Kabuli_Chr07	11725655	(G/A)
12339	CakSNP12339	*Kabuli*	Ca_Kabuli_Chr07	11730926	(T/C)
12340	CakSNP12340	*Kabuli*	Ca_Kabuli_Chr07	11730946	(A/C)
12341	CakSNP12341	*Kabuli*	Ca_Kabuli_Chr07	11735177	(T/C)
12342	CakSNP12342	*Kabuli*	Ca_Kabuli_Chr07	11735381	(T/C)
12343	CakSNP12343	*Kabuli*	Ca_Kabuli_Chr07	11735368	(G/C)
12344	CakSNP12344	*Kabuli*	Ca_Kabuli_Chr07	11736006	(C/A)
12345	CakSNP12345	*Kabuli*	Ca_Kabuli_Chr07	11792042	(G/A)
12346	CakSNP12346	*Kabuli*	Ca_Kabuli_Chr07	11792126	(T/A)
12347	CakSNP12347	*Kabuli*	Ca_Kabuli_Chr07	11792136	(G/A)
12348	CakSNP12348	*Kabuli*	Ca_Kabuli_Chr07	11803091	(T/G)
12349	CakSNP12349	*Kabuli*	Ca_Kabuli_Chr07	11812410	(G/A)

S.N.	SNP IDs	Cultivars	Chromosomes/scaffolds	Physical positions (bp)	SNPs
12350	CakSNP12350	*Kabuli*	Ca_Kabuli_Chr07	11812441	(A/C)
12351	CakSNP12351	*Kabuli*	Ca_Kabuli_Chr07	11858425	(C/T)
12352	CakSNP12352	*Kabuli*	Ca_Kabuli_Chr07	11886954	(A/G)
12353	CakSNP12353	*Kabuli*	Ca_Kabuli_Chr07	11902897	(A/G)
12354	CakSNP12354	*Kabuli*	Ca_Kabuli_Chr07	11902985	(G/T)
12355	CakSNP12355	*Kabuli*	Ca_Kabuli_Chr07	12010432	(G/A)
12356	CakSNP12356	*Kabuli*	Ca_Kabuli_Chr07	12010471	(C/T)
12357	CakSNP12357	*Kabuli*	Ca_Kabuli_Chr07	12010504	(G/A)
12358	CakSNP12358	*Kabuli*	Ca_Kabuli_Chr07	12010499	(C/T)
12359	CakSNP12359	*Kabuli*	Ca_Kabuli_Chr07	12010496	(G/A)
12360	CakSNP12360	*Kabuli*	Ca_Kabuli_Chr07	12010494	(G/A)
12361	CakSNP12361	*Kabuli*	Ca_Kabuli_Chr07	12010491	(G/T)
12362	CakSNP12362	*Kabuli*	Ca_Kabuli_Chr07	12010483	(C/T)
12363	CakSNP12363	*Kabuli*	Ca_Kabuli_Chr07	12010457	(C/T)
12364	CakSNP12364	*Kabuli*	Ca_Kabuli_Chr07	12010544	(G/A)
12365	CakSNP12365	*Kabuli*	Ca_Kabuli_Chr07	12010489	(C/T)
12366	CakSNP12366	*Kabuli*	Ca_Kabuli_Chr07	12030080	(A/C)
12367	CakSNP12367	*Kabuli*	Ca_Kabuli_Chr07	12071764	(T/C)
12368	CakSNP12368	*Kabuli*	Ca_Kabuli_Chr07	12071773	(A/T)
12369	CakSNP12369	*Kabuli*	Ca_Kabuli_Chr07	12203726	(T/G)
12370	CakSNP12370	*Kabuli*	Ca_Kabuli_Chr07	12203722	(T/C)
12371	CakSNP12371	*Kabuli*	Ca_Kabuli_Chr07	12203706	(T/C)
12372	CakSNP12372	*Kabuli*	Ca_Kabuli_Chr07	12203690	(C/T)
12373	CakSNP12373	*Kabuli*	Ca_Kabuli_Chr07	12244234	(C/T)
12374	CakSNP12374	*Kabuli*	Ca_Kabuli_Chr07	12244259	(G/A)
12375	CakSNP12375	*Kabuli*	Ca_Kabuli_Chr07	12284289	(T/A)
12376	CakSNP12376	*Kabuli*	Ca_Kabuli_Chr07	12284304	(A/G)
12377	CakSNP12377	*Kabuli*	Ca_Kabuli_Chr07	12385785	(C/T)
12378	CakSNP12378	*Kabuli*	Ca_Kabuli_Chr07	12390774	(G/A)
12379	CakSNP12379	*Kabuli*	Ca_Kabuli_Chr07	12481556	(G/C)
12380	CakSNP12380	*Kabuli*	Ca_Kabuli_Chr07	12481627	(A/C)
12381	CakSNP12381	*Kabuli*	Ca_Kabuli_Chr07	12481715	(A/G)
12382	CakSNP12382	*Kabuli*	Ca_Kabuli_Chr07	12481835	(C/T)
12383	CakSNP12383	*Kabuli*	Ca_Kabuli_Chr07	12482851	(G/A)
12384	CakSNP12384	*Kabuli*	Ca_Kabuli_Chr07	12482822	(A/G)
12385	CakSNP12385	*Kabuli*	Ca_Kabuli_Chr07	12482953	(A/C)
12386	CakSNP12386	*Kabuli*	Ca_Kabuli_Chr07	12483290	(A/C)
12387	CakSNP12387	*Kabuli*	Ca_Kabuli_Chr07	12492075	(G/A)

S.N.	SNP IDs	Cultivars	Chromosomes/scaffolds	Physical positions (bp)	SNPs
12388	CakSNP12388	*Kabuli*	Ca_Kabuli_Chr07	12492052	(C/T)
12389	CakSNP12389	*Kabuli*	Ca_Kabuli_Chr07	12492046	(A/T)
12390	CakSNP12390	*Kabuli*	Ca_Kabuli_Chr07	12616871	(T/C)
12391	CakSNP12391	*Kabuli*	Ca_Kabuli_Chr07	12616870	(G/A)
12392	CakSNP12392	*Kabuli*	Ca_Kabuli_Chr07	12616957	(C/T)
12393	CakSNP12393	*Kabuli*	Ca_Kabuli_Chr07	12616937	(C/T)
12394	CakSNP12394	*Kabuli*	Ca_Kabuli_Chr07	12623129	(T/C)
12395	CakSNP12395	*Kabuli*	Ca_Kabuli_Chr07	12623128	(G/A)
12396	CakSNP12396	*Kabuli*	Ca_Kabuli_Chr07	12623215	(C/T)
12397	CakSNP12397	*Kabuli*	Ca_Kabuli_Chr07	12623195	(C/T)
12398	CakSNP12398	*Kabuli*	Ca_Kabuli_Chr07	12628148	(A/G)
12399	CakSNP12399	*Kabuli*	Ca_Kabuli_Chr07	12628211	(A/G)
12400	CakSNP12400	*Kabuli*	Ca_Kabuli_Chr07	12628265	(T/A)
12401	CakSNP12401	*Kabuli*	Ca_Kabuli_Chr07	12629986	(A/G)
12402	CakSNP12402	*Kabuli*	Ca_Kabuli_Chr07	12633590	(A/C)
12403	CakSNP12403	*Kabuli*	Ca_Kabuli_Chr07	12633642	(T/G)
12404	CakSNP12404	*Kabuli*	Ca_Kabuli_Chr07	12633643	(A/T)
12405	CakSNP12405	*Kabuli*	Ca_Kabuli_Chr07	12681440	(T/C)
12406	CakSNP12406	*Kabuli*	Ca_Kabuli_Chr07	12718820	(C/G)
12407	CakSNP12407	*Kabuli*	Ca_Kabuli_Chr07	12718853	(G/A)
12408	CakSNP12408	*Kabuli*	Ca_Kabuli_Chr07	12730600	(C/A)
12409	CakSNP12409	*Kabuli*	Ca_Kabuli_Chr07	12737516	(T/C)
12410	CakSNP12410	*Kabuli*	Ca_Kabuli_Chr07	12893655	(C/T)
12411	CakSNP12411	*Kabuli*	Ca_Kabuli_Chr07	12895182	(A/G)
12412	CakSNP12412	*Kabuli*	Ca_Kabuli_Chr07	12898640	(A/C)
12413	CakSNP12413	*Kabuli*	Ca_Kabuli_Chr07	12898709	(T/A)
12414	CakSNP12414	*Kabuli*	Ca_Kabuli_Chr07	12898707	(G/A)
12415	CakSNP12415	*Kabuli*	Ca_Kabuli_Chr07	12899398	(T/C)
12416	CakSNP12416	*Kabuli*	Ca_Kabuli_Chr07	12913811	(T/A)
12417	CakSNP12417	*Kabuli*	Ca_Kabuli_Chr07	13020788	(C/T)
12418	CakSNP12418	*Kabuli*	Ca_Kabuli_Chr07	13020772	(A/G)
12419	CakSNP12419	*Kabuli*	Ca_Kabuli_Chr07	13021047	(A/G)
12420	CakSNP12420	*Kabuli*	Ca_Kabuli_Chr07	13021030	(A/G)
12421	CakSNP12421	*Kabuli*	Ca_Kabuli_Chr07	13059710	(C/A)
12422	CakSNP12422	*Kabuli*	Ca_Kabuli_Chr07	13059712	(C/A)
12423	CakSNP12423	*Kabuli*	Ca_Kabuli_Chr07	13059717	(T/A)
12424	CakSNP12424	*Kabuli*	Ca_Kabuli_Chr07	13059852	(G/A)
12425	CakSNP12425	*Kabuli*	Ca_Kabuli_Chr07	13172542	(A/G)

S.N.	SNP IDs	Cultivars	Chromosomes/scaffolds	Physical positions (bp)	SNPs
12426	CakSNP12426	*Kabuli*	Ca_Kabuli_Chr07	13220439	(G/A)
12427	CakSNP12427	*Kabuli*	Ca_Kabuli_Chr07	13220418	(A/G)
12428	CakSNP12428	*Kabuli*	Ca_Kabuli_Chr07	13262430	(C/T)
12429	CakSNP12429	*Kabuli*	Ca_Kabuli_Chr07	13281255	(T/C)
12430	CakSNP12430	*Kabuli*	Ca_Kabuli_Chr07	13281290	(A/G)
12431	CakSNP12431	*Kabuli*	Ca_Kabuli_Chr07	13281297	(C/G)
12432	CakSNP12432	*Kabuli*	Ca_Kabuli_Chr07	13281307	(C/T)
12433	CakSNP12433	*Kabuli*	Ca_Kabuli_Chr07	13281313	(T/C)
12434	CakSNP12434	*Kabuli*	Ca_Kabuli_Chr07	13281314	(G/A)
12435	CakSNP12435	*Kabuli*	Ca_Kabuli_Chr07	13281374	(G/T)
12436	CakSNP12436	*Kabuli*	Ca_Kabuli_Chr07	13281351	(T/A)
12437	CakSNP12437	*Kabuli*	Ca_Kabuli_Chr07	13288828	(G/A)
12438	CakSNP12438	*Kabuli*	Ca_Kabuli_Chr07	13288813	(G/A)
12439	CakSNP12439	*Kabuli*	Ca_Kabuli_Chr07	13288753	(A/C)
12440	CakSNP12440	*Kabuli*	Ca_Kabuli_Chr07	13319871	(A/G)
12441	CakSNP12441	*Kabuli*	Ca_Kabuli_Chr07	13514074	(C/T)
12442	CakSNP12442	*Kabuli*	Ca_Kabuli_Chr07	13605200	(A/C)
12443	CakSNP12443	*Kabuli*	Ca_Kabuli_Chr07	13605191	(A/C)
12444	CakSNP12444	*Kabuli*	Ca_Kabuli_Chr07	13605188	(A/C)
12445	CakSNP12445	*Kabuli*	Ca_Kabuli_Chr07	13605578	(C/G)
12446	CakSNP12446	*Kabuli*	Ca_Kabuli_Chr07	13667102	(C/T)
12447	CakSNP12447	*Kabuli*	Ca_Kabuli_Chr07	13667103	(T/G)
12448	CakSNP12448	*Kabuli*	Ca_Kabuli_Chr07	13730350	(A/C)
12449	CakSNP12449	*Kabuli*	Ca_Kabuli_Chr07	13757159	(A/C)
12450	CakSNP12450	*Kabuli*	Ca_Kabuli_Chr07	13776110	(A/C)
12451	CakSNP12451	*Kabuli*	Ca_Kabuli_Chr07	13781815	(T/C)
12452	CakSNP12452	*Kabuli*	Ca_Kabuli_Chr07	13819387	(C/T)
12453	CakSNP12453	*Kabuli*	Ca_Kabuli_Chr07	13881348	(G/A)
12454	CakSNP12454	*Kabuli*	Ca_Kabuli_Chr07	13893199	(T/C)
12455	CakSNP12455	*Kabuli*	Ca_Kabuli_Chr07	13893344	(G/C)
12456	CakSNP12456	*Kabuli*	Ca_Kabuli_Chr07	13893382	(G/T)
12457	CakSNP12457	*Kabuli*	Ca_Kabuli_Chr07	13893383	(C/T)
12458	CakSNP12458	*Kabuli*	Ca_Kabuli_Chr07	13903980	(T/G)
12459	CakSNP12459	*Kabuli*	Ca_Kabuli_Chr07	13904163	(T/C)
12460	CakSNP12460	*Kabuli*	Ca_Kabuli_Chr07	13913735	(A/G)
12461	CakSNP12461	*Kabuli*	Ca_Kabuli_Chr07	13921214	(C/T)
12462	CakSNP12462	*Kabuli*	Ca_Kabuli_Chr07	13921289	(T/C)
12463	CakSNP12463	*Kabuli*	Ca_Kabuli_Chr07	13949259	(A/G)

S.N.	SNP IDs	Cultivars	Chromosomes/scaffolds	Physical positions (bp)	SNPs
12464	CakSNP12464	*Kabuli*	Ca_Kabuli_Chr07	13949314	(G/A)
12465	CakSNP12465	*Kabuli*	Ca_Kabuli_Chr07	13951874	(T/C)
12466	CakSNP12466	*Kabuli*	Ca_Kabuli_Chr07	13951856	(T/C)
12467	CakSNP12467	*Kabuli*	Ca_Kabuli_Chr07	14030136	(C/T)
12468	CakSNP12468	*Kabuli*	Ca_Kabuli_Chr07	14051146	(G/T)
12469	CakSNP12469	*Kabuli*	Ca_Kabuli_Chr07	14179629	(T/G)
12470	CakSNP12470	*Kabuli*	Ca_Kabuli_Chr07	14179733	(C/A)
12471	CakSNP12471	*Kabuli*	Ca_Kabuli_Chr07	14212570	(C/A)
12472	CakSNP12472	*Kabuli*	Ca_Kabuli_Chr07	14212597	(C/G)
12473	CakSNP12473	*Kabuli*	Ca_Kabuli_Chr07	14233343	(T/G)
12474	CakSNP12474	*Kabuli*	Ca_Kabuli_Chr07	14233368	(T/C)
12475	CakSNP12475	*Kabuli*	Ca_Kabuli_Chr07	14281993	(G/T)
12476	CakSNP12476	*Kabuli*	Ca_Kabuli_Chr07	14282088	(T/C)
12477	CakSNP12477	*Kabuli*	Ca_Kabuli_Chr07	14320216	(T/C)
12478	CakSNP12478	*Kabuli*	Ca_Kabuli_Chr07	14328655	(T/C)
12479	CakSNP12479	*Kabuli*	Ca_Kabuli_Chr07	14328649	(T/C)
12480	CakSNP12480	*Kabuli*	Ca_Kabuli_Chr07	14329126	(C/T)
12481	CakSNP12481	*Kabuli*	Ca_Kabuli_Chr07	14329175	(G/A)
12482	CakSNP12482	*Kabuli*	Ca_Kabuli_Chr07	14329196	(G/A)
12483	CakSNP12483	*Kabuli*	Ca_Kabuli_Chr07	14329217	(C/T)
12484	CakSNP12484	*Kabuli*	Ca_Kabuli_Chr07	14329167	(A/T)
12485	CakSNP12485	*Kabuli*	Ca_Kabuli_Chr07	14345827	(T/C)
12486	CakSNP12486	*Kabuli*	Ca_Kabuli_Chr07	14345799	(T/A)
12487	CakSNP12487	*Kabuli*	Ca_Kabuli_Chr07	14470549	(C/G)
12488	CakSNP12488	*Kabuli*	Ca_Kabuli_Chr07	14518292	(A/T)
12489	CakSNP12489	*Kabuli*	Ca_Kabuli_Chr07	14526546	(A/G)
12490	CakSNP12490	*Kabuli*	Ca_Kabuli_Chr07	14526495	(T/C)
12491	CakSNP12491	*Kabuli*	Ca_Kabuli_Chr07	14526493	(C/T)
12492	CakSNP12492	*Kabuli*	Ca_Kabuli_Chr07	14526480	(T/C)
12493	CakSNP12493	*Kabuli*	Ca_Kabuli_Chr07	14526478	(G/C)
12494	CakSNP12494	*Kabuli*	Ca_Kabuli_Chr07	14558741	(C/A)
12495	CakSNP12495	*Kabuli*	Ca_Kabuli_Chr07	14558785	(C/T)
12496	CakSNP12496	*Kabuli*	Ca_Kabuli_Chr07	14559033	(G/A)
12497	CakSNP12497	*Kabuli*	Ca_Kabuli_Chr07	14666535	(T/A)
12498	CakSNP12498	*Kabuli*	Ca_Kabuli_Chr07	14668939	(G/A)
12499	CakSNP12499	*Kabuli*	Ca_Kabuli_Chr07	14668922	(A/C)
12500	CakSNP12500	*Kabuli*	Ca_Kabuli_Chr07	14685011	(G/C)
12501	CakSNP12501	*Kabuli*	Ca_Kabuli_Chr07	14685017	(C/T)

S.N.	SNP IDs	Cultivars	Chromosomes/scaffolds	Physical positions (bp)	SNPs
12502	CakSNP12502	*Kabuli*	Ca_Kabuli_Chr07	14685024	(A/G)
12503	CakSNP12503	*Kabuli*	Ca_Kabuli_Chr07	14685034	(T/G)
12504	CakSNP12504	*Kabuli*	Ca_Kabuli_Chr07	14712518	(C/A)
12505	CakSNP12505	*Kabuli*	Ca_Kabuli_Chr07	14712568	(C/A)
12506	CakSNP12506	*Kabuli*	Ca_Kabuli_Chr07	14712596	(T/C)
12507	CakSNP12507	*Kabuli*	Ca_Kabuli_Chr07	14712601	(C/T)
12508	CakSNP12508	*Kabuli*	Ca_Kabuli_Chr07	14748257	(T/A)
12509	CakSNP12509	*Kabuli*	Ca_Kabuli_Chr07	14775135	(C/G)
12510	CakSNP12510	*Kabuli*	Ca_Kabuli_Chr07	14782453	(A/G)
12511	CakSNP12511	*Kabuli*	Ca_Kabuli_Chr07	14899028	(C/A)
12512	CakSNP12512	*Kabuli*	Ca_Kabuli_Chr07	14899101	(T/C)
12513	CakSNP12513	*Kabuli*	Ca_Kabuli_Chr07	14933294	(T/C)
12514	CakSNP12514	*Kabuli*	Ca_Kabuli_Chr07	14933286	(G/A)
12515	CakSNP12515	*Kabuli*	Ca_Kabuli_Chr07	14983364	(T/G)
12516	CakSNP12516	*Kabuli*	Ca_Kabuli_Chr07	14997794	(G/A)
12517	CakSNP12517	*Kabuli*	Ca_Kabuli_Chr07	14997883	(T/C)
12518	CakSNP12518	*Kabuli*	Ca_Kabuli_Chr07	15028182	(T/G)
12519	CakSNP12519	*Kabuli*	Ca_Kabuli_Chr07	15039606	(T/A)
12520	CakSNP12520	*Kabuli*	Ca_Kabuli_Chr07	15069817	(G/A)
12521	CakSNP12521	*Kabuli*	Ca_Kabuli_Chr07	15070091	(T/A)
12522	CakSNP12522	*Kabuli*	Ca_Kabuli_Chr07	15119205	(T/C)
12523	CakSNP12523	*Kabuli*	Ca_Kabuli_Chr07	15125638	(G/C)
12524	CakSNP12524	*Kabuli*	Ca_Kabuli_Chr07	15125619	(T/G)
12525	CakSNP12525	*Kabuli*	Ca_Kabuli_Chr07	15185603	(A/G)
12526	CakSNP12526	*Kabuli*	Ca_Kabuli_Chr07	15201600	(A/G)
12527	CakSNP12527	*Kabuli*	Ca_Kabuli_Chr07	15399421	(T/C)
12528	CakSNP12528	*Kabuli*	Ca_Kabuli_Chr07	15441316	(A/T)
12529	CakSNP12529	*Kabuli*	Ca_Kabuli_Chr07	15463183	(G/T)
12530	CakSNP12530	*Kabuli*	Ca_Kabuli_Chr07	15463170	(G/A)
12531	CakSNP12531	*Kabuli*	Ca_Kabuli_Chr07	15463169	(G/A)
12532	CakSNP12532	*Kabuli*	Ca_Kabuli_Chr07	15530329	(G/T)
12533	CakSNP12533	*Kabuli*	Ca_Kabuli_Chr07	15530316	(G/A)
12534	CakSNP12534	*Kabuli*	Ca_Kabuli_Chr07	15530315	(G/A)
12535	CakSNP12535	*Kabuli*	Ca_Kabuli_Chr07	15568060	(G/T)
12536	CakSNP12536	*Kabuli*	Ca_Kabuli_Chr07	15568047	(G/A)
12537	CakSNP12537	*Kabuli*	Ca_Kabuli_Chr07	15568046	(G/A)
12538	CakSNP12538	*Kabuli*	Ca_Kabuli_Chr07	15622247	(G/A)
12539	CakSNP12539	*Kabuli*	Ca_Kabuli_Chr07	15622245	(T/A)

S.N.	SNP IDs	Cultivars	Chromosomes/scaffolds	Physical positions (bp)	SNPs
12540	CakSNP12540	*Kabuli*	Ca_Kabuli_Chr07	15762068	(A/T)
12541	CakSNP12541	*Kabuli*	Ca_Kabuli_Chr07	15826588	(A/C)
12542	CakSNP12542	*Kabuli*	Ca_Kabuli_Chr07	15856789	(A/G)
12543	CakSNP12543	*Kabuli*	Ca_Kabuli_Chr07	15856955	(T/G)
12544	CakSNP12544	*Kabuli*	Ca_Kabuli_Chr07	15860298	(T/C)
12545	CakSNP12545	*Kabuli*	Ca_Kabuli_Chr07	15887790	(T/C)
12546	CakSNP12546	*Kabuli*	Ca_Kabuli_Chr07	15966680	(A/G)
12547	CakSNP12547	*Kabuli*	Ca_Kabuli_Chr07	15966714	(G/A)
12548	CakSNP12548	*Kabuli*	Ca_Kabuli_Chr07	16008746	(T/C)
12549	CakSNP12549	*Kabuli*	Ca_Kabuli_Chr07	16008733	(A/G)
12550	CakSNP12550	*Kabuli*	Ca_Kabuli_Chr07	16289811	(C/T)
12551	CakSNP12551	*Kabuli*	Ca_Kabuli_Chr07	16289802	(C/T)
12552	CakSNP12552	*Kabuli*	Ca_Kabuli_Chr07	16289794	(G/T)
12553	CakSNP12553	*Kabuli*	Ca_Kabuli_Chr07	16324985	(T/C)
12554	CakSNP12554	*Kabuli*	Ca_Kabuli_Chr07	16331943	(A/G)
12555	CakSNP12555	*Kabuli*	Ca_Kabuli_Chr07	16381854	(T/C)
12556	CakSNP12556	*Kabuli*	Ca_Kabuli_Chr07	16381891	(T/G)
12557	CakSNP12557	*Kabuli*	Ca_Kabuli_Chr07	16381934	(T/C)
12558	CakSNP12558	*Kabuli*	Ca_Kabuli_Chr07	16466619	(T/C)
12559	CakSNP12559	*Kabuli*	Ca_Kabuli_Chr07	16581695	(C/G)
12560	CakSNP12560	*Kabuli*	Ca_Kabuli_Chr07	16626047	(T/G)
12561	CakSNP12561	*Kabuli*	Ca_Kabuli_Chr07	16626703	(A/G)
12562	CakSNP12562	*Kabuli*	Ca_Kabuli_Chr07	16665577	(A/G)
12563	CakSNP12563	*Kabuli*	Ca_Kabuli_Chr07	16735437	(G/A)
12564	CakSNP12564	*Kabuli*	Ca_Kabuli_Chr07	16735427	(A/G)
12565	CakSNP12565	*Kabuli*	Ca_Kabuli_Chr07	16735423	(G/C)
12566	CakSNP12566	*Kabuli*	Ca_Kabuli_Chr07	16816296	(G/C)
12567	CakSNP12567	*Kabuli*	Ca_Kabuli_Chr07	16821274	(A/C)
12568	CakSNP12568	*Kabuli*	Ca_Kabuli_Chr07	16829081	(C/A)
12569	CakSNP12569	*Kabuli*	Ca_Kabuli_Chr07	16897329	(A/C)
12570	CakSNP12570	*Kabuli*	Ca_Kabuli_Chr07	16897285	(T/C)
12571	CakSNP12571	*Kabuli*	Ca_Kabuli_Chr07	16936123	(C/G)
12572	CakSNP12572	*Kabuli*	Ca_Kabuli_Chr07	17017466	(C/G)
12573	CakSNP12573	*Kabuli*	Ca_Kabuli_Chr07	17017453	(A/G)
12574	CakSNP12574	*Kabuli*	Ca_Kabuli_Chr07	17017447	(A/G)
12575	CakSNP12575	*Kabuli*	Ca_Kabuli_Chr07	17017438	(A/T)
12576	CakSNP12576	*Kabuli*	Ca_Kabuli_Chr07	17017421	(G/A)
12577	CakSNP12577	*Kabuli*	Ca_Kabuli_Chr07	17017418	(T/G)

S.N.	SNP IDs	Cultivars	Chromosomes/scaffolds	Physical positions (bp)	SNPs
12578	CakSNP12578	*Kabuli*	Ca_Kabuli_Chr07	17038317	(G/A)
12579	CakSNP12579	*Kabuli*	Ca_Kabuli_Chr07	17064968	(T/C)
12580	CakSNP12580	*Kabuli*	Ca_Kabuli_Chr07	17088603	(A/G)
12581	CakSNP12581	*Kabuli*	Ca_Kabuli_Chr07	17140664	(C/T)
12582	CakSNP12582	*Kabuli*	Ca_Kabuli_Chr07	17178847	(A/C)
12583	CakSNP12583	*Kabuli*	Ca_Kabuli_Chr07	17181160	(G/C)
12584	CakSNP12584	*Kabuli*	Ca_Kabuli_Chr07	17228904	(T/G)
12585	CakSNP12585	*Kabuli*	Ca_Kabuli_Chr07	17318174	(G/T)
12586	CakSNP12586	*Kabuli*	Ca_Kabuli_Chr07	17539110	(G/A)
12587	CakSNP12587	*Kabuli*	Ca_Kabuli_Chr07	17539136	(C/A)
12588	CakSNP12588	*Kabuli*	Ca_Kabuli_Chr07	17539207	(G/A)
12589	CakSNP12589	*Kabuli*	Ca_Kabuli_Chr07	17586154	(C/T)
12590	CakSNP12590	*Kabuli*	Ca_Kabuli_Chr07	17591798	(T/A)
12591	CakSNP12591	*Kabuli*	Ca_Kabuli_Chr07	17591781	(G/A)
12592	CakSNP12592	*Kabuli*	Ca_Kabuli_Chr07	17591843	(G/T)
12593	CakSNP12593	*Kabuli*	Ca_Kabuli_Chr07	17591974	(A/G)
12594	CakSNP12594	*Kabuli*	Ca_Kabuli_Chr07	17591971	(A/G)
12595	CakSNP12595	*Kabuli*	Ca_Kabuli_Chr07	17591924	(T/C)
12596	CakSNP12596	*Kabuli*	Ca_Kabuli_Chr07	17592701	(C/A)
12597	CakSNP12597	*Kabuli*	Ca_Kabuli_Chr07	17597348	(T/C)
12598	CakSNP12598	*Kabuli*	Ca_Kabuli_Chr07	17597421	(A/G)
12599	CakSNP12599	*Kabuli*	Ca_Kabuli_Chr07	17597420	(C/T)
12600	CakSNP12600	*Kabuli*	Ca_Kabuli_Chr07	17624143	(G/A)
12601	CakSNP12601	*Kabuli*	Ca_Kabuli_Chr07	17624225	(T/C)
12602	CakSNP12602	*Kabuli*	Ca_Kabuli_Chr07	17766520	(T/C)
12603	CakSNP12603	*Kabuli*	Ca_Kabuli_Chr07	17766952	(T/A)
12604	CakSNP12604	*Kabuli*	Ca_Kabuli_Chr07	17766914	(C/A)
12605	CakSNP12605	*Kabuli*	Ca_Kabuli_Chr07	17779414	(A/G)
12606	CakSNP12606	*Kabuli*	Ca_Kabuli_Chr07	17779594	(T/C)
12607	CakSNP12607	*Kabuli*	Ca_Kabuli_Chr07	17779700	(A/T)
12608	CakSNP12608	*Kabuli*	Ca_Kabuli_Chr07	17782558	(C/T)
12609	CakSNP12609	*Kabuli*	Ca_Kabuli_Chr07	18011604	(G/C)
12610	CakSNP12610	*Kabuli*	Ca_Kabuli_Chr07	18090289	(T/A)
12611	CakSNP12611	*Kabuli*	Ca_Kabuli_Chr07	18265803	(A/C)
12612	CakSNP12612	*Kabuli*	Ca_Kabuli_Chr07	18265791	(G/C)
12613	CakSNP12613	*Kabuli*	Ca_Kabuli_Chr07	18265782	(G/C)
12614	CakSNP12614	*Kabuli*	Ca_Kabuli_Chr07	18319049	(T/G)
12615	CakSNP12615	*Kabuli*	Ca_Kabuli_Chr07	18319073	(T/G)

S.N.	SNP IDs	Cultivars	Chromosomes/scaffolds	Physical positions (bp)	SNPs
12616	CakSNP12616	*Kabuli*	Ca_Kabuli_Chr07	18365218	(G/A)
12617	CakSNP12617	*Kabuli*	Ca_Kabuli_Chr07	18430468	(C/T)
12618	CakSNP12618	*Kabuli*	Ca_Kabuli_Chr07	18469372	(G/A)
12619	CakSNP12619	*Kabuli*	Ca_Kabuli_Chr07	18469422	(C/T)
12620	CakSNP12620	*Kabuli*	Ca_Kabuli_Chr07	18638565	(A/G)
12621	CakSNP12621	*Kabuli*	Ca_Kabuli_Chr07	18642538	(A/G)
12622	CakSNP12622	*Kabuli*	Ca_Kabuli_Chr07	18658563	(C/G)
12623	CakSNP12623	*Kabuli*	Ca_Kabuli_Chr07	18696645	(G/A)
12624	CakSNP12624	*Kabuli*	Ca_Kabuli_Chr07	18796748	(T/C)
12625	CakSNP12625	*Kabuli*	Ca_Kabuli_Chr07	18796768	(A/G)
12626	CakSNP12626	*Kabuli*	Ca_Kabuli_Chr07	18910468	(A/G)
12627	CakSNP12627	*Kabuli*	Ca_Kabuli_Chr07	19042183	(C/T)
12628	CakSNP12628	*Kabuli*	Ca_Kabuli_Chr07	19189737	(A/C)
12629	CakSNP12629	*Kabuli*	Ca_Kabuli_Chr07	19217501	(A/C)
12630	CakSNP12630	*Kabuli*	Ca_Kabuli_Chr07	19217598	(G/C)
12631	CakSNP12631	*Kabuli*	Ca_Kabuli_Chr07	19232818	(T/A)
12632	CakSNP12632	*Kabuli*	Ca_Kabuli_Chr07	19290521	(G/A)
12633	CakSNP12633	*Kabuli*	Ca_Kabuli_Chr07	19470527	(T/C)
12634	CakSNP12634	*Kabuli*	Ca_Kabuli_Chr07	19472719	(G/A)
12635	CakSNP12635	*Kabuli*	Ca_Kabuli_Chr07	19472689	(T/A)
12636	CakSNP12636	*Kabuli*	Ca_Kabuli_Chr07	19542901	(A/G)
12637	CakSNP12637	*Kabuli*	Ca_Kabuli_Chr07	19549636	(C/T)
12638	CakSNP12638	*Kabuli*	Ca_Kabuli_Chr07	19707812	(T/A)
12639	CakSNP12639	*Kabuli*	Ca_Kabuli_Chr07	19707842	(G/A)
12640	CakSNP12640	*Kabuli*	Ca_Kabuli_Chr07	19818789	(C/A)
12641	CakSNP12641	*Kabuli*	Ca_Kabuli_Chr07	19818770	(T/C)
12642	CakSNP12642	*Kabuli*	Ca_Kabuli_Chr07	19818759	(G/C)
12643	CakSNP12643	*Kabuli*	Ca_Kabuli_Chr07	19818757	(G/C)
12644	CakSNP12644	*Kabuli*	Ca_Kabuli_Chr07	20031805	(T/C)
12645	CakSNP12645	*Kabuli*	Ca_Kabuli_Chr07	20032205	(A/C)
12646	CakSNP12646	*Kabuli*	Ca_Kabuli_Chr07	20082842	(T/C)
12647	CakSNP12647	*Kabuli*	Ca_Kabuli_Chr07	20083040	(A/T)
12648	CakSNP12648	*Kabuli*	Ca_Kabuli_Chr07	20099384	(C/T)
12649	CakSNP12649	*Kabuli*	Ca_Kabuli_Chr07	20111019	(T/C)
12650	CakSNP12650	*Kabuli*	Ca_Kabuli_Chr07	20111123	(T/C)
12651	CakSNP12651	*Kabuli*	Ca_Kabuli_Chr07	20112897	(G/A)
12652	CakSNP12652	*Kabuli*	Ca_Kabuli_Chr07	20135647	(C/G)
12653	CakSNP12653	*Kabuli*	Ca_Kabuli_Chr07	20168870	(T/A)

S.N.	SNP IDs	Cultivars	Chromosomes/scaffolds	Physical positions (bp)	SNPs
12654	CakSNP12654	*Kabuli*	Ca_Kabuli_Chr07	20252133	(T/C)
12655	CakSNP12655	*Kabuli*	Ca_Kabuli_Chr07	20252134	(G/A)
12656	CakSNP12656	*Kabuli*	Ca_Kabuli_Chr07	20252135	(A/G)
12657	CakSNP12657	*Kabuli*	Ca_Kabuli_Chr07	20252144	(G/A)
12658	CakSNP12658	*Kabuli*	Ca_Kabuli_Chr07	20252145	(A/G)
12659	CakSNP12659	*Kabuli*	Ca_Kabuli_Chr07	20252181	(C/T)
12660	CakSNP12660	*Kabuli*	Ca_Kabuli_Chr07	20252192	(G/A)
12661	CakSNP12661	*Kabuli*	Ca_Kabuli_Chr07	20252201	(C/A)
12662	CakSNP12662	*Kabuli*	Ca_Kabuli_Chr07	20252212	(C/T)
12663	CakSNP12663	*Kabuli*	Ca_Kabuli_Chr07	20252214	(C/G)
12664	CakSNP12664	*Kabuli*	Ca_Kabuli_Chr07	20252236	(G/A)
12665	CakSNP12665	*Kabuli*	Ca_Kabuli_Chr07	20252234	(A/C)
12666	CakSNP12666	*Kabuli*	Ca_Kabuli_Chr07	20252233	(C/T)
12667	CakSNP12667	*Kabuli*	Ca_Kabuli_Chr07	20252221	(T/C)
12668	CakSNP12668	*Kabuli*	Ca_Kabuli_Chr07	20252209	(G/A)
12669	CakSNP12669	*Kabuli*	Ca_Kabuli_Chr07	20252309	(C/T)
12670	CakSNP12670	*Kabuli*	Ca_Kabuli_Chr07	20252334	(A/T)
12671	CakSNP12671	*Kabuli*	Ca_Kabuli_Chr07	20252340	(A/G)
12672	CakSNP12672	*Kabuli*	Ca_Kabuli_Chr07	20362883	(G/A)
12673	CakSNP12673	*Kabuli*	Ca_Kabuli_Chr07	20362879	(C/T)
12674	CakSNP12674	*Kabuli*	Ca_Kabuli_Chr07	20362876	(G/A)
12675	CakSNP12675	*Kabuli*	Ca_Kabuli_Chr07	20362838	(T/C)
12676	CakSNP12676	*Kabuli*	Ca_Kabuli_Chr07	20414653	(G/A)
12677	CakSNP12677	*Kabuli*	Ca_Kabuli_Chr07	20414703	(C/A)
12678	CakSNP12678	*Kabuli*	Ca_Kabuli_Chr07	20486247	(T/G)
12679	CakSNP12679	*Kabuli*	Ca_Kabuli_Chr07	20486280	(G/C)
12680	CakSNP12680	*Kabuli*	Ca_Kabuli_Chr07	20496378	(G/A)
12681	CakSNP12681	*Kabuli*	Ca_Kabuli_Chr07	20496303	(C/T)
12682	CakSNP12682	*Kabuli*	Ca_Kabuli_Chr07	20496438	(C/T)
12683	CakSNP12683	*Kabuli*	Ca_Kabuli_Chr07	20498552	(T/C)
12684	CakSNP12684	*Kabuli*	Ca_Kabuli_Chr07	20498626	(T/G)
12685	CakSNP12685	*Kabuli*	Ca_Kabuli_Chr07	20540780	(C/T)
12686	CakSNP12686	*Kabuli*	Ca_Kabuli_Chr07	20540791	(G/A)
12687	CakSNP12687	*Kabuli*	Ca_Kabuli_Chr07	20543119	(T/C)
12688	CakSNP12688	*Kabuli*	Ca_Kabuli_Chr07	20550563	(G/A)
12689	CakSNP12689	*Kabuli*	Ca_Kabuli_Chr07	20550575	(C/G)
12690	CakSNP12690	*Kabuli*	Ca_Kabuli_Chr07	20552041	(G/A)
12691	CakSNP12691	*Kabuli*	Ca_Kabuli_Chr07	20552053	(C/G)

S.N.	SNP IDs	Cultivars	Chromosomes/scaffolds	Physical positions (bp)	SNPs
12692	CakSNP12692	*Kabuli*	Ca_Kabuli_Chr07	20556466	(G/C)
12693	CakSNP12693	*Kabuli*	Ca_Kabuli_Chr07	20556576	(C/T)
12694	CakSNP12694	*Kabuli*	Ca_Kabuli_Chr07	20563931	(G/A)
12695	CakSNP12695	*Kabuli*	Ca_Kabuli_Chr07	20564024	(G/A)
12696	CakSNP12696	*Kabuli*	Ca_Kabuli_Chr07	20564266	(G/A)
12697	CakSNP12697	*Kabuli*	Ca_Kabuli_Chr07	20564269	(C/T)
12698	CakSNP12698	*Kabuli*	Ca_Kabuli_Chr07	20564297	(A/G)
12699	CakSNP12699	*Kabuli*	Ca_Kabuli_Chr07	20579963	(G/T)
12700	CakSNP12700	*Kabuli*	Ca_Kabuli_Chr07	20579974	(A/T)
12701	CakSNP12701	*Kabuli*	Ca_Kabuli_Chr07	20585997	(T/G)
12702	CakSNP12702	*Kabuli*	Ca_Kabuli_Chr07	20605389	(G/A)
12703	CakSNP12703	*Kabuli*	Ca_Kabuli_Chr07	20605384	(C/G)
12704	CakSNP12704	*Kabuli*	Ca_Kabuli_Chr07	20605373	(C/A)
12705	CakSNP12705	*Kabuli*	Ca_Kabuli_Chr07	20633040	(A/G)
12706	CakSNP12706	*Kabuli*	Ca_Kabuli_Chr07	20637152	(G/A)
12707	CakSNP12707	*Kabuli*	Ca_Kabuli_Chr07	20917747	(A/G)
12708	CakSNP12708	*Kabuli*	Ca_Kabuli_Chr07	20917743	(T/G)
12709	CakSNP12709	*Kabuli*	Ca_Kabuli_Chr07	20917740	(A/G)
12710	CakSNP12710	*Kabuli*	Ca_Kabuli_Chr07	21073385	(A/C)
12711	CakSNP12711	*Kabuli*	Ca_Kabuli_Chr07	21081969	(A/G)
12712	CakSNP12712	*Kabuli*	Ca_Kabuli_Chr07	21099782	(G/T)
12713	CakSNP12713	*Kabuli*	Ca_Kabuli_Chr07	21402075	(A/G)
12714	CakSNP12714	*Kabuli*	Ca_Kabuli_Chr07	21402117	(A/C)
12715	CakSNP12715	*Kabuli*	Ca_Kabuli_Chr07	21595525	(C/T)
12716	CakSNP12716	*Kabuli*	Ca_Kabuli_Chr07	21595527	(T/G)
12717	CakSNP12717	*Kabuli*	Ca_Kabuli_Chr07	21616285	(A/G)
12718	CakSNP12718	*Kabuli*	Ca_Kabuli_Chr07	21616287	(T/G)
12719	CakSNP12719	*Kabuli*	Ca_Kabuli_Chr07	21616294	(T/G)
12720	CakSNP12720	*Kabuli*	Ca_Kabuli_Chr07	21626752	(T/C)
12721	CakSNP12721	*Kabuli*	Ca_Kabuli_Chr07	21633103	(C/G)
12722	CakSNP12722	*Kabuli*	Ca_Kabuli_Chr07	21639234	(G/A)
12723	CakSNP12723	*Kabuli*	Ca_Kabuli_Chr07	21639238	(G/A)
12724	CakSNP12724	*Kabuli*	Ca_Kabuli_Chr07	21639244	(T/G)
12725	CakSNP12725	*Kabuli*	Ca_Kabuli_Chr07	21639251	(T/G)
12726	CakSNP12726	*Kabuli*	Ca_Kabuli_Chr07	21639254	(T/G)
12727	CakSNP12727	*Kabuli*	Ca_Kabuli_Chr07	21639375	(T/G)
12728	CakSNP12728	*Kabuli*	Ca_Kabuli_Chr07	21644279	(C/T)
12729	CakSNP12729	*Kabuli*	Ca_Kabuli_Chr07	21644342	(G/A)

S.N.	SNP IDs	Cultivars	Chromosomes/scaffolds	Physical positions (bp)	SNPs
12730	CakSNP12730	*Kabuli*	Ca_Kabuli_Chr07	21713147	(T/G)
12731	CakSNP12731	*Kabuli*	Ca_Kabuli_Chr07	21744514	(T/C)
12732	CakSNP12732	*Kabuli*	Ca_Kabuli_Chr07	21918052	(G/C)
12733	CakSNP12733	*Kabuli*	Ca_Kabuli_Chr07	22707297	(T/C)
12734	CakSNP12734	*Kabuli*	Ca_Kabuli_Chr07	22721356	(A/C)
12735	CakSNP12735	*Kabuli*	Ca_Kabuli_Chr07	22738152	(C/G)
12736	CakSNP12736	*Kabuli*	Ca_Kabuli_Chr07	22738307	(G/C)
12737	CakSNP12737	*Kabuli*	Ca_Kabuli_Chr07	22738482	(A/G)
12738	CakSNP12738	*Kabuli*	Ca_Kabuli_Chr07	22808696	(A/G)
12739	CakSNP12739	*Kabuli*	Ca_Kabuli_Chr07	22808695	(C/T)
12740	CakSNP12740	*Kabuli*	Ca_Kabuli_Chr07	22808687	(C/T)
12741	CakSNP12741	*Kabuli*	Ca_Kabuli_Chr07	22808717	(C/T)
12742	CakSNP12742	*Kabuli*	Ca_Kabuli_Chr07	22808679	(A/C)
12743	CakSNP12743	*Kabuli*	Ca_Kabuli_Chr07	22839776	(G/A)
12744	CakSNP12744	*Kabuli*	Ca_Kabuli_Chr07	22875652	(A/G)
12745	CakSNP12745	*Kabuli*	Ca_Kabuli_Chr07	23087696	(T/C)
12746	CakSNP12746	*Kabuli*	Ca_Kabuli_Chr07	23087732	(T/C)
12747	CakSNP12747	*Kabuli*	Ca_Kabuli_Chr07	23439214	(A/G)
12748	CakSNP12748	*Kabuli*	Ca_Kabuli_Chr07	23439225	(C/A)
12749	CakSNP12749	*Kabuli*	Ca_Kabuli_Chr07	23574823	(C/T)
12750	CakSNP12750	*Kabuli*	Ca_Kabuli_Chr07	23575629	(G/A)
12751	CakSNP12751	*Kabuli*	Ca_Kabuli_Chr07	23616050	(A/G)
12752	CakSNP12752	*Kabuli*	Ca_Kabuli_Chr07	24021690	(T/A)
12753	CakSNP12753	*Kabuli*	Ca_Kabuli_Chr07	24181321	(G/A)
12754	CakSNP12754	*Kabuli*	Ca_Kabuli_Chr07	24190633	(G/A)
12755	CakSNP12755	*Kabuli*	Ca_Kabuli_Chr07	24190636	(G/A)
12756	CakSNP12756	*Kabuli*	Ca_Kabuli_Chr07	24190645	(A/T)
12757	CakSNP12757	*Kabuli*	Ca_Kabuli_Chr07	24190650	(G/A)
12758	CakSNP12758	*Kabuli*	Ca_Kabuli_Chr07	24190671	(C/T)
12759	CakSNP12759	*Kabuli*	Ca_Kabuli_Chr07	24245096	(C/T)
12760	CakSNP12760	*Kabuli*	Ca_Kabuli_Chr07	24415819	(T/A)
12761	CakSNP12761	*Kabuli*	Ca_Kabuli_Chr07	25218407	(C/T)
12762	CakSNP12762	*Kabuli*	Ca_Kabuli_Chr07	25362272	(T/C)
12763	CakSNP12763	*Kabuli*	Ca_Kabuli_Chr07	25428518	(T/C)
12764	CakSNP12764	*Kabuli*	Ca_Kabuli_Chr07	25428525	(C/G)
12765	CakSNP12765	*Kabuli*	Ca_Kabuli_Chr07	25428548	(G/T)
12766	CakSNP12766	*Kabuli*	Ca_Kabuli_Chr07	25428566	(T/C)
12767	CakSNP12767	*Kabuli*	Ca_Kabuli_Chr07	25428660	(G/A)

S.N.	SNP IDs	Cultivars	Chromosomes/scaffolds	Physical positions (bp)	SNPs
12768	CakSNP12768	*Kabuli*	Ca_Kabuli_Chr07	25569665	(T/G)
12769	CakSNP12769	*Kabuli*	Ca_Kabuli_Chr07	25569657	(T/C)
12770	CakSNP12770	*Kabuli*	Ca_Kabuli_Chr07	25740866	(G/T)
12771	CakSNP12771	*Kabuli*	Ca_Kabuli_Chr07	26070513	(G/T)
12772	CakSNP12772	*Kabuli*	Ca_Kabuli_Chr07	26070515	(A/G)
12773	CakSNP12773	*Kabuli*	Ca_Kabuli_Chr07	26094578	(T/C)
12774	CakSNP12774	*Kabuli*	Ca_Kabuli_Chr07	26094572	(C/T)
12775	CakSNP12775	*Kabuli*	Ca_Kabuli_Chr07	26094562	(G/T)
12776	CakSNP12776	*Kabuli*	Ca_Kabuli_Chr07	26094567	(G/A)
12777	CakSNP12777	*Kabuli*	Ca_Kabuli_Chr07	26094609	(C/T)
12778	CakSNP12778	*Kabuli*	Ca_Kabuli_Chr07	26137315	(T/C)
12779	CakSNP12779	*Kabuli*	Ca_Kabuli_Chr07	26146779	(C/T)
12780	CakSNP12780	*Kabuli*	Ca_Kabuli_Chr07	26226146	(G/C)
12781	CakSNP12781	*Kabuli*	Ca_Kabuli_Chr07	26487440	(A/C)
12782	CakSNP12782	*Kabuli*	Ca_Kabuli_Chr07	26692131	(T/G)
12783	CakSNP12783	*Kabuli*	Ca_Kabuli_Chr07	26692130	(C/T)
12784	CakSNP12784	*Kabuli*	Ca_Kabuli_Chr07	26692128	(A/C)
12785	CakSNP12785	*Kabuli*	Ca_Kabuli_Chr07	26692194	(C/A)
12786	CakSNP12786	*Kabuli*	Ca_Kabuli_Chr07	26692195	(G/T)
12787	CakSNP12787	*Kabuli*	Ca_Kabuli_Chr07	26694365	(G/A)
12788	CakSNP12788	*Kabuli*	Ca_Kabuli_Chr07	26694440	(C/A)
12789	CakSNP12789	*Kabuli*	Ca_Kabuli_Chr07	26694441	(G/A)
12790	CakSNP12790	*Kabuli*	Ca_Kabuli_Chr07	26897878	(A/G)
12791	CakSNP12791	*Kabuli*	Ca_Kabuli_Chr07	26897910	(A/G)
12792	CakSNP12792	*Kabuli*	Ca_Kabuli_Chr07	26897920	(A/G)
12793	CakSNP12793	*Kabuli*	Ca_Kabuli_Chr07	26897923	(G/A)
12794	CakSNP12794	*Kabuli*	Ca_Kabuli_Chr07	26897929	(T/C)
12795	CakSNP12795	*Kabuli*	Ca_Kabuli_Chr07	26897940	(A/G)
12796	CakSNP12796	*Kabuli*	Ca_Kabuli_Chr07	26897948	(A/T)
12797	CakSNP12797	*Kabuli*	Ca_Kabuli_Chr07	26940917	(G/A)
12798	CakSNP12798	*Kabuli*	Ca_Kabuli_Chr07	26940967	(A/G)
12799	CakSNP12799	*Kabuli*	Ca_Kabuli_Chr07	26940979	(A/G)
12800	CakSNP12800	*Kabuli*	Ca_Kabuli_Chr07	26940985	(T/G)
12801	CakSNP12801	*Kabuli*	Ca_Kabuli_Chr07	27050259	(C/G)
12802	CakSNP12802	*Kabuli*	Ca_Kabuli_Chr07	27176481	(C/A)
12803	CakSNP12803	*Kabuli*	Ca_Kabuli_Chr07	27210919	(C/T)
12804	CakSNP12804	*Kabuli*	Ca_Kabuli_Chr07	27210881	(C/T)
12805	CakSNP12805	*Kabuli*	Ca_Kabuli_Chr07	27210871	(C/A)

S.N.	SNP IDs	Cultivars	Chromosomes/scaffolds	Physical positions (bp)	SNPs
12806	CakSNP12806	*Kabuli*	Ca_Kabuli_Chr07	27210865	(T/C)
12807	CakSNP12807	*Kabuli*	Ca_Kabuli_Chr07	27210858	(C/T)
12808	CakSNP12808	*Kabuli*	Ca_Kabuli_Chr07	27210853	(A/G)
12809	CakSNP12809	*Kabuli*	Ca_Kabuli_Chr07	27210848	(A/T)
12810	CakSNP12810	*Kabuli*	Ca_Kabuli_Chr07	27210951	(T/C)
12811	CakSNP12811	*Kabuli*	Ca_Kabuli_Chr07	27210945	(C/T)
12812	CakSNP12812	*Kabuli*	Ca_Kabuli_Chr07	27210988	(C/T)
12813	CakSNP12813	*Kabuli*	Ca_Kabuli_Chr07	27210989	(C/T)
12814	CakSNP12814	*Kabuli*	Ca_Kabuli_Chr07	27210992	(C/A)
12815	CakSNP12815	*Kabuli*	Ca_Kabuli_Chr07	27211004	(T/C)
12816	CakSNP12816	*Kabuli*	Ca_Kabuli_Chr07	27211036	(G/A)
12817	CakSNP12817	*Kabuli*	Ca_Kabuli_Chr07	27211051	(C/A)
12818	CakSNP12818	*Kabuli*	Ca_Kabuli_Chr07	27211084	(G/T)
12819	CakSNP12819	*Kabuli*	Ca_Kabuli_Chr07	27211073	(C/T)
12820	CakSNP12820	*Kabuli*	Ca_Kabuli_Chr07	27211060	(C/T)
12821	CakSNP12821	*Kabuli*	Ca_Kabuli_Chr07	27633960	(C/A)
12822	CakSNP12822	*Kabuli*	Ca_Kabuli_Chr07	27735234	(A/G)
12823	CakSNP12823	*Kabuli*	Ca_Kabuli_Chr07	27899782	(T/C)
12824	CakSNP12824	*Kabuli*	Ca_Kabuli_Chr07	27899743	(G/A)
12825	CakSNP12825	*Kabuli*	Ca_Kabuli_Chr07	27971186	(T/G)
12826	CakSNP12826	*Kabuli*	Ca_Kabuli_Chr07	28676139	(T/C)
12827	CakSNP12827	*Kabuli*	Ca_Kabuli_Chr07	28931212	(A/C)
12828	CakSNP12828	*Kabuli*	Ca_Kabuli_Chr07	28931223	(C/A)
12829	CakSNP12829	*Kabuli*	Ca_Kabuli_Chr07	29150927	(A/C)
12830	CakSNP12830	*Kabuli*	Ca_Kabuli_Chr07	29153994	(T/C)
12831	CakSNP12831	*Kabuli*	Ca_Kabuli_Chr07	29154220	(C/A)
12832	CakSNP12832	*Kabuli*	Ca_Kabuli_Chr07	29157286	(A/G)
12833	CakSNP12833	*Kabuli*	Ca_Kabuli_Chr07	29168982	(G/A)
12834	CakSNP12834	*Kabuli*	Ca_Kabuli_Chr07	29168980	(C/T)
12835	CakSNP12835	*Kabuli*	Ca_Kabuli_Chr07	29190659	(T/G)
12836	CakSNP12836	*Kabuli*	Ca_Kabuli_Chr07	29225385	(T/G)
12837	CakSNP12837	*Kabuli*	Ca_Kabuli_Chr07	29225351	(T/G)
12838	CakSNP12838	*Kabuli*	Ca_Kabuli_Chr07	29225348	(G/C)
12839	CakSNP12839	*Kabuli*	Ca_Kabuli_Chr07	29229965	(A/G)
12840	CakSNP12840	*Kabuli*	Ca_Kabuli_Chr07	29313114	(G/T)
12841	CakSNP12841	*Kabuli*	Ca_Kabuli_Chr07	29313080	(T/C)
12842	CakSNP12842	*Kabuli*	Ca_Kabuli_Chr07	29313067	(G/T)
12843	CakSNP12843	*Kabuli*	Ca_Kabuli_Chr07	29313059	(G/A)

S.N.	SNP IDs	Cultivars	Chromosomes/scaffolds	Physical positions (bp)	SNPs
12844	CakSNP12844	*Kabuli*	Ca_Kabuli_Chr07	29313058	(T/C)
12845	CakSNP12845	*Kabuli*	Ca_Kabuli_Chr07	29313051	(T/C)
12846	CakSNP12846	*Kabuli*	Ca_Kabuli_Chr07	29313257	(G/A)
12847	CakSNP12847	*Kabuli*	Ca_Kabuli_Chr07	29434818	(T/C)
12848	CakSNP12848	*Kabuli*	Ca_Kabuli_Chr07	29482556	(G/A)
12849	CakSNP12849	*Kabuli*	Ca_Kabuli_Chr07	29484304	(G/A)
12850	CakSNP12850	*Kabuli*	Ca_Kabuli_Chr07	29607943	(C/T)
12851	CakSNP12851	*Kabuli*	Ca_Kabuli_Chr07	29613699	(C/T)
12852	CakSNP12852	*Kabuli*	Ca_Kabuli_Chr07	29625884	(T/C)
12853	CakSNP12853	*Kabuli*	Ca_Kabuli_Chr07	29705531	(T/G)
12854	CakSNP12854	*Kabuli*	Ca_Kabuli_Chr07	29705499	(T/C)
12855	CakSNP12855	*Kabuli*	Ca_Kabuli_Chr07	29796564	(G/T)
12856	CakSNP12856	*Kabuli*	Ca_Kabuli_Chr07	29801148	(A/G)
12857	CakSNP12857	*Kabuli*	Ca_Kabuli_Chr07	29801154	(T/C)
12858	CakSNP12858	*Kabuli*	Ca_Kabuli_Chr07	29837035	(G/A)
12859	CakSNP12859	*Kabuli*	Ca_Kabuli_Chr07	29838073	(C/T)
12860	CakSNP12860	*Kabuli*	Ca_Kabuli_Chr07	29848248	(T/C)
12861	CakSNP12861	*Kabuli*	Ca_Kabuli_Chr07	29848433	(C/T)
12862	CakSNP12862	*Kabuli*	Ca_Kabuli_Chr07	29849509	(C/T)
12863	CakSNP12863	*Kabuli*	Ca_Kabuli_Chr07	29849544	(A/G)
12864	CakSNP12864	*Kabuli*	Ca_Kabuli_Chr07	29884719	(G/T)
12865	CakSNP12865	*Kabuli*	Ca_Kabuli_Chr07	29884718	(C/A)
12866	CakSNP12866	*Kabuli*	Ca_Kabuli_Chr07	29925008	(G/A)
12867	CakSNP12867	*Kabuli*	Ca_Kabuli_Chr07	30004767	(C/T)
12868	CakSNP12868	*Kabuli*	Ca_Kabuli_Chr07	30004934	(A/C)
12869	CakSNP12869	*Kabuli*	Ca_Kabuli_Chr07	30026039	(T/G)
12870	CakSNP12870	*Kabuli*	Ca_Kabuli_Chr07	30026017	(C/T)
12871	CakSNP12871	*Kabuli*	Ca_Kabuli_Chr07	30029793	(A/C)
12872	CakSNP12872	*Kabuli*	Ca_Kabuli_Chr07	30029820	(T/C)
12873	CakSNP12873	*Kabuli*	Ca_Kabuli_Chr07	30060299	(C/A)
12874	CakSNP12874	*Kabuli*	Ca_Kabuli_Chr07	30085847	(C/T)
12875	CakSNP12875	*Kabuli*	Ca_Kabuli_Chr07	30085883	(T/G)
12876	CakSNP12876	*Kabuli*	Ca_Kabuli_Chr07	30085906	(C/T)
12877	CakSNP12877	*Kabuli*	Ca_Kabuli_Chr07	30085910	(T/A)
12878	CakSNP12878	*Kabuli*	Ca_Kabuli_Chr07	30099003	(C/T)
12879	CakSNP12879	*Kabuli*	Ca_Kabuli_Chr07	30107340	(A/G)
12880	CakSNP12880	*Kabuli*	Ca_Kabuli_Chr07	30398855	(A/C)
12881	CakSNP12881	*Kabuli*	Ca_Kabuli_Chr07	30403615	(A/G)

S.N.	SNP IDs	Cultivars	Chromosomes/scaffolds	Physical positions (bp)	SNPs
12882	CakSNP12882	*Kabuli*	Ca_Kabuli_Chr07	30403618	(G/A)
12883	CakSNP12883	*Kabuli*	Ca_Kabuli_Chr07	30403700	(C/T)
12884	CakSNP12884	*Kabuli*	Ca_Kabuli_Chr07	30403692	(G/A)
12885	CakSNP12885	*Kabuli*	Ca_Kabuli_Chr07	30403621	(G/T)
12886	CakSNP12886	*Kabuli*	Ca_Kabuli_Chr07	30691324	(A/C)
12887	CakSNP12887	*Kabuli*	Ca_Kabuli_Chr07	30691318	(A/C)
12888	CakSNP12888	*Kabuli*	Ca_Kabuli_Chr07	30768315	(G/A)
12889	CakSNP12889	*Kabuli*	Ca_Kabuli_Chr07	30850950	(A/C)
12890	CakSNP12890	*Kabuli*	Ca_Kabuli_Chr07	31190059	(G/A)
12891	CakSNP12891	*Kabuli*	Ca_Kabuli_Chr07	31193786	(T/G)
12892	CakSNP12892	*Kabuli*	Ca_Kabuli_Chr07	31314421	(T/A)
12893	CakSNP12893	*Kabuli*	Ca_Kabuli_Chr07	31324279	(A/G)
12894	CakSNP12894	*Kabuli*	Ca_Kabuli_Chr07	31401807	(T/C)
12895	CakSNP12895	*Kabuli*	Ca_Kabuli_Chr07	31442425	(A/C)
12896	CakSNP12896	*Kabuli*	Ca_Kabuli_Chr07	31612697	(T/A)
12897	CakSNP12897	*Kabuli*	Ca_Kabuli_Chr07	31639701	(C/T)
12898	CakSNP12898	*Kabuli*	Ca_Kabuli_Chr07	31830238	(A/G)
12899	CakSNP12899	*Kabuli*	Ca_Kabuli_Chr07	31851096	(T/A)
12900	CakSNP12900	*Kabuli*	Ca_Kabuli_Chr07	31873079	(G/T)
12901	CakSNP12901	*Kabuli*	Ca_Kabuli_Chr07	31996345	(A/C)
12902	CakSNP12902	*Kabuli*	Ca_Kabuli_Chr07	32017294	(G/A)
12903	CakSNP12903	*Kabuli*	Ca_Kabuli_Chr07	32017288	(G/T)
12904	CakSNP12904	*Kabuli*	Ca_Kabuli_Chr07	32017286	(T/G)
12905	CakSNP12905	*Kabuli*	Ca_Kabuli_Chr07	32017284	(C/T)
12906	CakSNP12906	*Kabuli*	Ca_Kabuli_Chr07	32017253	(G/A)
12907	CakSNP12907	*Kabuli*	Ca_Kabuli_Chr07	32017252	(G/A)
12908	CakSNP12908	*Kabuli*	Ca_Kabuli_Chr07	32017238	(C/T)
12909	CakSNP12909	*Kabuli*	Ca_Kabuli_Chr07	32017228	(G/A)
12910	CakSNP12910	*Kabuli*	Ca_Kabuli_Chr07	32017221	(C/T)
12911	CakSNP12911	*Kabuli*	Ca_Kabuli_Chr07	32017211	(C/A)
12912	CakSNP12912	*Kabuli*	Ca_Kabuli_Chr07	32127288	(T/A)
12913	CakSNP12913	*Kabuli*	Ca_Kabuli_Chr07	32283585	(T/G)
12914	CakSNP12914	*Kabuli*	Ca_Kabuli_Chr07	32309397	(A/T)
12915	CakSNP12915	*Kabuli*	Ca_Kabuli_Chr07	32309411	(G/T)
12916	CakSNP12916	*Kabuli*	Ca_Kabuli_Chr07	32309415	(C/T)
12917	CakSNP12917	*Kabuli*	Ca_Kabuli_Chr07	32309429	(C/T)
12918	CakSNP12918	*Kabuli*	Ca_Kabuli_Chr07	32309377	(C/T)
12919	CakSNP12919	*Kabuli*	Ca_Kabuli_Chr07	32309376	(C/G)

S.N.	SNP IDs	Cultivars	Chromosomes/scaffolds	Physical positions (bp)	SNPs
12920	CakSNP12920	*Kabuli*	Ca_Kabuli_Chr07	32399759	(C/T)
12921	CakSNP12921	*Kabuli*	Ca_Kabuli_Chr07	32404932	(T/C)
12922	CakSNP12922	*Kabuli*	Ca_Kabuli_Chr07	32451377	(A/C)
12923	CakSNP12923	*Kabuli*	Ca_Kabuli_Chr07	32522201	(A/G)
12924	CakSNP12924	*Kabuli*	Ca_Kabuli_Chr07	32522203	(A/G)
12925	CakSNP12925	*Kabuli*	Ca_Kabuli_Chr07	32522207	(T/G)
12926	CakSNP12926	*Kabuli*	Ca_Kabuli_Chr07	32522213	(A/G)
12927	CakSNP12927	*Kabuli*	Ca_Kabuli_Chr07	32522230	(T/G)
12928	CakSNP12928	*Kabuli*	Ca_Kabuli_Chr07	32557599	(G/A)
12929	CakSNP12929	*Kabuli*	Ca_Kabuli_Chr07	32669570	(A/G)
12930	CakSNP12930	*Kabuli*	Ca_Kabuli_Chr07	32754633	(T/G)
12931	CakSNP12931	*Kabuli*	Ca_Kabuli_Chr07	32755647	(A/C)
12932	CakSNP12932	*Kabuli*	Ca_Kabuli_Chr07	32755662	(G/T)
12933	CakSNP12933	*Kabuli*	Ca_Kabuli_Chr07	32784903	(T/G)
12934	CakSNP12934	*Kabuli*	Ca_Kabuli_Chr07	32799037	(A/G)
12935	CakSNP12935	*Kabuli*	Ca_Kabuli_Chr07	32807457	(T/A)
12936	CakSNP12936	*Kabuli*	Ca_Kabuli_Chr07	32807499	(A/G)
12937	CakSNP12937	*Kabuli*	Ca_Kabuli_Chr07	32874847	(C/T)
12938	CakSNP12938	*Kabuli*	Ca_Kabuli_Chr07	32874840	(C/T)
12939	CakSNP12939	*Kabuli*	Ca_Kabuli_Chr07	32875799	(T/A)
12940	CakSNP12940	*Kabuli*	Ca_Kabuli_Chr07	32942199	(A/G)
12941	CakSNP12941	*Kabuli*	Ca_Kabuli_Chr07	32942951	(T/G)
12942	CakSNP12942	*Kabuli*	Ca_Kabuli_Chr07	32989796	(T/A)
12943	CakSNP12943	*Kabuli*	Ca_Kabuli_Chr07	32989838	(C/T)
12944	CakSNP12944	*Kabuli*	Ca_Kabuli_Chr07	32989903	(A/G)
12945	CakSNP12945	*Kabuli*	Ca_Kabuli_Chr07	32989902	(C/A)
12946	CakSNP12946	*Kabuli*	Ca_Kabuli_Chr07	33065611	(G/T)
12947	CakSNP12947	*Kabuli*	Ca_Kabuli_Chr07	33121674	(G/C)
12948	CakSNP12948	*Kabuli*	Ca_Kabuli_Chr07	33121640	(C/A)
12949	CakSNP12949	*Kabuli*	Ca_Kabuli_Chr07	33122473	(A/C)
12950	CakSNP12950	*Kabuli*	Ca_Kabuli_Chr07	33182932	(G/A)
12951	CakSNP12951	*Kabuli*	Ca_Kabuli_Chr07	33267485	(T/C)
12952	CakSNP12952	*Kabuli*	Ca_Kabuli_Chr07	33267496	(T/A)
12953	CakSNP12953	*Kabuli*	Ca_Kabuli_Chr07	33335978	(C/A)
12954	CakSNP12954	*Kabuli*	Ca_Kabuli_Chr07	33364329	(C/T)
12955	CakSNP12955	*Kabuli*	Ca_Kabuli_Chr07	33364354	(G/A)
12956	CakSNP12956	*Kabuli*	Ca_Kabuli_Chr07	33378427	(C/T)
12957	CakSNP12957	*Kabuli*	Ca_Kabuli_Chr07	33411746	(C/A)

S.N.	SNP IDs	Cultivars	Chromosomes/scaffolds	Physical positions (bp)	SNPs
12958	CakSNP12958	*Kabuli*	Ca_Kabuli_Chr07	33465828	(C/A)
12959	CakSNP12959	*Kabuli*	Ca_Kabuli_Chr07	33465818	(G/A)
12960	CakSNP12960	*Kabuli*	Ca_Kabuli_Chr07	33465805	(T/C)
12961	CakSNP12961	*Kabuli*	Ca_Kabuli_Chr07	33465802	(C/A)
12962	CakSNP12962	*Kabuli*	Ca_Kabuli_Chr07	33465800	(T/A)
12963	CakSNP12963	*Kabuli*	Ca_Kabuli_Chr07	33465781	(G/T)
12964	CakSNP12964	*Kabuli*	Ca_Kabuli_Chr07	33465855	(A/C)
12965	CakSNP12965	*Kabuli*	Ca_Kabuli_Chr07	33480940	(C/T)
12966	CakSNP12966	*Kabuli*	Ca_Kabuli_Chr07	33573605	(C/T)
12967	CakSNP12967	*Kabuli*	Ca_Kabuli_Chr07	33573655	(A/C)
12968	CakSNP12968	*Kabuli*	Ca_Kabuli_Chr07	33619268	(G/C)
12969	CakSNP12969	*Kabuli*	Ca_Kabuli_Chr07	33641799	(G/T)
12970	CakSNP12970	*Kabuli*	Ca_Kabuli_Chr07	33641882	(A/T)
12971	CakSNP12971	*Kabuli*	Ca_Kabuli_Chr07	33674454	(T/C)
12972	CakSNP12972	*Kabuli*	Ca_Kabuli_Chr07	33744521	(C/G)
12973	CakSNP12973	*Kabuli*	Ca_Kabuli_Chr07	33753917	(G/A)
12974	CakSNP12974	*Kabuli*	Ca_Kabuli_Chr07	33761944	(G/A)
12975	CakSNP12975	*Kabuli*	Ca_Kabuli_Chr07	33762053	(T/G)
12976	CakSNP12976	*Kabuli*	Ca_Kabuli_Chr07	33821658	(C/T)
12977	CakSNP12977	*Kabuli*	Ca_Kabuli_Chr07	33865463	(T/C)
12978	CakSNP12978	*Kabuli*	Ca_Kabuli_Chr07	33865475	(G/A)
12979	CakSNP12979	*Kabuli*	Ca_Kabuli_Chr07	33867334	(C/T)
12980	CakSNP12980	*Kabuli*	Ca_Kabuli_Chr07	33867491	(C/T)
12981	CakSNP12981	*Kabuli*	Ca_Kabuli_Chr07	33867674	(C/T)
12982	CakSNP12982	*Kabuli*	Ca_Kabuli_Chr07	33869367	(G/A)
12983	CakSNP12983	*Kabuli*	Ca_Kabuli_Chr07	33879559	(C/G)
12984	CakSNP12984	*Kabuli*	Ca_Kabuli_Chr07	33910324	(C/A)
12985	CakSNP12985	*Kabuli*	Ca_Kabuli_Chr07	33910870	(C/A)
12986	CakSNP12986	*Kabuli*	Ca_Kabuli_Chr07	33912719	(T/G)
12987	CakSNP12987	*Kabuli*	Ca_Kabuli_Chr07	33912766	(G/T)
12988	CakSNP12988	*Kabuli*	Ca_Kabuli_Chr07	33912866	(T/C)
12989	CakSNP12989	*Kabuli*	Ca_Kabuli_Chr07	33912899	(T/A)
12990	CakSNP12990	*Kabuli*	Ca_Kabuli_Chr07	33912902	(A/T)
12991	CakSNP12991	*Kabuli*	Ca_Kabuli_Chr07	33912913	(A/T)
12992	CakSNP12992	*Kabuli*	Ca_Kabuli_Chr07	33912916	(G/T)
12993	CakSNP12993	*Kabuli*	Ca_Kabuli_Chr07	33912921	(T/G)
12994	CakSNP12994	*Kabuli*	Ca_Kabuli_Chr07	33912950	(C/T)
12995	CakSNP12995	*Kabuli*	Ca_Kabuli_Chr07	33912966	(T/C)

S.N.	SNP IDs	Cultivars	Chromosomes/scaffolds	Physical positions (bp)	SNPs
12996	CakSNP12996	*Kabuli*	Ca_Kabuli_Chr07	33913094	(A/G)
12997	CakSNP12997	*Kabuli*	Ca_Kabuli_Chr07	33913047	(C/T)
12998	CakSNP12998	*Kabuli*	Ca_Kabuli_Chr07	33913045	(G/T)
12999	CakSNP12999	*Kabuli*	Ca_Kabuli_Chr07	34107603	(C/A)
13000	CakSNP13000	*Kabuli*	Ca_Kabuli_Chr07	34152124	(G/C)
13001	CakSNP13001	*Kabuli*	Ca_Kabuli_Chr07	34271486	(A/G)
13002	CakSNP13002	*Kabuli*	Ca_Kabuli_Chr07	34277014	(G/T)
13003	CakSNP13003	*Kabuli*	Ca_Kabuli_Chr07	34277010	(A/G)
13004	CakSNP13004	*Kabuli*	Ca_Kabuli_Chr07	34279673	(G/A)
13005	CakSNP13005	*Kabuli*	Ca_Kabuli_Chr07	34279725	(A/G)
13006	CakSNP13006	*Kabuli*	Ca_Kabuli_Chr07	34279715	(A/C)
13007	CakSNP13007	*Kabuli*	Ca_Kabuli_Chr07	34318860	(C/T)
13008	CakSNP13008	*Kabuli*	Ca_Kabuli_Chr07	34318862	(A/G)
13009	CakSNP13009	*Kabuli*	Ca_Kabuli_Chr07	34474614	(A/C)
13010	CakSNP13010	*Kabuli*	Ca_Kabuli_Chr07	34801839	(A/T)
13011	CakSNP13011	*Kabuli*	Ca_Kabuli_Chr07	34857693	(C/T)
13012	CakSNP13012	*Kabuli*	Ca_Kabuli_Chr07	34947743	(A/C)
13013	CakSNP13013	*Kabuli*	Ca_Kabuli_Chr07	35016516	(G/C)
13014	CakSNP13014	*Kabuli*	Ca_Kabuli_Chr07	35072425	(A/T)
13015	CakSNP13015	*Kabuli*	Ca_Kabuli_Chr07	35072407	(G/A)
13016	CakSNP13016	*Kabuli*	Ca_Kabuli_Chr07	35072461	(A/T)
13017	CakSNP13017	*Kabuli*	Ca_Kabuli_Chr07	35074345	(T/A)
13018	CakSNP13018	*Kabuli*	Ca_Kabuli_Chr07	35074348	(T/C)
13019	CakSNP13019	*Kabuli*	Ca_Kabuli_Chr07	35074362	(T/C)
13020	CakSNP13020	*Kabuli*	Ca_Kabuli_Chr07	35112785	(T/C)
13021	CakSNP13021	*Kabuli*	Ca_Kabuli_Chr07	35114049	(C/T)
13022	CakSNP13022	*Kabuli*	Ca_Kabuli_Chr07	35134809	(A/C)
13023	CakSNP13023	*Kabuli*	Ca_Kabuli_Chr07	35134863	(T/C)
13024	CakSNP13024	*Kabuli*	Ca_Kabuli_Chr07	35181619	(T/G)
13025	CakSNP13025	*Kabuli*	Ca_Kabuli_Chr07	35181661	(T/C)
13026	CakSNP13026	*Kabuli*	Ca_Kabuli_Chr07	35181660	(G/A)
13027	CakSNP13027	*Kabuli*	Ca_Kabuli_Chr07	35181635	(G/C)
13028	CakSNP13028	*Kabuli*	Ca_Kabuli_Chr07	35227794	(A/C)
13029	CakSNP13029	*Kabuli*	Ca_Kabuli_Chr07	35258029	(T/G)
13030	CakSNP13030	*Kabuli*	Ca_Kabuli_Chr07	35263711	(A/T)
13031	CakSNP13031	*Kabuli*	Ca_Kabuli_Chr07	35263712	(G/T)
13032	CakSNP13032	*Kabuli*	Ca_Kabuli_Chr07	35263736	(C/G)
13033	CakSNP13033	*Kabuli*	Ca_Kabuli_Chr07	35263758	(A/G)

S.N.	SNP IDs	Cultivars	Chromosomes/scaffolds	Physical positions (bp)	SNPs
13034	CakSNP13034	*Kabuli*	Ca_Kabuli_Chr07	35274368	(A/G)
13035	CakSNP13035	*Kabuli*	Ca_Kabuli_Chr07	35274859	(T/G)
13036	CakSNP13036	*Kabuli*	Ca_Kabuli_Chr07	35274858	(G/A)
13037	CakSNP13037	*Kabuli*	Ca_Kabuli_Chr07	35274828	(A/G)
13038	CakSNP13038	*Kabuli*	Ca_Kabuli_Chr07	35279675	(A/G)
13039	CakSNP13039	*Kabuli*	Ca_Kabuli_Chr07	35280189	(G/A)
13040	CakSNP13040	*Kabuli*	Ca_Kabuli_Chr07	35280167	(A/G)
13041	CakSNP13041	*Kabuli*	Ca_Kabuli_Chr07	35576427	(C/A)
13042	CakSNP13042	*Kabuli*	Ca_Kabuli_Chr07	35619530	(G/A)
13043	CakSNP13043	*Kabuli*	Ca_Kabuli_Chr07	35619487	(G/A)
13044	CakSNP13044	*Kabuli*	Ca_Kabuli_Chr07	35619518	(G/C)
13045	CakSNP13045	*Kabuli*	Ca_Kabuli_Chr07	35622168	(A/G)
13046	CakSNP13046	*Kabuli*	Ca_Kabuli_Chr07	35637056	(T/C)
13047	CakSNP13047	*Kabuli*	Ca_Kabuli_Chr07	35680423	(A/C)
13048	CakSNP13048	*Kabuli*	Ca_Kabuli_Chr07	35799096	(A/C)
13049	CakSNP13049	*Kabuli*	Ca_Kabuli_Chr07	35799083	(T/A)
13050	CakSNP13050	*Kabuli*	Ca_Kabuli_Chr07	35799074	(G/C)
13051	CakSNP13051	*Kabuli*	Ca_Kabuli_Chr07	35799073	(T/C)
13052	CakSNP13052	*Kabuli*	Ca_Kabuli_Chr07	35807000	(A/G)
13053	CakSNP13053	*Kabuli*	Ca_Kabuli_Chr07	35806999	(C/G)
13054	CakSNP13054	*Kabuli*	Ca_Kabuli_Chr07	35806990	(A/T)
13055	CakSNP13055	*Kabuli*	Ca_Kabuli_Chr07	35806977	(T/G)
13056	CakSNP13056	*Kabuli*	Ca_Kabuli_Chr07	35911620	(T/G)
13057	CakSNP13057	*Kabuli*	Ca_Kabuli_Chr07	35911617	(A/C)
13058	CakSNP13058	*Kabuli*	Ca_Kabuli_Chr07	35911615	(C/T)
13059	CakSNP13059	*Kabuli*	Ca_Kabuli_Chr07	35911612	(T/G)
13060	CakSNP13060	*Kabuli*	Ca_Kabuli_Chr07	35911606	(T/C)
13061	CakSNP13061	*Kabuli*	Ca_Kabuli_Chr07	35947529	(T/C)
13062	CakSNP13062	*Kabuli*	Ca_Kabuli_Chr07	35947550	(C/T)
13063	CakSNP13063	*Kabuli*	Ca_Kabuli_Chr07	35947551	(A/G)
13064	CakSNP13064	*Kabuli*	Ca_Kabuli_Chr07	35959805	(C/T)
13065	CakSNP13065	*Kabuli*	Ca_Kabuli_Chr07	35959825	(G/A)
13066	CakSNP13066	*Kabuli*	Ca_Kabuli_Chr07	35959878	(G/A)
13067	CakSNP13067	*Kabuli*	Ca_Kabuli_Chr07	35959917	(C/T)
13068	CakSNP13068	*Kabuli*	Ca_Kabuli_Chr07	35959865	(T/C)
13069	CakSNP13069	*Kabuli*	Ca_Kabuli_Chr07	36711954	(C/A)
13070	CakSNP13070	*Kabuli*	Ca_Kabuli_Chr07	36715249	(T/C)
13071	CakSNP13071	*Kabuli*	Ca_Kabuli_Chr07	36745439	(T/G)

S.N.	SNP IDs	Cultivars	Chromosomes/scaffolds	Physical positions (bp)	SNPs
13072	CakSNP13072	*Kabuli*	Ca_Kabuli_Chr07	36916272	(T/G)
13073	CakSNP13073	*Kabuli*	Ca_Kabuli_Chr07	36916273	(T/A)
13074	CakSNP13074	*Kabuli*	Ca_Kabuli_Chr07	36998096	(T/C)
13075	CakSNP13075	*Kabuli*	Ca_Kabuli_Chr07	36998113	(C/T)
13076	CakSNP13076	*Kabuli*	Ca_Kabuli_Chr07	37058979	(C/T)
13077	CakSNP13077	*Kabuli*	Ca_Kabuli_Chr07	37187439	(A/G)
13078	CakSNP13078	*Kabuli*	Ca_Kabuli_Chr07	37626729	(G/T)
13079	CakSNP13079	*Kabuli*	Ca_Kabuli_Chr07	37626653	(A/C)
13080	CakSNP13080	*Kabuli*	Ca_Kabuli_Chr07	37626687	(A/G)
13081	CakSNP13081	*Kabuli*	Ca_Kabuli_Chr07	37626666	(C/T)
13082	CakSNP13082	*Kabuli*	Ca_Kabuli_Chr07	37703967	(G/A)
13083	CakSNP13083	*Kabuli*	Ca_Kabuli_Chr07	37703966	(C/T)
13084	CakSNP13084	*Kabuli*	Ca_Kabuli_Chr07	37703965	(C/T)
13085	CakSNP13085	*Kabuli*	Ca_Kabuli_Chr07	37703921	(G/T)
13086	CakSNP13086	*Kabuli*	Ca_Kabuli_Chr07	37703912	(G/A)
13087	CakSNP13087	*Kabuli*	Ca_Kabuli_Chr07	37703913	(C/G)
13088	CakSNP13088	*Kabuli*	Ca_Kabuli_Chr07	37703936	(C/T)
13089	CakSNP13089	*Kabuli*	Ca_Kabuli_Chr07	37857936	(G/T)
13090	CakSNP13090	*Kabuli*	Ca_Kabuli_Chr07	37857928	(A/T)
13091	CakSNP13091	*Kabuli*	Ca_Kabuli_Chr07	37857917	(G/A)
13092	CakSNP13092	*Kabuli*	Ca_Kabuli_Chr07	37857885	(C/T)
13093	CakSNP13093	*Kabuli*	Ca_Kabuli_Chr07	38047374	(A/G)
13094	CakSNP13094	*Kabuli*	Ca_Kabuli_Chr07	38047378	(C/A)
13095	CakSNP13095	*Kabuli*	Ca_Kabuli_Chr07	38143361	(A/G)
13096	CakSNP13096	*Kabuli*	Ca_Kabuli_Chr07	38143524	(T/A)
13097	CakSNP13097	*Kabuli*	Ca_Kabuli_Chr07	38143452	(G/A)
13098	CakSNP13098	*Kabuli*	Ca_Kabuli_Chr07	38460464	(G/A)
13099	CakSNP13099	*Kabuli*	Ca_Kabuli_Chr07	38460452	(C/T)
13100	CakSNP13100	*Kabuli*	Ca_Kabuli_Chr07	38460425	(G/A)
13101	CakSNP13101	*Kabuli*	Ca_Kabuli_Chr07	38460394	(C/A)
13102	CakSNP13102	*Kabuli*	Ca_Kabuli_Chr07	39181880	(G/A)
13103	CakSNP13103	*Kabuli*	Ca_Kabuli_Chr07	39182564	(A/T)
13104	CakSNP13104	*Kabuli*	Ca_Kabuli_Chr07	39339510	(T/G)
13105	CakSNP13105	*Kabuli*	Ca_Kabuli_Chr07	39339509	(G/A)
13106	CakSNP13106	*Kabuli*	Ca_Kabuli_Chr07	39339508	(G/A)
13107	CakSNP13107	*Kabuli*	Ca_Kabuli_Chr07	39339503	(C/A)
13108	CakSNP13108	*Kabuli*	Ca_Kabuli_Chr07	39339502	(C/A)
13109	CakSNP13109	*Kabuli*	Ca_Kabuli_Chr07	39339501	(G/C)

S.N.	SNP IDs	Cultivars	Chromosomes/scaffolds	Physical positions (bp)	SNPs
13110	CakSNP13110	*Kabuli*	Ca_Kabuli_Chr07	39339500	(G/T)
13111	CakSNP13111	*Kabuli*	Ca_Kabuli_Chr07	39339499	(C/T)
13112	CakSNP13112	*Kabuli*	Ca_Kabuli_Chr07	39339498	(G/T)
13113	CakSNP13113	*Kabuli*	Ca_Kabuli_Chr07	39339495	(G/T)
13114	CakSNP13114	*Kabuli*	Ca_Kabuli_Chr07	39399280	(T/C)
13115	CakSNP13115	*Kabuli*	Ca_Kabuli_Chr07	39680221	(A/C)
13116	CakSNP13116	*Kabuli*	Ca_Kabuli_Chr07	39680222	(G/A)
13117	CakSNP13117	*Kabuli*	Ca_Kabuli_Chr07	39768248	(T/C)
13118	CakSNP13118	*Kabuli*	Ca_Kabuli_Chr07	39944444	(T/C)
13119	CakSNP13119	*Kabuli*	Ca_Kabuli_Chr07	39944937	(T/C)
13120	CakSNP13120	*Kabuli*	Ca_Kabuli_Chr07	39944889	(T/C)
13121	CakSNP13121	*Kabuli*	Ca_Kabuli_Chr07	40092198	(G/A)
13122	CakSNP13122	*Kabuli*	Ca_Kabuli_Chr07	40092181	(C/T)
13123	CakSNP13123	*Kabuli*	Ca_Kabuli_Chr07	40092218	(A/T)
13124	CakSNP13124	*Kabuli*	Ca_Kabuli_Chr07	40092213	(G/A)
13125	CakSNP13125	*Kabuli*	Ca_Kabuli_Chr07	40092163	(T/C)
13126	CakSNP13126	*Kabuli*	Ca_Kabuli_Chr07	40092155	(T/A)
13127	CakSNP13127	*Kabuli*	Ca_Kabuli_Chr07	40092156	(C/A)
13128	CakSNP13128	*Kabuli*	Ca_Kabuli_Chr07	40092168	(G/A)
13129	CakSNP13129	*Kabuli*	Ca_Kabuli_Chr07	40092201	(G/A)
13130	CakSNP13130	*Kabuli*	Ca_Kabuli_Chr07	40092203	(T/G)
13131	CakSNP13131	*Kabuli*	Ca_Kabuli_Chr07	40092211	(C/T)
13132	CakSNP13132	*Kabuli*	Ca_Kabuli_Chr07	40092214	(C/G)
13133	CakSNP13133	*Kabuli*	Ca_Kabuli_Chr07	40092229	(C/A)
13134	CakSNP13134	*Kabuli*	Ca_Kabuli_Chr07	40092237	(C/T)
13135	CakSNP13135	*Kabuli*	Ca_Kabuli_Chr07	40092239	(G/A)
13136	CakSNP13136	*Kabuli*	Ca_Kabuli_Chr07	40092250	(A/C)
13137	CakSNP13137	*Kabuli*	Ca_Kabuli_Chr07	40092253	(C/T)
13138	CakSNP13138	*Kabuli*	Ca_Kabuli_Chr07	40092256	(T/C)
13139	CakSNP13139	*Kabuli*	Ca_Kabuli_Chr07	40092267	(G/C)
13140	CakSNP13140	*Kabuli*	Ca_Kabuli_Chr07	40183449	(C/A)
13141	CakSNP13141	*Kabuli*	Ca_Kabuli_Chr07	40183452	(A/C)
13142	CakSNP13142	*Kabuli*	Ca_Kabuli_Chr07	40241674	(A/C)
13143	CakSNP13143	*Kabuli*	Ca_Kabuli_Chr07	40242539	(A/G)
13144	CakSNP13144	*Kabuli*	Ca_Kabuli_Chr07	40242765	(A/G)
13145	CakSNP13145	*Kabuli*	Ca_Kabuli_Chr07	40243784	(G/A)
13146	CakSNP13146	*Kabuli*	Ca_Kabuli_Chr07	40323213	(C/A)
13147	CakSNP13147	*Kabuli*	Ca_Kabuli_Chr07	40323251	(T/A)

S.N.	SNP IDs	Cultivars	Chromosomes/scaffolds	Physical positions (bp)	SNPs
13148	CakSNP13148	*Kabuli*	Ca_Kabuli_Chr07	40429548	(A/G)
13149	CakSNP13149	*Kabuli*	Ca_Kabuli_Chr07	40429552	(C/T)
13150	CakSNP13150	*Kabuli*	Ca_Kabuli_Chr07	40429564	(G/T)
13151	CakSNP13151	*Kabuli*	Ca_Kabuli_Chr07	40429573	(G/T)
13152	CakSNP13152	*Kabuli*	Ca_Kabuli_Chr07	40429629	(G/T)
13153	CakSNP13153	*Kabuli*	Ca_Kabuli_Chr07	40429621	(G/T)
13154	CakSNP13154	*Kabuli*	Ca_Kabuli_Chr07	40429604	(C/T)
13155	CakSNP13155	*Kabuli*	Ca_Kabuli_Chr07	40429600	(C/T)
13156	CakSNP13156	*Kabuli*	Ca_Kabuli_Chr07	40429596	(G/A)
13157	CakSNP13157	*Kabuli*	Ca_Kabuli_Chr07	40429567	(G/A)
13158	CakSNP13158	*Kabuli*	Ca_Kabuli_Chr07	40456574	(T/C)
13159	CakSNP13159	*Kabuli*	Ca_Kabuli_Chr07	40645278	(A/G)
13160	CakSNP13160	*Kabuli*	Ca_Kabuli_Chr07	40645276	(A/G)
13161	CakSNP13161	*Kabuli*	Ca_Kabuli_Chr07	40645208	(C/G)
13162	CakSNP13162	*Kabuli*	Ca_Kabuli_Chr07	40889596	(A/C)
13163	CakSNP13163	*Kabuli*	Ca_Kabuli_Chr07	40889598	(C/G)
13164	CakSNP13164	*Kabuli*	Ca_Kabuli_Chr07	40889623	(G/A)
13165	CakSNP13165	*Kabuli*	Ca_Kabuli_Chr07	40889632	(C/T)
13166	CakSNP13166	*Kabuli*	Ca_Kabuli_Chr07	40889633	(G/A)
13167	CakSNP13167	*Kabuli*	Ca_Kabuli_Chr07	40889640	(G/A)
13168	CakSNP13168	*Kabuli*	Ca_Kabuli_Chr07	40889641	(C/T)
13169	CakSNP13169	*Kabuli*	Ca_Kabuli_Chr07	40889643	(G/A)
13170	CakSNP13170	*Kabuli*	Ca_Kabuli_Chr07	40889648	(T/A)
13171	CakSNP13171	*Kabuli*	Ca_Kabuli_Chr07	40889649	(G/A)
13172	CakSNP13172	*Kabuli*	Ca_Kabuli_Chr07	40889673	(C/A)
13173	CakSNP13173	*Kabuli*	Ca_Kabuli_Chr07	40889670	(C/T)
13174	CakSNP13174	*Kabuli*	Ca_Kabuli_Chr07	40889723	(C/G)
13175	CakSNP13175	*Kabuli*	Ca_Kabuli_Chr07	40889684	(C/T)
13176	CakSNP13176	*Kabuli*	Ca_Kabuli_Chr07	41282883	(A/T)
13177	CakSNP13177	*Kabuli*	Ca_Kabuli_Chr07	41282961	(G/T)
13178	CakSNP13178	*Kabuli*	Ca_Kabuli_Chr07	41282960	(A/C)
13179	CakSNP13179	*Kabuli*	Ca_Kabuli_Chr07	41282957	(G/A)
13180	CakSNP13180	*Kabuli*	Ca_Kabuli_Chr07	41325886	(A/G)
13181	CakSNP13181	*Kabuli*	Ca_Kabuli_Chr07	41339748	(C/A)
13182	CakSNP13182	*Kabuli*	Ca_Kabuli_Chr07	41349604	(T/C)
13183	CakSNP13183	*Kabuli*	Ca_Kabuli_Chr07	41387509	(A/C)
13184	CakSNP13184	*Kabuli*	Ca_Kabuli_Chr07	41387502	(A/C)
13185	CakSNP13185	*Kabuli*	Ca_Kabuli_Chr07	41387475	(A/C)

S.N.	SNP IDs	Cultivars	Chromosomes/scaffolds	Physical positions (bp)	SNPs
13186	CakSNP13186	*Kabuli*	Ca_Kabuli_Chr07	41657180	(G/T)
13187	CakSNP13187	*Kabuli*	Ca_Kabuli_Chr07	41657241	(T/C)
13188	CakSNP13188	*Kabuli*	Ca_Kabuli_Chr07	41821410	(T/C)
13189	CakSNP13189	*Kabuli*	Ca_Kabuli_Chr07	42166004	(C/T)
13190	CakSNP13190	*Kabuli*	Ca_Kabuli_Chr07	42268628	(A/C)
13191	CakSNP13191	*Kabuli*	Ca_Kabuli_Chr07	42272939	(G/C)
13192	CakSNP13192	*Kabuli*	Ca_Kabuli_Chr07	42355002	(T/G)
13193	CakSNP13193	*Kabuli*	Ca_Kabuli_Chr07	42354958	(G/A)
13194	CakSNP13194	*Kabuli*	Ca_Kabuli_Chr07	42354938	(C/T)
13195	CakSNP13195	*Kabuli*	Ca_Kabuli_Chr07	42354999	(C/T)
13196	CakSNP13196	*Kabuli*	Ca_Kabuli_Chr07	42355011	(G/T)
13197	CakSNP13197	*Kabuli*	Ca_Kabuli_Chr07	42566717	(A/G)
13198	CakSNP13198	*Kabuli*	Ca_Kabuli_Chr07	42846882	(C/T)
13199	CakSNP13199	*Kabuli*	Ca_Kabuli_Chr07	42851182	(T/C)
13200	CakSNP13200	*Kabuli*	Ca_Kabuli_Chr07	43080817	(T/C)
13201	CakSNP13201	*Kabuli*	Ca_Kabuli_Chr07	43118954	(A/C)
13202	CakSNP13202	*Kabuli*	Ca_Kabuli_Chr07	43119266	(C/T)
13203	CakSNP13203	*Kabuli*	Ca_Kabuli_Chr07	43210821	(A/T)
13204	CakSNP13204	*Kabuli*	Ca_Kabuli_Chr07	43646117	(G/A)
13205	CakSNP13205	*Kabuli*	Ca_Kabuli_Chr07	44232239	(G/A)
13206	CakSNP13206	*Kabuli*	Ca_Kabuli_Chr07	44232204	(T/A)
13207	CakSNP13207	*Kabuli*	Ca_Kabuli_Chr07	44232221	(C/T)
13208	CakSNP13208	*Kabuli*	Ca_Kabuli_Chr07	44232266	(C/T)
13209	CakSNP13209	*Kabuli*	Ca_Kabuli_Chr07	44540553	(T/G)
13210	CakSNP13210	*Kabuli*	Ca_Kabuli_Chr07	44540595	(G/A)
13211	CakSNP13211	*Kabuli*	Ca_Kabuli_Chr07	44540596	(C/A)
13212	CakSNP13212	*Kabuli*	Ca_Kabuli_Chr07	44540629	(G/A)
13213	CakSNP13213	*Kabuli*	Ca_Kabuli_Chr07	44540616	(C/A)
13214	CakSNP13214	*Kabuli*	Ca_Kabuli_Chr07	44612651	(C/A)
13215	CakSNP13215	*Kabuli*	Ca_Kabuli_Chr07	44747668	(C/T)
13216	CakSNP13216	*Kabuli*	Ca_Kabuli_Chr07	44765450	(C/T)
13217	CakSNP13217	*Kabuli*	Ca_Kabuli_Chr07	44765467	(G/T)
13218	CakSNP13218	*Kabuli*	Ca_Kabuli_Chr07	44765522	(A/C)
13219	CakSNP13219	*Kabuli*	Ca_Kabuli_Chr07	44877324	(A/C)
13220	CakSNP13220	*Kabuli*	Ca_Kabuli_Chr07	44926768	(C/T)
13221	CakSNP13221	*Kabuli*	Ca_Kabuli_Chr07	44926783	(C/A)
13222	CakSNP13222	*Kabuli*	Ca_Kabuli_Chr07	44926815	(C/T)
13223	CakSNP13223	*Kabuli*	Ca_Kabuli_Chr07	45147550	(A/G)

S.N.	SNP IDs	Cultivars	Chromosomes/scaffolds	Physical positions (bp)	SNPs
13224	CakSNP13224	*Kabuli*	Ca_Kabuli_Chr07	45280659	(T/A)
13225	CakSNP13225	*Kabuli*	Ca_Kabuli_Chr07	45289633	(T/C)
13226	CakSNP13226	*Kabuli*	Ca_Kabuli_Chr07	45936581	(C/A)
13227	CakSNP13227	*Kabuli*	Ca_Kabuli_Chr07	46053109	(G/A)
13228	CakSNP13228	*Kabuli*	Ca_Kabuli_Chr07	46394656	(A/G)
13229	CakSNP13229	*Kabuli*	Ca_Kabuli_Chr07	46485934	(A/G)
13230	CakSNP13230	*Kabuli*	Ca_Kabuli_Chr07	46747255	(A/C)
13231	CakSNP13231	*Kabuli*	Ca_Kabuli_Chr07	46747283	(T/C)
13232	CakSNP13232	*Kabuli*	Ca_Kabuli_Chr07	46747290	(G/T)
13233	CakSNP13233	*Kabuli*	Ca_Kabuli_Chr07	46747295	(G/C)
13234	CakSNP13234	*Kabuli*	Ca_Kabuli_Chr07	46747309	(C/T)
13235	CakSNP13235	*Kabuli*	Ca_Kabuli_Chr07	46747330	(G/A)
13236	CakSNP13236	*Kabuli*	Ca_Kabuli_Chr07	46823471	(T/G)
13237	CakSNP13237	*Kabuli*	Ca_Kabuli_Chr07	46823949	(G/T)
13238	CakSNP13238	*Kabuli*	Ca_Kabuli_Chr07	46826036	(T/A)
13239	CakSNP13239	*Kabuli*	Ca_Kabuli_Chr07	46826030	(T/C)
13240	CakSNP13240	*Kabuli*	Ca_Kabuli_Chr07	46826028	(C/A)
13241	CakSNP13241	*Kabuli*	Ca_Kabuli_Chr07	46826020	(G/A)
13242	CakSNP13242	*Kabuli*	Ca_Kabuli_Chr07	46826015	(G/A)
13243	CakSNP13243	*Kabuli*	Ca_Kabuli_Chr07	46826008	(A/T)
13244	CakSNP13244	*Kabuli*	Ca_Kabuli_Chr07	46825997	(A/G)
13245	CakSNP13245	*Kabuli*	Ca_Kabuli_Chr07	46825992	(T/G)
13246	CakSNP13246	*Kabuli*	Ca_Kabuli_Chr07	46825985	(T/G)
13247	CakSNP13247	*Kabuli*	Ca_Kabuli_Chr07	46825957	(A/T)
13248	CakSNP13248	*Kabuli*	Ca_Kabuli_Chr07	46825952	(A/G)
13249	CakSNP13249	*Kabuli*	Ca_Kabuli_Chr07	47127331	(T/G)
13250	CakSNP13250	*Kabuli*	Ca_Kabuli_Chr07	47127342	(T/G)
13251	CakSNP13251	*Kabuli*	Ca_Kabuli_Chr07	47127343	(C/T)
13252	CakSNP13252	*Kabuli*	Ca_Kabuli_Chr07	47159646	(A/C)
13253	CakSNP13253	*Kabuli*	Ca_Kabuli_Chr07	47335940	(G/A)
13254	CakSNP13254	*Kabuli*	Ca_Kabuli_Chr07	47335916	(C/G)
13255	CakSNP13255	*Kabuli*	Ca_Kabuli_Chr07	47524173	(A/C)
13256	CakSNP13256	*Kabuli*	Ca_Kabuli_Chr07	47554330	(C/G)
13257	CakSNP13257	*Kabuli*	Ca_Kabuli_Chr07	47694706	(A/C)
13258	CakSNP13258	*Kabuli*	Ca_Kabuli_Chr07	47839934	(G/A)
13259	CakSNP13259	*Kabuli*	Ca_Kabuli_Chr07	47839952	(G/T)
13260	CakSNP13260	*Kabuli*	Ca_Kabuli_Chr07	47840034	(A/G)
13261	CakSNP13261	*Kabuli*	Ca_Kabuli_Chr07	47840092	(A/C)

S.N.	SNP IDs	Cultivars	Chromosomes/scaffolds	Physical positions (bp)	SNPs
13262	CakSNP13262	*Kabuli*	Ca_Kabuli_Chr07	48102586	(G/T)
13263	CakSNP13263	*Kabuli*	Ca_Kabuli_Chr07	48200865	(A/G)
13264	CakSNP13264	*Kabuli*	Ca_Kabuli_Chr07	48200852	(G/A)
13265	CakSNP13265	*Kabuli*	Ca_Kabuli_Chr07	48200822	(G/A)
13266	CakSNP13266	*Kabuli*	Ca_Kabuli_Chr07	48200820	(A/C)
13267	CakSNP13267	*Kabuli*	Ca_Kabuli_Chr07	48200823	(G/A)
13268	CakSNP13268	*Kabuli*	Ca_Kabuli_Chr07	48200848	(T/G)
13269	CakSNP13269	*Kabuli*	Ca_Kabuli_Chr07	48509506	(G/T)
13270	CakSNP13270	*Kabuli*	Ca_Kabuli_Chr08	54095	(A/C)
13271	CakSNP13271	*Kabuli*	Ca_Kabuli_Chr08	85693	(T/G)
13272	CakSNP13272	*Kabuli*	Ca_Kabuli_Chr08	85697	(G/A)
13273	CakSNP13273	*Kabuli*	Ca_Kabuli_Chr08	85707	(C/A)
13274	CakSNP13274	*Kabuli*	Ca_Kabuli_Chr08	85710	(C/A)
13275	CakSNP13275	*Kabuli*	Ca_Kabuli_Chr08	158986	(C/G)
13276	CakSNP13276	*Kabuli*	Ca_Kabuli_Chr08	177457	(C/T)
13277	CakSNP13277	*Kabuli*	Ca_Kabuli_Chr08	177436	(A/G)
13278	CakSNP13278	*Kabuli*	Ca_Kabuli_Chr08	177405	(G/A)
13279	CakSNP13279	*Kabuli*	Ca_Kabuli_Chr08	184444	(G/A)
13280	CakSNP13280	*Kabuli*	Ca_Kabuli_Chr08	218813	(C/T)
13281	CakSNP13281	*Kabuli*	Ca_Kabuli_Chr08	218828	(T/G)
13282	CakSNP13282	*Kabuli*	Ca_Kabuli_Chr08	218957	(G/T)
13283	CakSNP13283	*Kabuli*	Ca_Kabuli_Chr08	218928	(A/G)
13284	CakSNP13284	*Kabuli*	Ca_Kabuli_Chr08	249654	(A/C)
13285	CakSNP13285	*Kabuli*	Ca_Kabuli_Chr08	253786	(G/A)
13286	CakSNP13286	*Kabuli*	Ca_Kabuli_Chr08	281622	(G/A)
13287	CakSNP13287	*Kabuli*	Ca_Kabuli_Chr08	281608	(T/A)
13288	CakSNP13288	*Kabuli*	Ca_Kabuli_Chr08	281630	(A/C)
13289	CakSNP13289	*Kabuli*	Ca_Kabuli_Chr08	283147	(C/T)
13290	CakSNP13290	*Kabuli*	Ca_Kabuli_Chr08	285779	(G/A)
13291	CakSNP13291	*Kabuli*	Ca_Kabuli_Chr08	356295	(A/T)
13292	CakSNP13292	*Kabuli*	Ca_Kabuli_Chr08	411967	(G/A)
13293	CakSNP13293	*Kabuli*	Ca_Kabuli_Chr08	499819	(A/C)
13294	CakSNP13294	*Kabuli*	Ca_Kabuli_Chr08	499890	(T/C)
13295	CakSNP13295	*Kabuli*	Ca_Kabuli_Chr08	510824	(G/A)
13296	CakSNP13296	*Kabuli*	Ca_Kabuli_Chr08	560477	(C/T)
13297	CakSNP13297	*Kabuli*	Ca_Kabuli_Chr08	560451	(C/T)
13298	CakSNP13298	*Kabuli*	Ca_Kabuli_Chr08	560604	(T/G)
13299	CakSNP13299	*Kabuli*	Ca_Kabuli_Chr08	560746	(A/G)

**S.N.**	**SNP IDs**	**Cultivars**	**Chromosomes/scaffolds**	**Physical positions (bp)**	**SNPs**
13300	CakSNP13300	*Kabuli*	Ca_Kabuli_Chr08	566112	(A/G)
13301	CakSNP13301	*Kabuli*	Ca_Kabuli_Chr08	566107	(C/T)
13302	CakSNP13302	*Kabuli*	Ca_Kabuli_Chr08	566094	(A/G)
13303	CakSNP13303	*Kabuli*	Ca_Kabuli_Chr08	596171	(C/T)
13304	CakSNP13304	*Kabuli*	Ca_Kabuli_Chr08	614324	(G/A)
13305	CakSNP13305	*Kabuli*	Ca_Kabuli_Chr08	614301	(C/A)
13306	CakSNP13306	*Kabuli*	Ca_Kabuli_Chr08	619605	(T/C)
13307	CakSNP13307	*Kabuli*	Ca_Kabuli_Chr08	634862	(C/T)
13308	CakSNP13308	*Kabuli*	Ca_Kabuli_Chr08	634843	(G/A)
13309	CakSNP13309	*Kabuli*	Ca_Kabuli_Chr08	634818	(G/T)
13310	CakSNP13310	*Kabuli*	Ca_Kabuli_Chr08	634921	(C/G)
13311	CakSNP13311	*Kabuli*	Ca_Kabuli_Chr08	649491	(A/C)
13312	CakSNP13312	*Kabuli*	Ca_Kabuli_Chr08	660610	(T/G)
13313	CakSNP13313	*Kabuli*	Ca_Kabuli_Chr08	666294	(G/A)
13314	CakSNP13314	*Kabuli*	Ca_Kabuli_Chr08	666288	(A/T)
13315	CakSNP13315	*Kabuli*	Ca_Kabuli_Chr08	666284	(T/A)
13316	CakSNP13316	*Kabuli*	Ca_Kabuli_Chr08	666274	(T/G)
13317	CakSNP13317	*Kabuli*	Ca_Kabuli_Chr08	672962	(C/T)
13318	CakSNP13318	*Kabuli*	Ca_Kabuli_Chr08	673820	(A/G)
13319	CakSNP13319	*Kabuli*	Ca_Kabuli_Chr08	674902	(T/G)
13320	CakSNP13320	*Kabuli*	Ca_Kabuli_Chr08	701803	(C/T)
13321	CakSNP13321	*Kabuli*	Ca_Kabuli_Chr08	701811	(T/G)
13322	CakSNP13322	*Kabuli*	Ca_Kabuli_Chr08	701837	(G/A)
13323	CakSNP13323	*Kabuli*	Ca_Kabuli_Chr08	706514	(T/C)
13324	CakSNP13324	*Kabuli*	Ca_Kabuli_Chr08	706482	(G/C)
13325	CakSNP13325	*Kabuli*	Ca_Kabuli_Chr08	706614	(C/T)
13326	CakSNP13326	*Kabuli*	Ca_Kabuli_Chr08	706793	(A/G)
13327	CakSNP13327	*Kabuli*	Ca_Kabuli_Chr08	706800	(G/A)
13328	CakSNP13328	*Kabuli*	Ca_Kabuli_Chr08	709063	(T/C)
13329	CakSNP13329	*Kabuli*	Ca_Kabuli_Chr08	709094	(T/C)
13330	CakSNP13330	*Kabuli*	Ca_Kabuli_Chr08	709124	(A/T)
13331	CakSNP13331	*Kabuli*	Ca_Kabuli_Chr08	714967	(A/G)
13332	CakSNP13332	*Kabuli*	Ca_Kabuli_Chr08	714974	(G/A)
13333	CakSNP13333	*Kabuli*	Ca_Kabuli_Chr08	777760	(A/T)
13334	CakSNP13334	*Kabuli*	Ca_Kabuli_Chr08	777772	(G/A)
13335	CakSNP13335	*Kabuli*	Ca_Kabuli_Chr08	777776	(C/G)
13336	CakSNP13336	*Kabuli*	Ca_Kabuli_Chr08	779589	(C/G)
13337	CakSNP13337	*Kabuli*	Ca_Kabuli_Chr08	779611	(T/C)

S.N.	SNP IDs	Cultivars	Chromosomes/scaffolds	Physical positions (bp)	SNPs
13338	CakSNP13338	*Kabuli*	Ca_Kabuli_Chr08	779640	(T/C)
13339	CakSNP13339	*Kabuli*	Ca_Kabuli_Chr08	817321	(T/G)
13340	CakSNP13340	*Kabuli*	Ca_Kabuli_Chr08	822302	(A/G)
13341	CakSNP13341	*Kabuli*	Ca_Kabuli_Chr08	822942	(T/G)
13342	CakSNP13342	*Kabuli*	Ca_Kabuli_Chr08	822976	(C/T)
13343	CakSNP13343	*Kabuli*	Ca_Kabuli_Chr08	839924	(C/G)
13344	CakSNP13344	*Kabuli*	Ca_Kabuli_Chr08	855332	(G/A)
13345	CakSNP13345	*Kabuli*	Ca_Kabuli_Chr08	866741	(A/C)
13346	CakSNP13346	*Kabuli*	Ca_Kabuli_Chr08	871666	(C/G)
13347	CakSNP13347	*Kabuli*	Ca_Kabuli_Chr08	875236	(T/G)
13348	CakSNP13348	*Kabuli*	Ca_Kabuli_Chr08	894184	(C/A)
13349	CakSNP13349	*Kabuli*	Ca_Kabuli_Chr08	895883	(G/A)
13350	CakSNP13350	*Kabuli*	Ca_Kabuli_Chr08	895947	(C/T)
13351	CakSNP13351	*Kabuli*	Ca_Kabuli_Chr08	897417	(T/C)
13352	CakSNP13352	*Kabuli*	Ca_Kabuli_Chr08	898089	(C/T)
13353	CakSNP13353	*Kabuli*	Ca_Kabuli_Chr08	898150	(C/A)
13354	CakSNP13354	*Kabuli*	Ca_Kabuli_Chr08	898152	(A/G)
13355	CakSNP13355	*Kabuli*	Ca_Kabuli_Chr08	898173	(G/T)
13356	CakSNP13356	*Kabuli*	Ca_Kabuli_Chr08	898164	(G/T)
13357	CakSNP13357	*Kabuli*	Ca_Kabuli_Chr08	898226	(C/G)
13358	CakSNP13358	*Kabuli*	Ca_Kabuli_Chr08	898251	(G/T)
13359	CakSNP13359	*Kabuli*	Ca_Kabuli_Chr08	919690	(T/C)
13360	CakSNP13360	*Kabuli*	Ca_Kabuli_Chr08	923820	(T/C)
13361	CakSNP13361	*Kabuli*	Ca_Kabuli_Chr08	924720	(G/T)
13362	CakSNP13362	*Kabuli*	Ca_Kabuli_Chr08	924681	(C/T)
13363	CakSNP13363	*Kabuli*	Ca_Kabuli_Chr08	924715	(A/G)
13364	CakSNP13364	*Kabuli*	Ca_Kabuli_Chr08	924732	(G/T)
13365	CakSNP13365	*Kabuli*	Ca_Kabuli_Chr08	974170	(T/C)
13366	CakSNP13366	*Kabuli*	Ca_Kabuli_Chr08	974159	(T/G)
13367	CakSNP13367	*Kabuli*	Ca_Kabuli_Chr08	1011333	(C/T)
13368	CakSNP13368	*Kabuli*	Ca_Kabuli_Chr08	1011320	(G/T)
13369	CakSNP13369	*Kabuli*	Ca_Kabuli_Chr08	1011327	(G/A)
13370	CakSNP13370	*Kabuli*	Ca_Kabuli_Chr08	1043905	(T/C)
13371	CakSNP13371	*Kabuli*	Ca_Kabuli_Chr08	1070728	(T/C)
13372	CakSNP13372	*Kabuli*	Ca_Kabuli_Chr08	1077334	(C/T)
13373	CakSNP13373	*Kabuli*	Ca_Kabuli_Chr08	1077430	(C/T)
13374	CakSNP13374	*Kabuli*	Ca_Kabuli_Chr08	1119568	(T/C)
13375	CakSNP13375	*Kabuli*	Ca_Kabuli_Chr08	1119781	(A/G)

S.N.	SNP IDs	Cultivars	Chromosomes/scaffolds	Physical positions (bp)	SNPs
13376	CakSNP13376	*Kabuli*	Ca_Kabuli_Chr08	1119773	(T/G)
13377	CakSNP13377	*Kabuli*	Ca_Kabuli_Chr08	1119761	(G/T)
13378	CakSNP13378	*Kabuli*	Ca_Kabuli_Chr08	1138275	(G/A)
13379	CakSNP13379	*Kabuli*	Ca_Kabuli_Chr08	1138394	(A/G)
13380	CakSNP13380	*Kabuli*	Ca_Kabuli_Chr08	1155597	(C/T)
13381	CakSNP13381	*Kabuli*	Ca_Kabuli_Chr08	1155636	(T/C)
13382	CakSNP13382	*Kabuli*	Ca_Kabuli_Chr08	1155639	(T/A)
13383	CakSNP13383	*Kabuli*	Ca_Kabuli_Chr08	1155641	(C/G)
13384	CakSNP13384	*Kabuli*	Ca_Kabuli_Chr08	1174369	(A/G)
13385	CakSNP13385	*Kabuli*	Ca_Kabuli_Chr08	1193297	(C/T)
13386	CakSNP13386	*Kabuli*	Ca_Kabuli_Chr08	1194886	(G/A)
13387	CakSNP13387	*Kabuli*	Ca_Kabuli_Chr08	1194888	(A/G)
13388	CakSNP13388	*Kabuli*	Ca_Kabuli_Chr08	1241739	(C/T)
13389	CakSNP13389	*Kabuli*	Ca_Kabuli_Chr08	1262576	(T/C)
13390	CakSNP13390	*Kabuli*	Ca_Kabuli_Chr08	1262561	(C/T)
13391	CakSNP13391	*Kabuli*	Ca_Kabuli_Chr08	1262660	(C/A)
13392	CakSNP13392	*Kabuli*	Ca_Kabuli_Chr08	1262686	(T/G)
13393	CakSNP13393	*Kabuli*	Ca_Kabuli_Chr08	1265537	(G/A)
13394	CakSNP13394	*Kabuli*	Ca_Kabuli_Chr08	1265535	(A/G)
13395	CakSNP13395	*Kabuli*	Ca_Kabuli_Chr08	1265509	(T/C)
13396	CakSNP13396	*Kabuli*	Ca_Kabuli_Chr08	1265595	(G/T)
13397	CakSNP13397	*Kabuli*	Ca_Kabuli_Chr08	1265579	(A/G)
13398	CakSNP13398	*Kabuli*	Ca_Kabuli_Chr08	1265577	(T/G)
13399	CakSNP13399	*Kabuli*	Ca_Kabuli_Chr08	1382266	(G/A)
13400	CakSNP13400	*Kabuli*	Ca_Kabuli_Chr08	1382433	(G/T)
13401	CakSNP13401	*Kabuli*	Ca_Kabuli_Chr08	1382454	(A/C)
13402	CakSNP13402	*Kabuli*	Ca_Kabuli_Chr08	1433436	(T/C)
13403	CakSNP13403	*Kabuli*	Ca_Kabuli_Chr08	1477523	(T/C)
13404	CakSNP13404	*Kabuli*	Ca_Kabuli_Chr08	1487202	(G/C)
13405	CakSNP13405	*Kabuli*	Ca_Kabuli_Chr08	1503621	(C/T)
13406	CakSNP13406	*Kabuli*	Ca_Kabuli_Chr08	1503595	(C/T)
13407	CakSNP13407	*Kabuli*	Ca_Kabuli_Chr08	1503588	(T/C)
13408	CakSNP13408	*Kabuli*	Ca_Kabuli_Chr08	1504859	(A/G)
13409	CakSNP13409	*Kabuli*	Ca_Kabuli_Chr08	1507709	(T/G)
13410	CakSNP13410	*Kabuli*	Ca_Kabuli_Chr08	1507782	(G/C)
13411	CakSNP13411	*Kabuli*	Ca_Kabuli_Chr08	1518285	(A/C)
13412	CakSNP13412	*Kabuli*	Ca_Kabuli_Chr08	1518297	(T/G)
13413	CakSNP13413	*Kabuli*	Ca_Kabuli_Chr08	1518355	(G/A)

S.N.	SNP IDs	Cultivars	Chromosomes/scaffolds	Physical positions (bp)	SNPs
13414	CakSNP13414	*Kabuli*	Ca_Kabuli_Chr08	1534461	(A/C)
13415	CakSNP13415	*Kabuli*	Ca_Kabuli_Chr08	1544916	(C/T)
13416	CakSNP13416	*Kabuli*	Ca_Kabuli_Chr08	1552082	(T/A)
13417	CakSNP13417	*Kabuli*	Ca_Kabuli_Chr08	1552143	(A/G)
13418	CakSNP13418	*Kabuli*	Ca_Kabuli_Chr08	1554964	(T/C)
13419	CakSNP13419	*Kabuli*	Ca_Kabuli_Chr08	1555017	(C/A)
13420	CakSNP13420	*Kabuli*	Ca_Kabuli_Chr08	1555122	(A/G)
13421	CakSNP13421	*Kabuli*	Ca_Kabuli_Chr08	1557492	(C/T)
13422	CakSNP13422	*Kabuli*	Ca_Kabuli_Chr08	1573877	(G/T)
13423	CakSNP13423	*Kabuli*	Ca_Kabuli_Chr08	1612107	(G/A)
13424	CakSNP13424	*Kabuli*	Ca_Kabuli_Chr08	1612099	(A/G)
13425	CakSNP13425	*Kabuli*	Ca_Kabuli_Chr08	1612221	(C/G)
13426	CakSNP13426	*Kabuli*	Ca_Kabuli_Chr08	1667832	(T/C)
13427	CakSNP13427	*Kabuli*	Ca_Kabuli_Chr08	1675895	(C/T)
13428	CakSNP13428	*Kabuli*	Ca_Kabuli_Chr08	1675888	(C/T)
13429	CakSNP13429	*Kabuli*	Ca_Kabuli_Chr08	1708743	(C/T)
13430	CakSNP13430	*Kabuli*	Ca_Kabuli_Chr08	1708860	(T/C)
13431	CakSNP13431	*Kabuli*	Ca_Kabuli_Chr08	1718032	(C/T)
13432	CakSNP13432	*Kabuli*	Ca_Kabuli_Chr08	1723593	(G/A)
13433	CakSNP13433	*Kabuli*	Ca_Kabuli_Chr08	1743097	(G/A)
13434	CakSNP13434	*Kabuli*	Ca_Kabuli_Chr08	1747523	(T/A)
13435	CakSNP13435	*Kabuli*	Ca_Kabuli_Chr08	1747522	(T/C)
13436	CakSNP13436	*Kabuli*	Ca_Kabuli_Chr08	1782034	(C/G)
13437	CakSNP13437	*Kabuli*	Ca_Kabuli_Chr08	1794755	(C/A)
13438	CakSNP13438	*Kabuli*	Ca_Kabuli_Chr08	1794756	(G/T)
13439	CakSNP13439	*Kabuli*	Ca_Kabuli_Chr08	1794759	(C/T)
13440	CakSNP13440	*Kabuli*	Ca_Kabuli_Chr08	1794810	(G/A)
13441	CakSNP13441	*Kabuli*	Ca_Kabuli_Chr08	1817388	(T/C)
13442	CakSNP13442	*Kabuli*	Ca_Kabuli_Chr08	1830230	(A/T)
13443	CakSNP13443	*Kabuli*	Ca_Kabuli_Chr08	1830348	(T/C)
13444	CakSNP13444	*Kabuli*	Ca_Kabuli_Chr08	1846478	(A/G)
13445	CakSNP13445	*Kabuli*	Ca_Kabuli_Chr08	1893613	(T/C)
13446	CakSNP13446	*Kabuli*	Ca_Kabuli_Chr08	1932975	(T/C)
13447	CakSNP13447	*Kabuli*	Ca_Kabuli_Chr08	1932981	(T/C)
13448	CakSNP13448	*Kabuli*	Ca_Kabuli_Chr08	1932990	(A/C)
13449	CakSNP13449	*Kabuli*	Ca_Kabuli_Chr08	1932993	(C/T)
13450	CakSNP13450	*Kabuli*	Ca_Kabuli_Chr08	1932997	(G/T)
13451	CakSNP13451	*Kabuli*	Ca_Kabuli_Chr08	1933026	(G/A)

S.N.	SNP IDs	Cultivars	Chromosomes/scaffolds	Physical positions (bp)	SNPs
13452	CakSNP13452	*Kabuli*	Ca_Kabuli_Chr08	1933064	(G/C)
13453	CakSNP13453	*Kabuli*	Ca_Kabuli_Chr08	1933053	(G/A)
13454	CakSNP13454	*Kabuli*	Ca_Kabuli_Chr08	1933052	(T/C)
13455	CakSNP13455	*Kabuli*	Ca_Kabuli_Chr08	1933039	(G/T)
13456	CakSNP13456	*Kabuli*	Ca_Kabuli_Chr08	1933035	(T/A)
13457	CakSNP13457	*Kabuli*	Ca_Kabuli_Chr08	1958433	(G/C)
13458	CakSNP13458	*Kabuli*	Ca_Kabuli_Chr08	1958415	(T/G)
13459	CakSNP13459	*Kabuli*	Ca_Kabuli_Chr08	1978964	(G/C)
13460	CakSNP13460	*Kabuli*	Ca_Kabuli_Chr08	1980189	(T/A)
13461	CakSNP13461	*Kabuli*	Ca_Kabuli_Chr08	2004509	(A/G)
13462	CakSNP13462	*Kabuli*	Ca_Kabuli_Chr08	2004577	(G/C)
13463	CakSNP13463	*Kabuli*	Ca_Kabuli_Chr08	2029405	(C/A)
13464	CakSNP13464	*Kabuli*	Ca_Kabuli_Chr08	2029384	(T/C)
13465	CakSNP13465	*Kabuli*	Ca_Kabuli_Chr08	2052263	(T/G)
13466	CakSNP13466	*Kabuli*	Ca_Kabuli_Chr08	2052286	(T/C)
13467	CakSNP13467	*Kabuli*	Ca_Kabuli_Chr08	2070694	(G/T)
13468	CakSNP13468	*Kabuli*	Ca_Kabuli_Chr08	2073807	(C/G)
13469	CakSNP13469	*Kabuli*	Ca_Kabuli_Chr08	2074619	(T/G)
13470	CakSNP13470	*Kabuli*	Ca_Kabuli_Chr08	2075296	(T/C)
13471	CakSNP13471	*Kabuli*	Ca_Kabuli_Chr08	2075290	(G/T)
13472	CakSNP13472	*Kabuli*	Ca_Kabuli_Chr08	2075278	(T/G)
13473	CakSNP13473	*Kabuli*	Ca_Kabuli_Chr08	2079723	(A/G)
13474	CakSNP13474	*Kabuli*	Ca_Kabuli_Chr08	2081228	(C/T)
13475	CakSNP13475	*Kabuli*	Ca_Kabuli_Chr08	2088903	(T/C)
13476	CakSNP13476	*Kabuli*	Ca_Kabuli_Chr08	2088892	(T/G)
13477	CakSNP13477	*Kabuli*	Ca_Kabuli_Chr08	2090852	(A/G)
13478	CakSNP13478	*Kabuli*	Ca_Kabuli_Chr08	2095607	(A/G)
13479	CakSNP13479	*Kabuli*	Ca_Kabuli_Chr08	2107370	(C/T)
13480	CakSNP13480	*Kabuli*	Ca_Kabuli_Chr08	2113823	(T/G)
13481	CakSNP13481	*Kabuli*	Ca_Kabuli_Chr08	2113816	(T/C)
13482	CakSNP13482	*Kabuli*	Ca_Kabuli_Chr08	2128450	(A/G)
13483	CakSNP13483	*Kabuli*	Ca_Kabuli_Chr08	2194927	(G/A)
13484	CakSNP13484	*Kabuli*	Ca_Kabuli_Chr08	2194898	(A/T)
13485	CakSNP13485	*Kabuli*	Ca_Kabuli_Chr08	2250195	(C/G)
13486	CakSNP13486	*Kabuli*	Ca_Kabuli_Chr08	2250148	(T/C)
13487	CakSNP13487	*Kabuli*	Ca_Kabuli_Chr08	2264108	(G/A)
13488	CakSNP13488	*Kabuli*	Ca_Kabuli_Chr08	2279413	(G/A)
13489	CakSNP13489	*Kabuli*	Ca_Kabuli_Chr08	2309552	(C/G)

S.N.	SNP IDs	Cultivars	Chromosomes/scaffolds	Physical positions (bp)	SNPs
13490	CakSNP13490	*Kabuli*	Ca_Kabuli_Chr08	2309549	(A/G)
13491	CakSNP13491	*Kabuli*	Ca_Kabuli_Chr08	2413731	(A/G)
13492	CakSNP13492	*Kabuli*	Ca_Kabuli_Chr08	2456352	(T/C)
13493	CakSNP13493	*Kabuli*	Ca_Kabuli_Chr08	2482834	(A/G)
13494	CakSNP13494	*Kabuli*	Ca_Kabuli_Chr08	2482849	(T/C)
13495	CakSNP13495	*Kabuli*	Ca_Kabuli_Chr08	2483021	(G/A)
13496	CakSNP13496	*Kabuli*	Ca_Kabuli_Chr08	2488709	(A/G)
13497	CakSNP13497	*Kabuli*	Ca_Kabuli_Chr08	2488769	(C/G)
13498	CakSNP13498	*Kabuli*	Ca_Kabuli_Chr08	2488772	(A/G)
13499	CakSNP13499	*Kabuli*	Ca_Kabuli_Chr08	2515612	(A/G)
13500	CakSNP13500	*Kabuli*	Ca_Kabuli_Chr08	2524188	(A/G)
13501	CakSNP13501	*Kabuli*	Ca_Kabuli_Chr08	2524148	(A/G)
13502	CakSNP13502	*Kabuli*	Ca_Kabuli_Chr08	2539375	(G/A)
13503	CakSNP13503	*Kabuli*	Ca_Kabuli_Chr08	2540644	(G/A)
13504	CakSNP13504	*Kabuli*	Ca_Kabuli_Chr08	2540704	(A/T)
13505	CakSNP13505	*Kabuli*	Ca_Kabuli_Chr08	2543156	(A/G)
13506	CakSNP13506	*Kabuli*	Ca_Kabuli_Chr08	2545070	(T/A)
13507	CakSNP13507	*Kabuli*	Ca_Kabuli_Chr08	2604759	(C/T)
13508	CakSNP13508	*Kabuli*	Ca_Kabuli_Chr08	2605423	(A/T)
13509	CakSNP13509	*Kabuli*	Ca_Kabuli_Chr08	2605460	(C/T)
13510	CakSNP13510	*Kabuli*	Ca_Kabuli_Chr08	2621132	(T/A)
13511	CakSNP13511	*Kabuli*	Ca_Kabuli_Chr08	2622009	(G/T)
13512	CakSNP13512	*Kabuli*	Ca_Kabuli_Chr08	2621987	(C/G)
13513	CakSNP13513	*Kabuli*	Ca_Kabuli_Chr08	2621973	(T/G)
13514	CakSNP13514	*Kabuli*	Ca_Kabuli_Chr08	2651439	(G/T)
13515	CakSNP13515	*Kabuli*	Ca_Kabuli_Chr08	2651425	(C/T)
13516	CakSNP13516	*Kabuli*	Ca_Kabuli_Chr08	2651422	(T/C)
13517	CakSNP13517	*Kabuli*	Ca_Kabuli_Chr08	2651393	(G/T)
13518	CakSNP13518	*Kabuli*	Ca_Kabuli_Chr08	2651373	(G/T)
13519	CakSNP13519	*Kabuli*	Ca_Kabuli_Chr08	2651921	(C/T)
13520	CakSNP13520	*Kabuli*	Ca_Kabuli_Chr08	2664625	(T/C)
13521	CakSNP13521	*Kabuli*	Ca_Kabuli_Chr08	2664711	(T/G)
13522	CakSNP13522	*Kabuli*	Ca_Kabuli_Chr08	2722673	(C/A)
13523	CakSNP13523	*Kabuli*	Ca_Kabuli_Chr08	2735849	(T/C)
13524	CakSNP13524	*Kabuli*	Ca_Kabuli_Chr08	2743075	(T/C)
13525	CakSNP13525	*Kabuli*	Ca_Kabuli_Chr08	2743088	(A/G)
13526	CakSNP13526	*Kabuli*	Ca_Kabuli_Chr08	2743109	(G/C)
13527	CakSNP13527	*Kabuli*	Ca_Kabuli_Chr08	2743139	(A/T)

S.N.	SNP IDs	Cultivars	Chromosomes/scaffolds	Physical positions (bp)	SNPs
13528	CakSNP13528	*Kabuli*	Ca_Kabuli_Chr08	2783576	(T/G)
13529	CakSNP13529	*Kabuli*	Ca_Kabuli_Chr08	2783655	(G/C)
13530	CakSNP13530	*Kabuli*	Ca_Kabuli_Chr08	2827822	(C/T)
13531	CakSNP13531	*Kabuli*	Ca_Kabuli_Chr08	2827984	(T/C)
13532	CakSNP13532	*Kabuli*	Ca_Kabuli_Chr08	2838179	(T/A)
13533	CakSNP13533	*Kabuli*	Ca_Kabuli_Chr08	2871095	(G/A)
13534	CakSNP13534	*Kabuli*	Ca_Kabuli_Chr08	2871064	(G/A)
13535	CakSNP13535	*Kabuli*	Ca_Kabuli_Chr08	2888353	(G/T)
13536	CakSNP13536	*Kabuli*	Ca_Kabuli_Chr08	2888317	(G/A)
13537	CakSNP13537	*Kabuli*	Ca_Kabuli_Chr08	2943631	(G/T)
13538	CakSNP13538	*Kabuli*	Ca_Kabuli_Chr08	2947498	(A/G)
13539	CakSNP13539	*Kabuli*	Ca_Kabuli_Chr08	2974759	(G/C)
13540	CakSNP13540	*Kabuli*	Ca_Kabuli_Chr08	2976709	(C/T)
13541	CakSNP13541	*Kabuli*	Ca_Kabuli_Chr08	2977543	(T/C)
13542	CakSNP13542	*Kabuli*	Ca_Kabuli_Chr08	2977628	(C/T)
13543	CakSNP13543	*Kabuli*	Ca_Kabuli_Chr08	2977603	(T/A)
13544	CakSNP13544	*Kabuli*	Ca_Kabuli_Chr08	3013928	(G/C)
13545	CakSNP13545	*Kabuli*	Ca_Kabuli_Chr08	3013917	(G/A)
13546	CakSNP13546	*Kabuli*	Ca_Kabuli_Chr08	3041947	(A/G)
13547	CakSNP13547	*Kabuli*	Ca_Kabuli_Chr08	3041981	(A/G)
13548	CakSNP13548	*Kabuli*	Ca_Kabuli_Chr08	3050452	(T/C)
13549	CakSNP13549	*Kabuli*	Ca_Kabuli_Chr08	3064477	(G/A)
13550	CakSNP13550	*Kabuli*	Ca_Kabuli_Chr08	3141617	(C/T)
13551	CakSNP13551	*Kabuli*	Ca_Kabuli_Chr08	3171324	(C/T)
13552	CakSNP13552	*Kabuli*	Ca_Kabuli_Chr08	3210879	(C/A)
13553	CakSNP13553	*Kabuli*	Ca_Kabuli_Chr08	3248044	(A/G)
13554	CakSNP13554	*Kabuli*	Ca_Kabuli_Chr08	3248104	(A/G)
13555	CakSNP13555	*Kabuli*	Ca_Kabuli_Chr08	3250707	(C/T)
13556	CakSNP13556	*Kabuli*	Ca_Kabuli_Chr08	3263822	(T/C)
13557	CakSNP13557	*Kabuli*	Ca_Kabuli_Chr08	3269124	(G/A)
13558	CakSNP13558	*Kabuli*	Ca_Kabuli_Chr08	3269118	(A/G)
13559	CakSNP13559	*Kabuli*	Ca_Kabuli_Chr08	3269088	(A/C)
13560	CakSNP13560	*Kabuli*	Ca_Kabuli_Chr08	3269085	(A/C)
13561	CakSNP13561	*Kabuli*	Ca_Kabuli_Chr08	3297410	(T/C)
13562	CakSNP13562	*Kabuli*	Ca_Kabuli_Chr08	3386165	(C/A)
13563	CakSNP13563	*Kabuli*	Ca_Kabuli_Chr08	3386181	(C/A)
13564	CakSNP13564	*Kabuli*	Ca_Kabuli_Chr08	3392929	(A/C)
13565	CakSNP13565	*Kabuli*	Ca_Kabuli_Chr08	3394796	(A/C)

S.N.	SNP IDs	Cultivars	Chromosomes/scaffolds	Physical positions (bp)	SNPs
13566	CakSNP13566	*Kabuli*	Ca_Kabuli_Chr08	3394833	(T/C)
13567	CakSNP13567	*Kabuli*	Ca_Kabuli_Chr08	3394931	(T/C)
13568	CakSNP13568	*Kabuli*	Ca_Kabuli_Chr08	3396820	(T/C)
13569	CakSNP13569	*Kabuli*	Ca_Kabuli_Chr08	3438274	(A/T)
13570	CakSNP13570	*Kabuli*	Ca_Kabuli_Chr08	3445568	(G/T)
13571	CakSNP13571	*Kabuli*	Ca_Kabuli_Chr08	3448194	(C/T)
13572	CakSNP13572	*Kabuli*	Ca_Kabuli_Chr08	3460627	(G/A)
13573	CakSNP13573	*Kabuli*	Ca_Kabuli_Chr08	3499010	(G/A)
13574	CakSNP13574	*Kabuli*	Ca_Kabuli_Chr08	3539690	(A/G)
13575	CakSNP13575	*Kabuli*	Ca_Kabuli_Chr08	3539723	(G/C)
13576	CakSNP13576	*Kabuli*	Ca_Kabuli_Chr08	3539745	(T/C)
13577	CakSNP13577	*Kabuli*	Ca_Kabuli_Chr08	3539851	(T/C)
13578	CakSNP13578	*Kabuli*	Ca_Kabuli_Chr08	3539838	(T/G)
13579	CakSNP13579	*Kabuli*	Ca_Kabuli_Chr08	3539832	(T/C)
13580	CakSNP13580	*Kabuli*	Ca_Kabuli_Chr08	3560271	(G/A)
13581	CakSNP13581	*Kabuli*	Ca_Kabuli_Chr08	3563692	(A/C)
13582	CakSNP13582	*Kabuli*	Ca_Kabuli_Chr08	3563825	(G/T)
13583	CakSNP13583	*Kabuli*	Ca_Kabuli_Chr08	3563824	(T/A)
13584	CakSNP13584	*Kabuli*	Ca_Kabuli_Chr08	3599091	(G/C)
13585	CakSNP13585	*Kabuli*	Ca_Kabuli_Chr08	3601517	(T/C)
13586	CakSNP13586	*Kabuli*	Ca_Kabuli_Chr08	3605513	(T/C)
13587	CakSNP13587	*Kabuli*	Ca_Kabuli_Chr08	3617810	(T/C)
13588	CakSNP13588	*Kabuli*	Ca_Kabuli_Chr08	3617757	(G/C)
13589	CakSNP13589	*Kabuli*	Ca_Kabuli_Chr08	3723085	(C/T)
13590	CakSNP13590	*Kabuli*	Ca_Kabuli_Chr08	3730369	(A/C)
13591	CakSNP13591	*Kabuli*	Ca_Kabuli_Chr08	3775945	(G/A)
13592	CakSNP13592	*Kabuli*	Ca_Kabuli_Chr08	3775960	(T/C)
13593	CakSNP13593	*Kabuli*	Ca_Kabuli_Chr08	3813978	(C/T)
13594	CakSNP13594	*Kabuli*	Ca_Kabuli_Chr08	3829425	(C/G)
13595	CakSNP13595	*Kabuli*	Ca_Kabuli_Chr08	3829576	(A/G)
13596	CakSNP13596	*Kabuli*	Ca_Kabuli_Chr08	3837237	(A/G)
13597	CakSNP13597	*Kabuli*	Ca_Kabuli_Chr08	3837306	(G/C)
13598	CakSNP13598	*Kabuli*	Ca_Kabuli_Chr08	3868511	(A/C)
13599	CakSNP13599	*Kabuli*	Ca_Kabuli_Chr08	3877130	(G/T)
13600	CakSNP13600	*Kabuli*	Ca_Kabuli_Chr08	3877176	(T/G)
13601	CakSNP13601	*Kabuli*	Ca_Kabuli_Chr08	3911930	(A/C)
13602	CakSNP13602	*Kabuli*	Ca_Kabuli_Chr08	3911876	(G/C)
13603	CakSNP13603	*Kabuli*	Ca_Kabuli_Chr08	3951767	(C/A)

**S.N.**	**SNP IDs**	**Cultivars**	**Chromosomes/scaffolds**	**Physical positions (bp)**	**SNPs**
13604	CakSNP13604	*Kabuli*	Ca_Kabuli_Chr08	3951768	(A/G)
13605	CakSNP13605	*Kabuli*	Ca_Kabuli_Chr08	3954853	(T/A)
13606	CakSNP13606	*Kabuli*	Ca_Kabuli_Chr08	4042166	(A/C)
13607	CakSNP13607	*Kabuli*	Ca_Kabuli_Chr08	4042233	(G/A)
13608	CakSNP13608	*Kabuli*	Ca_Kabuli_Chr08	4042424	(G/A)
13609	CakSNP13609	*Kabuli*	Ca_Kabuli_Chr08	4052732	(C/T)
13610	CakSNP13610	*Kabuli*	Ca_Kabuli_Chr08	4052702	(C/T)
13611	CakSNP13611	*Kabuli*	Ca_Kabuli_Chr08	4073528	(C/T)
13612	CakSNP13612	*Kabuli*	Ca_Kabuli_Chr08	4073603	(A/G)
13613	CakSNP13613	*Kabuli*	Ca_Kabuli_Chr08	4074135	(T/C)
13614	CakSNP13614	*Kabuli*	Ca_Kabuli_Chr08	4091946	(T/C)
13615	CakSNP13615	*Kabuli*	Ca_Kabuli_Chr08	4092348	(T/A)
13616	CakSNP13616	*Kabuli*	Ca_Kabuli_Chr08	4092350	(G/C)
13617	CakSNP13617	*Kabuli*	Ca_Kabuli_Chr08	4095131	(A/C)
13618	CakSNP13618	*Kabuli*	Ca_Kabuli_Chr08	4095119	(G/A)
13619	CakSNP13619	*Kabuli*	Ca_Kabuli_Chr08	4106580	(T/C)
13620	CakSNP13620	*Kabuli*	Ca_Kabuli_Chr08	4106583	(T/C)
13621	CakSNP13621	*Kabuli*	Ca_Kabuli_Chr08	4106644	(C/T)
13622	CakSNP13622	*Kabuli*	Ca_Kabuli_Chr08	4172342	(A/G)
13623	CakSNP13623	*Kabuli*	Ca_Kabuli_Chr08	4172368	(T/C)
13624	CakSNP13624	*Kabuli*	Ca_Kabuli_Chr08	4183744	(A/G)
13625	CakSNP13625	*Kabuli*	Ca_Kabuli_Chr08	4183733	(G/A)
13626	CakSNP13626	*Kabuli*	Ca_Kabuli_Chr08	4198214	(A/C)
13627	CakSNP13627	*Kabuli*	Ca_Kabuli_Chr08	4201285	(A/G)
13628	CakSNP13628	*Kabuli*	Ca_Kabuli_Chr08	4237620	(T/A)
13629	CakSNP13629	*Kabuli*	Ca_Kabuli_Chr08	4271620	(T/A)
13630	CakSNP13630	*Kabuli*	Ca_Kabuli_Chr08	4290690	(T/A)
13631	CakSNP13631	*Kabuli*	Ca_Kabuli_Chr08	4322058	(A/G)
13632	CakSNP13632	*Kabuli*	Ca_Kabuli_Chr08	4322202	(C/T)
13633	CakSNP13633	*Kabuli*	Ca_Kabuli_Chr08	4344459	(C/A)
13634	CakSNP13634	*Kabuli*	Ca_Kabuli_Chr08	4346552	(A/G)
13635	CakSNP13635	*Kabuli*	Ca_Kabuli_Chr08	4346749	(A/G)
13636	CakSNP13636	*Kabuli*	Ca_Kabuli_Chr08	4346811	(C/T)
13637	CakSNP13637	*Kabuli*	Ca_Kabuli_Chr08	4346827	(A/C)
13638	CakSNP13638	*Kabuli*	Ca_Kabuli_Chr08	4347535	(A/C)
13639	CakSNP13639	*Kabuli*	Ca_Kabuli_Chr08	4351751	(G/A)
13640	CakSNP13640	*Kabuli*	Ca_Kabuli_Chr08	4353397	(A/C)
13641	CakSNP13641	*Kabuli*	Ca_Kabuli_Chr08	4355388	(G/T)

S.N.	SNP IDs	Cultivars	Chromosomes/scaffolds	Physical positions (bp)	SNPs
13642	CakSNP13642	*Kabuli*	Ca_Kabuli_Chr08	4355531	(A/C)
13643	CakSNP13643	*Kabuli*	Ca_Kabuli_Chr08	4368163	(T/C)
13644	CakSNP13644	*Kabuli*	Ca_Kabuli_Chr08	4368184	(T/C)
13645	CakSNP13645	*Kabuli*	Ca_Kabuli_Chr08	4368213	(T/C)
13646	CakSNP13646	*Kabuli*	Ca_Kabuli_Chr08	4368403	(G/A)
13647	CakSNP13647	*Kabuli*	Ca_Kabuli_Chr08	4368463	(A/G)
13648	CakSNP13648	*Kabuli*	Ca_Kabuli_Chr08	4392707	(T/C)
13649	CakSNP13649	*Kabuli*	Ca_Kabuli_Chr08	4392767	(T/G)
13650	CakSNP13650	*Kabuli*	Ca_Kabuli_Chr08	4392818	(C/A)
13651	CakSNP13651	*Kabuli*	Ca_Kabuli_Chr08	4406956	(C/G)
13652	CakSNP13652	*Kabuli*	Ca_Kabuli_Chr08	4421609	(A/G)
13653	CakSNP13653	*Kabuli*	Ca_Kabuli_Chr08	4421629	(T/A)
13654	CakSNP13654	*Kabuli*	Ca_Kabuli_Chr08	4472423	(A/T)
13655	CakSNP13655	*Kabuli*	Ca_Kabuli_Chr08	4483130	(T/C)
13656	CakSNP13656	*Kabuli*	Ca_Kabuli_Chr08	4483274	(T/C)
13657	CakSNP13657	*Kabuli*	Ca_Kabuli_Chr08	4484606	(T/A)
13658	CakSNP13658	*Kabuli*	Ca_Kabuli_Chr08	4507729	(G/T)
13659	CakSNP13659	*Kabuli*	Ca_Kabuli_Chr08	4510845	(C/T)
13660	CakSNP13660	*Kabuli*	Ca_Kabuli_Chr08	4510810	(A/G)
13661	CakSNP13661	*Kabuli*	Ca_Kabuli_Chr08	4512311	(T/C)
13662	CakSNP13662	*Kabuli*	Ca_Kabuli_Chr08	4512436	(G/A)
13663	CakSNP13663	*Kabuli*	Ca_Kabuli_Chr08	4512492	(T/G)
13664	CakSNP13664	*Kabuli*	Ca_Kabuli_Chr08	4524914	(C/T)
13665	CakSNP13665	*Kabuli*	Ca_Kabuli_Chr08	4557334	(A/G)
13666	CakSNP13666	*Kabuli*	Ca_Kabuli_Chr08	4557328	(T/C)
13667	CakSNP13667	*Kabuli*	Ca_Kabuli_Chr08	4557401	(A/C)
13668	CakSNP13668	*Kabuli*	Ca_Kabuli_Chr08	4557407	(A/C)
13669	CakSNP13669	*Kabuli*	Ca_Kabuli_Chr08	4557440	(A/G)
13670	CakSNP13670	*Kabuli*	Ca_Kabuli_Chr08	4557488	(C/T)
13671	CakSNP13671	*Kabuli*	Ca_Kabuli_Chr08	4580363	(C/A)
13672	CakSNP13672	*Kabuli*	Ca_Kabuli_Chr08	4588869	(T/C)
13673	CakSNP13673	*Kabuli*	Ca_Kabuli_Chr08	4588915	(A/C)
13674	CakSNP13674	*Kabuli*	Ca_Kabuli_Chr08	4611094	(C/T)
13675	CakSNP13675	*Kabuli*	Ca_Kabuli_Chr08	4658502	(A/G)
13676	CakSNP13676	*Kabuli*	Ca_Kabuli_Chr08	4714566	(G/A)
13677	CakSNP13677	*Kabuli*	Ca_Kabuli_Chr08	4744388	(C/T)
13678	CakSNP13678	*Kabuli*	Ca_Kabuli_Chr08	4767567	(A/C)
13679	CakSNP13679	*Kabuli*	Ca_Kabuli_Chr08	4767586	(C/G)

S.N.	SNP IDs	Cultivars	Chromosomes/scaffolds	Physical positions (bp)	SNPs
13680	CakSNP13680	*Kabuli*	Ca_Kabuli_Chr08	4773205	(A/C)
13681	CakSNP13681	*Kabuli*	Ca_Kabuli_Chr08	4810954	(A/C)
13682	CakSNP13682	*Kabuli*	Ca_Kabuli_Chr08	4810968	(G/A)
13683	CakSNP13683	*Kabuli*	Ca_Kabuli_Chr08	4810986	(A/C)
13684	CakSNP13684	*Kabuli*	Ca_Kabuli_Chr08	4858262	(C/A)
13685	CakSNP13685	*Kabuli*	Ca_Kabuli_Chr08	4899964	(T/G)
13686	CakSNP13686	*Kabuli*	Ca_Kabuli_Chr08	4899983	(G/A)
13687	CakSNP13687	*Kabuli*	Ca_Kabuli_Chr08	4980949	(A/T)
13688	CakSNP13688	*Kabuli*	Ca_Kabuli_Chr08	4980916	(C/T)
13689	CakSNP13689	*Kabuli*	Ca_Kabuli_Chr08	5044456	(T/C)
13690	CakSNP13690	*Kabuli*	Ca_Kabuli_Chr08	5044423	(T/A)
13691	CakSNP13691	*Kabuli*	Ca_Kabuli_Chr08	5096194	(T/G)
13692	CakSNP13692	*Kabuli*	Ca_Kabuli_Chr08	5115451	(C/G)
13693	CakSNP13693	*Kabuli*	Ca_Kabuli_Chr08	5115498	(A/T)
13694	CakSNP13694	*Kabuli*	Ca_Kabuli_Chr08	5115512	(T/G)
13695	CakSNP13695	*Kabuli*	Ca_Kabuli_Chr08	5115522	(G/C)
13696	CakSNP13696	*Kabuli*	Ca_Kabuli_Chr08	5134706	(A/C)
13697	CakSNP13697	*Kabuli*	Ca_Kabuli_Chr08	5184227	(C/G)
13698	CakSNP13698	*Kabuli*	Ca_Kabuli_Chr08	5184276	(G/A)
13699	CakSNP13699	*Kabuli*	Ca_Kabuli_Chr08	5197004	(G/T)
13700	CakSNP13700	*Kabuli*	Ca_Kabuli_Chr08	5197254	(G/A)
13701	CakSNP13701	*Kabuli*	Ca_Kabuli_Chr08	5204956	(A/G)
13702	CakSNP13702	*Kabuli*	Ca_Kabuli_Chr08	5313792	(T/C)
13703	CakSNP13703	*Kabuli*	Ca_Kabuli_Chr08	5313782	(T/G)
13704	CakSNP13704	*Kabuli*	Ca_Kabuli_Chr08	5313774	(C/G)
13705	CakSNP13705	*Kabuli*	Ca_Kabuli_Chr08	5313770	(A/T)
13706	CakSNP13706	*Kabuli*	Ca_Kabuli_Chr08	5379556	(G/A)
13707	CakSNP13707	*Kabuli*	Ca_Kabuli_Chr08	5390415	(C/T)
13708	CakSNP13708	*Kabuli*	Ca_Kabuli_Chr08	5390602	(T/C)
13709	CakSNP13709	*Kabuli*	Ca_Kabuli_Chr08	5399503	(G/A)
13710	CakSNP13710	*Kabuli*	Ca_Kabuli_Chr08	5400188	(A/G)
13711	CakSNP13711	*Kabuli*	Ca_Kabuli_Chr08	5412501	(A/G)
13712	CakSNP13712	*Kabuli*	Ca_Kabuli_Chr08	5412489	(T/C)
13713	CakSNP13713	*Kabuli*	Ca_Kabuli_Chr08	5434223	(C/T)
13714	CakSNP13714	*Kabuli*	Ca_Kabuli_Chr08	5434225	(G/C)
13715	CakSNP13715	*Kabuli*	Ca_Kabuli_Chr08	5434232	(A/G)
13716	CakSNP13716	*Kabuli*	Ca_Kabuli_Chr08	5434238	(T/G)
13717	CakSNP13717	*Kabuli*	Ca_Kabuli_Chr08	5434242	(T/A)

S.N.	SNP IDs	Cultivars	Chromosomes/scaffolds	Physical positions (bp)	SNPs
13718	CakSNP13718	*Kabuli*	Ca_Kabuli_Chr08	5487769	(G/A)
13719	CakSNP13719	*Kabuli*	Ca_Kabuli_Chr08	5487830	(G/A)
13720	CakSNP13720	*Kabuli*	Ca_Kabuli_Chr08	5536418	(T/G)
13721	CakSNP13721	*Kabuli*	Ca_Kabuli_Chr08	5538696	(A/C)
13722	CakSNP13722	*Kabuli*	Ca_Kabuli_Chr08	5538695	(T/C)
13723	CakSNP13723	*Kabuli*	Ca_Kabuli_Chr08	5538683	(T/C)
13724	CakSNP13724	*Kabuli*	Ca_Kabuli_Chr08	5539044	(C/T)
13725	CakSNP13725	*Kabuli*	Ca_Kabuli_Chr08	5586106	(G/A)
13726	CakSNP13726	*Kabuli*	Ca_Kabuli_Chr08	5591654	(T/C)
13727	CakSNP13727	*Kabuli*	Ca_Kabuli_Chr08	5591788	(G/C)
13728	CakSNP13728	*Kabuli*	Ca_Kabuli_Chr08	5650080	(C/G)
13729	CakSNP13729	*Kabuli*	Ca_Kabuli_Chr08	5712245	(T/A)
13730	CakSNP13730	*Kabuli*	Ca_Kabuli_Chr08	5811179	(G/C)
13731	CakSNP13731	*Kabuli*	Ca_Kabuli_Chr08	5819008	(G/A)
13732	CakSNP13732	*Kabuli*	Ca_Kabuli_Chr08	5840141	(G/A)
13733	CakSNP13733	*Kabuli*	Ca_Kabuli_Chr08	5870455	(A/G)
13734	CakSNP13734	*Kabuli*	Ca_Kabuli_Chr08	5870664	(T/C)
13735	CakSNP13735	*Kabuli*	Ca_Kabuli_Chr08	5870749	(C/T)
13736	CakSNP13736	*Kabuli*	Ca_Kabuli_Chr08	5871358	(G/C)
13737	CakSNP13737	*Kabuli*	Ca_Kabuli_Chr08	5871361	(T/G)
13738	CakSNP13738	*Kabuli*	Ca_Kabuli_Chr08	5871364	(T/C)
13739	CakSNP13739	*Kabuli*	Ca_Kabuli_Chr08	5871367	(T/A)
13740	CakSNP13740	*Kabuli*	Ca_Kabuli_Chr08	5871421	(A/G)
13741	CakSNP13741	*Kabuli*	Ca_Kabuli_Chr08	5910629	(C/G)
13742	CakSNP13742	*Kabuli*	Ca_Kabuli_Chr08	5927870	(G/T)
13743	CakSNP13743	*Kabuli*	Ca_Kabuli_Chr08	5935435	(A/C)
13744	CakSNP13744	*Kabuli*	Ca_Kabuli_Chr08	5963855	(T/A)
13745	CakSNP13745	*Kabuli*	Ca_Kabuli_Chr08	5992761	(A/G)
13746	CakSNP13746	*Kabuli*	Ca_Kabuli_Chr08	6084594	(G/A)
13747	CakSNP13747	*Kabuli*	Ca_Kabuli_Chr08	6084591	(C/T)
13748	CakSNP13748	*Kabuli*	Ca_Kabuli_Chr08	6115961	(T/G)
13749	CakSNP13749	*Kabuli*	Ca_Kabuli_Chr08	6115965	(T/C)
13750	CakSNP13750	*Kabuli*	Ca_Kabuli_Chr08	6116096	(C/T)
13751	CakSNP13751	*Kabuli*	Ca_Kabuli_Chr08	6116097	(C/A)
13752	CakSNP13752	*Kabuli*	Ca_Kabuli_Chr08	6116101	(G/A)
13753	CakSNP13753	*Kabuli*	Ca_Kabuli_Chr08	6145326	(G/A)
13754	CakSNP13754	*Kabuli*	Ca_Kabuli_Chr08	6149097	(A/C)
13755	CakSNP13755	*Kabuli*	Ca_Kabuli_Chr08	6192944	(T/G)

S.N.	SNP IDs	Cultivars	Chromosomes/scaffolds	Physical positions (bp)	SNPs
13756	CakSNP13756	*Kabuli*	Ca_Kabuli_Chr08	6192986	(C/T)
13757	CakSNP13757	*Kabuli*	Ca_Kabuli_Chr08	6193078	(G/A)
13758	CakSNP13758	*Kabuli*	Ca_Kabuli_Chr08	6218250	(G/A)
13759	CakSNP13759	*Kabuli*	Ca_Kabuli_Chr08	6218275	(C/T)
13760	CakSNP13760	*Kabuli*	Ca_Kabuli_Chr08	6218298	(A/G)
13761	CakSNP13761	*Kabuli*	Ca_Kabuli_Chr08	6218299	(T/A)
13762	CakSNP13762	*Kabuli*	Ca_Kabuli_Chr08	6266432	(A/G)
13763	CakSNP13763	*Kabuli*	Ca_Kabuli_Chr08	6372236	(A/T)
13764	CakSNP13764	*Kabuli*	Ca_Kabuli_Chr08	6372230	(G/A)
13765	CakSNP13765	*Kabuli*	Ca_Kabuli_Chr08	6372223	(G/A)
13766	CakSNP13766	*Kabuli*	Ca_Kabuli_Chr08	6374794	(C/A)
13767	CakSNP13767	*Kabuli*	Ca_Kabuli_Chr08	6437758	(T/G)
13768	CakSNP13768	*Kabuli*	Ca_Kabuli_Chr08	6503723	(T/C)
13769	CakSNP13769	*Kabuli*	Ca_Kabuli_Chr08	6525633	(A/T)
13770	CakSNP13770	*Kabuli*	Ca_Kabuli_Chr08	6526612	(T/C)
13771	CakSNP13771	*Kabuli*	Ca_Kabuli_Chr08	6544248	(G/T)
13772	CakSNP13772	*Kabuli*	Ca_Kabuli_Chr08	6544278	(C/A)
13773	CakSNP13773	*Kabuli*	Ca_Kabuli_Chr08	6624006	(C/T)
13774	CakSNP13774	*Kabuli*	Ca_Kabuli_Chr08	6630663	(C/T)
13775	CakSNP13775	*Kabuli*	Ca_Kabuli_Chr08	6630661	(T/C)
13776	CakSNP13776	*Kabuli*	Ca_Kabuli_Chr08	6664509	(T/A)
13777	CakSNP13777	*Kabuli*	Ca_Kabuli_Chr08	6673855	(C/T)
13778	CakSNP13778	*Kabuli*	Ca_Kabuli_Chr08	6673891	(A/G)
13779	CakSNP13779	*Kabuli*	Ca_Kabuli_Chr08	6674024	(G/A)
13780	CakSNP13780	*Kabuli*	Ca_Kabuli_Chr08	6673956	(G/A)
13781	CakSNP13781	*Kabuli*	Ca_Kabuli_Chr08	6708869	(G/T)
13782	CakSNP13782	*Kabuli*	Ca_Kabuli_Chr08	6708945	(A/G)
13783	CakSNP13783	*Kabuli*	Ca_Kabuli_Chr08	6747091	(T/C)
13784	CakSNP13784	*Kabuli*	Ca_Kabuli_Chr08	6747995	(A/G)
13785	CakSNP13785	*Kabuli*	Ca_Kabuli_Chr08	6748136	(C/T)
13786	CakSNP13786	*Kabuli*	Ca_Kabuli_Chr08	6748294	(T/C)
13787	CakSNP13787	*Kabuli*	Ca_Kabuli_Chr08	6780592	(T/G)
13788	CakSNP13788	*Kabuli*	Ca_Kabuli_Chr08	6940139	(A/G)
13789	CakSNP13789	*Kabuli*	Ca_Kabuli_Chr08	6942651	(G/T)
13790	CakSNP13790	*Kabuli*	Ca_Kabuli_Chr08	6943710	(A/G)
13791	CakSNP13791	*Kabuli*	Ca_Kabuli_Chr08	6968036	(A/G)
13792	CakSNP13792	*Kabuli*	Ca_Kabuli_Chr08	6986090	(G/A)
13793	CakSNP13793	*Kabuli*	Ca_Kabuli_Chr08	6986130	(A/G)

S.N.	SNP IDs	Cultivars	Chromosomes/scaffolds	Physical positions (bp)	SNPs
13794	CakSNP13794	*Kabuli*	Ca_Kabuli_Chr08	6987609	(G/T)
13795	CakSNP13795	*Kabuli*	Ca_Kabuli_Chr08	6987615	(A/C)
13796	CakSNP13796	*Kabuli*	Ca_Kabuli_Chr08	6989342	(G/T)
13797	CakSNP13797	*Kabuli*	Ca_Kabuli_Chr08	6989433	(A/G)
13798	CakSNP13798	*Kabuli*	Ca_Kabuli_Chr08	7013049	(G/A)
13799	CakSNP13799	*Kabuli*	Ca_Kabuli_Chr08	7037715	(G/A)
13800	CakSNP13800	*Kabuli*	Ca_Kabuli_Chr08	7041901	(A/C)
13801	CakSNP13801	*Kabuli*	Ca_Kabuli_Chr08	7047509	(T/G)
13802	CakSNP13802	*Kabuli*	Ca_Kabuli_Chr08	7047469	(T/A)
13803	CakSNP13803	*Kabuli*	Ca_Kabuli_Chr08	7099455	(C/T)
13804	CakSNP13804	*Kabuli*	Ca_Kabuli_Chr08	7099569	(T/C)
13805	CakSNP13805	*Kabuli*	Ca_Kabuli_Chr08	7099530	(C/A)
13806	CakSNP13806	*Kabuli*	Ca_Kabuli_Chr08	7099756	(A/G)
13807	CakSNP13807	*Kabuli*	Ca_Kabuli_Chr08	7162826	(T/G)
13808	CakSNP13808	*Kabuli*	Ca_Kabuli_Chr08	7162976	(A/G)
13809	CakSNP13809	*Kabuli*	Ca_Kabuli_Chr08	7174066	(C/T)
13810	CakSNP13810	*Kabuli*	Ca_Kabuli_Chr08	7175086	(A/C)
13811	CakSNP13811	*Kabuli*	Ca_Kabuli_Chr08	7191825	(A/G)
13812	CakSNP13812	*Kabuli*	Ca_Kabuli_Chr08	7193946	(C/T)
13813	CakSNP13813	*Kabuli*	Ca_Kabuli_Chr08	7193960	(A/T)
13814	CakSNP13814	*Kabuli*	Ca_Kabuli_Chr08	7295151	(T/C)
13815	CakSNP13815	*Kabuli*	Ca_Kabuli_Chr08	7295206	(A/G)
13816	CakSNP13816	*Kabuli*	Ca_Kabuli_Chr08	7295177	(G/T)
13817	CakSNP13817	*Kabuli*	Ca_Kabuli_Chr08	7307737	(G/T)
13818	CakSNP13818	*Kabuli*	Ca_Kabuli_Chr08	7307787	(A/T)
13819	CakSNP13819	*Kabuli*	Ca_Kabuli_Chr08	7357621	(A/G)
13820	CakSNP13820	*Kabuli*	Ca_Kabuli_Chr08	7357583	(T/C)
13821	CakSNP13821	*Kabuli*	Ca_Kabuli_Chr08	7503013	(T/A)
13822	CakSNP13822	*Kabuli*	Ca_Kabuli_Chr08	7503010	(T/C)
13823	CakSNP13823	*Kabuli*	Ca_Kabuli_Chr08	7506004	(A/G)
13824	CakSNP13824	*Kabuli*	Ca_Kabuli_Chr08	7519758	(C/A)
13825	CakSNP13825	*Kabuli*	Ca_Kabuli_Chr08	7519815	(G/A)
13826	CakSNP13826	*Kabuli*	Ca_Kabuli_Chr08	7526956	(A/G)
13827	CakSNP13827	*Kabuli*	Ca_Kabuli_Chr08	7563385	(C/G)
13828	CakSNP13828	*Kabuli*	Ca_Kabuli_Chr08	7563370	(T/C)
13829	CakSNP13829	*Kabuli*	Ca_Kabuli_Chr08	7563373	(T/A)
13830	CakSNP13830	*Kabuli*	Ca_Kabuli_Chr08	7566548	(T/C)
13831	CakSNP13831	*Kabuli*	Ca_Kabuli_Chr08	7614202	(A/C)

S.N.	SNP IDs	Cultivars	Chromosomes/scaffolds	Physical positions (bp)	SNPs
13832	CakSNP13832	*Kabuli*	Ca_Kabuli_Chr08	7628690	(T/A)
13833	CakSNP13833	*Kabuli*	Ca_Kabuli_Chr08	7665291	(T/C)
13834	CakSNP13834	*Kabuli*	Ca_Kabuli_Chr08	7665383	(G/T)
13835	CakSNP13835	*Kabuli*	Ca_Kabuli_Chr08	7694747	(A/G)
13836	CakSNP13836	*Kabuli*	Ca_Kabuli_Chr08	7695990	(A/T)
13837	CakSNP13837	*Kabuli*	Ca_Kabuli_Chr08	7695989	(T/A)
13838	CakSNP13838	*Kabuli*	Ca_Kabuli_Chr08	7697886	(G/C)
13839	CakSNP13839	*Kabuli*	Ca_Kabuli_Chr08	7726921	(G/A)
13840	CakSNP13840	*Kabuli*	Ca_Kabuli_Chr08	7726894	(G/T)
13841	CakSNP13841	*Kabuli*	Ca_Kabuli_Chr08	7726879	(A/G)
13842	CakSNP13842	*Kabuli*	Ca_Kabuli_Chr08	7728085	(T/G)
13843	CakSNP13843	*Kabuli*	Ca_Kabuli_Chr08	7728076	(T/C)
13844	CakSNP13844	*Kabuli*	Ca_Kabuli_Chr08	7729693	(T/C)
13845	CakSNP13845	*Kabuli*	Ca_Kabuli_Chr08	7733508	(C/T)
13846	CakSNP13846	*Kabuli*	Ca_Kabuli_Chr08	7733573	(A/C)
13847	CakSNP13847	*Kabuli*	Ca_Kabuli_Chr08	7733574	(G/A)
13848	CakSNP13848	*Kabuli*	Ca_Kabuli_Chr08	7733670	(C/T)
13849	CakSNP13849	*Kabuli*	Ca_Kabuli_Chr08	7733647	(G/T)
13850	CakSNP13850	*Kabuli*	Ca_Kabuli_Chr08	7776466	(G/A)
13851	CakSNP13851	*Kabuli*	Ca_Kabuli_Chr08	7776592	(T/C)
13852	CakSNP13852	*Kabuli*	Ca_Kabuli_Chr08	7776897	(A/T)
13853	CakSNP13853	*Kabuli*	Ca_Kabuli_Chr08	7776934	(G/T)
13854	CakSNP13854	*Kabuli*	Ca_Kabuli_Chr08	7776970	(T/G)
13855	CakSNP13855	*Kabuli*	Ca_Kabuli_Chr08	7777023	(G/A)
13856	CakSNP13856	*Kabuli*	Ca_Kabuli_Chr08	7807065	(T/C)
13857	CakSNP13857	*Kabuli*	Ca_Kabuli_Chr08	7978269	(C/T)
13858	CakSNP13858	*Kabuli*	Ca_Kabuli_Chr08	8029283	(T/A)
13859	CakSNP13859	*Kabuli*	Ca_Kabuli_Chr08	8039078	(A/T)
13860	CakSNP13860	*Kabuli*	Ca_Kabuli_Chr08	8059732	(A/G)
13861	CakSNP13861	*Kabuli*	Ca_Kabuli_Chr08	8066006	(C/A)
13862	CakSNP13862	*Kabuli*	Ca_Kabuli_Chr08	8066005	(A/G)
13863	CakSNP13863	*Kabuli*	Ca_Kabuli_Chr08	8104804	(G/T)
13864	CakSNP13864	*Kabuli*	Ca_Kabuli_Chr08	8104801	(T/C)
13865	CakSNP13865	*Kabuli*	Ca_Kabuli_Chr08	8104884	(T/A)
13866	CakSNP13866	*Kabuli*	Ca_Kabuli_Chr08	8104892	(C/T)
13867	CakSNP13867	*Kabuli*	Ca_Kabuli_Chr08	8106804	(A/G)
13868	CakSNP13868	*Kabuli*	Ca_Kabuli_Chr08	8106851	(G/A)
13869	CakSNP13869	*Kabuli*	Ca_Kabuli_Chr08	8114316	(A/C)

S.N.	SNP IDs	Cultivars	Chromosomes/scaffolds	Physical positions (bp)	SNPs
13870	CakSNP13870	*Kabuli*	Ca_Kabuli_Chr08	8194206	(C/T)
13871	CakSNP13871	*Kabuli*	Ca_Kabuli_Chr08	8194232	(C/A)
13872	CakSNP13872	*Kabuli*	Ca_Kabuli_Chr08	8194234	(C/A)
13873	CakSNP13873	*Kabuli*	Ca_Kabuli_Chr08	8194342	(T/C)
13874	CakSNP13874	*Kabuli*	Ca_Kabuli_Chr08	8194464	(A/G)
13875	CakSNP13875	*Kabuli*	Ca_Kabuli_Chr08	8194436	(T/A)
13876	CakSNP13876	*Kabuli*	Ca_Kabuli_Chr08	8202726	(G/A)
13877	CakSNP13877	*Kabuli*	Ca_Kabuli_Chr08	8270488	(T/C)
13878	CakSNP13878	*Kabuli*	Ca_Kabuli_Chr08	8288041	(A/G)
13879	CakSNP13879	*Kabuli*	Ca_Kabuli_Chr08	8293716	(G/A)
13880	CakSNP13880	*Kabuli*	Ca_Kabuli_Chr08	8293704	(T/C)
13881	CakSNP13881	*Kabuli*	Ca_Kabuli_Chr08	8293693	(T/C)
13882	CakSNP13882	*Kabuli*	Ca_Kabuli_Chr08	8318110	(A/G)
13883	CakSNP13883	*Kabuli*	Ca_Kabuli_Chr08	8389117	(C/T)
13884	CakSNP13884	*Kabuli*	Ca_Kabuli_Chr08	8458383	(G/A)
13885	CakSNP13885	*Kabuli*	Ca_Kabuli_Chr08	8533479	(G/T)
13886	CakSNP13886	*Kabuli*	Ca_Kabuli_Chr08	8533978	(G/A)
13887	CakSNP13887	*Kabuli*	Ca_Kabuli_Chr08	8560330	(C/A)
13888	CakSNP13888	*Kabuli*	Ca_Kabuli_Chr08	8560331	(C/G)
13889	CakSNP13889	*Kabuli*	Ca_Kabuli_Chr08	8560332	(A/C)
13890	CakSNP13890	*Kabuli*	Ca_Kabuli_Chr08	8560326	(G/T)
13891	CakSNP13891	*Kabuli*	Ca_Kabuli_Chr08	8661327	(T/C)
13892	CakSNP13892	*Kabuli*	Ca_Kabuli_Chr08	8670242	(T/G)
13893	CakSNP13893	*Kabuli*	Ca_Kabuli_Chr08	8772131	(C/G)
13894	CakSNP13894	*Kabuli*	Ca_Kabuli_Chr08	8772124	(G/A)
13895	CakSNP13895	*Kabuli*	Ca_Kabuli_Chr08	8853602	(G/A)
13896	CakSNP13896	*Kabuli*	Ca_Kabuli_Chr08	8853664	(A/G)
13897	CakSNP13897	*Kabuli*	Ca_Kabuli_Chr08	8856046	(C/T)
13898	CakSNP13898	*Kabuli*	Ca_Kabuli_Chr08	8856047	(C/T)
13899	CakSNP13899	*Kabuli*	Ca_Kabuli_Chr08	8856150	(A/G)
13900	CakSNP13900	*Kabuli*	Ca_Kabuli_Chr08	8856143	(G/A)
13901	CakSNP13901	*Kabuli*	Ca_Kabuli_Chr08	8864079	(C/T)
13902	CakSNP13902	*Kabuli*	Ca_Kabuli_Chr08	8864157	(G/A)
13903	CakSNP13903	*Kabuli*	Ca_Kabuli_Chr08	8888412	(A/G)
13904	CakSNP13904	*Kabuli*	Ca_Kabuli_Chr08	8888414	(G/T)
13905	CakSNP13905	*Kabuli*	Ca_Kabuli_Chr08	8888426	(T/C)
13906	CakSNP13906	*Kabuli*	Ca_Kabuli_Chr08	8888476	(A/C)
13907	CakSNP13907	*Kabuli*	Ca_Kabuli_Chr08	8904346	(C/A)

S.N.	SNP IDs	Cultivars	Chromosomes/scaffolds	Physical positions (bp)	SNPs
13908	CakSNP13908	*Kabuli*	Ca_Kabuli_Chr08	8904366	(A/G)
13909	CakSNP13909	*Kabuli*	Ca_Kabuli_Chr08	8914018	(A/C)
13910	CakSNP13910	*Kabuli*	Ca_Kabuli_Chr08	9097585	(A/C)
13911	CakSNP13911	*Kabuli*	Ca_Kabuli_Chr08	9099798	(G/T)
13912	CakSNP13912	*Kabuli*	Ca_Kabuli_Chr08	9100111	(C/T)
13913	CakSNP13913	*Kabuli*	Ca_Kabuli_Chr08	9109896	(T/A)
13914	CakSNP13914	*Kabuli*	Ca_Kabuli_Chr08	9109907	(A/T)
13915	CakSNP13915	*Kabuli*	Ca_Kabuli_Chr08	9109920	(C/A)
13916	CakSNP13916	*Kabuli*	Ca_Kabuli_Chr08	9112178	(G/A)
13917	CakSNP13917	*Kabuli*	Ca_Kabuli_Chr08	9112172	(A/C)
13918	CakSNP13918	*Kabuli*	Ca_Kabuli_Chr08	9118582	(C/T)
13919	CakSNP13919	*Kabuli*	Ca_Kabuli_Chr08	9118667	(A/G)
13920	CakSNP13920	*Kabuli*	Ca_Kabuli_Chr08	9143840	(C/A)
13921	CakSNP13921	*Kabuli*	Ca_Kabuli_Chr08	9151722	(G/T)
13922	CakSNP13922	*Kabuli*	Ca_Kabuli_Chr08	9389347	(G/A)
13923	CakSNP13923	*Kabuli*	Ca_Kabuli_Chr08	10004481	(T/C)
13924	CakSNP13924	*Kabuli*	Ca_Kabuli_Chr08	10155981	(A/C)
13925	CakSNP13925	*Kabuli*	Ca_Kabuli_Chr08	10258892	(T/G)
13926	CakSNP13926	*Kabuli*	Ca_Kabuli_Chr08	10486601	(A/C)
13927	CakSNP13927	*Kabuli*	Ca_Kabuli_Chr08	10556124	(T/C)
13928	CakSNP13928	*Kabuli*	Ca_Kabuli_Chr08	10668422	(A/C)
13929	CakSNP13929	*Kabuli*	Ca_Kabuli_Chr08	10728369	(G/C)
13930	CakSNP13930	*Kabuli*	Ca_Kabuli_Chr08	10733404	(A/G)
13931	CakSNP13931	*Kabuli*	Ca_Kabuli_Chr08	10733503	(C/T)
13932	CakSNP13932	*Kabuli*	Ca_Kabuli_Chr08	10733661	(G/A)
13933	CakSNP13933	*Kabuli*	Ca_Kabuli_Chr08	10733747	(C/T)
13934	CakSNP13934	*Kabuli*	Ca_Kabuli_Chr08	10759078	(G/A)
13935	CakSNP13935	*Kabuli*	Ca_Kabuli_Chr08	10759060	(G/A)
13936	CakSNP13936	*Kabuli*	Ca_Kabuli_Chr08	10759056	(C/T)
13937	CakSNP13937	*Kabuli*	Ca_Kabuli_Chr08	10759102	(C/T)
13938	CakSNP13938	*Kabuli*	Ca_Kabuli_Chr08	10763656	(A/T)
13939	CakSNP13939	*Kabuli*	Ca_Kabuli_Chr08	10953767	(C/T)
13940	CakSNP13940	*Kabuli*	Ca_Kabuli_Chr08	11015680	(G/T)
13941	CakSNP13941	*Kabuli*	Ca_Kabuli_Chr08	11015678	(C/G)
13942	CakSNP13942	*Kabuli*	Ca_Kabuli_Chr08	11015666	(G/C)
13943	CakSNP13943	*Kabuli*	Ca_Kabuli_Chr08	11066644	(C/T)
13944	CakSNP13944	*Kabuli*	Ca_Kabuli_Chr08	11066728	(G/A)
13945	CakSNP13945	*Kabuli*	Ca_Kabuli_Chr08	11087143	(A/G)

S.N.	SNP IDs	Cultivars	Chromosomes/scaffolds	Physical positions (bp)	SNPs
13946	CakSNP13946	*Kabuli*	Ca_Kabuli_Chr08	11087169	(C/T)
13947	CakSNP13947	*Kabuli*	Ca_Kabuli_Chr08	11087302	(A/T)
13948	CakSNP13948	*Kabuli*	Ca_Kabuli_Chr08	11087250	(T/C)
13949	CakSNP13949	*Kabuli*	Ca_Kabuli_Chr08	11087247	(T/C)
13950	CakSNP13950	*Kabuli*	Ca_Kabuli_Chr08	11186919	(C/G)
13951	CakSNP13951	*Kabuli*	Ca_Kabuli_Chr08	11187037	(T/C)
13952	CakSNP13952	*Kabuli*	Ca_Kabuli_Chr08	11186986	(A/C)
13953	CakSNP13953	*Kabuli*	Ca_Kabuli_Chr08	11186974	(A/C)
13954	CakSNP13954	*Kabuli*	Ca_Kabuli_Chr08	11231812	(A/G)
13955	CakSNP13955	*Kabuli*	Ca_Kabuli_Chr08	11240258	(C/A)
13956	CakSNP13956	*Kabuli*	Ca_Kabuli_Chr08	11243263	(A/C)
13957	CakSNP13957	*Kabuli*	Ca_Kabuli_Chr08	11243256	(A/C)
13958	CakSNP13958	*Kabuli*	Ca_Kabuli_Chr08	11266447	(G/A)
13959	CakSNP13959	*Kabuli*	Ca_Kabuli_Chr08	11310460	(G/A)
13960	CakSNP13960	*Kabuli*	Ca_Kabuli_Chr08	11310480	(T/A)
13961	CakSNP13961	*Kabuli*	Ca_Kabuli_Chr08	11332934	(C/T)
13962	CakSNP13962	*Kabuli*	Ca_Kabuli_Chr08	11332940	(T/C)
13963	CakSNP13963	*Kabuli*	Ca_Kabuli_Chr08	11333057	(C/G)
13964	CakSNP13964	*Kabuli*	Ca_Kabuli_Chr08	11334190	(A/T)
13965	CakSNP13965	*Kabuli*	Ca_Kabuli_Chr08	11334210	(C/T)
13966	CakSNP13966	*Kabuli*	Ca_Kabuli_Chr08	11349891	(T/A)
13967	CakSNP13967	*Kabuli*	Ca_Kabuli_Chr08	11349995	(A/G)
13968	CakSNP13968	*Kabuli*	Ca_Kabuli_Chr08	11403805	(G/A)
13969	CakSNP13969	*Kabuli*	Ca_Kabuli_Chr08	11403754	(G/A)
13970	CakSNP13970	*Kabuli*	Ca_Kabuli_Chr08	11562814	(A/C)
13971	CakSNP13971	*Kabuli*	Ca_Kabuli_Chr08	11562833	(A/G)
13972	CakSNP13972	*Kabuli*	Ca_Kabuli_Chr08	11631836	(C/T)
13973	CakSNP13973	*Kabuli*	Ca_Kabuli_Chr08	11656700	(G/T)
13974	CakSNP13974	*Kabuli*	Ca_Kabuli_Chr08	11656687	(A/G)
13975	CakSNP13975	*Kabuli*	Ca_Kabuli_Chr08	11656675	(T/C)
13976	CakSNP13976	*Kabuli*	Ca_Kabuli_Chr08	11656670	(A/G)
13977	CakSNP13977	*Kabuli*	Ca_Kabuli_Chr08	11696021	(G/C)
13978	CakSNP13978	*Kabuli*	Ca_Kabuli_Chr08	11735882	(A/C)
13979	CakSNP13979	*Kabuli*	Ca_Kabuli_Chr08	11962280	(T/C)
13980	CakSNP13980	*Kabuli*	Ca_Kabuli_Chr08	12875217	(A/G)
13981	CakSNP13981	*Kabuli*	Ca_Kabuli_Chr08	13248268	(T/C)
13982	CakSNP13982	*Kabuli*	Ca_Kabuli_Chr08	13745335	(A/G)
13983	CakSNP13983	*Kabuli*	Ca_Kabuli_Chr08	13840865	(A/C)

S.N.	SNP IDs	Cultivars	Chromosomes/scaffolds	Physical positions (bp)	SNPs
13984	CakSNP13984	*Kabuli*	Ca_Kabuli_Chr08	13860922	(C/G)
13985	CakSNP13985	*Kabuli*	Ca_Kabuli_Chr08	13864026	(T/G)
13986	CakSNP13986	*Kabuli*	Ca_Kabuli_Chr08	13864077	(T/C)
13987	CakSNP13987	*Kabuli*	Ca_Kabuli_Chr08	13997385	(C/T)
13988	CakSNP13988	*Kabuli*	Ca_Kabuli_Chr08	13998102	(A/G)
13989	CakSNP13989	*Kabuli*	Ca_Kabuli_Chr08	14006709	(G/A)
13990	CakSNP13990	*Kabuli*	Ca_Kabuli_Chr08	14006694	(C/T)
13991	CakSNP13991	*Kabuli*	Ca_Kabuli_Chr08	14007431	(A/G)
13992	CakSNP13992	*Kabuli*	Ca_Kabuli_Chr08	14124588	(C/A)
13993	CakSNP13993	*Kabuli*	Ca_Kabuli_Chr08	14129040	(G/A)
13994	CakSNP13994	*Kabuli*	Ca_Kabuli_Chr08	14325980	(C/A)
13995	CakSNP13995	*Kabuli*	Ca_Kabuli_Chr08	14437006	(C/A)
13996	CakSNP13996	*Kabuli*	Ca_Kabuli_Chr08	14444424	(G/A)
13997	CakSNP13997	*Kabuli*	Ca_Kabuli_Chr08	14444438	(A/C)
13998	CakSNP13998	*Kabuli*	Ca_Kabuli_Chr08	14445205	(C/A)
13999	CakSNP13999	*Kabuli*	Ca_Kabuli_Chr08	14460426	(T/C)
14000	CakSNP14000	*Kabuli*	Ca_Kabuli_Chr08	14528882	(T/G)
14001	CakSNP14001	*Kabuli*	Ca_Kabuli_Chr08	14528966	(C/G)
14002	CakSNP14002	*Kabuli*	Ca_Kabuli_Chr08	14613302	(G/A)
14003	CakSNP14003	*Kabuli*	Ca_Kabuli_Chr08	14640967	(A/C)
14004	CakSNP14004	*Kabuli*	Ca_Kabuli_Chr08	14715495	(T/G)
14005	CakSNP14005	*Kabuli*	Ca_Kabuli_Chr08	14753763	(T/C)
14006	CakSNP14006	*Kabuli*	Ca_Kabuli_Chr08	14753750	(T/C)
14007	CakSNP14007	*Kabuli*	Ca_Kabuli_Chr08	14754849	(A/T)
14008	CakSNP14008	*Kabuli*	Ca_Kabuli_Chr08	14759784	(G/T)
14009	CakSNP14009	*Kabuli*	Ca_Kabuli_Chr08	14760479	(C/G)
14010	CakSNP14010	*Kabuli*	Ca_Kabuli_Chr08	14760497	(G/A)
14011	CakSNP14011	*Kabuli*	Ca_Kabuli_Chr08	14760503	(A/T)
14012	CakSNP14012	*Kabuli*	Ca_Kabuli_Chr08	14821416	(G/A)
14013	CakSNP14013	*Kabuli*	Ca_Kabuli_Chr08	15000109	(A/G)
14014	CakSNP14014	*Kabuli*	Ca_Kabuli_Chr08	15034465	(T/C)
14015	CakSNP14015	*Kabuli*	Ca_Kabuli_Chr08	15131175	(G/A)
14016	CakSNP14016	*Kabuli*	Ca_Kabuli_Chr08	15192125	(A/G)
14017	CakSNP14017	*Kabuli*	Ca_Kabuli_Chr08	15207944	(C/T)
14018	CakSNP14018	*Kabuli*	Ca_Kabuli_Chr08	15207976	(A/G)
14019	CakSNP14019	*Kabuli*	Ca_Kabuli_Chr08	15220986	(C/G)
14020	CakSNP14020	*Kabuli*	Ca_Kabuli_Chr08	15421764	(T/C)
14021	CakSNP14021	*Kabuli*	Ca_Kabuli_Chr08	15421744	(T/G)

S.N.	SNP IDs	Cultivars	Chromosomes/scaffolds	Physical positions (bp)	SNPs
14022	CakSNP14022	*Kabuli*	Ca_Kabuli_Chr08	15421719	(C/G)
14023	CakSNP14023	*Kabuli*	Ca_Kabuli_Chr08	15422163	(A/T)
14024	CakSNP14024	*Kabuli*	Ca_Kabuli_Chr08	15546669	(T/C)
14025	CakSNP14025	*Kabuli*	Ca_Kabuli_Chr08	15546671	(A/C)
14026	CakSNP14026	*Kabuli*	Ca_Kabuli_Chr08	15546696	(C/T)
14027	CakSNP14027	*Kabuli*	Ca_Kabuli_Chr08	15546710	(T/A)
14028	CakSNP14028	*Kabuli*	Ca_Kabuli_Chr08	15546758	(G/C)
14029	CakSNP14029	*Kabuli*	Ca_Kabuli_Chr08	15553251	(A/G)
14030	CakSNP14030	*Kabuli*	Ca_Kabuli_Chr08	15555771	(A/C)
14031	CakSNP14031	*Kabuli*	Ca_Kabuli_Chr08	15699220	(T/A)
14032	CakSNP14032	*Kabuli*	Ca_Kabuli_Chr08	15699180	(T/C)
14033	CakSNP14033	*Kabuli*	Ca_Kabuli_Chr08	15741863	(C/G)
14034	CakSNP14034	*Kabuli*	Ca_Kabuli_Chr08	15764739	(C/T)
14035	CakSNP14035	*Kabuli*	Ca_Kabuli_Chr08	15764748	(G/A)
14036	CakSNP14036	*Kabuli*	Ca_Kabuli_Chr08	15764823	(G/A)
14037	CakSNP14037	*Kabuli*	Ca_Kabuli_Chr08	15764808	(C/A)
14038	CakSNP14038	*Kabuli*	Ca_Kabuli_Chr08	15764874	(G/A)
14039	CakSNP14039	*Kabuli*	Ca_Kabuli_Chr08	15857176	(G/A)
14040	CakSNP14040	*Kabuli*	Ca_Kabuli_Chr08	15857192	(A/G)
14041	CakSNP14041	*Kabuli*	Ca_Kabuli_Chr08	15857359	(T/G)
14042	CakSNP14042	*Kabuli*	Ca_Kabuli_Chr08	15863509	(T/C)
14043	CakSNP14043	*Kabuli*	Ca_Kabuli_Chr08	15873071	(A/C)
14044	CakSNP14044	*Kabuli*	Ca_Kabuli_Chr08	15873133	(A/G)
14045	CakSNP14045	*Kabuli*	Ca_Kabuli_Chr08	16004976	(G/C)
14046	CakSNP14046	*Kabuli*	Ca_Kabuli_Chr08	16005046	(C/T)
14047	CakSNP14047	*Kabuli*	Ca_Kabuli_Chr08	16005078	(A/G)
14048	CakSNP14048	*Kabuli*	Ca_Kabuli_Chr08	16005235	(A/G)
14049	CakSNP14049	*Kabuli*	Ca_Kabuli_Chr08	16005253	(G/A)
14050	CakSNP14050	*Kabuli*	Ca_Kabuli_Chr08	16020239	(C/A)
14051	CakSNP14051	*Kabuli*	Ca_Kabuli_Chr08	16020277	(T/G)
14052	CakSNP14052	*Kabuli*	Ca_Kabuli_Chr08	16020323	(G/T)
14053	CakSNP14053	*Kabuli*	Ca_Kabuli_Chr08	16020305	(G/A)
14054	CakSNP14054	*Kabuli*	Ca_Kabuli_Chr08	16022432	(C/T)
14055	CakSNP14055	*Kabuli*	Ca_Kabuli_Chr08	16027974	(A/T)
14056	CakSNP14056	*Kabuli*	Ca_Kabuli_Chr08	16028064	(G/T)
14057	CakSNP14057	*Kabuli*	Ca_Kabuli_Chr08	16028062	(G/A)
14058	CakSNP14058	*Kabuli*	Ca_Kabuli_Chr08	16028040	(A/G)
14059	CakSNP14059	*Kabuli*	Ca_Kabuli_Chr08	16033779	(T/G)

S.N.	SNP IDs	Cultivars	Chromosomes/scaffolds	Physical positions (bp)	SNPs
14060	CakSNP14060	*Kabuli*	Ca_Kabuli_Chr08	16076161	(C/G)
14061	CakSNP14061	*Kabuli*	Ca_Kabuli_Chr08	16076168	(A/G)
14062	CakSNP14062	*Kabuli*	Ca_Kabuli_Chr08	16102413	(C/T)
14063	CakSNP14063	*Kabuli*	Ca_Kabuli_Chr08	16102395	(G/A)
14064	CakSNP14064	*Kabuli*	Ca_Kabuli_Chr08	16102363	(T/C)
14065	CakSNP14065	*Kabuli*	Ca_Kabuli_Chr08	16102362	(G/T)
14066	CakSNP14066	*Kabuli*	Ca_Kabuli_Chr08	16102348	(A/T)
14067	CakSNP14067	*Kabuli*	Ca_Kabuli_Chr08	16102343	(C/T)
14068	CakSNP14068	*Kabuli*	Ca_Kabuli_Chr08	16102335	(A/T)
14069	CakSNP14069	*Kabuli*	Ca_Kabuli_Chr08	16102414	(G/T)
14070	CakSNP14070	*Kabuli*	Ca_Kabuli_Chr08	16144279	(T/A)
14071	CakSNP14071	*Kabuli*	Ca_Kabuli_Chr08	16145275	(A/G)
14072	CakSNP14072	*Kabuli*	Ca_Kabuli_Chr08	16145419	(A/G)
14073	CakSNP14073	*Kabuli*	Ca_Kabuli_Chr08	16145411	(C/G)
14074	CakSNP14074	*Kabuli*	Ca_Kabuli_Chr08	16145370	(C/A)
14075	CakSNP14075	*Kabuli*	Ca_Kabuli_Chr08	16157015	(C/T)
14076	CakSNP14076	*Kabuli*	Ca_Kabuli_Chr08	16157134	(A/C)
14077	CakSNP14077	*Kabuli*	Ca_Kabuli_Chr08	16158594	(A/C)
14078	CakSNP14078	*Kabuli*	Ca_Kabuli_Chr08	16264310	(G/T)
14079	CakSNP14079	*Kabuli*	Ca_Kabuli_Chr08	16268878	(T/C)
14080	CakSNP14080	*Kabuli*	Ca_Kabuli_Chr08	16278758	(T/C)
14081	CakSNP14081	*Kabuli*	Ca_Kabuli_Chr08	16283660	(C/T)
14082	CakSNP14082	*Kabuli*	Ca_Kabuli_Chr08	16283661	(A/G)
14083	CakSNP14083	*Kabuli*	Ca_Kabuli_Chr08	16283722	(A/G)
14084	CakSNP14084	*Kabuli*	Ca_Kabuli_Chr08	16283738	(G/C)
14085	CakSNP14085	*Kabuli*	Ca_Kabuli_Chr08	16283859	(T/C)
14086	CakSNP14086	*Kabuli*	Ca_Kabuli_Chr08	16284269	(G/A)
14087	CakSNP14087	*Kabuli*	Ca_Kabuli_Chr08	16284279	(A/C)
14088	CakSNP14088	*Kabuli*	Ca_Kabuli_Chr08	16284306	(C/T)
14089	CakSNP14089	*Kabuli*	Ca_Kabuli_Chr08	16309857	(G/T)
14090	CakSNP14090	*Kabuli*	Ca_Kabuli_Chr08	16321524	(A/G)
14091	CakSNP14091	*Kabuli*	Ca_Kabuli_Chr08	16321545	(G/T)
14092	CakSNP14092	*Kabuli*	Ca_Kabuli_Chr08	16366490	(G/A)
14093	CakSNP14093	*Kabuli*	Ca_Kabuli_Chr08	16381928	(T/G)
14094	CakSNP14094	*Kabuli*	Ca_Kabuli_Chr08	16411550	(A/G)
14095	CakSNP14095	*Kabuli*	Ca_Kabuli_Chr08	16412672	(T/C)
14096	CakSNP14096	*Kabuli*	Ca_Kabuli_Chr08	16421407	(G/A)
14097	CakSNP14097	*Kabuli*	Ca_Kabuli_Chr08	16421441	(C/A)

S.N.	SNP IDs	Cultivars	Chromosomes/scaffolds	Physical positions (bp)	SNPs
14098	CakSNP14098	*Kabuli*	Ca_Kabuli_Chr08	16421442	(C/G)
14099	CakSNP14099	*Kabuli*	Ca_Kabuli_Chr08	16421545	(G/T)
14100	CakSNP14100	*Kabuli*	Ca_Kabuli_Chr08	16421497	(C/T)
14101	CakSNP14101	*Kabuli*	Ca_Kabuli_Chr08	16421595	(A/C)
14102	CakSNP14102	*Kabuli*	Ca_Kabuli_Chr08	16432907	(T/G)
14103	CakSNP14103	*Kabuli*	Ca_Kabuli_Chr08	16472919	(T/A)
14104	CakSNP14104	*Kabuli*	Ca_Kabuli_Chr08	16472932	(C/T)
14105	CakSNP14105	*Kabuli*	Ca_Kabuli_Chr08	16472933	(G/C)
14106	CakSNP14106	*Kabuli*	Ca_Kabuli_Chr08	16472999	(A/C)
14107	CakSNP14107	*Kabuli*	Ca_Kabuli_Chr08	16473047	(C/G)
14108	CakSNP14108	*Kabuli*	Ca_Kabuli_Chr08	16473139	(C/T)
14109	CakSNP14109	*Kabuli*	Ca_Kabuli_Chr08	16473154	(T/A)
14110	CakSNP14110	*Kabuli*	Ca_Kabuli_Chr08	16473157	(C/T)
14111	CakSNP14111	*Kabuli*	Ca_Kabuli_Chr08	16473311	(A/G)
14112	CakSNP14112	*Kabuli*	Ca_Kabuli_Chr08	16473290	(A/G)
14113	CakSNP14113	*Kabuli*	Ca_Kabuli_Chr08	16473920	(A/G)
14114	CakSNP14114	*Kabuli*	Ca_Kabuli_Chr08	16473939	(A/G)
14115	CakSNP14115	*Kabuli*	Ca_Kabuli_Chr08	16473987	(C/T)
14116	CakSNP14116	*Kabuli*	Ca_Kabuli_C11058086	341	(G/A)
14117	CakSNP14117	*Kabuli*	Ca_Kabuli_C11058274	143	(T/A)
14118	CakSNP14118	*Kabuli*	Ca_Kabuli_C11062332	412	(G/A)
14119	CakSNP14119	*Kabuli*	Ca_Kabuli_C11062436	381	(T/A)
14120	CakSNP14120	*Kabuli*	Ca_Kabuli_C11062546	677	(G/T)
14121	CakSNP14121	*Kabuli*	Ca_Kabuli_C11076702	989	(G/A)
14122	CakSNP14122	*Kabuli*	Ca_Kabuli_C11078452	885	(G/A)
14123	CakSNP14123	*Kabuli*	Ca_Kabuli_C11079766	1418	(A/C)
14124	CakSNP14124	*Kabuli*	Ca_Kabuli_C11079766	1422	(G/C)
14125	CakSNP14125	*Kabuli*	Ca_Kabuli_C11079766	1412	(A/T)
14126	CakSNP14126	*Kabuli*	Ca_Kabuli_C11080716	1568	(A/C)
14127	CakSNP14127	*Kabuli*	Ca_Kabuli_C11084624	656	(G/A)
14128	CakSNP14128	*Kabuli*	Ca_Kabuli_C11084992	516	(A/G)
14129	CakSNP14129	*Kabuli*	Ca_Kabuli_C11084992	537	(G/T)
14130	CakSNP14130	*Kabuli*	Ca_Kabuli_C11086066	641	(A/C)
14131	CakSNP14131	*Kabuli*	Ca_Kabuli_C11086066	624	(A/C)
14132	CakSNP14132	*Kabuli*	Ca_Kabuli_C11088694	514	(G/C)
14133	CakSNP14133	*Kabuli*	Ca_Kabuli_C11119498	682	(T/A)
14134	CakSNP14134	*Kabuli*	Ca_Kabuli_C11119498	686	(C/A)
14135	CakSNP14135	*Kabuli*	Ca_Kabuli_C11119498	689	(C/A)

S.N.	SNP IDs	Cultivars	Chromosomes/scaffolds	Physical positions (bp)	SNPs
14136	CakSNP14136	*Kabuli*	Ca_Kabuli_C11119498	693	(G/A)
14137	CakSNP14137	*Kabuli*	Ca_Kabuli_C11132768	2177	(G/A)
14138	CakSNP14138	*Kabuli*	Ca_Kabuli_C11142214	2902	(G/T)
14139	CakSNP14139	*Kabuli*	Ca_Kabuli_C11142252	3299	(A/C)
14140	CakSNP14140	*Kabuli*	Ca_Kabuli_C11143608	1389	(A/T)
14141	CakSNP14141	*Kabuli*	Ca_Kabuli_C11147634	3004	(C/A)
14142	CakSNP14142	*Kabuli*	Ca_Kabuli_C11147634	3028	(C/T)
14143	CakSNP14143	*Kabuli*	Ca_Kabuli_C11149092	2181	(A/G)
14144	CakSNP14144	*Kabuli*	Ca_Kabuli_C11149092	2281	(T/C)
14145	CakSNP14145	*Kabuli*	Ca_Kabuli_C11149092	2474	(T/A)
14146	CakSNP14146	*Kabuli*	Ca_Kabuli_C11149092	3190	(T/G)
14147	CakSNP14147	*Kabuli*	Ca_Kabuli_C11149092	3284	(C/G)
14148	CakSNP14148	*Kabuli*	Ca_Kabuli_C11153606	4275	(C/T)
14149	CakSNP14149	*Kabuli*	Ca_Kabuli_C11157320	766	(C/G)
14150	CakSNP14150	*Kabuli*	Ca_Kabuli_C11157320	2169	(T/A)
14151	CakSNP14151	*Kabuli*	Ca_Kabuli_C11157320	2152	(G/C)
14152	CakSNP14152	*Kabuli*	Ca_Kabuli_C11158050	2186	(A/T)
14153	CakSNP14153	*Kabuli*	Ca_Kabuli_C11160518	4522	(A/G)
14154	CakSNP14154	*Kabuli*	Ca_Kabuli_C11162020	3914	(C/T)
14155	CakSNP14155	*Kabuli*	Ca_Kabuli_C11162020	3926	(T/G)
14156	CakSNP14156	*Kabuli*	Ca_Kabuli_C11162020	5515	(G/A)
14157	CakSNP14157	*Kabuli*	Ca_Kabuli_C11164954	5778	(T/A)
14158	CakSNP14158	*Kabuli*	Ca_Kabuli_C11164954	5784	(G/A)
14159	CakSNP14159	*Kabuli*	Ca_Kabuli_C11165474	6285	(T/A)
14160	CakSNP14160	*Kabuli*	Ca_Kabuli_C11165890	154	(C/G)
14161	CakSNP14161	*Kabuli*	Ca_Kabuli_C11174756	8931	(C/G)
14162	CakSNP14162	*Kabuli*	Ca_Kabuli_C11175430	987	(A/G)
14163	CakSNP14163	*Kabuli*	Ca_Kabuli_C11175430	3929	(T/G)
14164	CakSNP14164	*Kabuli*	Ca_Kabuli_C11175430	3936	(G/T)
14165	CakSNP14165	*Kabuli*	Ca_Kabuli_C11175430	3952	(T/C)
14166	CakSNP14166	*Kabuli*	Ca_Kabuli_C11176688	4197	(G/C)
14167	CakSNP14167	*Kabuli*	Ca_Kabuli_C11177172	2529	(T/G)
14168	CakSNP14168	*Kabuli*	Ca_Kabuli_C11177172	2515	(T/A)
14169	CakSNP14169	*Kabuli*	Ca_Kabuli_C11177172	5324	(G/A)
14170	CakSNP14170	*Kabuli*	Ca_Kabuli_C11177748	1264	(T/A)
14171	CakSNP14171	*Kabuli*	Ca_Kabuli_C11178532	2107	(A/T)
14172	CakSNP14172	*Kabuli*	Ca_Kabuli_C11178532	3524	(A/G)
14173	CakSNP14173	*Kabuli*	Ca_Kabuli_C11181782	6215	(A/G)

S.N.	SNP IDs	Cultivars	Chromosomes/scaffolds	Physical positions (bp)	SNPs
14174	CakSNP14174	*Kabuli*	Ca_Kabuli_C11181840	19192	(A/G)
14175	CakSNP14175	*Kabuli*	Ca_Kabuli_C11181840	19202	(T/C)
14176	CakSNP14176	*Kabuli*	Ca_Kabuli_Scaffold1006	40651	(T/A)
14177	CakSNP14177	*Kabuli*	Ca_Kabuli_Scaffold1006	382579	(C/T)
14178	CakSNP14178	*Kabuli*	Ca_Kabuli_Scaffold1006	436923	(T/C)
14179	CakSNP14179	*Kabuli*	Ca_Kabuli_Scaffold1006	644893	(A/C)
14180	CakSNP14180	*Kabuli*	Ca_Kabuli_Scaffold1006	667620	(A/G)
14181	CakSNP14181	*Kabuli*	Ca_Kabuli_Scaffold1006	667713	(G/A)
14182	CakSNP14182	*Kabuli*	Ca_Kabuli_Scaffold1006	674495	(A/G)
14183	CakSNP14183	*Kabuli*	Ca_Kabuli_Scaffold1006	674823	(G/A)
14184	CakSNP14184	*Kabuli*	Ca_Kabuli_Scaffold1006	674826	(C/T)
14185	CakSNP14185	*Kabuli*	Ca_Kabuli_Scaffold1006	674844	(T/C)
14186	CakSNP14186	*Kabuli*	Ca_Kabuli_Scaffold1006	674847	(T/G)
14187	CakSNP14187	*Kabuli*	Ca_Kabuli_Scaffold1006	674862	(G/A)
14188	CakSNP14188	*Kabuli*	Ca_Kabuli_Scaffold1006	674865	(T/C)
14189	CakSNP14189	*Kabuli*	Ca_Kabuli_Scaffold1006	674871	(A/G)
14190	CakSNP14190	*Kabuli*	Ca_Kabuli_Scaffold1006	674881	(G/A)
14191	CakSNP14191	*Kabuli*	Ca_Kabuli_Scaffold1006	674981	(G/A)
14192	CakSNP14192	*Kabuli*	Ca_Kabuli_Scaffold1006	674946	(G/A)
14193	CakSNP14193	*Kabuli*	Ca_Kabuli_Scaffold1010	90092	(G/A)
14194	CakSNP14194	*Kabuli*	Ca_Kabuli_Scaffold1013	56140	(G/A)
14195	CakSNP14195	*Kabuli*	Ca_Kabuli_Scaffold1013	56116	(C/T)
14196	CakSNP14196	*Kabuli*	Ca_Kabuli_Scaffold1013	56114	(C/A)
14197	CakSNP14197	*Kabuli*	Ca_Kabuli_Scaffold1013	56075	(T/A)
14198	CakSNP14198	*Kabuli*	Ca_Kabuli_Scaffold1013	56074	(C/T)
14199	CakSNP14199	*Kabuli*	Ca_Kabuli_Scaffold1013	56069	(C/T)
14200	CakSNP14200	*Kabuli*	Ca_Kabuli_Scaffold10186	416	(C/T)
14201	CakSNP14201	*Kabuli*	Ca_Kabuli_Scaffold10186	410	(A/C)
14202	CakSNP14202	*Kabuli*	Ca_Kabuli_Scaffold10186	378	(A/T)
14203	CakSNP14203	*Kabuli*	Ca_Kabuli_Scaffold10186	354	(T/A)
14204	CakSNP14204	*Kabuli*	Ca_Kabuli_Scaffold10186	397	(G/A)
14205	CakSNP14205	*Kabuli*	Ca_Kabuli_Scaffold10186	573	(T/G)
14206	CakSNP14206	*Kabuli*	Ca_Kabuli_Scaffold102	82102	(T/C)
14207	CakSNP14207	*Kabuli*	Ca_Kabuli_Scaffold102	82107	(C/A)
14208	CakSNP14208	*Kabuli*	Ca_Kabuli_Scaffold1027	256612	(A/C)
14209	CakSNP14209	*Kabuli*	Ca_Kabuli_Scaffold1034	73684	(G/A)
14210	CakSNP14210	*Kabuli*	Ca_Kabuli_Scaffold1034	73695	(G/T)
14211	CakSNP14211	*Kabuli*	Ca_Kabuli_Scaffold10420	19974	(A/G)

S.N.	SNP IDs	Cultivars	Chromosomes/scaffolds	Physical positions (bp)	SNPs
14212	CakSNP14212	*Kabuli*	Ca_Kabuli_Scaffold10420	31513	(C/T)
14213	CakSNP14213	*Kabuli*	Ca_Kabuli_Scaffold10420	31563	(A/G)
14214	CakSNP14214	*Kabuli*	Ca_Kabuli_Scaffold10420	31817	(C/T)
14215	CakSNP14215	*Kabuli*	Ca_Kabuli_Scaffold10420	40193	(G/T)
14216	CakSNP14216	*Kabuli*	Ca_Kabuli_Scaffold1043	16328	(A/T)
14217	CakSNP14217	*Kabuli*	Ca_Kabuli_Scaffold1043	16331	(G/T)
14218	CakSNP14218	*Kabuli*	Ca_Kabuli_Scaffold1043	16333	(A/T)
14219	CakSNP14219	*Kabuli*	Ca_Kabuli_Scaffold1043	16313	(A/G)
14220	CakSNP14220	*Kabuli*	Ca_Kabuli_Scaffold1047	235352	(T/C)
14221	CakSNP14221	*Kabuli*	Ca_Kabuli_Scaffold1047	577874	(T/G)
14222	CakSNP14222	*Kabuli*	Ca_Kabuli_Scaffold1050	3398	(G/A)
14223	CakSNP14223	*Kabuli*	Ca_Kabuli_Scaffold1050	3415	(C/T)
14224	CakSNP14224	*Kabuli*	Ca_Kabuli_Scaffold1050	3463	(C/T)
14225	CakSNP14225	*Kabuli*	Ca_Kabuli_Scaffold1050	14057	(C/A)
14226	CakSNP14226	*Kabuli*	Ca_Kabuli_Scaffold1050	126986	(T/G)
14227	CakSNP14227	*Kabuli*	Ca_Kabuli_Scaffold1050	127022	(G/A)
14228	CakSNP14228	*Kabuli*	Ca_Kabuli_Scaffold1050	127023	(G/T)
14229	CakSNP14229	*Kabuli*	Ca_Kabuli_Scaffold1050	127054	(C/T)
14230	CakSNP14230	*Kabuli*	Ca_Kabuli_Scaffold1050	128763	(A/G)
14231	CakSNP14231	*Kabuli*	Ca_Kabuli_Scaffold1055	344459	(G/A)
14232	CakSNP14232	*Kabuli*	Ca_Kabuli_Scaffold1055	344443	(G/A)
14233	CakSNP14233	*Kabuli*	Ca_Kabuli_Scaffold1055	344399	(C/T)
14234	CakSNP14234	*Kabuli*	Ca_Kabuli_Scaffold1060	3278	(A/C)
14235	CakSNP14235	*Kabuli*	Ca_Kabuli_Scaffold1060	3310	(G/T)
14236	CakSNP14236	*Kabuli*	Ca_Kabuli_Scaffold1060	394867	(C/T)
14237	CakSNP14237	*Kabuli*	Ca_Kabuli_Scaffold1060	395029	(C/T)
14238	CakSNP14238	*Kabuli*	Ca_Kabuli_Scaffold1060	395042	(A/G)
14239	CakSNP14239	*Kabuli*	Ca_Kabuli_Scaffold1061	63613	(A/G)
14240	CakSNP14240	*Kabuli*	Ca_Kabuli_Scaffold1061	63620	(C/T)
14241	CakSNP14241	*Kabuli*	Ca_Kabuli_Scaffold1065	142678	(A/C)
14242	CakSNP14242	*Kabuli*	Ca_Kabuli_Scaffold1067	38623	(C/A)
14243	CakSNP14243	*Kabuli*	Ca_Kabuli_Scaffold1081	768	(A/C)
14244	CakSNP14244	*Kabuli*	Ca_Kabuli_Scaffold1081	798	(T/C)
14245	CakSNP14245	*Kabuli*	Ca_Kabuli_Scaffold1081	800	(T/C)
14246	CakSNP14246	*Kabuli*	Ca_Kabuli_Scaffold1081	805	(A/C)
14247	CakSNP14247	*Kabuli*	Ca_Kabuli_Scaffold1081	818	(A/C)
14248	CakSNP14248	*Kabuli*	Ca_Kabuli_Scaffold1089	1154	(C/A)
14249	CakSNP14249	*Kabuli*	Ca_Kabuli_Scaffold1089	1240	(T/G)

S.N.	SNP IDs	Cultivars	Chromosomes/scaffolds	Physical positions (bp)	SNPs
14250	CakSNP14250	*Kabuli*	Ca_Kabuli_Scaffold1089	1281	(G/A)
14251	CakSNP14251	*Kabuli*	Ca_Kabuli_Scaffold1089	1278	(T/C)
14252	CakSNP14252	*Kabuli*	Ca_Kabuli_Scaffold1089	3160	(C/A)
14253	CakSNP14253	*Kabuli*	Ca_Kabuli_Scaffold1089	3173	(G/T)
14254	CakSNP14254	*Kabuli*	Ca_Kabuli_Scaffold1089	3204	(G/C)
14255	CakSNP14255	*Kabuli*	Ca_Kabuli_Scaffold1089	3236	(T/G)
14256	CakSNP14256	*Kabuli*	Ca_Kabuli_Scaffold1089	3249	(T/G)
14257	CakSNP14257	*Kabuli*	Ca_Kabuli_Scaffold1089	3248	(C/T)
14258	CakSNP14258	*Kabuli*	Ca_Kabuli_Scaffold1089	3240	(G/C)
14259	CakSNP14259	*Kabuli*	Ca_Kabuli_Scaffold1089	3228	(C/T)
14260	CakSNP14260	*Kabuli*	Ca_Kabuli_Scaffold1089	3191	(G/A)
14261	CakSNP14261	*Kabuli*	Ca_Kabuli_Scaffold1089	3274	(T/C)
14262	CakSNP14262	*Kabuli*	Ca_Kabuli_Scaffold1089	137675	(T/C)
14263	CakSNP14263	*Kabuli*	Ca_Kabuli_Scaffold1089	137679	(G/T)
14264	CakSNP14264	*Kabuli*	Ca_Kabuli_Scaffold109_1	156485	(C/T)
14265	CakSNP14265	*Kabuli*	Ca_Kabuli_Scaffold109_1	230701	(G/C)
14266	CakSNP14266	*Kabuli*	Ca_Kabuli_Scaffold109_1	244141	(A/C)
14267	CakSNP14267	*Kabuli*	Ca_Kabuli_Scaffold109_1	244150	(C/T)
14268	CakSNP14268	*Kabuli*	Ca_Kabuli_Scaffold109_1	244156	(A/C)
14269	CakSNP14269	*Kabuli*	Ca_Kabuli_Scaffold109_1	244176	(A/T)
14270	CakSNP14270	*Kabuli*	Ca_Kabuli_Scaffold109_1	328757	(C/T)
14271	CakSNP14271	*Kabuli*	Ca_Kabuli_Scaffold109_1	343415	(G/A)
14272	CakSNP14272	*Kabuli*	Ca_Kabuli_Scaffold109_1	343454	(G/A)
14273	CakSNP14273	*Kabuli*	Ca_Kabuli_Scaffold109_1	385548	(T/C)
14274	CakSNP14274	*Kabuli*	Ca_Kabuli_Scaffold109_1	385578	(C/G)
14275	CakSNP14275	*Kabuli*	Ca_Kabuli_Scaffold109_1	385602	(C/T)
14276	CakSNP14276	*Kabuli*	Ca_Kabuli_Scaffold109_1	385660	(G/A)
14277	CakSNP14277	*Kabuli*	Ca_Kabuli_Scaffold109_1	385653	(C/T)
14278	CakSNP14278	*Kabuli*	Ca_Kabuli_Scaffold109_1	442149	(A/T)
14279	CakSNP14279	*Kabuli*	Ca_Kabuli_Scaffold109_1	535504	(G/C)
14280	CakSNP14280	*Kabuli*	Ca_Kabuli_Scaffold109_1	586181	(A/C)
14281	CakSNP14281	*Kabuli*	Ca_Kabuli_Scaffold109_1	586286	(C/T)
14282	CakSNP14282	*Kabuli*	Ca_Kabuli_Scaffold109_1	696869	(A/C)
14283	CakSNP14283	*Kabuli*	Ca_Kabuli_Scaffold109_1	734137	(G/A)
14284	CakSNP14284	*Kabuli*	Ca_Kabuli_Scaffold109_1	734141	(C/T)
14285	CakSNP14285	*Kabuli*	Ca_Kabuli_Scaffold109_1	741607	(G/A)
14286	CakSNP14286	*Kabuli*	Ca_Kabuli_Scaffold109_1	763160	(G/A)
14287	CakSNP14287	*Kabuli*	Ca_Kabuli_Scaffold1109	150672	(G/A)

S.N.	SNP IDs	Cultivars	Chromosomes/scaffolds	Physical positions (bp)	SNPs
14288	CakSNP14288	*Kabuli*	Ca_Kabuli_Scaffold1109	150720	(G/A)
14289	CakSNP14289	*Kabuli*	Ca_Kabuli_Scaffold1109	150676	(C/T)
14290	CakSNP14290	*Kabuli*	Ca_Kabuli_Scaffold1115	6234	(G/C)
14291	CakSNP14291	*Kabuli*	Ca_Kabuli_Scaffold1115	131039	(G/A)
14292	CakSNP14292	*Kabuli*	Ca_Kabuli_Scaffold1115	153349	(T/C)
14293	CakSNP14293	*Kabuli*	Ca_Kabuli_Scaffold1118	2215	(T/C)
14294	CakSNP14294	*Kabuli*	Ca_Kabuli_Scaffold1118	2224	(T/A)
14295	CakSNP14295	*Kabuli*	Ca_Kabuli_Scaffold1118	2225	(C/T)
14296	CakSNP14296	*Kabuli*	Ca_Kabuli_Scaffold1118	2226	(T/G)
14297	CakSNP14297	*Kabuli*	Ca_Kabuli_Scaffold1118	2315	(T/C)
14298	CakSNP14298	*Kabuli*	Ca_Kabuli_Scaffold1118	9568	(A/G)
14299	CakSNP14299	*Kabuli*	Ca_Kabuli_Scaffold1118	9556	(C/T)
14300	CakSNP14300	*Kabuli*	Ca_Kabuli_Scaffold1128	94196	(T/A)
14301	CakSNP14301	*Kabuli*	Ca_Kabuli_Scaffold1128	112702	(G/A)
14302	CakSNP14302	*Kabuli*	Ca_Kabuli_Scaffold1128	341416	(T/A)
14303	CakSNP14303	*Kabuli*	Ca_Kabuli_Scaffold1128	360567	(T/A)
14304	CakSNP14304	*Kabuli*	Ca_Kabuli_Scaffold1128	360529	(A/C)
14305	CakSNP14305	*Kabuli*	Ca_Kabuli_Scaffold1128	423553	(A/G)
14306	CakSNP14306	*Kabuli*	Ca_Kabuli_Scaffold1132	26497	(C/T)
14307	CakSNP14307	*Kabuli*	Ca_Kabuli_Scaffold1132	26492	(G/A)
14308	CakSNP14308	*Kabuli*	Ca_Kabuli_Scaffold1134	50630	(T/C)
14309	CakSNP14309	*Kabuli*	Ca_Kabuli_Scaffold1151	228312	(C/G)
14310	CakSNP14310	*Kabuli*	Ca_Kabuli_Scaffold1151	228304	(A/T)
14311	CakSNP14311	*Kabuli*	Ca_Kabuli_Scaffold1151	228291	(C/T)
14312	CakSNP14312	*Kabuli*	Ca_Kabuli_Scaffold1152	9541	(A/G)
14313	CakSNP14313	*Kabuli*	Ca_Kabuli_Scaffold1176_2	52186	(C/T)
14314	CakSNP14314	*Kabuli*	Ca_Kabuli_Scaffold1176_2	52146	(T/C)
14315	CakSNP14315	*Kabuli*	Ca_Kabuli_Scaffold1176_2	52142	(G/T)
14316	CakSNP14316	*Kabuli*	Ca_Kabuli_Scaffold1176_2	52125	(C/T)
14317	CakSNP14317	*Kabuli*	Ca_Kabuli_Scaffold1176_2	52124	(C/T)
14318	CakSNP14318	*Kabuli*	Ca_Kabuli_Scaffold1176_2	330840	(C/A)
14319	CakSNP14319	*Kabuli*	Ca_Kabuli_Scaffold118	206562	(A/G)
14320	CakSNP14320	*Kabuli*	Ca_Kabuli_Scaffold118	303784	(A/C)
14321	CakSNP14321	*Kabuli*	Ca_Kabuli_Scaffold118	332673	(T/C)
14322	CakSNP14322	*Kabuli*	Ca_Kabuli_Scaffold1180	114508	(C/A)
14323	CakSNP14323	*Kabuli*	Ca_Kabuli_Scaffold1185	944	(G/A)
14324	CakSNP14324	*Kabuli*	Ca_Kabuli_Scaffold1185	1110	(G/A)
14325	CakSNP14325	*Kabuli*	Ca_Kabuli_Scaffold1185	1134	(T/A)

**S.N.**	**SNP IDs**	**Cultivars**	**Chromosomes/scaffolds**	**Physical positions (bp)**	**SNPs**
14326	CakSNP14326	*Kabuli*	Ca_Kabuli_Scaffold1185	1098	(T/C)
14327	CakSNP14327	*Kabuli*	Ca_Kabuli_Scaffold119	12259	(A/G)
14328	CakSNP14328	*Kabuli*	Ca_Kabuli_Scaffold119	12263	(C/A)
14329	CakSNP14329	*Kabuli*	Ca_Kabuli_Scaffold119	12267	(C/A)
14330	CakSNP14330	*Kabuli*	Ca_Kabuli_Scaffold119	12279	(G/A)
14331	CakSNP14331	*Kabuli*	Ca_Kabuli_Scaffold1196	70506	(G/A)
14332	CakSNP14332	*Kabuli*	Ca_Kabuli_Scaffold1196	70552	(A/C)
14333	CakSNP14333	*Kabuli*	Ca_Kabuli_Scaffold1196	70549	(G/A)
14334	CakSNP14334	*Kabuli*	Ca_Kabuli_Scaffold1196	70547	(G/C)
14335	CakSNP14335	*Kabuli*	Ca_Kabuli_Scaffold1196	70535	(G/A)
14336	CakSNP14336	*Kabuli*	Ca_Kabuli_Scaffold1196	70530	(C/T)
14337	CakSNP14337	*Kabuli*	Ca_Kabuli_Scaffold1196	70526	(T/C)
14338	CakSNP14338	*Kabuli*	Ca_Kabuli_Scaffold1196	70520	(G/A)
14339	CakSNP14339	*Kabuli*	Ca_Kabuli_Scaffold1196	70503	(C/T)
14340	CakSNP14340	*Kabuli*	Ca_Kabuli_Scaffold1196	203001	(G/A)
14341	CakSNP14341	*Kabuli*	Ca_Kabuli_Scaffold1197	219637	(A/T)
14342	CakSNP14342	*Kabuli*	Ca_Kabuli_Scaffold1197	344239	(A/C)
14343	CakSNP14343	*Kabuli*	Ca_Kabuli_Scaffold1197	402506	(T/C)
14344	CakSNP14344	*Kabuli*	Ca_Kabuli_Scaffold1197	407641	(G/A)
14345	CakSNP14345	*Kabuli*	Ca_Kabuli_Scaffold1197	407658	(C/G)
14346	CakSNP14346	*Kabuli*	Ca_Kabuli_Scaffold1197	407691	(T/C)
14347	CakSNP14347	*Kabuli*	Ca_Kabuli_Scaffold1197	407695	(T/C)
14348	CakSNP14348	*Kabuli*	Ca_Kabuli_Scaffold1197	447890	(C/T)
14349	CakSNP14349	*Kabuli*	Ca_Kabuli_Scaffold1197	520164	(T/A)
14350	CakSNP14350	*Kabuli*	Ca_Kabuli_Scaffold1197	520153	(C/G)
14351	CakSNP14351	*Kabuli*	Ca_Kabuli_Scaffold1197	520148	(A/T)
14352	CakSNP14352	*Kabuli*	Ca_Kabuli_Scaffold12	107756	(G/A)
14353	CakSNP14353	*Kabuli*	Ca_Kabuli_Scaffold12	200573	(G/C)
14354	CakSNP14354	*Kabuli*	Ca_Kabuli_Scaffold12	331877	(G/A)
14355	CakSNP14355	*Kabuli*	Ca_Kabuli_Scaffold12	331890	(A/G)
14356	CakSNP14356	*Kabuli*	Ca_Kabuli_Scaffold12	332909	(C/T)
14357	CakSNP14357	*Kabuli*	Ca_Kabuli_Scaffold12	332921	(G/A)
14358	CakSNP14358	*Kabuli*	Ca_Kabuli_Scaffold12	332963	(C/T)
14359	CakSNP14359	*Kabuli*	Ca_Kabuli_Scaffold1202	46904	(C/T)
14360	CakSNP14360	*Kabuli*	Ca_Kabuli_Scaffold1202	46945	(G/A)
14361	CakSNP14361	*Kabuli*	Ca_Kabuli_Scaffold1215	40741	(G/T)
14362	CakSNP14362	*Kabuli*	Ca_Kabuli_Scaffold1215	40793	(A/G)
14363	CakSNP14363	*Kabuli*	Ca_Kabuli_Scaffold1215	40797	(A/T)

S.N.	SNP IDs	Cultivars	Chromosomes/scaffolds	Physical positions (bp)	SNPs
14364	CakSNP14364	*Kabuli*	Ca_Kabuli_Scaffold1215	40817	(G/A)
14365	CakSNP14365	*Kabuli*	Ca_Kabuli_Scaffold1215	40814	(A/T)
14366	CakSNP14366	*Kabuli*	Ca_Kabuli_Scaffold1219	34791	(G/A)
14367	CakSNP14367	*Kabuli*	Ca_Kabuli_Scaffold1219	85015	(A/C)
14368	CakSNP14368	*Kabuli*	Ca_Kabuli_Scaffold1259	71428	(C/T)
14369	CakSNP14369	*Kabuli*	Ca_Kabuli_Scaffold1259	71574	(A/T)
14370	CakSNP14370	*Kabuli*	Ca_Kabuli_Scaffold1259	71649	(C/T)
14371	CakSNP14371	*Kabuli*	Ca_Kabuli_Scaffold127	28809	(A/T)
14372	CakSNP14372	*Kabuli*	Ca_Kabuli_Scaffold127	28780	(G/A)
14373	CakSNP14373	*Kabuli*	Ca_Kabuli_Scaffold127	28778	(C/T)
14374	CakSNP14374	*Kabuli*	Ca_Kabuli_Scaffold127	28776	(T/G)
14375	CakSNP14375	*Kabuli*	Ca_Kabuli_Scaffold127	28772	(G/A)
14376	CakSNP14376	*Kabuli*	Ca_Kabuli_Scaffold1272	57303	(C/A)
14377	CakSNP14377	*Kabuli*	Ca_Kabuli_Scaffold1272	57356	(C/G)
14378	CakSNP14378	*Kabuli*	Ca_Kabuli_Scaffold128	19256	(A/C)
14379	CakSNP14379	*Kabuli*	Ca_Kabuli_Scaffold128	19315	(C/T)
14380	CakSNP14380	*Kabuli*	Ca_Kabuli_Scaffold128	52881	(G/A)
14381	CakSNP14381	*Kabuli*	Ca_Kabuli_Scaffold128	52855	(G/C)
14382	CakSNP14382	*Kabuli*	Ca_Kabuli_Scaffold128	52844	(A/T)
14383	CakSNP14383	*Kabuli*	Ca_Kabuli_Scaffold128	263590	(G/T)
14384	CakSNP14384	*Kabuli*	Ca_Kabuli_Scaffold128	263586	(T/A)
14385	CakSNP14385	*Kabuli*	Ca_Kabuli_Scaffold128	263572	(T/A)
14386	CakSNP14386	*Kabuli*	Ca_Kabuli_Scaffold128	334180	(G/T)
14387	CakSNP14387	*Kabuli*	Ca_Kabuli_Scaffold128	334129	(G/A)
14388	CakSNP14388	*Kabuli*	Ca_Kabuli_Scaffold128	593782	(T/C)
14389	CakSNP14389	*Kabuli*	Ca_Kabuli_Scaffold128	860975	(G/A)
14390	CakSNP14390	*Kabuli*	Ca_Kabuli_Scaffold128	860981	(T/A)
14391	CakSNP14391	*Kabuli*	Ca_Kabuli_Scaffold128	860983	(A/T)
14392	CakSNP14392	*Kabuli*	Ca_Kabuli_Scaffold128	860987	(C/T)
14393	CakSNP14393	*Kabuli*	Ca_Kabuli_Scaffold128	861003	(C/A)
14394	CakSNP14394	*Kabuli*	Ca_Kabuli_Scaffold128	861010	(C/T)
14395	CakSNP14395	*Kabuli*	Ca_Kabuli_Scaffold128	861022	(T/C)
14396	CakSNP14396	*Kabuli*	Ca_Kabuli_Scaffold128	861028	(T/C)
14397	CakSNP14397	*Kabuli*	Ca_Kabuli_Scaffold128	861072	(G/T)
14398	CakSNP14398	*Kabuli*	Ca_Kabuli_Scaffold128	861053	(G/A)
14399	CakSNP14399	*Kabuli*	Ca_Kabuli_Scaffold128	861036	(G/A)
14400	CakSNP14400	*Kabuli*	Ca_Kabuli_Scaffold128	861015	(C/T)
14401	CakSNP14401	*Kabuli*	Ca_Kabuli_Scaffold1281	211456	(A/T)

S.N.	SNP IDs	Cultivars	Chromosomes/scaffolds	Physical positions (bp)	SNPs
14402	CakSNP14402	*Kabuli*	Ca_Kabuli_Scaffold1281	239742	(T/C)
14403	CakSNP14403	*Kabuli*	Ca_Kabuli_Scaffold1281	244153	(A/C)
14404	CakSNP14404	*Kabuli*	Ca_Kabuli_Scaffold1281	258565	(G/A)
14405	CakSNP14405	*Kabuli*	Ca_Kabuli_Scaffold1281	316037	(A/G)
14406	CakSNP14406	*Kabuli*	Ca_Kabuli_Scaffold1281	396888	(G/A)
14407	CakSNP14407	*Kabuli*	Ca_Kabuli_Scaffold1281	396996	(C/T)
14408	CakSNP14408	*Kabuli*	Ca_Kabuli_Scaffold1281	396992	(A/G)
14409	CakSNP14409	*Kabuli*	Ca_Kabuli_Scaffold1281	398693	(G/A)
14410	CakSNP14410	*Kabuli*	Ca_Kabuli_Scaffold1281	398729	(T/G)
14411	CakSNP14411	*Kabuli*	Ca_Kabuli_Scaffold1281	398887	(G/A)
14412	CakSNP14412	*Kabuli*	Ca_Kabuli_Scaffold1281	408214	(C/T)
14413	CakSNP14413	*Kabuli*	Ca_Kabuli_Scaffold1281	408170	(C/T)
14414	CakSNP14414	*Kabuli*	Ca_Kabuli_Scaffold1281	526446	(C/T)
14415	CakSNP14415	*Kabuli*	Ca_Kabuli_Scaffold1281	526442	(T/C)
14416	CakSNP14416	*Kabuli*	Ca_Kabuli_Scaffold1281	527012	(C/T)
14417	CakSNP14417	*Kabuli*	Ca_Kabuli_Scaffold1281	527008	(T/C)
14418	CakSNP14418	*Kabuli*	Ca_Kabuli_Scaffold1285	68330	(G/A)
14419	CakSNP14419	*Kabuli*	Ca_Kabuli_Scaffold1285	68327	(G/T)
14420	CakSNP14420	*Kabuli*	Ca_Kabuli_Scaffold1285	68324	(T/C)
14421	CakSNP14421	*Kabuli*	Ca_Kabuli_Scaffold1285	68315	(T/C)
14422	CakSNP14422	*Kabuli*	Ca_Kabuli_Scaffold1285	68311	(G/A)
14423	CakSNP14423	*Kabuli*	Ca_Kabuli_Scaffold1285	96310	(A/C)
14424	CakSNP14424	*Kabuli*	Ca_Kabuli_Scaffold1285	96358	(T/C)
14425	CakSNP14425	*Kabuli*	Ca_Kabuli_Scaffold1285	96429	(C/T)
14426	CakSNP14426	*Kabuli*	Ca_Kabuli_Scaffold1285	96428	(G/A)
14427	CakSNP14427	*Kabuli*	Ca_Kabuli_Scaffold1285	96414	(C/T)
14428	CakSNP14428	*Kabuli*	Ca_Kabuli_Scaffold1285	96411	(G/A)
14429	CakSNP14429	*Kabuli*	Ca_Kabuli_Scaffold1285	96409	(C/T)
14430	CakSNP14430	*Kabuli*	Ca_Kabuli_Scaffold1285	96399	(C/T)
14431	CakSNP14431	*Kabuli*	Ca_Kabuli_Scaffold1285	105553	(T/C)
14432	CakSNP14432	*Kabuli*	Ca_Kabuli_Scaffold1285	105507	(A/T)
14433	CakSNP14433	*Kabuli*	Ca_Kabuli_Scaffold1285	105504	(G/A)
14434	CakSNP14434	*Kabuli*	Ca_Kabuli_Scaffold1285	192068	(T/G)
14435	CakSNP14435	*Kabuli*	Ca_Kabuli_Scaffold1285	240762	(C/T)
14436	CakSNP14436	*Kabuli*	Ca_Kabuli_Scaffold1285	240735	(G/A)
14437	CakSNP14437	*Kabuli*	Ca_Kabuli_Scaffold1285	240730	(T/C)
14438	CakSNP14438	*Kabuli*	Ca_Kabuli_Scaffold1285	240723	(C/T)
14439	CakSNP14439	*Kabuli*	Ca_Kabuli_Scaffold1301_1	43433	(T/C)

S.N.	SNP IDs	Cultivars	Chromosomes/scaffolds	Physical positions (bp)	SNPs
14440	CakSNP14440	*Kabuli*	Ca_Kabuli_Scaffold1301_1	45565	(A/C)
14441	CakSNP14441	*Kabuli*	Ca_Kabuli_Scaffold1301_1	45566	(G/A)
14442	CakSNP14442	*Kabuli*	Ca_Kabuli_Scaffold1301_1	58243	(A/G)
14443	CakSNP14443	*Kabuli*	Ca_Kabuli_Scaffold1301_1	58337	(C/T)
14444	CakSNP14444	*Kabuli*	Ca_Kabuli_Scaffold1301_1	58460	(A/T)
14445	CakSNP14445	*Kabuli*	Ca_Kabuli_Scaffold1301_1	63148	(T/C)
14446	CakSNP14446	*Kabuli*	Ca_Kabuli_Scaffold1301_1	95495	(G/A)
14447	CakSNP14447	*Kabuli*	Ca_Kabuli_Scaffold1301_1	95497	(G/A)
14448	CakSNP14448	*Kabuli*	Ca_Kabuli_Scaffold1301_1	95602	(T/C)
14449	CakSNP14449	*Kabuli*	Ca_Kabuli_Scaffold1301_1	95533	(C/G)
14450	CakSNP14450	*Kabuli*	Ca_Kabuli_Scaffold1301_1	122496	(T/A)
14451	CakSNP14451	*Kabuli*	Ca_Kabuli_Scaffold1301_1	122563	(T/C)
14452	CakSNP14452	*Kabuli*	Ca_Kabuli_Scaffold1301_1	154342	(A/C)
14453	CakSNP14453	*Kabuli*	Ca_Kabuli_Scaffold1301_1	157390	(A/G)
14454	CakSNP14454	*Kabuli*	Ca_Kabuli_Scaffold1301_1	218538	(G/A)
14455	CakSNP14455	*Kabuli*	Ca_Kabuli_Scaffold1308	44260	(A/G)
14456	CakSNP14456	*Kabuli*	Ca_Kabuli_Scaffold1309	49259	(G/A)
14457	CakSNP14457	*Kabuli*	Ca_Kabuli_Scaffold1312	98400	(T/C)
14458	CakSNP14458	*Kabuli*	Ca_Kabuli_Scaffold1313	18173	(A/G)
14459	CakSNP14459	*Kabuli*	Ca_Kabuli_Scaffold1313	18412	(G/A)
14460	CakSNP14460	*Kabuli*	Ca_Kabuli_Scaffold1313	227732	(G/A)
14461	CakSNP14461	*Kabuli*	Ca_Kabuli_Scaffold1313	227744	(C/T)
14462	CakSNP14462	*Kabuli*	Ca_Kabuli_Scaffold1315	115480	(G/A)
14463	CakSNP14463	*Kabuli*	Ca_Kabuli_Scaffold1315	115476	(A/C)
14464	CakSNP14464	*Kabuli*	Ca_Kabuli_Scaffold1315	115471	(G/A)
14465	CakSNP14465	*Kabuli*	Ca_Kabuli_Scaffold1315	115446	(C/A)
14466	CakSNP14466	*Kabuli*	Ca_Kabuli_Scaffold1315	115481	(G/T)
14467	CakSNP14467	*Kabuli*	Ca_Kabuli_Scaffold132	153364	(A/G)
14468	CakSNP14468	*Kabuli*	Ca_Kabuli_Scaffold132	153361	(A/G)
14469	CakSNP14469	*Kabuli*	Ca_Kabuli_Scaffold1324	228151	(G/A)
14470	CakSNP14470	*Kabuli*	Ca_Kabuli_Scaffold1324	228159	(A/C)
14471	CakSNP14471	*Kabuli*	Ca_Kabuli_Scaffold1324	262208	(C/G)
14472	CakSNP14472	*Kabuli*	Ca_Kabuli_Scaffold134	218290	(C/T)
14473	CakSNP14473	*Kabuli*	Ca_Kabuli_Scaffold134	218352	(T/G)
14474	CakSNP14474	*Kabuli*	Ca_Kabuli_Scaffold134	218336	(T/G)
14475	CakSNP14475	*Kabuli*	Ca_Kabuli_Scaffold134	223318	(C/T)
14476	CakSNP14476	*Kabuli*	Ca_Kabuli_Scaffold134	223303	(T/C)
14477	CakSNP14477	*Kabuli*	Ca_Kabuli_Scaffold134	233007	(C/T)

**S.N.**	**SNP IDs**	**Cultivars**	**Chromosomes/scaffolds**	**Physical positions (bp)**	**SNPs**
14478	CakSNP14478	*Kabuli*	Ca_Kabuli_Scaffold134	275661	(T/A)
14479	CakSNP14479	*Kabuli*	Ca_Kabuli_Scaffold1348_1	84644	(G/C)
14480	CakSNP14480	*Kabuli*	Ca_Kabuli_Scaffold1348_1	134575	(T/C)
14481	CakSNP14481	*Kabuli*	Ca_Kabuli_Scaffold1348_1	134572	(G/A)
14482	CakSNP14482	*Kabuli*	Ca_Kabuli_Scaffold1348_1	294458	(A/G)
14483	CakSNP14483	*Kabuli*	Ca_Kabuli_Scaffold1348_1	294456	(A/G)
14484	CakSNP14484	*Kabuli*	Ca_Kabuli_Scaffold1348_1	380156	(A/T)
14485	CakSNP14485	*Kabuli*	Ca_Kabuli_Scaffold1348_1	380225	(T/G)
14486	CakSNP14486	*Kabuli*	Ca_Kabuli_Scaffold1348_1	380227	(C/G)
14487	CakSNP14487	*Kabuli*	Ca_Kabuli_Scaffold1348_1	380244	(C/G)
14488	CakSNP14488	*Kabuli*	Ca_Kabuli_Scaffold1348_1	380289	(C/T)
14489	CakSNP14489	*Kabuli*	Ca_Kabuli_Scaffold1348_1	380336	(G/C)
14490	CakSNP14490	*Kabuli*	Ca_Kabuli_Scaffold1348_1	381026	(G/T)
14491	CakSNP14491	*Kabuli*	Ca_Kabuli_Scaffold1348_1	452011	(G/T)
14492	CakSNP14492	*Kabuli*	Ca_Kabuli_Scaffold1348_1	454342	(G/T)
14493	CakSNP14493	*Kabuli*	Ca_Kabuli_Scaffold1348_1	515602	(A/C)
14494	CakSNP14494	*Kabuli*	Ca_Kabuli_Scaffold1348_1	535984	(G/T)
14495	CakSNP14495	*Kabuli*	Ca_Kabuli_Scaffold1348_1	552192	(C/A)
14496	CakSNP14496	*Kabuli*	Ca_Kabuli_Scaffold1348_1	552299	(T/C)
14497	CakSNP14497	*Kabuli*	Ca_Kabuli_Scaffold1348_1	552342	(G/A)
14498	CakSNP14498	*Kabuli*	Ca_Kabuli_Scaffold1348_1	552328	(A/G)
14499	CakSNP14499	*Kabuli*	Ca_Kabuli_Scaffold1348_1	553863	(G/T)
14500	CakSNP14500	*Kabuli*	Ca_Kabuli_Scaffold1348_1	553978	(C/T)
14501	CakSNP14501	*Kabuli*	Ca_Kabuli_Scaffold1348_1	554003	(G/C)
14502	CakSNP14502	*Kabuli*	Ca_Kabuli_Scaffold1348_1	554134	(A/G)
14503	CakSNP14503	*Kabuli*	Ca_Kabuli_Scaffold1348_1	554215	(T/G)
14504	CakSNP14504	*Kabuli*	Ca_Kabuli_Scaffold1348_1	651906	(A/T)
14505	CakSNP14505	*Kabuli*	Ca_Kabuli_Scaffold1348_1	652067	(A/G)
14506	CakSNP14506	*Kabuli*	Ca_Kabuli_Scaffold1348_1	667541	(A/C)
14507	CakSNP14507	*Kabuli*	Ca_Kabuli_Scaffold1348_1	667504	(C/T)
14508	CakSNP14508	*Kabuli*	Ca_Kabuli_Scaffold1348_1	679305	(T/C)
14509	CakSNP14509	*Kabuli*	Ca_Kabuli_Scaffold1348_1	680734	(G/T)
14510	CakSNP14510	*Kabuli*	Ca_Kabuli_Scaffold1348_1	680730	(C/T)
14511	CakSNP14511	*Kabuli*	Ca_Kabuli_Scaffold1348_1	680718	(A/T)
14512	CakSNP14512	*Kabuli*	Ca_Kabuli_Scaffold1348_1	708276	(G/A)
14513	CakSNP14513	*Kabuli*	Ca_Kabuli_Scaffold1348_1	708302	(G/T)
14514	CakSNP14514	*Kabuli*	Ca_Kabuli_Scaffold1348_1	708344	(G/A)
14515	CakSNP14515	*Kabuli*	Ca_Kabuli_Scaffold1348_1	732450	(A/C)

S.N.	SNP IDs	Cultivars	Chromosomes/scaffolds	Physical positions (bp)	SNPs
14516	CakSNP14516	*Kabuli*	Ca_Kabuli_Scaffold1348_1	732454	(T/G)
14517	CakSNP14517	*Kabuli*	Ca_Kabuli_Scaffold1348_1	732460	(T/A)
14518	CakSNP14518	*Kabuli*	Ca_Kabuli_Scaffold1348_1	732496	(G/T)
14519	CakSNP14519	*Kabuli*	Ca_Kabuli_Scaffold1348_1	735782	(C/T)
14520	CakSNP14520	*Kabuli*	Ca_Kabuli_Scaffold1348_1	735812	(G/A)
14521	CakSNP14521	*Kabuli*	Ca_Kabuli_Scaffold1348_1	750170	(C/A)
14522	CakSNP14522	*Kabuli*	Ca_Kabuli_Scaffold1348_1	750162	(T/C)
14523	CakSNP14523	*Kabuli*	Ca_Kabuli_Scaffold1348_1	750213	(C/A)
14524	CakSNP14524	*Kabuli*	Ca_Kabuli_Scaffold1348_1	771029	(T/A)
14525	CakSNP14525	*Kabuli*	Ca_Kabuli_Scaffold1348_1	871724	(G/A)
14526	CakSNP14526	*Kabuli*	Ca_Kabuli_Scaffold1348_1	909909	(A/G)
14527	CakSNP14527	*Kabuli*	Ca_Kabuli_Scaffold1348_1	909967	(C/G)
14528	CakSNP14528	*Kabuli*	Ca_Kabuli_Scaffold1348_1	909965	(T/A)
14529	CakSNP14529	*Kabuli*	Ca_Kabuli_Scaffold1348_1	909959	(A/C)
14530	CakSNP14530	*Kabuli*	Ca_Kabuli_Scaffold1348_1	924164	(T/G)
14531	CakSNP14531	*Kabuli*	Ca_Kabuli_Scaffold1348_1	934720	(C/A)
14532	CakSNP14532	*Kabuli*	Ca_Kabuli_Scaffold1348_1	934791	(C/T)
14533	CakSNP14533	*Kabuli*	Ca_Kabuli_Scaffold1348_1	950995	(A/G)
14534	CakSNP14534	*Kabuli*	Ca_Kabuli_Scaffold1348_1	959608	(A/C)
14535	CakSNP14535	*Kabuli*	Ca_Kabuli_Scaffold1348_1	959828	(G/A)
14536	CakSNP14536	*Kabuli*	Ca_Kabuli_Scaffold1348_1	959780	(G/A)
14537	CakSNP14537	*Kabuli*	Ca_Kabuli_Scaffold1348_1	970927	(A/G)
14538	CakSNP14538	*Kabuli*	Ca_Kabuli_Scaffold1348_1	981964	(T/C)
14539	CakSNP14539	*Kabuli*	Ca_Kabuli_Scaffold1348_1	983101	(T/G)
14540	CakSNP14540	*Kabuli*	Ca_Kabuli_Scaffold1348_1	1020258	(C/T)
14541	CakSNP14541	*Kabuli*	Ca_Kabuli_Scaffold1348_1	1023898	(G/A)
14542	CakSNP14542	*Kabuli*	Ca_Kabuli_Scaffold1348_1	1023923	(G/A)
14543	CakSNP14543	*Kabuli*	Ca_Kabuli_Scaffold1348_1	1023954	(G/C)
14544	CakSNP14544	*Kabuli*	Ca_Kabuli_Scaffold1348_1	1066818	(A/C)
14545	CakSNP14545	*Kabuli*	Ca_Kabuli_Scaffold1348_1	1066783	(C/T)
14546	CakSNP14546	*Kabuli*	Ca_Kabuli_Scaffold1348_1	1066776	(T/C)
14547	CakSNP14547	*Kabuli*	Ca_Kabuli_Scaffold1348_1	1072750	(C/T)
14548	CakSNP14548	*Kabuli*	Ca_Kabuli_Scaffold1348_1	1145082	(A/C)
14549	CakSNP14549	*Kabuli*	Ca_Kabuli_Scaffold1348_1	1145124	(T/C)
14550	CakSNP14550	*Kabuli*	Ca_Kabuli_Scaffold1348_1	1207442	(G/A)
14551	CakSNP14551	*Kabuli*	Ca_Kabuli_Scaffold1348_1	1236956	(A/G)
14552	CakSNP14552	*Kabuli*	Ca_Kabuli_Scaffold1348_1	1263560	(T/G)
14553	CakSNP14553	*Kabuli*	Ca_Kabuli_Scaffold1348_1	1265627	(T/C)

S.N.	SNP IDs	Cultivars	Chromosomes/scaffolds	Physical positions (bp)	SNPs
14554	CakSNP14554	*Kabuli*	Ca_Kabuli_Scaffold1348_1	1265635	(T/G)
14555	CakSNP14555	*Kabuli*	Ca_Kabuli_Scaffold1348_1	1273341	(G/A)
14556	CakSNP14556	*Kabuli*	Ca_Kabuli_Scaffold1348_1	1273388	(G/A)
14557	CakSNP14557	*Kabuli*	Ca_Kabuli_Scaffold1348_1	1288448	(A/G)
14558	CakSNP14558	*Kabuli*	Ca_Kabuli_Scaffold1348_1	1294675	(A/T)
14559	CakSNP14559	*Kabuli*	Ca_Kabuli_Scaffold1348_1	1296453	(G/A)
14560	CakSNP14560	*Kabuli*	Ca_Kabuli_Scaffold1348_1	1330256	(A/G)
14561	CakSNP14561	*Kabuli*	Ca_Kabuli_Scaffold1348_1	1330288	(A/G)
14562	CakSNP14562	*Kabuli*	Ca_Kabuli_Scaffold1348_1	1330400	(A/G)
14563	CakSNP14563	*Kabuli*	Ca_Kabuli_Scaffold1348_1	1346914	(G/T)
14564	CakSNP14564	*Kabuli*	Ca_Kabuli_Scaffold1348_1	1346943	(G/A)
14565	CakSNP14565	*Kabuli*	Ca_Kabuli_Scaffold1348_1	1372535	(G/A)
14566	CakSNP14566	*Kabuli*	Ca_Kabuli_Scaffold1348_1	1403452	(G/A)
14567	CakSNP14567	*Kabuli*	Ca_Kabuli_Scaffold1348_1	1403480	(A/G)
14568	CakSNP14568	*Kabuli*	Ca_Kabuli_Scaffold1351	172111	(G/A)
14569	CakSNP14569	*Kabuli*	Ca_Kabuli_Scaffold1351	172110	(T/C)
14570	CakSNP14570	*Kabuli*	Ca_Kabuli_Scaffold1351	177159	(G/A)
14571	CakSNP14571	*Kabuli*	Ca_Kabuli_Scaffold1351	177172	(G/A)
14572	CakSNP14572	*Kabuli*	Ca_Kabuli_Scaffold1351	177178	(T/C)
14573	CakSNP14573	*Kabuli*	Ca_Kabuli_Scaffold1351	177181	(A/G)
14574	CakSNP14574	*Kabuli*	Ca_Kabuli_Scaffold1351	177194	(G/A)
14575	CakSNP14575	*Kabuli*	Ca_Kabuli_Scaffold1351	177202	(G/A)
14576	CakSNP14576	*Kabuli*	Ca_Kabuli_Scaffold1351	177171	(T/G)
14577	CakSNP14577	*Kabuli*	Ca_Kabuli_Scaffold1351	177193	(T/C)
14578	CakSNP14578	*Kabuli*	Ca_Kabuli_Scaffold1351	177256	(T/G)
14579	CakSNP14579	*Kabuli*	Ca_Kabuli_Scaffold1351	177248	(G/T)
14580	CakSNP14580	*Kabuli*	Ca_Kabuli_Scaffold1351	177247	(G/A)
14581	CakSNP14581	*Kabuli*	Ca_Kabuli_Scaffold1351	177242	(G/A)
14582	CakSNP14582	*Kabuli*	Ca_Kabuli_Scaffold1351	177241	(T/G)
14583	CakSNP14583	*Kabuli*	Ca_Kabuli_Scaffold1351	177240	(C/A)
14584	CakSNP14584	*Kabuli*	Ca_Kabuli_Scaffold1351	177235	(G/T)
14585	CakSNP14585	*Kabuli*	Ca_Kabuli_Scaffold1351	177234	(A/C)
14586	CakSNP14586	*Kabuli*	Ca_Kabuli_Scaffold1351	177210	(T/A)
14587	CakSNP14587	*Kabuli*	Ca_Kabuli_Scaffold1351	177200	(T/G)
14588	CakSNP14588	*Kabuli*	Ca_Kabuli_Scaffold1351	177191	(C/T)
14589	CakSNP14589	*Kabuli*	Ca_Kabuli_Scaffold1351	177188	(G/T)
14590	CakSNP14590	*Kabuli*	Ca_Kabuli_Scaffold1351	204656	(C/A)
14591	CakSNP14591	*Kabuli*	Ca_Kabuli_Scaffold1351	335227	(T/C)

S.N.	SNP IDs	Cultivars	Chromosomes/scaffolds	Physical positions (bp)	SNPs
14592	CakSNP14592	*Kabuli*	Ca_Kabuli_Scaffold1351	335239	(G/T)
14593	CakSNP14593	*Kabuli*	Ca_Kabuli_Scaffold1351	367576	(A/G)
14594	CakSNP14594	*Kabuli*	Ca_Kabuli_Scaffold1351	367578	(G/A)
14595	CakSNP14595	*Kabuli*	Ca_Kabuli_Scaffold1351	367745	(G/A)
14596	CakSNP14596	*Kabuli*	Ca_Kabuli_Scaffold1369	8015	(T/A)
14597	CakSNP14597	*Kabuli*	Ca_Kabuli_Scaffold1369	8115	(T/G)
14598	CakSNP14598	*Kabuli*	Ca_Kabuli_Scaffold1369	8154	(T/G)
14599	CakSNP14599	*Kabuli*	Ca_Kabuli_Scaffold1369	8221	(T/G)
14600	CakSNP14600	*Kabuli*	Ca_Kabuli_Scaffold1369	8327	(G/A)
14601	CakSNP14601	*Kabuli*	Ca_Kabuli_Scaffold1369	31000	(A/G)
14602	CakSNP14602	*Kabuli*	Ca_Kabuli_Scaffold1390	98354	(A/G)
14603	CakSNP14603	*Kabuli*	Ca_Kabuli_Scaffold1401	89077	(T/C)
14604	CakSNP14604	*Kabuli*	Ca_Kabuli_Scaffold1401	89074	(T/C)
14605	CakSNP14605	*Kabuli*	Ca_Kabuli_Scaffold1417	4863	(A/G)
14606	CakSNP14606	*Kabuli*	Ca_Kabuli_Scaffold1417	4906	(G/A)
14607	CakSNP14607	*Kabuli*	Ca_Kabuli_Scaffold1417	4917	(G/T)
14608	CakSNP14608	*Kabuli*	Ca_Kabuli_Scaffold1417	76627	(A/C)
14609	CakSNP14609	*Kabuli*	Ca_Kabuli_Scaffold1417	230210	(T/G)
14610	CakSNP14610	*Kabuli*	Ca_Kabuli_Scaffold1419	24845	(C/T)
14611	CakSNP14611	*Kabuli*	Ca_Kabuli_Scaffold143	18492	(A/G)
14612	CakSNP14612	*Kabuli*	Ca_Kabuli_Scaffold1439	242972	(A/G)
14613	CakSNP14613	*Kabuli*	Ca_Kabuli_Scaffold1448	47306	(A/G)
14614	CakSNP14614	*Kabuli*	Ca_Kabuli_Scaffold1448	74436	(C/T)
14615	CakSNP14615	*Kabuli*	Ca_Kabuli_Scaffold1448	74434	(C/T)
14616	CakSNP14616	*Kabuli*	Ca_Kabuli_Scaffold1448	74529	(C/T)
14617	CakSNP14617	*Kabuli*	Ca_Kabuli_Scaffold1448	76809	(G/A)
14618	CakSNP14618	*Kabuli*	Ca_Kabuli_Scaffold1449	55474	(T/G)
14619	CakSNP14619	*Kabuli*	Ca_Kabuli_Scaffold1449	55529	(G/A)
14620	CakSNP14620	*Kabuli*	Ca_Kabuli_Scaffold1449	55532	(G/A)
14621	CakSNP14621	*Kabuli*	Ca_Kabuli_Scaffold1452	3501	(C/A)
14622	CakSNP14622	*Kabuli*	Ca_Kabuli_Scaffold1452	3496	(A/T)
14623	CakSNP14623	*Kabuli*	Ca_Kabuli_Scaffold1452	3495	(C/A)
14624	CakSNP14624	*Kabuli*	Ca_Kabuli_Scaffold1452	3737	(C/A)
14625	CakSNP14625	*Kabuli*	Ca_Kabuli_Scaffold1462	1556	(T/G)
14626	CakSNP14626	*Kabuli*	Ca_Kabuli_Scaffold1466	90846	(C/A)
14627	CakSNP14627	*Kabuli*	Ca_Kabuli_Scaffold1466	165472	(C/T)
14628	CakSNP14628	*Kabuli*	Ca_Kabuli_Scaffold1466	322287	(G/A)
14629	CakSNP14629	*Kabuli*	Ca_Kabuli_Scaffold1466	322315	(G/T)

S.N.	SNP IDs	Cultivars	Chromosomes/scaffolds	Physical positions (bp)	SNPs
14630	CakSNP14630	*Kabuli*	Ca_Kabuli_Scaffold1466	322450	(G/T)
14631	CakSNP14631	*Kabuli*	Ca_Kabuli_Scaffold1466	322448	(T/G)
14632	CakSNP14632	*Kabuli*	Ca_Kabuli_Scaffold1466	322421	(C/T)
14633	CakSNP14633	*Kabuli*	Ca_Kabuli_Scaffold1466	322415	(G/A)
14634	CakSNP14634	*Kabuli*	Ca_Kabuli_Scaffold1466	322414	(G/A)
14635	CakSNP14635	*Kabuli*	Ca_Kabuli_Scaffold1466	322400	(C/T)
14636	CakSNP14636	*Kabuli*	Ca_Kabuli_Scaffold1466	322390	(G/A)
14637	CakSNP14637	*Kabuli*	Ca_Kabuli_Scaffold1466	322383	(C/T)
14638	CakSNP14638	*Kabuli*	Ca_Kabuli_Scaffold1467	205779	(A/G)
14639	CakSNP14639	*Kabuli*	Ca_Kabuli_Scaffold1467	205803	(C/T)
14640	CakSNP14640	*Kabuli*	Ca_Kabuli_Scaffold1467	205833	(G/A)
14641	CakSNP14641	*Kabuli*	Ca_Kabuli_Scaffold1467	205840	(G/A)
14642	CakSNP14642	*Kabuli*	Ca_Kabuli_Scaffold1467	205845	(A/G)
14643	CakSNP14643	*Kabuli*	Ca_Kabuli_Scaffold1467	206121	(G/A)
14644	CakSNP14644	*Kabuli*	Ca_Kabuli_Scaffold1467	206118	(C/T)
14645	CakSNP14645	*Kabuli*	Ca_Kabuli_Scaffold1467	206100	(G/A)
14646	CakSNP14646	*Kabuli*	Ca_Kabuli_Scaffold1467	206094	(T/C)
14647	CakSNP14647	*Kabuli*	Ca_Kabuli_Scaffold1467	206085	(T/C)
14648	CakSNP14648	*Kabuli*	Ca_Kabuli_Scaffold1467	206068	(A/G)
14649	CakSNP14649	*Kabuli*	Ca_Kabuli_Scaffold1467	206067	(T/G)
14650	CakSNP14650	*Kabuli*	Ca_Kabuli_Scaffold1475	4820	(G/A)
14651	CakSNP14651	*Kabuli*	Ca_Kabuli_Scaffold1475	4796	(C/T)
14652	CakSNP14652	*Kabuli*	Ca_Kabuli_Scaffold1475	4794	(C/A)
14653	CakSNP14653	*Kabuli*	Ca_Kabuli_Scaffold1475	4755	(T/A)
14654	CakSNP14654	*Kabuli*	Ca_Kabuli_Scaffold1475	4754	(C/T)
14655	CakSNP14655	*Kabuli*	Ca_Kabuli_Scaffold1475	4749	(C/T)
14656	CakSNP14656	*Kabuli*	Ca_Kabuli_Scaffold1475	4873	(G/T)
14657	CakSNP14657	*Kabuli*	Ca_Kabuli_Scaffold1475	4845	(A/T)
14658	CakSNP14658	*Kabuli*	Ca_Kabuli_Scaffold1475	4833	(G/A)
14659	CakSNP14659	*Kabuli*	Ca_Kabuli_Scaffold1475	4818	(C/T)
14660	CakSNP14660	*Kabuli*	Ca_Kabuli_Scaffold1483	56686	(A/T)
14661	CakSNP14661	*Kabuli*	Ca_Kabuli_Scaffold1483	56721	(G/C)
14662	CakSNP14662	*Kabuli*	Ca_Kabuli_Scaffold1493	81740	(C/T)
14663	CakSNP14663	*Kabuli*	Ca_Kabuli_Scaffold1493	81784	(C/A)
14664	CakSNP14664	*Kabuli*	Ca_Kabuli_Scaffold1493	81789	(G/A)
14665	CakSNP14665	*Kabuli*	Ca_Kabuli_Scaffold1496	289997	(G/T)
14666	CakSNP14666	*Kabuli*	Ca_Kabuli_Scaffold1496	290018	(C/A)
14667	CakSNP14667	*Kabuli*	Ca_Kabuli_Scaffold1498	3474	(A/C)

S.N.	SNP IDs	Cultivars	Chromosomes/scaffolds	Physical positions (bp)	SNPs
14668	CakSNP14668	*Kabuli*	Ca_Kabuli_Scaffold1498	3657	(C/T)
14669	CakSNP14669	*Kabuli*	Ca_Kabuli_Scaffold1498	3641	(T/C)
14670	CakSNP14670	*Kabuli*	Ca_Kabuli_Scaffold1504	160623	(G/T)
14671	CakSNP14671	*Kabuli*	Ca_Kabuli_Scaffold1504	189700	(A/G)
14672	CakSNP14672	*Kabuli*	Ca_Kabuli_Scaffold1504	189699	(C/T)
14673	CakSNP14673	*Kabuli*	Ca_Kabuli_Scaffold1504	189681	(C/T)
14674	CakSNP14674	*Kabuli*	Ca_Kabuli_Scaffold1504	189664	(G/A)
14675	CakSNP14675	*Kabuli*	Ca_Kabuli_Scaffold1504	189663	(G/A)
14676	CakSNP14676	*Kabuli*	Ca_Kabuli_Scaffold1504	197789	(G/A)
14677	CakSNP14677	*Kabuli*	Ca_Kabuli_Scaffold1504	197790	(G/A)
14678	CakSNP14678	*Kabuli*	Ca_Kabuli_Scaffold1504	197819	(T/G)
14679	CakSNP14679	*Kabuli*	Ca_Kabuli_Scaffold1505	33297	(A/C)
14680	CakSNP14680	*Kabuli*	Ca_Kabuli_Scaffold151	267060	(T/G)
14681	CakSNP14681	*Kabuli*	Ca_Kabuli_Scaffold151	275743	(T/A)
14682	CakSNP14682	*Kabuli*	Ca_Kabuli_Scaffold1521	20734	(C/A)
14683	CakSNP14683	*Kabuli*	Ca_Kabuli_Scaffold1521	20779	(T/C)
14684	CakSNP14684	*Kabuli*	Ca_Kabuli_Scaffold1521	20851	(C/T)
14685	CakSNP14685	*Kabuli*	Ca_Kabuli_Scaffold1545	111476	(G/A)
14686	CakSNP14686	*Kabuli*	Ca_Kabuli_Scaffold1545	113148	(T/C)
14687	CakSNP14687	*Kabuli*	Ca_Kabuli_Scaffold1553	5078	(G/A)
14688	CakSNP14688	*Kabuli*	Ca_Kabuli_Scaffold1553	5115	(C/T)
14689	CakSNP14689	*Kabuli*	Ca_Kabuli_Scaffold1553	5125	(A/G)
14690	CakSNP14690	*Kabuli*	Ca_Kabuli_Scaffold157	100228	(A/G)
14691	CakSNP14691	*Kabuli*	Ca_Kabuli_Scaffold157	100529	(T/C)
14692	CakSNP14692	*Kabuli*	Ca_Kabuli_Scaffold157	102779	(C/T)
14693	CakSNP14693	*Kabuli*	Ca_Kabuli_Scaffold157	106331	(G/A)
14694	CakSNP14694	*Kabuli*	Ca_Kabuli_Scaffold157	106349	(C/T)
14695	CakSNP14695	*Kabuli*	Ca_Kabuli_Scaffold157	133602	(A/G)
14696	CakSNP14696	*Kabuli*	Ca_Kabuli_Scaffold157	133583	(T/G)
14697	CakSNP14697	*Kabuli*	Ca_Kabuli_Scaffold157	141880	(A/G)
14698	CakSNP14698	*Kabuli*	Ca_Kabuli_Scaffold157	142444	(A/T)
14699	CakSNP14699	*Kabuli*	Ca_Kabuli_Scaffold157	142455	(T/C)
14700	CakSNP14700	*Kabuli*	Ca_Kabuli_Scaffold157	142470	(G/T)
14701	CakSNP14701	*Kabuli*	Ca_Kabuli_Scaffold157	151081	(G/A)
14702	CakSNP14702	*Kabuli*	Ca_Kabuli_Scaffold157	151199	(T/A)
14703	CakSNP14703	*Kabuli*	Ca_Kabuli_Scaffold157	151192	(T/C)
14704	CakSNP14704	*Kabuli*	Ca_Kabuli_Scaffold157	151177	(A/G)
14705	CakSNP14705	*Kabuli*	Ca_Kabuli_Scaffold157	151131	(T/A)

S.N.	SNP IDs	Cultivars	Chromosomes/scaffolds	Physical positions (bp)	SNPs
14706	CakSNP14706	*Kabuli*	Ca_Kabuli_Scaffold157	213380	(C/G)
14707	CakSNP14707	*Kabuli*	Ca_Kabuli_Scaffold157	213398	(T/G)
14708	CakSNP14708	*Kabuli*	Ca_Kabuli_Scaffold157	213404	(T/G)
14709	CakSNP14709	*Kabuli*	Ca_Kabuli_Scaffold157	213518	(T/C)
14710	CakSNP14710	*Kabuli*	Ca_Kabuli_Scaffold157	213487	(G/C)
14711	CakSNP14711	*Kabuli*	Ca_Kabuli_Scaffold157	213477	(G/A)
14712	CakSNP14712	*Kabuli*	Ca_Kabuli_Scaffold157	229917	(G/A)
14713	CakSNP14713	*Kabuli*	Ca_Kabuli_Scaffold157	229900	(G/A)
14714	CakSNP14714	*Kabuli*	Ca_Kabuli_Scaffold157	321386	(C/T)
14715	CakSNP14715	*Kabuli*	Ca_Kabuli_Scaffold157	326434	(A/G)
14716	CakSNP14716	*Kabuli*	Ca_Kabuli_Scaffold157	326467	(C/T)
14717	CakSNP14717	*Kabuli*	Ca_Kabuli_Scaffold157	326494	(A/G)
14718	CakSNP14718	*Kabuli*	Ca_Kabuli_Scaffold157	326497	(A/G)
14719	CakSNP14719	*Kabuli*	Ca_Kabuli_Scaffold157	352687	(C/T)
14720	CakSNP14720	*Kabuli*	Ca_Kabuli_Scaffold157	352712	(G/A)
14721	CakSNP14721	*Kabuli*	Ca_Kabuli_Scaffold157	352747	(T/C)
14722	CakSNP14722	*Kabuli*	Ca_Kabuli_Scaffold157	355222	(G/A)
14723	CakSNP14723	*Kabuli*	Ca_Kabuli_Scaffold157	469198	(A/C)
14724	CakSNP14724	*Kabuli*	Ca_Kabuli_Scaffold157	535057	(T/G)
14725	CakSNP14725	*Kabuli*	Ca_Kabuli_Scaffold1575	2230	(C/T)
14726	CakSNP14726	*Kabuli*	Ca_Kabuli_Scaffold1580	71861	(G/T)
14727	CakSNP14727	*Kabuli*	Ca_Kabuli_Scaffold1580	119410	(T/C)
14728	CakSNP14728	*Kabuli*	Ca_Kabuli_Scaffold1580	119678	(T/C)
14729	CakSNP14729	*Kabuli*	Ca_Kabuli_Scaffold1580	163148	(A/T)
14730	CakSNP14730	*Kabuli*	Ca_Kabuli_Scaffold1580	163194	(T/C)
14731	CakSNP14731	*Kabuli*	Ca_Kabuli_Scaffold1580	380649	(A/T)
14732	CakSNP14732	*Kabuli*	Ca_Kabuli_Scaffold1585	59010	(C/A)
14733	CakSNP14733	*Kabuli*	Ca_Kabuli_Scaffold1603	11828	(C/G)
14734	CakSNP14734	*Kabuli*	Ca_Kabuli_Scaffold1607	107222	(T/C)
14735	CakSNP14735	*Kabuli*	Ca_Kabuli_Scaffold1613	48917	(T/G)
14736	CakSNP14736	*Kabuli*	Ca_Kabuli_Scaffold1613	107664	(T/A)
14737	CakSNP14737	*Kabuli*	Ca_Kabuli_Scaffold1613	107750	(T/A)
14738	CakSNP14738	*Kabuli*	Ca_Kabuli_Scaffold1631	202418	(A/G)
14739	CakSNP14739	*Kabuli*	Ca_Kabuli_Scaffold1635	83227	(G/C)
14740	CakSNP14740	*Kabuli*	Ca_Kabuli_Scaffold1635	83345	(A/T)
14741	CakSNP14741	*Kabuli*	Ca_Kabuli_Scaffold1654	26432	(G/A)
14742	CakSNP14742	*Kabuli*	Ca_Kabuli_Scaffold1659	303945	(T/G)
14743	CakSNP14743	*Kabuli*	Ca_Kabuli_Scaffold1661	70434	(A/G)

S.N.	SNP IDs	Cultivars	Chromosomes/scaffolds	Physical positions (bp)	SNPs
14744	CakSNP14744	*Kabuli*	Ca_Kabuli_Scaffold1664	94989	(G/A)
14745	CakSNP14745	*Kabuli*	Ca_Kabuli_Scaffold1664	94984	(A/T)
14746	CakSNP14746	*Kabuli*	Ca_Kabuli_Scaffold1664	94930	(G/C)
14747	CakSNP14747	*Kabuli*	Ca_Kabuli_Scaffold167	11018	(T/A)
14748	CakSNP14748	*Kabuli*	Ca_Kabuli_Scaffold167	11009	(A/T)
14749	CakSNP14749	*Kabuli*	Ca_Kabuli_Scaffold167	137869	(C/T)
14750	CakSNP14750	*Kabuli*	Ca_Kabuli_Scaffold167	137848	(A/G)
14751	CakSNP14751	*Kabuli*	Ca_Kabuli_Scaffold167	239687	(G/A)
14752	CakSNP14752	*Kabuli*	Ca_Kabuli_Scaffold167	327829	(T/C)
14753	CakSNP14753	*Kabuli*	Ca_Kabuli_Scaffold1687	82935	(A/C)
14754	CakSNP14754	*Kabuli*	Ca_Kabuli_Scaffold1699	27556	(A/C)
14755	CakSNP14755	*Kabuli*	Ca_Kabuli_Scaffold1699	27607	(T/G)
14756	CakSNP14756	*Kabuli*	Ca_Kabuli_Scaffold1708	69664	(A/T)
14757	CakSNP14757	*Kabuli*	Ca_Kabuli_Scaffold171	96763	(T/C)
14758	CakSNP14758	*Kabuli*	Ca_Kabuli_Scaffold171	183718	(T/C)
14759	CakSNP14759	*Kabuli*	Ca_Kabuli_Scaffold171	183720	(C/T)
14760	CakSNP14760	*Kabuli*	Ca_Kabuli_Scaffold171	183721	(G/A)
14761	CakSNP14761	*Kabuli*	Ca_Kabuli_Scaffold171	183727	(C/T)
14762	CakSNP14762	*Kabuli*	Ca_Kabuli_Scaffold171	183759	(T/C)
14763	CakSNP14763	*Kabuli*	Ca_Kabuli_Scaffold171	186125	(C/A)
14764	CakSNP14764	*Kabuli*	Ca_Kabuli_Scaffold171	208050	(A/G)
14765	CakSNP14765	*Kabuli*	Ca_Kabuli_Scaffold171	209964	(G/T)
14766	CakSNP14766	*Kabuli*	Ca_Kabuli_Scaffold1718	302	(G/A)
14767	CakSNP14767	*Kabuli*	Ca_Kabuli_Scaffold1718	295	(G/A)
14768	CakSNP14768	*Kabuli*	Ca_Kabuli_Scaffold1718	289	(C/T)
14769	CakSNP14769	*Kabuli*	Ca_Kabuli_Scaffold1718	268	(C/G)
14770	CakSNP14770	*Kabuli*	Ca_Kabuli_Scaffold1718	3898	(G/C)
14771	CakSNP14771	*Kabuli*	Ca_Kabuli_Scaffold1718	21521	(A/G)
14772	CakSNP14772	*Kabuli*	Ca_Kabuli_Scaffold1718	40669	(T/C)
14773	CakSNP14773	*Kabuli*	Ca_Kabuli_Scaffold1722	65999	(C/A)
14774	CakSNP14774	*Kabuli*	Ca_Kabuli_Scaffold1722	135589	(C/T)
14775	CakSNP14775	*Kabuli*	Ca_Kabuli_Scaffold1728	33301	(A/C)
14776	CakSNP14776	*Kabuli*	Ca_Kabuli_Scaffold174	17761	(A/C)
14777	CakSNP14777	*Kabuli*	Ca_Kabuli_Scaffold174	32366	(T/C)
14778	CakSNP14778	*Kabuli*	Ca_Kabuli_Scaffold174	44014	(T/G)
14779	CakSNP14779	*Kabuli*	Ca_Kabuli_Scaffold174	61454	(T/C)
14780	CakSNP14780	*Kabuli*	Ca_Kabuli_Scaffold174	263899	(G/A)
14781	CakSNP14781	*Kabuli*	Ca_Kabuli_Scaffold174	335077	(C/T)

S.N.	SNP IDs	Cultivars	Chromosomes/scaffolds	Physical positions (bp)	SNPs
14782	CakSNP14782	*Kabuli*	Ca_Kabuli_Scaffold174	341322	(C/T)
14783	CakSNP14783	*Kabuli*	Ca_Kabuli_Scaffold174	457627	(G/A)
14784	CakSNP14784	*Kabuli*	Ca_Kabuli_Scaffold1750	15510	(C/T)
14785	CakSNP14785	*Kabuli*	Ca_Kabuli_Scaffold1750	137106	(A/C)
14786	CakSNP14786	*Kabuli*	Ca_Kabuli_Scaffold1750	204638	(G/A)
14787	CakSNP14787	*Kabuli*	Ca_Kabuli_Scaffold1751	189367	(T/G)
14788	CakSNP14788	*Kabuli*	Ca_Kabuli_Scaffold1751	192417	(C/A)
14789	CakSNP14789	*Kabuli*	Ca_Kabuli_Scaffold1751	192713	(G/T)
14790	CakSNP14790	*Kabuli*	Ca_Kabuli_Scaffold1751	193026	(T/A)
14791	CakSNP14791	*Kabuli*	Ca_Kabuli_Scaffold1751	193013	(T/G)
14792	CakSNP14792	*Kabuli*	Ca_Kabuli_Scaffold1751	266583	(G/A)
14793	CakSNP14793	*Kabuli*	Ca_Kabuli_Scaffold1751	496626	(G/A)
14794	CakSNP14794	*Kabuli*	Ca_Kabuli_Scaffold1751	528896	(A/C)
14795	CakSNP14795	*Kabuli*	Ca_Kabuli_Scaffold1751	529005	(C/T)
14796	CakSNP14796	*Kabuli*	Ca_Kabuli_Scaffold1751	528979	(T/C)
14797	CakSNP14797	*Kabuli*	Ca_Kabuli_Scaffold1751	528973	(C/G)
14798	CakSNP14798	*Kabuli*	Ca_Kabuli_Scaffold1751	625364	(G/A)
14799	CakSNP14799	*Kabuli*	Ca_Kabuli_Scaffold1751	625362	(C/T)
14800	CakSNP14800	*Kabuli*	Ca_Kabuli_Scaffold1751	625355	(C/T)
14801	CakSNP14801	*Kabuli*	Ca_Kabuli_Scaffold1751	625342	(C/T)
14802	CakSNP14802	*Kabuli*	Ca_Kabuli_Scaffold1751	625335	(G/A)
14803	CakSNP14803	*Kabuli*	Ca_Kabuli_Scaffold1751	625395	(A/G)
14804	CakSNP14804	*Kabuli*	Ca_Kabuli_Scaffold1751	625394	(C/T)
14805	CakSNP14805	*Kabuli*	Ca_Kabuli_Scaffold1751	625389	(A/C)
14806	CakSNP14806	*Kabuli*	Ca_Kabuli_Scaffold1751	625378	(G/A)
14807	CakSNP14807	*Kabuli*	Ca_Kabuli_Scaffold1751	625375	(G/A)
14808	CakSNP14808	*Kabuli*	Ca_Kabuli_Scaffold1751	625366	(T/C)
14809	CakSNP14809	*Kabuli*	Ca_Kabuli_Scaffold1751	625365	(T/C)
14810	CakSNP14810	*Kabuli*	Ca_Kabuli_Scaffold1751	625332	(A/C)
14811	CakSNP14811	*Kabuli*	Ca_Kabuli_Scaffold1751	625396	(G/A)
14812	CakSNP14812	*Kabuli*	Ca_Kabuli_Scaffold1776	740	(C/T)
14813	CakSNP14813	*Kabuli*	Ca_Kabuli_Scaffold1777	164715	(G/A)
14814	CakSNP14814	*Kabuli*	Ca_Kabuli_Scaffold1777	164722	(C/G)
14815	CakSNP14815	*Kabuli*	Ca_Kabuli_Scaffold1777	164747	(G/A)
14816	CakSNP14816	*Kabuli*	Ca_Kabuli_Scaffold1777	164791	(T/G)
14817	CakSNP14817	*Kabuli*	Ca_Kabuli_Scaffold1777	164758	(C/T)
14818	CakSNP14818	*Kabuli*	Ca_Kabuli_Scaffold1777	164753	(A/G)
14819	CakSNP14819	*Kabuli*	Ca_Kabuli_Scaffold1777	164730	(T/G)

**S.N.**	**SNP IDs**	**Cultivars**	**Chromosomes/scaffolds**	**Physical positions (bp)**	**SNPs**
14820	CakSNP14820	*Kabuli*	Ca_Kabuli_Scaffold1777	169037	(G/T)
14821	CakSNP14821	*Kabuli*	Ca_Kabuli_Scaffold1777	343173	(C/T)
14822	CakSNP14822	*Kabuli*	Ca_Kabuli_Scaffold1777	343185	(G/A)
14823	CakSNP14823	*Kabuli*	Ca_Kabuli_Scaffold1777	448166	(A/G)
14824	CakSNP14824	*Kabuli*	Ca_Kabuli_Scaffold1777	448176	(C/T)
14825	CakSNP14825	*Kabuli*	Ca_Kabuli_Scaffold1777	448187	(G/T)
14826	CakSNP14826	*Kabuli*	Ca_Kabuli_Scaffold1777	448216	(C/T)
14827	CakSNP14827	*Kabuli*	Ca_Kabuli_Scaffold1779	957	(G/A)
14828	CakSNP14828	*Kabuli*	Ca_Kabuli_Scaffold1779	896	(A/G)
14829	CakSNP14829	*Kabuli*	Ca_Kabuli_Scaffold1779	890	(G/A)
14830	CakSNP14830	*Kabuli*	Ca_Kabuli_Scaffold1802	17068	(A/G)
14831	CakSNP14831	*Kabuli*	Ca_Kabuli_Scaffold1802	17033	(A/G)
14832	CakSNP14832	*Kabuli*	Ca_Kabuli_Scaffold1802	17111	(C/T)
14833	CakSNP14833	*Kabuli*	Ca_Kabuli_Scaffold1802	17117	(T/C)
14834	CakSNP14834	*Kabuli*	Ca_Kabuli_Scaffold1802	17120	(C/T)
14835	CakSNP14835	*Kabuli*	Ca_Kabuli_Scaffold1802	17123	(G/T)
14836	CakSNP14836	*Kabuli*	Ca_Kabuli_Scaffold1802	17132	(C/T)
14837	CakSNP14837	*Kabuli*	Ca_Kabuli_Scaffold1802	17150	(T/C)
14838	CakSNP14838	*Kabuli*	Ca_Kabuli_Scaffold1802	17151	(T/C)
14839	CakSNP14839	*Kabuli*	Ca_Kabuli_Scaffold1802	17168	(T/C)
14840	CakSNP14840	*Kabuli*	Ca_Kabuli_Scaffold1802	17177	(T/A)
14841	CakSNP14841	*Kabuli*	Ca_Kabuli_Scaffold1835	9398	(C/A)
14842	CakSNP14842	*Kabuli*	Ca_Kabuli_Scaffold1835	10541	(C/G)
14843	CakSNP14843	*Kabuli*	Ca_Kabuli_Scaffold1835	10563	(T/C)
14844	CakSNP14844	*Kabuli*	Ca_Kabuli_Scaffold1844	41899	(C/A)
14845	CakSNP14845	*Kabuli*	Ca_Kabuli_Scaffold1845	16292	(C/G)
14846	CakSNP14846	*Kabuli*	Ca_Kabuli_Scaffold1845	16317	(C/G)
14847	CakSNP14847	*Kabuli*	Ca_Kabuli_Scaffold1845	40886	(T/A)
14848	CakSNP14848	*Kabuli*	Ca_Kabuli_Scaffold1845	40872	(T/G)
14849	CakSNP14849	*Kabuli*	Ca_Kabuli_Scaffold1845	40871	(T/A)
14850	CakSNP14850	*Kabuli*	Ca_Kabuli_Scaffold1845	40842	(T/G)
14851	CakSNP14851	*Kabuli*	Ca_Kabuli_Scaffold1845	40821	(T/G)
14852	CakSNP14852	*Kabuli*	Ca_Kabuli_Scaffold1848	583	(A/G)
14853	CakSNP14853	*Kabuli*	Ca_Kabuli_Scaffold1848	565	(T/C)
14854	CakSNP14854	*Kabuli*	Ca_Kabuli_Scaffold1848	562	(G/C)
14855	CakSNP14855	*Kabuli*	Ca_Kabuli_Scaffold1848	532	(G/C)
14856	CakSNP14856	*Kabuli*	Ca_Kabuli_Scaffold186	11965	(A/G)
14857	CakSNP14857	*Kabuli*	Ca_Kabuli_Scaffold186	279942	(A/G)

S.N.	SNP IDs	Cultivars	Chromosomes/scaffolds	Physical positions (bp)	SNPs
14858	CakSNP14858	*Kabuli*	Ca_Kabuli_Scaffold186	280062	(T/C)
14859	CakSNP14859	*Kabuli*	Ca_Kabuli_Scaffold186	280047	(A/G)
14860	CakSNP14860	*Kabuli*	Ca_Kabuli_Scaffold186	280023	(C/T)
14861	CakSNP14861	*Kabuli*	Ca_Kabuli_Scaffold186	280015	(T/C)
14862	CakSNP14862	*Kabuli*	Ca_Kabuli_Scaffold186	280009	(G/T)
14863	CakSNP14863	*Kabuli*	Ca_Kabuli_Scaffold1866	106318	(T/A)
14864	CakSNP14864	*Kabuli*	Ca_Kabuli_Scaffold1866	106330	(A/T)
14865	CakSNP14865	*Kabuli*	Ca_Kabuli_Scaffold1866	106371	(C/A)
14866	CakSNP14866	*Kabuli*	Ca_Kabuli_Scaffold1866	106374	(A/T)
14867	CakSNP14867	*Kabuli*	Ca_Kabuli_Scaffold1866	106402	(C/T)
14868	CakSNP14868	*Kabuli*	Ca_Kabuli_Scaffold1866	110550	(A/G)
14869	CakSNP14869	*Kabuli*	Ca_Kabuli_Scaffold1866	110570	(G/A)
14870	CakSNP14870	*Kabuli*	Ca_Kabuli_Scaffold1866	110578	(C/A)
14871	CakSNP14871	*Kabuli*	Ca_Kabuli_Scaffold1899	148	(T/G)
14872	CakSNP14872	*Kabuli*	Ca_Kabuli_Scaffold1899	187	(A/G)
14873	CakSNP14873	*Kabuli*	Ca_Kabuli_Scaffold1899	267	(T/A)
14874	CakSNP14874	*Kabuli*	Ca_Kabuli_Scaffold1899	200	(G/A)
14875	CakSNP14875	*Kabuli*	Ca_Kabuli_Scaffold1918	216231	(T/G)
14876	CakSNP14876	*Kabuli*	Ca_Kabuli_Scaffold1918	216213	(T/C)
14877	CakSNP14877	*Kabuli*	Ca_Kabuli_Scaffold1918	216180	(C/A)
14878	CakSNP14878	*Kabuli*	Ca_Kabuli_Scaffold1918	237787	(G/A)
14879	CakSNP14879	*Kabuli*	Ca_Kabuli_Scaffold1918	245929	(T/C)
14880	CakSNP14880	*Kabuli*	Ca_Kabuli_Scaffold1928	103092	(A/G)
14881	CakSNP14881	*Kabuli*	Ca_Kabuli_Scaffold1928	103114	(G/A)
14882	CakSNP14882	*Kabuli*	Ca_Kabuli_Scaffold193	38279	(C/A)
14883	CakSNP14883	*Kabuli*	Ca_Kabuli_Scaffold193	186538	(C/T)
14884	CakSNP14884	*Kabuli*	Ca_Kabuli_Scaffold193	186523	(T/C)
14885	CakSNP14885	*Kabuli*	Ca_Kabuli_Scaffold193	186515	(T/G)
14886	CakSNP14886	*Kabuli*	Ca_Kabuli_Scaffold193	186510	(C/A)
14887	CakSNP14887	*Kabuli*	Ca_Kabuli_Scaffold193	186584	(A/G)
14888	CakSNP14888	*Kabuli*	Ca_Kabuli_Scaffold193	186582	(C/A)
14889	CakSNP14889	*Kabuli*	Ca_Kabuli_Scaffold193	186587	(G/T)
14890	CakSNP14890	*Kabuli*	Ca_Kabuli_Scaffold193	186588	(C/G)
14891	CakSNP14891	*Kabuli*	Ca_Kabuli_Scaffold193	186595	(C/T)
14892	CakSNP14892	*Kabuli*	Ca_Kabuli_Scaffold193	186598	(A/G)
14893	CakSNP14893	*Kabuli*	Ca_Kabuli_Scaffold193	186611	(G/A)
14894	CakSNP14894	*Kabuli*	Ca_Kabuli_Scaffold193	186639	(C/T)
14895	CakSNP14895	*Kabuli*	Ca_Kabuli_Scaffold193	186651	(T/C)

**S.N.**	**SNP IDs**	**Cultivars**	**Chromosomes/scaffolds**	**Physical positions (bp)**	**SNPs**
14896	CakSNP14896	*Kabuli*	Ca_Kabuli_Scaffold193	186657	(G/A)
14897	CakSNP14897	*Kabuli*	Ca_Kabuli_Scaffold193	186661	(T/C)
14898	CakSNP14898	*Kabuli*	Ca_Kabuli_Scaffold193	242763	(T/C)
14899	CakSNP14899	*Kabuli*	Ca_Kabuli_Scaffold193	242733	(G/T)
14900	CakSNP14900	*Kabuli*	Ca_Kabuli_Scaffold193	244735	(T/G)
14901	CakSNP14901	*Kabuli*	Ca_Kabuli_Scaffold1943	163784	(G/A)
14902	CakSNP14902	*Kabuli*	Ca_Kabuli_Scaffold1943	163780	(C/T)
14903	CakSNP14903	*Kabuli*	Ca_Kabuli_Scaffold1959	84160	(G/A)
14904	CakSNP14904	*Kabuli*	Ca_Kabuli_Scaffold1964	54613	(G/A)
14905	CakSNP14905	*Kabuli*	Ca_Kabuli_Scaffold1964	54604	(T/C)
14906	CakSNP14906	*Kabuli*	Ca_Kabuli_Scaffold1964	67416	(G/C)
14907	CakSNP14907	*Kabuli*	Ca_Kabuli_Scaffold198	631160	(G/A)
14908	CakSNP14908	*Kabuli*	Ca_Kabuli_Scaffold198	631174	(C/T)
14909	CakSNP14909	*Kabuli*	Ca_Kabuli_Scaffold198	631185	(G/A)
14910	CakSNP14910	*Kabuli*	Ca_Kabuli_Scaffold198	641266	(C/T)
14911	CakSNP14911	*Kabuli*	Ca_Kabuli_Scaffold198	641252	(A/G)
14912	CakSNP14912	*Kabuli*	Ca_Kabuli_Scaffold198	641243	(A/G)
14913	CakSNP14913	*Kabuli*	Ca_Kabuli_Scaffold1981	265110	(A/C)
14914	CakSNP14914	*Kabuli*	Ca_Kabuli_Scaffold1981	265126	(G/T)
14915	CakSNP14915	*Kabuli*	Ca_Kabuli_Scaffold1981	265216	(A/G)
14916	CakSNP14916	*Kabuli*	Ca_Kabuli_Scaffold1981	265191	(T/G)
14917	CakSNP14917	*Kabuli*	Ca_Kabuli_Scaffold1981	269879	(A/G)
14918	CakSNP14918	*Kabuli*	Ca_Kabuli_Scaffold1981	385683	(T/A)
14919	CakSNP14919	*Kabuli*	Ca_Kabuli_Scaffold1984	212011	(G/A)
14920	CakSNP14920	*Kabuli*	Ca_Kabuli_Scaffold1984	215969	(G/C)
14921	CakSNP14921	*Kabuli*	Ca_Kabuli_Scaffold1984	292507	(A/C)
14922	CakSNP14922	*Kabuli*	Ca_Kabuli_Scaffold1984	292511	(G/C)
14923	CakSNP14923	*Kabuli*	Ca_Kabuli_Scaffold1985	98611	(C/T)
14924	CakSNP14924	*Kabuli*	Ca_Kabuli_Scaffold1985	98659	(C/T)
14925	CakSNP14925	*Kabuli*	Ca_Kabuli_Scaffold1991	62009	(C/A)
14926	CakSNP14926	*Kabuli*	Ca_Kabuli_Scaffold1991	62121	(G/A)
14927	CakSNP14927	*Kabuli*	Ca_Kabuli_Scaffold1991	62085	(A/C)
14928	CakSNP14928	*Kabuli*	Ca_Kabuli_Scaffold1991	62115	(G/A)
14929	CakSNP14929	*Kabuli*	Ca_Kabuli_Scaffold1991	62131	(G/A)
14930	CakSNP14930	*Kabuli*	Ca_Kabuli_Scaffold1991	62154	(T/A)
14931	CakSNP14931	*Kabuli*	Ca_Kabuli_Scaffold1991	62147	(G/A)
14932	CakSNP14932	*Kabuli*	Ca_Kabuli_Scaffold1991	62422	(G/A)
14933	CakSNP14933	*Kabuli*	Ca_Kabuli_Scaffold2	137309	(T/G)

S.N.	SNP IDs	Cultivars	Chromosomes/scaffolds	Physical positions (bp)	SNPs
14934	CakSNP14934	*Kabuli*	Ca_Kabuli_Scaffold2	504258	(C/T)
14935	CakSNP14935	*Kabuli*	Ca_Kabuli_Scaffold2	504253	(C/T)
14936	CakSNP14936	*Kabuli*	Ca_Kabuli_Scaffold2	504248	(G/A)
14937	CakSNP14937	*Kabuli*	Ca_Kabuli_Scaffold202	16084	(T/C)
14938	CakSNP14938	*Kabuli*	Ca_Kabuli_Scaffold202	92532	(C/A)
14939	CakSNP14939	*Kabuli*	Ca_Kabuli_Scaffold202	96456	(T/C)
14940	CakSNP14940	*Kabuli*	Ca_Kabuli_Scaffold202	96444	(G/A)
14941	CakSNP14941	*Kabuli*	Ca_Kabuli_Scaffold202	96532	(G/T)
14942	CakSNP14942	*Kabuli*	Ca_Kabuli_Scaffold202	96589	(A/T)
14943	CakSNP14943	*Kabuli*	Ca_Kabuli_Scaffold202	96580	(T/G)
14944	CakSNP14944	*Kabuli*	Ca_Kabuli_Scaffold202	96576	(A/G)
14945	CakSNP14945	*Kabuli*	Ca_Kabuli_Scaffold202	99123	(C/T)
14946	CakSNP14946	*Kabuli*	Ca_Kabuli_Scaffold2027	100867	(G/C)
14947	CakSNP14947	*Kabuli*	Ca_Kabuli_Scaffold2027	152763	(C/A)
14948	CakSNP14948	*Kabuli*	Ca_Kabuli_Scaffold2030	13855	(G/A)
14949	CakSNP14949	*Kabuli*	Ca_Kabuli_Scaffold2030	13822	(C/T)
14950	CakSNP14950	*Kabuli*	Ca_Kabuli_Scaffold2030	13807	(G/A)
14951	CakSNP14951	*Kabuli*	Ca_Kabuli_Scaffold2030	13889	(C/T)
14952	CakSNP14952	*Kabuli*	Ca_Kabuli_Scaffold2030	13874	(G/T)
14953	CakSNP14953	*Kabuli*	Ca_Kabuli_Scaffold2033	115044	(C/T)
14954	CakSNP14954	*Kabuli*	Ca_Kabuli_Scaffold2033	118686	(C/T)
14955	CakSNP14955	*Kabuli*	Ca_Kabuli_Scaffold2033	118779	(G/T)
14956	CakSNP14956	*Kabuli*	Ca_Kabuli_Scaffold2033	118760	(T/G)
14957	CakSNP14957	*Kabuli*	Ca_Kabuli_Scaffold2033	119057	(T/G)
14958	CakSNP14958	*Kabuli*	Ca_Kabuli_Scaffold2033	119166	(T/C)
14959	CakSNP14959	*Kabuli*	Ca_Kabuli_Scaffold2033	119645	(G/A)
14960	CakSNP14960	*Kabuli*	Ca_Kabuli_Scaffold2033	119648	(G/A)
14961	CakSNP14961	*Kabuli*	Ca_Kabuli_Scaffold2033	119703	(C/T)
14962	CakSNP14962	*Kabuli*	Ca_Kabuli_Scaffold2033	119715	(G/T)
14963	CakSNP14963	*Kabuli*	Ca_Kabuli_Scaffold2033	119728	(G/A)
14964	CakSNP14964	*Kabuli*	Ca_Kabuli_Scaffold2039	116734	(A/G)
14965	CakSNP14965	*Kabuli*	Ca_Kabuli_Scaffold2040	139597	(A/G)
14966	CakSNP14966	*Kabuli*	Ca_Kabuli_Scaffold2040	139585	(A/C)
14967	CakSNP14967	*Kabuli*	Ca_Kabuli_Scaffold2040	139584	(G/A)
14968	CakSNP14968	*Kabuli*	Ca_Kabuli_Scaffold2040	139581	(G/A)
14969	CakSNP14969	*Kabuli*	Ca_Kabuli_Scaffold2040	139560	(G/T)
14970	CakSNP14970	*Kabuli*	Ca_Kabuli_Scaffold2040	139550	(G/A)
14971	CakSNP14971	*Kabuli*	Ca_Kabuli_Scaffold2040	139548	(C/T)

S.N.	SNP IDs	Cultivars	Chromosomes/scaffolds	Physical positions (bp)	SNPs
14972	CakSNP14972	*Kabuli*	Ca_Kabuli_Scaffold2040	139544	(C/A)
14973	CakSNP14973	*Kabuli*	Ca_Kabuli_Scaffold2040	139541	(T/C)
14974	CakSNP14974	*Kabuli*	Ca_Kabuli_Scaffold2040	139538	(G/A)
14975	CakSNP14975	*Kabuli*	Ca_Kabuli_Scaffold2040	139545	(G/A)
14976	CakSNP14976	*Kabuli*	Ca_Kabuli_Scaffold2040	139603	(C/T)
14977	CakSNP14977	*Kabuli*	Ca_Kabuli_Scaffold205	74448	(T/G)
14978	CakSNP14978	*Kabuli*	Ca_Kabuli_Scaffold205	74430	(A/G)
14979	CakSNP14979	*Kabuli*	Ca_Kabuli_Scaffold205	74397	(G/A)
14980	CakSNP14980	*Kabuli*	Ca_Kabuli_Scaffold205	74399	(C/T)
14981	CakSNP14981	*Kabuli*	Ca_Kabuli_Scaffold205	74424	(T/C)
14982	CakSNP14982	*Kabuli*	Ca_Kabuli_Scaffold205	74432	(G/A)
14983	CakSNP14983	*Kabuli*	Ca_Kabuli_Scaffold205	74433	(G/A)
14984	CakSNP14984	*Kabuli*	Ca_Kabuli_Scaffold206	58349	(C/A)
14985	CakSNP14985	*Kabuli*	Ca_Kabuli_Scaffold206	62985	(G/A)
14986	CakSNP14986	*Kabuli*	Ca_Kabuli_Scaffold208	344503	(G/T)
14987	CakSNP14987	*Kabuli*	Ca_Kabuli_Scaffold208	344515	(A/G)
14988	CakSNP14988	*Kabuli*	Ca_Kabuli_Scaffold208	344519	(A/T)
14989	CakSNP14989	*Kabuli*	Ca_Kabuli_Scaffold208	344533	(C/T)
14990	CakSNP14990	*Kabuli*	Ca_Kabuli_Scaffold208	344556	(T/G)
14991	CakSNP14991	*Kabuli*	Ca_Kabuli_Scaffold2097	54210	(A/C)
14992	CakSNP14992	*Kabuli*	Ca_Kabuli_Scaffold210	293799	(G/A)
14993	CakSNP14993	*Kabuli*	Ca_Kabuli_Scaffold210	355284	(G/T)
14994	CakSNP14994	*Kabuli*	Ca_Kabuli_Scaffold210	533483	(G/A)
14995	CakSNP14995	*Kabuli*	Ca_Kabuli_Scaffold210	533416	(G/A)
14996	CakSNP14996	*Kabuli*	Ca_Kabuli_Scaffold210	617431	(T/A)
14997	CakSNP14997	*Kabuli*	Ca_Kabuli_Scaffold2104	61757	(T/C)
14998	CakSNP14998	*Kabuli*	Ca_Kabuli_Scaffold2104	61745	(T/G)
14999	CakSNP14999	*Kabuli*	Ca_Kabuli_Scaffold2104	61734	(T/G)
15000	CakSNP15000	*Kabuli*	Ca_Kabuli_Scaffold2104	61723	(G/A)
15001	CakSNP15001	*Kabuli*	Ca_Kabuli_Scaffold2104	61710	(C/T)
15002	CakSNP15002	*Kabuli*	Ca_Kabuli_Scaffold2104	61705	(C/T)
15003	CakSNP15003	*Kabuli*	Ca_Kabuli_Scaffold2152	372404	(A/T)
15004	CakSNP15004	*Kabuli*	Ca_Kabuli_Scaffold2166	228653	(G/C)
15005	CakSNP15005	*Kabuli*	Ca_Kabuli_Scaffold2166	228710	(G/A)
15006	CakSNP15006	*Kabuli*	Ca_Kabuli_Scaffold2170	4821	(G/A)
15007	CakSNP15007	*Kabuli*	Ca_Kabuli_Scaffold2192	67485	(A/G)
15008	CakSNP15008	*Kabuli*	Ca_Kabuli_Scaffold2192	67589	(A/C)
15009	CakSNP15009	*Kabuli*	Ca_Kabuli_Scaffold221	53841	(T/G)

S.N.	SNP IDs	Cultivars	Chromosomes/scaffolds	Physical positions (bp)	SNPs
15010	CakSNP15010	*Kabuli*	Ca_Kabuli_Scaffold2248	5538	(A/T)
15011	CakSNP15011	*Kabuli*	Ca_Kabuli_Scaffold2248	5562	(C/T)
15012	CakSNP15012	*Kabuli*	Ca_Kabuli_Scaffold2248	5570	(T/C)
15013	CakSNP15013	*Kabuli*	Ca_Kabuli_Scaffold2248	5520	(C/A)
15014	CakSNP15014	*Kabuli*	Ca_Kabuli_Scaffold2249	4446	(A/C)
15015	CakSNP15015	*Kabuli*	Ca_Kabuli_Scaffold2269	58091	(A/T)
15016	CakSNP15016	*Kabuli*	Ca_Kabuli_Scaffold2269	123799	(A/C)
15017	CakSNP15017	*Kabuli*	Ca_Kabuli_Scaffold2269	126013	(A/C)
15018	CakSNP15018	*Kabuli*	Ca_Kabuli_Scaffold2301	70721	(A/G)
15019	CakSNP15019	*Kabuli*	Ca_Kabuli_Scaffold2330	27894	(A/C)
15020	CakSNP15020	*Kabuli*	Ca_Kabuli_Scaffold2330	64157	(T/C)
15021	CakSNP15021	*Kabuli*	Ca_Kabuli_Scaffold2330	65482	(T/G)
15022	CakSNP15022	*Kabuli*	Ca_Kabuli_Scaffold2330	65617	(G/A)
15023	CakSNP15023	*Kabuli*	Ca_Kabuli_Scaffold2330	65650	(C/T)
15024	CakSNP15024	*Kabuli*	Ca_Kabuli_Scaffold2330	83061	(C/T)
15025	CakSNP15025	*Kabuli*	Ca_Kabuli_Scaffold2330	83064	(A/G)
15026	CakSNP15026	*Kabuli*	Ca_Kabuli_Scaffold2330	83077	(G/C)
15027	CakSNP15027	*Kabuli*	Ca_Kabuli_Scaffold2330	83112	(A/G)
15028	CakSNP15028	*Kabuli*	Ca_Kabuli_Scaffold2330	83116	(G/T)
15029	CakSNP15029	*Kabuli*	Ca_Kabuli_Scaffold235	67258	(G/T)
15030	CakSNP15030	*Kabuli*	Ca_Kabuli_Scaffold2371	139149	(A/G)
15031	CakSNP15031	*Kabuli*	Ca_Kabuli_Scaffold2373	3818	(A/G)
15032	CakSNP15032	*Kabuli*	Ca_Kabuli_Scaffold2373	3815	(T/A)
15033	CakSNP15033	*Kabuli*	Ca_Kabuli_Scaffold2373	3811	(C/T)
15034	CakSNP15034	*Kabuli*	Ca_Kabuli_Scaffold2373	3793	(C/T)
15035	CakSNP15035	*Kabuli*	Ca_Kabuli_Scaffold2373	3923	(T/C)
15036	CakSNP15036	*Kabuli*	Ca_Kabuli_Scaffold2373	3871	(A/T)
15037	CakSNP15037	*Kabuli*	Ca_Kabuli_Scaffold2373	3862	(C/T)
15038	CakSNP15038	*Kabuli*	Ca_Kabuli_Scaffold2373	12916	(C/A)
15039	CakSNP15039	*Kabuli*	Ca_Kabuli_Scaffold2373	12969	(G/C)
15040	CakSNP15040	*Kabuli*	Ca_Kabuli_Scaffold2373	12978	(A/T)
15041	CakSNP15041	*Kabuli*	Ca_Kabuli_Scaffold2373	62495	(C/T)
15042	CakSNP15042	*Kabuli*	Ca_Kabuli_Scaffold2373	62494	(A/G)
15043	CakSNP15043	*Kabuli*	Ca_Kabuli_Scaffold2373	62473	(G/A)
15044	CakSNP15044	*Kabuli*	Ca_Kabuli_Scaffold2373	62434	(G/A)
15045	CakSNP15045	*Kabuli*	Ca_Kabuli_Scaffold2373	62433	(A/G)
15046	CakSNP15046	*Kabuli*	Ca_Kabuli_Scaffold2373	127884	(A/C)
15047	CakSNP15047	*Kabuli*	Ca_Kabuli_Scaffold2373	127851	(C/T)

S.N.	SNP IDs	Cultivars	Chromosomes/scaffolds	Physical positions (bp)	SNPs
15048	CakSNP15048	*Kabuli*	Ca_Kabuli_Scaffold2373	127905	(C/T)
15049	CakSNP15049	*Kabuli*	Ca_Kabuli_Scaffold2373	127870	(T/G)
15050	CakSNP15050	*Kabuli*	Ca_Kabuli_Scaffold2373	127855	(A/C)
15051	CakSNP15051	*Kabuli*	Ca_Kabuli_Scaffold2392	35402	(A/C)
15052	CakSNP15052	*Kabuli*	Ca_Kabuli_Scaffold2392	198120	(G/T)
15053	CakSNP15053	*Kabuli*	Ca_Kabuli_Scaffold2392	314304	(T/C)
15054	CakSNP15054	*Kabuli*	Ca_Kabuli_Scaffold2392	314322	(T/C)
15055	CakSNP15055	*Kabuli*	Ca_Kabuli_Scaffold2392	314323	(G/A)
15056	CakSNP15056	*Kabuli*	Ca_Kabuli_Scaffold2392	314343	(G/A)
15057	CakSNP15057	*Kabuli*	Ca_Kabuli_Scaffold2392	314344	(T/C)
15058	CakSNP15058	*Kabuli*	Ca_Kabuli_Scaffold2392	314349	(C/G)
15059	CakSNP15059	*Kabuli*	Ca_Kabuli_Scaffold2392	314353	(G/A)
15060	CakSNP15060	*Kabuli*	Ca_Kabuli_Scaffold2392	314345	(G/A)
15061	CakSNP15061	*Kabuli*	Ca_Kabuli_Scaffold2392	314401	(A/C)
15062	CakSNP15062	*Kabuli*	Ca_Kabuli_Scaffold240	6231	(T/A)
15063	CakSNP15063	*Kabuli*	Ca_Kabuli_Scaffold240	6193	(G/A)
15064	CakSNP15064	*Kabuli*	Ca_Kabuli_Scaffold240	6152	(C/T)
15065	CakSNP15065	*Kabuli*	Ca_Kabuli_Scaffold240	119157	(G/A)
15066	CakSNP15066	*Kabuli*	Ca_Kabuli_Scaffold240	119301	(T/C)
15067	CakSNP15067	*Kabuli*	Ca_Kabuli_Scaffold240	119297	(C/A)
15068	CakSNP15068	*Kabuli*	Ca_Kabuli_Scaffold240	119278	(T/C)
15069	CakSNP15069	*Kabuli*	Ca_Kabuli_Scaffold240	119269	(C/T)
15070	CakSNP15070	*Kabuli*	Ca_Kabuli_Scaffold240	119267	(G/C)
15071	CakSNP15071	*Kabuli*	Ca_Kabuli_Scaffold240	119265	(C/G)
15072	CakSNP15072	*Kabuli*	Ca_Kabuli_Scaffold2415	117840	(A/G)
15073	CakSNP15073	*Kabuli*	Ca_Kabuli_Scaffold2415	117871	(A/C)
15074	CakSNP15074	*Kabuli*	Ca_Kabuli_Scaffold2415	117882	(A/G)
15075	CakSNP15075	*Kabuli*	Ca_Kabuli_Scaffold2415	117970	(G/A)
15076	CakSNP15076	*Kabuli*	Ca_Kabuli_Scaffold242	293872	(A/G)
15077	CakSNP15077	*Kabuli*	Ca_Kabuli_Scaffold250	38270	(A/G)
15078	CakSNP15078	*Kabuli*	Ca_Kabuli_Scaffold2501	287	(C/T)
15079	CakSNP15079	*Kabuli*	Ca_Kabuli_Scaffold2501	282	(C/A)
15080	CakSNP15080	*Kabuli*	Ca_Kabuli_Scaffold2501	251	(T/G)
15081	CakSNP15081	*Kabuli*	Ca_Kabuli_Scaffold2501	242	(C/G)
15082	CakSNP15082	*Kabuli*	Ca_Kabuli_Scaffold2516	58947	(T/C)
15083	CakSNP15083	*Kabuli*	Ca_Kabuli_Scaffold2521	57762	(T/C)
15084	CakSNP15084	*Kabuli*	Ca_Kabuli_Scaffold2521	57725	(G/A)
15085	CakSNP15085	*Kabuli*	Ca_Kabuli_Scaffold2521	57709	(G/T)

S.N.	SNP IDs	Cultivars	Chromosomes/scaffolds	Physical positions (bp)	SNPs
15086	CakSNP15086	*Kabuli*	Ca_Kabuli_Scaffold2557	110378	(C/T)
15087	CakSNP15087	*Kabuli*	Ca_Kabuli_Scaffold2557	110414	(T/G)
15088	CakSNP15088	*Kabuli*	Ca_Kabuli_Scaffold2557	110437	(C/T)
15089	CakSNP15089	*Kabuli*	Ca_Kabuli_Scaffold2557	110441	(T/A)
15090	CakSNP15090	*Kabuli*	Ca_Kabuli_Scaffold2569	58152	(T/G)
15091	CakSNP15091	*Kabuli*	Ca_Kabuli_Scaffold2575	101222	(C/T)
15092	CakSNP15092	*Kabuli*	Ca_Kabuli_Scaffold2617	467848	(C/T)
15093	CakSNP15093	*Kabuli*	Ca_Kabuli_Scaffold263	85006	(G/A)
15094	CakSNP15094	*Kabuli*	Ca_Kabuli_Scaffold263	85003	(C/T)
15095	CakSNP15095	*Kabuli*	Ca_Kabuli_Scaffold263	84998	(G/A)
15096	CakSNP15096	*Kabuli*	Ca_Kabuli_Scaffold263	84976	(A/G)
15097	CakSNP15097	*Kabuli*	Ca_Kabuli_Scaffold263	84970	(T/C)
15098	CakSNP15098	*Kabuli*	Ca_Kabuli_Scaffold263	84952	(T/G)
15099	CakSNP15099	*Kabuli*	Ca_Kabuli_Scaffold2720	30699	(C/T)
15100	CakSNP15100	*Kabuli*	Ca_Kabuli_Scaffold2720	30701	(G/A)
15101	CakSNP15101	*Kabuli*	Ca_Kabuli_Scaffold2720	30760	(T/C)
15102	CakSNP15102	*Kabuli*	Ca_Kabuli_Scaffold2720	177550	(G/T)
15103	CakSNP15103	*Kabuli*	Ca_Kabuli_Scaffold2720	177578	(T/C)
15104	CakSNP15104	*Kabuli*	Ca_Kabuli_Scaffold2722	52416	(G/T)
15105	CakSNP15105	*Kabuli*	Ca_Kabuli_Scaffold2722	52397	(G/T)
15106	CakSNP15106	*Kabuli*	Ca_Kabuli_Scaffold2722	67309	(C/A)
15107	CakSNP15107	*Kabuli*	Ca_Kabuli_Scaffold2722	75609	(A/G)
15108	CakSNP15108	*Kabuli*	Ca_Kabuli_Scaffold2728	1371	(G/A)
15109	CakSNP15109	*Kabuli*	Ca_Kabuli_Scaffold273	202514	(T/G)
15110	CakSNP15110	*Kabuli*	Ca_Kabuli_Scaffold2738	1307	(G/A)
15111	CakSNP15111	*Kabuli*	Ca_Kabuli_Scaffold275	208095	(G/A)
15112	CakSNP15112	*Kabuli*	Ca_Kabuli_Scaffold275	208067	(C/A)
15113	CakSNP15113	*Kabuli*	Ca_Kabuli_Scaffold275	208786	(G/C)
15114	CakSNP15114	*Kabuli*	Ca_Kabuli_Scaffold275	230606	(A/G)
15115	CakSNP15115	*Kabuli*	Ca_Kabuli_Scaffold275	347422	(C/A)
15116	CakSNP15116	*Kabuli*	Ca_Kabuli_Scaffold275	347384	(G/A)
15117	CakSNP15117	*Kabuli*	Ca_Kabuli_Scaffold275	347361	(C/T)
15118	CakSNP15118	*Kabuli*	Ca_Kabuli_Scaffold275	479497	(C/T)
15119	CakSNP15119	*Kabuli*	Ca_Kabuli_Scaffold275	562610	(C/T)
15120	CakSNP15120	*Kabuli*	Ca_Kabuli_Scaffold2763	4688	(A/C)
15121	CakSNP15121	*Kabuli*	Ca_Kabuli_Scaffold2763	4706	(G/T)
15122	CakSNP15122	*Kabuli*	Ca_Kabuli_Scaffold2763	4719	(G/A)
15123	CakSNP15123	*Kabuli*	Ca_Kabuli_Scaffold2763	4732	(C/G)

S.N.	SNP IDs	Cultivars	Chromosomes/scaffolds	Physical positions (bp)	SNPs
15124	CakSNP15124	*Kabuli*	Ca_Kabuli_Scaffold2763	4738	(C/G)
15125	CakSNP15125	*Kabuli*	Ca_Kabuli_Scaffold2763	4804	(A/T)
15126	CakSNP15126	*Kabuli*	Ca_Kabuli_Scaffold2763	4800	(T/A)
15127	CakSNP15127	*Kabuli*	Ca_Kabuli_Scaffold2763	4787	(A/G)
15128	CakSNP15128	*Kabuli*	Ca_Kabuli_Scaffold2763	4782	(C/T)
15129	CakSNP15129	*Kabuli*	Ca_Kabuli_Scaffold2763	25756	(T/G)
15130	CakSNP15130	*Kabuli*	Ca_Kabuli_Scaffold2763	25769	(A/G)
15131	CakSNP15131	*Kabuli*	Ca_Kabuli_Scaffold2763	25771	(T/G)
15132	CakSNP15132	*Kabuli*	Ca_Kabuli_Scaffold2763	43207	(G/A)
15133	CakSNP15133	*Kabuli*	Ca_Kabuli_Scaffold2763	49533	(T/A)
15134	CakSNP15134	*Kabuli*	Ca_Kabuli_Scaffold2763	82193	(C/A)
15135	CakSNP15135	*Kabuli*	Ca_Kabuli_Scaffold2763	82232	(C/T)
15136	CakSNP15136	*Kabuli*	Ca_Kabuli_Scaffold2763	82249	(G/A)
15137	CakSNP15137	*Kabuli*	Ca_Kabuli_Scaffold2763	82250	(T/C)
15138	CakSNP15138	*Kabuli*	Ca_Kabuli_Scaffold2763	82253	(G/C)
15139	CakSNP15139	*Kabuli*	Ca_Kabuli_Scaffold2763	82262	(G/C)
15140	CakSNP15140	*Kabuli*	Ca_Kabuli_Scaffold2763	82261	(C/G)
15141	CakSNP15141	*Kabuli*	Ca_Kabuli_Scaffold2763	82265	(G/T)
15142	CakSNP15142	*Kabuli*	Ca_Kabuli_Scaffold2763	82302	(T/A)
15143	CakSNP15143	*Kabuli*	Ca_Kabuli_Scaffold2763	82319	(T/C)
15144	CakSNP15144	*Kabuli*	Ca_Kabuli_Scaffold2763	82328	(A/G)
15145	CakSNP15145	*Kabuli*	Ca_Kabuli_Scaffold2763	82342	(C/T)
15146	CakSNP15146	*Kabuli*	Ca_Kabuli_Scaffold2763	82369	(G/A)
15147	CakSNP15147	*Kabuli*	Ca_Kabuli_Scaffold2763	82417	(C/T)
15148	CakSNP15148	*Kabuli*	Ca_Kabuli_Scaffold2763	82397	(T/C)
15149	CakSNP15149	*Kabuli*	Ca_Kabuli_Scaffold2763	95823	(G/A)
15150	CakSNP15150	*Kabuli*	Ca_Kabuli_Scaffold2763	99364	(A/G)
15151	CakSNP15151	*Kabuli*	Ca_Kabuli_Scaffold2763	99368	(C/G)
15152	CakSNP15152	*Kabuli*	Ca_Kabuli_Scaffold2763	99549	(C/A)
15153	CakSNP15153	*Kabuli*	Ca_Kabuli_Scaffold2763	104549	(G/C)
15154	CakSNP15154	*Kabuli*	Ca_Kabuli_Scaffold2763	104583	(T/C)
15155	CakSNP15155	*Kabuli*	Ca_Kabuli_Scaffold2763	107087	(A/G)
15156	CakSNP15156	*Kabuli*	Ca_Kabuli_Scaffold2763	107033	(T/A)
15157	CakSNP15157	*Kabuli*	Ca_Kabuli_Scaffold2763	107314	(A/G)
15158	CakSNP15158	*Kabuli*	Ca_Kabuli_Scaffold2763	107437	(T/C)
15159	CakSNP15159	*Kabuli*	Ca_Kabuli_Scaffold2763	107376	(C/T)
15160	CakSNP15160	*Kabuli*	Ca_Kabuli_Scaffold2763	107413	(A/G)
15161	CakSNP15161	*Kabuli*	Ca_Kabuli_Scaffold2763	108560	(T/G)

**S.N.**	**SNP IDs**	**Cultivars**	**Chromosomes/scaffolds**	**Physical positions (bp)**	**SNPs**
15162	CakSNP15162	*Kabuli*	Ca_Kabuli_Scaffold2763	108599	(T/A)
15163	CakSNP15163	*Kabuli*	Ca_Kabuli_Scaffold2763	108763	(T/G)
15164	CakSNP15164	*Kabuli*	Ca_Kabuli_Scaffold2763	117440	(C/T)
15165	CakSNP15165	*Kabuli*	Ca_Kabuli_Scaffold2763	117430	(A/C)
15166	CakSNP15166	*Kabuli*	Ca_Kabuli_Scaffold2763	125716	(C/T)
15167	CakSNP15167	*Kabuli*	Ca_Kabuli_Scaffold280	90875	(G/A)
15168	CakSNP15168	*Kabuli*	Ca_Kabuli_Scaffold280	90950	(C/A)
15169	CakSNP15169	*Kabuli*	Ca_Kabuli_Scaffold280	99357	(A/C)
15170	CakSNP15170	*Kabuli*	Ca_Kabuli_Scaffold280	101752	(G/C)
15171	CakSNP15171	*Kabuli*	Ca_Kabuli_Scaffold280	111160	(C/A)
15172	CakSNP15172	*Kabuli*	Ca_Kabuli_Scaffold2812	56649	(C/T)
15173	CakSNP15173	*Kabuli*	Ca_Kabuli_Scaffold2812	56658	(G/A)
15174	CakSNP15174	*Kabuli*	Ca_Kabuli_Scaffold2812	56672	(C/G)
15175	CakSNP15175	*Kabuli*	Ca_Kabuli_Scaffold2812	56702	(G/A)
15176	CakSNP15176	*Kabuli*	Ca_Kabuli_Scaffold2812	56714	(G/C)
15177	CakSNP15177	*Kabuli*	Ca_Kabuli_Scaffold2812	56648	(C/G)
15178	CakSNP15178	*Kabuli*	Ca_Kabuli_Scaffold2812	89844	(A/C)
15179	CakSNP15179	*Kabuli*	Ca_Kabuli_Scaffold2812	89940	(C/T)
15180	CakSNP15180	*Kabuli*	Ca_Kabuli_Scaffold2812	89888	(T/A)
15181	CakSNP15181	*Kabuli*	Ca_Kabuli_Scaffold2812	89887	(A/T)
15182	CakSNP15182	*Kabuli*	Ca_Kabuli_Scaffold2815	25626	(C/G)
15183	CakSNP15183	*Kabuli*	Ca_Kabuli_Scaffold2815	25655	(T/G)
15184	CakSNP15184	*Kabuli*	Ca_Kabuli_Scaffold2815	25668	(T/G)
15185	CakSNP15185	*Kabuli*	Ca_Kabuli_Scaffold2827	171	(C/T)
15186	CakSNP15186	*Kabuli*	Ca_Kabuli_Scaffold2827	172	(G/A)
15187	CakSNP15187	*Kabuli*	Ca_Kabuli_Scaffold2827	179	(T/G)
15188	CakSNP15188	*Kabuli*	Ca_Kabuli_Scaffold2827	210	(A/G)
15189	CakSNP15189	*Kabuli*	Ca_Kabuli_Scaffold2827	285	(T/G)
15190	CakSNP15190	*Kabuli*	Ca_Kabuli_Scaffold2827	258	(T/C)
15191	CakSNP15191	*Kabuli*	Ca_Kabuli_Scaffold2827	240	(T/G)
15192	CakSNP15192	*Kabuli*	Ca_Kabuli_Scaffold2827	222	(C/T)
15193	CakSNP15193	*Kabuli*	Ca_Kabuli_Scaffold284	242841	(A/T)
15194	CakSNP15194	*Kabuli*	Ca_Kabuli_Scaffold284	242778	(T/C)
15195	CakSNP15195	*Kabuli*	Ca_Kabuli_Scaffold284	522764	(T/G)
15196	CakSNP15196	*Kabuli*	Ca_Kabuli_Scaffold284	613764	(G/A)
15197	CakSNP15197	*Kabuli*	Ca_Kabuli_Scaffold284	613861	(C/T)
15198	CakSNP15198	*Kabuli*	Ca_Kabuli_Scaffold284	613850	(C/T)
15199	CakSNP15199	*Kabuli*	Ca_Kabuli_Scaffold284	613811	(G/A)

S.N.	SNP IDs	Cultivars	Chromosomes/scaffolds	Physical positions (bp)	SNPs
15200	CakSNP15200	*Kabuli*	Ca_Kabuli_Scaffold284	826690	(T/C)
15201	CakSNP15201	*Kabuli*	Ca_Kabuli_Scaffold284	826644	(G/A)
15202	CakSNP15202	*Kabuli*	Ca_Kabuli_Scaffold2845	7107	(G/A)
15203	CakSNP15203	*Kabuli*	Ca_Kabuli_Scaffold2848	2403	(C/A)
15204	CakSNP15204	*Kabuli*	Ca_Kabuli_Scaffold2848	2444	(C/A)
15205	CakSNP15205	*Kabuli*	Ca_Kabuli_Scaffold2848	2517	(G/A)
15206	CakSNP15206	*Kabuli*	Ca_Kabuli_Scaffold2848	2464	(A/G)
15207	CakSNP15207	*Kabuli*	Ca_Kabuli_Scaffold2853	14852	(C/G)
15208	CakSNP15208	*Kabuli*	Ca_Kabuli_Scaffold2853	14916	(A/G)
15209	CakSNP15209	*Kabuli*	Ca_Kabuli_Scaffold2853	14917	(G/A)
15210	CakSNP15210	*Kabuli*	Ca_Kabuli_Scaffold2853	15100	(T/C)
15211	CakSNP15211	*Kabuli*	Ca_Kabuli_Scaffold2853	15086	(G/A)
15212	CakSNP15212	*Kabuli*	Ca_Kabuli_Scaffold287	3224	(G/A)
15213	CakSNP15213	*Kabuli*	Ca_Kabuli_Scaffold287	163600	(G/C)
15214	CakSNP15214	*Kabuli*	Ca_Kabuli_Scaffold287	163591	(C/A)
15215	CakSNP15215	*Kabuli*	Ca_Kabuli_Scaffold287	163576	(G/A)
15216	CakSNP15216	*Kabuli*	Ca_Kabuli_Scaffold290	179998	(T/C)
15217	CakSNP15217	*Kabuli*	Ca_Kabuli_Scaffold290	179993	(C/T)
15218	CakSNP15218	*Kabuli*	Ca_Kabuli_Scaffold290	179984	(G/T)
15219	CakSNP15219	*Kabuli*	Ca_Kabuli_Scaffold290	179940	(G/A)
15220	CakSNP15220	*Kabuli*	Ca_Kabuli_Scaffold290	179937	(C/G)
15221	CakSNP15221	*Kabuli*	Ca_Kabuli_Scaffold290	179969	(A/T)
15222	CakSNP15222	*Kabuli*	Ca_Kabuli_Scaffold290	211634	(A/C)
15223	CakSNP15223	*Kabuli*	Ca_Kabuli_Scaffold290	212536	(A/C)
15224	CakSNP15224	*Kabuli*	Ca_Kabuli_Scaffold290	212514	(C/T)
15225	CakSNP15225	*Kabuli*	Ca_Kabuli_Scaffold290	255161	(G/A)
15226	CakSNP15226	*Kabuli*	Ca_Kabuli_Scaffold290	728010	(T/A)
15227	CakSNP15227	*Kabuli*	Ca_Kabuli_Scaffold290	824906	(C/T)
15228	CakSNP15228	*Kabuli*	Ca_Kabuli_Scaffold2907	31202	(G/A)
15229	CakSNP15229	*Kabuli*	Ca_Kabuli_Scaffold2950	32650	(C/T)
15230	CakSNP15230	*Kabuli*	Ca_Kabuli_Scaffold2950	32703	(A/C)
15231	CakSNP15231	*Kabuli*	Ca_Kabuli_Scaffold2950	32739	(C/A)
15232	CakSNP15232	*Kabuli*	Ca_Kabuli_Scaffold2950	32776	(T/A)
15233	CakSNP15233	*Kabuli*	Ca_Kabuli_Scaffold2950	33081	(A/G)
15234	CakSNP15234	*Kabuli*	Ca_Kabuli_Scaffold2950	33207	(C/T)
15235	CakSNP15235	*Kabuli*	Ca_Kabuli_Scaffold296	41422	(T/G)
15236	CakSNP15236	*Kabuli*	Ca_Kabuli_Scaffold296	59691	(G/A)
15237	CakSNP15237	*Kabuli*	Ca_Kabuli_Scaffold296	59758	(A/G)

S.N.	SNP IDs	Cultivars	Chromosomes/scaffolds	Physical positions (bp)	SNPs
15238	CakSNP15238	*Kabuli*	Ca_Kabuli_Scaffold296	59883	(A/C)
15239	CakSNP15239	*Kabuli*	Ca_Kabuli_Scaffold296	361664	(T/C)
15240	CakSNP15240	*Kabuli*	Ca_Kabuli_Scaffold299	199050	(A/T)
15241	CakSNP15241	*Kabuli*	Ca_Kabuli_Scaffold299	199067	(A/T)
15242	CakSNP15242	*Kabuli*	Ca_Kabuli_Scaffold299	199068	(G/T)
15243	CakSNP15243	*Kabuli*	Ca_Kabuli_Scaffold299	199087	(G/A)
15244	CakSNP15244	*Kabuli*	Ca_Kabuli_Scaffold299	199073	(G/A)
15245	CakSNP15245	*Kabuli*	Ca_Kabuli_Scaffold299	204540	(G/A)
15246	CakSNP15246	*Kabuli*	Ca_Kabuli_Scaffold300	111914	(C/T)
15247	CakSNP15247	*Kabuli*	Ca_Kabuli_Scaffold300	111924	(C/T)
15248	CakSNP15248	*Kabuli*	Ca_Kabuli_Scaffold300	111926	(G/A)
15249	CakSNP15249	*Kabuli*	Ca_Kabuli_Scaffold300	111933	(C/T)
15250	CakSNP15250	*Kabuli*	Ca_Kabuli_Scaffold300	111936	(C/A)
15251	CakSNP15251	*Kabuli*	Ca_Kabuli_Scaffold300	111972	(A/T)
15252	CakSNP15252	*Kabuli*	Ca_Kabuli_Scaffold300	111973	(A/T)
15253	CakSNP15253	*Kabuli*	Ca_Kabuli_Scaffold300	111976	(C/T)
15254	CakSNP15254	*Kabuli*	Ca_Kabuli_Scaffold300	112011	(T/C)
15255	CakSNP15255	*Kabuli*	Ca_Kabuli_Scaffold300	112006	(G/A)
15256	CakSNP15256	*Kabuli*	Ca_Kabuli_Scaffold300	112005	(G/A)
15257	CakSNP15257	*Kabuli*	Ca_Kabuli_Scaffold300	111959	(C/T)
15258	CakSNP15258	*Kabuli*	Ca_Kabuli_Scaffold300	111964	(C/T)
15259	CakSNP15259	*Kabuli*	Ca_Kabuli_Scaffold3016	32790	(C/T)
15260	CakSNP15260	*Kabuli*	Ca_Kabuli_Scaffold303	18213	(G/A)
15261	CakSNP15261	*Kabuli*	Ca_Kabuli_Scaffold305	49263	(G/A)
15262	CakSNP15262	*Kabuli*	Ca_Kabuli_Scaffold305	49266	(T/G)
15263	CakSNP15263	*Kabuli*	Ca_Kabuli_Scaffold305	49278	(C/A)
15264	CakSNP15264	*Kabuli*	Ca_Kabuli_Scaffold305	138929	(G/A)
15265	CakSNP15265	*Kabuli*	Ca_Kabuli_Scaffold306	15514	(C/T)
15266	CakSNP15266	*Kabuli*	Ca_Kabuli_Scaffold306	19215	(C/A)
15267	CakSNP15267	*Kabuli*	Ca_Kabuli_Scaffold306	85993	(T/A)
15268	CakSNP15268	*Kabuli*	Ca_Kabuli_Scaffold306	88389	(T/A)
15269	CakSNP15269	*Kabuli*	Ca_Kabuli_Scaffold3084	4409	(G/A)
15270	CakSNP15270	*Kabuli*	Ca_Kabuli_Scaffold308_2	9421	(G/A)
15271	CakSNP15271	*Kabuli*	Ca_Kabuli_Scaffold308_2	143696	(C/G)
15272	CakSNP15272	*Kabuli*	Ca_Kabuli_Scaffold308_2	143813	(C/G)
15273	CakSNP15273	*Kabuli*	Ca_Kabuli_Scaffold308_2	144354	(C/T)
15274	CakSNP15274	*Kabuli*	Ca_Kabuli_Scaffold308_2	144508	(C/T)
15275	CakSNP15275	*Kabuli*	Ca_Kabuli_Scaffold308_2	145639	(G/T)

S.N.	SNP IDs	Cultivars	Chromosomes/scaffolds	Physical positions (bp)	SNPs
15276	CakSNP15276	*Kabuli*	Ca_Kabuli_Scaffold308_2	152546	(A/G)
15277	CakSNP15277	*Kabuli*	Ca_Kabuli_Scaffold308_2	152576	(G/T)
15278	CakSNP15278	*Kabuli*	Ca_Kabuli_Scaffold311	86652	(C/A)
15279	CakSNP15279	*Kabuli*	Ca_Kabuli_Scaffold311	86677	(C/T)
15280	CakSNP15280	*Kabuli*	Ca_Kabuli_Scaffold311	86658	(C/T)
15281	CakSNP15281	*Kabuli*	Ca_Kabuli_Scaffold311	86670	(G/A)
15282	CakSNP15282	*Kabuli*	Ca_Kabuli_Scaffold3116	159	(T/G)
15283	CakSNP15283	*Kabuli*	Ca_Kabuli_Scaffold314	432453	(T/C)
15284	CakSNP15284	*Kabuli*	Ca_Kabuli_Scaffold314	432471	(A/G)
15285	CakSNP15285	*Kabuli*	Ca_Kabuli_Scaffold314	535269	(A/C)
15286	CakSNP15286	*Kabuli*	Ca_Kabuli_Scaffold314	535266	(A/C)
15287	CakSNP15287	*Kabuli*	Ca_Kabuli_Scaffold314	535263	(T/C)
15288	CakSNP15288	*Kabuli*	Ca_Kabuli_Scaffold314	610535	(C/G)
15289	CakSNP15289	*Kabuli*	Ca_Kabuli_Scaffold314	624361	(C/T)
15290	CakSNP15290	*Kabuli*	Ca_Kabuli_Scaffold3155	7438	(A/C)
15291	CakSNP15291	*Kabuli*	Ca_Kabuli_Scaffold3155	7442	(T/C)
15292	CakSNP15292	*Kabuli*	Ca_Kabuli_Scaffold3155	7443	(A/G)
15293	CakSNP15293	*Kabuli*	Ca_Kabuli_Scaffold3155	7444	(A/G)
15294	CakSNP15294	*Kabuli*	Ca_Kabuli_Scaffold3155	7455	(T/G)
15295	CakSNP15295	*Kabuli*	Ca_Kabuli_Scaffold3155	7462	(A/G)
15296	CakSNP15296	*Kabuli*	Ca_Kabuli_Scaffold3155	7464	(T/A)
15297	CakSNP15297	*Kabuli*	Ca_Kabuli_Scaffold3155	7472	(A/G)
15298	CakSNP15298	*Kabuli*	Ca_Kabuli_Scaffold3155	7477	(C/G)
15299	CakSNP15299	*Kabuli*	Ca_Kabuli_Scaffold3155	7480	(G/A)
15300	CakSNP15300	*Kabuli*	Ca_Kabuli_Scaffold3155	9404	(C/T)
15301	CakSNP15301	*Kabuli*	Ca_Kabuli_Scaffold3155	9746	(A/T)
15302	CakSNP15302	*Kabuli*	Ca_Kabuli_Scaffold3170	56261	(A/G)
15303	CakSNP15303	*Kabuli*	Ca_Kabuli_Scaffold322	55475	(G/A)
15304	CakSNP15304	*Kabuli*	Ca_Kabuli_Scaffold322	55479	(G/T)
15305	CakSNP15305	*Kabuli*	Ca_Kabuli_Scaffold322	55589	(A/T)
15306	CakSNP15306	*Kabuli*	Ca_Kabuli_Scaffold3228	2926	(C/T)
15307	CakSNP15307	*Kabuli*	Ca_Kabuli_Scaffold3228	23138	(A/G)
15308	CakSNP15308	*Kabuli*	Ca_Kabuli_Scaffold3228	23190	(G/C)
15309	CakSNP15309	*Kabuli*	Ca_Kabuli_Scaffold3228	23315	(C/A)
15310	CakSNP15310	*Kabuli*	Ca_Kabuli_Scaffold3228	23270	(A/C)
15311	CakSNP15311	*Kabuli*	Ca_Kabuli_Scaffold324	15569	(A/C)
15312	CakSNP15312	*Kabuli*	Ca_Kabuli_Scaffold324	15574	(G/A)
15313	CakSNP15313	*Kabuli*	Ca_Kabuli_Scaffold324	15575	(G/C)

S.N.	SNP IDs	Cultivars	Chromosomes/scaffolds	Physical positions (bp)	SNPs
15314	CakSNP15314	*Kabuli*	Ca_Kabuli_Scaffold324	15581	(G/C)
15315	CakSNP15315	*Kabuli*	Ca_Kabuli_Scaffold324	15590	(G/T)
15316	CakSNP15316	*Kabuli*	Ca_Kabuli_Scaffold324	15593	(G/A)
15317	CakSNP15317	*Kabuli*	Ca_Kabuli_Scaffold324	15594	(C/A)
15318	CakSNP15318	*Kabuli*	Ca_Kabuli_Scaffold324	15614	(C/T)
15319	CakSNP15319	*Kabuli*	Ca_Kabuli_Scaffold324	15619	(A/G)
15320	CakSNP15320	*Kabuli*	Ca_Kabuli_Scaffold324	15636	(G/A)
15321	CakSNP15321	*Kabuli*	Ca_Kabuli_Scaffold3254_2	15616	(A/G)
15322	CakSNP15322	*Kabuli*	Ca_Kabuli_Scaffold3254_2	28089	(T/C)
15323	CakSNP15323	*Kabuli*	Ca_Kabuli_Scaffold3254_2	31210	(T/A)
15324	CakSNP15324	*Kabuli*	Ca_Kabuli_Scaffold3254_2	31217	(C/T)
15325	CakSNP15325	*Kabuli*	Ca_Kabuli_Scaffold3254_2	31219	(A/C)
15326	CakSNP15326	*Kabuli*	Ca_Kabuli_Scaffold3254_2	31224	(T/G)
15327	CakSNP15327	*Kabuli*	Ca_Kabuli_Scaffold3254_2	31229	(G/T)
15328	CakSNP15328	*Kabuli*	Ca_Kabuli_Scaffold3254_2	31242	(C/A)
15329	CakSNP15329	*Kabuli*	Ca_Kabuli_Scaffold3254_2	31344	(A/G)
15330	CakSNP15330	*Kabuli*	Ca_Kabuli_Scaffold3254_2	31325	(T/A)
15331	CakSNP15331	*Kabuli*	Ca_Kabuli_Scaffold3254_2	31322	(G/A)
15332	CakSNP15332	*Kabuli*	Ca_Kabuli_Scaffold3254_2	31311	(G/A)
15333	CakSNP15333	*Kabuli*	Ca_Kabuli_Scaffold3254_2	31303	(T/C)
15334	CakSNP15334	*Kabuli*	Ca_Kabuli_Scaffold3254_2	39157	(T/A)
15335	CakSNP15335	*Kabuli*	Ca_Kabuli_Scaffold3254_2	46644	(G/A)
15336	CakSNP15336	*Kabuli*	Ca_Kabuli_Scaffold3254_2	186218	(G/A)
15337	CakSNP15337	*Kabuli*	Ca_Kabuli_Scaffold3254_2	186203	(C/G)
15338	CakSNP15338	*Kabuli*	Ca_Kabuli_Scaffold3254_2	186243	(A/C)
15339	CakSNP15339	*Kabuli*	Ca_Kabuli_Scaffold3284	2735	(T/C)
15340	CakSNP15340	*Kabuli*	Ca_Kabuli_Scaffold332	203691	(T/A)
15341	CakSNP15341	*Kabuli*	Ca_Kabuli_Scaffold332	204783	(A/G)
15342	CakSNP15342	*Kabuli*	Ca_Kabuli_Scaffold332	204785	(G/A)
15343	CakSNP15343	*Kabuli*	Ca_Kabuli_Scaffold332	204952	(G/A)
15344	CakSNP15344	*Kabuli*	Ca_Kabuli_Scaffold3321	17935	(C/A)
15345	CakSNP15345	*Kabuli*	Ca_Kabuli_Scaffold3337	17963	(C/T)
15346	CakSNP15346	*Kabuli*	Ca_Kabuli_Scaffold3337	17999	(T/G)
15347	CakSNP15347	*Kabuli*	Ca_Kabuli_Scaffold3337	18022	(C/T)
15348	CakSNP15348	*Kabuli*	Ca_Kabuli_Scaffold3337	18026	(T/A)
15349	CakSNP15349	*Kabuli*	Ca_Kabuli_Scaffold3337	17959	(A/G)
15350	CakSNP15350	*Kabuli*	Ca_Kabuli_Scaffold3337	17956	(G/A)
15351	CakSNP15351	*Kabuli*	Ca_Kabuli_Scaffold334	43160	(G/A)

S.N.	SNP IDs	Cultivars	Chromosomes/scaffolds	Physical positions (bp)	SNPs
15352	CakSNP15352	*Kabuli*	Ca_Kabuli_Scaffold335	60951	(T/C)
15353	CakSNP15353	*Kabuli*	Ca_Kabuli_Scaffold335	60952	(T/C)
15354	CakSNP15354	*Kabuli*	Ca_Kabuli_Scaffold335	60956	(T/C)
15355	CakSNP15355	*Kabuli*	Ca_Kabuli_Scaffold335	60957	(T/C)
15356	CakSNP15356	*Kabuli*	Ca_Kabuli_Scaffold335	60960	(T/C)
15357	CakSNP15357	*Kabuli*	Ca_Kabuli_Scaffold335	60961	(C/T)
15358	CakSNP15358	*Kabuli*	Ca_Kabuli_Scaffold335	60962	(T/G)
15359	CakSNP15359	*Kabuli*	Ca_Kabuli_Scaffold335	60906	(T/G)
15360	CakSNP15360	*Kabuli*	Ca_Kabuli_Scaffold335	135942	(T/A)
15361	CakSNP15361	*Kabuli*	Ca_Kabuli_Scaffold335	136017	(C/G)
15362	CakSNP15362	*Kabuli*	Ca_Kabuli_Scaffold335	135969	(C/T)
15363	CakSNP15363	*Kabuli*	Ca_Kabuli_Scaffold335	135976	(C/T)
15364	CakSNP15364	*Kabuli*	Ca_Kabuli_Scaffold335	136077	(G/C)
15365	CakSNP15365	*Kabuli*	Ca_Kabuli_Scaffold335	136076	(G/C)
15366	CakSNP15366	*Kabuli*	Ca_Kabuli_Scaffold335	136073	(G/A)
15367	CakSNP15367	*Kabuli*	Ca_Kabuli_Scaffold335	136051	(T/A)
15368	CakSNP15368	*Kabuli*	Ca_Kabuli_Scaffold335	136030	(G/A)
15369	CakSNP15369	*Kabuli*	Ca_Kabuli_Scaffold335	136129	(T/C)
15370	CakSNP15370	*Kabuli*	Ca_Kabuli_Scaffold335	136109	(G/T)
15371	CakSNP15371	*Kabuli*	Ca_Kabuli_Scaffold335	136106	(G/A)
15372	CakSNP15372	*Kabuli*	Ca_Kabuli_Scaffold335	136096	(T/C)
15373	CakSNP15373	*Kabuli*	Ca_Kabuli_Scaffold335	136091	(G/A)
15374	CakSNP15374	*Kabuli*	Ca_Kabuli_Scaffold336	468863	(T/A)
15375	CakSNP15375	*Kabuli*	Ca_Kabuli_Scaffold336	563363	(C/T)
15376	CakSNP15376	*Kabuli*	Ca_Kabuli_Scaffold336	618427	(C/T)
15377	CakSNP15377	*Kabuli*	Ca_Kabuli_Scaffold336	771169	(C/T)
15378	CakSNP15378	*Kabuli*	Ca_Kabuli_Scaffold336	771175	(C/T)
15379	CakSNP15379	*Kabuli*	Ca_Kabuli_Scaffold3362	69878	(C/A)
15380	CakSNP15380	*Kabuli*	Ca_Kabuli_Scaffold338	14666	(T/G)
15381	CakSNP15381	*Kabuli*	Ca_Kabuli_Scaffold3397	45363	(T/C)
15382	CakSNP15382	*Kabuli*	Ca_Kabuli_Scaffold342	311411	(G/A)
15383	CakSNP15383	*Kabuli*	Ca_Kabuli_Scaffold342	311394	(A/C)
15384	CakSNP15384	*Kabuli*	Ca_Kabuli_Scaffold342	359376	(A/C)
15385	CakSNP15385	*Kabuli*	Ca_Kabuli_Scaffold3422	13376	(T/A)
15386	CakSNP15386	*Kabuli*	Ca_Kabuli_Scaffold346_1	67110	(G/T)
15387	CakSNP15387	*Kabuli*	Ca_Kabuli_Scaffold346_1	105461	(A/G)
15388	CakSNP15388	*Kabuli*	Ca_Kabuli_Scaffold346_1	105521	(A/C)
15389	CakSNP15389	*Kabuli*	Ca_Kabuli_Scaffold346_1	105523	(G/A)

S.N.	SNP IDs	Cultivars	Chromosomes/scaffolds	Physical positions (bp)	SNPs
15390	CakSNP15390	*Kabuli*	Ca_Kabuli_Scaffold346_1	105546	(C/T)
15391	CakSNP15391	*Kabuli*	Ca_Kabuli_Scaffold349	51381	(A/T)
15392	CakSNP15392	*Kabuli*	Ca_Kabuli_Scaffold349	51396	(G/A)
15393	CakSNP15393	*Kabuli*	Ca_Kabuli_Scaffold349	51414	(C/T)
15394	CakSNP15394	*Kabuli*	Ca_Kabuli_Scaffold349	51435	(C/T)
15395	CakSNP15395	*Kabuli*	Ca_Kabuli_Scaffold349	51548	(T/C)
15396	CakSNP15396	*Kabuli*	Ca_Kabuli_Scaffold349	51637	(T/A)
15397	CakSNP15397	*Kabuli*	Ca_Kabuli_Scaffold349	51624	(G/A)
15398	CakSNP15398	*Kabuli*	Ca_Kabuli_Scaffold349	174027	(G/C)
15399	CakSNP15399	*Kabuli*	Ca_Kabuli_Scaffold349	174039	(A/T)
15400	CakSNP15400	*Kabuli*	Ca_Kabuli_Scaffold3514	42134	(G/A)
15401	CakSNP15401	*Kabuli*	Ca_Kabuli_Scaffold3514	42135	(G/T)
15402	CakSNP15402	*Kabuli*	Ca_Kabuli_Scaffold3514	42156	(G/A)
15403	CakSNP15403	*Kabuli*	Ca_Kabuli_Scaffold3514	42163	(T/C)
15404	CakSNP15404	*Kabuli*	Ca_Kabuli_Scaffold3514	42164	(A/G)
15405	CakSNP15405	*Kabuli*	Ca_Kabuli_Scaffold3514	42175	(G/A)
15406	CakSNP15406	*Kabuli*	Ca_Kabuli_Scaffold352	22672	(C/T)
15407	CakSNP15407	*Kabuli*	Ca_Kabuli_Scaffold352	22675	(G/A)
15408	CakSNP15408	*Kabuli*	Ca_Kabuli_Scaffold352	22693	(C/T)
15409	CakSNP15409	*Kabuli*	Ca_Kabuli_Scaffold352	22708	(A/G)
15410	CakSNP15410	*Kabuli*	Ca_Kabuli_Scaffold352	22787	(A/G)
15411	CakSNP15411	*Kabuli*	Ca_Kabuli_Scaffold352	22777	(T/G)
15412	CakSNP15412	*Kabuli*	Ca_Kabuli_Scaffold352	22726	(A/C)
15413	CakSNP15413	*Kabuli*	Ca_Kabuli_Scaffold36	167556	(C/T)
15414	CakSNP15414	*Kabuli*	Ca_Kabuli_Scaffold36	226511	(A/G)
15415	CakSNP15415	*Kabuli*	Ca_Kabuli_Scaffold362	84943	(A/G)
15416	CakSNP15416	*Kabuli*	Ca_Kabuli_Scaffold362	84945	(T/C)
15417	CakSNP15417	*Kabuli*	Ca_Kabuli_Scaffold362	84954	(T/C)
15418	CakSNP15418	*Kabuli*	Ca_Kabuli_Scaffold362	84972	(C/A)
15419	CakSNP15419	*Kabuli*	Ca_Kabuli_Scaffold362	84988	(T/G)
15420	CakSNP15420	*Kabuli*	Ca_Kabuli_Scaffold362	84994	(G/A)
15421	CakSNP15421	*Kabuli*	Ca_Kabuli_Scaffold362	164620	(C/T)
15422	CakSNP15422	*Kabuli*	Ca_Kabuli_Scaffold362	164789	(T/C)
15423	CakSNP15423	*Kabuli*	Ca_Kabuli_Scaffold362	164787	(C/T)
15424	CakSNP15424	*Kabuli*	Ca_Kabuli_Scaffold362	196556	(G/A)
15425	CakSNP15425	*Kabuli*	Ca_Kabuli_Scaffold362	219176	(T/C)
15426	CakSNP15426	*Kabuli*	Ca_Kabuli_Scaffold366	61810	(C/T)
15427	CakSNP15427	*Kabuli*	Ca_Kabuli_Scaffold369	162735	(T/G)

S.N.	SNP IDs	Cultivars	Chromosomes/scaffolds	Physical positions (bp)	SNPs
15428	CakSNP15428	*Kabuli*	Ca_Kabuli_Scaffold3693	81068	(C/T)
15429	CakSNP15429	*Kabuli*	Ca_Kabuli_Scaffold3693	81080	(C/A)
15430	CakSNP15430	*Kabuli*	Ca_Kabuli_Scaffold3693	81204	(C/T)
15431	CakSNP15431	*Kabuli*	Ca_Kabuli_Scaffold3724	68378	(G/A)
15432	CakSNP15432	*Kabuli*	Ca_Kabuli_Scaffold374	132907	(A/C)
15433	CakSNP15433	*Kabuli*	Ca_Kabuli_Scaffold374	132896	(T/C)
15434	CakSNP15434	*Kabuli*	Ca_Kabuli_Scaffold374	132873	(A/G)
15435	CakSNP15435	*Kabuli*	Ca_Kabuli_Scaffold374	132859	(C/T)
15436	CakSNP15436	*Kabuli*	Ca_Kabuli_Scaffold374	132858	(G/A)
15437	CakSNP15437	*Kabuli*	Ca_Kabuli_Scaffold374	132849	(A/C)
15438	CakSNP15438	*Kabuli*	Ca_Kabuli_Scaffold374	132843	(C/T)
15439	CakSNP15439	*Kabuli*	Ca_Kabuli_Scaffold374	198368	(T/C)
15440	CakSNP15440	*Kabuli*	Ca_Kabuli_Scaffold377	393535	(G/T)
15441	CakSNP15441	*Kabuli*	Ca_Kabuli_Scaffold377	393538	(G/A)
15442	CakSNP15442	*Kabuli*	Ca_Kabuli_Scaffold379	69075	(A/C)
15443	CakSNP15443	*Kabuli*	Ca_Kabuli_Scaffold379	144442	(G/A)
15444	CakSNP15444	*Kabuli*	Ca_Kabuli_Scaffold379	177306	(T/C)
15445	CakSNP15445	*Kabuli*	Ca_Kabuli_Scaffold379	177354	(G/A)
15446	CakSNP15446	*Kabuli*	Ca_Kabuli_Scaffold379	177340	(C/T)
15447	CakSNP15447	*Kabuli*	Ca_Kabuli_Scaffold379	227608	(A/T)
15448	CakSNP15448	*Kabuli*	Ca_Kabuli_Scaffold379	227604	(C/A)
15449	CakSNP15449	*Kabuli*	Ca_Kabuli_Scaffold379	227597	(T/A)
15450	CakSNP15450	*Kabuli*	Ca_Kabuli_Scaffold38	69499	(A/G)
15451	CakSNP15451	*Kabuli*	Ca_Kabuli_Scaffold38	413691	(G/A)
15452	CakSNP15452	*Kabuli*	Ca_Kabuli_Scaffold38	413713	(A/C)
15453	CakSNP15453	*Kabuli*	Ca_Kabuli_Scaffold38	413719	(G/A)
15454	CakSNP15454	*Kabuli*	Ca_Kabuli_Scaffold38	457392	(C/A)
15455	CakSNP15455	*Kabuli*	Ca_Kabuli_Scaffold38	457596	(T/A)
15456	CakSNP15456	*Kabuli*	Ca_Kabuli_Scaffold38	520372	(T/G)
15457	CakSNP15457	*Kabuli*	Ca_Kabuli_Scaffold38	520311	(G/A)
15458	CakSNP15458	*Kabuli*	Ca_Kabuli_Scaffold38	520377	(T/C)
15459	CakSNP15459	*Kabuli*	Ca_Kabuli_Scaffold38	537059	(A/T)
15460	CakSNP15460	*Kabuli*	Ca_Kabuli_Scaffold38	537063	(C/T)
15461	CakSNP15461	*Kabuli*	Ca_Kabuli_Scaffold3819	450	(T/A)
15462	CakSNP15462	*Kabuli*	Ca_Kabuli_Scaffold382	36686	(A/T)
15463	CakSNP15463	*Kabuli*	Ca_Kabuli_Scaffold382	36703	(A/T)
15464	CakSNP15464	*Kabuli*	Ca_Kabuli_Scaffold382	36704	(G/T)
15465	CakSNP15465	*Kabuli*	Ca_Kabuli_Scaffold382	36709	(G/A)

S.N.	SNP IDs	Cultivars	Chromosomes/scaffolds	Physical positions (bp)	SNPs
15466	CakSNP15466	*Kabuli*	Ca_Kabuli_Scaffold382	36674	(G/A)
15467	CakSNP15467	*Kabuli*	Ca_Kabuli_Scaffold3824	26207	(G/A)
15468	CakSNP15468	*Kabuli*	Ca_Kabuli_Scaffold3824	26267	(G/A)
15469	CakSNP15469	*Kabuli*	Ca_Kabuli_Scaffold3824	26327	(G/A)
15470	CakSNP15470	*Kabuli*	Ca_Kabuli_Scaffold3824	26387	(G/A)
15471	CakSNP15471	*Kabuli*	Ca_Kabuli_Scaffold3865	12265	(T/A)
15472	CakSNP15472	*Kabuli*	Ca_Kabuli_Scaffold3865	65093	(T/C)
15473	CakSNP15473	*Kabuli*	Ca_Kabuli_Scaffold3865	65127	(C/A)
15474	CakSNP15474	*Kabuli*	Ca_Kabuli_Scaffold3865	65129	(T/A)
15475	CakSNP15475	*Kabuli*	Ca_Kabuli_Scaffold387	3263	(G/A)
15476	CakSNP15476	*Kabuli*	Ca_Kabuli_Scaffold387	3219	(C/A)
15477	CakSNP15477	*Kabuli*	Ca_Kabuli_Scaffold387	3309	(T/C)
15478	CakSNP15478	*Kabuli*	Ca_Kabuli_Scaffold387	3316	(G/A)
15479	CakSNP15479	*Kabuli*	Ca_Kabuli_Scaffold3945	3367	(G/A)
15480	CakSNP15480	*Kabuli*	Ca_Kabuli_Scaffold3945	3371	(G/A)
15481	CakSNP15481	*Kabuli*	Ca_Kabuli_Scaffold3945	3880	(G/T)
15482	CakSNP15482	*Kabuli*	Ca_Kabuli_Scaffold3945	3879	(T/C)
15483	CakSNP15483	*Kabuli*	Ca_Kabuli_Scaffold3945	7260	(T/A)
15484	CakSNP15484	*Kabuli*	Ca_Kabuli_Scaffold3945	13468	(C/T)
15485	CakSNP15485	*Kabuli*	Ca_Kabuli_Scaffold3945	13440	(C/T)
15486	CakSNP15486	*Kabuli*	Ca_Kabuli_Scaffold3945	13892	(C/G)
15487	CakSNP15487	*Kabuli*	Ca_Kabuli_Scaffold3945	18891	(A/T)
15488	CakSNP15488	*Kabuli*	Ca_Kabuli_Scaffold3945	18892	(G/C)
15489	CakSNP15489	*Kabuli*	Ca_Kabuli_Scaffold3945	19359	(G/A)
15490	CakSNP15490	*Kabuli*	Ca_Kabuli_Scaffold3945	19366	(G/T)
15491	CakSNP15491	*Kabuli*	Ca_Kabuli_Scaffold3945	19367	(A/T)
15492	CakSNP15492	*Kabuli*	Ca_Kabuli_Scaffold3945	19368	(A/C)
15493	CakSNP15493	*Kabuli*	Ca_Kabuli_Scaffold396	130566	(T/C)
15494	CakSNP15494	*Kabuli*	Ca_Kabuli_Scaffold396	130671	(A/G)
15495	CakSNP15495	*Kabuli*	Ca_Kabuli_Scaffold396	130639	(C/A)
15496	CakSNP15496	*Kabuli*	Ca_Kabuli_Scaffold396	130636	(G/A)
15497	CakSNP15497	*Kabuli*	Ca_Kabuli_Scaffold396	130635	(G/T)
15498	CakSNP15498	*Kabuli*	Ca_Kabuli_Scaffold396	130624	(A/G)
15499	CakSNP15499	*Kabuli*	Ca_Kabuli_Scaffold396	130621	(C/A)
15500	CakSNP15500	*Kabuli*	Ca_Kabuli_Scaffold396	195890	(A/G)
15501	CakSNP15501	*Kabuli*	Ca_Kabuli_Scaffold396	297325	(G/C)
15502	CakSNP15502	*Kabuli*	Ca_Kabuli_Scaffold398	246956	(C/T)
15503	CakSNP15503	*Kabuli*	Ca_Kabuli_Scaffold398	247004	(C/T)

S.N.	SNP IDs	Cultivars	Chromosomes/scaffolds	Physical positions (bp)	SNPs
15504	CakSNP15504	*Kabuli*	Ca_Kabuli_Scaffold398	441570	(C/T)
15505	CakSNP15505	*Kabuli*	Ca_Kabuli_Scaffold398	441605	(C/A)
15506	CakSNP15506	*Kabuli*	Ca_Kabuli_Scaffold40	386189	(A/C)
15507	CakSNP15507	*Kabuli*	Ca_Kabuli_Scaffold40	497729	(T/C)
15508	CakSNP15508	*Kabuli*	Ca_Kabuli_Scaffold40	542951	(C/G)
15509	CakSNP15509	*Kabuli*	Ca_Kabuli_Scaffold40	548453	(A/G)
15510	CakSNP15510	*Kabuli*	Ca_Kabuli_Scaffold40	636910	(C/T)
15511	CakSNP15511	*Kabuli*	Ca_Kabuli_Scaffold40	691226	(C/G)
15512	CakSNP15512	*Kabuli*	Ca_Kabuli_Scaffold40	871303	(A/C)
15513	CakSNP15513	*Kabuli*	Ca_Kabuli_Scaffold40	911602	(T/G)
15514	CakSNP15514	*Kabuli*	Ca_Kabuli_Scaffold40	968798	(G/A)
15515	CakSNP15515	*Kabuli*	Ca_Kabuli_Scaffold40	996604	(T/C)
15516	CakSNP15516	*Kabuli*	Ca_Kabuli_Scaffold40	1014875	(G/A)
15517	CakSNP15517	*Kabuli*	Ca_Kabuli_Scaffold40	1014834	(T/A)
15518	CakSNP15518	*Kabuli*	Ca_Kabuli_Scaffold40	1014826	(C/T)
15519	CakSNP15519	*Kabuli*	Ca_Kabuli_Scaffold40	1014805	(C/T)
15520	CakSNP15520	*Kabuli*	Ca_Kabuli_Scaffold40	1039218	(T/C)
15521	CakSNP15521	*Kabuli*	Ca_Kabuli_Scaffold40	1039233	(A/T)
15522	CakSNP15522	*Kabuli*	Ca_Kabuli_Scaffold40	1049594	(G/A)
15523	CakSNP15523	*Kabuli*	Ca_Kabuli_Scaffold40	1050111	(C/T)
15524	CakSNP15524	*Kabuli*	Ca_Kabuli_Scaffold40	1050164	(C/T)
15525	CakSNP15525	*Kabuli*	Ca_Kabuli_Scaffold40	1050255	(T/C)
15526	CakSNP15526	*Kabuli*	Ca_Kabuli_Scaffold40	1050175	(G/C)
15527	CakSNP15527	*Kabuli*	Ca_Kabuli_Scaffold40	1052597	(T/C)
15528	CakSNP15528	*Kabuli*	Ca_Kabuli_Scaffold40	1052983	(G/A)
15529	CakSNP15529	*Kabuli*	Ca_Kabuli_Scaffold40	1053215	(G/A)
15530	CakSNP15530	*Kabuli*	Ca_Kabuli_Scaffold401	49065	(G/A)
15531	CakSNP15531	*Kabuli*	Ca_Kabuli_Scaffold4011	93334	(T/C)
15532	CakSNP15532	*Kabuli*	Ca_Kabuli_Scaffold4011	93347	(C/T)
15533	CakSNP15533	*Kabuli*	Ca_Kabuli_Scaffold4011	96223	(G/C)
15534	CakSNP15534	*Kabuli*	Ca_Kabuli_Scaffold4011	97602	(T/C)
15535	CakSNP15535	*Kabuli*	Ca_Kabuli_Scaffold4011	130271	(G/A)
15536	CakSNP15536	*Kabuli*	Ca_Kabuli_Scaffold4011	130213	(G/A)
15537	CakSNP15537	*Kabuli*	Ca_Kabuli_Scaffold404	273203	(C/A)
15538	CakSNP15538	*Kabuli*	Ca_Kabuli_Scaffold404	273209	(C/A)
15539	CakSNP15539	*Kabuli*	Ca_Kabuli_Scaffold404	273220	(G/A)
15540	CakSNP15540	*Kabuli*	Ca_Kabuli_Scaffold404	273245	(C/T)
15541	CakSNP15541	*Kabuli*	Ca_Kabuli_Scaffold404	285941	(G/A)

S.N.	SNP IDs	Cultivars	Chromosomes/scaffolds	Physical positions (bp)	SNPs
15542	CakSNP15542	*Kabuli*	Ca_Kabuli_Scaffold404	285952	(T/C)
15543	CakSNP15543	*Kabuli*	Ca_Kabuli_Scaffold404	285958	(C/G)
15544	CakSNP15544	*Kabuli*	Ca_Kabuli_Scaffold404	285987	(G/A)
15545	CakSNP15545	*Kabuli*	Ca_Kabuli_Scaffold404	285999	(T/G)
15546	CakSNP15546	*Kabuli*	Ca_Kabuli_Scaffold404	286013	(T/A)
15547	CakSNP15547	*Kabuli*	Ca_Kabuli_Scaffold404	355859	(G/A)
15548	CakSNP15548	*Kabuli*	Ca_Kabuli_Scaffold4057	16503	(C/G)
15549	CakSNP15549	*Kabuli*	Ca_Kabuli_Scaffold41	14657	(C/A)
15550	CakSNP15550	*Kabuli*	Ca_Kabuli_Scaffold41	122583	(G/C)
15551	CakSNP15551	*Kabuli*	Ca_Kabuli_Scaffold4102	1035	(C/G)
15552	CakSNP15552	*Kabuli*	Ca_Kabuli_Scaffold4102	1136	(A/G)
15553	CakSNP15553	*Kabuli*	Ca_Kabuli_Scaffold4102	1160	(T/C)
15554	CakSNP15554	*Kabuli*	Ca_Kabuli_Scaffold4102	1163	(G/T)
15555	CakSNP15555	*Kabuli*	Ca_Kabuli_Scaffold4102	1346	(C/T)
15556	CakSNP15556	*Kabuli*	Ca_Kabuli_Scaffold411	60078	(C/T)
15557	CakSNP15557	*Kabuli*	Ca_Kabuli_Scaffold411	323122	(T/C)
15558	CakSNP15558	*Kabuli*	Ca_Kabuli_Scaffold411	323140	(T/C)
15559	CakSNP15559	*Kabuli*	Ca_Kabuli_Scaffold411	323141	(G/A)
15560	CakSNP15560	*Kabuli*	Ca_Kabuli_Scaffold411	323147	(T/C)
15561	CakSNP15561	*Kabuli*	Ca_Kabuli_Scaffold411	323161	(G/A)
15562	CakSNP15562	*Kabuli*	Ca_Kabuli_Scaffold411	323162	(T/C)
15563	CakSNP15563	*Kabuli*	Ca_Kabuli_Scaffold411	323167	(C/G)
15564	CakSNP15564	*Kabuli*	Ca_Kabuli_Scaffold411	323179	(T/G)
15565	CakSNP15565	*Kabuli*	Ca_Kabuli_Scaffold411	323114	(C/G)
15566	CakSNP15566	*Kabuli*	Ca_Kabuli_Scaffold411	323215	(T/C)
15567	CakSNP15567	*Kabuli*	Ca_Kabuli_Scaffold415	8479	(A/C)
15568	CakSNP15568	*Kabuli*	Ca_Kabuli_Scaffold415	8529	(G/A)
15569	CakSNP15569	*Kabuli*	Ca_Kabuli_Scaffold418	6092	(C/G)
15570	CakSNP15570	*Kabuli*	Ca_Kabuli_Scaffold418	6094	(T/G)
15571	CakSNP15571	*Kabuli*	Ca_Kabuli_Scaffold418	13769	(T/G)
15572	CakSNP15572	*Kabuli*	Ca_Kabuli_Scaffold418	13804	(T/G)
15573	CakSNP15573	*Kabuli*	Ca_Kabuli_Scaffold418	13808	(A/G)
15574	CakSNP15574	*Kabuli*	Ca_Kabuli_Scaffold418	13811	(G/A)
15575	CakSNP15575	*Kabuli*	Ca_Kabuli_Scaffold418	13812	(C/A)
15576	CakSNP15576	*Kabuli*	Ca_Kabuli_Scaffold418	151140	(G/T)
15577	CakSNP15577	*Kabuli*	Ca_Kabuli_Scaffold418	151205	(C/T)
15578	CakSNP15578	*Kabuli*	Ca_Kabuli_Scaffold418	151256	(G/A)
15579	CakSNP15579	*Kabuli*	Ca_Kabuli_Scaffold419	73537	(T/G)

S.N.	SNP IDs	Cultivars	Chromosomes/scaffolds	Physical positions (bp)	SNPs
15580	CakSNP15580	*Kabuli*	Ca_Kabuli_Scaffold420	34145	(T/G)
15581	CakSNP15581	*Kabuli*	Ca_Kabuli_Scaffold420	42532	(G/A)
15582	CakSNP15582	*Kabuli*	Ca_Kabuli_Scaffold420	42739	(C/T)
15583	CakSNP15583	*Kabuli*	Ca_Kabuli_Scaffold420	42811	(C/T)
15584	CakSNP15584	*Kabuli*	Ca_Kabuli_Scaffold420	42877	(T/C)
15585	CakSNP15585	*Kabuli*	Ca_Kabuli_Scaffold420	43372	(C/T)
15586	CakSNP15586	*Kabuli*	Ca_Kabuli_Scaffold420	92756	(C/T)
15587	CakSNP15587	*Kabuli*	Ca_Kabuli_Scaffold420	92759	(A/G)
15588	CakSNP15588	*Kabuli*	Ca_Kabuli_Scaffold420	92784	(C/T)
15589	CakSNP15589	*Kabuli*	Ca_Kabuli_Scaffold420	92813	(G/T)
15590	CakSNP15590	*Kabuli*	Ca_Kabuli_Scaffold420	92865	(G/T)
15591	CakSNP15591	*Kabuli*	Ca_Kabuli_Scaffold420	92846	(C/T)
15592	CakSNP15592	*Kabuli*	Ca_Kabuli_Scaffold420	92801	(A/C)
15593	CakSNP15593	*Kabuli*	Ca_Kabuli_Scaffold420	149744	(C/G)
15594	CakSNP15594	*Kabuli*	Ca_Kabuli_Scaffold420	242536	(G/T)
15595	CakSNP15595	*Kabuli*	Ca_Kabuli_Scaffold420	242503	(C/T)
15596	CakSNP15596	*Kabuli*	Ca_Kabuli_Scaffold420	242487	(A/G)
15597	CakSNP15597	*Kabuli*	Ca_Kabuli_Scaffold420	242922	(A/C)
15598	CakSNP15598	*Kabuli*	Ca_Kabuli_Scaffold420	254661	(T/G)
15599	CakSNP15599	*Kabuli*	Ca_Kabuli_Scaffold420	341146	(T/A)
15600	CakSNP15600	*Kabuli*	Ca_Kabuli_Scaffold420	341184	(A/G)
15601	CakSNP15601	*Kabuli*	Ca_Kabuli_Scaffold420	341202	(T/G)
15602	CakSNP15602	*Kabuli*	Ca_Kabuli_Scaffold420	341206	(G/A)
15603	CakSNP15603	*Kabuli*	Ca_Kabuli_Scaffold421_1	25389	(G/A)
15604	CakSNP15604	*Kabuli*	Ca_Kabuli_Scaffold421_1	71804	(A/G)
15605	CakSNP15605	*Kabuli*	Ca_Kabuli_Scaffold421_1	71770	(G/T)
15606	CakSNP15606	*Kabuli*	Ca_Kabuli_Scaffold421_2	142859	(G/C)
15607	CakSNP15607	*Kabuli*	Ca_Kabuli_Scaffold421_2	341095	(C/A)
15608	CakSNP15608	*Kabuli*	Ca_Kabuli_Scaffold421_2	341136	(C/A)
15609	CakSNP15609	*Kabuli*	Ca_Kabuli_Scaffold421_2	446961	(G/A)
15610	CakSNP15610	*Kabuli*	Ca_Kabuli_Scaffold421_2	446976	(C/A)
15611	CakSNP15611	*Kabuli*	Ca_Kabuli_Scaffold421_2	446979	(C/T)
15612	CakSNP15612	*Kabuli*	Ca_Kabuli_Scaffold424	32402	(C/G)
15613	CakSNP15613	*Kabuli*	Ca_Kabuli_Scaffold4331	4044	(T/G)
15614	CakSNP15614	*Kabuli*	Ca_Kabuli_Scaffold44	66309	(A/C)
15615	CakSNP15615	*Kabuli*	Ca_Kabuli_Scaffold44	66280	(G/A)
15616	CakSNP15616	*Kabuli*	Ca_Kabuli_Scaffold44	87412	(G/A)
15617	CakSNP15617	*Kabuli*	Ca_Kabuli_Scaffold4414	276	(C/T)

S.N.	SNP IDs	Cultivars	Chromosomes/scaffolds	Physical positions (bp)	SNPs
15618	CakSNP15618	*Kabuli*	Ca_Kabuli_Scaffold4452	20003	(G/A)
15619	CakSNP15619	*Kabuli*	Ca_Kabuli_Scaffold4452	20005	(G/A)
15620	CakSNP15620	*Kabuli*	Ca_Kabuli_Scaffold4452	20038	(A/G)
15621	CakSNP15621	*Kabuli*	Ca_Kabuli_Scaffold4452	20010	(G/A)
15622	CakSNP15622	*Kabuli*	Ca_Kabuli_Scaffold4452	29414	(G/T)
15623	CakSNP15623	*Kabuli*	Ca_Kabuli_Scaffold4452	36715	(A/T)
15624	CakSNP15624	*Kabuli*	Ca_Kabuli_Scaffold4452	69950	(A/C)
15625	CakSNP15625	*Kabuli*	Ca_Kabuli_Scaffold4452	70041	(T/C)
15626	CakSNP15626	*Kabuli*	Ca_Kabuli_Scaffold450	132367	(C/T)
15627	CakSNP15627	*Kabuli*	Ca_Kabuli_Scaffold450	132427	(A/T)
15628	CakSNP15628	*Kabuli*	Ca_Kabuli_Scaffold450	197287	(G/A)
15629	CakSNP15629	*Kabuli*	Ca_Kabuli_Scaffold450	197272	(G/A)
15630	CakSNP15630	*Kabuli*	Ca_Kabuli_Scaffold450	197262	(T/G)
15631	CakSNP15631	*Kabuli*	Ca_Kabuli_Scaffold450	197230	(C/A)
15632	CakSNP15632	*Kabuli*	Ca_Kabuli_Scaffold450	197226	(G/A)
15633	CakSNP15633	*Kabuli*	Ca_Kabuli_Scaffold450	250303	(G/T)
15634	CakSNP15634	*Kabuli*	Ca_Kabuli_Scaffold451	103555	(G/C)
15635	CakSNP15635	*Kabuli*	Ca_Kabuli_Scaffold451	103596	(A/G)
15636	CakSNP15636	*Kabuli*	Ca_Kabuli_Scaffold451	117231	(A/G)
15637	CakSNP15637	*Kabuli*	Ca_Kabuli_Scaffold451	117300	(C/A)
15638	CakSNP15638	*Kabuli*	Ca_Kabuli_Scaffold4511	40072	(C/A)
15639	CakSNP15639	*Kabuli*	Ca_Kabuli_Scaffold4511	40014	(C/T)
15640	CakSNP15640	*Kabuli*	Ca_Kabuli_Scaffold4511	40089	(C/T)
15641	CakSNP15641	*Kabuli*	Ca_Kabuli_Scaffold452	1892	(G/A)
15642	CakSNP15642	*Kabuli*	Ca_Kabuli_Scaffold452	1881	(A/G)
15643	CakSNP15643	*Kabuli*	Ca_Kabuli_Scaffold452	1841	(A/G)
15644	CakSNP15644	*Kabuli*	Ca_Kabuli_Scaffold452	1837	(A/C)
15645	CakSNP15645	*Kabuli*	Ca_Kabuli_Scaffold452	1836	(A/G)
15646	CakSNP15646	*Kabuli*	Ca_Kabuli_Scaffold452	1835	(C/T)
15647	CakSNP15647	*Kabuli*	Ca_Kabuli_Scaffold453	303193	(T/C)
15648	CakSNP15648	*Kabuli*	Ca_Kabuli_Scaffold453	303201	(G/C)
15649	CakSNP15649	*Kabuli*	Ca_Kabuli_Scaffold453	303246	(A/G)
15650	CakSNP15650	*Kabuli*	Ca_Kabuli_Scaffold453	303248	(C/T)
15651	CakSNP15651	*Kabuli*	Ca_Kabuli_Scaffold453	303259	(C/G)
15652	CakSNP15652	*Kabuli*	Ca_Kabuli_Scaffold46	39438	(C/A)
15653	CakSNP15653	*Kabuli*	Ca_Kabuli_Scaffold46	39470	(C/T)
15654	CakSNP15654	*Kabuli*	Ca_Kabuli_Scaffold461	20652	(C/A)
15655	CakSNP15655	*Kabuli*	Ca_Kabuli_Scaffold461	20629	(A/T)

S.N.	SNP IDs	Cultivars	Chromosomes/scaffolds	Physical positions (bp)	SNPs
15656	CakSNP15656	*Kabuli*	Ca_Kabuli_Scaffold461	20603	(C/T)
15657	CakSNP15657	*Kabuli*	Ca_Kabuli_Scaffold461	20599	(C/A)
15658	CakSNP15658	*Kabuli*	Ca_Kabuli_Scaffold4620	2699	(T/C)
15659	CakSNP15659	*Kabuli*	Ca_Kabuli_Scaffold4620	23442	(T/C)
15660	CakSNP15660	*Kabuli*	Ca_Kabuli_Scaffold4641	511	(G/A)
15661	CakSNP15661	*Kabuli*	Ca_Kabuli_Scaffold4641	547	(A/C)
15662	CakSNP15662	*Kabuli*	Ca_Kabuli_Scaffold4641	553	(C/A)
15663	CakSNP15663	*Kabuli*	Ca_Kabuli_Scaffold4641	556	(G/C)
15664	CakSNP15664	*Kabuli*	Ca_Kabuli_Scaffold4662	7683	(T/C)
15665	CakSNP15665	*Kabuli*	Ca_Kabuli_Scaffold4662	7651	(T/C)
15666	CakSNP15666	*Kabuli*	Ca_Kabuli_Scaffold4662	7638	(C/T)
15667	CakSNP15667	*Kabuli*	Ca_Kabuli_Scaffold4662	7632	(A/G)
15668	CakSNP15668	*Kabuli*	Ca_Kabuli_Scaffold4695	5685	(A/G)
15669	CakSNP15669	*Kabuli*	Ca_Kabuli_Scaffold470	7175	(A/G)
15670	CakSNP15670	*Kabuli*	Ca_Kabuli_Scaffold473	24996	(G/A)
15671	CakSNP15671	*Kabuli*	Ca_Kabuli_Scaffold473	24984	(T/C)
15672	CakSNP15672	*Kabuli*	Ca_Kabuli_Scaffold473	24973	(T/C)
15673	CakSNP15673	*Kabuli*	Ca_Kabuli_Scaffold475	135286	(C/A)
15674	CakSNP15674	*Kabuli*	Ca_Kabuli_Scaffold475	135290	(G/A)
15675	CakSNP15675	*Kabuli*	Ca_Kabuli_Scaffold477	51898	(C/T)
15676	CakSNP15676	*Kabuli*	Ca_Kabuli_Scaffold4777	10546	(G/T)
15677	CakSNP15677	*Kabuli*	Ca_Kabuli_Scaffold4777	16224	(A/T)
15678	CakSNP15678	*Kabuli*	Ca_Kabuli_Scaffold4777	16239	(G/T)
15679	CakSNP15679	*Kabuli*	Ca_Kabuli_Scaffold4777	55347	(G/T)
15680	CakSNP15680	*Kabuli*	Ca_Kabuli_Scaffold4777	72624	(G/A)
15681	CakSNP15681	*Kabuli*	Ca_Kabuli_Scaffold4777	76950	(C/A)
15682	CakSNP15682	*Kabuli*	Ca_Kabuli_Scaffold4777	106820	(A/G)
15683	CakSNP15683	*Kabuli*	Ca_Kabuli_Scaffold4777	112193	(A/C)
15684	CakSNP15684	*Kabuli*	Ca_Kabuli_Scaffold48	261719	(A/G)
15685	CakSNP15685	*Kabuli*	Ca_Kabuli_Scaffold48	420925	(A/G)
15686	CakSNP15686	*Kabuli*	Ca_Kabuli_Scaffold480	67375	(T/C)
15687	CakSNP15687	*Kabuli*	Ca_Kabuli_Scaffold4836	5866	(T/A)
15688	CakSNP15688	*Kabuli*	Ca_Kabuli_Scaffold4836	5891	(A/T)
15689	CakSNP15689	*Kabuli*	Ca_Kabuli_Scaffold4836	5909	(G/A)
15690	CakSNP15690	*Kabuli*	Ca_Kabuli_Scaffold4836	5846	(C/T)
15691	CakSNP15691	*Kabuli*	Ca_Kabuli_Scaffold484	203947	(C/T)
15692	CakSNP15692	*Kabuli*	Ca_Kabuli_Scaffold484	209786	(A/C)
15693	CakSNP15693	*Kabuli*	Ca_Kabuli_Scaffold484	243567	(C/T)

S.N.	SNP IDs	Cultivars	Chromosomes/scaffolds	Physical positions (bp)	SNPs
15694	CakSNP15694	*Kabuli*	Ca_Kabuli_Scaffold484	243575	(T/A)
15695	CakSNP15695	*Kabuli*	Ca_Kabuli_Scaffold484	647868	(C/T)
15696	CakSNP15696	*Kabuli*	Ca_Kabuli_Scaffold484	647866	(A/G)
15697	CakSNP15697	*Kabuli*	Ca_Kabuli_Scaffold484	647809	(A/T)
15698	CakSNP15698	*Kabuli*	Ca_Kabuli_Scaffold484	660749	(C/G)
15699	CakSNP15699	*Kabuli*	Ca_Kabuli_Scaffold485	103572	(C/T)
15700	CakSNP15700	*Kabuli*	Ca_Kabuli_Scaffold485	103546	(A/G)
15701	CakSNP15701	*Kabuli*	Ca_Kabuli_Scaffold485	103528	(A/C)
15702	CakSNP15702	*Kabuli*	Ca_Kabuli_Scaffold485	123578	(T/C)
15703	CakSNP15703	*Kabuli*	Ca_Kabuli_Scaffold485	123642	(G/A)
15704	CakSNP15704	*Kabuli*	Ca_Kabuli_Scaffold495	128200	(A/T)
15705	CakSNP15705	*Kabuli*	Ca_Kabuli_Scaffold496	53652	(G/A)
15706	CakSNP15706	*Kabuli*	Ca_Kabuli_Scaffold496	53657	(A/T)
15707	CakSNP15707	*Kabuli*	Ca_Kabuli_Scaffold496	53616	(G/T)
15708	CakSNP15708	*Kabuli*	Ca_Kabuli_Scaffold496	53587	(A/G)
15709	CakSNP15709	*Kabuli*	Ca_Kabuli_Scaffold496	175429	(G/A)
15710	CakSNP15710	*Kabuli*	Ca_Kabuli_Scaffold496	175493	(G/A)
15711	CakSNP15711	*Kabuli*	Ca_Kabuli_Scaffold498	146772	(A/C)
15712	CakSNP15712	*Kabuli*	Ca_Kabuli_Scaffold498	148658	(G/T)
15713	CakSNP15713	*Kabuli*	Ca_Kabuli_Scaffold498	164638	(T/C)
15714	CakSNP15714	*Kabuli*	Ca_Kabuli_Scaffold498	164615	(G/C)
15715	CakSNP15715	*Kabuli*	Ca_Kabuli_Scaffold498	164590	(T/A)
15716	CakSNP15716	*Kabuli*	Ca_Kabuli_Scaffold50	153593	(T/A)
15717	CakSNP15717	*Kabuli*	Ca_Kabuli_Scaffold50	153599	(C/G)
15718	CakSNP15718	*Kabuli*	Ca_Kabuli_Scaffold50	153631	(C/T)
15719	CakSNP15719	*Kabuli*	Ca_Kabuli_Scaffold50	153734	(A/C)
15720	CakSNP15720	*Kabuli*	Ca_Kabuli_Scaffold50	153851	(T/C)
15721	CakSNP15721	*Kabuli*	Ca_Kabuli_Scaffold50	191625	(T/C)
15722	CakSNP15722	*Kabuli*	Ca_Kabuli_Scaffold50	201219	(C/G)
15723	CakSNP15723	*Kabuli*	Ca_Kabuli_Scaffold50	448866	(G/A)
15724	CakSNP15724	*Kabuli*	Ca_Kabuli_Scaffold510	12378	(T/A)
15725	CakSNP15725	*Kabuli*	Ca_Kabuli_Scaffold510	12383	(G/T)
15726	CakSNP15726	*Kabuli*	Ca_Kabuli_Scaffold510	12394	(C/A)
15727	CakSNP15727	*Kabuli*	Ca_Kabuli_Scaffold510	12414	(C/T)
15728	CakSNP15728	*Kabuli*	Ca_Kabuli_Scaffold510	12434	(C/T)
15729	CakSNP15729	*Kabuli*	Ca_Kabuli_Scaffold511	10450	(T/G)
15730	CakSNP15730	*Kabuli*	Ca_Kabuli_Scaffold511	10520	(G/T)
15731	CakSNP15731	*Kabuli*	Ca_Kabuli_Scaffold511	173376	(C/A)

S.N.	SNP IDs	Cultivars	Chromosomes/scaffolds	Physical positions (bp)	SNPs
15732	CakSNP15732	*Kabuli*	Ca_Kabuli_Scaffold511	173377	(A/G)
15733	CakSNP15733	*Kabuli*	Ca_Kabuli_Scaffold511	173378	(G/A)
15734	CakSNP15734	*Kabuli*	Ca_Kabuli_Scaffold513	7188	(A/C)
15735	CakSNP15735	*Kabuli*	Ca_Kabuli_Scaffold5163	2301	(A/G)
15736	CakSNP15736	*Kabuli*	Ca_Kabuli_Scaffold5185	1091	(G/A)
15737	CakSNP15737	*Kabuli*	Ca_Kabuli_Scaffold520	143359	(C/T)
15738	CakSNP15738	*Kabuli*	Ca_Kabuli_Scaffold520	143358	(G/A)
15739	CakSNP15739	*Kabuli*	Ca_Kabuli_Scaffold520	143356	(C/G)
15740	CakSNP15740	*Kabuli*	Ca_Kabuli_Scaffold520	143342	(G/A)
15741	CakSNP15741	*Kabuli*	Ca_Kabuli_Scaffold520	143336	(T/C)
15742	CakSNP15742	*Kabuli*	Ca_Kabuli_Scaffold520	143334	(G/A)
15743	CakSNP15743	*Kabuli*	Ca_Kabuli_Scaffold520	143333	(A/T)
15744	CakSNP15744	*Kabuli*	Ca_Kabuli_Scaffold520	143324	(G/A)
15745	CakSNP15745	*Kabuli*	Ca_Kabuli_Scaffold520	143317	(T/A)
15746	CakSNP15746	*Kabuli*	Ca_Kabuli_Scaffold520	143300	(G/A)
15747	CakSNP15747	*Kabuli*	Ca_Kabuli_Scaffold520	143294	(G/A)
15748	CakSNP15748	*Kabuli*	Ca_Kabuli_Scaffold520	143291	(G/A)
15749	CakSNP15749	*Kabuli*	Ca_Kabuli_Scaffold520	143375	(G/A)
15750	CakSNP15750	*Kabuli*	Ca_Kabuli_Scaffold520	143372	(G/A)
15751	CakSNP15751	*Kabuli*	Ca_Kabuli_Scaffold520	143355	(C/T)
15752	CakSNP15752	*Kabuli*	Ca_Kabuli_Scaffold520	143390	(A/G)
15753	CakSNP15753	*Kabuli*	Ca_Kabuli_Scaffold520	143391	(A/C)
15754	CakSNP15754	*Kabuli*	Ca_Kabuli_Scaffold528	191764	(A/G)
15755	CakSNP15755	*Kabuli*	Ca_Kabuli_Scaffold528	191835	(A/G)
15756	CakSNP15756	*Kabuli*	Ca_Kabuli_Scaffold528	232014	(A/C)
15757	CakSNP15757	*Kabuli*	Ca_Kabuli_Scaffold53	49273	(A/G)
15758	CakSNP15758	*Kabuli*	Ca_Kabuli_Scaffold53	123293	(C/A)
15759	CakSNP15759	*Kabuli*	Ca_Kabuli_Scaffold531	2668	(C/T)
15760	CakSNP15760	*Kabuli*	Ca_Kabuli_Scaffold531	2674	(C/T)
15761	CakSNP15761	*Kabuli*	Ca_Kabuli_Scaffold531	65242	(T/G)
15762	CakSNP15762	*Kabuli*	Ca_Kabuli_Scaffold531	101520	(C/G)
15763	CakSNP15763	*Kabuli*	Ca_Kabuli_Scaffold531	124019	(T/G)
15764	CakSNP15764	*Kabuli*	Ca_Kabuli_Scaffold5328	70	(G/A)
15765	CakSNP15765	*Kabuli*	Ca_Kabuli_Scaffold5328	85	(C/T)
15766	CakSNP15766	*Kabuli*	Ca_Kabuli_Scaffold5328	86	(C/T)
15767	CakSNP15767	*Kabuli*	Ca_Kabuli_Scaffold5328	117	(C/T)
15768	CakSNP15768	*Kabuli*	Ca_Kabuli_Scaffold535	156836	(G/A)
15769	CakSNP15769	*Kabuli*	Ca_Kabuli_Scaffold5358	2561	(C/A)

S.N.	SNP IDs	Cultivars	Chromosomes/scaffolds	Physical positions (bp)	SNPs
15770	CakSNP15770	*Kabuli*	Ca_Kabuli_Scaffold5358	3329	(C/A)
15771	CakSNP15771	*Kabuli*	Ca_Kabuli_Scaffold537	216656	(A/G)
15772	CakSNP15772	*Kabuli*	Ca_Kabuli_Scaffold537	216669	(T/A)
15773	CakSNP15773	*Kabuli*	Ca_Kabuli_Scaffold537	216846	(C/T)
15774	CakSNP15774	*Kabuli*	Ca_Kabuli_Scaffold537	216829	(C/G)
15775	CakSNP15775	*Kabuli*	Ca_Kabuli_Scaffold537	216778	(T/A)
15776	CakSNP15776	*Kabuli*	Ca_Kabuli_Scaffold537	223108	(T/C)
15777	CakSNP15777	*Kabuli*	Ca_Kabuli_Scaffold543	20780	(T/G)
15778	CakSNP15778	*Kabuli*	Ca_Kabuli_Scaffold543	20819	(T/C)
15779	CakSNP15779	*Kabuli*	Ca_Kabuli_Scaffold543	20827	(G/T)
15780	CakSNP15780	*Kabuli*	Ca_Kabuli_Scaffold543	143169	(C/T)
15781	CakSNP15781	*Kabuli*	Ca_Kabuli_Scaffold543	143182	(G/A)
15782	CakSNP15782	*Kabuli*	Ca_Kabuli_Scaffold543	143185	(A/G)
15783	CakSNP15783	*Kabuli*	Ca_Kabuli_Scaffold543	143191	(G/A)
15784	CakSNP15784	*Kabuli*	Ca_Kabuli_Scaffold543	266748	(A/C)
15785	CakSNP15785	*Kabuli*	Ca_Kabuli_Scaffold543	266729	(A/C)
15786	CakSNP15786	*Kabuli*	Ca_Kabuli_Scaffold543	266848	(A/C)
15787	CakSNP15787	*Kabuli*	Ca_Kabuli_Scaffold545	17699	(A/C)
15788	CakSNP15788	*Kabuli*	Ca_Kabuli_Scaffold545	138848	(T/C)
15789	CakSNP15789	*Kabuli*	Ca_Kabuli_Scaffold545	139118	(A/T)
15790	CakSNP15790	*Kabuli*	Ca_Kabuli_Scaffold545	146720	(A/G)
15791	CakSNP15791	*Kabuli*	Ca_Kabuli_Scaffold545	147549	(G/A)
15792	CakSNP15792	*Kabuli*	Ca_Kabuli_Scaffold545	147568	(T/G)
15793	CakSNP15793	*Kabuli*	Ca_Kabuli_Scaffold545	226799	(G/A)
15794	CakSNP15794	*Kabuli*	Ca_Kabuli_Scaffold545	226769	(G/C)
15795	CakSNP15795	*Kabuli*	Ca_Kabuli_Scaffold548	17462	(G/A)
15796	CakSNP15796	*Kabuli*	Ca_Kabuli_Scaffold548	231396	(G/A)
15797	CakSNP15797	*Kabuli*	Ca_Kabuli_Scaffold548	231338	(G/A)
15798	CakSNP15798	*Kabuli*	Ca_Kabuli_Scaffold5511	353	(G/A)
15799	CakSNP15799	*Kabuli*	Ca_Kabuli_Scaffold5511	361	(G/A)
15800	CakSNP15800	*Kabuli*	Ca_Kabuli_Scaffold5511	366	(G/A)
15801	CakSNP15801	*Kabuli*	Ca_Kabuli_Scaffold5511	376	(C/T)
15802	CakSNP15802	*Kabuli*	Ca_Kabuli_Scaffold5511	387	(T/C)
15803	CakSNP15803	*Kabuli*	Ca_Kabuli_Scaffold5511	388	(G/A)
15804	CakSNP15804	*Kabuli*	Ca_Kabuli_Scaffold5511	365	(T/G)
15805	CakSNP15805	*Kabuli*	Ca_Kabuli_Scaffold5511	396	(G/A)
15806	CakSNP15806	*Kabuli*	Ca_Kabuli_Scaffold5511	458	(G/A)
15807	CakSNP15807	*Kabuli*	Ca_Kabuli_Scaffold5511	447	(A/C)

S.N.	SNP IDs	Cultivars	Chromosomes/scaffolds	Physical positions (bp)	SNPs
15808	CakSNP15808	*Kabuli*	Ca_Kabuli_Scaffold5511	434	(C/T)
15809	CakSNP15809	*Kabuli*	Ca_Kabuli_Scaffold5511	404	(T/G)
15810	CakSNP15810	*Kabuli*	Ca_Kabuli_Scaffold553	302105	(T/C)
15811	CakSNP15811	*Kabuli*	Ca_Kabuli_Scaffold553	302098	(T/C)
15812	CakSNP15812	*Kabuli*	Ca_Kabuli_Scaffold553	310715	(A/G)
15813	CakSNP15813	*Kabuli*	Ca_Kabuli_Scaffold553	310759	(T/A)
15814	CakSNP15814	*Kabuli*	Ca_Kabuli_Scaffold553	402702	(T/C)
15815	CakSNP15815	*Kabuli*	Ca_Kabuli_Scaffold553	441845	(T/C)
15816	CakSNP15816	*Kabuli*	Ca_Kabuli_Scaffold553	441881	(T/C)
15817	CakSNP15817	*Kabuli*	Ca_Kabuli_Scaffold553	441898	(G/A)
15818	CakSNP15818	*Kabuli*	Ca_Kabuli_Scaffold553	550789	(G/C)
15819	CakSNP15819	*Kabuli*	Ca_Kabuli_Scaffold553	607503	(G/T)
15820	CakSNP15820	*Kabuli*	Ca_Kabuli_Scaffold553	607572	(T/C)
15821	CakSNP15821	*Kabuli*	Ca_Kabuli_Scaffold553	794383	(A/G)
15822	CakSNP15822	*Kabuli*	Ca_Kabuli_Scaffold553	794404	(G/A)
15823	CakSNP15823	*Kabuli*	Ca_Kabuli_Scaffold553	794431	(T/C)
15824	CakSNP15824	*Kabuli*	Ca_Kabuli_Scaffold553	794439	(G/T)
15825	CakSNP15825	*Kabuli*	Ca_Kabuli_Scaffold561	46046	(T/G)
15826	CakSNP15826	*Kabuli*	Ca_Kabuli_Scaffold561	49174	(G/A)
15827	CakSNP15827	*Kabuli*	Ca_Kabuli_Scaffold561	95414	(A/C)
15828	CakSNP15828	*Kabuli*	Ca_Kabuli_Scaffold561	95386	(G/A)
15829	CakSNP15829	*Kabuli*	Ca_Kabuli_Scaffold562	221706	(T/C)
15830	CakSNP15830	*Kabuli*	Ca_Kabuli_Scaffold562	221696	(T/G)
15831	CakSNP15831	*Kabuli*	Ca_Kabuli_Scaffold562	221686	(C/A)
15832	CakSNP15832	*Kabuli*	Ca_Kabuli_Scaffold562	221713	(G/A)
15833	CakSNP15833	*Kabuli*	Ca_Kabuli_Scaffold562	221714	(C/T)
15834	CakSNP15834	*Kabuli*	Ca_Kabuli_Scaffold562	221735	(G/A)
15835	CakSNP15835	*Kabuli*	Ca_Kabuli_Scaffold562	221795	(C/T)
15836	CakSNP15836	*Kabuli*	Ca_Kabuli_Scaffold562	221748	(C/A)
15837	CakSNP15837	*Kabuli*	Ca_Kabuli_Scaffold562	221758	(G/A)
15838	CakSNP15838	*Kabuli*	Ca_Kabuli_Scaffold562	221769	(T/A)
15839	CakSNP15839	*Kabuli*	Ca_Kabuli_Scaffold562	221778	(G/A)
15840	CakSNP15840	*Kabuli*	Ca_Kabuli_Scaffold562	221779	(G/A)
15841	CakSNP15841	*Kabuli*	Ca_Kabuli_Scaffold562	221788	(G/A)
15842	CakSNP15842	*Kabuli*	Ca_Kabuli_Scaffold562	221828	(T/A)
15843	CakSNP15843	*Kabuli*	Ca_Kabuli_Scaffold562	221792	(C/A)
15844	CakSNP15844	*Kabuli*	Ca_Kabuli_Scaffold562	255362	(G/A)
15845	CakSNP15845	*Kabuli*	Ca_Kabuli_Scaffold562	255295	(G/A)

S.N.	SNP IDs	Cultivars	Chromosomes/scaffolds	Physical positions (bp)	SNPs
15846	CakSNP15846	*Kabuli*	Ca_Kabuli_Scaffold562	289080	(C/A)
15847	CakSNP15847	*Kabuli*	Ca_Kabuli_Scaffold562	290071	(C/T)
15848	CakSNP15848	*Kabuli*	Ca_Kabuli_Scaffold5714	24882	(A/C)
15849	CakSNP15849	*Kabuli*	Ca_Kabuli_Scaffold5714	36359	(G/C)
15850	CakSNP15850	*Kabuli*	Ca_Kabuli_Scaffold5730	239	(G/A)
15851	CakSNP15851	*Kabuli*	Ca_Kabuli_Scaffold5730	192	(A/G)
15852	CakSNP15852	*Kabuli*	Ca_Kabuli_Scaffold5730	227	(G/A)
15853	CakSNP15853	*Kabuli*	Ca_Kabuli_Scaffold575	36084	(T/C)
15854	CakSNP15854	*Kabuli*	Ca_Kabuli_Scaffold575	36135	(T/C)
15855	CakSNP15855	*Kabuli*	Ca_Kabuli_Scaffold575	36238	(C/T)
15856	CakSNP15856	*Kabuli*	Ca_Kabuli_Scaffold575	45254	(C/T)
15857	CakSNP15857	*Kabuli*	Ca_Kabuli_Scaffold575	45250	(T/C)
15858	CakSNP15858	*Kabuli*	Ca_Kabuli_Scaffold575	45239	(G/T)
15859	CakSNP15859	*Kabuli*	Ca_Kabuli_Scaffold575	45236	(G/A)
15860	CakSNP15860	*Kabuli*	Ca_Kabuli_Scaffold575	45233	(G/A)
15861	CakSNP15861	*Kabuli*	Ca_Kabuli_Scaffold575	45215	(C/A)
15862	CakSNP15862	*Kabuli*	Ca_Kabuli_Scaffold575	45192	(C/T)
15863	CakSNP15863	*Kabuli*	Ca_Kabuli_Scaffold575	45279	(G/C)
15864	CakSNP15864	*Kabuli*	Ca_Kabuli_Scaffold575	45245	(G/A)
15865	CakSNP15865	*Kabuli*	Ca_Kabuli_Scaffold575	45237	(C/T)
15866	CakSNP15866	*Kabuli*	Ca_Kabuli_Scaffold575	45234	(C/T)
15867	CakSNP15867	*Kabuli*	Ca_Kabuli_Scaffold575	110772	(G/T)
15868	CakSNP15868	*Kabuli*	Ca_Kabuli_Scaffold575	110785	(C/T)
15869	CakSNP15869	*Kabuli*	Ca_Kabuli_Scaffold575	110765	(C/T)
15870	CakSNP15870	*Kabuli*	Ca_Kabuli_Scaffold575	110759	(C/T)
15871	CakSNP15871	*Kabuli*	Ca_Kabuli_Scaffold575	110753	(G/A)
15872	CakSNP15872	*Kabuli*	Ca_Kabuli_Scaffold575	117287	(T/G)
15873	CakSNP15873	*Kabuli*	Ca_Kabuli_Scaffold5792	65166	(A/C)
15874	CakSNP15874	*Kabuli*	Ca_Kabuli_Scaffold5792	86779	(C/A)
15875	CakSNP15875	*Kabuli*	Ca_Kabuli_Scaffold5792	86716	(T/C)
15876	CakSNP15876	*Kabuli*	Ca_Kabuli_Scaffold584	53203	(G/T)
15877	CakSNP15877	*Kabuli*	Ca_Kabuli_Scaffold589	72891	(T/G)
15878	CakSNP15878	*Kabuli*	Ca_Kabuli_Scaffold590	88429	(A/T)
15879	CakSNP15879	*Kabuli*	Ca_Kabuli_Scaffold590	88420	(T/C)
15880	CakSNP15880	*Kabuli*	Ca_Kabuli_Scaffold598	300825	(A/C)
15881	CakSNP15881	*Kabuli*	Ca_Kabuli_Scaffold598	300940	(C/T)
15882	CakSNP15882	*Kabuli*	Ca_Kabuli_Scaffold598	404254	(G/C)
15883	CakSNP15883	*Kabuli*	Ca_Kabuli_Scaffold598	404275	(G/T)

S.N.	SNP IDs	Cultivars	Chromosomes/scaffolds	Physical positions (bp)	SNPs
15884	CakSNP15884	*Kabuli*	Ca_Kabuli_Scaffold599	349605	(T/G)
15885	CakSNP15885	*Kabuli*	Ca_Kabuli_Scaffold5997	742	(G/C)
15886	CakSNP15886	*Kabuli*	Ca_Kabuli_Scaffold5997	679	(C/T)
15887	CakSNP15887	*Kabuli*	Ca_Kabuli_Scaffold601	55483	(A/G)
15888	CakSNP15888	*Kabuli*	Ca_Kabuli_Scaffold601	71998	(T/A)
15889	CakSNP15889	*Kabuli*	Ca_Kabuli_Scaffold605	23769	(A/C)
15890	CakSNP15890	*Kabuli*	Ca_Kabuli_Scaffold605	23773	(T/C)
15891	CakSNP15891	*Kabuli*	Ca_Kabuli_Scaffold605	23774	(A/G)
15892	CakSNP15892	*Kabuli*	Ca_Kabuli_Scaffold605	23775	(A/G)
15893	CakSNP15893	*Kabuli*	Ca_Kabuli_Scaffold605	23793	(A/G)
15894	CakSNP15894	*Kabuli*	Ca_Kabuli_Scaffold605	23795	(T/A)
15895	CakSNP15895	*Kabuli*	Ca_Kabuli_Scaffold605	23803	(A/G)
15896	CakSNP15896	*Kabuli*	Ca_Kabuli_Scaffold605	23808	(C/G)
15897	CakSNP15897	*Kabuli*	Ca_Kabuli_Scaffold605	23811	(G/A)
15898	CakSNP15898	*Kabuli*	Ca_Kabuli_Scaffold605	23816	(T/A)
15899	CakSNP15899	*Kabuli*	Ca_Kabuli_Scaffold605	84761	(A/C)
15900	CakSNP15900	*Kabuli*	Ca_Kabuli_Scaffold605	84765	(T/C)
15901	CakSNP15901	*Kabuli*	Ca_Kabuli_Scaffold605	84766	(A/G)
15902	CakSNP15902	*Kabuli*	Ca_Kabuli_Scaffold605	84767	(A/G)
15903	CakSNP15903	*Kabuli*	Ca_Kabuli_Scaffold605	84785	(A/G)
15904	CakSNP15904	*Kabuli*	Ca_Kabuli_Scaffold605	84787	(T/A)
15905	CakSNP15905	*Kabuli*	Ca_Kabuli_Scaffold605	84795	(A/G)
15906	CakSNP15906	*Kabuli*	Ca_Kabuli_Scaffold605	84800	(C/G)
15907	CakSNP15907	*Kabuli*	Ca_Kabuli_Scaffold605	84803	(G/A)
15908	CakSNP15908	*Kabuli*	Ca_Kabuli_Scaffold605	84807	(A/G)
15909	CakSNP15909	*Kabuli*	Ca_Kabuli_Scaffold62	46079	(C/T)
15910	CakSNP15910	*Kabuli*	Ca_Kabuli_Scaffold62	46085	(C/T)
15911	CakSNP15911	*Kabuli*	Ca_Kabuli_Scaffold62	46087	(G/A)
15912	CakSNP15912	*Kabuli*	Ca_Kabuli_Scaffold62	46088	(C/T)
15913	CakSNP15913	*Kabuli*	Ca_Kabuli_Scaffold624	254901	(C/T)
15914	CakSNP15914	*Kabuli*	Ca_Kabuli_Scaffold624	254950	(G/A)
15915	CakSNP15915	*Kabuli*	Ca_Kabuli_Scaffold624	254959	(T/C)
15916	CakSNP15916	*Kabuli*	Ca_Kabuli_Scaffold624	254971	(G/A)
15917	CakSNP15917	*Kabuli*	Ca_Kabuli_Scaffold624	254992	(C/T)
15918	CakSNP15918	*Kabuli*	Ca_Kabuli_Scaffold624	254942	(A/T)
15919	CakSNP15919	*Kabuli*	Ca_Kabuli_Scaffold6339	190	(T/G)
15920	CakSNP15920	*Kabuli*	Ca_Kabuli_Scaffold6367	6825	(G/T)
15921	CakSNP15921	*Kabuli*	Ca_Kabuli_Scaffold6367	10856	(T/G)

S.N.	SNP IDs	Cultivars	Chromosomes/scaffolds	Physical positions (bp)	SNPs
15922	CakSNP15922	*Kabuli*	Ca_Kabuli_Scaffold6367	10842	(T/A)
15923	CakSNP15923	*Kabuli*	Ca_Kabuli_Scaffold6367	13651	(G/A)
15924	CakSNP15924	*Kabuli*	Ca_Kabuli_Scaffold6367	23667	(G/A)
15925	CakSNP15925	*Kabuli*	Ca_Kabuli_Scaffold6367	25674	(T/C)
15926	CakSNP15926	*Kabuli*	Ca_Kabuli_Scaffold637	107671	(G/C)
15927	CakSNP15927	*Kabuli*	Ca_Kabuli_Scaffold637	107661	(A/C)
15928	CakSNP15928	*Kabuli*	Ca_Kabuli_Scaffold637	107636	(T/C)
15929	CakSNP15929	*Kabuli*	Ca_Kabuli_Scaffold637	107613	(C/G)
15930	CakSNP15930	*Kabuli*	Ca_Kabuli_Scaffold6403	1553	(T/A)
15931	CakSNP15931	*Kabuli*	Ca_Kabuli_Scaffold642	28715	(A/T)
15932	CakSNP15932	*Kabuli*	Ca_Kabuli_Scaffold642	615176	(T/C)
15933	CakSNP15933	*Kabuli*	Ca_Kabuli_Scaffold645	91908	(C/T)
15934	CakSNP15934	*Kabuli*	Ca_Kabuli_Scaffold653	145231	(A/G)
15935	CakSNP15935	*Kabuli*	Ca_Kabuli_Scaffold653	183054	(T/C)
15936	CakSNP15936	*Kabuli*	Ca_Kabuli_Scaffold653	183063	(G/A)
15937	CakSNP15937	*Kabuli*	Ca_Kabuli_Scaffold653	183069	(C/A)
15938	CakSNP15938	*Kabuli*	Ca_Kabuli_Scaffold653	183074	(C/G)
15939	CakSNP15939	*Kabuli*	Ca_Kabuli_Scaffold653	183078	(T/G)
15940	CakSNP15940	*Kabuli*	Ca_Kabuli_Scaffold653	183091	(T/C)
15941	CakSNP15941	*Kabuli*	Ca_Kabuli_Scaffold653	183143	(T/A)
15942	CakSNP15942	*Kabuli*	Ca_Kabuli_Scaffold653	183135	(T/C)
15943	CakSNP15943	*Kabuli*	Ca_Kabuli_Scaffold653	183112	(T/C)
15944	CakSNP15944	*Kabuli*	Ca_Kabuli_Scaffold653	189129	(C/T)
15945	CakSNP15945	*Kabuli*	Ca_Kabuli_Scaffold653	197878	(A/T)
15946	CakSNP15946	*Kabuli*	Ca_Kabuli_Scaffold661	180364	(G/T)
15947	CakSNP15947	*Kabuli*	Ca_Kabuli_Scaffold661	180362	(A/C)
15948	CakSNP15948	*Kabuli*	Ca_Kabuli_Scaffold661	180348	(C/T)
15949	CakSNP15949	*Kabuli*	Ca_Kabuli_Scaffold661	180302	(G/T)
15950	CakSNP15950	*Kabuli*	Ca_Kabuli_Scaffold661	311222	(G/T)
15951	CakSNP15951	*Kabuli*	Ca_Kabuli_Scaffold661	311182	(C/T)
15952	CakSNP15952	*Kabuli*	Ca_Kabuli_Scaffold663	16068	(G/A)
15953	CakSNP15953	*Kabuli*	Ca_Kabuli_Scaffold663	16042	(C/A)
15954	CakSNP15954	*Kabuli*	Ca_Kabuli_Scaffold663	16029	(G/A)
15955	CakSNP15955	*Kabuli*	Ca_Kabuli_Scaffold663	16056	(C/T)
15956	CakSNP15956	*Kabuli*	Ca_Kabuli_Scaffold663	16059	(G/A)
15957	CakSNP15957	*Kabuli*	Ca_Kabuli_Scaffold663	16061	(A/G)
15958	CakSNP15958	*Kabuli*	Ca_Kabuli_Scaffold663	156613	(G/C)
15959	CakSNP15959	*Kabuli*	Ca_Kabuli_Scaffold674	91979	(T/A)

S.N.	SNP IDs	Cultivars	Chromosomes/scaffolds	Physical positions (bp)	SNPs
15960	CakSNP15960	*Kabuli*	Ca_Kabuli_Scaffold674	360388	(C/T)
15961	CakSNP15961	*Kabuli*	Ca_Kabuli_Scaffold674	360401	(A/G)
15962	CakSNP15962	*Kabuli*	Ca_Kabuli_Scaffold674	360421	(G/A)
15963	CakSNP15963	*Kabuli*	Ca_Kabuli_Scaffold674	360406	(C/T)
15964	CakSNP15964	*Kabuli*	Ca_Kabuli_Scaffold674	360349	(G/A)
15965	CakSNP15965	*Kabuli*	Ca_Kabuli_Scaffold674	360464	(G/A)
15966	CakSNP15966	*Kabuli*	Ca_Kabuli_Scaffold674	360457	(G/C)
15967	CakSNP15967	*Kabuli*	Ca_Kabuli_Scaffold674	360411	(G/A)
15968	CakSNP15968	*Kabuli*	Ca_Kabuli_Scaffold674	360408	(G/T)
15969	CakSNP15969	*Kabuli*	Ca_Kabuli_Scaffold674	608623	(T/G)
15970	CakSNP15970	*Kabuli*	Ca_Kabuli_Scaffold674	652382	(G/A)
15971	CakSNP15971	*Kabuli*	Ca_Kabuli_Scaffold674	652373	(A/G)
15972	CakSNP15972	*Kabuli*	Ca_Kabuli_Scaffold674	826136	(T/C)
15973	CakSNP15973	*Kabuli*	Ca_Kabuli_Scaffold681	18376	(C/G)
15974	CakSNP15974	*Kabuli*	Ca_Kabuli_Scaffold682	157238	(G/C)
15975	CakSNP15975	*Kabuli*	Ca_Kabuli_Scaffold682	578200	(T/G)
15976	CakSNP15976	*Kabuli*	Ca_Kabuli_Scaffold684	78894	(G/A)
15977	CakSNP15977	*Kabuli*	Ca_Kabuli_Scaffold684	78883	(T/G)
15978	CakSNP15978	*Kabuli*	Ca_Kabuli_Scaffold684	78871	(C/G)
15979	CakSNP15979	*Kabuli*	Ca_Kabuli_Scaffold684	78842	(G/A)
15980	CakSNP15980	*Kabuli*	Ca_Kabuli_Scaffold684	78844	(A/G)
15981	CakSNP15981	*Kabuli*	Ca_Kabuli_Scaffold684	78864	(C/T)
15982	CakSNP15982	*Kabuli*	Ca_Kabuli_Scaffold684	78893	(C/T)
15983	CakSNP15983	*Kabuli*	Ca_Kabuli_Scaffold686	206111	(G/A)
15984	CakSNP15984	*Kabuli*	Ca_Kabuli_Scaffold686	206044	(T/C)
15985	CakSNP15985	*Kabuli*	Ca_Kabuli_Scaffold686	206042	(A/G)
15986	CakSNP15986	*Kabuli*	Ca_Kabuli_Scaffold702	247644	(G/C)
15987	CakSNP15987	*Kabuli*	Ca_Kabuli_Scaffold711	153694	(C/T)
15988	CakSNP15988	*Kabuli*	Ca_Kabuli_Scaffold711	153680	(C/A)
15989	CakSNP15989	*Kabuli*	Ca_Kabuli_Scaffold711	153667	(G/A)
15990	CakSNP15990	*Kabuli*	Ca_Kabuli_Scaffold711	153697	(G/A)
15991	CakSNP15991	*Kabuli*	Ca_Kabuli_Scaffold711	153699	(A/G)
15992	CakSNP15992	*Kabuli*	Ca_Kabuli_Scaffold711	153706	(G/A)
15993	CakSNP15993	*Kabuli*	Ca_Kabuli_Scaffold7127	1878	(C/T)
15994	CakSNP15994	*Kabuli*	Ca_Kabuli_Scaffold7144	234	(A/T)
15995	CakSNP15995	*Kabuli*	Ca_Kabuli_Scaffold716	128208	(G/T)
15996	CakSNP15996	*Kabuli*	Ca_Kabuli_Scaffold716	212326	(G/A)
15997	CakSNP15997	*Kabuli*	Ca_Kabuli_Scaffold716	212374	(G/A)

S.N.	SNP IDs	Cultivars	Chromosomes/scaffolds	Physical positions (bp)	SNPs
15998	CakSNP15998	*Kabuli*	Ca_Kabuli_Scaffold716	269220	(T/C)
15999	CakSNP15999	*Kabuli*	Ca_Kabuli_Scaffold716	269230	(A/C)
16000	CakSNP16000	*Kabuli*	Ca_Kabuli_Scaffold716	269242	(A/G)
16001	CakSNP16001	*Kabuli*	Ca_Kabuli_Scaffold716	269266	(G/T)
16002	CakSNP16002	*Kabuli*	Ca_Kabuli_Scaffold716	269271	(G/A)
16003	CakSNP16003	*Kabuli*	Ca_Kabuli_Scaffold716	269281	(T/G)
16004	CakSNP16004	*Kabuli*	Ca_Kabuli_Scaffold716	269287	(G/A)
16005	CakSNP16005	*Kabuli*	Ca_Kabuli_Scaffold716	269335	(G/A)
16006	CakSNP16006	*Kabuli*	Ca_Kabuli_Scaffold716	269332	(C/T)
16007	CakSNP16007	*Kabuli*	Ca_Kabuli_Scaffold716	269314	(G/A)
16008	CakSNP16008	*Kabuli*	Ca_Kabuli_Scaffold716	269299	(T/C)
16009	CakSNP16009	*Kabuli*	Ca_Kabuli_Scaffold719	11850	(C/T)
16010	CakSNP16010	*Kabuli*	Ca_Kabuli_Scaffold720	60880	(G/A)
16011	CakSNP16011	*Kabuli*	Ca_Kabuli_Scaffold720	370624	(T/G)
16012	CakSNP16012	*Kabuli*	Ca_Kabuli_Scaffold720	370639	(T/A)
16013	CakSNP16013	*Kabuli*	Ca_Kabuli_Scaffold720	370725	(G/A)
16014	CakSNP16014	*Kabuli*	Ca_Kabuli_Scaffold724	25019	(G/A)
16015	CakSNP16015	*Kabuli*	Ca_Kabuli_Scaffold724	25722	(G/C)
16016	CakSNP16016	*Kabuli*	Ca_Kabuli_Scaffold731	315093	(G/A)
16017	CakSNP16017	*Kabuli*	Ca_Kabuli_Scaffold731	315114	(C/T)
16018	CakSNP16018	*Kabuli*	Ca_Kabuli_Scaffold731	315135	(G/T)
16019	CakSNP16019	*Kabuli*	Ca_Kabuli_Scaffold731	315138	(C/T)
16020	CakSNP16020	*Kabuli*	Ca_Kabuli_Scaffold731	315141	(G/A)
16021	CakSNP16021	*Kabuli*	Ca_Kabuli_Scaffold731	332497	(G/A)
16022	CakSNP16022	*Kabuli*	Ca_Kabuli_Scaffold731	332468	(G/T)
16023	CakSNP16023	*Kabuli*	Ca_Kabuli_Scaffold731	332467	(G/C)
16024	CakSNP16024	*Kabuli*	Ca_Kabuli_Scaffold731	332465	(T/A)
16025	CakSNP16025	*Kabuli*	Ca_Kabuli_Scaffold731	332464	(G/T)
16026	CakSNP16026	*Kabuli*	Ca_Kabuli_Scaffold731	332458	(A/T)
16027	CakSNP16027	*Kabuli*	Ca_Kabuli_Scaffold731	332528	(G/C)
16028	CakSNP16028	*Kabuli*	Ca_Kabuli_Scaffold731	357674	(G/A)
16029	CakSNP16029	*Kabuli*	Ca_Kabuli_Scaffold731	357645	(G/T)
16030	CakSNP16030	*Kabuli*	Ca_Kabuli_Scaffold731	357644	(G/C)
16031	CakSNP16031	*Kabuli*	Ca_Kabuli_Scaffold731	357642	(T/A)
16032	CakSNP16032	*Kabuli*	Ca_Kabuli_Scaffold731	357641	(G/T)
16033	CakSNP16033	*Kabuli*	Ca_Kabuli_Scaffold731	357635	(A/T)
16034	CakSNP16034	*Kabuli*	Ca_Kabuli_Scaffold731	357715	(A/T)
16035	CakSNP16035	*Kabuli*	Ca_Kabuli_Scaffold731	362123	(G/A)

S.N.	SNP IDs	Cultivars	Chromosomes/scaffolds	Physical positions (bp)	SNPs
16036	CakSNP16036	*Kabuli*	Ca_Kabuli_Scaffold731	362118	(C/A)
16037	CakSNP16037	*Kabuli*	Ca_Kabuli_Scaffold731	362114	(A/C)
16038	CakSNP16038	*Kabuli*	Ca_Kabuli_Scaffold731	362548	(T/A)
16039	CakSNP16039	*Kabuli*	Ca_Kabuli_Scaffold731	362553	(C/T)
16040	CakSNP16040	*Kabuli*	Ca_Kabuli_Scaffold731	362554	(T/A)
16041	CakSNP16041	*Kabuli*	Ca_Kabuli_Scaffold731	362555	(T/G)
16042	CakSNP16042	*Kabuli*	Ca_Kabuli_Scaffold731	362556	(C/A)
16043	CakSNP16043	*Kabuli*	Ca_Kabuli_Scaffold731	362574	(T/C)
16044	CakSNP16044	*Kabuli*	Ca_Kabuli_Scaffold731	362581	(A/G)
16045	CakSNP16045	*Kabuli*	Ca_Kabuli_Scaffold731	362582	(T/C)
16046	CakSNP16046	*Kabuli*	Ca_Kabuli_Scaffold731	362594	(G/T)
16047	CakSNP16047	*Kabuli*	Ca_Kabuli_Scaffold731	362610	(C/T)
16048	CakSNP16048	*Kabuli*	Ca_Kabuli_Scaffold731	372255	(G/A)
16049	CakSNP16049	*Kabuli*	Ca_Kabuli_Scaffold731	372253	(G/T)
16050	CakSNP16050	*Kabuli*	Ca_Kabuli_Scaffold731	372250	(G/A)
16051	CakSNP16051	*Kabuli*	Ca_Kabuli_Scaffold731	372244	(C/T)
16052	CakSNP16052	*Kabuli*	Ca_Kabuli_Scaffold731	372243	(A/T)
16053	CakSNP16053	*Kabuli*	Ca_Kabuli_Scaffold731	372215	(A/G)
16054	CakSNP16054	*Kabuli*	Ca_Kabuli_Scaffold731	372209	(G/C)
16055	CakSNP16055	*Kabuli*	Ca_Kabuli_Scaffold731	372183	(C/T)
16056	CakSNP16056	*Kabuli*	Ca_Kabuli_Scaffold731	372959	(A/C)
16057	CakSNP16057	*Kabuli*	Ca_Kabuli_Scaffold7355	1606	(C/G)
16058	CakSNP16058	*Kabuli*	Ca_Kabuli_Scaffold7355	1690	(T/C)
16059	CakSNP16059	*Kabuli*	Ca_Kabuli_Scaffold7355	3608	(A/G)
16060	CakSNP16060	*Kabuli*	Ca_Kabuli_Scaffold7355	3602	(T/C)
16061	CakSNP16061	*Kabuli*	Ca_Kabuli_Scaffold7355	3578	(C/T)
16062	CakSNP16062	*Kabuli*	Ca_Kabuli_Scaffold7355	3572	(A/G)
16063	CakSNP16063	*Kabuli*	Ca_Kabuli_Scaffold7355	3566	(C/T)
16064	CakSNP16064	*Kabuli*	Ca_Kabuli_Scaffold752	382375	(G/A)
16065	CakSNP16065	*Kabuli*	Ca_Kabuli_Scaffold752	497452	(T/A)
16066	CakSNP16066	*Kabuli*	Ca_Kabuli_Scaffold752	497454	(A/G)
16067	CakSNP16067	*Kabuli*	Ca_Kabuli_Scaffold753	8312	(A/G)
16068	CakSNP16068	*Kabuli*	Ca_Kabuli_Scaffold758	16480	(G/T)
16069	CakSNP16069	*Kabuli*	Ca_Kabuli_Scaffold7650	2686	(C/T)
16070	CakSNP16070	*Kabuli*	Ca_Kabuli_Scaffold7715	3434	(A/T)
16071	CakSNP16071	*Kabuli*	Ca_Kabuli_Scaffold772	53192	(C/T)
16072	CakSNP16072	*Kabuli*	Ca_Kabuli_Scaffold772	53196	(C/T)
16073	CakSNP16073	*Kabuli*	Ca_Kabuli_Scaffold775	89238	(T/C)

S.N.	SNP IDs	Cultivars	Chromosomes/scaffolds	Physical positions (bp)	SNPs
16074	CakSNP16074	*Kabuli*	Ca_Kabuli_Scaffold775	89239	(C/T)
16075	CakSNP16075	*Kabuli*	Ca_Kabuli_Scaffold775	279828	(A/G)
16076	CakSNP16076	*Kabuli*	Ca_Kabuli_Scaffold775	356107	(C/A)
16077	CakSNP16077	*Kabuli*	Ca_Kabuli_Scaffold775	448604	(G/A)
16078	CakSNP16078	*Kabuli*	Ca_Kabuli_Scaffold775	448743	(T/C)
16079	CakSNP16079	*Kabuli*	Ca_Kabuli_Scaffold776	188906	(C/T)
16080	CakSNP16080	*Kabuli*	Ca_Kabuli_Scaffold776	188895	(C/T)
16081	CakSNP16081	*Kabuli*	Ca_Kabuli_Scaffold776	188888	(G/A)
16082	CakSNP16082	*Kabuli*	Ca_Kabuli_Scaffold7765	503	(C/T)
16083	CakSNP16083	*Kabuli*	Ca_Kabuli_Scaffold7765	536	(C/A)
16084	CakSNP16084	*Kabuli*	Ca_Kabuli_Scaffold7765	549	(G/A)
16085	CakSNP16085	*Kabuli*	Ca_Kabuli_Scaffold783	201617	(T/C)
16086	CakSNP16086	*Kabuli*	Ca_Kabuli_Scaffold783	244209	(T/C)
16087	CakSNP16087	*Kabuli*	Ca_Kabuli_Scaffold7842	727	(C/T)
16088	CakSNP16088	*Kabuli*	Ca_Kabuli_Scaffold7842	1078	(G/A)
16089	CakSNP16089	*Kabuli*	Ca_Kabuli_Scaffold7842	1075	(C/T)
16090	CakSNP16090	*Kabuli*	Ca_Kabuli_Scaffold7842	1068	(C/A)
16091	CakSNP16091	*Kabuli*	Ca_Kabuli_Scaffold7842	1042	(T/C)
16092	CakSNP16092	*Kabuli*	Ca_Kabuli_Scaffold7842	1024	(T/G)
16093	CakSNP16093	*Kabuli*	Ca_Kabuli_Scaffold787	4363	(A/G)
16094	CakSNP16094	*Kabuli*	Ca_Kabuli_Scaffold794	50197	(T/G)
16095	CakSNP16095	*Kabuli*	Ca_Kabuli_Scaffold794	50201	(T/G)
16096	CakSNP16096	*Kabuli*	Ca_Kabuli_Scaffold794	50225	(C/G)
16097	CakSNP16097	*Kabuli*	Ca_Kabuli_Scaffold794	50239	(G/A)
16098	CakSNP16098	*Kabuli*	Ca_Kabuli_Scaffold794	50240	(C/A)
16099	CakSNP16099	*Kabuli*	Ca_Kabuli_Scaffold794	50236	(A/G)
16100	CakSNP16100	*Kabuli*	Ca_Kabuli_Scaffold799	3956	(G/A)
16101	CakSNP16101	*Kabuli*	Ca_Kabuli_Scaffold799	3930	(A/G)
16102	CakSNP16102	*Kabuli*	Ca_Kabuli_Scaffold799	3928	(C/T)
16103	CakSNP16103	*Kabuli*	Ca_Kabuli_Scaffold801	67115	(C/T)
16104	CakSNP16104	*Kabuli*	Ca_Kabuli_Scaffold806	129263	(A/G)
16105	CakSNP16105	*Kabuli*	Ca_Kabuli_Scaffold806	129265	(G/T)
16106	CakSNP16106	*Kabuli*	Ca_Kabuli_Scaffold809	48678	(T/C)
16107	CakSNP16107	*Kabuli*	Ca_Kabuli_Scaffold809	48650	(C/A)
16108	CakSNP16108	*Kabuli*	Ca_Kabuli_Scaffold809	53417	(C/G)
16109	CakSNP16109	*Kabuli*	Ca_Kabuli_Scaffold809	53425	(T/C)
16110	CakSNP16110	*Kabuli*	Ca_Kabuli_Scaffold809	53431	(G/A)
16111	CakSNP16111	*Kabuli*	Ca_Kabuli_Scaffold809	53444	(G/A)

S.N.	SNP IDs	Cultivars	Chromosomes/scaffolds	Physical positions (bp)	SNPs
16112	CakSNP16112	*Kabuli*	Ca_Kabuli_Scaffold809	53474	(G/A)
16113	CakSNP16113	*Kabuli*	Ca_Kabuli_Scaffold809	53464	(G/A)
16114	CakSNP16114	*Kabuli*	Ca_Kabuli_Scaffold809	53470	(C/G)
16115	CakSNP16115	*Kabuli*	Ca_Kabuli_Scaffold811	81373	(C/T)
16116	CakSNP16116	*Kabuli*	Ca_Kabuli_Scaffold812	34686	(C/T)
16117	CakSNP16117	*Kabuli*	Ca_Kabuli_Scaffold812	246208	(A/C)
16118	CakSNP16118	*Kabuli*	Ca_Kabuli_Scaffold812	332746	(T/C)
16119	CakSNP16119	*Kabuli*	Ca_Kabuli_Scaffold812	332758	(T/C)
16120	CakSNP16120	*Kabuli*	Ca_Kabuli_Scaffold812	528815	(C/G)
16121	CakSNP16121	*Kabuli*	Ca_Kabuli_Scaffold8226	21624	(G/A)
16122	CakSNP16122	*Kabuli*	Ca_Kabuli_Scaffold8226	21592	(T/G)
16123	CakSNP16123	*Kabuli*	Ca_Kabuli_Scaffold8226	21590	(T/C)
16124	CakSNP16124	*Kabuli*	Ca_Kabuli_Scaffold8226	21572	(T/A)
16125	CakSNP16125	*Kabuli*	Ca_Kabuli_Scaffold842	13898	(T/C)
16126	CakSNP16126	*Kabuli*	Ca_Kabuli_Scaffold842	13905	(C/G)
16127	CakSNP16127	*Kabuli*	Ca_Kabuli_Scaffold842	13928	(G/T)
16128	CakSNP16128	*Kabuli*	Ca_Kabuli_Scaffold842	13946	(T/C)
16129	CakSNP16129	*Kabuli*	Ca_Kabuli_Scaffold845	28172	(G/T)
16130	CakSNP16130	*Kabuli*	Ca_Kabuli_Scaffold845	45557	(A/G)
16131	CakSNP16131	*Kabuli*	Ca_Kabuli_Scaffold845	71041	(A/G)
16132	CakSNP16132	*Kabuli*	Ca_Kabuli_Scaffold845	102612	(A/G)
16133	CakSNP16133	*Kabuli*	Ca_Kabuli_Scaffold845	129326	(C/G)
16134	CakSNP16134	*Kabuli*	Ca_Kabuli_Scaffold845	129370	(A/G)
16135	CakSNP16135	*Kabuli*	Ca_Kabuli_Scaffold845	130569	(G/C)
16136	CakSNP16136	*Kabuli*	Ca_Kabuli_Scaffold845	131911	(A/G)
16137	CakSNP16137	*Kabuli*	Ca_Kabuli_Scaffold845	136796	(A/G)
16138	CakSNP16138	*Kabuli*	Ca_Kabuli_Scaffold845	139268	(G/A)
16139	CakSNP16139	*Kabuli*	Ca_Kabuli_Scaffold845	154556	(T/C)
16140	CakSNP16140	*Kabuli*	Ca_Kabuli_Scaffold845	205934	(T/C)
16141	CakSNP16141	*Kabuli*	Ca_Kabuli_Scaffold848	37282	(G/A)
16142	CakSNP16142	*Kabuli*	Ca_Kabuli_Scaffold848	37309	(G/A)
16143	CakSNP16143	*Kabuli*	Ca_Kabuli_Scaffold848	39934	(T/G)
16144	CakSNP16144	*Kabuli*	Ca_Kabuli_Scaffold848	39968	(C/T)
16145	CakSNP16145	*Kabuli*	Ca_Kabuli_Scaffold848	39984	(G/T)
16146	CakSNP16146	*Kabuli*	Ca_Kabuli_Scaffold848	39985	(C/T)
16147	CakSNP16147	*Kabuli*	Ca_Kabuli_Scaffold848	39990	(A/G)
16148	CakSNP16148	*Kabuli*	Ca_Kabuli_Scaffold848	39994	(C/T)
16149	CakSNP16149	*Kabuli*	Ca_Kabuli_Scaffold848	89976	(C/A)

S.N.	SNP IDs	Cultivars	Chromosomes/scaffolds	Physical positions (bp)	SNPs
16150	CakSNP16150	*Kabuli*	Ca_Kabuli_Scaffold848	114078	(T/C)
16151	CakSNP16151	*Kabuli*	Ca_Kabuli_Scaffold848	158548	(T/C)
16152	CakSNP16152	*Kabuli*	Ca_Kabuli_Scaffold848	158557	(G/A)
16153	CakSNP16153	*Kabuli*	Ca_Kabuli_Scaffold848	158569	(C/T)
16154	CakSNP16154	*Kabuli*	Ca_Kabuli_Scaffold848	158570	(A/G)
16155	CakSNP16155	*Kabuli*	Ca_Kabuli_Scaffold848	158611	(C/T)
16156	CakSNP16156	*Kabuli*	Ca_Kabuli_Scaffold848	158638	(G/A)
16157	CakSNP16157	*Kabuli*	Ca_Kabuli_Scaffold848	158626	(G/T)
16158	CakSNP16158	*Kabuli*	Ca_Kabuli_Scaffold848	158594	(A/G)
16159	CakSNP16159	*Kabuli*	Ca_Kabuli_Scaffold857	5227	(G/A)
16160	CakSNP16160	*Kabuli*	Ca_Kabuli_Scaffold8586	4415	(A/G)
16161	CakSNP16161	*Kabuli*	Ca_Kabuli_Scaffold859	9382	(A/G)
16162	CakSNP16162	*Kabuli*	Ca_Kabuli_Scaffold8641	502	(G/A)
16163	CakSNP16163	*Kabuli*	Ca_Kabuli_Scaffold8641	472	(C/T)
16164	CakSNP16164	*Kabuli*	Ca_Kabuli_Scaffold8641	466	(C/T)
16165	CakSNP16165	*Kabuli*	Ca_Kabuli_Scaffold8641	460	(T/C)
16166	CakSNP16166	*Kabuli*	Ca_Kabuli_Scaffold8646	2056	(A/T)
16167	CakSNP16167	*Kabuli*	Ca_Kabuli_Scaffold8646	2073	(G/A)
16168	CakSNP16168	*Kabuli*	Ca_Kabuli_Scaffold8648	3938	(C/T)
16169	CakSNP16169	*Kabuli*	Ca_Kabuli_Scaffold8648	3977	(A/G)
16170	CakSNP16170	*Kabuli*	Ca_Kabuli_Scaffold8648	4058	(C/A)
16171	CakSNP16171	*Kabuli*	Ca_Kabuli_Scaffold8648	4050	(A/G)
16172	CakSNP16172	*Kabuli*	Ca_Kabuli_Scaffold8648	4036	(A/C)
16173	CakSNP16173	*Kabuli*	Ca_Kabuli_Scaffold8648	4013	(G/A)
16174	CakSNP16174	*Kabuli*	Ca_Kabuli_Scaffold8648	3992	(G/A)
16175	CakSNP16175	*Kabuli*	Ca_Kabuli_Scaffold866	27325	(C/T)
16176	CakSNP16176	*Kabuli*	Ca_Kabuli_Scaffold866	27293	(T/G)
16177	CakSNP16177	*Kabuli*	Ca_Kabuli_Scaffold866	27279	(C/T)
16178	CakSNP16178	*Kabuli*	Ca_Kabuli_Scaffold87	182677	(C/G)
16179	CakSNP16179	*Kabuli*	Ca_Kabuli_Scaffold87	182724	(G/A)
16180	CakSNP16180	*Kabuli*	Ca_Kabuli_Scaffold87	182769	(G/C)
16181	CakSNP16181	*Kabuli*	Ca_Kabuli_Scaffold87	182788	(A/C)
16182	CakSNP16182	*Kabuli*	Ca_Kabuli_Scaffold873	265386	(C/T)
16183	CakSNP16183	*Kabuli*	Ca_Kabuli_Scaffold873	265395	(C/A)
16184	CakSNP16184	*Kabuli*	Ca_Kabuli_Scaffold873	265396	(C/T)
16185	CakSNP16185	*Kabuli*	Ca_Kabuli_Scaffold873	265399	(C/A)
16186	CakSNP16186	*Kabuli*	Ca_Kabuli_Scaffold873	265458	(C/T)
16187	CakSNP16187	*Kabuli*	Ca_Kabuli_Scaffold873	265406	(T/C)

S.N.	SNP IDs	Cultivars	Chromosomes/scaffolds	Physical positions (bp)	SNPs
16188	CakSNP16188	*Kabuli*	Ca_Kabuli_Scaffold875	49179	(T/C)
16189	CakSNP16189	*Kabuli*	Ca_Kabuli_Scaffold875	51703	(C/G)
16190	CakSNP16190	*Kabuli*	Ca_Kabuli_Scaffold875	51702	(T/A)
16191	CakSNP16191	*Kabuli*	Ca_Kabuli_Scaffold875	51701	(T/C)
16192	CakSNP16192	*Kabuli*	Ca_Kabuli_Scaffold875	51714	(T/A)
16193	CakSNP16193	*Kabuli*	Ca_Kabuli_Scaffold875	51704	(C/A)
16194	CakSNP16194	*Kabuli*	Ca_Kabuli_Scaffold875	62738	(A/G)
16195	CakSNP16195	*Kabuli*	Ca_Kabuli_Scaffold876	152311	(G/T)
16196	CakSNP16196	*Kabuli*	Ca_Kabuli_Scaffold876	188605	(C/G)
16197	CakSNP16197	*Kabuli*	Ca_Kabuli_Scaffold876	297059	(T/C)
16198	CakSNP16198	*Kabuli*	Ca_Kabuli_Scaffold876	297024	(T/G)
16199	CakSNP16199	*Kabuli*	Ca_Kabuli_Scaffold876	297023	(G/A)
16200	CakSNP16200	*Kabuli*	Ca_Kabuli_Scaffold876	297022	(G/A)
16201	CakSNP16201	*Kabuli*	Ca_Kabuli_Scaffold876	297003	(C/T)
16202	CakSNP16202	*Kabuli*	Ca_Kabuli_Scaffold876	296995	(C/T)
16203	CakSNP16203	*Kabuli*	Ca_Kabuli_Scaffold877	9813	(G/A)
16204	CakSNP16204	*Kabuli*	Ca_Kabuli_Scaffold877	9851	(C/T)
16205	CakSNP16205	*Kabuli*	Ca_Kabuli_Scaffold88	9982	(C/A)
16206	CakSNP16206	*Kabuli*	Ca_Kabuli_Scaffold88	16398	(C/T)
16207	CakSNP16207	*Kabuli*	Ca_Kabuli_Scaffold88	110743	(A/G)
16208	CakSNP16208	*Kabuli*	Ca_Kabuli_Scaffold88	240551	(T/C)
16209	CakSNP16209	*Kabuli*	Ca_Kabuli_Scaffold88	240550	(G/A)
16210	CakSNP16210	*Kabuli*	Ca_Kabuli_Scaffold88	240525	(A/G)
16211	CakSNP16211	*Kabuli*	Ca_Kabuli_Scaffold88	274006	(A/G)
16212	CakSNP16212	*Kabuli*	Ca_Kabuli_Scaffold882	117376	(G/A)
16213	CakSNP16213	*Kabuli*	Ca_Kabuli_Scaffold882	118266	(G/T)
16214	CakSNP16214	*Kabuli*	Ca_Kabuli_Scaffold882	232549	(C/A)
16215	CakSNP16215	*Kabuli*	Ca_Kabuli_Scaffold882	232570	(T/G)
16216	CakSNP16216	*Kabuli*	Ca_Kabuli_Scaffold882	232605	(T/A)
16217	CakSNP16217	*Kabuli*	Ca_Kabuli_Scaffold882	358449	(T/A)
16218	CakSNP16218	*Kabuli*	Ca_Kabuli_Scaffold882	570871	(T/A)
16219	CakSNP16219	*Kabuli*	Ca_Kabuli_Scaffold882	571210	(A/T)
16220	CakSNP16220	*Kabuli*	Ca_Kabuli_Scaffold882	573367	(T/A)
16221	CakSNP16221	*Kabuli*	Ca_Kabuli_Scaffold882	573706	(A/T)
16222	CakSNP16222	*Kabuli*	Ca_Kabuli_Scaffold887_1	26025	(A/C)
16223	CakSNP16223	*Kabuli*	Ca_Kabuli_Scaffold887_1	26134	(C/A)
16224	CakSNP16224	*Kabuli*	Ca_Kabuli_Scaffold887_1	26121	(A/C)
16225	CakSNP16225	*Kabuli*	Ca_Kabuli_Scaffold887_1	26108	(G/T)

S.N.	SNP IDs	Cultivars	Chromosomes/scaffolds	Physical positions (bp)	SNPs
16226	CakSNP16226	*Kabuli*	Ca_Kabuli_Scaffold887_1	139386	(T/C)
16227	CakSNP16227	*Kabuli*	Ca_Kabuli_Scaffold887_1	139396	(G/C)
16228	CakSNP16228	*Kabuli*	Ca_Kabuli_Scaffold887_1	139580	(G/T)
16229	CakSNP16229	*Kabuli*	Ca_Kabuli_Scaffold887_1	144885	(T/C)
16230	CakSNP16230	*Kabuli*	Ca_Kabuli_Scaffold887_1	260488	(A/C)
16231	CakSNP16231	*Kabuli*	Ca_Kabuli_Scaffold892	24349	(T/C)
16232	CakSNP16232	*Kabuli*	Ca_Kabuli_Scaffold892	24323	(A/G)
16233	CakSNP16233	*Kabuli*	Ca_Kabuli_Scaffold892	24317	(G/T)
16234	CakSNP16234	*Kabuli*	Ca_Kabuli_Scaffold892	24316	(T/A)
16235	CakSNP16235	*Kabuli*	Ca_Kabuli_Scaffold892	35527	(T/C)
16236	CakSNP16236	*Kabuli*	Ca_Kabuli_Scaffold892	35501	(A/G)
16237	CakSNP16237	*Kabuli*	Ca_Kabuli_Scaffold892	35495	(G/T)
16238	CakSNP16238	*Kabuli*	Ca_Kabuli_Scaffold892	35494	(T/A)
16239	CakSNP16239	*Kabuli*	Ca_Kabuli_Scaffold892	35489	(A/G)
16240	CakSNP16240	*Kabuli*	Ca_Kabuli_Scaffold892	35514	(G/T)
16241	CakSNP16241	*Kabuli*	Ca_Kabuli_Scaffold895	18621	(C/A)
16242	CakSNP16242	*Kabuli*	Ca_Kabuli_Scaffold895	18693	(G/C)
16243	CakSNP16243	*Kabuli*	Ca_Kabuli_Scaffold895	20978	(T/C)
16244	CakSNP16244	*Kabuli*	Ca_Kabuli_Scaffold895	158250	(A/G)
16245	CakSNP16245	*Kabuli*	Ca_Kabuli_Scaffold901	91817	(C/T)
16246	CakSNP16246	*Kabuli*	Ca_Kabuli_Scaffold901	91849	(T/C)
16247	CakSNP16247	*Kabuli*	Ca_Kabuli_Scaffold908	15845	(C/A)
16248	CakSNP16248	*Kabuli*	Ca_Kabuli_Scaffold908	15841	(G/A)
16249	CakSNP16249	*Kabuli*	Ca_Kabuli_Scaffold908	15840	(G/A)
16250	CakSNP16250	*Kabuli*	Ca_Kabuli_Scaffold908	15816	(C/T)
16251	CakSNP16251	*Kabuli*	Ca_Kabuli_Scaffold908	15813	(G/T)
16252	CakSNP16252	*Kabuli*	Ca_Kabuli_Scaffold908	15810	(A/G)
16253	CakSNP16253	*Kabuli*	Ca_Kabuli_Scaffold908	15799	(C/T)
16254	CakSNP16254	*Kabuli*	Ca_Kabuli_Scaffold908	15786	(G/A)
16255	CakSNP16255	*Kabuli*	Ca_Kabuli_Scaffold908	15785	(G/A)
16256	CakSNP16256	*Kabuli*	Ca_Kabuli_Scaffold908	15822	(C/T)
16257	CakSNP16257	*Kabuli*	Ca_Kabuli_Scaffold908	15834	(C/A)
16258	CakSNP16258	*Kabuli*	Ca_Kabuli_Scaffold913	14282	(A/C)
16259	CakSNP16259	*Kabuli*	Ca_Kabuli_Scaffold913	332494	(G/C)
16260	CakSNP16260	*Kabuli*	Ca_Kabuli_Scaffold913	403513	(G/A)
16261	CakSNP16261	*Kabuli*	Ca_Kabuli_Scaffold913	403572	(C/G)
16262	CakSNP16262	*Kabuli*	Ca_Kabuli_Scaffold913	403553	(T/C)
16263	CakSNP16263	*Kabuli*	Ca_Kabuli_Scaffold913	403531	(A/C)

S.N.	SNP IDs	Cultivars	Chromosomes/scaffolds	Physical positions (bp)	SNPs
16264	CakSNP16264	*Kabuli*	Ca_Kabuli_Scaffold914	18826	(A/G)
16265	CakSNP16265	*Kabuli*	Ca_Kabuli_Scaffold914	18810	(C/A)
16266	CakSNP16266	*Kabuli*	Ca_Kabuli_Scaffold914	18809	(C/T)
16267	CakSNP16267	*Kabuli*	Ca_Kabuli_Scaffold914	18750	(C/A)
16268	CakSNP16268	*Kabuli*	Ca_Kabuli_Scaffold914	38792	(A/G)
16269	CakSNP16269	*Kabuli*	Ca_Kabuli_Scaffold914	38776	(C/A)
16270	CakSNP16270	*Kabuli*	Ca_Kabuli_Scaffold914	38775	(C/T)
16271	CakSNP16271	*Kabuli*	Ca_Kabuli_Scaffold914	38716	(C/A)
16272	CakSNP16272	*Kabuli*	Ca_Kabuli_Scaffold914	53125	(T/A)
16273	CakSNP16273	*Kabuli*	Ca_Kabuli_Scaffold914	53132	(C/T)
16274	CakSNP16274	*Kabuli*	Ca_Kabuli_Scaffold914	53270	(A/G)
16275	CakSNP16275	*Kabuli*	Ca_Kabuli_Scaffold916	1036	(A/G)
16276	CakSNP16276	*Kabuli*	Ca_Kabuli_Scaffold916	275497	(T/C)
16277	CakSNP16277	*Kabuli*	Ca_Kabuli_Scaffold916	275510	(A/T)
16278	CakSNP16278	*Kabuli*	Ca_Kabuli_Scaffold916	275512	(G/T)
16279	CakSNP16279	*Kabuli*	Ca_Kabuli_Scaffold916	275519	(A/C)
16280	CakSNP16280	*Kabuli*	Ca_Kabuli_Scaffold916	275493	(A/T)
16281	CakSNP16281	*Kabuli*	Ca_Kabuli_Scaffold919	119274	(T/C)
16282	CakSNP16282	*Kabuli*	Ca_Kabuli_Scaffold922	111921	(C/A)
16283	CakSNP16283	*Kabuli*	Ca_Kabuli_Scaffold922	121629	(T/C)
16284	CakSNP16284	*Kabuli*	Ca_Kabuli_Scaffold93	205677	(G/A)
16285	CakSNP16285	*Kabuli*	Ca_Kabuli_Scaffold93	506983	(C/A)
16286	CakSNP16286	*Kabuli*	Ca_Kabuli_Scaffold93	578138	(G/A)
16287	CakSNP16287	*Kabuli*	Ca_Kabuli_Scaffold93	578146	(A/G)
16288	CakSNP16288	*Kabuli*	Ca_Kabuli_Scaffold93	578158	(A/G)
16289	CakSNP16289	*Kabuli*	Ca_Kabuli_Scaffold93	640373	(A/C)
16290	CakSNP16290	*Kabuli*	Ca_Kabuli_Scaffold93	640389	(G/T)
16291	CakSNP16291	*Kabuli*	Ca_Kabuli_Scaffold93	640430	(C/T)
16292	CakSNP16292	*Kabuli*	Ca_Kabuli_Scaffold93	640487	(A/C)
16293	CakSNP16293	*Kabuli*	Ca_Kabuli_Scaffold93	640459	(G/T)
16294	CakSNP16294	*Kabuli*	Ca_Kabuli_Scaffold93	640448	(T/C)
16295	CakSNP16295	*Kabuli*	Ca_Kabuli_Scaffold93	640445	(C/T)
16296	CakSNP16296	*Kabuli*	Ca_Kabuli_Scaffold93	640444	(G/T)
16297	CakSNP16297	*Kabuli*	Ca_Kabuli_Scaffold93	645328	(A/C)
16298	CakSNP16298	*Kabuli*	Ca_Kabuli_Scaffold93	645344	(G/T)
16299	CakSNP16299	*Kabuli*	Ca_Kabuli_Scaffold93	645442	(A/C)
16300	CakSNP16300	*Kabuli*	Ca_Kabuli_Scaffold93	645414	(G/T)
16301	CakSNP16301	*Kabuli*	Ca_Kabuli_Scaffold93	645403	(T/C)

S.N.	SNP IDs	Cultivars	Chromosomes/scaffolds	Physical positions (bp)	SNPs
16302	CakSNP16302	*Kabuli*	Ca_Kabuli_Scaffold93	645400	(C/T)
16303	CakSNP16303	*Kabuli*	Ca_Kabuli_Scaffold93	645399	(G/T)
16304	CakSNP16304	*Kabuli*	Ca_Kabuli_Scaffold93	645402	(G/A)
16305	CakSNP16305	*Kabuli*	Ca_Kabuli_Scaffold93	651020	(C/T)
16306	CakSNP16306	*Kabuli*	Ca_Kabuli_Scaffold93	651018	(C/T)
16307	CakSNP16307	*Kabuli*	Ca_Kabuli_Scaffold93	651009	(A/G)
16308	CakSNP16308	*Kabuli*	Ca_Kabuli_Scaffold93	650981	(T/C)
16309	CakSNP16309	*Kabuli*	Ca_Kabuli_Scaffold93	650968	(G/A)
16310	CakSNP16310	*Kabuli*	Ca_Kabuli_Scaffold93	650958	(G/A)
16311	CakSNP16311	*Kabuli*	Ca_Kabuli_Scaffold93	650955	(T/C)
16312	CakSNP16312	*Kabuli*	Ca_Kabuli_Scaffold931	13212	(T/C)
16313	CakSNP16313	*Kabuli*	Ca_Kabuli_Scaffold9323	5263	(A/G)
16314	CakSNP16314	*Kabuli*	Ca_Kabuli_Scaffold937	132087	(C/G)
16315	CakSNP16315	*Kabuli*	Ca_Kabuli_Scaffold937	134580	(C/G)
16316	CakSNP16316	*Kabuli*	Ca_Kabuli_Scaffold937	134707	(G/A)
16317	CakSNP16317	*Kabuli*	Ca_Kabuli_Scaffold948	12188	(C/T)
16318	CakSNP16318	*Kabuli*	Ca_Kabuli_Scaffold948	21152	(C/T)
16319	CakSNP16319	*Kabuli*	Ca_Kabuli_Scaffold953	42329	(T/C)
16320	CakSNP16320	*Kabuli*	Ca_Kabuli_Scaffold953	57413	(A/T)
16321	CakSNP16321	*Kabuli*	Ca_Kabuli_Scaffold956	21441	(C/A)
16322	CakSNP16322	*Kabuli*	Ca_Kabuli_Scaffold956	27612	(A/G)
16323	CakSNP16323	*Kabuli*	Ca_Kabuli_Scaffold956	27710	(T/G)
16324	CakSNP16324	*Kabuli*	Ca_Kabuli_Scaffold956	27693	(T/C)
16325	CakSNP16325	*Kabuli*	Ca_Kabuli_Scaffold956	28015	(A/C)
16326	CakSNP16326	*Kabuli*	Ca_Kabuli_Scaffold956	28014	(T/A)
16327	CakSNP16327	*Kabuli*	Ca_Kabuli_Scaffold956	29736	(T/A)
16328	CakSNP16328	*Kabuli*	Ca_Kabuli_Scaffold959	56173	(C/T)
16329	CakSNP16329	*Kabuli*	Ca_Kabuli_Scaffold959	56159	(G/A)
16330	CakSNP16330	*Kabuli*	Ca_Kabuli_Scaffold959	56156	(G/A)
16331	CakSNP16331	*Kabuli*	Ca_Kabuli_Scaffold959	56117	(G/A)
16332	CakSNP16332	*Kabuli*	Ca_Kabuli_Scaffold962	149524	(C/T)
16333	CakSNP16333	*Kabuli*	Ca_Kabuli_Scaffold962	149545	(C/T)
16334	CakSNP16334	*Kabuli*	Ca_Kabuli_Scaffold962	149580	(G/A)
16335	CakSNP16335	*Kabuli*	Ca_Kabuli_Scaffold962	149585	(A/G)
16336	CakSNP16336	*Kabuli*	Ca_Kabuli_Scaffold962	149645	(G/T)
16337	CakSNP16337	*Kabuli*	Ca_Kabuli_Scaffold962	149631	(G/A)
16338	CakSNP16338	*Kabuli*	Ca_Kabuli_Scaffold962	149589	(G/A)
16339	CakSNP16339	*Kabuli*	Ca_Kabuli_Scaffold962	149583	(G/A)

S.N.	SNP IDs	Cultivars	Chromosomes/scaffolds	Physical positions (bp)	SNPs
16340	CakSNP16340	*Kabuli*	Ca_Kabuli_Scaffold962	149577	(G/T)
16341	CakSNP16341	*Kabuli*	Ca_Kabuli_Scaffold962	149689	(G/A)
16342	CakSNP16342	*Kabuli*	Ca_Kabuli_Scaffold962	149687	(A/T)
16343	CakSNP16343	*Kabuli*	Ca_Kabuli_Scaffold962	149676	(T/C)
16344	CakSNP16344	*Kabuli*	Ca_Kabuli_Scaffold962	149661	(G/A)
16345	CakSNP16345	*Kabuli*	Ca_Kabuli_Scaffold962	149649	(C/T)
16346	CakSNP16346	*Kabuli*	Ca_Kabuli_Scaffold962	149644	(C/T)
16347	CakSNP16347	*Kabuli*	Ca_Kabuli_Scaffold962	149628	(G/A)
16348	CakSNP16348	*Kabuli*	Ca_Kabuli_Scaffold962	149613	(G/T)
16349	CakSNP16349	*Kabuli*	Ca_Kabuli_Scaffold963	77099	(C/T)
16350	CakSNP16350	*Kabuli*	Ca_Kabuli_Scaffold98	287956	(T/A)
16351	CakSNP16351	*Kabuli*	Ca_Kabuli_Scaffold98	287933	(T/C)
16352	CakSNP16352	*Kabuli*	Ca_Kabuli_Scaffold98	388565	(T/C)
16353	CakSNP16353	*Kabuli*	Ca_Kabuli_Scaffold98	434956	(A/G)
16354	CakSNP16354	*Kabuli*	Ca_Kabuli_Scaffold98	440243	(G/C)
16355	CakSNP16355	*Kabuli*	Ca_Kabuli_Scaffold98	447314	(G/C)
16356	CakSNP16356	*Kabuli*	Ca_Kabuli_Scaffold98	447311	(G/A)
16357	CakSNP16357	*Kabuli*	Ca_Kabuli_Scaffold98	485305	(G/C)
16358	CakSNP16358	*Kabuli*	Ca_Kabuli_Scaffold98	485302	(G/A)
16359	CakSNP16359	*Kabuli*	Ca_Kabuli_Scaffold98	533710	(T/C)
16360	CakSNP16360	*Kabuli*	Ca_Kabuli_Scaffold98	533677	(T/A)
16361	CakSNP16361	*Kabuli*	Ca_Kabuli_Scaffold98	533812	(G/C)
16362	CakSNP16362	*Kabuli*	Ca_Kabuli_Scaffold98	562979	(G/A)
16363	CakSNP16363	*Kabuli*	Ca_Kabuli_Scaffold98	563016	(T/C)
16364	CakSNP16364	*Kabuli*	Ca_Kabuli_Scaffold98	563048	(A/C)
16365	CakSNP16365	*Kabuli*	Ca_Kabuli_Scaffold981	84091	(G/A)
16366	CakSNP16366	*Kabuli*	Ca_Kabuli_Scaffold981	84088	(C/G)
16367	CakSNP16367	*Kabuli*	Ca_Kabuli_Scaffold981	84087	(C/A)
16368	CakSNP16368	*Kabuli*	Ca_Kabuli_Scaffold981	84072	(G/A)
16369	CakSNP16369	*Kabuli*	Ca_Kabuli_Scaffold981	84057	(A/G)
16370	CakSNP16370	*Kabuli*	Ca_Kabuli_Scaffold981	84034	(C/T)
16371	CakSNP16371	*Kabuli*	Ca_Kabuli_Scaffold981	84026	(G/C)
16372	CakSNP16372	*Kabuli*	Ca_Kabuli_Scaffold981	84019	(C/T)
16373	CakSNP16373	*Kabuli*	Ca_Kabuli_Scaffold981	84007	(C/G)
16374	CakSNP16374	*Kabuli*	Ca_Kabuli_Scaffold999	77761	(A/T)
16375	CakSNP16375	*Kabuli*	Ca_Kabuli_Scaffold999	77763	(C/T)
16376	CakSNP16376	*Kabuli*	Ca_Kabuli_Scaffold999	77767	(A/T)
16377	CakSNP16377	*Kabuli*	Ca_Kabuli_denovo	1061	(T/A)

S.N.	SNP IDs	Cultivars	Chromosomes/scaffolds	Physical positions (bp)	SNPs
16378	CakSNP16378	*Kabuli*	Ca_Kabuli_denovo	1965	(G/T)
16379	CakSNP16379	*Kabuli*	Ca_Kabuli_denovo	1975	(G/A)
16380	CakSNP16380	*Kabuli*	Ca_Kabuli_denovo	2003	(A/G)
16381	CakSNP16381	*Kabuli*	Ca_Kabuli_denovo	2012	(T/G)
16382	CakSNP16382	*Kabuli*	Ca_Kabuli_denovo	2047	(T/G)
16383	CakSNP16383	*Kabuli*	Ca_Kabuli_denovo	3355	(C/A)
16384	CakSNP16384	*Kabuli*	Ca_Kabuli_denovo	3357	(T/G)
16385	CakSNP16385	*Kabuli*	Ca_Kabuli_denovo	3369	(C/G)
16386	CakSNP16386	*Kabuli*	Ca_Kabuli_denovo	3376	(C/G)
16387	CakSNP16387	*Kabuli*	Ca_Kabuli_denovo	3391	(A/G)
16388	CakSNP16388	*Kabuli*	Ca_Kabuli_denovo	3398	(A/G)
16389	CakSNP16389	*Kabuli*	Ca_Kabuli_denovo	3408	(C/G)
16390	CakSNP16390	*Kabuli*	Ca_Kabuli_denovo	3410	(G/T)
16391	CakSNP16391	*Kabuli*	Ca_Kabuli_denovo	3417	(G/A)
16392	CakSNP16392	*Kabuli*	Ca_Kabuli_denovo	3427	(G/C)
16393	CakSNP16393	*Kabuli*	Ca_Kabuli_denovo	3439	(C/G)
16394	CakSNP16394	*Kabuli*	Ca_Kabuli_denovo	3710	(A/T)
16395	CakSNP16395	*Kabuli*	Ca_Kabuli_denovo	3711	(T/G)
16396	CakSNP16396	*Kabuli*	Ca_Kabuli_denovo	3712	(G/C)
16397	CakSNP16397	*Kabuli*	Ca_Kabuli_denovo	3714	(C/G)
16398	CakSNP16398	*Kabuli*	Ca_Kabuli_denovo	3715	(A/G)
16399	CakSNP16399	*Kabuli*	Ca_Kabuli_denovo	3716	(G/A)
16400	CakSNP16400	*Kabuli*	Ca_Kabuli_denovo	3717	(A/G)
16401	CakSNP16401	*Kabuli*	Ca_Kabuli_denovo	3720	(G/T)
16402	CakSNP16402	*Kabuli*	Ca_Kabuli_denovo	3721	(A/C)
16403	CakSNP16403	*Kabuli*	Ca_Kabuli_denovo	3967	(G/A)
16404	CakSNP16404	*Kabuli*	Ca_Kabuli_denovo	3968	(G/A)
16405	CakSNP16405	*Kabuli*	Ca_Kabuli_denovo	3969	(A/G)
16406	CakSNP16406	*Kabuli*	Ca_Kabuli_denovo	3970	(A/G)
16407	CakSNP16407	*Kabuli*	Ca_Kabuli_denovo	3971	(G/A)
16408	CakSNP16408	*Kabuli*	Ca_Kabuli_denovo	3972	(C/A)
16409	CakSNP16409	*Kabuli*	Ca_Kabuli_denovo	3975	(T/G)
16410	CakSNP16410	*Kabuli*	Ca_Kabuli_denovo	4061	(G/A)
16411	CakSNP16411	*Kabuli*	Ca_Kabuli_denovo	4063	(G/A)
16412	CakSNP16412	*Kabuli*	Ca_Kabuli_denovo	4064	(A/G)
16413	CakSNP16413	*Kabuli*	Ca_Kabuli_denovo	4065	(G/C)
16414	CakSNP16414	*Kabuli*	Ca_Kabuli_denovo	4066	(C/G)
16415	CakSNP16415	*Kabuli*	Ca_Kabuli_denovo	4068	(T/G)

S.N.	SNP IDs	Cultivars	Chromosomes/scaffolds	Physical positions (bp)	SNPs
16416	CakSNP16416	*Kabuli*	Ca_Kabuli_denovo	4287	(C/G)
16417	CakSNP16417	*Kabuli*	Ca_Kabuli_denovo	4288	(G/A)
16418	CakSNP16418	*Kabuli*	Ca_Kabuli_denovo	4295	(A/G)
16419	CakSNP16419	*Kabuli*	Ca_Kabuli_denovo	4299	(T/C)
16420	CakSNP16420	*Kabuli*	Ca_Kabuli_denovo	4724	(A/G)
16421	CakSNP16421	*Kabuli*	Ca_Kabuli_denovo	4725	(G/C)
16422	CakSNP16422	*Kabuli*	Ca_Kabuli_denovo	4727	(G/A)
16423	CakSNP16423	*Kabuli*	Ca_Kabuli_denovo	4728	(A/G)
16424	CakSNP16424	*Kabuli*	Ca_Kabuli_denovo	4731	(C/A)
16425	CakSNP16425	*Kabuli*	Ca_Kabuli_denovo	4732	(C/T)
16426	CakSNP16426	*Kabuli*	Ca_Kabuli_denovo	5106	(C/G)
16427	CakSNP16427	*Kabuli*	Ca_Kabuli_denovo	5108	(T/G)
16428	CakSNP16428	*Kabuli*	Ca_Kabuli_denovo	5349	(T/G)
16429	CakSNP16429	*Kabuli*	Ca_Kabuli_denovo	5394	(C/G)
16430	CakSNP16430	*Kabuli*	Ca_Kabuli_denovo	5650	(C/G)
16431	CakSNP16431	*Kabuli*	Ca_Kabuli_denovo	5652	(G/T)
16432	CakSNP16432	*Kabuli*	Ca_Kabuli_denovo	5654	(C/T)
16433	CakSNP16433	*Kabuli*	Ca_Kabuli_denovo	5655	(A/C)
16434	CakSNP16434	*Kabuli*	Ca_Kabuli_denovo	5746	(G/C)
16435	CakSNP16435	*Kabuli*	Ca_Kabuli_denovo	5747	(C/G)
16436	CakSNP16436	*Kabuli*	Ca_Kabuli_denovo	5749	(G/T)
16437	CakSNP16437	*Kabuli*	Ca_Kabuli_denovo	5751	(C/T)
16438	CakSNP16438	*Kabuli*	Ca_Kabuli_denovo	5752	(A/C)
16439	CakSNP16439	*Kabuli*	Ca_Kabuli_denovo	5851	(C/G)
16440	CakSNP16440	*Kabuli*	Ca_Kabuli_denovo	5853	(G/T)
16441	CakSNP16441	*Kabuli*	Ca_Kabuli_denovo	5855	(C/T)
16442	CakSNP16442	*Kabuli*	Ca_Kabuli_denovo	5856	(A/C)
16443	CakSNP16443	*Kabuli*	Ca_Kabuli_denovo	6702	(T/G)
16444	CakSNP16444	*Kabuli*	Ca_Kabuli_denovo	6708	(C/G)
16445	CakSNP16445	*Kabuli*	Ca_Kabuli_denovo	6727	(A/G)
16446	CakSNP16446	*Kabuli*	Ca_Kabuli_denovo	6728	(C/G)
16447	CakSNP16447	*Kabuli*	Ca_Kabuli_denovo	6739	(T/G)
16448	CakSNP16448	*Kabuli*	Ca_Kabuli_denovo	6761	(T/G)
16449	CakSNP16449	*Kabuli*	Ca_Kabuli_denovo	6773	(T/G)
16450	CakSNP16450	*Kabuli*	Ca_Kabuli_denovo	7246	(C/A)
16451	CakSNP16451	*Kabuli*	Ca_Kabuli_denovo	7247	(G/C)
16452	CakSNP16452	*Kabuli*	Ca_Kabuli_denovo	7248	(A/C)
16453	CakSNP16453	*Kabuli*	Ca_Kabuli_denovo	7251	(C/T)

S.N.	SNP IDs	Cultivars	Chromosomes/scaffolds	Physical positions (bp)	SNPs
16454	CakSNP16454	*Kabuli*	Ca_Kabuli_denovo	7253	(T/G)
16455	CakSNP16455	*Kabuli*	Ca_Kabuli_denovo	7254	(C/A)
16456	CakSNP16456	*Kabuli*	Ca_Kabuli_denovo	7255	(G/T)
16457	CakSNP16457	*Kabuli*	Ca_Kabuli_denovo	7380	(A/T)
16458	CakSNP16458	*Kabuli*	Ca_Kabuli_denovo	7690	(A/G)
16459	CakSNP16459	*Kabuli*	Ca_Kabuli_denovo	7691	(C/G)
16460	CakSNP16460	*Kabuli*	Ca_Kabuli_denovo	7883	(G/T)
16461	CakSNP16461	*Kabuli*	Ca_Kabuli_denovo	7937	(C/G)
16462	CakSNP16462	*Kabuli*	Ca_Kabuli_denovo	7944	(T/A)
16463	CakSNP16463	*Kabuli*	Ca_Kabuli_denovo	7976	(T/G)
16464	CakSNP16464	*Kabuli*	Ca_Kabuli_denovo	7999	(T/C)
16465	CakSNP16465	*Kabuli*	Ca_Kabuli_denovo	8051	(C/A)
16466	CakSNP16466	*Kabuli*	Ca_Kabuli_denovo	8069	(T/G)
16467	CakSNP16467	*Kabuli*	Ca_Kabuli_denovo	8088	(C/A)
16468	CakSNP16468	*Kabuli*	Ca_Kabuli_denovo	8136	(A/G)
16469	CakSNP16469	*Kabuli*	Ca_Kabuli_denovo	8162	(T/G)
16470	CakSNP16470	*Kabuli*	Ca_Kabuli_denovo	8537	(A/G)
16471	CakSNP16471	*Kabuli*	Ca_Kabuli_denovo	8539	(A/C)
16472	CakSNP16472	*Kabuli*	Ca_Kabuli_denovo	8541	(T/G)
16473	CakSNP16473	*Kabuli*	Ca_Kabuli_denovo	9009	(G/A)
16474	CakSNP16474	*Kabuli*	Ca_Kabuli_denovo	9010	(A/G)
16475	CakSNP16475	*Kabuli*	Ca_Kabuli_denovo	9011	(A/G)
16476	CakSNP16476	*Kabuli*	Ca_Kabuli_denovo	9012	(C/A)
16477	CakSNP16477	*Kabuli*	Ca_Kabuli_denovo	11242	(C/G)
16478	CakSNP16478	*Kabuli*	Ca_Kabuli_denovo	11244	(T/G)
16479	CakSNP16479	*Kabuli*	Ca_Kabuli_denovo	11246	(C/T)
16480	CakSNP16480	*Kabuli*	Ca_Kabuli_denovo	11711	(G/T)
16481	CakSNP16481	*Kabuli*	Ca_Kabuli_denovo	12623	(A/G)
16482	CakSNP16482	*Kabuli*	Ca_Kabuli_denovo	12624	(G/C)
16483	CakSNP16483	*Kabuli*	Ca_Kabuli_denovo	12625	(C/G)
16484	CakSNP16484	*Kabuli*	Ca_Kabuli_denovo	12627	(T/G)
16485	CakSNP16485	*Kabuli*	Ca_Kabuli_denovo	12629	(C/T)
16486	CakSNP16486	*Kabuli*	Ca_Kabuli_denovo	12630	(A/C)
16487	CakSNP16487	*Kabuli*	Ca_Kabuli_denovo	13473	(G/A)
16488	CakSNP16488	*Kabuli*	Ca_Kabuli_denovo	13475	(G/A)
16489	CakSNP16489	*Kabuli*	Ca_Kabuli_denovo	13476	(A/G)
16490	CakSNP16490	*Kabuli*	Ca_Kabuli_denovo	14124	(A/C)
16491	CakSNP16491	*Kabuli*	Ca_Kabuli_denovo	14560	(C/A)

S.N.	SNP IDs	Cultivars	Chromosomes/scaffolds	Physical positions (bp)	SNPs
16492	CakSNP16492	*Kabuli*	Ca_Kabuli_denovo	14672	(G/A)
16493	CakSNP16493	*Kabuli*	Ca_Kabuli_denovo	14674	(G/A)
16494	CakSNP16494	*Kabuli*	Ca_Kabuli_denovo	15543	(G/A)
16495	CakSNP16495	*Kabuli*	Ca_Kabuli_denovo	15553	(C/A)
16496	CakSNP16496	*Kabuli*	Ca_Kabuli_denovo	15580	(G/A)
16497	CakSNP16497	*Kabuli*	Ca_Kabuli_denovo	17223	(C/G)
16498	CakSNP16498	*Kabuli*	Ca_Kabuli_denovo	17468	(A/G)
16499	CakSNP16499	*Kabuli*	Ca_Kabuli_denovo	17469	(G/C)
16500	CakSNP16500	*Kabuli*	Ca_Kabuli_denovo	17470	(C/G)
16501	CakSNP16501	*Kabuli*	Ca_Kabuli_denovo	17472	(T/G)
16502	CakSNP16502	*Kabuli*	Ca_Kabuli_denovo	17733	(G/C)
16503	CakSNP16503	*Kabuli*	Ca_Kabuli_denovo	17734	(C/G)
16504	CakSNP16504	*Kabuli*	Ca_Kabuli_denovo	17736	(T/G)
16505	CakSNP16505	*Kabuli*	Ca_Kabuli_denovo	17738	(C/T)
16506	CakSNP16506	*Kabuli*	Ca_Kabuli_denovo	18017	(G/A)
16507	CakSNP16507	*Kabuli*	Ca_Kabuli_denovo	18019	(G/A)
16508	CakSNP16508	*Kabuli*	Ca_Kabuli_denovo	18050	(G/C)
16509	CakSNP16509	*Kabuli*	Ca_Kabuli_denovo	18291	(C/G)
16510	CakSNP16510	*Kabuli*	Ca_Kabuli_denovo	18293	(G/T)
16511	CakSNP16511	*Kabuli*	Ca_Kabuli_denovo	18295	(C/T)
16512	CakSNP16512	*Kabuli*	Ca_Kabuli_denovo	18296	(A/C)
16513	CakSNP16513	*Kabuli*	Ca_Kabuli_denovo	18297	(G/A)
16514	CakSNP16514	*Kabuli*	Ca_Kabuli_denovo	18394	(T/G)
16515	CakSNP16515	*Kabuli*	Ca_Kabuli_denovo	18450	(G/A)
16516	CakSNP16516	*Kabuli*	Ca_Kabuli_denovo	19005	(C/T)
16517	CakSNP16517	*Kabuli*	Ca_Kabuli_denovo	19041	(T/C)
16518	CakSNP16518	*Kabuli*	Ca_Kabuli_denovo	19048	(G/C)
16519	CakSNP16519	*Kabuli*	Ca_Kabuli_denovo	20080	(C/G)
16520	CakSNP16520	*Kabuli*	Ca_Kabuli_denovo	20081	(G/A)
16521	CakSNP16521	*Kabuli*	Ca_Kabuli_denovo	20082	(A/T)
16522	CakSNP16522	*Kabuli*	Ca_Kabuli_denovo	20083	(G/C)
16523	CakSNP16523	*Kabuli*	Ca_Kabuli_denovo	20084	(T/A)
16524	CakSNP16524	*Kabuli*	Ca_Kabuli_denovo	20086	(G/C)
16525	CakSNP16525	*Kabuli*	Ca_Kabuli_denovo	20087	(T/G)
16526	CakSNP16526	*Kabuli*	Ca_Kabuli_denovo	21257	(C/A)
16527	CakSNP16527	*Kabuli*	Ca_Kabuli_denovo	22000	(T/G)
16528	CakSNP16528	*Kabuli*	Ca_Kabuli_denovo	22013	(A/G)
16529	CakSNP16529	*Kabuli*	Ca_Kabuli_denovo	22039	(G/T)

S.N.	SNP IDs	Cultivars	Chromosomes/scaffolds	Physical positions (bp)	SNPs
16530	CakSNP16530	*Kabuli*	Ca_Kabuli_denovo	22242	(A/G)
16531	CakSNP16531	*Kabuli*	Ca_Kabuli_denovo	22320	(T/A)
16532	CakSNP16532	*Kabuli*	Ca_Kabuli_denovo	27708	(C/G)
16533	CakSNP16533	*Kabuli*	Ca_Kabuli_denovo	27709	(G/A)
16534	CakSNP16534	*Kabuli*	Ca_Kabuli_denovo	27710	(G/A)
16535	CakSNP16535	*Kabuli*	Ca_Kabuli_denovo	27711	(A/G)
16536	CakSNP16536	*Kabuli*	Ca_Kabuli_denovo	27712	(C/A)
16537	CakSNP16537	*Kabuli*	Ca_Kabuli_denovo	27714	(G/C)
16538	CakSNP16538	*Kabuli*	Ca_Kabuli_denovo	27715	(A/G)
16539	CakSNP16539	*Kabuli*	Ca_Kabuli_denovo	28816	(G/T)
16540	CakSNP16540	*Kabuli*	Ca_Kabuli_denovo	28818	(C/T)
16541	CakSNP16541	*Kabuli*	Ca_Kabuli_denovo	29384	(A/G)
16542	CakSNP16542	*Kabuli*	Ca_Kabuli_denovo	32328	(G/A)
16543	CakSNP16543	*Kabuli*	Ca_Kabuli_denovo	32330	(G/A)
16544	CakSNP16544	*Kabuli*	Ca_Kabuli_denovo	32331	(A/G)
16545	CakSNP16545	*Kabuli*	Ca_Kabuli_denovo	33189	(G/A)
16546	CakSNP16546	*Kabuli*	Ca_Kabuli_denovo	34679	(G/T)
16547	CakSNP16547	*Kabuli*	Ca_Kabuli_denovo	34680	(C/G)
16548	CakSNP16548	*Kabuli*	Ca_Kabuli_denovo	34682	(C/G)
16549	CakSNP16549	*Kabuli*	Ca_Kabuli_denovo	34683	(G/A)
16550	CakSNP16550	*Kabuli*	Ca_Kabuli_denovo	34684	(A/G)
16551	CakSNP16551	*Kabuli*	Ca_Kabuli_denovo	34685	(G/A)
16552	CakSNP16552	*Kabuli*	Ca_Kabuli_denovo	34858	(G/A)
16553	CakSNP16553	*Kabuli*	Ca_Kabuli_denovo	34860	(G/A)
16554	CakSNP16554	*Kabuli*	Ca_Kabuli_denovo	35627	(T/G)
16555	CakSNP16555	*Kabuli*	Ca_Kabuli_denovo	35634	(A/G)
16556	CakSNP16556	*Kabuli*	Ca_Kabuli_denovo	35645	(C/A)
16557	CakSNP16557	*Kabuli*	Ca_Kabuli_denovo	35661	(G/A)
16558	CakSNP16558	*Kabuli*	Ca_Kabuli_denovo	35679	(C/G)
16559	CakSNP16559	*Kabuli*	Ca_Kabuli_denovo	36295	(C/G)
16560	CakSNP16560	*Kabuli*	Ca_Kabuli_denovo	36358	(G/C)
16561	CakSNP16561	*Kabuli*	Ca_Kabuli_denovo	36359	(T/A)
16562	CakSNP16562	*Kabuli*	Ca_Kabuli_denovo	36361	(G/C)
16563	CakSNP16563	*Kabuli*	Ca_Kabuli_denovo	36362	(T/G)
16564	CakSNP16564	*Kabuli*	Ca_Kabuli_denovo	36544	(T/G)
16565	CakSNP16565	*Kabuli*	Ca_Kabuli_denovo	36545	(G/C)
16566	CakSNP16566	*Kabuli*	Ca_Kabuli_denovo	36547	(G/C)
16567	CakSNP16567	*Kabuli*	Ca_Kabuli_denovo	36548	(A/G)

S.N.	SNP IDs	Cultivars	Chromosomes/scaffolds	Physical positions (bp)	SNPs
16568	CakSNP16568	*Kabuli*	Ca_Kabuli_denovo	36549	(G/A)
16569	CakSNP16569	*Kabuli*	Ca_Kabuli_denovo	36550	(A/G)
16570	CakSNP16570	*Kabuli*	Ca_Kabuli_denovo	36620	(G/C)
16571	CakSNP16571	*Kabuli*	Ca_Kabuli_denovo	36621	(C/A)
16572	CakSNP16572	*Kabuli*	Ca_Kabuli_denovo	36622	(A/G)
16573	CakSNP16573	*Kabuli*	Ca_Kabuli_denovo	36624	(A/G)
16574	CakSNP16574	*Kabuli*	Ca_Kabuli_denovo	36626	(T/A)
16575	CakSNP16575	*Kabuli*	Ca_Kabuli_denovo	36627	(G/T)
16576	CakSNP16576	*Kabuli*	Ca_Kabuli_denovo	36628	(C/G)
16577	CakSNP16577	*Kabuli*	Ca_Kabuli_denovo	36630	(G/C)
16578	CakSNP16578	*Kabuli*	Ca_Kabuli_denovo	37457	(G/C)
16579	CakSNP16579	*Kabuli*	Ca_Kabuli_denovo	37458	(C/A)
16580	CakSNP16580	*Kabuli*	Ca_Kabuli_denovo	37459	(A/G)
16581	CakSNP16581	*Kabuli*	Ca_Kabuli_denovo	37461	(G/A)
16582	CakSNP16582	*Kabuli*	Ca_Kabuli_denovo	37463	(A/T)
16583	CakSNP16583	*Kabuli*	Ca_Kabuli_denovo	37464	(T/G)
16584	CakSNP16584	*Kabuli*	Ca_Kabuli_denovo	37465	(C/G)
16585	CakSNP16585	*Kabuli*	Ca_Kabuli_denovo	37467	(G/C)
16586	CakSNP16586	*Kabuli*	Ca_Kabuli_denovo	37468	(A/G)
16587	CakSNP16587	*Kabuli*	Ca_Kabuli_denovo	37469	(G/A)
16588	CakSNP16588	*Kabuli*	Ca_Kabuli_denovo	38106	(G/A)
16589	CakSNP16589	*Kabuli*	Ca_Kabuli_denovo	38108	(T/G)
16590	CakSNP16590	*Kabuli*	Ca_Kabuli_denovo	38109	(T/A)
16591	CakSNP16591	*Kabuli*	Ca_Kabuli_denovo	38110	(C/A)
16592	CakSNP16592	*Kabuli*	Ca_Kabuli_denovo	38111	(T/A)
16593	CakSNP16593	*Kabuli*	Ca_Kabuli_denovo	38114	(C/A)
16594	CakSNP16594	*Kabuli*	Ca_Kabuli_denovo	38932	(G/A)
16595	CakSNP16595	*Kabuli*	Ca_Kabuli_denovo	38934	(G/A)
16596	CakSNP16596	*Kabuli*	Ca_Kabuli_denovo	40259	(A/G)
16597	CakSNP16597	*Kabuli*	Ca_Kabuli_denovo	40260	(G/C)
16598	CakSNP16598	*Kabuli*	Ca_Kabuli_denovo	40261	(C/A)
16599	CakSNP16599	*Kabuli*	Ca_Kabuli_denovo	40262	(A/G)
16600	CakSNP16600	*Kabuli*	Ca_Kabuli_denovo	40264	(G/A)
16601	CakSNP16601	*Kabuli*	Ca_Kabuli_denovo	40266	(T/A)
16602	CakSNP16602	*Kabuli*	Ca_Kabuli_denovo	40267	(G/T)
16603	CakSNP16603	*Kabuli*	Ca_Kabuli_denovo	40268	(C/G)
16604	CakSNP16604	*Kabuli*	Ca_Kabuli_denovo	40270	(G/C)
16605	CakSNP16605	*Kabuli*	Ca_Kabuli_denovo	41456	(T/C)

S.N.	SNP IDs	Cultivars	Chromosomes/scaffolds	Physical positions (bp)	SNPs
16606	CakSNP16606	*Kabuli*	Ca_Kabuli_denovo	41457	(T/A)
16607	CakSNP16607	*Kabuli*	Ca_Kabuli_denovo	41458	(G/C)
16608	CakSNP16608	*Kabuli*	Ca_Kabuli_denovo	41460	(A/G)
16609	CakSNP16609	*Kabuli*	Ca_Kabuli_denovo	41888	(A/T)
16610	CakSNP16610	*Kabuli*	Ca_Kabuli_denovo	41895	(C/A)
16611	CakSNP16611	*Kabuli*	Ca_Kabuli_denovo	44072	(T/G)
16612	CakSNP16612	*Kabuli*	Ca_Kabuli_denovo	44074	(C/T)
16613	CakSNP16613	*Kabuli*	Ca_Kabuli_denovo	45632	(A/C)
16614	CakSNP16614	*Kabuli*	Ca_Kabuli_denovo	45635	(C/T)
16615	CakSNP16615	*Kabuli*	Ca_Kabuli_denovo	45637	(T/G)
16616	CakSNP16616	*Kabuli*	Ca_Kabuli_denovo	45639	(G/A)
16617	CakSNP16617	*Kabuli*	Ca_Kabuli_denovo	48879	(T/G)
16618	CakSNP16618	*Kabuli*	Ca_Kabuli_denovo	49259	(G/A)
16619	CakSNP16619	*Kabuli*	Ca_Kabuli_denovo	49260	(A/G)
16620	CakSNP16620	*Kabuli*	Ca_Kabuli_denovo	49261	(A/G)
16621	CakSNP16621	*Kabuli*	Ca_Kabuli_denovo	49262	(G/A)
16622	CakSNP16622	*Kabuli*	Ca_Kabuli_denovo	49263	(C/A)
16623	CakSNP16623	*Kabuli*	Ca_Kabuli_denovo	49265	(G/C)
16624	CakSNP16624	*Kabuli*	Ca_Kabuli_denovo	49932	(C/G)
16625	CakSNP16625	*Kabuli*	Ca_Kabuli_denovo	49934	(T/G)
16626	CakSNP16626	*Kabuli*	Ca_Kabuli_denovo	49936	(C/T)
16627	CakSNP16627	*Kabuli*	Ca_Kabuli_denovo	50705	(T/G)
16628	CakSNP16628	*Kabuli*	Ca_Kabuli_denovo	50712	(T/C)
16629	CakSNP16629	*Kabuli*	Ca_Kabuli_denovo	50914	(G/A)
16630	CakSNP16630	*Kabuli*	Ca_Kabuli_denovo	51290	(C/G)
16631	CakSNP16631	*Kabuli*	Ca_Kabuli_denovo	51357	(T/G)
16632	CakSNP16632	*Kabuli*	Ca_Kabuli_denovo	51491	(A/G)
16633	CakSNP16633	*Kabuli*	Ca_Kabuli_denovo	51528	(T/C)
16634	CakSNP16634	*Kabuli*	Ca_Kabuli_denovo	51691	(G/C)
16635	CakSNP16635	*Kabuli*	Ca_Kabuli_denovo	51720	(T/G)
16636	CakSNP16636	*Kabuli*	Ca_Kabuli_denovo	51737	(C/G)
16637	CakSNP16637	*Kabuli*	Ca_Kabuli_denovo	51993	(T/G)
16638	CakSNP16638	*Kabuli*	Ca_Kabuli_denovo	52004	(C/A)
16639	CakSNP16639	*Kabuli*	Ca_Kabuli_denovo	52027	(G/T)
16640	CakSNP16640	*Kabuli*	Ca_Kabuli_denovo	52098	(A/G)
16641	CakSNP16641	*Kabuli*	Ca_Kabuli_denovo	52113	(G/T)
16642	CakSNP16642	*Kabuli*	Ca_Kabuli_denovo	52290	(A/G)
16643	CakSNP16643	*Kabuli*	Ca_Kabuli_denovo	52303	(A/C)

S.N.	SNP IDs	Cultivars	Chromosomes/scaffolds	Physical positions (bp)	SNPs
16644	CakSNP16644	*Kabuli*	Ca_Kabuli_denovo	53001	(C/A)
16645	CakSNP16645	*Kabuli*	Ca_Kabuli_denovo	53002	(A/G)
16646	CakSNP16646	*Kabuli*	Ca_Kabuli_denovo	53003	(G/C)
16647	CakSNP16647	*Kabuli*	Ca_Kabuli_denovo	53207	(T/G)
16648	CakSNP16648	*Kabuli*	Ca_Kabuli_denovo	53219	(C/A)
16649	CakSNP16649	*Kabuli*	Ca_Kabuli_denovo	53399	(G/C)
16650	CakSNP16650	*Kabuli*	Ca_Kabuli_denovo	53594	(G/A)
16651	CakSNP16651	*Kabuli*	Ca_Kabuli_denovo	53782	(C/A)
16652	CakSNP16652	*Kabuli*	Ca_Kabuli_denovo	53972	(G/A)
16653	CakSNP16653	*Kabuli*	Ca_Kabuli_denovo	54229	(T/G)
16654	CakSNP16654	*Kabuli*	Ca_Kabuli_denovo	54262	(A/G)
16655	CakSNP16655	*Kabuli*	Ca_Kabuli_denovo	54267	(T/G)
16656	CakSNP16656	*Kabuli*	Ca_Kabuli_denovo	54513	(C/G)
16657	CakSNP16657	*Kabuli*	Ca_Kabuli_denovo	54540	(A/G)
16658	CakSNP16658	*Kabuli*	Ca_Kabuli_denovo	54870	(A/C)
16659	CakSNP16659	*Kabuli*	Ca_Kabuli_denovo	54877	(A/G)
16660	CakSNP16660	*Kabuli*	Ca_Kabuli_denovo	54880	(T/C)
16661	CakSNP16661	*Kabuli*	Ca_Kabuli_denovo	54888	(C/G)
16662	CakSNP16662	*Kabuli*	Ca_Kabuli_denovo	54895	(T/G)
16663	CakSNP16663	*Kabuli*	Ca_Kabuli_denovo	54900	(C/G)
16664	CakSNP16664	*Kabuli*	Ca_Kabuli_denovo	54909	(T/G)
16665	CakSNP16665	*Kabuli*	Ca_Kabuli_denovo	54910	(C/G)
16666	CakSNP16666	*Kabuli*	Ca_Kabuli_denovo	54911	(A/G)
16667	CakSNP16667	*Kabuli*	Ca_Kabuli_denovo	54915	(T/A)
16668	CakSNP16668	*Kabuli*	Ca_Kabuli_denovo	54932	(T/G)
16669	CakSNP16669	*Kabuli*	Ca_Kabuli_denovo	55406	(T/C)
16670	CakSNP16670	*Kabuli*	Ca_Kabuli_denovo	55407	(T/A)
16671	CakSNP16671	*Kabuli*	Ca_Kabuli_denovo	55408	(G/C)
16672	CakSNP16672	*Kabuli*	Ca_Kabuli_denovo	55409	(C/A)
16673	CakSNP16673	*Kabuli*	Ca_Kabuli_denovo	55504	(G/C)
16674	CakSNP16674	*Kabuli*	Ca_Kabuli_denovo	55505	(G/A)
16675	CakSNP16675	*Kabuli*	Ca_Kabuli_denovo	55506	(A/G)
16676	CakSNP16676	*Kabuli*	Ca_Kabuli_denovo	56193	(T/G)
16677	CakSNP16677	*Kabuli*	Ca_Kabuli_denovo	56223	(T/G)
16678	CakSNP16678	*Kabuli*	Ca_Kabuli_denovo	56260	(G/C)
16679	CakSNP16679	*Kabuli*	Ca_Kabuli_denovo	56261	(G/A)
16680	CakSNP16680	*Kabuli*	Ca_Kabuli_denovo	56262	(A/C)
16681	CakSNP16681	*Kabuli*	Ca_Kabuli_denovo	56263	(T/C)

S.N.	SNP IDs	Cultivars	Chromosomes/scaffolds	Physical positions (bp)	SNPs
16682	CakSNP16682	*Kabuli*	Ca_Kabuli_denovo	56264	(C/G)
16683	CakSNP16683	*Kabuli*	Ca_Kabuli_denovo	56266	(C/T)
16684	CakSNP16684	*Kabuli*	Ca_Kabuli_denovo	56344	(G/C)
16685	CakSNP16685	*Kabuli*	Ca_Kabuli_denovo	56345	(A/G)
16686	CakSNP16686	*Kabuli*	Ca_Kabuli_denovo	56442	(C/A)
16687	CakSNP16687	*Kabuli*	Ca_Kabuli_denovo	56443	(G/C)
16688	CakSNP16688	*Kabuli*	Ca_Kabuli_denovo	56444	(A/G)
16689	CakSNP16689	*Kabuli*	Ca_Kabuli_denovo	56620	(C/A)
16690	CakSNP16690	*Kabuli*	Ca_Kabuli_denovo	56621	(A/T)
16691	CakSNP16691	*Kabuli*	Ca_Kabuli_denovo	56623	(G/C)
16692	CakSNP16692	*Kabuli*	Ca_Kabuli_denovo	56624	(C/A)
16693	CakSNP16693	*Kabuli*	Ca_Kabuli_denovo	56625	(G/A)
16694	CakSNP16694	*Kabuli*	Ca_Kabuli_denovo	56721	(C/A)
16695	CakSNP16695	*Kabuli*	Ca_Kabuli_denovo	56723	(C/G)
16696	CakSNP16696	*Kabuli*	Ca_Kabuli_denovo	56724	(G/A)
16697	CakSNP16697	*Kabuli*	Ca_Kabuli_denovo	56811	(A/T)
16698	CakSNP16698	*Kabuli*	Ca_Kabuli_denovo	56812	(T/G)
16699	CakSNP16699	*Kabuli*	Ca_Kabuli_denovo	56813	(G/C)
16700	CakSNP16700	*Kabuli*	Ca_Kabuli_denovo	56815	(C/G)
16701	CakSNP16701	*Kabuli*	Ca_Kabuli_denovo	56816	(G/A)
16702	CakSNP16702	*Kabuli*	Ca_Kabuli_denovo	56817	(G/A)
16703	CakSNP16703	*Kabuli*	Ca_Kabuli_denovo	56818	(A/G)
16704	CakSNP16704	*Kabuli*	Ca_Kabuli_denovo	56900	(A/G)
16705	CakSNP16705	*Kabuli*	Ca_Kabuli_denovo	56902	(G/A)
16706	CakSNP16706	*Kabuli*	Ca_Kabuli_denovo	56904	(A/T)
16707	CakSNP16707	*Kabuli*	Ca_Kabuli_denovo	56905	(T/G)
16708	CakSNP16708	*Kabuli*	Ca_Kabuli_denovo	56906	(G/C)
16709	CakSNP16709	*Kabuli*	Ca_Kabuli_denovo	56908	(C/G)
16710	CakSNP16710	*Kabuli*	Ca_Kabuli_denovo	56909	(G/A)
16711	CakSNP16711	*Kabuli*	Ca_Kabuli_denovo	56910	(A/G)
16712	CakSNP16712	*Kabuli*	Ca_Kabuli_denovo	56911	(G/A)
16713	CakSNP16713	*Kabuli*	Ca_Kabuli_denovo	56912	(A/C)
16714	CakSNP16714	*Kabuli*	Ca_Kabuli_denovo	56913	(T/C)
16715	CakSNP16715	*Kabuli*	Ca_Kabuli_denovo	56915	(G/C)
16716	CakSNP16716	*Kabuli*	Ca_Kabuli_denovo	56916	(T/A)
16717	CakSNP16717	*Kabuli*	Ca_Kabuli_denovo	57097	(T/G)
16718	CakSNP16718	*Kabuli*	Ca_Kabuli_denovo	57098	(C/A)
16719	CakSNP16719	*Kabuli*	Ca_Kabuli_denovo	57100	(C/T)

S.N.	SNP IDs	Cultivars	Chromosomes/scaffolds	Physical positions (bp)	SNPs
16720	CakSNP16720	*Kabuli*	Ca_Kabuli_denovo	57101	(G/C)
16721	CakSNP16721	*Kabuli*	Ca_Kabuli_denovo	57170	(C/G)
16722	CakSNP16722	*Kabuli*	Ca_Kabuli_denovo	57172	(G/T)
16723	CakSNP16723	*Kabuli*	Ca_Kabuli_denovo	57174	(T/C)
16724	CakSNP16724	*Kabuli*	Ca_Kabuli_denovo	57175	(C/A)
16725	CakSNP16725	*Kabuli*	Ca_Kabuli_denovo	57176	(A/G)
16726	CakSNP16726	*Kabuli*	Ca_Kabuli_denovo	57177	(C/G)
16727	CakSNP16727	*Kabuli*	Ca_Kabuli_denovo	57178	(A/C)
16728	CakSNP16728	*Kabuli*	Ca_Kabuli_denovo	57193	(G/T)
16729	CakSNP16729	*Kabuli*	Ca_Kabuli_denovo	57194	(A/C)
16730	CakSNP16730	*Kabuli*	Ca_Kabuli_denovo	57195	(T/G)
16731	CakSNP16731	*Kabuli*	Ca_Kabuli_denovo	57196	(C/T)
16732	CakSNP16732	*Kabuli*	Ca_Kabuli_denovo	57271	(C/A)
16733	CakSNP16733	*Kabuli*	Ca_Kabuli_denovo	57272	(A/G)
16734	CakSNP16734	*Kabuli*	Ca_Kabuli_denovo	57275	(A/G)
16735	CakSNP16735	*Kabuli*	Ca_Kabuli_denovo	57276	(A/T)
16736	CakSNP16736	*Kabuli*	Ca_Kabuli_denovo	57277	(G/T)
16737	CakSNP16737	*Kabuli*	Ca_Kabuli_denovo	57278	(A/C)
16738	CakSNP16738	*Kabuli*	Ca_Kabuli_denovo	57280	(C/G)
16739	CakSNP16739	*Kabuli*	Ca_Kabuli_denovo	57281	(G/A)
16740	CakSNP16740	*Kabuli*	Ca_Kabuli_denovo	57282	(A/G)
16741	CakSNP16741	*Kabuli*	Ca_Kabuli_denovo	57283	(T/A)
16742	CakSNP16742	*Kabuli*	Ca_Kabuli_denovo	57285	(T/C)
16743	CakSNP16743	*Kabuli*	Ca_Kabuli_denovo	57286	(C/G)
16744	CakSNP16744	*Kabuli*	Ca_Kabuli_denovo	57287	(G/A)
16745	CakSNP16745	*Kabuli*	Ca_Kabuli_denovo	57289	(A/C)
16746	CakSNP16746	*Kabuli*	Ca_Kabuli_denovo	57955	(A/G)
16747	CakSNP16747	*Kabuli*	Ca_Kabuli_denovo	57990	(T/G)
16748	CakSNP16748	*Kabuli*	Ca_Kabuli_denovo	58026	(A/C)
16749	CakSNP16749	*Kabuli*	Ca_Kabuli_denovo	58029	(C/T)
16750	CakSNP16750	*Kabuli*	Ca_Kabuli_denovo	58031	(T/G)
16751	CakSNP16751	*Kabuli*	Ca_Kabuli_denovo	58032	(C/A)
16752	CakSNP16752	*Kabuli*	Ca_Kabuli_denovo	58033	(G/T)
16753	CakSNP16753	*Kabuli*	Ca_Kabuli_denovo	58046	(T/G)
16754	CakSNP16754	*Kabuli*	Ca_Kabuli_denovo	58761	(G/A)
16755	CakSNP16755	*Kabuli*	Ca_Kabuli_denovo	58763	(G/A)
16756	CakSNP16756	*Kabuli*	Ca_Kabuli_denovo	58765	(G/A)
16757	CakSNP16757	*Kabuli*	Ca_Kabuli_denovo	59113	(G/T)

S.N.	SNP IDs	Cultivars	Chromosomes/scaffolds	Physical positions (bp)	SNPs
16758	CakSNP16758	*Kabuli*	Ca_Kabuli_denovo	60062	(C/G)
16759	CakSNP16759	*Kabuli*	Ca_Kabuli_denovo	60064	(T/G)
16760	CakSNP16760	*Kabuli*	Ca_Kabuli_denovo	60066	(C/T)
16761	CakSNP16761	*Kabuli*	Ca_Kabuli_denovo	60711	(G/A)
16762	CakSNP16762	*Kabuli*	Ca_Kabuli_denovo	60712	(A/G)
16763	CakSNP16763	*Kabuli*	Ca_Kabuli_denovo	62231	(G/A)
16764	CakSNP16764	*Kabuli*	Ca_Kabuli_denovo	62282	(G/A)
16765	CakSNP16765	*Kabuli*	Ca_Kabuli_denovo	62560	(G/A)
16766	CakSNP16766	*Kabuli*	Ca_Kabuli_denovo	62562	(G/A)
16767	CakSNP16767	*Kabuli*	Ca_Kabuli_denovo	63303	(C/G)
16768	CakSNP16768	*Kabuli*	Ca_Kabuli_denovo	63305	(T/G)
16769	CakSNP16769	*Kabuli*	Ca_Kabuli_denovo	63307	(C/T)
16770	CakSNP16770	*Kabuli*	Ca_Kabuli_denovo	64427	(T/C)
16771	CakSNP16771	*Kabuli*	Ca_Kabuli_denovo	64428	(T/A)
16772	CakSNP16772	*Kabuli*	Ca_Kabuli_denovo	64429	(G/C)
16773	CakSNP16773	*Kabuli*	Ca_Kabuli_denovo	64525	(C/G)
16774	CakSNP16774	*Kabuli*	Ca_Kabuli_denovo	64526	(G/A)
16775	CakSNP16775	*Kabuli*	Ca_Kabuli_denovo	66001	(G/A)
16776	CakSNP16776	*Kabuli*	Ca_Kabuli_denovo	66003	(G/A)
16777	CakSNP16777	*Kabuli*	Ca_Kabuli_denovo	66373	(G/A)
16778	CakSNP16778	*Kabuli*	Ca_Kabuli_denovo	67505	(A/G)
16779	CakSNP16779	*Kabuli*	Ca_Kabuli_denovo	67506	(C/G)
16780	CakSNP16780	*Kabuli*	Ca_Kabuli_denovo	67912	(G/T)
16781	CakSNP16781	*Kabuli*	Ca_Kabuli_denovo	68709	(G/A)
16782	CakSNP16782	*Kabuli*	Ca_Kabuli_denovo	68710	(C/G)
16783	CakSNP16783	*Kabuli*	Ca_Kabuli_denovo	68711	(A/C)
16784	CakSNP16784	*Kabuli*	Ca_Kabuli_denovo	68712	(A/G)
16785	CakSNP16785	*Kabuli*	Ca_Kabuli_denovo	68714	(G/A)
16786	CakSNP16786	*Kabuli*	Ca_Kabuli_denovo	68716	(A/T)
16787	CakSNP16787	*Kabuli*	Ca_Kabuli_denovo	69930	(C/G)
16788	CakSNP16788	*Kabuli*	Ca_Kabuli_denovo	69932	(G/T)
16789	CakSNP16789	*Kabuli*	Ca_Kabuli_denovo	69934	(C/T)
16790	CakSNP16790	*Kabuli*	Ca_Kabuli_denovo	69935	(A/C)
16791	CakSNP16791	*Kabuli*	Ca_Kabuli_denovo	69936	(G/A)
16792	CakSNP16792	*Kabuli*	Ca_Kabuli_denovo	69937	(C/G)
16793	CakSNP16793	*Kabuli*	Ca_Kabuli_denovo	71409	(G/A)
16794	CakSNP16794	*Kabuli*	Ca_Kabuli_denovo	71411	(G/A)
16795	CakSNP16795	*Kabuli*	Ca_Kabuli_denovo	72745	(G/T)

S.N.	SNP IDs	Cultivars	Chromosomes/scaffolds	Physical positions (bp)	SNPs
16796	CakSNP16796	*Kabuli*	Ca_Kabuli_denovo	74388	(G/C)
16797	CakSNP16797	*Kabuli*	Ca_Kabuli_denovo	74389	(C/G)
16798	CakSNP16798	*Kabuli*	Ca_Kabuli_denovo	74391	(T/G)
16799	CakSNP16799	*Kabuli*	Ca_Kabuli_denovo	74393	(C/T)
16800	CakSNP16800	*Kabuli*	Ca_Kabuli_denovo	74394	(A/C)
16801	CakSNP16801	*Kabuli*	Ca_Kabuli_denovo	74952	(C/G)
16802	CakSNP16802	*Kabuli*	Ca_Kabuli_denovo	74954	(G/T)
16803	CakSNP16803	*Kabuli*	Ca_Kabuli_denovo	74956	(C/T)
16804	CakSNP16804	*Kabuli*	Ca_Kabuli_denovo	74957	(A/C)
16805	CakSNP16805	*Kabuli*	Ca_Kabuli_denovo	74958	(G/A)
16806	CakSNP16806	*Kabuli*	Ca_Kabuli_denovo	74959	(C/G)
16807	CakSNP16807	*Kabuli*	Ca_Kabuli_denovo	74972	(A/G)
16808	CakSNP16808	*Kabuli*	Ca_Kabuli_denovo	75008	(G/A)
16809	CakSNP16809	*Kabuli*	Ca_Kabuli_denovo	76173	(A/G)
16810	CakSNP16810	*Kabuli*	Ca_Kabuli_denovo	76178	(G/A)
16811	CakSNP16811	*Kabuli*	Ca_Kabuli_denovo	76193	(C/G)
16812	CakSNP16812	*Kabuli*	Ca_Kabuli_denovo	76204	(C/G)
16813	CakSNP16813	*Kabuli*	Ca_Kabuli_denovo	76218	(T/G)
16814	CakSNP16814	*Kabuli*	Ca_Kabuli_denovo	76228	(G/C)
16815	CakSNP16815	*Kabuli*	Ca_Kabuli_denovo	76288	(C/T)
16816	CakSNP16816	*Kabuli*	Ca_Kabuli_denovo	76295	(G/T)
16817	CakSNP16817	*Kabuli*	Ca_Kabuli_denovo	76452	(T/A)
16818	CakSNP16818	*Kabuli*	Ca_Kabuli_denovo	76513	(A/G)
16819	CakSNP16819	*Kabuli*	Ca_Kabuli_denovo	76517	(T/C)
16820	CakSNP16820	*Kabuli*	Ca_Kabuli_denovo	76530	(T/G)
16821	CakSNP16821	*Kabuli*	Ca_Kabuli_denovo	76561	(G/T)
16822	CakSNP16822	*Kabuli*	Ca_Kabuli_denovo	76579	(G/A)
16823	CakSNP16823	*Kabuli*	Ca_Kabuli_denovo	77007	(T/A)
16824	CakSNP16824	*Kabuli*	Ca_Kabuli_denovo	77013	(A/T)
16825	CakSNP16825	*Kabuli*	Ca_Kabuli_denovo	77051	(T/A)
16826	CakSNP16826	*Kabuli*	Ca_Kabuli_denovo	77063	(A/G)
16827	CakSNP16827	*Kabuli*	Ca_Kabuli_denovo	78895	(C/T)
16828	CakSNP16828	*Kabuli*	Ca_Kabuli_denovo	78946	(C/T)
16829	CakSNP16829	*Kabuli*	Ca_Kabuli_denovo	79307	(T/A)
16830	CakSNP16830	*Kabuli*	Ca_Kabuli_denovo	79308	(T/G)
16831	CakSNP16831	*Kabuli*	Ca_Kabuli_denovo	79309	(C/G)
16832	CakSNP16832	*Kabuli*	Ca_Kabuli_denovo	79311	(A/G)
16833	CakSNP16833	*Kabuli*	Ca_Kabuli_denovo	79312	(T/C)

S.N.	SNP IDs	Cultivars	Chromosomes/scaffolds	Physical positions (bp)	SNPs
16834	CakSNP16834	*Kabuli*	Ca_Kabuli_denovo	79313	(G/A)
16835	CakSNP16835	*Kabuli*	Ca_Kabuli_denovo	83072	(C/T)
16836	CakSNP16836	*Kabuli*	Ca_Kabuli_denovo	83103	(C/G)
16837	CakSNP16837	*Kabuli*	Ca_Kabuli_denovo	83773	(C/G)
16838	CakSNP16838	*Kabuli*	Ca_Kabuli_denovo	83775	(A/G)
16839	CakSNP16839	*Kabuli*	Ca_Kabuli_denovo	83776	(T/C)
16840	CakSNP16840	*Kabuli*	Ca_Kabuli_denovo	83777	(G/A)
16841	CakSNP16841	*Kabuli*	Ca_Kabuli_denovo	83885	(A/C)
16842	CakSNP16842	*Kabuli*	Ca_Kabuli_denovo	84999	(T/G)
16843	CakSNP16843	*Kabuli*	Ca_Kabuli_denovo	85000	(G/A)
16844	CakSNP16844	*Kabuli*	Ca_Kabuli_denovo	85001	(C/G)
16845	CakSNP16845	*Kabuli*	Ca_Kabuli_denovo	85003	(G/C)
16846	CakSNP16846	*Kabuli*	Ca_Kabuli_denovo	89165	(G/A)
16847	CakSNP16847	*Kabuli*	Ca_Kabuli_denovo	89167	(G/A)
16848	CakSNP16848	*Kabuli*	Ca_Kabuli_denovo	89731	(C/G)
16849	CakSNP16849	*Kabuli*	Ca_Kabuli_denovo	89733	(A/G)
16850	CakSNP16850	*Kabuli*	Ca_Kabuli_denovo	89829	(G/A)
16851	CakSNP16851	*Kabuli*	Ca_Kabuli_denovo	89830	(G/A)
16852	CakSNP16852	*Kabuli*	Ca_Kabuli_denovo	90103	(G/A)
16853	CakSNP16853	*Kabuli*	Ca_Kabuli_denovo	90105	(G/A)
16854	CakSNP16854	*Kabuli*	Ca_Kabuli_denovo	90106	(A/G)
16855	CakSNP16855	*Kabuli*	Ca_Kabuli_denovo	90209	(A/C)
16856	CakSNP16856	*Kabuli*	Ca_Kabuli_denovo	90210	(G/T)
16857	CakSNP16857	*Kabuli*	Ca_Kabuli_denovo	90211	(A/G)
16858	CakSNP16858	*Kabuli*	Ca_Kabuli_denovo	90488	(A/C)
16859	CakSNP16859	*Kabuli*	Ca_Kabuli_denovo	92231	(G/A)
16860	CakSNP16860	*Kabuli*	Ca_Kabuli_denovo	92233	(G/A)
16861	CakSNP16861	*Kabuli*	Ca_Kabuli_denovo	92989	(T/A)
16862	CakSNP16862	*Kabuli*	Ca_Kabuli_denovo	92990	(G/T)
16863	CakSNP16863	*Kabuli*	Ca_Kabuli_denovo	94682	(G/A)
16864	CakSNP16864	*Kabuli*	Ca_Kabuli_denovo	94731	(G/A)
16865	CakSNP16865	*Kabuli*	Ca_Kabuli_denovo	96656	(C/T)
16866	CakSNP16866	*Kabuli*	Ca_Kabuli_denovo	96658	(A/C)
16867	CakSNP16867	*Kabuli*	Ca_Kabuli_denovo	96660	(G/A)
16868	CakSNP16868	*Kabuli*	Ca_Kabuli_denovo	96989	(A/G)
16869	CakSNP16869	*Kabuli*	Ca_Kabuli_denovo	96991	(A/G)
16870	CakSNP16870	*Kabuli*	Ca_Kabuli_denovo	96993	(T/A)
16871	CakSNP16871	*Kabuli*	Ca_Kabuli_denovo	96994	(G/T)

**S.N.**	**SNP IDs**	**Cultivars**	**Chromosomes/scaffolds**	**Physical positions (bp)**	**SNPs**
16872	CakSNP16872	*Kabuli*	Ca_Kabuli_denovo	96995	(C/G)
16873	CakSNP16873	*Kabuli*	Ca_Kabuli_denovo	98504	(A/G)
16874	CakSNP16874	*Kabuli*	Ca_Kabuli_denovo	98519	(A/G)
16875	CakSNP16875	*Kabuli*	Ca_Kabuli_denovo	98520	(A/G)
16876	CakSNP16876	*Kabuli*	Ca_Kabuli_denovo	98532	(G/A)
16877	CakSNP16877	*Kabuli*	Ca_Kabuli_denovo	98534	(C/G)
16878	CakSNP16878	*Kabuli*	Ca_Kabuli_denovo	98536	(C/G)
16879	CakSNP16879	*Kabuli*	Ca_Kabuli_denovo	98537	(A/G)
16880	CakSNP16880	*Kabuli*	Ca_Kabuli_denovo	98550	(A/C)
16881	CakSNP16881	*Kabuli*	Ca_Kabuli_denovo	98564	(A/G)
16882	CakSNP16882	*Kabuli*	Ca_Kabuli_denovo	98567	(A/G)
16883	CakSNP16883	*Kabuli*	Ca_Kabuli_denovo	98874	(T/A)
16884	CakSNP16884	*Kabuli*	Ca_Kabuli_denovo	98885	(T/C)
16885	CakSNP16885	*Kabuli*	Ca_Kabuli_denovo	98887	(T/C)
16886	CakSNP16886	*Kabuli*	Ca_Kabuli_denovo	98913	(G/T)
16887	CakSNP16887	*Kabuli*	Ca_Kabuli_denovo	99120	(T/C)
16888	CakSNP16888	*Kabuli*	Ca_Kabuli_denovo	99137	(A/G)
16889	CakSNP16889	*Kabuli*	Ca_Kabuli_denovo	99210	(C/G)
16890	CakSNP16890	*Kabuli*	Ca_Kabuli_denovo	99212	(T/G)
16891	CakSNP16891	*Kabuli*	Ca_Kabuli_denovo	99214	(C/T)
16892	CakSNP16892	*Kabuli*	Ca_Kabuli_denovo	99215	(A/C)
16893	CakSNP16893	*Kabuli*	Ca_Kabuli_denovo	99985	(A/G)
16894	CakSNP16894	*Kabuli*	Ca_Kabuli_denovo	99996	(A/G)
16895	CakSNP16895	*Kabuli*	Ca_Kabuli_denovo	99997	(A/G)
16896	CakSNP16896	*Kabuli*	Ca_Kabuli_denovo	100026	(C/G)
16897	CakSNP16897	*Kabuli*	Ca_Kabuli_denovo	100032	(A/G)
16898	CakSNP16898	*Kabuli*	Ca_Kabuli_denovo	100035	(A/T)
16899	CakSNP16899	*Kabuli*	Ca_Kabuli_denovo	100038	(C/G)
16900	CakSNP16900	*Kabuli*	Ca_Kabuli_denovo	100039	(C/G)
16901	CakSNP16901	*Kabuli*	Ca_Kabuli_denovo	100051	(A/G)
16902	CakSNP16902	*Kabuli*	Ca_Kabuli_denovo	100054	(A/G)
16903	CakSNP16903	*Kabuli*	Ca_Kabuli_denovo	100063	(A/G)
16904	CakSNP16904	*Kabuli*	Ca_Kabuli_denovo	101348	(T/G)
16905	CakSNP16905	*Kabuli*	Ca_Kabuli_denovo	101357	(A/G)
16906	CakSNP16906	*Kabuli*	Ca_Kabuli_denovo	101358	(A/C)
16907	CakSNP16907	*Kabuli*	Ca_Kabuli_denovo	101359	(T/C)
16908	CakSNP16908	*Kabuli*	Ca_Kabuli_denovo	101361	(A/C)
16909	CakSNP16909	*Kabuli*	Ca_Kabuli_denovo	101362	(G/C)

S.N.	SNP IDs	Cultivars	Chromosomes/scaffolds	Physical positions (bp)	SNPs
16910	CakSNP16910	*Kabuli*	Ca_Kabuli_denovo	101363	(A/G)
16911	CakSNP16911	*Kabuli*	Ca_Kabuli_denovo	101364	(C/A)
16912	CakSNP16912	*Kabuli*	Ca_Kabuli_denovo	101367	(A/C)
16913	CakSNP16913	*Kabuli*	Ca_Kabuli_denovo	101371	(C/T)
16914	CakSNP16914	*Kabuli*	Ca_Kabuli_denovo	101919	(C/A)
16915	CakSNP16915	*Kabuli*	Ca_Kabuli_denovo	101920	(C/T)
16916	CakSNP16916	*Kabuli*	Ca_Kabuli_denovo	101921	(G/C)
16917	CakSNP16917	*Kabuli*	Ca_Kabuli_denovo	102039	(A/G)
16918	CakSNP16918	*Kabuli*	Ca_Kabuli_denovo	102047	(G/A)
16919	CakSNP16919	*Kabuli*	Ca_Kabuli_denovo	102091	(A/C)
16920	CakSNP16920	*Kabuli*	Ca_Kabuli_denovo	102099	(C/T)
16921	CakSNP16921	*Kabuli*	Ca_Kabuli_denovo	102171	(G/A)
16922	CakSNP16922	*Kabuli*	Ca_Kabuli_denovo	102173	(G/A)
16923	CakSNP16923	*Kabuli*	Ca_Kabuli_denovo	105285	(C/G)
16924	CakSNP16924	*Kabuli*	Ca_Kabuli_denovo	105303	(C/G)
16925	CakSNP16925	*Kabuli*	Ca_Kabuli_denovo	105305	(T/C)
16926	CakSNP16926	*Kabuli*	Ca_Kabuli_denovo	105319	(G/T)
16927	CakSNP16927	*Kabuli*	Ca_Kabuli_denovo	105320	(T/G)
16928	CakSNP16928	*Kabuli*	Ca_Kabuli_denovo	105321	(C/A)
16929	CakSNP16929	*Kabuli*	Ca_Kabuli_denovo	105326	(T/G)
16930	CakSNP16930	*Kabuli*	Ca_Kabuli_denovo	105336	(A/G)
16931	CakSNP16931	*Kabuli*	Ca_Kabuli_denovo	105339	(C/G)
16932	CakSNP16932	*Kabuli*	Ca_Kabuli_denovo	105790	(G/T)
16933	CakSNP16933	*Kabuli*	Ca_Kabuli_denovo	105792	(T/C)
16934	CakSNP16934	*Kabuli*	Ca_Kabuli_denovo	105795	(A/C)
16935	CakSNP16935	*Kabuli*	Ca_Kabuli_denovo	105819	(G/T)
16936	CakSNP16936	*Kabuli*	Ca_Kabuli_denovo	106278	(A/G)
16937	CakSNP16937	*Kabuli*	Ca_Kabuli_denovo	106280	(C/G)
16938	CakSNP16938	*Kabuli*	Ca_Kabuli_denovo	106282	(T/G)
16939	CakSNP16939	*Kabuli*	Ca_Kabuli_denovo	106284	(C/T)
16940	CakSNP16940	*Kabuli*	Ca_Kabuli_denovo	107119	(C/G)
16941	CakSNP16941	*Kabuli*	Ca_Kabuli_denovo	107120	(A/C)
16942	CakSNP16942	*Kabuli*	Ca_Kabuli_denovo	107121	(G/C)
16943	CakSNP16943	*Kabuli*	Ca_Kabuli_denovo	107124	(G/A)
16944	CakSNP16944	*Kabuli*	Ca_Kabuli_denovo	107125	(A/T)
16945	CakSNP16945	*Kabuli*	Ca_Kabuli_denovo	110466	(G/A)
16946	CakSNP16946	*Kabuli*	Ca_Kabuli_denovo	110468	(G/A)
16947	CakSNP16947	*Kabuli*	Ca_Kabuli_denovo	111292	(C/G)

S.N.	SNP IDs	Cultivars	Chromosomes/scaffolds	Physical positions (bp)	SNPs
16948	CakSNP16948	*Kabuli*	Ca_Kabuli_denovo	111294	(T/G)
16949	CakSNP16949	*Kabuli*	Ca_Kabuli_denovo	111296	(C/T)
16950	CakSNP16950	*Kabuli*	Ca_Kabuli_denovo	115118	(A/C)
16951	CakSNP16951	*Kabuli*	Ca_Kabuli_denovo	115119	(G/C)
16952	CakSNP16952	*Kabuli*	Ca_Kabuli_denovo	115122	(G/A)
16953	CakSNP16953	*Kabuli*	Ca_Kabuli_denovo	115123	(A/T)
16954	CakSNP16954	*Kabuli*	Ca_Kabuli_denovo	115219	(G/C)
16955	CakSNP16955	*Kabuli*	Ca_Kabuli_denovo	115220	(A/G)
16956	CakSNP16956	*Kabuli*	Ca_Kabuli_denovo	115768	(C/A)
16957	CakSNP16957	*Kabuli*	Ca_Kabuli_denovo	115769	(T/A)
16958	CakSNP16958	*Kabuli*	Ca_Kabuli_denovo	115771	(C/G)
16959	CakSNP16959	*Kabuli*	Ca_Kabuli_denovo	116794	(G/A)
16960	CakSNP16960	*Kabuli*	Ca_Kabuli_denovo	116795	(A/T)
16961	CakSNP16961	*Kabuli*	Ca_Kabuli_denovo	116796	(G/A)
16962	CakSNP16962	*Kabuli*	Ca_Kabuli_denovo	116797	(C/T)
16963	CakSNP16963	*Kabuli*	Ca_Kabuli_denovo	116798	(C/G)
16964	CakSNP16964	*Kabuli*	Ca_Kabuli_denovo	116954	(A/G)
16965	CakSNP16965	*Kabuli*	Ca_Kabuli_denovo	117155	(A/G)
16966	CakSNP16966	*Kabuli*	Ca_Kabuli_denovo	117163	(T/C)
16967	CakSNP16967	*Kabuli*	Ca_Kabuli_denovo	117344	(T/C)
16968	CakSNP16968	*Kabuli*	Ca_Kabuli_denovo	117345	(T/A)
16969	CakSNP16969	*Kabuli*	Ca_Kabuli_denovo	117346	(G/C)
16970	CakSNP16970	*Kabuli*	Ca_Kabuli_denovo	117347	(G/A)
16971	CakSNP16971	*Kabuli*	Ca_Kabuli_denovo	117802	(A/G)
16972	CakSNP16972	*Kabuli*	Ca_Kabuli_denovo	117804	(A/G)
16973	CakSNP16973	*Kabuli*	Ca_Kabuli_denovo	117806	(T/C)
16974	CakSNP16974	*Kabuli*	Ca_Kabuli_denovo	117807	(T/G)
16975	CakSNP16975	*Kabuli*	Ca_Kabuli_denovo	117901	(G/C)
16976	CakSNP16976	*Kabuli*	Ca_Kabuli_denovo	117996	(T/G)
16977	CakSNP16977	*Kabuli*	Ca_Kabuli_denovo	118000	(G/C)
16978	CakSNP16978	*Kabuli*	Ca_Kabuli_denovo	118002	(A/G)
16979	CakSNP16979	*Kabuli*	Ca_Kabuli_denovo	119682	(C/G)
16980	CakSNP16980	*Kabuli*	Ca_Kabuli_denovo	119765	(A/G)
16981	CakSNP16981	*Kabuli*	Ca_Kabuli_denovo	119767	(A/C)
16982	CakSNP16982	*Kabuli*	Ca_Kabuli_denovo	122783	(T/C)
16983	CakSNP16983	*Kabuli*	Ca_Kabuli_denovo	122839	(T/G)
16984	CakSNP16984	*Kabuli*	Ca_Kabuli_denovo	124009	(G/T)
16985	CakSNP16985	*Kabuli*	Ca_Kabuli_denovo	124031	(A/G)

S.N.	SNP IDs	Cultivars	Chromosomes/scaffolds	Physical positions (bp)	SNPs
16986	CakSNP16986	*Kabuli*	Ca_Kabuli_denovo	124227	(G/T)
16987	CakSNP16987	*Kabuli*	Ca_Kabuli_denovo	124274	(G/A)
16988	CakSNP16988	*Kabuli*	Ca_Kabuli_denovo	124738	(T/A)
16989	CakSNP16989	*Kabuli*	Ca_Kabuli_denovo	124751	(T/A)
16990	CakSNP16990	*Kabuli*	Ca_Kabuli_denovo	124772	(C/G)
16991	CakSNP16991	*Kabuli*	Ca_Kabuli_denovo	124780	(A/G)
16992	CakSNP16992	*Kabuli*	Ca_Kabuli_denovo	125616	(A/G)
16993	CakSNP16993	*Kabuli*	Ca_Kabuli_denovo	125617	(T/G)
16994	CakSNP16994	*Kabuli*	Ca_Kabuli_denovo	125618	(T/C)
16995	CakSNP16995	*Kabuli*	Ca_Kabuli_denovo	127024	(A/G)
16996	CakSNP16996	*Kabuli*	Ca_Kabuli_denovo	127401	(T/G)
16997	CakSNP16997	*Kabuli*	Ca_Kabuli_denovo	127409	(G/T)
16998	CakSNP16998	*Kabuli*	Ca_Kabuli_denovo	127410	(G/T)
16999	CakSNP16999	*Kabuli*	Ca_Kabuli_denovo	127411	(G/C)
17000	CakSNP17000	*Kabuli*	Ca_Kabuli_denovo	127493	(A/G)
17001	CakSNP17001	*Kabuli*	Ca_Kabuli_denovo	127494	(A/G)
17002	CakSNP17002	*Kabuli*	Ca_Kabuli_denovo	129540	(A/C)
17003	CakSNP17003	*Kabuli*	Ca_Kabuli_denovo	129899	(T/G)
17004	CakSNP17004	*Kabuli*	Ca_Kabuli_denovo	129902	(A/C)
17005	CakSNP17005	*Kabuli*	Ca_Kabuli_denovo	129903	(G/A)
17006	CakSNP17006	*Kabuli*	Ca_Kabuli_denovo	129904	(C/G)
17007	CakSNP17007	*Kabuli*	Ca_Kabuli_denovo	129905	(C/A)
17008	CakSNP17008	*Kabuli*	Ca_Kabuli_denovo	129906	(A/G)
17009	CakSNP17009	*Kabuli*	Ca_Kabuli_denovo	129908	(G/A)
17010	CakSNP17010	*Kabuli*	Ca_Kabuli_denovo	130839	(A/C)
17011	CakSNP17011	*Kabuli*	Ca_Kabuli_denovo	130840	(T/G)
17012	CakSNP17012	*Kabuli*	Ca_Kabuli_denovo	130841	(G/A)
17013	CakSNP17013	*Kabuli*	Ca_Kabuli_denovo	130842	(C/T)
17014	CakSNP17014	*Kabuli*	Ca_Kabuli_denovo	133633	(A/G)
17015	CakSNP17015	*Kabuli*	Ca_Kabuli_denovo	133634	(A/G)
17016	CakSNP17016	*Kabuli*	Ca_Kabuli_denovo	136132	(G/A)
17017	CakSNP17017	*Kabuli*	Ca_Kabuli_denovo	136134	(G/A)
17018	CakSNP17018	*Kabuli*	Ca_Kabuli_denovo	138972	(A/G)
17019	CakSNP17019	*Kabuli*	Ca_Kabuli_denovo	139763	(T/G)
17020	CakSNP17020	*Kabuli*	Ca_Kabuli_denovo	139764	(G/C)
17021	CakSNP17021	*Kabuli*	Ca_Kabuli_denovo	140441	(G/T)
17022	CakSNP17022	*Kabuli*	Ca_Kabuli_denovo	140457	(T/G)
17023	CakSNP17023	*Kabuli*	Ca_Kabuli_denovo	140481	(A/G)

S.N.	SNP IDs	Cultivars	Chromosomes/scaffolds	Physical positions (bp)	SNPs
17024	CakSNP17024	*Kabuli*	Ca_Kabuli_denovo	143491	(C/G)
17025	CakSNP17025	*Kabuli*	Ca_Kabuli_denovo	143493	(T/G)
17026	CakSNP17026	*Kabuli*	Ca_Kabuli_denovo	143495	(C/T)
17027	CakSNP17027	*Kabuli*	Ca_Kabuli_denovo	143496	(A/C)
17028	CakSNP17028	*Kabuli*	Ca_Kabuli_denovo	143497	(G/A)
17029	CakSNP17029	*Kabuli*	Ca_Kabuli_denovo	146274	(G/A)
17030	CakSNP17030	*Kabuli*	Ca_Kabuli_denovo	146364	(A/G)
17031	CakSNP17031	*Kabuli*	Ca_Kabuli_denovo	146747	(A/G)
17032	CakSNP17032	*Kabuli*	Ca_Kabuli_denovo	146749	(G/A)
17033	CakSNP17033	*Kabuli*	Ca_Kabuli_denovo	146751	(T/A)
17034	CakSNP17034	*Kabuli*	Ca_Kabuli_denovo	146753	(C/G)
17035	CakSNP17035	*Kabuli*	Ca_Kabuli_denovo	146755	(G/C)
17036	CakSNP17036	*Kabuli*	Ca_Kabuli_denovo	147027	(A/G)
17037	CakSNP17037	*Kabuli*	Ca_Kabuli_denovo	147029	(A/G)
17038	CakSNP17038	*Kabuli*	Ca_Kabuli_denovo	147031	(A/T)
17039	CakSNP17039	*Kabuli*	Ca_Kabuli_denovo	147032	(G/T)
17040	CakSNP17040	*Kabuli*	Ca_Kabuli_denovo	147033	(C/G)
17041	CakSNP17041	*Kabuli*	Ca_Kabuli_denovo	149439	(A/C)
17042	CakSNP17042	*Kabuli*	Ca_Kabuli_denovo	149440	(G/T)
17043	CakSNP17043	*Kabuli*	Ca_Kabuli_denovo	149628	(C/G)
17044	CakSNP17044	*Kabuli*	Ca_Kabuli_denovo	149629	(C/A)
17045	CakSNP17045	*Kabuli*	Ca_Kabuli_denovo	149632	(C/G)
17046	CakSNP17046	*Kabuli*	Ca_Kabuli_denovo	149809	(A/G)
17047	CakSNP17047	*Kabuli*	Ca_Kabuli_denovo	149811	(A/G)
17048	CakSNP17048	*Kabuli*	Ca_Kabuli_denovo	149813	(T/A)
17049	CakSNP17049	*Kabuli*	Ca_Kabuli_denovo	150092	(A/G)
17050	CakSNP17050	*Kabuli*	Ca_Kabuli_denovo	150094	(G/A)
17051	CakSNP17051	*Kabuli*	Ca_Kabuli_denovo	150096	(T/A)
17052	CakSNP17052	*Kabuli*	Ca_Kabuli_denovo	150097	(G/T)
17053	CakSNP17053	*Kabuli*	Ca_Kabuli_denovo	150098	(C/G)
17054	CakSNP17054	*Kabuli*	Ca_Kabuli_denovo	150669	(A/G)
17055	CakSNP17055	*Kabuli*	Ca_Kabuli_denovo	150688	(T/G)
17056	CakSNP17056	*Kabuli*	Ca_Kabuli_denovo	150703	(A/G)
17057	CakSNP17057	*Kabuli*	Ca_Kabuli_denovo	150706	(T/A)
17058	CakSNP17058	*Kabuli*	Ca_Kabuli_denovo	150720	(A/G)
17059	CakSNP17059	*Kabuli*	Ca_Kabuli_denovo	150730	(A/G)
17060	CakSNP17060	*Kabuli*	Ca_Kabuli_denovo	150734	(G/A)
17061	CakSNP17061	*Kabuli*	Ca_Kabuli_denovo	153852	(A/G)

S.N.	SNP IDs	Cultivars	Chromosomes/scaffolds	Physical positions (bp)	SNPs
17062	CakSNP17062	*Kabuli*	Ca_Kabuli_denovo	154095	(C/G)
17063	CakSNP17063	*Kabuli*	Ca_Kabuli_denovo	154096	(G/A)
17064	CakSNP17064	*Kabuli*	Ca_Kabuli_denovo	154097	(T/A)
17065	CakSNP17065	*Kabuli*	Ca_Kabuli_denovo	154098	(C/T)
17066	CakSNP17066	*Kabuli*	Ca_Kabuli_denovo	154640	(G/A)
17067	CakSNP17067	*Kabuli*	Ca_Kabuli_denovo	155679	(A/G)
17068	CakSNP17068	*Kabuli*	Ca_Kabuli_denovo	155681	(A/G)
17069	CakSNP17069	*Kabuli*	Ca_Kabuli_denovo	155683	(A/T)
17070	CakSNP17070	*Kabuli*	Ca_Kabuli_denovo	156229	(A/G)
17071	CakSNP17071	*Kabuli*	Ca_Kabuli_denovo	156322	(T/G)
17072	CakSNP17072	*Kabuli*	Ca_Kabuli_denovo	156323	(G/A)
17073	CakSNP17073	*Kabuli*	Ca_Kabuli_denovo	156324	(C/G)
17074	CakSNP17074	*Kabuli*	Ca_Kabuli_denovo	156325	(A/C)
17075	CakSNP17075	*Kabuli*	Ca_Kabuli_denovo	156492	(A/G)
17076	CakSNP17076	*Kabuli*	Ca_Kabuli_denovo	156493	(G/A)
17077	CakSNP17077	*Kabuli*	Ca_Kabuli_denovo	156494	(C/G)
17078	CakSNP17078	*Kabuli*	Ca_Kabuli_denovo	157819	(C/G)
17079	CakSNP17079	*Kabuli*	Ca_Kabuli_denovo	157821	(G/A)
17080	CakSNP17080	*Kabuli*	Ca_Kabuli_denovo	157822	(A/G)
17081	CakSNP17081	*Kabuli*	Ca_Kabuli_denovo	157997	(G/C)
17082	CakSNP17082	*Kabuli*	Ca_Kabuli_denovo	158850	(C/A)
17083	CakSNP17083	*Kabuli*	Ca_Kabuli_denovo	159015	(G/A)
17084	CakSNP17084	*Kabuli*	Ca_Kabuli_denovo	159016	(G/C)
17085	CakSNP17085	*Kabuli*	Ca_Kabuli_denovo	159017	(A/C)
17086	CakSNP17086	*Kabuli*	Ca_Kabuli_denovo	159018	(A/G)
17087	CakSNP17087	*Kabuli*	Ca_Kabuli_denovo	159019	(T/A)
17088	CakSNP17088	*Kabuli*	Ca_Kabuli_denovo	159020	(G/T)
17089	CakSNP17089	*Kabuli*	Ca_Kabuli_denovo	164804	(T/G)
17090	CakSNP17090	*Kabuli*	Ca_Kabuli_denovo	164847	(T/C)
17091	CakSNP17091	*Kabuli*	Ca_Kabuli_denovo	164877	(A/G)
17092	CakSNP17092	*Kabuli*	Ca_Kabuli_denovo	164886	(G/C)
17093	CakSNP17093	*Kabuli*	Ca_Kabuli_denovo	165283	(C/G)
17094	CakSNP17094	*Kabuli*	Ca_Kabuli_denovo	165296	(C/A)
17095	CakSNP17095	*Kabuli*	Ca_Kabuli_denovo	165324	(A/G)
17096	CakSNP17096	*Kabuli*	Ca_Kabuli_denovo	165355	(T/G)
17097	CakSNP17097	*Kabuli*	Ca_Kabuli_denovo	166177	(A/G)
17098	CakSNP17098	*Kabuli*	Ca_Kabuli_denovo	166179	(G/A)
17099	CakSNP17099	*Kabuli*	Ca_Kabuli_denovo	166181	(T/A)

S.N.	SNP IDs	Cultivars	Chromosomes/scaffolds	Physical positions (bp)	SNPs
17100	CakSNP17100	*Kabuli*	Ca_Kabuli_denovo	166736	(C/G)
17101	CakSNP17101	*Kabuli*	Ca_Kabuli_denovo	166737	(A/G)
17102	CakSNP17102	*Kabuli*	Ca_Kabuli_denovo	166738	(G/A)
17103	CakSNP17103	*Kabuli*	Ca_Kabuli_denovo	166758	(G/C)
17104	CakSNP17104	*Kabuli*	Ca_Kabuli_denovo	166767	(A/G)
17105	CakSNP17105	*Kabuli*	Ca_Kabuli_denovo	166786	(A/G)
17106	CakSNP17106	*Kabuli*	Ca_Kabuli_denovo	166809	(A/G)
17107	CakSNP17107	*Kabuli*	Ca_Kabuli_denovo	166828	(A/C)
17108	CakSNP17108	*Kabuli*	Ca_Kabuli_denovo	166836	(A/C)
17109	CakSNP17109	*Kabuli*	Ca_Kabuli_denovo	166843	(G/T)
17110	CakSNP17110	*Kabuli*	Ca_Kabuli_denovo	167563	(G/C)
17111	CakSNP17111	*Kabuli*	Ca_Kabuli_denovo	167564	(C/A)
17112	CakSNP17112	*Kabuli*	Ca_Kabuli_denovo	167565	(A/G)
17113	CakSNP17113	*Kabuli*	Ca_Kabuli_denovo	167567	(A/G)
17114	CakSNP17114	*Kabuli*	Ca_Kabuli_denovo	167569	(T/A)
17115	CakSNP17115	*Kabuli*	Ca_Kabuli_denovo	169044	(A/G)
17116	CakSNP17116	*Kabuli*	Ca_Kabuli_denovo	169046	(A/G)
17117	CakSNP17117	*Kabuli*	Ca_Kabuli_denovo	169047	(G/A)
17118	CakSNP17118	*Kabuli*	Ca_Kabuli_denovo	171930	(G/A)
17119	CakSNP17119	*Kabuli*	Ca_Kabuli_denovo	171932	(G/A)
17120	CakSNP17120	*Kabuli*	Ca_Kabuli_denovo	171933	(A/G)
17121	CakSNP17121	*Kabuli*	Ca_Kabuli_denovo	171983	(T/A)
17122	CakSNP17122	*Kabuli*	Ca_Kabuli_denovo	171999	(G/T)
17123	CakSNP17123	*Kabuli*	Ca_Kabuli_denovo	172017	(A/G)
17124	CakSNP17124	*Kabuli*	Ca_Kabuli_denovo	172035	(T/A)
17125	CakSNP17125	*Kabuli*	Ca_Kabuli_denovo	172044	(A/G)
17126	CakSNP17126	*Kabuli*	Ca_Kabuli_denovo	173451	(A/G)
17127	CakSNP17127	*Kabuli*	Ca_Kabuli_denovo	173462	(C/G)
17128	CakSNP17128	*Kabuli*	Ca_Kabuli_denovo	173472	(C/G)
17129	CakSNP17129	*Kabuli*	Ca_Kabuli_denovo	174632	(C/A)
17130	CakSNP17130	*Kabuli*	Ca_Kabuli_denovo	174633	(A/G)
17131	CakSNP17131	*Kabuli*	Ca_Kabuli_denovo	174635	(A/G)
17132	CakSNP17132	*Kabuli*	Ca_Kabuli_denovo	175138	(C/G)
17133	CakSNP17133	*Kabuli*	Ca_Kabuli_denovo	175160	(A/G)
17134	CakSNP17134	*Kabuli*	Ca_Kabuli_denovo	175170	(T/G)
17135	CakSNP17135	*Kabuli*	Ca_Kabuli_denovo	176799	(T/G)
17136	CakSNP17136	*Kabuli*	Ca_Kabuli_denovo	176814	(T/A)
17137	CakSNP17137	*Kabuli*	Ca_Kabuli_denovo	176822	(T/G)

S.N.	SNP IDs	Cultivars	Chromosomes/scaffolds	Physical positions (bp)	SNPs
17138	CakSNP17138	*Kabuli*	Ca_Kabuli_denovo	176824	(C/G)
17139	CakSNP17139	*Kabuli*	Ca_Kabuli_denovo	176831	(T/G)
17140	CakSNP17140	*Kabuli*	Ca_Kabuli_denovo	176835	(T/G)
17141	CakSNP17141	*Kabuli*	Ca_Kabuli_denovo	176837	(A/G)
17142	CakSNP17142	*Kabuli*	Ca_Kabuli_denovo	176839	(T/G)
17143	CakSNP17143	*Kabuli*	Ca_Kabuli_denovo	176874	(T/A)
17144	CakSNP17144	*Kabuli*	Ca_Kabuli_denovo	178198	(T/G)
17145	CakSNP17145	*Kabuli*	Ca_Kabuli_denovo	178200	(T/G)
17146	CakSNP17146	*Kabuli*	Ca_Kabuli_denovo	178205	(T/G)
17147	CakSNP17147	*Kabuli*	Ca_Kabuli_denovo	178212	(C/T)
17148	CakSNP17148	*Kabuli*	Ca_Kabuli_denovo	178217	(T/G)
17149	CakSNP17149	*Kabuli*	Ca_Kabuli_denovo	178238	(C/G)
17150	CakSNP17150	*Kabuli*	Ca_Kabuli_denovo	178243	(A/G)
17151	CakSNP17151	*Kabuli*	Ca_Kabuli_denovo	178249	(T/G)
17152	CakSNP17152	*Kabuli*	Ca_Kabuli_denovo	178251	(T/G)
17153	CakSNP17153	*Kabuli*	Ca_Kabuli_denovo	178276	(T/C)
17154	CakSNP17154	*Kabuli*	Ca_Kabuli_denovo	178736	(G/A)
17155	CakSNP17155	*Kabuli*	Ca_Kabuli_denovo	178737	(C/T)
17156	CakSNP17156	*Kabuli*	Ca_Kabuli_denovo	179759	(C/T)
17157	CakSNP17157	*Kabuli*	Ca_Kabuli_denovo	180307	(C/A)
17158	CakSNP17158	*Kabuli*	Ca_Kabuli_denovo	180309	(T/G)
17159	CakSNP17159	*Kabuli*	Ca_Kabuli_denovo	180571	(G/A)
17160	CakSNP17160	*Kabuli*	Ca_Kabuli_denovo	180573	(G/A)
17161	CakSNP17161	*Kabuli*	Ca_Kabuli_denovo	180686	(A/G)
17162	CakSNP17162	*Kabuli*	Ca_Kabuli_denovo	180689	(G/C)
17163	CakSNP17163	*Kabuli*	Ca_Kabuli_denovo	181636	(T/A)
17164	CakSNP17164	*Kabuli*	Ca_Kabuli_denovo	181639	(C/G)
17165	CakSNP17165	*Kabuli*	Ca_Kabuli_denovo	181670	(A/G)
17166	CakSNP17166	*Kabuli*	Ca_Kabuli_denovo	181675	(G/A)
17167	CakSNP17167	*Kabuli*	Ca_Kabuli_denovo	181679	(G/A)
17168	CakSNP17168	*Kabuli*	Ca_Kabuli_denovo	181698	(G/A)
17169	CakSNP17169	*Kabuli*	Ca_Kabuli_denovo	181720	(C/G)
17170	CakSNP17170	*Kabuli*	Ca_Kabuli_denovo	182277	(A/G)
17171	CakSNP17171	*Kabuli*	Ca_Kabuli_denovo	182279	(A/T)
17172	CakSNP17172	*Kabuli*	Ca_Kabuli_denovo	182280	(G/T)
17173	CakSNP17173	*Kabuli*	Ca_Kabuli_denovo	182281	(C/G)
17174	CakSNP17174	*Kabuli*	Ca_Kabuli_denovo	182628	(G/T)
17175	CakSNP17175	*Kabuli*	Ca_Kabuli_denovo	183987	(G/C)

S.N.	SNP IDs	Cultivars	Chromosomes/scaffolds	Physical positions (bp)	SNPs
17176	CakSNP17176	*Kabuli*	Ca_Kabuli_denovo	183990	(A/G)
17177	CakSNP17177	*Kabuli*	Ca_Kabuli_denovo	184118	(G/C)
17178	CakSNP17178	*Kabuli*	Ca_Kabuli_denovo	184181	(T/G)
17179	CakSNP17179	*Kabuli*	Ca_Kabuli_denovo	184211	(T/G)
17180	CakSNP17180	*Kabuli*	Ca_Kabuli_denovo	184231	(C/A)
17181	CakSNP17181	*Kabuli*	Ca_Kabuli_denovo	184333	(C/G)
17182	CakSNP17182	*Kabuli*	Ca_Kabuli_denovo	184359	(G/T)
17183	CakSNP17183	*Kabuli*	Ca_Kabuli_denovo	184367	(T/G)
17184	CakSNP17184	*Kabuli*	Ca_Kabuli_denovo	184370	(C/G)
17185	CakSNP17185	*Kabuli*	Ca_Kabuli_denovo	184372	(C/G)
17186	CakSNP17186	*Kabuli*	Ca_Kabuli_denovo	184427	(C/G)
17187	CakSNP17187	*Kabuli*	Ca_Kabuli_denovo	184440	(C/G)
17188	CakSNP17188	*Kabuli*	Ca_Kabuli_denovo	184460	(T/G)
17189	CakSNP17189	*Kabuli*	Ca_Kabuli_denovo	184487	(C/A)
17190	CakSNP17190	*Kabuli*	Ca_Kabuli_denovo	184509	(C/T)
17191	CakSNP17191	*Kabuli*	Ca_Kabuli_denovo	184521	(T/A)
17192	CakSNP17192	*Kabuli*	Ca_Kabuli_denovo	184541	(C/G)
17193	CakSNP17193	*Kabuli*	Ca_Kabuli_denovo	184553	(T/G)
17194	CakSNP17194	*Kabuli*	Ca_Kabuli_denovo	184583	(T/G)
17195	CakSNP17195	*Kabuli*	Ca_Kabuli_denovo	185564	(C/G)
17196	CakSNP17196	*Kabuli*	Ca_Kabuli_denovo	185568	(T/G)
17197	CakSNP17197	*Kabuli*	Ca_Kabuli_denovo	185573	(G/A)
17198	CakSNP17198	*Kabuli*	Ca_Kabuli_denovo	185577	(T/G)
17199	CakSNP17199	*Kabuli*	Ca_Kabuli_denovo	185579	(A/G)
17200	CakSNP17200	*Kabuli*	Ca_Kabuli_denovo	185583	(A/G)
17201	CakSNP17201	*Kabuli*	Ca_Kabuli_denovo	185634	(A/G)
17202	CakSNP17202	*Kabuli*	Ca_Kabuli_denovo	185645	(C/G)
17203	CakSNP17203	*Kabuli*	Ca_Kabuli_denovo	185653	(A/G)
17204	CakSNP17204	*Kabuli*	Ca_Kabuli_denovo	185657	(C/G)
17205	CakSNP17205	*Kabuli*	Ca_Kabuli_denovo	185669	(T/G)
17206	CakSNP17206	*Kabuli*	Ca_Kabuli_denovo	185674	(C/G)
17207	CakSNP17207	*Kabuli*	Ca_Kabuli_denovo	185679	(T/A)
17208	CakSNP17208	*Kabuli*	Ca_Kabuli_denovo	185682	(T/G)
17209	CakSNP17209	*Kabuli*	Ca_Kabuli_denovo	187188	(A/G)
17210	CakSNP17210	*Kabuli*	Ca_Kabuli_denovo	188302	(G/A)
17211	CakSNP17211	*Kabuli*	Ca_Kabuli_denovo	188305	(C/A)
17212	CakSNP17212	*Kabuli*	Ca_Kabuli_denovo	188306	(A/G)
17213	CakSNP17213	*Kabuli*	Ca_Kabuli_denovo	188308	(C/A)

S.N.	SNP IDs	Cultivars	Chromosomes/scaffolds	Physical positions (bp)	SNPs
17214	CakSNP17214	*Kabuli*	Ca_Kabuli_denovo	188310	(G/T)
17215	CakSNP17215	*Kabuli*	Ca_Kabuli_denovo	188312	(A/C)
17216	CakSNP17216	*Kabuli*	Ca_Kabuli_denovo	188313	(C/A)
17217	CakSNP17217	*Kabuli*	Ca_Kabuli_denovo	190388	(C/G)
17218	CakSNP17218	*Kabuli*	Ca_Kabuli_denovo	190404	(C/G)
17219	CakSNP17219	*Kabuli*	Ca_Kabuli_denovo	194914	(A/G)
17220	CakSNP17220	*Kabuli*	Ca_Kabuli_denovo	194915	(C/G)
17221	CakSNP17221	*Kabuli*	Ca_Kabuli_denovo	195589	(A/G)
17222	CakSNP17222	*Kabuli*	Ca_Kabuli_denovo	195605	(C/G)
17223	CakSNP17223	*Kabuli*	Ca_Kabuli_denovo	195642	(C/G)
17224	CakSNP17224	*Kabuli*	Ca_Kabuli_denovo	195663	(T/A)
17225	CakSNP17225	*Kabuli*	Ca_Kabuli_denovo	195669	(T/G)
17226	CakSNP17226	*Kabuli*	Ca_Kabuli_denovo	196773	(C/G)
17227	CakSNP17227	*Kabuli*	Ca_Kabuli_denovo	196775	(T/G)
17228	CakSNP17228	*Kabuli*	Ca_Kabuli_denovo	196777	(C/T)
17229	CakSNP17229	*Kabuli*	Ca_Kabuli_denovo	197792	(A/G)
17230	CakSNP17230	*Kabuli*	Ca_Kabuli_denovo	197793	(G/C)
17231	CakSNP17231	*Kabuli*	Ca_Kabuli_denovo	197795	(A/G)
17232	CakSNP17232	*Kabuli*	Ca_Kabuli_denovo	197796	(G/A)
17233	CakSNP17233	*Kabuli*	Ca_Kabuli_denovo	197799	(C/A)
17234	CakSNP17234	*Kabuli*	Ca_Kabuli_denovo	197800	(C/T)
17235	CakSNP17235	*Kabuli*	Ca_Kabuli_denovo	197802	(A/C)
17236	CakSNP17236	*Kabuli*	Ca_Kabuli_denovo	197991	(C/G)
17237	CakSNP17237	*Kabuli*	Ca_Kabuli_denovo	197992	(G/A)
17238	CakSNP17238	*Kabuli*	Ca_Kabuli_denovo	197993	(A/T)
17239	CakSNP17239	*Kabuli*	Ca_Kabuli_denovo	197994	(C/T)
17240	CakSNP17240	*Kabuli*	Ca_Kabuli_denovo	197995	(T/C)
17241	CakSNP17241	*Kabuli*	Ca_Kabuli_denovo	197996	(C/T)
17242	CakSNP17242	*Kabuli*	Ca_Kabuli_denovo	198162	(T/G)
17243	CakSNP17243	*Kabuli*	Ca_Kabuli_denovo	198166	(C/G)
17244	CakSNP17244	*Kabuli*	Ca_Kabuli_denovo	198168	(A/G)
17245	CakSNP17245	*Kabuli*	Ca_Kabuli_denovo	198170	(G/A)
17246	CakSNP17246	*Kabuli*	Ca_Kabuli_denovo	198171	(C/A)
17247	CakSNP17247	*Kabuli*	Ca_Kabuli_denovo	198358	(T/G)
17248	CakSNP17248	*Kabuli*	Ca_Kabuli_denovo	198365	(A/G)
17249	CakSNP17249	*Kabuli*	Ca_Kabuli_denovo	198366	(C/A)
17250	CakSNP17250	*Kabuli*	Ca_Kabuli_denovo	198367	(C/T)
17251	CakSNP17251	*Kabuli*	Ca_Kabuli_denovo	198370	(C/T)

S.N.	SNP IDs	Cultivars	Chromosomes/scaffolds	Physical positions (bp)	SNPs
17252	CakSNP17252	*Kabuli*	Ca_Kabuli_denovo	198431	(G/A)
17253	CakSNP17253	*Kabuli*	Ca_Kabuli_denovo	198433	(A/G)
17254	CakSNP17254	*Kabuli*	Ca_Kabuli_denovo	198434	(G/A)
17255	CakSNP17255	*Kabuli*	Ca_Kabuli_denovo	198435	(A/G)
17256	CakSNP17256	*Kabuli*	Ca_Kabuli_denovo	198436	(G/C)
17257	CakSNP17257	*Kabuli*	Ca_Kabuli_denovo	198437	(C/G)
17258	CakSNP17258	*Kabuli*	Ca_Kabuli_denovo	198439	(G/T)
17259	CakSNP17259	*Kabuli*	Ca_Kabuli_denovo	198441	(T/C)
17260	CakSNP17260	*Kabuli*	Ca_Kabuli_denovo	198442	(C/A)
17261	CakSNP17261	*Kabuli*	Ca_Kabuli_denovo	198443	(A/G)
17262	CakSNP17262	*Kabuli*	Ca_Kabuli_denovo	198444	(G/C)
17263	CakSNP17263	*Kabuli*	Ca_Kabuli_denovo	198445	(C/A)
17264	CakSNP17264	*Kabuli*	Ca_Kabuli_denovo	198446	(A/G)
17265	CakSNP17265	*Kabuli*	Ca_Kabuli_denovo	198448	(G/A)
17266	CakSNP17266	*Kabuli*	Ca_Kabuli_denovo	198450	(A/T)
17267	CakSNP17267	*Kabuli*	Ca_Kabuli_denovo	198451	(T/G)
17268	CakSNP17268	*Kabuli*	Ca_Kabuli_denovo	198452	(G/C)
17269	CakSNP17269	*Kabuli*	Ca_Kabuli_denovo	198454	(C/G)
17270	CakSNP17270	*Kabuli*	Ca_Kabuli_denovo	198455	(G/A)
17271	CakSNP17271	*Kabuli*	Ca_Kabuli_denovo	198456	(A/G)
17272	CakSNP17272	*Kabuli*	Ca_Kabuli_denovo	198457	(G/A)
17273	CakSNP17273	*Kabuli*	Ca_Kabuli_denovo	198458	(A/C)
17274	CakSNP17274	*Kabuli*	Ca_Kabuli_denovo	198842	(T/G)
17275	CakSNP17275	*Kabuli*	Ca_Kabuli_denovo	199140	(C/A)
17276	CakSNP17276	*Kabuli*	Ca_Kabuli_denovo	199240	(T/C)
17277	CakSNP17277	*Kabuli*	Ca_Kabuli_denovo	199747	(C/G)
17278	CakSNP17278	*Kabuli*	Ca_Kabuli_denovo	200458	(A/G)
17279	CakSNP17279	*Kabuli*	Ca_Kabuli_denovo	200459	(T/G)
17280	CakSNP17280	*Kabuli*	Ca_Kabuli_denovo	200728	(T/C)
17281	CakSNP17281	*Kabuli*	Ca_Kabuli_denovo	200743	(C/G)
17282	CakSNP17282	*Kabuli*	Ca_Kabuli_denovo	200890	(T/A)
17283	CakSNP17283	*Kabuli*	Ca_Kabuli_denovo	200910	(G/A)
17284	CakSNP17284	*Kabuli*	Ca_Kabuli_denovo	201180	(G/T)
17285	CakSNP17285	*Kabuli*	Ca_Kabuli_denovo	201201	(A/G)
17286	CakSNP17286	*Kabuli*	Ca_Kabuli_denovo	201287	(A/C)
17287	CakSNP17287	*Kabuli*	Ca_Kabuli_denovo	201365	(A/T)
17288	CakSNP17288	*Kabuli*	Ca_Kabuli_denovo	201444	(T/G)
17289	CakSNP17289	*Kabuli*	Ca_Kabuli_denovo	201979	(G/A)

S.N.	SNP IDs	Cultivars	Chromosomes/scaffolds	Physical positions (bp)	SNPs
17290	CakSNP17290	*Kabuli*	Ca_Kabuli_denovo	201981	(G/A)
17291	CakSNP17291	*Kabuli*	Ca_Kabuli_denovo	201982	(A/G)
17292	CakSNP17292	*Kabuli*	Ca_Kabuli_denovo	202419	(T/G)
17293	CakSNP17293	*Kabuli*	Ca_Kabuli_denovo	202456	(T/G)
17294	CakSNP17294	*Kabuli*	Ca_Kabuli_denovo	202459	(C/T)
17295	CakSNP17295	*Kabuli*	Ca_Kabuli_denovo	202460	(G/C)
17296	CakSNP17296	*Kabuli*	Ca_Kabuli_denovo	202461	(T/G)
17297	CakSNP17297	*Kabuli*	Ca_Kabuli_denovo	202523	(G/A)
17298	CakSNP17298	*Kabuli*	Ca_Kabuli_denovo	202525	(A/G)
17299	CakSNP17299	*Kabuli*	Ca_Kabuli_denovo	202526	(G/A)
17300	CakSNP17300	*Kabuli*	Ca_Kabuli_denovo	202527	(A/G)
17301	CakSNP17301	*Kabuli*	Ca_Kabuli_denovo	202529	(C/G)
17302	CakSNP17302	*Kabuli*	Ca_Kabuli_denovo	202531	(G/T)
17303	CakSNP17303	*Kabuli*	Ca_Kabuli_denovo	202533	(T/C)
17304	CakSNP17304	*Kabuli*	Ca_Kabuli_denovo	202534	(C/A)
17305	CakSNP17305	*Kabuli*	Ca_Kabuli_denovo	202535	(A/G)
17306	CakSNP17306	*Kabuli*	Ca_Kabuli_denovo	202536	(G/C)
17307	CakSNP17307	*Kabuli*	Ca_Kabuli_denovo	202537	(C/A)
17308	CakSNP17308	*Kabuli*	Ca_Kabuli_denovo	202538	(A/G)
17309	CakSNP17309	*Kabuli*	Ca_Kabuli_denovo	202540	(G/A)
17310	CakSNP17310	*Kabuli*	Ca_Kabuli_denovo	202542	(A/T)
17311	CakSNP17311	*Kabuli*	Ca_Kabuli_denovo	202544	(G/C)
17312	CakSNP17312	*Kabuli*	Ca_Kabuli_denovo	202546	(C/G)
17313	CakSNP17313	*Kabuli*	Ca_Kabuli_denovo	202547	(G/A)
17314	CakSNP17314	*Kabuli*	Ca_Kabuli_denovo	202548	(A/G)
17315	CakSNP17315	*Kabuli*	Ca_Kabuli_denovo	202549	(G/A)
17316	CakSNP17316	*Kabuli*	Ca_Kabuli_denovo	202550	(A/T)
17317	CakSNP17317	*Kabuli*	Ca_Kabuli_denovo	202551	(C/A)
17318	CakSNP17318	*Kabuli*	Ca_Kabuli_denovo	202553	(C/G)
17319	CakSNP17319	*Kabuli*	Ca_Kabuli_denovo	204292	(A/G)
17320	CakSNP17320	*Kabuli*	Ca_Kabuli_denovo	204293	(G/C)
17321	CakSNP17321	*Kabuli*	Ca_Kabuli_denovo	207676	(T/A)
17322	CakSNP17322	*Kabuli*	Ca_Kabuli_denovo	207681	(T/G)
17323	CakSNP17323	*Kabuli*	Ca_Kabuli_denovo	207690	(C/G)
17324	CakSNP17324	*Kabuli*	Ca_Kabuli_denovo	207698	(A/G)
17325	CakSNP17325	*Kabuli*	Ca_Kabuli_denovo	207703	(T/A)
17326	CakSNP17326	*Kabuli*	Ca_Kabuli_denovo	207712	(C/G)
17327	CakSNP17327	*Kabuli*	Ca_Kabuli_denovo	207716	(C/G)

S.N.	SNP IDs	Cultivars	Chromosomes/scaffolds	Physical positions (bp)	SNPs
17328	CakSNP17328	*Kabuli*	Ca_Kabuli_denovo	207737	(T/G)
17329	CakSNP17329	*Kabuli*	Ca_Kabuli_denovo	208035	(G/A)
17330	CakSNP17330	*Kabuli*	Ca_Kabuli_denovo	208036	(A/T)
17331	CakSNP17331	*Kabuli*	Ca_Kabuli_denovo	208037	(C/T)
17332	CakSNP17332	*Kabuli*	Ca_Kabuli_denovo	208042	(A/T)
17333	CakSNP17333	*Kabuli*	Ca_Kabuli_denovo	209330	(A/G)
17334	CakSNP17334	*Kabuli*	Ca_Kabuli_denovo	209332	(C/T)
17335	CakSNP17335	*Kabuli*	Ca_Kabuli_denovo	209333	(C/G)
17336	CakSNP17336	*Kabuli*	Ca_Kabuli_denovo	209334	(G/C)
17337	CakSNP17337	*Kabuli*	Ca_Kabuli_denovo	210208	(A/T)
17338	CakSNP17338	*Kabuli*	Ca_Kabuli_denovo	210216	(T/C)
17339	CakSNP17339	*Kabuli*	Ca_Kabuli_denovo	210225	(G/A)
17340	CakSNP17340	*Kabuli*	Ca_Kabuli_denovo	210237	(A/T)
17341	CakSNP17341	*Kabuli*	Ca_Kabuli_denovo	215170	(G/A)
17342	CakSNP17342	*Kabuli*	Ca_Kabuli_denovo	215678	(T/G)
17343	CakSNP17343	*Kabuli*	Ca_Kabuli_denovo	215689	(C/A)
17344	CakSNP17344	*Kabuli*	Ca_Kabuli_denovo	215699	(A/G)
17345	CakSNP17345	*Kabuli*	Ca_Kabuli_denovo	215705	(T/G)
17346	CakSNP17346	*Kabuli*	Ca_Kabuli_denovo	215723	(A/G)
17347	CakSNP17347	*Kabuli*	Ca_Kabuli_denovo	215731	(C/G)
17348	CakSNP17348	*Kabuli*	Ca_Kabuli_denovo	215752	(G/A)
17349	CakSNP17349	*Kabuli*	Ca_Kabuli_denovo	215757	(G/T)
17350	CakSNP17350	*Kabuli*	Ca_Kabuli_denovo	217228	(C/A)
17351	CakSNP17351	*Kabuli*	Ca_Kabuli_denovo	217229	(A/T)
17352	CakSNP17352	*Kabuli*	Ca_Kabuli_denovo	217232	(C/A)
17353	CakSNP17353	*Kabuli*	Ca_Kabuli_denovo	220674	(C/G)
17354	CakSNP17354	*Kabuli*	Ca_Kabuli_denovo	220676	(T/G)
17355	CakSNP17355	*Kabuli*	Ca_Kabuli_denovo	220678	(C/T)
17356	CakSNP17356	*Kabuli*	Ca_Kabuli_denovo	220679	(A/C)
17357	CakSNP17357	*Kabuli*	Ca_Kabuli_denovo	222258	(C/G)
17358	CakSNP17358	*Kabuli*	Ca_Kabuli_denovo	222260	(T/G)
17359	CakSNP17359	*Kabuli*	Ca_Kabuli_denovo	222262	(C/T)
17360	CakSNP17360	*Kabuli*	Ca_Kabuli_denovo	223208	(C/A)
17361	CakSNP17361	*Kabuli*	Ca_Kabuli_denovo	223209	(T/A)
17362	CakSNP17362	*Kabuli*	Ca_Kabuli_denovo	223211	(C/G)
17363	CakSNP17363	*Kabuli*	Ca_Kabuli_denovo	223224	(G/A)
17364	CakSNP17364	*Kabuli*	Ca_Kabuli_denovo	223241	(T/G)
17365	CakSNP17365	*Kabuli*	Ca_Kabuli_denovo	223243	(A/G)

S.N.	SNP IDs	Cultivars	Chromosomes/scaffolds	Physical positions (bp)	SNPs
17366	CakSNP17366	*Kabuli*	Ca_Kabuli_denovo	223248	(A/C)
17367	CakSNP17367	*Kabuli*	Ca_Kabuli_denovo	223251	(T/G)
17368	CakSNP17368	*Kabuli*	Ca_Kabuli_denovo	223258	(A/G)
17369	CakSNP17369	*Kabuli*	Ca_Kabuli_denovo	223261	(G/A)
17370	CakSNP17370	*Kabuli*	Ca_Kabuli_denovo	224544	(T/C)
17371	CakSNP17371	*Kabuli*	Ca_Kabuli_denovo	224555	(G/A)
17372	CakSNP17372	*Kabuli*	Ca_Kabuli_denovo	224557	(T/C)
17373	CakSNP17373	*Kabuli*	Ca_Kabuli_denovo	224865	(C/A)
17374	CakSNP17374	*Kabuli*	Ca_Kabuli_denovo	224867	(C/G)
17375	CakSNP17375	*Kabuli*	Ca_Kabuli_denovo	224868	(G/A)
17376	CakSNP17376	*Kabuli*	Ca_Kabuli_denovo	225042	(A/G)
17377	CakSNP17377	*Kabuli*	Ca_Kabuli_denovo	225043	(A/G)
17378	CakSNP17378	*Kabuli*	Ca_Kabuli_denovo	225408	(A/C)
17379	CakSNP17379	*Kabuli*	Ca_Kabuli_denovo	225409	(C/A)
17380	CakSNP17380	*Kabuli*	Ca_Kabuli_denovo	225414	(T/A)
17381	CakSNP17381	*Kabuli*	Ca_Kabuli_denovo	225417	(G/T)
17382	CakSNP17382	*Kabuli*	Ca_Kabuli_denovo	225422	(G/C)
17383	CakSNP17383	*Kabuli*	Ca_Kabuli_denovo	225425	(T/G)
17384	CakSNP17384	*Kabuli*	Ca_Kabuli_denovo	230711	(G/T)
17385	CakSNP17385	*Kabuli*	Ca_Kabuli_denovo	230712	(C/T)
17386	CakSNP17386	*Kabuli*	Ca_Kabuli_denovo	232397	(G/C)
17387	CakSNP17387	*Kabuli*	Ca_Kabuli_denovo	232398	(C/G)
17388	CakSNP17388	*Kabuli*	Ca_Kabuli_denovo	232399	(C/A)
17389	CakSNP17389	*Kabuli*	Ca_Kabuli_denovo	232400	(T/G)
17390	CakSNP17390	*Kabuli*	Ca_Kabuli_denovo	232401	(C/A)
17391	CakSNP17391	*Kabuli*	Ca_Kabuli_denovo	235464	(C/A)
17392	CakSNP17392	*Kabuli*	Ca_Kabuli_denovo	238447	(A/G)
17393	CakSNP17393	*Kabuli*	Ca_Kabuli_denovo	238449	(A/G)
17394	CakSNP17394	*Kabuli*	Ca_Kabuli_denovo	238451	(T/A)
17395	CakSNP17395	*Kabuli*	Ca_Kabuli_denovo	238452	(G/T)
17396	CakSNP17396	*Kabuli*	Ca_Kabuli_denovo	239728	(G/T)
17397	CakSNP17397	*Kabuli*	Ca_Kabuli_denovo	240943	(C/T)
17398	CakSNP17398	*Kabuli*	Ca_Kabuli_denovo	248697	(A/C)
17399	CakSNP17399	*Kabuli*	Ca_Kabuli_denovo	248719	(T/C)
17400	CakSNP17400	*Kabuli*	Ca_Kabuli_denovo	248775	(T/G)
17401	CakSNP17401	*Kabuli*	Ca_Kabuli_denovo	256228	(A/G)
17402	CakSNP17402	*Kabuli*	Ca_Kabuli_denovo	256262	(T/G)
17403	CakSNP17403	*Kabuli*	Ca_Kabuli_denovo	256272	(G/A)

S.N.	SNP IDs	Cultivars	Chromosomes/scaffolds	Physical positions (bp)	SNPs
17404	CakSNP17404	*Kabuli*	Ca_Kabuli_denovo	264802	(G/C)
17405	CakSNP17405	*Kabuli*	Ca_Kabuli_denovo	264812	(T/G)
17406	CakSNP17406	*Kabuli*	Ca_Kabuli_denovo	264815	(C/G)
17407	CakSNP17407	*Kabuli*	Ca_Kabuli_denovo	264817	(G/C)
17408	CakSNP17408	*Kabuli*	Ca_Kabuli_denovo	264819	(A/G)
17409	CakSNP17409	*Kabuli*	Ca_Kabuli_denovo	265972	(A/G)
17410	CakSNP17410	*Kabuli*	Ca_Kabuli_denovo	268126	(G/T)
17411	CakSNP17411	*Kabuli*	Ca_Kabuli_denovo	268171	(T/C)
17412	CakSNP17412	*Kabuli*	Ca_Kabuli_denovo	273531	(A/G)
17413	CakSNP17413	*Kabuli*	Ca_Kabuli_denovo	273541	(C/G)
17414	CakSNP17414	*Kabuli*	Ca_Kabuli_denovo	273543	(G/C)
17415	CakSNP17415	*Kabuli*	Ca_Kabuli_denovo	275658	(T/G)
17416	CakSNP17416	*Kabuli*	Ca_Kabuli_denovo	275671	(A/G)
17417	CakSNP17417	*Kabuli*	Ca_Kabuli_denovo	275698	(T/G)
17418	CakSNP17418	*Kabuli*	Ca_Kabuli_denovo	281765	(C/G)
17419	CakSNP17419	*Kabuli*	Ca_Kabuli_denovo	281766	(G/C)
17420	CakSNP17420	*Kabuli*	Ca_Kabuli_denovo	281767	(G/A)
17421	CakSNP17421	*Kabuli*	Ca_Kabuli_denovo	281768	(T/G)
17422	CakSNP17422	*Kabuli*	Ca_Kabuli_denovo	281769	(T/G)
17423	CakSNP17423	*Kabuli*	Ca_Kabuli_denovo	281770	(A/C)
17424	CakSNP17424	*Kabuli*	Ca_Kabuli_denovo	290341	(T/A)
17425	CakSNP17425	*Kabuli*	Ca_Kabuli_denovo	290342	(T/G)
17426	CakSNP17426	*Kabuli*	Ca_Kabuli_denovo	290344	(C/T)
17427	CakSNP17427	*Kabuli*	Ca_Kabuli_denovo	290345	(G/T)
17428	CakSNP17428	*Kabuli*	Ca_Kabuli_denovo	299355	(A/T)
17429	CakSNP17429	*Kabuli*	Ca_Kabuli_denovo	299356	(C/T)
17430	CakSNP17430	*Kabuli*	Ca_Kabuli_denovo	300954	(A/G)
17431	CakSNP17431	*Kabuli*	Ca_Kabuli_denovo	300963	(A/G)
17432	CakSNP17432	*Kabuli*	Ca_Kabuli_denovo	300979	(A/G)
17433	CakSNP17433	*Kabuli*	Ca_Kabuli_denovo	300980	(T/G)
17434	CakSNP17434	*Kabuli*	Ca_Kabuli_denovo	300982	(T/G)
17435	CakSNP17435	*Kabuli*	Ca_Kabuli_denovo	300994	(A/G)
17436	CakSNP17436	*Kabuli*	Ca_Kabuli_denovo	301008	(A/G)
17437	CakSNP17437	*Kabuli*	Ca_Kabuli_denovo	301011	(C/G)
17438	CakSNP17438	*Kabuli*	Ca_Kabuli_denovo	301012	(C/G)
17439	CakSNP17439	*Kabuli*	Ca_Kabuli_denovo	301021	(A/G)
17440	CakSNP17440	*Kabuli*	Ca_Kabuli_denovo	301027	(A/G)
17441	CakSNP17441	*Kabuli*	Ca_Kabuli_denovo	301037	(A/G)

S.N.	SNP IDs	Cultivars	Chromosomes/scaffolds	Physical positions (bp)	SNPs
17442	CakSNP17442	*Kabuli*	Ca_Kabuli_denovo	305833	(T/G)
17443	CakSNP17443	*Kabuli*	Ca_Kabuli_denovo	309250	(C/G)
17444	CakSNP17444	*Kabuli*	Ca_Kabuli_denovo	309283	(A/G)
17445	CakSNP17445	*Kabuli*	Ca_Kabuli_denovo	309293	(C/G)
17446	CakSNP17446	*Kabuli*	Ca_Kabuli_denovo	312821	(A/G)
17447	CakSNP17447	*Kabuli*	Ca_Kabuli_denovo	313026	(T/G)
17448	CakSNP17448	*Kabuli*	Ca_Kabuli_denovo	313030	(G/C)
17449	CakSNP17449	*Kabuli*	Ca_Kabuli_denovo	313032	(A/G)
17450	CakSNP17450	*Kabuli*	Ca_Kabuli_denovo	313033	(T/G)
17451	CakSNP17451	*Kabuli*	Ca_Kabuli_denovo	313034	(G/A)
17452	CakSNP17452	*Kabuli*	Ca_Kabuli_denovo	315534	(G/C)
17453	CakSNP17453	*Kabuli*	Ca_Kabuli_denovo	315535	(C/G)
17454	CakSNP17454	*Kabuli*	Ca_Kabuli_denovo	316184	(G/C)
17455	CakSNP17455	*Kabuli*	Ca_Kabuli_denovo	319444	(A/G)
17456	CakSNP17456	*Kabuli*	Ca_Kabuli_denovo	324057	(T/G)
17457	CakSNP17457	*Kabuli*	Ca_Kabuli_denovo	325498	(A/G)
17458	CakSNP17458	*Kabuli*	Ca_Kabuli_denovo	325500	(G/A)
17459	CakSNP17459	*Kabuli*	Ca_Kabuli_denovo	327160	(G/A)
17460	CakSNP17460	*Kabuli*	Ca_Kabuli_denovo	327161	(A/T)
17461	CakSNP17461	*Kabuli*	Ca_Kabuli_denovo	327162	(G/A)
17462	CakSNP17462	*Kabuli*	Ca_Kabuli_denovo	328553	(A/G)
17463	CakSNP17463	*Kabuli*	Ca_Kabuli_denovo	328554	(A/G)
17464	CakSNP17464	*Kabuli*	Ca_Kabuli_denovo	328555	(G/T)
17465	CakSNP17465	*Kabuli*	Ca_Kabuli_denovo	328556	(A/C)
17466	CakSNP17466	*Kabuli*	Ca_Kabuli_denovo	328867	(G/A)
17467	CakSNP17467	*Kabuli*	Ca_Kabuli_denovo	328887	(A/G)
17468	CakSNP17468	*Kabuli*	Ca_Kabuli_denovo	328899	(A/C)
17469	CakSNP17469	*Kabuli*	Ca_Kabuli_denovo	328908	(C/A)
17470	CakSNP17470	*Kabuli*	Ca_Kabuli_denovo	329973	(C/A)
17471	CakSNP17471	*Kabuli*	Ca_Kabuli_denovo	329977	(A/G)
17472	CakSNP17472	*Kabuli*	Ca_Kabuli_denovo	329986	(A/G)
17473	CakSNP17473	*Kabuli*	Ca_Kabuli_denovo	329993	(T/G)
17474	CakSNP17474	*Kabuli*	Ca_Kabuli_denovo	329999	(G/A)
17475	CakSNP17475	*Kabuli*	Ca_Kabuli_denovo	330000	(A/G)
17476	CakSNP17476	*Kabuli*	Ca_Kabuli_denovo	332747	(G/T)
17477	CakSNP17477	*Kabuli*	Ca_Kabuli_denovo	332748	(A/C)
17478	CakSNP17478	*Kabuli*	Ca_Kabuli_denovo	332751	(G/C)
17479	CakSNP17479	*Kabuli*	Ca_Kabuli_denovo	332755	(G/C)

S.N.	SNP IDs	Cultivars	Chromosomes/scaffolds	Physical positions (bp)	SNPs
17480	CakSNP17480	*Kabuli*	Ca_Kabuli_denovo	337250	(T/C)
17481	CakSNP17481	*Kabuli*	Ca_Kabuli_denovo	337257	(T/G)
17482	CakSNP17482	*Kabuli*	Ca_Kabuli_denovo	337269	(T/G)
17483	CakSNP17483	*Kabuli*	Ca_Kabuli_denovo	337289	(T/G)
17484	CakSNP17484	*Kabuli*	Ca_Kabuli_denovo	337405	(T/C)
17485	CakSNP17485	*Kabuli*	Ca_Kabuli_denovo	341733	(T/G)
17486	CakSNP17486	*Kabuli*	Ca_Kabuli_denovo	341774	(A/G)
17487	CakSNP17487	*Kabuli*	Ca_Kabuli_denovo	348683	(C/A)
17488	CakSNP17488	*Kabuli*	Ca_Kabuli_denovo	348717	(C/G)
17489	CakSNP17489	*Kabuli*	Ca_Kabuli_denovo	352430	(T/G)
17490	CakSNP17490	*Kabuli*	Ca_Kabuli_denovo	352452	(T/G)
17491	CakSNP17491	*Kabuli*	Ca_Kabuli_denovo	352471	(C/A)
17492	CakSNP17492	*Kabuli*	Ca_Kabuli_denovo	356259	(G/A)
17493	CakSNP17493	*Kabuli*	Ca_Kabuli_denovo	356260	(G/A)
17494	CakSNP17494	*Kabuli*	Ca_Kabuli_denovo	356262	(T/G)
17495	CakSNP17495	*Kabuli*	Ca_Kabuli_denovo	356264	(A/C)
17496	CakSNP17496	*Kabuli*	Ca_Kabuli_denovo	356266	(A/C)
17497	CakSNP17497	*Kabuli*	Ca_Kabuli_denovo	356835	(G/A)
17498	CakSNP17498	*Kabuli*	Ca_Kabuli_denovo	356836	(T/A)
17499	CakSNP17499	*Kabuli*	Ca_Kabuli_denovo	357513	(C/A)
17500	CakSNP17500	*Kabuli*	Ca_Kabuli_denovo	357533	(T/G)
17501	CakSNP17501	*Kabuli*	Ca_Kabuli_denovo	357578	(G/C)
17502	CakSNP17502	*Kabuli*	Ca_Kabuli_denovo	357580	(G/A)
17503	CakSNP17503	*Kabuli*	Ca_Kabuli_denovo	357581	(A/T)
17504	CakSNP17504	*Kabuli*	Ca_Kabuli_denovo	357582	(C/A)
17505	CakSNP17505	*Kabuli*	Ca_Kabuli_denovo	357583	(T/C)
17506	CakSNP17506	*Kabuli*	Ca_Kabuli_denovo	357584	(C/G)
17507	CakSNP17507	*Kabuli*	Ca_Kabuli_denovo	357585	(G/T)
17508	CakSNP17508	*Kabuli*	Ca_Kabuli_denovo	357586	(T/C)
17509	CakSNP17509	*Kabuli*	Ca_Kabuli_denovo	358284	(T/G)
17510	CakSNP17510	*Kabuli*	Ca_Kabuli_denovo	358878	(C/G)
17511	CakSNP17511	*Kabuli*	Ca_Kabuli_denovo	358879	(A/C)
17512	CakSNP17512	*Kabuli*	Ca_Kabuli_denovo	358882	(C/G)
17513	CakSNP17513	*Kabuli*	Ca_Kabuli_denovo	359691	(C/G)
17514	CakSNP17514	*Kabuli*	Ca_Kabuli_denovo	359693	(G/A)
17515	CakSNP17515	*Kabuli*	Ca_Kabuli_denovo	359695	(A/G)
17516	CakSNP17516	*Kabuli*	Ca_Kabuli_denovo	359696	(G/A)
17517	CakSNP17517	*Kabuli*	Ca_Kabuli_denovo	359697	(A/G)

S.N.	SNP IDs	Cultivars	Chromosomes/scaffolds	Physical positions (bp)	SNPs
17518	CakSNP17518	*Kabuli*	Ca_Kabuli_denovo	359698	(G/C)
17519	CakSNP17519	*Kabuli*	Ca_Kabuli_denovo	359699	(C/G)
17520	CakSNP17520	*Kabuli*	Ca_Kabuli_denovo	359701	(G/T)
17521	CakSNP17521	*Kabuli*	Ca_Kabuli_denovo	359703	(T/C)
17522	CakSNP17522	*Kabuli*	Ca_Kabuli_denovo	359704	(C/A)
17523	CakSNP17523	*Kabuli*	Ca_Kabuli_denovo	359705	(A/G)
17524	CakSNP17524	*Kabuli*	Ca_Kabuli_denovo	359706	(G/C)
17525	CakSNP17525	*Kabuli*	Ca_Kabuli_denovo	359707	(C/A)
17526	CakSNP17526	*Kabuli*	Ca_Kabuli_denovo	359708	(A/G)
17527	CakSNP17527	*Kabuli*	Ca_Kabuli_denovo	359710	(G/A)
17528	CakSNP17528	*Kabuli*	Ca_Kabuli_denovo	359712	(A/T)
17529	CakSNP17529	*Kabuli*	Ca_Kabuli_denovo	359713	(T/G)
17530	CakSNP17530	*Kabuli*	Ca_Kabuli_denovo	359714	(G/C)
17531	CakSNP17531	*Kabuli*	Ca_Kabuli_denovo	359716	(C/G)
17532	CakSNP17532	*Kabuli*	Ca_Kabuli_denovo	359717	(G/A)
17533	CakSNP17533	*Kabuli*	Ca_Kabuli_denovo	359718	(A/G)
17534	CakSNP17534	*Kabuli*	Ca_Kabuli_denovo	359719	(G/A)
17535	CakSNP17535	*Kabuli*	Ca_Kabuli_denovo	359720	(A/C)
17536	CakSNP17536	*Kabuli*	Ca_Kabuli_denovo	359722	(C/G)
17537	CakSNP17537	*Kabuli*	Ca_Kabuli_denovo	359724	(G/T)
17538	CakSNP17538	*Kabuli*	Ca_Kabuli_denovo	359725	(A/C)
17539	CakSNP17539	*Kabuli*	Ca_Kabuli_denovo	364778	(T/G)
17540	CakSNP17540	*Kabuli*	Ca_Kabuli_denovo	364839	(A/C)
17541	CakSNP17541	*Kabuli*	Ca_Kabuli_denovo	366496	(A/G)
17542	CakSNP17542	*Kabuli*	Ca_Kabuli_denovo	366498	(C/A)
17543	CakSNP17543	*Kabuli*	Ca_Kabuli_denovo	369650	(A/G)
17544	CakSNP17544	*Kabuli*	Ca_Kabuli_denovo	375270	(G/A)
17545	CakSNP17545	*Kabuli*	Ca_Kabuli_denovo	375282	(T/G)
17546	CakSNP17546	*Kabuli*	Ca_Kabuli_denovo	381255	(T/G)
17547	CakSNP17547	*Kabuli*	Ca_Kabuli_denovo	381258	(T/G)
17548	CakSNP17548	*Kabuli*	Ca_Kabuli_denovo	381275	(T/G)
17549	CakSNP17549	*Kabuli*	Ca_Kabuli_denovo	382497	(G/A)
17550	CakSNP17550	*Kabuli*	Ca_Kabuli_denovo	386867	(G/C)
17551	CakSNP17551	*Kabuli*	Ca_Kabuli_denovo	386970	(G/C)
17552	CakSNP17552	*Kabuli*	Ca_Kabuli_denovo	389031	(T/G)
17553	CakSNP17553	*Kabuli*	Ca_Kabuli_denovo	389037	(A/G)
17554	CakSNP17554	*Kabuli*	Ca_Kabuli_denovo	389049	(C/G)
17555	CakSNP17555	*Kabuli*	Ca_Kabuli_denovo	389058	(T/G)

S.N.	SNP IDs	Cultivars	Chromosomes/scaffolds	Physical positions (bp)	SNPs
17556	CakSNP17556	*Kabuli*	Ca_Kabuli_denovo	389063	(T/G)
17557	CakSNP17557	*Kabuli*	Ca_Kabuli_denovo	389071	(C/G)
17558	CakSNP17558	*Kabuli*	Ca_Kabuli_denovo	389076	(A/G)
17559	CakSNP17559	*Kabuli*	Ca_Kabuli_denovo	389084	(C/G)
17560	CakSNP17560	*Kabuli*	Ca_Kabuli_denovo	392493	(T/G)
17561	CakSNP17561	*Kabuli*	Ca_Kabuli_denovo	396462	(A/T)
17562	CakSNP17562	*Kabuli*	Ca_Kabuli_denovo	396485	(T/G)
17563	CakSNP17563	*Kabuli*	Ca_Kabuli_denovo	396487	(T/G)
17564	CakSNP17564	*Kabuli*	Ca_Kabuli_denovo	396520	(T/G)
17565	CakSNP17565	*Kabuli*	Ca_Kabuli_denovo	396522	(T/G)
17566	CakSNP17566	*Kabuli*	Ca_Kabuli_denovo	396537	(T/A)
17567	CakSNP17567	*Kabuli*	Ca_Kabuli_denovo	401937	(C/G)
17568	CakSNP17568	*Kabuli*	Ca_Kabuli_denovo	401939	(C/G)
17569	CakSNP17569	*Kabuli*	Ca_Kabuli_denovo	401940	(G/A)
17570	CakSNP17570	*Kabuli*	Ca_Kabuli_denovo	401941	(A/G)
17571	CakSNP17571	*Kabuli*	Ca_Kabuli_denovo	405694	(G/C)
17572	CakSNP17572	*Kabuli*	Ca_Kabuli_denovo	408057	(A/G)
17573	CakSNP17573	*Kabuli*	Ca_Kabuli_denovo	408058	(T/G)
17574	CakSNP17574	*Kabuli*	Ca_Kabuli_denovo	408059	(T/C)
17575	CakSNP17575	*Kabuli*	Ca_Kabuli_denovo	408060	(C/G)
17576	CakSNP17576	*Kabuli*	Ca_Kabuli_denovo	409398	(A/G)
17577	CakSNP17577	*Kabuli*	Ca_Kabuli_denovo	409408	(C/G)
17578	CakSNP17578	*Kabuli*	Ca_Kabuli_denovo	409485	(A/G)
17579	CakSNP17579	*Kabuli*	Ca_Kabuli_denovo	409491	(A/G)
17580	CakSNP17580	*Kabuli*	Ca_Kabuli_denovo	409503	(A/G)
17581	CakSNP17581	*Kabuli*	Ca_Kabuli_denovo	409516	(C/A)
17582	CakSNP17582	*Kabuli*	Ca_Kabuli_denovo	409517	(C/G)
17583	CakSNP17583	*Kabuli*	Ca_Kabuli_denovo	409538	(T/G)
17584	CakSNP17584	*Kabuli*	Ca_Kabuli_denovo	412542	(A/G)
17585	CakSNP17585	*Kabuli*	Ca_Kabuli_denovo	412543	(G/C)
17586	CakSNP17586	*Kabuli*	Ca_Kabuli_denovo	412544	(A/C)
17587	CakSNP17587	*Kabuli*	Ca_Kabuli_denovo	412545	(G/A)
17588	CakSNP17588	*Kabuli*	Ca_Kabuli_denovo	416852	(C/G)
17589	CakSNP17589	*Kabuli*	Ca_Kabuli_denovo	416855	(T/G)
17590	CakSNP17590	*Kabuli*	Ca_Kabuli_denovo	416876	(T/G)
17591	CakSNP17591	*Kabuli*	Ca_Kabuli_denovo	416887	(A/G)
17592	CakSNP17592	*Kabuli*	Ca_Kabuli_denovo	416915	(A/G)
17593	CakSNP17593	*Kabuli*	Ca_Kabuli_denovo	416999	(A/T)

S.N.	SNP IDs	Cultivars	Chromosomes/scaffolds	Physical positions (bp)	SNPs
17594	CakSNP17594	*Kabuli*	Ca_Kabuli_denovo	417000	(A/C)
17595	CakSNP17595	*Kabuli*	Ca_Kabuli_denovo	420045	(A/G)
17596	CakSNP17596	*Kabuli*	Ca_Kabuli_denovo	420049	(A/G)
17597	CakSNP17597	*Kabuli*	Ca_Kabuli_denovo	420278	(A/G)
17598	CakSNP17598	*Kabuli*	Ca_Kabuli_denovo	420290	(C/G)
17599	CakSNP17599	*Kabuli*	Ca_Kabuli_denovo	420297	(C/G)
17600	CakSNP17600	*Kabuli*	Ca_Kabuli_denovo	420313	(A/G)
17601	CakSNP17601	*Kabuli*	Ca_Kabuli_denovo	420318	(C/G)
17602	CakSNP17602	*Kabuli*	Ca_Kabuli_denovo	420329	(A/G)
17603	CakSNP17603	*Kabuli*	Ca_Kabuli_denovo	420360	(A/G)
17604	CakSNP17604	*Kabuli*	Ca_Kabuli_denovo	422246	(C/G)
17605	CakSNP17605	*Kabuli*	Ca_Kabuli_denovo	422283	(G/A)
17606	CakSNP17606	*Kabuli*	Ca_Kabuli_denovo	422291	(T/G)
17607	CakSNP17607	*Kabuli*	Ca_Kabuli_denovo	422304	(C/A)
17608	CakSNP17608	*Kabuli*	Ca_Kabuli_denovo	425114	(A/G)
17609	CakSNP17609	*Kabuli*	Ca_Kabuli_denovo	425192	(A/G)
17610	CakSNP17610	*Kabuli*	Ca_Kabuli_denovo	427123	(T/G)
17611	CakSNP17611	*Kabuli*	Ca_Kabuli_denovo	440472	(A/G)
17612	CakSNP17612	*Kabuli*	Ca_Kabuli_denovo	441668	(T/G)
17613	CakSNP17613	*Kabuli*	Ca_Kabuli_denovo	442080	(T/G)
17614	CakSNP17614	*Kabuli*	Ca_Kabuli_denovo	442120	(T/C)
17615	CakSNP17615	*Kabuli*	Ca_Kabuli_denovo	442121	(C/A)
17616	CakSNP17616	*Kabuli*	Ca_Kabuli_denovo	442122	(T/A)
17617	CakSNP17617	*Kabuli*	Ca_Kabuli_denovo	442213	(C/T)
17618	CakSNP17618	*Kabuli*	Ca_Kabuli_denovo	442216	(C/T)
17619	CakSNP17619	*Kabuli*	Ca_Kabuli_denovo	442391	(G/C)
17620	CakSNP17620	*Kabuli*	Ca_Kabuli_denovo	442393	(C/G)
17621	CakSNP17621	*Kabuli*	Ca_Kabuli_denovo	442394	(G/A)
17622	CakSNP17622	*Kabuli*	Ca_Kabuli_denovo	442395	(G/A)
17623	CakSNP17623	*Kabuli*	Ca_Kabuli_denovo	442398	(C/T)
17624	CakSNP17624	*Kabuli*	Ca_Kabuli_denovo	442399	(G/C)
17625	CakSNP17625	*Kabuli*	Ca_Kabuli_denovo	442400	(A/G)
17626	CakSNP17626	*Kabuli*	Ca_Kabuli_denovo	442402	(C/A)
17627	CakSNP17627	*Kabuli*	Ca_Kabuli_denovo	443991	(T/G)
17628	CakSNP17628	*Kabuli*	Ca_Kabuli_denovo	444015	(T/C)
17629	CakSNP17629	*Kabuli*	Ca_Kabuli_denovo	444023	(T/G)
17630	CakSNP17630	*Kabuli*	Ca_Kabuli_denovo	444031	(A/G)
17631	CakSNP17631	*Kabuli*	Ca_Kabuli_denovo	453291	(C/G)

S.N.	SNP IDs	Cultivars	Chromosomes/scaffolds	Physical positions (bp)	SNPs
17632	CakSNP17632	*Kabuli*	Ca_Kabuli_denovo	453300	(A/G)
17633	CakSNP17633	*Kabuli*	Ca_Kabuli_denovo	453318	(A/G)
17634	CakSNP17634	*Kabuli*	Ca_Kabuli_denovo	453861	(G/A)
17635	CakSNP17635	*Kabuli*	Ca_Kabuli_denovo	453862	(G/A)
17636	CakSNP17636	*Kabuli*	Ca_Kabuli_denovo	453900	(G/A)
17637	CakSNP17637	*Kabuli*	Ca_Kabuli_denovo	454466	(C/G)
17638	CakSNP17638	*Kabuli*	Ca_Kabuli_denovo	468460	(G/A)
17639	CakSNP17639	*Kabuli*	Ca_Kabuli_denovo	469180	(A/G)
17640	CakSNP17640	*Kabuli*	Ca_Kabuli_denovo	476805	(G/C)
17641	CakSNP17641	*Kabuli*	Ca_Kabuli_denovo	476807	(C/G)
17642	CakSNP17642	*Kabuli*	Ca_Kabuli_denovo	476808	(A/G)
17643	CakSNP17643	*Kabuli*	Ca_Kabuli_denovo	476809	(G/A)
17644	CakSNP17644	*Kabuli*	Ca_Kabuli_denovo	476811	(C/A)
17645	CakSNP17645	*Kabuli*	Ca_Kabuli_denovo	479038	(C/A)
17646	CakSNP17646	*Kabuli*	Ca_Kabuli_denovo	479043	(A/G)
17647	CakSNP17647	*Kabuli*	Ca_Kabuli_denovo	489994	(T/G)
17648	CakSNP17648	*Kabuli*	Ca_Kabuli_denovo	490709	(G/C)
17649	CakSNP17649	*Kabuli*	Ca_Kabuli_denovo	490829	(C/A)
17650	CakSNP17650	*Kabuli*	Ca_Kabuli_denovo	490851	(A/C)
17651	CakSNP17651	*Kabuli*	Ca_Kabuli_denovo	490863	(T/G)
17652	CakSNP17652	*Kabuli*	Ca_Kabuli_denovo	490944	(C/A)
17653	CakSNP17653	*Kabuli*	Ca_Kabuli_denovo	490945	(A/T)
17654	CakSNP17654	*Kabuli*	Ca_Kabuli_denovo	490946	(G/T)
17655	CakSNP17655	*Kabuli*	Ca_Kabuli_denovo	490947	(A/C)
17656	CakSNP17656	*Kabuli*	Ca_Kabuli_denovo	491027	(G/T)
17657	CakSNP17657	*Kabuli*	Ca_Kabuli_denovo	492735	(A/T)
17658	CakSNP17658	*Kabuli*	Ca_Kabuli_denovo	500805	(A/C)
17659	CakSNP17659	*Kabuli*	Ca_Kabuli_denovo	504532	(T/G)
17660	CakSNP17660	*Kabuli*	Ca_Kabuli_denovo	504535	(C/G)
17661	CakSNP17661	*Kabuli*	Ca_Kabuli_denovo	504543	(T/G)
17662	CakSNP17662	*Kabuli*	Ca_Kabuli_denovo	504570	(A/G)
17663	CakSNP17663	*Kabuli*	Ca_Kabuli_denovo	504576	(T/G)
17664	CakSNP17664	*Kabuli*	Ca_Kabuli_denovo	504608	(G/A)
17665	CakSNP17665	*Kabuli*	Ca_Kabuli_denovo	504615	(C/T)
17666	CakSNP17666	*Kabuli*	Ca_Kabuli_denovo	514036	(G/C)
17667	CakSNP17667	*Kabuli*	Ca_Kabuli_denovo	514044	(T/C)
17668	CakSNP17668	*Kabuli*	Ca_Kabuli_denovo	520412	(A/G)
17669	CakSNP17669	*Kabuli*	Ca_Kabuli_denovo	520413	(G/A)

S.N.	SNP IDs	Cultivars	Chromosomes/scaffolds	Physical positions (bp)	SNPs
17670	CakSNP17670	*Kabuli*	Ca_Kabuli_denovo	520414	(G/T)
17671	CakSNP17671	*Kabuli*	Ca_Kabuli_denovo	520415	(A/C)
17672	CakSNP17672	*Kabuli*	Ca_Kabuli_denovo	520883	(G/A)
17673	CakSNP17673	*Kabuli*	Ca_Kabuli_denovo	523230	(A/G)
17674	CakSNP17674	*Kabuli*	Ca_Kabuli_denovo	523242	(T/A)
17675	CakSNP17675	*Kabuli*	Ca_Kabuli_denovo	525830	(C/A)
17676	CakSNP17676	*Kabuli*	Ca_Kabuli_denovo	525854	(T/A)
17677	CakSNP17677	*Kabuli*	Ca_Kabuli_denovo	525877	(G/C)
17678	CakSNP17678	*Kabuli*	Ca_Kabuli_denovo	533049	(A/T)
17679	CakSNP17679	*Kabuli*	Ca_Kabuli_denovo	533050	(G/T)
17680	CakSNP17680	*Kabuli*	Ca_Kabuli_denovo	533054	(C/T)
17681	CakSNP17681	*Kabuli*	Ca_Kabuli_denovo	534272	(A/G)
17682	CakSNP17682	*Kabuli*	Ca_Kabuli_denovo	554834	(G/C)
17683	CakSNP17683	*Kabuli*	Ca_Kabuli_denovo	554836	(A/G)
17684	CakSNP17684	*Kabuli*	Ca_Kabuli_denovo	554837	(G/A)
17685	CakSNP17685	*Kabuli*	Ca_Kabuli_denovo	554838	(G/A)
17686	CakSNP17686	*Kabuli*	Ca_Kabuli_denovo	554839	(A/G)
17687	CakSNP17687	*Kabuli*	Ca_Kabuli_denovo	565001	(C/G)
17688	CakSNP17688	*Kabuli*	Ca_Kabuli_denovo	565008	(T/G)
17689	CakSNP17689	*Kabuli*	Ca_Kabuli_denovo	565056	(A/G)
17690	CakSNP17690	*Kabuli*	Ca_Kabuli_denovo	565065	(C/G)
17691	CakSNP17691	*Kabuli*	Ca_Kabuli_denovo	566223	(A/G)
17692	CakSNP17692	*Kabuli*	Ca_Kabuli_denovo	566225	(T/G)
17693	CakSNP17693	*Kabuli*	Ca_Kabuli_denovo	566238	(C/A)
17694	CakSNP17694	*Kabuli*	Ca_Kabuli_denovo	566255	(T/C)
17695	CakSNP17695	*Kabuli*	Ca_Kabuli_denovo	575792	(T/G)
17696	CakSNP17696	*Kabuli*	Ca_Kabuli_denovo	575809	(A/G)
17697	CakSNP17697	*Kabuli*	Ca_Kabuli_denovo	736077	(A/C)
17698	CakSNP17698	*Kabuli*	Ca_Kabuli_denovo	861305	(T/C)
17699	CakSNP17699	*Kabuli*	Ca_Kabuli_denovo	861308	(G/C)
17700	CakSNP17700	*Kabuli*	Ca_Kabuli_denovo	930198	(T/A)
17701	CakSNP17701	*Kabuli*	Ca_Kabuli_denovo	930227	(T/G)
17702	CakSNP17702	*Kabuli*	Ca_Kabuli_denovo	930235	(A/G)
17703	CakSNP17703	*Kabuli*	Ca_Kabuli_denovo	930257	(T/G)
17704	CakSNP17704	*Kabuli*	Ca_Kabuli_denovo	930278	(G/C)
17705	CakSNP17705	*Kabuli*	Ca_Kabuli_denovo	930279	(G/A)
17706	CakSNP17706	*Kabuli*	Ca_Kabuli_denovo	933718	(C/A)
17707	CakSNP17707	*Kabuli*	Ca_Kabuli_denovo	933719	(T/G)

S.N.	SNP IDs	Cultivars	Chromosomes/scaffolds	Physical positions (bp)	SNPs
17708	CakSNP17708	*Kabuli*	Ca_Kabuli_denovo	933720	(C/A)
17709	CakSNP17709	*Kabuli*	Ca_Kabuli_denovo	965223	(A/G)
17710	CakSNP17710	*Kabuli*	Ca_Kabuli_denovo	1158419	(G/C)
17711	CakSNP17711	*Kabuli*	Ca_Kabuli_denovo	1158466	(C/G)
17712	CakSNP17712	*Kabuli*	Ca_Kabuli_denovo	1188058	(C/A)
17713	CakSNP17713	*Kabuli*	Ca_Kabuli_denovo	1188059	(A/C)
17714	CakSNP17714	*Kabuli*	Ca_Kabuli_denovo	1188060	(G/C)
17715	CakSNP17715	*Kabuli*	Ca_Kabuli_denovo	1188063	(T/A)
17716	CakSNP17716	*Kabuli*	Ca_Kabuli_denovo	1188064	(C/T)
17717	CakSNP17717	*Kabuli*	Ca_Kabuli_denovo	1286765	(C/A)
17718	CakSNP17718	*Kabuli*	Ca_Kabuli_denovo	1286799	(T/G)
17719	CakSNP17719	*Kabuli*	Ca_Kabuli_denovo	1286839	(C/A)
17720	CakSNP17720	*Kabuli*	Ca_Kabuli_denovo	1286848	(T/G)
17721	CakSNP17721	*Kabuli*	Ca_Kabuli_denovo	1286873	(T/A)
17722	CakSNP17722	*Kabuli*	Ca_Kabuli_denovo	1286885	(A/G)
17723	CakSNP17723	*Kabuli*	Ca_Kabuli_denovo	1286892	(T/G)
17724	CakSNP17724	*Kabuli*	Ca_Kabuli_denovo	1286899	(A/G)
17725	CakSNP17725	*Kabuli*	Ca_Kabuli_denovo	1286901	(C/A)
17726	CakSNP17726	*Kabuli*	Ca_Kabuli_denovo	1541967	(C/G)
17727	CakSNP17727	*Kabuli*	Ca_Kabuli_denovo	1542153	(T/G)
17728	CakSNP17728	*Kabuli*	Ca_Kabuli_denovo	1602962	(T/A)
17729	CakSNP17729	*Kabuli*	Ca_Kabuli_denovo	1603019	(T/C)
17730	CakSNP17730	*Kabuli*	Ca_Kabuli_denovo	1603020	(T/A)
17731	CakSNP17731	*Kabuli*	Ca_Kabuli_denovo	1603038	(A/C)
17732	CakSNP17732	*Kabuli*	Ca_Kabuli_denovo	1603067	(C/G)
17733	CakSNP17733	*Kabuli*	Ca_Kabuli_denovo	1603073	(G/T)
17734	CakSNP17734	*Kabuli*	Ca_Kabuli_denovo	1603120	(G/C)
17735	CakSNP17735	*Kabuli*	Ca_Kabuli_denovo	1604436	(A/G)
17736	CakSNP17736	*Kabuli*	Ca_Kabuli_denovo	1604633	(G/A)
17737	CakSNP17737	*Kabuli*	Ca_Kabuli_denovo	1604645	(G/A)
17738	CakSNP17738	*Kabuli*	Ca_Kabuli_denovo	1604682	(T/G)
17739	CakSNP17739	*Kabuli*	Ca_Kabuli_denovo	1607526	(A/G)
17740	CakSNP17740	*Kabuli*	Ca_Kabuli_denovo	1621430	(C/G)
17741	CakSNP17741	*Kabuli*	Ca_Kabuli_denovo	1621431	(C/G)
17742	CakSNP17742	*Kabuli*	Ca_Kabuli_denovo	1622000	(T/A)
17743	CakSNP17743	*Kabuli*	Ca_Kabuli_denovo	1622001	(T/G)
17744	CakSNP17744	*Kabuli*	Ca_Kabuli_denovo	1622005	(G/C)
17745	CakSNP17745	*Kabuli*	Ca_Kabuli_denovo	1627804	(A/G)

S.N.	SNP IDs	Cultivars	Chromosomes/scaffolds	Physical positions (bp)	SNPs
17746	CakSNP17746	*Kabuli*	Ca_Kabuli_denovo	1627832	(C/G)
17747	CakSNP17747	*Kabuli*	Ca_Kabuli_denovo	1627845	(C/G)
17748	CakSNP17748	*Kabuli*	Ca_Kabuli_denovo	1629193	(C/G)
17749	CakSNP17749	*Kabuli*	Ca_Kabuli_denovo	1629194	(G/T)
17750	CakSNP17750	*Kabuli*	Ca_Kabuli_denovo	1629209	(C/G)
17751	CakSNP17751	*Kabuli*	Ca_Kabuli_denovo	1629225	(T/G)
17752	CakSNP17752	*Kabuli*	Ca_Kabuli_denovo	1645344	(A/C)
17753	CakSNP17753	*Kabuli*	Ca_Kabuli_denovo	1646822	(T/A)
17754	CakSNP17754	*Kabuli*	Ca_Kabuli_denovo	1646824	(C/G)
17755	CakSNP17755	*Kabuli*	Ca_Kabuli_denovo	1646825	(C/A)
17756	CakSNP17756	*Kabuli*	Ca_Kabuli_denovo	1646826	(C/G)
17757	CakSNP17757	*Kabuli*	Ca_Kabuli_denovo	1651702	(C/G)
17758	CakSNP17758	*Kabuli*	Ca_Kabuli_denovo	1651703	(G/A)
17759	CakSNP17759	*Kabuli*	Ca_Kabuli_denovo	1651738	(A/G)
17760	CakSNP17760	*Kabuli*	Ca_Kabuli_denovo	1657001	(C/G)
17761	CakSNP17761	*Kabuli*	Ca_Kabuli_denovo	1657040	(T/G)
17762	CakSNP17762	*Kabuli*	Ca_Kabuli_denovo	1662755	(C/G)
17763	CakSNP17763	*Kabuli*	Ca_Kabuli_denovo	1662758	(A/G)
17764	CakSNP17764	*Kabuli*	Ca_Kabuli_denovo	1662759	(T/G)
17765	CakSNP17765	*Kabuli*	Ca_Kabuli_denovo	1662766	(G/A)
17766	CakSNP17766	*Kabuli*	Ca_Kabuli_denovo	1662768	(A/G)
17767	CakSNP17767	*Kabuli*	Ca_Kabuli_denovo	1662796	(G/A)
17768	CakSNP17768	*Kabuli*	Ca_Kabuli_denovo	1664534	(T/G)
17769	CakSNP17769	*Kabuli*	Ca_Kabuli_denovo	1664562	(T/G)
17770	CakSNP17770	*Kabuli*	Ca_Kabuli_denovo	1664573	(A/G)
17771	CakSNP17771	*Kabuli*	Ca_Kabuli_denovo	1665734	(A/G)
17772	CakSNP17772	*Kabuli*	Ca_Kabuli_denovo	1665742	(G/A)
17773	CakSNP17773	*Kabuli*	Ca_Kabuli_denovo	1665774	(C/G)
17774	CakSNP17774	*Kabuli*	Ca_Kabuli_denovo	1665815	(T/G)
17775	CakSNP17775	*Kabuli*	Ca_Kabuli_denovo	1670929	(C/T)
17776	CakSNP17776	*Kabuli*	Ca_Kabuli_denovo	1670930	(C/G)
17777	CakSNP17777	*Kabuli*	Ca_Kabuli_denovo	1670931	(G/C)
17778	CakSNP17778	*Kabuli*	Ca_Kabuli_denovo	1670932	(A/C)
17779	CakSNP17779	*Kabuli*	Ca_Kabuli_denovo	1674472	(T/G)
17780	CakSNP17780	*Kabuli*	Ca_Kabuli_denovo	1674473	(T/G)
17781	CakSNP17781	*Kabuli*	Ca_Kabuli_denovo	1674498	(G/A)
17782	CakSNP17782	*Kabuli*	Ca_Kabuli_denovo	1674513	(A/G)
17783	CakSNP17783	*Kabuli*	Ca_Kabuli_denovo	1674786	(T/G)

S.N.	SNP IDs	Cultivars	Chromosomes/scaffolds	Physical positions (bp)	SNPs
17784	CakSNP17784	*Kabuli*	Ca_Kabuli_denovo	1674794	(G/T)
17785	CakSNP17785	*Kabuli*	Ca_Kabuli_denovo	1674824	(T/G)
17786	CakSNP17786	*Kabuli*	Ca_Kabuli_denovo	1676619	(A/C)
17787	CakSNP17787	*Kabuli*	Ca_Kabuli_denovo	1676622	(C/A)
17788	CakSNP17788	*Kabuli*	Ca_Kabuli_denovo	1676628	(C/G)
17789	CakSNP17789	*Kabuli*	Ca_Kabuli_denovo	1676641	(T/A)
17790	CakSNP17790	*Kabuli*	Ca_Kabuli_denovo	1676642	(T/G)
17791	CakSNP17791	*Kabuli*	Ca_Kabuli_denovo	1676643	(T/G)
17792	CakSNP17792	*Kabuli*	Ca_Kabuli_denovo	1676647	(A/G)
17793	CakSNP17793	*Kabuli*	Ca_Kabuli_denovo	1676665	(T/G)
17794	CakSNP17794	*Kabuli*	Ca_Kabuli_denovo	1676692	(T/G)
17795	CakSNP17795	*Kabuli*	Ca_Kabuli_denovo	1679945	(C/T)
17796	CakSNP17796	*Kabuli*	Ca_Kabuli_denovo	1684919	(A/T)
17797	CakSNP17797	*Kabuli*	Ca_Kabuli_denovo	1684920	(G/T)
17798	CakSNP17798	*Kabuli*	Ca_Kabuli_denovo	1684923	(T/G)
17799	CakSNP17799	*Kabuli*	Ca_Kabuli_denovo	1685743	(A/C)
17800	CakSNP17800	*Kabuli*	Ca_Kabuli_denovo	1685744	(C/T)
17801	CakSNP17801	*Kabuli*	Ca_Kabuli_denovo	1685745	(T/C)
17802	CakSNP17802	*Kabuli*	Ca_Kabuli_denovo	1687665	(A/G)
17803	CakSNP17803	*Kabuli*	Ca_Kabuli_denovo	1687667	(A/G)
17804	CakSNP17804	*Kabuli*	Ca_Kabuli_denovo	1687669	(A/T)
17805	CakSNP17805	*Kabuli*	Ca_Kabuli_denovo	1688595	(A/T)
17806	CakSNP17806	*Kabuli*	Ca_Kabuli_denovo	1688596	(T/C)
17807	CakSNP17807	*Kabuli*	Ca_Kabuli_denovo	1688598	(C/A)
17808	CakSNP17808	*Kabuli*	Ca_Kabuli_denovo	1688599	(A/T)
17809	CakSNP17809	*Kabuli*	Ca_Kabuli_denovo	1688600	(C/A)
17810	CakSNP17810	*Kabuli*	Ca_Kabuli_denovo	1690593	(G/A)
17811	CakSNP17811	*Kabuli*	Ca_Kabuli_denovo	1690595	(T/G)
17812	CakSNP17812	*Kabuli*	Ca_Kabuli_denovo	1690605	(T/A)
17813	CakSNP17813	*Kabuli*	Ca_Kabuli_denovo	1696698	(T/G)
17814	CakSNP17814	*Kabuli*	Ca_Kabuli_denovo	1696705	(C/G)
17815	CakSNP17815	*Kabuli*	Ca_Kabuli_denovo	1698932	(G/C)
17816	CakSNP17816	*Kabuli*	Ca_Kabuli_denovo	1698934	(A/G)
17817	CakSNP17817	*Kabuli*	Ca_Kabuli_denovo	1698960	(A/G)
17818	CakSNP17818	*Kabuli*	Ca_Kabuli_denovo	1699496	(C/G)
17819	CakSNP17819	*Kabuli*	Ca_Kabuli_denovo	1699498	(C/G)
17820	CakSNP17820	*Kabuli*	Ca_Kabuli_denovo	1699521	(T/G)
17821	CakSNP17821	*Kabuli*	Ca_Kabuli_denovo	1699556	(G/T)

S.N.	SNP IDs	Cultivars	Chromosomes/scaffolds	Physical positions (bp)	SNPs
17822	CakSNP17822	*Kabuli*	Ca_Kabuli_denovo	1699569	(A/T)
17823	CakSNP17823	*Kabuli*	Ca_Kabuli_denovo	1707083	(C/A)
17824	CakSNP17824	*Kabuli*	Ca_Kabuli_denovo	1707085	(C/G)
17825	CakSNP17825	*Kabuli*	Ca_Kabuli_denovo	1707086	(A/T)
17826	CakSNP17826	*Kabuli*	Ca_Kabuli_denovo	1707560	(C/A)
17827	CakSNP17827	*Kabuli*	Ca_Kabuli_denovo	1707561	(A/G)
17828	CakSNP17828	*Kabuli*	Ca_Kabuli_denovo	1707563	(A/G)
17829	CakSNP17829	*Kabuli*	Ca_Kabuli_denovo	1707565	(T/A)
17830	CakSNP17830	*Kabuli*	Ca_Kabuli_denovo	1732322	(A/G)
17831	CakSNP17831	*Kabuli*	Ca_Kabuli_denovo	1732338	(C/T)
17832	CakSNP17832	*Kabuli*	Ca_Kabuli_denovo	1734760	(G/C)
17833	CakSNP17833	*Kabuli*	Ca_Kabuli_denovo	1735490	(C/G)
17834	CakSNP17834	*Kabuli*	Ca_Kabuli_denovo	1735502	(C/T)
17835	CakSNP17835	*Kabuli*	Ca_Kabuli_denovo	1735523	(T/C)
17836	CakSNP17836	*Kabuli*	Ca_Kabuli_denovo	1735891	(T/G)
17837	CakSNP17837	*Kabuli*	Ca_Kabuli_denovo	1735929	(T/A)
17838	CakSNP17838	*Kabuli*	Ca_Kabuli_denovo	1735956	(G/C)
17839	CakSNP17839	*Kabuli*	Ca_Kabuli_denovo	1735988	(A/G)
17840	CakSNP17840	*Kabuli*	Ca_Kabuli_denovo	1737810	(A/G)
17841	CakSNP17841	*Kabuli*	Ca_Kabuli_denovo	1737898	(T/G)
17842	CakSNP17842	*Kabuli*	Ca_Kabuli_denovo	1737939	(G/A)
17843	CakSNP17843	*Kabuli*	Ca_Kabuli_denovo	1737964	(C/A)
17844	CakSNP17844	*Kabuli*	Ca_Kabuli_denovo	1739167	(G/C)
17845	CakSNP17845	*Kabuli*	Ca_Kabuli_denovo	1739168	(A/C)
17846	CakSNP17846	*Kabuli*	Ca_Kabuli_denovo	1739955	(G/A)
17847	CakSNP17847	*Kabuli*	Ca_Kabuli_denovo	1740013	(T/G)
17848	CakSNP17848	*Kabuli*	Ca_Kabuli_denovo	1741581	(G/A)
17849	CakSNP17849	*Kabuli*	Ca_Kabuli_denovo	1745745	(C/T)
17850	CakSNP17850	*Kabuli*	Ca_Kabuli_denovo	1745967	(G/T)
17851	CakSNP17851	*Kabuli*	Ca_Kabuli_denovo	1773627	(C/T)
17852	CakSNP17852	*Kabuli*	Ca_Kabuli_denovo	1773675	(T/G)
17853	CakSNP17853	*Kabuli*	Ca_Kabuli_denovo	1778702	(T/A)
17854	CakSNP17854	*Kabuli*	Ca_Kabuli_denovo	1778708	(G/A)
17855	CakSNP17855	*Kabuli*	Ca_Kabuli_denovo	1782502	(C/T)
17856	CakSNP17856	*Kabuli*	Ca_Kabuli_denovo	1782521	(T/C)
17857	CakSNP17857	*Kabuli*	Ca_Kabuli_denovo	1785710	(T/C)
17858	CakSNP17858	*Kabuli*	Ca_Kabuli_denovo	1799281	(G/T)
17859	CakSNP17859	*Kabuli*	Ca_Kabuli_denovo	1835504	(G/A)

S.N.	SNP IDs	Cultivars	Chromosomes/scaffolds	Physical positions (bp)	SNPs
17860	CakSNP17860	*Kabuli*	Ca_Kabuli_denovo	1848623	(T/C)
17861	CakSNP17861	*Kabuli*	Ca_Kabuli_denovo	1865044	(C/G)
17862	CakSNP17862	*Kabuli*	Ca_Kabuli_denovo	1865051	(T/C)
17863	CakSNP17863	*Kabuli*	Ca_Kabuli_denovo	1877655	(A/C)
17864	CakSNP17864	*Kabuli*	Ca_Kabuli_denovo	1885551	(C/T)
17865	CakSNP17865	*Kabuli*	Ca_Kabuli_denovo	1885575	(G/A)
17866	CakSNP17866	*Kabuli*	Ca_Kabuli_denovo	1892464	(C/T)
17867	CakSNP17867	*Kabuli*	Ca_Kabuli_denovo	1892470	(A/T)
17868	CakSNP17868	*Kabuli*	Ca_Kabuli_denovo	1898838	(A/G)
17869	CakSNP17869	*Kabuli*	Ca_Kabuli_denovo	1898842	(G/A)
17870	CakSNP17870	*Kabuli*	Ca_Kabuli_denovo	1907169	(C/T)
17871	CakSNP17871	*Kabuli*	Ca_Kabuli_denovo	1954938	(C/T)
17872	CakSNP17872	*Kabuli*	Ca_Kabuli_denovo	1973101	(A/C)
17873	CakSNP17873	*Kabuli*	Ca_Kabuli_denovo	1981659	(C/G)
17874	CakSNP17874	*Kabuli*	Ca_Kabuli_denovo	1981685	(C/T)
17875	CakSNP17875	*Kabuli*	Ca_Kabuli_denovo	2017269	(C/T)
17876	CakSNP17876	*Kabuli*	Ca_Kabuli_denovo	2017293	(G/A)
17877	CakSNP17877	*Kabuli*	Ca_Kabuli_denovo	2082507	(C/T)
17878	CakSNP17878	*Kabuli*	Ca_Kabuli_denovo	2082510	(T/C)
17879	CakSNP17879	*Kabuli*	Ca_Kabuli_denovo	2083031	(G/C)
17880	CakSNP17880	*Kabuli*	Ca_Kabuli_denovo	2083034	(G/A)
17881	CakSNP17881	*Kabuli*	Ca_Kabuli_denovo	2083043	(T/G)
17882	CakSNP17882	*Kabuli*	Ca_Kabuli_denovo	2086223	(A/C)
17883	CakSNP17883	*Kabuli*	Ca_Kabuli_denovo	2089458	(A/G)
17884	CakSNP17884	*Kabuli*	Ca_Kabuli_denovo	2089483	(G/C)
17885	CakSNP17885	*Kabuli*	Ca_Kabuli_denovo	2103496	(C/G)
17886	CakSNP17886	*Kabuli*	Ca_Kabuli_denovo	2117543	(A/G)
17887	CakSNP17887	*Kabuli*	Ca_Kabuli_denovo	2179176	(A/C)
17888	CakSNP17888	*Kabuli*	Ca_Kabuli_denovo	2196538	(T/A)
17889	CakSNP17889	*Kabuli*	Ca_Kabuli_denovo	2196959	(G/A)
17890	CakSNP17890	*Kabuli*	Ca_Kabuli_denovo	2196969	(G/A)
17891	CakSNP17891	*Kabuli*	Ca_Kabuli_denovo	2204023	(C/T)
17892	CakSNP17892	*Kabuli*	Ca_Kabuli_denovo	2212621	(C/T)
17893	CakSNP17893	*Kabuli*	Ca_Kabuli_denovo	2212895	(C/T)
17894	CakSNP17894	*Kabuli*	Ca_Kabuli_denovo	2238931	(C/T)
17895	CakSNP17895	*Kabuli*	Ca_Kabuli_denovo	2238976	(C/T)
17896	CakSNP17896	*Kabuli*	Ca_Kabuli_denovo	2272968	(G/A)
17897	CakSNP17897	*Kabuli*	Ca_Kabuli_denovo	2289863	(C/A)

S.N.	SNP IDs	Cultivars	Chromosomes/scaffolds	Physical positions (bp)	SNPs
17898	CakSNP17898	*Kabuli*	Ca_Kabuli_denovo	2329264	(G/A)
17899	CakSNP17899	*Kabuli*	Ca_Kabuli_denovo	2339668	(G/A)
17900	CakSNP17900	*Kabuli*	Ca_Kabuli_denovo	2345594	(A/T)
17901	CakSNP17901	*Kabuli*	Ca_Kabuli_denovo	2349919	(G/T)
17902	CakSNP17902	*Kabuli*	Ca_Kabuli_denovo	2349923	(T/G)
17903	CakSNP17903	*Kabuli*	Ca_Kabuli_denovo	2352980	(T/G)
17904	CakSNP17904	*Kabuli*	Ca_Kabuli_denovo	2368635	(A/T)
17905	CakSNP17905	*Kabuli*	Ca_Kabuli_denovo	2377565	(G/T)
17906	CakSNP17906	*Kabuli*	Ca_Kabuli_denovo	2378145	(G/A)
17907	CakSNP17907	*Kabuli*	Ca_Kabuli_denovo	2422055	(G/A)
17908	CakSNP17908	*Kabuli*	Ca_Kabuli_denovo	2424690	(C/T)
17909	CakSNP17909	*Kabuli*	Ca_Kabuli_denovo	2436733	(T/G)
17910	CakSNP17910	*Kabuli*	Ca_Kabuli_denovo	2442765	(G/C)
17911	CakSNP17911	*Kabuli*	Ca_Kabuli_denovo	2472151	(C/G)
17912	CakSNP17912	*Kabuli*	Ca_Kabuli_denovo	2472157	(T/C)
17913	CakSNP17913	*Kabuli*	Ca_Kabuli_denovo	2472160	(A/C)
17914	CakSNP17914	*Kabuli*	Ca_Kabuli_denovo	2481362	(C/G)
17915	CakSNP17915	*Kabuli*	Ca_Kabuli_denovo	2481371	(G/C)
17916	CakSNP17916	*Kabuli*	Ca_Kabuli_denovo	2484130	(G/C)
17917	CakSNP17917	*Kabuli*	Ca_Kabuli_denovo	2493981	(A/C)
17918	CakSNP17918	*Kabuli*	Ca_Kabuli_denovo	2521639	(T/C)
17919	CakSNP17919	*Kabuli*	Ca_Kabuli_denovo	2532212	(C/T)
17920	CakSNP17920	*Kabuli*	Ca_Kabuli_denovo	2534679	(T/C)
17921	CakSNP17921	*Kabuli*	Ca_Kabuli_denovo	2543790	(C/G)
17922	CakSNP17922	*Kabuli*	Ca_Kabuli_denovo	2543805	(G/A)
17923	CakSNP17923	*Kabuli*	Ca_Kabuli_denovo	2557532	(T/G)
17924	CakSNP17924	*Kabuli*	Ca_Kabuli_denovo	2566186	(C/T)
17925	CakSNP17925	*Kabuli*	Ca_Kabuli_denovo	2586184	(T/C)
17926	CakSNP17926	*Kabuli*	Ca_Kabuli_denovo	2596306	(G/A)
17927	CakSNP17927	*Kabuli*	Ca_Kabuli_denovo	2599278	(C/T)
17928	CakSNP17928	*Kabuli*	Ca_Kabuli_denovo	2599284	(T/C)
17929	CakSNP17929	*Kabuli*	Ca_Kabuli_denovo	2810251	(C/G)
17930	CakSNP17930	*Kabuli*	Ca_Kabuli_denovo	2822788	(C/T)
17931	CakSNP17931	*Kabuli*	Ca_Kabuli_denovo	2822794	(G/A)
17932	CakSNP17932	*Kabuli*	Ca_Kabuli_denovo	2841190	(C/G)
17933	CakSNP17933	*Kabuli*	Ca_Kabuli_denovo	2899276	(A/G)
17934	CakSNP17934	*Kabuli*	Ca_Kabuli_denovo	2905320	(A/C)
17935	CakSNP17935	*Kabuli*	Ca_Kabuli_denovo	2920107	(A/C)

S.N.	SNP IDs	Cultivars	Chromosomes/scaffolds	Physical positions (bp)	SNPs
17936	CakSNP17936	*Kabuli*	Ca_Kabuli_denovo	2943170	(A/C)
17937	CakSNP17937	*Kabuli*	Ca_Kabuli_denovo	2950044	(T/C)
17938	CakSNP17938	*Kabuli*	Ca_Kabuli_denovo	2967013	(A/G)
17939	CakSNP17939	*Kabuli*	Ca_Kabuli_denovo	2986733	(G/C)
17940	CakSNP17940	*Kabuli*	Ca_Kabuli_denovo	2988473	(G/A)
17941	CakSNP17941	*Kabuli*	Ca_Kabuli_denovo	3002912	(G/C)
17942	CakSNP17942	*Kabuli*	Ca_Kabuli_denovo	3004600	(C/G)
17943	CakSNP17943	*Kabuli*	Ca_Kabuli_denovo	3028757	(A/G)
17944	CakSNP17944	*Kabuli*	Ca_Kabuli_denovo	3058052	(G/A)
17945	CakSNP17945	*Kabuli*	Ca_Kabuli_denovo	3067960	(A/G)
17946	CakSNP17946	*Kabuli*	Ca_Kabuli_denovo	3075746	(C/G)
17947	CakSNP17947	*Kabuli*	Ca_Kabuli_denovo	3075749	(A/G)
17948	CakSNP17948	*Kabuli*	Ca_Kabuli_denovo	3082312	(T/A)
17949	CakSNP17949	*Kabuli*	Ca_Kabuli_denovo	3102755	(C/T)
17950	CakSNP17950	*Kabuli*	Ca_Kabuli_denovo	3112730	(C/T)
17951	CakSNP17951	*Kabuli*	Ca_Kabuli_denovo	3119860	(G/A)
17952	CakSNP17952	*Kabuli*	Ca_Kabuli_denovo	3119864	(A/G)
17953	CakSNP17953	*Kabuli*	Ca_Kabuli_denovo	3122405	(A/G)
17954	CakSNP17954	*Kabuli*	Ca_Kabuli_denovo	3158126	(G/T)
17955	CakSNP17955	*Kabuli*	Ca_Kabuli_denovo	3158176	(G/A)
17956	CakSNP17956	*Kabuli*	Ca_Kabuli_denovo	3195629	(G/A)
17957	CakSNP17957	*Kabuli*	Ca_Kabuli_denovo	3234354	(G/A)
17958	CakSNP17958	*Kabuli*	Ca_Kabuli_denovo	3236873	(A/G)
17959	CakSNP17959	*Kabuli*	Ca_Kabuli_denovo	3236875	(T/C)
17960	CakSNP17960	*Kabuli*	Ca_Kabuli_denovo	3236878	(T/G)
17961	CakSNP17961	*Kabuli*	Ca_Kabuli_denovo	3303558	(A/C)
17962	CakSNP17962	*Kabuli*	Ca_Kabuli_denovo	3303631	(G/C)
17963	CakSNP17963	*Kabuli*	Ca_Kabuli_denovo	3315370	(A/G)
17964	CakSNP17964	*Kabuli*	Ca_Kabuli_denovo	3315379	(A/G)
17965	CakSNP17965	*Kabuli*	Ca_Kabuli_denovo	3366344	(C/T)
17966	CakSNP17966	*Kabuli*	Ca_Kabuli_denovo	3366389	(G/T)
17967	CakSNP17967	*Kabuli*	Ca_Kabuli_denovo	3376003	(A/G)
17968	CakSNP17968	*Kabuli*	Ca_Kabuli_denovo	3376309	(T/C)
17969	CakSNP17969	*Kabuli*	Ca_Kabuli_denovo	3381616	(T/C)
17970	CakSNP17970	*Kabuli*	Ca_Kabuli_denovo	3383148	(T/C)
17971	CakSNP17971	*Kabuli*	Ca_Kabuli_denovo	3397038	(T/C)
17972	CakSNP17972	*Kabuli*	Ca_Kabuli_denovo	3401848	(A/C)
17973	CakSNP17973	*Kabuli*	Ca_Kabuli_denovo	3419633	(C/G)

S.N.	SNP IDs	Cultivars	Chromosomes/scaffolds	Physical positions (bp)	SNPs
17974	CakSNP17974	*Kabuli*	Ca_Kabuli_denovo	3427074	(A/G)
17975	CakSNP17975	*Kabuli*	Ca_Kabuli_denovo	3438889	(C/T)
17976	CakSNP17976	*Kabuli*	Ca_Kabuli_denovo	3520157	(T/C)
17977	CakSNP17977	*Kabuli*	Ca_Kabuli_denovo	3520187	(A/G)
17978	CakSNP17978	*Kabuli*	Ca_Kabuli_denovo	3563125	(C/T)
17979	CakSNP17979	*Kabuli*	Ca_Kabuli_denovo	3569168	(A/G)
17980	CakSNP17980	*Kabuli*	Ca_Kabuli_denovo	3569183	(A/G)
17981	CakSNP17981	*Kabuli*	Ca_Kabuli_denovo	3593979	(G/T)
17982	CakSNP17982	*Kabuli*	Ca_Kabuli_denovo	3608269	(C/T)
17983	CakSNP17983	*Kabuli*	Ca_Kabuli_denovo	3608277	(G/T)
17984	CakSNP17984	*Kabuli*	Ca_Kabuli_denovo	3619659	(C/T)
17985	CakSNP17985	*Kabuli*	Ca_Kabuli_denovo	3619740	(A/G)
17986	CakSNP17986	*Kabuli*	Ca_Kabuli_denovo	3621163	(G/A)
17987	CakSNP17987	*Kabuli*	Ca_Kabuli_denovo	3621166	(T/G)
17988	CakSNP17988	*Kabuli*	Ca_Kabuli_denovo	3627246	(T/A)
17989	CakSNP17989	*Kabuli*	Ca_Kabuli_denovo	3640534	(G/A)
17990	CakSNP17990	*Kabuli*	Ca_Kabuli_denovo	3640537	(A/G)
17991	CakSNP17991	*Kabuli*	Ca_Kabuli_denovo	3643962	(C/T)
17992	CakSNP17992	*Kabuli*	Ca_Kabuli_denovo	3649832	(T/G)
17993	CakSNP17993	*Kabuli*	Ca_Kabuli_denovo	3649844	(A/G)
17994	CakSNP17994	*Kabuli*	Ca_Kabuli_denovo	3656017	(A/C)
17995	CakSNP17995	*Kabuli*	Ca_Kabuli_denovo	3733774	(C/T)
17996	CakSNP17996	*Kabuli*	Ca_Kabuli_denovo	3733795	(C/G)
17997	CakSNP17997	*Kabuli*	Ca_Kabuli_denovo	3733959	(G/A)
17998	CakSNP17998	*Kabuli*	Ca_Kabuli_denovo	3865215	(A/G)
17999	CakSNP17999	*Kabuli*	Ca_Kabuli_denovo	3882576	(G/A)
18000	CakSNP18000	*Kabuli*	Ca_Kabuli_denovo	3882602	(G/C)
18001	CakSNP18001	*Kabuli*	Ca_Kabuli_denovo	3891592	(A/G)
18002	CakSNP18002	*Kabuli*	Ca_Kabuli_denovo	3926567	(T/C)
18003	CakSNP18003	*Kabuli*	Ca_Kabuli_denovo	3932481	(T/A)
18004	CakSNP18004	*Kabuli*	Ca_Kabuli_denovo	3962628	(C/A)
18005	CakSNP18005	*Kabuli*	Ca_Kabuli_denovo	3971486	(C/G)
18006	CakSNP18006	*Kabuli*	Ca_Kabuli_denovo	3971511	(T/C)
18007	CakSNP18007	*Kabuli*	Ca_Kabuli_denovo	3985191	(G/A)
18008	CakSNP18008	*Kabuli*	Ca_Kabuli_denovo	3985194	(C/T)
18009	CakSNP18009	*Kabuli*	Ca_Kabuli_denovo	3988996	(G/C)
18010	CakSNP18010	*Kabuli*	Ca_Kabuli_denovo	3989017	(G/A)
18011	CakSNP18011	*Kabuli*	Ca_Kabuli_denovo	4058620	(G/C)

S.N.	SNP IDs	Cultivars	Chromosomes/scaffolds	Physical positions (bp)	SNPs
18012	CakSNP18012	*Kabuli*	Ca_Kabuli_denovo	4058680	(A/C)
18013	CakSNP18013	*Kabuli*	Ca_Kabuli_denovo	4069641	(G/C)
18014	CakSNP18014	*Kabuli*	Ca_Kabuli_denovo	4069659	(C/T)
18015	CakSNP18015	*Kabuli*	Ca_Kabuli_denovo	4074763	(A/G)
18016	CakSNP18016	*Kabuli*	Ca_Kabuli_denovo	4074764	(C/G)
18017	CakSNP18017	*Kabuli*	Ca_Kabuli_denovo	4102951	(A/G)
18018	CakSNP18018	*Kabuli*	Ca_Kabuli_denovo	4131618	(A/C)
18019	CakSNP18019	*Kabuli*	Ca_Kabuli_denovo	4327961	(T/C)
18020	CakSNP18020	*Kabuli*	Ca_Kabuli_denovo	4354794	(C/T)
18021	CakSNP18021	*Kabuli*	Ca_Kabuli_denovo	4354804	(C/T)
18022	CakSNP18022	*Kabuli*	Ca_Kabuli_denovo	4384538	(T/C)
18023	CakSNP18023	*Kabuli*	Ca_Kabuli_denovo	4423469	(A/G)
18024	CakSNP18024	*Kabuli*	Ca_Kabuli_denovo	4423478	(C/G)
18025	CakSNP18025	*Kabuli*	Ca_Kabuli_denovo	4462086	(C/A)
18026	CakSNP18026	*Kabuli*	Ca_Kabuli_denovo	4531890	(A/C)
18027	CakSNP18027	*Kabuli*	Ca_Kabuli_denovo	4533618	(T/C)
18028	CakSNP18028	*Kabuli*	Ca_Kabuli_denovo	4565146	(G/A)
18029	CakSNP18029	*Kabuli*	Ca_Kabuli_denovo	4575382	(C/T)
18030	CakSNP18030	*Kabuli*	Ca_Kabuli_denovo	4581997	(C/G)
18031	CakSNP18031	*Kabuli*	Ca_Kabuli_denovo	4581998	(C/A)
18032	CakSNP18032	*Kabuli*	Ca_Kabuli_denovo	4633334	(G/A)
18033	CakSNP18033	*Kabuli*	Ca_Kabuli_denovo	4634189	(A/C)
18034	CakSNP18034	*Kabuli*	Ca_Kabuli_denovo	4645619	(G/A)
18035	CakSNP18035	*Kabuli*	Ca_Kabuli_denovo	4659307	(G/C)
18036	CakSNP18036	*Kabuli*	Ca_Kabuli_denovo	4659309	(C/A)
18037	CakSNP18037	*Kabuli*	Ca_Kabuli_denovo	4659328	(G/A)
18038	CakSNP18038	*Kabuli*	Ca_Kabuli_denovo	4704245	(G/A)
18039	CakSNP18039	*Kabuli*	Ca_Kabuli_denovo	4704305	(C/T)
18040	CakSNP18040	*Kabuli*	Ca_Kabuli_denovo	4738377	(T/A)
18041	CakSNP18041	*Kabuli*	Ca_Kabuli_denovo	4738379	(G/T)
18042	CakSNP18042	*Kabuli*	Ca_Kabuli_denovo	4738831	(G/A)
18043	CakSNP18043	*Kabuli*	Ca_Kabuli_denovo	4756479	(G/A)
18044	CakSNP18044	*Kabuli*	Ca_Kabuli_denovo	4763483	(C/A)
18045	CakSNP18045	*Kabuli*	Ca_Kabuli_denovo	4769505	(C/A)
18046	CakSNP18046	*Kabuli*	Ca_Kabuli_denovo	4769506	(A/G)
18047	CakSNP18047	*Kabuli*	Ca_Kabuli_denovo	4779386	(G/T)
18048	CakSNP18048	*Kabuli*	Ca_Kabuli_denovo	4779389	(G/A)
18049	CakSNP18049	*Kabuli*	Ca_Kabuli_denovo	4801328	(G/A)

S.N.	SNP IDs	Cultivars	Chromosomes/scaffolds	Physical positions (bp)	SNPs
18050	CakSNP18050	*Kabuli*	Ca_Kabuli_denovo	4801337	(G/A)
18051	CakSNP18051	*Kabuli*	Ca_Kabuli_denovo	4801346	(C/T)
18052	CakSNP18052	*Kabuli*	Ca_Kabuli_denovo	4804447	(A/G)
18053	CakSNP18053	*Kabuli*	Ca_Kabuli_denovo	4829417	(G/C)
18054	CakSNP18054	*Kabuli*	Ca_Kabuli_denovo	4829418	(T/C)
18055	CakSNP18055	*Kabuli*	Ca_Kabuli_denovo	4837576	(C/T)
18056	CakSNP18056	*Kabuli*	Ca_Kabuli_denovo	4848221	(C/T)
18057	CakSNP18057	*Kabuli*	Ca_Kabuli_denovo	4848227	(G/A)
18058	CakSNP18058	*Kabuli*	Ca_Kabuli_denovo	4849347	(C/G)
18059	CakSNP18059	*Kabuli*	Ca_Kabuli_denovo	4870801	(G/A)
18060	CakSNP18060	*Kabuli*	Ca_Kabuli_denovo	4910257	(G/A)
18061	CakSNP18061	*Kabuli*	Ca_Kabuli_denovo	4931630	(C/T)
18062	CakSNP18062	*Kabuli*	Ca_Kabuli_denovo	4956229	(A/G)
18063	CakSNP18063	*Kabuli*	Ca_Kabuli_denovo	4970754	(A/C)
18064	CakSNP18064	*Kabuli*	Ca_Kabuli_denovo	4984720	(G/A)
18065	CakSNP18065	*Kabuli*	Ca_Kabuli_denovo	4984729	(C/T)
18066	CakSNP18066	*Kabuli*	Ca_Kabuli_denovo	4986581	(T/C)
18067	CakSNP18067	*Kabuli*	Ca_Kabuli_denovo	4997664	(G/A)
18068	CakSNP18068	*Kabuli*	Ca_Kabuli_denovo	4999327	(A/G)
18069	CakSNP18069	*Kabuli*	Ca_Kabuli_denovo	4999329	(G/A)
18070	CakSNP18070	*Kabuli*	Ca_Kabuli_denovo	4999334	(G/C)
18071	CakSNP18071	*Kabuli*	Ca_Kabuli_denovo	4999336	(C/G)
18072	CakSNP18072	*Kabuli*	Ca_Kabuli_denovo	4999337	(A/C)
18073	CakSNP18073	*Kabuli*	Ca_Kabuli_denovo	4999367	(T/G)
18074	CakSNP18074	*Kabuli*	Ca_Kabuli_denovo	5021916	(A/T)
18075	CakSNP18075	*Kabuli*	Ca_Kabuli_denovo	5021954	(C/T)
18076	CakSNP18076	*Kabuli*	Ca_Kabuli_denovo	5036439	(C/A)
18077	CakSNP18077	*Kabuli*	Ca_Kabuli_denovo	5085488	(C/T)
18078	CakSNP18078	*Kabuli*	Ca_Kabuli_denovo	5104591	(A/G)
18079	CakSNP18079	*Kabuli*	Ca_Kabuli_denovo	5109203	(T/C)
18080	CakSNP18080	*Kabuli*	Ca_Kabuli_denovo	5128398	(C/G)
18081	CakSNP18081	*Kabuli*	Ca_Kabuli_denovo	5128413	(A/G)
18082	CakSNP18082	*Kabuli*	Ca_Kabuli_denovo	5154944	(C/G)
18083	CakSNP18083	*Kabuli*	Ca_Kabuli_denovo	5155600	(C/G)
18084	CakSNP18084	*Kabuli*	Ca_Kabuli_denovo	5191898	(C/T)
18085	CakSNP18085	*Kabuli*	Ca_Kabuli_denovo	5196708	(T/A)
18086	CakSNP18086	*Kabuli*	Ca_Kabuli_denovo	5229436	(G/C)
18087	CakSNP18087	*Kabuli*	Ca_Kabuli_denovo	5230153	(C/G)

S.N.	SNP IDs	Cultivars	Chromosomes/scaffolds	Physical positions (bp)	SNPs
18088	CakSNP18088	*Kabuli*	Ca_Kabuli_denovo	5230160	(T/C)
18089	CakSNP18089	*Kabuli*	Ca_Kabuli_denovo	5236751	(G/A)
18090	CakSNP18090	*Kabuli*	Ca_Kabuli_denovo	5236789	(T/A)
18091	CakSNP18091	*Kabuli*	Ca_Kabuli_denovo	5238535	(T/A)
18092	CakSNP18092	*Kabuli*	Ca_Kabuli_denovo	5238598	(A/G)
18093	CakSNP18093	*Kabuli*	Ca_Kabuli_denovo	5263444	(C/T)
18094	CakSNP18094	*Kabuli*	Ca_Kabuli_denovo	5273594	(G/A)
18095	CakSNP18095	*Kabuli*	Ca_Kabuli_denovo	5273656	(C/G)
18096	CakSNP18096	*Kabuli*	Ca_Kabuli_denovo	5275340	(G/A)
18097	CakSNP18097	*Kabuli*	Ca_Kabuli_denovo	5280137	(G/A)
18098	CakSNP18098	*Kabuli*	Ca_Kabuli_denovo	5305760	(G/C)
18099	CakSNP18099	*Kabuli*	Ca_Kabuli_denovo	5329084	(G/C)
18100	CakSNP18100	*Kabuli*	Ca_Kabuli_denovo	5329559	(T/C)
18101	CakSNP18101	*Kabuli*	Ca_Kabuli_denovo	5329562	(C/G)
18102	CakSNP18102	*Kabuli*	Ca_Kabuli_denovo	5332558	(C/T)
18103	CakSNP18103	*Kabuli*	Ca_Kabuli_denovo	5332561	(C/A)
18104	CakSNP18104	*Kabuli*	Ca_Kabuli_denovo	5337094	(G/C)
18105	CakSNP18105	*Kabuli*	Ca_Kabuli_denovo	5357564	(C/T)
18106	CakSNP18106	*Kabuli*	Ca_Kabuli_denovo	5358806	(A/G)
18107	CakSNP18107	*Kabuli*	Ca_Kabuli_denovo	5388664	(A/T)
18108	CakSNP18108	*Kabuli*	Ca_Kabuli_denovo	5402790	(G/A)
18109	CakSNP18109	*Kabuli*	Ca_Kabuli_denovo	5402793	(C/T)
18110	CakSNP18110	*Kabuli*	Ca_Kabuli_denovo	5470445	(A/C)
18111	CakSNP18111	*Kabuli*	Ca_Kabuli_denovo	5470446	(G/C)
18112	CakSNP18112	*Kabuli*	Ca_Kabuli_denovo	5470447	(A/G)
18113	CakSNP18113	*Kabuli*	Ca_Kabuli_denovo	5471395	(C/T)
18114	CakSNP18114	*Kabuli*	Ca_Kabuli_denovo	5477856	(C/A)
18115	CakSNP18115	*Kabuli*	Ca_Kabuli_denovo	5529333	(C/G)
18116	CakSNP18116	*Kabuli*	Ca_Kabuli_denovo	5529359	(C/T)
18117	CakSNP18117	*Kabuli*	Ca_Kabuli_denovo	5558990	(C/T)
18118	CakSNP18118	*Kabuli*	Ca_Kabuli_denovo	5559026	(C/A)
18119	CakSNP18119	*Kabuli*	Ca_Kabuli_denovo	5568535	(C/G)
18120	CakSNP18120	*Kabuli*	Ca_Kabuli_denovo	5582991	(A/T)
18121	CakSNP18121	*Kabuli*	Ca_Kabuli_denovo	5592088	(C/T)
18122	CakSNP18122	*Kabuli*	Ca_Kabuli_denovo	5592090	(G/T)
18123	CakSNP18123	*Kabuli*	Ca_Kabuli_denovo	5596926	(A/C)
18124	CakSNP18124	*Kabuli*	Ca_Kabuli_denovo	5681190	(A/T)
18125	CakSNP18125	*Kabuli*	Ca_Kabuli_denovo	5681191	(C/T)

S.N.	SNP IDs	Cultivars	Chromosomes/scaffolds	Physical positions (bp)	SNPs
18126	CakSNP18126	*Kabuli*	Ca_Kabuli_denovo	5725564	(C/T)
18127	CakSNP18127	*Kabuli*	Ca_Kabuli_denovo	5725609	(G/A)
18128	CakSNP18128	*Kabuli*	Ca_Kabuli_denovo	5766882	(T/G)
18129	CakSNP18129	*Kabuli*	Ca_Kabuli_denovo	5766883	(T/A)
18130	CakSNP18130	*Kabuli*	Ca_Kabuli_denovo	5773213	(C/T)
18131	CakSNP18131	*Kabuli*	Ca_Kabuli_denovo	5781658	(C/G)
18132	CakSNP18132	*Kabuli*	Ca_Kabuli_denovo	5806886	(T/C)
18133	CakSNP18133	*Kabuli*	Ca_Kabuli_denovo	5816220	(A/C)
18134	CakSNP18134	*Kabuli*	Ca_Kabuli_denovo	5819283	(G/C)
18135	CakSNP18135	*Kabuli*	Ca_Kabuli_denovo	5848818	(T/A)
18136	CakSNP18136	*Kabuli*	Ca_Kabuli_denovo	5856817	(G/A)
18137	CakSNP18137	*Kabuli*	Ca_Kabuli_denovo	5863013	(C/A)
18138	CakSNP18138	*Kabuli*	Ca_Kabuli_denovo	5863024	(G/A)
18139	CakSNP18139	*Kabuli*	Ca_Kabuli_denovo	5864127	(G/A)
18140	CakSNP18140	*Kabuli*	Ca_Kabuli_denovo	5864153	(G/C)
18141	CakSNP18141	*Kabuli*	Ca_Kabuli_denovo	5873238	(C/A)
18142	CakSNP18142	*Kabuli*	Ca_Kabuli_denovo	5873308	(A/G)
18143	CakSNP18143	*Kabuli*	Ca_Kabuli_denovo	5879375	(G/T)
18144	CakSNP18144	*Kabuli*	Ca_Kabuli_denovo	5879387	(C/G)
18145	CakSNP18145	*Kabuli*	Ca_Kabuli_denovo	5879389	(C/A)
18146	CakSNP18146	*Kabuli*	Ca_Kabuli_denovo	5879418	(T/G)
18147	CakSNP18147	*Kabuli*	Ca_Kabuli_denovo	5879435	(C/A)
18148	CakSNP18148	*Kabuli*	Ca_Kabuli_denovo	5879445	(G/T)
18149	CakSNP18149	*Kabuli*	Ca_Kabuli_denovo	5879451	(C/A)
18150	CakSNP18150	*Kabuli*	Ca_Kabuli_denovo	5890893	(T/A)
18151	CakSNP18151	*Kabuli*	Ca_Kabuli_denovo	5890894	(G/A)
18152	CakSNP18152	*Kabuli*	Ca_Kabuli_denovo	5890895	(G/T)
18153	CakSNP18153	*Kabuli*	Ca_Kabuli_denovo	5890898	(C/T)
18154	CakSNP18154	*Kabuli*	Ca_Kabuli_denovo	5890899	(A/T)
18155	CakSNP18155	*Kabuli*	Ca_Kabuli_denovo	5919819	(C/G)
18156	CakSNP18156	*Kabuli*	Ca_Kabuli_denovo	5952126	(A/G)
18157	CakSNP18157	*Kabuli*	Ca_Kabuli_denovo	5952154	(A/C)
18158	CakSNP18158	*Kabuli*	Ca_Kabuli_denovo	5952171	(A/G)
18159	CakSNP18159	*Kabuli*	Ca_Kabuli_denovo	5963529	(G/A)
18160	CakSNP18160	*Kabuli*	Ca_Kabuli_denovo	5963530	(T/A)
18161	CakSNP18161	*Kabuli*	Ca_Kabuli_denovo	5963531	(T/C)
18162	CakSNP18162	*Kabuli*	Ca_Kabuli_denovo	5963533	(G/A)
18163	CakSNP18163	*Kabuli*	Ca_Kabuli_denovo	5971199	(T/G)

S.N.	SNP IDs	Cultivars	Chromosomes/scaffolds	Physical positions (bp)	SNPs
18164	CakSNP18164	*Kabuli*	Ca_Kabuli_denovo	6010227	(T/A)
18165	CakSNP18165	*Kabuli*	Ca_Kabuli_denovo	6010251	(T/G)
18166	CakSNP18166	*Kabuli*	Ca_Kabuli_denovo	6010287	(G/T)
18167	CakSNP18167	*Kabuli*	Ca_Kabuli_denovo	6010311	(A/G)
18168	CakSNP18168	*Kabuli*	Ca_Kabuli_denovo	6010573	(G/A)
18169	CakSNP18169	*Kabuli*	Ca_Kabuli_denovo	6012251	(A/C)
18170	CakSNP18170	*Kabuli*	Ca_Kabuli_denovo	6014521	(C/T)
18171	CakSNP18171	*Kabuli*	Ca_Kabuli_denovo	6014733	(T/G)
18172	CakSNP18172	*Kabuli*	Ca_Kabuli_denovo	6014753	(T/G)
18173	CakSNP18173	*Kabuli*	Ca_Kabuli_denovo	6014754	(T/A)
18174	CakSNP18174	*Kabuli*	Ca_Kabuli_denovo	6014921	(A/C)
18175	CakSNP18175	*Kabuli*	Ca_Kabuli_denovo	6017294	(G/T)
18176	CakSNP18176	*Kabuli*	Ca_Kabuli_denovo	6017306	(T/G)
18177	CakSNP18177	*Kabuli*	Ca_Kabuli_denovo	6017326	(C/G)
18178	CakSNP18178	*Kabuli*	Ca_Kabuli_denovo	6017328	(T/G)
18179	CakSNP18179	*Kabuli*	Ca_Kabuli_denovo	6017335	(A/G)
18180	CakSNP18180	*Kabuli*	Ca_Kabuli_denovo	6017341	(A/G)
18181	CakSNP18181	*Kabuli*	Ca_Kabuli_denovo	6017344	(C/A)
18182	CakSNP18182	*Kabuli*	Ca_Kabuli_denovo	6017345	(C/G)
18183	CakSNP18183	*Kabuli*	Ca_Kabuli_denovo	6017348	(T/G)
18184	CakSNP18184	*Kabuli*	Ca_Kabuli_denovo	6034062	(T/C)
18185	CakSNP18185	*Kabuli*	Ca_Kabuli_denovo	6034071	(A/G)
18186	CakSNP18186	*Kabuli*	Ca_Kabuli_denovo	6043616	(A/G)
18187	CakSNP18187	*Kabuli*	Ca_Kabuli_denovo	6043627	(G/T)
18188	CakSNP18188	*Kabuli*	Ca_Kabuli_denovo	6047989	(T/G)
18189	CakSNP18189	*Kabuli*	Ca_Kabuli_denovo	6051272	(T/C)
18190	CakSNP18190	*Kabuli*	Ca_Kabuli_denovo	6057777	(G/A)
18191	CakSNP18191	*Kabuli*	Ca_Kabuli_denovo	6059536	(A/G)
18192	CakSNP18192	*Kabuli*	Ca_Kabuli_denovo	6059551	(A/G)
18193	CakSNP18193	*Kabuli*	Ca_Kabuli_denovo	6059588	(A/C)
18194	CakSNP18194	*Kabuli*	Ca_Kabuli_denovo	6098413	(G/A)
18195	CakSNP18195	*Kabuli*	Ca_Kabuli_denovo	6103875	(G/A)
18196	CakSNP18196	*Kabuli*	Ca_Kabuli_denovo	6117175	(C/A)
18197	CakSNP18197	*Kabuli*	Ca_Kabuli_denovo	6117188	(C/A)
18198	CakSNP18198	*Kabuli*	Ca_Kabuli_denovo	6117213	(C/G)
18199	CakSNP18199	*Kabuli*	Ca_Kabuli_denovo	6117215	(A/G)
18200	CakSNP18200	*Kabuli*	Ca_Kabuli_denovo	6165667	(T/C)
18201	CakSNP18201	*Kabuli*	Ca_Kabuli_denovo	6182275	(T/G)

S.N.	SNP IDs	Cultivars	Chromosomes/scaffolds	Physical positions (bp)	SNPs
18202	CakSNP18202	*Kabuli*	Ca_Kabuli_denovo	6182281	(G/C)
18203	CakSNP18203	*Kabuli*	Ca_Kabuli_denovo	6190775	(T/C)
18204	CakSNP18204	*Kabuli*	Ca_Kabuli_denovo	6191143	(G/C)
18205	CakSNP18205	*Kabuli*	Ca_Kabuli_denovo	6191170	(G/A)
18206	CakSNP18206	*Kabuli*	Ca_Kabuli_denovo	6237722	(G/A)
18207	CakSNP18207	*Kabuli*	Ca_Kabuli_denovo	6258541	(C/G)
18208	CakSNP18208	*Kabuli*	Ca_Kabuli_denovo	6264608	(C/T)
18209	CakSNP18209	*Kabuli*	Ca_Kabuli_denovo	6300610	(A/G)
18210	CakSNP18210	*Kabuli*	Ca_Kabuli_denovo	6300657	(T/C)
18211	CakSNP18211	*Kabuli*	Ca_Kabuli_denovo	6310736	(G/C)
18212	CakSNP18212	*Kabuli*	Ca_Kabuli_denovo	6310744	(T/C)
18213	CakSNP18213	*Kabuli*	Ca_Kabuli_denovo	6321154	(G/A)
18214	CakSNP18214	*Kabuli*	Ca_Kabuli_denovo	6355193	(T/G)
18215	CakSNP18215	*Kabuli*	Ca_Kabuli_denovo	6368181	(G/A)
18216	CakSNP18216	*Kabuli*	Ca_Kabuli_denovo	6377203	(C/G)
18217	CakSNP18217	*Kabuli*	Ca_Kabuli_denovo	6399060	(G/A)
18218	CakSNP18218	*Kabuli*	Ca_Kabuli_denovo	6402317	(G/C)
18219	CakSNP18219	*Kabuli*	Ca_Kabuli_denovo	6402384	(C/T)
18220	CakSNP18220	*Kabuli*	Ca_Kabuli_denovo	6402397	(A/C)
18221	CakSNP18221	*Kabuli*	Ca_Kabuli_denovo	6409872	(T/A)
18222	CakSNP18222	*Kabuli*	Ca_Kabuli_denovo	6411767	(C/G)
18223	CakSNP18223	*Kabuli*	Ca_Kabuli_denovo	6411770	(C/T)
18224	CakSNP18224	*Kabuli*	Ca_Kabuli_denovo	6419754	(G/A)
18225	CakSNP18225	*Kabuli*	Ca_Kabuli_denovo	6427894	(T/C)
18226	CakSNP18226	*Kabuli*	Ca_Kabuli_denovo	6462028	(C/T)
18227	CakSNP18227	*Kabuli*	Ca_Kabuli_denovo	6475346	(G/A)
18228	CakSNP18228	*Kabuli*	Ca_Kabuli_denovo	6475348	(T/C)
18229	CakSNP18229	*Kabuli*	Ca_Kabuli_denovo	6480559	(A/G)
18230	CakSNP18230	*Kabuli*	Ca_Kabuli_denovo	6480580	(C/G)
18231	CakSNP18231	*Kabuli*	Ca_Kabuli_denovo	6486758	(G/A)
18232	CakSNP18232	*Kabuli*	Ca_Kabuli_denovo	6486782	(C/T)
18233	CakSNP18233	*Kabuli*	Ca_Kabuli_denovo	6526925	(A/C)
18234	CakSNP18234	*Kabuli*	Ca_Kabuli_denovo	6531409	(T/C)
18235	CakSNP18235	*Kabuli*	Ca_Kabuli_denovo	6545167	(G/C)
18236	CakSNP18236	*Kabuli*	Ca_Kabuli_denovo	6545175	(A/G)
18237	CakSNP18237	*Kabuli*	Ca_Kabuli_denovo	6552139	(C/T)
18238	CakSNP18238	*Kabuli*	Ca_Kabuli_denovo	6552142	(G/T)
18239	CakSNP18239	*Kabuli*	Ca_Kabuli_denovo	6552157	(G/T)

S.N.	SNP IDs	Cultivars	Chromosomes/scaffolds	Physical positions (bp)	SNPs
18240	CakSNP18240	*Kabuli*	Ca_Kabuli_denovo	6552161	(T/A)
18241	CakSNP18241	*Kabuli*	Ca_Kabuli_denovo	6559984	(C/T)
18242	CakSNP18242	*Kabuli*	Ca_Kabuli_denovo	6559996	(G/A)
18243	CakSNP18243	*Kabuli*	Ca_Kabuli_denovo	6560006	(A/G)
18244	CakSNP18244	*Kabuli*	Ca_Kabuli_denovo	6565348	(T/C)
18245	CakSNP18245	*Kabuli*	Ca_Kabuli_denovo	6581608	(A/C)
18246	CakSNP18246	*Kabuli*	Ca_Kabuli_denovo	6583973	(T/G)
18247	CakSNP18247	*Kabuli*	Ca_Kabuli_denovo	6590097	(C/T)
18248	CakSNP18248	*Kabuli*	Ca_Kabuli_denovo	6590129	(G/A)
18249	CakSNP18249	*Kabuli*	Ca_Kabuli_denovo	6590156	(T/G)
18250	CakSNP18250	*Kabuli*	Ca_Kabuli_denovo	6599573	(A/G)
18251	CakSNP18251	*Kabuli*	Ca_Kabuli_denovo	6612721	(A/G)
18252	CakSNP18252	*Kabuli*	Ca_Kabuli_denovo	6663521	(C/G)
18253	CakSNP18253	*Kabuli*	Ca_Kabuli_denovo	6663527	(G/A)
18254	CakSNP18254	*Kabuli*	Ca_Kabuli_denovo	6688010	(C/G)
18255	CakSNP18255	*Kabuli*	Ca_Kabuli_denovo	6706345	(A/G)
18256	CakSNP18256	*Kabuli*	Ca_Kabuli_denovo	6707272	(T/C)
18257	CakSNP18257	*Kabuli*	Ca_Kabuli_denovo	6711061	(A/G)
18258	CakSNP18258	*Kabuli*	Ca_Kabuli_denovo	6712133	(C/G)
18259	CakSNP18259	*Kabuli*	Ca_Kabuli_denovo	6712135	(G/C)
18260	CakSNP18260	*Kabuli*	Ca_Kabuli_denovo	6712138	(C/A)
18261	CakSNP18261	*Kabuli*	Ca_Kabuli_denovo	6712141	(A/T)
18262	CakSNP18262	*Kabuli*	Ca_Kabuli_denovo	6712146	(G/T)
18263	CakSNP18263	*Kabuli*	Ca_Kabuli_denovo	6712147	(T/G)
18264	CakSNP18264	*Kabuli*	Ca_Kabuli_denovo	6720667	(A/G)
18265	CakSNP18265	*Kabuli*	Ca_Kabuli_denovo	6785596	(T/A)
18266	CakSNP18266	*Kabuli*	Ca_Kabuli_denovo	6791052	(C/A)
18267	CakSNP18267	*Kabuli*	Ca_Kabuli_denovo	6791071	(A/C)
18268	CakSNP18268	*Kabuli*	Ca_Kabuli_denovo	6800910	(A/T)
18269	CakSNP18269	*Kabuli*	Ca_Kabuli_denovo	6800913	(A/G)
18270	CakSNP18270	*Kabuli*	Ca_Kabuli_denovo	6800915	(G/A)
18271	CakSNP18271	*Kabuli*	Ca_Kabuli_denovo	6800927	(G/C)
18272	CakSNP18272	*Kabuli*	Ca_Kabuli_denovo	6800944	(G/C)
18273	CakSNP18273	*Kabuli*	Ca_Kabuli_denovo	6800949	(T/C)
18274	CakSNP18274	*Kabuli*	Ca_Kabuli_denovo	6811156	(G/T)
18275	CakSNP18275	*Kabuli*	Ca_Kabuli_denovo	6811456	(A/T)
18276	CakSNP18276	*Kabuli*	Ca_Kabuli_denovo	6811472	(A/T)
18277	CakSNP18277	*Kabuli*	Ca_Kabuli_denovo	6813703	(A/G)

S.N.	SNP IDs	Cultivars	Chromosomes/scaffolds	Physical positions (bp)	SNPs
18278	CakSNP18278	*Kabuli*	Ca_Kabuli_denovo	6861843	(T/C)
18279	CakSNP18279	*Kabuli*	Ca_Kabuli_denovo	6861855	(G/A)
18280	CakSNP18280	*Kabuli*	Ca_Kabuli_denovo	6869394	(T/C)
18281	CakSNP18281	*Kabuli*	Ca_Kabuli_denovo	6870117	(C/T)
18282	CakSNP18282	*Kabuli*	Ca_Kabuli_denovo	6870139	(G/A)
18283	CakSNP18283	*Kabuli*	Ca_Kabuli_denovo	6873819	(A/T)
18284	CakSNP18284	*Kabuli*	Ca_Kabuli_denovo	6873823	(T/G)
18285	CakSNP18285	*Kabuli*	Ca_Kabuli_denovo	6873825	(T/C)
18286	CakSNP18286	*Kabuli*	Ca_Kabuli_denovo	6873826	(C/T)
18287	CakSNP18287	*Kabuli*	Ca_Kabuli_denovo	6874179	(C/A)
18288	CakSNP18288	*Kabuli*	Ca_Kabuli_denovo	6874189	(G/T)
18289	CakSNP18289	*Kabuli*	Ca_Kabuli_denovo	6877071	(G/A)
18290	CakSNP18290	*Kabuli*	Ca_Kabuli_denovo	6882102	(C/G)
18291	CakSNP18291	*Kabuli*	Ca_Kabuli_denovo	6882109	(T/C)
18292	CakSNP18292	*Kabuli*	Ca_Kabuli_denovo	6882113	(T/A)
18293	CakSNP18293	*Kabuli*	Ca_Kabuli_denovo	6882804	(T/A)
18294	CakSNP18294	*Kabuli*	Ca_Kabuli_denovo	6894538	(C/G)
18295	CakSNP18295	*Kabuli*	Ca_Kabuli_denovo	6894541	(A/G)
18296	CakSNP18296	*Kabuli*	Ca_Kabuli_denovo	6894542	(C/A)
18297	CakSNP18297	*Kabuli*	Ca_Kabuli_denovo	6898197	(A/G)
18298	CakSNP18298	*Kabuli*	Ca_Kabuli_denovo	6907485	(T/A)
18299	CakSNP18299	*Kabuli*	Ca_Kabuli_denovo	6907494	(A/G)
18300	CakSNP18300	*Kabuli*	Ca_Kabuli_denovo	6916345	(C/G)
18301	CakSNP18301	*Kabuli*	Ca_Kabuli_denovo	6916346	(T/A)
18302	CakSNP18302	*Kabuli*	Ca_Kabuli_denovo	6916357	(C/T)
18303	CakSNP18303	*Kabuli*	Ca_Kabuli_denovo	6916359	(T/C)
18304	CakSNP18304	*Kabuli*	Ca_Kabuli_denovo	6918579	(A/G)
18305	CakSNP18305	*Kabuli*	Ca_Kabuli_denovo	6918593	(C/G)
18306	CakSNP18306	*Kabuli*	Ca_Kabuli_denovo	6923821	(A/T)
18307	CakSNP18307	*Kabuli*	Ca_Kabuli_denovo	6929548	(A/T)
18308	CakSNP18308	*Kabuli*	Ca_Kabuli_denovo	6939673	(G/A)
18309	CakSNP18309	*Kabuli*	Ca_Kabuli_denovo	6939691	(A/T)
18310	CakSNP18310	*Kabuli*	Ca_Kabuli_denovo	6944977	(A/C)
18311	CakSNP18311	*Kabuli*	Ca_Kabuli_denovo	6945035	(C/G)
18312	CakSNP18312	*Kabuli*	Ca_Kabuli_denovo	6945036	(A/G)
18313	CakSNP18313	*Kabuli*	Ca_Kabuli_denovo	6956695	(C/T)
18314	CakSNP18314	*Kabuli*	Ca_Kabuli_denovo	6956707	(A/G)
18315	CakSNP18315	*Kabuli*	Ca_Kabuli_denovo	6959682	(C/G)

S.N.	SNP IDs	Cultivars	Chromosomes/scaffolds	Physical positions (bp)	SNPs
18316	CakSNP18316	*Kabuli*	Ca_Kabuli_denovo	6959727	(T/C)
18317	CakSNP18317	*Kabuli*	Ca_Kabuli_denovo	6960786	(G/A)
18318	CakSNP18318	*Kabuli*	Ca_Kabuli_denovo	6960796	(C/A)
18319	CakSNP18319	*Kabuli*	Ca_Kabuli_denovo	6960820	(C/G)
18320	CakSNP18320	*Kabuli*	Ca_Kabuli_denovo	6975387	(A/G)
18321	CakSNP18321	*Kabuli*	Ca_Kabuli_denovo	6975388	(T/A)
18322	CakSNP18322	*Kabuli*	Ca_Kabuli_denovo	6975430	(T/C)
18323	CakSNP18323	*Kabuli*	Ca_Kabuli_denovo	6975851	(T/C)
18324	CakSNP18324	*Kabuli*	Ca_Kabuli_denovo	6975869	(C/G)
18325	CakSNP18325	*Kabuli*	Ca_Kabuli_denovo	6976418	(C/G)
18326	CakSNP18326	*Kabuli*	Ca_Kabuli_denovo	6976441	(T/C)
18327	CakSNP18327	*Kabuli*	Ca_Kabuli_denovo	6985651	(T/C)
18328	CakSNP18328	*Kabuli*	Ca_Kabuli_denovo	6998248	(A/G)
18329	CakSNP18329	*Kabuli*	Ca_Kabuli_denovo	6998250	(C/A)
18330	CakSNP18330	*Kabuli*	Ca_Kabuli_denovo	7000764	(A/T)
18331	CakSNP18331	*Kabuli*	Ca_Kabuli_denovo	7012691	(A/G)
18332	CakSNP18332	*Kabuli*	Ca_Kabuli_denovo	7017046	(T/C)
18333	CakSNP18333	*Kabuli*	Ca_Kabuli_denovo	7017051	(A/G)
18334	CakSNP18334	*Kabuli*	Ca_Kabuli_denovo	7017052	(T/C)
18335	CakSNP18335	*Kabuli*	Ca_Kabuli_denovo	7017053	(A/C)
18336	CakSNP18336	*Kabuli*	Ca_Kabuli_denovo	7017058	(G/C)
18337	CakSNP18337	*Kabuli*	Ca_Kabuli_denovo	7017061	(A/T)
18338	CakSNP18338	*Kabuli*	Ca_Kabuli_denovo	7017065	(T/C)
18339	CakSNP18339	*Kabuli*	Ca_Kabuli_denovo	7017071	(C/T)
18340	CakSNP18340	*Kabuli*	Ca_Kabuli_denovo	7017079	(C/G)
18341	CakSNP18341	*Kabuli*	Ca_Kabuli_denovo	7017080	(A/G)
18342	CakSNP18342	*Kabuli*	Ca_Kabuli_denovo	7017081	(G/T)
18343	CakSNP18343	*Kabuli*	Ca_Kabuli_denovo	7069247	(G/A)
18344	CakSNP18344	*Kabuli*	Ca_Kabuli_denovo	7069273	(C/T)
18345	CakSNP18345	*Kabuli*	Ca_Kabuli_denovo	7069930	(G/C)
18346	CakSNP18346	*Kabuli*	Ca_Kabuli_denovo	7070108	(A/G)
18347	CakSNP18347	*Kabuli*	Ca_Kabuli_denovo	7070110	(G/A)
18348	CakSNP18348	*Kabuli*	Ca_Kabuli_denovo	7070111	(A/G)
18349	CakSNP18349	*Kabuli*	Ca_Kabuli_denovo	7070113	(C/G)
18350	CakSNP18350	*Kabuli*	Ca_Kabuli_denovo	7070115	(T/G)
18351	CakSNP18351	*Kabuli*	Ca_Kabuli_denovo	7070117	(C/T)
18352	CakSNP18352	*Kabuli*	Ca_Kabuli_denovo	7086244	(A/G)
18353	CakSNP18353	*Kabuli*	Ca_Kabuli_denovo	7103380	(A/C)

S.N.	SNP IDs	Cultivars	Chromosomes/scaffolds	Physical positions (bp)	SNPs
18354	CakSNP18354	*Kabuli*	Ca_Kabuli_denovo	7127589	(G/A)
18355	CakSNP18355	*Kabuli*	Ca_Kabuli_denovo	7131343	(G/A)
18356	CakSNP18356	*Kabuli*	Ca_Kabuli_denovo	7141293	(A/G)
18357	CakSNP18357	*Kabuli*	Ca_Kabuli_denovo	7141324	(A/G)
18358	CakSNP18358	*Kabuli*	Ca_Kabuli_denovo	7143296	(G/A)
18359	CakSNP18359	*Kabuli*	Ca_Kabuli_denovo	7143640	(G/A)
18360	CakSNP18360	*Kabuli*	Ca_Kabuli_denovo	7143649	(G/A)
18361	CakSNP18361	*Kabuli*	Ca_Kabuli_denovo	7143693	(T/C)
18362	CakSNP18362	*Kabuli*	Ca_Kabuli_denovo	7146142	(C/T)
18363	CakSNP18363	*Kabuli*	Ca_Kabuli_denovo	7146173	(C/T)
18364	CakSNP18364	*Kabuli*	Ca_Kabuli_denovo	7149196	(C/T)
18365	CakSNP18365	*Kabuli*	Ca_Kabuli_denovo	7152489	(C/T)
18366	CakSNP18366	*Kabuli*	Ca_Kabuli_denovo	7183627	(A/G)
18367	CakSNP18367	*Kabuli*	Ca_Kabuli_denovo	7191526	(C/T)
18368	CakSNP18368	*Kabuli*	Ca_Kabuli_denovo	7246580	(C/T)
18369	CakSNP18369	*Kabuli*	Ca_Kabuli_denovo	7254020	(C/G)
18370	CakSNP18370	*Kabuli*	Ca_Kabuli_denovo	7254039	(T/C)
18371	CakSNP18371	*Kabuli*	Ca_Kabuli_denovo	7257747	(G/C)
18372	CakSNP18372	*Kabuli*	Ca_Kabuli_denovo	7267238	(C/T)
18373	CakSNP18373	*Kabuli*	Ca_Kabuli_denovo	7267256	(C/A)
18374	CakSNP18374	*Kabuli*	Ca_Kabuli_denovo	7267260	(A/T)
18375	CakSNP18375	*Kabuli*	Ca_Kabuli_denovo	7311958	(G/A)
18376	CakSNP18376	*Kabuli*	Ca_Kabuli_denovo	7328061	(A/G)
18377	CakSNP18377	*Kabuli*	Ca_Kabuli_denovo	7344433	(T/A)
18378	CakSNP18378	*Kabuli*	Ca_Kabuli_denovo	7369005	(G/A)
18379	CakSNP18379	*Kabuli*	Ca_Kabuli_denovo	7375018	(T/C)
18380	CakSNP18380	*Kabuli*	Ca_Kabuli_denovo	7382267	(T/C)
18381	CakSNP18381	*Kabuli*	Ca_Kabuli_denovo	7382310	(G/T)
18382	CakSNP18382	*Kabuli*	Ca_Kabuli_denovo	7391587	(C/G)
18383	CakSNP18383	*Kabuli*	Ca_Kabuli_denovo	7391613	(T/G)
18384	CakSNP18384	*Kabuli*	Ca_Kabuli_denovo	7391949	(C/T)
18385	CakSNP18385	*Kabuli*	Ca_Kabuli_denovo	7401051	(C/G)
18386	CakSNP18386	*Kabuli*	Ca_Kabuli_denovo	7401054	(G/T)
18387	CakSNP18387	*Kabuli*	Ca_Kabuli_denovo	7401077	(A/G)
18388	CakSNP18388	*Kabuli*	Ca_Kabuli_denovo	7401092	(G/A)
18389	CakSNP18389	*Kabuli*	Ca_Kabuli_denovo	7401100	(A/G)
18390	CakSNP18390	*Kabuli*	Ca_Kabuli_denovo	7401103	(C/T)
18391	CakSNP18391	*Kabuli*	Ca_Kabuli_denovo	7401244	(T/A)

S.N.	SNP IDs	Cultivars	Chromosomes/scaffolds	Physical positions (bp)	SNPs
18392	CakSNP18392	*Kabuli*	Ca_Kabuli_denovo	7416312	(C/T)
18393	CakSNP18393	*Kabuli*	Ca_Kabuli_denovo	7416345	(A/G)
18394	CakSNP18394	*Kabuli*	Ca_Kabuli_denovo	7427788	(G/A)
18395	CakSNP18395	*Kabuli*	Ca_Kabuli_denovo	7430435	(C/T)
18396	CakSNP18396	*Kabuli*	Ca_Kabuli_denovo	7430455	(A/T)
18397	CakSNP18397	*Kabuli*	Ca_Kabuli_denovo	7430464	(A/G)
18398	CakSNP18398	*Kabuli*	Ca_Kabuli_denovo	7452895	(C/G)
18399	CakSNP18399	*Kabuli*	Ca_Kabuli_denovo	7467529	(G/T)
18400	CakSNP18400	*Kabuli*	Ca_Kabuli_denovo	7470943	(T/G)
18401	CakSNP18401	*Kabuli*	Ca_Kabuli_denovo	7473234	(A/G)
18402	CakSNP18402	*Kabuli*	Ca_Kabuli_denovo	7477213	(G/A)
18403	CakSNP18403	*Kabuli*	Ca_Kabuli_denovo	7477273	(A/G)
18404	CakSNP18404	*Kabuli*	Ca_Kabuli_denovo	7477350	(C/T)
18405	CakSNP18405	*Kabuli*	Ca_Kabuli_denovo	7480213	(C/G)
18406	CakSNP18406	*Kabuli*	Ca_Kabuli_denovo	7480219	(C/T)
18407	CakSNP18407	*Kabuli*	Ca_Kabuli_denovo	7482161	(T/G)
18408	CakSNP18408	*Kabuli*	Ca_Kabuli_denovo	7494282	(T/A)
18409	CakSNP18409	*Kabuli*	Ca_Kabuli_denovo	7496672	(C/G)
18410	CakSNP18410	*Kabuli*	Ca_Kabuli_denovo	7496846	(A/G)
18411	CakSNP18411	*Kabuli*	Ca_Kabuli_denovo	7496849	(C/G)
18412	CakSNP18412	*Kabuli*	Ca_Kabuli_denovo	7500180	(G/A)
18413	CakSNP18413	*Kabuli*	Ca_Kabuli_denovo	7503626	(G/A)
18414	CakSNP18414	*Kabuli*	Ca_Kabuli_denovo	7504369	(C/G)
18415	CakSNP18415	*Kabuli*	Ca_Kabuli_denovo	7508116	(T/C)
18416	CakSNP18416	*Kabuli*	Ca_Kabuli_denovo	7511720	(C/G)
18417	CakSNP18417	*Kabuli*	Ca_Kabuli_denovo	7511741	(G/A)
18418	CakSNP18418	*Kabuli*	Ca_Kabuli_denovo	7511744	(G/C)
18419	CakSNP18419	*Kabuli*	Ca_Kabuli_denovo	7516002	(A/G)
18420	CakSNP18420	*Kabuli*	Ca_Kabuli_denovo	7516053	(G/A)
18421	CakSNP18421	*Kabuli*	Ca_Kabuli_denovo	7516067	(T/C)
18422	CakSNP18422	*Kabuli*	Ca_Kabuli_denovo	7518038	(A/G)
18423	CakSNP18423	*Kabuli*	Ca_Kabuli_denovo	7518231	(C/T)
18424	CakSNP18424	*Kabuli*	Ca_Kabuli_denovo	7519248	(G/A)
18425	CakSNP18425	*Kabuli*	Ca_Kabuli_denovo	7520456	(A/G)
18426	CakSNP18426	*Kabuli*	Ca_Kabuli_denovo	7520467	(T/C)
18427	CakSNP18427	*Kabuli*	Ca_Kabuli_denovo	7522835	(G/A)
18428	CakSNP18428	*Kabuli*	Ca_Kabuli_denovo	7532746	(T/C)
18429	CakSNP18429	*Kabuli*	Ca_Kabuli_denovo	7534604	(C/T)

S.N.	SNP IDs	Cultivars	Chromosomes/scaffolds	Physical positions (bp)	SNPs
18430	CakSNP18430	*Kabuli*	Ca_Kabuli_denovo	7537117	(G/A)
18431	CakSNP18431	*Kabuli*	Ca_Kabuli_denovo	7537138	(C/T)
18432	CakSNP18432	*Kabuli*	Ca_Kabuli_denovo	7539822	(C/T)
18433	CakSNP18433	*Kabuli*	Ca_Kabuli_denovo	7539825	(C/G)
18434	CakSNP18434	*Kabuli*	Ca_Kabuli_denovo	7546313	(A/G)
18435	CakSNP18435	*Kabuli*	Ca_Kabuli_denovo	7548311	(A/G)
18436	CakSNP18436	*Kabuli*	Ca_Kabuli_denovo	7549471	(T/C)
18437	CakSNP18437	*Kabuli*	Ca_Kabuli_denovo	7549507	(T/G)
18438	CakSNP18438	*Kabuli*	Ca_Kabuli_denovo	7553420	(A/G)
18439	CakSNP18439	*Kabuli*	Ca_Kabuli_denovo	7557108	(C/G)
18440	CakSNP18440	*Kabuli*	Ca_Kabuli_denovo	7557111	(G/C)
18441	CakSNP18441	*Kabuli*	Ca_Kabuli_denovo	7563251	(A/G)
18442	CakSNP18442	*Kabuli*	Ca_Kabuli_denovo	7563332	(C/G)
18443	CakSNP18443	*Kabuli*	Ca_Kabuli_denovo	7569104	(T/A)
18444	CakSNP18444	*Kabuli*	Ca_Kabuli_denovo	7569136	(A/C)
18445	CakSNP18445	*Kabuli*	Ca_Kabuli_denovo	7571356	(C/G)
18446	CakSNP18446	*Kabuli*	Ca_Kabuli_denovo	7581291	(T/C)
18447	CakSNP18447	*Kabuli*	Ca_Kabuli_denovo	7583443	(T/G)
18448	CakSNP18448	*Kabuli*	Ca_Kabuli_denovo	7586717	(T/C)
18449	CakSNP18449	*Kabuli*	Ca_Kabuli_denovo	7587722	(T/G)
18450	CakSNP18450	*Kabuli*	Ca_Kabuli_denovo	7590523	(A/G)
18451	CakSNP18451	*Kabuli*	Ca_Kabuli_denovo	7597523	(G/A)
18452	CakSNP18452	*Kabuli*	Ca_Kabuli_denovo	7597543	(G/T)
18453	CakSNP18453	*Kabuli*	Ca_Kabuli_denovo	7599351	(A/G)
18454	CakSNP18454	*Kabuli*	Ca_Kabuli_denovo	7599355	(G/A)
18455	CakSNP18455	*Kabuli*	Ca_Kabuli_denovo	7607382	(C/T)
18456	CakSNP18456	*Kabuli*	Ca_Kabuli_denovo	7619139	(G/A)
18457	CakSNP18457	*Kabuli*	Ca_Kabuli_denovo	7619163	(G/A)
18458	CakSNP18458	*Kabuli*	Ca_Kabuli_denovo	7621274	(A/G)
18459	CakSNP18459	*Kabuli*	Ca_Kabuli_denovo	7621346	(T/C)
18460	CakSNP18460	*Kabuli*	Ca_Kabuli_denovo	7632661	(A/G)
18461	CakSNP18461	*Kabuli*	Ca_Kabuli_denovo	7632682	(C/T)
18462	CakSNP18462	*Kabuli*	Ca_Kabuli_denovo	7634464	(G/A)
18463	CakSNP18463	*Kabuli*	Ca_Kabuli_denovo	7638848	(C/G)
18464	CakSNP18464	*Kabuli*	Ca_Kabuli_denovo	7638917	(A/C)
18465	CakSNP18465	*Kabuli*	Ca_Kabuli_denovo	7642288	(A/G)
18466	CakSNP18466	*Kabuli*	Ca_Kabuli_denovo	7644157	(G/A)
18467	CakSNP18467	*Kabuli*	Ca_Kabuli_denovo	7644247	(C/G)

S.N.	SNP IDs	Cultivars	Chromosomes/scaffolds	Physical positions (bp)	SNPs
18468	CakSNP18468	*Kabuli*	Ca_Kabuli_denovo	7644265	(C/T)
18469	CakSNP18469	*Kabuli*	Ca_Kabuli_denovo	7644275	(G/A)
18470	CakSNP18470	*Kabuli*	Ca_Kabuli_denovo	7644306	(G/A)
18471	CakSNP18471	*Kabuli*	Ca_Kabuli_denovo	7647077	(G/A)
18472	CakSNP18472	*Kabuli*	Ca_Kabuli_denovo	7659948	(T/A)
18473	CakSNP18473	*Kabuli*	Ca_Kabuli_denovo	7660004	(A/G)
18474	CakSNP18474	*Kabuli*	Ca_Kabuli_denovo	7661617	(G/T)
18475	CakSNP18475	*Kabuli*	Ca_Kabuli_denovo	7662572	(C/G)
18476	CakSNP18476	*Kabuli*	Ca_Kabuli_denovo	7662608	(A/G)
18477	CakSNP18477	*Kabuli*	Ca_Kabuli_denovo	7663940	(A/C)
18478	CakSNP18478	*Kabuli*	Ca_Kabuli_denovo	7663942	(A/G)
18479	CakSNP18479	*Kabuli*	Ca_Kabuli_denovo	7665276	(G/A)
18480	CakSNP18480	*Kabuli*	Ca_Kabuli_denovo	7668261	(C/T)
18481	CakSNP18481	*Kabuli*	Ca_Kabuli_denovo	7668301	(T/C)
18482	CakSNP18482	*Kabuli*	Ca_Kabuli_denovo	7671493	(C/T)
18483	CakSNP18483	*Kabuli*	Ca_Kabuli_denovo	7671508	(T/C)
18484	CakSNP18484	*Kabuli*	Ca_Kabuli_denovo	7674683	(A/C)
18485	CakSNP18485	*Kabuli*	Ca_Kabuli_denovo	7679874	(G/C)
18486	CakSNP18486	*Kabuli*	Ca_Kabuli_denovo	7681981	(G/T)
18487	CakSNP18487	*Kabuli*	Ca_Kabuli_denovo	7686913	(A/C)
18488	CakSNP18488	*Kabuli*	Ca_Kabuli_denovo	7686915	(G/A)
18489	CakSNP18489	*Kabuli*	Ca_Kabuli_denovo	7689434	(G/A)
18490	CakSNP18490	*Kabuli*	Ca_Kabuli_denovo	7689491	(G/A)
18491	CakSNP18491	*Kabuli*	Ca_Kabuli_denovo	7694767	(C/T)
18492	CakSNP18492	*Kabuli*	Ca_Kabuli_denovo	7694773	(T/C)
18493	CakSNP18493	*Kabuli*	Ca_Kabuli_denovo	7696282	(C/T)
18494	CakSNP18494	*Kabuli*	Ca_Kabuli_denovo	7698789	(G/C)
18495	CakSNP18495	*Kabuli*	Ca_Kabuli_denovo	7707323	(G/A)
18496	CakSNP18496	*Kabuli*	Ca_Kabuli_denovo	7713250	(C/T)
18497	CakSNP18497	*Kabuli*	Ca_Kabuli_denovo	7713283	(C/A)
18498	CakSNP18498	*Kabuli*	Ca_Kabuli_denovo	7714197	(A/G)
18499	CakSNP18499	*Kabuli*	Ca_Kabuli_denovo	7719731	(G/A)
18500	CakSNP18500	*Kabuli*	Ca_Kabuli_denovo	7722023	(T/G)
18501	CakSNP18501	*Kabuli*	Ca_Kabuli_denovo	7722035	(G/T)
18502	CakSNP18502	*Kabuli*	Ca_Kabuli_denovo	7736605	(G/A)
18503	CakSNP18503	*Kabuli*	Ca_Kabuli_denovo	7740036	(T/A)
18504	CakSNP18504	*Kabuli*	Ca_Kabuli_denovo	7742921	(A/G)
18505	CakSNP18505	*Kabuli*	Ca_Kabuli_denovo	7742924	(T/G)

S.N.	SNP IDs	Cultivars	Chromosomes/scaffolds	Physical positions (bp)	SNPs
18506	CakSNP18506	*Kabuli*	Ca_Kabuli_denovo	7744414	(C/T)
18507	CakSNP18507	*Kabuli*	Ca_Kabuli_denovo	7749013	(C/T)
18508	CakSNP18508	*Kabuli*	Ca_Kabuli_denovo	7755838	(A/G)
18509	CakSNP18509	*Kabuli*	Ca_Kabuli_denovo	7757665	(G/T)
18510	CakSNP18510	*Kabuli*	Ca_Kabuli_denovo	7763638	(A/C)
18511	CakSNP18511	*Kabuli*	Ca_Kabuli_denovo	7763641	(T/A)
18512	CakSNP18512	*Kabuli*	Ca_Kabuli_denovo	7773005	(C/G)
18513	CakSNP18513	*Kabuli*	Ca_Kabuli_denovo	7773497	(C/T)
18514	CakSNP18514	*Kabuli*	Ca_Kabuli_denovo	7775131	(C/A)
18515	CakSNP18515	*Kabuli*	Ca_Kabuli_denovo	7777349	(G/A)
18516	CakSNP18516	*Kabuli*	Ca_Kabuli_denovo	7777355	(C/G)
18517	CakSNP18517	*Kabuli*	Ca_Kabuli_denovo	7780110	(T/C)
18518	CakSNP18518	*Kabuli*	Ca_Kabuli_denovo	7780147	(T/C)
18519	CakSNP18519	*Kabuli*	Ca_Kabuli_denovo	7780424	(T/G)
18520	CakSNP18520	*Kabuli*	Ca_Kabuli_denovo	7783550	(T/C)
18521	CakSNP18521	*Kabuli*	Ca_Kabuli_denovo	7784298	(A/G)
18522	CakSNP18522	*Kabuli*	Ca_Kabuli_denovo	7784355	(G/A)
18523	CakSNP18523	*Kabuli*	Ca_Kabuli_denovo	7786066	(A/G)
18524	CakSNP18524	*Kabuli*	Ca_Kabuli_denovo	7786092	(T/C)
18525	CakSNP18525	*Kabuli*	Ca_Kabuli_denovo	7788204	(T/C)
18526	CakSNP18526	*Kabuli*	Ca_Kabuli_denovo	7789221	(T/C)
18527	CakSNP18527	*Kabuli*	Ca_Kabuli_denovo	7789301	(C/T)
18528	CakSNP18528	*Kabuli*	Ca_Kabuli_denovo	7793891	(G/C)
18529	CakSNP18529	*Kabuli*	Ca_Kabuli_denovo	7793893	(C/A)
18530	CakSNP18530	*Kabuli*	Ca_Kabuli_denovo	7797598	(A/G)
18531	CakSNP18531	*Kabuli*	Ca_Kabuli_denovo	7801499	(T/C)
18532	CakSNP18532	*Kabuli*	Ca_Kabuli_denovo	7803930	(G/A)
18533	CakSNP18533	*Kabuli*	Ca_Kabuli_denovo	7803960	(T/C)
18534	CakSNP18534	*Kabuli*	Ca_Kabuli_denovo	7808478	(T/C)
18535	CakSNP18535	*Kabuli*	Ca_Kabuli_denovo	7811193	(T/C)
18536	CakSNP18536	*Kabuli*	Ca_Kabuli_denovo	7811229	(T/C)
18537	CakSNP18537	*Kabuli*	Ca_Kabuli_denovo	7815667	(G/T)
18538	CakSNP18538	*Kabuli*	Ca_Kabuli_denovo	7817621	(G/C)
18539	CakSNP18539	*Kabuli*	Ca_Kabuli_denovo	7817663	(T/C)
18540	CakSNP18540	*Kabuli*	Ca_Kabuli_denovo	7819661	(A/G)
18541	CakSNP18541	*Kabuli*	Ca_Kabuli_denovo	7819688	(C/T)
18542	CakSNP18542	*Kabuli*	Ca_Kabuli_denovo	7821392	(A/T)
18543	CakSNP18543	*Kabuli*	Ca_Kabuli_denovo	7822168	(T/C)

S.N.	SNP IDs	Cultivars	Chromosomes/scaffolds	Physical positions (bp)	SNPs
18544	CakSNP18544	*Kabuli*	Ca_Kabuli_denovo	7823280	(G/A)
18545	CakSNP18545	*Kabuli*	Ca_Kabuli_denovo	7825450	(C/A)
18546	CakSNP18546	*Kabuli*	Ca_Kabuli_denovo	7832200	(T/G)
18547	CakSNP18547	*Kabuli*	Ca_Kabuli_denovo	7833031	(G/A)
18548	CakSNP18548	*Kabuli*	Ca_Kabuli_denovo	7842059	(A/G)
18549	CakSNP18549	*Kabuli*	Ca_Kabuli_denovo	7845807	(C/A)
18550	CakSNP18550	*Kabuli*	Ca_Kabuli_denovo	7845827	(C/A)
18551	CakSNP18551	*Kabuli*	Ca_Kabuli_denovo	7851012	(G/A)
18552	CakSNP18552	*Kabuli*	Ca_Kabuli_denovo	7851036	(T/C)
18553	CakSNP18553	*Kabuli*	Ca_Kabuli_denovo	7851360	(G/A)
18554	CakSNP18554	*Kabuli*	Ca_Kabuli_denovo	7851364	(C/G)
18555	CakSNP18555	*Kabuli*	Ca_Kabuli_denovo	7851413	(A/G)
18556	CakSNP18556	*Kabuli*	Ca_Kabuli_denovo	7855438	(T/C)
18557	CakSNP18557	*Kabuli*	Ca_Kabuli_denovo	7855500	(A/G)
18558	CakSNP18558	*Kabuli*	Ca_Kabuli_denovo	7858167	(T/C)
18559	CakSNP18559	*Kabuli*	Ca_Kabuli_denovo	7866944	(C/T)
18560	CakSNP18560	*Kabuli*	Ca_Kabuli_denovo	7867542	(A/G)
18561	CakSNP18561	*Kabuli*	Ca_Kabuli_denovo	7867799	(A/C)
18562	CakSNP18562	*Kabuli*	Ca_Kabuli_denovo	7876080	(A/T)
18563	CakSNP18563	*Kabuli*	Ca_Kabuli_denovo	7880716	(A/T)
18564	CakSNP18564	*Kabuli*	Ca_Kabuli_denovo	7880717	(C/T)
18565	CakSNP18565	*Kabuli*	Ca_Kabuli_denovo	7881296	(G/A)
18566	CakSNP18566	*Kabuli*	Ca_Kabuli_denovo	7881314	(G/A)
18567	CakSNP18567	*Kabuli*	Ca_Kabuli_denovo	7887564	(C/T)
18568	CakSNP18568	*Kabuli*	Ca_Kabuli_denovo	7888564	(A/G)
18569	CakSNP18569	*Kabuli*	Ca_Kabuli_denovo	7892719	(T/C)
18570	CakSNP18570	*Kabuli*	Ca_Kabuli_denovo	7894702	(C/A)
18571	CakSNP18571	*Kabuli*	Ca_Kabuli_denovo	7894708	(G/C)
18572	CakSNP18572	*Kabuli*	Ca_Kabuli_denovo	7897351	(G/A)
18573	CakSNP18573	*Kabuli*	Ca_Kabuli_denovo	7897373	(A/C)
18574	CakSNP18574	*Kabuli*	Ca_Kabuli_denovo	7900400	(T/C)
18575	CakSNP18575	*Kabuli*	Ca_Kabuli_denovo	7901584	(A/G)
18576	CakSNP18576	*Kabuli*	Ca_Kabuli_denovo	7901585	(T/C)
18577	CakSNP18577	*Kabuli*	Ca_Kabuli_denovo	7910500	(C/T)
18578	CakSNP18578	*Kabuli*	Ca_Kabuli_denovo	7910554	(G/A)
18579	CakSNP18579	*Kabuli*	Ca_Kabuli_denovo	7920197	(C/T)
18580	CakSNP18580	*Kabuli*	Ca_Kabuli_denovo	7920209	(G/A)
18581	CakSNP18581	*Kabuli*	Ca_Kabuli_denovo	7930088	(T/G)

S.N.	SNP IDs	Cultivars	Chromosomes/scaffolds	Physical positions (bp)	SNPs
18582	CakSNP18582	*Kabuli*	Ca_Kabuli_denovo	7936283	(C/T)
18583	CakSNP18583	*Kabuli*	Ca_Kabuli_denovo	7936329	(T/G)
18584	CakSNP18584	*Kabuli*	Ca_Kabuli_denovo	7941133	(C/T)
18585	CakSNP18585	*Kabuli*	Ca_Kabuli_denovo	7941147	(T/C)
18586	CakSNP18586	*Kabuli*	Ca_Kabuli_denovo	7945108	(G/A)
18587	CakSNP18587	*Kabuli*	Ca_Kabuli_denovo	7949240	(G/C)
18588	CakSNP18588	*Kabuli*	Ca_Kabuli_denovo	7949247	(G/C)
18589	CakSNP18589	*Kabuli*	Ca_Kabuli_denovo	7952539	(A/G)
18590	CakSNP18590	*Kabuli*	Ca_Kabuli_denovo	7952565	(T/C)
18591	CakSNP18591	*Kabuli*	Ca_Kabuli_denovo	7953341	(G/C)
18592	CakSNP18592	*Kabuli*	Ca_Kabuli_denovo	7955422	(T/C)
18593	CakSNP18593	*Kabuli*	Ca_Kabuli_denovo	7955973	(G/C)
18594	CakSNP18594	*Kabuli*	Ca_Kabuli_denovo	7955994	(G/T)
18595	CakSNP18595	*Kabuli*	Ca_Kabuli_denovo	7956631	(T/C)
18596	CakSNP18596	*Kabuli*	Ca_Kabuli_denovo	7960635	(C/T)
18597	CakSNP18597	*Kabuli*	Ca_Kabuli_denovo	7962856	(C/T)
18598	CakSNP18598	*Kabuli*	Ca_Kabuli_denovo	7962883	(G/C)
18599	CakSNP18599	*Kabuli*	Ca_Kabuli_denovo	7963369	(A/G)
18600	CakSNP18600	*Kabuli*	Ca_Kabuli_denovo	7963390	(G/C)
18601	CakSNP18601	*Kabuli*	Ca_Kabuli_denovo	7970950	(G/C)
18602	CakSNP18602	*Kabuli*	Ca_Kabuli_denovo	7974031	(G/C)
18603	CakSNP18603	*Kabuli*	Ca_Kabuli_denovo	7974052	(C/G)
18604	CakSNP18604	*Kabuli*	Ca_Kabuli_denovo	7982597	(A/G)
18605	CakSNP18605	*Kabuli*	Ca_Kabuli_denovo	7988467	(G/A)
18606	CakSNP18606	*Kabuli*	Ca_Kabuli_denovo	7995056	(A/G)
18607	CakSNP18607	*Kabuli*	Ca_Kabuli_denovo	7995068	(C/T)
18608	CakSNP18608	*Kabuli*	Ca_Kabuli_denovo	7997461	(T/C)
18609	CakSNP18609	*Kabuli*	Ca_Kabuli_denovo	7999842	(T/G)
18610	CakSNP18610	*Kabuli*	Ca_Kabuli_denovo	7999848	(T/A)
18611	CakSNP18611	*Kabuli*	Ca_Kabuli_denovo	8001503	(T/C)
18612	CakSNP18612	*Kabuli*	Ca_Kabuli_denovo	8003727	(A/G)
18613	CakSNP18613	*Kabuli*	Ca_Kabuli_denovo	8003736	(A/G)
18614	CakSNP18614	*Kabuli*	Ca_Kabuli_denovo	8011242	(G/C)
18615	CakSNP18615	*Kabuli*	Ca_Kabuli_denovo	8011262	(C/T)
18616	CakSNP18616	*Kabuli*	Ca_Kabuli_denovo	8013352	(C/T)
18617	CakSNP18617	*Kabuli*	Ca_Kabuli_denovo	8019793	(C/A)
18618	CakSNP18618	*Kabuli*	Ca_Kabuli_denovo	8022307	(G/A)
18619	CakSNP18619	*Kabuli*	Ca_Kabuli_denovo	8022322	(C/G)

S.N.	SNP IDs	Cultivars	Chromosomes/scaffolds	Physical positions (bp)	SNPs
18620	CakSNP18620	*Kabuli*	Ca_Kabuli_denovo	8022996	(A/G)
18621	CakSNP18621	*Kabuli*	Ca_Kabuli_denovo	8026787	(T/G)
18622	CakSNP18622	*Kabuli*	Ca_Kabuli_denovo	8026830	(G/C)
18623	CakSNP18623	*Kabuli*	Ca_Kabuli_denovo	8027198	(T/C)
18624	CakSNP18624	*Kabuli*	Ca_Kabuli_denovo	8027199	(T/C)
18625	CakSNP18625	*Kabuli*	Ca_Kabuli_denovo	8039817	(T/C)
18626	CakSNP18626	*Kabuli*	Ca_Kabuli_denovo	8041747	(T/C)
18627	CakSNP18627	*Kabuli*	Ca_Kabuli_denovo	8041759	(C/A)
18628	CakSNP18628	*Kabuli*	Ca_Kabuli_denovo	8043209	(A/G)
18629	CakSNP18629	*Kabuli*	Ca_Kabuli_denovo	8043253	(T/C)
18630	CakSNP18630	*Kabuli*	Ca_Kabuli_denovo	8044069	(A/G)
18631	CakSNP18631	*Kabuli*	Ca_Kabuli_denovo	8044129	(G/A)
18632	CakSNP18632	*Kabuli*	Ca_Kabuli_denovo	8045895	(A/G)
18633	CakSNP18633	*Kabuli*	Ca_Kabuli_denovo	8051714	(T/C)
18634	CakSNP18634	*Kabuli*	Ca_Kabuli_denovo	8057562	(G/A)
18635	CakSNP18635	*Kabuli*	Ca_Kabuli_denovo	8063940	(A/T)
18636	CakSNP18636	*Kabuli*	Ca_Kabuli_denovo	8069087	(C/A)
18637	CakSNP18637	*Kabuli*	Ca_Kabuli_denovo	8069369	(G/C)
18638	CakSNP18638	*Kabuli*	Ca_Kabuli_denovo	8070193	(G/C)
18639	CakSNP18639	*Kabuli*	Ca_Kabuli_denovo	8070240	(T/C)
18640	CakSNP18640	*Kabuli*	Ca_Kabuli_denovo	8072550	(C/T)
18641	CakSNP18641	*Kabuli*	Ca_Kabuli_denovo	8082932	(G/A)
18642	CakSNP18642	*Kabuli*	Ca_Kabuli_denovo	8083232	(A/G)
18643	CakSNP18643	*Kabuli*	Ca_Kabuli_denovo	8087512	(A/G)
18644	CakSNP18644	*Kabuli*	Ca_Kabuli_denovo	8087573	(A/G)
18645	CakSNP18645	*Kabuli*	Ca_Kabuli_denovo	8088037	(C/G)
18646	CakSNP18646	*Kabuli*	Ca_Kabuli_denovo	8088041	(T/C)
18647	CakSNP18647	*Kabuli*	Ca_Kabuli_denovo	8088319	(C/T)
18648	CakSNP18648	*Kabuli*	Ca_Kabuli_denovo	8088322	(G/C)
18649	CakSNP18649	*Kabuli*	Ca_Kabuli_denovo	8094162	(A/C)
18650	CakSNP18650	*Kabuli*	Ca_Kabuli_denovo	8098520	(A/G)
18651	CakSNP18651	*Kabuli*	Ca_Kabuli_denovo	8105899	(T/G)
18652	CakSNP18652	*Kabuli*	Ca_Kabuli_denovo	8105968	(T/C)
18653	CakSNP18653	*Kabuli*	Ca_Kabuli_denovo	8109763	(A/C)
18654	CakSNP18654	*Kabuli*	Ca_Kabuli_denovo	8112263	(A/G)
18655	CakSNP18655	*Kabuli*	Ca_Kabuli_denovo	8112296	(A/C)
18656	CakSNP18656	*Kabuli*	Ca_Kabuli_denovo	8112297	(G/C)
18657	CakSNP18657	*Kabuli*	Ca_Kabuli_denovo	8115320	(A/G)

S.N.	SNP IDs	Cultivars	Chromosomes/scaffolds	Physical positions (bp)	SNPs
18658	CakSNP18658	*Kabuli*	Ca_Kabuli_denovo	8122096	(T/C)
18659	CakSNP18659	*Kabuli*	Ca_Kabuli_denovo	8130858	(G/A)
18660	CakSNP18660	*Kabuli*	Ca_Kabuli_denovo	8130859	(C/G)
18661	CakSNP18661	*Kabuli*	Ca_Kabuli_denovo	8137324	(C/T)
18662	CakSNP18662	*Kabuli*	Ca_Kabuli_denovo	8137330	(T/G)
18663	CakSNP18663	*Kabuli*	Ca_Kabuli_denovo	8141933	(A/G)
18664	CakSNP18664	*Kabuli*	Ca_Kabuli_denovo	8144021	(A/C)
18665	CakSNP18665	*Kabuli*	Ca_Kabuli_denovo	8144078	(G/A)
18666	CakSNP18666	*Kabuli*	Ca_Kabuli_denovo	8148859	(G/A)
18667	CakSNP18667	*Kabuli*	Ca_Kabuli_denovo	8148868	(C/A)
18668	CakSNP18668	*Kabuli*	Ca_Kabuli_denovo	8149952	(C/G)
18669	CakSNP18669	*Kabuli*	Ca_Kabuli_denovo	8149954	(A/G)
18670	CakSNP18670	*Kabuli*	Ca_Kabuli_denovo	8154225	(C/A)
18671	CakSNP18671	*Kabuli*	Ca_Kabuli_denovo	8158725	(C/T)
18672	CakSNP18672	*Kabuli*	Ca_Kabuli_denovo	8158779	(T/C)
18673	CakSNP18673	*Kabuli*	Ca_Kabuli_denovo	8159110	(A/G)
18674	CakSNP18674	*Kabuli*	Ca_Kabuli_denovo	8159113	(A/G)
18675	CakSNP18675	*Kabuli*	Ca_Kabuli_denovo	8168485	(A/G)
18676	CakSNP18676	*Kabuli*	Ca_Kabuli_denovo	8169341	(C/G)
18677	CakSNP18677	*Kabuli*	Ca_Kabuli_denovo	8171387	(G/C)
18678	CakSNP18678	*Kabuli*	Ca_Kabuli_denovo	8178754	(T/C)
18679	CakSNP18679	*Kabuli*	Ca_Kabuli_denovo	8184848	(C/A)
18680	CakSNP18680	*Kabuli*	Ca_Kabuli_denovo	8189540	(A/C)
18681	CakSNP18681	*Kabuli*	Ca_Kabuli_denovo	8196300	(T/C)
18682	CakSNP18682	*Kabuli*	Ca_Kabuli_denovo	8196341	(C/G)
18683	CakSNP18683	*Kabuli*	Ca_Kabuli_denovo	8203367	(G/A)
18684	CakSNP18684	*Kabuli*	Ca_Kabuli_denovo	8207563	(C/T)
18685	CakSNP18685	*Kabuli*	Ca_Kabuli_denovo	8210958	(C/G)
18686	CakSNP18686	*Kabuli*	Ca_Kabuli_denovo	8211907	(A/G)
18687	CakSNP18687	*Kabuli*	Ca_Kabuli_denovo	8214133	(A/G)
18688	CakSNP18688	*Kabuli*	Ca_Kabuli_denovo	8214554	(T/C)
18689	CakSNP18689	*Kabuli*	Ca_Kabuli_denovo	8214590	(A/G)
18690	CakSNP18690	*Kabuli*	Ca_Kabuli_denovo	8217362	(A/G)
18691	CakSNP18691	*Kabuli*	Ca_Kabuli_denovo	8218000	(C/G)
18692	CakSNP18692	*Kabuli*	Ca_Kabuli_denovo	8218005	(A/C)
18693	CakSNP18693	*Kabuli*	Ca_Kabuli_denovo	8219090	(G/T)
18694	CakSNP18694	*Kabuli*	Ca_Kabuli_denovo	8223822	(C/G)
18695	CakSNP18695	*Kabuli*	Ca_Kabuli_denovo	8226812	(C/T)

S.N.	SNP IDs	Cultivars	Chromosomes/scaffolds	Physical positions (bp)	SNPs
18696	CakSNP18696	*Kabuli*	Ca_Kabuli_denovo	8230958	(C/G)
18697	CakSNP18697	*Kabuli*	Ca_Kabuli_denovo	8230966	(A/T)
18698	CakSNP18698	*Kabuli*	Ca_Kabuli_denovo	8232844	(G/A)
18699	CakSNP18699	*Kabuli*	Ca_Kabuli_denovo	8232880	(G/A)
18700	CakSNP18700	*Kabuli*	Ca_Kabuli_denovo	8234144	(A/G)
18701	CakSNP18701	*Kabuli*	Ca_Kabuli_denovo	8234153	(C/G)
18702	CakSNP18702	*Kabuli*	Ca_Kabuli_denovo	8241946	(C/T)
18703	CakSNP18703	*Kabuli*	Ca_Kabuli_denovo	8241952	(C/T)
18704	CakSNP18704	*Kabuli*	Ca_Kabuli_denovo	8242371	(G/A)
18705	CakSNP18705	*Kabuli*	Ca_Kabuli_denovo	8243836	(T/C)
18706	CakSNP18706	*Kabuli*	Ca_Kabuli_denovo	8244667	(C/T)
18707	CakSNP18707	*Kabuli*	Ca_Kabuli_denovo	8249352	(C/A)
18708	CakSNP18708	*Kabuli*	Ca_Kabuli_denovo	8256558	(C/T)
18709	CakSNP18709	*Kabuli*	Ca_Kabuli_denovo	8256561	(C/T)
18710	CakSNP18710	*Kabuli*	Ca_Kabuli_denovo	8257059	(C/T)
18711	CakSNP18711	*Kabuli*	Ca_Kabuli_denovo	8257088	(A/G)
18712	CakSNP18712	*Kabuli*	Ca_Kabuli_denovo	8261284	(C/G)
18713	CakSNP18713	*Kabuli*	Ca_Kabuli_denovo	8266889	(C/T)
18714	CakSNP18714	*Kabuli*	Ca_Kabuli_denovo	8269821	(A/G)
18715	CakSNP18715	*Kabuli*	Ca_Kabuli_denovo	8271190	(C/G)
18716	CakSNP18716	*Kabuli*	Ca_Kabuli_denovo	8271195	(G/C)
18717	CakSNP18717	*Kabuli*	Ca_Kabuli_denovo	8283720	(C/T)
18718	CakSNP18718	*Kabuli*	Ca_Kabuli_denovo	8283723	(T/C)
18719	CakSNP18719	*Kabuli*	Ca_Kabuli_denovo	8287800	(G/T)
18720	CakSNP18720	*Kabuli*	Ca_Kabuli_denovo	8289104	(C/T)
18721	CakSNP18721	*Kabuli*	Ca_Kabuli_denovo	8293206	(G/T)
18722	CakSNP18722	*Kabuli*	Ca_Kabuli_denovo	8297749	(A/C)
18723	CakSNP18723	*Kabuli*	Ca_Kabuli_denovo	8300470	(T/G)
18724	CakSNP18724	*Kabuli*	Ca_Kabuli_denovo	8300931	(A/G)
18725	CakSNP18725	*Kabuli*	Ca_Kabuli_denovo	8300939	(T/A)
18726	CakSNP18726	*Kabuli*	Ca_Kabuli_denovo	8303497	(T/C)
18727	CakSNP18727	*Kabuli*	Ca_Kabuli_denovo	8303505	(C/G)
18728	CakSNP18728	*Kabuli*	Ca_Kabuli_denovo	8305655	(A/G)
18729	CakSNP18729	*Kabuli*	Ca_Kabuli_denovo	8305661	(G/A)
18730	CakSNP18730	*Kabuli*	Ca_Kabuli_denovo	8306153	(G/A)
18731	CakSNP18731	*Kabuli*	Ca_Kabuli_denovo	8317400	(G/C)
18732	CakSNP18732	*Kabuli*	Ca_Kabuli_denovo	8317564	(G/C)
18733	CakSNP18733	*Kabuli*	Ca_Kabuli_denovo	8317582	(C/T)

S.N.	SNP IDs	Cultivars	Chromosomes/scaffolds	Physical positions (bp)	SNPs
18734	CakSNP18734	*Kabuli*	Ca_Kabuli_denovo	8321061	(C/T)
18735	CakSNP18735	*Kabuli*	Ca_Kabuli_denovo	8322931	(G/A)
18736	CakSNP18736	*Kabuli*	Ca_Kabuli_denovo	8325194	(C/T)
18737	CakSNP18737	*Kabuli*	Ca_Kabuli_denovo	8325213	(C/A)
18738	CakSNP18738	*Kabuli*	Ca_Kabuli_denovo	8327042	(C/T)
18739	CakSNP18739	*Kabuli*	Ca_Kabuli_denovo	8327887	(A/G)
18740	CakSNP18740	*Kabuli*	Ca_Kabuli_denovo	8327890	(A/G)
18741	CakSNP18741	*Kabuli*	Ca_Kabuli_denovo	8328850	(G/A)
18742	CakSNP18742	*Kabuli*	Ca_Kabuli_denovo	8329853	(G/T)
18743	CakSNP18743	*Kabuli*	Ca_Kabuli_denovo	8329859	(G/A)
18744	CakSNP18744	*Kabuli*	Ca_Kabuli_denovo	8331379	(C/T)
18745	CakSNP18745	*Kabuli*	Ca_Kabuli_denovo	8335396	(T/C)
18746	CakSNP18746	*Kabuli*	Ca_Kabuli_denovo	8335402	(T/G)
18747	CakSNP18747	*Kabuli*	Ca_Kabuli_denovo	8336646	(G/C)
18748	CakSNP18748	*Kabuli*	Ca_Kabuli_denovo	8336655	(T/C)
18749	CakSNP18749	*Kabuli*	Ca_Kabuli_denovo	8336819	(G/A)
18750	CakSNP18750	*Kabuli*	Ca_Kabuli_denovo	8336864	(A/G)
18751	CakSNP18751	*Kabuli*	Ca_Kabuli_denovo	8337449	(T/G)
18752	CakSNP18752	*Kabuli*	Ca_Kabuli_denovo	8343165	(A/G)
18753	CakSNP18753	*Kabuli*	Ca_Kabuli_denovo	8349391	(G/C)
18754	CakSNP18754	*Kabuli*	Ca_Kabuli_denovo	8349400	(C/T)
18755	CakSNP18755	*Kabuli*	Ca_Kabuli_denovo	8350117	(T/G)
18756	CakSNP18756	*Kabuli*	Ca_Kabuli_denovo	8350148	(C/G)
18757	CakSNP18757	*Kabuli*	Ca_Kabuli_denovo	8353015	(G/T)
18758	CakSNP18758	*Kabuli*	Ca_Kabuli_denovo	8354669	(A/T)
18759	CakSNP18759	*Kabuli*	Ca_Kabuli_denovo	8354690	(T/C)
18760	CakSNP18760	*Kabuli*	Ca_Kabuli_denovo	8355827	(T/G)
18761	CakSNP18761	*Kabuli*	Ca_Kabuli_denovo	8360991	(C/A)
18762	CakSNP18762	*Kabuli*	Ca_Kabuli_denovo	8361061	(G/A)
18763	CakSNP18763	*Kabuli*	Ca_Kabuli_denovo	8365868	(T/C)
18764	CakSNP18764	*Kabuli*	Ca_Kabuli_denovo	8365899	(G/A)
18765	CakSNP18765	*Kabuli*	Ca_Kabuli_denovo	8379869	(A/C)
18766	CakSNP18766	*Kabuli*	Ca_Kabuli_denovo	8380718	(C/G)
18767	CakSNP18767	*Kabuli*	Ca_Kabuli_denovo	8380722	(G/A)
18768	CakSNP18768	*Kabuli*	Ca_Kabuli_denovo	8391995	(A/G)
18769	CakSNP18769	*Kabuli*	Ca_Kabuli_denovo	8392015	(A/C)
18770	CakSNP18770	*Kabuli*	Ca_Kabuli_denovo	8400805	(C/T)
18771	CakSNP18771	*Kabuli*	Ca_Kabuli_denovo	8405558	(A/G)

S.N.	SNP IDs	Cultivars	Chromosomes/scaffolds	Physical positions (bp)	SNPs
18772	CakSNP18772	*Kabuli*	Ca_Kabuli_denovo	8405615	(T/C)
18773	CakSNP18773	*Kabuli*	Ca_Kabuli_denovo	8409196	(T/C)
18774	CakSNP18774	*Kabuli*	Ca_Kabuli_denovo	8409374	(T/C)
18775	CakSNP18775	*Kabuli*	Ca_Kabuli_denovo	8411035	(T/G)
18776	CakSNP18776	*Kabuli*	Ca_Kabuli_denovo	8411059	(T/G)
18777	CakSNP18777	*Kabuli*	Ca_Kabuli_denovo	8413267	(G/C)
18778	CakSNP18778	*Kabuli*	Ca_Kabuli_denovo	8413291	(A/C)
18779	CakSNP18779	*Kabuli*	Ca_Kabuli_denovo	8416177	(T/C)
18780	CakSNP18780	*Kabuli*	Ca_Kabuli_denovo	8416180	(T/C)
18781	CakSNP18781	*Kabuli*	Ca_Kabuli_denovo	8417405	(T/G)
18782	CakSNP18782	*Kabuli*	Ca_Kabuli_denovo	8417948	(G/T)
18783	CakSNP18783	*Kabuli*	Ca_Kabuli_denovo	8418116	(G/C)
18784	CakSNP18784	*Kabuli*	Ca_Kabuli_denovo	8418159	(T/C)
18785	CakSNP18785	*Kabuli*	Ca_Kabuli_denovo	8419456	(C/G)
18786	CakSNP18786	*Kabuli*	Ca_Kabuli_denovo	8420072	(A/C)
18787	CakSNP18787	*Kabuli*	Ca_Kabuli_denovo	8422556	(T/C)
18788	CakSNP18788	*Kabuli*	Ca_Kabuli_denovo	8423156	(T/C)
18789	CakSNP18789	*Kabuli*	Ca_Kabuli_denovo	8429766	(A/C)
18790	CakSNP18790	*Kabuli*	Ca_Kabuli_denovo	8436507	(C/T)
18791	CakSNP18791	*Kabuli*	Ca_Kabuli_denovo	8436561	(G/A)
18792	CakSNP18792	*Kabuli*	Ca_Kabuli_denovo	8438196	(T/G)
18793	CakSNP18793	*Kabuli*	Ca_Kabuli_denovo	8438207	(G/C)
18794	CakSNP18794	*Kabuli*	Ca_Kabuli_denovo	8448425	(G/C)
18795	CakSNP18795	*Kabuli*	Ca_Kabuli_denovo	8448815	(C/A)
18796	CakSNP18796	*Kabuli*	Ca_Kabuli_denovo	8452890	(T/G)
18797	CakSNP18797	*Kabuli*	Ca_Kabuli_denovo	8454266	(T/C)
18798	CakSNP18798	*Kabuli*	Ca_Kabuli_denovo	8460560	(C/A)
18799	CakSNP18799	*Kabuli*	Ca_Kabuli_denovo	8461914	(C/G)
18800	CakSNP18800	*Kabuli*	Ca_Kabuli_denovo	8466689	(A/G)
18801	CakSNP18801	*Kabuli*	Ca_Kabuli_denovo	8469963	(A/G)
18802	CakSNP18802	*Kabuli*	Ca_Kabuli_denovo	8469975	(C/A)
18803	CakSNP18803	*Kabuli*	Ca_Kabuli_denovo	8479007	(G/T)
18804	CakSNP18804	*Kabuli*	Ca_Kabuli_denovo	8479010	(C/G)
18805	CakSNP18805	*Kabuli*	Ca_Kabuli_denovo	8480432	(C/G)
18806	CakSNP18806	*Kabuli*	Ca_Kabuli_denovo	8480589	(C/T)
18807	CakSNP18807	*Kabuli*	Ca_Kabuli_denovo	8483863	(T/C)
18808	CakSNP18808	*Kabuli*	Ca_Kabuli_denovo	8486588	(G/T)
18809	CakSNP18809	*Kabuli*	Ca_Kabuli_denovo	8489504	(G/A)

S.N.	SNP IDs	Cultivars	Chromosomes/scaffolds	Physical positions (bp)	SNPs
18810	CakSNP18810	*Kabuli*	Ca_Kabuli_denovo	8492446	(C/G)
18811	CakSNP18811	*Kabuli*	Ca_Kabuli_denovo	8492462	(A/T)
18812	CakSNP18812	*Kabuli*	Ca_Kabuli_denovo	8496663	(T/G)
18813	CakSNP18813	*Kabuli*	Ca_Kabuli_denovo	8503771	(T/C)
18814	CakSNP18814	*Kabuli*	Ca_Kabuli_denovo	8503785	(T/G)
18815	CakSNP18815	*Kabuli*	Ca_Kabuli_denovo	8503798	(A/G)
18816	CakSNP18816	*Kabuli*	Ca_Kabuli_denovo	8505482	(T/C)
18817	CakSNP18817	*Kabuli*	Ca_Kabuli_denovo	8505765	(G/A)
18818	CakSNP18818	*Kabuli*	Ca_Kabuli_denovo	8509639	(A/G)
18819	CakSNP18819	*Kabuli*	Ca_Kabuli_denovo	8509669	(G/A)
18820	CakSNP18820	*Kabuli*	Ca_Kabuli_denovo	8513119	(A/C)
18821	CakSNP18821	*Kabuli*	Ca_Kabuli_denovo	8530754	(T/C)
18822	CakSNP18822	*Kabuli*	Ca_Kabuli_denovo	8530755	(T/G)
18823	CakSNP18823	*Kabuli*	Ca_Kabuli_denovo	8531674	(C/T)
18824	CakSNP18824	*Kabuli*	Ca_Kabuli_denovo	8531701	(A/T)
18825	CakSNP18825	*Kabuli*	Ca_Kabuli_denovo	8531753	(G/C)
18826	CakSNP18826	*Kabuli*	Ca_Kabuli_denovo	8531795	(G/A)
18827	CakSNP18827	*Kabuli*	Ca_Kabuli_denovo	8533432	(C/T)
18828	CakSNP18828	*Kabuli*	Ca_Kabuli_denovo	8533441	(A/C)
18829	CakSNP18829	*Kabuli*	Ca_Kabuli_denovo	8533970	(A/C)
18830	CakSNP18830	*Kabuli*	Ca_Kabuli_denovo	8533990	(T/C)
18831	CakSNP18831	*Kabuli*	Ca_Kabuli_denovo	8534449	(G/A)
18832	CakSNP18832	*Kabuli*	Ca_Kabuli_denovo	8550060	(A/G)
18833	CakSNP18833	*Kabuli*	Ca_Kabuli_denovo	8554253	(A/T)
18834	CakSNP18834	*Kabuli*	Ca_Kabuli_denovo	8554325	(G/A)
18835	CakSNP18835	*Kabuli*	Ca_Kabuli_denovo	8556197	(G/T)
18836	CakSNP18836	*Kabuli*	Ca_Kabuli_denovo	8556200	(A/G)
18837	CakSNP18837	*Kabuli*	Ca_Kabuli_denovo	8556235	(C/T)
18838	CakSNP18838	*Kabuli*	Ca_Kabuli_denovo	8560474	(C/G)
18839	CakSNP18839	*Kabuli*	Ca_Kabuli_denovo	8560544	(A/C)
18840	CakSNP18840	*Kabuli*	Ca_Kabuli_denovo	8562341	(G/C)
18841	CakSNP18841	*Kabuli*	Ca_Kabuli_denovo	8566053	(G/T)
18842	CakSNP18842	*Kabuli*	Ca_Kabuli_denovo	8566099	(C/T)
18843	CakSNP18843	*Kabuli*	Ca_Kabuli_denovo	8571259	(A/G)
18844	CakSNP18844	*Kabuli*	Ca_Kabuli_denovo	8573599	(A/T)
18845	CakSNP18845	*Kabuli*	Ca_Kabuli_denovo	8578805	(A/C)
18846	CakSNP18846	*Kabuli*	Ca_Kabuli_denovo	8580661	(G/C)
18847	CakSNP18847	*Kabuli*	Ca_Kabuli_denovo	8580697	(G/T)

S.N.	SNP IDs	Cultivars	Chromosomes/scaffolds	Physical positions (bp)	SNPs
18848	CakSNP18848	*Kabuli*	Ca_Kabuli_denovo	8581281	(G/A)
18849	CakSNP18849	*Kabuli*	Ca_Kabuli_denovo	8584477	(A/G)
18850	CakSNP18850	*Kabuli*	Ca_Kabuli_denovo	8584479	(C/A)
18851	CakSNP18851	*Kabuli*	Ca_Kabuli_denovo	8584858	(T/C)
18852	CakSNP18852	*Kabuli*	Ca_Kabuli_denovo	8584877	(G/A)
18853	CakSNP18853	*Kabuli*	Ca_Kabuli_denovo	8593352	(G/C)
18854	CakSNP18854	*Kabuli*	Ca_Kabuli_denovo	8593359	(A/C)
18855	CakSNP18855	*Kabuli*	Ca_Kabuli_denovo	8603638	(C/T)
18856	CakSNP18856	*Kabuli*	Ca_Kabuli_denovo	8603707	(C/A)
18857	CakSNP18857	*Kabuli*	Ca_Kabuli_denovo	8607036	(T/C)
18858	CakSNP18858	*Kabuli*	Ca_Kabuli_denovo	8623968	(C/A)
18859	CakSNP18859	*Kabuli*	Ca_Kabuli_denovo	8626120	(T/C)
18860	CakSNP18860	*Kabuli*	Ca_Kabuli_denovo	8626123	(C/T)
18861	CakSNP18861	*Kabuli*	Ca_Kabuli_denovo	8629836	(A/C)
18862	CakSNP18862	*Kabuli*	Ca_Kabuli_denovo	8629837	(G/C)
18863	CakSNP18863	*Kabuli*	Ca_Kabuli_denovo	8629839	(T/C)
18864	CakSNP18864	*Kabuli*	Ca_Kabuli_denovo	8630141	(T/G)
18865	CakSNP18865	*Kabuli*	Ca_Kabuli_denovo	8630199	(G/C)
18866	CakSNP18866	*Kabuli*	Ca_Kabuli_denovo	8633226	(G/A)
18867	CakSNP18867	*Kabuli*	Ca_Kabuli_denovo	8633271	(G/T)
18868	CakSNP18868	*Kabuli*	Ca_Kabuli_denovo	8634180	(T/C)
18869	CakSNP18869	*Kabuli*	Ca_Kabuli_denovo	8634188	(A/G)
18870	CakSNP18870	*Kabuli*	Ca_Kabuli_denovo	8642988	(A/C)
18871	CakSNP18871	*Kabuli*	Ca_Kabuli_denovo	8652511	(T/A)
18872	CakSNP18872	*Kabuli*	Ca_Kabuli_denovo	8656524	(G/C)
18873	CakSNP18873	*Kabuli*	Ca_Kabuli_denovo	8657680	(G/T)
18874	CakSNP18874	*Kabuli*	Ca_Kabuli_denovo	8661307	(A/G)
18875	CakSNP18875	*Kabuli*	Ca_Kabuli_denovo	8661312	(G/A)
18876	CakSNP18876	*Kabuli*	Ca_Kabuli_denovo	8665424	(G/A)
18877	CakSNP18877	*Kabuli*	Ca_Kabuli_denovo	8677202	(C/T)
18878	CakSNP18878	*Kabuli*	Ca_Kabuli_denovo	8677253	(T/C)
18879	CakSNP18879	*Kabuli*	Ca_Kabuli_denovo	8683447	(T/C)
18880	CakSNP18880	*Kabuli*	Ca_Kabuli_denovo	8683473	(T/C)
18881	CakSNP18881	*Kabuli*	Ca_Kabuli_denovo	8688554	(G/A)
18882	CakSNP18882	*Kabuli*	Ca_Kabuli_denovo	8692558	(A/G)
18883	CakSNP18883	*Kabuli*	Ca_Kabuli_denovo	8697009	(A/G)
18884	CakSNP18884	*Kabuli*	Ca_Kabuli_denovo	8701182	(C/T)
18885	CakSNP18885	*Kabuli*	Ca_Kabuli_denovo	8706399	(C/T)

S.N.	SNP IDs	Cultivars	Chromosomes/scaffolds	Physical positions (bp)	SNPs
18886	CakSNP18886	*Kabuli*	Ca_Kabuli_denovo	8712116	(C/T)
18887	CakSNP18887	*Kabuli*	Ca_Kabuli_denovo	8712137	(C/G)
18888	CakSNP18888	*Kabuli*	Ca_Kabuli_denovo	8713270	(C/A)
18889	CakSNP18889	*Kabuli*	Ca_Kabuli_denovo	8713287	(T/G)
18890	CakSNP18890	*Kabuli*	Ca_Kabuli_denovo	8714821	(G/A)
18891	CakSNP18891	*Kabuli*	Ca_Kabuli_denovo	8717740	(T/C)
18892	CakSNP18892	*Kabuli*	Ca_Kabuli_denovo	8717768	(G/C)
18893	CakSNP18893	*Kabuli*	Ca_Kabuli_denovo	8718318	(T/G)
18894	CakSNP18894	*Kabuli*	Ca_Kabuli_denovo	8723450	(C/T)
18895	CakSNP18895	*Kabuli*	Ca_Kabuli_denovo	8728039	(C/T)
18896	CakSNP18896	*Kabuli*	Ca_Kabuli_denovo	8728049	(T/G)
18897	CakSNP18897	*Kabuli*	Ca_Kabuli_denovo	8733667	(G/T)
18898	CakSNP18898	*Kabuli*	Ca_Kabuli_denovo	8737564	(A/G)
18899	CakSNP18899	*Kabuli*	Ca_Kabuli_denovo	8740252	(C/T)
18900	CakSNP18900	*Kabuli*	Ca_Kabuli_denovo	8741945	(G/A)
18901	CakSNP18901	*Kabuli*	Ca_Kabuli_denovo	8743682	(C/A)
18902	CakSNP18902	*Kabuli*	Ca_Kabuli_denovo	8745896	(T/G)
18903	CakSNP18903	*Kabuli*	Ca_Kabuli_denovo	8746884	(A/G)
18904	CakSNP18904	*Kabuli*	Ca_Kabuli_denovo	8749660	(G/A)
18905	CakSNP18905	*Kabuli*	Ca_Kabuli_denovo	8749690	(C/G)
18906	CakSNP18906	*Kabuli*	Ca_Kabuli_denovo	8751619	(C/A)
18907	CakSNP18907	*Kabuli*	Ca_Kabuli_denovo	8751625	(C/T)
18908	CakSNP18908	*Kabuli*	Ca_Kabuli_denovo	8754866	(C/G)
18909	CakSNP18909	*Kabuli*	Ca_Kabuli_denovo	8756405	(T/A)
18910	CakSNP18910	*Kabuli*	Ca_Kabuli_denovo	8762906	(T/C)
18911	CakSNP18911	*Kabuli*	Ca_Kabuli_denovo	8764006	(C/G)
18912	CakSNP18912	*Kabuli*	Ca_Kabuli_denovo	8773383	(C/A)
18913	CakSNP18913	*Kabuli*	Ca_Kabuli_denovo	8783399	(C/T)
18914	CakSNP18914	*Kabuli*	Ca_Kabuli_denovo	8785543	(T/A)
18915	CakSNP18915	*Kabuli*	Ca_Kabuli_denovo	8785573	(G/A)
18916	CakSNP18916	*Kabuli*	Ca_Kabuli_denovo	8785912	(A/G)
18917	CakSNP18917	*Kabuli*	Ca_Kabuli_denovo	8785983	(A/C)
18918	CakSNP18918	*Kabuli*	Ca_Kabuli_denovo	8786375	(C/T)
18919	CakSNP18919	*Kabuli*	Ca_Kabuli_denovo	8786405	(T/C)
18920	CakSNP18920	*Kabuli*	Ca_Kabuli_denovo	8790559	(G/A)
18921	CakSNP18921	*Kabuli*	Ca_Kabuli_denovo	8793365	(T/C)
18922	CakSNP18922	*Kabuli*	Ca_Kabuli_denovo	8797171	(C/A)
18923	CakSNP18923	*Kabuli*	Ca_Kabuli_denovo	8797219	(T/C)

S.N.	SNP IDs	Cultivars	Chromosomes/scaffolds	Physical positions (bp)	SNPs
18924	CakSNP18924	*Kabuli*	Ca_Kabuli_denovo	8802899	(C/T)
18925	CakSNP18925	*Kabuli*	Ca_Kabuli_denovo	8802968	(C/A)
18926	CakSNP18926	*Kabuli*	Ca_Kabuli_denovo	8802970	(C/G)
18927	CakSNP18927	*Kabuli*	Ca_Kabuli_denovo	8817440	(T/C)
18928	CakSNP18928	*Kabuli*	Ca_Kabuli_denovo	8817510	(C/A)
18929	CakSNP18929	*Kabuli*	Ca_Kabuli_denovo	8821101	(C/T)
18930	CakSNP18930	*Kabuli*	Ca_Kabuli_denovo	8835782	(A/C)
18931	CakSNP18931	*Kabuli*	Ca_Kabuli_denovo	8837267	(C/G)
18932	CakSNP18932	*Kabuli*	Ca_Kabuli_denovo	8837276	(C/G)
18933	CakSNP18933	*Kabuli*	Ca_Kabuli_denovo	8839300	(G/C)
18934	CakSNP18934	*Kabuli*	Ca_Kabuli_denovo	8839943	(C/T)
18935	CakSNP18935	*Kabuli*	Ca_Kabuli_denovo	8839950	(G/A)
18936	CakSNP18936	*Kabuli*	Ca_Kabuli_denovo	8841891	(C/G)
18937	CakSNP18937	*Kabuli*	Ca_Kabuli_denovo	8843216	(T/C)
18938	CakSNP18938	*Kabuli*	Ca_Kabuli_denovo	8846463	(T/C)
18939	CakSNP18939	*Kabuli*	Ca_Kabuli_denovo	8847644	(T/G)
18940	CakSNP18940	*Kabuli*	Ca_Kabuli_denovo	8847757	(A/T)
18941	CakSNP18941	*Kabuli*	Ca_Kabuli_denovo	8847796	(A/G)
18942	CakSNP18942	*Kabuli*	Ca_Kabuli_denovo	8852097	(T/G)
18943	CakSNP18943	*Kabuli*	Ca_Kabuli_denovo	8855774	(G/C)
18944	CakSNP18944	*Kabuli*	Ca_Kabuli_denovo	8856170	(A/C)
18945	CakSNP18945	*Kabuli*	Ca_Kabuli_denovo	8857655	(A/T)
18946	CakSNP18946	*Kabuli*	Ca_Kabuli_denovo	8858498	(A/G)
18947	CakSNP18947	*Kabuli*	Ca_Kabuli_denovo	8864720	(T/G)
18948	CakSNP18948	*Kabuli*	Ca_Kabuli_denovo	8869813	(G/A)
18949	CakSNP18949	*Kabuli*	Ca_Kabuli_denovo	8881500	(A/C)
18950	CakSNP18950	*Kabuli*	Ca_Kabuli_denovo	8881501	(G/A)
18951	CakSNP18951	*Kabuli*	Ca_Kabuli_denovo	8882179	(C/T)
18952	CakSNP18952	*Kabuli*	Ca_Kabuli_denovo	8882231	(G/A)
18953	CakSNP18953	*Kabuli*	Ca_Kabuli_denovo	8885140	(A/C)
18954	CakSNP18954	*Kabuli*	Ca_Kabuli_denovo	8885204	(C/T)
18955	CakSNP18955	*Kabuli*	Ca_Kabuli_denovo	8887438	(T/G)
18956	CakSNP18956	*Kabuli*	Ca_Kabuli_denovo	8891101	(C/T)
18957	CakSNP18957	*Kabuli*	Ca_Kabuli_denovo	8898477	(T/C)
18958	CakSNP18958	*Kabuli*	Ca_Kabuli_denovo	8903642	(A/G)
18959	CakSNP18959	*Kabuli*	Ca_Kabuli_denovo	8903702	(G/A)
18960	CakSNP18960	*Kabuli*	Ca_Kabuli_denovo	8904259	(T/G)
18961	CakSNP18961	*Kabuli*	Ca_Kabuli_denovo	8910990	(A/G)

S.N.	SNP IDs	Cultivars	Chromosomes/scaffolds	Physical positions (bp)	SNPs
18962	CakSNP18962	*Kabuli*	Ca_Kabuli_denovo	8911135	(G/A)
18963	CakSNP18963	*Kabuli*	Ca_Kabuli_denovo	8911140	(G/A)
18964	CakSNP18964	*Kabuli*	Ca_Kabuli_denovo	8916332	(C/A)
18965	CakSNP18965	*Kabuli*	Ca_Kabuli_denovo	8918015	(A/G)
18966	CakSNP18966	*Kabuli*	Ca_Kabuli_denovo	8918028	(C/T)
18967	CakSNP18967	*Kabuli*	Ca_Kabuli_denovo	8918993	(C/T)
18968	CakSNP18968	*Kabuli*	Ca_Kabuli_denovo	8922620	(C/G)
18969	CakSNP18969	*Kabuli*	Ca_Kabuli_denovo	8922642	(T/G)
18970	CakSNP18970	*Kabuli*	Ca_Kabuli_denovo	8923604	(A/G)
18971	CakSNP18971	*Kabuli*	Ca_Kabuli_denovo	8935945	(G/T)
18972	CakSNP18972	*Kabuli*	Ca_Kabuli_denovo	8941714	(G/A)
18973	CakSNP18973	*Kabuli*	Ca_Kabuli_denovo	8941759	(G/A)
18974	CakSNP18974	*Kabuli*	Ca_Kabuli_denovo	8946705	(C/T)
18975	CakSNP18975	*Kabuli*	Ca_Kabuli_denovo	8946759	(C/T)
18976	CakSNP18976	*Kabuli*	Ca_Kabuli_denovo	8955101	(C/T)
18977	CakSNP18977	*Kabuli*	Ca_Kabuli_denovo	8956392	(T/C)
18978	CakSNP18978	*Kabuli*	Ca_Kabuli_denovo	8956416	(A/G)
18979	CakSNP18979	*Kabuli*	Ca_Kabuli_denovo	8958868	(C/A)
18980	CakSNP18980	*Kabuli*	Ca_Kabuli_denovo	8962549	(T/C)
18981	CakSNP18981	*Kabuli*	Ca_Kabuli_denovo	8969882	(C/G)
18982	CakSNP18982	*Kabuli*	Ca_Kabuli_denovo	8969927	(G/A)
18983	CakSNP18983	*Kabuli*	Ca_Kabuli_denovo	8972239	(C/G)
18984	CakSNP18984	*Kabuli*	Ca_Kabuli_denovo	8979187	(A/G)
18985	CakSNP18985	*Kabuli*	Ca_Kabuli_denovo	8991933	(T/C)
18986	CakSNP18986	*Kabuli*	Ca_Kabuli_denovo	8998274	(C/T)
18987	CakSNP18987	*Kabuli*	Ca_Kabuli_denovo	8998283	(G/C)
18988	CakSNP18988	*Kabuli*	Ca_Kabuli_denovo	8998729	(C/G)
18989	CakSNP18989	*Kabuli*	Ca_Kabuli_denovo	8998743	(G/T)
18990	CakSNP18990	*Kabuli*	Ca_Kabuli_denovo	8999684	(C/T)
18991	CakSNP18991	*Kabuli*	Ca_Kabuli_denovo	8999695	(T/C)
18992	CakSNP18992	*Kabuli*	Ca_Kabuli_denovo	9007535	(C/A)
18993	CakSNP18993	*Kabuli*	Ca_Kabuli_denovo	9007546	(A/C)
18994	CakSNP18994	*Kabuli*	Ca_Kabuli_denovo	9008111	(G/A)
18995	CakSNP18995	*Kabuli*	Ca_Kabuli_denovo	9008153	(A/G)
18996	CakSNP18996	*Kabuli*	Ca_Kabuli_denovo	9009853	(T/G)
18997	CakSNP18997	*Kabuli*	Ca_Kabuli_denovo	9010359	(T/C)
18998	CakSNP18998	*Kabuli*	Ca_Kabuli_denovo	9016466	(A/C)
18999	CakSNP18999	*Kabuli*	Ca_Kabuli_denovo	9016479	(C/T)

S.N.	SNP IDs	Cultivars	Chromosomes/scaffolds	Physical positions (bp)	SNPs
19000	CakSNP19000	*Kabuli*	Ca_Kabuli_denovo	9020583	(C/G)
19001	CakSNP19001	*Kabuli*	Ca_Kabuli_denovo	9020621	(A/G)
19002	CakSNP19002	*Kabuli*	Ca_Kabuli_denovo	9020928	(A/G)
19003	CakSNP19003	*Kabuli*	Ca_Kabuli_denovo	9021373	(C/T)
19004	CakSNP19004	*Kabuli*	Ca_Kabuli_denovo	9025243	(C/A)
19005	CakSNP19005	*Kabuli*	Ca_Kabuli_denovo	9025246	(G/C)
19006	CakSNP19006	*Kabuli*	Ca_Kabuli_denovo	9026799	(A/C)
19007	CakSNP19007	*Kabuli*	Ca_Kabuli_denovo	9028405	(C/G)
19008	CakSNP19008	*Kabuli*	Ca_Kabuli_denovo	9029661	(C/T)
19009	CakSNP19009	*Kabuli*	Ca_Kabuli_denovo	9030509	(T/C)
19010	CakSNP19010	*Kabuli*	Ca_Kabuli_denovo	9034404	(A/G)
19011	CakSNP19011	*Kabuli*	Ca_Kabuli_denovo	9034429	(A/C)
19012	CakSNP19012	*Kabuli*	Ca_Kabuli_denovo	9043273	(C/G)
19013	CakSNP19013	*Kabuli*	Ca_Kabuli_denovo	9043321	(C/T)
19014	CakSNP19014	*Kabuli*	Ca_Kabuli_denovo	9050711	(C/G)
19015	CakSNP19015	*Kabuli*	Ca_Kabuli_denovo	9050747	(G/A)
19016	CakSNP19016	*Kabuli*	Ca_Kabuli_denovo	9056377	(A/G)
19017	CakSNP19017	*Kabuli*	Ca_Kabuli_denovo	9067796	(A/G)
19018	CakSNP19018	*Kabuli*	Ca_Kabuli_denovo	9067797	(A/G)
19019	CakSNP19019	*Kabuli*	Ca_Kabuli_denovo	9068479	(A/G)
19020	CakSNP19020	*Kabuli*	Ca_Kabuli_denovo	9071784	(G/A)
19021	CakSNP19021	*Kabuli*	Ca_Kabuli_denovo	9077263	(A/C)
19022	CakSNP19022	*Kabuli*	Ca_Kabuli_denovo	9077474	(T/G)
19023	CakSNP19023	*Kabuli*	Ca_Kabuli_denovo	9077488	(T/C)
19024	CakSNP19024	*Kabuli*	Ca_Kabuli_denovo	9077513	(T/G)
19025	CakSNP19025	*Kabuli*	Ca_Kabuli_denovo	9077529	(T/G)
19026	CakSNP19026	*Kabuli*	Ca_Kabuli_denovo	9077813	(C/T)
19027	CakSNP19027	*Kabuli*	Ca_Kabuli_denovo	9081380	(G/A)
19028	CakSNP19028	*Kabuli*	Ca_Kabuli_denovo	9081383	(C/T)
19029	CakSNP19029	*Kabuli*	Ca_Kabuli_denovo	9085000	(T/C)
19030	CakSNP19030	*Kabuli*	Ca_Kabuli_denovo	9085051	(T/C)
19031	CakSNP19031	*Kabuli*	Ca_Kabuli_denovo	9087707	(T/C)
19032	CakSNP19032	*Kabuli*	Ca_Kabuli_denovo	9089170	(A/C)
19033	CakSNP19033	*Kabuli*	Ca_Kabuli_denovo	9089960	(G/A)
19034	CakSNP19034	*Kabuli*	Ca_Kabuli_denovo	9093663	(G/A)
19035	CakSNP19035	*Kabuli*	Ca_Kabuli_denovo	9094477	(C/T)
19036	CakSNP19036	*Kabuli*	Ca_Kabuli_denovo	9096275	(G/A)
19037	CakSNP19037	*Kabuli*	Ca_Kabuli_denovo	9096314	(A/G)

S.N.	SNP IDs	Cultivars	Chromosomes/scaffolds	Physical positions (bp)	SNPs
19038	CakSNP19038	*Kabuli*	Ca_Kabuli_denovo	9099899	(G/C)
19039	CakSNP19039	*Kabuli*	Ca_Kabuli_denovo	9099908	(C/G)
19040	CakSNP19040	*Kabuli*	Ca_Kabuli_denovo	9101826	(A/G)
19041	CakSNP19041	*Kabuli*	Ca_Kabuli_denovo	9110057	(T/G)
19042	CakSNP19042	*Kabuli*	Ca_Kabuli_denovo	9116771	(G/A)
19043	CakSNP19043	*Kabuli*	Ca_Kabuli_denovo	9116864	(C/T)
19044	CakSNP19044	*Kabuli*	Ca_Kabuli_denovo	9116876	(C/G)
19045	CakSNP19045	*Kabuli*	Ca_Kabuli_denovo	9118126	(A/G)
19046	CakSNP19046	*Kabuli*	Ca_Kabuli_denovo	9119868	(A/C)
19047	CakSNP19047	*Kabuli*	Ca_Kabuli_denovo	9119949	(G/A)
19048	CakSNP19048	*Kabuli*	Ca_Kabuli_denovo	9120924	(C/T)
19049	CakSNP19049	*Kabuli*	Ca_Kabuli_denovo	9120942	(C/G)
19050	CakSNP19050	*Kabuli*	Ca_Kabuli_denovo	9124892	(A/G)
19051	CakSNP19051	*Kabuli*	Ca_Kabuli_denovo	9124895	(G/T)
19052	CakSNP19052	*Kabuli*	Ca_Kabuli_denovo	9125565	(C/T)
19053	CakSNP19053	*Kabuli*	Ca_Kabuli_denovo	9128353	(A/G)
19054	CakSNP19054	*Kabuli*	Ca_Kabuli_denovo	9128371	(C/T)
19055	CakSNP19055	*Kabuli*	Ca_Kabuli_denovo	9130620	(G/T)
19056	CakSNP19056	*Kabuli*	Ca_Kabuli_denovo	9140141	(G/A)
19057	CakSNP19057	*Kabuli*	Ca_Kabuli_denovo	9140451	(G/C)
19058	CakSNP19058	*Kabuli*	Ca_Kabuli_denovo	9140463	(A/G)
19059	CakSNP19059	*Kabuli*	Ca_Kabuli_denovo	9142027	(T/C)
19060	CakSNP19060	*Kabuli*	Ca_Kabuli_denovo	9147040	(G/C)
19061	CakSNP19061	*Kabuli*	Ca_Kabuli_denovo	9147082	(G/C)
19062	CakSNP19062	*Kabuli*	Ca_Kabuli_denovo	9148606	(G/C)
19063	CakSNP19063	*Kabuli*	Ca_Kabuli_denovo	9150620	(G/C)
19064	CakSNP19064	*Kabuli*	Ca_Kabuli_denovo	9156588	(G/T)
19065	CakSNP19065	*Kabuli*	Ca_Kabuli_denovo	9156594	(C/T)
19066	CakSNP19066	*Kabuli*	Ca_Kabuli_denovo	9158920	(T/G)
19067	CakSNP19067	*Kabuli*	Ca_Kabuli_denovo	9160264	(G/C)
19068	CakSNP19068	*Kabuli*	Ca_Kabuli_denovo	9160267	(T/G)
19069	CakSNP19069	*Kabuli*	Ca_Kabuli_denovo	9172662	(A/C)
19070	CakSNP19070	*Kabuli*	Ca_Kabuli_denovo	9173737	(C/T)
19071	CakSNP19071	*Kabuli*	Ca_Kabuli_denovo	9173759	(C/A)
19072	CakSNP19072	*Kabuli*	Ca_Kabuli_denovo	9176774	(A/C)
19073	CakSNP19073	*Kabuli*	Ca_Kabuli_denovo	9176776	(A/G)
19074	CakSNP19074	*Kabuli*	Ca_Kabuli_denovo	9177251	(T/G)
19075	CakSNP19075	*Kabuli*	Ca_Kabuli_denovo	9177255	(A/C)

S.N.	SNP IDs	Cultivars	Chromosomes/scaffolds	Physical positions (bp)	SNPs
19076	CakSNP19076	*Kabuli*	Ca_Kabuli_denovo	9185606	(A/C)
19077	CakSNP19077	*Kabuli*	Ca_Kabuli_denovo	9185607	(G/C)
19078	CakSNP19078	*Kabuli*	Ca_Kabuli_denovo	9185932	(C/T)
19079	CakSNP19079	*Kabuli*	Ca_Kabuli_denovo	9187737	(T/G)
19080	CakSNP19080	*Kabuli*	Ca_Kabuli_denovo	9192222	(G/A)
19081	CakSNP19081	*Kabuli*	Ca_Kabuli_denovo	9192798	(G/A)
19082	CakSNP19082	*Kabuli*	Ca_Kabuli_denovo	9192851	(T/C)
19083	CakSNP19083	*Kabuli*	Ca_Kabuli_denovo	9198550	(A/G)
19084	CakSNP19084	*Kabuli*	Ca_Kabuli_denovo	9198616	(C/G)
19085	CakSNP19085	*Kabuli*	Ca_Kabuli_denovo	9201182	(T/C)
19086	CakSNP19086	*Kabuli*	Ca_Kabuli_denovo	9203603	(T/C)
19087	CakSNP19087	*Kabuli*	Ca_Kabuli_denovo	9205951	(A/G)
19088	CakSNP19088	*Kabuli*	Ca_Kabuli_denovo	9208178	(C/A)
19089	CakSNP19089	*Kabuli*	Ca_Kabuli_denovo	9208179	(A/G)
19090	CakSNP19090	*Kabuli*	Ca_Kabuli_denovo	9209336	(T/G)
19091	CakSNP19091	*Kabuli*	Ca_Kabuli_denovo	9214280	(G/C)
19092	CakSNP19092	*Kabuli*	Ca_Kabuli_denovo	9224701	(A/G)
19093	CakSNP19093	*Kabuli*	Ca_Kabuli_denovo	9224756	(A/G)
19094	CakSNP19094	*Kabuli*	Ca_Kabuli_denovo	9226486	(T/C)
19095	CakSNP19095	*Kabuli*	Ca_Kabuli_denovo	9239246	(T/C)
19096	CakSNP19096	*Kabuli*	Ca_Kabuli_denovo	9239264	(A/G)
19097	CakSNP19097	*Kabuli*	Ca_Kabuli_denovo	9239699	(G/A)
19098	CakSNP19098	*Kabuli*	Ca_Kabuli_denovo	9239708	(G/A)
19099	CakSNP19099	*Kabuli*	Ca_Kabuli_denovo	9248504	(T/C)
19100	CakSNP19100	*Kabuli*	Ca_Kabuli_denovo	9248531	(C/G)
19101	CakSNP19101	*Kabuli*	Ca_Kabuli_denovo	9250624	(A/G)
19102	CakSNP19102	*Kabuli*	Ca_Kabuli_denovo	9251558	(C/G)
19103	CakSNP19103	*Kabuli*	Ca_Kabuli_denovo	9252627	(C/T)
19104	CakSNP19104	*Kabuli*	Ca_Kabuli_denovo	9260377	(A/G)
19105	CakSNP19105	*Kabuli*	Ca_Kabuli_denovo	9275027	(C/T)
19106	CakSNP19106	*Kabuli*	Ca_Kabuli_denovo	9279086	(G/A)
19107	CakSNP19107	*Kabuli*	Ca_Kabuli_denovo	9279103	(C/G)
19108	CakSNP19108	*Kabuli*	Ca_Kabuli_denovo	9280476	(G/T)
19109	CakSNP19109	*Kabuli*	Ca_Kabuli_denovo	9285521	(A/G)
19110	CakSNP19110	*Kabuli*	Ca_Kabuli_denovo	9285552	(A/C)
19111	CakSNP19111	*Kabuli*	Ca_Kabuli_denovo	9290097	(C/T)
19112	CakSNP19112	*Kabuli*	Ca_Kabuli_denovo	9290124	(T/C)
19113	CakSNP19113	*Kabuli*	Ca_Kabuli_denovo	9291022	(T/C)

S.N.	SNP IDs	Cultivars	Chromosomes/scaffolds	Physical positions (bp)	SNPs
19114	CakSNP19114	*Kabuli*	Ca_Kabuli_denovo	9291386	(G/T)
19115	CakSNP19115	*Kabuli*	Ca_Kabuli_denovo	9295107	(C/T)
19116	CakSNP19116	*Kabuli*	Ca_Kabuli_denovo	9302031	(A/G)
19117	CakSNP19117	*Kabuli*	Ca_Kabuli_denovo	9303206	(G/C)
19118	CakSNP19118	*Kabuli*	Ca_Kabuli_denovo	9303255	(G/A)
19119	CakSNP19119	*Kabuli*	Ca_Kabuli_denovo	9306274	(T/C)
19120	CakSNP19120	*Kabuli*	Ca_Kabuli_denovo	9311223	(T/C)
19121	CakSNP19121	*Kabuli*	Ca_Kabuli_denovo	9311232	(T/C)
19122	CakSNP19122	*Kabuli*	Ca_Kabuli_denovo	9313124	(G/A)
19123	CakSNP19123	*Kabuli*	Ca_Kabuli_denovo	9313760	(A/C)
19124	CakSNP19124	*Kabuli*	Ca_Kabuli_denovo	9313761	(G/C)
19125	CakSNP19125	*Kabuli*	Ca_Kabuli_denovo	9313763	(T/A)
19126	CakSNP19126	*Kabuli*	Ca_Kabuli_denovo	9313764	(C/T)
19127	CakSNP19127	*Kabuli*	Ca_Kabuli_denovo	9323010	(A/G)
19128	CakSNP19128	*Kabuli*	Ca_Kabuli_denovo	9325321	(G/A)
19129	CakSNP19129	*Kabuli*	Ca_Kabuli_denovo	9326340	(C/G)
19130	CakSNP19130	*Kabuli*	Ca_Kabuli_denovo	9326358	(C/T)
19131	CakSNP19131	*Kabuli*	Ca_Kabuli_denovo	9327287	(G/A)
19132	CakSNP19132	*Kabuli*	Ca_Kabuli_denovo	9329987	(A/G)
19133	CakSNP19133	*Kabuli*	Ca_Kabuli_denovo	9331268	(T/A)
19134	CakSNP19134	*Kabuli*	Ca_Kabuli_denovo	9331322	(G/A)
19135	CakSNP19135	*Kabuli*	Ca_Kabuli_denovo	9336282	(C/T)
19136	CakSNP19136	*Kabuli*	Ca_Kabuli_denovo	9336314	(A/G)
19137	CakSNP19137	*Kabuli*	Ca_Kabuli_denovo	9337079	(A/G)
19138	CakSNP19138	*Kabuli*	Ca_Kabuli_denovo	9337886	(G/A)
19139	CakSNP19139	*Kabuli*	Ca_Kabuli_denovo	9337907	(G/A)
19140	CakSNP19140	*Kabuli*	Ca_Kabuli_denovo	9341919	(G/A)
19141	CakSNP19141	*Kabuli*	Ca_Kabuli_denovo	9343441	(C/T)
19142	CakSNP19142	*Kabuli*	Ca_Kabuli_denovo	9343510	(A/C)
19143	CakSNP19143	*Kabuli*	Ca_Kabuli_denovo	9343631	(G/A)
19144	CakSNP19144	*Kabuli*	Ca_Kabuli_denovo	9349136	(G/A)
19145	CakSNP19145	*Kabuli*	Ca_Kabuli_denovo	9349164	(C/T)
19146	CakSNP19146	*Kabuli*	Ca_Kabuli_denovo	9360445	(T/C)
19147	CakSNP19147	*Kabuli*	Ca_Kabuli_denovo	9360845	(C/G)
19148	CakSNP19148	*Kabuli*	Ca_Kabuli_denovo	9361697	(A/G)
19149	CakSNP19149	*Kabuli*	Ca_Kabuli_denovo	9362542	(T/G)
19150	CakSNP19150	*Kabuli*	Ca_Kabuli_denovo	9364583	(A/G)
19151	CakSNP19151	*Kabuli*	Ca_Kabuli_denovo	9364589	(C/G)

S.N.	SNP IDs	Cultivars	Chromosomes/scaffolds	Physical positions (bp)	SNPs
19152	CakSNP19152	*Kabuli*	Ca_Kabuli_denovo	9370733	(A/C)
19153	CakSNP19153	*Kabuli*	Ca_Kabuli_denovo	9373782	(A/G)
19154	CakSNP19154	*Kabuli*	Ca_Kabuli_denovo	9378726	(T/C)
19155	CakSNP19155	*Kabuli*	Ca_Kabuli_denovo	9380666	(A/G)
19156	CakSNP19156	*Kabuli*	Ca_Kabuli_denovo	9380693	(T/C)
19157	CakSNP19157	*Kabuli*	Ca_Kabuli_denovo	9383066	(T/C)
19158	CakSNP19158	*Kabuli*	Ca_Kabuli_denovo	9386351	(T/C)
19159	CakSNP19159	*Kabuli*	Ca_Kabuli_denovo	9389346	(C/T)
19160	CakSNP19160	*Kabuli*	Ca_Kabuli_denovo	9393532	(C/T)
19161	CakSNP19161	*Kabuli*	Ca_Kabuli_denovo	9394058	(G/A)
19162	CakSNP19162	*Kabuli*	Ca_Kabuli_denovo	9395983	(G/T)
19163	CakSNP19163	*Kabuli*	Ca_Kabuli_denovo	9396052	(G/A)
19164	CakSNP19164	*Kabuli*	Ca_Kabuli_denovo	9396286	(T/C)
19165	CakSNP19165	*Kabuli*	Ca_Kabuli_denovo	9396295	(T/C)
19166	CakSNP19166	*Kabuli*	Ca_Kabuli_denovo	9397435	(C/T)
19167	CakSNP19167	*Kabuli*	Ca_Kabuli_denovo	9397440	(T/A)
19168	CakSNP19168	*Kabuli*	Ca_Kabuli_denovo	9404461	(G/A)
19169	CakSNP19169	*Kabuli*	Ca_Kabuli_denovo	9404509	(T/C)
19170	CakSNP19170	*Kabuli*	Ca_Kabuli_denovo	9410160	(A/G)
19171	CakSNP19171	*Kabuli*	Ca_Kabuli_denovo	9412751	(G/C)
19172	CakSNP19172	*Kabuli*	Ca_Kabuli_denovo	9412760	(A/G)
19173	CakSNP19173	*Kabuli*	Ca_Kabuli_denovo	9414379	(T/G)
19174	CakSNP19174	*Kabuli*	Ca_Kabuli_denovo	9415076	(A/G)
19175	CakSNP19175	*Kabuli*	Ca_Kabuli_denovo	9415133	(G/A)
19176	CakSNP19176	*Kabuli*	Ca_Kabuli_denovo	9418681	(T/A)
19177	CakSNP19177	*Kabuli*	Ca_Kabuli_denovo	9421464	(A/C)
19178	CakSNP19178	*Kabuli*	Ca_Kabuli_denovo	9421521	(T/C)
19179	CakSNP19179	*Kabuli*	Ca_Kabuli_denovo	9425870	(T/C)
19180	CakSNP19180	*Kabuli*	Ca_Kabuli_denovo	9429363	(A/C)
19181	CakSNP19181	*Kabuli*	Ca_Kabuli_denovo	9432400	(T/C)
19182	CakSNP19182	*Kabuli*	Ca_Kabuli_denovo	9434348	(T/C)
19183	CakSNP19183	*Kabuli*	Ca_Kabuli_denovo	9434372	(T/G)
19184	CakSNP19184	*Kabuli*	Ca_Kabuli_denovo	9435488	(T/G)
19185	CakSNP19185	*Kabuli*	Ca_Kabuli_denovo	9437375	(A/G)
19186	CakSNP19186	*Kabuli*	Ca_Kabuli_denovo	9437396	(C/G)
19187	CakSNP19187	*Kabuli*	Ca_Kabuli_denovo	9437960	(C/T)
19188	CakSNP19188	*Kabuli*	Ca_Kabuli_denovo	9437977	(A/G)
19189	CakSNP19189	*Kabuli*	Ca_Kabuli_denovo	9438865	(G/C)

S.N.	SNP IDs	Cultivars	Chromosomes/scaffolds	Physical positions (bp)	SNPs
19190	CakSNP19190	*Kabuli*	Ca_Kabuli_denovo	9442353	(G/C)
19191	CakSNP19191	*Kabuli*	Ca_Kabuli_denovo	9442362	(A/G)
19192	CakSNP19192	*Kabuli*	Ca_Kabuli_denovo	9444085	(T/C)
19193	CakSNP19193	*Kabuli*	Ca_Kabuli_denovo	9448354	(G/T)
19194	CakSNP19194	*Kabuli*	Ca_Kabuli_denovo	9452904	(G/A)
19195	CakSNP19195	*Kabuli*	Ca_Kabuli_denovo	9456475	(T/C)
19196	CakSNP19196	*Kabuli*	Ca_Kabuli_denovo	9456505	(T/G)
19197	CakSNP19197	*Kabuli*	Ca_Kabuli_denovo	9457401	(G/C)
19198	CakSNP19198	*Kabuli*	Ca_Kabuli_denovo	9462647	(A/G)
19199	CakSNP19199	*Kabuli*	Ca_Kabuli_denovo	9462650	(C/G)
19200	CakSNP19200	*Kabuli*	Ca_Kabuli_denovo	9469427	(G/C)
19201	CakSNP19201	*Kabuli*	Ca_Kabuli_denovo	9475012	(A/G)
19202	CakSNP19202	*Kabuli*	Ca_Kabuli_denovo	9479197	(G/C)
19203	CakSNP19203	*Kabuli*	Ca_Kabuli_denovo	9479611	(A/T)
19204	CakSNP19204	*Kabuli*	Ca_Kabuli_denovo	9482483	(T/C)
19205	CakSNP19205	*Kabuli*	Ca_Kabuli_denovo	9493569	(T/G)
19206	CakSNP19206	*Kabuli*	Ca_Kabuli_denovo	9493584	(T/G)
19207	CakSNP19207	*Kabuli*	Ca_Kabuli_denovo	9495891	(C/G)
19208	CakSNP19208	*Kabuli*	Ca_Kabuli_denovo	9495933	(T/G)
19209	CakSNP19209	*Kabuli*	Ca_Kabuli_denovo	9501269	(T/C)
19210	CakSNP19210	*Kabuli*	Ca_Kabuli_denovo	9503039	(G/T)
19211	CakSNP19211	*Kabuli*	Ca_Kabuli_denovo	9510912	(A/G)
19212	CakSNP19212	*Kabuli*	Ca_Kabuli_denovo	9511963	(C/T)
19213	CakSNP19213	*Kabuli*	Ca_Kabuli_denovo	9511976	(G/A)
19214	CakSNP19214	*Kabuli*	Ca_Kabuli_denovo	9513069	(C/T)
19215	CakSNP19215	*Kabuli*	Ca_Kabuli_denovo	9513373	(T/C)
19216	CakSNP19216	*Kabuli*	Ca_Kabuli_denovo	9513845	(G/A)
19217	CakSNP19217	*Kabuli*	Ca_Kabuli_denovo	9518015	(G/A)
19218	CakSNP19218	*Kabuli*	Ca_Kabuli_denovo	9519581	(C/T)
19219	CakSNP19219	*Kabuli*	Ca_Kabuli_denovo	9519617	(C/T)
19220	CakSNP19220	*Kabuli*	Ca_Kabuli_denovo	9519815	(A/G)
19221	CakSNP19221	*Kabuli*	Ca_Kabuli_denovo	9519824	(A/G)
19222	CakSNP19222	*Kabuli*	Ca_Kabuli_denovo	9522876	(C/G)
19223	CakSNP19223	*Kabuli*	Ca_Kabuli_denovo	9523870	(C/G)
19224	CakSNP19224	*Kabuli*	Ca_Kabuli_denovo	9523885	(C/T)
19225	CakSNP19225	*Kabuli*	Ca_Kabuli_denovo	9523894	(C/A)
19226	CakSNP19226	*Kabuli*	Ca_Kabuli_denovo	9524048	(A/G)
19227	CakSNP19227	*Kabuli*	Ca_Kabuli_denovo	9527559	(T/A)

**S.N.**	**SNP IDs**	**Cultivars**	**Chromosomes/scaffolds**	**Physical positions (bp)**	**SNPs**
19228	CakSNP19228	*Kabuli*	Ca_Kabuli_denovo	9533107	(C/T)
19229	CakSNP19229	*Kabuli*	Ca_Kabuli_denovo	9533143	(C/T)
19230	CakSNP19230	*Kabuli*	Ca_Kabuli_denovo	9533629	(G/A)
19231	CakSNP19231	*Kabuli*	Ca_Kabuli_denovo	9533635	(A/G)
19232	CakSNP19232	*Kabuli*	Ca_Kabuli_denovo	9533903	(G/C)
19233	CakSNP19233	*Kabuli*	Ca_Kabuli_denovo	9533953	(G/T)
19234	CakSNP19234	*Kabuli*	Ca_Kabuli_denovo	9535405	(A/C)
19235	CakSNP19235	*Kabuli*	Ca_Kabuli_denovo	9535430	(A/C)
19236	CakSNP19236	*Kabuli*	Ca_Kabuli_denovo	9545146	(T/G)
19237	CakSNP19237	*Kabuli*	Ca_Kabuli_denovo	9545486	(A/T)
19238	CakSNP19238	*Kabuli*	Ca_Kabuli_denovo	9551035	(G/C)
19239	CakSNP19239	*Kabuli*	Ca_Kabuli_denovo	9554120	(A/G)
19240	CakSNP19240	*Kabuli*	Ca_Kabuli_denovo	9555017	(C/T)
19241	CakSNP19241	*Kabuli*	Ca_Kabuli_denovo	9555080	(T/C)
19242	CakSNP19242	*Kabuli*	Ca_Kabuli_denovo	9565532	(C/T)
19243	CakSNP19243	*Kabuli*	Ca_Kabuli_denovo	9565561	(T/G)
19244	CakSNP19244	*Kabuli*	Ca_Kabuli_denovo	9573858	(T/C)
19245	CakSNP19245	*Kabuli*	Ca_Kabuli_denovo	9585179	(T/C)
19246	CakSNP19246	*Kabuli*	Ca_Kabuli_denovo	9586184	(G/C)
19247	CakSNP19247	*Kabuli*	Ca_Kabuli_denovo	9595407	(C/G)
19248	CakSNP19248	*Kabuli*	Ca_Kabuli_denovo	9595430	(C/T)
19249	CakSNP19249	*Kabuli*	Ca_Kabuli_denovo	9595612	(T/G)
19250	CakSNP19250	*Kabuli*	Ca_Kabuli_denovo	9595624	(C/G)
19251	CakSNP19251	*Kabuli*	Ca_Kabuli_denovo	9602675	(T/C)
19252	CakSNP19252	*Kabuli*	Ca_Kabuli_denovo	9607619	(G/A)
19253	CakSNP19253	*Kabuli*	Ca_Kabuli_denovo	9608629	(C/T)
19254	CakSNP19254	*Kabuli*	Ca_Kabuli_denovo	9611662	(C/T)
19255	CakSNP19255	*Kabuli*	Ca_Kabuli_denovo	9611695	(A/C)
19256	CakSNP19256	*Kabuli*	Ca_Kabuli_denovo	9612481	(C/T)
19257	CakSNP19257	*Kabuli*	Ca_Kabuli_denovo	9615028	(G/T)
19258	CakSNP19258	*Kabuli*	Ca_Kabuli_denovo	9619205	(G/A)
19259	CakSNP19259	*Kabuli*	Ca_Kabuli_denovo	9622636	(A/C)
19260	CakSNP19260	*Kabuli*	Ca_Kabuli_denovo	9624793	(T/C)
19261	CakSNP19261	*Kabuli*	Ca_Kabuli_denovo	9626464	(G/A)
19262	CakSNP19262	*Kabuli*	Ca_Kabuli_denovo	9626469	(G/A)
19263	CakSNP19263	*Kabuli*	Ca_Kabuli_denovo	9626640	(G/T)
19264	CakSNP19264	*Kabuli*	Ca_Kabuli_denovo	9626684	(C/G)
19265	CakSNP19265	*Kabuli*	Ca_Kabuli_denovo	9627699	(T/C)

S.N.	SNP IDs	Cultivars	Chromosomes/scaffolds	Physical positions (bp)	SNPs
19266	CakSNP19266	*Kabuli*	Ca_Kabuli_denovo	9628193	(A/G)
19267	CakSNP19267	*Kabuli*	Ca_Kabuli_denovo	9628195	(T/G)
19268	CakSNP19268	*Kabuli*	Ca_Kabuli_denovo	9633018	(A/C)
19269	CakSNP19269	*Kabuli*	Ca_Kabuli_denovo	9633019	(G/A)
19270	CakSNP19270	*Kabuli*	Ca_Kabuli_denovo	9634980	(C/G)
19271	CakSNP19271	*Kabuli*	Ca_Kabuli_denovo	9634986	(C/T)
19272	CakSNP19272	*Kabuli*	Ca_Kabuli_denovo	9635479	(G/A)
19273	CakSNP19273	*Kabuli*	Ca_Kabuli_denovo	9639661	(A/G)
19274	CakSNP19274	*Kabuli*	Ca_Kabuli_denovo	9639705	(C/A)
19275	CakSNP19275	*Kabuli*	Ca_Kabuli_denovo	9648137	(G/A)
19276	CakSNP19276	*Kabuli*	Ca_Kabuli_denovo	9650779	(A/G)
19277	CakSNP19277	*Kabuli*	Ca_Kabuli_denovo	9653500	(G/A)
19278	CakSNP19278	*Kabuli*	Ca_Kabuli_denovo	9653548	(G/T)
19279	CakSNP19279	*Kabuli*	Ca_Kabuli_denovo	9655416	(G/C)
19280	CakSNP19280	*Kabuli*	Ca_Kabuli_denovo	9655928	(C/G)
19281	CakSNP19281	*Kabuli*	Ca_Kabuli_denovo	9658278	(G/A)
19282	CakSNP19282	*Kabuli*	Ca_Kabuli_denovo	9660064	(C/G)
19283	CakSNP19283	*Kabuli*	Ca_Kabuli_denovo	9661613	(G/C)
19284	CakSNP19284	*Kabuli*	Ca_Kabuli_denovo	9664866	(C/T)
19285	CakSNP19285	*Kabuli*	Ca_Kabuli_denovo	9664901	(G/C)
19286	CakSNP19286	*Kabuli*	Ca_Kabuli_denovo	9668776	(G/T)
19287	CakSNP19287	*Kabuli*	Ca_Kabuli_denovo	9672444	(T/C)
19288	CakSNP19288	*Kabuli*	Ca_Kabuli_denovo	9672812	(G/A)
19289	CakSNP19289	*Kabuli*	Ca_Kabuli_denovo	9678908	(A/G)
19290	CakSNP19290	*Kabuli*	Ca_Kabuli_denovo	9678917	(C/T)
19291	CakSNP19291	*Kabuli*	Ca_Kabuli_denovo	9679714	(T/G)
19292	CakSNP19292	*Kabuli*	Ca_Kabuli_denovo	9681415	(G/C)
19293	CakSNP19293	*Kabuli*	Ca_Kabuli_denovo	9685223	(G/C)
19294	CakSNP19294	*Kabuli*	Ca_Kabuli_denovo	9689620	(A/G)
19295	CakSNP19295	*Kabuli*	Ca_Kabuli_denovo	9689670	(A/G)
19296	CakSNP19296	*Kabuli*	Ca_Kabuli_denovo	9699577	(C/T)
19297	CakSNP19297	*Kabuli*	Ca_Kabuli_denovo	9702980	(T/C)
19298	CakSNP19298	*Kabuli*	Ca_Kabuli_denovo	9702994	(T/C)
19299	CakSNP19299	*Kabuli*	Ca_Kabuli_denovo	9718247	(G/A)
19300	CakSNP19300	*Kabuli*	Ca_Kabuli_denovo	9718330	(C/A)
19301	CakSNP19301	*Kabuli*	Ca_Kabuli_denovo	9718375	(C/T)
19302	CakSNP19302	*Kabuli*	Ca_Kabuli_denovo	9719868	(T/C)
19303	CakSNP19303	*Kabuli*	Ca_Kabuli_denovo	9720016	(T/C)

S.N.	SNP IDs	Cultivars	Chromosomes/scaffolds	Physical positions (bp)	SNPs
19304	CakSNP19304	*Kabuli*	Ca_Kabuli_denovo	9720034	(A/G)
19305	CakSNP19305	*Kabuli*	Ca_Kabuli_denovo	9725329	(A/C)
19306	CakSNP19306	*Kabuli*	Ca_Kabuli_denovo	9726829	(G/T)
19307	CakSNP19307	*Kabuli*	Ca_Kabuli_denovo	9726834	(T/C)
19308	CakSNP19308	*Kabuli*	Ca_Kabuli_denovo	9730519	(A/G)
19309	CakSNP19309	*Kabuli*	Ca_Kabuli_denovo	9730534	(C/A)
19310	CakSNP19310	*Kabuli*	Ca_Kabuli_denovo	9737591	(A/G)
19311	CakSNP19311	*Kabuli*	Ca_Kabuli_denovo	9738624	(A/C)
19312	CakSNP19312	*Kabuli*	Ca_Kabuli_denovo	9738660	(G/A)
19313	CakSNP19313	*Kabuli*	Ca_Kabuli_denovo	9739260	(A/C)
19314	CakSNP19314	*Kabuli*	Ca_Kabuli_denovo	9739831	(G/A)
19315	CakSNP19315	*Kabuli*	Ca_Kabuli_denovo	9741292	(C/A)
19316	CakSNP19316	*Kabuli*	Ca_Kabuli_denovo	9751264	(G/C)
19317	CakSNP19317	*Kabuli*	Ca_Kabuli_denovo	9751267	(C/T)
19318	CakSNP19318	*Kabuli*	Ca_Kabuli_denovo	9751700	(A/C)
19319	CakSNP19319	*Kabuli*	Ca_Kabuli_denovo	9751701	(G/A)
19320	CakSNP19320	*Kabuli*	Ca_Kabuli_denovo	9758471	(C/T)
19321	CakSNP19321	*Kabuli*	Ca_Kabuli_denovo	9765484	(C/G)
19322	CakSNP19322	*Kabuli*	Ca_Kabuli_denovo	9765523	(T/C)
19323	CakSNP19323	*Kabuli*	Ca_Kabuli_denovo	9766654	(C/T)
19324	CakSNP19324	*Kabuli*	Ca_Kabuli_denovo	9780824	(T/C)
19325	CakSNP19325	*Kabuli*	Ca_Kabuli_denovo	9780872	(T/C)
19326	CakSNP19326	*Kabuli*	Ca_Kabuli_denovo	9781196	(C/T)
19327	CakSNP19327	*Kabuli*	Ca_Kabuli_denovo	9781236	(A/G)
19328	CakSNP19328	*Kabuli*	Ca_Kabuli_denovo	9783341	(A/G)
19329	CakSNP19329	*Kabuli*	Ca_Kabuli_denovo	9786959	(G/C)
19330	CakSNP19330	*Kabuli*	Ca_Kabuli_denovo	9788350	(G/A)
19331	CakSNP19331	*Kabuli*	Ca_Kabuli_denovo	9788437	(T/C)
19332	CakSNP19332	*Kabuli*	Ca_Kabuli_denovo	9793854	(C/G)
19333	CakSNP19333	*Kabuli*	Ca_Kabuli_denovo	9802771	(T/G)
19334	CakSNP19334	*Kabuli*	Ca_Kabuli_denovo	9804090	(G/A)
19335	CakSNP19335	*Kabuli*	Ca_Kabuli_denovo	9804109	(C/T)
19336	CakSNP19336	*Kabuli*	Ca_Kabuli_denovo	9804576	(G/A)
19337	CakSNP19337	*Kabuli*	Ca_Kabuli_denovo	9809489	(T/C)
19338	CakSNP19338	*Kabuli*	Ca_Kabuli_denovo	9809493	(T/C)
19339	CakSNP19339	*Kabuli*	Ca_Kabuli_denovo	9815146	(G/T)
19340	CakSNP19340	*Kabuli*	Ca_Kabuli_denovo	9815151	(A/T)
19341	CakSNP19341	*Kabuli*	Ca_Kabuli_denovo	9823378	(C/G)

S.N.	SNP IDs	Cultivars	Chromosomes/scaffolds	Physical positions (bp)	SNPs
19342	CakSNP19342	*Kabuli*	Ca_Kabuli_denovo	9830944	(C/T)
19343	CakSNP19343	*Kabuli*	Ca_Kabuli_denovo	9830977	(T/C)
19344	CakSNP19344	*Kabuli*	Ca_Kabuli_denovo	9832549	(G/A)
19345	CakSNP19345	*Kabuli*	Ca_Kabuli_denovo	9833972	(G/A)
19346	CakSNP19346	*Kabuli*	Ca_Kabuli_denovo	9836572	(G/T)
19347	CakSNP19347	*Kabuli*	Ca_Kabuli_denovo	9845280	(A/C)
19348	CakSNP19348	*Kabuli*	Ca_Kabuli_denovo	9845868	(T/C)
19349	CakSNP19349	*Kabuli*	Ca_Kabuli_denovo	9847577	(C/A)
19350	CakSNP19350	*Kabuli*	Ca_Kabuli_denovo	9847585	(G/A)
19351	CakSNP19351	*Kabuli*	Ca_Kabuli_denovo	9848680	(G/C)
19352	CakSNP19352	*Kabuli*	Ca_Kabuli_denovo	9850046	(A/G)
19353	CakSNP19353	*Kabuli*	Ca_Kabuli_denovo	9853101	(A/C)
19354	CakSNP19354	*Kabuli*	Ca_Kabuli_denovo	9863140	(A/G)
19355	CakSNP19355	*Kabuli*	Ca_Kabuli_denovo	9863513	(T/C)
19356	CakSNP19356	*Kabuli*	Ca_Kabuli_denovo	9863525	(G/C)
19357	CakSNP19357	*Kabuli*	Ca_Kabuli_denovo	9865957	(C/T)
19358	CakSNP19358	*Kabuli*	Ca_Kabuli_denovo	9870575	(T/C)
19359	CakSNP19359	*Kabuli*	Ca_Kabuli_denovo	9872528	(T/G)
19360	CakSNP19360	*Kabuli*	Ca_Kabuli_denovo	9872591	(C/G)
19361	CakSNP19361	*Kabuli*	Ca_Kabuli_denovo	9878441	(A/G)
19362	CakSNP19362	*Kabuli*	Ca_Kabuli_denovo	9880795	(C/G)
19363	CakSNP19363	*Kabuli*	Ca_Kabuli_denovo	9881788	(G/T)
19364	CakSNP19364	*Kabuli*	Ca_Kabuli_denovo	9881800	(A/G)
19365	CakSNP19365	*Kabuli*	Ca_Kabuli_denovo	9884524	(T/G)
19366	CakSNP19366	*Kabuli*	Ca_Kabuli_denovo	9885838	(G/C)
19367	CakSNP19367	*Kabuli*	Ca_Kabuli_denovo	9889813	(T/C)
19368	CakSNP19368	*Kabuli*	Ca_Kabuli_denovo	9891114	(G/T)
19369	CakSNP19369	*Kabuli*	Ca_Kabuli_denovo	9891158	(T/C)
19370	CakSNP19370	*Kabuli*	Ca_Kabuli_denovo	9891452	(A/G)
19371	CakSNP19371	*Kabuli*	Ca_Kabuli_denovo	9896914	(G/A)
19372	CakSNP19372	*Kabuli*	Ca_Kabuli_denovo	9896968	(G/A)
19373	CakSNP19373	*Kabuli*	Ca_Kabuli_denovo	9899452	(C/G)
19374	CakSNP19374	*Kabuli*	Ca_Kabuli_denovo	9901759	(C/T)
19375	CakSNP19375	*Kabuli*	Ca_Kabuli_denovo	9901779	(G/C)
19376	CakSNP19376	*Kabuli*	Ca_Kabuli_denovo	9908975	(T/C)
19377	CakSNP19377	*Kabuli*	Ca_Kabuli_denovo	9913858	(G/T)
19378	CakSNP19378	*Kabuli*	Ca_Kabuli_denovo	9913864	(G/A)
19379	CakSNP19379	*Kabuli*	Ca_Kabuli_denovo	9914830	(G/T)

S.N.	SNP IDs	Cultivars	Chromosomes/scaffolds	Physical positions (bp)	SNPs
19380	CakSNP19380	*Kabuli*	Ca_Kabuli_denovo	9915716	(C/G)
19381	CakSNP19381	*Kabuli*	Ca_Kabuli_denovo	9915762	(T/G)
19382	CakSNP19382	*Kabuli*	Ca_Kabuli_denovo	9915772	(C/G)
19383	CakSNP19383	*Kabuli*	Ca_Kabuli_denovo	9918090	(C/A)
19384	CakSNP19384	*Kabuli*	Ca_Kabuli_denovo	9918838	(C/G)
19385	CakSNP19385	*Kabuli*	Ca_Kabuli_denovo	9918841	(A/G)
19386	CakSNP19386	*Kabuli*	Ca_Kabuli_denovo	9918853	(C/T)
19387	CakSNP19387	*Kabuli*	Ca_Kabuli_denovo	9923995	(C/T)
19388	CakSNP19388	*Kabuli*	Ca_Kabuli_denovo	9924598	(C/T)
19389	CakSNP19389	*Kabuli*	Ca_Kabuli_denovo	9924640	(T/A)
19390	CakSNP19390	*Kabuli*	Ca_Kabuli_denovo	9924819	(G/T)
19391	CakSNP19391	*Kabuli*	Ca_Kabuli_denovo	9924862	(G/A)
19392	CakSNP19392	*Kabuli*	Ca_Kabuli_denovo	9929093	(C/T)
19393	CakSNP19393	*Kabuli*	Ca_Kabuli_denovo	9932690	(G/A)
19394	CakSNP19394	*Kabuli*	Ca_Kabuli_denovo	9932696	(G/C)
19395	CakSNP19395	*Kabuli*	Ca_Kabuli_denovo	9933865	(G/C)
19396	CakSNP19396	*Kabuli*	Ca_Kabuli_denovo	9933889	(A/G)
19397	CakSNP19397	*Kabuli*	Ca_Kabuli_denovo	9937838	(T/C)
19398	CakSNP19398	*Kabuli*	Ca_Kabuli_denovo	9938641	(A/G)
19399	CakSNP19399	*Kabuli*	Ca_Kabuli_denovo	9938665	(G/C)
19400	CakSNP19400	*Kabuli*	Ca_Kabuli_denovo	9940609	(C/T)
19401	CakSNP19401	*Kabuli*	Ca_Kabuli_denovo	9942826	(T/C)
19402	CakSNP19402	*Kabuli*	Ca_Kabuli_denovo	9942846	(A/G)
19403	CakSNP19403	*Kabuli*	Ca_Kabuli_denovo	9947560	(A/C)
19404	CakSNP19404	*Kabuli*	Ca_Kabuli_denovo	9951000	(A/G)
19405	CakSNP19405	*Kabuli*	Ca_Kabuli_denovo	9962011	(G/A)
19406	CakSNP19406	*Kabuli*	Ca_Kabuli_denovo	9962035	(C/T)
19407	CakSNP19407	*Kabuli*	Ca_Kabuli_denovo	9973611	(G/T)
19408	CakSNP19408	*Kabuli*	Ca_Kabuli_denovo	9973617	(T/C)
19409	CakSNP19409	*Kabuli*	Ca_Kabuli_denovo	9973620	(A/G)
19410	CakSNP19410	*Kabuli*	Ca_Kabuli_denovo	9974899	(G/A)
19411	CakSNP19411	*Kabuli*	Ca_Kabuli_denovo	9976962	(T/C)
19412	CakSNP19412	*Kabuli*	Ca_Kabuli_denovo	9976966	(A/G)
19413	CakSNP19413	*Kabuli*	Ca_Kabuli_denovo	9981376	(A/C)
19414	CakSNP19414	*Kabuli*	Ca_Kabuli_denovo	9981406	(G/T)
19415	CakSNP19415	*Kabuli*	Ca_Kabuli_denovo	9985529	(C/G)
19416	CakSNP19416	*Kabuli*	Ca_Kabuli_denovo	9985544	(C/G)
19417	CakSNP19417	*Kabuli*	Ca_Kabuli_denovo	9996966	(G/A)

S.N.	SNP IDs	Cultivars	Chromosomes/scaffolds	Physical positions (bp)	SNPs
19418	CakSNP19418	*Kabuli*	Ca_Kabuli_denovo	9996979	(G/T)
19419	CakSNP19419	*Kabuli*	Ca_Kabuli_denovo	10003680	(G/T)
19420	CakSNP19420	*Kabuli*	Ca_Kabuli_denovo	10008824	(T/C)
19421	CakSNP19421	*Kabuli*	Ca_Kabuli_denovo	10009526	(T/C)
19422	CakSNP19422	*Kabuli*	Ca_Kabuli_denovo	10012784	(A/G)
19423	CakSNP19423	*Kabuli*	Ca_Kabuli_denovo	10012811	(G/C)
19424	CakSNP19424	*Kabuli*	Ca_Kabuli_denovo	10013047	(C/T)
19425	CakSNP19425	*Kabuli*	Ca_Kabuli_denovo	10013311	(A/C)
19426	CakSNP19426	*Kabuli*	Ca_Kabuli_denovo	10013608	(T/C)
19427	CakSNP19427	*Kabuli*	Ca_Kabuli_denovo	10013617	(T/G)
19428	CakSNP19428	*Kabuli*	Ca_Kabuli_denovo	10015026	(C/G)
19429	CakSNP19429	*Kabuli*	Ca_Kabuli_denovo	10015729	(T/G)
19430	CakSNP19430	*Kabuli*	Ca_Kabuli_denovo	10016049	(C/A)
19431	CakSNP19431	*Kabuli*	Ca_Kabuli_denovo	10019860	(A/G)
19432	CakSNP19432	*Kabuli*	Ca_Kabuli_denovo	10019905	(A/G)
19433	CakSNP19433	*Kabuli*	Ca_Kabuli_denovo	10022721	(T/C)
19434	CakSNP19434	*Kabuli*	Ca_Kabuli_denovo	10022740	(C/A)
19435	CakSNP19435	*Kabuli*	Ca_Kabuli_denovo	10032574	(T/C)
19436	CakSNP19436	*Kabuli*	Ca_Kabuli_denovo	10032577	(T/G)
19437	CakSNP19437	*Kabuli*	Ca_Kabuli_denovo	10039375	(C/G)
19438	CakSNP19438	*Kabuli*	Ca_Kabuli_denovo	10039400	(C/T)
19439	CakSNP19439	*Kabuli*	Ca_Kabuli_denovo	10040960	(T/C)
19440	CakSNP19440	*Kabuli*	Ca_Kabuli_denovo	10041262	(T/G)
19441	CakSNP19441	*Kabuli*	Ca_Kabuli_denovo	10043768	(T/C)
19442	CakSNP19442	*Kabuli*	Ca_Kabuli_denovo	10045315	(A/G)
19443	CakSNP19443	*Kabuli*	Ca_Kabuli_denovo	10045339	(C/G)
19444	CakSNP19444	*Kabuli*	Ca_Kabuli_denovo	10061400	(T/C)
19445	CakSNP19445	*Kabuli*	Ca_Kabuli_denovo	10061401	(T/C)
19446	CakSNP19446	*Kabuli*	Ca_Kabuli_denovo	10061405	(C/G)
19447	CakSNP19447	*Kabuli*	Ca_Kabuli_denovo	10068192	(T/C)
19448	CakSNP19448	*Kabuli*	Ca_Kabuli_denovo	10070360	(G/C)
19449	CakSNP19449	*Kabuli*	Ca_Kabuli_denovo	10077413	(C/T)
19450	CakSNP19450	*Kabuli*	Ca_Kabuli_denovo	10077418	(C/T)
19451	CakSNP19451	*Kabuli*	Ca_Kabuli_denovo	10078621	(C/T)
19452	CakSNP19452	*Kabuli*	Ca_Kabuli_denovo	10084025	(T/C)
19453	CakSNP19453	*Kabuli*	Ca_Kabuli_denovo	10086513	(A/G)
19454	CakSNP19454	*Kabuli*	Ca_Kabuli_denovo	10086534	(C/T)
19455	CakSNP19455	*Kabuli*	Ca_Kabuli_denovo	10088290	(T/C)

S.N.	SNP IDs	Cultivars	Chromosomes/scaffolds	Physical positions (bp)	SNPs
19456	CakSNP19456	*Kabuli*	Ca_Kabuli_denovo	10090741	(G/C)
19457	CakSNP19457	*Kabuli*	Ca_Kabuli_denovo	10090793	(G/A)
19458	CakSNP19458	*Kabuli*	Ca_Kabuli_denovo	10091452	(G/C)
19459	CakSNP19459	*Kabuli*	Ca_Kabuli_denovo	10091458	(C/T)
19460	CakSNP19460	*Kabuli*	Ca_Kabuli_denovo	10093966	(A/G)
19461	CakSNP19461	*Kabuli*	Ca_Kabuli_denovo	10093969	(C/A)
19462	CakSNP19462	*Kabuli*	Ca_Kabuli_denovo	10096801	(A/G)
19463	CakSNP19463	*Kabuli*	Ca_Kabuli_denovo	10096810	(C/T)
19464	CakSNP19464	*Kabuli*	Ca_Kabuli_denovo	10097986	(T/C)
19465	CakSNP19465	*Kabuli*	Ca_Kabuli_denovo	10101482	(C/T)
19466	CakSNP19466	*Kabuli*	Ca_Kabuli_denovo	10101496	(C/T)
19467	CakSNP19467	*Kabuli*	Ca_Kabuli_denovo	10103354	(T/G)
19468	CakSNP19468	*Kabuli*	Ca_Kabuli_denovo	10103399	(A/C)
19469	CakSNP19469	*Kabuli*	Ca_Kabuli_denovo	10104830	(C/T)
19470	CakSNP19470	*Kabuli*	Ca_Kabuli_denovo	10104864	(G/A)
19471	CakSNP19471	*Kabuli*	Ca_Kabuli_denovo	10109044	(C/G)
19472	CakSNP19472	*Kabuli*	Ca_Kabuli_denovo	10112518	(A/C)
19473	CakSNP19473	*Kabuli*	Ca_Kabuli_denovo	10112928	(A/C)
19474	CakSNP19474	*Kabuli*	Ca_Kabuli_denovo	10112950	(G/C)
19475	CakSNP19475	*Kabuli*	Ca_Kabuli_denovo	10117322	(C/T)
19476	CakSNP19476	*Kabuli*	Ca_Kabuli_denovo	10117331	(T/G)
19477	CakSNP19477	*Kabuli*	Ca_Kabuli_denovo	10124748	(A/G)
19478	CakSNP19478	*Kabuli*	Ca_Kabuli_denovo	10124801	(G/A)
19479	CakSNP19479	*Kabuli*	Ca_Kabuli_denovo	10129566	(G/C)
19480	CakSNP19480	*Kabuli*	Ca_Kabuli_denovo	10129575	(T/C)
19481	CakSNP19481	*Kabuli*	Ca_Kabuli_denovo	10137026	(C/G)
19482	CakSNP19482	*Kabuli*	Ca_Kabuli_denovo	10137050	(T/C)
19483	CakSNP19483	*Kabuli*	Ca_Kabuli_denovo	10138779	(A/C)
19484	CakSNP19484	*Kabuli*	Ca_Kabuli_denovo	10141306	(G/T)
19485	CakSNP19485	*Kabuli*	Ca_Kabuli_denovo	10141307	(T/C)
19486	CakSNP19486	*Kabuli*	Ca_Kabuli_denovo	10141954	(A/G)
19487	CakSNP19487	*Kabuli*	Ca_Kabuli_denovo	10142443	(C/T)
19488	CakSNP19488	*Kabuli*	Ca_Kabuli_denovo	10145154	(A/G)
19489	CakSNP19489	*Kabuli*	Ca_Kabuli_denovo	10149666	(T/C)
19490	CakSNP19490	*Kabuli*	Ca_Kabuli_denovo	10149781	(G/A)
19491	CakSNP19491	*Kabuli*	Ca_Kabuli_denovo	10149790	(A/G)
19492	CakSNP19492	*Kabuli*	Ca_Kabuli_denovo	10149841	(T/C)
19493	CakSNP19493	*Kabuli*	Ca_Kabuli_denovo	10149869	(A/C)

S.N.	SNP IDs	Cultivars	Chromosomes/scaffolds	Physical positions (bp)	SNPs
19494	CakSNP19494	*Kabuli*	Ca_Kabuli_denovo	10156177	(T/C)
19495	CakSNP19495	*Kabuli*	Ca_Kabuli_denovo	10156216	(G/C)
19496	CakSNP19496	*Kabuli*	Ca_Kabuli_denovo	10168295	(G/A)
19497	CakSNP19497	*Kabuli*	Ca_Kabuli_denovo	10168335	(A/G)
19498	CakSNP19498	*Kabuli*	Ca_Kabuli_denovo	10169966	(G/A)
19499	CakSNP19499	*Kabuli*	Ca_Kabuli_denovo	10169996	(A/G)
19500	CakSNP19500	*Kabuli*	Ca_Kabuli_denovo	10171792	(A/C)
19501	CakSNP19501	*Kabuli*	Ca_Kabuli_denovo	10171816	(A/G)
19502	CakSNP19502	*Kabuli*	Ca_Kabuli_denovo	10175611	(A/C)
19503	CakSNP19503	*Kabuli*	Ca_Kabuli_denovo	10177607	(A/G)
19504	CakSNP19504	*Kabuli*	Ca_Kabuli_denovo	10177613	(T/C)
19505	CakSNP19505	*Kabuli*	Ca_Kabuli_denovo	10178789	(G/C)
19506	CakSNP19506	*Kabuli*	Ca_Kabuli_denovo	10178804	(C/T)
19507	CakSNP19507	*Kabuli*	Ca_Kabuli_denovo	10190673	(A/G)
19508	CakSNP19508	*Kabuli*	Ca_Kabuli_denovo	10194406	(C/T)
19509	CakSNP19509	*Kabuli*	Ca_Kabuli_denovo	10194475	(A/G)
19510	CakSNP19510	*Kabuli*	Ca_Kabuli_denovo	10205609	(G/A)
19511	CakSNP19511	*Kabuli*	Ca_Kabuli_denovo	10210572	(G/T)
19512	CakSNP19512	*Kabuli*	Ca_Kabuli_denovo	10213833	(A/C)
19513	CakSNP19513	*Kabuli*	Ca_Kabuli_denovo	10217093	(C/G)
19514	CakSNP19514	*Kabuli*	Ca_Kabuli_denovo	10217104	(A/C)
19515	CakSNP19515	*Kabuli*	Ca_Kabuli_denovo	10225142	(C/T)
19516	CakSNP19516	*Kabuli*	Ca_Kabuli_denovo	10225149	(G/A)
19517	CakSNP19517	*Kabuli*	Ca_Kabuli_denovo	10228761	(A/G)
19518	CakSNP19518	*Kabuli*	Ca_Kabuli_denovo	10228762	(G/A)
19519	CakSNP19519	*Kabuli*	Ca_Kabuli_denovo	10230849	(G/T)
19520	CakSNP19520	*Kabuli*	Ca_Kabuli_denovo	10230851	(T/C)
19521	CakSNP19521	*Kabuli*	Ca_Kabuli_denovo	10236737	(C/T)
19522	CakSNP19522	*Kabuli*	Ca_Kabuli_denovo	10254467	(C/T)
19523	CakSNP19523	*Kabuli*	Ca_Kabuli_denovo	10255898	(A/G)
19524	CakSNP19524	*Kabuli*	Ca_Kabuli_denovo	10256594	(C/G)
19525	CakSNP19525	*Kabuli*	Ca_Kabuli_denovo	10257196	(A/G)
19526	CakSNP19526	*Kabuli*	Ca_Kabuli_denovo	10261646	(T/C)
19527	CakSNP19527	*Kabuli*	Ca_Kabuli_denovo	10261700	(G/C)
19528	CakSNP19528	*Kabuli*	Ca_Kabuli_denovo	10262876	(C/T)
19529	CakSNP19529	*Kabuli*	Ca_Kabuli_denovo	10263678	(A/G)
19530	CakSNP19530	*Kabuli*	Ca_Kabuli_denovo	10265917	(C/T)
19531	CakSNP19531	*Kabuli*	Ca_Kabuli_denovo	10265974	(C/A)

S.N.	SNP IDs	Cultivars	Chromosomes/scaffolds	Physical positions (bp)	SNPs
19532	CakSNP19532	*Kabuli*	Ca_Kabuli_denovo	10269550	(C/T)
19533	CakSNP19533	*Kabuli*	Ca_Kabuli_denovo	10269607	(T/G)
19534	CakSNP19534	*Kabuli*	Ca_Kabuli_denovo	10274756	(G/T)
19535	CakSNP19535	*Kabuli*	Ca_Kabuli_denovo	10274772	(T/C)
19536	CakSNP19536	*Kabuli*	Ca_Kabuli_denovo	10279030	(C/T)
19537	CakSNP19537	*Kabuli*	Ca_Kabuli_denovo	10280444	(A/G)
19538	CakSNP19538	*Kabuli*	Ca_Kabuli_denovo	10286367	(T/C)
19539	CakSNP19539	*Kabuli*	Ca_Kabuli_denovo	10288899	(T/A)
19540	CakSNP19540	*Kabuli*	Ca_Kabuli_denovo	10288969	(C/T)
19541	CakSNP19541	*Kabuli*	Ca_Kabuli_denovo	10290935	(A/G)
19542	CakSNP19542	*Kabuli*	Ca_Kabuli_denovo	10291504	(G/A)
19543	CakSNP19543	*Kabuli*	Ca_Kabuli_denovo	10291534	(C/T)
19544	CakSNP19544	*Kabuli*	Ca_Kabuli_denovo	10292621	(T/C)
19545	CakSNP19545	*Kabuli*	Ca_Kabuli_denovo	10300039	(G/A)
19546	CakSNP19546	*Kabuli*	Ca_Kabuli_denovo	10309223	(C/G)
19547	CakSNP19547	*Kabuli*	Ca_Kabuli_denovo	10313066	(G/A)
19548	CakSNP19548	*Kabuli*	Ca_Kabuli_denovo	10313087	(A/G)
19549	CakSNP19549	*Kabuli*	Ca_Kabuli_denovo	10313600	(T/C)
19550	CakSNP19550	*Kabuli*	Ca_Kabuli_denovo	10320550	(G/C)
19551	CakSNP19551	*Kabuli*	Ca_Kabuli_denovo	10320553	(C/T)
19552	CakSNP19552	*Kabuli*	Ca_Kabuli_denovo	10321987	(T/A)
19553	CakSNP19553	*Kabuli*	Ca_Kabuli_denovo	10322002	(T/C)
19554	CakSNP19554	*Kabuli*	Ca_Kabuli_denovo	10324130	(C/G)
19555	CakSNP19555	*Kabuli*	Ca_Kabuli_denovo	10324205	(G/C)
19556	CakSNP19556	*Kabuli*	Ca_Kabuli_denovo	10328878	(T/C)
19557	CakSNP19557	*Kabuli*	Ca_Kabuli_denovo	10329076	(C/T)
19558	CakSNP19558	*Kabuli*	Ca_Kabuli_denovo	10332868	(G/A)
19559	CakSNP19559	*Kabuli*	Ca_Kabuli_denovo	10336212	(C/T)
19560	CakSNP19560	*Kabuli*	Ca_Kabuli_denovo	10336214	(C/T)
19561	CakSNP19561	*Kabuli*	Ca_Kabuli_denovo	10336794	(G/C)
19562	CakSNP19562	*Kabuli*	Ca_Kabuli_denovo	10339873	(C/T)
19563	CakSNP19563	*Kabuli*	Ca_Kabuli_denovo	10340283	(T/C)
19564	CakSNP19564	*Kabuli*	Ca_Kabuli_denovo	10349134	(G/T)
19565	CakSNP19565	*Kabuli*	Ca_Kabuli_denovo	10352881	(A/C)
19566	CakSNP19566	*Kabuli*	Ca_Kabuli_denovo	10352883	(A/G)
19567	CakSNP19567	*Kabuli*	Ca_Kabuli_denovo	10359340	(T/G)
19568	CakSNP19568	*Kabuli*	Ca_Kabuli_denovo	10362271	(C/T)
19569	CakSNP19569	*Kabuli*	Ca_Kabuli_denovo	10362307	(A/C)

S.N.	SNP IDs	Cultivars	Chromosomes/scaffolds	Physical positions (bp)	SNPs
19570	CakSNP19570	*Kabuli*	Ca_Kabuli_denovo	10371987	(T/G)
19571	CakSNP19571	*Kabuli*	Ca_Kabuli_denovo	10382340	(G/C)
19572	CakSNP19572	*Kabuli*	Ca_Kabuli_denovo	10382402	(T/C)
19573	CakSNP19573	*Kabuli*	Ca_Kabuli_denovo	10386409	(C/T)
19574	CakSNP19574	*Kabuli*	Ca_Kabuli_denovo	10387229	(G/A)
19575	CakSNP19575	*Kabuli*	Ca_Kabuli_denovo	10388512	(A/G)
19576	CakSNP19576	*Kabuli*	Ca_Kabuli_denovo	10389471	(G/A)
19577	CakSNP19577	*Kabuli*	Ca_Kabuli_denovo	10397284	(C/T)
19578	CakSNP19578	*Kabuli*	Ca_Kabuli_denovo	10397306	(C/G)
19579	CakSNP19579	*Kabuli*	Ca_Kabuli_denovo	10398136	(C/T)
19580	CakSNP19580	*Kabuli*	Ca_Kabuli_denovo	10400788	(G/C)
19581	CakSNP19581	*Kabuli*	Ca_Kabuli_denovo	10404768	(A/G)
19582	CakSNP19582	*Kabuli*	Ca_Kabuli_denovo	10404818	(G/A)
19583	CakSNP19583	*Kabuli*	Ca_Kabuli_denovo	10407328	(C/G)
19584	CakSNP19584	*Kabuli*	Ca_Kabuli_denovo	10413612	(A/G)
19585	CakSNP19585	*Kabuli*	Ca_Kabuli_denovo	10415506	(T/C)
19586	CakSNP19586	*Kabuli*	Ca_Kabuli_denovo	10417123	(G/C)
19587	CakSNP19587	*Kabuli*	Ca_Kabuli_denovo	10417126	(A/G)
19588	CakSNP19588	*Kabuli*	Ca_Kabuli_denovo	10420396	(C/T)
19589	CakSNP19589	*Kabuli*	Ca_Kabuli_denovo	10420453	(T/G)
19590	CakSNP19590	*Kabuli*	Ca_Kabuli_denovo	10421757	(G/A)
19591	CakSNP19591	*Kabuli*	Ca_Kabuli_denovo	10421896	(C/A)
19592	CakSNP19592	*Kabuli*	Ca_Kabuli_denovo	10423550	(C/T)
19593	CakSNP19593	*Kabuli*	Ca_Kabuli_denovo	10423580	(T/C)
19594	CakSNP19594	*Kabuli*	Ca_Kabuli_denovo	10426889	(T/C)
19595	CakSNP19595	*Kabuli*	Ca_Kabuli_denovo	10426931	(G/A)
19596	CakSNP19596	*Kabuli*	Ca_Kabuli_denovo	10427834	(G/C)
19597	CakSNP19597	*Kabuli*	Ca_Kabuli_denovo	10427835	(C/T)
19598	CakSNP19598	*Kabuli*	Ca_Kabuli_denovo	10427855	(T/C)
19599	CakSNP19599	*Kabuli*	Ca_Kabuli_denovo	10428671	(G/A)
19600	CakSNP19600	*Kabuli*	Ca_Kabuli_denovo	10428677	(G/T)
19601	CakSNP19601	*Kabuli*	Ca_Kabuli_denovo	10433563	(T/G)
19602	CakSNP19602	*Kabuli*	Ca_Kabuli_denovo	10434056	(G/A)
19603	CakSNP19603	*Kabuli*	Ca_Kabuli_denovo	10434071	(G/T)
19604	CakSNP19604	*Kabuli*	Ca_Kabuli_denovo	10434715	(T/C)
19605	CakSNP19605	*Kabuli*	Ca_Kabuli_denovo	10438263	(A/C)
19606	CakSNP19606	*Kabuli*	Ca_Kabuli_denovo	10438309	(A/C)
19607	CakSNP19607	*Kabuli*	Ca_Kabuli_denovo	10449585	(A/T)

S.N.	SNP IDs	Cultivars	Chromosomes/scaffolds	Physical positions (bp)	SNPs
19608	CakSNP19608	*Kabuli*	Ca_Kabuli_denovo	10453372	(G/A)
19609	CakSNP19609	*Kabuli*	Ca_Kabuli_denovo	10457782	(A/G)
19610	CakSNP19610	*Kabuli*	Ca_Kabuli_denovo	10462185	(T/G)
19611	CakSNP19611	*Kabuli*	Ca_Kabuli_denovo	10463932	(A/G)
19612	CakSNP19612	*Kabuli*	Ca_Kabuli_denovo	10466745	(C/T)
19613	CakSNP19613	*Kabuli*	Ca_Kabuli_denovo	10466768	(C/T)
19614	CakSNP19614	*Kabuli*	Ca_Kabuli_denovo	10471964	(A/G)
19615	CakSNP19615	*Kabuli*	Ca_Kabuli_denovo	10474267	(A/C)
19616	CakSNP19616	*Kabuli*	Ca_Kabuli_denovo	10474993	(G/A)
19617	CakSNP19617	*Kabuli*	Ca_Kabuli_denovo	10475020	(C/G)
19618	CakSNP19618	*Kabuli*	Ca_Kabuli_denovo	10476143	(A/C)
19619	CakSNP19619	*Kabuli*	Ca_Kabuli_denovo	10478166	(G/A)
19620	CakSNP19620	*Kabuli*	Ca_Kabuli_denovo	10478171	(C/T)
19621	CakSNP19621	*Kabuli*	Ca_Kabuli_denovo	10480186	(G/A)
19622	CakSNP19622	*Kabuli*	Ca_Kabuli_denovo	10480247	(G/A)
19623	CakSNP19623	*Kabuli*	Ca_Kabuli_denovo	10480562	(A/G)
19624	CakSNP19624	*Kabuli*	Ca_Kabuli_denovo	10487489	(C/T)
19625	CakSNP19625	*Kabuli*	Ca_Kabuli_denovo	10487501	(C/T)
19626	CakSNP19626	*Kabuli*	Ca_Kabuli_denovo	10487719	(C/A)
19627	CakSNP19627	*Kabuli*	Ca_Kabuli_denovo	10488191	(G/A)
19628	CakSNP19628	*Kabuli*	Ca_Kabuli_denovo	10488218	(T/C)
19629	CakSNP19629	*Kabuli*	Ca_Kabuli_denovo	10490833	(T/G)
19630	CakSNP19630	*Kabuli*	Ca_Kabuli_denovo	10490857	(C/T)
19631	CakSNP19631	*Kabuli*	Ca_Kabuli_denovo	10491343	(G/A)
19632	CakSNP19632	*Kabuli*	Ca_Kabuli_denovo	10491421	(A/C)
19633	CakSNP19633	*Kabuli*	Ca_Kabuli_denovo	10504586	(T/A)
19634	CakSNP19634	*Kabuli*	Ca_Kabuli_denovo	10509436	(C/T)
19635	CakSNP19635	*Kabuli*	Ca_Kabuli_denovo	10520172	(A/G)
19636	CakSNP19636	*Kabuli*	Ca_Kabuli_denovo	10521045	(C/T)
19637	CakSNP19637	*Kabuli*	Ca_Kabuli_denovo	10522423	(C/T)
19638	CakSNP19638	*Kabuli*	Ca_Kabuli_denovo	10526136	(T/C)
19639	CakSNP19639	*Kabuli*	Ca_Kabuli_denovo	10526145	(C/T)
19640	CakSNP19640	*Kabuli*	Ca_Kabuli_denovo	10527066	(C/A)
19641	CakSNP19641	*Kabuli*	Ca_Kabuli_denovo	10528013	(A/G)
19642	CakSNP19642	*Kabuli*	Ca_Kabuli_denovo	10538248	(G/A)
19643	CakSNP19643	*Kabuli*	Ca_Kabuli_denovo	10544632	(A/G)
19644	CakSNP19644	*Kabuli*	Ca_Kabuli_denovo	10546885	(A/G)
19645	CakSNP19645	*Kabuli*	Ca_Kabuli_denovo	10555780	(C/A)

S.N.	SNP IDs	Cultivars	Chromosomes/scaffolds	Physical positions (bp)	SNPs
19646	CakSNP19646	*Kabuli*	Ca_Kabuli_denovo	10558499	(C/T)
19647	CakSNP19647	*Kabuli*	Ca_Kabuli_denovo	10558812	(T/C)
19648	CakSNP19648	*Kabuli*	Ca_Kabuli_denovo	10559975	(A/G)
19649	CakSNP19649	*Kabuli*	Ca_Kabuli_denovo	10563440	(G/A)
19650	CakSNP19650	*Kabuli*	Ca_Kabuli_denovo	10566203	(C/G)
19651	CakSNP19651	*Kabuli*	Ca_Kabuli_denovo	10580621	(G/A)
19652	CakSNP19652	*Kabuli*	Ca_Kabuli_denovo	10583596	(G/A)
19653	CakSNP19653	*Kabuli*	Ca_Kabuli_denovo	10586396	(G/A)
19654	CakSNP19654	*Kabuli*	Ca_Kabuli_denovo	10586476	(A/G)
19655	CakSNP19655	*Kabuli*	Ca_Kabuli_denovo	10587347	(T/C)
19656	CakSNP19656	*Kabuli*	Ca_Kabuli_denovo	10587505	(C/G)
19657	CakSNP19657	*Kabuli*	Ca_Kabuli_denovo	10597388	(C/G)
19658	CakSNP19658	*Kabuli*	Ca_Kabuli_denovo	10597948	(T/C)
19659	CakSNP19659	*Kabuli*	Ca_Kabuli_denovo	10601860	(C/T)
19660	CakSNP19660	*Kabuli*	Ca_Kabuli_denovo	10602584	(A/G)
19661	CakSNP19661	*Kabuli*	Ca_Kabuli_denovo	10604019	(C/T)
19662	CakSNP19662	*Kabuli*	Ca_Kabuli_denovo	10608370	(C/T)
19663	CakSNP19663	*Kabuli*	Ca_Kabuli_denovo	10609543	(G/A)
19664	CakSNP19664	*Kabuli*	Ca_Kabuli_denovo	10612490	(G/A)
19665	CakSNP19665	*Kabuli*	Ca_Kabuli_denovo	10613546	(T/G)
19666	CakSNP19666	*Kabuli*	Ca_Kabuli_denovo	10614294	(C/T)
19667	CakSNP19667	*Kabuli*	Ca_Kabuli_denovo	10614905	(G/A)
19668	CakSNP19668	*Kabuli*	Ca_Kabuli_denovo	10619587	(G/A)
19669	CakSNP19669	*Kabuli*	Ca_Kabuli_denovo	10623248	(A/G)
19670	CakSNP19670	*Kabuli*	Ca_Kabuli_denovo	10625948	(C/A)
19671	CakSNP19671	*Kabuli*	Ca_Kabuli_denovo	10625976	(A/T)
19672	CakSNP19672	*Kabuli*	Ca_Kabuli_denovo	10633747	(C/T)
19673	CakSNP19673	*Kabuli*	Ca_Kabuli_denovo	10634473	(A/G)
19674	CakSNP19674	*Kabuli*	Ca_Kabuli_denovo	10634539	(C/G)
19675	CakSNP19675	*Kabuli*	Ca_Kabuli_denovo	10635906	(G/A)
19676	CakSNP19676	*Kabuli*	Ca_Kabuli_denovo	10636722	(T/G)
19677	CakSNP19677	*Kabuli*	Ca_Kabuli_denovo	10636753	(T/C)
19678	CakSNP19678	*Kabuli*	Ca_Kabuli_denovo	10638379	(C/T)
19679	CakSNP19679	*Kabuli*	Ca_Kabuli_denovo	10638416	(C/T)
19680	CakSNP19680	*Kabuli*	Ca_Kabuli_denovo	10639123	(T/C)
19681	CakSNP19681	*Kabuli*	Ca_Kabuli_denovo	10639199	(A/C)
19682	CakSNP19682	*Kabuli*	Ca_Kabuli_denovo	10639200	(G/T)
19683	CakSNP19683	*Kabuli*	Ca_Kabuli_denovo	10639201	(A/C)

S.N.	SNP IDs	Cultivars	Chromosomes/scaffolds	Physical positions (bp)	SNPs
19684	CakSNP19684	*Kabuli*	Ca_Kabuli_denovo	10645075	(G/A)
19685	CakSNP19685	*Kabuli*	Ca_Kabuli_denovo	10648894	(A/C)
19686	CakSNP19686	*Kabuli*	Ca_Kabuli_denovo	10648964	(A/G)
19687	CakSNP19687	*Kabuli*	Ca_Kabuli_denovo	10651838	(C/T)
19688	CakSNP19688	*Kabuli*	Ca_Kabuli_denovo	10652481	(A/G)
19689	CakSNP19689	*Kabuli*	Ca_Kabuli_denovo	10652486	(T/C)
19690	CakSNP19690	*Kabuli*	Ca_Kabuli_denovo	10660520	(G/A)
19691	CakSNP19691	*Kabuli*	Ca_Kabuli_denovo	10675025	(A/G)
19692	CakSNP19692	*Kabuli*	Ca_Kabuli_denovo	10675052	(G/A)
19693	CakSNP19693	*Kabuli*	Ca_Kabuli_denovo	10675879	(A/G)
19694	CakSNP19694	*Kabuli*	Ca_Kabuli_denovo	10682359	(A/G)
19695	CakSNP19695	*Kabuli*	Ca_Kabuli_denovo	10685319	(T/A)
19696	CakSNP19696	*Kabuli*	Ca_Kabuli_denovo	10690207	(C/A)
19697	CakSNP19697	*Kabuli*	Ca_Kabuli_denovo	10690236	(T/C)
19698	CakSNP19698	*Kabuli*	Ca_Kabuli_denovo	10704771	(C/T)
19699	CakSNP19699	*Kabuli*	Ca_Kabuli_denovo	10704772	(C/T)
19700	CakSNP19700	*Kabuli*	Ca_Kabuli_denovo	10709211	(G/A)
19701	CakSNP19701	*Kabuli*	Ca_Kabuli_denovo	10709212	(C/T)
19702	CakSNP19702	*Kabuli*	Ca_Kabuli_denovo	10711513	(C/T)
19703	CakSNP19703	*Kabuli*	Ca_Kabuli_denovo	10717240	(C/T)
19704	CakSNP19704	*Kabuli*	Ca_Kabuli_denovo	10725261	(T/C)
19705	CakSNP19705	*Kabuli*	Ca_Kabuli_denovo	10725280	(A/G)
19706	CakSNP19706	*Kabuli*	Ca_Kabuli_denovo	10726837	(T/G)
19707	CakSNP19707	*Kabuli*	Ca_Kabuli_denovo	10726857	(A/G)
19708	CakSNP19708	*Kabuli*	Ca_Kabuli_denovo	10727523	(C/T)
19709	CakSNP19709	*Kabuli*	Ca_Kabuli_denovo	10737510	(A/C)
19710	CakSNP19710	*Kabuli*	Ca_Kabuli_denovo	10737559	(A/G)
19711	CakSNP19711	*Kabuli*	Ca_Kabuli_denovo	10744775	(G/A)
19712	CakSNP19712	*Kabuli*	Ca_Kabuli_denovo	10748089	(T/C)
19713	CakSNP19713	*Kabuli*	Ca_Kabuli_denovo	10748469	(T/G)
19714	CakSNP19714	*Kabuli*	Ca_Kabuli_denovo	10750888	(A/G)
19715	CakSNP19715	*Kabuli*	Ca_Kabuli_denovo	10750911	(T/C)
19716	CakSNP19716	*Kabuli*	Ca_Kabuli_denovo	10750941	(G/A)
19717	CakSNP19717	*Kabuli*	Ca_Kabuli_denovo	10765332	(C/T)
19718	CakSNP19718	*Kabuli*	Ca_Kabuli_denovo	10774154	(C/A)
19719	CakSNP19719	*Kabuli*	Ca_Kabuli_denovo	10774193	(A/G)
19720	CakSNP19720	*Kabuli*	Ca_Kabuli_denovo	10779799	(C/G)
19721	CakSNP19721	*Kabuli*	Ca_Kabuli_denovo	10779817	(C/T)

S.N.	SNP IDs	Cultivars	Chromosomes/scaffolds	Physical positions (bp)	SNPs
19722	CakSNP19722	*Kabuli*	Ca_Kabuli_denovo	10784206	(C/G)
19723	CakSNP19723	*Kabuli*	Ca_Kabuli_denovo	10785716	(C/G)
19724	CakSNP19724	*Kabuli*	Ca_Kabuli_denovo	10793538	(C/T)
19725	CakSNP19725	*Kabuli*	Ca_Kabuli_denovo	10801968	(C/G)
19726	CakSNP19726	*Kabuli*	Ca_Kabuli_denovo	10802297	(T/C)
19727	CakSNP19727	*Kabuli*	Ca_Kabuli_denovo	10802312	(A/G)
19728	CakSNP19728	*Kabuli*	Ca_Kabuli_denovo	10804689	(A/C)
19729	CakSNP19729	*Kabuli*	Ca_Kabuli_denovo	10804725	(A/C)
19730	CakSNP19730	*Kabuli*	Ca_Kabuli_denovo	10815305	(C/T)
19731	CakSNP19731	*Kabuli*	Ca_Kabuli_denovo	10816024	(A/G)
19732	CakSNP19732	*Kabuli*	Ca_Kabuli_denovo	10820299	(T/C)
19733	CakSNP19733	*Kabuli*	Ca_Kabuli_denovo	10820305	(T/G)
19734	CakSNP19734	*Kabuli*	Ca_Kabuli_denovo	10827752	(T/C)
19735	CakSNP19735	*Kabuli*	Ca_Kabuli_denovo	10827766	(C/G)
19736	CakSNP19736	*Kabuli*	Ca_Kabuli_denovo	10829958	(G/C)
19737	CakSNP19737	*Kabuli*	Ca_Kabuli_denovo	10830004	(C/T)
19738	CakSNP19738	*Kabuli*	Ca_Kabuli_denovo	10836808	(T/G)
19739	CakSNP19739	*Kabuli*	Ca_Kabuli_denovo	10837882	(A/G)
19740	CakSNP19740	*Kabuli*	Ca_Kabuli_denovo	10837892	(A/C)
19741	CakSNP19741	*Kabuli*	Ca_Kabuli_denovo	10843751	(G/A)
19742	CakSNP19742	*Kabuli*	Ca_Kabuli_denovo	10852994	(G/T)
19743	CakSNP19743	*Kabuli*	Ca_Kabuli_denovo	10853440	(C/A)
19744	CakSNP19744	*Kabuli*	Ca_Kabuli_denovo	10855184	(A/C)
19745	CakSNP19745	*Kabuli*	Ca_Kabuli_denovo	10859383	(A/G)
19746	CakSNP19746	*Kabuli*	Ca_Kabuli_denovo	10859422	(C/T)
19747	CakSNP19747	*Kabuli*	Ca_Kabuli_denovo	10863716	(A/C)
19748	CakSNP19748	*Kabuli*	Ca_Kabuli_denovo	10863767	(C/T)
19749	CakSNP19749	*Kabuli*	Ca_Kabuli_denovo	10867451	(C/A)
19750	CakSNP19750	*Kabuli*	Ca_Kabuli_denovo	10868177	(C/T)
19751	CakSNP19751	*Kabuli*	Ca_Kabuli_denovo	10871629	(C/T)
19752	CakSNP19752	*Kabuli*	Ca_Kabuli_denovo	10871630	(G/A)
19753	CakSNP19753	*Kabuli*	Ca_Kabuli_denovo	10872857	(C/A)
19754	CakSNP19754	*Kabuli*	Ca_Kabuli_denovo	10872867	(T/G)
19755	CakSNP19755	*Kabuli*	Ca_Kabuli_denovo	10873672	(G/C)
19756	CakSNP19756	*Kabuli*	Ca_Kabuli_denovo	10879509	(A/G)
19757	CakSNP19757	*Kabuli*	Ca_Kabuli_denovo	10880917	(G/C)
19758	CakSNP19758	*Kabuli*	Ca_Kabuli_denovo	10882337	(C/G)
19759	CakSNP19759	*Kabuli*	Ca_Kabuli_denovo	10884357	(A/G)

S.N.	SNP IDs	Cultivars	Chromosomes/scaffolds	Physical positions (bp)	SNPs
19760	CakSNP19760	*Kabuli*	Ca_Kabuli_denovo	10884407	(A/T)
19761	CakSNP19761	*Kabuli*	Ca_Kabuli_denovo	10894351	(C/A)
19762	CakSNP19762	*Kabuli*	Ca_Kabuli_denovo	10894895	(A/G)
19763	CakSNP19763	*Kabuli*	Ca_Kabuli_denovo	10894913	(T/C)
19764	CakSNP19764	*Kabuli*	Ca_Kabuli_denovo	10897663	(C/T)
19765	CakSNP19765	*Kabuli*	Ca_Kabuli_denovo	10905659	(A/G)
19766	CakSNP19766	*Kabuli*	Ca_Kabuli_denovo	10929918	(A/T)
19767	CakSNP19767	*Kabuli*	Ca_Kabuli_denovo	10930163	(G/T)
19768	CakSNP19768	*Kabuli*	Ca_Kabuli_denovo	10935880	(C/T)
19769	CakSNP19769	*Kabuli*	Ca_Kabuli_denovo	10935937	(A/C)
19770	CakSNP19770	*Kabuli*	Ca_Kabuli_denovo	10936993	(A/G)
19771	CakSNP19771	*Kabuli*	Ca_Kabuli_denovo	10942394	(A/G)
19772	CakSNP19772	*Kabuli*	Ca_Kabuli_denovo	10942431	(A/G)
19773	CakSNP19773	*Kabuli*	Ca_Kabuli_denovo	10943375	(T/C)
19774	CakSNP19774	*Kabuli*	Ca_Kabuli_denovo	10943443	(C/T)
19775	CakSNP19775	*Kabuli*	Ca_Kabuli_denovo	10945287	(G/A)
19776	CakSNP19776	*Kabuli*	Ca_Kabuli_denovo	10945318	(G/C)
19777	CakSNP19777	*Kabuli*	Ca_Kabuli_denovo	10951393	(G/T)
19778	CakSNP19778	*Kabuli*	Ca_Kabuli_denovo	10951395	(A/G)
19779	CakSNP19779	*Kabuli*	Ca_Kabuli_denovo	10957310	(C/T)
19780	CakSNP19780	*Kabuli*	Ca_Kabuli_denovo	10958307	(C/T)
19781	CakSNP19781	*Kabuli*	Ca_Kabuli_denovo	10958312	(C/A)
19782	CakSNP19782	*Kabuli*	Ca_Kabuli_denovo	10959965	(C/G)
19783	CakSNP19783	*Kabuli*	Ca_Kabuli_denovo	10960754	(G/A)
19784	CakSNP19784	*Kabuli*	Ca_Kabuli_denovo	10964203	(C/T)
19785	CakSNP19785	*Kabuli*	Ca_Kabuli_denovo	10964227	(G/A)
19786	CakSNP19786	*Kabuli*	Ca_Kabuli_denovo	10971033	(G/A)
19787	CakSNP19787	*Kabuli*	Ca_Kabuli_denovo	10971053	(G/C)
19788	CakSNP19788	*Kabuli*	Ca_Kabuli_denovo	10977079	(A/C)
19789	CakSNP19789	*Kabuli*	Ca_Kabuli_denovo	10978239	(A/G)
19790	CakSNP19790	*Kabuli*	Ca_Kabuli_denovo	10978244	(G/A)
19791	CakSNP19791	*Kabuli*	Ca_Kabuli_denovo	10978256	(C/G)
19792	CakSNP19792	*Kabuli*	Ca_Kabuli_denovo	10985386	(T/C)
19793	CakSNP19793	*Kabuli*	Ca_Kabuli_denovo	10985566	(A/G)
19794	CakSNP19794	*Kabuli*	Ca_Kabuli_denovo	10985567	(T/C)
19795	CakSNP19795	*Kabuli*	Ca_Kabuli_denovo	10986704	(C/G)
19796	CakSNP19796	*Kabuli*	Ca_Kabuli_denovo	10986710	(C/G)
19797	CakSNP19797	*Kabuli*	Ca_Kabuli_denovo	10987146	(T/G)

S.N.	SNP IDs	Cultivars	Chromosomes/scaffolds	Physical positions (bp)	SNPs
19798	CakSNP19798	*Kabuli*	Ca_Kabuli_denovo	10987152	(T/A)
19799	CakSNP19799	*Kabuli*	Ca_Kabuli_denovo	10989989	(T/G)
19800	CakSNP19800	*Kabuli*	Ca_Kabuli_denovo	10994002	(C/T)
19801	CakSNP19801	*Kabuli*	Ca_Kabuli_denovo	10994032	(T/C)
19802	CakSNP19802	*Kabuli*	Ca_Kabuli_denovo	10996772	(C/T)
19803	CakSNP19803	*Kabuli*	Ca_Kabuli_denovo	11000849	(G/C)
19804	CakSNP19804	*Kabuli*	Ca_Kabuli_denovo	11000856	(C/G)
19805	CakSNP19805	*Kabuli*	Ca_Kabuli_denovo	11009676	(C/T)
19806	CakSNP19806	*Kabuli*	Ca_Kabuli_denovo	11015742	(G/A)
19807	CakSNP19807	*Kabuli*	Ca_Kabuli_denovo	11018664	(C/A)
19808	CakSNP19808	*Kabuli*	Ca_Kabuli_denovo	11018718	(C/G)
19809	CakSNP19809	*Kabuli*	Ca_Kabuli_denovo	11022673	(C/G)
19810	CakSNP19810	*Kabuli*	Ca_Kabuli_denovo	11022685	(T/C)
19811	CakSNP19811	*Kabuli*	Ca_Kabuli_denovo	11022766	(C/T)
19812	CakSNP19812	*Kabuli*	Ca_Kabuli_denovo	11025360	(A/C)
19813	CakSNP19813	*Kabuli*	Ca_Kabuli_denovo	11025765	(C/G)
19814	CakSNP19814	*Kabuli*	Ca_Kabuli_denovo	11035425	(C/T)
19815	CakSNP19815	*Kabuli*	Ca_Kabuli_denovo	11035455	(C/G)
19816	CakSNP19816	*Kabuli*	Ca_Kabuli_denovo	11038835	(C/G)
19817	CakSNP19817	*Kabuli*	Ca_Kabuli_denovo	11038859	(T/C)
19818	CakSNP19818	*Kabuli*	Ca_Kabuli_denovo	11041618	(G/A)
19819	CakSNP19819	*Kabuli*	Ca_Kabuli_denovo	11041631	(A/G)
19820	CakSNP19820	*Kabuli*	Ca_Kabuli_denovo	11049997	(A/G)
19821	CakSNP19821	*Kabuli*	Ca_Kabuli_denovo	11050024	(G/A)
19822	CakSNP19822	*Kabuli*	Ca_Kabuli_denovo	11052623	(T/G)
19823	CakSNP19823	*Kabuli*	Ca_Kabuli_denovo	11052626	(T/C)
19824	CakSNP19824	*Kabuli*	Ca_Kabuli_denovo	11059845	(G/A)
19825	CakSNP19825	*Kabuli*	Ca_Kabuli_denovo	11060901	(G/C)
19826	CakSNP19826	*Kabuli*	Ca_Kabuli_denovo	11063106	(T/A)
19827	CakSNP19827	*Kabuli*	Ca_Kabuli_denovo	11078845	(G/C)
19828	CakSNP19828	*Kabuli*	Ca_Kabuli_denovo	11081534	(T/C)
19829	CakSNP19829	*Kabuli*	Ca_Kabuli_denovo	11081662	(C/T)
19830	CakSNP19830	*Kabuli*	Ca_Kabuli_denovo	11083492	(G/A)
19831	CakSNP19831	*Kabuli*	Ca_Kabuli_denovo	11083548	(G/A)
19832	CakSNP19832	*Kabuli*	Ca_Kabuli_denovo	11086369	(G/A)
19833	CakSNP19833	*Kabuli*	Ca_Kabuli_denovo	11099095	(G/A)
19834	CakSNP19834	*Kabuli*	Ca_Kabuli_denovo	11101450	(A/G)
19835	CakSNP19835	*Kabuli*	Ca_Kabuli_denovo	11101462	(T/C)

S.N.	SNP IDs	Cultivars	Chromosomes/scaffolds	Physical positions (bp)	SNPs
19836	CakSNP19836	*Kabuli*	Ca_Kabuli_denovo	11114537	(G/C)
19837	CakSNP19837	*Kabuli*	Ca_Kabuli_denovo	11116590	(A/G)
19838	CakSNP19838	*Kabuli*	Ca_Kabuli_denovo	11116620	(T/C)
19839	CakSNP19839	*Kabuli*	Ca_Kabuli_denovo	11117655	(A/C)
19840	CakSNP19840	*Kabuli*	Ca_Kabuli_denovo	11127124	(T/C)
19841	CakSNP19841	*Kabuli*	Ca_Kabuli_denovo	11129839	(G/A)
19842	CakSNP19842	*Kabuli*	Ca_Kabuli_denovo	11132036	(G/C)
19843	CakSNP19843	*Kabuli*	Ca_Kabuli_denovo	11136790	(A/G)
19844	CakSNP19844	*Kabuli*	Ca_Kabuli_denovo	11136841	(C/T)
19845	CakSNP19845	*Kabuli*	Ca_Kabuli_denovo	11137317	(G/A)
19846	CakSNP19846	*Kabuli*	Ca_Kabuli_denovo	11137921	(G/T)
19847	CakSNP19847	*Kabuli*	Ca_Kabuli_denovo	11143568	(A/G)
19848	CakSNP19848	*Kabuli*	Ca_Kabuli_denovo	11144773	(A/G)
19849	CakSNP19849	*Kabuli*	Ca_Kabuli_denovo	11144842	(A/C)
19850	CakSNP19850	*Kabuli*	Ca_Kabuli_denovo	11146079	(C/A)
19851	CakSNP19851	*Kabuli*	Ca_Kabuli_denovo	11161889	(G/T)
19852	CakSNP19852	*Kabuli*	Ca_Kabuli_denovo	11162483	(G/T)
19853	CakSNP19853	*Kabuli*	Ca_Kabuli_denovo	11162500	(A/G)
19854	CakSNP19854	*Kabuli*	Ca_Kabuli_denovo	11165328	(A/G)
19855	CakSNP19855	*Kabuli*	Ca_Kabuli_denovo	11165347	(A/C)
19856	CakSNP19856	*Kabuli*	Ca_Kabuli_denovo	11171823	(G/T)
19857	CakSNP19857	*Kabuli*	Ca_Kabuli_denovo	11172625	(A/G)
19858	CakSNP19858	*Kabuli*	Ca_Kabuli_denovo	11172634	(A/G)
19859	CakSNP19859	*Kabuli*	Ca_Kabuli_denovo	11174544	(G/T)
19860	CakSNP19860	*Kabuli*	Ca_Kabuli_denovo	11174567	(A/G)
19861	CakSNP19861	*Kabuli*	Ca_Kabuli_denovo	11177330	(C/G)
19862	CakSNP19862	*Kabuli*	Ca_Kabuli_denovo	11185106	(C/G)
19863	CakSNP19863	*Kabuli*	Ca_Kabuli_denovo	11188583	(G/C)
19864	CakSNP19864	*Kabuli*	Ca_Kabuli_denovo	11193535	(C/G)
19865	CakSNP19865	*Kabuli*	Ca_Kabuli_denovo	11193573	(G/A)
19866	CakSNP19866	*Kabuli*	Ca_Kabuli_denovo	11195851	(G/T)
19867	CakSNP19867	*Kabuli*	Ca_Kabuli_denovo	11204126	(C/T)
19868	CakSNP19868	*Kabuli*	Ca_Kabuli_denovo	11204171	(A/C)
19869	CakSNP19869	*Kabuli*	Ca_Kabuli_denovo	11205211	(G/A)
19870	CakSNP19870	*Kabuli*	Ca_Kabuli_denovo	11205220	(C/A)
19871	CakSNP19871	*Kabuli*	Ca_Kabuli_denovo	11205976	(A/G)
19872	CakSNP19872	*Kabuli*	Ca_Kabuli_denovo	11205991	(C/T)
19873	CakSNP19873	*Kabuli*	Ca_Kabuli_denovo	11207799	(T/C)

S.N.	SNP IDs	Cultivars	Chromosomes/scaffolds	Physical positions (bp)	SNPs
19874	CakSNP19874	*Kabuli*	Ca_Kabuli_denovo	11210096	(T/C)
19875	CakSNP19875	*Kabuli*	Ca_Kabuli_denovo	11212029	(T/C)
19876	CakSNP19876	*Kabuli*	Ca_Kabuli_denovo	11212077	(A/G)
19877	CakSNP19877	*Kabuli*	Ca_Kabuli_denovo	11212273	(G/C)
19878	CakSNP19878	*Kabuli*	Ca_Kabuli_denovo	11212276	(A/G)
19879	CakSNP19879	*Kabuli*	Ca_Kabuli_denovo	11226427	(A/G)
19880	CakSNP19880	*Kabuli*	Ca_Kabuli_denovo	11226478	(C/G)
19881	CakSNP19881	*Kabuli*	Ca_Kabuli_denovo	11232178	(C/A)
19882	CakSNP19882	*Kabuli*	Ca_Kabuli_denovo	11232208	(T/C)
19883	CakSNP19883	*Kabuli*	Ca_Kabuli_denovo	11235535	(T/C)
19884	CakSNP19884	*Kabuli*	Ca_Kabuli_denovo	11238073	(G/A)
19885	CakSNP19885	*Kabuli*	Ca_Kabuli_denovo	11238076	(C/G)
19886	CakSNP19886	*Kabuli*	Ca_Kabuli_denovo	11243846	(C/T)
19887	CakSNP19887	*Kabuli*	Ca_Kabuli_denovo	11247995	(G/A)
19888	CakSNP19888	*Kabuli*	Ca_Kabuli_denovo	11251636	(A/G)
19889	CakSNP19889	*Kabuli*	Ca_Kabuli_denovo	11253863	(C/T)
19890	CakSNP19890	*Kabuli*	Ca_Kabuli_denovo	11253937	(C/T)
19891	CakSNP19891	*Kabuli*	Ca_Kabuli_denovo	11253941	(G/T)
19892	CakSNP19892	*Kabuli*	Ca_Kabuli_denovo	11254529	(C/A)
19893	CakSNP19893	*Kabuli*	Ca_Kabuli_denovo	11254535	(G/C)
19894	CakSNP19894	*Kabuli*	Ca_Kabuli_denovo	11257861	(A/G)
19895	CakSNP19895	*Kabuli*	Ca_Kabuli_denovo	11257890	(T/C)
19896	CakSNP19896	*Kabuli*	Ca_Kabuli_denovo	11260640	(C/T)
19897	CakSNP19897	*Kabuli*	Ca_Kabuli_denovo	11261285	(T/G)
19898	CakSNP19898	*Kabuli*	Ca_Kabuli_denovo	11263910	(C/T)
19899	CakSNP19899	*Kabuli*	Ca_Kabuli_denovo	11264488	(A/G)
19900	CakSNP19900	*Kabuli*	Ca_Kabuli_denovo	11266340	(C/G)
19901	CakSNP19901	*Kabuli*	Ca_Kabuli_denovo	11268597	(A/G)
19902	CakSNP19902	*Kabuli*	Ca_Kabuli_denovo	11269385	(C/A)
19903	CakSNP19903	*Kabuli*	Ca_Kabuli_denovo	11274782	(G/A)
19904	CakSNP19904	*Kabuli*	Ca_Kabuli_denovo	11274830	(A/G)
19905	CakSNP19905	*Kabuli*	Ca_Kabuli_denovo	11276474	(C/T)
19906	CakSNP19906	*Kabuli*	Ca_Kabuli_denovo	11281562	(A/G)
19907	CakSNP19907	*Kabuli*	Ca_Kabuli_denovo	11282591	(A/T)
19908	CakSNP19908	*Kabuli*	Ca_Kabuli_denovo	11284197	(C/T)
19909	CakSNP19909	*Kabuli*	Ca_Kabuli_denovo	11284218	(A/G)
19910	CakSNP19910	*Kabuli*	Ca_Kabuli_denovo	11294318	(T/C)
19911	CakSNP19911	*Kabuli*	Ca_Kabuli_denovo	11294360	(T/C)

S.N.	SNP IDs	Cultivars	Chromosomes/scaffolds	Physical positions (bp)	SNPs
19912	CakSNP19912	*Kabuli*	Ca_Kabuli_denovo	11298034	(A/G)
19913	CakSNP19913	*Kabuli*	Ca_Kabuli_denovo	11302009	(A/G)
19914	CakSNP19914	*Kabuli*	Ca_Kabuli_denovo	11303066	(G/A)
19915	CakSNP19915	*Kabuli*	Ca_Kabuli_denovo	11303067	(C/G)
19916	CakSNP19916	*Kabuli*	Ca_Kabuli_denovo	11303075	(A/G)
19917	CakSNP19917	*Kabuli*	Ca_Kabuli_denovo	11305242	(C/G)
19918	CakSNP19918	*Kabuli*	Ca_Kabuli_denovo	11311346	(G/A)
19919	CakSNP19919	*Kabuli*	Ca_Kabuli_denovo	11311355	(C/G)
19920	CakSNP19920	*Kabuli*	Ca_Kabuli_denovo	11313010	(G/A)
19921	CakSNP19921	*Kabuli*	Ca_Kabuli_denovo	11313019	(G/T)
19922	CakSNP19922	*Kabuli*	Ca_Kabuli_denovo	11320821	(C/T)
19923	CakSNP19923	*Kabuli*	Ca_Kabuli_denovo	11320852	(C/T)
19924	CakSNP19924	*Kabuli*	Ca_Kabuli_denovo	11320859	(C/G)
19925	CakSNP19925	*Kabuli*	Ca_Kabuli_denovo	11321893	(C/A)
19926	CakSNP19926	*Kabuli*	Ca_Kabuli_denovo	11322134	(A/G)
19927	CakSNP19927	*Kabuli*	Ca_Kabuli_denovo	11322171	(A/G)
19928	CakSNP19928	*Kabuli*	Ca_Kabuli_denovo	11322424	(G/A)
19929	CakSNP19929	*Kabuli*	Ca_Kabuli_denovo	11328453	(T/C)
19930	CakSNP19930	*Kabuli*	Ca_Kabuli_denovo	11328457	(A/G)
19931	CakSNP19931	*Kabuli*	Ca_Kabuli_denovo	11330932	(A/C)
19932	CakSNP19932	*Kabuli*	Ca_Kabuli_denovo	11335776	(T/C)
19933	CakSNP19933	*Kabuli*	Ca_Kabuli_denovo	11335791	(T/C)
19934	CakSNP19934	*Kabuli*	Ca_Kabuli_denovo	11336858	(T/C)
19935	CakSNP19935	*Kabuli*	Ca_Kabuli_denovo	11337191	(G/A)
19936	CakSNP19936	*Kabuli*	Ca_Kabuli_denovo	11338105	(G/C)
19937	CakSNP19937	*Kabuli*	Ca_Kabuli_denovo	11338164	(A/T)
19938	CakSNP19938	*Kabuli*	Ca_Kabuli_denovo	11338385	(G/A)
19939	CakSNP19939	*Kabuli*	Ca_Kabuli_denovo	11338391	(C/G)
19940	CakSNP19940	*Kabuli*	Ca_Kabuli_denovo	11346296	(G/T)
19941	CakSNP19941	*Kabuli*	Ca_Kabuli_denovo	11353664	(T/C)
19942	CakSNP19942	*Kabuli*	Ca_Kabuli_denovo	11361169	(G/C)
19943	CakSNP19943	*Kabuli*	Ca_Kabuli_denovo	11361193	(C/A)
19944	CakSNP19944	*Kabuli*	Ca_Kabuli_denovo	11361211	(A/G)
19945	CakSNP19945	*Kabuli*	Ca_Kabuli_denovo	11361500	(G/C)
19946	CakSNP19946	*Kabuli*	Ca_Kabuli_denovo	11361520	(C/T)
19947	CakSNP19947	*Kabuli*	Ca_Kabuli_denovo	11375757	(C/T)
19948	CakSNP19948	*Kabuli*	Ca_Kabuli_denovo	11378866	(C/T)
19949	CakSNP19949	*Kabuli*	Ca_Kabuli_denovo	11378886	(G/C)

S.N.	SNP IDs	Cultivars	Chromosomes/scaffolds	Physical positions (bp)	SNPs
19950	CakSNP19950	*Kabuli*	Ca_Kabuli_denovo	11386105	(C/T)
19951	CakSNP19951	*Kabuli*	Ca_Kabuli_denovo	11386154	(C/G)
19952	CakSNP19952	*Kabuli*	Ca_Kabuli_denovo	11389200	(G/C)
19953	CakSNP19953	*Kabuli*	Ca_Kabuli_denovo	11393292	(C/T)
19954	CakSNP19954	*Kabuli*	Ca_Kabuli_denovo	11400208	(C/G)
19955	CakSNP19955	*Kabuli*	Ca_Kabuli_denovo	11400582	(C/A)
19956	CakSNP19956	*Kabuli*	Ca_Kabuli_denovo	11402566	(C/T)
19957	CakSNP19957	*Kabuli*	Ca_Kabuli_denovo	11403286	(A/G)
19958	CakSNP19958	*Kabuli*	Ca_Kabuli_denovo	11407953	(A/G)
19959	CakSNP19959	*Kabuli*	Ca_Kabuli_denovo	11416225	(A/G)
19960	CakSNP19960	*Kabuli*	Ca_Kabuli_denovo	11430755	(A/G)
19961	CakSNP19961	*Kabuli*	Ca_Kabuli_denovo	11434250	(T/G)
19962	CakSNP19962	*Kabuli*	Ca_Kabuli_denovo	11441548	(G/C)
19963	CakSNP19963	*Kabuli*	Ca_Kabuli_denovo	11441557	(T/C)
19964	CakSNP19964	*Kabuli*	Ca_Kabuli_denovo	11452594	(T/C)
19965	CakSNP19965	*Kabuli*	Ca_Kabuli_denovo	11452603	(A/G)
19966	CakSNP19966	*Kabuli*	Ca_Kabuli_denovo	11455379	(G/T)
19967	CakSNP19967	*Kabuli*	Ca_Kabuli_denovo	11455475	(T/C)
19968	CakSNP19968	*Kabuli*	Ca_Kabuli_denovo	11455484	(C/G)
19969	CakSNP19969	*Kabuli*	Ca_Kabuli_denovo	11467373	(G/A)
19970	CakSNP19970	*Kabuli*	Ca_Kabuli_denovo	11483928	(G/A)
19971	CakSNP19971	*Kabuli*	Ca_Kabuli_denovo	11485151	(G/A)
19972	CakSNP19972	*Kabuli*	Ca_Kabuli_denovo	11485417	(C/A)
19973	CakSNP19973	*Kabuli*	Ca_Kabuli_denovo	11489691	(A/G)
19974	CakSNP19974	*Kabuli*	Ca_Kabuli_denovo	11489727	(A/G)
19975	CakSNP19975	*Kabuli*	Ca_Kabuli_denovo	11492137	(T/C)
19976	CakSNP19976	*Kabuli*	Ca_Kabuli_denovo	11500226	(C/T)
19977	CakSNP19977	*Kabuli*	Ca_Kabuli_denovo	11500286	(A/G)
19978	CakSNP19978	*Kabuli*	Ca_Kabuli_denovo	11500518	(C/T)
19979	CakSNP19979	*Kabuli*	Ca_Kabuli_denovo	11500549	(G/A)
19980	CakSNP19980	*Kabuli*	Ca_Kabuli_denovo	11509689	(A/G)
19981	CakSNP19981	*Kabuli*	Ca_Kabuli_denovo	11509710	(T/A)
19982	CakSNP19982	*Kabuli*	Ca_Kabuli_denovo	11520113	(T/C)
19983	CakSNP19983	*Kabuli*	Ca_Kabuli_denovo	11520121	(T/G)
19984	CakSNP19984	*Kabuli*	Ca_Kabuli_denovo	11522244	(T/C)
19985	CakSNP19985	*Kabuli*	Ca_Kabuli_denovo	11522247	(G/A)
19986	CakSNP19986	*Kabuli*	Ca_Kabuli_denovo	11524862	(A/G)
19987	CakSNP19987	*Kabuli*	Ca_Kabuli_denovo	11524904	(A/G)

S.N.	SNP IDs	Cultivars	Chromosomes/scaffolds	Physical positions (bp)	SNPs
19988	CakSNP19988	*Kabuli*	Ca_Kabuli_denovo	11526181	(G/C)
19989	CakSNP19989	*Kabuli*	Ca_Kabuli_denovo	11526429	(C/T)
19990	CakSNP19990	*Kabuli*	Ca_Kabuli_denovo	11526440	(C/T)
19991	CakSNP19991	*Kabuli*	Ca_Kabuli_denovo	11536624	(T/G)
19992	CakSNP19992	*Kabuli*	Ca_Kabuli_denovo	11537381	(G/A)
19993	CakSNP19993	*Kabuli*	Ca_Kabuli_denovo	11539912	(A/G)
19994	CakSNP19994	*Kabuli*	Ca_Kabuli_denovo	11539928	(C/A)
19995	CakSNP19995	*Kabuli*	Ca_Kabuli_denovo	11543736	(G/A)
19996	CakSNP19996	*Kabuli*	Ca_Kabuli_denovo	11546270	(G/A)
19997	CakSNP19997	*Kabuli*	Ca_Kabuli_denovo	11546282	(G/A)
19998	CakSNP19998	*Kabuli*	Ca_Kabuli_denovo	11560747	(A/T)
19999	CakSNP19999	*Kabuli*	Ca_Kabuli_denovo	11560777	(T/A)
20000	CakSNP20000	*Kabuli*	Ca_Kabuli_denovo	11562092	(A/C)
20001	CakSNP20001	*Kabuli*	Ca_Kabuli_denovo	11566483	(T/C)
20002	CakSNP20002	*Kabuli*	Ca_Kabuli_denovo	11570439	(G/C)
20003	CakSNP20003	*Kabuli*	Ca_Kabuli_denovo	11571669	(C/T)
20004	CakSNP20004	*Kabuli*	Ca_Kabuli_denovo	11571683	(C/T)
20005	CakSNP20005	*Kabuli*	Ca_Kabuli_denovo	11572107	(C/T)
20006	CakSNP20006	*Kabuli*	Ca_Kabuli_denovo	11576597	(G/A)
20007	CakSNP20007	*Kabuli*	Ca_Kabuli_denovo	11582680	(A/G)
20008	CakSNP20008	*Kabuli*	Ca_Kabuli_denovo	11584675	(A/G)
20009	CakSNP20009	*Kabuli*	Ca_Kabuli_denovo	11594624	(T/C)
20010	CakSNP20010	*Kabuli*	Ca_Kabuli_denovo	11596364	(G/A)
20011	CakSNP20011	*Kabuli*	Ca_Kabuli_denovo	11596442	(A/G)
20012	CakSNP20012	*Kabuli*	Ca_Kabuli_denovo	11597952	(C/T)
20013	CakSNP20013	*Kabuli*	Ca_Kabuli_denovo	11597994	(A/G)
20014	CakSNP20014	*Kabuli*	Ca_Kabuli_denovo	11601006	(G/T)
20015	CakSNP20015	*Kabuli*	Ca_Kabuli_denovo	11602335	(G/C)
20016	CakSNP20016	*Kabuli*	Ca_Kabuli_denovo	11602781	(C/G)
20017	CakSNP20017	*Kabuli*	Ca_Kabuli_denovo	11603266	(T/A)
20018	CakSNP20018	*Kabuli*	Ca_Kabuli_denovo	11621388	(A/G)
20019	CakSNP20019	*Kabuli*	Ca_Kabuli_denovo	11622352	(G/C)
20020	CakSNP20020	*Kabuli*	Ca_Kabuli_denovo	11622365	(A/T)
20021	CakSNP20021	*Kabuli*	Ca_Kabuli_denovo	11628118	(T/C)
20022	CakSNP20022	*Kabuli*	Ca_Kabuli_denovo	11628136	(A/C)
20023	CakSNP20023	*Kabuli*	Ca_Kabuli_denovo	11629142	(G/C)
20024	CakSNP20024	*Kabuli*	Ca_Kabuli_denovo	11629160	(T/G)
20025	CakSNP20025	*Kabuli*	Ca_Kabuli_denovo	11632641	(T/G)

S.N.	SNP IDs	Cultivars	Chromosomes/scaffolds	Physical positions (bp)	SNPs
20026	CakSNP20026	*Kabuli*	Ca_Kabuli_denovo	11632708	(T/C)
20027	CakSNP20027	*Kabuli*	Ca_Kabuli_denovo	11633909	(A/G)
20028	CakSNP20028	*Kabuli*	Ca_Kabuli_denovo	11635456	(T/C)
20029	CakSNP20029	*Kabuli*	Ca_Kabuli_denovo	11635493	(G/C)
20030	CakSNP20030	*Kabuli*	Ca_Kabuli_denovo	11637531	(C/T)
20031	CakSNP20031	*Kabuli*	Ca_Kabuli_denovo	11647295	(G/A)
20032	CakSNP20032	*Kabuli*	Ca_Kabuli_denovo	11647988	(A/G)
20033	CakSNP20033	*Kabuli*	Ca_Kabuli_denovo	11653902	(C/A)
20034	CakSNP20034	*Kabuli*	Ca_Kabuli_denovo	11654696	(G/T)
20035	CakSNP20035	*Kabuli*	Ca_Kabuli_denovo	11654699	(G/A)
20036	CakSNP20036	*Kabuli*	Ca_Kabuli_denovo	11657390	(G/A)
20037	CakSNP20037	*Kabuli*	Ca_Kabuli_denovo	11657404	(G/A)
20038	CakSNP20038	*Kabuli*	Ca_Kabuli_denovo	11657818	(C/T)
20039	CakSNP20039	*Kabuli*	Ca_Kabuli_denovo	11657827	(C/G)
20040	CakSNP20040	*Kabuli*	Ca_Kabuli_denovo	11658673	(G/C)
20041	CakSNP20041	*Kabuli*	Ca_Kabuli_denovo	11658712	(G/T)
20042	CakSNP20042	*Kabuli*	Ca_Kabuli_denovo	11658756	(C/G)
20043	CakSNP20043	*Kabuli*	Ca_Kabuli_denovo	11659710	(C/G)
20044	CakSNP20044	*Kabuli*	Ca_Kabuli_denovo	11663334	(T/C)
20045	CakSNP20045	*Kabuli*	Ca_Kabuli_denovo	11665747	(C/T)
20046	CakSNP20046	*Kabuli*	Ca_Kabuli_denovo	11672834	(T/C)
20047	CakSNP20047	*Kabuli*	Ca_Kabuli_denovo	11672842	(G/A)
20048	CakSNP20048	*Kabuli*	Ca_Kabuli_denovo	11676813	(G/C)
20049	CakSNP20049	*Kabuli*	Ca_Kabuli_denovo	11677203	(G/T)
20050	CakSNP20050	*Kabuli*	Ca_Kabuli_denovo	11677211	(C/T)
20051	CakSNP20051	*Kabuli*	Ca_Kabuli_denovo	11677396	(A/G)
20052	CakSNP20052	*Kabuli*	Ca_Kabuli_denovo	11678405	(A/G)
20053	CakSNP20053	*Kabuli*	Ca_Kabuli_denovo	11681261	(C/A)
20054	CakSNP20054	*Kabuli*	Ca_Kabuli_denovo	11681262	(T/G)
20055	CakSNP20055	*Kabuli*	Ca_Kabuli_denovo	11691933	(A/G)
20056	CakSNP20056	*Kabuli*	Ca_Kabuli_denovo	11691993	(C/A)
20057	CakSNP20057	*Kabuli*	Ca_Kabuli_denovo	11700734	(G/A)
20058	CakSNP20058	*Kabuli*	Ca_Kabuli_denovo	11705216	(A/C)
20059	CakSNP20059	*Kabuli*	Ca_Kabuli_denovo	11705234	(G/A)
20060	CakSNP20060	*Kabuli*	Ca_Kabuli_denovo	11718358	(T/G)
20061	CakSNP20061	*Kabuli*	Ca_Kabuli_denovo	11727596	(A/G)
20062	CakSNP20062	*Kabuli*	Ca_Kabuli_denovo	11729357	(A/T)
20063	CakSNP20063	*Kabuli*	Ca_Kabuli_denovo	11729711	(C/A)

S.N.	SNP IDs	Cultivars	Chromosomes/scaffolds	Physical positions (bp)	SNPs
20064	CakSNP20064	*Kabuli*	Ca_Kabuli_denovo	11731601	(C/T)
20065	CakSNP20065	*Kabuli*	Ca_Kabuli_denovo	11732939	(G/A)
20066	CakSNP20066	*Kabuli*	Ca_Kabuli_denovo	11751802	(T/C)
20067	CakSNP20067	*Kabuli*	Ca_Kabuli_denovo	11751841	(C/T)
20068	CakSNP20068	*Kabuli*	Ca_Kabuli_denovo	11756035	(G/T)
20069	CakSNP20069	*Kabuli*	Ca_Kabuli_denovo	11757946	(C/T)
20070	CakSNP20070	*Kabuli*	Ca_Kabuli_denovo	11758845	(C/T)
20071	CakSNP20071	*Kabuli*	Ca_Kabuli_denovo	11758846	(G/A)
20072	CakSNP20072	*Kabuli*	Ca_Kabuli_denovo	11765477	(T/A)
20073	CakSNP20073	*Kabuli*	Ca_Kabuli_denovo	11767720	(T/C)
20074	CakSNP20074	*Kabuli*	Ca_Kabuli_denovo	11767747	(C/G)
20075	CakSNP20075	*Kabuli*	Ca_Kabuli_denovo	11773745	(C/G)
20076	CakSNP20076	*Kabuli*	Ca_Kabuli_denovo	11777816	(G/C)
20077	CakSNP20077	*Kabuli*	Ca_Kabuli_denovo	11785619	(G/A)
20078	CakSNP20078	*Kabuli*	Ca_Kabuli_denovo	11785637	(C/G)
20079	CakSNP20079	*Kabuli*	Ca_Kabuli_denovo	11789993	(G/T)
20080	CakSNP20080	*Kabuli*	Ca_Kabuli_denovo	11789999	(A/C)
20081	CakSNP20081	*Kabuli*	Ca_Kabuli_denovo	11792609	(C/T)
20082	CakSNP20082	*Kabuli*	Ca_Kabuli_denovo	11792678	(A/C)
20083	CakSNP20083	*Kabuli*	Ca_Kabuli_denovo	11792680	(A/T)
20084	CakSNP20084	*Kabuli*	Ca_Kabuli_denovo	11797038	(T/C)
20085	CakSNP20085	*Kabuli*	Ca_Kabuli_denovo	11797045	(C/A)
20086	CakSNP20086	*Kabuli*	Ca_Kabuli_denovo	11797720	(C/T)
20087	CakSNP20087	*Kabuli*	Ca_Kabuli_denovo	11798474	(A/G)
20088	CakSNP20088	*Kabuli*	Ca_Kabuli_denovo	11800425	(C/T)
20089	CakSNP20089	*Kabuli*	Ca_Kabuli_denovo	11800433	(C/A)
20090	CakSNP20090	*Kabuli*	Ca_Kabuli_denovo	11801910	(G/C)
20091	CakSNP20091	*Kabuli*	Ca_Kabuli_denovo	11801949	(A/G)
20092	CakSNP20092	*Kabuli*	Ca_Kabuli_denovo	11801959	(C/G)
20093	CakSNP20093	*Kabuli*	Ca_Kabuli_denovo	11813358	(T/C)
20094	CakSNP20094	*Kabuli*	Ca_Kabuli_denovo	11813400	(T/A)
20095	CakSNP20095	*Kabuli*	Ca_Kabuli_denovo	11818388	(C/G)
20096	CakSNP20096	*Kabuli*	Ca_Kabuli_denovo	11818997	(A/C)
20097	CakSNP20097	*Kabuli*	Ca_Kabuli_denovo	11818999	(A/C)
20098	CakSNP20098	*Kabuli*	Ca_Kabuli_denovo	11821809	(C/T)
20099	CakSNP20099	*Kabuli*	Ca_Kabuli_denovo	11826617	(G/T)
20100	CakSNP20100	*Kabuli*	Ca_Kabuli_denovo	11826835	(C/T)
20101	CakSNP20101	*Kabuli*	Ca_Kabuli_denovo	11826865	(T/C)

S.N.	SNP IDs	Cultivars	Chromosomes/scaffolds	Physical positions (bp)	SNPs
20102	CakSNP20102	*Kabuli*	Ca_Kabuli_denovo	11828227	(C/A)
20103	CakSNP20103	*Kabuli*	Ca_Kabuli_denovo	11832922	(A/G)
20104	CakSNP20104	*Kabuli*	Ca_Kabuli_denovo	11836267	(T/C)
20105	CakSNP20105	*Kabuli*	Ca_Kabuli_denovo	11836286	(C/T)
20106	CakSNP20106	*Kabuli*	Ca_Kabuli_denovo	11839483	(T/C)
20107	CakSNP20107	*Kabuli*	Ca_Kabuli_denovo	11841284	(A/G)
20108	CakSNP20108	*Kabuli*	Ca_Kabuli_denovo	11842163	(T/C)
20109	CakSNP20109	*Kabuli*	Ca_Kabuli_denovo	11842187	(T/C)
20110	CakSNP20110	*Kabuli*	Ca_Kabuli_denovo	11844115	(G/C)
20111	CakSNP20111	*Kabuli*	Ca_Kabuli_denovo	11848053	(T/C)
20112	CakSNP20112	*Kabuli*	Ca_Kabuli_denovo	11850589	(C/G)
20113	CakSNP20113	*Kabuli*	Ca_Kabuli_denovo	11851324	(T/C)
20114	CakSNP20114	*Kabuli*	Ca_Kabuli_denovo	11851331	(G/A)
20115	CakSNP20115	*Kabuli*	Ca_Kabuli_denovo	11853095	(T/A)
20116	CakSNP20116	*Kabuli*	Ca_Kabuli_denovo	11855554	(A/G)
20117	CakSNP20117	*Kabuli*	Ca_Kabuli_denovo	11855592	(A/T)
20118	CakSNP20118	*Kabuli*	Ca_Kabuli_denovo	11857112	(A/G)
20119	CakSNP20119	*Kabuli*	Ca_Kabuli_denovo	11858823	(C/G)
20120	CakSNP20120	*Kabuli*	Ca_Kabuli_denovo	11858841	(C/T)
20121	CakSNP20121	*Kabuli*	Ca_Kabuli_denovo	11867607	(G/A)
20122	CakSNP20122	*Kabuli*	Ca_Kabuli_denovo	11868238	(A/G)
20123	CakSNP20123	*Kabuli*	Ca_Kabuli_denovo	11868277	(C/T)
20124	CakSNP20124	*Kabuli*	Ca_Kabuli_denovo	11870258	(T/C)
20125	CakSNP20125	*Kabuli*	Ca_Kabuli_denovo	11879386	(A/C)
20126	CakSNP20126	*Kabuli*	Ca_Kabuli_denovo	11879392	(T/C)
20127	CakSNP20127	*Kabuli*	Ca_Kabuli_denovo	11887761	(T/C)
20128	CakSNP20128	*Kabuli*	Ca_Kabuli_denovo	11887771	(T/C)
20129	CakSNP20129	*Kabuli*	Ca_Kabuli_denovo	11888785	(T/C)
20130	CakSNP20130	*Kabuli*	Ca_Kabuli_denovo	11888809	(T/C)
20131	CakSNP20131	*Kabuli*	Ca_Kabuli_denovo	11901103	(A/T)
20132	CakSNP20132	*Kabuli*	Ca_Kabuli_denovo	11904032	(G/T)
20133	CakSNP20133	*Kabuli*	Ca_Kabuli_denovo	11905598	(T/A)
20134	CakSNP20134	*Kabuli*	Ca_Kabuli_denovo	11905613	(C/G)
20135	CakSNP20135	*Kabuli*	Ca_Kabuli_denovo	11909810	(G/C)
20136	CakSNP20136	*Kabuli*	Ca_Kabuli_denovo	11913469	(A/G)
20137	CakSNP20137	*Kabuli*	Ca_Kabuli_denovo	11917788	(G/C)
20138	CakSNP20138	*Kabuli*	Ca_Kabuli_denovo	11918472	(T/C)
20139	CakSNP20139	*Kabuli*	Ca_Kabuli_denovo	11921650	(T/C)

S.N.	SNP IDs	Cultivars	Chromosomes/scaffolds	Physical positions (bp)	SNPs
20140	CakSNP20140	*Kabuli*	Ca_Kabuli_denovo	11921653	(C/T)
20141	CakSNP20141	*Kabuli*	Ca_Kabuli_denovo	11926343	(G/A)
20142	CakSNP20142	*Kabuli*	Ca_Kabuli_denovo	11926414	(C/G)
20143	CakSNP20143	*Kabuli*	Ca_Kabuli_denovo	11928286	(C/G)
20144	CakSNP20144	*Kabuli*	Ca_Kabuli_denovo	11928288	(A/G)
20145	CakSNP20145	*Kabuli*	Ca_Kabuli_denovo	11932767	(C/T)
20146	CakSNP20146	*Kabuli*	Ca_Kabuli_denovo	11933776	(T/C)
20147	CakSNP20147	*Kabuli*	Ca_Kabuli_denovo	11933779	(T/C)
20148	CakSNP20148	*Kabuli*	Ca_Kabuli_denovo	11934806	(A/G)
20149	CakSNP20149	*Kabuli*	Ca_Kabuli_denovo	11934835	(C/T)
20150	CakSNP20150	*Kabuli*	Ca_Kabuli_denovo	11935833	(C/T)
20151	CakSNP20151	*Kabuli*	Ca_Kabuli_denovo	11936229	(A/T)
20152	CakSNP20152	*Kabuli*	Ca_Kabuli_denovo	11937312	(A/G)
20153	CakSNP20153	*Kabuli*	Ca_Kabuli_denovo	11937322	(A/C)
20154	CakSNP20154	*Kabuli*	Ca_Kabuli_denovo	11937520	(G/T)
20155	CakSNP20155	*Kabuli*	Ca_Kabuli_denovo	11941139	(A/G)
20156	CakSNP20156	*Kabuli*	Ca_Kabuli_denovo	11942242	(T/A)
20157	CakSNP20157	*Kabuli*	Ca_Kabuli_denovo	11942299	(C/T)
20158	CakSNP20158	*Kabuli*	Ca_Kabuli_denovo	11947730	(T/A)
20159	CakSNP20159	*Kabuli*	Ca_Kabuli_denovo	11953049	(C/G)
20160	CakSNP20160	*Kabuli*	Ca_Kabuli_denovo	11962838	(A/G)
20161	CakSNP20161	*Kabuli*	Ca_Kabuli_denovo	11962840	(G/A)
20162	CakSNP20162	*Kabuli*	Ca_Kabuli_denovo	11965507	(A/G)
20163	CakSNP20163	*Kabuli*	Ca_Kabuli_denovo	11965510	(G/A)
20164	CakSNP20164	*Kabuli*	Ca_Kabuli_denovo	11970885	(C/G)
20165	CakSNP20165	*Kabuli*	Ca_Kabuli_denovo	11976757	(C/G)
20166	CakSNP20166	*Kabuli*	Ca_Kabuli_denovo	11983367	(T/C)
20167	CakSNP20167	*Kabuli*	Ca_Kabuli_denovo	11983391	(C/A)
20168	CakSNP20168	*Kabuli*	Ca_Kabuli_denovo	11986506	(G/A)
20169	CakSNP20169	*Kabuli*	Ca_Kabuli_denovo	11986536	(A/G)
20170	CakSNP20170	*Kabuli*	Ca_Kabuli_denovo	11986557	(A/G)
20171	CakSNP20171	*Kabuli*	Ca_Kabuli_denovo	11987561	(G/C)
20172	CakSNP20172	*Kabuli*	Ca_Kabuli_denovo	11993728	(G/A)
20173	CakSNP20173	*Kabuli*	Ca_Kabuli_denovo	12005056	(T/C)
20174	CakSNP20174	*Kabuli*	Ca_Kabuli_denovo	12008099	(T/C)
20175	CakSNP20175	*Kabuli*	Ca_Kabuli_denovo	12008111	(T/C)
20176	CakSNP20176	*Kabuli*	Ca_Kabuli_denovo	12010534	(A/T)
20177	CakSNP20177	*Kabuli*	Ca_Kabuli_denovo	12018239	(A/G)

S.N.	SNP IDs	Cultivars	Chromosomes/scaffolds	Physical positions (bp)	SNPs
20178	CakSNP20178	*Kabuli*	Ca_Kabuli_denovo	12019804	(C/T)
20179	CakSNP20179	*Kabuli*	Ca_Kabuli_denovo	12024854	(T/G)
20180	CakSNP20180	*Kabuli*	Ca_Kabuli_denovo	12027996	(C/G)
20181	CakSNP20181	*Kabuli*	Ca_Kabuli_denovo	12028061	(A/G)
20182	CakSNP20182	*Kabuli*	Ca_Kabuli_denovo	12033965	(G/A)
20183	CakSNP20183	*Kabuli*	Ca_Kabuli_denovo	12033971	(C/T)
20184	CakSNP20184	*Kabuli*	Ca_Kabuli_denovo	12036188	(A/G)
20185	CakSNP20185	*Kabuli*	Ca_Kabuli_denovo	12036224	(T/A)
20186	CakSNP20186	*Kabuli*	Ca_Kabuli_denovo	12037582	(A/G)
20187	CakSNP20187	*Kabuli*	Ca_Kabuli_denovo	12037583	(T/C)
20188	CakSNP20188	*Kabuli*	Ca_Kabuli_denovo	12041729	(T/G)
20189	CakSNP20189	*Kabuli*	Ca_Kabuli_denovo	12049740	(G/A)
20190	CakSNP20190	*Kabuli*	Ca_Kabuli_denovo	12054052	(T/G)
20191	CakSNP20191	*Kabuli*	Ca_Kabuli_denovo	12063452	(G/A)
20192	CakSNP20192	*Kabuli*	Ca_Kabuli_denovo	12063460	(C/T)
20193	CakSNP20193	*Kabuli*	Ca_Kabuli_denovo	12065643	(T/G)
20194	CakSNP20194	*Kabuli*	Ca_Kabuli_denovo	12066145	(T/C)
20195	CakSNP20195	*Kabuli*	Ca_Kabuli_denovo	12075499	(A/T)
20196	CakSNP20196	*Kabuli*	Ca_Kabuli_denovo	12075514	(A/T)
20197	CakSNP20197	*Kabuli*	Ca_Kabuli_denovo	12075516	(C/G)
20198	CakSNP20198	*Kabuli*	Ca_Kabuli_denovo	12075524	(C/G)
20199	CakSNP20199	*Kabuli*	Ca_Kabuli_denovo	12076633	(T/C)
20200	CakSNP20200	*Kabuli*	Ca_Kabuli_denovo	12076636	(A/G)
20201	CakSNP20201	*Kabuli*	Ca_Kabuli_denovo	12077380	(C/T)
20202	CakSNP20202	*Kabuli*	Ca_Kabuli_denovo	12079082	(G/A)
20203	CakSNP20203	*Kabuli*	Ca_Kabuli_denovo	12079352	(G/A)
20204	CakSNP20204	*Kabuli*	Ca_Kabuli_denovo	12079358	(G/C)
20205	CakSNP20205	*Kabuli*	Ca_Kabuli_denovo	12083899	(T/C)
20206	CakSNP20206	*Kabuli*	Ca_Kabuli_denovo	12083908	(G/T)
20207	CakSNP20207	*Kabuli*	Ca_Kabuli_denovo	12090488	(C/G)
20208	CakSNP20208	*Kabuli*	Ca_Kabuli_denovo	12100423	(C/T)
20209	CakSNP20209	*Kabuli*	Ca_Kabuli_denovo	12100504	(C/A)
20210	CakSNP20210	*Kabuli*	Ca_Kabuli_denovo	12105637	(T/C)
20211	CakSNP20211	*Kabuli*	Ca_Kabuli_denovo	12105669	(G/A)
20212	CakSNP20212	*Kabuli*	Ca_Kabuli_denovo	12108912	(G/A)
20213	CakSNP20213	*Kabuli*	Ca_Kabuli_denovo	12118782	(G/T)
20214	CakSNP20214	*Kabuli*	Ca_Kabuli_denovo	12118820	(T/A)
20215	CakSNP20215	*Kabuli*	Ca_Kabuli_denovo	12123015	(A/C)

S.N.	SNP IDs	Cultivars	Chromosomes/scaffolds	Physical positions (bp)	SNPs
20216	CakSNP20216	*Kabuli*	Ca_Kabuli_denovo	12123016	(G/C)
20217	CakSNP20217	*Kabuli*	Ca_Kabuli_denovo	12128221	(A/C)
20218	CakSNP20218	*Kabuli*	Ca_Kabuli_denovo	12129661	(T/C)
20219	CakSNP20219	*Kabuli*	Ca_Kabuli_denovo	12132152	(C/T)
20220	CakSNP20220	*Kabuli*	Ca_Kabuli_denovo	12140555	(C/T)
20221	CakSNP20221	*Kabuli*	Ca_Kabuli_denovo	12140573	(C/A)
20222	CakSNP20222	*Kabuli*	Ca_Kabuli_denovo	12141836	(G/A)
20223	CakSNP20223	*Kabuli*	Ca_Kabuli_denovo	12142574	(A/G)
20224	CakSNP20224	*Kabuli*	Ca_Kabuli_denovo	12151348	(A/G)
20225	CakSNP20225	*Kabuli*	Ca_Kabuli_denovo	12151375	(A/G)
20226	CakSNP20226	*Kabuli*	Ca_Kabuli_denovo	12157446	(T/G)
20227	CakSNP20227	*Kabuli*	Ca_Kabuli_denovo	12157447	(T/A)
20228	CakSNP20228	*Kabuli*	Ca_Kabuli_denovo	12157570	(G/A)
20229	CakSNP20229	*Kabuli*	Ca_Kabuli_denovo	12160442	(T/G)
20230	CakSNP20230	*Kabuli*	Ca_Kabuli_denovo	12160463	(C/T)
20231	CakSNP20231	*Kabuli*	Ca_Kabuli_denovo	12160547	(T/C)
20232	CakSNP20232	*Kabuli*	Ca_Kabuli_denovo	12160588	(A/G)
20233	CakSNP20233	*Kabuli*	Ca_Kabuli_denovo	12161049	(A/C)
20234	CakSNP20234	*Kabuli*	Ca_Kabuli_denovo	12163984	(G/C)
20235	CakSNP20235	*Kabuli*	Ca_Kabuli_denovo	12170666	(T/C)
20236	CakSNP20236	*Kabuli*	Ca_Kabuli_denovo	12174208	(C/G)
20237	CakSNP20237	*Kabuli*	Ca_Kabuli_denovo	12178363	(A/G)
20238	CakSNP20238	*Kabuli*	Ca_Kabuli_denovo	12181987	(G/A)
20239	CakSNP20239	*Kabuli*	Ca_Kabuli_denovo	12182026	(C/G)
20240	CakSNP20240	*Kabuli*	Ca_Kabuli_denovo	12186730	(T/C)
20241	CakSNP20241	*Kabuli*	Ca_Kabuli_denovo	12186760	(C/G)
20242	CakSNP20242	*Kabuli*	Ca_Kabuli_denovo	12187344	(A/G)
20243	CakSNP20243	*Kabuli*	Ca_Kabuli_denovo	12192872	(C/T)
20244	CakSNP20244	*Kabuli*	Ca_Kabuli_denovo	12201334	(A/G)
20245	CakSNP20245	*Kabuli*	Ca_Kabuli_denovo	12202915	(A/G)
20246	CakSNP20246	*Kabuli*	Ca_Kabuli_denovo	12209776	(T/C)
20247	CakSNP20247	*Kabuli*	Ca_Kabuli_denovo	12215372	(G/T)
20248	CakSNP20248	*Kabuli*	Ca_Kabuli_denovo	12216070	(C/T)
20249	CakSNP20249	*Kabuli*	Ca_Kabuli_denovo	12217982	(G/A)
20250	CakSNP20250	*Kabuli*	Ca_Kabuli_denovo	12234821	(C/T)
20251	CakSNP20251	*Kabuli*	Ca_Kabuli_denovo	12236422	(T/C)
20252	CakSNP20252	*Kabuli*	Ca_Kabuli_denovo	12239018	(C/G)
20253	CakSNP20253	*Kabuli*	Ca_Kabuli_denovo	12240512	(A/G)

S.N.	SNP IDs	Cultivars	Chromosomes/scaffolds	Physical positions (bp)	SNPs
20254	CakSNP20254	*Kabuli*	Ca_Kabuli_denovo	12240549	(A/G)
20255	CakSNP20255	*Kabuli*	Ca_Kabuli_denovo	12243380	(A/G)
20256	CakSNP20256	*Kabuli*	Ca_Kabuli_denovo	12248626	(A/G)
20257	CakSNP20257	*Kabuli*	Ca_Kabuli_denovo	12251127	(C/A)
20258	CakSNP20258	*Kabuli*	Ca_Kabuli_denovo	12252623	(A/G)
20259	CakSNP20259	*Kabuli*	Ca_Kabuli_denovo	12257991	(T/C)
20260	CakSNP20260	*Kabuli*	Ca_Kabuli_denovo	12257993	(T/G)
20261	CakSNP20261	*Kabuli*	Ca_Kabuli_denovo	12264308	(G/T)
20262	CakSNP20262	*Kabuli*	Ca_Kabuli_denovo	12266786	(A/G)
20263	CakSNP20263	*Kabuli*	Ca_Kabuli_denovo	12269505	(G/C)
20264	CakSNP20264	*Kabuli*	Ca_Kabuli_denovo	12292363	(G/C)
20265	CakSNP20265	*Kabuli*	Ca_Kabuli_denovo	12298060	(C/G)
20266	CakSNP20266	*Kabuli*	Ca_Kabuli_denovo	12298745	(C/T)
20267	CakSNP20267	*Kabuli*	Ca_Kabuli_denovo	12301582	(A/C)
20268	CakSNP20268	*Kabuli*	Ca_Kabuli_denovo	12309140	(G/A)
20269	CakSNP20269	*Kabuli*	Ca_Kabuli_denovo	12309148	(A/G)
20270	CakSNP20270	*Kabuli*	Ca_Kabuli_denovo	12318085	(T/C)
20271	CakSNP20271	*Kabuli*	Ca_Kabuli_denovo	12318112	(T/C)
20272	CakSNP20272	*Kabuli*	Ca_Kabuli_denovo	12319732	(G/C)
20273	CakSNP20273	*Kabuli*	Ca_Kabuli_denovo	12323652	(G/C)
20274	CakSNP20274	*Kabuli*	Ca_Kabuli_denovo	12329986	(G/A)
20275	CakSNP20275	*Kabuli*	Ca_Kabuli_denovo	12329992	(C/T)
20276	CakSNP20276	*Kabuli*	Ca_Kabuli_denovo	12331347	(C/G)
20277	CakSNP20277	*Kabuli*	Ca_Kabuli_denovo	12331761	(G/C)
20278	CakSNP20278	*Kabuli*	Ca_Kabuli_denovo	12331775	(C/G)
20279	CakSNP20279	*Kabuli*	Ca_Kabuli_denovo	12343528	(T/C)
20280	CakSNP20280	*Kabuli*	Ca_Kabuli_denovo	12343563	(T/C)
20281	CakSNP20281	*Kabuli*	Ca_Kabuli_denovo	12348038	(C/G)
20282	CakSNP20282	*Kabuli*	Ca_Kabuli_denovo	12348044	(C/G)
20283	CakSNP20283	*Kabuli*	Ca_Kabuli_denovo	12356015	(T/C)
20284	CakSNP20284	*Kabuli*	Ca_Kabuli_denovo	12360701	(T/G)
20285	CakSNP20285	*Kabuli*	Ca_Kabuli_denovo	12360722	(C/T)
20286	CakSNP20286	*Kabuli*	Ca_Kabuli_denovo	12364826	(G/T)
20287	CakSNP20287	*Kabuli*	Ca_Kabuli_denovo	12365459	(A/C)
20288	CakSNP20288	*Kabuli*	Ca_Kabuli_denovo	12368369	(C/T)
20289	CakSNP20289	*Kabuli*	Ca_Kabuli_denovo	12380551	(A/T)
20290	CakSNP20290	*Kabuli*	Ca_Kabuli_denovo	12380579	(G/C)
20291	CakSNP20291	*Kabuli*	Ca_Kabuli_denovo	12381201	(C/T)

S.N.	SNP IDs	Cultivars	Chromosomes/scaffolds	Physical positions (bp)	SNPs
20292	CakSNP20292	*Kabuli*	Ca_Kabuli_denovo	12381391	(C/T)
20293	CakSNP20293	*Kabuli*	Ca_Kabuli_denovo	12385312	(G/C)
20294	CakSNP20294	*Kabuli*	Ca_Kabuli_denovo	12385354	(A/C)
20295	CakSNP20295	*Kabuli*	Ca_Kabuli_denovo	12386113	(C/G)
20296	CakSNP20296	*Kabuli*	Ca_Kabuli_denovo	12387661	(C/A)
20297	CakSNP20297	*Kabuli*	Ca_Kabuli_denovo	12387670	(T/C)
20298	CakSNP20298	*Kabuli*	Ca_Kabuli_denovo	12391385	(G/A)
20299	CakSNP20299	*Kabuli*	Ca_Kabuli_denovo	12391414	(G/A)
20300	CakSNP20300	*Kabuli*	Ca_Kabuli_denovo	12394679	(T/C)
20301	CakSNP20301	*Kabuli*	Ca_Kabuli_denovo	12394728	(G/C)
20302	CakSNP20302	*Kabuli*	Ca_Kabuli_denovo	12394732	(C/T)
20303	CakSNP20303	*Kabuli*	Ca_Kabuli_denovo	12404927	(A/G)
20304	CakSNP20304	*Kabuli*	Ca_Kabuli_denovo	12411157	(C/G)
20305	CakSNP20305	*Kabuli*	Ca_Kabuli_denovo	12415971	(C/T)
20306	CakSNP20306	*Kabuli*	Ca_Kabuli_denovo	12417288	(C/A)
20307	CakSNP20307	*Kabuli*	Ca_Kabuli_denovo	12417294	(G/C)
20308	CakSNP20308	*Kabuli*	Ca_Kabuli_denovo	12420048	(G/C)
20309	CakSNP20309	*Kabuli*	Ca_Kabuli_denovo	12422409	(C/G)
20310	CakSNP20310	*Kabuli*	Ca_Kabuli_denovo	12422413	(A/C)
20311	CakSNP20311	*Kabuli*	Ca_Kabuli_denovo	12424936	(C/T)
20312	CakSNP20312	*Kabuli*	Ca_Kabuli_denovo	12424968	(A/T)
20313	CakSNP20313	*Kabuli*	Ca_Kabuli_denovo	12431462	(C/T)
20314	CakSNP20314	*Kabuli*	Ca_Kabuli_denovo	12440976	(C/T)
20315	CakSNP20315	*Kabuli*	Ca_Kabuli_denovo	12449927	(T/C)
20316	CakSNP20316	*Kabuli*	Ca_Kabuli_denovo	12449999	(C/T)
20317	CakSNP20317	*Kabuli*	Ca_Kabuli_denovo	12450760	(A/G)
20318	CakSNP20318	*Kabuli*	Ca_Kabuli_denovo	12450818	(G/C)
20319	CakSNP20319	*Kabuli*	Ca_Kabuli_denovo	12450967	(C/T)
20320	CakSNP20320	*Kabuli*	Ca_Kabuli_denovo	12450989	(A/G)
20321	CakSNP20321	*Kabuli*	Ca_Kabuli_denovo	12467411	(A/G)
20322	CakSNP20322	*Kabuli*	Ca_Kabuli_denovo	12468098	(C/G)
20323	CakSNP20323	*Kabuli*	Ca_Kabuli_denovo	12471420	(T/C)
20324	CakSNP20324	*Kabuli*	Ca_Kabuli_denovo	12472768	(G/A)
20325	CakSNP20325	*Kabuli*	Ca_Kabuli_denovo	12473457	(A/G)
20326	CakSNP20326	*Kabuli*	Ca_Kabuli_denovo	12484829	(T/G)
20327	CakSNP20327	*Kabuli*	Ca_Kabuli_denovo	12485751	(A/G)
20328	CakSNP20328	*Kabuli*	Ca_Kabuli_denovo	12485766	(C/T)
20329	CakSNP20329	*Kabuli*	Ca_Kabuli_denovo	12486851	(A/G)

S.N.	SNP IDs	Cultivars	Chromosomes/scaffolds	Physical positions (bp)	SNPs
20330	CakSNP20330	*Kabuli*	Ca_Kabuli_denovo	12486854	(G/A)
20331	CakSNP20331	*Kabuli*	Ca_Kabuli_denovo	12487619	(C/T)
20332	CakSNP20332	*Kabuli*	Ca_Kabuli_denovo	12487668	(A/G)
20333	CakSNP20333	*Kabuli*	Ca_Kabuli_denovo	12496808	(C/T)
20334	CakSNP20334	*Kabuli*	Ca_Kabuli_denovo	12500463	(G/A)
20335	CakSNP20335	*Kabuli*	Ca_Kabuli_denovo	12500466	(G/C)
20336	CakSNP20336	*Kabuli*	Ca_Kabuli_denovo	12502746	(A/T)
20337	CakSNP20337	*Kabuli*	Ca_Kabuli_denovo	12505149	(G/T)
20338	CakSNP20338	*Kabuli*	Ca_Kabuli_denovo	12506116	(A/C)
20339	CakSNP20339	*Kabuli*	Ca_Kabuli_denovo	12506158	(A/G)
20340	CakSNP20340	*Kabuli*	Ca_Kabuli_denovo	12507925	(C/T)
20341	CakSNP20341	*Kabuli*	Ca_Kabuli_denovo	12514887	(A/C)
20342	CakSNP20342	*Kabuli*	Ca_Kabuli_denovo	12527488	(G/C)
20343	CakSNP20343	*Kabuli*	Ca_Kabuli_denovo	12529187	(A/G)
20344	CakSNP20344	*Kabuli*	Ca_Kabuli_denovo	12530009	(C/T)
20345	CakSNP20345	*Kabuli*	Ca_Kabuli_denovo	12539987	(C/T)
20346	CakSNP20346	*Kabuli*	Ca_Kabuli_denovo	12541576	(C/G)
20347	CakSNP20347	*Kabuli*	Ca_Kabuli_denovo	12550942	(C/T)
20348	CakSNP20348	*Kabuli*	Ca_Kabuli_denovo	12562412	(C/T)
20349	CakSNP20349	*Kabuli*	Ca_Kabuli_denovo	12562414	(G/A)
20350	CakSNP20350	*Kabuli*	Ca_Kabuli_denovo	12567144	(T/C)
20351	CakSNP20351	*Kabuli*	Ca_Kabuli_denovo	12568213	(A/G)
20352	CakSNP20352	*Kabuli*	Ca_Kabuli_denovo	12568222	(G/A)
20353	CakSNP20353	*Kabuli*	Ca_Kabuli_denovo	12572669	(A/C)
20354	CakSNP20354	*Kabuli*	Ca_Kabuli_denovo	12572691	(C/T)
20355	CakSNP20355	*Kabuli*	Ca_Kabuli_denovo	12578294	(T/C)
20356	CakSNP20356	*Kabuli*	Ca_Kabuli_denovo	12578300	(A/C)
20357	CakSNP20357	*Kabuli*	Ca_Kabuli_denovo	12583593	(T/C)
20358	CakSNP20358	*Kabuli*	Ca_Kabuli_denovo	12588224	(G/A)
20359	CakSNP20359	*Kabuli*	Ca_Kabuli_denovo	12597602	(C/T)
20360	CakSNP20360	*Kabuli*	Ca_Kabuli_denovo	12597680	(C/T)
20361	CakSNP20361	*Kabuli*	Ca_Kabuli_denovo	12597698	(A/G)
20362	CakSNP20362	*Kabuli*	Ca_Kabuli_denovo	12598854	(G/A)
20363	CakSNP20363	*Kabuli*	Ca_Kabuli_denovo	12614448	(C/G)
20364	CakSNP20364	*Kabuli*	Ca_Kabuli_denovo	12614475	(T/G)
20365	CakSNP20365	*Kabuli*	Ca_Kabuli_denovo	12620203	(T/G)
20366	CakSNP20366	*Kabuli*	Ca_Kabuli_denovo	12625088	(G/A)
20367	CakSNP20367	*Kabuli*	Ca_Kabuli_denovo	12630071	(G/A)

S.N.	SNP IDs	Cultivars	Chromosomes/scaffolds	Physical positions (bp)	SNPs
20368	CakSNP20368	*Kabuli*	Ca_Kabuli_denovo	12630136	(C/G)
20369	CakSNP20369	*Kabuli*	Ca_Kabuli_denovo	12647378	(C/G)
20370	CakSNP20370	*Kabuli*	Ca_Kabuli_denovo	12647381	(C/T)
20371	CakSNP20371	*Kabuli*	Ca_Kabuli_denovo	12649202	(G/C)
20372	CakSNP20372	*Kabuli*	Ca_Kabuli_denovo	12649208	(G/A)
20373	CakSNP20373	*Kabuli*	Ca_Kabuli_denovo	12655462	(A/C)
20374	CakSNP20374	*Kabuli*	Ca_Kabuli_denovo	12655487	(A/C)
20375	CakSNP20375	*Kabuli*	Ca_Kabuli_denovo	12669133	(G/A)
20376	CakSNP20376	*Kabuli*	Ca_Kabuli_denovo	12672676	(T/C)
20377	CakSNP20377	*Kabuli*	Ca_Kabuli_denovo	12680590	(T/G)
20378	CakSNP20378	*Kabuli*	Ca_Kabuli_denovo	12680643	(T/C)
20379	CakSNP20379	*Kabuli*	Ca_Kabuli_denovo	12681610	(G/T)
20380	CakSNP20380	*Kabuli*	Ca_Kabuli_denovo	12683863	(C/A)
20381	CakSNP20381	*Kabuli*	Ca_Kabuli_denovo	12684098	(A/G)
20382	CakSNP20382	*Kabuli*	Ca_Kabuli_denovo	12684100	(C/T)
20383	CakSNP20383	*Kabuli*	Ca_Kabuli_denovo	12686753	(A/G)
20384	CakSNP20384	*Kabuli*	Ca_Kabuli_denovo	12686758	(C/A)
20385	CakSNP20385	*Kabuli*	Ca_Kabuli_denovo	12688024	(C/T)
20386	CakSNP20386	*Kabuli*	Ca_Kabuli_denovo	12688476	(G/C)
20387	CakSNP20387	*Kabuli*	Ca_Kabuli_denovo	12688489	(G/T)
20388	CakSNP20388	*Kabuli*	Ca_Kabuli_denovo	12689864	(G/A)
20389	CakSNP20389	*Kabuli*	Ca_Kabuli_denovo	12689914	(C/T)
20390	CakSNP20390	*Kabuli*	Ca_Kabuli_denovo	12691912	(C/A)
20391	CakSNP20391	*Kabuli*	Ca_Kabuli_denovo	12691926	(C/G)
20392	CakSNP20392	*Kabuli*	Ca_Kabuli_denovo	12692387	(A/G)
20393	CakSNP20393	*Kabuli*	Ca_Kabuli_denovo	12701219	(C/T)
20394	CakSNP20394	*Kabuli*	Ca_Kabuli_denovo	12701233	(A/G)
20395	CakSNP20395	*Kabuli*	Ca_Kabuli_denovo	12701423	(G/A)
20396	CakSNP20396	*Kabuli*	Ca_Kabuli_denovo	12711354	(C/G)
20397	CakSNP20397	*Kabuli*	Ca_Kabuli_denovo	12711370	(T/C)
20398	CakSNP20398	*Kabuli*	Ca_Kabuli_denovo	12711403	(G/C)
20399	CakSNP20399	*Kabuli*	Ca_Kabuli_denovo	12712367	(A/G)
20400	CakSNP20400	*Kabuli*	Ca_Kabuli_denovo	12712436	(A/G)
20401	CakSNP20401	*Kabuli*	Ca_Kabuli_denovo	12712492	(C/G)
20402	CakSNP20402	*Kabuli*	Ca_Kabuli_denovo	12719308	(C/T)
20403	CakSNP20403	*Kabuli*	Ca_Kabuli_denovo	12728194	(C/A)
20404	CakSNP20404	*Kabuli*	Ca_Kabuli_denovo	12728235	(C/G)
20405	CakSNP20405	*Kabuli*	Ca_Kabuli_denovo	12728761	(G/C)

S.N.	SNP IDs	Cultivars	Chromosomes/scaffolds	Physical positions (bp)	SNPs
20406	CakSNP20406	*Kabuli*	Ca_Kabuli_denovo	12728774	(G/C)
20407	CakSNP20407	*Kabuli*	Ca_Kabuli_denovo	12730181	(C/T)
20408	CakSNP20408	*Kabuli*	Ca_Kabuli_denovo	12735810	(A/G)
20409	CakSNP20409	*Kabuli*	Ca_Kabuli_denovo	12735840	(G/A)
20410	CakSNP20410	*Kabuli*	Ca_Kabuli_denovo	12738518	(C/T)
20411	CakSNP20411	*Kabuli*	Ca_Kabuli_denovo	12738520	(T/C)
20412	CakSNP20412	*Kabuli*	Ca_Kabuli_denovo	12739001	(C/G)
20413	CakSNP20413	*Kabuli*	Ca_Kabuli_denovo	12739011	(G/C)
20414	CakSNP20414	*Kabuli*	Ca_Kabuli_denovo	12739895	(T/G)
20415	CakSNP20415	*Kabuli*	Ca_Kabuli_denovo	12740080	(T/G)
20416	CakSNP20416	*Kabuli*	Ca_Kabuli_denovo	12740138	(G/C)
20417	CakSNP20417	*Kabuli*	Ca_Kabuli_denovo	12748520	(T/C)
20418	CakSNP20418	*Kabuli*	Ca_Kabuli_denovo	12754598	(G/A)
20419	CakSNP20419	*Kabuli*	Ca_Kabuli_denovo	12760788	(G/T)
20420	CakSNP20420	*Kabuli*	Ca_Kabuli_denovo	12763262	(C/T)
20421	CakSNP20421	*Kabuli*	Ca_Kabuli_denovo	12765002	(G/C)
20422	CakSNP20422	*Kabuli*	Ca_Kabuli_denovo	12765006	(G/A)
20423	CakSNP20423	*Kabuli*	Ca_Kabuli_denovo	12767039	(A/G)
20424	CakSNP20424	*Kabuli*	Ca_Kabuli_denovo	12769214	(A/T)
20425	CakSNP20425	*Kabuli*	Ca_Kabuli_denovo	12770684	(A/G)
20426	CakSNP20426	*Kabuli*	Ca_Kabuli_denovo	12770747	(C/G)
20427	CakSNP20427	*Kabuli*	Ca_Kabuli_denovo	12772557	(A/G)
20428	CakSNP20428	*Kabuli*	Ca_Kabuli_denovo	12772584	(T/C)
20429	CakSNP20429	*Kabuli*	Ca_Kabuli_denovo	12776581	(G/A)
20430	CakSNP20430	*Kabuli*	Ca_Kabuli_denovo	12780073	(C/A)
20431	CakSNP20431	*Kabuli*	Ca_Kabuli_denovo	12785221	(C/A)
20432	CakSNP20432	*Kabuli*	Ca_Kabuli_denovo	12785230	(G/C)
20433	CakSNP20433	*Kabuli*	Ca_Kabuli_denovo	12785482	(C/G)
20434	CakSNP20434	*Kabuli*	Ca_Kabuli_denovo	12785515	(A/G)
20435	CakSNP20435	*Kabuli*	Ca_Kabuli_denovo	12791066	(A/G)
20436	CakSNP20436	*Kabuli*	Ca_Kabuli_denovo	12791067	(A/G)
20437	CakSNP20437	*Kabuli*	Ca_Kabuli_denovo	12793473	(T/C)
20438	CakSNP20438	*Kabuli*	Ca_Kabuli_denovo	12793863	(G/A)
20439	CakSNP20439	*Kabuli*	Ca_Kabuli_denovo	12793884	(C/G)
20440	CakSNP20440	*Kabuli*	Ca_Kabuli_denovo	12795878	(C/T)
20441	CakSNP20441	*Kabuli*	Ca_Kabuli_denovo	12805396	(T/C)
20442	CakSNP20442	*Kabuli*	Ca_Kabuli_denovo	12812076	(C/G)
20443	CakSNP20443	*Kabuli*	Ca_Kabuli_denovo	12812111	(A/C)

S.N.	SNP IDs	Cultivars	Chromosomes/scaffolds	Physical positions (bp)	SNPs
20444	CakSNP20444	*Kabuli*	Ca_Kabuli_denovo	12817660	(C/T)
20445	CakSNP20445	*Kabuli*	Ca_Kabuli_denovo	12818175	(T/G)
20446	CakSNP20446	*Kabuli*	Ca_Kabuli_denovo	12829473	(G/A)
20447	CakSNP20447	*Kabuli*	Ca_Kabuli_denovo	12830550	(C/G)
20448	CakSNP20448	*Kabuli*	Ca_Kabuli_denovo	12830559	(G/A)
20449	CakSNP20449	*Kabuli*	Ca_Kabuli_denovo	12832898	(C/A)
20450	CakSNP20450	*Kabuli*	Ca_Kabuli_denovo	12832925	(C/G)
20451	CakSNP20451	*Kabuli*	Ca_Kabuli_denovo	12843699	(C/T)
20452	CakSNP20452	*Kabuli*	Ca_Kabuli_denovo	12843736	(T/A)
20453	CakSNP20453	*Kabuli*	Ca_Kabuli_denovo	12845113	(G/T)
20454	CakSNP20454	*Kabuli*	Ca_Kabuli_denovo	12848545	(T/C)
20455	CakSNP20455	*Kabuli*	Ca_Kabuli_denovo	12850100	(T/C)
20456	CakSNP20456	*Kabuli*	Ca_Kabuli_denovo	12850561	(A/G)
20457	CakSNP20457	*Kabuli*	Ca_Kabuli_denovo	12850591	(C/T)
20458	CakSNP20458	*Kabuli*	Ca_Kabuli_denovo	12854008	(A/G)
20459	CakSNP20459	*Kabuli*	Ca_Kabuli_denovo	12854011	(G/C)
20460	CakSNP20460	*Kabuli*	Ca_Kabuli_denovo	12856834	(T/G)
20461	CakSNP20461	*Kabuli*	Ca_Kabuli_denovo	12860525	(G/A)
20462	CakSNP20462	*Kabuli*	Ca_Kabuli_denovo	12862348	(T/G)
20463	CakSNP20463	*Kabuli*	Ca_Kabuli_denovo	12863560	(A/G)
20464	CakSNP20464	*Kabuli*	Ca_Kabuli_denovo	12865448	(C/T)
20465	CakSNP20465	*Kabuli*	Ca_Kabuli_denovo	12865468	(C/T)
20466	CakSNP20466	*Kabuli*	Ca_Kabuli_denovo	12869255	(A/G)
20467	CakSNP20467	*Kabuli*	Ca_Kabuli_denovo	12871791	(C/G)
20468	CakSNP20468	*Kabuli*	Ca_Kabuli_denovo	12872788	(G/A)
20469	CakSNP20469	*Kabuli*	Ca_Kabuli_denovo	12872842	(T/C)
20470	CakSNP20470	*Kabuli*	Ca_Kabuli_denovo	12876043	(A/G)
20471	CakSNP20471	*Kabuli*	Ca_Kabuli_denovo	12888592	(A/C)
20472	CakSNP20472	*Kabuli*	Ca_Kabuli_denovo	12897255	(A/G)
20473	CakSNP20473	*Kabuli*	Ca_Kabuli_denovo	12899024	(T/C)
20474	CakSNP20474	*Kabuli*	Ca_Kabuli_denovo	12903398	(C/A)
20475	CakSNP20475	*Kabuli*	Ca_Kabuli_denovo	12903437	(C/A)
20476	CakSNP20476	*Kabuli*	Ca_Kabuli_denovo	12904085	(T/C)
20477	CakSNP20477	*Kabuli*	Ca_Kabuli_denovo	12904118	(A/G)
20478	CakSNP20478	*Kabuli*	Ca_Kabuli_denovo	12910271	(C/T)
20479	CakSNP20479	*Kabuli*	Ca_Kabuli_denovo	12910286	(A/G)
20480	CakSNP20480	*Kabuli*	Ca_Kabuli_denovo	12911244	(T/G)
20481	CakSNP20481	*Kabuli*	Ca_Kabuli_denovo	12911275	(A/G)

S.N.	SNP IDs	Cultivars	Chromosomes/scaffolds	Physical positions (bp)	SNPs
20482	CakSNP20482	*Kabuli*	Ca_Kabuli_denovo	12914398	(C/T)
20483	CakSNP20483	*Kabuli*	Ca_Kabuli_denovo	12914424	(C/A)
20484	CakSNP20484	*Kabuli*	Ca_Kabuli_denovo	12927462	(A/G)
20485	CakSNP20485	*Kabuli*	Ca_Kabuli_denovo	12933253	(T/C)
20486	CakSNP20486	*Kabuli*	Ca_Kabuli_denovo	12935128	(T/C)
20487	CakSNP20487	*Kabuli*	Ca_Kabuli_denovo	12935673	(C/G)
20488	CakSNP20488	*Kabuli*	Ca_Kabuli_denovo	12936805	(T/C)
20489	CakSNP20489	*Kabuli*	Ca_Kabuli_denovo	12939572	(T/C)
20490	CakSNP20490	*Kabuli*	Ca_Kabuli_denovo	12939580	(G/A)
20491	CakSNP20491	*Kabuli*	Ca_Kabuli_denovo	12974203	(T/C)
20492	CakSNP20492	*Kabuli*	Ca_Kabuli_denovo	12975846	(T/C)
20493	CakSNP20493	*Kabuli*	Ca_Kabuli_denovo	12976965	(C/G)
20494	CakSNP20494	*Kabuli*	Ca_Kabuli_denovo	12977012	(C/G)
20495	CakSNP20495	*Kabuli*	Ca_Kabuli_denovo	12980401	(G/A)
20496	CakSNP20496	*Kabuli*	Ca_Kabuli_denovo	12980404	(G/C)
20497	CakSNP20497	*Kabuli*	Ca_Kabuli_denovo	12981713	(C/T)
20498	CakSNP20498	*Kabuli*	Ca_Kabuli_denovo	12983088	(A/G)
20499	CakSNP20499	*Kabuli*	Ca_Kabuli_denovo	12997717	(G/A)
20500	CakSNP20500	*Kabuli*	Ca_Kabuli_denovo	13000283	(A/C)
20501	CakSNP20501	*Kabuli*	Ca_Kabuli_denovo	13013951	(A/C)
20502	CakSNP20502	*Kabuli*	Ca_Kabuli_denovo	13013952	(G/A)
20503	CakSNP20503	*Kabuli*	Ca_Kabuli_denovo	13013955	(C/T)
20504	CakSNP20504	*Kabuli*	Ca_Kabuli_denovo	13014166	(T/C)
20505	CakSNP20505	*Kabuli*	Ca_Kabuli_denovo	13016388	(C/T)
20506	CakSNP20506	*Kabuli*	Ca_Kabuli_denovo	13016391	(G/C)
20507	CakSNP20507	*Kabuli*	Ca_Kabuli_denovo	13020665	(G/A)
20508	CakSNP20508	*Kabuli*	Ca_Kabuli_denovo	13021311	(G/A)
20509	CakSNP20509	*Kabuli*	Ca_Kabuli_denovo	13028389	(A/G)
20510	CakSNP20510	*Kabuli*	Ca_Kabuli_denovo	13028463	(G/A)
20511	CakSNP20511	*Kabuli*	Ca_Kabuli_denovo	13030260	(G/A)
20512	CakSNP20512	*Kabuli*	Ca_Kabuli_denovo	13031950	(A/C)
20513	CakSNP20513	*Kabuli*	Ca_Kabuli_denovo	13032980	(G/A)
20514	CakSNP20514	*Kabuli*	Ca_Kabuli_denovo	13033007	(C/T)
20515	CakSNP20515	*Kabuli*	Ca_Kabuli_denovo	13044230	(G/C)
20516	CakSNP20516	*Kabuli*	Ca_Kabuli_denovo	13047420	(G/A)
20517	CakSNP20517	*Kabuli*	Ca_Kabuli_denovo	13048862	(C/T)
20518	CakSNP20518	*Kabuli*	Ca_Kabuli_denovo	13049519	(T/C)
20519	CakSNP20519	*Kabuli*	Ca_Kabuli_denovo	13051397	(C/G)

S.N.	SNP IDs	Cultivars	Chromosomes/scaffolds	Physical positions (bp)	SNPs
20520	CakSNP20520	*Kabuli*	Ca_Kabuli_denovo	13055832	(A/G)
20521	CakSNP20521	*Kabuli*	Ca_Kabuli_denovo	13060385	(C/A)
20522	CakSNP20522	*Kabuli*	Ca_Kabuli_denovo	13060407	(G/C)
20523	CakSNP20523	*Kabuli*	Ca_Kabuli_denovo	13064433	(G/A)
20524	CakSNP20524	*Kabuli*	Ca_Kabuli_denovo	13065262	(G/C)
20525	CakSNP20525	*Kabuli*	Ca_Kabuli_denovo	13082032	(G/C)
20526	CakSNP20526	*Kabuli*	Ca_Kabuli_denovo	13088614	(G/C)
20527	CakSNP20527	*Kabuli*	Ca_Kabuli_denovo	13101486	(C/G)
20528	CakSNP20528	*Kabuli*	Ca_Kabuli_denovo	13101540	(G/C)
20529	CakSNP20529	*Kabuli*	Ca_Kabuli_denovo	13107551	(T/G)
20530	CakSNP20530	*Kabuli*	Ca_Kabuli_denovo	13108297	(C/T)
20531	CakSNP20531	*Kabuli*	Ca_Kabuli_denovo	13108376	(A/G)
20532	CakSNP20532	*Kabuli*	Ca_Kabuli_denovo	13109480	(G/C)
20533	CakSNP20533	*Kabuli*	Ca_Kabuli_denovo	13109533	(C/G)
20534	CakSNP20534	*Kabuli*	Ca_Kabuli_denovo	13113390	(A/T)
20535	CakSNP20535	*Kabuli*	Ca_Kabuli_denovo	13113393	(T/C)
20536	CakSNP20536	*Kabuli*	Ca_Kabuli_denovo	13113408	(G/A)
20537	CakSNP20537	*Kabuli*	Ca_Kabuli_denovo	13114873	(A/C)
20538	CakSNP20538	*Kabuli*	Ca_Kabuli_denovo	13115242	(T/C)
20539	CakSNP20539	*Kabuli*	Ca_Kabuli_denovo	13117588	(T/G)
20540	CakSNP20540	*Kabuli*	Ca_Kabuli_denovo	13117614	(A/G)
20541	CakSNP20541	*Kabuli*	Ca_Kabuli_denovo	13119370	(T/C)
20542	CakSNP20542	*Kabuli*	Ca_Kabuli_denovo	13121866	(T/C)
20543	CakSNP20543	*Kabuli*	Ca_Kabuli_denovo	13121875	(A/G)
20544	CakSNP20544	*Kabuli*	Ca_Kabuli_denovo	13123297	(A/G)
20545	CakSNP20545	*Kabuli*	Ca_Kabuli_denovo	13124552	(A/G)
20546	CakSNP20546	*Kabuli*	Ca_Kabuli_denovo	13124612	(G/A)
20547	CakSNP20547	*Kabuli*	Ca_Kabuli_denovo	13127534	(G/T)
20548	CakSNP20548	*Kabuli*	Ca_Kabuli_denovo	13127558	(C/G)
20549	CakSNP20549	*Kabuli*	Ca_Kabuli_denovo	13131624	(G/A)
20550	CakSNP20550	*Kabuli*	Ca_Kabuli_denovo	13131674	(G/A)
20551	CakSNP20551	*Kabuli*	Ca_Kabuli_denovo	13138680	(A/G)
20552	CakSNP20552	*Kabuli*	Ca_Kabuli_denovo	13138756	(G/A)
20553	CakSNP20553	*Kabuli*	Ca_Kabuli_denovo	13141952	(T/C)
20554	CakSNP20554	*Kabuli*	Ca_Kabuli_denovo	13143666	(C/G)
20555	CakSNP20555	*Kabuli*	Ca_Kabuli_denovo	13145752	(T/G)
20556	CakSNP20556	*Kabuli*	Ca_Kabuli_denovo	13145765	(C/T)
20557	CakSNP20557	*Kabuli*	Ca_Kabuli_denovo	13146212	(A/T)

S.N.	SNP IDs	Cultivars	Chromosomes/scaffolds	Physical positions (bp)	SNPs
20558	CakSNP20558	*Kabuli*	Ca_Kabuli_denovo	13146263	(T/A)
20559	CakSNP20559	*Kabuli*	Ca_Kabuli_denovo	13147196	(A/G)
20560	CakSNP20560	*Kabuli*	Ca_Kabuli_denovo	13147202	(G/A)
20561	CakSNP20561	*Kabuli*	Ca_Kabuli_denovo	13155547	(G/T)
20562	CakSNP20562	*Kabuli*	Ca_Kabuli_denovo	13162784	(G/C)
20563	CakSNP20563	*Kabuli*	Ca_Kabuli_denovo	13169513	(C/T)
20564	CakSNP20564	*Kabuli*	Ca_Kabuli_denovo	13169527	(C/T)
20565	CakSNP20565	*Kabuli*	Ca_Kabuli_denovo	13171259	(A/G)
20566	CakSNP20566	*Kabuli*	Ca_Kabuli_denovo	13171283	(A/G)
20567	CakSNP20567	*Kabuli*	Ca_Kabuli_denovo	13172040	(A/G)
20568	CakSNP20568	*Kabuli*	Ca_Kabuli_denovo	13173271	(C/T)
20569	CakSNP20569	*Kabuli*	Ca_Kabuli_denovo	13173276	(G/A)
20570	CakSNP20570	*Kabuli*	Ca_Kabuli_denovo	13178980	(C/G)
20571	CakSNP20571	*Kabuli*	Ca_Kabuli_denovo	13179014	(C/G)
20572	CakSNP20572	*Kabuli*	Ca_Kabuli_denovo	13182701	(C/G)
20573	CakSNP20573	*Kabuli*	Ca_Kabuli_denovo	13184993	(G/C)
20574	CakSNP20574	*Kabuli*	Ca_Kabuli_denovo	13187926	(C/T)
20575	CakSNP20575	*Kabuli*	Ca_Kabuli_denovo	13189933	(A/G)
20576	CakSNP20576	*Kabuli*	Ca_Kabuli_denovo	13194655	(A/C)
20577	CakSNP20577	*Kabuli*	Ca_Kabuli_denovo	13198712	(T/C)
20578	CakSNP20578	*Kabuli*	Ca_Kabuli_denovo	13198718	(C/T)
20579	CakSNP20579	*Kabuli*	Ca_Kabuli_denovo	13203576	(T/G)
20580	CakSNP20580	*Kabuli*	Ca_Kabuli_denovo	13205570	(T/C)
20581	CakSNP20581	*Kabuli*	Ca_Kabuli_denovo	13205676	(A/C)
20582	CakSNP20582	*Kabuli*	Ca_Kabuli_denovo	13206342	(G/C)
20583	CakSNP20583	*Kabuli*	Ca_Kabuli_denovo	13208511	(A/C)
20584	CakSNP20584	*Kabuli*	Ca_Kabuli_denovo	13208512	(G/A)
20585	CakSNP20585	*Kabuli*	Ca_Kabuli_denovo	13211264	(A/G)
20586	CakSNP20586	*Kabuli*	Ca_Kabuli_denovo	13219900	(G/T)
20587	CakSNP20587	*Kabuli*	Ca_Kabuli_denovo	13220434	(C/T)
20588	CakSNP20588	*Kabuli*	Ca_Kabuli_denovo	13220440	(A/G)
20589	CakSNP20589	*Kabuli*	Ca_Kabuli_denovo	13223422	(C/G)
20590	CakSNP20590	*Kabuli*	Ca_Kabuli_denovo	13223458	(C/T)
20591	CakSNP20591	*Kabuli*	Ca_Kabuli_denovo	13233187	(T/A)
20592	CakSNP20592	*Kabuli*	Ca_Kabuli_denovo	13233773	(C/T)
20593	CakSNP20593	*Kabuli*	Ca_Kabuli_denovo	13236038	(A/C)
20594	CakSNP20594	*Kabuli*	Ca_Kabuli_denovo	13236125	(T/C)
20595	CakSNP20595	*Kabuli*	Ca_Kabuli_denovo	13236608	(A/G)

S.N.	SNP IDs	Cultivars	Chromosomes/scaffolds	Physical positions (bp)	SNPs
20596	CakSNP20596	*Kabuli*	Ca_Kabuli_denovo	13243606	(G/T)
20597	CakSNP20597	*Kabuli*	Ca_Kabuli_denovo	13243624	(C/A)
20598	CakSNP20598	*Kabuli*	Ca_Kabuli_denovo	13255115	(G/A)
20599	CakSNP20599	*Kabuli*	Ca_Kabuli_denovo	13256716	(C/T)
20600	CakSNP20600	*Kabuli*	Ca_Kabuli_denovo	13257182	(A/G)
20601	CakSNP20601	*Kabuli*	Ca_Kabuli_denovo	13257218	(C/T)
20602	CakSNP20602	*Kabuli*	Ca_Kabuli_denovo	13257741	(G/C)
20603	CakSNP20603	*Kabuli*	Ca_Kabuli_denovo	13257752	(C/T)
20604	CakSNP20604	*Kabuli*	Ca_Kabuli_denovo	13262646	(C/T)
20605	CakSNP20605	*Kabuli*	Ca_Kabuli_denovo	13262682	(G/C)
20606	CakSNP20606	*Kabuli*	Ca_Kabuli_denovo	13263857	(C/G)
20607	CakSNP20607	*Kabuli*	Ca_Kabuli_denovo	13263881	(T/C)
20608	CakSNP20608	*Kabuli*	Ca_Kabuli_denovo	13265069	(T/C)
20609	CakSNP20609	*Kabuli*	Ca_Kabuli_denovo	13265087	(G/C)
20610	CakSNP20610	*Kabuli*	Ca_Kabuli_denovo	13265898	(A/G)
20611	CakSNP20611	*Kabuli*	Ca_Kabuli_denovo	13265931	(G/A)
20612	CakSNP20612	*Kabuli*	Ca_Kabuli_denovo	13266100	(T/C)
20613	CakSNP20613	*Kabuli*	Ca_Kabuli_denovo	13266130	(A/T)
20614	CakSNP20614	*Kabuli*	Ca_Kabuli_denovo	13268055	(T/C)
20615	CakSNP20615	*Kabuli*	Ca_Kabuli_denovo	13268076	(G/T)
20616	CakSNP20616	*Kabuli*	Ca_Kabuli_denovo	13271245	(A/G)
20617	CakSNP20617	*Kabuli*	Ca_Kabuli_denovo	13271253	(T/C)
20618	CakSNP20618	*Kabuli*	Ca_Kabuli_denovo	13274178	(C/A)
20619	CakSNP20619	*Kabuli*	Ca_Kabuli_denovo	13274216	(C/G)
20620	CakSNP20620	*Kabuli*	Ca_Kabuli_denovo	13281091	(T/A)
20621	CakSNP20621	*Kabuli*	Ca_Kabuli_denovo	13283664	(C/A)
20622	CakSNP20622	*Kabuli*	Ca_Kabuli_denovo	13291126	(C/A)
20623	CakSNP20623	*Kabuli*	Ca_Kabuli_denovo	13298005	(C/T)
20624	CakSNP20624	*Kabuli*	Ca_Kabuli_denovo	13299371	(A/T)
20625	CakSNP20625	*Kabuli*	Ca_Kabuli_denovo	13299943	(G/A)
20626	CakSNP20626	*Kabuli*	Ca_Kabuli_denovo	13300502	(C/T)
20627	CakSNP20627	*Kabuli*	Ca_Kabuli_denovo	13300525	(C/T)
20628	CakSNP20628	*Kabuli*	Ca_Kabuli_denovo	13302088	(G/T)
20629	CakSNP20629	*Kabuli*	Ca_Kabuli_denovo	13302097	(A/G)
20630	CakSNP20630	*Kabuli*	Ca_Kabuli_denovo	13305822	(C/T)
20631	CakSNP20631	*Kabuli*	Ca_Kabuli_denovo	13309255	(A/G)
20632	CakSNP20632	*Kabuli*	Ca_Kabuli_denovo	13309265	(T/C)
20633	CakSNP20633	*Kabuli*	Ca_Kabuli_denovo	13318048	(T/C)

S.N.	SNP IDs	Cultivars	Chromosomes/scaffolds	Physical positions (bp)	SNPs
20634	CakSNP20634	*Kabuli*	Ca_Kabuli_denovo	13321431	(G/C)
20635	CakSNP20635	*Kabuli*	Ca_Kabuli_denovo	13321444	(T/A)
20636	CakSNP20636	*Kabuli*	Ca_Kabuli_denovo	13326443	(T/A)
20637	CakSNP20637	*Kabuli*	Ca_Kabuli_denovo	13326470	(A/G)
20638	CakSNP20638	*Kabuli*	Ca_Kabuli_denovo	13331285	(A/G)
20639	CakSNP20639	*Kabuli*	Ca_Kabuli_denovo	13331312	(C/G)
20640	CakSNP20640	*Kabuli*	Ca_Kabuli_denovo	13338641	(A/C)
20641	CakSNP20641	*Kabuli*	Ca_Kabuli_denovo	13338649	(T/G)
20642	CakSNP20642	*Kabuli*	Ca_Kabuli_denovo	13342906	(A/G)
20643	CakSNP20643	*Kabuli*	Ca_Kabuli_denovo	13347586	(G/C)
20644	CakSNP20644	*Kabuli*	Ca_Kabuli_denovo	13347619	(C/T)
20645	CakSNP20645	*Kabuli*	Ca_Kabuli_denovo	13350368	(G/C)
20646	CakSNP20646	*Kabuli*	Ca_Kabuli_denovo	13350390	(A/G)
20647	CakSNP20647	*Kabuli*	Ca_Kabuli_denovo	13350733	(A/G)
20648	CakSNP20648	*Kabuli*	Ca_Kabuli_denovo	13350766	(T/C)
20649	CakSNP20649	*Kabuli*	Ca_Kabuli_denovo	13351946	(A/G)
20650	CakSNP20650	*Kabuli*	Ca_Kabuli_denovo	13354654	(C/G)
20651	CakSNP20651	*Kabuli*	Ca_Kabuli_denovo	13361155	(T/C)
20652	CakSNP20652	*Kabuli*	Ca_Kabuli_denovo	13368691	(G/T)
20653	CakSNP20653	*Kabuli*	Ca_Kabuli_denovo	13373609	(C/T)
20654	CakSNP20654	*Kabuli*	Ca_Kabuli_denovo	13373661	(G/A)
20655	CakSNP20655	*Kabuli*	Ca_Kabuli_denovo	13375276	(T/C)
20656	CakSNP20656	*Kabuli*	Ca_Kabuli_denovo	13376177	(C/T)
20657	CakSNP20657	*Kabuli*	Ca_Kabuli_denovo	13376187	(C/T)
20658	CakSNP20658	*Kabuli*	Ca_Kabuli_denovo	13390756	(T/C)
20659	CakSNP20659	*Kabuli*	Ca_Kabuli_denovo	13390759	(A/G)
20660	CakSNP20660	*Kabuli*	Ca_Kabuli_denovo	13400505	(T/C)
20661	CakSNP20661	*Kabuli*	Ca_Kabuli_denovo	13400514	(C/A)
20662	CakSNP20662	*Kabuli*	Ca_Kabuli_denovo	13402676	(C/T)
20663	CakSNP20663	*Kabuli*	Ca_Kabuli_denovo	13402685	(C/T)
20664	CakSNP20664	*Kabuli*	Ca_Kabuli_denovo	13402948	(T/C)
20665	CakSNP20665	*Kabuli*	Ca_Kabuli_denovo	13402956	(G/C)
20666	CakSNP20666	*Kabuli*	Ca_Kabuli_denovo	13402957	(C/T)
20667	CakSNP20667	*Kabuli*	Ca_Kabuli_denovo	13403374	(C/T)
20668	CakSNP20668	*Kabuli*	Ca_Kabuli_denovo	13404299	(C/T)
20669	CakSNP20669	*Kabuli*	Ca_Kabuli_denovo	13405999	(T/C)
20670	CakSNP20670	*Kabuli*	Ca_Kabuli_denovo	13408133	(G/A)
20671	CakSNP20671	*Kabuli*	Ca_Kabuli_denovo	13413523	(C/T)

S.N.	SNP IDs	Cultivars	Chromosomes/scaffolds	Physical positions (bp)	SNPs
20672	CakSNP20672	*Kabuli*	Ca_Kabuli_denovo	13421129	(A/G)
20673	CakSNP20673	*Kabuli*	Ca_Kabuli_denovo	13423674	(G/A)
20674	CakSNP20674	*Kabuli*	Ca_Kabuli_denovo	13424125	(A/T)
20675	CakSNP20675	*Kabuli*	Ca_Kabuli_denovo	13425959	(C/G)
20676	CakSNP20676	*Kabuli*	Ca_Kabuli_denovo	13431139	(T/C)
20677	CakSNP20677	*Kabuli*	Ca_Kabuli_denovo	13434517	(C/G)
20678	CakSNP20678	*Kabuli*	Ca_Kabuli_denovo	13437430	(T/C)
20679	CakSNP20679	*Kabuli*	Ca_Kabuli_denovo	13438296	(C/A)
20680	CakSNP20680	*Kabuli*	Ca_Kabuli_denovo	13444653	(A/G)
20681	CakSNP20681	*Kabuli*	Ca_Kabuli_denovo	13449681	(C/T)
20682	CakSNP20682	*Kabuli*	Ca_Kabuli_denovo	13450012	(G/A)
20683	CakSNP20683	*Kabuli*	Ca_Kabuli_denovo	13451539	(T/C)
20684	CakSNP20684	*Kabuli*	Ca_Kabuli_denovo	13451572	(G/T)
20685	CakSNP20685	*Kabuli*	Ca_Kabuli_denovo	13452839	(A/G)
20686	CakSNP20686	*Kabuli*	Ca_Kabuli_denovo	13462372	(A/C)
20687	CakSNP20687	*Kabuli*	Ca_Kabuli_denovo	13465634	(C/G)
20688	CakSNP20688	*Kabuli*	Ca_Kabuli_denovo	13467173	(G/A)
20689	CakSNP20689	*Kabuli*	Ca_Kabuli_denovo	13475729	(C/T)
20690	CakSNP20690	*Kabuli*	Ca_Kabuli_denovo	13475780	(T/C)
20691	CakSNP20691	*Kabuli*	Ca_Kabuli_denovo	13477849	(T/C)
20692	CakSNP20692	*Kabuli*	Ca_Kabuli_denovo	13477879	(C/T)
20693	CakSNP20693	*Kabuli*	Ca_Kabuli_denovo	13482416	(C/T)
20694	CakSNP20694	*Kabuli*	Ca_Kabuli_denovo	13483653	(C/G)
20695	CakSNP20695	*Kabuli*	Ca_Kabuli_denovo	13483915	(C/T)
20696	CakSNP20696	*Kabuli*	Ca_Kabuli_denovo	13483957	(C/T)
20697	CakSNP20697	*Kabuli*	Ca_Kabuli_denovo	13484597	(G/A)
20698	CakSNP20698	*Kabuli*	Ca_Kabuli_denovo	13486160	(G/A)
20699	CakSNP20699	*Kabuli*	Ca_Kabuli_denovo	13486322	(C/T)
20700	CakSNP20700	*Kabuli*	Ca_Kabuli_denovo	13487264	(C/T)
20701	CakSNP20701	*Kabuli*	Ca_Kabuli_denovo	13487276	(C/T)
20702	CakSNP20702	*Kabuli*	Ca_Kabuli_denovo	13489767	(C/G)
20703	CakSNP20703	*Kabuli*	Ca_Kabuli_denovo	13489770	(A/G)
20704	CakSNP20704	*Kabuli*	Ca_Kabuli_denovo	13489794	(C/T)
20705	CakSNP20705	*Kabuli*	Ca_Kabuli_denovo	13494557	(G/C)
20706	CakSNP20706	*Kabuli*	Ca_Kabuli_denovo	13494571	(A/G)
20707	CakSNP20707	*Kabuli*	Ca_Kabuli_denovo	13500382	(C/A)
20708	CakSNP20708	*Kabuli*	Ca_Kabuli_denovo	13511809	(A/G)
20709	CakSNP20709	*Kabuli*	Ca_Kabuli_denovo	13511857	(A/G)

S.N.	SNP IDs	Cultivars	Chromosomes/scaffolds	Physical positions (bp)	SNPs
20710	CakSNP20710	*Kabuli*	Ca_Kabuli_denovo	13515765	(T/C)
20711	CakSNP20711	*Kabuli*	Ca_Kabuli_denovo	13528252	(G/A)
20712	CakSNP20712	*Kabuli*	Ca_Kabuli_denovo	13528261	(G/A)
20713	CakSNP20713	*Kabuli*	Ca_Kabuli_denovo	13536814	(A/G)
20714	CakSNP20714	*Kabuli*	Ca_Kabuli_denovo	13536909	(C/T)
20715	CakSNP20715	*Kabuli*	Ca_Kabuli_denovo	13536915	(G/C)
20716	CakSNP20716	*Kabuli*	Ca_Kabuli_denovo	13539057	(G/C)
20717	CakSNP20717	*Kabuli*	Ca_Kabuli_denovo	13544201	(G/A)
20718	CakSNP20718	*Kabuli*	Ca_Kabuli_denovo	13550350	(T/C)
20719	CakSNP20719	*Kabuli*	Ca_Kabuli_denovo	13560452	(G/A)
20720	CakSNP20720	*Kabuli*	Ca_Kabuli_denovo	13565367	(C/G)
20721	CakSNP20721	*Kabuli*	Ca_Kabuli_denovo	13565390	(A/G)
20722	CakSNP20722	*Kabuli*	Ca_Kabuli_denovo	13575988	(T/C)
20723	CakSNP20723	*Kabuli*	Ca_Kabuli_denovo	13576027	(G/T)
20724	CakSNP20724	*Kabuli*	Ca_Kabuli_denovo	13602770	(G/T)
20725	CakSNP20725	*Kabuli*	Ca_Kabuli_denovo	13604914	(T/C)
20726	CakSNP20726	*Kabuli*	Ca_Kabuli_denovo	13604935	(C/T)
20727	CakSNP20727	*Kabuli*	Ca_Kabuli_denovo	13606541	(A/G)
20728	CakSNP20728	*Kabuli*	Ca_Kabuli_denovo	13613180	(T/C)
20729	CakSNP20729	*Kabuli*	Ca_Kabuli_denovo	13613224	(A/G)
20730	CakSNP20730	*Kabuli*	Ca_Kabuli_denovo	13613225	(A/G)
20731	CakSNP20731	*Kabuli*	Ca_Kabuli_denovo	13613231	(C/G)
20732	CakSNP20732	*Kabuli*	Ca_Kabuli_denovo	13617653	(C/T)
20733	CakSNP20733	*Kabuli*	Ca_Kabuli_denovo	13617683	(A/T)
20734	CakSNP20734	*Kabuli*	Ca_Kabuli_denovo	13623029	(G/A)
20735	CakSNP20735	*Kabuli*	Ca_Kabuli_denovo	13623080	(G/C)
20736	CakSNP20736	*Kabuli*	Ca_Kabuli_denovo	13625592	(C/G)
20737	CakSNP20737	*Kabuli*	Ca_Kabuli_denovo	13625871	(G/T)
20738	CakSNP20738	*Kabuli*	Ca_Kabuli_denovo	13626117	(C/T)
20739	CakSNP20739	*Kabuli*	Ca_Kabuli_denovo	13629263	(T/C)
20740	CakSNP20740	*Kabuli*	Ca_Kabuli_denovo	13629308	(C/T)
20741	CakSNP20741	*Kabuli*	Ca_Kabuli_denovo	13632321	(T/C)
20742	CakSNP20742	*Kabuli*	Ca_Kabuli_denovo	13632391	(A/G)
20743	CakSNP20743	*Kabuli*	Ca_Kabuli_denovo	13641188	(A/G)
20744	CakSNP20744	*Kabuli*	Ca_Kabuli_denovo	13641203	(G/A)
20745	CakSNP20745	*Kabuli*	Ca_Kabuli_denovo	13641400	(G/A)
20746	CakSNP20746	*Kabuli*	Ca_Kabuli_denovo	13643385	(G/C)
20747	CakSNP20747	*Kabuli*	Ca_Kabuli_denovo	13643444	(A/G)

S.N.	SNP IDs	Cultivars	Chromosomes/scaffolds	Physical positions (bp)	SNPs
20748	CakSNP20748	*Kabuli*	Ca_Kabuli_denovo	13651337	(T/G)
20749	CakSNP20749	*Kabuli*	Ca_Kabuli_denovo	13655503	(A/G)
20750	CakSNP20750	*Kabuli*	Ca_Kabuli_denovo	13655518	(A/G)
20751	CakSNP20751	*Kabuli*	Ca_Kabuli_denovo	13666470	(C/T)
20752	CakSNP20752	*Kabuli*	Ca_Kabuli_denovo	13666480	(A/G)
20753	CakSNP20753	*Kabuli*	Ca_Kabuli_denovo	13673377	(T/C)
20754	CakSNP20754	*Kabuli*	Ca_Kabuli_denovo	13675125	(C/T)
20755	CakSNP20755	*Kabuli*	Ca_Kabuli_denovo	13675292	(G/A)
20756	CakSNP20756	*Kabuli*	Ca_Kabuli_denovo	13677592	(A/C)
20757	CakSNP20757	*Kabuli*	Ca_Kabuli_denovo	13687869	(G/C)
20758	CakSNP20758	*Kabuli*	Ca_Kabuli_denovo	13691846	(G/A)
20759	CakSNP20759	*Kabuli*	Ca_Kabuli_denovo	13691919	(T/A)
20760	CakSNP20760	*Kabuli*	Ca_Kabuli_denovo	13698536	(C/T)
20761	CakSNP20761	*Kabuli*	Ca_Kabuli_denovo	13698551	(G/C)
20762	CakSNP20762	*Kabuli*	Ca_Kabuli_denovo	13701338	(G/C)
20763	CakSNP20763	*Kabuli*	Ca_Kabuli_denovo	13701377	(C/T)
20764	CakSNP20764	*Kabuli*	Ca_Kabuli_denovo	13704680	(T/C)
20765	CakSNP20765	*Kabuli*	Ca_Kabuli_denovo	13706750	(T/C)
20766	CakSNP20766	*Kabuli*	Ca_Kabuli_denovo	13706908	(T/C)
20767	CakSNP20767	*Kabuli*	Ca_Kabuli_denovo	13706944	(C/T)
20768	CakSNP20768	*Kabuli*	Ca_Kabuli_denovo	13709609	(A/G)
20769	CakSNP20769	*Kabuli*	Ca_Kabuli_denovo	13710121	(G/T)
20770	CakSNP20770	*Kabuli*	Ca_Kabuli_denovo	13711298	(T/C)
20771	CakSNP20771	*Kabuli*	Ca_Kabuli_denovo	13711325	(C/G)
20772	CakSNP20772	*Kabuli*	Ca_Kabuli_denovo	13718021	(G/A)
20773	CakSNP20773	*Kabuli*	Ca_Kabuli_denovo	13721788	(T/C)
20774	CakSNP20774	*Kabuli*	Ca_Kabuli_denovo	13721815	(C/T)
20775	CakSNP20775	*Kabuli*	Ca_Kabuli_denovo	13732598	(C/T)
20776	CakSNP20776	*Kabuli*	Ca_Kabuli_denovo	13732658	(C/T)
20777	CakSNP20777	*Kabuli*	Ca_Kabuli_denovo	13736037	(G/A)
20778	CakSNP20778	*Kabuli*	Ca_Kabuli_denovo	13736092	(C/G)
20779	CakSNP20779	*Kabuli*	Ca_Kabuli_denovo	13739207	(A/G)
20780	CakSNP20780	*Kabuli*	Ca_Kabuli_denovo	13739213	(C/T)
20781	CakSNP20781	*Kabuli*	Ca_Kabuli_denovo	13740208	(A/G)
20782	CakSNP20782	*Kabuli*	Ca_Kabuli_denovo	13745703	(T/C)
20783	CakSNP20783	*Kabuli*	Ca_Kabuli_denovo	13745704	(T/C)
20784	CakSNP20784	*Kabuli*	Ca_Kabuli_denovo	13757579	(G/T)
20785	CakSNP20785	*Kabuli*	Ca_Kabuli_denovo	13762433	(G/A)

S.N.	SNP IDs	Cultivars	Chromosomes/scaffolds	Physical positions (bp)	SNPs
20786	CakSNP20786	*Kabuli*	Ca_Kabuli_denovo	13762450	(C/T)
20787	CakSNP20787	*Kabuli*	Ca_Kabuli_denovo	13764490	(C/A)
20788	CakSNP20788	*Kabuli*	Ca_Kabuli_denovo	13770447	(C/T)
20789	CakSNP20789	*Kabuli*	Ca_Kabuli_denovo	13770484	(T/C)
20790	CakSNP20790	*Kabuli*	Ca_Kabuli_denovo	13788645	(T/G)
20791	CakSNP20791	*Kabuli*	Ca_Kabuli_denovo	13788697	(G/A)
20792	CakSNP20792	*Kabuli*	Ca_Kabuli_denovo	13788721	(T/C)
20793	CakSNP20793	*Kabuli*	Ca_Kabuli_denovo	13794543	(T/C)
20794	CakSNP20794	*Kabuli*	Ca_Kabuli_denovo	13799629	(G/C)
20795	CakSNP20795	*Kabuli*	Ca_Kabuli_denovo	13799704	(T/C)
20796	CakSNP20796	*Kabuli*	Ca_Kabuli_denovo	13817434	(G/A)
20797	CakSNP20797	*Kabuli*	Ca_Kabuli_denovo	13819277	(C/A)
20798	CakSNP20798	*Kabuli*	Ca_Kabuli_denovo	13819286	(G/C)
20799	CakSNP20799	*Kabuli*	Ca_Kabuli_denovo	13820035	(C/T)
20800	CakSNP20800	*Kabuli*	Ca_Kabuli_denovo	13826976	(G/T)
20801	CakSNP20801	*Kabuli*	Ca_Kabuli_denovo	13826995	(G/A)
20802	CakSNP20802	*Kabuli*	Ca_Kabuli_denovo	13831242	(A/T)
20803	CakSNP20803	*Kabuli*	Ca_Kabuli_denovo	13838447	(T/C)
20804	CakSNP20804	*Kabuli*	Ca_Kabuli_denovo	13841687	(C/T)
20805	CakSNP20805	*Kabuli*	Ca_Kabuli_denovo	13841700	(A/G)
20806	CakSNP20806	*Kabuli*	Ca_Kabuli_denovo	13841822	(T/G)
20807	CakSNP20807	*Kabuli*	Ca_Kabuli_denovo	13849092	(C/T)
20808	CakSNP20808	*Kabuli*	Ca_Kabuli_denovo	13851470	(A/G)
20809	CakSNP20809	*Kabuli*	Ca_Kabuli_denovo	13854412	(G/C)
20810	CakSNP20810	*Kabuli*	Ca_Kabuli_denovo	13868476	(A/C)
20811	CakSNP20811	*Kabuli*	Ca_Kabuli_denovo	13868494	(C/G)
20812	CakSNP20812	*Kabuli*	Ca_Kabuli_denovo	13868588	(G/A)
20813	CakSNP20813	*Kabuli*	Ca_Kabuli_denovo	13870026	(T/C)
20814	CakSNP20814	*Kabuli*	Ca_Kabuli_denovo	13870068	(A/C)
20815	CakSNP20815	*Kabuli*	Ca_Kabuli_denovo	13878412	(A/C)
20816	CakSNP20816	*Kabuli*	Ca_Kabuli_denovo	13879822	(G/T)
20817	CakSNP20817	*Kabuli*	Ca_Kabuli_denovo	13882414	(G/A)
20818	CakSNP20818	*Kabuli*	Ca_Kabuli_denovo	13882450	(T/C)
20819	CakSNP20819	*Kabuli*	Ca_Kabuli_denovo	13884785	(T/C)
20820	CakSNP20820	*Kabuli*	Ca_Kabuli_denovo	13885854	(T/C)
20821	CakSNP20821	*Kabuli*	Ca_Kabuli_denovo	13892083	(A/G)
20822	CakSNP20822	*Kabuli*	Ca_Kabuli_denovo	13892084	(C/A)
20823	CakSNP20823	*Kabuli*	Ca_Kabuli_denovo	13898027	(G/A)

S.N.	SNP IDs	Cultivars	Chromosomes/scaffolds	Physical positions (bp)	SNPs
20824	CakSNP20824	*Kabuli*	Ca_Kabuli_denovo	13901395	(T/C)
20825	CakSNP20825	*Kabuli*	Ca_Kabuli_denovo	13902570	(A/C)
20826	CakSNP20826	*Kabuli*	Ca_Kabuli_denovo	13902571	(A/G)
20827	CakSNP20827	*Kabuli*	Ca_Kabuli_denovo	13911052	(T/A)
20828	CakSNP20828	*Kabuli*	Ca_Kabuli_denovo	13911082	(G/A)
20829	CakSNP20829	*Kabuli*	Ca_Kabuli_denovo	13912343	(T/C)
20830	CakSNP20830	*Kabuli*	Ca_Kabuli_denovo	13912361	(A/G)
20831	CakSNP20831	*Kabuli*	Ca_Kabuli_denovo	13915445	(G/A)
20832	CakSNP20832	*Kabuli*	Ca_Kabuli_denovo	13928830	(C/T)
20833	CakSNP20833	*Kabuli*	Ca_Kabuli_denovo	13935332	(G/A)
20834	CakSNP20834	*Kabuli*	Ca_Kabuli_denovo	13938219	(G/A)
20835	CakSNP20835	*Kabuli*	Ca_Kabuli_denovo	13938220	(G/A)
20836	CakSNP20836	*Kabuli*	Ca_Kabuli_denovo	13942437	(G/A)
20837	CakSNP20837	*Kabuli*	Ca_Kabuli_denovo	13942467	(G/A)
20838	CakSNP20838	*Kabuli*	Ca_Kabuli_denovo	13945517	(C/T)
20839	CakSNP20839	*Kabuli*	Ca_Kabuli_denovo	13948830	(T/C)
20840	CakSNP20840	*Kabuli*	Ca_Kabuli_denovo	13955773	(T/C)
20841	CakSNP20841	*Kabuli*	Ca_Kabuli_denovo	13955776	(G/C)
20842	CakSNP20842	*Kabuli*	Ca_Kabuli_denovo	13958314	(A/T)
20843	CakSNP20843	*Kabuli*	Ca_Kabuli_denovo	13960720	(C/T)
20844	CakSNP20844	*Kabuli*	Ca_Kabuli_denovo	13963694	(C/A)
20845	CakSNP20845	*Kabuli*	Ca_Kabuli_denovo	13963760	(A/G)
20846	CakSNP20846	*Kabuli*	Ca_Kabuli_denovo	13965291	(C/G)
20847	CakSNP20847	*Kabuli*	Ca_Kabuli_denovo	13965316	(C/T)
20848	CakSNP20848	*Kabuli*	Ca_Kabuli_denovo	13974263	(G/A)
20849	CakSNP20849	*Kabuli*	Ca_Kabuli_denovo	13977184	(G/A)
20850	CakSNP20850	*Kabuli*	Ca_Kabuli_denovo	13978845	(A/G)
20851	CakSNP20851	*Kabuli*	Ca_Kabuli_denovo	13978865	(A/G)
20852	CakSNP20852	*Kabuli*	Ca_Kabuli_denovo	13978866	(C/G)
20853	CakSNP20853	*Kabuli*	Ca_Kabuli_denovo	13980992	(G/A)
20854	CakSNP20854	*Kabuli*	Ca_Kabuli_denovo	13981007	(T/C)
20855	CakSNP20855	*Kabuli*	Ca_Kabuli_denovo	13988791	(G/C)
20856	CakSNP20856	*Kabuli*	Ca_Kabuli_denovo	13988810	(C/G)
20857	CakSNP20857	*Kabuli*	Ca_Kabuli_denovo	13989848	(A/G)
20858	CakSNP20858	*Kabuli*	Ca_Kabuli_denovo	13989891	(G/A)
20859	CakSNP20859	*Kabuli*	Ca_Kabuli_denovo	13991399	(T/C)
20860	CakSNP20860	*Kabuli*	Ca_Kabuli_denovo	13991444	(A/G)
20861	CakSNP20861	*Kabuli*	Ca_Kabuli_denovo	13991699	(A/G)

S.N.	SNP IDs	Cultivars	Chromosomes/scaffolds	Physical positions (bp)	SNPs
20862	CakSNP20862	*Kabuli*	Ca_Kabuli_denovo	13993112	(G/A)
20863	CakSNP20863	*Kabuli*	Ca_Kabuli_denovo	13994280	(C/T)
20864	CakSNP20864	*Kabuli*	Ca_Kabuli_denovo	13994560	(C/G)
20865	CakSNP20865	*Kabuli*	Ca_Kabuli_denovo	13994609	(A/G)
20866	CakSNP20866	*Kabuli*	Ca_Kabuli_denovo	13997710	(C/A)
20867	CakSNP20867	*Kabuli*	Ca_Kabuli_denovo	14013165	(T/C)
20868	CakSNP20868	*Kabuli*	Ca_Kabuli_denovo	14024171	(A/G)
20869	CakSNP20869	*Kabuli*	Ca_Kabuli_denovo	14024173	(T/C)
20870	CakSNP20870	*Kabuli*	Ca_Kabuli_denovo	14031255	(G/C)
20871	CakSNP20871	*Kabuli*	Ca_Kabuli_denovo	14031282	(A/G)
20872	CakSNP20872	*Kabuli*	Ca_Kabuli_denovo	14038882	(A/G)
20873	CakSNP20873	*Kabuli*	Ca_Kabuli_denovo	14047682	(A/G)
20874	CakSNP20874	*Kabuli*	Ca_Kabuli_denovo	14047694	(G/A)
20875	CakSNP20875	*Kabuli*	Ca_Kabuli_denovo	14051292	(C/A)
20876	CakSNP20876	*Kabuli*	Ca_Kabuli_denovo	14054007	(C/T)
20877	CakSNP20877	*Kabuli*	Ca_Kabuli_denovo	14054913	(C/T)
20878	CakSNP20878	*Kabuli*	Ca_Kabuli_denovo	14063953	(G/C)
20879	CakSNP20879	*Kabuli*	Ca_Kabuli_denovo	14069422	(A/G)
20880	CakSNP20880	*Kabuli*	Ca_Kabuli_denovo	14069482	(C/T)
20881	CakSNP20881	*Kabuli*	Ca_Kabuli_denovo	14070071	(A/G)
20882	CakSNP20882	*Kabuli*	Ca_Kabuli_denovo	14070080	(T/C)
20883	CakSNP20883	*Kabuli*	Ca_Kabuli_denovo	14090644	(C/G)
20884	CakSNP20884	*Kabuli*	Ca_Kabuli_denovo	14094627	(C/T)
20885	CakSNP20885	*Kabuli*	Ca_Kabuli_denovo	14094666	(A/G)
20886	CakSNP20886	*Kabuli*	Ca_Kabuli_denovo	14095149	(T/G)
20887	CakSNP20887	*Kabuli*	Ca_Kabuli_denovo	14097307	(T/G)
20888	CakSNP20888	*Kabuli*	Ca_Kabuli_denovo	14097992	(G/A)
20889	CakSNP20889	*Kabuli*	Ca_Kabuli_denovo	14099100	(A/G)
20890	CakSNP20890	*Kabuli*	Ca_Kabuli_denovo	14101707	(G/C)
20891	CakSNP20891	*Kabuli*	Ca_Kabuli_denovo	14102440	(C/T)
20892	CakSNP20892	*Kabuli*	Ca_Kabuli_denovo	14102489	(G/T)
20893	CakSNP20893	*Kabuli*	Ca_Kabuli_denovo	14104688	(T/A)
20894	CakSNP20894	*Kabuli*	Ca_Kabuli_denovo	14109844	(G/A)
20895	CakSNP20895	*Kabuli*	Ca_Kabuli_denovo	14109866	(A/C)
20896	CakSNP20896	*Kabuli*	Ca_Kabuli_denovo	14109868	(A/C)
20897	CakSNP20897	*Kabuli*	Ca_Kabuli_denovo	14112239	(C/T)
20898	CakSNP20898	*Kabuli*	Ca_Kabuli_denovo	14112675	(G/T)
20899	CakSNP20899	*Kabuli*	Ca_Kabuli_denovo	14112693	(C/T)

S.N.	SNP IDs	Cultivars	Chromosomes/scaffolds	Physical positions (bp)	SNPs
20900	CakSNP20900	*Kabuli*	Ca_Kabuli_denovo	14115751	(C/T)
20901	CakSNP20901	*Kabuli*	Ca_Kabuli_denovo	14121780	(G/T)
20902	CakSNP20902	*Kabuli*	Ca_Kabuli_denovo	14121828	(C/T)
20903	CakSNP20903	*Kabuli*	Ca_Kabuli_denovo	14123266	(C/T)
20904	CakSNP20904	*Kabuli*	Ca_Kabuli_denovo	14132147	(C/G)
20905	CakSNP20905	*Kabuli*	Ca_Kabuli_denovo	14134459	(C/T)
20906	CakSNP20906	*Kabuli*	Ca_Kabuli_denovo	14136009	(A/G)
20907	CakSNP20907	*Kabuli*	Ca_Kabuli_denovo	14136078	(A/G)
20908	CakSNP20908	*Kabuli*	Ca_Kabuli_denovo	14141764	(T/C)
20909	CakSNP20909	*Kabuli*	Ca_Kabuli_denovo	14146205	(T/C)
20910	CakSNP20910	*Kabuli*	Ca_Kabuli_denovo	14146512	(A/T)
20911	CakSNP20911	*Kabuli*	Ca_Kabuli_denovo	14146533	(C/T)
20912	CakSNP20912	*Kabuli*	Ca_Kabuli_denovo	14151634	(A/G)
20913	CakSNP20913	*Kabuli*	Ca_Kabuli_denovo	14162150	(G/C)
20914	CakSNP20914	*Kabuli*	Ca_Kabuli_denovo	14162186	(G/A)
20915	CakSNP20915	*Kabuli*	Ca_Kabuli_denovo	14162459	(C/T)
20916	CakSNP20916	*Kabuli*	Ca_Kabuli_denovo	14165612	(A/G)
20917	CakSNP20917	*Kabuli*	Ca_Kabuli_denovo	14170796	(C/T)
20918	CakSNP20918	*Kabuli*	Ca_Kabuli_denovo	14170853	(G/A)
20919	CakSNP20919	*Kabuli*	Ca_Kabuli_denovo	14173237	(C/T)
20920	CakSNP20920	*Kabuli*	Ca_Kabuli_denovo	14175735	(C/G)
20921	CakSNP20921	*Kabuli*	Ca_Kabuli_denovo	14178519	(A/C)
20922	CakSNP20922	*Kabuli*	Ca_Kabuli_denovo	14178529	(C/T)
20923	CakSNP20923	*Kabuli*	Ca_Kabuli_denovo	14180447	(A/G)
20924	CakSNP20924	*Kabuli*	Ca_Kabuli_denovo	14186276	(T/C)
20925	CakSNP20925	*Kabuli*	Ca_Kabuli_denovo	14188666	(T/C)
20926	CakSNP20926	*Kabuli*	Ca_Kabuli_denovo	14204954	(T/C)
20927	CakSNP20927	*Kabuli*	Ca_Kabuli_denovo	14204970	(A/G)
20928	CakSNP20928	*Kabuli*	Ca_Kabuli_denovo	14210876	(A/G)
20929	CakSNP20929	*Kabuli*	Ca_Kabuli_denovo	14210938	(C/T)
20930	CakSNP20930	*Kabuli*	Ca_Kabuli_denovo	14219449	(A/G)
20931	CakSNP20931	*Kabuli*	Ca_Kabuli_denovo	14219489	(G/A)
20932	CakSNP20932	*Kabuli*	Ca_Kabuli_denovo	14219623	(T/C)
20933	CakSNP20933	*Kabuli*	Ca_Kabuli_denovo	14221319	(G/A)
20934	CakSNP20934	*Kabuli*	Ca_Kabuli_denovo	14221501	(T/C)
20935	CakSNP20935	*Kabuli*	Ca_Kabuli_denovo	14229007	(C/A)
20936	CakSNP20936	*Kabuli*	Ca_Kabuli_denovo	14231935	(T/C)
20937	CakSNP20937	*Kabuli*	Ca_Kabuli_denovo	14231950	(G/T)

S.N.	SNP IDs	Cultivars	Chromosomes/scaffolds	Physical positions (bp)	SNPs
20938	CakSNP20938	*Kabuli*	Ca_Kabuli_denovo	14235265	(T/G)
20939	CakSNP20939	*Kabuli*	Ca_Kabuli_denovo	14237166	(G/A)
20940	CakSNP20940	*Kabuli*	Ca_Kabuli_denovo	14237557	(A/C)
20941	CakSNP20941	*Kabuli*	Ca_Kabuli_denovo	14241384	(A/G)
20942	CakSNP20942	*Kabuli*	Ca_Kabuli_denovo	14244846	(A/G)
20943	CakSNP20943	*Kabuli*	Ca_Kabuli_denovo	14246524	(T/G)
20944	CakSNP20944	*Kabuli*	Ca_Kabuli_denovo	14251791	(G/C)
20945	CakSNP20945	*Kabuli*	Ca_Kabuli_denovo	14251806	(A/G)
20946	CakSNP20946	*Kabuli*	Ca_Kabuli_denovo	14255378	(G/A)
20947	CakSNP20947	*Kabuli*	Ca_Kabuli_denovo	14257965	(C/A)
20948	CakSNP20948	*Kabuli*	Ca_Kabuli_denovo	14264833	(G/C)
20949	CakSNP20949	*Kabuli*	Ca_Kabuli_denovo	14264890	(T/C)
20950	CakSNP20950	*Kabuli*	Ca_Kabuli_denovo	14265359	(A/G)
20951	CakSNP20951	*Kabuli*	Ca_Kabuli_denovo	14275421	(G/T)
20952	CakSNP20952	*Kabuli*	Ca_Kabuli_denovo	14276179	(C/T)
20953	CakSNP20953	*Kabuli*	Ca_Kabuli_denovo	14276201	(G/A)
20954	CakSNP20954	*Kabuli*	Ca_Kabuli_denovo	14282422	(T/G)
20955	CakSNP20955	*Kabuli*	Ca_Kabuli_denovo	14282784	(G/A)
20956	CakSNP20956	*Kabuli*	Ca_Kabuli_denovo	14282796	(G/A)
20957	CakSNP20957	*Kabuli*	Ca_Kabuli_denovo	14286253	(T/C)
20958	CakSNP20958	*Kabuli*	Ca_Kabuli_denovo	14286269	(T/C)
20959	CakSNP20959	*Kabuli*	Ca_Kabuli_denovo	14289279	(C/G)
20960	CakSNP20960	*Kabuli*	Ca_Kabuli_denovo	14289309	(A/G)
20961	CakSNP20961	*Kabuli*	Ca_Kabuli_denovo	14292246	(C/G)
20962	CakSNP20962	*Kabuli*	Ca_Kabuli_denovo	14296223	(C/T)
20963	CakSNP20963	*Kabuli*	Ca_Kabuli_denovo	14297461	(G/C)
20964	CakSNP20964	*Kabuli*	Ca_Kabuli_denovo	14297530	(G/C)
20965	CakSNP20965	*Kabuli*	Ca_Kabuli_denovo	14303045	(C/T)
20966	CakSNP20966	*Kabuli*	Ca_Kabuli_denovo	14307152	(A/G)
20967	CakSNP20967	*Kabuli*	Ca_Kabuli_denovo	14308158	(C/G)
20968	CakSNP20968	*Kabuli*	Ca_Kabuli_denovo	14308820	(C/T)
20969	CakSNP20969	*Kabuli*	Ca_Kabuli_denovo	14310581	(A/C)
20970	CakSNP20970	*Kabuli*	Ca_Kabuli_denovo	14310632	(T/C)
20971	CakSNP20971	*Kabuli*	Ca_Kabuli_denovo	14311760	(A/G)
20972	CakSNP20972	*Kabuli*	Ca_Kabuli_denovo	14313417	(C/G)
20973	CakSNP20973	*Kabuli*	Ca_Kabuli_denovo	14320707	(G/A)
20974	CakSNP20974	*Kabuli*	Ca_Kabuli_denovo	14320709	(T/A)
20975	CakSNP20975	*Kabuli*	Ca_Kabuli_denovo	14328623	(A/C)

S.N.	SNP IDs	Cultivars	Chromosomes/scaffolds	Physical positions (bp)	SNPs
20976	CakSNP20976	*Kabuli*	Ca_Kabuli_denovo	14328917	(G/T)
20977	CakSNP20977	*Kabuli*	Ca_Kabuli_denovo	14333931	(A/G)
20978	CakSNP20978	*Kabuli*	Ca_Kabuli_denovo	14337745	(A/G)
20979	CakSNP20979	*Kabuli*	Ca_Kabuli_denovo	14337746	(T/C)
20980	CakSNP20980	*Kabuli*	Ca_Kabuli_denovo	14341574	(A/G)
20981	CakSNP20981	*Kabuli*	Ca_Kabuli_denovo	14341617	(A/C)
20982	CakSNP20982	*Kabuli*	Ca_Kabuli_denovo	14346701	(A/G)
20983	CakSNP20983	*Kabuli*	Ca_Kabuli_denovo	14346747	(G/T)
20984	CakSNP20984	*Kabuli*	Ca_Kabuli_denovo	14347271	(T/C)
20985	CakSNP20985	*Kabuli*	Ca_Kabuli_denovo	14348435	(A/T)
20986	CakSNP20986	*Kabuli*	Ca_Kabuli_denovo	14352328	(T/C)
20987	CakSNP20987	*Kabuli*	Ca_Kabuli_denovo	14352669	(A/G)
20988	CakSNP20988	*Kabuli*	Ca_Kabuli_denovo	14353348	(G/T)
20989	CakSNP20989	*Kabuli*	Ca_Kabuli_denovo	14353366	(G/A)
20990	CakSNP20990	*Kabuli*	Ca_Kabuli_denovo	14364703	(C/G)
20991	CakSNP20991	*Kabuli*	Ca_Kabuli_denovo	14369102	(C/G)
20992	CakSNP20992	*Kabuli*	Ca_Kabuli_denovo	14369921	(T/C)
20993	CakSNP20993	*Kabuli*	Ca_Kabuli_denovo	14380810	(C/G)
20994	CakSNP20994	*Kabuli*	Ca_Kabuli_denovo	14380823	(T/C)
20995	CakSNP20995	*Kabuli*	Ca_Kabuli_denovo	14383046	(C/T)
20996	CakSNP20996	*Kabuli*	Ca_Kabuli_denovo	14388649	(G/A)
20997	CakSNP20997	*Kabuli*	Ca_Kabuli_denovo	14388663	(T/A)
20998	CakSNP20998	*Kabuli*	Ca_Kabuli_denovo	14394158	(C/T)
20999	CakSNP20999	*Kabuli*	Ca_Kabuli_denovo	14397942	(G/A)
21000	CakSNP21000	*Kabuli*	Ca_Kabuli_denovo	14401170	(T/C)
21001	CakSNP21001	*Kabuli*	Ca_Kabuli_denovo	14402239	(T/G)
21002	CakSNP21002	*Kabuli*	Ca_Kabuli_denovo	14403229	(T/G)
21003	CakSNP21003	*Kabuli*	Ca_Kabuli_denovo	14405364	(C/T)
21004	CakSNP21004	*Kabuli*	Ca_Kabuli_denovo	14405406	(T/C)
21005	CakSNP21005	*Kabuli*	Ca_Kabuli_denovo	14406925	(C/G)
21006	CakSNP21006	*Kabuli*	Ca_Kabuli_denovo	14406958	(A/G)
21007	CakSNP21007	*Kabuli*	Ca_Kabuli_denovo	14406974	(G/C)
21008	CakSNP21008	*Kabuli*	Ca_Kabuli_denovo	14407215	(C/A)
21009	CakSNP21009	*Kabuli*	Ca_Kabuli_denovo	14407218	(T/C)
21010	CakSNP21010	*Kabuli*	Ca_Kabuli_denovo	14414831	(A/C)
21011	CakSNP21011	*Kabuli*	Ca_Kabuli_denovo	14416795	(G/A)
21012	CakSNP21012	*Kabuli*	Ca_Kabuli_denovo	14429719	(T/C)
21013	CakSNP21013	*Kabuli*	Ca_Kabuli_denovo	14433591	(C/T)

S.N.	SNP IDs	Cultivars	Chromosomes/scaffolds	Physical positions (bp)	SNPs
21014	CakSNP21014	*Kabuli*	Ca_Kabuli_denovo	14437122	(T/G)
21015	CakSNP21015	*Kabuli*	Ca_Kabuli_denovo	14437433	(T/C)
21016	CakSNP21016	*Kabuli*	Ca_Kabuli_denovo	14442419	(T/C)
21017	CakSNP21017	*Kabuli*	Ca_Kabuli_denovo	14442430	(C/T)
21018	CakSNP21018	*Kabuli*	Ca_Kabuli_denovo	14443760	(T/G)
21019	CakSNP21019	*Kabuli*	Ca_Kabuli_denovo	14443778	(A/G)
21020	CakSNP21020	*Kabuli*	Ca_Kabuli_denovo	14444490	(T/C)
21021	CakSNP21021	*Kabuli*	Ca_Kabuli_denovo	14444536	(C/G)
21022	CakSNP21022	*Kabuli*	Ca_Kabuli_denovo	14445505	(A/C)
21023	CakSNP21023	*Kabuli*	Ca_Kabuli_denovo	14448509	(T/G)
21024	CakSNP21024	*Kabuli*	Ca_Kabuli_denovo	14449521	(A/T)
21025	CakSNP21025	*Kabuli*	Ca_Kabuli_denovo	14449530	(G/A)
21026	CakSNP21026	*Kabuli*	Ca_Kabuli_denovo	14452270	(T/C)
21027	CakSNP21027	*Kabuli*	Ca_Kabuli_denovo	14457529	(C/T)
21028	CakSNP21028	*Kabuli*	Ca_Kabuli_denovo	14467081	(T/G)
21029	CakSNP21029	*Kabuli*	Ca_Kabuli_denovo	14469056	(T/C)
21030	CakSNP21030	*Kabuli*	Ca_Kabuli_denovo	14469127	(C/G)
21031	CakSNP21031	*Kabuli*	Ca_Kabuli_denovo	14469420	(A/G)
21032	CakSNP21032	*Kabuli*	Ca_Kabuli_denovo	14483569	(C/T)
21033	CakSNP21033	*Kabuli*	Ca_Kabuli_denovo	14495553	(A/G)
21034	CakSNP21034	*Kabuli*	Ca_Kabuli_denovo	14495559	(T/G)
21035	CakSNP21035	*Kabuli*	Ca_Kabuli_denovo	14513052	(T/C)
21036	CakSNP21036	*Kabuli*	Ca_Kabuli_denovo	14513906	(G/T)
21037	CakSNP21037	*Kabuli*	Ca_Kabuli_denovo	14514776	(T/A)
21038	CakSNP21038	*Kabuli*	Ca_Kabuli_denovo	14514787	(T/C)
21039	CakSNP21039	*Kabuli*	Ca_Kabuli_denovo	14515738	(G/C)
21040	CakSNP21040	*Kabuli*	Ca_Kabuli_denovo	14530041	(A/C)
21041	CakSNP21041	*Kabuli*	Ca_Kabuli_denovo	14537319	(T/C)
21042	CakSNP21042	*Kabuli*	Ca_Kabuli_denovo	14543760	(C/T)
21043	CakSNP21043	*Kabuli*	Ca_Kabuli_denovo	14546700	(A/G)
21044	CakSNP21044	*Kabuli*	Ca_Kabuli_denovo	14547594	(C/T)
21045	CakSNP21045	*Kabuli*	Ca_Kabuli_denovo	14550152	(G/A)
21046	CakSNP21046	*Kabuli*	Ca_Kabuli_denovo	14551303	(G/T)
21047	CakSNP21047	*Kabuli*	Ca_Kabuli_denovo	14561183	(A/G)
21048	CakSNP21048	*Kabuli*	Ca_Kabuli_denovo	14562250	(C/T)
21049	CakSNP21049	*Kabuli*	Ca_Kabuli_denovo	14566607	(A/G)
21050	CakSNP21050	*Kabuli*	Ca_Kabuli_denovo	14570440	(C/G)
21051	CakSNP21051	*Kabuli*	Ca_Kabuli_denovo	14570663	(G/T)

S.N.	SNP IDs	Cultivars	Chromosomes/scaffolds	Physical positions (bp)	SNPs
21052	CakSNP21052	*Kabuli*	Ca_Kabuli_denovo	14571952	(T/A)
21053	CakSNP21053	*Kabuli*	Ca_Kabuli_denovo	14572744	(T/C)
21054	CakSNP21054	*Kabuli*	Ca_Kabuli_denovo	14573976	(C/T)
21055	CakSNP21055	*Kabuli*	Ca_Kabuli_denovo	14578027	(A/C)
21056	CakSNP21056	*Kabuli*	Ca_Kabuli_denovo	14578231	(A/G)
21057	CakSNP21057	*Kabuli*	Ca_Kabuli_denovo	14578249	(C/T)
21058	CakSNP21058	*Kabuli*	Ca_Kabuli_denovo	14578273	(G/A)
21059	CakSNP21059	*Kabuli*	Ca_Kabuli_denovo	14582337	(C/T)
21060	CakSNP21060	*Kabuli*	Ca_Kabuli_denovo	14587510	(T/C)
21061	CakSNP21061	*Kabuli*	Ca_Kabuli_denovo	14589670	(G/A)
21062	CakSNP21062	*Kabuli*	Ca_Kabuli_denovo	14589693	(G/C)
21063	CakSNP21063	*Kabuli*	Ca_Kabuli_denovo	14590243	(A/C)
21064	CakSNP21064	*Kabuli*	Ca_Kabuli_denovo	14596822	(T/G)
21065	CakSNP21065	*Kabuli*	Ca_Kabuli_denovo	14596886	(T/C)
21066	CakSNP21066	*Kabuli*	Ca_Kabuli_denovo	14597698	(T/C)
21067	CakSNP21067	*Kabuli*	Ca_Kabuli_denovo	14599704	(A/G)
21068	CakSNP21068	*Kabuli*	Ca_Kabuli_denovo	14609759	(C/G)
21069	CakSNP21069	*Kabuli*	Ca_Kabuli_denovo	14609792	(T/C)
21070	CakSNP21070	*Kabuli*	Ca_Kabuli_denovo	14614842	(G/T)
21071	CakSNP21071	*Kabuli*	Ca_Kabuli_denovo	14615031	(T/G)
21072	CakSNP21072	*Kabuli*	Ca_Kabuli_denovo	14617523	(T/C)
21073	CakSNP21073	*Kabuli*	Ca_Kabuli_denovo	14619462	(T/G)
21074	CakSNP21074	*Kabuli*	Ca_Kabuli_denovo	14619490	(T/C)
21075	CakSNP21075	*Kabuli*	Ca_Kabuli_denovo	14620013	(C/T)
21076	CakSNP21076	*Kabuli*	Ca_Kabuli_denovo	14631563	(T/G)
21077	CakSNP21077	*Kabuli*	Ca_Kabuli_denovo	14636201	(G/A)
21078	CakSNP21078	*Kabuli*	Ca_Kabuli_denovo	14636204	(A/G)
21079	CakSNP21079	*Kabuli*	Ca_Kabuli_denovo	14636266	(G/A)
21080	CakSNP21080	*Kabuli*	Ca_Kabuli_denovo	14636270	(A/C)
21081	CakSNP21081	*Kabuli*	Ca_Kabuli_denovo	14641177	(A/C)
21082	CakSNP21082	*Kabuli*	Ca_Kabuli_denovo	14648080	(C/G)
21083	CakSNP21083	*Kabuli*	Ca_Kabuli_denovo	14648723	(T/C)
21084	CakSNP21084	*Kabuli*	Ca_Kabuli_denovo	14648727	(C/T)
21085	CakSNP21085	*Kabuli*	Ca_Kabuli_denovo	14652198	(G/T)
21086	CakSNP21086	*Kabuli*	Ca_Kabuli_denovo	14660915	(A/G)
21087	CakSNP21087	*Kabuli*	Ca_Kabuli_denovo	14668713	(A/C)
21088	CakSNP21088	*Kabuli*	Ca_Kabuli_denovo	14670691	(C/T)
21089	CakSNP21089	*Kabuli*	Ca_Kabuli_denovo	14670709	(C/T)

**S.N.**	**SNP IDs**	**Cultivars**	**Chromosomes/scaffolds**	**Physical positions (bp)**	**SNPs**
21090	CakSNP21090	*Kabuli*	Ca_Kabuli_denovo	14673778	(T/C)
21091	CakSNP21091	*Kabuli*	Ca_Kabuli_denovo	14682478	(C/G)
21092	CakSNP21092	*Kabuli*	Ca_Kabuli_denovo	14682505	(T/C)
21093	CakSNP21093	*Kabuli*	Ca_Kabuli_denovo	14685915	(G/A)
21094	CakSNP21094	*Kabuli*	Ca_Kabuli_denovo	14689649	(G/C)
21095	CakSNP21095	*Kabuli*	Ca_Kabuli_denovo	14696938	(A/G)
21096	CakSNP21096	*Kabuli*	Ca_Kabuli_denovo	14698942	(T/C)
21097	CakSNP21097	*Kabuli*	Ca_Kabuli_denovo	14703530	(C/G)
21098	CakSNP21098	*Kabuli*	Ca_Kabuli_denovo	14703575	(A/G)
21099	CakSNP21099	*Kabuli*	Ca_Kabuli_denovo	14704072	(T/C)
21100	CakSNP21100	*Kabuli*	Ca_Kabuli_denovo	14704120	(T/C)
21101	CakSNP21101	*Kabuli*	Ca_Kabuli_denovo	14704731	(C/T)
21102	CakSNP21102	*Kabuli*	Ca_Kabuli_denovo	14704732	(T/C)
21103	CakSNP21103	*Kabuli*	Ca_Kabuli_denovo	14715112	(A/G)
21104	CakSNP21104	*Kabuli*	Ca_Kabuli_denovo	14715215	(G/A)
21105	CakSNP21105	*Kabuli*	Ca_Kabuli_denovo	14715220	(C/G)
21106	CakSNP21106	*Kabuli*	Ca_Kabuli_denovo	14726401	(A/G)
21107	CakSNP21107	*Kabuli*	Ca_Kabuli_denovo	14728979	(G/T)
21108	CakSNP21108	*Kabuli*	Ca_Kabuli_denovo	14729758	(C/G)
21109	CakSNP21109	*Kabuli*	Ca_Kabuli_denovo	14729891	(A/T)
21110	CakSNP21110	*Kabuli*	Ca_Kabuli_denovo	14735636	(T/G)
21111	CakSNP21111	*Kabuli*	Ca_Kabuli_denovo	14736247	(A/G)
21112	CakSNP21112	*Kabuli*	Ca_Kabuli_denovo	14736289	(C/T)
21113	CakSNP21113	*Kabuli*	Ca_Kabuli_denovo	14741708	(A/C)
21114	CakSNP21114	*Kabuli*	Ca_Kabuli_denovo	14743692	(C/G)
21115	CakSNP21115	*Kabuli*	Ca_Kabuli_denovo	14743756	(T/A)
21116	CakSNP21116	*Kabuli*	Ca_Kabuli_denovo	14745532	(A/C)
21117	CakSNP21117	*Kabuli*	Ca_Kabuli_denovo	14746118	(A/G)
21118	CakSNP21118	*Kabuli*	Ca_Kabuli_denovo	14746154	(A/G)
21119	CakSNP21119	*Kabuli*	Ca_Kabuli_denovo	14748412	(G/A)
21120	CakSNP21120	*Kabuli*	Ca_Kabuli_denovo	14748417	(G/C)
21121	CakSNP21121	*Kabuli*	Ca_Kabuli_denovo	14749636	(G/A)
21122	CakSNP21122	*Kabuli*	Ca_Kabuli_denovo	14749704	(T/G)
21123	CakSNP21123	*Kabuli*	Ca_Kabuli_denovo	14749705	(G/T)
21124	CakSNP21124	*Kabuli*	Ca_Kabuli_denovo	14752787	(T/C)
21125	CakSNP21125	*Kabuli*	Ca_Kabuli_denovo	14755314	(C/T)
21126	CakSNP21126	*Kabuli*	Ca_Kabuli_denovo	14757725	(C/G)
21127	CakSNP21127	*Kabuli*	Ca_Kabuli_denovo	14759576	(C/T)

S.N.	SNP IDs	Cultivars	Chromosomes/scaffolds	Physical positions (bp)	SNPs
21128	CakSNP21128	*Kabuli*	Ca_Kabuli_denovo	14782372	(C/T)
21129	CakSNP21129	*Kabuli*	Ca_Kabuli_denovo	14782399	(C/T)
21130	CakSNP21130	*Kabuli*	Ca_Kabuli_denovo	14787071	(G/T)
21131	CakSNP21131	*Kabuli*	Ca_Kabuli_denovo	14788336	(T/C)
21132	CakSNP21132	*Kabuli*	Ca_Kabuli_denovo	14792248	(G/C)
21133	CakSNP21133	*Kabuli*	Ca_Kabuli_denovo	14792775	(G/A)
21134	CakSNP21134	*Kabuli*	Ca_Kabuli_denovo	14801212	(C/G)
21135	CakSNP21135	*Kabuli*	Ca_Kabuli_denovo	14801218	(C/T)
21136	CakSNP21136	*Kabuli*	Ca_Kabuli_denovo	14805457	(T/C)
21137	CakSNP21137	*Kabuli*	Ca_Kabuli_denovo	14805477	(G/C)
21138	CakSNP21138	*Kabuli*	Ca_Kabuli_denovo	14805855	(T/C)
21139	CakSNP21139	*Kabuli*	Ca_Kabuli_denovo	14805865	(A/G)
21140	CakSNP21140	*Kabuli*	Ca_Kabuli_denovo	14811884	(G/C)
21141	CakSNP21141	*Kabuli*	Ca_Kabuli_denovo	14825428	(C/G)
21142	CakSNP21142	*Kabuli*	Ca_Kabuli_denovo	14825440	(G/C)
21143	CakSNP21143	*Kabuli*	Ca_Kabuli_denovo	14833055	(C/A)
21144	CakSNP21144	*Kabuli*	Ca_Kabuli_denovo	14833103	(A/G)
21145	CakSNP21145	*Kabuli*	Ca_Kabuli_denovo	14834045	(G/C)
21146	CakSNP21146	*Kabuli*	Ca_Kabuli_denovo	14838314	(G/C)
21147	CakSNP21147	*Kabuli*	Ca_Kabuli_denovo	14841355	(G/A)
21148	CakSNP21148	*Kabuli*	Ca_Kabuli_denovo	14841976	(C/T)
21149	CakSNP21149	*Kabuli*	Ca_Kabuli_denovo	14845633	(A/C)
21150	CakSNP21150	*Kabuli*	Ca_Kabuli_denovo	14861241	(T/C)
21151	CakSNP21151	*Kabuli*	Ca_Kabuli_denovo	14861255	(T/C)
21152	CakSNP21152	*Kabuli*	Ca_Kabuli_denovo	14864054	(G/T)
21153	CakSNP21153	*Kabuli*	Ca_Kabuli_denovo	14864425	(C/T)
21154	CakSNP21154	*Kabuli*	Ca_Kabuli_denovo	14872625	(C/T)
21155	CakSNP21155	*Kabuli*	Ca_Kabuli_denovo	14872631	(C/T)
21156	CakSNP21156	*Kabuli*	Ca_Kabuli_denovo	14877101	(G/A)
21157	CakSNP21157	*Kabuli*	Ca_Kabuli_denovo	14877962	(G/A)
21158	CakSNP21158	*Kabuli*	Ca_Kabuli_denovo	14878041	(C/G)
21159	CakSNP21159	*Kabuli*	Ca_Kabuli_denovo	14881946	(C/T)
21160	CakSNP21160	*Kabuli*	Ca_Kabuli_denovo	14883255	(A/C)
21161	CakSNP21161	*Kabuli*	Ca_Kabuli_denovo	14883746	(C/T)
21162	CakSNP21162	*Kabuli*	Ca_Kabuli_denovo	14883748	(G/A)
21163	CakSNP21163	*Kabuli*	Ca_Kabuli_denovo	14886823	(T/C)
21164	CakSNP21164	*Kabuli*	Ca_Kabuli_denovo	14888070	(A/G)
21165	CakSNP21165	*Kabuli*	Ca_Kabuli_denovo	14891224	(G/A)

S.N.	SNP IDs	Cultivars	Chromosomes/scaffolds	Physical positions (bp)	SNPs
21166	CakSNP21166	*Kabuli*	Ca_Kabuli_denovo	14893670	(A/C)
21167	CakSNP21167	*Kabuli*	Ca_Kabuli_denovo	14893855	(T/G)
21168	CakSNP21168	*Kabuli*	Ca_Kabuli_denovo	14895580	(A/C)
21169	CakSNP21169	*Kabuli*	Ca_Kabuli_denovo	14898622	(C/T)
21170	CakSNP21170	*Kabuli*	Ca_Kabuli_denovo	14898764	(C/A)
21171	CakSNP21171	*Kabuli*	Ca_Kabuli_denovo	14899131	(T/G)
21172	CakSNP21172	*Kabuli*	Ca_Kabuli_denovo	14899150	(T/C)
21173	CakSNP21173	*Kabuli*	Ca_Kabuli_denovo	14899484	(T/C)
21174	CakSNP21174	*Kabuli*	Ca_Kabuli_denovo	14905145	(C/G)
21175	CakSNP21175	*Kabuli*	Ca_Kabuli_denovo	14906147	(C/G)
21176	CakSNP21176	*Kabuli*	Ca_Kabuli_denovo	14907584	(C/T)
21177	CakSNP21177	*Kabuli*	Ca_Kabuli_denovo	14909654	(T/G)
21178	CakSNP21178	*Kabuli*	Ca_Kabuli_denovo	14909803	(T/G)
21179	CakSNP21179	*Kabuli*	Ca_Kabuli_denovo	14912143	(A/G)
21180	CakSNP21180	*Kabuli*	Ca_Kabuli_denovo	14912144	(A/G)
21181	CakSNP21181	*Kabuli*	Ca_Kabuli_denovo	14913605	(C/T)
21182	CakSNP21182	*Kabuli*	Ca_Kabuli_denovo	14914323	(G/A)
21183	CakSNP21183	*Kabuli*	Ca_Kabuli_denovo	14916673	(T/C)
21184	CakSNP21184	*Kabuli*	Ca_Kabuli_denovo	14916715	(A/G)
21185	CakSNP21185	*Kabuli*	Ca_Kabuli_denovo	14918881	(G/C)
21186	CakSNP21186	*Kabuli*	Ca_Kabuli_denovo	14926796	(A/G)
21187	CakSNP21187	*Kabuli*	Ca_Kabuli_denovo	14927585	(T/C)
21188	CakSNP21188	*Kabuli*	Ca_Kabuli_denovo	14929577	(A/C)
21189	CakSNP21189	*Kabuli*	Ca_Kabuli_denovo	14935159	(C/A)
21190	CakSNP21190	*Kabuli*	Ca_Kabuli_denovo	14942882	(A/G)
21191	CakSNP21191	*Kabuli*	Ca_Kabuli_denovo	14942888	(A/C)
21192	CakSNP21192	*Kabuli*	Ca_Kabuli_denovo	14943646	(G/A)
21193	CakSNP21193	*Kabuli*	Ca_Kabuli_denovo	14950163	(T/G)
21194	CakSNP21194	*Kabuli*	Ca_Kabuli_denovo	14962978	(T/C)
21195	CakSNP21195	*Kabuli*	Ca_Kabuli_denovo	14968986	(C/A)
21196	CakSNP21196	*Kabuli*	Ca_Kabuli_denovo	14971785	(C/T)
21197	CakSNP21197	*Kabuli*	Ca_Kabuli_denovo	14974801	(G/A)
21198	CakSNP21198	*Kabuli*	Ca_Kabuli_denovo	14974806	(G/A)
21199	CakSNP21199	*Kabuli*	Ca_Kabuli_denovo	14977193	(T/C)
21200	CakSNP21200	*Kabuli*	Ca_Kabuli_denovo	14978299	(A/C)
21201	CakSNP21201	*Kabuli*	Ca_Kabuli_denovo	14981709	(C/T)
21202	CakSNP21202	*Kabuli*	Ca_Kabuli_denovo	14981741	(A/G)
21203	CakSNP21203	*Kabuli*	Ca_Kabuli_denovo	14982459	(G/A)

S.N.	SNP IDs	Cultivars	Chromosomes/scaffolds	Physical positions (bp)	SNPs
21204	CakSNP21204	*Kabuli*	Ca_Kabuli_denovo	14982465	(A/C)
21205	CakSNP21205	*Kabuli*	Ca_Kabuli_denovo	14985193	(G/A)
21206	CakSNP21206	*Kabuli*	Ca_Kabuli_denovo	14985249	(G/A)
21207	CakSNP21207	*Kabuli*	Ca_Kabuli_denovo	14991461	(T/C)
21208	CakSNP21208	*Kabuli*	Ca_Kabuli_denovo	14993274	(T/G)
21209	CakSNP21209	*Kabuli*	Ca_Kabuli_denovo	14998061	(T/C)
21210	CakSNP21210	*Kabuli*	Ca_Kabuli_denovo	15002955	(T/C)
21211	CakSNP21211	*Kabuli*	Ca_Kabuli_denovo	15006029	(T/A)
21212	CakSNP21212	*Kabuli*	Ca_Kabuli_denovo	15007070	(G/T)
21213	CakSNP21213	*Kabuli*	Ca_Kabuli_denovo	15011020	(G/A)
21214	CakSNP21214	*Kabuli*	Ca_Kabuli_denovo	15012544	(C/A)
21215	CakSNP21215	*Kabuli*	Ca_Kabuli_denovo	15012589	(C/T)
21216	CakSNP21216	*Kabuli*	Ca_Kabuli_denovo	15019523	(T/C)
21217	CakSNP21217	*Kabuli*	Ca_Kabuli_denovo	15019572	(G/T)
21218	CakSNP21218	*Kabuli*	Ca_Kabuli_denovo	15029564	(G/A)
21219	CakSNP21219	*Kabuli*	Ca_Kabuli_denovo	15029594	(G/A)
21220	CakSNP21220	*Kabuli*	Ca_Kabuli_denovo	15029893	(T/G)
21221	CakSNP21221	*Kabuli*	Ca_Kabuli_denovo	15029901	(T/C)
21222	CakSNP21222	*Kabuli*	Ca_Kabuli_denovo	15033418	(G/A)
21223	CakSNP21223	*Kabuli*	Ca_Kabuli_denovo	15048640	(G/C)
21224	CakSNP21224	*Kabuli*	Ca_Kabuli_denovo	15053938	(G/C)
21225	CakSNP21225	*Kabuli*	Ca_Kabuli_denovo	15053965	(T/C)
21226	CakSNP21226	*Kabuli*	Ca_Kabuli_denovo	15055093	(G/C)
21227	CakSNP21227	*Kabuli*	Ca_Kabuli_denovo	15055828	(C/A)
21228	CakSNP21228	*Kabuli*	Ca_Kabuli_denovo	15056085	(T/C)
21229	CakSNP21229	*Kabuli*	Ca_Kabuli_denovo	15056124	(C/T)
21230	CakSNP21230	*Kabuli*	Ca_Kabuli_denovo	15057672	(A/G)
21231	CakSNP21231	*Kabuli*	Ca_Kabuli_denovo	15058226	(C/A)
21232	CakSNP21232	*Kabuli*	Ca_Kabuli_denovo	15061165	(A/C)
21233	CakSNP21233	*Kabuli*	Ca_Kabuli_denovo	15063461	(C/T)
21234	CakSNP21234	*Kabuli*	Ca_Kabuli_denovo	15064384	(C/T)
21235	CakSNP21235	*Kabuli*	Ca_Kabuli_denovo	15068536	(G/C)
21236	CakSNP21236	*Kabuli*	Ca_Kabuli_denovo	15072879	(C/T)
21237	CakSNP21237	*Kabuli*	Ca_Kabuli_denovo	15072882	(A/C)
21238	CakSNP21238	*Kabuli*	Ca_Kabuli_denovo	15076991	(G/C)
21239	CakSNP21239	*Kabuli*	Ca_Kabuli_denovo	15077061	(T/C)
21240	CakSNP21240	*Kabuli*	Ca_Kabuli_denovo	15078562	(T/C)
21241	CakSNP21241	*Kabuli*	Ca_Kabuli_denovo	15078586	(G/C)

S.N.	SNP IDs	Cultivars	Chromosomes/scaffolds	Physical positions (bp)	SNPs
21242	CakSNP21242	*Kabuli*	Ca_Kabuli_denovo	15078864	(A/G)
21243	CakSNP21243	*Kabuli*	Ca_Kabuli_denovo	15079985	(C/A)
21244	CakSNP21244	*Kabuli*	Ca_Kabuli_denovo	15079994	(A/G)
21245	CakSNP21245	*Kabuli*	Ca_Kabuli_denovo	15080664	(G/A)
21246	CakSNP21246	*Kabuli*	Ca_Kabuli_denovo	15081288	(C/T)
21247	CakSNP21247	*Kabuli*	Ca_Kabuli_denovo	15081298	(G/A)
21248	CakSNP21248	*Kabuli*	Ca_Kabuli_denovo	15084526	(C/T)
21249	CakSNP21249	*Kabuli*	Ca_Kabuli_denovo	15084576	(C/T)
21250	CakSNP21250	*Kabuli*	Ca_Kabuli_denovo	15097373	(A/C)
21251	CakSNP21251	*Kabuli*	Ca_Kabuli_denovo	15101968	(T/C)
21252	CakSNP21252	*Kabuli*	Ca_Kabuli_denovo	15109364	(G/A)
21253	CakSNP21253	*Kabuli*	Ca_Kabuli_denovo	15111061	(T/C)
21254	CakSNP21254	*Kabuli*	Ca_Kabuli_denovo	15121640	(T/C)
21255	CakSNP21255	*Kabuli*	Ca_Kabuli_denovo	15122674	(A/C)
21256	CakSNP21256	*Kabuli*	Ca_Kabuli_denovo	15125029	(A/C)
21257	CakSNP21257	*Kabuli*	Ca_Kabuli_denovo	15125256	(A/G)
21258	CakSNP21258	*Kabuli*	Ca_Kabuli_denovo	15128950	(C/G)
21259	CakSNP21259	*Kabuli*	Ca_Kabuli_denovo	15136143	(G/A)
21260	CakSNP21260	*Kabuli*	Ca_Kabuli_denovo	15146085	(T/C)
21261	CakSNP21261	*Kabuli*	Ca_Kabuli_denovo	15146116	(C/G)
21262	CakSNP21262	*Kabuli*	Ca_Kabuli_denovo	15147215	(G/C)
21263	CakSNP21263	*Kabuli*	Ca_Kabuli_denovo	15148861	(A/C)
21264	CakSNP21264	*Kabuli*	Ca_Kabuli_denovo	15148863	(A/C)
21265	CakSNP21265	*Kabuli*	Ca_Kabuli_denovo	15149093	(G/T)
21266	CakSNP21266	*Kabuli*	Ca_Kabuli_denovo	15149102	(C/T)
21267	CakSNP21267	*Kabuli*	Ca_Kabuli_denovo	15162466	(C/G)
21268	CakSNP21268	*Kabuli*	Ca_Kabuli_denovo	15162535	(C/G)
21269	CakSNP21269	*Kabuli*	Ca_Kabuli_denovo	15172412	(A/G)
21270	CakSNP21270	*Kabuli*	Ca_Kabuli_denovo	15173713	(G/T)
21271	CakSNP21271	*Kabuli*	Ca_Kabuli_denovo	15173771	(T/G)
21272	CakSNP21272	*Kabuli*	Ca_Kabuli_denovo	15175666	(T/C)
21273	CakSNP21273	*Kabuli*	Ca_Kabuli_denovo	15178364	(A/C)
21274	CakSNP21274	*Kabuli*	Ca_Kabuli_denovo	15178367	(C/T)
21275	CakSNP21275	*Kabuli*	Ca_Kabuli_denovo	15181189	(C/G)
21276	CakSNP21276	*Kabuli*	Ca_Kabuli_denovo	15193542	(A/G)
21277	CakSNP21277	*Kabuli*	Ca_Kabuli_denovo	15193595	(T/G)
21278	CakSNP21278	*Kabuli*	Ca_Kabuli_denovo	15194474	(A/C)
21279	CakSNP21279	*Kabuli*	Ca_Kabuli_denovo	15194482	(A/G)

S.N.	SNP IDs	Cultivars	Chromosomes/scaffolds	Physical positions (bp)	SNPs
21280	CakSNP21280	*Kabuli*	Ca_Kabuli_denovo	15198160	(T/C)
21281	CakSNP21281	*Kabuli*	Ca_Kabuli_denovo	15198174	(G/A)
21282	CakSNP21282	*Kabuli*	Ca_Kabuli_denovo	15198245	(A/G)
21283	CakSNP21283	*Kabuli*	Ca_Kabuli_denovo	15199468	(T/C)
21284	CakSNP21284	*Kabuli*	Ca_Kabuli_denovo	15199489	(T/C)
21285	CakSNP21285	*Kabuli*	Ca_Kabuli_denovo	15199495	(G/A)
21286	CakSNP21286	*Kabuli*	Ca_Kabuli_denovo	15206923	(G/A)
21287	CakSNP21287	*Kabuli*	Ca_Kabuli_denovo	15206935	(A/G)
21288	CakSNP21288	*Kabuli*	Ca_Kabuli_denovo	15209330	(A/G)
21289	CakSNP21289	*Kabuli*	Ca_Kabuli_denovo	15210193	(G/A)
21290	CakSNP21290	*Kabuli*	Ca_Kabuli_denovo	15210344	(C/T)
21291	CakSNP21291	*Kabuli*	Ca_Kabuli_denovo	15211610	(A/G)
21292	CakSNP21292	*Kabuli*	Ca_Kabuli_denovo	15212128	(T/C)
21293	CakSNP21293	*Kabuli*	Ca_Kabuli_denovo	15212149	(T/C)
21294	CakSNP21294	*Kabuli*	Ca_Kabuli_denovo	15212161	(G/C)
21295	CakSNP21295	*Kabuli*	Ca_Kabuli_denovo	15213006	(A/T)
21296	CakSNP21296	*Kabuli*	Ca_Kabuli_denovo	15226225	(G/A)
21297	CakSNP21297	*Kabuli*	Ca_Kabuli_denovo	15228338	(A/C)
21298	CakSNP21298	*Kabuli*	Ca_Kabuli_denovo	15233099	(A/G)
21299	CakSNP21299	*Kabuli*	Ca_Kabuli_denovo	15237165	(T/C)
21300	CakSNP21300	*Kabuli*	Ca_Kabuli_denovo	15238403	(C/G)
21301	CakSNP21301	*Kabuli*	Ca_Kabuli_denovo	15244019	(C/T)
21302	CakSNP21302	*Kabuli*	Ca_Kabuli_denovo	15245249	(G/C)
21303	CakSNP21303	*Kabuli*	Ca_Kabuli_denovo	15250791	(A/C)
21304	CakSNP21304	*Kabuli*	Ca_Kabuli_denovo	15261871	(A/G)
21305	CakSNP21305	*Kabuli*	Ca_Kabuli_denovo	15262432	(T/C)
21306	CakSNP21306	*Kabuli*	Ca_Kabuli_denovo	15262463	(T/C)
21307	CakSNP21307	*Kabuli*	Ca_Kabuli_denovo	15267483	(C/T)
21308	CakSNP21308	*Kabuli*	Ca_Kabuli_denovo	15267510	(T/C)
21309	CakSNP21309	*Kabuli*	Ca_Kabuli_denovo	15270517	(A/G)
21310	CakSNP21310	*Kabuli*	Ca_Kabuli_denovo	15270541	(G/C)
21311	CakSNP21311	*Kabuli*	Ca_Kabuli_denovo	15271966	(G/T)
21312	CakSNP21312	*Kabuli*	Ca_Kabuli_denovo	15277962	(C/A)
21313	CakSNP21313	*Kabuli*	Ca_Kabuli_denovo	15278013	(A/C)
21314	CakSNP21314	*Kabuli*	Ca_Kabuli_denovo	15278377	(C/G)
21315	CakSNP21315	*Kabuli*	Ca_Kabuli_denovo	15278389	(T/C)
21316	CakSNP21316	*Kabuli*	Ca_Kabuli_denovo	15281124	(A/C)
21317	CakSNP21317	*Kabuli*	Ca_Kabuli_denovo	15281657	(C/A)

S.N.	SNP IDs	Cultivars	Chromosomes/scaffolds	Physical positions (bp)	SNPs
21318	CakSNP21318	*Kabuli*	Ca_Kabuli_denovo	15281660	(C/G)
21319	CakSNP21319	*Kabuli*	Ca_Kabuli_denovo	15285712	(T/A)
21320	CakSNP21320	*Kabuli*	Ca_Kabuli_denovo	15286336	(C/T)
21321	CakSNP21321	*Kabuli*	Ca_Kabuli_denovo	15289465	(C/T)
21322	CakSNP21322	*Kabuli*	Ca_Kabuli_denovo	15289612	(A/G)
21323	CakSNP21323	*Kabuli*	Ca_Kabuli_denovo	15298450	(G/T)
21324	CakSNP21324	*Kabuli*	Ca_Kabuli_denovo	15298471	(T/A)
21325	CakSNP21325	*Kabuli*	Ca_Kabuli_denovo	15301323	(C/T)
21326	CakSNP21326	*Kabuli*	Ca_Kabuli_denovo	15301328	(C/T)
21327	CakSNP21327	*Kabuli*	Ca_Kabuli_denovo	15307943	(G/C)
21328	CakSNP21328	*Kabuli*	Ca_Kabuli_denovo	15308754	(C/G)
21329	CakSNP21329	*Kabuli*	Ca_Kabuli_denovo	15310682	(A/C)
21330	CakSNP21330	*Kabuli*	Ca_Kabuli_denovo	15310711	(T/C)
21331	CakSNP21331	*Kabuli*	Ca_Kabuli_denovo	15310717	(G/A)
21332	CakSNP21332	*Kabuli*	Ca_Kabuli_denovo	15311372	(A/T)
21333	CakSNP21333	*Kabuli*	Ca_Kabuli_denovo	15321205	(G/A)
21334	CakSNP21334	*Kabuli*	Ca_Kabuli_denovo	15325303	(C/G)
21335	CakSNP21335	*Kabuli*	Ca_Kabuli_denovo	15325309	(A/C)
21336	CakSNP21336	*Kabuli*	Ca_Kabuli_denovo	15328163	(G/C)
21337	CakSNP21337	*Kabuli*	Ca_Kabuli_denovo	15339934	(C/T)
21338	CakSNP21338	*Kabuli*	Ca_Kabuli_denovo	15341482	(A/G)
21339	CakSNP21339	*Kabuli*	Ca_Kabuli_denovo	15342107	(G/A)
21340	CakSNP21340	*Kabuli*	Ca_Kabuli_denovo	15343248	(C/G)
21341	CakSNP21341	*Kabuli*	Ca_Kabuli_denovo	15343266	(A/G)
21342	CakSNP21342	*Kabuli*	Ca_Kabuli_denovo	15345196	(T/C)
21343	CakSNP21343	*Kabuli*	Ca_Kabuli_denovo	15345239	(T/A)
21344	CakSNP21344	*Kabuli*	Ca_Kabuli_denovo	15349210	(C/T)
21345	CakSNP21345	*Kabuli*	Ca_Kabuli_denovo	15349219	(C/T)
21346	CakSNP21346	*Kabuli*	Ca_Kabuli_denovo	15350042	(C/T)
21347	CakSNP21347	*Kabuli*	Ca_Kabuli_denovo	15350057	(C/G)
21348	CakSNP21348	*Kabuli*	Ca_Kabuli_denovo	15352453	(G/A)
21349	CakSNP21349	*Kabuli*	Ca_Kabuli_denovo	15352531	(A/G)
21350	CakSNP21350	*Kabuli*	Ca_Kabuli_denovo	15357660	(G/C)
21351	CakSNP21351	*Kabuli*	Ca_Kabuli_denovo	15357668	(C/T)
21352	CakSNP21352	*Kabuli*	Ca_Kabuli_denovo	15358078	(C/T)
21353	CakSNP21353	*Kabuli*	Ca_Kabuli_denovo	15358348	(C/T)
21354	CakSNP21354	*Kabuli*	Ca_Kabuli_denovo	15361024	(C/T)
21355	CakSNP21355	*Kabuli*	Ca_Kabuli_denovo	15361027	(A/G)

S.N.	SNP IDs	Cultivars	Chromosomes/scaffolds	Physical positions (bp)	SNPs
21356	CakSNP21356	*Kabuli*	Ca_Kabuli_denovo	15361033	(A/C)
21357	CakSNP21357	*Kabuli*	Ca_Kabuli_denovo	15363103	(C/T)
21358	CakSNP21358	*Kabuli*	Ca_Kabuli_denovo	15363133	(A/G)
21359	CakSNP21359	*Kabuli*	Ca_Kabuli_denovo	15366397	(G/A)
21360	CakSNP21360	*Kabuli*	Ca_Kabuli_denovo	15368258	(T/C)
21361	CakSNP21361	*Kabuli*	Ca_Kabuli_denovo	15368306	(A/G)
21362	CakSNP21362	*Kabuli*	Ca_Kabuli_denovo	15379238	(T/C)
21363	CakSNP21363	*Kabuli*	Ca_Kabuli_denovo	15379241	(G/A)
21364	CakSNP21364	*Kabuli*	Ca_Kabuli_denovo	15383419	(T/C)
21365	CakSNP21365	*Kabuli*	Ca_Kabuli_denovo	15386024	(G/A)
21366	CakSNP21366	*Kabuli*	Ca_Kabuli_denovo	15386083	(C/A)
21367	CakSNP21367	*Kabuli*	Ca_Kabuli_denovo	15387519	(C/T)
21368	CakSNP21368	*Kabuli*	Ca_Kabuli_denovo	15390193	(T/G)
21369	CakSNP21369	*Kabuli*	Ca_Kabuli_denovo	15390614	(G/C)
21370	CakSNP21370	*Kabuli*	Ca_Kabuli_denovo	15390631	(G/C)
21371	CakSNP21371	*Kabuli*	Ca_Kabuli_denovo	15390776	(C/G)
21372	CakSNP21372	*Kabuli*	Ca_Kabuli_denovo	15390791	(G/A)
21373	CakSNP21373	*Kabuli*	Ca_Kabuli_denovo	15391286	(G/A)
21374	CakSNP21374	*Kabuli*	Ca_Kabuli_denovo	15391314	(A/C)
21375	CakSNP21375	*Kabuli*	Ca_Kabuli_denovo	15397292	(G/C)
21376	CakSNP21376	*Kabuli*	Ca_Kabuli_denovo	15397334	(C/G)
21377	CakSNP21377	*Kabuli*	Ca_Kabuli_denovo	15400127	(G/A)
21378	CakSNP21378	*Kabuli*	Ca_Kabuli_denovo	15400133	(A/C)
21379	CakSNP21379	*Kabuli*	Ca_Kabuli_denovo	15401000	(A/G)
21380	CakSNP21380	*Kabuli*	Ca_Kabuli_denovo	15401033	(G/T)
21381	CakSNP21381	*Kabuli*	Ca_Kabuli_denovo	15406282	(A/C)
21382	CakSNP21382	*Kabuli*	Ca_Kabuli_denovo	15406287	(G/C)
21383	CakSNP21383	*Kabuli*	Ca_Kabuli_denovo	15406288	(G/T)
21384	CakSNP21384	*Kabuli*	Ca_Kabuli_denovo	15407414	(T/G)
21385	CakSNP21385	*Kabuli*	Ca_Kabuli_denovo	15407444	(A/G)
21386	CakSNP21386	*Kabuli*	Ca_Kabuli_denovo	15414998	(A/G)
21387	CakSNP21387	*Kabuli*	Ca_Kabuli_denovo	15417467	(A/G)
21388	CakSNP21388	*Kabuli*	Ca_Kabuli_denovo	15424718	(C/G)
21389	CakSNP21389	*Kabuli*	Ca_Kabuli_denovo	15425714	(A/G)
21390	CakSNP21390	*Kabuli*	Ca_Kabuli_denovo	15426125	(A/G)
21391	CakSNP21391	*Kabuli*	Ca_Kabuli_denovo	15428336	(G/C)
21392	CakSNP21392	*Kabuli*	Ca_Kabuli_denovo	15430885	(T/G)
21393	CakSNP21393	*Kabuli*	Ca_Kabuli_denovo	15431873	(G/C)

S.N.	SNP IDs	Cultivars	Chromosomes/scaffolds	Physical positions (bp)	SNPs
21394	CakSNP21394	*Kabuli*	Ca_Kabuli_denovo	15431888	(C/T)
21395	CakSNP21395	*Kabuli*	Ca_Kabuli_denovo	15431906	(C/G)
21396	CakSNP21396	*Kabuli*	Ca_Kabuli_denovo	15431918	(C/T)
21397	CakSNP21397	*Kabuli*	Ca_Kabuli_denovo	15440132	(A/C)
21398	CakSNP21398	*Kabuli*	Ca_Kabuli_denovo	15441290	(A/C)
21399	CakSNP21399	*Kabuli*	Ca_Kabuli_denovo	15441305	(A/G)
21400	CakSNP21400	*Kabuli*	Ca_Kabuli_denovo	15455235	(G/T)
21401	CakSNP21401	*Kabuli*	Ca_Kabuli_denovo	15455296	(C/G)
21402	CakSNP21402	*Kabuli*	Ca_Kabuli_denovo	15456996	(G/A)
21403	CakSNP21403	*Kabuli*	Ca_Kabuli_denovo	15458395	(C/T)
21404	CakSNP21404	*Kabuli*	Ca_Kabuli_denovo	15458942	(G/A)
21405	CakSNP21405	*Kabuli*	Ca_Kabuli_denovo	15460422	(A/G)
21406	CakSNP21406	*Kabuli*	Ca_Kabuli_denovo	15460460	(G/A)
21407	CakSNP21407	*Kabuli*	Ca_Kabuli_denovo	15464054	(C/T)
21408	CakSNP21408	*Kabuli*	Ca_Kabuli_denovo	15464063	(A/G)
21409	CakSNP21409	*Kabuli*	Ca_Kabuli_denovo	15464257	(C/T)
21410	CakSNP21410	*Kabuli*	Ca_Kabuli_denovo	15464599	(A/G)
21411	CakSNP21411	*Kabuli*	Ca_Kabuli_denovo	15468646	(C/T)
21412	CakSNP21412	*Kabuli*	Ca_Kabuli_denovo	15469155	(C/T)
21413	CakSNP21413	*Kabuli*	Ca_Kabuli_denovo	15474029	(A/G)
21414	CakSNP21414	*Kabuli*	Ca_Kabuli_denovo	15474040	(T/G)
21415	CakSNP21415	*Kabuli*	Ca_Kabuli_denovo	15474052	(C/A)
21416	CakSNP21416	*Kabuli*	Ca_Kabuli_denovo	15474082	(G/T)
21417	CakSNP21417	*Kabuli*	Ca_Kabuli_denovo	15475209	(C/G)
21418	CakSNP21418	*Kabuli*	Ca_Kabuli_denovo	15475225	(T/C)
21419	CakSNP21419	*Kabuli*	Ca_Kabuli_denovo	15478410	(T/G)
21420	CakSNP21420	*Kabuli*	Ca_Kabuli_denovo	15480813	(C/T)
21421	CakSNP21421	*Kabuli*	Ca_Kabuli_denovo	15480822	(C/T)
21422	CakSNP21422	*Kabuli*	Ca_Kabuli_denovo	15491990	(T/C)
21423	CakSNP21423	*Kabuli*	Ca_Kabuli_denovo	15491996	(G/A)
21424	CakSNP21424	*Kabuli*	Ca_Kabuli_denovo	15499304	(G/T)
21425	CakSNP21425	*Kabuli*	Ca_Kabuli_denovo	15499316	(T/C)
21426	CakSNP21426	*Kabuli*	Ca_Kabuli_denovo	15499338	(T/G)
21427	CakSNP21427	*Kabuli*	Ca_Kabuli_denovo	15506868	(C/T)
21428	CakSNP21428	*Kabuli*	Ca_Kabuli_denovo	15507953	(G/T)
21429	CakSNP21429	*Kabuli*	Ca_Kabuli_denovo	15507965	(A/G)
21430	CakSNP21430	*Kabuli*	Ca_Kabuli_denovo	15521689	(T/C)
21431	CakSNP21431	*Kabuli*	Ca_Kabuli_denovo	15522761	(G/A)

S.N.	SNP IDs	Cultivars	Chromosomes/scaffolds	Physical positions (bp)	SNPs
21432	CakSNP21432	*Kabuli*	Ca_Kabuli_denovo	15522773	(G/A)
21433	CakSNP21433	*Kabuli*	Ca_Kabuli_denovo	15524716	(G/T)
21434	CakSNP21434	*Kabuli*	Ca_Kabuli_denovo	15524717	(T/C)
21435	CakSNP21435	*Kabuli*	Ca_Kabuli_denovo	15524990	(T/C)
21436	CakSNP21436	*Kabuli*	Ca_Kabuli_denovo	15526190	(A/G)
21437	CakSNP21437	*Kabuli*	Ca_Kabuli_denovo	15530285	(T/G)
21438	CakSNP21438	*Kabuli*	Ca_Kabuli_denovo	15531595	(T/C)
21439	CakSNP21439	*Kabuli*	Ca_Kabuli_denovo	15552032	(A/G)
21440	CakSNP21440	*Kabuli*	Ca_Kabuli_denovo	15564979	(C/T)
21441	CakSNP21441	*Kabuli*	Ca_Kabuli_denovo	15566724	(T/A)
21442	CakSNP21442	*Kabuli*	Ca_Kabuli_denovo	15568183	(T/C)
21443	CakSNP21443	*Kabuli*	Ca_Kabuli_denovo	15568192	(G/T)
21444	CakSNP21444	*Kabuli*	Ca_Kabuli_denovo	15575379	(A/G)
21445	CakSNP21445	*Kabuli*	Ca_Kabuli_denovo	15575382	(G/C)
21446	CakSNP21446	*Kabuli*	Ca_Kabuli_denovo	15576520	(G/C)
21447	CakSNP21447	*Kabuli*	Ca_Kabuli_denovo	15582787	(T/C)
21448	CakSNP21448	*Kabuli*	Ca_Kabuli_denovo	15582800	(C/G)
21449	CakSNP21449	*Kabuli*	Ca_Kabuli_denovo	15590817	(A/G)
21450	CakSNP21450	*Kabuli*	Ca_Kabuli_denovo	15591832	(T/C)
21451	CakSNP21451	*Kabuli*	Ca_Kabuli_denovo	15595473	(C/A)
21452	CakSNP21452	*Kabuli*	Ca_Kabuli_denovo	15597791	(G/A)
21453	CakSNP21453	*Kabuli*	Ca_Kabuli_denovo	15597799	(C/A)
21454	CakSNP21454	*Kabuli*	Ca_Kabuli_denovo	15603179	(A/G)
21455	CakSNP21455	*Kabuli*	Ca_Kabuli_denovo	15603193	(T/C)
21456	CakSNP21456	*Kabuli*	Ca_Kabuli_denovo	15606500	(G/A)
21457	CakSNP21457	*Kabuli*	Ca_Kabuli_denovo	15609823	(G/A)
21458	CakSNP21458	*Kabuli*	Ca_Kabuli_denovo	15611115	(G/C)
21459	CakSNP21459	*Kabuli*	Ca_Kabuli_denovo	15611121	(C/T)
21460	CakSNP21460	*Kabuli*	Ca_Kabuli_denovo	15620902	(C/T)
21461	CakSNP21461	*Kabuli*	Ca_Kabuli_denovo	15620920	(G/T)
21462	CakSNP21462	*Kabuli*	Ca_Kabuli_denovo	15620944	(C/G)
21463	CakSNP21463	*Kabuli*	Ca_Kabuli_denovo	15621127	(G/A)
21464	CakSNP21464	*Kabuli*	Ca_Kabuli_denovo	15628387	(T/C)
21465	CakSNP21465	*Kabuli*	Ca_Kabuli_denovo	15631264	(G/T)
21466	CakSNP21466	*Kabuli*	Ca_Kabuli_denovo	15631270	(A/G)
21467	CakSNP21467	*Kabuli*	Ca_Kabuli_denovo	15631562	(C/A)
21468	CakSNP21468	*Kabuli*	Ca_Kabuli_denovo	15633158	(C/A)
21469	CakSNP21469	*Kabuli*	Ca_Kabuli_denovo	15637429	(G/T)

S.N.	SNP IDs	Cultivars	Chromosomes/scaffolds	Physical positions (bp)	SNPs
21470	CakSNP21470	*Kabuli*	Ca_Kabuli_denovo	15638521	(C/G)
21471	CakSNP21471	*Kabuli*	Ca_Kabuli_denovo	15638548	(A/G)
21472	CakSNP21472	*Kabuli*	Ca_Kabuli_denovo	15640495	(C/T)
21473	CakSNP21473	*Kabuli*	Ca_Kabuli_denovo	15643259	(G/A)
21474	CakSNP21474	*Kabuli*	Ca_Kabuli_denovo	15643271	(T/C)
21475	CakSNP21475	*Kabuli*	Ca_Kabuli_denovo	15643735	(C/T)
21476	CakSNP21476	*Kabuli*	Ca_Kabuli_denovo	15643743	(A/G)
21477	CakSNP21477	*Kabuli*	Ca_Kabuli_denovo	15643912	(T/C)
21478	CakSNP21478	*Kabuli*	Ca_Kabuli_denovo	15643960	(C/T)
21479	CakSNP21479	*Kabuli*	Ca_Kabuli_denovo	15644967	(A/C)
21480	CakSNP21480	*Kabuli*	Ca_Kabuli_denovo	15647635	(A/G)
21481	CakSNP21481	*Kabuli*	Ca_Kabuli_denovo	15650816	(G/A)
21482	CakSNP21482	*Kabuli*	Ca_Kabuli_denovo	15656144	(T/C)
21483	CakSNP21483	*Kabuli*	Ca_Kabuli_denovo	15662922	(A/G)
21484	CakSNP21484	*Kabuli*	Ca_Kabuli_denovo	15662923	(A/G)
21485	CakSNP21485	*Kabuli*	Ca_Kabuli_denovo	15668904	(C/G)
21486	CakSNP21486	*Kabuli*	Ca_Kabuli_denovo	15672757	(G/A)
21487	CakSNP21487	*Kabuli*	Ca_Kabuli_denovo	15672819	(C/A)
21488	CakSNP21488	*Kabuli*	Ca_Kabuli_denovo	15676871	(A/G)
21489	CakSNP21489	*Kabuli*	Ca_Kabuli_denovo	15679048	(C/T)
21490	CakSNP21490	*Kabuli*	Ca_Kabuli_denovo	15682338	(A/G)
21491	CakSNP21491	*Kabuli*	Ca_Kabuli_denovo	15686389	(G/A)
21492	CakSNP21492	*Kabuli*	Ca_Kabuli_denovo	15689140	(T/G)
21493	CakSNP21493	*Kabuli*	Ca_Kabuli_denovo	15689179	(A/G)
21494	CakSNP21494	*Kabuli*	Ca_Kabuli_denovo	15693017	(A/G)
21495	CakSNP21495	*Kabuli*	Ca_Kabuli_denovo	15693033	(A/C)
21496	CakSNP21496	*Kabuli*	Ca_Kabuli_denovo	15696764	(T/G)
21497	CakSNP21497	*Kabuli*	Ca_Kabuli_denovo	15710031	(A/C)
21498	CakSNP21498	*Kabuli*	Ca_Kabuli_denovo	15724677	(T/C)
21499	CakSNP21499	*Kabuli*	Ca_Kabuli_denovo	15729201	(C/T)
21500	CakSNP21500	*Kabuli*	Ca_Kabuli_denovo	15730162	(T/C)
21501	CakSNP21501	*Kabuli*	Ca_Kabuli_denovo	15731996	(T/C)
21502	CakSNP21502	*Kabuli*	Ca_Kabuli_denovo	15732030	(C/T)
21503	CakSNP21503	*Kabuli*	Ca_Kabuli_denovo	15737951	(G/C)
21504	CakSNP21504	*Kabuli*	Ca_Kabuli_denovo	15738002	(A/G)
21505	CakSNP21505	*Kabuli*	Ca_Kabuli_denovo	15738908	(T/C)
21506	CakSNP21506	*Kabuli*	Ca_Kabuli_denovo	15738922	(T/C)
21507	CakSNP21507	*Kabuli*	Ca_Kabuli_denovo	15742863	(C/T)

S.N.	SNP IDs	Cultivars	Chromosomes/scaffolds	Physical positions (bp)	SNPs
21508	CakSNP21508	*Kabuli*	Ca_Kabuli_denovo	15746595	(T/G)
21509	CakSNP21509	*Kabuli*	Ca_Kabuli_denovo	15746721	(A/G)
21510	CakSNP21510	*Kabuli*	Ca_Kabuli_denovo	15748786	(T/C)
21511	CakSNP21511	*Kabuli*	Ca_Kabuli_denovo	15750852	(A/C)
21512	CakSNP21512	*Kabuli*	Ca_Kabuli_denovo	15750903	(T/A)
21513	CakSNP21513	*Kabuli*	Ca_Kabuli_denovo	15750933	(C/T)
21514	CakSNP21514	*Kabuli*	Ca_Kabuli_denovo	15756831	(C/A)
21515	CakSNP21515	*Kabuli*	Ca_Kabuli_denovo	15760646	(C/G)
21516	CakSNP21516	*Kabuli*	Ca_Kabuli_denovo	15760923	(A/C)
21517	CakSNP21517	*Kabuli*	Ca_Kabuli_denovo	15766715	(C/T)
21518	CakSNP21518	*Kabuli*	Ca_Kabuli_denovo	15766718	(G/T)
21519	CakSNP21519	*Kabuli*	Ca_Kabuli_denovo	15769597	(C/T)
21520	CakSNP21520	*Kabuli*	Ca_Kabuli_denovo	15775001	(G/A)
21521	CakSNP21521	*Kabuli*	Ca_Kabuli_denovo	15777288	(T/G)
21522	CakSNP21522	*Kabuli*	Ca_Kabuli_denovo	15779882	(G/C)
21523	CakSNP21523	*Kabuli*	Ca_Kabuli_denovo	15780781	(C/T)
21524	CakSNP21524	*Kabuli*	Ca_Kabuli_denovo	15781142	(G/A)
21525	CakSNP21525	*Kabuli*	Ca_Kabuli_denovo	15781160	(G/A)
21526	CakSNP21526	*Kabuli*	Ca_Kabuli_denovo	15791427	(C/A)
21527	CakSNP21527	*Kabuli*	Ca_Kabuli_denovo	15791472	(C/G)
21528	CakSNP21528	*Kabuli*	Ca_Kabuli_denovo	15792255	(C/T)
21529	CakSNP21529	*Kabuli*	Ca_Kabuli_denovo	15806405	(G/A)
21530	CakSNP21530	*Kabuli*	Ca_Kabuli_denovo	15816444	(G/C)
21531	CakSNP21531	*Kabuli*	Ca_Kabuli_denovo	15816474	(A/C)
21532	CakSNP21532	*Kabuli*	Ca_Kabuli_denovo	15817830	(A/G)
21533	CakSNP21533	*Kabuli*	Ca_Kabuli_denovo	15820384	(T/C)
21534	CakSNP21534	*Kabuli*	Ca_Kabuli_denovo	15827646	(G/C)
21535	CakSNP21535	*Kabuli*	Ca_Kabuli_denovo	15835243	(A/T)
21536	CakSNP21536	*Kabuli*	Ca_Kabuli_denovo	15840418	(C/T)
21537	CakSNP21537	*Kabuli*	Ca_Kabuli_denovo	15843233	(T/A)
21538	CakSNP21538	*Kabuli*	Ca_Kabuli_denovo	15846580	(C/T)
21539	CakSNP21539	*Kabuli*	Ca_Kabuli_denovo	15846593	(T/C)
21540	CakSNP21540	*Kabuli*	Ca_Kabuli_denovo	15848981	(A/G)
21541	CakSNP21541	*Kabuli*	Ca_Kabuli_denovo	15848984	(G/C)
21542	CakSNP21542	*Kabuli*	Ca_Kabuli_denovo	15853479	(G/C)
21543	CakSNP21543	*Kabuli*	Ca_Kabuli_denovo	15855332	(G/T)
21544	CakSNP21544	*Kabuli*	Ca_Kabuli_denovo	15857901	(A/G)
21545	CakSNP21545	*Kabuli*	Ca_Kabuli_denovo	15858482	(A/G)

S.N.	SNP IDs	Cultivars	Chromosomes/scaffolds	Physical positions (bp)	SNPs
21546	CakSNP21546	*Kabuli*	Ca_Kabuli_denovo	15858484	(C/G)
21547	CakSNP21547	*Kabuli*	Ca_Kabuli_denovo	15866668	(A/G)
21548	CakSNP21548	*Kabuli*	Ca_Kabuli_denovo	15867686	(T/G)
21549	CakSNP21549	*Kabuli*	Ca_Kabuli_denovo	15873998	(C/T)
21550	CakSNP21550	*Kabuli*	Ca_Kabuli_denovo	15874929	(G/A)
21551	CakSNP21551	*Kabuli*	Ca_Kabuli_denovo	15874948	(A/G)
21552	CakSNP21552	*Kabuli*	Ca_Kabuli_denovo	15875007	(G/A)
21553	CakSNP21553	*Kabuli*	Ca_Kabuli_denovo	15876783	(A/G)
21554	CakSNP21554	*Kabuli*	Ca_Kabuli_denovo	15884976	(C/G)
21555	CakSNP21555	*Kabuli*	Ca_Kabuli_denovo	15887807	(G/A)
21556	CakSNP21556	*Kabuli*	Ca_Kabuli_denovo	15892953	(T/C)
21557	CakSNP21557	*Kabuli*	Ca_Kabuli_denovo	15893254	(T/C)
21558	CakSNP21558	*Kabuli*	Ca_Kabuli_denovo	15893260	(A/G)
21559	CakSNP21559	*Kabuli*	Ca_Kabuli_denovo	15893327	(A/C)
21560	CakSNP21560	*Kabuli*	Ca_Kabuli_denovo	15893329	(A/C)
21561	CakSNP21561	*Kabuli*	Ca_Kabuli_denovo	15897534	(A/G)
21562	CakSNP21562	*Kabuli*	Ca_Kabuli_denovo	15897602	(C/T)
21563	CakSNP21563	*Kabuli*	Ca_Kabuli_denovo	15903773	(G/T)
21564	CakSNP21564	*Kabuli*	Ca_Kabuli_denovo	15903815	(A/G)
21565	CakSNP21565	*Kabuli*	Ca_Kabuli_denovo	15905649	(C/G)
21566	CakSNP21566	*Kabuli*	Ca_Kabuli_denovo	15907232	(T/C)
21567	CakSNP21567	*Kabuli*	Ca_Kabuli_denovo	15908094	(G/C)
21568	CakSNP21568	*Kabuli*	Ca_Kabuli_denovo	15911035	(T/C)
21569	CakSNP21569	*Kabuli*	Ca_Kabuli_denovo	15924073	(C/T)
21570	CakSNP21570	*Kabuli*	Ca_Kabuli_denovo	15929633	(T/A)
21571	CakSNP21571	*Kabuli*	Ca_Kabuli_denovo	15929674	(T/C)
21572	CakSNP21572	*Kabuli*	Ca_Kabuli_denovo	15930740	(G/A)
21573	CakSNP21573	*Kabuli*	Ca_Kabuli_denovo	15930808	(C/T)
21574	CakSNP21574	*Kabuli*	Ca_Kabuli_denovo	15931690	(C/G)
21575	CakSNP21575	*Kabuli*	Ca_Kabuli_denovo	15931714	(G/T)
21576	CakSNP21576	*Kabuli*	Ca_Kabuli_denovo	15932330	(A/G)
21577	CakSNP21577	*Kabuli*	Ca_Kabuli_denovo	15934836	(C/T)
21578	CakSNP21578	*Kabuli*	Ca_Kabuli_denovo	15937570	(A/G)
21579	CakSNP21579	*Kabuli*	Ca_Kabuli_denovo	15951873	(G/A)
21580	CakSNP21580	*Kabuli*	Ca_Kabuli_denovo	15952688	(A/G)
21581	CakSNP21581	*Kabuli*	Ca_Kabuli_denovo	15952703	(A/G)
21582	CakSNP21582	*Kabuli*	Ca_Kabuli_denovo	15970245	(G/A)
21583	CakSNP21583	*Kabuli*	Ca_Kabuli_denovo	15972570	(G/T)

S.N.	SNP IDs	Cultivars	Chromosomes/scaffolds	Physical positions (bp)	SNPs
21584	CakSNP21584	*Kabuli*	Ca_Kabuli_denovo	15972591	(C/G)
21585	CakSNP21585	*Kabuli*	Ca_Kabuli_denovo	15977624	(G/T)
21586	CakSNP21586	*Kabuli*	Ca_Kabuli_denovo	15980240	(A/T)
21587	CakSNP21587	*Kabuli*	Ca_Kabuli_denovo	15992568	(C/G)
21588	CakSNP21588	*Kabuli*	Ca_Kabuli_denovo	15996800	(A/C)
21589	CakSNP21589	*Kabuli*	Ca_Kabuli_denovo	15997796	(A/T)
21590	CakSNP21590	*Kabuli*	Ca_Kabuli_denovo	15997817	(G/A)
21591	CakSNP21591	*Kabuli*	Ca_Kabuli_denovo	16007356	(G/C)
21592	CakSNP21592	*Kabuli*	Ca_Kabuli_denovo	16016229	(T/C)
21593	CakSNP21593	*Kabuli*	Ca_Kabuli_denovo	16018177	(C/T)
21594	CakSNP21594	*Kabuli*	Ca_Kabuli_denovo	16019462	(G/C)
21595	CakSNP21595	*Kabuli*	Ca_Kabuli_denovo	16032952	(G/C)
21596	CakSNP21596	*Kabuli*	Ca_Kabuli_denovo	16035104	(T/A)
21597	CakSNP21597	*Kabuli*	Ca_Kabuli_denovo	16035142	(T/C)
21598	CakSNP21598	*Kabuli*	Ca_Kabuli_denovo	16042128	(A/C)
21599	CakSNP21599	*Kabuli*	Ca_Kabuli_denovo	16045168	(G/T)
21600	CakSNP21600	*Kabuli*	Ca_Kabuli_denovo	16046908	(A/G)
21601	CakSNP21601	*Kabuli*	Ca_Kabuli_denovo	16051304	(C/T)
21602	CakSNP21602	*Kabuli*	Ca_Kabuli_denovo	16053222	(G/A)
21603	CakSNP21603	*Kabuli*	Ca_Kabuli_denovo	16059216	(G/C)
21604	CakSNP21604	*Kabuli*	Ca_Kabuli_denovo	16063343	(G/A)
21605	CakSNP21605	*Kabuli*	Ca_Kabuli_denovo	16063447	(T/G)
21606	CakSNP21606	*Kabuli*	Ca_Kabuli_denovo	16063460	(A/C)
21607	CakSNP21607	*Kabuli*	Ca_Kabuli_denovo	16064860	(C/G)
21608	CakSNP21608	*Kabuli*	Ca_Kabuli_denovo	16072684	(A/G)
21609	CakSNP21609	*Kabuli*	Ca_Kabuli_denovo	16077406	(A/C)
21610	CakSNP21610	*Kabuli*	Ca_Kabuli_denovo	16083241	(T/G)
21611	CakSNP21611	*Kabuli*	Ca_Kabuli_denovo	16088745	(T/C)
21612	CakSNP21612	*Kabuli*	Ca_Kabuli_denovo	16088999	(A/C)
21613	CakSNP21613	*Kabuli*	Ca_Kabuli_denovo	16089001	(A/C)
21614	CakSNP21614	*Kabuli*	Ca_Kabuli_denovo	16091858	(T/G)
21615	CakSNP21615	*Kabuli*	Ca_Kabuli_denovo	16104177	(G/A)
21616	CakSNP21616	*Kabuli*	Ca_Kabuli_denovo	16107450	(C/T)
21617	CakSNP21617	*Kabuli*	Ca_Kabuli_denovo	16107506	(G/A)
21618	CakSNP21618	*Kabuli*	Ca_Kabuli_denovo	16114582	(A/G)
21619	CakSNP21619	*Kabuli*	Ca_Kabuli_denovo	16115155	(C/T)
21620	CakSNP21620	*Kabuli*	Ca_Kabuli_denovo	16115164	(C/A)
21621	CakSNP21621	*Kabuli*	Ca_Kabuli_denovo	16116836	(T/G)

S.N.	SNP IDs	Cultivars	Chromosomes/scaffolds	Physical positions (bp)	SNPs
21622	CakSNP21622	*Kabuli*	Ca_Kabuli_denovo	16116890	(T/G)
21623	CakSNP21623	*Kabuli*	Ca_Kabuli_denovo	16118811	(T/C)
21624	CakSNP21624	*Kabuli*	Ca_Kabuli_denovo	16118838	(G/A)
21625	CakSNP21625	*Kabuli*	Ca_Kabuli_denovo	16119632	(A/G)
21626	CakSNP21626	*Kabuli*	Ca_Kabuli_denovo	16134211	(G/A)
21627	CakSNP21627	*Kabuli*	Ca_Kabuli_denovo	16134249	(G/A)
21628	CakSNP21628	*Kabuli*	Ca_Kabuli_denovo	16134580	(G/A)
21629	CakSNP21629	*Kabuli*	Ca_Kabuli_denovo	16143666	(A/G)
21630	CakSNP21630	*Kabuli*	Ca_Kabuli_denovo	16144928	(A/G)
21631	CakSNP21631	*Kabuli*	Ca_Kabuli_denovo	16146300	(C/G)
21632	CakSNP21632	*Kabuli*	Ca_Kabuli_denovo	16152938	(G/A)
21633	CakSNP21633	*Kabuli*	Ca_Kabuli_denovo	16159144	(T/C)
21634	CakSNP21634	*Kabuli*	Ca_Kabuli_denovo	16165826	(C/G)
21635	CakSNP21635	*Kabuli*	Ca_Kabuli_denovo	16165837	(G/A)
21636	CakSNP21636	*Kabuli*	Ca_Kabuli_denovo	16167983	(C/T)
21637	CakSNP21637	*Kabuli*	Ca_Kabuli_denovo	16171075	(G/A)
21638	CakSNP21638	*Kabuli*	Ca_Kabuli_denovo	16173457	(T/G)
21639	CakSNP21639	*Kabuli*	Ca_Kabuli_denovo	16173469	(A/G)
21640	CakSNP21640	*Kabuli*	Ca_Kabuli_denovo	16180337	(A/T)
21641	CakSNP21641	*Kabuli*	Ca_Kabuli_denovo	16180369	(G/C)
21642	CakSNP21642	*Kabuli*	Ca_Kabuli_denovo	16180679	(T/C)
21643	CakSNP21643	*Kabuli*	Ca_Kabuli_denovo	16180694	(G/A)
21644	CakSNP21644	*Kabuli*	Ca_Kabuli_denovo	16180697	(G/A)
21645	CakSNP21645	*Kabuli*	Ca_Kabuli_denovo	16192364	(T/G)
21646	CakSNP21646	*Kabuli*	Ca_Kabuli_denovo	16195262	(A/G)
21647	CakSNP21647	*Kabuli*	Ca_Kabuli_denovo	16195265	(C/A)
21648	CakSNP21648	*Kabuli*	Ca_Kabuli_denovo	16202129	(C/G)
21649	CakSNP21649	*Kabuli*	Ca_Kabuli_denovo	16202257	(G/T)
21650	CakSNP21650	*Kabuli*	Ca_Kabuli_denovo	16203514	(A/G)
21651	CakSNP21651	*Kabuli*	Ca_Kabuli_denovo	16212822	(A/G)
21652	CakSNP21652	*Kabuli*	Ca_Kabuli_denovo	16212833	(G/T)
21653	CakSNP21653	*Kabuli*	Ca_Kabuli_denovo	16223803	(C/T)
21654	CakSNP21654	*Kabuli*	Ca_Kabuli_denovo	16223835	(A/T)
21655	CakSNP21655	*Kabuli*	Ca_Kabuli_denovo	16226517	(T/G)
21656	CakSNP21656	*Kabuli*	Ca_Kabuli_denovo	16226520	(A/C)
21657	CakSNP21657	*Kabuli*	Ca_Kabuli_denovo	16228102	(T/G)
21658	CakSNP21658	*Kabuli*	Ca_Kabuli_denovo	16231770	(C/T)
21659	CakSNP21659	*Kabuli*	Ca_Kabuli_denovo	16232648	(C/A)

S.N.	SNP IDs	Cultivars	Chromosomes/scaffolds	Physical positions (bp)	SNPs
21660	CakSNP21660	*Kabuli*	Ca_Kabuli_denovo	16234566	(G/T)
21661	CakSNP21661	*Kabuli*	Ca_Kabuli_denovo	16234575	(G/A)
21662	CakSNP21662	*Kabuli*	Ca_Kabuli_denovo	16240526	(G/A)
21663	CakSNP21663	*Kabuli*	Ca_Kabuli_denovo	16244719	(A/T)
21664	CakSNP21664	*Kabuli*	Ca_Kabuli_denovo	16247333	(G/A)
21665	CakSNP21665	*Kabuli*	Ca_Kabuli_denovo	16247374	(C/T)
21666	CakSNP21666	*Kabuli*	Ca_Kabuli_denovo	16260126	(G/C)
21667	CakSNP21667	*Kabuli*	Ca_Kabuli_denovo	16260137	(A/G)
21668	CakSNP21668	*Kabuli*	Ca_Kabuli_denovo	16260601	(C/T)
21669	CakSNP21669	*Kabuli*	Ca_Kabuli_denovo	16260619	(G/A)
21670	CakSNP21670	*Kabuli*	Ca_Kabuli_denovo	16262566	(G/C)
21671	CakSNP21671	*Kabuli*	Ca_Kabuli_denovo	16263211	(C/T)
21672	CakSNP21672	*Kabuli*	Ca_Kabuli_denovo	16263606	(A/T)
21673	CakSNP21673	*Kabuli*	Ca_Kabuli_denovo	16263609	(T/C)
21674	CakSNP21674	*Kabuli*	Ca_Kabuli_denovo	16265172	(C/G)
21675	CakSNP21675	*Kabuli*	Ca_Kabuli_denovo	16266042	(T/G)
21676	CakSNP21676	*Kabuli*	Ca_Kabuli_denovo	16280261	(C/A)
21677	CakSNP21677	*Kabuli*	Ca_Kabuli_denovo	16280269	(C/T)
21678	CakSNP21678	*Kabuli*	Ca_Kabuli_denovo	16285150	(C/T)
21679	CakSNP21679	*Kabuli*	Ca_Kabuli_denovo	16285196	(C/T)
21680	CakSNP21680	*Kabuli*	Ca_Kabuli_denovo	16285228	(G/C)
21681	CakSNP21681	*Kabuli*	Ca_Kabuli_denovo	16287350	(G/C)
21682	CakSNP21682	*Kabuli*	Ca_Kabuli_denovo	16287361	(C/T)
21683	CakSNP21683	*Kabuli*	Ca_Kabuli_denovo	16293927	(C/T)
21684	CakSNP21684	*Kabuli*	Ca_Kabuli_denovo	16304638	(C/G)
21685	CakSNP21685	*Kabuli*	Ca_Kabuli_denovo	16304644	(G/A)
21686	CakSNP21686	*Kabuli*	Ca_Kabuli_denovo	16309405	(C/T)
21687	CakSNP21687	*Kabuli*	Ca_Kabuli_denovo	16310098	(A/C)
21688	CakSNP21688	*Kabuli*	Ca_Kabuli_denovo	16311865	(A/G)
21689	CakSNP21689	*Kabuli*	Ca_Kabuli_denovo	16318334	(A/C)
21690	CakSNP21690	*Kabuli*	Ca_Kabuli_denovo	16321322	(G/A)
21691	CakSNP21691	*Kabuli*	Ca_Kabuli_denovo	16321357	(G/T)
21692	CakSNP21692	*Kabuli*	Ca_Kabuli_denovo	16323646	(C/T)
21693	CakSNP21693	*Kabuli*	Ca_Kabuli_denovo	16323653	(G/A)
21694	CakSNP21694	*Kabuli*	Ca_Kabuli_denovo	16324174	(T/C)
21695	CakSNP21695	*Kabuli*	Ca_Kabuli_denovo	16324198	(G/C)
21696	CakSNP21696	*Kabuli*	Ca_Kabuli_denovo	16326296	(A/G)
21697	CakSNP21697	*Kabuli*	Ca_Kabuli_denovo	16326326	(T/C)

S.N.	SNP IDs	Cultivars	Chromosomes/scaffolds	Physical positions (bp)	SNPs
21698	CakSNP21698	*Kabuli*	Ca_Kabuli_denovo	16327612	(G/T)
21699	CakSNP21699	*Kabuli*	Ca_Kabuli_denovo	16329733	(T/G)
21700	CakSNP21700	*Kabuli*	Ca_Kabuli_denovo	16329739	(T/C)
21701	CakSNP21701	*Kabuli*	Ca_Kabuli_denovo	16331645	(T/C)
21702	CakSNP21702	*Kabuli*	Ca_Kabuli_denovo	16332497	(A/G)
21703	CakSNP21703	*Kabuli*	Ca_Kabuli_denovo	16335146	(A/G)
21704	CakSNP21704	*Kabuli*	Ca_Kabuli_denovo	16337697	(A/C)
21705	CakSNP21705	*Kabuli*	Ca_Kabuli_denovo	16338451	(T/C)
21706	CakSNP21706	*Kabuli*	Ca_Kabuli_denovo	16338474	(T/G)
21707	CakSNP21707	*Kabuli*	Ca_Kabuli_denovo	16345856	(C/G)
21708	CakSNP21708	*Kabuli*	Ca_Kabuli_denovo	16360422	(C/T)
21709	CakSNP21709	*Kabuli*	Ca_Kabuli_denovo	16366147	(A/C)
21710	CakSNP21710	*Kabuli*	Ca_Kabuli_denovo	16366177	(T/C)
21711	CakSNP21711	*Kabuli*	Ca_Kabuli_denovo	16374055	(C/T)
21712	CakSNP21712	*Kabuli*	Ca_Kabuli_denovo	16374136	(C/A)
21713	CakSNP21713	*Kabuli*	Ca_Kabuli_denovo	16379800	(G/A)
21714	CakSNP21714	*Kabuli*	Ca_Kabuli_denovo	16380555	(A/G)
21715	CakSNP21715	*Kabuli*	Ca_Kabuli_denovo	16382611	(C/T)
21716	CakSNP21716	*Kabuli*	Ca_Kabuli_denovo	16386909	(T/G)
21717	CakSNP21717	*Kabuli*	Ca_Kabuli_denovo	16386939	(A/G)
21718	CakSNP21718	*Kabuli*	Ca_Kabuli_denovo	16395993	(A/C)
21719	CakSNP21719	*Kabuli*	Ca_Kabuli_denovo	16395994	(G/A)
21720	CakSNP21720	*Kabuli*	Ca_Kabuli_denovo	16396510	(A/G)
21721	CakSNP21721	*Kabuli*	Ca_Kabuli_denovo	16396541	(A/G)
21722	CakSNP21722	*Kabuli*	Ca_Kabuli_denovo	16399727	(C/G)
21723	CakSNP21723	*Kabuli*	Ca_Kabuli_denovo	16400456	(A/C)
21724	CakSNP21724	*Kabuli*	Ca_Kabuli_denovo	16400458	(A/G)
21725	CakSNP21725	*Kabuli*	Ca_Kabuli_denovo	16403710	(T/G)
21726	CakSNP21726	*Kabuli*	Ca_Kabuli_denovo	16407355	(T/G)
21727	CakSNP21727	*Kabuli*	Ca_Kabuli_denovo	16407358	(G/C)
21728	CakSNP21728	*Kabuli*	Ca_Kabuli_denovo	16408072	(T/C)
21729	CakSNP21729	*Kabuli*	Ca_Kabuli_denovo	16419017	(G/T)
21730	CakSNP21730	*Kabuli*	Ca_Kabuli_denovo	16419044	(T/C)
21731	CakSNP21731	*Kabuli*	Ca_Kabuli_denovo	16422703	(G/C)
21732	CakSNP21732	*Kabuli*	Ca_Kabuli_denovo	16423796	(C/G)
21733	CakSNP21733	*Kabuli*	Ca_Kabuli_denovo	16431153	(A/G)
21734	CakSNP21734	*Kabuli*	Ca_Kabuli_denovo	16431161	(G/A)
21735	CakSNP21735	*Kabuli*	Ca_Kabuli_denovo	16432329	(A/G)

S.N.	SNP IDs	Cultivars	Chromosomes/scaffolds	Physical positions (bp)	SNPs
21736	CakSNP21736	*Kabuli*	Ca_Kabuli_denovo	16433327	(G/A)
21737	CakSNP21737	*Kabuli*	Ca_Kabuli_denovo	16433379	(A/G)
21738	CakSNP21738	*Kabuli*	Ca_Kabuli_denovo	16433532	(T/C)
21739	CakSNP21739	*Kabuli*	Ca_Kabuli_denovo	16433951	(A/G)
21740	CakSNP21740	*Kabuli*	Ca_Kabuli_denovo	16433958	(T/C)
21741	CakSNP21741	*Kabuli*	Ca_Kabuli_denovo	16433964	(T/C)
21742	CakSNP21742	*Kabuli*	Ca_Kabuli_denovo	16438515	(G/C)
21743	CakSNP21743	*Kabuli*	Ca_Kabuli_denovo	16438572	(G/T)
21744	CakSNP21744	*Kabuli*	Ca_Kabuli_denovo	16439398	(G/C)
21745	CakSNP21745	*Kabuli*	Ca_Kabuli_denovo	16439416	(T/C)
21746	CakSNP21746	*Kabuli*	Ca_Kabuli_denovo	16442634	(G/A)
21747	CakSNP21747	*Kabuli*	Ca_Kabuli_denovo	16442868	(A/T)
21748	CakSNP21748	*Kabuli*	Ca_Kabuli_denovo	16443463	(G/A)
21749	CakSNP21749	*Kabuli*	Ca_Kabuli_denovo	16443487	(C/T)
21750	CakSNP21750	*Kabuli*	Ca_Kabuli_denovo	16446001	(T/C)
21751	CakSNP21751	*Kabuli*	Ca_Kabuli_denovo	16446028	(A/T)
21752	CakSNP21752	*Kabuli*	Ca_Kabuli_denovo	16446612	(C/G)
21753	CakSNP21753	*Kabuli*	Ca_Kabuli_denovo	16446660	(C/T)
21754	CakSNP21754	*Kabuli*	Ca_Kabuli_denovo	16448652	(C/T)
21755	CakSNP21755	*Kabuli*	Ca_Kabuli_denovo	16448673	(C/T)
21756	CakSNP21756	*Kabuli*	Ca_Kabuli_denovo	16450928	(C/T)
21757	CakSNP21757	*Kabuli*	Ca_Kabuli_denovo	16450936	(G/C)
21758	CakSNP21758	*Kabuli*	Ca_Kabuli_denovo	16454737	(A/G)
21759	CakSNP21759	*Kabuli*	Ca_Kabuli_denovo	16454742	(C/T)
21760	CakSNP21760	*Kabuli*	Ca_Kabuli_denovo	16463528	(C/G)
21761	CakSNP21761	*Kabuli*	Ca_Kabuli_denovo	16463533	(A/C)
21762	CakSNP21762	*Kabuli*	Ca_Kabuli_denovo	16466561	(G/C)
21763	CakSNP21763	*Kabuli*	Ca_Kabuli_denovo	16478129	(C/T)
21764	CakSNP21764	*Kabuli*	Ca_Kabuli_denovo	16480207	(G/A)
21765	CakSNP21765	*Kabuli*	Ca_Kabuli_denovo	16481213	(T/C)
21766	CakSNP21766	*Kabuli*	Ca_Kabuli_denovo	16481216	(C/G)
21767	CakSNP21767	*Kabuli*	Ca_Kabuli_denovo	16488089	(C/G)
21768	CakSNP21768	*Kabuli*	Ca_Kabuli_denovo	16491339	(G/A)
21769	CakSNP21769	*Kabuli*	Ca_Kabuli_denovo	16493110	(C/G)
21770	CakSNP21770	*Kabuli*	Ca_Kabuli_denovo	16493176	(T/C)
21771	CakSNP21771	*Kabuli*	Ca_Kabuli_denovo	16493859	(C/T)
21772	CakSNP21772	*Kabuli*	Ca_Kabuli_denovo	16493864	(G/A)
21773	CakSNP21773	*Kabuli*	Ca_Kabuli_denovo	16495795	(T/G)

S.N.	SNP IDs	Cultivars	Chromosomes/scaffolds	Physical positions (bp)	SNPs
21774	CakSNP21774	*Kabuli*	Ca_Kabuli_denovo	16495801	(A/G)
21775	CakSNP21775	*Kabuli*	Ca_Kabuli_denovo	16499520	(A/G)
21776	CakSNP21776	*Kabuli*	Ca_Kabuli_denovo	16499555	(C/A)
21777	CakSNP21777	*Kabuli*	Ca_Kabuli_denovo	16500677	(A/G)
21778	CakSNP21778	*Kabuli*	Ca_Kabuli_denovo	16500683	(A/G)
21779	CakSNP21779	*Kabuli*	Ca_Kabuli_denovo	16501449	(T/G)
21780	CakSNP21780	*Kabuli*	Ca_Kabuli_denovo	16503695	(G/C)
21781	CakSNP21781	*Kabuli*	Ca_Kabuli_denovo	16506410	(C/G)
21782	CakSNP21782	*Kabuli*	Ca_Kabuli_denovo	16506452	(C/T)
21783	CakSNP21783	*Kabuli*	Ca_Kabuli_denovo	16506584	(C/G)
21784	CakSNP21784	*Kabuli*	Ca_Kabuli_denovo	16506595	(A/C)
21785	CakSNP21785	*Kabuli*	Ca_Kabuli_denovo	16506801	(C/T)
21786	CakSNP21786	*Kabuli*	Ca_Kabuli_denovo	16506848	(T/G)
21787	CakSNP21787	*Kabuli*	Ca_Kabuli_denovo	16507564	(T/G)
21788	CakSNP21788	*Kabuli*	Ca_Kabuli_denovo	16507589	(T/G)
21789	CakSNP21789	*Kabuli*	Ca_Kabuli_denovo	16511992	(G/A)
21790	CakSNP21790	*Kabuli*	Ca_Kabuli_denovo	16516079	(T/C)
21791	CakSNP21791	*Kabuli*	Ca_Kabuli_denovo	16518646	(A/G)
21792	CakSNP21792	*Kabuli*	Ca_Kabuli_denovo	16521054	(G/C)
21793	CakSNP21793	*Kabuli*	Ca_Kabuli_denovo	16523681	(A/G)
21794	CakSNP21794	*Kabuli*	Ca_Kabuli_denovo	16527540	(A/G)
21795	CakSNP21795	*Kabuli*	Ca_Kabuli_denovo	16527703	(T/C)
21796	CakSNP21796	*Kabuli*	Ca_Kabuli_denovo	16527716	(G/A)
21797	CakSNP21797	*Kabuli*	Ca_Kabuli_denovo	16531688	(G/C)
21798	CakSNP21798	*Kabuli*	Ca_Kabuli_denovo	16531733	(G/C)
21799	CakSNP21799	*Kabuli*	Ca_Kabuli_denovo	16535101	(C/A)
21800	CakSNP21800	*Kabuli*	Ca_Kabuli_denovo	16537066	(C/A)
21801	CakSNP21801	*Kabuli*	Ca_Kabuli_denovo	16538701	(C/T)
21802	CakSNP21802	*Kabuli*	Ca_Kabuli_denovo	16538725	(G/T)
21803	CakSNP21803	*Kabuli*	Ca_Kabuli_denovo	16540897	(G/T)
21804	CakSNP21804	*Kabuli*	Ca_Kabuli_denovo	16540932	(T/C)
21805	CakSNP21805	*Kabuli*	Ca_Kabuli_denovo	16541788	(T/C)
21806	CakSNP21806	*Kabuli*	Ca_Kabuli_denovo	16541815	(C/G)
21807	CakSNP21807	*Kabuli*	Ca_Kabuli_denovo	16541818	(C/T)
21808	CakSNP21808	*Kabuli*	Ca_Kabuli_denovo	16542667	(T/C)
21809	CakSNP21809	*Kabuli*	Ca_Kabuli_denovo	16543444	(T/C)
21810	CakSNP21810	*Kabuli*	Ca_Kabuli_denovo	16548536	(G/A)
21811	CakSNP21811	*Kabuli*	Ca_Kabuli_denovo	16548706	(T/G)

S.N.	SNP IDs	Cultivars	Chromosomes/scaffolds	Physical positions (bp)	SNPs
21812	CakSNP21812	*Kabuli*	Ca_Kabuli_denovo	16548709	(C/T)
21813	CakSNP21813	*Kabuli*	Ca_Kabuli_denovo	16550594	(A/G)
21814	CakSNP21814	*Kabuli*	Ca_Kabuli_denovo	16550597	(G/C)
21815	CakSNP21815	*Kabuli*	Ca_Kabuli_denovo	16550753	(T/G)
21816	CakSNP21816	*Kabuli*	Ca_Kabuli_denovo	16551596	(T/C)
21817	CakSNP21817	*Kabuli*	Ca_Kabuli_denovo	16551608	(C/T)
21818	CakSNP21818	*Kabuli*	Ca_Kabuli_denovo	16551984	(C/G)
21819	CakSNP21819	*Kabuli*	Ca_Kabuli_denovo	16552001	(C/G)
21820	CakSNP21820	*Kabuli*	Ca_Kabuli_denovo	16555320	(A/G)
21821	CakSNP21821	*Kabuli*	Ca_Kabuli_denovo	16555331	(A/G)
21822	CakSNP21822	*Kabuli*	Ca_Kabuli_denovo	16555347	(C/T)
21823	CakSNP21823	*Kabuli*	Ca_Kabuli_denovo	16563232	(C/G)
21824	CakSNP21824	*Kabuli*	Ca_Kabuli_denovo	16568993	(G/T)
21825	CakSNP21825	*Kabuli*	Ca_Kabuli_denovo	16568994	(T/C)
21826	CakSNP21826	*Kabuli*	Ca_Kabuli_denovo	16573820	(G/C)
21827	CakSNP21827	*Kabuli*	Ca_Kabuli_denovo	16573891	(C/T)
21828	CakSNP21828	*Kabuli*	Ca_Kabuli_denovo	16573900	(C/G)
21829	CakSNP21829	*Kabuli*	Ca_Kabuli_denovo	16573903	(C/T)
21830	CakSNP21830	*Kabuli*	Ca_Kabuli_denovo	16575551	(A/G)
21831	CakSNP21831	*Kabuli*	Ca_Kabuli_denovo	16577951	(G/A)
21832	CakSNP21832	*Kabuli*	Ca_Kabuli_denovo	16577959	(G/T)
21833	CakSNP21833	*Kabuli*	Ca_Kabuli_denovo	16578568	(C/T)
21834	CakSNP21834	*Kabuli*	Ca_Kabuli_denovo	16585761	(C/T)
21835	CakSNP21835	*Kabuli*	Ca_Kabuli_denovo	16589452	(G/A)
21836	CakSNP21836	*Kabuli*	Ca_Kabuli_denovo	16590210	(G/C)
21837	CakSNP21837	*Kabuli*	Ca_Kabuli_denovo	16590229	(C/G)
21838	CakSNP21838	*Kabuli*	Ca_Kabuli_denovo	16611748	(C/T)
21839	CakSNP21839	*Kabuli*	Ca_Kabuli_denovo	16613548	(A/G)
21840	CakSNP21840	*Kabuli*	Ca_Kabuli_denovo	16616090	(G/A)
21841	CakSNP21841	*Kabuli*	Ca_Kabuli_denovo	16616105	(A/G)
21842	CakSNP21842	*Kabuli*	Ca_Kabuli_denovo	16616806	(C/A)
21843	CakSNP21843	*Kabuli*	Ca_Kabuli_denovo	16619801	(C/A)
21844	CakSNP21844	*Kabuli*	Ca_Kabuli_denovo	16623076	(A/G)
21845	CakSNP21845	*Kabuli*	Ca_Kabuli_denovo	16626028	(C/A)
21846	CakSNP21846	*Kabuli*	Ca_Kabuli_denovo	16626043	(A/G)
21847	CakSNP21847	*Kabuli*	Ca_Kabuli_denovo	16629782	(C/T)
21848	CakSNP21848	*Kabuli*	Ca_Kabuli_denovo	16643323	(A/G)
21849	CakSNP21849	*Kabuli*	Ca_Kabuli_denovo	16648155	(A/G)

S.N.	SNP IDs	Cultivars	Chromosomes/scaffolds	Physical positions (bp)	SNPs
21850	CakSNP21850	*Kabuli*	Ca_Kabuli_denovo	16653157	(T/C)
21851	CakSNP21851	*Kabuli*	Ca_Kabuli_denovo	16665169	(T/C)
21852	CakSNP21852	*Kabuli*	Ca_Kabuli_denovo	16669271	(T/G)
21853	CakSNP21853	*Kabuli*	Ca_Kabuli_denovo	16669353	(G/C)
21854	CakSNP21854	*Kabuli*	Ca_Kabuli_denovo	16669354	(G/C)
21855	CakSNP21855	*Kabuli*	Ca_Kabuli_denovo	16672658	(C/G)
21856	CakSNP21856	*Kabuli*	Ca_Kabuli_denovo	16673523	(C/T)
21857	CakSNP21857	*Kabuli*	Ca_Kabuli_denovo	16673532	(T/A)
21858	CakSNP21858	*Kabuli*	Ca_Kabuli_denovo	16673632	(C/A)
21859	CakSNP21859	*Kabuli*	Ca_Kabuli_denovo	16678096	(A/G)
21860	CakSNP21860	*Kabuli*	Ca_Kabuli_denovo	16678143	(G/T)
21861	CakSNP21861	*Kabuli*	Ca_Kabuli_denovo	16691722	(T/A)
21862	CakSNP21862	*Kabuli*	Ca_Kabuli_denovo	16700868	(G/C)
21863	CakSNP21863	*Kabuli*	Ca_Kabuli_denovo	16706187	(G/C)
21864	CakSNP21864	*Kabuli*	Ca_Kabuli_denovo	16706241	(G/A)
21865	CakSNP21865	*Kabuli*	Ca_Kabuli_denovo	16707329	(G/A)
21866	CakSNP21866	*Kabuli*	Ca_Kabuli_denovo	16707356	(A/C)
21867	CakSNP21867	*Kabuli*	Ca_Kabuli_denovo	16708337	(A/G)
21868	CakSNP21868	*Kabuli*	Ca_Kabuli_denovo	16715210	(A/G)
21869	CakSNP21869	*Kabuli*	Ca_Kabuli_denovo	16715216	(A/C)
21870	CakSNP21870	*Kabuli*	Ca_Kabuli_denovo	16720077	(T/G)
21871	CakSNP21871	*Kabuli*	Ca_Kabuli_denovo	16720096	(C/A)
21872	CakSNP21872	*Kabuli*	Ca_Kabuli_denovo	16723012	(G/A)
21873	CakSNP21873	*Kabuli*	Ca_Kabuli_denovo	16724754	(A/G)
21874	CakSNP21874	*Kabuli*	Ca_Kabuli_denovo	16724832	(T/C)
21875	CakSNP21875	*Kabuli*	Ca_Kabuli_denovo	16725436	(T/C)
21876	CakSNP21876	*Kabuli*	Ca_Kabuli_denovo	16728254	(A/C)
21877	CakSNP21877	*Kabuli*	Ca_Kabuli_denovo	16734735	(A/G)
21878	CakSNP21878	*Kabuli*	Ca_Kabuli_denovo	16738628	(C/T)
21879	CakSNP21879	*Kabuli*	Ca_Kabuli_denovo	16741741	(T/C)
21880	CakSNP21880	*Kabuli*	Ca_Kabuli_denovo	16745312	(T/G)
21881	CakSNP21881	*Kabuli*	Ca_Kabuli_denovo	16745357	(C/T)
21882	CakSNP21882	*Kabuli*	Ca_Kabuli_denovo	16749543	(C/T)
21883	CakSNP21883	*Kabuli*	Ca_Kabuli_denovo	16753524	(T/C)
21884	CakSNP21884	*Kabuli*	Ca_Kabuli_denovo	16756754	(T/G)
21885	CakSNP21885	*Kabuli*	Ca_Kabuli_denovo	16756778	(C/G)
21886	CakSNP21886	*Kabuli*	Ca_Kabuli_denovo	16759066	(A/C)
21887	CakSNP21887	*Kabuli*	Ca_Kabuli_denovo	16763119	(G/C)

S.N.	SNP IDs	Cultivars	Chromosomes/scaffolds	Physical positions (bp)	SNPs
21888	CakSNP21888	*Kabuli*	Ca_Kabuli_denovo	16768916	(C/T)
21889	CakSNP21889	*Kabuli*	Ca_Kabuli_denovo	16770864	(C/T)
21890	CakSNP21890	*Kabuli*	Ca_Kabuli_denovo	16773167	(G/A)
21891	CakSNP21891	*Kabuli*	Ca_Kabuli_denovo	16773169	(T/C)
21892	CakSNP21892	*Kabuli*	Ca_Kabuli_denovo	16775909	(G/C)
21893	CakSNP21893	*Kabuli*	Ca_Kabuli_denovo	16792384	(G/T)
21894	CakSNP21894	*Kabuli*	Ca_Kabuli_denovo	16792385	(T/C)
21895	CakSNP21895	*Kabuli*	Ca_Kabuli_denovo	16795334	(A/C)
21896	CakSNP21896	*Kabuli*	Ca_Kabuli_denovo	16796009	(G/C)
21897	CakSNP21897	*Kabuli*	Ca_Kabuli_denovo	16797381	(A/C)
21898	CakSNP21898	*Kabuli*	Ca_Kabuli_denovo	16798070	(T/G)
21899	CakSNP21899	*Kabuli*	Ca_Kabuli_denovo	16800967	(G/C)
21900	CakSNP21900	*Kabuli*	Ca_Kabuli_denovo	16800982	(G/A)
21901	CakSNP21901	*Kabuli*	Ca_Kabuli_denovo	16805285	(A/C)
21902	CakSNP21902	*Kabuli*	Ca_Kabuli_denovo	16806851	(A/G)
21903	CakSNP21903	*Kabuli*	Ca_Kabuli_denovo	16810319	(C/G)
21904	CakSNP21904	*Kabuli*	Ca_Kabuli_denovo	16810328	(G/T)
21905	CakSNP21905	*Kabuli*	Ca_Kabuli_denovo	16811019	(A/G)
21906	CakSNP21906	*Kabuli*	Ca_Kabuli_denovo	16817728	(C/T)
21907	CakSNP21907	*Kabuli*	Ca_Kabuli_denovo	16818477	(G/T)
21908	CakSNP21908	*Kabuli*	Ca_Kabuli_denovo	16822925	(G/A)
21909	CakSNP21909	*Kabuli*	Ca_Kabuli_denovo	16839161	(T/C)
21910	CakSNP21910	*Kabuli*	Ca_Kabuli_denovo	16847104	(G/A)
21911	CakSNP21911	*Kabuli*	Ca_Kabuli_denovo	16847905	(T/G)
21912	CakSNP21912	*Kabuli*	Ca_Kabuli_denovo	16847915	(T/C)
21913	CakSNP21913	*Kabuli*	Ca_Kabuli_denovo	16848678	(A/G)
21914	CakSNP21914	*Kabuli*	Ca_Kabuli_denovo	16848818	(C/G)
21915	CakSNP21915	*Kabuli*	Ca_Kabuli_denovo	16849886	(C/A)
21916	CakSNP21916	*Kabuli*	Ca_Kabuli_denovo	16849916	(G/C)
21917	CakSNP21917	*Kabuli*	Ca_Kabuli_denovo	16854862	(C/T)
21918	CakSNP21918	*Kabuli*	Ca_Kabuli_denovo	16854880	(A/G)
21919	CakSNP21919	*Kabuli*	Ca_Kabuli_denovo	16856071	(T/C)
21920	CakSNP21920	*Kabuli*	Ca_Kabuli_denovo	16857724	(G/T)
21921	CakSNP21921	*Kabuli*	Ca_Kabuli_denovo	16866134	(C/T)
21922	CakSNP21922	*Kabuli*	Ca_Kabuli_denovo	16866191	(T/G)
21923	CakSNP21923	*Kabuli*	Ca_Kabuli_denovo	16867690	(G/A)
21924	CakSNP21924	*Kabuli*	Ca_Kabuli_denovo	16884460	(G/C)
21925	CakSNP21925	*Kabuli*	Ca_Kabuli_denovo	16890964	(T/C)

**S.N.**	**SNP IDs**	**Cultivars**	**Chromosomes/scaffolds**	**Physical positions (bp)**	**SNPs**
21926	CakSNP21926	*Kabuli*	Ca_Kabuli_denovo	16890974	(A/G)
21927	CakSNP21927	*Kabuli*	Ca_Kabuli_denovo	16900104	(G/A)
21928	CakSNP21928	*Kabuli*	Ca_Kabuli_denovo	16900127	(G/A)
21929	CakSNP21929	*Kabuli*	Ca_Kabuli_denovo	16901597	(T/G)
21930	CakSNP21930	*Kabuli*	Ca_Kabuli_denovo	16901600	(T/A)
21931	CakSNP21931	*Kabuli*	Ca_Kabuli_denovo	16901701	(C/T)
21932	CakSNP21932	*Kabuli*	Ca_Kabuli_denovo	16904740	(A/C)
21933	CakSNP21933	*Kabuli*	Ca_Kabuli_denovo	16909393	(C/G)
21934	CakSNP21934	*Kabuli*	Ca_Kabuli_denovo	16909447	(C/A)
21935	CakSNP21935	*Kabuli*	Ca_Kabuli_denovo	16913920	(G/T)
21936	CakSNP21936	*Kabuli*	Ca_Kabuli_denovo	16917202	(G/C)
21937	CakSNP21937	*Kabuli*	Ca_Kabuli_denovo	16917212	(G/A)
21938	CakSNP21938	*Kabuli*	Ca_Kabuli_denovo	16921376	(A/G)
21939	CakSNP21939	*Kabuli*	Ca_Kabuli_denovo	16929094	(C/T)
21940	CakSNP21940	*Kabuli*	Ca_Kabuli_denovo	16930382	(T/C)
21941	CakSNP21941	*Kabuli*	Ca_Kabuli_denovo	16936177	(A/G)
21942	CakSNP21942	*Kabuli*	Ca_Kabuli_denovo	16938854	(T/C)
21943	CakSNP21943	*Kabuli*	Ca_Kabuli_denovo	16938863	(A/G)
21944	CakSNP21944	*Kabuli*	Ca_Kabuli_denovo	16947610	(G/A)
21945	CakSNP21945	*Kabuli*	Ca_Kabuli_denovo	16947664	(T/C)
21946	CakSNP21946	*Kabuli*	Ca_Kabuli_denovo	16947792	(G/A)
21947	CakSNP21947	*Kabuli*	Ca_Kabuli_denovo	16950010	(T/C)
21948	CakSNP21948	*Kabuli*	Ca_Kabuli_denovo	16959044	(C/G)
21949	CakSNP21949	*Kabuli*	Ca_Kabuli_denovo	16959070	(C/G)
21950	CakSNP21950	*Kabuli*	Ca_Kabuli_denovo	16959077	(G/A)
21951	CakSNP21951	*Kabuli*	Ca_Kabuli_denovo	16972762	(T/C)
21952	CakSNP21952	*Kabuli*	Ca_Kabuli_denovo	16973548	(C/T)
21953	CakSNP21953	*Kabuli*	Ca_Kabuli_denovo	16973590	(C/G)
21954	CakSNP21954	*Kabuli*	Ca_Kabuli_denovo	16984599	(C/G)
21955	CakSNP21955	*Kabuli*	Ca_Kabuli_denovo	16984785	(C/G)
21956	CakSNP21956	*Kabuli*	Ca_Kabuli_denovo	16984812	(T/C)
21957	CakSNP21957	*Kabuli*	Ca_Kabuli_denovo	17004468	(G/A)
21958	CakSNP21958	*Kabuli*	Ca_Kabuli_denovo	17004504	(C/T)
21959	CakSNP21959	*Kabuli*	Ca_Kabuli_denovo	17004540	(C/T)
21960	CakSNP21960	*Kabuli*	Ca_Kabuli_denovo	17005753	(G/A)
21961	CakSNP21961	*Kabuli*	Ca_Kabuli_denovo	17005756	(A/G)
21962	CakSNP21962	*Kabuli*	Ca_Kabuli_denovo	17016529	(C/T)
21963	CakSNP21963	*Kabuli*	Ca_Kabuli_denovo	17016536	(G/T)

S.N.	SNP IDs	Cultivars	Chromosomes/scaffolds	Physical positions (bp)	SNPs
21964	CakSNP21964	*Kabuli*	Ca_Kabuli_denovo	17016995	(A/T)
21965	CakSNP21965	*Kabuli*	Ca_Kabuli_denovo	17021436	(T/G)
21966	CakSNP21966	*Kabuli*	Ca_Kabuli_denovo	17021442	(G/T)
21967	CakSNP21967	*Kabuli*	Ca_Kabuli_denovo	17024552	(C/A)
21968	CakSNP21968	*Kabuli*	Ca_Kabuli_denovo	17034564	(T/C)
21969	CakSNP21969	*Kabuli*	Ca_Kabuli_denovo	17034585	(G/A)
21970	CakSNP21970	*Kabuli*	Ca_Kabuli_denovo	17035472	(G/A)
21971	CakSNP21971	*Kabuli*	Ca_Kabuli_denovo	17039549	(C/G)
21972	CakSNP21972	*Kabuli*	Ca_Kabuli_denovo	17039551	(T/G)
21973	CakSNP21973	*Kabuli*	Ca_Kabuli_denovo	17042770	(C/G)
21974	CakSNP21974	*Kabuli*	Ca_Kabuli_denovo	17043941	(G/A)
21975	CakSNP21975	*Kabuli*	Ca_Kabuli_denovo	17045500	(T/G)
21976	CakSNP21976	*Kabuli*	Ca_Kabuli_denovo	17050364	(G/A)
21977	CakSNP21977	*Kabuli*	Ca_Kabuli_denovo	17052384	(T/G)
21978	CakSNP21978	*Kabuli*	Ca_Kabuli_denovo	17056471	(C/T)
21979	CakSNP21979	*Kabuli*	Ca_Kabuli_denovo	17056477	(C/G)
21980	CakSNP21980	*Kabuli*	Ca_Kabuli_denovo	17057732	(C/T)
21981	CakSNP21981	*Kabuli*	Ca_Kabuli_denovo	17057823	(T/C)
21982	CakSNP21982	*Kabuli*	Ca_Kabuli_denovo	17057850	(G/A)
21983	CakSNP21983	*Kabuli*	Ca_Kabuli_denovo	17069033	(A/C)
21984	CakSNP21984	*Kabuli*	Ca_Kabuli_denovo	17069653	(C/T)
21985	CakSNP21985	*Kabuli*	Ca_Kabuli_denovo	17069674	(T/C)
21986	CakSNP21986	*Kabuli*	Ca_Kabuli_denovo	17072513	(T/C)
21987	CakSNP21987	*Kabuli*	Ca_Kabuli_denovo	17072515	(G/C)
21988	CakSNP21988	*Kabuli*	Ca_Kabuli_denovo	17072813	(T/C)
21989	CakSNP21989	*Kabuli*	Ca_Kabuli_denovo	17074228	(A/G)
21990	CakSNP21990	*Kabuli*	Ca_Kabuli_denovo	17074250	(T/A)
21991	CakSNP21991	*Kabuli*	Ca_Kabuli_denovo	17074307	(A/T)
21992	CakSNP21992	*Kabuli*	Ca_Kabuli_denovo	17077410	(A/G)
21993	CakSNP21993	*Kabuli*	Ca_Kabuli_denovo	17082462	(T/C)
21994	CakSNP21994	*Kabuli*	Ca_Kabuli_denovo	17082822	(A/G)
21995	CakSNP21995	*Kabuli*	Ca_Kabuli_denovo	17083441	(G/T)
21996	CakSNP21996	*Kabuli*	Ca_Kabuli_denovo	17085224	(T/C)
21997	CakSNP21997	*Kabuli*	Ca_Kabuli_denovo	17094726	(G/A)
21998	CakSNP21998	*Kabuli*	Ca_Kabuli_denovo	17095757	(A/G)
21999	CakSNP21999	*Kabuli*	Ca_Kabuli_denovo	17100012	(C/G)
22000	CakSNP22000	*Kabuli*	Ca_Kabuli_denovo	17100020	(A/C)
22001	CakSNP22001	*Kabuli*	Ca_Kabuli_denovo	17100026	(C/G)

S.N.	SNP IDs	Cultivars	Chromosomes/scaffolds	Physical positions (bp)	SNPs
22002	CakSNP22002	*Kabuli*	Ca_Kabuli_denovo	17100254	(A/T)
22003	CakSNP22003	*Kabuli*	Ca_Kabuli_denovo	17100270	(C/G)
22004	CakSNP22004	*Kabuli*	Ca_Kabuli_denovo	17100331	(T/C)
22005	CakSNP22005	*Kabuli*	Ca_Kabuli_denovo	17105131	(T/G)
22006	CakSNP22006	*Kabuli*	Ca_Kabuli_denovo	17124123	(G/C)
22007	CakSNP22007	*Kabuli*	Ca_Kabuli_denovo	17124179	(G/A)
22008	CakSNP22008	*Kabuli*	Ca_Kabuli_denovo	17135908	(C/G)
22009	CakSNP22009	*Kabuli*	Ca_Kabuli_denovo	17135911	(T/C)
22010	CakSNP22010	*Kabuli*	Ca_Kabuli_denovo	17137643	(A/C)
22011	CakSNP22011	*Kabuli*	Ca_Kabuli_denovo	17145502	(C/T)
22012	CakSNP22012	*Kabuli*	Ca_Kabuli_denovo	17149807	(C/G)
22013	CakSNP22013	*Kabuli*	Ca_Kabuli_denovo	17153306	(G/A)
22014	CakSNP22014	*Kabuli*	Ca_Kabuli_denovo	17153538	(A/G)
22015	CakSNP22015	*Kabuli*	Ca_Kabuli_denovo	17153539	(T/C)
22016	CakSNP22016	*Kabuli*	Ca_Kabuli_denovo	17158844	(T/G)
22017	CakSNP22017	*Kabuli*	Ca_Kabuli_denovo	17158845	(C/A)
22018	CakSNP22018	*Kabuli*	Ca_Kabuli_denovo	17164315	(G/T)
22019	CakSNP22019	*Kabuli*	Ca_Kabuli_denovo	17169559	(C/T)
22020	CakSNP22020	*Kabuli*	Ca_Kabuli_denovo	17173889	(C/T)
22021	CakSNP22021	*Kabuli*	Ca_Kabuli_denovo	17175017	(C/G)
22022	CakSNP22022	*Kabuli*	Ca_Kabuli_denovo	17175032	(C/T)
22023	CakSNP22023	*Kabuli*	Ca_Kabuli_denovo	17183208	(C/T)
22024	CakSNP22024	*Kabuli*	Ca_Kabuli_denovo	17183478	(C/G)
22025	CakSNP22025	*Kabuli*	Ca_Kabuli_denovo	17183481	(T/C)
22026	CakSNP22026	*Kabuli*	Ca_Kabuli_denovo	17191143	(A/C)
22027	CakSNP22027	*Kabuli*	Ca_Kabuli_denovo	17191144	(G/A)
22028	CakSNP22028	*Kabuli*	Ca_Kabuli_denovo	17193826	(G/A)
22029	CakSNP22029	*Kabuli*	Ca_Kabuli_denovo	17194069	(G/T)
22030	CakSNP22030	*Kabuli*	Ca_Kabuli_denovo	17197111	(T/G)
22031	CakSNP22031	*Kabuli*	Ca_Kabuli_denovo	17197112	(C/T)
22032	CakSNP22032	*Kabuli*	Ca_Kabuli_denovo	17211956	(G/A)
22033	CakSNP22033	*Kabuli*	Ca_Kabuli_denovo	17221166	(T/C)
22034	CakSNP22034	*Kabuli*	Ca_Kabuli_denovo	17222126	(G/T)
22035	CakSNP22035	*Kabuli*	Ca_Kabuli_denovo	17228590	(A/G)
22036	CakSNP22036	*Kabuli*	Ca_Kabuli_denovo	17228617	(C/G)
22037	CakSNP22037	*Kabuli*	Ca_Kabuli_denovo	17229757	(T/C)
22038	CakSNP22038	*Kabuli*	Ca_Kabuli_denovo	17229851	(A/G)
22039	CakSNP22039	*Kabuli*	Ca_Kabuli_denovo	17229856	(T/C)

S.N.	SNP IDs	Cultivars	Chromosomes/scaffolds	Physical positions (bp)	SNPs
22040	CakSNP22040	*Kabuli*	Ca_Kabuli_denovo	17230023	(C/T)
22041	CakSNP22041	*Kabuli*	Ca_Kabuli_denovo	17232772	(G/A)
22042	CakSNP22042	*Kabuli*	Ca_Kabuli_denovo	17234123	(C/T)
22043	CakSNP22043	*Kabuli*	Ca_Kabuli_denovo	17234152	(T/G)
22044	CakSNP22044	*Kabuli*	Ca_Kabuli_denovo	17234682	(G/A)
22045	CakSNP22045	*Kabuli*	Ca_Kabuli_denovo	17234713	(C/A)
22046	CakSNP22046	*Kabuli*	Ca_Kabuli_denovo	17236651	(C/T)
22047	CakSNP22047	*Kabuli*	Ca_Kabuli_denovo	17236681	(T/C)
22048	CakSNP22048	*Kabuli*	Ca_Kabuli_denovo	17237146	(A/G)
22049	CakSNP22049	*Kabuli*	Ca_Kabuli_denovo	17259898	(C/G)
22050	CakSNP22050	*Kabuli*	Ca_Kabuli_denovo	17263412	(T/G)
22051	CakSNP22051	*Kabuli*	Ca_Kabuli_denovo	17264715	(G/C)
22052	CakSNP22052	*Kabuli*	Ca_Kabuli_denovo	17264725	(T/C)
22053	CakSNP22053	*Kabuli*	Ca_Kabuli_denovo	17264921	(G/A)
22054	CakSNP22054	*Kabuli*	Ca_Kabuli_denovo	17265470	(A/G)
22055	CakSNP22055	*Kabuli*	Ca_Kabuli_denovo	17270108	(G/A)
22056	CakSNP22056	*Kabuli*	Ca_Kabuli_denovo	17275057	(G/A)
22057	CakSNP22057	*Kabuli*	Ca_Kabuli_denovo	17275063	(A/G)
22058	CakSNP22058	*Kabuli*	Ca_Kabuli_denovo	17284324	(A/G)
22059	CakSNP22059	*Kabuli*	Ca_Kabuli_denovo	17286294	(C/T)
22060	CakSNP22060	*Kabuli*	Ca_Kabuli_denovo	17286314	(A/G)
22061	CakSNP22061	*Kabuli*	Ca_Kabuli_denovo	17288522	(C/T)
22062	CakSNP22062	*Kabuli*	Ca_Kabuli_denovo	17288523	(T/C)
22063	CakSNP22063	*Kabuli*	Ca_Kabuli_denovo	17289626	(T/G)
22064	CakSNP22064	*Kabuli*	Ca_Kabuli_denovo	17290132	(C/T)
22065	CakSNP22065	*Kabuli*	Ca_Kabuli_denovo	17291686	(A/C)
22066	CakSNP22066	*Kabuli*	Ca_Kabuli_denovo	17297188	(C/T)
22067	CakSNP22067	*Kabuli*	Ca_Kabuli_denovo	17297200	(G/A)
22068	CakSNP22068	*Kabuli*	Ca_Kabuli_denovo	17309055	(A/G)
22069	CakSNP22069	*Kabuli*	Ca_Kabuli_denovo	17310208	(T/C)
22070	CakSNP22070	*Kabuli*	Ca_Kabuli_denovo	17313517	(C/A)
22071	CakSNP22071	*Kabuli*	Ca_Kabuli_denovo	17313528	(A/C)
22072	CakSNP22072	*Kabuli*	Ca_Kabuli_denovo	17313529	(G/C)
22073	CakSNP22073	*Kabuli*	Ca_Kabuli_denovo	17317567	(C/T)
22074	CakSNP22074	*Kabuli*	Ca_Kabuli_denovo	17318919	(T/G)
22075	CakSNP22075	*Kabuli*	Ca_Kabuli_denovo	17319872	(C/T)
22076	CakSNP22076	*Kabuli*	Ca_Kabuli_denovo	17321416	(C/A)
22077	CakSNP22077	*Kabuli*	Ca_Kabuli_denovo	17330189	(T/C)

S.N.	SNP IDs	Cultivars	Chromosomes/scaffolds	Physical positions (bp)	SNPs
22078	CakSNP22078	*Kabuli*	Ca_Kabuli_denovo	17330572	(T/G)
22079	CakSNP22079	*Kabuli*	Ca_Kabuli_denovo	17335969	(T/C)
22080	CakSNP22080	*Kabuli*	Ca_Kabuli_denovo	17335978	(C/T)
22081	CakSNP22081	*Kabuli*	Ca_Kabuli_denovo	17336333	(C/T)
22082	CakSNP22082	*Kabuli*	Ca_Kabuli_denovo	17336409	(T/C)
22083	CakSNP22083	*Kabuli*	Ca_Kabuli_denovo	17339624	(A/G)
22084	CakSNP22084	*Kabuli*	Ca_Kabuli_denovo	17347047	(G/T)
22085	CakSNP22085	*Kabuli*	Ca_Kabuli_denovo	17347065	(G/C)
22086	CakSNP22086	*Kabuli*	Ca_Kabuli_denovo	17349179	(C/A)
22087	CakSNP22087	*Kabuli*	Ca_Kabuli_denovo	17350379	(C/T)
22088	CakSNP22088	*Kabuli*	Ca_Kabuli_denovo	17350403	(G/C)
22089	CakSNP22089	*Kabuli*	Ca_Kabuli_denovo	17355331	(C/T)
22090	CakSNP22090	*Kabuli*	Ca_Kabuli_denovo	17355343	(T/G)
22091	CakSNP22091	*Kabuli*	Ca_Kabuli_denovo	17363326	(A/G)
22092	CakSNP22092	*Kabuli*	Ca_Kabuli_denovo	17363362	(C/G)
22093	CakSNP22093	*Kabuli*	Ca_Kabuli_denovo	17363588	(G/C)
22094	CakSNP22094	*Kabuli*	Ca_Kabuli_denovo	17363607	(G/A)
22095	CakSNP22095	*Kabuli*	Ca_Kabuli_denovo	17365595	(A/G)
22096	CakSNP22096	*Kabuli*	Ca_Kabuli_denovo	17367130	(T/G)
22097	CakSNP22097	*Kabuli*	Ca_Kabuli_denovo	17372448	(G/A)
22098	CakSNP22098	*Kabuli*	Ca_Kabuli_denovo	17372474	(G/T)
22099	CakSNP22099	*Kabuli*	Ca_Kabuli_denovo	17374148	(T/C)
22100	CakSNP22100	*Kabuli*	Ca_Kabuli_denovo	17374150	(G/A)
22101	CakSNP22101	*Kabuli*	Ca_Kabuli_denovo	17374982	(C/G)
22102	CakSNP22102	*Kabuli*	Ca_Kabuli_denovo	17383714	(G/C)
22103	CakSNP22103	*Kabuli*	Ca_Kabuli_denovo	17390158	(C/T)
22104	CakSNP22104	*Kabuli*	Ca_Kabuli_denovo	17391946	(G/A)
22105	CakSNP22105	*Kabuli*	Ca_Kabuli_denovo	17392021	(A/G)
22106	CakSNP22106	*Kabuli*	Ca_Kabuli_denovo	17396458	(G/A)
22107	CakSNP22107	*Kabuli*	Ca_Kabuli_denovo	17400281	(G/C)
22108	CakSNP22108	*Kabuli*	Ca_Kabuli_denovo	17418177	(G/A)
22109	CakSNP22109	*Kabuli*	Ca_Kabuli_denovo	17418195	(G/A)
22110	CakSNP22110	*Kabuli*	Ca_Kabuli_denovo	17423923	(A/C)
22111	CakSNP22111	*Kabuli*	Ca_Kabuli_denovo	17425180	(T/A)
22112	CakSNP22112	*Kabuli*	Ca_Kabuli_denovo	17425534	(G/C)
22113	CakSNP22113	*Kabuli*	Ca_Kabuli_denovo	17426379	(C/T)
22114	CakSNP22114	*Kabuli*	Ca_Kabuli_denovo	17427763	(A/G)
22115	CakSNP22115	*Kabuli*	Ca_Kabuli_denovo	17427807	(G/A)

S.N.	SNP IDs	Cultivars	Chromosomes/scaffolds	Physical positions (bp)	SNPs
22116	CakSNP22116	*Kabuli*	Ca_Kabuli_denovo	17429423	(A/G)
22117	CakSNP22117	*Kabuli*	Ca_Kabuli_denovo	17429444	(G/A)
22118	CakSNP22118	*Kabuli*	Ca_Kabuli_denovo	17432893	(G/A)
22119	CakSNP22119	*Kabuli*	Ca_Kabuli_denovo	17438905	(A/G)
22120	CakSNP22120	*Kabuli*	Ca_Kabuli_denovo	17454408	(G/A)
22121	CakSNP22121	*Kabuli*	Ca_Kabuli_denovo	17454527	(C/G)
22122	CakSNP22122	*Kabuli*	Ca_Kabuli_denovo	17454584	(G/A)
22123	CakSNP22123	*Kabuli*	Ca_Kabuli_denovo	17458338	(C/G)
22124	CakSNP22124	*Kabuli*	Ca_Kabuli_denovo	17458378	(A/C)
22125	CakSNP22125	*Kabuli*	Ca_Kabuli_denovo	17460854	(C/A)
22126	CakSNP22126	*Kabuli*	Ca_Kabuli_denovo	17462015	(T/C)
22127	CakSNP22127	*Kabuli*	Ca_Kabuli_denovo	17462108	(G/T)
22128	CakSNP22128	*Kabuli*	Ca_Kabuli_denovo	17463999	(T/G)
22129	CakSNP22129	*Kabuli*	Ca_Kabuli_denovo	17464342	(A/G)
22130	CakSNP22130	*Kabuli*	Ca_Kabuli_denovo	17465423	(C/T)
22131	CakSNP22131	*Kabuli*	Ca_Kabuli_denovo	17465429	(A/G)
22132	CakSNP22132	*Kabuli*	Ca_Kabuli_denovo	17467389	(A/G)
22133	CakSNP22133	*Kabuli*	Ca_Kabuli_denovo	17468575	(A/T)
22134	CakSNP22134	*Kabuli*	Ca_Kabuli_denovo	17472963	(C/A)
22135	CakSNP22135	*Kabuli*	Ca_Kabuli_denovo	17475553	(A/G)
22136	CakSNP22136	*Kabuli*	Ca_Kabuli_denovo	17475769	(A/C)
22137	CakSNP22137	*Kabuli*	Ca_Kabuli_denovo	17484863	(A/G)
22138	CakSNP22138	*Kabuli*	Ca_Kabuli_denovo	17484890	(C/A)
22139	CakSNP22139	*Kabuli*	Ca_Kabuli_denovo	17490392	(G/A)
22140	CakSNP22140	*Kabuli*	Ca_Kabuli_denovo	17491797	(C/T)
22141	CakSNP22141	*Kabuli*	Ca_Kabuli_denovo	17494871	(A/T)
22142	CakSNP22142	*Kabuli*	Ca_Kabuli_denovo	17495308	(T/C)
22143	CakSNP22143	*Kabuli*	Ca_Kabuli_denovo	17495506	(A/T)
22144	CakSNP22144	*Kabuli*	Ca_Kabuli_denovo	17495658	(A/G)
22145	CakSNP22145	*Kabuli*	Ca_Kabuli_denovo	17495911	(C/A)
22146	CakSNP22146	*Kabuli*	Ca_Kabuli_denovo	17495980	(C/G)
22147	CakSNP22147	*Kabuli*	Ca_Kabuli_denovo	17496047	(A/G)
22148	CakSNP22148	*Kabuli*	Ca_Kabuli_denovo	17496086	(C/T)
22149	CakSNP22149	*Kabuli*	Ca_Kabuli_denovo	17498540	(G/C)
22150	CakSNP22150	*Kabuli*	Ca_Kabuli_denovo	17502327	(C/G)
22151	CakSNP22151	*Kabuli*	Ca_Kabuli_denovo	17502354	(C/T)
22152	CakSNP22152	*Kabuli*	Ca_Kabuli_denovo	17509865	(C/G)
22153	CakSNP22153	*Kabuli*	Ca_Kabuli_denovo	17509874	(G/T)

S.N.	SNP IDs	Cultivars	Chromosomes/scaffolds	Physical positions (bp)	SNPs
22154	CakSNP22154	*Kabuli*	Ca_Kabuli_denovo	17512372	(G/C)
22155	CakSNP22155	*Kabuli*	Ca_Kabuli_denovo	17512443	(A/G)
22156	CakSNP22156	*Kabuli*	Ca_Kabuli_denovo	17514272	(T/G)
22157	CakSNP22157	*Kabuli*	Ca_Kabuli_denovo	17514319	(A/C)
22158	CakSNP22158	*Kabuli*	Ca_Kabuli_denovo	17515725	(T/C)
22159	CakSNP22159	*Kabuli*	Ca_Kabuli_denovo	17515728	(A/G)
22160	CakSNP22160	*Kabuli*	Ca_Kabuli_denovo	17516278	(A/G)
22161	CakSNP22161	*Kabuli*	Ca_Kabuli_denovo	17517744	(A/G)
22162	CakSNP22162	*Kabuli*	Ca_Kabuli_denovo	17519081	(A/G)
22163	CakSNP22163	*Kabuli*	Ca_Kabuli_denovo	17519153	(C/G)
22164	CakSNP22164	*Kabuli*	Ca_Kabuli_denovo	17522335	(A/G)
22165	CakSNP22165	*Kabuli*	Ca_Kabuli_denovo	17522341	(C/G)
22166	CakSNP22166	*Kabuli*	Ca_Kabuli_denovo	17543443	(A/G)
22167	CakSNP22167	*Kabuli*	Ca_Kabuli_denovo	17554118	(A/G)
22168	CakSNP22168	*Kabuli*	Ca_Kabuli_denovo	17554879	(G/A)
22169	CakSNP22169	*Kabuli*	Ca_Kabuli_denovo	17555105	(G/A)
22170	CakSNP22170	*Kabuli*	Ca_Kabuli_denovo	17555680	(C/T)
22171	CakSNP22171	*Kabuli*	Ca_Kabuli_denovo	17563276	(A/C)
22172	CakSNP22172	*Kabuli*	Ca_Kabuli_denovo	17563281	(G/A)
22173	CakSNP22173	*Kabuli*	Ca_Kabuli_denovo	17568185	(A/G)
22174	CakSNP22174	*Kabuli*	Ca_Kabuli_denovo	17568195	(A/G)
22175	CakSNP22175	*Kabuli*	Ca_Kabuli_denovo	17568481	(A/C)
22176	CakSNP22176	*Kabuli*	Ca_Kabuli_denovo	17568536	(A/G)
22177	CakSNP22177	*Kabuli*	Ca_Kabuli_denovo	17569512	(C/G)
22178	CakSNP22178	*Kabuli*	Ca_Kabuli_denovo	17571190	(T/C)
22179	CakSNP22179	*Kabuli*	Ca_Kabuli_denovo	17575857	(G/C)
22180	CakSNP22180	*Kabuli*	Ca_Kabuli_denovo	17575861	(A/G)
22181	CakSNP22181	*Kabuli*	Ca_Kabuli_denovo	17575862	(A/G)
22182	CakSNP22182	*Kabuli*	Ca_Kabuli_denovo	17575990	(A/G)
22183	CakSNP22183	*Kabuli*	Ca_Kabuli_denovo	17576348	(T/C)
22184	CakSNP22184	*Kabuli*	Ca_Kabuli_denovo	17577311	(G/T)
22185	CakSNP22185	*Kabuli*	Ca_Kabuli_denovo	17577949	(T/G)
22186	CakSNP22186	*Kabuli*	Ca_Kabuli_denovo	17578019	(C/G)
22187	CakSNP22187	*Kabuli*	Ca_Kabuli_denovo	17587745	(A/G)
22188	CakSNP22188	*Kabuli*	Ca_Kabuli_denovo	17587759	(A/G)
22189	CakSNP22189	*Kabuli*	Ca_Kabuli_denovo	17588013	(C/A)
22190	CakSNP22190	*Kabuli*	Ca_Kabuli_denovo	17588037	(A/G)
22191	CakSNP22191	*Kabuli*	Ca_Kabuli_denovo	17588065	(C/T)

S.N.	SNP IDs	Cultivars	Chromosomes/scaffolds	Physical positions (bp)	SNPs
22192	CakSNP22192	*Kabuli*	Ca_Kabuli_denovo	17589665	(C/T)
22193	CakSNP22193	*Kabuli*	Ca_Kabuli_denovo	17589862	(C/G)
22194	CakSNP22194	*Kabuli*	Ca_Kabuli_denovo	17593933	(G/A)
22195	CakSNP22195	*Kabuli*	Ca_Kabuli_denovo	17593960	(A/G)
22196	CakSNP22196	*Kabuli*	Ca_Kabuli_denovo	17605730	(G/A)
22197	CakSNP22197	*Kabuli*	Ca_Kabuli_denovo	17609678	(G/C)
22198	CakSNP22198	*Kabuli*	Ca_Kabuli_denovo	17609709	(A/G)
22199	CakSNP22199	*Kabuli*	Ca_Kabuli_denovo	17610652	(A/G)
22200	CakSNP22200	*Kabuli*	Ca_Kabuli_denovo	17610664	(G/C)
22201	CakSNP22201	*Kabuli*	Ca_Kabuli_denovo	17610812	(G/A)
22202	CakSNP22202	*Kabuli*	Ca_Kabuli_denovo	17610820	(C/T)
22203	CakSNP22203	*Kabuli*	Ca_Kabuli_denovo	17611216	(C/T)
22204	CakSNP22204	*Kabuli*	Ca_Kabuli_denovo	17611222	(A/G)
22205	CakSNP22205	*Kabuli*	Ca_Kabuli_denovo	17621283	(C/T)
22206	CakSNP22206	*Kabuli*	Ca_Kabuli_denovo	17621351	(T/C)
22207	CakSNP22207	*Kabuli*	Ca_Kabuli_denovo	17625679	(A/G)
22208	CakSNP22208	*Kabuli*	Ca_Kabuli_denovo	17626513	(T/G)
22209	CakSNP22209	*Kabuli*	Ca_Kabuli_denovo	17627257	(C/T)
22210	CakSNP22210	*Kabuli*	Ca_Kabuli_denovo	17634324	(G/T)
22211	CakSNP22211	*Kabuli*	Ca_Kabuli_denovo	17634364	(G/A)
22212	CakSNP22212	*Kabuli*	Ca_Kabuli_denovo	17643307	(G/A)
22213	CakSNP22213	*Kabuli*	Ca_Kabuli_denovo	17645604	(A/G)
22214	CakSNP22214	*Kabuli*	Ca_Kabuli_denovo	17648275	(C/T)
22215	CakSNP22215	*Kabuli*	Ca_Kabuli_denovo	17651790	(C/T)
22216	CakSNP22216	*Kabuli*	Ca_Kabuli_denovo	17651792	(G/C)
22217	CakSNP22217	*Kabuli*	Ca_Kabuli_denovo	17653212	(C/A)
22218	CakSNP22218	*Kabuli*	Ca_Kabuli_denovo	17653385	(A/G)
22219	CakSNP22219	*Kabuli*	Ca_Kabuli_denovo	17653412	(T/C)
22220	CakSNP22220	*Kabuli*	Ca_Kabuli_denovo	17655910	(G/A)
22221	CakSNP22221	*Kabuli*	Ca_Kabuli_denovo	17659610	(C/T)
22222	CakSNP22222	*Kabuli*	Ca_Kabuli_denovo	17660788	(G/T)
22223	CakSNP22223	*Kabuli*	Ca_Kabuli_denovo	17664464	(G/A)
22224	CakSNP22224	*Kabuli*	Ca_Kabuli_denovo	17664469	(C/T)
22225	CakSNP22225	*Kabuli*	Ca_Kabuli_denovo	17667464	(A/C)
22226	CakSNP22226	*Kabuli*	Ca_Kabuli_denovo	17667796	(C/G)
22227	CakSNP22227	*Kabuli*	Ca_Kabuli_denovo	17667823	(A/G)
22228	CakSNP22228	*Kabuli*	Ca_Kabuli_denovo	17668198	(T/C)
22229	CakSNP22229	*Kabuli*	Ca_Kabuli_denovo	17675941	(C/T)

S.N.	SNP IDs	Cultivars	Chromosomes/scaffolds	Physical positions (bp)	SNPs
22230	CakSNP22230	*Kabuli*	Ca_Kabuli_denovo	17675953	(C/G)
22231	CakSNP22231	*Kabuli*	Ca_Kabuli_denovo	17681320	(T/C)
22232	CakSNP22232	*Kabuli*	Ca_Kabuli_denovo	17686750	(C/T)
22233	CakSNP22233	*Kabuli*	Ca_Kabuli_denovo	17686802	(C/T)
22234	CakSNP22234	*Kabuli*	Ca_Kabuli_denovo	17690386	(A/G)
22235	CakSNP22235	*Kabuli*	Ca_Kabuli_denovo	17690457	(T/G)
22236	CakSNP22236	*Kabuli*	Ca_Kabuli_denovo	17695830	(T/C)
22237	CakSNP22237	*Kabuli*	Ca_Kabuli_denovo	17696236	(A/G)
22238	CakSNP22238	*Kabuli*	Ca_Kabuli_denovo	17703446	(C/T)
22239	CakSNP22239	*Kabuli*	Ca_Kabuli_denovo	17707089	(T/G)
22240	CakSNP22240	*Kabuli*	Ca_Kabuli_denovo	17714825	(A/C)
22241	CakSNP22241	*Kabuli*	Ca_Kabuli_denovo	17719103	(A/C)
22242	CakSNP22242	*Kabuli*	Ca_Kabuli_denovo	17729630	(G/A)
22243	CakSNP22243	*Kabuli*	Ca_Kabuli_denovo	17729707	(G/T)
22244	CakSNP22244	*Kabuli*	Ca_Kabuli_denovo	17730593	(G/C)
22245	CakSNP22245	*Kabuli*	Ca_Kabuli_denovo	17730605	(A/C)
22246	CakSNP22246	*Kabuli*	Ca_Kabuli_denovo	17730836	(A/C)
22247	CakSNP22247	*Kabuli*	Ca_Kabuli_denovo	17737454	(G/A)
22248	CakSNP22248	*Kabuli*	Ca_Kabuli_denovo	17737466	(A/G)
22249	CakSNP22249	*Kabuli*	Ca_Kabuli_denovo	17741720	(G/C)
22250	CakSNP22250	*Kabuli*	Ca_Kabuli_denovo	17743579	(C/T)
22251	CakSNP22251	*Kabuli*	Ca_Kabuli_denovo	17745440	(T/C)
22252	CakSNP22252	*Kabuli*	Ca_Kabuli_denovo	17745478	(C/T)
22253	CakSNP22253	*Kabuli*	Ca_Kabuli_denovo	17746537	(T/G)
22254	CakSNP22254	*Kabuli*	Ca_Kabuli_denovo	17749390	(C/G)
22255	CakSNP22255	*Kabuli*	Ca_Kabuli_denovo	17749399	(T/C)
22256	CakSNP22256	*Kabuli*	Ca_Kabuli_denovo	17753739	(T/A)
22257	CakSNP22257	*Kabuli*	Ca_Kabuli_denovo	17760588	(A/G)
22258	CakSNP22258	*Kabuli*	Ca_Kabuli_denovo	17760620	(C/T)
22259	CakSNP22259	*Kabuli*	Ca_Kabuli_denovo	17760650	(T/G)
22260	CakSNP22260	*Kabuli*	Ca_Kabuli_denovo	17761956	(G/A)
22261	CakSNP22261	*Kabuli*	Ca_Kabuli_denovo	17765524	(G/T)
22262	CakSNP22262	*Kabuli*	Ca_Kabuli_denovo	17765543	(G/C)
22263	CakSNP22263	*Kabuli*	Ca_Kabuli_denovo	17781649	(G/C)
22264	CakSNP22264	*Kabuli*	Ca_Kabuli_denovo	17785598	(G/C)
22265	CakSNP22265	*Kabuli*	Ca_Kabuli_denovo	17785614	(T/G)
22266	CakSNP22266	*Kabuli*	Ca_Kabuli_denovo	17785643	(T/C)
22267	CakSNP22267	*Kabuli*	Ca_Kabuli_denovo	17787071	(A/G)

S.N.	SNP IDs	Cultivars	Chromosomes/scaffolds	Physical positions (bp)	SNPs
22268	CakSNP22268	*Kabuli*	Ca_Kabuli_denovo	17790731	(G/A)
22269	CakSNP22269	*Kabuli*	Ca_Kabuli_denovo	17792770	(C/T)
22270	CakSNP22270	*Kabuli*	Ca_Kabuli_denovo	17792777	(G/A)
22271	CakSNP22271	*Kabuli*	Ca_Kabuli_denovo	17792957	(C/T)
22272	CakSNP22272	*Kabuli*	Ca_Kabuli_denovo	17792964	(G/A)
22273	CakSNP22273	*Kabuli*	Ca_Kabuli_denovo	17809880	(T/C)
22274	CakSNP22274	*Kabuli*	Ca_Kabuli_denovo	17809913	(G/A)
22275	CakSNP22275	*Kabuli*	Ca_Kabuli_denovo	17813020	(G/A)
22276	CakSNP22276	*Kabuli*	Ca_Kabuli_denovo	17813023	(C/G)
22277	CakSNP22277	*Kabuli*	Ca_Kabuli_denovo	17814716	(C/T)
22278	CakSNP22278	*Kabuli*	Ca_Kabuli_denovo	17814788	(C/G)
22279	CakSNP22279	*Kabuli*	Ca_Kabuli_denovo	17818318	(C/A)
22280	CakSNP22280	*Kabuli*	Ca_Kabuli_denovo	17830073	(C/T)
22281	CakSNP22281	*Kabuli*	Ca_Kabuli_denovo	17831657	(A/G)
22282	CakSNP22282	*Kabuli*	Ca_Kabuli_denovo	17831663	(G/T)
22283	CakSNP22283	*Kabuli*	Ca_Kabuli_denovo	17832301	(G/A)
22284	CakSNP22284	*Kabuli*	Ca_Kabuli_denovo	17832314	(G/A)
22285	CakSNP22285	*Kabuli*	Ca_Kabuli_denovo	17837354	(T/C)
22286	CakSNP22286	*Kabuli*	Ca_Kabuli_denovo	17838454	(G/A)
22287	CakSNP22287	*Kabuli*	Ca_Kabuli_denovo	17844685	(A/C)
22288	CakSNP22288	*Kabuli*	Ca_Kabuli_denovo	17844706	(T/C)
22289	CakSNP22289	*Kabuli*	Ca_Kabuli_denovo	17846171	(C/T)
22290	CakSNP22290	*Kabuli*	Ca_Kabuli_denovo	17846249	(A/G)
22291	CakSNP22291	*Kabuli*	Ca_Kabuli_denovo	17846303	(C/T)
22292	CakSNP22292	*Kabuli*	Ca_Kabuli_denovo	17848306	(T/C)
22293	CakSNP22293	*Kabuli*	Ca_Kabuli_denovo	17852659	(G/A)
22294	CakSNP22294	*Kabuli*	Ca_Kabuli_denovo	17863496	(T/C)
22295	CakSNP22295	*Kabuli*	Ca_Kabuli_denovo	17863520	(G/C)
22296	CakSNP22296	*Kabuli*	Ca_Kabuli_denovo	17864834	(C/G)
22297	CakSNP22297	*Kabuli*	Ca_Kabuli_denovo	17865968	(T/C)
22298	CakSNP22298	*Kabuli*	Ca_Kabuli_denovo	17866529	(C/G)
22299	CakSNP22299	*Kabuli*	Ca_Kabuli_denovo	17870735	(G/A)
22300	CakSNP22300	*Kabuli*	Ca_Kabuli_denovo	17870747	(A/G)
22301	CakSNP22301	*Kabuli*	Ca_Kabuli_denovo	17871285	(G/A)
22302	CakSNP22302	*Kabuli*	Ca_Kabuli_denovo	17871294	(C/T)
22303	CakSNP22303	*Kabuli*	Ca_Kabuli_denovo	17872306	(A/C)
22304	CakSNP22304	*Kabuli*	Ca_Kabuli_denovo	17872945	(A/T)
22305	CakSNP22305	*Kabuli*	Ca_Kabuli_denovo	17872976	(A/G)

S.N.	SNP IDs	Cultivars	Chromosomes/scaffolds	Physical positions (bp)	SNPs
22306	CakSNP22306	*Kabuli*	Ca_Kabuli_denovo	17884474	(T/G)
22307	CakSNP22307	*Kabuli*	Ca_Kabuli_denovo	17889232	(G/C)
22308	CakSNP22308	*Kabuli*	Ca_Kabuli_denovo	17889257	(A/G)
22309	CakSNP22309	*Kabuli*	Ca_Kabuli_denovo	17891779	(T/C)
22310	CakSNP22310	*Kabuli*	Ca_Kabuli_denovo	17892202	(T/C)
22311	CakSNP22311	*Kabuli*	Ca_Kabuli_denovo	17901128	(C/T)
22312	CakSNP22312	*Kabuli*	Ca_Kabuli_denovo	17901641	(A/G)
22313	CakSNP22313	*Kabuli*	Ca_Kabuli_denovo	17901648	(T/A)
22314	CakSNP22314	*Kabuli*	Ca_Kabuli_denovo	17910343	(A/G)
22315	CakSNP22315	*Kabuli*	Ca_Kabuli_denovo	17910397	(A/C)
22316	CakSNP22316	*Kabuli*	Ca_Kabuli_denovo	17913667	(A/G)
22317	CakSNP22317	*Kabuli*	Ca_Kabuli_denovo	17913745	(T/C)
22318	CakSNP22318	*Kabuli*	Ca_Kabuli_denovo	17919055	(A/C)
22319	CakSNP22319	*Kabuli*	Ca_Kabuli_denovo	17925237	(G/A)
22320	CakSNP22320	*Kabuli*	Ca_Kabuli_denovo	17925240	(T/G)
22321	CakSNP22321	*Kabuli*	Ca_Kabuli_denovo	17928555	(G/T)
22322	CakSNP22322	*Kabuli*	Ca_Kabuli_denovo	17928573	(C/G)
22323	CakSNP22323	*Kabuli*	Ca_Kabuli_denovo	17932364	(C/T)
22324	CakSNP22324	*Kabuli*	Ca_Kabuli_denovo	17935615	(C/A)
22325	CakSNP22325	*Kabuli*	Ca_Kabuli_denovo	17935649	(T/G)
22326	CakSNP22326	*Kabuli*	Ca_Kabuli_denovo	17935692	(C/T)
22327	CakSNP22327	*Kabuli*	Ca_Kabuli_denovo	17935701	(A/G)
22328	CakSNP22328	*Kabuli*	Ca_Kabuli_denovo	17942231	(A/G)
22329	CakSNP22329	*Kabuli*	Ca_Kabuli_denovo	17949666	(A/G)
22330	CakSNP22330	*Kabuli*	Ca_Kabuli_denovo	17950774	(A/G)
22331	CakSNP22331	*Kabuli*	Ca_Kabuli_denovo	17957321	(C/A)
22332	CakSNP22332	*Kabuli*	Ca_Kabuli_denovo	17957334	(C/G)
22333	CakSNP22333	*Kabuli*	Ca_Kabuli_denovo	17963997	(T/G)
22334	CakSNP22334	*Kabuli*	Ca_Kabuli_denovo	17968728	(G/A)
22335	CakSNP22335	*Kabuli*	Ca_Kabuli_denovo	17969758	(G/C)
22336	CakSNP22336	*Kabuli*	Ca_Kabuli_denovo	17971133	(G/C)
22337	CakSNP22337	*Kabuli*	Ca_Kabuli_denovo	17971802	(G/T)
22338	CakSNP22338	*Kabuli*	Ca_Kabuli_denovo	17971807	(G/C)
22339	CakSNP22339	*Kabuli*	Ca_Kabuli_denovo	17972205	(A/G)
22340	CakSNP22340	*Kabuli*	Ca_Kabuli_denovo	17974116	(G/A)
22341	CakSNP22341	*Kabuli*	Ca_Kabuli_denovo	17974129	(C/T)
22342	CakSNP22342	*Kabuli*	Ca_Kabuli_denovo	17985164	(C/T)
22343	CakSNP22343	*Kabuli*	Ca_Kabuli_denovo	17986886	(T/A)

S.N.	SNP IDs	Cultivars	Chromosomes/scaffolds	Physical positions (bp)	SNPs
22344	CakSNP22344	*Kabuli*	Ca_Kabuli_denovo	17991233	(C/G)
22345	CakSNP22345	*Kabuli*	Ca_Kabuli_denovo	17991279	(G/T)
22346	CakSNP22346	*Kabuli*	Ca_Kabuli_denovo	17999993	(A/G)
22347	CakSNP22347	*Kabuli*	Ca_Kabuli_denovo	18000376	(T/C)
22348	CakSNP22348	*Kabuli*	Ca_Kabuli_denovo	18009494	(G/A)
22349	CakSNP22349	*Kabuli*	Ca_Kabuli_denovo	18009530	(G/A)
22350	CakSNP22350	*Kabuli*	Ca_Kabuli_denovo	18015260	(C/T)
22351	CakSNP22351	*Kabuli*	Ca_Kabuli_denovo	18015266	(T/C)
22352	CakSNP22352	*Kabuli*	Ca_Kabuli_denovo	18019036	(G/T)
22353	CakSNP22353	*Kabuli*	Ca_Kabuli_denovo	18019054	(G/C)
22354	CakSNP22354	*Kabuli*	Ca_Kabuli_denovo	18021175	(A/C)
22355	CakSNP22355	*Kabuli*	Ca_Kabuli_denovo	18021208	(C/T)
22356	CakSNP22356	*Kabuli*	Ca_Kabuli_denovo	18022504	(A/G)
22357	CakSNP22357	*Kabuli*	Ca_Kabuli_denovo	18030218	(A/G)
22358	CakSNP22358	*Kabuli*	Ca_Kabuli_denovo	18030245	(G/C)
22359	CakSNP22359	*Kabuli*	Ca_Kabuli_denovo	18045236	(A/G)
22360	CakSNP22360	*Kabuli*	Ca_Kabuli_denovo	18045254	(G/C)
22361	CakSNP22361	*Kabuli*	Ca_Kabuli_denovo	18049288	(A/G)
22362	CakSNP22362	*Kabuli*	Ca_Kabuli_denovo	18057460	(A/G)
22363	CakSNP22363	*Kabuli*	Ca_Kabuli_denovo	18057493	(A/G)
22364	CakSNP22364	*Kabuli*	Ca_Kabuli_denovo	18062095	(T/C)
22365	CakSNP22365	*Kabuli*	Ca_Kabuli_denovo	18062116	(C/T)
22366	CakSNP22366	*Kabuli*	Ca_Kabuli_denovo	18063677	(T/G)
22367	CakSNP22367	*Kabuli*	Ca_Kabuli_denovo	18065873	(T/G)
22368	CakSNP22368	*Kabuli*	Ca_Kabuli_denovo	18073526	(A/C)
22369	CakSNP22369	*Kabuli*	Ca_Kabuli_denovo	18073553	(C/T)
22370	CakSNP22370	*Kabuli*	Ca_Kabuli_denovo	18075293	(G/C)
22371	CakSNP22371	*Kabuli*	Ca_Kabuli_denovo	18078197	(G/A)
22372	CakSNP22372	*Kabuli*	Ca_Kabuli_denovo	18078269	(C/T)
22373	CakSNP22373	*Kabuli*	Ca_Kabuli_denovo	18085071	(C/T)
22374	CakSNP22374	*Kabuli*	Ca_Kabuli_denovo	18086849	(A/C)
22375	CakSNP22375	*Kabuli*	Ca_Kabuli_denovo	18095969	(C/T)
22376	CakSNP22376	*Kabuli*	Ca_Kabuli_denovo	18096648	(T/C)
22377	CakSNP22377	*Kabuli*	Ca_Kabuli_denovo	18100045	(T/C)
22378	CakSNP22378	*Kabuli*	Ca_Kabuli_denovo	18100062	(G/C)
22379	CakSNP22379	*Kabuli*	Ca_Kabuli_denovo	18109396	(C/A)
22380	CakSNP22380	*Kabuli*	Ca_Kabuli_denovo	18109402	(T/C)
22381	CakSNP22381	*Kabuli*	Ca_Kabuli_denovo	18111873	(G/A)

S.N.	SNP IDs	Cultivars	Chromosomes/scaffolds	Physical positions (bp)	SNPs
22382	CakSNP22382	*Kabuli*	Ca_Kabuli_denovo	18111898	(C/T)
22383	CakSNP22383	*Kabuli*	Ca_Kabuli_denovo	18113153	(C/A)
22384	CakSNP22384	*Kabuli*	Ca_Kabuli_denovo	18113171	(G/A)
22385	CakSNP22385	*Kabuli*	Ca_Kabuli_denovo	18113367	(C/A)
22386	CakSNP22386	*Kabuli*	Ca_Kabuli_denovo	18113809	(G/A)
22387	CakSNP22387	*Kabuli*	Ca_Kabuli_denovo	18115789	(T/A)
22388	CakSNP22388	*Kabuli*	Ca_Kabuli_denovo	18115810	(G/C)
22389	CakSNP22389	*Kabuli*	Ca_Kabuli_denovo	18116597	(T/C)
22390	CakSNP22390	*Kabuli*	Ca_Kabuli_denovo	18116666	(A/T)
22391	CakSNP22391	*Kabuli*	Ca_Kabuli_denovo	18118195	(T/G)
22392	CakSNP22392	*Kabuli*	Ca_Kabuli_denovo	18120678	(A/C)
22393	CakSNP22393	*Kabuli*	Ca_Kabuli_denovo	18126053	(T/C)
22394	CakSNP22394	*Kabuli*	Ca_Kabuli_denovo	18127484	(G/C)
22395	CakSNP22395	*Kabuli*	Ca_Kabuli_denovo	18130665	(G/A)
22396	CakSNP22396	*Kabuli*	Ca_Kabuli_denovo	18131139	(C/A)
22397	CakSNP22397	*Kabuli*	Ca_Kabuli_denovo	18131168	(G/A)
22398	CakSNP22398	*Kabuli*	Ca_Kabuli_denovo	18132154	(A/G)
22399	CakSNP22399	*Kabuli*	Ca_Kabuli_denovo	18136870	(T/G)
22400	CakSNP22400	*Kabuli*	Ca_Kabuli_denovo	18136875	(G/C)
22401	CakSNP22401	*Kabuli*	Ca_Kabuli_denovo	18143261	(C/T)
22402	CakSNP22402	*Kabuli*	Ca_Kabuli_denovo	18145646	(T/C)
22403	CakSNP22403	*Kabuli*	Ca_Kabuli_denovo	18145676	(C/T)
22404	CakSNP22404	*Kabuli*	Ca_Kabuli_denovo	18150116	(A/G)
22405	CakSNP22405	*Kabuli*	Ca_Kabuli_denovo	18150140	(G/T)
22406	CakSNP22406	*Kabuli*	Ca_Kabuli_denovo	18152696	(G/C)
22407	CakSNP22407	*Kabuli*	Ca_Kabuli_denovo	18155798	(A/G)
22408	CakSNP22408	*Kabuli*	Ca_Kabuli_denovo	18155801	(A/G)
22409	CakSNP22409	*Kabuli*	Ca_Kabuli_denovo	18157850	(A/C)
22410	CakSNP22410	*Kabuli*	Ca_Kabuli_denovo	18159030	(C/T)
22411	CakSNP22411	*Kabuli*	Ca_Kabuli_denovo	18161129	(C/G)
22412	CakSNP22412	*Kabuli*	Ca_Kabuli_denovo	18164824	(T/A)
22413	CakSNP22413	*Kabuli*	Ca_Kabuli_denovo	18166118	(G/C)
22414	CakSNP22414	*Kabuli*	Ca_Kabuli_denovo	18166365	(T/C)
22415	CakSNP22415	*Kabuli*	Ca_Kabuli_denovo	18166405	(G/A)
22416	CakSNP22416	*Kabuli*	Ca_Kabuli_denovo	18167705	(A/T)
22417	CakSNP22417	*Kabuli*	Ca_Kabuli_denovo	18169954	(C/T)
22418	CakSNP22418	*Kabuli*	Ca_Kabuli_denovo	18170385	(A/G)
22419	CakSNP22419	*Kabuli*	Ca_Kabuli_denovo	18173531	(C/T)

S.N.	SNP IDs	Cultivars	Chromosomes/scaffolds	Physical positions (bp)	SNPs
22420	CakSNP22420	*Kabuli*	Ca_Kabuli_denovo	18178644	(G/T)
22421	CakSNP22421	*Kabuli*	Ca_Kabuli_denovo	18178846	(C/T)
22422	CakSNP22422	*Kabuli*	Ca_Kabuli_denovo	18183712	(T/G)
22423	CakSNP22423	*Kabuli*	Ca_Kabuli_denovo	18185898	(T/C)
22424	CakSNP22424	*Kabuli*	Ca_Kabuli_denovo	18185916	(A/G)
22425	CakSNP22425	*Kabuli*	Ca_Kabuli_denovo	18196616	(T/C)
22426	CakSNP22426	*Kabuli*	Ca_Kabuli_denovo	18196660	(G/C)
22427	CakSNP22427	*Kabuli*	Ca_Kabuli_denovo	18199026	(C/T)
22428	CakSNP22428	*Kabuli*	Ca_Kabuli_denovo	18209704	(G/A)
22429	CakSNP22429	*Kabuli*	Ca_Kabuli_denovo	18209734	(A/C)
22430	CakSNP22430	*Kabuli*	Ca_Kabuli_denovo	18214361	(T/C)
22431	CakSNP22431	*Kabuli*	Ca_Kabuli_denovo	18214422	(A/C)
22432	CakSNP22432	*Kabuli*	Ca_Kabuli_denovo	18214627	(C/G)
22433	CakSNP22433	*Kabuli*	Ca_Kabuli_denovo	18214630	(C/T)
22434	CakSNP22434	*Kabuli*	Ca_Kabuli_denovo	18215680	(A/T)
22435	CakSNP22435	*Kabuli*	Ca_Kabuli_denovo	18215725	(G/A)
22436	CakSNP22436	*Kabuli*	Ca_Kabuli_denovo	18225599	(A/G)
22437	CakSNP22437	*Kabuli*	Ca_Kabuli_denovo	18225626	(G/A)
22438	CakSNP22438	*Kabuli*	Ca_Kabuli_denovo	18226822	(C/T)
22439	CakSNP22439	*Kabuli*	Ca_Kabuli_denovo	18229442	(C/A)
22440	CakSNP22440	*Kabuli*	Ca_Kabuli_denovo	18232712	(C/T)
22441	CakSNP22441	*Kabuli*	Ca_Kabuli_denovo	18232715	(A/G)
22442	CakSNP22442	*Kabuli*	Ca_Kabuli_denovo	18240760	(G/C)
22443	CakSNP22443	*Kabuli*	Ca_Kabuli_denovo	18240772	(C/T)
22444	CakSNP22444	*Kabuli*	Ca_Kabuli_denovo	18244217	(G/C)
22445	CakSNP22445	*Kabuli*	Ca_Kabuli_denovo	18244244	(A/G)
22446	CakSNP22446	*Kabuli*	Ca_Kabuli_denovo	18249035	(G/T)
22447	CakSNP22447	*Kabuli*	Ca_Kabuli_denovo	18254434	(C/G)
22448	CakSNP22448	*Kabuli*	Ca_Kabuli_denovo	18254479	(A/G)
22449	CakSNP22449	*Kabuli*	Ca_Kabuli_denovo	18255571	(T/C)
22450	CakSNP22450	*Kabuli*	Ca_Kabuli_denovo	18255580	(G/T)
22451	CakSNP22451	*Kabuli*	Ca_Kabuli_denovo	18255938	(T/C)
22452	CakSNP22452	*Kabuli*	Ca_Kabuli_denovo	18259685	(T/C)
22453	CakSNP22453	*Kabuli*	Ca_Kabuli_denovo	18259712	(G/C)
22454	CakSNP22454	*Kabuli*	Ca_Kabuli_denovo	18260199	(G/A)
22455	CakSNP22455	*Kabuli*	Ca_Kabuli_denovo	18260208	(A/C)
22456	CakSNP22456	*Kabuli*	Ca_Kabuli_denovo	18268558	(T/G)
22457	CakSNP22457	*Kabuli*	Ca_Kabuli_denovo	18268564	(T/C)

S.N.	SNP IDs	Cultivars	Chromosomes/scaffolds	Physical positions (bp)	SNPs
22458	CakSNP22458	*Kabuli*	Ca_Kabuli_denovo	18269034	(A/G)
22459	CakSNP22459	*Kabuli*	Ca_Kabuli_denovo	18270676	(T/C)
22460	CakSNP22460	*Kabuli*	Ca_Kabuli_denovo	18270688	(T/G)
22461	CakSNP22461	*Kabuli*	Ca_Kabuli_denovo	18270828	(G/A)
22462	CakSNP22462	*Kabuli*	Ca_Kabuli_denovo	18270846	(C/G)
22463	CakSNP22463	*Kabuli*	Ca_Kabuli_denovo	18274147	(T/C)
22464	CakSNP22464	*Kabuli*	Ca_Kabuli_denovo	18275905	(T/C)
22465	CakSNP22465	*Kabuli*	Ca_Kabuli_denovo	18276932	(A/G)
22466	CakSNP22466	*Kabuli*	Ca_Kabuli_denovo	18279539	(C/T)
22467	CakSNP22467	*Kabuli*	Ca_Kabuli_denovo	18284434	(G/A)
22468	CakSNP22468	*Kabuli*	Ca_Kabuli_denovo	18284503	(G/C)
22469	CakSNP22469	*Kabuli*	Ca_Kabuli_denovo	18288645	(T/C)
22470	CakSNP22470	*Kabuli*	Ca_Kabuli_denovo	18292460	(G/A)
22471	CakSNP22471	*Kabuli*	Ca_Kabuli_denovo	18293034	(A/G)
22472	CakSNP22472	*Kabuli*	Ca_Kabuli_denovo	18303244	(T/C)
22473	CakSNP22473	*Kabuli*	Ca_Kabuli_denovo	18312877	(G/A)
22474	CakSNP22474	*Kabuli*	Ca_Kabuli_denovo	18315388	(T/C)
22475	CakSNP22475	*Kabuli*	Ca_Kabuli_denovo	18326563	(C/G)
22476	CakSNP22476	*Kabuli*	Ca_Kabuli_denovo	18326575	(C/T)
22477	CakSNP22477	*Kabuli*	Ca_Kabuli_denovo	18326977	(G/A)
22478	CakSNP22478	*Kabuli*	Ca_Kabuli_denovo	18330217	(T/C)
22479	CakSNP22479	*Kabuli*	Ca_Kabuli_denovo	18330247	(C/T)
22480	CakSNP22480	*Kabuli*	Ca_Kabuli_denovo	18349380	(C/G)
22481	CakSNP22481	*Kabuli*	Ca_Kabuli_denovo	18351620	(T/G)
22482	CakSNP22482	*Kabuli*	Ca_Kabuli_denovo	18352549	(T/C)
22483	CakSNP22483	*Kabuli*	Ca_Kabuli_denovo	18352558	(A/C)
22484	CakSNP22484	*Kabuli*	Ca_Kabuli_denovo	18354146	(A/G)
22485	CakSNP22485	*Kabuli*	Ca_Kabuli_denovo	18354163	(T/C)
22486	CakSNP22486	*Kabuli*	Ca_Kabuli_denovo	18357472	(A/G)
22487	CakSNP22487	*Kabuli*	Ca_Kabuli_denovo	18368995	(G/A)
22488	CakSNP22488	*Kabuli*	Ca_Kabuli_denovo	18369476	(G/T)
22489	CakSNP22489	*Kabuli*	Ca_Kabuli_denovo	18369482	(T/C)
22490	CakSNP22490	*Kabuli*	Ca_Kabuli_denovo	18371972	(A/C)
22491	CakSNP22491	*Kabuli*	Ca_Kabuli_denovo	18371974	(T/C)
22492	CakSNP22492	*Kabuli*	Ca_Kabuli_denovo	18378184	(A/G)
22493	CakSNP22493	*Kabuli*	Ca_Kabuli_denovo	18378189	(G/A)
22494	CakSNP22494	*Kabuli*	Ca_Kabuli_denovo	18379267	(G/A)
22495	CakSNP22495	*Kabuli*	Ca_Kabuli_denovo	18380355	(A/G)

S.N.	SNP IDs	Cultivars	Chromosomes/scaffolds	Physical positions (bp)	SNPs
22496	CakSNP22496	*Kabuli*	Ca_Kabuli_denovo	18382345	(G/C)
22497	CakSNP22497	*Kabuli*	Ca_Kabuli_denovo	18383189	(C/T)
22498	CakSNP22498	*Kabuli*	Ca_Kabuli_denovo	18387158	(T/C)
22499	CakSNP22499	*Kabuli*	Ca_Kabuli_denovo	18387172	(G/A)
22500	CakSNP22500	*Kabuli*	Ca_Kabuli_denovo	18395703	(G/A)
22501	CakSNP22501	*Kabuli*	Ca_Kabuli_denovo	18399965	(G/C)
22502	CakSNP22502	*Kabuli*	Ca_Kabuli_denovo	18399994	(T/C)
22503	CakSNP22503	*Kabuli*	Ca_Kabuli_denovo	18400927	(G/C)
22504	CakSNP22504	*Kabuli*	Ca_Kabuli_denovo	18400934	(G/A)
22505	CakSNP22505	*Kabuli*	Ca_Kabuli_denovo	18411242	(T/C)
22506	CakSNP22506	*Kabuli*	Ca_Kabuli_denovo	18411256	(G/A)
22507	CakSNP22507	*Kabuli*	Ca_Kabuli_denovo	18416998	(G/C)
22508	CakSNP22508	*Kabuli*	Ca_Kabuli_denovo	18416999	(C/G)
22509	CakSNP22509	*Kabuli*	Ca_Kabuli_denovo	18417740	(C/G)
22510	CakSNP22510	*Kabuli*	Ca_Kabuli_denovo	18417754	(T/C)
22511	CakSNP22511	*Kabuli*	Ca_Kabuli_denovo	18423214	(C/T)
22512	CakSNP22512	*Kabuli*	Ca_Kabuli_denovo	18423619	(C/T)
22513	CakSNP22513	*Kabuli*	Ca_Kabuli_denovo	18423628	(T/C)
22514	CakSNP22514	*Kabuli*	Ca_Kabuli_denovo	18423994	(G/A)
22515	CakSNP22515	*Kabuli*	Ca_Kabuli_denovo	18428416	(G/T)
22516	CakSNP22516	*Kabuli*	Ca_Kabuli_denovo	18429367	(T/G)
22517	CakSNP22517	*Kabuli*	Ca_Kabuli_denovo	18429383	(A/G)
22518	CakSNP22518	*Kabuli*	Ca_Kabuli_denovo	18432130	(T/C)
22519	CakSNP22519	*Kabuli*	Ca_Kabuli_denovo	18432188	(T/C)
22520	CakSNP22520	*Kabuli*	Ca_Kabuli_denovo	18437366	(C/A)
22521	CakSNP22521	*Kabuli*	Ca_Kabuli_denovo	18438204	(A/G)
22522	CakSNP22522	*Kabuli*	Ca_Kabuli_denovo	18440440	(G/A)
22523	CakSNP22523	*Kabuli*	Ca_Kabuli_denovo	18451604	(G/A)
22524	CakSNP22524	*Kabuli*	Ca_Kabuli_denovo	18451622	(C/A)
22525	CakSNP22525	*Kabuli*	Ca_Kabuli_denovo	18455332	(G/A)
22526	CakSNP22526	*Kabuli*	Ca_Kabuli_denovo	18455350	(C/A)
22527	CakSNP22527	*Kabuli*	Ca_Kabuli_denovo	18455356	(T/C)
22528	CakSNP22528	*Kabuli*	Ca_Kabuli_denovo	18459497	(C/G)
22529	CakSNP22529	*Kabuli*	Ca_Kabuli_denovo	18462101	(A/C)
22530	CakSNP22530	*Kabuli*	Ca_Kabuli_denovo	18464620	(A/G)
22531	CakSNP22531	*Kabuli*	Ca_Kabuli_denovo	18464648	(G/A)
22532	CakSNP22532	*Kabuli*	Ca_Kabuli_denovo	18466527	(G/C)
22533	CakSNP22533	*Kabuli*	Ca_Kabuli_denovo	18466539	(T/C)

S.N.	SNP IDs	Cultivars	Chromosomes/scaffolds	Physical positions (bp)	SNPs
22534	CakSNP22534	*Kabuli*	Ca_Kabuli_denovo	18469553	(G/T)
22535	CakSNP22535	*Kabuli*	Ca_Kabuli_denovo	18470860	(T/C)
22536	CakSNP22536	*Kabuli*	Ca_Kabuli_denovo	18474096	(A/G)
22537	CakSNP22537	*Kabuli*	Ca_Kabuli_denovo	18476944	(T/G)
22538	CakSNP22538	*Kabuli*	Ca_Kabuli_denovo	18477935	(A/G)
22539	CakSNP22539	*Kabuli*	Ca_Kabuli_denovo	18479017	(G/C)
22540	CakSNP22540	*Kabuli*	Ca_Kabuli_denovo	18484876	(C/T)
22541	CakSNP22541	*Kabuli*	Ca_Kabuli_denovo	18492425	(G/C)
22542	CakSNP22542	*Kabuli*	Ca_Kabuli_denovo	18492458	(A/G)
22543	CakSNP22543	*Kabuli*	Ca_Kabuli_denovo	18492911	(G/C)
22544	CakSNP22544	*Kabuli*	Ca_Kabuli_denovo	18497528	(G/A)
22545	CakSNP22545	*Kabuli*	Ca_Kabuli_denovo	18497537	(T/C)
22546	CakSNP22546	*Kabuli*	Ca_Kabuli_denovo	18501632	(G/A)
22547	CakSNP22547	*Kabuli*	Ca_Kabuli_denovo	18501638	(G/A)
22548	CakSNP22548	*Kabuli*	Ca_Kabuli_denovo	18504613	(C/A)
22549	CakSNP22549	*Kabuli*	Ca_Kabuli_denovo	18509084	(C/T)
22550	CakSNP22550	*Kabuli*	Ca_Kabuli_denovo	18512044	(A/C)
22551	CakSNP22551	*Kabuli*	Ca_Kabuli_denovo	18512047	(G/A)
22552	CakSNP22552	*Kabuli*	Ca_Kabuli_denovo	18512138	(A/G)
22553	CakSNP22553	*Kabuli*	Ca_Kabuli_denovo	18515042	(A/G)
22554	CakSNP22554	*Kabuli*	Ca_Kabuli_denovo	18518254	(A/C)
22555	CakSNP22555	*Kabuli*	Ca_Kabuli_denovo	18520034	(C/G)
22556	CakSNP22556	*Kabuli*	Ca_Kabuli_denovo	18520055	(T/C)
22557	CakSNP22557	*Kabuli*	Ca_Kabuli_denovo	18524435	(C/T)
22558	CakSNP22558	*Kabuli*	Ca_Kabuli_denovo	18524445	(A/C)
22559	CakSNP22559	*Kabuli*	Ca_Kabuli_denovo	18524597	(C/G)
22560	CakSNP22560	*Kabuli*	Ca_Kabuli_denovo	18529410	(T/C)
22561	CakSNP22561	*Kabuli*	Ca_Kabuli_denovo	18539598	(C/A)
22562	CakSNP22562	*Kabuli*	Ca_Kabuli_denovo	18542780	(T/A)
22563	CakSNP22563	*Kabuli*	Ca_Kabuli_denovo	18543466	(C/G)
22564	CakSNP22564	*Kabuli*	Ca_Kabuli_denovo	18543550	(G/T)
22565	CakSNP22565	*Kabuli*	Ca_Kabuli_denovo	18543835	(T/C)
22566	CakSNP22566	*Kabuli*	Ca_Kabuli_denovo	18545659	(A/G)
22567	CakSNP22567	*Kabuli*	Ca_Kabuli_denovo	18552228	(C/G)
22568	CakSNP22568	*Kabuli*	Ca_Kabuli_denovo	18558448	(C/A)
22569	CakSNP22569	*Kabuli*	Ca_Kabuli_denovo	18559193	(C/T)
22570	CakSNP22570	*Kabuli*	Ca_Kabuli_denovo	18559231	(G/A)
22571	CakSNP22571	*Kabuli*	Ca_Kabuli_denovo	18559927	(G/A)

S.N.	SNP IDs	Cultivars	Chromosomes/scaffolds	Physical positions (bp)	SNPs
22572	CakSNP22572	*Kabuli*	Ca_Kabuli_denovo	18563930	(G/A)
22573	CakSNP22573	*Kabuli*	Ca_Kabuli_denovo	18563978	(C/T)
22574	CakSNP22574	*Kabuli*	Ca_Kabuli_denovo	18566071	(C/T)
22575	CakSNP22575	*Kabuli*	Ca_Kabuli_denovo	18568152	(T/C)
22576	CakSNP22576	*Kabuli*	Ca_Kabuli_denovo	18568157	(T/C)
22577	CakSNP22577	*Kabuli*	Ca_Kabuli_denovo	18569485	(C/T)
22578	CakSNP22578	*Kabuli*	Ca_Kabuli_denovo	18569488	(T/C)
22579	CakSNP22579	*Kabuli*	Ca_Kabuli_denovo	18573632	(G/A)
22580	CakSNP22580	*Kabuli*	Ca_Kabuli_denovo	18579933	(C/T)
22581	CakSNP22581	*Kabuli*	Ca_Kabuli_denovo	18581122	(A/C)
22582	CakSNP22582	*Kabuli*	Ca_Kabuli_denovo	18586183	(G/T)
22583	CakSNP22583	*Kabuli*	Ca_Kabuli_denovo	18586211	(A/G)
22584	CakSNP22584	*Kabuli*	Ca_Kabuli_denovo	18587105	(C/T)
22585	CakSNP22585	*Kabuli*	Ca_Kabuli_denovo	18589602	(T/C)
22586	CakSNP22586	*Kabuli*	Ca_Kabuli_denovo	18591097	(A/G)
22587	CakSNP22587	*Kabuli*	Ca_Kabuli_denovo	18592866	(C/G)
22588	CakSNP22588	*Kabuli*	Ca_Kabuli_denovo	18592869	(C/T)
22589	CakSNP22589	*Kabuli*	Ca_Kabuli_denovo	18596290	(T/C)
22590	CakSNP22590	*Kabuli*	Ca_Kabuli_denovo	18596296	(T/G)
22591	CakSNP22591	*Kabuli*	Ca_Kabuli_denovo	18597427	(G/A)
22592	CakSNP22592	*Kabuli*	Ca_Kabuli_denovo	18597485	(C/T)
22593	CakSNP22593	*Kabuli*	Ca_Kabuli_denovo	18598249	(C/G)
22594	CakSNP22594	*Kabuli*	Ca_Kabuli_denovo	18606359	(C/A)
22595	CakSNP22595	*Kabuli*	Ca_Kabuli_denovo	18608180	(G/A)
22596	CakSNP22596	*Kabuli*	Ca_Kabuli_denovo	18608195	(T/C)
22597	CakSNP22597	*Kabuli*	Ca_Kabuli_denovo	18614037	(T/C)
22598	CakSNP22598	*Kabuli*	Ca_Kabuli_denovo	18618464	(A/G)
22599	CakSNP22599	*Kabuli*	Ca_Kabuli_denovo	18620233	(T/C)
22600	CakSNP22600	*Kabuli*	Ca_Kabuli_denovo	18623050	(G/A)
22601	CakSNP22601	*Kabuli*	Ca_Kabuli_denovo	18623064	(G/A)
22602	CakSNP22602	*Kabuli*	Ca_Kabuli_denovo	18623808	(T/C)
22603	CakSNP22603	*Kabuli*	Ca_Kabuli_denovo	18624662	(G/A)
22604	CakSNP22604	*Kabuli*	Ca_Kabuli_denovo	18624688	(T/C)
22605	CakSNP22605	*Kabuli*	Ca_Kabuli_denovo	18627935	(A/G)
22606	CakSNP22606	*Kabuli*	Ca_Kabuli_denovo	18632591	(A/C)
22607	CakSNP22607	*Kabuli*	Ca_Kabuli_denovo	18633248	(G/A)
22608	CakSNP22608	*Kabuli*	Ca_Kabuli_denovo	18639938	(A/G)
22609	CakSNP22609	*Kabuli*	Ca_Kabuli_denovo	18641122	(C/G)

S.N.	SNP IDs	Cultivars	Chromosomes/scaffolds	Physical positions (bp)	SNPs
22610	CakSNP22610	*Kabuli*	Ca_Kabuli_denovo	18641197	(C/G)
22611	CakSNP22611	*Kabuli*	Ca_Kabuli_denovo	18645319	(C/T)
22612	CakSNP22612	*Kabuli*	Ca_Kabuli_denovo	18646865	(T/C)
22613	CakSNP22613	*Kabuli*	Ca_Kabuli_denovo	18650506	(G/A)
22614	CakSNP22614	*Kabuli*	Ca_Kabuli_denovo	18651436	(C/A)
22615	CakSNP22615	*Kabuli*	Ca_Kabuli_denovo	18651511	(T/C)
22616	CakSNP22616	*Kabuli*	Ca_Kabuli_denovo	18663629	(G/A)
22617	CakSNP22617	*Kabuli*	Ca_Kabuli_denovo	18663648	(T/A)
22618	CakSNP22618	*Kabuli*	Ca_Kabuli_denovo	18670780	(T/G)
22619	CakSNP22619	*Kabuli*	Ca_Kabuli_denovo	18671340	(T/C)
22620	CakSNP22620	*Kabuli*	Ca_Kabuli_denovo	18671551	(A/G)
22621	CakSNP22621	*Kabuli*	Ca_Kabuli_denovo	18671557	(G/C)
22622	CakSNP22622	*Kabuli*	Ca_Kabuli_denovo	18675353	(A/G)
22623	CakSNP22623	*Kabuli*	Ca_Kabuli_denovo	18675369	(G/C)
22624	CakSNP22624	*Kabuli*	Ca_Kabuli_denovo	18676288	(G/A)
22625	CakSNP22625	*Kabuli*	Ca_Kabuli_denovo	18676421	(T/C)
22626	CakSNP22626	*Kabuli*	Ca_Kabuli_denovo	18676427	(T/C)
22627	CakSNP22627	*Kabuli*	Ca_Kabuli_denovo	18678872	(A/G)
22628	CakSNP22628	*Kabuli*	Ca_Kabuli_denovo	18680021	(C/T)
22629	CakSNP22629	*Kabuli*	Ca_Kabuli_denovo	18680071	(C/A)
22630	CakSNP22630	*Kabuli*	Ca_Kabuli_denovo	18680107	(A/C)
22631	CakSNP22631	*Kabuli*	Ca_Kabuli_denovo	18685944	(A/C)
22632	CakSNP22632	*Kabuli*	Ca_Kabuli_denovo	18690041	(C/A)
22633	CakSNP22633	*Kabuli*	Ca_Kabuli_denovo	18690059	(A/T)
22634	CakSNP22634	*Kabuli*	Ca_Kabuli_denovo	18696550	(C/T)
22635	CakSNP22635	*Kabuli*	Ca_Kabuli_denovo	18696580	(G/A)
22636	CakSNP22636	*Kabuli*	Ca_Kabuli_denovo	18700033	(G/C)
22637	CakSNP22637	*Kabuli*	Ca_Kabuli_denovo	18704924	(A/C)
22638	CakSNP22638	*Kabuli*	Ca_Kabuli_denovo	18705814	(A/C)
22639	CakSNP22639	*Kabuli*	Ca_Kabuli_denovo	18712035	(G/T)
22640	CakSNP22640	*Kabuli*	Ca_Kabuli_denovo	18713763	(A/T)
22641	CakSNP22641	*Kabuli*	Ca_Kabuli_denovo	18713777	(G/A)
22642	CakSNP22642	*Kabuli*	Ca_Kabuli_denovo	18713783	(T/C)
22643	CakSNP22643	*Kabuli*	Ca_Kabuli_denovo	18716914	(A/G)
22644	CakSNP22644	*Kabuli*	Ca_Kabuli_denovo	18722230	(G/T)
22645	CakSNP22645	*Kabuli*	Ca_Kabuli_denovo	18724934	(G/A)
22646	CakSNP22646	*Kabuli*	Ca_Kabuli_denovo	18726509	(A/G)
22647	CakSNP22647	*Kabuli*	Ca_Kabuli_denovo	18727704	(A/G)

S.N.	SNP IDs	Cultivars	Chromosomes/scaffolds	Physical positions (bp)	SNPs
22648	CakSNP22648	*Kabuli*	Ca_Kabuli_denovo	18727738	(G/C)
22649	CakSNP22649	*Kabuli*	Ca_Kabuli_denovo	18728749	(T/G)
22650	CakSNP22650	*Kabuli*	Ca_Kabuli_denovo	18728774	(T/G)
22651	CakSNP22651	*Kabuli*	Ca_Kabuli_denovo	18733070	(C/T)
22652	CakSNP22652	*Kabuli*	Ca_Kabuli_denovo	18733079	(C/G)
22653	CakSNP22653	*Kabuli*	Ca_Kabuli_denovo	18739407	(A/C)
22654	CakSNP22654	*Kabuli*	Ca_Kabuli_denovo	18739408	(A/G)
22655	CakSNP22655	*Kabuli*	Ca_Kabuli_denovo	18747163	(G/A)
22656	CakSNP22656	*Kabuli*	Ca_Kabuli_denovo	18748718	(C/A)
22657	CakSNP22657	*Kabuli*	Ca_Kabuli_denovo	18748786	(T/C)
22658	CakSNP22658	*Kabuli*	Ca_Kabuli_denovo	18753052	(A/G)
22659	CakSNP22659	*Kabuli*	Ca_Kabuli_denovo	18756362	(C/T)
22660	CakSNP22660	*Kabuli*	Ca_Kabuli_denovo	18762726	(G/A)
22661	CakSNP22661	*Kabuli*	Ca_Kabuli_denovo	18762744	(C/A)
22662	CakSNP22662	*Kabuli*	Ca_Kabuli_denovo	18763792	(G/T)
22663	CakSNP22663	*Kabuli*	Ca_Kabuli_denovo	18770479	(C/T)
22664	CakSNP22664	*Kabuli*	Ca_Kabuli_denovo	18770536	(T/C)
22665	CakSNP22665	*Kabuli*	Ca_Kabuli_denovo	18770586	(C/T)
22666	CakSNP22666	*Kabuli*	Ca_Kabuli_denovo	18773521	(G/A)
22667	CakSNP22667	*Kabuli*	Ca_Kabuli_denovo	18775835	(T/C)
22668	CakSNP22668	*Kabuli*	Ca_Kabuli_denovo	18775837	(A/G)
22669	CakSNP22669	*Kabuli*	Ca_Kabuli_denovo	18777081	(C/G)
22670	CakSNP22670	*Kabuli*	Ca_Kabuli_denovo	18787985	(C/G)
22671	CakSNP22671	*Kabuli*	Ca_Kabuli_denovo	18788003	(T/C)
22672	CakSNP22672	*Kabuli*	Ca_Kabuli_denovo	18790370	(A/C)
22673	CakSNP22673	*Kabuli*	Ca_Kabuli_denovo	18791098	(C/T)
22674	CakSNP22674	*Kabuli*	Ca_Kabuli_denovo	18791101	(C/A)
22675	CakSNP22675	*Kabuli*	Ca_Kabuli_denovo	18793121	(T/C)
22676	CakSNP22676	*Kabuli*	Ca_Kabuli_denovo	18803897	(A/G)
22677	CakSNP22677	*Kabuli*	Ca_Kabuli_denovo	18803935	(G/C)
22678	CakSNP22678	*Kabuli*	Ca_Kabuli_denovo	18806483	(C/T)
22679	CakSNP22679	*Kabuli*	Ca_Kabuli_denovo	18808206	(T/G)
22680	CakSNP22680	*Kabuli*	Ca_Kabuli_denovo	18808227	(T/G)
22681	CakSNP22681	*Kabuli*	Ca_Kabuli_denovo	18809542	(C/T)
22682	CakSNP22682	*Kabuli*	Ca_Kabuli_denovo	18812003	(G/T)
22683	CakSNP22683	*Kabuli*	Ca_Kabuli_denovo	18813549	(G/A)
22684	CakSNP22684	*Kabuli*	Ca_Kabuli_denovo	18814122	(G/A)
22685	CakSNP22685	*Kabuli*	Ca_Kabuli_denovo	18816705	(T/C)

S.N.	SNP IDs	Cultivars	Chromosomes/scaffolds	Physical positions (bp)	SNPs
22686	CakSNP22686	*Kabuli*	Ca_Kabuli_denovo	18816713	(G/A)
22687	CakSNP22687	*Kabuli*	Ca_Kabuli_denovo	18828492	(G/A)
22688	CakSNP22688	*Kabuli*	Ca_Kabuli_denovo	18829915	(C/A)
22689	CakSNP22689	*Kabuli*	Ca_Kabuli_denovo	18829924	(A/G)
22690	CakSNP22690	*Kabuli*	Ca_Kabuli_denovo	18832361	(G/T)
22691	CakSNP22691	*Kabuli*	Ca_Kabuli_denovo	18832368	(G/C)
22692	CakSNP22692	*Kabuli*	Ca_Kabuli_denovo	18834096	(C/G)
22693	CakSNP22693	*Kabuli*	Ca_Kabuli_denovo	18835613	(T/C)
22694	CakSNP22694	*Kabuli*	Ca_Kabuli_denovo	18836177	(C/T)
22695	CakSNP22695	*Kabuli*	Ca_Kabuli_denovo	18841637	(A/G)
22696	CakSNP22696	*Kabuli*	Ca_Kabuli_denovo	18841640	(T/G)
22697	CakSNP22697	*Kabuli*	Ca_Kabuli_denovo	18849251	(G/A)
22698	CakSNP22698	*Kabuli*	Ca_Kabuli_denovo	18850197	(A/G)
22699	CakSNP22699	*Kabuli*	Ca_Kabuli_denovo	18850203	(C/T)
22700	CakSNP22700	*Kabuli*	Ca_Kabuli_denovo	18850655	(G/A)
22701	CakSNP22701	*Kabuli*	Ca_Kabuli_denovo	18850691	(G/C)
22702	CakSNP22702	*Kabuli*	Ca_Kabuli_denovo	18852209	(A/G)
22703	CakSNP22703	*Kabuli*	Ca_Kabuli_denovo	18852950	(A/G)
22704	CakSNP22704	*Kabuli*	Ca_Kabuli_denovo	18856357	(T/C)
22705	CakSNP22705	*Kabuli*	Ca_Kabuli_denovo	18857764	(C/T)
22706	CakSNP22706	*Kabuli*	Ca_Kabuli_denovo	18857773	(T/C)
22707	CakSNP22707	*Kabuli*	Ca_Kabuli_denovo	18860305	(A/G)
22708	CakSNP22708	*Kabuli*	Ca_Kabuli_denovo	18867206	(A/G)
22709	CakSNP22709	*Kabuli*	Ca_Kabuli_denovo	18869392	(G/A)
22710	CakSNP22710	*Kabuli*	Ca_Kabuli_denovo	18869409	(A/C)
22711	CakSNP22711	*Kabuli*	Ca_Kabuli_denovo	18872425	(C/T)
22712	CakSNP22712	*Kabuli*	Ca_Kabuli_denovo	18872431	(T/C)
22713	CakSNP22713	*Kabuli*	Ca_Kabuli_denovo	18872443	(T/C)
22714	CakSNP22714	*Kabuli*	Ca_Kabuli_denovo	18873043	(G/A)
22715	CakSNP22715	*Kabuli*	Ca_Kabuli_denovo	18873726	(T/C)
22716	CakSNP22716	*Kabuli*	Ca_Kabuli_denovo	18873766	(G/A)
22717	CakSNP22717	*Kabuli*	Ca_Kabuli_denovo	18878910	(T/A)
22718	CakSNP22718	*Kabuli*	Ca_Kabuli_denovo	18879388	(G/T)
22719	CakSNP22719	*Kabuli*	Ca_Kabuli_denovo	18880593	(T/C)
22720	CakSNP22720	*Kabuli*	Ca_Kabuli_denovo	18881435	(T/G)
22721	CakSNP22721	*Kabuli*	Ca_Kabuli_denovo	18882519	(T/C)
22722	CakSNP22722	*Kabuli*	Ca_Kabuli_denovo	18891748	(T/G)
22723	CakSNP22723	*Kabuli*	Ca_Kabuli_denovo	18891754	(T/C)

S.N.	SNP IDs	Cultivars	Chromosomes/scaffolds	Physical positions (bp)	SNPs
22724	CakSNP22724	*Kabuli*	Ca_Kabuli_denovo	18892953	(A/T)
22725	CakSNP22725	*Kabuli*	Ca_Kabuli_denovo	18896152	(A/G)
22726	CakSNP22726	*Kabuli*	Ca_Kabuli_denovo	18898353	(C/A)
22727	CakSNP22727	*Kabuli*	Ca_Kabuli_denovo	18898800	(C/T)
22728	CakSNP22728	*Kabuli*	Ca_Kabuli_denovo	18899861	(A/G)
22729	CakSNP22729	*Kabuli*	Ca_Kabuli_denovo	18899909	(C/G)
22730	CakSNP22730	*Kabuli*	Ca_Kabuli_denovo	18900482	(A/C)
22731	CakSNP22731	*Kabuli*	Ca_Kabuli_denovo	18900483	(G/T)
22732	CakSNP22732	*Kabuli*	Ca_Kabuli_denovo	18900484	(A/G)
22733	CakSNP22733	*Kabuli*	Ca_Kabuli_denovo	18905479	(G/A)
22734	CakSNP22734	*Kabuli*	Ca_Kabuli_denovo	18911372	(C/G)
22735	CakSNP22735	*Kabuli*	Ca_Kabuli_denovo	18911381	(A/G)
22736	CakSNP22736	*Kabuli*	Ca_Kabuli_denovo	18924368	(C/A)
22737	CakSNP22737	*Kabuli*	Ca_Kabuli_denovo	18927874	(G/C)
22738	CakSNP22738	*Kabuli*	Ca_Kabuli_denovo	18930752	(A/G)
22739	CakSNP22739	*Kabuli*	Ca_Kabuli_denovo	18930761	(T/C)
22740	CakSNP22740	*Kabuli*	Ca_Kabuli_denovo	18930885	(A/G)
22741	CakSNP22741	*Kabuli*	Ca_Kabuli_denovo	18930894	(A/G)
22742	CakSNP22742	*Kabuli*	Ca_Kabuli_denovo	18934921	(C/T)
22743	CakSNP22743	*Kabuli*	Ca_Kabuli_denovo	18934964	(C/T)
22744	CakSNP22744	*Kabuli*	Ca_Kabuli_denovo	18935278	(C/T)
22745	CakSNP22745	*Kabuli*	Ca_Kabuli_denovo	18938911	(G/A)
22746	CakSNP22746	*Kabuli*	Ca_Kabuli_denovo	18938964	(G/C)
22747	CakSNP22747	*Kabuli*	Ca_Kabuli_denovo	18940227	(G/C)
22748	CakSNP22748	*Kabuli*	Ca_Kabuli_denovo	18949813	(C/T)
22749	CakSNP22749	*Kabuli*	Ca_Kabuli_denovo	18951660	(G/C)
22750	CakSNP22750	*Kabuli*	Ca_Kabuli_denovo	18951699	(G/C)
22751	CakSNP22751	*Kabuli*	Ca_Kabuli_denovo	18951736	(A/G)
22752	CakSNP22752	*Kabuli*	Ca_Kabuli_denovo	18951760	(A/G)
22753	CakSNP22753	*Kabuli*	Ca_Kabuli_denovo	18968953	(G/C)
22754	CakSNP22754	*Kabuli*	Ca_Kabuli_denovo	18969406	(A/G)
22755	CakSNP22755	*Kabuli*	Ca_Kabuli_denovo	18969451	(A/G)
22756	CakSNP22756	*Kabuli*	Ca_Kabuli_denovo	18975086	(G/A)
22757	CakSNP22757	*Kabuli*	Ca_Kabuli_denovo	18975094	(T/C)
22758	CakSNP22758	*Kabuli*	Ca_Kabuli_denovo	18975107	(T/C)
22759	CakSNP22759	*Kabuli*	Ca_Kabuli_denovo	18975761	(T/C)
22760	CakSNP22760	*Kabuli*	Ca_Kabuli_denovo	18975813	(A/G)
22761	CakSNP22761	*Kabuli*	Ca_Kabuli_denovo	18976289	(G/A)

S.N.	SNP IDs	Cultivars	Chromosomes/scaffolds	Physical positions (bp)	SNPs
22762	CakSNP22762	*Kabuli*	Ca_Kabuli_denovo	18976292	(G/A)
22763	CakSNP22763	*Kabuli*	Ca_Kabuli_denovo	18977055	(C/G)
22764	CakSNP22764	*Kabuli*	Ca_Kabuli_denovo	18978483	(T/C)
22765	CakSNP22765	*Kabuli*	Ca_Kabuli_denovo	18981320	(T/C)
22766	CakSNP22766	*Kabuli*	Ca_Kabuli_denovo	18987252	(G/T)
22767	CakSNP22767	*Kabuli*	Ca_Kabuli_denovo	18992585	(G/A)
22768	CakSNP22768	*Kabuli*	Ca_Kabuli_denovo	18997411	(T/C)
22769	CakSNP22769	*Kabuli*	Ca_Kabuli_denovo	19005025	(A/C)
22770	CakSNP22770	*Kabuli*	Ca_Kabuli_denovo	19005067	(G/T)
22771	CakSNP22771	*Kabuli*	Ca_Kabuli_denovo	19006159	(T/C)
22772	CakSNP22772	*Kabuli*	Ca_Kabuli_denovo	19008722	(A/T)
22773	CakSNP22773	*Kabuli*	Ca_Kabuli_denovo	19010698	(T/C)
22774	CakSNP22774	*Kabuli*	Ca_Kabuli_denovo	19010774	(G/A)
22775	CakSNP22775	*Kabuli*	Ca_Kabuli_denovo	19016555	(G/A)
22776	CakSNP22776	*Kabuli*	Ca_Kabuli_denovo	19016573	(A/G)
22777	CakSNP22777	*Kabuli*	Ca_Kabuli_denovo	19017747	(T/G)
22778	CakSNP22778	*Kabuli*	Ca_Kabuli_denovo	19019311	(C/G)
22779	CakSNP22779	*Kabuli*	Ca_Kabuli_denovo	19026033	(A/C)
22780	CakSNP22780	*Kabuli*	Ca_Kabuli_denovo	19026034	(G/C)
22781	CakSNP22781	*Kabuli*	Ca_Kabuli_denovo	19032739	(G/A)
22782	CakSNP22782	*Kabuli*	Ca_Kabuli_denovo	19034113	(A/T)
22783	CakSNP22783	*Kabuli*	Ca_Kabuli_denovo	19040086	(T/C)
22784	CakSNP22784	*Kabuli*	Ca_Kabuli_denovo	19040170	(G/T)
22785	CakSNP22785	*Kabuli*	Ca_Kabuli_denovo	19045310	(G/A)
22786	CakSNP22786	*Kabuli*	Ca_Kabuli_denovo	19049477	(T/C)
22787	CakSNP22787	*Kabuli*	Ca_Kabuli_denovo	19050458	(A/T)
22788	CakSNP22788	*Kabuli*	Ca_Kabuli_denovo	19056692	(A/G)
22789	CakSNP22789	*Kabuli*	Ca_Kabuli_denovo	19056836	(C/T)
22790	CakSNP22790	*Kabuli*	Ca_Kabuli_denovo	19056854	(G/C)
22791	CakSNP22791	*Kabuli*	Ca_Kabuli_denovo	19063347	(C/G)
22792	CakSNP22792	*Kabuli*	Ca_Kabuli_denovo	19066239	(C/A)
22793	CakSNP22793	*Kabuli*	Ca_Kabuli_denovo	19069211	(T/C)
22794	CakSNP22794	*Kabuli*	Ca_Kabuli_denovo	19069247	(A/G)
22795	CakSNP22795	*Kabuli*	Ca_Kabuli_denovo	19069451	(T/C)
22796	CakSNP22796	*Kabuli*	Ca_Kabuli_denovo	19079071	(C/T)
22797	CakSNP22797	*Kabuli*	Ca_Kabuli_denovo	19079086	(C/G)
22798	CakSNP22798	*Kabuli*	Ca_Kabuli_denovo	19087156	(T/G)
22799	CakSNP22799	*Kabuli*	Ca_Kabuli_denovo	19088934	(C/G)

S.N.	SNP IDs	Cultivars	Chromosomes/scaffolds	Physical positions (bp)	SNPs
22800	CakSNP22800	*Kabuli*	Ca_Kabuli_denovo	19088942	(C/T)
22801	CakSNP22801	*Kabuli*	Ca_Kabuli_denovo	19089410	(G/A)
22802	CakSNP22802	*Kabuli*	Ca_Kabuli_denovo	19089454	(G/T)
22803	CakSNP22803	*Kabuli*	Ca_Kabuli_denovo	19090604	(G/A)
22804	CakSNP22804	*Kabuli*	Ca_Kabuli_denovo	19091010	(T/C)
22805	CakSNP22805	*Kabuli*	Ca_Kabuli_denovo	19091040	(A/C)
22806	CakSNP22806	*Kabuli*	Ca_Kabuli_denovo	19091083	(T/C)
22807	CakSNP22807	*Kabuli*	Ca_Kabuli_denovo	19093687	(G/T)
22808	CakSNP22808	*Kabuli*	Ca_Kabuli_denovo	19096458	(A/G)
22809	CakSNP22809	*Kabuli*	Ca_Kabuli_denovo	19108084	(G/A)
22810	CakSNP22810	*Kabuli*	Ca_Kabuli_denovo	19108134	(G/A)
22811	CakSNP22811	*Kabuli*	Ca_Kabuli_denovo	19108151	(G/C)
22812	CakSNP22812	*Kabuli*	Ca_Kabuli_denovo	19110319	(C/T)
22813	CakSNP22813	*Kabuli*	Ca_Kabuli_denovo	19110332	(A/G)
22814	CakSNP22814	*Kabuli*	Ca_Kabuli_denovo	19110398	(C/A)
22815	CakSNP22815	*Kabuli*	Ca_Kabuli_denovo	19126783	(T/A)
22816	CakSNP22816	*Kabuli*	Ca_Kabuli_denovo	19132339	(A/C)
22817	CakSNP22817	*Kabuli*	Ca_Kabuli_denovo	19132396	(T/C)
22818	CakSNP22818	*Kabuli*	Ca_Kabuli_denovo	19135975	(G/A)
22819	CakSNP22819	*Kabuli*	Ca_Kabuli_denovo	19136023	(A/G)
22820	CakSNP22820	*Kabuli*	Ca_Kabuli_denovo	19138028	(T/C)
22821	CakSNP22821	*Kabuli*	Ca_Kabuli_denovo	19138056	(G/A)
22822	CakSNP22822	*Kabuli*	Ca_Kabuli_denovo	19147683	(A/G)
22823	CakSNP22823	*Kabuli*	Ca_Kabuli_denovo	19147701	(T/C)
22824	CakSNP22824	*Kabuli*	Ca_Kabuli_denovo	19149268	(C/T)
22825	CakSNP22825	*Kabuli*	Ca_Kabuli_denovo	19154228	(G/A)
22826	CakSNP22826	*Kabuli*	Ca_Kabuli_denovo	19154258	(A/G)
22827	CakSNP22827	*Kabuli*	Ca_Kabuli_denovo	19155218	(T/C)
22828	CakSNP22828	*Kabuli*	Ca_Kabuli_denovo	19155611	(A/G)
22829	CakSNP22829	*Kabuli*	Ca_Kabuli_denovo	19155614	(G/A)
22830	CakSNP22830	*Kabuli*	Ca_Kabuli_denovo	19156125	(T/C)
22831	CakSNP22831	*Kabuli*	Ca_Kabuli_denovo	19158322	(T/C)
22832	CakSNP22832	*Kabuli*	Ca_Kabuli_denovo	19160518	(C/T)
22833	CakSNP22833	*Kabuli*	Ca_Kabuli_denovo	19160524	(T/A)
22834	CakSNP22834	*Kabuli*	Ca_Kabuli_denovo	19161853	(C/A)
22835	CakSNP22835	*Kabuli*	Ca_Kabuli_denovo	19163941	(C/G)
22836	CakSNP22836	*Kabuli*	Ca_Kabuli_denovo	19169207	(G/T)
22837	CakSNP22837	*Kabuli*	Ca_Kabuli_denovo	19169216	(C/T)

**S.N.**	**SNP IDs**	**Cultivars**	**Chromosomes/scaffolds**	**Physical positions (bp)**	**SNPs**
22838	CakSNP22838	*Kabuli*	Ca_Kabuli_denovo	19171720	(G/A)
22839	CakSNP22839	*Kabuli*	Ca_Kabuli_denovo	19171726	(A/G)
22840	CakSNP22840	*Kabuli*	Ca_Kabuli_denovo	19177383	(C/A)
22841	CakSNP22841	*Kabuli*	Ca_Kabuli_denovo	19177386	(C/A)
22842	CakSNP22842	*Kabuli*	Ca_Kabuli_denovo	19184471	(C/T)
22843	CakSNP22843	*Kabuli*	Ca_Kabuli_denovo	19185765	(A/G)
22844	CakSNP22844	*Kabuli*	Ca_Kabuli_denovo	19185789	(A/G)
22845	CakSNP22845	*Kabuli*	Ca_Kabuli_denovo	19191108	(A/C)
22846	CakSNP22846	*Kabuli*	Ca_Kabuli_denovo	19191109	(A/G)
22847	CakSNP22847	*Kabuli*	Ca_Kabuli_denovo	19192438	(G/A)
22848	CakSNP22848	*Kabuli*	Ca_Kabuli_denovo	19192486	(C/T)
22849	CakSNP22849	*Kabuli*	Ca_Kabuli_denovo	19195741	(A/G)
22850	CakSNP22850	*Kabuli*	Ca_Kabuli_denovo	19195744	(G/A)
22851	CakSNP22851	*Kabuli*	Ca_Kabuli_denovo	19198315	(T/C)
22852	CakSNP22852	*Kabuli*	Ca_Kabuli_denovo	19198343	(T/G)
22853	CakSNP22853	*Kabuli*	Ca_Kabuli_denovo	19200266	(A/G)
22854	CakSNP22854	*Kabuli*	Ca_Kabuli_denovo	19201724	(T/G)
22855	CakSNP22855	*Kabuli*	Ca_Kabuli_denovo	19206320	(T/C)
22856	CakSNP22856	*Kabuli*	Ca_Kabuli_denovo	19223412	(C/T)
22857	CakSNP22857	*Kabuli*	Ca_Kabuli_denovo	19223413	(G/T)
22858	CakSNP22858	*Kabuli*	Ca_Kabuli_denovo	19229621	(A/G)
22859	CakSNP22859	*Kabuli*	Ca_Kabuli_denovo	19234774	(T/C)
22860	CakSNP22860	*Kabuli*	Ca_Kabuli_denovo	19238116	(A/C)
22861	CakSNP22861	*Kabuli*	Ca_Kabuli_denovo	19238124	(C/G)
22862	CakSNP22862	*Kabuli*	Ca_Kabuli_denovo	19238946	(C/T)
22863	CakSNP22863	*Kabuli*	Ca_Kabuli_denovo	19240781	(T/C)
22864	CakSNP22864	*Kabuli*	Ca_Kabuli_denovo	19240800	(G/A)
22865	CakSNP22865	*Kabuli*	Ca_Kabuli_denovo	19247383	(G/C)
22866	CakSNP22866	*Kabuli*	Ca_Kabuli_denovo	19248118	(A/C)
22867	CakSNP22867	*Kabuli*	Ca_Kabuli_denovo	19251244	(C/T)
22868	CakSNP22868	*Kabuli*	Ca_Kabuli_denovo	19254548	(C/A)
22869	CakSNP22869	*Kabuli*	Ca_Kabuli_denovo	19254587	(A/G)
22870	CakSNP22870	*Kabuli*	Ca_Kabuli_denovo	19256586	(C/T)
22871	CakSNP22871	*Kabuli*	Ca_Kabuli_denovo	19274063	(T/G)
22872	CakSNP22872	*Kabuli*	Ca_Kabuli_denovo	19275686	(C/T)
22873	CakSNP22873	*Kabuli*	Ca_Kabuli_denovo	19275704	(T/C)
22874	CakSNP22874	*Kabuli*	Ca_Kabuli_denovo	19279916	(T/G)
22875	CakSNP22875	*Kabuli*	Ca_Kabuli_denovo	19284224	(A/G)

S.N.	SNP IDs	Cultivars	Chromosomes/scaffolds	Physical positions (bp)	SNPs
22876	CakSNP22876	*Kabuli*	Ca_Kabuli_denovo	19284257	(G/C)
22877	CakSNP22877	*Kabuli*	Ca_Kabuli_denovo	19287074	(C/T)
22878	CakSNP22878	*Kabuli*	Ca_Kabuli_denovo	19294446	(G/A)
22879	CakSNP22879	*Kabuli*	Ca_Kabuli_denovo	19294650	(G/A)
22880	CakSNP22880	*Kabuli*	Ca_Kabuli_denovo	19295751	(T/C)
22881	CakSNP22881	*Kabuli*	Ca_Kabuli_denovo	19302256	(A/G)
22882	CakSNP22882	*Kabuli*	Ca_Kabuli_denovo	19303277	(T/C)
22883	CakSNP22883	*Kabuli*	Ca_Kabuli_denovo	19304858	(T/C)
22884	CakSNP22884	*Kabuli*	Ca_Kabuli_denovo	19304894	(T/C)
22885	CakSNP22885	*Kabuli*	Ca_Kabuli_denovo	19304897	(C/T)
22886	CakSNP22886	*Kabuli*	Ca_Kabuli_denovo	19315474	(G/A)
22887	CakSNP22887	*Kabuli*	Ca_Kabuli_denovo	19315480	(G/A)
22888	CakSNP22888	*Kabuli*	Ca_Kabuli_denovo	19323757	(A/G)
22889	CakSNP22889	*Kabuli*	Ca_Kabuli_denovo	19328354	(C/A)
22890	CakSNP22890	*Kabuli*	Ca_Kabuli_denovo	19331804	(G/A)
22891	CakSNP22891	*Kabuli*	Ca_Kabuli_denovo	19332683	(G/T)
22892	CakSNP22892	*Kabuli*	Ca_Kabuli_denovo	19335471	(C/G)
22893	CakSNP22893	*Kabuli*	Ca_Kabuli_denovo	19346404	(T/C)
22894	CakSNP22894	*Kabuli*	Ca_Kabuli_denovo	19348192	(A/C)
22895	CakSNP22895	*Kabuli*	Ca_Kabuli_denovo	19348233	(C/G)
22896	CakSNP22896	*Kabuli*	Ca_Kabuli_denovo	19349645	(T/G)
22897	CakSNP22897	*Kabuli*	Ca_Kabuli_denovo	19350085	(G/A)
22898	CakSNP22898	*Kabuli*	Ca_Kabuli_denovo	19352147	(G/A)
22899	CakSNP22899	*Kabuli*	Ca_Kabuli_denovo	19356539	(C/T)
22900	CakSNP22900	*Kabuli*	Ca_Kabuli_denovo	19361046	(A/G)
22901	CakSNP22901	*Kabuli*	Ca_Kabuli_denovo	19367179	(T/C)
22902	CakSNP22902	*Kabuli*	Ca_Kabuli_denovo	19370551	(T/C)
22903	CakSNP22903	*Kabuli*	Ca_Kabuli_denovo	19374728	(A/G)
22904	CakSNP22904	*Kabuli*	Ca_Kabuli_denovo	19376336	(G/A)
22905	CakSNP22905	*Kabuli*	Ca_Kabuli_denovo	19376363	(G/A)
22906	CakSNP22906	*Kabuli*	Ca_Kabuli_denovo	19379073	(C/T)
22907	CakSNP22907	*Kabuli*	Ca_Kabuli_denovo	19379306	(C/A)
22908	CakSNP22908	*Kabuli*	Ca_Kabuli_denovo	19379735	(T/C)
22909	CakSNP22909	*Kabuli*	Ca_Kabuli_denovo	19385230	(C/G)
22910	CakSNP22910	*Kabuli*	Ca_Kabuli_denovo	19385511	(G/C)
22911	CakSNP22911	*Kabuli*	Ca_Kabuli_denovo	19385523	(A/G)
22912	CakSNP22912	*Kabuli*	Ca_Kabuli_denovo	19390162	(C/T)
22913	CakSNP22913	*Kabuli*	Ca_Kabuli_denovo	19390333	(T/C)

S.N.	SNP IDs	Cultivars	Chromosomes/scaffolds	Physical positions (bp)	SNPs
22914	CakSNP22914	*Kabuli*	Ca_Kabuli_denovo	19391252	(C/T)
22915	CakSNP22915	*Kabuli*	Ca_Kabuli_denovo	19398054	(C/A)
22916	CakSNP22916	*Kabuli*	Ca_Kabuli_denovo	19398085	(T/C)
22917	CakSNP22917	*Kabuli*	Ca_Kabuli_denovo	19398318	(G/A)
22918	CakSNP22918	*Kabuli*	Ca_Kabuli_denovo	19400571	(C/T)
22919	CakSNP22919	*Kabuli*	Ca_Kabuli_denovo	19401445	(G/C)
22920	CakSNP22920	*Kabuli*	Ca_Kabuli_denovo	19401475	(A/G)
22921	CakSNP22921	*Kabuli*	Ca_Kabuli_denovo	19402744	(C/G)
22922	CakSNP22922	*Kabuli*	Ca_Kabuli_denovo	19407853	(C/G)
22923	CakSNP22923	*Kabuli*	Ca_Kabuli_denovo	19416866	(C/T)
22924	CakSNP22924	*Kabuli*	Ca_Kabuli_denovo	19421553	(C/T)
22925	CakSNP22925	*Kabuli*	Ca_Kabuli_denovo	19421556	(A/G)
22926	CakSNP22926	*Kabuli*	Ca_Kabuli_denovo	19422615	(G/A)
22927	CakSNP22927	*Kabuli*	Ca_Kabuli_denovo	19422629	(G/A)
22928	CakSNP22928	*Kabuli*	Ca_Kabuli_denovo	19429950	(T/G)
22929	CakSNP22929	*Kabuli*	Ca_Kabuli_denovo	19429960	(G/A)
22930	CakSNP22930	*Kabuli*	Ca_Kabuli_denovo	19430010	(C/G)
22931	CakSNP22931	*Kabuli*	Ca_Kabuli_denovo	19430159	(G/A)
22932	CakSNP22932	*Kabuli*	Ca_Kabuli_denovo	19430182	(C/A)
22933	CakSNP22933	*Kabuli*	Ca_Kabuli_denovo	19432428	(G/T)
22934	CakSNP22934	*Kabuli*	Ca_Kabuli_denovo	19434335	(A/G)
22935	CakSNP22935	*Kabuli*	Ca_Kabuli_denovo	19435393	(G/T)
22936	CakSNP22936	*Kabuli*	Ca_Kabuli_denovo	19435406	(T/C)
22937	CakSNP22937	*Kabuli*	Ca_Kabuli_denovo	19446380	(G/C)
22938	CakSNP22938	*Kabuli*	Ca_Kabuli_denovo	19449849	(A/G)
22939	CakSNP22939	*Kabuli*	Ca_Kabuli_denovo	19459653	(A/G)
22940	CakSNP22940	*Kabuli*	Ca_Kabuli_denovo	19459666	(G/C)
22941	CakSNP22941	*Kabuli*	Ca_Kabuli_denovo	19461469	(A/G)
22942	CakSNP22942	*Kabuli*	Ca_Kabuli_denovo	19467652	(C/G)
22943	CakSNP22943	*Kabuli*	Ca_Kabuli_denovo	19482253	(T/C)
22944	CakSNP22944	*Kabuli*	Ca_Kabuli_denovo	19482283	(G/C)
22945	CakSNP22945	*Kabuli*	Ca_Kabuli_denovo	19490764	(A/G)
22946	CakSNP22946	*Kabuli*	Ca_Kabuli_denovo	19490794	(G/A)
22947	CakSNP22947	*Kabuli*	Ca_Kabuli_denovo	19494880	(C/T)
22948	CakSNP22948	*Kabuli*	Ca_Kabuli_denovo	19496641	(C/G)
22949	CakSNP22949	*Kabuli*	Ca_Kabuli_denovo	19496704	(C/T)
22950	CakSNP22950	*Kabuli*	Ca_Kabuli_denovo	19497856	(G/A)
22951	CakSNP22951	*Kabuli*	Ca_Kabuli_denovo	19497910	(C/T)

S.N.	SNP IDs	Cultivars	Chromosomes/scaffolds	Physical positions (bp)	SNPs
22952	CakSNP22952	*Kabuli*	Ca_Kabuli_denovo	19499330	(G/T)
22953	CakSNP22953	*Kabuli*	Ca_Kabuli_denovo	19499333	(A/G)
22954	CakSNP22954	*Kabuli*	Ca_Kabuli_denovo	19500292	(C/T)
22955	CakSNP22955	*Kabuli*	Ca_Kabuli_denovo	19500454	(A/G)
22956	CakSNP22956	*Kabuli*	Ca_Kabuli_denovo	19500513	(G/A)
22957	CakSNP22957	*Kabuli*	Ca_Kabuli_denovo	19504817	(C/T)
22958	CakSNP22958	*Kabuli*	Ca_Kabuli_denovo	19504856	(T/G)
22959	CakSNP22959	*Kabuli*	Ca_Kabuli_denovo	19505980	(A/G)
22960	CakSNP22960	*Kabuli*	Ca_Kabuli_denovo	19508060	(C/T)
22961	CakSNP22961	*Kabuli*	Ca_Kabuli_denovo	19508077	(G/T)
22962	CakSNP22962	*Kabuli*	Ca_Kabuli_denovo	19508114	(A/G)
22963	CakSNP22963	*Kabuli*	Ca_Kabuli_denovo	19509112	(C/A)
22964	CakSNP22964	*Kabuli*	Ca_Kabuli_denovo	19515877	(T/C)
22965	CakSNP22965	*Kabuli*	Ca_Kabuli_denovo	19519198	(A/G)
22966	CakSNP22966	*Kabuli*	Ca_Kabuli_denovo	19519203	(G/A)
22967	CakSNP22967	*Kabuli*	Ca_Kabuli_denovo	19521213	(C/G)
22968	CakSNP22968	*Kabuli*	Ca_Kabuli_denovo	19521958	(T/A)
22969	CakSNP22969	*Kabuli*	Ca_Kabuli_denovo	19521965	(A/G)
22970	CakSNP22970	*Kabuli*	Ca_Kabuli_denovo	19525451	(G/A)
22971	CakSNP22971	*Kabuli*	Ca_Kabuli_denovo	19526859	(G/C)
22972	CakSNP22972	*Kabuli*	Ca_Kabuli_denovo	19526871	(T/C)
22973	CakSNP22973	*Kabuli*	Ca_Kabuli_denovo	19532983	(T/C)
22974	CakSNP22974	*Kabuli*	Ca_Kabuli_denovo	19537330	(A/G)
22975	CakSNP22975	*Kabuli*	Ca_Kabuli_denovo	19547897	(G/A)
22976	CakSNP22976	*Kabuli*	Ca_Kabuli_denovo	19549075	(C/T)
22977	CakSNP22977	*Kabuli*	Ca_Kabuli_denovo	19549145	(A/G)
22978	CakSNP22978	*Kabuli*	Ca_Kabuli_denovo	19554267	(A/G)
22979	CakSNP22979	*Kabuli*	Ca_Kabuli_denovo	19558394	(C/T)
22980	CakSNP22980	*Kabuli*	Ca_Kabuli_denovo	19558883	(A/G)
22981	CakSNP22981	*Kabuli*	Ca_Kabuli_denovo	19559530	(T/C)
22982	CakSNP22982	*Kabuli*	Ca_Kabuli_denovo	19569592	(C/G)
22983	CakSNP22983	*Kabuli*	Ca_Kabuli_denovo	19578021	(C/T)
22984	CakSNP22984	*Kabuli*	Ca_Kabuli_denovo	19580031	(C/G)
22985	CakSNP22985	*Kabuli*	Ca_Kabuli_denovo	19583318	(G/A)
22986	CakSNP22986	*Kabuli*	Ca_Kabuli_denovo	19584237	(C/G)
22987	CakSNP22987	*Kabuli*	Ca_Kabuli_denovo	19592563	(A/C)
22988	CakSNP22988	*Kabuli*	Ca_Kabuli_denovo	19594005	(A/T)
22989	CakSNP22989	*Kabuli*	Ca_Kabuli_denovo	19594048	(G/C)

S.N.	SNP IDs	Cultivars	Chromosomes/scaffolds	Physical positions (bp)	SNPs
22990	CakSNP22990	*Kabuli*	Ca_Kabuli_denovo	19596700	(C/G)
22991	CakSNP22991	*Kabuli*	Ca_Kabuli_denovo	19596703	(G/A)
22992	CakSNP22992	*Kabuli*	Ca_Kabuli_denovo	19596726	(G/C)
22993	CakSNP22993	*Kabuli*	Ca_Kabuli_denovo	19599063	(G/A)
22994	CakSNP22994	*Kabuli*	Ca_Kabuli_denovo	19607491	(A/C)
22995	CakSNP22995	*Kabuli*	Ca_Kabuli_denovo	19607505	(G/C)
22996	CakSNP22996	*Kabuli*	Ca_Kabuli_denovo	19607787	(T/C)
22997	CakSNP22997	*Kabuli*	Ca_Kabuli_denovo	19607832	(G/A)
22998	CakSNP22998	*Kabuli*	Ca_Kabuli_denovo	19609996	(C/T)
22999	CakSNP22999	*Kabuli*	Ca_Kabuli_denovo	19610895	(C/G)
23000	CakSNP23000	*Kabuli*	Ca_Kabuli_denovo	19613068	(C/T)
23001	CakSNP23001	*Kabuli*	Ca_Kabuli_denovo	19629262	(A/C)
23002	CakSNP23002	*Kabuli*	Ca_Kabuli_denovo	19630852	(G/A)
23003	CakSNP23003	*Kabuli*	Ca_Kabuli_denovo	19632166	(T/C)
23004	CakSNP23004	*Kabuli*	Ca_Kabuli_denovo	19632919	(C/T)
23005	CakSNP23005	*Kabuli*	Ca_Kabuli_denovo	19634087	(T/C)
23006	CakSNP23006	*Kabuli*	Ca_Kabuli_denovo	19640222	(A/G)
23007	CakSNP23007	*Kabuli*	Ca_Kabuli_denovo	19640228	(C/T)
23008	CakSNP23008	*Kabuli*	Ca_Kabuli_denovo	19645993	(C/T)
23009	CakSNP23009	*Kabuli*	Ca_Kabuli_denovo	19652525	(G/A)
23010	CakSNP23010	*Kabuli*	Ca_Kabuli_denovo	19657004	(G/A)
23011	CakSNP23011	*Kabuli*	Ca_Kabuli_denovo	19658542	(T/C)
23012	CakSNP23012	*Kabuli*	Ca_Kabuli_denovo	19659001	(T/C)
23013	CakSNP23013	*Kabuli*	Ca_Kabuli_denovo	19661154	(C/G)
23014	CakSNP23014	*Kabuli*	Ca_Kabuli_denovo	19664674	(T/A)
23015	CakSNP23015	*Kabuli*	Ca_Kabuli_denovo	19664737	(T/G)
23016	CakSNP23016	*Kabuli*	Ca_Kabuli_denovo	19666531	(A/C)
23017	CakSNP23017	*Kabuli*	Ca_Kabuli_denovo	19666855	(A/G)
23018	CakSNP23018	*Kabuli*	Ca_Kabuli_denovo	19666861	(C/A)
23019	CakSNP23019	*Kabuli*	Ca_Kabuli_denovo	19670099	(G/C)
23020	CakSNP23020	*Kabuli*	Ca_Kabuli_denovo	19687054	(T/C)
23021	CakSNP23021	*Kabuli*	Ca_Kabuli_denovo	19692882	(G/T)
23022	CakSNP23022	*Kabuli*	Ca_Kabuli_denovo	19692883	(A/C)
23023	CakSNP23023	*Kabuli*	Ca_Kabuli_denovo	19694833	(A/G)
23024	CakSNP23024	*Kabuli*	Ca_Kabuli_denovo	19694872	(G/T)
23025	CakSNP23025	*Kabuli*	Ca_Kabuli_denovo	19695841	(C/T)
23026	CakSNP23026	*Kabuli*	Ca_Kabuli_denovo	19695891	(C/T)
23027	CakSNP23027	*Kabuli*	Ca_Kabuli_denovo	19699736	(G/A)

S.N.	SNP IDs	Cultivars	Chromosomes/scaffolds	Physical positions (bp)	SNPs
23028	CakSNP23028	*Kabuli*	Ca_Kabuli_denovo	19713310	(A/G)
23029	CakSNP23029	*Kabuli*	Ca_Kabuli_denovo	19713350	(T/C)
23030	CakSNP23030	*Kabuli*	Ca_Kabuli_denovo	19715189	(C/G)
23031	CakSNP23031	*Kabuli*	Ca_Kabuli_denovo	19715195	(C/T)
23032	CakSNP23032	*Kabuli*	Ca_Kabuli_denovo	19720566	(C/G)
23033	CakSNP23033	*Kabuli*	Ca_Kabuli_denovo	19720598	(T/A)
23034	CakSNP23034	*Kabuli*	Ca_Kabuli_denovo	19721704	(G/A)
23035	CakSNP23035	*Kabuli*	Ca_Kabuli_denovo	19721717	(C/T)
23036	CakSNP23036	*Kabuli*	Ca_Kabuli_denovo	19728777	(G/A)
23037	CakSNP23037	*Kabuli*	Ca_Kabuli_denovo	19728792	(A/G)
23038	CakSNP23038	*Kabuli*	Ca_Kabuli_denovo	19731087	(C/G)
23039	CakSNP23039	*Kabuli*	Ca_Kabuli_denovo	19734795	(G/A)
23040	CakSNP23040	*Kabuli*	Ca_Kabuli_denovo	19734828	(C/G)
23041	CakSNP23041	*Kabuli*	Ca_Kabuli_denovo	19738169	(A/C)
23042	CakSNP23042	*Kabuli*	Ca_Kabuli_denovo	19741005	(T/G)
23043	CakSNP23043	*Kabuli*	Ca_Kabuli_denovo	19744691	(C/T)
23044	CakSNP23044	*Kabuli*	Ca_Kabuli_denovo	19750612	(G/A)
23045	CakSNP23045	*Kabuli*	Ca_Kabuli_denovo	19756173	(A/G)
23046	CakSNP23046	*Kabuli*	Ca_Kabuli_denovo	19761671	(G/A)
23047	CakSNP23047	*Kabuli*	Ca_Kabuli_denovo	19761695	(A/T)
23048	CakSNP23048	*Kabuli*	Ca_Kabuli_denovo	19762343	(C/T)
23049	CakSNP23049	*Kabuli*	Ca_Kabuli_denovo	19762346	(T/C)
23050	CakSNP23050	*Kabuli*	Ca_Kabuli_denovo	19764134	(G/C)
23051	CakSNP23051	*Kabuli*	Ca_Kabuli_denovo	19769234	(G/T)
23052	CakSNP23052	*Kabuli*	Ca_Kabuli_denovo	19774332	(C/T)
23053	CakSNP23053	*Kabuli*	Ca_Kabuli_denovo	19776405	(T/G)
23054	CakSNP23054	*Kabuli*	Ca_Kabuli_denovo	19776889	(T/G)
23055	CakSNP23055	*Kabuli*	Ca_Kabuli_denovo	19781120	(C/T)
23056	CakSNP23056	*Kabuli*	Ca_Kabuli_denovo	19788782	(A/G)
23057	CakSNP23057	*Kabuli*	Ca_Kabuli_denovo	19788806	(C/G)
23058	CakSNP23058	*Kabuli*	Ca_Kabuli_denovo	19793263	(G/A)
23059	CakSNP23059	*Kabuli*	Ca_Kabuli_denovo	19794417	(A/G)
23060	CakSNP23060	*Kabuli*	Ca_Kabuli_denovo	19797482	(C/T)
23061	CakSNP23061	*Kabuli*	Ca_Kabuli_denovo	19797497	(T/C)
23062	CakSNP23062	*Kabuli*	Ca_Kabuli_denovo	19806614	(T/G)
23063	CakSNP23063	*Kabuli*	Ca_Kabuli_denovo	19806651	(G/C)
23064	CakSNP23064	*Kabuli*	Ca_Kabuli_denovo	19808095	(A/C)
23065	CakSNP23065	*Kabuli*	Ca_Kabuli_denovo	19808101	(A/G)

S.N.	SNP IDs	Cultivars	Chromosomes/scaffolds	Physical positions (bp)	SNPs
23066	CakSNP23066	*Kabuli*	Ca_Kabuli_denovo	19814186	(C/A)
23067	CakSNP23067	*Kabuli*	Ca_Kabuli_denovo	19825655	(A/G)
23068	CakSNP23068	*Kabuli*	Ca_Kabuli_denovo	19825684	(C/G)
23069	CakSNP23069	*Kabuli*	Ca_Kabuli_denovo	19831346	(T/C)
23070	CakSNP23070	*Kabuli*	Ca_Kabuli_denovo	19831394	(A/G)
23071	CakSNP23071	*Kabuli*	Ca_Kabuli_denovo	19833191	(C/T)
23072	CakSNP23072	*Kabuli*	Ca_Kabuli_denovo	19835711	(C/T)
23073	CakSNP23073	*Kabuli*	Ca_Kabuli_denovo	19837847	(T/C)
23074	CakSNP23074	*Kabuli*	Ca_Kabuli_denovo	19837862	(C/A)
23075	CakSNP23075	*Kabuli*	Ca_Kabuli_denovo	19838759	(T/C)
23076	CakSNP23076	*Kabuli*	Ca_Kabuli_denovo	19842335	(T/G)
23077	CakSNP23077	*Kabuli*	Ca_Kabuli_denovo	19842348	(G/A)
23078	CakSNP23078	*Kabuli*	Ca_Kabuli_denovo	19848450	(G/C)
23079	CakSNP23079	*Kabuli*	Ca_Kabuli_denovo	19848471	(T/C)
23080	CakSNP23080	*Kabuli*	Ca_Kabuli_denovo	19852642	(C/T)
23081	CakSNP23081	*Kabuli*	Ca_Kabuli_denovo	19852670	(C/G)
23082	CakSNP23082	*Kabuli*	Ca_Kabuli_denovo	19857655	(C/T)
23083	CakSNP23083	*Kabuli*	Ca_Kabuli_denovo	19857667	(T/C)
23084	CakSNP23084	*Kabuli*	Ca_Kabuli_denovo	19858311	(C/G)
23085	CakSNP23085	*Kabuli*	Ca_Kabuli_denovo	19858321	(G/C)
23086	CakSNP23086	*Kabuli*	Ca_Kabuli_denovo	19866905	(G/C)
23087	CakSNP23087	*Kabuli*	Ca_Kabuli_denovo	19873840	(C/A)
23088	CakSNP23088	*Kabuli*	Ca_Kabuli_denovo	19873946	(T/C)
23089	CakSNP23089	*Kabuli*	Ca_Kabuli_denovo	19875607	(T/C)
23090	CakSNP23090	*Kabuli*	Ca_Kabuli_denovo	19878301	(T/A)
23091	CakSNP23091	*Kabuli*	Ca_Kabuli_denovo	19878370	(C/T)
23092	CakSNP23092	*Kabuli*	Ca_Kabuli_denovo	19879644	(A/G)
23093	CakSNP23093	*Kabuli*	Ca_Kabuli_denovo	19879658	(C/T)
23094	CakSNP23094	*Kabuli*	Ca_Kabuli_denovo	19880110	(G/A)
23095	CakSNP23095	*Kabuli*	Ca_Kabuli_denovo	19888886	(T/G)
23096	CakSNP23096	*Kabuli*	Ca_Kabuli_denovo	19895426	(C/T)
23097	CakSNP23097	*Kabuli*	Ca_Kabuli_denovo	19895429	(C/T)
23098	CakSNP23098	*Kabuli*	Ca_Kabuli_denovo	19896219	(A/G)
23099	CakSNP23099	*Kabuli*	Ca_Kabuli_denovo	19897592	(A/G)
23100	CakSNP23100	*Kabuli*	Ca_Kabuli_denovo	19898670	(C/T)
23101	CakSNP23101	*Kabuli*	Ca_Kabuli_denovo	19900442	(G/A)
23102	CakSNP23102	*Kabuli*	Ca_Kabuli_denovo	19901441	(A/G)
23103	CakSNP23103	*Kabuli*	Ca_Kabuli_denovo	19904162	(G/C)

S.N.	SNP IDs	Cultivars	Chromosomes/scaffolds	Physical positions (bp)	SNPs
23104	CakSNP23104	*Kabuli*	Ca_Kabuli_denovo	19906133	(C/T)
23105	CakSNP23105	*Kabuli*	Ca_Kabuli_denovo	19906160	(C/G)
23106	CakSNP23106	*Kabuli*	Ca_Kabuli_denovo	19913298	(A/G)
23107	CakSNP23107	*Kabuli*	Ca_Kabuli_denovo	19913325	(T/G)
23108	CakSNP23108	*Kabuli*	Ca_Kabuli_denovo	19917542	(T/C)
23109	CakSNP23109	*Kabuli*	Ca_Kabuli_denovo	19917611	(T/G)
23110	CakSNP23110	*Kabuli*	Ca_Kabuli_denovo	19917912	(C/T)
23111	CakSNP23111	*Kabuli*	Ca_Kabuli_denovo	19917922	(G/A)
23112	CakSNP23112	*Kabuli*	Ca_Kabuli_denovo	19919217	(T/C)
23113	CakSNP23113	*Kabuli*	Ca_Kabuli_denovo	19919234	(G/C)
23114	CakSNP23114	*Kabuli*	Ca_Kabuli_denovo	19921011	(C/A)
23115	CakSNP23115	*Kabuli*	Ca_Kabuli_denovo	19921041	(A/G)
23116	CakSNP23116	*Kabuli*	Ca_Kabuli_denovo	19926525	(G/C)
23117	CakSNP23117	*Kabuli*	Ca_Kabuli_denovo	19931580	(C/A)
23118	CakSNP23118	*Kabuli*	Ca_Kabuli_denovo	19931658	(C/G)
23119	CakSNP23119	*Kabuli*	Ca_Kabuli_denovo	19937324	(G/A)
23120	CakSNP23120	*Kabuli*	Ca_Kabuli_denovo	19937333	(G/C)
23121	CakSNP23121	*Kabuli*	Ca_Kabuli_denovo	19938269	(C/T)
23122	CakSNP23122	*Kabuli*	Ca_Kabuli_denovo	19942267	(A/C)
23123	CakSNP23123	*Kabuli*	Ca_Kabuli_denovo	19945803	(G/T)
23124	CakSNP23124	*Kabuli*	Ca_Kabuli_denovo	19948365	(C/G)
23125	CakSNP23125	*Kabuli*	Ca_Kabuli_denovo	19948387	(C/A)
23126	CakSNP23126	*Kabuli*	Ca_Kabuli_denovo	19948522	(C/T)
23127	CakSNP23127	*Kabuli*	Ca_Kabuli_denovo	19948538	(T/A)
23128	CakSNP23128	*Kabuli*	Ca_Kabuli_denovo	19952071	(T/A)
23129	CakSNP23129	*Kabuli*	Ca_Kabuli_denovo	19953836	(C/T)
23130	CakSNP23130	*Kabuli*	Ca_Kabuli_denovo	19953839	(T/G)
23131	CakSNP23131	*Kabuli*	Ca_Kabuli_denovo	19954188	(C/G)
23132	CakSNP23132	*Kabuli*	Ca_Kabuli_denovo	19955948	(G/C)
23133	CakSNP23133	*Kabuli*	Ca_Kabuli_denovo	19956797	(C/T)
23134	CakSNP23134	*Kabuli*	Ca_Kabuli_denovo	19958097	(C/G)
23135	CakSNP23135	*Kabuli*	Ca_Kabuli_denovo	19958902	(A/G)
23136	CakSNP23136	*Kabuli*	Ca_Kabuli_denovo	19963409	(G/A)
23137	CakSNP23137	*Kabuli*	Ca_Kabuli_denovo	19968457	(G/A)
23138	CakSNP23138	*Kabuli*	Ca_Kabuli_denovo	19975434	(T/A)
23139	CakSNP23139	*Kabuli*	Ca_Kabuli_denovo	19975893	(G/A)
23140	CakSNP23140	*Kabuli*	Ca_Kabuli_denovo	19975905	(G/C)
23141	CakSNP23141	*Kabuli*	Ca_Kabuli_denovo	19984691	(C/T)

S.N.	SNP IDs	Cultivars	Chromosomes/scaffolds	Physical positions (bp)	SNPs
23142	CakSNP23142	*Kabuli*	Ca_Kabuli_denovo	19989240	(G/A)
23143	CakSNP23143	*Kabuli*	Ca_Kabuli_denovo	19989273	(A/G)
23144	CakSNP23144	*Kabuli*	Ca_Kabuli_denovo	19997189	(A/C)
23145	CakSNP23145	*Kabuli*	Ca_Kabuli_denovo	19997192	(C/G)
23146	CakSNP23146	*Kabuli*	Ca_Kabuli_denovo	19998340	(T/C)
23147	CakSNP23147	*Kabuli*	Ca_Kabuli_denovo	20000681	(G/A)
23148	CakSNP23148	*Kabuli*	Ca_Kabuli_denovo	20000697	(T/C)
23149	CakSNP23149	*Kabuli*	Ca_Kabuli_denovo	20000708	(T/C)
23150	CakSNP23150	*Kabuli*	Ca_Kabuli_denovo	20005079	(G/A)
23151	CakSNP23151	*Kabuli*	Ca_Kabuli_denovo	20017910	(C/G)
23152	CakSNP23152	*Kabuli*	Ca_Kabuli_denovo	20017928	(T/C)
23153	CakSNP23153	*Kabuli*	Ca_Kabuli_denovo	20018908	(C/G)
23154	CakSNP23154	*Kabuli*	Ca_Kabuli_denovo	20018920	(C/T)
23155	CakSNP23155	*Kabuli*	Ca_Kabuli_denovo	20024896	(C/G)
23156	CakSNP23156	*Kabuli*	Ca_Kabuli_denovo	20024947	(C/A)
23157	CakSNP23157	*Kabuli*	Ca_Kabuli_denovo	20025608	(A/G)
23158	CakSNP23158	*Kabuli*	Ca_Kabuli_denovo	20025654	(G/A)
23159	CakSNP23159	*Kabuli*	Ca_Kabuli_denovo	20026305	(T/C)
23160	CakSNP23160	*Kabuli*	Ca_Kabuli_denovo	20027451	(G/C)
23161	CakSNP23161	*Kabuli*	Ca_Kabuli_denovo	20035598	(C/T)
23162	CakSNP23162	*Kabuli*	Ca_Kabuli_denovo	20035633	(G/C)
23163	CakSNP23163	*Kabuli*	Ca_Kabuli_denovo	20038648	(G/C)
23164	CakSNP23164	*Kabuli*	Ca_Kabuli_denovo	20038704	(A/G)
23165	CakSNP23165	*Kabuli*	Ca_Kabuli_denovo	20040215	(T/A)
23166	CakSNP23166	*Kabuli*	Ca_Kabuli_denovo	20042344	(T/C)
23167	CakSNP23167	*Kabuli*	Ca_Kabuli_denovo	20044804	(C/G)
23168	CakSNP23168	*Kabuli*	Ca_Kabuli_denovo	20044831	(C/T)
23169	CakSNP23169	*Kabuli*	Ca_Kabuli_denovo	20048966	(G/A)
23170	CakSNP23170	*Kabuli*	Ca_Kabuli_denovo	20056233	(G/C)
23171	CakSNP23171	*Kabuli*	Ca_Kabuli_denovo	20063573	(G/A)
23172	CakSNP23172	*Kabuli*	Ca_Kabuli_denovo	20068026	(G/C)
23173	CakSNP23173	*Kabuli*	Ca_Kabuli_denovo	20069077	(C/G)
23174	CakSNP23174	*Kabuli*	Ca_Kabuli_denovo	20074843	(C/A)
23175	CakSNP23175	*Kabuli*	Ca_Kabuli_denovo	20078987	(G/T)
23176	CakSNP23176	*Kabuli*	Ca_Kabuli_denovo	20081620	(C/A)
23177	CakSNP23177	*Kabuli*	Ca_Kabuli_denovo	20083394	(T/A)
23178	CakSNP23178	*Kabuli*	Ca_Kabuli_denovo	20083396	(T/C)
23179	CakSNP23179	*Kabuli*	Ca_Kabuli_denovo	20085531	(T/C)

S.N.	SNP IDs	Cultivars	Chromosomes/scaffolds	Physical positions (bp)	SNPs
23180	CakSNP23180	*Kabuli*	Ca_Kabuli_denovo	20085546	(G/T)
23181	CakSNP23181	*Kabuli*	Ca_Kabuli_denovo	20087213	(T/C)
23182	CakSNP23182	*Kabuli*	Ca_Kabuli_denovo	20087214	(T/G)
23183	CakSNP23183	*Kabuli*	Ca_Kabuli_denovo	20101340	(C/G)
23184	CakSNP23184	*Kabuli*	Ca_Kabuli_denovo	20104647	(A/C)
23185	CakSNP23185	*Kabuli*	Ca_Kabuli_denovo	20104648	(G/C)
23186	CakSNP23186	*Kabuli*	Ca_Kabuli_denovo	20110626	(G/T)
23187	CakSNP23187	*Kabuli*	Ca_Kabuli_denovo	20110644	(C/T)
23188	CakSNP23188	*Kabuli*	Ca_Kabuli_denovo	20116483	(C/T)
23189	CakSNP23189	*Kabuli*	Ca_Kabuli_denovo	20119877	(A/G)
23190	CakSNP23190	*Kabuli*	Ca_Kabuli_denovo	20123545	(T/C)
23191	CakSNP23191	*Kabuli*	Ca_Kabuli_denovo	20123553	(A/G)
23192	CakSNP23192	*Kabuli*	Ca_Kabuli_denovo	20126594	(T/G)
23193	CakSNP23193	*Kabuli*	Ca_Kabuli_denovo	20126610	(G/C)
23194	CakSNP23194	*Kabuli*	Ca_Kabuli_denovo	20129287	(A/G)
23195	CakSNP23195	*Kabuli*	Ca_Kabuli_denovo	20129288	(A/T)
23196	CakSNP23196	*Kabuli*	Ca_Kabuli_denovo	20129305	(C/G)
23197	CakSNP23197	*Kabuli*	Ca_Kabuli_denovo	20131006	(G/A)
23198	CakSNP23198	*Kabuli*	Ca_Kabuli_denovo	20131170	(A/T)
23199	CakSNP23199	*Kabuli*	Ca_Kabuli_denovo	20134798	(A/C)
23200	CakSNP23200	*Kabuli*	Ca_Kabuli_denovo	20136511	(A/G)
23201	CakSNP23201	*Kabuli*	Ca_Kabuli_denovo	20137771	(G/A)
23202	CakSNP23202	*Kabuli*	Ca_Kabuli_denovo	20141248	(C/G)
23203	CakSNP23203	*Kabuli*	Ca_Kabuli_denovo	20142644	(G/A)
23204	CakSNP23204	*Kabuli*	Ca_Kabuli_denovo	20145625	(A/G)
23205	CakSNP23205	*Kabuli*	Ca_Kabuli_denovo	20145669	(C/G)
23206	CakSNP23206	*Kabuli*	Ca_Kabuli_denovo	20145687	(G/T)
23207	CakSNP23207	*Kabuli*	Ca_Kabuli_denovo	20150919	(C/T)
23208	CakSNP23208	*Kabuli*	Ca_Kabuli_denovo	20152100	(A/T)
23209	CakSNP23209	*Kabuli*	Ca_Kabuli_denovo	20156472	(T/C)
23210	CakSNP23210	*Kabuli*	Ca_Kabuli_denovo	20157125	(G/A)
23211	CakSNP23211	*Kabuli*	Ca_Kabuli_denovo	20157152	(T/C)
23212	CakSNP23212	*Kabuli*	Ca_Kabuli_denovo	20158810	(T/C)
23213	CakSNP23213	*Kabuli*	Ca_Kabuli_denovo	20159644	(T/C)
23214	CakSNP23214	*Kabuli*	Ca_Kabuli_denovo	20159683	(G/T)
23215	CakSNP23215	*Kabuli*	Ca_Kabuli_denovo	20161993	(T/C)
23216	CakSNP23216	*Kabuli*	Ca_Kabuli_denovo	20162803	(C/T)
23217	CakSNP23217	*Kabuli*	Ca_Kabuli_denovo	20162851	(C/T)

S.N.	SNP IDs	Cultivars	Chromosomes/scaffolds	Physical positions (bp)	SNPs
23218	CakSNP23218	*Kabuli*	Ca_Kabuli_denovo	20164653	(T/C)
23219	CakSNP23219	*Kabuli*	Ca_Kabuli_denovo	20164656	(G/A)
23220	CakSNP23220	*Kabuli*	Ca_Kabuli_denovo	20170406	(A/G)
23221	CakSNP23221	*Kabuli*	Ca_Kabuli_denovo	20170473	(A/C)
23222	CakSNP23222	*Kabuli*	Ca_Kabuli_denovo	20180858	(T/G)
23223	CakSNP23223	*Kabuli*	Ca_Kabuli_denovo	20194916	(G/C)
23224	CakSNP23224	*Kabuli*	Ca_Kabuli_denovo	20194934	(A/C)
23225	CakSNP23225	*Kabuli*	Ca_Kabuli_denovo	20196346	(T/C)
23226	CakSNP23226	*Kabuli*	Ca_Kabuli_denovo	20200393	(T/C)
23227	CakSNP23227	*Kabuli*	Ca_Kabuli_denovo	20202734	(A/T)
23228	CakSNP23228	*Kabuli*	Ca_Kabuli_denovo	20202761	(A/G)
23229	CakSNP23229	*Kabuli*	Ca_Kabuli_denovo	20212724	(A/G)
23230	CakSNP23230	*Kabuli*	Ca_Kabuli_denovo	20212763	(G/A)
23231	CakSNP23231	*Kabuli*	Ca_Kabuli_denovo	20219887	(G/A)
23232	CakSNP23232	*Kabuli*	Ca_Kabuli_denovo	20220084	(G/A)
23233	CakSNP23233	*Kabuli*	Ca_Kabuli_denovo	20220098	(C/G)
23234	CakSNP23234	*Kabuli*	Ca_Kabuli_denovo	20230591	(C/T)
23235	CakSNP23235	*Kabuli*	Ca_Kabuli_denovo	20230657	(C/T)
23236	CakSNP23236	*Kabuli*	Ca_Kabuli_denovo	20236388	(A/G)
23237	CakSNP23237	*Kabuli*	Ca_Kabuli_denovo	20240633	(T/G)
23238	CakSNP23238	*Kabuli*	Ca_Kabuli_denovo	20240645	(C/G)
23239	CakSNP23239	*Kabuli*	Ca_Kabuli_denovo	20241466	(T/C)
23240	CakSNP23240	*Kabuli*	Ca_Kabuli_denovo	20241469	(G/A)
23241	CakSNP23241	*Kabuli*	Ca_Kabuli_denovo	20244579	(G/A)
23242	CakSNP23242	*Kabuli*	Ca_Kabuli_denovo	20249119	(C/A)
23243	CakSNP23243	*Kabuli*	Ca_Kabuli_denovo	20249316	(T/C)
23244	CakSNP23244	*Kabuli*	Ca_Kabuli_denovo	20249645	(T/C)
23245	CakSNP23245	*Kabuli*	Ca_Kabuli_denovo	20249668	(C/A)
23246	CakSNP23246	*Kabuli*	Ca_Kabuli_denovo	20257527	(C/G)
23247	CakSNP23247	*Kabuli*	Ca_Kabuli_denovo	20257528	(G/A)
23248	CakSNP23248	*Kabuli*	Ca_Kabuli_denovo	20260076	(C/G)
23249	CakSNP23249	*Kabuli*	Ca_Kabuli_denovo	20260108	(G/A)
23250	CakSNP23250	*Kabuli*	Ca_Kabuli_denovo	20264839	(T/C)
23251	CakSNP23251	*Kabuli*	Ca_Kabuli_denovo	20269376	(C/T)
23252	CakSNP23252	*Kabuli*	Ca_Kabuli_denovo	20269682	(T/G)
23253	CakSNP23253	*Kabuli*	Ca_Kabuli_denovo	20272800	(A/C)
23254	CakSNP23254	*Kabuli*	Ca_Kabuli_denovo	20272866	(C/G)
23255	CakSNP23255	*Kabuli*	Ca_Kabuli_denovo	20274219	(C/T)

S.N.	SNP IDs	Cultivars	Chromosomes/scaffolds	Physical positions (bp)	SNPs
23256	CakSNP23256	*Kabuli*	Ca_Kabuli_denovo	20281838	(T/G)
23257	CakSNP23257	*Kabuli*	Ca_Kabuli_denovo	20283791	(G/A)
23258	CakSNP23258	*Kabuli*	Ca_Kabuli_denovo	20286365	(C/T)
23259	CakSNP23259	*Kabuli*	Ca_Kabuli_denovo	20286656	(G/A)
23260	CakSNP23260	*Kabuli*	Ca_Kabuli_denovo	20287498	(C/T)
23261	CakSNP23261	*Kabuli*	Ca_Kabuli_denovo	20287522	(T/C)
23262	CakSNP23262	*Kabuli*	Ca_Kabuli_denovo	20295694	(T/C)
23263	CakSNP23263	*Kabuli*	Ca_Kabuli_denovo	20295877	(A/G)
23264	CakSNP23264	*Kabuli*	Ca_Kabuli_denovo	20297276	(C/G)
23265	CakSNP23265	*Kabuli*	Ca_Kabuli_denovo	20297287	(T/C)
23266	CakSNP23266	*Kabuli*	Ca_Kabuli_denovo	20304659	(G/C)
23267	CakSNP23267	*Kabuli*	Ca_Kabuli_denovo	20307614	(C/T)
23268	CakSNP23268	*Kabuli*	Ca_Kabuli_denovo	20308015	(C/T)
23269	CakSNP23269	*Kabuli*	Ca_Kabuli_denovo	20309478	(T/C)
23270	CakSNP23270	*Kabuli*	Ca_Kabuli_denovo	20309524	(C/T)
23271	CakSNP23271	*Kabuli*	Ca_Kabuli_denovo	20309582	(A/C)
23272	CakSNP23272	*Kabuli*	Ca_Kabuli_denovo	20313491	(T/C)
23273	CakSNP23273	*Kabuli*	Ca_Kabuli_denovo	20317455	(G/T)
23274	CakSNP23274	*Kabuli*	Ca_Kabuli_denovo	20318783	(C/T)
23275	CakSNP23275	*Kabuli*	Ca_Kabuli_denovo	20319883	(T/G)
23276	CakSNP23276	*Kabuli*	Ca_Kabuli_denovo	20321059	(T/G)
23277	CakSNP23277	*Kabuli*	Ca_Kabuli_denovo	20333539	(G/T)
23278	CakSNP23278	*Kabuli*	Ca_Kabuli_denovo	20333593	(G/C)
23279	CakSNP23279	*Kabuli*	Ca_Kabuli_denovo	20338885	(G/A)
23280	CakSNP23280	*Kabuli*	Ca_Kabuli_denovo	20339572	(A/T)
23281	CakSNP23281	*Kabuli*	Ca_Kabuli_denovo	20355762	(C/T)
23282	CakSNP23282	*Kabuli*	Ca_Kabuli_denovo	20359839	(A/G)
23283	CakSNP23283	*Kabuli*	Ca_Kabuli_denovo	20360563	(C/T)
23284	CakSNP23284	*Kabuli*	Ca_Kabuli_denovo	20362457	(C/T)
23285	CakSNP23285	*Kabuli*	Ca_Kabuli_denovo	20367494	(C/T)
23286	CakSNP23286	*Kabuli*	Ca_Kabuli_denovo	20368379	(A/T)
23287	CakSNP23287	*Kabuli*	Ca_Kabuli_denovo	20370821	(T/C)
23288	CakSNP23288	*Kabuli*	Ca_Kabuli_denovo	20375797	(A/G)
23289	CakSNP23289	*Kabuli*	Ca_Kabuli_denovo	20375808	(G/A)
23290	CakSNP23290	*Kabuli*	Ca_Kabuli_denovo	20380039	(G/C)
23291	CakSNP23291	*Kabuli*	Ca_Kabuli_denovo	20386266	(G/A)
23292	CakSNP23292	*Kabuli*	Ca_Kabuli_denovo	20386332	(A/G)
23293	CakSNP23293	*Kabuli*	Ca_Kabuli_denovo	20393328	(C/T)

S.N.	SNP IDs	Cultivars	Chromosomes/scaffolds	Physical positions (bp)	SNPs
23294	CakSNP23294	*Kabuli*	Ca_Kabuli_denovo	20396493	(A/G)
23295	CakSNP23295	*Kabuli*	Ca_Kabuli_denovo	20407673	(G/C)
23296	CakSNP23296	*Kabuli*	Ca_Kabuli_denovo	20407694	(C/G)
23297	CakSNP23297	*Kabuli*	Ca_Kabuli_denovo	20411690	(A/G)
23298	CakSNP23298	*Kabuli*	Ca_Kabuli_denovo	20416894	(G/A)
23299	CakSNP23299	*Kabuli*	Ca_Kabuli_denovo	20416922	(T/C)
23300	CakSNP23300	*Kabuli*	Ca_Kabuli_denovo	20418994	(G/A)
23301	CakSNP23301	*Kabuli*	Ca_Kabuli_denovo	20424469	(A/G)
23302	CakSNP23302	*Kabuli*	Ca_Kabuli_denovo	20424475	(T/G)
23303	CakSNP23303	*Kabuli*	Ca_Kabuli_denovo	20426830	(C/G)
23304	CakSNP23304	*Kabuli*	Ca_Kabuli_denovo	20426890	(G/C)
23305	CakSNP23305	*Kabuli*	Ca_Kabuli_denovo	20450527	(C/T)
23306	CakSNP23306	*Kabuli*	Ca_Kabuli_denovo	20450551	(T/C)
23307	CakSNP23307	*Kabuli*	Ca_Kabuli_denovo	20462740	(T/C)
23308	CakSNP23308	*Kabuli*	Ca_Kabuli_denovo	20463827	(T/C)
23309	CakSNP23309	*Kabuli*	Ca_Kabuli_denovo	20463839	(C/T)
23310	CakSNP23310	*Kabuli*	Ca_Kabuli_denovo	20464124	(C/T)
23311	CakSNP23311	*Kabuli*	Ca_Kabuli_denovo	20464330	(G/T)
23312	CakSNP23312	*Kabuli*	Ca_Kabuli_denovo	20464352	(G/A)
23313	CakSNP23313	*Kabuli*	Ca_Kabuli_denovo	20466839	(C/T)
23314	CakSNP23314	*Kabuli*	Ca_Kabuli_denovo	20471359	(G/C)
23315	CakSNP23315	*Kabuli*	Ca_Kabuli_denovo	20473596	(G/A)
23316	CakSNP23316	*Kabuli*	Ca_Kabuli_denovo	20473597	(T/C)
23317	CakSNP23317	*Kabuli*	Ca_Kabuli_denovo	20474659	(A/G)
23318	CakSNP23318	*Kabuli*	Ca_Kabuli_denovo	20474660	(G/A)
23319	CakSNP23319	*Kabuli*	Ca_Kabuli_denovo	20486435	(C/A)
23320	CakSNP23320	*Kabuli*	Ca_Kabuli_denovo	20486468	(A/G)
23321	CakSNP23321	*Kabuli*	Ca_Kabuli_denovo	20490102	(C/A)
23322	CakSNP23322	*Kabuli*	Ca_Kabuli_denovo	20501163	(C/T)
23323	CakSNP23323	*Kabuli*	Ca_Kabuli_denovo	20511427	(A/C)
23324	CakSNP23324	*Kabuli*	Ca_Kabuli_denovo	20511808	(G/A)
23325	CakSNP23325	*Kabuli*	Ca_Kabuli_denovo	20516470	(C/T)
23326	CakSNP23326	*Kabuli*	Ca_Kabuli_denovo	20517433	(C/T)
23327	CakSNP23327	*Kabuli*	Ca_Kabuli_denovo	20522644	(C/T)
23328	CakSNP23328	*Kabuli*	Ca_Kabuli_denovo	20522665	(T/A)
23329	CakSNP23329	*Kabuli*	Ca_Kabuli_denovo	20541587	(C/G)
23330	CakSNP23330	*Kabuli*	Ca_Kabuli_denovo	20542211	(A/G)
23331	CakSNP23331	*Kabuli*	Ca_Kabuli_denovo	20546414	(A/G)

S.N.	SNP IDs	Cultivars	Chromosomes/scaffolds	Physical positions (bp)	SNPs
23332	CakSNP23332	*Kabuli*	Ca_Kabuli_denovo	20546702	(G/A)
23333	CakSNP23333	*Kabuli*	Ca_Kabuli_denovo	20546714	(G/A)
23334	CakSNP23334	*Kabuli*	Ca_Kabuli_denovo	20549051	(C/G)
23335	CakSNP23335	*Kabuli*	Ca_Kabuli_denovo	20554469	(G/T)
23336	CakSNP23336	*Kabuli*	Ca_Kabuli_denovo	20555467	(T/G)
23337	CakSNP23337	*Kabuli*	Ca_Kabuli_denovo	20555488	(T/C)
23338	CakSNP23338	*Kabuli*	Ca_Kabuli_denovo	20567620	(G/T)
23339	CakSNP23339	*Kabuli*	Ca_Kabuli_denovo	20567674	(G/C)
23340	CakSNP23340	*Kabuli*	Ca_Kabuli_denovo	20568646	(A/G)
23341	CakSNP23341	*Kabuli*	Ca_Kabuli_denovo	20569289	(G/A)
23342	CakSNP23342	*Kabuli*	Ca_Kabuli_denovo	20571818	(T/C)
23343	CakSNP23343	*Kabuli*	Ca_Kabuli_denovo	20571867	(C/A)
23344	CakSNP23344	*Kabuli*	Ca_Kabuli_denovo	20577212	(G/A)
23345	CakSNP23345	*Kabuli*	Ca_Kabuli_denovo	20577218	(C/T)
23346	CakSNP23346	*Kabuli*	Ca_Kabuli_denovo	20581578	(G/A)
23347	CakSNP23347	*Kabuli*	Ca_Kabuli_denovo	20584509	(T/C)
23348	CakSNP23348	*Kabuli*	Ca_Kabuli_denovo	20588181	(G/C)
23349	CakSNP23349	*Kabuli*	Ca_Kabuli_denovo	20588184	(T/C)
23350	CakSNP23350	*Kabuli*	Ca_Kabuli_denovo	20588933	(A/G)
23351	CakSNP23351	*Kabuli*	Ca_Kabuli_denovo	20588955	(C/T)
23352	CakSNP23352	*Kabuli*	Ca_Kabuli_denovo	20591069	(C/T)
23353	CakSNP23353	*Kabuli*	Ca_Kabuli_denovo	20591637	(G/A)
23354	CakSNP23354	*Kabuli*	Ca_Kabuli_denovo	20591679	(C/G)
23355	CakSNP23355	*Kabuli*	Ca_Kabuli_denovo	20597291	(C/T)
23356	CakSNP23356	*Kabuli*	Ca_Kabuli_denovo	20603510	(C/G)
23357	CakSNP23357	*Kabuli*	Ca_Kabuli_denovo	20608014	(T/C)
23358	CakSNP23358	*Kabuli*	Ca_Kabuli_denovo	20608016	(G/C)
23359	CakSNP23359	*Kabuli*	Ca_Kabuli_denovo	20608286	(G/A)
23360	CakSNP23360	*Kabuli*	Ca_Kabuli_denovo	20608691	(A/G)
23361	CakSNP23361	*Kabuli*	Ca_Kabuli_denovo	20610844	(G/A)
23362	CakSNP23362	*Kabuli*	Ca_Kabuli_denovo	20618225	(C/A)
23363	CakSNP23363	*Kabuli*	Ca_Kabuli_denovo	20620363	(T/C)
23364	CakSNP23364	*Kabuli*	Ca_Kabuli_denovo	20621301	(A/G)
23365	CakSNP23365	*Kabuli*	Ca_Kabuli_denovo	20622012	(G/A)
23366	CakSNP23366	*Kabuli*	Ca_Kabuli_denovo	20624246	(T/C)
23367	CakSNP23367	*Kabuli*	Ca_Kabuli_denovo	20624249	(A/G)
23368	CakSNP23368	*Kabuli*	Ca_Kabuli_denovo	20626274	(T/C)
23369	CakSNP23369	*Kabuli*	Ca_Kabuli_denovo	20626284	(G/A)

S.N.	SNP IDs	Cultivars	Chromosomes/scaffolds	Physical positions (bp)	SNPs
23370	CakSNP23370	*Kabuli*	Ca_Kabuli_denovo	20629298	(G/A)
23371	CakSNP23371	*Kabuli*	Ca_Kabuli_denovo	20629762	(T/C)
23372	CakSNP23372	*Kabuli*	Ca_Kabuli_denovo	20629816	(C/T)
23373	CakSNP23373	*Kabuli*	Ca_Kabuli_denovo	20629867	(T/C)
23374	CakSNP23374	*Kabuli*	Ca_Kabuli_denovo	20631581	(A/C)
23375	CakSNP23375	*Kabuli*	Ca_Kabuli_denovo	20632830	(A/T)
23376	CakSNP23376	*Kabuli*	Ca_Kabuli_denovo	20632874	(G/A)
23377	CakSNP23377	*Kabuli*	Ca_Kabuli_denovo	20644947	(G/A)
23378	CakSNP23378	*Kabuli*	Ca_Kabuli_denovo	20647462	(G/A)
23379	CakSNP23379	*Kabuli*	Ca_Kabuli_denovo	20647463	(T/A)
23380	CakSNP23380	*Kabuli*	Ca_Kabuli_denovo	20649939	(C/T)
23381	CakSNP23381	*Kabuli*	Ca_Kabuli_denovo	20649985	(G/A)
23382	CakSNP23382	*Kabuli*	Ca_Kabuli_denovo	20651807	(T/C)
23383	CakSNP23383	*Kabuli*	Ca_Kabuli_denovo	20652253	(G/A)
23384	CakSNP23384	*Kabuli*	Ca_Kabuli_denovo	20652319	(C/G)
23385	CakSNP23385	*Kabuli*	Ca_Kabuli_denovo	20655181	(A/G)
23386	CakSNP23386	*Kabuli*	Ca_Kabuli_denovo	20655205	(C/T)
23387	CakSNP23387	*Kabuli*	Ca_Kabuli_denovo	20658706	(G/C)
23388	CakSNP23388	*Kabuli*	Ca_Kabuli_denovo	20659695	(A/G)
23389	CakSNP23389	*Kabuli*	Ca_Kabuli_denovo	20660543	(A/G)
23390	CakSNP23390	*Kabuli*	Ca_Kabuli_denovo	20661734	(G/A)
23391	CakSNP23391	*Kabuli*	Ca_Kabuli_denovo	20663129	(C/T)
23392	CakSNP23392	*Kabuli*	Ca_Kabuli_denovo	20663431	(A/G)
23393	CakSNP23393	*Kabuli*	Ca_Kabuli_denovo	20663481	(G/C)
23394	CakSNP23394	*Kabuli*	Ca_Kabuli_denovo	20664732	(A/G)
23395	CakSNP23395	*Kabuli*	Ca_Kabuli_denovo	20666474	(G/A)
23396	CakSNP23396	*Kabuli*	Ca_Kabuli_denovo	20666533	(T/C)
23397	CakSNP23397	*Kabuli*	Ca_Kabuli_denovo	20671435	(G/T)
23398	CakSNP23398	*Kabuli*	Ca_Kabuli_denovo	20671438	(T/C)
23399	CakSNP23399	*Kabuli*	Ca_Kabuli_denovo	20671699	(G/A)
23400	CakSNP23400	*Kabuli*	Ca_Kabuli_denovo	20674290	(T/C)
23401	CakSNP23401	*Kabuli*	Ca_Kabuli_denovo	20674470	(G/T)
23402	CakSNP23402	*Kabuli*	Ca_Kabuli_denovo	20674471	(G/T)
23403	CakSNP23403	*Kabuli*	Ca_Kabuli_denovo	20674987	(G/T)
23404	CakSNP23404	*Kabuli*	Ca_Kabuli_denovo	20675794	(T/G)
23405	CakSNP23405	*Kabuli*	Ca_Kabuli_denovo	20675819	(T/G)
23406	CakSNP23406	*Kabuli*	Ca_Kabuli_denovo	20676549	(T/C)
23407	CakSNP23407	*Kabuli*	Ca_Kabuli_denovo	20679128	(T/G)

S.N.	SNP IDs	Cultivars	Chromosomes/scaffolds	Physical positions (bp)	SNPs
23408	CakSNP23408	*Kabuli*	Ca_Kabuli_denovo	20681826	(C/T)
23409	CakSNP23409	*Kabuli*	Ca_Kabuli_denovo	20681874	(G/C)
23410	CakSNP23410	*Kabuli*	Ca_Kabuli_denovo	20682581	(T/C)
23411	CakSNP23411	*Kabuli*	Ca_Kabuli_denovo	20682638	(C/T)
23412	CakSNP23412	*Kabuli*	Ca_Kabuli_denovo	20692335	(G/C)
23413	CakSNP23413	*Kabuli*	Ca_Kabuli_denovo	20692407	(A/C)
23414	CakSNP23414	*Kabuli*	Ca_Kabuli_denovo	20693892	(G/T)
23415	CakSNP23415	*Kabuli*	Ca_Kabuli_denovo	20697315	(A/G)
23416	CakSNP23416	*Kabuli*	Ca_Kabuli_denovo	20698586	(G/T)
23417	CakSNP23417	*Kabuli*	Ca_Kabuli_denovo	20702486	(G/C)
23418	CakSNP23418	*Kabuli*	Ca_Kabuli_denovo	20702493	(C/G)
23419	CakSNP23419	*Kabuli*	Ca_Kabuli_denovo	20706779	(G/T)
23420	CakSNP23420	*Kabuli*	Ca_Kabuli_denovo	20706816	(A/T)
23421	CakSNP23421	*Kabuli*	Ca_Kabuli_denovo	20710940	(G/A)
23422	CakSNP23422	*Kabuli*	Ca_Kabuli_denovo	20712713	(G/C)
23423	CakSNP23423	*Kabuli*	Ca_Kabuli_denovo	20712728	(C/T)
23424	CakSNP23424	*Kabuli*	Ca_Kabuli_denovo	20713275	(G/A)
23425	CakSNP23425	*Kabuli*	Ca_Kabuli_denovo	20715271	(G/A)
23426	CakSNP23426	*Kabuli*	Ca_Kabuli_denovo	20720557	(A/G)
23427	CakSNP23427	*Kabuli*	Ca_Kabuli_denovo	20723873	(C/G)
23428	CakSNP23428	*Kabuli*	Ca_Kabuli_denovo	20723876	(A/G)
23429	CakSNP23429	*Kabuli*	Ca_Kabuli_denovo	20725341	(A/G)
23430	CakSNP23430	*Kabuli*	Ca_Kabuli_denovo	20725359	(C/G)
23431	CakSNP23431	*Kabuli*	Ca_Kabuli_denovo	20727759	(C/G)
23432	CakSNP23432	*Kabuli*	Ca_Kabuli_denovo	20727764	(G/C)
23433	CakSNP23433	*Kabuli*	Ca_Kabuli_denovo	20740597	(A/G)
23434	CakSNP23434	*Kabuli*	Ca_Kabuli_denovo	20740642	(C/G)
23435	CakSNP23435	*Kabuli*	Ca_Kabuli_denovo	20743843	(A/G)
23436	CakSNP23436	*Kabuli*	Ca_Kabuli_denovo	20760598	(T/G)
23437	CakSNP23437	*Kabuli*	Ca_Kabuli_denovo	20765039	(A/C)
23438	CakSNP23438	*Kabuli*	Ca_Kabuli_denovo	20765041	(A/G)
23439	CakSNP23439	*Kabuli*	Ca_Kabuli_denovo	20773882	(C/A)
23440	CakSNP23440	*Kabuli*	Ca_Kabuli_denovo	20777272	(G/T)
23441	CakSNP23441	*Kabuli*	Ca_Kabuli_denovo	20777274	(T/C)
23442	CakSNP23442	*Kabuli*	Ca_Kabuli_denovo	20782360	(T/C)
23443	CakSNP23443	*Kabuli*	Ca_Kabuli_denovo	20789033	(T/C)
23444	CakSNP23444	*Kabuli*	Ca_Kabuli_denovo	20790116	(A/C)
23445	CakSNP23445	*Kabuli*	Ca_Kabuli_denovo	20790127	(C/T)

S.N.	SNP IDs	Cultivars	Chromosomes/scaffolds	Physical positions (bp)	SNPs
23446	CakSNP23446	*Kabuli*	Ca_Kabuli_denovo	20800334	(G/C)
23447	CakSNP23447	*Kabuli*	Ca_Kabuli_denovo	20801433	(G/A)
23448	CakSNP23448	*Kabuli*	Ca_Kabuli_denovo	20801457	(A/C)
23449	CakSNP23449	*Kabuli*	Ca_Kabuli_denovo	20806916	(G/A)
23450	CakSNP23450	*Kabuli*	Ca_Kabuli_denovo	20820671	(G/A)
23451	CakSNP23451	*Kabuli*	Ca_Kabuli_denovo	20820733	(T/C)
23452	CakSNP23452	*Kabuli*	Ca_Kabuli_denovo	20822191	(C/G)
23453	CakSNP23453	*Kabuli*	Ca_Kabuli_denovo	20827366	(C/G)
23454	CakSNP23454	*Kabuli*	Ca_Kabuli_denovo	20827408	(C/G)
23455	CakSNP23455	*Kabuli*	Ca_Kabuli_denovo	20828077	(G/T)
23456	CakSNP23456	*Kabuli*	Ca_Kabuli_denovo	20840036	(A/T)
23457	CakSNP23457	*Kabuli*	Ca_Kabuli_denovo	20848569	(C/T)
23458	CakSNP23458	*Kabuli*	Ca_Kabuli_denovo	20850730	(C/G)
23459	CakSNP23459	*Kabuli*	Ca_Kabuli_denovo	20858746	(C/T)
23460	CakSNP23460	*Kabuli*	Ca_Kabuli_denovo	20858776	(G/T)
23461	CakSNP23461	*Kabuli*	Ca_Kabuli_denovo	20859779	(C/G)
23462	CakSNP23462	*Kabuli*	Ca_Kabuli_denovo	20859806	(A/C)
23463	CakSNP23463	*Kabuli*	Ca_Kabuli_denovo	20865497	(C/T)
23464	CakSNP23464	*Kabuli*	Ca_Kabuli_denovo	20869412	(A/G)
23465	CakSNP23465	*Kabuli*	Ca_Kabuli_denovo	20870518	(G/T)
23466	CakSNP23466	*Kabuli*	Ca_Kabuli_denovo	20870556	(T/C)
23467	CakSNP23467	*Kabuli*	Ca_Kabuli_denovo	20877900	(G/C)
23468	CakSNP23468	*Kabuli*	Ca_Kabuli_denovo	20881291	(A/C)
23469	CakSNP23469	*Kabuli*	Ca_Kabuli_denovo	20889434	(C/T)
23470	CakSNP23470	*Kabuli*	Ca_Kabuli_denovo	20889463	(A/G)
23471	CakSNP23471	*Kabuli*	Ca_Kabuli_denovo	20890276	(G/A)
23472	CakSNP23472	*Kabuli*	Ca_Kabuli_denovo	20890735	(C/G)
23473	CakSNP23473	*Kabuli*	Ca_Kabuli_denovo	20891812	(G/T)
23474	CakSNP23474	*Kabuli*	Ca_Kabuli_denovo	20896658	(T/C)
23475	CakSNP23475	*Kabuli*	Ca_Kabuli_denovo	20899817	(G/A)
23476	CakSNP23476	*Kabuli*	Ca_Kabuli_denovo	20899873	(C/T)
23477	CakSNP23477	*Kabuli*	Ca_Kabuli_denovo	20901410	(G/A)
23478	CakSNP23478	*Kabuli*	Ca_Kabuli_denovo	20902136	(G/T)
23479	CakSNP23479	*Kabuli*	Ca_Kabuli_denovo	20902138	(C/G)
23480	CakSNP23480	*Kabuli*	Ca_Kabuli_denovo	20905216	(C/G)
23481	CakSNP23481	*Kabuli*	Ca_Kabuli_denovo	20909680	(A/G)
23482	CakSNP23482	*Kabuli*	Ca_Kabuli_denovo	20918685	(A/G)
23483	CakSNP23483	*Kabuli*	Ca_Kabuli_denovo	20920301	(T/C)

S.N.	SNP IDs	Cultivars	Chromosomes/scaffolds	Physical positions (bp)	SNPs
23484	CakSNP23484	*Kabuli*	Ca_Kabuli_denovo	20927066	(C/T)
23485	CakSNP23485	*Kabuli*	Ca_Kabuli_denovo	20927075	(C/G)
23486	CakSNP23486	*Kabuli*	Ca_Kabuli_denovo	20930235	(A/G)
23487	CakSNP23487	*Kabuli*	Ca_Kabuli_denovo	20931160	(C/G)
23488	CakSNP23488	*Kabuli*	Ca_Kabuli_denovo	20932009	(C/G)
23489	CakSNP23489	*Kabuli*	Ca_Kabuli_denovo	20932069	(A/G)
23490	CakSNP23490	*Kabuli*	Ca_Kabuli_denovo	20935272	(C/T)
23491	CakSNP23491	*Kabuli*	Ca_Kabuli_denovo	20939058	(G/A)
23492	CakSNP23492	*Kabuli*	Ca_Kabuli_denovo	20942369	(T/G)
23493	CakSNP23493	*Kabuli*	Ca_Kabuli_denovo	20943817	(C/G)
23494	CakSNP23494	*Kabuli*	Ca_Kabuli_denovo	20943829	(C/T)
23495	CakSNP23495	*Kabuli*	Ca_Kabuli_denovo	20947352	(G/A)
23496	CakSNP23496	*Kabuli*	Ca_Kabuli_denovo	20952554	(G/C)
23497	CakSNP23497	*Kabuli*	Ca_Kabuli_denovo	20954312	(C/T)
23498	CakSNP23498	*Kabuli*	Ca_Kabuli_denovo	20954315	(T/C)
23499	CakSNP23499	*Kabuli*	Ca_Kabuli_denovo	20956282	(G/C)
23500	CakSNP23500	*Kabuli*	Ca_Kabuli_denovo	20956459	(C/T)
23501	CakSNP23501	*Kabuli*	Ca_Kabuli_denovo	20957959	(T/C)
23502	CakSNP23502	*Kabuli*	Ca_Kabuli_denovo	20959789	(T/A)
23503	CakSNP23503	*Kabuli*	Ca_Kabuli_denovo	20962222	(G/A)
23504	CakSNP23504	*Kabuli*	Ca_Kabuli_denovo	20962285	(A/G)
23505	CakSNP23505	*Kabuli*	Ca_Kabuli_denovo	20967037	(G/A)
23506	CakSNP23506	*Kabuli*	Ca_Kabuli_denovo	20975342	(A/G)
23507	CakSNP23507	*Kabuli*	Ca_Kabuli_denovo	20976175	(G/A)
23508	CakSNP23508	*Kabuli*	Ca_Kabuli_denovo	20976187	(A/G)
23509	CakSNP23509	*Kabuli*	Ca_Kabuli_denovo	20976285	(G/A)
23510	CakSNP23510	*Kabuli*	Ca_Kabuli_denovo	20978728	(G/A)
23511	CakSNP23511	*Kabuli*	Ca_Kabuli_denovo	20980830	(T/C)
23512	CakSNP23512	*Kabuli*	Ca_Kabuli_denovo	20980872	(T/C)
23513	CakSNP23513	*Kabuli*	Ca_Kabuli_denovo	20982688	(A/G)
23514	CakSNP23514	*Kabuli*	Ca_Kabuli_denovo	20982709	(G/T)
23515	CakSNP23515	*Kabuli*	Ca_Kabuli_denovo	20986531	(C/T)
23516	CakSNP23516	*Kabuli*	Ca_Kabuli_denovo	20987836	(A/G)
23517	CakSNP23517	*Kabuli*	Ca_Kabuli_denovo	20990243	(A/G)
23518	CakSNP23518	*Kabuli*	Ca_Kabuli_denovo	20990278	(C/A)
23519	CakSNP23519	*Kabuli*	Ca_Kabuli_denovo	20994487	(A/G)
23520	CakSNP23520	*Kabuli*	Ca_Kabuli_denovo	20994648	(A/C)
23521	CakSNP23521	*Kabuli*	Ca_Kabuli_denovo	20994656	(T/C)

S.N.	SNP IDs	Cultivars	Chromosomes/scaffolds	Physical positions (bp)	SNPs
23522	CakSNP23522	*Kabuli*	Ca_Kabuli_denovo	20996617	(A/G)
23523	CakSNP23523	*Kabuli*	Ca_Kabuli_denovo	21002777	(T/C)
23524	CakSNP23524	*Kabuli*	Ca_Kabuli_denovo	21005000	(G/A)
23525	CakSNP23525	*Kabuli*	Ca_Kabuli_denovo	21005039	(G/A)
23526	CakSNP23526	*Kabuli*	Ca_Kabuli_denovo	21005600	(G/C)
23527	CakSNP23527	*Kabuli*	Ca_Kabuli_denovo	21005601	(C/T)
23528	CakSNP23528	*Kabuli*	Ca_Kabuli_denovo	21008057	(T/G)
23529	CakSNP23529	*Kabuli*	Ca_Kabuli_denovo	21008099	(G/C)
23530	CakSNP23530	*Kabuli*	Ca_Kabuli_denovo	21008633	(A/C)
23531	CakSNP23531	*Kabuli*	Ca_Kabuli_denovo	21008674	(C/G)
23532	CakSNP23532	*Kabuli*	Ca_Kabuli_denovo	21012356	(C/T)
23533	CakSNP23533	*Kabuli*	Ca_Kabuli_denovo	21012365	(C/T)
23534	CakSNP23534	*Kabuli*	Ca_Kabuli_denovo	21017170	(C/G)
23535	CakSNP23535	*Kabuli*	Ca_Kabuli_denovo	21017204	(G/A)
23536	CakSNP23536	*Kabuli*	Ca_Kabuli_denovo	21017243	(A/G)
23537	CakSNP23537	*Kabuli*	Ca_Kabuli_denovo	21020518	(G/A)
23538	CakSNP23538	*Kabuli*	Ca_Kabuli_denovo	21020548	(A/T)
23539	CakSNP23539	*Kabuli*	Ca_Kabuli_denovo	21024546	(T/C)
23540	CakSNP23540	*Kabuli*	Ca_Kabuli_denovo	21030016	(C/T)
23541	CakSNP23541	*Kabuli*	Ca_Kabuli_denovo	21044341	(C/T)
23542	CakSNP23542	*Kabuli*	Ca_Kabuli_denovo	21047977	(T/C)
23543	CakSNP23543	*Kabuli*	Ca_Kabuli_denovo	21061094	(T/A)
23544	CakSNP23544	*Kabuli*	Ca_Kabuli_denovo	21068365	(G/C)
23545	CakSNP23545	*Kabuli*	Ca_Kabuli_denovo	21068404	(G/T)
23546	CakSNP23546	*Kabuli*	Ca_Kabuli_denovo	21071286	(C/T)
23547	CakSNP23547	*Kabuli*	Ca_Kabuli_denovo	21077257	(A/G)
23548	CakSNP23548	*Kabuli*	Ca_Kabuli_denovo	21077281	(T/C)
23549	CakSNP23549	*Kabuli*	Ca_Kabuli_denovo	21082230	(G/T)
23550	CakSNP23550	*Kabuli*	Ca_Kabuli_denovo	21082236	(G/T)
23551	CakSNP23551	*Kabuli*	Ca_Kabuli_denovo	21082241	(T/G)
23552	CakSNP23552	*Kabuli*	Ca_Kabuli_denovo	21082258	(T/G)
23553	CakSNP23553	*Kabuli*	Ca_Kabuli_denovo	21084346	(T/G)
23554	CakSNP23554	*Kabuli*	Ca_Kabuli_denovo	21084718	(T/G)
23555	CakSNP23555	*Kabuli*	Ca_Kabuli_denovo	21084761	(A/G)
23556	CakSNP23556	*Kabuli*	Ca_Kabuli_denovo	21090649	(T/C)
23557	CakSNP23557	*Kabuli*	Ca_Kabuli_denovo	21090888	(C/G)
23558	CakSNP23558	*Kabuli*	Ca_Kabuli_denovo	21090964	(T/G)
23559	CakSNP23559	*Kabuli*	Ca_Kabuli_denovo	21090985	(G/A)

S.N.	SNP IDs	Cultivars	Chromosomes/scaffolds	Physical positions (bp)	SNPs
23560	CakSNP23560	*Kabuli*	Ca_Kabuli_denovo	21100610	(C/T)
23561	CakSNP23561	*Kabuli*	Ca_Kabuli_denovo	21106642	(C/A)
23562	CakSNP23562	*Kabuli*	Ca_Kabuli_denovo	21106699	(A/C)
23563	CakSNP23563	*Kabuli*	Ca_Kabuli_denovo	21110848	(T/C)
23564	CakSNP23564	*Kabuli*	Ca_Kabuli_denovo	21113900	(T/C)
23565	CakSNP23565	*Kabuli*	Ca_Kabuli_denovo	21115327	(A/G)
23566	CakSNP23566	*Kabuli*	Ca_Kabuli_denovo	21115333	(A/G)
23567	CakSNP23567	*Kabuli*	Ca_Kabuli_denovo	21117191	(C/A)
23568	CakSNP23568	*Kabuli*	Ca_Kabuli_denovo	21117193	(A/G)
23569	CakSNP23569	*Kabuli*	Ca_Kabuli_denovo	21126652	(G/A)
23570	CakSNP23570	*Kabuli*	Ca_Kabuli_denovo	21126679	(A/G)
23571	CakSNP23571	*Kabuli*	Ca_Kabuli_denovo	21134870	(G/C)
23572	CakSNP23572	*Kabuli*	Ca_Kabuli_denovo	21144962	(T/A)
23573	CakSNP23573	*Kabuli*	Ca_Kabuli_denovo	21144985	(C/A)
23574	CakSNP23574	*Kabuli*	Ca_Kabuli_denovo	21147449	(T/C)
23575	CakSNP23575	*Kabuli*	Ca_Kabuli_denovo	21150332	(C/T)
23576	CakSNP23576	*Kabuli*	Ca_Kabuli_denovo	21154187	(C/T)
23577	CakSNP23577	*Kabuli*	Ca_Kabuli_denovo	21158231	(C/G)
23578	CakSNP23578	*Kabuli*	Ca_Kabuli_denovo	21160880	(G/A)
23579	CakSNP23579	*Kabuli*	Ca_Kabuli_denovo	21162260	(T/C)
23580	CakSNP23580	*Kabuli*	Ca_Kabuli_denovo	21162266	(A/G)
23581	CakSNP23581	*Kabuli*	Ca_Kabuli_denovo	21169177	(C/T)
23582	CakSNP23582	*Kabuli*	Ca_Kabuli_denovo	21173866	(C/A)
23583	CakSNP23583	*Kabuli*	Ca_Kabuli_denovo	21176854	(A/G)
23584	CakSNP23584	*Kabuli*	Ca_Kabuli_denovo	21176884	(G/A)
23585	CakSNP23585	*Kabuli*	Ca_Kabuli_denovo	21178432	(G/A)
23586	CakSNP23586	*Kabuli*	Ca_Kabuli_denovo	21179437	(A/G)
23587	CakSNP23587	*Kabuli*	Ca_Kabuli_denovo	21179446	(C/G)
23588	CakSNP23588	*Kabuli*	Ca_Kabuli_denovo	21181786	(T/C)
23589	CakSNP23589	*Kabuli*	Ca_Kabuli_denovo	21184619	(T/C)
23590	CakSNP23590	*Kabuli*	Ca_Kabuli_denovo	21184628	(T/C)
23591	CakSNP23591	*Kabuli*	Ca_Kabuli_denovo	21186065	(G/A)
23592	CakSNP23592	*Kabuli*	Ca_Kabuli_denovo	21186539	(G/T)
23593	CakSNP23593	*Kabuli*	Ca_Kabuli_denovo	21190874	(G/T)
23594	CakSNP23594	*Kabuli*	Ca_Kabuli_denovo	21194997	(C/T)
23595	CakSNP23595	*Kabuli*	Ca_Kabuli_denovo	21199835	(G/C)
23596	CakSNP23596	*Kabuli*	Ca_Kabuli_denovo	21200833	(T/G)
23597	CakSNP23597	*Kabuli*	Ca_Kabuli_denovo	21204235	(G/C)

S.N.	SNP IDs	Cultivars	Chromosomes/scaffolds	Physical positions (bp)	SNPs
23598	CakSNP23598	*Kabuli*	Ca_Kabuli_denovo	21208112	(C/A)
23599	CakSNP23599	*Kabuli*	Ca_Kabuli_denovo	21208135	(A/G)
23600	CakSNP23600	*Kabuli*	Ca_Kabuli_denovo	21213971	(C/T)
23601	CakSNP23601	*Kabuli*	Ca_Kabuli_denovo	21221451	(T/G)
23602	CakSNP23602	*Kabuli*	Ca_Kabuli_denovo	21225048	(C/G)
23603	CakSNP23603	*Kabuli*	Ca_Kabuli_denovo	21225421	(G/C)
23604	CakSNP23604	*Kabuli*	Ca_Kabuli_denovo	21231167	(T/G)
23605	CakSNP23605	*Kabuli*	Ca_Kabuli_denovo	21232376	(C/T)
23606	CakSNP23606	*Kabuli*	Ca_Kabuli_denovo	21232394	(C/T)
23607	CakSNP23607	*Kabuli*	Ca_Kabuli_denovo	21244441	(C/T)
23608	CakSNP23608	*Kabuli*	Ca_Kabuli_denovo	21250052	(G/A)
23609	CakSNP23609	*Kabuli*	Ca_Kabuli_denovo	21250842	(G/A)
23610	CakSNP23610	*Kabuli*	Ca_Kabuli_denovo	21251182	(T/G)
23611	CakSNP23611	*Kabuli*	Ca_Kabuli_denovo	21254233	(T/C)
23612	CakSNP23612	*Kabuli*	Ca_Kabuli_denovo	21255077	(G/A)
23613	CakSNP23613	*Kabuli*	Ca_Kabuli_denovo	21255104	(G/C)
23614	CakSNP23614	*Kabuli*	Ca_Kabuli_denovo	21256294	(T/C)
23615	CakSNP23615	*Kabuli*	Ca_Kabuli_denovo	21262614	(C/G)
23616	CakSNP23616	*Kabuli*	Ca_Kabuli_denovo	21262652	(T/C)
23617	CakSNP23617	*Kabuli*	Ca_Kabuli_denovo	21267786	(A/G)
23618	CakSNP23618	*Kabuli*	Ca_Kabuli_denovo	21270928	(G/A)
23619	CakSNP23619	*Kabuli*	Ca_Kabuli_denovo	21271173	(G/A)
23620	CakSNP23620	*Kabuli*	Ca_Kabuli_denovo	21271385	(C/A)
23621	CakSNP23621	*Kabuli*	Ca_Kabuli_denovo	21272775	(A/G)
23622	CakSNP23622	*Kabuli*	Ca_Kabuli_denovo	21277217	(C/T)
23623	CakSNP23623	*Kabuli*	Ca_Kabuli_denovo	21277218	(C/T)
23624	CakSNP23624	*Kabuli*	Ca_Kabuli_denovo	21279072	(C/T)
23625	CakSNP23625	*Kabuli*	Ca_Kabuli_denovo	21279083	(A/G)
23626	CakSNP23626	*Kabuli*	Ca_Kabuli_denovo	21282944	(G/A)
23627	CakSNP23627	*Kabuli*	Ca_Kabuli_denovo	21283436	(C/T)
23628	CakSNP23628	*Kabuli*	Ca_Kabuli_denovo	21284173	(C/G)
23629	CakSNP23629	*Kabuli*	Ca_Kabuli_denovo	21295065	(G/C)
23630	CakSNP23630	*Kabuli*	Ca_Kabuli_denovo	21295070	(T/C)
23631	CakSNP23631	*Kabuli*	Ca_Kabuli_denovo	21296946	(T/A)
23632	CakSNP23632	*Kabuli*	Ca_Kabuli_denovo	21296983	(G/A)
23633	CakSNP23633	*Kabuli*	Ca_Kabuli_denovo	21298616	(T/C)
23634	CakSNP23634	*Kabuli*	Ca_Kabuli_denovo	21307635	(G/A)
23635	CakSNP23635	*Kabuli*	Ca_Kabuli_denovo	21308677	(T/G)

S.N.	SNP IDs	Cultivars	Chromosomes/scaffolds	Physical positions (bp)	SNPs
23636	CakSNP23636	*Kabuli*	Ca_Kabuli_denovo	21318213	(A/C)
23637	CakSNP23637	*Kabuli*	Ca_Kabuli_denovo	21329472	(A/G)
23638	CakSNP23638	*Kabuli*	Ca_Kabuli_denovo	21331823	(C/A)
23639	CakSNP23639	*Kabuli*	Ca_Kabuli_denovo	21331826	(C/G)
23640	CakSNP23640	*Kabuli*	Ca_Kabuli_denovo	21332074	(C/T)
23641	CakSNP23641	*Kabuli*	Ca_Kabuli_denovo	21332076	(A/G)
23642	CakSNP23642	*Kabuli*	Ca_Kabuli_denovo	21341423	(C/T)
23643	CakSNP23643	*Kabuli*	Ca_Kabuli_denovo	21341964	(T/C)
23644	CakSNP23644	*Kabuli*	Ca_Kabuli_denovo	21343141	(C/T)
23645	CakSNP23645	*Kabuli*	Ca_Kabuli_denovo	21344835	(G/C)
23646	CakSNP23646	*Kabuli*	Ca_Kabuli_denovo	21348438	(G/A)
23647	CakSNP23647	*Kabuli*	Ca_Kabuli_denovo	21348450	(T/C)
23648	CakSNP23648	*Kabuli*	Ca_Kabuli_denovo	21349005	(C/A)
23649	CakSNP23649	*Kabuli*	Ca_Kabuli_denovo	21351422	(A/T)
23650	CakSNP23650	*Kabuli*	Ca_Kabuli_denovo	21356826	(T/C)
23651	CakSNP23651	*Kabuli*	Ca_Kabuli_denovo	21356851	(C/G)
23652	CakSNP23652	*Kabuli*	Ca_Kabuli_denovo	21358482	(G/A)
23653	CakSNP23653	*Kabuli*	Ca_Kabuli_denovo	21358524	(C/T)
23654	CakSNP23654	*Kabuli*	Ca_Kabuli_denovo	21361581	(C/T)
23655	CakSNP23655	*Kabuli*	Ca_Kabuli_denovo	21366756	(G/T)
23656	CakSNP23656	*Kabuli*	Ca_Kabuli_denovo	21367515	(G/C)
23657	CakSNP23657	*Kabuli*	Ca_Kabuli_denovo	21367533	(T/C)
23658	CakSNP23658	*Kabuli*	Ca_Kabuli_denovo	21367537	(C/A)
23659	CakSNP23659	*Kabuli*	Ca_Kabuli_denovo	21372130	(G/A)
23660	CakSNP23660	*Kabuli*	Ca_Kabuli_denovo	21377916	(G/A)
23661	CakSNP23661	*Kabuli*	Ca_Kabuli_denovo	21377970	(T/C)
23662	CakSNP23662	*Kabuli*	Ca_Kabuli_denovo	21380467	(C/T)
23663	CakSNP23663	*Kabuli*	Ca_Kabuli_denovo	21381098	(G/A)
23664	CakSNP23664	*Kabuli*	Ca_Kabuli_denovo	21381144	(C/A)
23665	CakSNP23665	*Kabuli*	Ca_Kabuli_denovo	21384365	(C/T)
23666	CakSNP23666	*Kabuli*	Ca_Kabuli_denovo	21384367	(G/A)
23667	CakSNP23667	*Kabuli*	Ca_Kabuli_denovo	21385344	(T/G)
23668	CakSNP23668	*Kabuli*	Ca_Kabuli_denovo	21393480	(A/G)
23669	CakSNP23669	*Kabuli*	Ca_Kabuli_denovo	21393528	(G/A)
23670	CakSNP23670	*Kabuli*	Ca_Kabuli_denovo	21399407	(A/T)
23671	CakSNP23671	*Kabuli*	Ca_Kabuli_denovo	21399412	(G/A)
23672	CakSNP23672	*Kabuli*	Ca_Kabuli_denovo	21400517	(C/T)
23673	CakSNP23673	*Kabuli*	Ca_Kabuli_denovo	21400573	(C/T)

S.N.	SNP IDs	Cultivars	Chromosomes/scaffolds	Physical positions (bp)	SNPs
23674	CakSNP23674	*Kabuli*	Ca_Kabuli_denovo	21407519	(A/G)
23675	CakSNP23675	*Kabuli*	Ca_Kabuli_denovo	21421791	(A/G)
23676	CakSNP23676	*Kabuli*	Ca_Kabuli_denovo	21424958	(T/G)
23677	CakSNP23677	*Kabuli*	Ca_Kabuli_denovo	21440438	(T/G)
23678	CakSNP23678	*Kabuli*	Ca_Kabuli_denovo	21445256	(G/A)
23679	CakSNP23679	*Kabuli*	Ca_Kabuli_denovo	21445286	(A/G)
23680	CakSNP23680	*Kabuli*	Ca_Kabuli_denovo	21451496	(T/C)
23681	CakSNP23681	*Kabuli*	Ca_Kabuli_denovo	21452183	(A/G)
23682	CakSNP23682	*Kabuli*	Ca_Kabuli_denovo	21455208	(T/C)
23683	CakSNP23683	*Kabuli*	Ca_Kabuli_denovo	21455244	(C/A)
23684	CakSNP23684	*Kabuli*	Ca_Kabuli_denovo	21459122	(C/A)
23685	CakSNP23685	*Kabuli*	Ca_Kabuli_denovo	21459187	(G/A)
23686	CakSNP23686	*Kabuli*	Ca_Kabuli_denovo	21467527	(T/G)
23687	CakSNP23687	*Kabuli*	Ca_Kabuli_denovo	21467545	(C/T)
23688	CakSNP23688	*Kabuli*	Ca_Kabuli_denovo	21475924	(A/G)
23689	CakSNP23689	*Kabuli*	Ca_Kabuli_denovo	21478754	(G/A)
23690	CakSNP23690	*Kabuli*	Ca_Kabuli_denovo	21482973	(G/C)
23691	CakSNP23691	*Kabuli*	Ca_Kabuli_denovo	21484952	(C/T)
23692	CakSNP23692	*Kabuli*	Ca_Kabuli_denovo	21490667	(C/T)
23693	CakSNP23693	*Kabuli*	Ca_Kabuli_denovo	21491669	(C/G)
23694	CakSNP23694	*Kabuli*	Ca_Kabuli_denovo	21493435	(C/A)
23695	CakSNP23695	*Kabuli*	Ca_Kabuli_denovo	21501063	(A/G)
23696	CakSNP23696	*Kabuli*	Ca_Kabuli_denovo	21503867	(A/G)
23697	CakSNP23697	*Kabuli*	Ca_Kabuli_denovo	21505441	(A/T)
23698	CakSNP23698	*Kabuli*	Ca_Kabuli_denovo	21506174	(G/A)
23699	CakSNP23699	*Kabuli*	Ca_Kabuli_denovo	21506198	(C/T)
23700	CakSNP23700	*Kabuli*	Ca_Kabuli_denovo	21510879	(G/C)
23701	CakSNP23701	*Kabuli*	Ca_Kabuli_denovo	21512410	(G/A)
23702	CakSNP23702	*Kabuli*	Ca_Kabuli_denovo	21512440	(T/C)
23703	CakSNP23703	*Kabuli*	Ca_Kabuli_denovo	21517339	(T/C)
23704	CakSNP23704	*Kabuli*	Ca_Kabuli_denovo	21517364	(G/A)
23705	CakSNP23705	*Kabuli*	Ca_Kabuli_denovo	21521543	(T/C)
23706	CakSNP23706	*Kabuli*	Ca_Kabuli_denovo	21529901	(C/T)
23707	CakSNP23707	*Kabuli*	Ca_Kabuli_denovo	21533729	(C/A)
23708	CakSNP23708	*Kabuli*	Ca_Kabuli_denovo	21536504	(G/A)
23709	CakSNP23709	*Kabuli*	Ca_Kabuli_denovo	21536561	(C/G)
23710	CakSNP23710	*Kabuli*	Ca_Kabuli_denovo	21536878	(T/C)
23711	CakSNP23711	*Kabuli*	Ca_Kabuli_denovo	21541280	(C/T)

S.N.	SNP IDs	Cultivars	Chromosomes/scaffolds	Physical positions (bp)	SNPs
23712	CakSNP23712	*Kabuli*	Ca_Kabuli_denovo	21541298	(C/T)
23713	CakSNP23713	*Kabuli*	Ca_Kabuli_denovo	21541870	(T/C)
23714	CakSNP23714	*Kabuli*	Ca_Kabuli_denovo	21549651	(C/G)
23715	CakSNP23715	*Kabuli*	Ca_Kabuli_denovo	21558166	(C/T)
23716	CakSNP23716	*Kabuli*	Ca_Kabuli_denovo	21559750	(A/T)
23717	CakSNP23717	*Kabuli*	Ca_Kabuli_denovo	21560733	(T/G)
23718	CakSNP23718	*Kabuli*	Ca_Kabuli_denovo	21561543	(G/T)
23719	CakSNP23719	*Kabuli*	Ca_Kabuli_denovo	21565201	(A/G)
23720	CakSNP23720	*Kabuli*	Ca_Kabuli_denovo	21565213	(A/T)
23721	CakSNP23721	*Kabuli*	Ca_Kabuli_denovo	21576483	(G/A)
23722	CakSNP23722	*Kabuli*	Ca_Kabuli_denovo	21577694	(T/G)
23723	CakSNP23723	*Kabuli*	Ca_Kabuli_denovo	21577730	(G/A)
23724	CakSNP23724	*Kabuli*	Ca_Kabuli_denovo	21577736	(A/G)
23725	CakSNP23725	*Kabuli*	Ca_Kabuli_denovo	21577930	(G/T)
23726	CakSNP23726	*Kabuli*	Ca_Kabuli_denovo	21587730	(C/A)
23727	CakSNP23727	*Kabuli*	Ca_Kabuli_denovo	21587742	(G/C)
23728	CakSNP23728	*Kabuli*	Ca_Kabuli_denovo	21588175	(C/G)
23729	CakSNP23729	*Kabuli*	Ca_Kabuli_denovo	21588402	(G/T)
23730	CakSNP23730	*Kabuli*	Ca_Kabuli_denovo	21588479	(A/G)
23731	CakSNP23731	*Kabuli*	Ca_Kabuli_denovo	21589510	(C/A)
23732	CakSNP23732	*Kabuli*	Ca_Kabuli_denovo	21591421	(C/T)
23733	CakSNP23733	*Kabuli*	Ca_Kabuli_denovo	21595077	(C/T)
23734	CakSNP23734	*Kabuli*	Ca_Kabuli_denovo	21595111	(C/T)
23735	CakSNP23735	*Kabuli*	Ca_Kabuli_denovo	21595640	(C/G)
23736	CakSNP23736	*Kabuli*	Ca_Kabuli_denovo	21611670	(T/A)
23737	CakSNP23737	*Kabuli*	Ca_Kabuli_denovo	21611698	(T/G)
23738	CakSNP23738	*Kabuli*	Ca_Kabuli_denovo	21633676	(C/T)
23739	CakSNP23739	*Kabuli*	Ca_Kabuli_denovo	21633691	(T/C)
23740	CakSNP23740	*Kabuli*	Ca_Kabuli_denovo	21651168	(C/T)
23741	CakSNP23741	*Kabuli*	Ca_Kabuli_denovo	21651210	(A/G)
23742	CakSNP23742	*Kabuli*	Ca_Kabuli_denovo	21652622	(C/G)
23743	CakSNP23743	*Kabuli*	Ca_Kabuli_denovo	21655049	(A/C)
23744	CakSNP23744	*Kabuli*	Ca_Kabuli_denovo	21655050	(G/C)
23745	CakSNP23745	*Kabuli*	Ca_Kabuli_denovo	21659196	(C/G)
23746	CakSNP23746	*Kabuli*	Ca_Kabuli_denovo	21664740	(T/C)
23747	CakSNP23747	*Kabuli*	Ca_Kabuli_denovo	21675772	(C/T)
23748	CakSNP23748	*Kabuli*	Ca_Kabuli_denovo	21676093	(C/G)
23749	CakSNP23749	*Kabuli*	Ca_Kabuli_denovo	21676147	(T/G)

S.N.	SNP IDs	Cultivars	Chromosomes/scaffolds	Physical positions (bp)	SNPs
23750	CakSNP23750	*Kabuli*	Ca_Kabuli_denovo	21676158	(T/G)
23751	CakSNP23751	*Kabuli*	Ca_Kabuli_denovo	21683180	(A/T)
23752	CakSNP23752	*Kabuli*	Ca_Kabuli_denovo	21683186	(G/A)
23753	CakSNP23753	*Kabuli*	Ca_Kabuli_denovo	21690203	(T/G)
23754	CakSNP23754	*Kabuli*	Ca_Kabuli_denovo	21691850	(T/C)
23755	CakSNP23755	*Kabuli*	Ca_Kabuli_denovo	21691851	(T/C)
23756	CakSNP23756	*Kabuli*	Ca_Kabuli_denovo	21701022	(C/G)
23757	CakSNP23757	*Kabuli*	Ca_Kabuli_denovo	21702075	(G/A)
23758	CakSNP23758	*Kabuli*	Ca_Kabuli_denovo	21702811	(T/G)
23759	CakSNP23759	*Kabuli*	Ca_Kabuli_denovo	21705038	(C/G)
23760	CakSNP23760	*Kabuli*	Ca_Kabuli_denovo	21707912	(C/A)
23761	CakSNP23761	*Kabuli*	Ca_Kabuli_denovo	21713585	(T/C)
23762	CakSNP23762	*Kabuli*	Ca_Kabuli_denovo	21713624	(A/G)
23763	CakSNP23763	*Kabuli*	Ca_Kabuli_denovo	21717589	(T/C)
23764	CakSNP23764	*Kabuli*	Ca_Kabuli_denovo	21717626	(A/G)
23765	CakSNP23765	*Kabuli*	Ca_Kabuli_denovo	21724227	(A/G)
23766	CakSNP23766	*Kabuli*	Ca_Kabuli_denovo	21724237	(T/C)
23767	CakSNP23767	*Kabuli*	Ca_Kabuli_denovo	21725945	(A/C)
23768	CakSNP23768	*Kabuli*	Ca_Kabuli_denovo	21725951	(A/G)
23769	CakSNP23769	*Kabuli*	Ca_Kabuli_denovo	21726047	(C/A)
23770	CakSNP23770	*Kabuli*	Ca_Kabuli_denovo	21726056	(C/T)
23771	CakSNP23771	*Kabuli*	Ca_Kabuli_denovo	21726967	(G/A)
23772	CakSNP23772	*Kabuli*	Ca_Kabuli_denovo	21729583	(A/G)
23773	CakSNP23773	*Kabuli*	Ca_Kabuli_denovo	21730951	(C/T)
23774	CakSNP23774	*Kabuli*	Ca_Kabuli_denovo	21730966	(C/T)
23775	CakSNP23775	*Kabuli*	Ca_Kabuli_denovo	21745463	(A/T)
23776	CakSNP23776	*Kabuli*	Ca_Kabuli_denovo	21745487	(T/C)
23777	CakSNP23777	*Kabuli*	Ca_Kabuli_denovo	21748246	(C/T)
23778	CakSNP23778	*Kabuli*	Ca_Kabuli_denovo	21748466	(G/C)
23779	CakSNP23779	*Kabuli*	Ca_Kabuli_denovo	21759102	(T/G)
23780	CakSNP23780	*Kabuli*	Ca_Kabuli_denovo	21759330	(C/T)
23781	CakSNP23781	*Kabuli*	Ca_Kabuli_denovo	21759386	(C/T)
23782	CakSNP23782	*Kabuli*	Ca_Kabuli_denovo	21761283	(T/C)
23783	CakSNP23783	*Kabuli*	Ca_Kabuli_denovo	21762479	(A/C)
23784	CakSNP23784	*Kabuli*	Ca_Kabuli_denovo	21764026	(C/G)
23785	CakSNP23785	*Kabuli*	Ca_Kabuli_denovo	21765047	(G/A)
23786	CakSNP23786	*Kabuli*	Ca_Kabuli_denovo	21768651	(C/A)
23787	CakSNP23787	*Kabuli*	Ca_Kabuli_denovo	21768663	(G/A)

S.N.	SNP IDs	Cultivars	Chromosomes/scaffolds	Physical positions (bp)	SNPs
23788	CakSNP23788	*Kabuli*	Ca_Kabuli_denovo	21778525	(G/C)
23789	CakSNP23789	*Kabuli*	Ca_Kabuli_denovo	21778543	(A/G)
23790	CakSNP23790	*Kabuli*	Ca_Kabuli_denovo	21778657	(T/C)
23791	CakSNP23791	*Kabuli*	Ca_Kabuli_denovo	21788450	(C/G)
23792	CakSNP23792	*Kabuli*	Ca_Kabuli_denovo	21788489	(A/G)
23793	CakSNP23793	*Kabuli*	Ca_Kabuli_denovo	21798025	(A/C)
23794	CakSNP23794	*Kabuli*	Ca_Kabuli_denovo	21798919	(T/G)
23795	CakSNP23795	*Kabuli*	Ca_Kabuli_denovo	21800681	(A/G)
23796	CakSNP23796	*Kabuli*	Ca_Kabuli_denovo	21801770	(G/T)
23797	CakSNP23797	*Kabuli*	Ca_Kabuli_denovo	21801797	(C/T)
23798	CakSNP23798	*Kabuli*	Ca_Kabuli_denovo	21804246	(G/A)
23799	CakSNP23799	*Kabuli*	Ca_Kabuli_denovo	21804254	(C/G)
23800	CakSNP23800	*Kabuli*	Ca_Kabuli_denovo	21805673	(C/T)
23801	CakSNP23801	*Kabuli*	Ca_Kabuli_denovo	21807860	(G/T)
23802	CakSNP23802	*Kabuli*	Ca_Kabuli_denovo	21807880	(T/G)
23803	CakSNP23803	*Kabuli*	Ca_Kabuli_denovo	21808391	(C/G)
23804	CakSNP23804	*Kabuli*	Ca_Kabuli_denovo	21811794	(A/C)
23805	CakSNP23805	*Kabuli*	Ca_Kabuli_denovo	21811800	(A/G)
23806	CakSNP23806	*Kabuli*	Ca_Kabuli_denovo	21815122	(G/C)
23807	CakSNP23807	*Kabuli*	Ca_Kabuli_denovo	21815146	(G/A)
23808	CakSNP23808	*Kabuli*	Ca_Kabuli_denovo	21817157	(C/T)
23809	CakSNP23809	*Kabuli*	Ca_Kabuli_denovo	21817167	(A/G)
23810	CakSNP23810	*Kabuli*	Ca_Kabuli_denovo	21818738	(T/C)
23811	CakSNP23811	*Kabuli*	Ca_Kabuli_denovo	21818765	(T/C)
23812	CakSNP23812	*Kabuli*	Ca_Kabuli_denovo	21822406	(T/C)
23813	CakSNP23813	*Kabuli*	Ca_Kabuli_denovo	21822476	(A/G)
23814	CakSNP23814	*Kabuli*	Ca_Kabuli_denovo	21822488	(G/C)
23815	CakSNP23815	*Kabuli*	Ca_Kabuli_denovo	21824609	(T/C)
23816	CakSNP23816	*Kabuli*	Ca_Kabuli_denovo	21824648	(C/G)
23817	CakSNP23817	*Kabuli*	Ca_Kabuli_denovo	21826085	(A/G)
23818	CakSNP23818	*Kabuli*	Ca_Kabuli_denovo	21826147	(C/T)
23819	CakSNP23819	*Kabuli*	Ca_Kabuli_denovo	21827387	(A/C)
23820	CakSNP23820	*Kabuli*	Ca_Kabuli_denovo	21830766	(T/C)
23821	CakSNP23821	*Kabuli*	Ca_Kabuli_denovo	21830792	(A/C)
23822	CakSNP23822	*Kabuli*	Ca_Kabuli_denovo	21832533	(G/A)
23823	CakSNP23823	*Kabuli*	Ca_Kabuli_denovo	21832563	(A/G)
23824	CakSNP23824	*Kabuli*	Ca_Kabuli_denovo	21833724	(A/G)
23825	CakSNP23825	*Kabuli*	Ca_Kabuli_denovo	21833757	(C/T)

S.N.	SNP IDs	Cultivars	Chromosomes/scaffolds	Physical positions (bp)	SNPs
23826	CakSNP23826	*Kabuli*	Ca_Kabuli_denovo	21833915	(T/C)
23827	CakSNP23827	*Kabuli*	Ca_Kabuli_denovo	21839422	(A/G)
23828	CakSNP23828	*Kabuli*	Ca_Kabuli_denovo	21842425	(C/T)
23829	CakSNP23829	*Kabuli*	Ca_Kabuli_denovo	21857124	(G/A)
23830	CakSNP23830	*Kabuli*	Ca_Kabuli_denovo	21860971	(C/G)
23831	CakSNP23831	*Kabuli*	Ca_Kabuli_denovo	21860983	(G/T)
23832	CakSNP23832	*Kabuli*	Ca_Kabuli_denovo	21862785	(A/G)
23833	CakSNP23833	*Kabuli*	Ca_Kabuli_denovo	21863197	(T/G)
23834	CakSNP23834	*Kabuli*	Ca_Kabuli_denovo	21863232	(A/G)
23835	CakSNP23835	*Kabuli*	Ca_Kabuli_denovo	21868496	(C/T)
23836	CakSNP23836	*Kabuli*	Ca_Kabuli_denovo	21876586	(T/C)
23837	CakSNP23837	*Kabuli*	Ca_Kabuli_denovo	21876590	(G/C)
23838	CakSNP23838	*Kabuli*	Ca_Kabuli_denovo	21876611	(G/A)
23839	CakSNP23839	*Kabuli*	Ca_Kabuli_denovo	21880120	(C/T)
23840	CakSNP23840	*Kabuli*	Ca_Kabuli_denovo	21880140	(G/C)
23841	CakSNP23841	*Kabuli*	Ca_Kabuli_denovo	21882551	(C/T)
23842	CakSNP23842	*Kabuli*	Ca_Kabuli_denovo	21883006	(C/G)
23843	CakSNP23843	*Kabuli*	Ca_Kabuli_denovo	21883012	(G/T)
23844	CakSNP23844	*Kabuli*	Ca_Kabuli_denovo	21885351	(G/C)
23845	CakSNP23845	*Kabuli*	Ca_Kabuli_denovo	21886844	(G/A)
23846	CakSNP23846	*Kabuli*	Ca_Kabuli_denovo	21886849	(C/T)
23847	CakSNP23847	*Kabuli*	Ca_Kabuli_denovo	21888586	(G/A)
23848	CakSNP23848	*Kabuli*	Ca_Kabuli_denovo	21893756	(C/T)
23849	CakSNP23849	*Kabuli*	Ca_Kabuli_denovo	21894934	(T/C)
23850	CakSNP23850	*Kabuli*	Ca_Kabuli_denovo	21894955	(A/G)
23851	CakSNP23851	*Kabuli*	Ca_Kabuli_denovo	21898260	(G/A)
23852	CakSNP23852	*Kabuli*	Ca_Kabuli_denovo	21898280	(A/G)
23853	CakSNP23853	*Kabuli*	Ca_Kabuli_denovo	21903472	(C/G)
23854	CakSNP23854	*Kabuli*	Ca_Kabuli_denovo	21903496	(T/C)
23855	CakSNP23855	*Kabuli*	Ca_Kabuli_denovo	21905287	(A/G)
23856	CakSNP23856	*Kabuli*	Ca_Kabuli_denovo	21905310	(G/A)
23857	CakSNP23857	*Kabuli*	Ca_Kabuli_denovo	21907451	(A/C)
23858	CakSNP23858	*Kabuli*	Ca_Kabuli_denovo	21907453	(A/C)
23859	CakSNP23859	*Kabuli*	Ca_Kabuli_denovo	21913758	(T/C)
23860	CakSNP23860	*Kabuli*	Ca_Kabuli_denovo	21915275	(G/A)
23861	CakSNP23861	*Kabuli*	Ca_Kabuli_denovo	21915344	(A/T)
23862	CakSNP23862	*Kabuli*	Ca_Kabuli_denovo	21916354	(G/C)
23863	CakSNP23863	*Kabuli*	Ca_Kabuli_denovo	21916363	(G/C)

S.N.	SNP IDs	Cultivars	Chromosomes/scaffolds	Physical positions (bp)	SNPs
23864	CakSNP23864	*Kabuli*	Ca_Kabuli_denovo	21917642	(C/T)
23865	CakSNP23865	*Kabuli*	Ca_Kabuli_denovo	21923496	(C/T)
23866	CakSNP23866	*Kabuli*	Ca_Kabuli_denovo	21926068	(G/A)
23867	CakSNP23867	*Kabuli*	Ca_Kabuli_denovo	21929425	(G/A)
23868	CakSNP23868	*Kabuli*	Ca_Kabuli_denovo	21931570	(C/T)
23869	CakSNP23869	*Kabuli*	Ca_Kabuli_denovo	21931574	(C/G)
23870	CakSNP23870	*Kabuli*	Ca_Kabuli_denovo	21933266	(G/C)
23871	CakSNP23871	*Kabuli*	Ca_Kabuli_denovo	21943045	(C/T)
23872	CakSNP23872	*Kabuli*	Ca_Kabuli_denovo	21943062	(C/T)
23873	CakSNP23873	*Kabuli*	Ca_Kabuli_denovo	21944105	(T/G)
23874	CakSNP23874	*Kabuli*	Ca_Kabuli_denovo	21947457	(T/C)
23875	CakSNP23875	*Kabuli*	Ca_Kabuli_denovo	21959356	(C/T)
23876	CakSNP23876	*Kabuli*	Ca_Kabuli_denovo	21959383	(T/C)
23877	CakSNP23877	*Kabuli*	Ca_Kabuli_denovo	21959872	(G/T)
23878	CakSNP23878	*Kabuli*	Ca_Kabuli_denovo	21968331	(A/G)
23879	CakSNP23879	*Kabuli*	Ca_Kabuli_denovo	21968856	(T/C)
23880	CakSNP23880	*Kabuli*	Ca_Kabuli_denovo	21968860	(A/G)
23881	CakSNP23881	*Kabuli*	Ca_Kabuli_denovo	21976577	(G/A)
23882	CakSNP23882	*Kabuli*	Ca_Kabuli_denovo	21976623	(C/A)
23883	CakSNP23883	*Kabuli*	Ca_Kabuli_denovo	21978703	(A/C)
23884	CakSNP23884	*Kabuli*	Ca_Kabuli_denovo	21978727	(A/C)
23885	CakSNP23885	*Kabuli*	Ca_Kabuli_denovo	21983999	(A/G)
23886	CakSNP23886	*Kabuli*	Ca_Kabuli_denovo	21984023	(A/G)
23887	CakSNP23887	*Kabuli*	Ca_Kabuli_denovo	21984026	(G/A)
23888	CakSNP23888	*Kabuli*	Ca_Kabuli_denovo	21984665	(A/T)
23889	CakSNP23889	*Kabuli*	Ca_Kabuli_denovo	21984673	(T/C)
23890	CakSNP23890	*Kabuli*	Ca_Kabuli_denovo	21984871	(A/G)
23891	CakSNP23891	*Kabuli*	Ca_Kabuli_denovo	21984916	(A/G)
23892	CakSNP23892	*Kabuli*	Ca_Kabuli_denovo	21993694	(A/G)
23893	CakSNP23893	*Kabuli*	Ca_Kabuli_denovo	21993700	(A/C)
23894	CakSNP23894	*Kabuli*	Ca_Kabuli_denovo	21995519	(T/G)
23895	CakSNP23895	*Kabuli*	Ca_Kabuli_denovo	21999081	(C/G)
23896	CakSNP23896	*Kabuli*	Ca_Kabuli_denovo	21999091	(G/T)
23897	CakSNP23897	*Kabuli*	Ca_Kabuli_denovo	22001325	(C/A)
23898	CakSNP23898	*Kabuli*	Ca_Kabuli_denovo	22001329	(G/A)
23899	CakSNP23899	*Kabuli*	Ca_Kabuli_denovo	22003126	(A/G)
23900	CakSNP23900	*Kabuli*	Ca_Kabuli_denovo	22005228	(A/G)
23901	CakSNP23901	*Kabuli*	Ca_Kabuli_denovo	22010799	(C/A)

S.N.	SNP IDs	Cultivars	Chromosomes/scaffolds	Physical positions (bp)	SNPs
23902	CakSNP23902	*Kabuli*	Ca_Kabuli_denovo	22010803	(A/C)
23903	CakSNP23903	*Kabuli*	Ca_Kabuli_denovo	22011716	(T/G)
23904	CakSNP23904	*Kabuli*	Ca_Kabuli_denovo	22011794	(C/T)
23905	CakSNP23905	*Kabuli*	Ca_Kabuli_denovo	22016922	(A/C)
23906	CakSNP23906	*Kabuli*	Ca_Kabuli_denovo	22020149	(C/T)
23907	CakSNP23907	*Kabuli*	Ca_Kabuli_denovo	22020151	(G/A)
23908	CakSNP23908	*Kabuli*	Ca_Kabuli_denovo	22026815	(G/A)
23909	CakSNP23909	*Kabuli*	Ca_Kabuli_denovo	22027645	(C/T)
23910	CakSNP23910	*Kabuli*	Ca_Kabuli_denovo	22028368	(C/T)
23911	CakSNP23911	*Kabuli*	Ca_Kabuli_denovo	22028371	(A/G)
23912	CakSNP23912	*Kabuli*	Ca_Kabuli_denovo	22028386	(T/C)
23913	CakSNP23913	*Kabuli*	Ca_Kabuli_denovo	22028745	(T/G)
23914	CakSNP23914	*Kabuli*	Ca_Kabuli_denovo	22031438	(T/G)
23915	CakSNP23915	*Kabuli*	Ca_Kabuli_denovo	22040585	(G/T)
23916	CakSNP23916	*Kabuli*	Ca_Kabuli_denovo	22044687	(C/T)
23917	CakSNP23917	*Kabuli*	Ca_Kabuli_denovo	22048634	(C/A)
23918	CakSNP23918	*Kabuli*	Ca_Kabuli_denovo	22048658	(G/A)
23919	CakSNP23919	*Kabuli*	Ca_Kabuli_denovo	22054161	(A/G)
23920	CakSNP23920	*Kabuli*	Ca_Kabuli_denovo	22057228	(T/C)
23921	CakSNP23921	*Kabuli*	Ca_Kabuli_denovo	22059344	(C/G)
23922	CakSNP23922	*Kabuli*	Ca_Kabuli_denovo	22059356	(A/G)
23923	CakSNP23923	*Kabuli*	Ca_Kabuli_denovo	22070684	(T/G)
23924	CakSNP23924	*Kabuli*	Ca_Kabuli_denovo	22071511	(G/A)
23925	CakSNP23925	*Kabuli*	Ca_Kabuli_denovo	22072977	(T/G)
23926	CakSNP23926	*Kabuli*	Ca_Kabuli_denovo	22074563	(A/T)
23927	CakSNP23927	*Kabuli*	Ca_Kabuli_denovo	22078330	(T/C)
23928	CakSNP23928	*Kabuli*	Ca_Kabuli_denovo	22078518	(T/C)
23929	CakSNP23929	*Kabuli*	Ca_Kabuli_denovo	22078563	(G/C)
23930	CakSNP23930	*Kabuli*	Ca_Kabuli_denovo	22082588	(T/C)
23931	CakSNP23931	*Kabuli*	Ca_Kabuli_denovo	22082621	(C/T)
23932	CakSNP23932	*Kabuli*	Ca_Kabuli_denovo	22083454	(C/T)
23933	CakSNP23933	*Kabuli*	Ca_Kabuli_denovo	22086659	(T/G)
23934	CakSNP23934	*Kabuli*	Ca_Kabuli_denovo	22093836	(G/A)
23935	CakSNP23935	*Kabuli*	Ca_Kabuli_denovo	22095169	(C/T)
23936	CakSNP23936	*Kabuli*	Ca_Kabuli_denovo	22099049	(A/T)
23937	CakSNP23937	*Kabuli*	Ca_Kabuli_denovo	22099091	(G/A)
23938	CakSNP23938	*Kabuli*	Ca_Kabuli_denovo	22105626	(T/C)
23939	CakSNP23939	*Kabuli*	Ca_Kabuli_denovo	22109925	(C/G)

S.N.	SNP IDs	Cultivars	Chromosomes/scaffolds	Physical positions (bp)	SNPs
23940	CakSNP23940	*Kabuli*	Ca_Kabuli_denovo	22109955	(T/C)
23941	CakSNP23941	*Kabuli*	Ca_Kabuli_denovo	22115972	(C/G)
23942	CakSNP23942	*Kabuli*	Ca_Kabuli_denovo	22127527	(C/G)
23943	CakSNP23943	*Kabuli*	Ca_Kabuli_denovo	22145936	(C/A)
23944	CakSNP23944	*Kabuli*	Ca_Kabuli_denovo	22145951	(A/G)
23945	CakSNP23945	*Kabuli*	Ca_Kabuli_denovo	22147873	(A/G)
23946	CakSNP23946	*Kabuli*	Ca_Kabuli_denovo	22150196	(A/G)
23947	CakSNP23947	*Kabuli*	Ca_Kabuli_denovo	22150199	(G/C)
23948	CakSNP23948	*Kabuli*	Ca_Kabuli_denovo	22154593	(C/T)
23949	CakSNP23949	*Kabuli*	Ca_Kabuli_denovo	22155073	(T/A)
23950	CakSNP23950	*Kabuli*	Ca_Kabuli_denovo	22155343	(G/C)
23951	CakSNP23951	*Kabuli*	Ca_Kabuli_denovo	22155358	(C/A)
23952	CakSNP23952	*Kabuli*	Ca_Kabuli_denovo	22156352	(G/A)
23953	CakSNP23953	*Kabuli*	Ca_Kabuli_denovo	22156384	(C/G)
23954	CakSNP23954	*Kabuli*	Ca_Kabuli_denovo	22162484	(A/G)
23955	CakSNP23955	*Kabuli*	Ca_Kabuli_denovo	22166305	(T/C)
23956	CakSNP23956	*Kabuli*	Ca_Kabuli_denovo	22175865	(C/T)
23957	CakSNP23957	*Kabuli*	Ca_Kabuli_denovo	22175884	(T/C)
23958	CakSNP23958	*Kabuli*	Ca_Kabuli_denovo	22177441	(A/C)
23959	CakSNP23959	*Kabuli*	Ca_Kabuli_denovo	22177732	(T/C)
23960	CakSNP23960	*Kabuli*	Ca_Kabuli_denovo	22177762	(T/C)
23961	CakSNP23961	*Kabuli*	Ca_Kabuli_denovo	22185743	(C/T)
23962	CakSNP23962	*Kabuli*	Ca_Kabuli_denovo	22185763	(T/G)
23963	CakSNP23963	*Kabuli*	Ca_Kabuli_denovo	22187682	(T/C)
23964	CakSNP23964	*Kabuli*	Ca_Kabuli_denovo	22187734	(G/T)
23965	CakSNP23965	*Kabuli*	Ca_Kabuli_denovo	22191206	(C/A)
23966	CakSNP23966	*Kabuli*	Ca_Kabuli_denovo	22191233	(G/C)
23967	CakSNP23967	*Kabuli*	Ca_Kabuli_denovo	22194019	(T/C)
23968	CakSNP23968	*Kabuli*	Ca_Kabuli_denovo	22194078	(G/C)
23969	CakSNP23969	*Kabuli*	Ca_Kabuli_denovo	22199275	(T/C)
23970	CakSNP23970	*Kabuli*	Ca_Kabuli_denovo	22204436	(G/A)
23971	CakSNP23971	*Kabuli*	Ca_Kabuli_denovo	22204475	(A/G)
23972	CakSNP23972	*Kabuli*	Ca_Kabuli_denovo	22206003	(G/A)
23973	CakSNP23973	*Kabuli*	Ca_Kabuli_denovo	22210115	(C/G)
23974	CakSNP23974	*Kabuli*	Ca_Kabuli_denovo	22213095	(C/A)
23975	CakSNP23975	*Kabuli*	Ca_Kabuli_denovo	22213116	(A/G)
23976	CakSNP23976	*Kabuli*	Ca_Kabuli_denovo	22213901	(T/C)
23977	CakSNP23977	*Kabuli*	Ca_Kabuli_denovo	22213930	(G/C)

S.N.	SNP IDs	Cultivars	Chromosomes/scaffolds	Physical positions (bp)	SNPs
23978	CakSNP23978	*Kabuli*	Ca_Kabuli_denovo	22216043	(A/G)
23979	CakSNP23979	*Kabuli*	Ca_Kabuli_denovo	22223582	(G/A)
23980	CakSNP23980	*Kabuli*	Ca_Kabuli_denovo	22223614	(T/C)
23981	CakSNP23981	*Kabuli*	Ca_Kabuli_denovo	22223700	(T/A)
23982	CakSNP23982	*Kabuli*	Ca_Kabuli_denovo	22223707	(A/C)
23983	CakSNP23983	*Kabuli*	Ca_Kabuli_denovo	22224352	(G/T)
23984	CakSNP23984	*Kabuli*	Ca_Kabuli_denovo	22224391	(G/T)
23985	CakSNP23985	*Kabuli*	Ca_Kabuli_denovo	22236255	(C/T)
23986	CakSNP23986	*Kabuli*	Ca_Kabuli_denovo	22236273	(T/G)
23987	CakSNP23987	*Kabuli*	Ca_Kabuli_denovo	22237102	(G/A)
23988	CakSNP23988	*Kabuli*	Ca_Kabuli_denovo	22242259	(C/T)
23989	CakSNP23989	*Kabuli*	Ca_Kabuli_denovo	22242265	(C/T)
23990	CakSNP23990	*Kabuli*	Ca_Kabuli_denovo	22258645	(A/G)
23991	CakSNP23991	*Kabuli*	Ca_Kabuli_denovo	22266067	(G/C)
23992	CakSNP23992	*Kabuli*	Ca_Kabuli_denovo	22274978	(T/C)
23993	CakSNP23993	*Kabuli*	Ca_Kabuli_denovo	22277631	(G/C)
23994	CakSNP23994	*Kabuli*	Ca_Kabuli_denovo	22277682	(C/T)
23995	CakSNP23995	*Kabuli*	Ca_Kabuli_denovo	22279871	(C/A)
23996	CakSNP23996	*Kabuli*	Ca_Kabuli_denovo	22279916	(C/T)
23997	CakSNP23997	*Kabuli*	Ca_Kabuli_denovo	22280798	(G/A)
23998	CakSNP23998	*Kabuli*	Ca_Kabuli_denovo	22288948	(T/C)
23999	CakSNP23999	*Kabuli*	Ca_Kabuli_denovo	22290167	(C/A)
24000	CakSNP24000	*Kabuli*	Ca_Kabuli_denovo	22290169	(A/G)
24001	CakSNP24001	*Kabuli*	Ca_Kabuli_denovo	22297049	(C/A)
24002	CakSNP24002	*Kabuli*	Ca_Kabuli_denovo	22297053	(G/C)
24003	CakSNP24003	*Kabuli*	Ca_Kabuli_denovo	22297315	(T/C)
24004	CakSNP24004	*Kabuli*	Ca_Kabuli_denovo	22297334	(A/G)
24005	CakSNP24005	*Kabuli*	Ca_Kabuli_denovo	22302898	(G/A)
24006	CakSNP24006	*Kabuli*	Ca_Kabuli_denovo	22309863	(A/C)
24007	CakSNP24007	*Kabuli*	Ca_Kabuli_denovo	22315446	(A/T)
24008	CakSNP24008	*Kabuli*	Ca_Kabuli_denovo	22315449	(A/C)
24009	CakSNP24009	*Kabuli*	Ca_Kabuli_denovo	22324880	(T/G)
24010	CakSNP24010	*Kabuli*	Ca_Kabuli_denovo	22324883	(G/A)
24011	CakSNP24011	*Kabuli*	Ca_Kabuli_denovo	22328246	(A/G)
24012	CakSNP24012	*Kabuli*	Ca_Kabuli_denovo	22328273	(C/T)
24013	CakSNP24013	*Kabuli*	Ca_Kabuli_denovo	22332214	(G/T)
24014	CakSNP24014	*Kabuli*	Ca_Kabuli_denovo	22337605	(C/A)
24015	CakSNP24015	*Kabuli*	Ca_Kabuli_denovo	22340311	(C/A)

S.N.	SNP IDs	Cultivars	Chromosomes/scaffolds	Physical positions (bp)	SNPs
24016	CakSNP24016	*Kabuli*	Ca_Kabuli_denovo	22340317	(T/G)
24017	CakSNP24017	*Kabuli*	Ca_Kabuli_denovo	22342839	(G/C)
24018	CakSNP24018	*Kabuli*	Ca_Kabuli_denovo	22343917	(T/C)
24019	CakSNP24019	*Kabuli*	Ca_Kabuli_denovo	22343947	(A/C)
24020	CakSNP24020	*Kabuli*	Ca_Kabuli_denovo	22344811	(T/C)
24021	CakSNP24021	*Kabuli*	Ca_Kabuli_denovo	22347675	(T/C)
24022	CakSNP24022	*Kabuli*	Ca_Kabuli_denovo	22355630	(A/G)
24023	CakSNP24023	*Kabuli*	Ca_Kabuli_denovo	22355651	(A/C)
24024	CakSNP24024	*Kabuli*	Ca_Kabuli_denovo	22356787	(T/C)
24025	CakSNP24025	*Kabuli*	Ca_Kabuli_denovo	22359477	(T/A)
24026	CakSNP24026	*Kabuli*	Ca_Kabuli_denovo	22364608	(C/T)
24027	CakSNP24027	*Kabuli*	Ca_Kabuli_denovo	22370629	(G/A)
24028	CakSNP24028	*Kabuli*	Ca_Kabuli_denovo	22370640	(C/T)
24029	CakSNP24029	*Kabuli*	Ca_Kabuli_denovo	22379899	(G/C)
24030	CakSNP24030	*Kabuli*	Ca_Kabuli_denovo	22383680	(A/G)
24031	CakSNP24031	*Kabuli*	Ca_Kabuli_denovo	22384415	(A/C)
24032	CakSNP24032	*Kabuli*	Ca_Kabuli_denovo	22388447	(T/G)
24033	CakSNP24033	*Kabuli*	Ca_Kabuli_denovo	22390212	(A/C)
24034	CakSNP24034	*Kabuli*	Ca_Kabuli_denovo	22390214	(A/C)
24035	CakSNP24035	*Kabuli*	Ca_Kabuli_denovo	22396027	(G/A)
24036	CakSNP24036	*Kabuli*	Ca_Kabuli_denovo	22399918	(G/C)
24037	CakSNP24037	*Kabuli*	Ca_Kabuli_denovo	22402729	(A/G)
24038	CakSNP24038	*Kabuli*	Ca_Kabuli_denovo	22409040	(G/A)
24039	CakSNP24039	*Kabuli*	Ca_Kabuli_denovo	22409090	(T/G)
24040	CakSNP24040	*Kabuli*	Ca_Kabuli_denovo	22415065	(A/G)
24041	CakSNP24041	*Kabuli*	Ca_Kabuli_denovo	22415068	(T/C)
24042	CakSNP24042	*Kabuli*	Ca_Kabuli_denovo	22421870	(G/A)
24043	CakSNP24043	*Kabuli*	Ca_Kabuli_denovo	22423241	(C/G)
24044	CakSNP24044	*Kabuli*	Ca_Kabuli_denovo	22423263	(G/T)
24045	CakSNP24045	*Kabuli*	Ca_Kabuli_denovo	22424926	(G/C)
24046	CakSNP24046	*Kabuli*	Ca_Kabuli_denovo	22426360	(C/G)
24047	CakSNP24047	*Kabuli*	Ca_Kabuli_denovo	22427046	(A/T)
24048	CakSNP24048	*Kabuli*	Ca_Kabuli_denovo	22431626	(C/A)
24049	CakSNP24049	*Kabuli*	Ca_Kabuli_denovo	22431652	(C/T)
24050	CakSNP24050	*Kabuli*	Ca_Kabuli_denovo	22432380	(C/T)
24051	CakSNP24051	*Kabuli*	Ca_Kabuli_denovo	22432382	(C/G)
24052	CakSNP24052	*Kabuli*	Ca_Kabuli_denovo	22433280	(G/A)
24053	CakSNP24053	*Kabuli*	Ca_Kabuli_denovo	22433295	(A/G)

S.N.	SNP IDs	Cultivars	Chromosomes/scaffolds	Physical positions (bp)	SNPs
24054	CakSNP24054	*Kabuli*	Ca_Kabuli_denovo	22436951	(C/G)
24055	CakSNP24055	*Kabuli*	Ca_Kabuli_denovo	22436975	(T/G)
24056	CakSNP24056	*Kabuli*	Ca_Kabuli_denovo	22439163	(C/T)
24057	CakSNP24057	*Kabuli*	Ca_Kabuli_denovo	22443182	(T/C)
24058	CakSNP24058	*Kabuli*	Ca_Kabuli_denovo	22444030	(C/G)
24059	CakSNP24059	*Kabuli*	Ca_Kabuli_denovo	22449467	(G/T)
24060	CakSNP24060	*Kabuli*	Ca_Kabuli_denovo	22449512	(C/G)
24061	CakSNP24061	*Kabuli*	Ca_Kabuli_denovo	22450397	(C/G)
24062	CakSNP24062	*Kabuli*	Ca_Kabuli_denovo	22451704	(C/T)
24063	CakSNP24063	*Kabuli*	Ca_Kabuli_denovo	22452438	(A/G)
24064	CakSNP24064	*Kabuli*	Ca_Kabuli_denovo	22452449	(C/G)
24065	CakSNP24065	*Kabuli*	Ca_Kabuli_denovo	22453019	(T/C)
24066	CakSNP24066	*Kabuli*	Ca_Kabuli_denovo	22453049	(A/G)
24067	CakSNP24067	*Kabuli*	Ca_Kabuli_denovo	22453067	(G/A)
24068	CakSNP24068	*Kabuli*	Ca_Kabuli_denovo	22454097	(A/G)
24069	CakSNP24069	*Kabuli*	Ca_Kabuli_denovo	22454100	(G/A)
24070	CakSNP24070	*Kabuli*	Ca_Kabuli_denovo	22462238	(C/G)
24071	CakSNP24071	*Kabuli*	Ca_Kabuli_denovo	22462279	(T/C)
24072	CakSNP24072	*Kabuli*	Ca_Kabuli_denovo	22463261	(G/C)
24073	CakSNP24073	*Kabuli*	Ca_Kabuli_denovo	22464550	(C/A)
24074	CakSNP24074	*Kabuli*	Ca_Kabuli_denovo	22464930	(T/C)
24075	CakSNP24075	*Kabuli*	Ca_Kabuli_denovo	22464938	(G/C)
24076	CakSNP24076	*Kabuli*	Ca_Kabuli_denovo	22465107	(T/C)
24077	CakSNP24077	*Kabuli*	Ca_Kabuli_denovo	22465162	(A/G)
24078	CakSNP24078	*Kabuli*	Ca_Kabuli_denovo	22466096	(G/T)
24079	CakSNP24079	*Kabuli*	Ca_Kabuli_denovo	22476717	(A/C)
24080	CakSNP24080	*Kabuli*	Ca_Kabuli_denovo	22476744	(T/C)
24081	CakSNP24081	*Kabuli*	Ca_Kabuli_denovo	22479050	(A/G)
24082	CakSNP24082	*Kabuli*	Ca_Kabuli_denovo	22479122	(T/C)
24083	CakSNP24083	*Kabuli*	Ca_Kabuli_denovo	22480360	(C/T)
24084	CakSNP24084	*Kabuli*	Ca_Kabuli_denovo	22480405	(C/T)
24085	CakSNP24085	*Kabuli*	Ca_Kabuli_denovo	22486514	(G/A)
24086	CakSNP24086	*Kabuli*	Ca_Kabuli_denovo	22486522	(C/T)
24087	CakSNP24087	*Kabuli*	Ca_Kabuli_denovo	22493767	(T/C)
24088	CakSNP24088	*Kabuli*	Ca_Kabuli_denovo	22504660	(C/T)
24089	CakSNP24089	*Kabuli*	Ca_Kabuli_denovo	22514807	(C/A)
24090	CakSNP24090	*Kabuli*	Ca_Kabuli_denovo	22516434	(A/G)
24091	CakSNP24091	*Kabuli*	Ca_Kabuli_denovo	22520823	(G/C)

S.N.	SNP IDs	Cultivars	Chromosomes/scaffolds	Physical positions (bp)	SNPs
24092	CakSNP24092	*Kabuli*	Ca_Kabuli_denovo	22520875	(T/C)
24093	CakSNP24093	*Kabuli*	Ca_Kabuli_denovo	22521766	(G/C)
24094	CakSNP24094	*Kabuli*	Ca_Kabuli_denovo	22521796	(C/T)
24095	CakSNP24095	*Kabuli*	Ca_Kabuli_denovo	22529928	(A/G)
24096	CakSNP24096	*Kabuli*	Ca_Kabuli_denovo	22531422	(A/C)
24097	CakSNP24097	*Kabuli*	Ca_Kabuli_denovo	22531463	(A/G)
24098	CakSNP24098	*Kabuli*	Ca_Kabuli_denovo	22534289	(A/G)
24099	CakSNP24099	*Kabuli*	Ca_Kabuli_denovo	22542381	(T/G)
24100	CakSNP24100	*Kabuli*	Ca_Kabuli_denovo	22542441	(G/A)
24101	CakSNP24101	*Kabuli*	Ca_Kabuli_denovo	22546566	(A/C)
24102	CakSNP24102	*Kabuli*	Ca_Kabuli_denovo	22556801	(T/C)
24103	CakSNP24103	*Kabuli*	Ca_Kabuli_denovo	22561762	(G/C)
24104	CakSNP24104	*Kabuli*	Ca_Kabuli_denovo	22561771	(T/G)
24105	CakSNP24105	*Kabuli*	Ca_Kabuli_denovo	22567017	(T/C)
24106	CakSNP24106	*Kabuli*	Ca_Kabuli_denovo	22567026	(G/A)
24107	CakSNP24107	*Kabuli*	Ca_Kabuli_denovo	22567059	(T/C)
24108	CakSNP24108	*Kabuli*	Ca_Kabuli_denovo	22567219	(T/C)
24109	CakSNP24109	*Kabuli*	Ca_Kabuli_denovo	22567221	(C/T)
24110	CakSNP24110	*Kabuli*	Ca_Kabuli_denovo	22568144	(C/G)
24111	CakSNP24111	*Kabuli*	Ca_Kabuli_denovo	22568186	(C/A)
24112	CakSNP24112	*Kabuli*	Ca_Kabuli_denovo	22568434	(C/G)
24113	CakSNP24113	*Kabuli*	Ca_Kabuli_denovo	22578643	(C/T)
24114	CakSNP24114	*Kabuli*	Ca_Kabuli_denovo	22579945	(G/C)
24115	CakSNP24115	*Kabuli*	Ca_Kabuli_denovo	22581626	(T/C)
24116	CakSNP24116	*Kabuli*	Ca_Kabuli_denovo	22586002	(C/T)
24117	CakSNP24117	*Kabuli*	Ca_Kabuli_denovo	22590746	(G/A)
24118	CakSNP24118	*Kabuli*	Ca_Kabuli_denovo	22590761	(T/C)
24119	CakSNP24119	*Kabuli*	Ca_Kabuli_denovo	22602100	(C/T)
24120	CakSNP24120	*Kabuli*	Ca_Kabuli_denovo	22614086	(C/T)
24121	CakSNP24121	*Kabuli*	Ca_Kabuli_denovo	22614088	(C/G)
24122	CakSNP24122	*Kabuli*	Ca_Kabuli_denovo	22615811	(G/T)
24123	CakSNP24123	*Kabuli*	Ca_Kabuli_denovo	22635214	(G/C)
24124	CakSNP24124	*Kabuli*	Ca_Kabuli_denovo	22635612	(T/C)
24125	CakSNP24125	*Kabuli*	Ca_Kabuli_denovo	22635633	(G/A)
24126	CakSNP24126	*Kabuli*	Ca_Kabuli_denovo	22639106	(A/G)
24127	CakSNP24127	*Kabuli*	Ca_Kabuli_denovo	22639177	(T/C)
24128	CakSNP24128	*Kabuli*	Ca_Kabuli_denovo	22642069	(G/C)
24129	CakSNP24129	*Kabuli*	Ca_Kabuli_denovo	22643291	(C/G)

S.N.	SNP IDs	Cultivars	Chromosomes/scaffolds	Physical positions (bp)	SNPs
24130	CakSNP24130	*Kabuli*	Ca_Kabuli_denovo	22643294	(C/G)
24131	CakSNP24131	*Kabuli*	Ca_Kabuli_denovo	22647446	(C/G)
24132	CakSNP24132	*Kabuli*	Ca_Kabuli_denovo	22647449	(T/C)
24133	CakSNP24133	*Kabuli*	Ca_Kabuli_denovo	22650472	(A/G)
24134	CakSNP24134	*Kabuli*	Ca_Kabuli_denovo	22651126	(A/G)
24135	CakSNP24135	*Kabuli*	Ca_Kabuli_denovo	22651157	(A/G)
24136	CakSNP24136	*Kabuli*	Ca_Kabuli_denovo	22658903	(C/T)
24137	CakSNP24137	*Kabuli*	Ca_Kabuli_denovo	22658906	(C/T)
24138	CakSNP24138	*Kabuli*	Ca_Kabuli_denovo	22663217	(G/C)
24139	CakSNP24139	*Kabuli*	Ca_Kabuli_denovo	22663232	(T/C)
24140	CakSNP24140	*Kabuli*	Ca_Kabuli_denovo	22665794	(C/G)
24141	CakSNP24141	*Kabuli*	Ca_Kabuli_denovo	22667929	(T/C)
24142	CakSNP24142	*Kabuli*	Ca_Kabuli_denovo	22698389	(C/G)
24143	CakSNP24143	*Kabuli*	Ca_Kabuli_denovo	22701613	(A/T)
24144	CakSNP24144	*Kabuli*	Ca_Kabuli_denovo	22701626	(C/T)
24145	CakSNP24145	*Kabuli*	Ca_Kabuli_denovo	22704938	(C/G)
24146	CakSNP24146	*Kabuli*	Ca_Kabuli_denovo	22706294	(A/G)
24147	CakSNP24147	*Kabuli*	Ca_Kabuli_denovo	22706309	(G/A)
24148	CakSNP24148	*Kabuli*	Ca_Kabuli_denovo	22711654	(T/C)
24149	CakSNP24149	*Kabuli*	Ca_Kabuli_denovo	22712338	(G/C)
24150	CakSNP24150	*Kabuli*	Ca_Kabuli_denovo	22722943	(T/C)
24151	CakSNP24151	*Kabuli*	Ca_Kabuli_denovo	22722952	(G/C)
24152	CakSNP24152	*Kabuli*	Ca_Kabuli_denovo	22725798	(G/C)
24153	CakSNP24153	*Kabuli*	Ca_Kabuli_denovo	22725833	(G/T)
24154	CakSNP24154	*Kabuli*	Ca_Kabuli_denovo	22733117	(A/G)
24155	CakSNP24155	*Kabuli*	Ca_Kabuli_denovo	22733125	(C/G)
24156	CakSNP24156	*Kabuli*	Ca_Kabuli_denovo	22735553	(A/G)
24157	CakSNP24157	*Kabuli*	Ca_Kabuli_denovo	22735578	(A/G)
24158	CakSNP24158	*Kabuli*	Ca_Kabuli_denovo	22749173	(G/T)
24159	CakSNP24159	*Kabuli*	Ca_Kabuli_denovo	22749177	(G/C)
24160	CakSNP24160	*Kabuli*	Ca_Kabuli_denovo	22749186	(T/G)
24161	CakSNP24161	*Kabuli*	Ca_Kabuli_denovo	22762717	(C/G)
24162	CakSNP24162	*Kabuli*	Ca_Kabuli_denovo	22762805	(T/G)
24163	CakSNP24163	*Kabuli*	Ca_Kabuli_denovo	22762824	(G/T)
24164	CakSNP24164	*Kabuli*	Ca_Kabuli_denovo	22762857	(C/G)
24165	CakSNP24165	*Kabuli*	Ca_Kabuli_denovo	22767279	(T/G)
24166	CakSNP24166	*Kabuli*	Ca_Kabuli_denovo	22767322	(C/A)
24167	CakSNP24167	*Kabuli*	Ca_Kabuli_denovo	22770414	(T/C)

S.N.	SNP IDs	Cultivars	Chromosomes/scaffolds	Physical positions (bp)	SNPs
24168	CakSNP24168	*Kabuli*	Ca_Kabuli_denovo	22770468	(A/G)
24169	CakSNP24169	*Kabuli*	Ca_Kabuli_denovo	22772193	(G/C)
24170	CakSNP24170	*Kabuli*	Ca_Kabuli_denovo	22772208	(T/G)
24171	CakSNP24171	*Kabuli*	Ca_Kabuli_denovo	22774872	(A/G)
24172	CakSNP24172	*Kabuli*	Ca_Kabuli_denovo	22774887	(C/A)
24173	CakSNP24173	*Kabuli*	Ca_Kabuli_denovo	22774929	(T/G)
24174	CakSNP24174	*Kabuli*	Ca_Kabuli_denovo	22774938	(C/G)
24175	CakSNP24175	*Kabuli*	Ca_Kabuli_denovo	22779099	(G/T)
24176	CakSNP24176	*Kabuli*	Ca_Kabuli_denovo	22786218	(T/G)
24177	CakSNP24177	*Kabuli*	Ca_Kabuli_denovo	22786232	(A/G)
24178	CakSNP24178	*Kabuli*	Ca_Kabuli_denovo	22786258	(G/A)
24179	CakSNP24179	*Kabuli*	Ca_Kabuli_denovo	22786269	(A/G)
24180	CakSNP24180	*Kabuli*	Ca_Kabuli_denovo	23389902	(A/G)
24181	CakSNP24181	*Kabuli*	Ca_Kabuli_denovo	26279213	(G/C)
24182	CakSNP24182	*Kabuli*	Ca_Kabuli_denovo	26279215	(G/T)
24183	CakSNP24183	*Kabuli*	Ca_Kabuli_denovo	26279249	(T/C)
24184	CakSNP24184	*Kabuli*	Ca_Kabuli_denovo	28463588	(A/G)
24185	CakSNP24185	*Kabuli*	Ca_Kabuli_denovo	28463605	(G/A)
24186	CakSNP24186	*Kabuli*	Ca_Kabuli_denovo	30521758	(C/T)
24187	CakSNP24187	*Kabuli*	Ca_Kabuli_denovo	30521760	(T/C)
24188	CakSNP24188	*Kabuli*	Ca_Kabuli_denovo	30521761	(G/A)
24189	CakSNP24189	*Kabuli*	Ca_Kabuli_denovo	30521763	(T/C)
24190	CakSNP24190	*Kabuli*	Ca_Kabuli_denovo	30542416	(G/A)
24191	CakSNP24191	*Kabuli*	Ca_Kabuli_denovo	30542450	(A/G)
24192	CakSNP24192	*Kabuli*	Ca_Kabuli_denovo	30542467	(C/G)
24193	CakSNP24193	*Kabuli*	Ca_Kabuli_denovo	30542477	(A/G)
24194	CakSNP24194	*Kabuli*	Ca_Kabuli_denovo	30544320	(T/G)
24195	CakSNP24195	*Kabuli*	Ca_Kabuli_denovo	30544340	(T/C)
24196	CakSNP24196	*Kabuli*	Ca_Kabuli_denovo	30544349	(A/G)
24197	CakSNP24197	*Kabuli*	Ca_Kabuli_denovo	30544978	(T/A)
24198	CakSNP24198	*Kabuli*	Ca_Kabuli_denovo	30544987	(T/A)
24199	CakSNP24199	*Kabuli*	Ca_Kabuli_denovo	30544997	(G/C)
24200	CakSNP24200	*Kabuli*	Ca_Kabuli_denovo	30556074	(T/C)
24201	CakSNP24201	*Kabuli*	Ca_Kabuli_denovo	30556076	(T/C)
24202	CakSNP24202	*Kabuli*	Ca_Kabuli_denovo	30556080	(T/A)
24203	CakSNP24203	*Kabuli*	Ca_Kabuli_denovo	30556081	(C/A)
24204	CakSNP24204	*Kabuli*	Ca_Kabuli_denovo	30562605	(A/G)
24205	CakSNP24205	*Kabuli*	Ca_Kabuli_denovo	30562663	(G/A)

S.N.	SNP IDs	Cultivars	Chromosomes/scaffolds	Physical positions (bp)	SNPs
24206	CakSNP24206	*Kabuli*	Ca_Kabuli_denovo	30564215	(C/G)
24207	CakSNP24207	*Kabuli*	Ca_Kabuli_denovo	30564217	(T/G)
24208	CakSNP24208	*Kabuli*	Ca_Kabuli_denovo	30565114	(T/G)
24209	CakSNP24209	*Kabuli*	Ca_Kabuli_denovo	30565484	(C/A)
24210	CakSNP24210	*Kabuli*	Ca_Kabuli_denovo	30565552	(G/T)
24211	CakSNP24211	*Kabuli*	Ca_Kabuli_denovo	30585112	(C/G)
24212	CakSNP24212	*Kabuli*	Ca_Kabuli_denovo	30595715	(C/G)
24213	CakSNP24213	*Kabuli*	Ca_Kabuli_denovo	30595726	(C/G)
24214	CakSNP24214	*Kabuli*	Ca_Kabuli_denovo	30595772	(A/G)
24215	CakSNP24215	*Kabuli*	Ca_Kabuli_denovo	30595815	(C/A)
24216	CakSNP24216	*Kabuli*	Ca_Kabuli_denovo	30595832	(T/A)
24217	CakSNP24217	*Kabuli*	Ca_Kabuli_denovo	30608911	(G/A)
24218	CakSNP24218	*Kabuli*	Ca_Kabuli_denovo	30608992	(G/T)
24219	CakSNP24219	*Kabuli*	Ca_Kabuli_denovo	30608994	(T/C)
24220	CakSNP24220	*Kabuli*	Ca_Kabuli_denovo	30624438	(A/T)
24221	CakSNP24221	*Kabuli*	Ca_Kabuli_denovo	30624449	(G/A)
24222	CakSNP24222	*Kabuli*	Ca_Kabuli_denovo	30654546	(A/G)
24223	CakSNP24223	*Kabuli*	Ca_Kabuli_denovo	30654549	(A/C)
24224	CakSNP24224	*Kabuli*	Ca_Kabuli_denovo	30654551	(C/G)
24225	CakSNP24225	*Kabuli*	Ca_Kabuli_denovo	30654552	(T/G)
24226	CakSNP24226	*Kabuli*	Ca_Kabuli_denovo	30689389	(C/T)
24227	CakSNP24227	*Kabuli*	Ca_Kabuli_denovo	30692542	(C/T)
24228	CakSNP24228	*Kabuli*	Ca_Kabuli_denovo	30701261	(G/A)
24229	CakSNP24229	*Kabuli*	Ca_Kabuli_denovo	30701345	(T/A)
24230	CakSNP24230	*Kabuli*	Ca_Kabuli_denovo	30713916	(A/G)
24231	CakSNP24231	*Kabuli*	Ca_Kabuli_denovo	30713943	(A/G)
24232	CakSNP24232	*Kabuli*	Ca_Kabuli_denovo	30717672	(T/G)
24233	CakSNP24233	*Kabuli*	Ca_Kabuli_denovo	30720426	(T/G)
24234	CakSNP24234	*Kabuli*	Ca_Kabuli_denovo	30720438	(G/T)
24235	CakSNP24235	*Kabuli*	Ca_Kabuli_denovo	30720473	(A/G)
24236	CakSNP24236	*Kabuli*	Ca_Kabuli_denovo	30722929	(G/T)
24237	CakSNP24237	*Kabuli*	Ca_Kabuli_denovo	30722957	(T/G)
24238	CakSNP24238	*Kabuli*	Ca_Kabuli_denovo	30722963	(A/G)
24239	CakSNP24239	*Kabuli*	Ca_Kabuli_denovo	30722980	(G/A)
24240	CakSNP24240	*Kabuli*	Ca_Kabuli_denovo	30726959	(G/A)
24241	CakSNP24241	*Kabuli*	Ca_Kabuli_denovo	30726961	(G/A)
24242	CakSNP24242	*Kabuli*	Ca_Kabuli_denovo	30726962	(A/G)
24243	CakSNP24243	*Kabuli*	Ca_Kabuli_denovo	30726963	(G/A)

S.N.	SNP IDs	Cultivars	Chromosomes/scaffolds	Physical positions (bp)	SNPs
24244	CakSNP24244	*Kabuli*	Ca_Kabuli_denovo	30728437	(T/G)
24245	CakSNP24245	*Kabuli*	Ca_Kabuli_denovo	30728439	(A/G)
24246	CakSNP24246	*Kabuli*	Ca_Kabuli_denovo	30728469	(A/G)
24247	CakSNP24247	*Kabuli*	Ca_Kabuli_denovo	30730294	(G/C)
24248	CakSNP24248	*Kabuli*	Ca_Kabuli_denovo	30730741	(C/A)
24249	CakSNP24249	*Kabuli*	Ca_Kabuli_denovo	30730742	(C/G)
24250	CakSNP24250	*Kabuli*	Ca_Kabuli_denovo	30730743	(A/C)
24251	CakSNP24251	*Kabuli*	Ca_Kabuli_denovo	30730744	(G/A)
24252	CakSNP24252	*Kabuli*	Ca_Kabuli_denovo	30730790	(A/C)
24253	CakSNP24253	*Kabuli*	Ca_Kabuli_denovo	30731368	(C/G)
24254	CakSNP24254	*Kabuli*	Ca_Kabuli_denovo	30731905	(T/G)
24255	CakSNP24255	*Kabuli*	Ca_Kabuli_denovo	30731938	(T/G)
24256	CakSNP24256	*Kabuli*	Ca_Kabuli_denovo	30733630	(T/G)
24257	CakSNP24257	*Kabuli*	Ca_Kabuli_denovo	30733690	(C/G)
24258	CakSNP24258	*Kabuli*	Ca_Kabuli_denovo	30733694	(A/T)
24259	CakSNP24259	*Kabuli*	Ca_Kabuli_denovo	30733703	(G/T)
24260	CakSNP24260	*Kabuli*	Ca_Kabuli_denovo	30742989	(T/G)
24261	CakSNP24261	*Kabuli*	Ca_Kabuli_denovo	30742991	(A/C)
24262	CakSNP24262	*Kabuli*	Ca_Kabuli_denovo	30742993	(A/C)
24263	CakSNP24263	*Kabuli*	Ca_Kabuli_denovo	30742994	(G/A)
24264	CakSNP24264	*Kabuli*	Ca_Kabuli_denovo	30742995	(A/G)
24265	CakSNP24265	*Kabuli*	Ca_Kabuli_denovo	30746022	(G/A)
24266	CakSNP24266	*Kabuli*	Ca_Kabuli_denovo	30746046	(A/G)
24267	CakSNP24267	*Kabuli*	Ca_Kabuli_denovo	30752607	(A/G)
24268	CakSNP24268	*Kabuli*	Ca_Kabuli_denovo	30756090	(A/G)
24269	CakSNP24269	*Kabuli*	Ca_Kabuli_denovo	30756108	(A/C)
24270	CakSNP24270	*Kabuli*	Ca_Kabuli_denovo	30756117	(A/G)
24271	CakSNP24271	*Kabuli*	Ca_Kabuli_denovo	30760173	(G/C)
24272	CakSNP24272	*Kabuli*	Ca_Kabuli_denovo	30760215	(C/G)
24273	CakSNP24273	*Kabuli*	Ca_Kabuli_denovo	30765681	(T/C)
24274	CakSNP24274	*Kabuli*	Ca_Kabuli_denovo	30765682	(C/G)
24275	CakSNP24275	*Kabuli*	Ca_Kabuli_denovo	30765685	(A/C)
24276	CakSNP24276	*Kabuli*	Ca_Kabuli_denovo	30765686	(A/C)
24277	CakSNP24277	*Kabuli*	Ca_Kabuli_denovo	30766057	(G/A)
24278	CakSNP24278	*Kabuli*	Ca_Kabuli_denovo	30768470	(A/T)
24279	CakSNP24279	*Kabuli*	Ca_Kabuli_denovo	30772256	(C/G)
24280	CakSNP24280	*Kabuli*	Ca_Kabuli_denovo	30772258	(G/T)
24281	CakSNP24281	*Kabuli*	Ca_Kabuli_denovo	30772260	(C/T)

S.N.	SNP IDs	Cultivars	Chromosomes/scaffolds	Physical positions (bp)	SNPs
24282	CakSNP24282	*Kabuli*	Ca_Kabuli_denovo	30772261	(A/C)
24283	CakSNP24283	*Kabuli*	Ca_Kabuli_denovo	30772262	(G/A)
24284	CakSNP24284	*Kabuli*	Ca_Kabuli_denovo	30774518	(A/G)
24285	CakSNP24285	*Kabuli*	Ca_Kabuli_denovo	30774519	(G/A)
24286	CakSNP24286	*Kabuli*	Ca_Kabuli_denovo	30774521	(A/C)
24287	CakSNP24287	*Kabuli*	Ca_Kabuli_denovo	30774523	(G/A)
24288	CakSNP24288	*Kabuli*	Ca_Kabuli_denovo	30774524	(T/A)
24289	CakSNP24289	*Kabuli*	Ca_Kabuli_denovo	30774525	(C/A)
24290	CakSNP24290	*Kabuli*	Ca_Kabuli_denovo	30775207	(A/G)
24291	CakSNP24291	*Kabuli*	Ca_Kabuli_denovo	30775239	(A/G)
24292	CakSNP24292	*Kabuli*	Ca_Kabuli_denovo	30775240	(T/G)
24293	CakSNP24293	*Kabuli*	Ca_Kabuli_denovo	30775682	(C/G)
24294	CakSNP24294	*Kabuli*	Ca_Kabuli_denovo	30775707	(C/G)
24295	CakSNP24295	*Kabuli*	Ca_Kabuli_denovo	30775728	(A/G)
24296	CakSNP24296	*Kabuli*	Ca_Kabuli_denovo	30776185	(C/G)
24297	CakSNP24297	*Kabuli*	Ca_Kabuli_denovo	30776186	(A/G)
24298	CakSNP24298	*Kabuli*	Ca_Kabuli_denovo	30776187	(A/T)
24299	CakSNP24299	*Kabuli*	Ca_Kabuli_denovo	30776188	(C/T)
24300	CakSNP24300	*Kabuli*	Ca_Kabuli_denovo	30780155	(C/A)
24301	CakSNP24301	*Kabuli*	Ca_Kabuli_denovo	30780159	(A/G)
24302	CakSNP24302	*Kabuli*	Ca_Kabuli_denovo	30780168	(T/G)
24303	CakSNP24303	*Kabuli*	Ca_Kabuli_denovo	30780869	(T/G)
24304	CakSNP24304	*Kabuli*	Ca_Kabuli_denovo	30780871	(C/G)
24305	CakSNP24305	*Kabuli*	Ca_Kabuli_denovo	30780877	(A/G)
24306	CakSNP24306	*Kabuli*	Ca_Kabuli_denovo	30780881	(C/G)
24307	CakSNP24307	*Kabuli*	Ca_Kabuli_denovo	30780893	(T/G)
24308	CakSNP24308	*Kabuli*	Ca_Kabuli_denovo	30786169	(A/G)
24309	CakSNP24309	*Kabuli*	Ca_Kabuli_denovo	30786176	(G/A)
24310	CakSNP24310	*Kabuli*	Ca_Kabuli_denovo	30786230	(T/G)
24311	CakSNP24311	*Kabuli*	Ca_Kabuli_denovo	30786234	(A/G)
24312	CakSNP24312	*Kabuli*	Ca_Kabuli_denovo	30792759	(T/C)
24313	CakSNP24313	*Kabuli*	Ca_Kabuli_denovo	30792760	(A/C)
24314	CakSNP24314	*Kabuli*	Ca_Kabuli_denovo	30792764	(A/C)
24315	CakSNP24315	*Kabuli*	Ca_Kabuli_denovo	30792765	(A/G)
24316	CakSNP24316	*Kabuli*	Ca_Kabuli_denovo	30797378	(T/G)
24317	CakSNP24317	*Kabuli*	Ca_Kabuli_denovo	30797410	(A/G)
24318	CakSNP24318	*Kabuli*	Ca_Kabuli_denovo	30798168	(C/G)
24319	CakSNP24319	*Kabuli*	Ca_Kabuli_denovo	30798174	(A/G)

S.N.	SNP IDs	Cultivars	Chromosomes/scaffolds	Physical positions (bp)	SNPs
24320	CakSNP24320	*Kabuli*	Ca_Kabuli_denovo	30798186	(G/A)
24321	CakSNP24321	*Kabuli*	Ca_Kabuli_denovo	30798221	(A/G)
24322	CakSNP24322	*Kabuli*	Ca_Kabuli_denovo	30806118	(A/G)
24323	CakSNP24323	*Kabuli*	Ca_Kabuli_denovo	30807009	(C/G)
24324	CakSNP24324	*Kabuli*	Ca_Kabuli_denovo	30814984	(C/A)
24325	CakSNP24325	*Kabuli*	Ca_Kabuli_denovo	30814986	(G/T)
24326	CakSNP24326	*Kabuli*	Ca_Kabuli_denovo	30814988	(C/A)
24327	CakSNP24327	*Kabuli*	Ca_Kabuli_denovo	30814989	(C/A)
24328	CakSNP24328	*Kabuli*	Ca_Kabuli_denovo	30814991	(A/G)
24329	CakSNP24329	*Kabuli*	Ca_Kabuli_denovo	30817556	(C/G)
24330	CakSNP24330	*Kabuli*	Ca_Kabuli_denovo	30820587	(G/A)
24331	CakSNP24331	*Kabuli*	Ca_Kabuli_denovo	30820674	(A/G)
24332	CakSNP24332	*Kabuli*	Ca_Kabuli_denovo	30820693	(T/G)
24333	CakSNP24333	*Kabuli*	Ca_Kabuli_denovo	30820725	(A/G)
24334	CakSNP24334	*Kabuli*	Ca_Kabuli_denovo	30820857	(G/A)
24335	CakSNP24335	*Kabuli*	Ca_Kabuli_denovo	30820885	(T/G)
24336	CakSNP24336	*Kabuli*	Ca_Kabuli_denovo	30820935	(G/C)
24337	CakSNP24337	*Kabuli*	Ca_Kabuli_denovo	30821907	(A/G)
24338	CakSNP24338	*Kabuli*	Ca_Kabuli_denovo	30821961	(T/G)
24339	CakSNP24339	*Kabuli*	Ca_Kabuli_denovo	30824952	(C/A)
24340	CakSNP24340	*Kabuli*	Ca_Kabuli_denovo	30825037	(G/T)
24341	CakSNP24341	*Kabuli*	Ca_Kabuli_denovo	30825073	(C/G)
24342	CakSNP24342	*Kabuli*	Ca_Kabuli_denovo	30825087	(T/G)
24343	CakSNP24343	*Kabuli*	Ca_Kabuli_denovo	30825459	(C/G)
24344	CakSNP24344	*Kabuli*	Ca_Kabuli_denovo	30826626	(A/G)
24345	CakSNP24345	*Kabuli*	Ca_Kabuli_denovo	30826708	(T/A)
24346	CakSNP24346	*Kabuli*	Ca_Kabuli_denovo	30826821	(C/A)
24347	CakSNP24347	*Kabuli*	Ca_Kabuli_denovo	30826826	(T/G)
24348	CakSNP24348	*Kabuli*	Ca_Kabuli_denovo	30826840	(G/T)
24349	CakSNP24349	*Kabuli*	Ca_Kabuli_denovo	30826859	(A/G)
24350	CakSNP24350	*Kabuli*	Ca_Kabuli_denovo	30826896	(T/G)
24351	CakSNP24351	*Kabuli*	Ca_Kabuli_denovo	30829200	(G/A)
24352	CakSNP24352	*Kabuli*	Ca_Kabuli_denovo	30829213	(C/G)
24353	CakSNP24353	*Kabuli*	Ca_Kabuli_denovo	30831665	(G/T)
24354	CakSNP24354	*Kabuli*	Ca_Kabuli_denovo	30832766	(C/G)
24355	CakSNP24355	*Kabuli*	Ca_Kabuli_denovo	30833069	(G/A)
24356	CakSNP24356	*Kabuli*	Ca_Kabuli_denovo	30835039	(G/A)
24357	CakSNP24357	*Kabuli*	Ca_Kabuli_denovo	30835060	(G/C)

S.N.	SNP IDs	Cultivars	Chromosomes/scaffolds	Physical positions (bp)	SNPs
24358	CakSNP24358	*Kabuli*	Ca_Kabuli_denovo	30836884	(A/G)
24359	CakSNP24359	*Kabuli*	Ca_Kabuli_denovo	30836903	(T/C)
24360	CakSNP24360	*Kabuli*	Ca_Kabuli_denovo	30836937	(T/A)
24361	CakSNP24361	*Kabuli*	Ca_Kabuli_denovo	30836941	(A/C)
24362	CakSNP24362	*Kabuli*	Ca_Kabuli_denovo	30846063	(T/G)
24363	CakSNP24363	*Kabuli*	Ca_Kabuli_denovo	30846909	(T/G)
24364	CakSNP24364	*Kabuli*	Ca_Kabuli_denovo	30886176	(A/G)
24365	CakSNP24365	*Kabuli*	Ca_Kabuli_denovo	30886221	(C/T)
24366	CakSNP24366	*Kabuli*	Ca_Kabuli_denovo	30914188	(A/T)
24367	CakSNP24367	*Kabuli*	Ca_Kabuli_denovo	30914189	(T/C)
24368	CakSNP24368	*Kabuli*	Ca_Kabuli_denovo	30914190	(C/G)
24369	CakSNP24369	*Kabuli*	Ca_Kabuli_denovo	30914192	(A/G)
24370	CakSNP24370	*Kabuli*	Ca_Kabuli_denovo	30914194	(G/A)
24371	CakSNP24371	*Kabuli*	Ca_Kabuli_denovo	30914211	(T/A)
24372	CakSNP24372	*Kabuli*	Ca_Kabuli_denovo	30914217	(G/A)
24373	CakSNP24373	*Kabuli*	Ca_Kabuli_denovo	30914223	(T/C)
24374	CakSNP24374	*Kabuli*	Ca_Kabuli_denovo	30914224	(C/T)
24375	CakSNP24375	*Kabuli*	Ca_Kabuli_denovo	30915856	(G/A)
24376	CakSNP24376	*Kabuli*	Ca_Kabuli_denovo	30920569	(C/A)
24377	CakSNP24377	*Kabuli*	Ca_Kabuli_denovo	30920603	(A/G)
24378	CakSNP24378	*Kabuli*	Ca_Kabuli_denovo	30920620	(C/G)
24379	CakSNP24379	*Kabuli*	Ca_Kabuli_denovo	30920641	(T/G)
24380	CakSNP24380	*Kabuli*	Ca_Kabuli_denovo	30948570	(A/G)
24381	CakSNP24381	*Kabuli*	Ca_Kabuli_denovo	30948583	(C/T)
24382	CakSNP24382	*Kabuli*	Ca_Kabuli_denovo	39369006	(C/G)
24383	CakSNP24383	*Kabuli*	Ca_Kabuli_denovo	43194868	(T/A)
24384	CakSNP24384	*Kabuli*	Ca_Kabuli_denovo	43209602	(C/T)
24385	CakSNP24385	*Kabuli*	Ca_Kabuli_denovo	43209612	(T/G)
24386	CakSNP24386	*Kabuli*	Ca_Kabuli_denovo	43706574	(C/T)
24387	CakSNP24387	*Kabuli*	Ca_Kabuli_denovo	43711784	(A/C)
24388	CakSNP24388	*Kabuli*	Ca_Kabuli_denovo	43711813	(A/G)
24389	CakSNP24389	*Kabuli*	Ca_Kabuli_denovo	43990689	(T/C)
24390	CakSNP24390	*Kabuli*	Ca_Kabuli_denovo	43990690	(C/G)
24391	CakSNP24391	*Kabuli*	Ca_Kabuli_denovo	43990720	(C/A)
24392	CakSNP24392	*Kabuli*	Ca_Kabuli_denovo	43990743	(G/A)
24393	CakSNP24393	*Kabuli*	Ca_Kabuli_denovo	43990748	(T/G)
24394	CakSNP24394	*Kabuli*	Ca_Kabuli_denovo	43990751	(C/T)
24395	CakSNP24395	*Kabuli*	Ca_Kabuli_denovo	44029557	(G/C)

S.N.	SNP IDs	Cultivars	Chromosomes/scaffolds	Physical positions (bp)	SNPs
24396	CakSNP24396	*Kabuli*	Ca_Kabuli_denovo	44029593	(G/T)
24397	CakSNP24397	*Kabuli*	Ca_Kabuli_denovo	44071541	(A/G)
24398	CakSNP24398	*Kabuli*	Ca_Kabuli_denovo	44449557	(A/G)
24399	CakSNP24399	*Kabuli*	Ca_Kabuli_denovo	44449575	(A/T)
24400	CakSNP24400	*Kabuli*	Ca_Kabuli_denovo	44449577	(G/A)
24401	CakSNP24401	*Kabuli*	Ca_Kabuli_denovo	44449597	(C/T)
24402	CakSNP24402	*Kabuli*	Ca_Kabuli_denovo	67025915	(G/T)
24403	CakSNP24403	*Kabuli*	Ca_Kabuli_denovo	67025928	(G/T)
24404	CakSNP24404	*Kabuli*	Ca_Kabuli_denovo	67030173	(T/G)
24405	CakSNP24405	*Kabuli*	Ca_Kabuli_denovo	67030216	(A/G)
24406	CadSNP1	*Desi*	Ca_Desi_Chr01	179189	(A/C)
24407	CadSNP2	*Desi*	Ca_Desi_Chr01	382862	(T/C)
24408	CadSNP3	*Desi*	Ca_Desi_Chr01	382872	(G/A)
24409	CadSNP4	*Desi*	Ca_Desi_Chr01	382898	(T/C)
24410	CadSNP5	*Desi*	Ca_Desi_Chr01	436895	(A/C)
24411	CadSNP6	*Desi*	Ca_Desi_Chr01	461870	(A/G)
24412	CadSNP7	*Desi*	Ca_Desi_Chr01	498862	(A/G)
24413	CadSNP8	*Desi*	Ca_Desi_Chr01	517512	(T/C)
24414	CadSNP9	*Desi*	Ca_Desi_Chr01	517439	(A/G)
24415	CadSNP10	*Desi*	Ca_Desi_Chr01	527099	(A/G)
24416	CadSNP11	*Desi*	Ca_Desi_Chr01	527113	(G/T)
24417	CadSNP12	*Desi*	Ca_Desi_Chr01	527118	(T/C)
24418	CadSNP13	*Desi*	Ca_Desi_Chr01	527135	(G/A)
24419	CadSNP14	*Desi*	Ca_Desi_Chr01	527142	(G/T)
24420	CadSNP15	*Desi*	Ca_Desi_Chr01	527181	(C/A)
24421	CadSNP16	*Desi*	Ca_Desi_Chr01	527176	(T/C)
24422	CadSNP17	*Desi*	Ca_Desi_Chr01	527104	(G/T)
24423	CadSNP18	*Desi*	Ca_Desi_Chr01	533122	(G/A)
24424	CadSNP19	*Desi*	Ca_Desi_Chr01	533125	(C/T)
24425	CadSNP20	*Desi*	Ca_Desi_Chr01	533127	(T/A)
24426	CadSNP21	*Desi*	Ca_Desi_Chr01	533182	(T/A)
24427	CadSNP22	*Desi*	Ca_Desi_Chr01	537937	(T/C)
24428	CadSNP23	*Desi*	Ca_Desi_Chr01	537971	(A/G)
24429	CadSNP24	*Desi*	Ca_Desi_Chr01	538012	(C/T)
24430	CadSNP25	*Desi*	Ca_Desi_Chr01	538086	(G/T)
24431	CadSNP26	*Desi*	Ca_Desi_Chr01	548203	(T/C)
24432	CadSNP27	*Desi*	Ca_Desi_Chr01	551496	(T/G)
24433	CadSNP28	*Desi*	Ca_Desi_Chr01	559934	(C/T)

S.N.	SNP IDs	Cultivars	Chromosomes/scaffolds	Physical positions (bp)	SNPs
24434	CadSNP29	*Desi*	Ca_Desi_Chr01	559964	(C/T)
24435	CadSNP30	*Desi*	Ca_Desi_Chr01	620260	(T/G)
24436	CadSNP31	*Desi*	Ca_Desi_Chr01	620310	(C/T)
24437	CadSNP32	*Desi*	Ca_Desi_Chr01	647919	(G/T)
24438	CadSNP33	*Desi*	Ca_Desi_Chr01	648015	(G/A)
24439	CadSNP34	*Desi*	Ca_Desi_Chr01	664787	(T/G)
24440	CadSNP35	*Desi*	Ca_Desi_Chr01	685373	(T/C)
24441	CadSNP36	*Desi*	Ca_Desi_Chr01	704852	(A/G)
24442	CadSNP37	*Desi*	Ca_Desi_Chr01	704814	(C/T)
24443	CadSNP38	*Desi*	Ca_Desi_Chr01	704812	(C/G)
24444	CadSNP39	*Desi*	Ca_Desi_Chr01	739330	(C/T)
24445	CadSNP40	*Desi*	Ca_Desi_Chr01	739365	(G/A)
24446	CadSNP41	*Desi*	Ca_Desi_Chr01	739371	(T/A)
24447	CadSNP42	*Desi*	Ca_Desi_Chr01	739452	(T/C)
24448	CadSNP43	*Desi*	Ca_Desi_Chr01	739437	(G/A)
24449	CadSNP44	*Desi*	Ca_Desi_Chr01	756894	(A/G)
24450	CadSNP45	*Desi*	Ca_Desi_Chr01	757353	(A/C)
24451	CadSNP46	*Desi*	Ca_Desi_Chr01	757501	(T/A)
24452	CadSNP47	*Desi*	Ca_Desi_Chr01	766231	(C/A)
24453	CadSNP48	*Desi*	Ca_Desi_Chr01	766221	(T/C)
24454	CadSNP49	*Desi*	Ca_Desi_Chr01	766202	(T/C)
24455	CadSNP50	*Desi*	Ca_Desi_Chr01	766200	(A/G)
24456	CadSNP51	*Desi*	Ca_Desi_Chr01	766199	(A/T)
24457	CadSNP52	*Desi*	Ca_Desi_Chr01	785375	(C/A)
24458	CadSNP53	*Desi*	Ca_Desi_Chr01	785650	(G/A)
24459	CadSNP54	*Desi*	Ca_Desi_Chr01	786321	(A/T)
24460	CadSNP55	*Desi*	Ca_Desi_Chr01	791180	(A/C)
24461	CadSNP56	*Desi*	Ca_Desi_Chr01	807232	(T/C)
24462	CadSNP57	*Desi*	Ca_Desi_Chr01	807195	(C/T)
24463	CadSNP58	*Desi*	Ca_Desi_Chr01	836330	(G/T)
24464	CadSNP59	*Desi*	Ca_Desi_Chr01	836351	(C/T)
24465	CadSNP60	*Desi*	Ca_Desi_Chr01	836381	(T/C)
24466	CadSNP61	*Desi*	Ca_Desi_Chr01	836365	(C/T)
24467	CadSNP62	*Desi*	Ca_Desi_Chr01	836796	(C/G)
24468	CadSNP63	*Desi*	Ca_Desi_Chr01	836859	(G/A)
24469	CadSNP64	*Desi*	Ca_Desi_Chr01	836842	(T/G)
24470	CadSNP65	*Desi*	Ca_Desi_Chr01	845508	(C/T)
24471	CadSNP66	*Desi*	Ca_Desi_Chr01	872280	(C/T)

S.N.	SNP IDs	Cultivars	Chromosomes/scaffolds	Physical positions (bp)	SNPs
24472	CadSNP67	*Desi*	Ca_Desi_Chr01	872247	(G/A)
24473	CadSNP68	*Desi*	Ca_Desi_Chr01	885647	(A/C)
24474	CadSNP69	*Desi*	Ca_Desi_Chr01	885643	(C/T)
24475	CadSNP70	*Desi*	Ca_Desi_Chr01	885745	(G/T)
24476	CadSNP71	*Desi*	Ca_Desi_Chr01	965708	(T/C)
24477	CadSNP72	*Desi*	Ca_Desi_Chr01	965885	(T/C)
24478	CadSNP73	*Desi*	Ca_Desi_Chr01	965884	(G/A)
24479	CadSNP74	*Desi*	Ca_Desi_Chr01	965854	(T/C)
24480	CadSNP75	*Desi*	Ca_Desi_Chr01	968375	(T/C)
24481	CadSNP76	*Desi*	Ca_Desi_Chr01	973945	(G/T)
24482	CadSNP77	*Desi*	Ca_Desi_Chr01	973954	(C/T)
24483	CadSNP78	*Desi*	Ca_Desi_Chr01	978116	(T/C)
24484	CadSNP79	*Desi*	Ca_Desi_Chr01	978133	(T/A)
24485	CadSNP80	*Desi*	Ca_Desi_Chr01	983043	(C/A)
24486	CadSNP81	*Desi*	Ca_Desi_Chr01	983173	(G/A)
24487	CadSNP82	*Desi*	Ca_Desi_Chr01	983203	(G/T)
24488	CadSNP83	*Desi*	Ca_Desi_Chr01	992175	(G/A)
24489	CadSNP84	*Desi*	Ca_Desi_Chr01	992174	(C/T)
24490	CadSNP85	*Desi*	Ca_Desi_Chr01	999613	(T/C)
24491	CadSNP86	*Desi*	Ca_Desi_Chr01	1025432	(T/C)
24492	CadSNP87	*Desi*	Ca_Desi_Chr01	1025458	(C/T)
24493	CadSNP88	*Desi*	Ca_Desi_Chr01	1025593	(C/A)
24494	CadSNP89	*Desi*	Ca_Desi_Chr01	1025590	(A/G)
24495	CadSNP90	*Desi*	Ca_Desi_Chr01	1069295	(A/C)
24496	CadSNP91	*Desi*	Ca_Desi_Chr01	1069436	(G/A)
24497	CadSNP92	*Desi*	Ca_Desi_Chr01	1069425	(A/C)
24498	CadSNP93	*Desi*	Ca_Desi_Chr01	1069410	(A/T)
24499	CadSNP94	*Desi*	Ca_Desi_Chr01	1069403	(A/G)
24500	CadSNP95	*Desi*	Ca_Desi_Chr01	1071218	(C/T)
24501	CadSNP96	*Desi*	Ca_Desi_Chr01	1071230	(C/T)
24502	CadSNP97	*Desi*	Ca_Desi_Chr01	1071343	(G/A)
24503	CadSNP98	*Desi*	Ca_Desi_Chr01	1071275	(T/C)
24504	CadSNP99	*Desi*	Ca_Desi_Chr01	1072524	(A/T)
24505	CadSNP100	*Desi*	Ca_Desi_Chr01	1072532	(C/T)
24506	CadSNP101	*Desi*	Ca_Desi_Chr01	1072623	(G/A)
24507	CadSNP102	*Desi*	Ca_Desi_Chr01	1072955	(A/G)
24508	CadSNP103	*Desi*	Ca_Desi_Chr01	1072973	(T/C)
24509	CadSNP104	*Desi*	Ca_Desi_Chr01	1072976	(A/G)

S.N.	SNP IDs	Cultivars	Chromosomes/scaffolds	Physical positions (bp)	SNPs
24510	CadSNP105	*Desi*	Ca_Desi_Chr01	1073000	(A/T)
24511	CadSNP106	*Desi*	Ca_Desi_Chr01	1073059	(G/A)
24512	CadSNP107	*Desi*	Ca_Desi_Chr01	1073054	(A/G)
24513	CadSNP108	*Desi*	Ca_Desi_Chr01	1073048	(G/A)
24514	CadSNP109	*Desi*	Ca_Desi_Chr01	1126922	(G/C)
24515	CadSNP110	*Desi*	Ca_Desi_Chr01	1140358	(A/G)
24516	CadSNP111	*Desi*	Ca_Desi_Chr01	1140485	(A/T)
24517	CadSNP112	*Desi*	Ca_Desi_Chr01	1145410	(A/G)
24518	CadSNP113	*Desi*	Ca_Desi_Chr01	1145493	(G/T)
24519	CadSNP114	*Desi*	Ca_Desi_Chr01	1146141	(C/T)
24520	CadSNP115	*Desi*	Ca_Desi_Chr01	1146138	(T/G)
24521	CadSNP116	*Desi*	Ca_Desi_Chr01	1146211	(C/T)
24522	CadSNP117	*Desi*	Ca_Desi_Chr01	1146466	(C/T)
24523	CadSNP118	*Desi*	Ca_Desi_Chr01	1146471	(A/G)
24524	CadSNP119	*Desi*	Ca_Desi_Chr01	1146489	(C/T)
24525	CadSNP120	*Desi*	Ca_Desi_Chr01	1146490	(G/A)
24526	CadSNP121	*Desi*	Ca_Desi_Chr01	1146659	(C/T)
24527	CadSNP122	*Desi*	Ca_Desi_Chr01	1146626	(C/A)
24528	CadSNP123	*Desi*	Ca_Desi_Chr01	1146611	(C/G)
24529	CadSNP124	*Desi*	Ca_Desi_Chr01	1156112	(A/G)
24530	CadSNP125	*Desi*	Ca_Desi_Chr01	1189681	(C/G)
24531	CadSNP126	*Desi*	Ca_Desi_Chr01	1189628	(C/A)
24532	CadSNP127	*Desi*	Ca_Desi_Chr01	1189627	(T/A)
24533	CadSNP128	*Desi*	Ca_Desi_Chr01	1189626	(G/C)
24534	CadSNP129	*Desi*	Ca_Desi_Chr01	1234998	(A/T)
24535	CadSNP130	*Desi*	Ca_Desi_Chr01	1234995	(G/A)
24536	CadSNP131	*Desi*	Ca_Desi_Chr01	1234986	(A/G)
24537	CadSNP132	*Desi*	Ca_Desi_Chr01	1234985	(A/G)
24538	CadSNP133	*Desi*	Ca_Desi_Chr01	1234977	(G/T)
24539	CadSNP134	*Desi*	Ca_Desi_Chr01	1271491	(C/T)
24540	CadSNP135	*Desi*	Ca_Desi_Chr01	1271498	(A/T)
24541	CadSNP136	*Desi*	Ca_Desi_Chr01	1271686	(G/A)
24542	CadSNP137	*Desi*	Ca_Desi_Chr01	1271654	(T/G)
24543	CadSNP138	*Desi*	Ca_Desi_Chr01	1375921	(C/G)
24544	CadSNP139	*Desi*	Ca_Desi_Chr01	1384553	(C/T)
24545	CadSNP140	*Desi*	Ca_Desi_Chr01	1389951	(C/T)
24546	CadSNP141	*Desi*	Ca_Desi_Chr01	1390763	(T/C)
24547	CadSNP142	*Desi*	Ca_Desi_Chr01	1445548	(T/C)

S.N.	SNP IDs	Cultivars	Chromosomes/scaffolds	Physical positions (bp)	SNPs
24548	CadSNP143	*Desi*	Ca_Desi_Chr01	1451002	(A/G)
24549	CadSNP144	*Desi*	Ca_Desi_Chr01	1451028	(A/T)
24550	CadSNP145	*Desi*	Ca_Desi_Chr01	1463203	(T/C)
24551	CadSNP146	*Desi*	Ca_Desi_Chr01	1463234	(C/T)
24552	CadSNP147	*Desi*	Ca_Desi_Chr01	1463270	(A/C)
24553	CadSNP148	*Desi*	Ca_Desi_Chr01	1463335	(C/T)
24554	CadSNP149	*Desi*	Ca_Desi_Chr01	1479425	(T/C)
24555	CadSNP150	*Desi*	Ca_Desi_Chr01	1479437	(T/C)
24556	CadSNP151	*Desi*	Ca_Desi_Chr01	1480187	(T/C)
24557	CadSNP152	*Desi*	Ca_Desi_Chr01	1486086	(G/C)
24558	CadSNP153	*Desi*	Ca_Desi_Chr01	1486057	(A/C)
24559	CadSNP154	*Desi*	Ca_Desi_Chr01	1522221	(T/C)
24560	CadSNP155	*Desi*	Ca_Desi_Chr01	1526742	(G/T)
24561	CadSNP156	*Desi*	Ca_Desi_Chr01	1526741	(C/A)
24562	CadSNP157	*Desi*	Ca_Desi_Chr01	1553951	(A/G)
24563	CadSNP158	*Desi*	Ca_Desi_Chr01	1553958	(A/G)
24564	CadSNP159	*Desi*	Ca_Desi_Chr01	1598211	(C/T)
24565	CadSNP160	*Desi*	Ca_Desi_Chr01	1627093	(G/T)
24566	CadSNP161	*Desi*	Ca_Desi_Chr01	1632231	(G/A)
24567	CadSNP162	*Desi*	Ca_Desi_Chr01	1638965	(T/A)
24568	CadSNP163	*Desi*	Ca_Desi_Chr01	1639227	(C/G)
24569	CadSNP164	*Desi*	Ca_Desi_Chr01	1727845	(T/A)
24570	CadSNP165	*Desi*	Ca_Desi_Chr01	1787370	(A/T)
24571	CadSNP166	*Desi*	Ca_Desi_Chr01	1815665	(T/C)
24572	CadSNP167	*Desi*	Ca_Desi_Chr01	1826921	(A/G)
24573	CadSNP168	*Desi*	Ca_Desi_Chr01	1828118	(A/G)
24574	CadSNP169	*Desi*	Ca_Desi_Chr01	1828194	(C/G)
24575	CadSNP170	*Desi*	Ca_Desi_Chr01	1828281	(A/C)
24576	CadSNP171	*Desi*	Ca_Desi_Chr01	1838149	(C/G)
24577	CadSNP172	*Desi*	Ca_Desi_Chr01	1839807	(A/G)
24578	CadSNP173	*Desi*	Ca_Desi_Chr01	1842557	(C/T)
24579	CadSNP174	*Desi*	Ca_Desi_Chr01	1842545	(T/C)
24580	CadSNP175	*Desi*	Ca_Desi_Chr01	1851780	(A/T)
24581	CadSNP176	*Desi*	Ca_Desi_Chr01	1881482	(A/G)
24582	CadSNP177	*Desi*	Ca_Desi_Chr01	1881584	(A/G)
24583	CadSNP178	*Desi*	Ca_Desi_Chr01	1904344	(T/C)
24584	CadSNP179	*Desi*	Ca_Desi_Chr01	1904458	(G/A)
24585	CadSNP180	*Desi*	Ca_Desi_Chr01	1904473	(C/T)

S.N.	SNP IDs	Cultivars	Chromosomes/scaffolds	Physical positions (bp)	SNPs
24586	CadSNP181	*Desi*	Ca_Desi_Chr01	1906298	(A/C)
24587	CadSNP182	*Desi*	Ca_Desi_Chr01	1906300	(T/G)
24588	CadSNP183	*Desi*	Ca_Desi_Chr01	1906308	(A/G)
24589	CadSNP184	*Desi*	Ca_Desi_Chr01	1906324	(A/C)
24590	CadSNP185	*Desi*	Ca_Desi_Chr01	1906333	(A/C)
24591	CadSNP186	*Desi*	Ca_Desi_Chr01	1906363	(G/A)
24592	CadSNP187	*Desi*	Ca_Desi_Chr01	1906417	(G/A)
24593	CadSNP188	*Desi*	Ca_Desi_Chr01	1912864	(T/A)
24594	CadSNP189	*Desi*	Ca_Desi_Chr01	1912850	(A/C)
24595	CadSNP190	*Desi*	Ca_Desi_Chr01	1912820	(C/T)
24596	CadSNP191	*Desi*	Ca_Desi_Chr01	1920014	(C/G)
24597	CadSNP192	*Desi*	Ca_Desi_Chr01	1920100	(A/G)
24598	CadSNP193	*Desi*	Ca_Desi_Chr01	1936976	(C/T)
24599	CadSNP194	*Desi*	Ca_Desi_Chr01	1937117	(A/G)
24600	CadSNP195	*Desi*	Ca_Desi_Chr01	1937351	(C/T)
24601	CadSNP196	*Desi*	Ca_Desi_Chr01	1960896	(A/C)
24602	CadSNP197	*Desi*	Ca_Desi_Chr01	1960988	(A/T)
24603	CadSNP198	*Desi*	Ca_Desi_Chr01	1971575	(C/T)
24604	CadSNP199	*Desi*	Ca_Desi_Chr01	1982239	(C/A)
24605	CadSNP200	*Desi*	Ca_Desi_Chr01	1982267	(T/G)
24606	CadSNP201	*Desi*	Ca_Desi_Chr01	2021813	(A/G)
24607	CadSNP202	*Desi*	Ca_Desi_Chr01	2021754	(T/A)
24608	CadSNP203	*Desi*	Ca_Desi_Chr01	2044121	(A/G)
24609	CadSNP204	*Desi*	Ca_Desi_Chr01	2067290	(C/A)
24610	CadSNP205	*Desi*	Ca_Desi_Chr01	2084464	(G/A)
24611	CadSNP206	*Desi*	Ca_Desi_Chr01	2084449	(A/G)
24612	CadSNP207	*Desi*	Ca_Desi_Chr01	2084396	(T/G)
24613	CadSNP208	*Desi*	Ca_Desi_Chr01	2084474	(C/A)
24614	CadSNP209	*Desi*	Ca_Desi_Chr01	2084506	(G/T)
24615	CadSNP210	*Desi*	Ca_Desi_Chr01	2084548	(C/G)
24616	CadSNP211	*Desi*	Ca_Desi_Chr01	2185003	(C/T)
24617	CadSNP212	*Desi*	Ca_Desi_Chr01	2213640	(A/G)
24618	CadSNP213	*Desi*	Ca_Desi_Chr01	2213694	(C/T)
24619	CadSNP214	*Desi*	Ca_Desi_Chr01	2213700	(C/T)
24620	CadSNP215	*Desi*	Ca_Desi_Chr01	2213762	(T/C)
24621	CadSNP216	*Desi*	Ca_Desi_Chr01	2213817	(A/G)
24622	CadSNP217	*Desi*	Ca_Desi_Chr01	2214078	(A/T)
24623	CadSNP218	*Desi*	Ca_Desi_Chr01	2214038	(A/T)

S.N.	SNP IDs	Cultivars	Chromosomes/scaffolds	Physical positions (bp)	SNPs
24624	CadSNP219	*Desi*	Ca_Desi_Chr01	2214019	(A/G)
24625	CadSNP220	*Desi*	Ca_Desi_Chr01	2249232	(A/G)
24626	CadSNP221	*Desi*	Ca_Desi_Chr01	2263604	(C/A)
24627	CadSNP222	*Desi*	Ca_Desi_Chr01	2281554	(T/C)
24628	CadSNP223	*Desi*	Ca_Desi_Chr01	2281552	(C/T)
24629	CadSNP224	*Desi*	Ca_Desi_Chr01	2292933	(T/C)
24630	CadSNP225	*Desi*	Ca_Desi_Chr01	2322362	(G/A)
24631	CadSNP226	*Desi*	Ca_Desi_Chr01	2322455	(A/C)
24632	CadSNP227	*Desi*	Ca_Desi_Chr01	2327411	(T/G)
24633	CadSNP228	*Desi*	Ca_Desi_Chr01	2349214	(C/A)
24634	CadSNP229	*Desi*	Ca_Desi_Chr01	2349258	(G/A)
24635	CadSNP230	*Desi*	Ca_Desi_Chr01	2349306	(C/T)
24636	CadSNP231	*Desi*	Ca_Desi_Chr01	2350002	(T/C)
24637	CadSNP232	*Desi*	Ca_Desi_Chr01	2356768	(A/G)
24638	CadSNP233	*Desi*	Ca_Desi_Chr01	2356772	(A/T)
24639	CadSNP234	*Desi*	Ca_Desi_Chr01	2438649	(T/A)
24640	CadSNP235	*Desi*	Ca_Desi_Chr01	2438719	(T/A)
24641	CadSNP236	*Desi*	Ca_Desi_Chr01	2440248	(C/T)
24642	CadSNP237	*Desi*	Ca_Desi_Chr01	2448997	(C/T)
24643	CadSNP238	*Desi*	Ca_Desi_Chr01	2456844	(T/G)
24644	CadSNP239	*Desi*	Ca_Desi_Chr01	2457014	(A/C)
24645	CadSNP240	*Desi*	Ca_Desi_Chr01	2471770	(G/T)
24646	CadSNP241	*Desi*	Ca_Desi_Chr01	2471753	(G/A)
24647	CadSNP242	*Desi*	Ca_Desi_Chr01	2471803	(A/G)
24648	CadSNP243	*Desi*	Ca_Desi_Chr01	2471865	(T/C)
24649	CadSNP244	*Desi*	Ca_Desi_Chr01	2471996	(G/A)
24650	CadSNP245	*Desi*	Ca_Desi_Chr01	2471946	(C/A)
24651	CadSNP246	*Desi*	Ca_Desi_Chr01	2471938	(G/T)
24652	CadSNP247	*Desi*	Ca_Desi_Chr01	2471924	(G/A)
24653	CadSNP248	*Desi*	Ca_Desi_Chr01	2471923	(C/A)
24654	CadSNP249	*Desi*	Ca_Desi_Chr01	2491534	(T/C)
24655	CadSNP250	*Desi*	Ca_Desi_Chr01	2491494	(G/A)
24656	CadSNP251	*Desi*	Ca_Desi_Chr01	2491479	(T/C)
24657	CadSNP252	*Desi*	Ca_Desi_Chr01	2536793	(A/G)
24658	CadSNP253	*Desi*	Ca_Desi_Chr01	2541722	(T/C)
24659	CadSNP254	*Desi*	Ca_Desi_Chr01	2553114	(C/T)
24660	CadSNP255	*Desi*	Ca_Desi_Chr01	2553120	(A/C)
24661	CadSNP256	*Desi*	Ca_Desi_Chr01	2553187	(A/G)

S.N.	SNP IDs	Cultivars	Chromosomes/scaffolds	Physical positions (bp)	SNPs
24662	CadSNP257	*Desi*	Ca_Desi_Chr01	2553150	(C/T)
24663	CadSNP258	*Desi*	Ca_Desi_Chr01	2554326	(T/G)
24664	CadSNP259	*Desi*	Ca_Desi_Chr01	2555171	(C/G)
24665	CadSNP260	*Desi*	Ca_Desi_Chr01	2573180	(A/C)
24666	CadSNP261	*Desi*	Ca_Desi_Chr01	2593870	(C/T)
24667	CadSNP262	*Desi*	Ca_Desi_Chr01	2637647	(C/G)
24668	CadSNP263	*Desi*	Ca_Desi_Chr01	2637591	(T/A)
24669	CadSNP264	*Desi*	Ca_Desi_Chr01	2637890	(G/C)
24670	CadSNP265	*Desi*	Ca_Desi_Chr01	2637849	(C/T)
24671	CadSNP266	*Desi*	Ca_Desi_Chr01	2775191	(G/A)
24672	CadSNP267	*Desi*	Ca_Desi_Chr01	2824614	(G/A)
24673	CadSNP268	*Desi*	Ca_Desi_Chr01	2856666	(T/C)
24674	CadSNP269	*Desi*	Ca_Desi_Chr01	2887261	(C/G)
24675	CadSNP270	*Desi*	Ca_Desi_Chr01	3010959	(T/C)
24676	CadSNP271	*Desi*	Ca_Desi_Chr01	3011053	(T/G)
24677	CadSNP272	*Desi*	Ca_Desi_Chr01	3073657	(C/T)
24678	CadSNP273	*Desi*	Ca_Desi_Chr01	3083080	(G/T)
24679	CadSNP274	*Desi*	Ca_Desi_Chr01	3088883	(G/T)
24680	CadSNP275	*Desi*	Ca_Desi_Chr01	3151378	(A/C)
24681	CadSNP276	*Desi*	Ca_Desi_Chr01	3151834	(G/C)
24682	CadSNP277	*Desi*	Ca_Desi_Chr01	3192625	(C/T)
24683	CadSNP278	*Desi*	Ca_Desi_Chr01	3192622	(C/G)
24684	CadSNP279	*Desi*	Ca_Desi_Chr01	3192562	(C/G)
24685	CadSNP280	*Desi*	Ca_Desi_Chr01	3328510	(C/A)
24686	CadSNP281	*Desi*	Ca_Desi_Chr01	3328604	(G/A)
24687	CadSNP282	*Desi*	Ca_Desi_Chr01	3366300	(T/C)
24688	CadSNP283	*Desi*	Ca_Desi_Chr01	3368165	(A/C)
24689	CadSNP284	*Desi*	Ca_Desi_Chr01	3370851	(A/T)
24690	CadSNP285	*Desi*	Ca_Desi_Chr01	3370914	(G/A)
24691	CadSNP286	*Desi*	Ca_Desi_Chr01	3390238	(G/T)
24692	CadSNP287	*Desi*	Ca_Desi_Chr01	3425891	(C/T)
24693	CadSNP288	*Desi*	Ca_Desi_Chr01	3427492	(G/A)
24694	CadSNP289	*Desi*	Ca_Desi_Chr01	3435226	(G/A)
24695	CadSNP290	*Desi*	Ca_Desi_Chr01	3435227	(C/T)
24696	CadSNP291	*Desi*	Ca_Desi_Chr01	3439224	(T/C)
24697	CadSNP292	*Desi*	Ca_Desi_Chr01	3440875	(C/A)
24698	CadSNP293	*Desi*	Ca_Desi_Chr01	3444610	(A/G)
24699	CadSNP294	*Desi*	Ca_Desi_Chr01	3444681	(G/T)

S.N.	SNP IDs	Cultivars	Chromosomes/scaffolds	Physical positions (bp)	SNPs
24700	CadSNP295	*Desi*	Ca_Desi_Chr01	3444708	(T/C)
24701	CadSNP296	*Desi*	Ca_Desi_Chr01	3444696	(C/A)
24702	CadSNP297	*Desi*	Ca_Desi_Chr01	3452397	(C/T)
24703	CadSNP298	*Desi*	Ca_Desi_Chr01	3545305	(T/G)
24704	CadSNP299	*Desi*	Ca_Desi_Chr01	3545332	(T/G)
24705	CadSNP300	*Desi*	Ca_Desi_Chr01	3585574	(G/T)
24706	CadSNP301	*Desi*	Ca_Desi_Chr01	3606976	(C/T)
24707	CadSNP302	*Desi*	Ca_Desi_Chr01	3633117	(T/C)
24708	CadSNP303	*Desi*	Ca_Desi_Chr01	3674413	(A/C)
24709	CadSNP304	*Desi*	Ca_Desi_Chr01	3674854	(A/C)
24710	CadSNP305	*Desi*	Ca_Desi_Chr01	3675786	(T/C)
24711	CadSNP306	*Desi*	Ca_Desi_Chr01	3677607	(G/A)
24712	CadSNP307	*Desi*	Ca_Desi_Chr01	3743152	(G/A)
24713	CadSNP308	*Desi*	Ca_Desi_Chr01	3771750	(C/T)
24714	CadSNP309	*Desi*	Ca_Desi_Chr01	3771899	(T/C)
24715	CadSNP310	*Desi*	Ca_Desi_Chr01	3783317	(G/A)
24716	CadSNP311	*Desi*	Ca_Desi_Chr01	3786526	(T/C)
24717	CadSNP312	*Desi*	Ca_Desi_Chr01	3786523	(C/T)
24718	CadSNP313	*Desi*	Ca_Desi_Chr01	3804312	(G/T)
24719	CadSNP314	*Desi*	Ca_Desi_Chr01	3877417	(G/A)
24720	CadSNP315	*Desi*	Ca_Desi_Chr01	3877438	(A/T)
24721	CadSNP316	*Desi*	Ca_Desi_Chr01	3911580	(A/G)
24722	CadSNP317	*Desi*	Ca_Desi_Chr01	3953451	(C/T)
24723	CadSNP318	*Desi*	Ca_Desi_Chr01	3963327	(T/C)
24724	CadSNP319	*Desi*	Ca_Desi_Chr01	3993197	(C/T)
24725	CadSNP320	*Desi*	Ca_Desi_Chr01	3993242	(T/G)
24726	CadSNP321	*Desi*	Ca_Desi_Chr01	3994131	(G/A)
24727	CadSNP322	*Desi*	Ca_Desi_Chr01	3995579	(C/T)
24728	CadSNP323	*Desi*	Ca_Desi_Chr01	3996356	(C/T)
24729	CadSNP324	*Desi*	Ca_Desi_Chr01	3996916	(C/T)
24730	CadSNP325	*Desi*	Ca_Desi_Chr01	5004852	(G/A)
24731	CadSNP326	*Desi*	Ca_Desi_Chr01	5004855	(A/C)
24732	CadSNP327	*Desi*	Ca_Desi_Chr01	5004857	(G/C)
24733	CadSNP328	*Desi*	Ca_Desi_Chr01	5004917	(C/A)
24734	CadSNP329	*Desi*	Ca_Desi_Chr01	5022262	(G/T)
24735	CadSNP330	*Desi*	Ca_Desi_Chr01	5022319	(G/A)
24736	CadSNP331	*Desi*	Ca_Desi_Chr01	5022390	(C/T)
24737	CadSNP332	*Desi*	Ca_Desi_Chr01	5039591	(A/G)

S.N.	SNP IDs	Cultivars	Chromosomes/scaffolds	Physical positions (bp)	SNPs
24738	CadSNP333	*Desi*	Ca_Desi_Chr01	5039614	(C/A)
24739	CadSNP334	*Desi*	Ca_Desi_Chr01	5040610	(A/G)
24740	CadSNP335	*Desi*	Ca_Desi_Chr01	5040663	(A/G)
24741	CadSNP336	*Desi*	Ca_Desi_Chr01	5056212	(T/C)
24742	CadSNP337	*Desi*	Ca_Desi_Chr01	5056260	(A/C)
24743	CadSNP338	*Desi*	Ca_Desi_Chr01	5056328	(T/C)
24744	CadSNP339	*Desi*	Ca_Desi_Chr01	5059326	(A/G)
24745	CadSNP340	*Desi*	Ca_Desi_Chr01	5061066	(T/A)
24746	CadSNP341	*Desi*	Ca_Desi_Chr01	5061125	(G/C)
24747	CadSNP342	*Desi*	Ca_Desi_Chr01	5069716	(A/G)
24748	CadSNP343	*Desi*	Ca_Desi_Chr01	5069743	(A/G)
24749	CadSNP344	*Desi*	Ca_Desi_Chr01	5094757	(G/C)
24750	CadSNP345	*Desi*	Ca_Desi_Chr01	5094752	(G/A)
24751	CadSNP346	*Desi*	Ca_Desi_Chr01	5161857	(C/T)
24752	CadSNP347	*Desi*	Ca_Desi_Chr01	5197254	(A/G)
24753	CadSNP348	*Desi*	Ca_Desi_Chr01	5197445	(C/A)
24754	CadSNP349	*Desi*	Ca_Desi_Chr01	5202104	(T/C)
24755	CadSNP350	*Desi*	Ca_Desi_Chr01	5202057	(A/T)
24756	CadSNP351	*Desi*	Ca_Desi_Chr01	5218664	(T/C)
24757	CadSNP352	*Desi*	Ca_Desi_Chr01	5227055	(C/A)
24758	CadSNP353	*Desi*	Ca_Desi_Chr01	5240171	(G/A)
24759	CadSNP354	*Desi*	Ca_Desi_Chr01	5292807	(A/G)
24760	CadSNP355	*Desi*	Ca_Desi_Chr01	5292810	(A/T)
24761	CadSNP356	*Desi*	Ca_Desi_Chr01	5348734	(A/G)
24762	CadSNP357	*Desi*	Ca_Desi_Chr01	5348941	(T/C)
24763	CadSNP358	*Desi*	Ca_Desi_Chr01	5428980	(C/A)
24764	CadSNP359	*Desi*	Ca_Desi_Chr01	5446596	(A/C)
24765	CadSNP360	*Desi*	Ca_Desi_Chr01	5446549	(A/C)
24766	CadSNP361	*Desi*	Ca_Desi_Chr01	5498868	(A/G)
24767	CadSNP362	*Desi*	Ca_Desi_Chr01	5498890	(A/G)
24768	CadSNP363	*Desi*	Ca_Desi_Chr01	5506578	(G/C)
24769	CadSNP364	*Desi*	Ca_Desi_Chr01	5511110	(A/T)
24770	CadSNP365	*Desi*	Ca_Desi_Chr01	5511140	(A/T)
24771	CadSNP366	*Desi*	Ca_Desi_Chr01	5511162	(T/G)
24772	CadSNP367	*Desi*	Ca_Desi_Chr01	5511149	(T/C)
24773	CadSNP368	*Desi*	Ca_Desi_Chr01	5537208	(C/T)
24774	CadSNP369	*Desi*	Ca_Desi_Chr01	5537211	(G/A)
24775	CadSNP370	*Desi*	Ca_Desi_Chr01	5537394	(A/G)

**S.N.**	**SNP IDs**	**Cultivars**	**Chromosomes/scaffolds**	**Physical positions (bp)**	**SNPs**
24776	CadSNP371	*Desi*	Ca_Desi_Chr01	5537397	(T/A)
24777	CadSNP372	*Desi*	Ca_Desi_Chr01	5537508	(C/T)
24778	CadSNP373	*Desi*	Ca_Desi_Chr01	5555967	(C/T)
24779	CadSNP374	*Desi*	Ca_Desi_Chr01	5556081	(G/A)
24780	CadSNP375	*Desi*	Ca_Desi_Chr01	5568121	(A/C)
24781	CadSNP376	*Desi*	Ca_Desi_Chr01	5580120	(C/T)
24782	CadSNP377	*Desi*	Ca_Desi_Chr01	5580193	(C/T)
24783	CadSNP378	*Desi*	Ca_Desi_Chr01	5605911	(G/C)
24784	CadSNP379	*Desi*	Ca_Desi_Chr01	5605923	(G/T)
24785	CadSNP380	*Desi*	Ca_Desi_Chr01	5613829	(C/A)
24786	CadSNP381	*Desi*	Ca_Desi_Chr01	5644131	(G/C)
24787	CadSNP382	*Desi*	Ca_Desi_Chr01	5645053	(C/T)
24788	CadSNP383	*Desi*	Ca_Desi_Chr01	5645050	(T/C)
24789	CadSNP384	*Desi*	Ca_Desi_Chr01	5645015	(T/C)
24790	CadSNP385	*Desi*	Ca_Desi_Chr01	5667771	(A/G)
24791	CadSNP386	*Desi*	Ca_Desi_Chr01	5676601	(T/C)
24792	CadSNP387	*Desi*	Ca_Desi_Chr01	5704265	(C/A)
24793	CadSNP388	*Desi*	Ca_Desi_Chr01	5753424	(G/A)
24794	CadSNP389	*Desi*	Ca_Desi_Chr01	5753449	(T/G)
24795	CadSNP390	*Desi*	Ca_Desi_Chr01	5753484	(T/G)
24796	CadSNP391	*Desi*	Ca_Desi_Chr01	5776338	(C/A)
24797	CadSNP392	*Desi*	Ca_Desi_Chr01	5776339	(A/G)
24798	CadSNP393	*Desi*	Ca_Desi_Chr01	5776369	(T/C)
24799	CadSNP394	*Desi*	Ca_Desi_Chr01	5776540	(T/C)
24800	CadSNP395	*Desi*	Ca_Desi_Chr01	5778096	(C/G)
24801	CadSNP396	*Desi*	Ca_Desi_Chr01	5807567	(C/G)
24802	CadSNP397	*Desi*	Ca_Desi_Chr01	5807642	(G/A)
24803	CadSNP398	*Desi*	Ca_Desi_Chr01	5823260	(C/T)
24804	CadSNP399	*Desi*	Ca_Desi_Chr01	5853627	(T/A)
24805	CadSNP400	*Desi*	Ca_Desi_Chr01	5853706	(G/A)
24806	CadSNP401	*Desi*	Ca_Desi_Chr01	5854373	(C/T)
24807	CadSNP402	*Desi*	Ca_Desi_Chr01	6037738	(T/G)
24808	CadSNP403	*Desi*	Ca_Desi_Chr01	6072236	(A/G)
24809	CadSNP404	*Desi*	Ca_Desi_Chr01	6072205	(A/G)
24810	CadSNP405	*Desi*	Ca_Desi_Chr01	6080835	(G/C)
24811	CadSNP406	*Desi*	Ca_Desi_Chr01	6080870	(T/C)
24812	CadSNP407	*Desi*	Ca_Desi_Chr01	6084027	(C/T)
24813	CadSNP408	*Desi*	Ca_Desi_Chr01	6083987	(T/G)

S.N.	SNP IDs	Cultivars	Chromosomes/scaffolds	Physical positions (bp)	SNPs
24814	CadSNP409	*Desi*	Ca_Desi_Chr01	6107667	(T/C)
24815	CadSNP410	*Desi*	Ca_Desi_Chr01	6107673	(A/C)
24816	CadSNP411	*Desi*	Ca_Desi_Chr01	6108004	(C/A)
24817	CadSNP412	*Desi*	Ca_Desi_Chr01	6108008	(T/G)
24818	CadSNP413	*Desi*	Ca_Desi_Chr01	6108016	(C/T)
24819	CadSNP414	*Desi*	Ca_Desi_Chr01	6108035	(T/A)
24820	CadSNP415	*Desi*	Ca_Desi_Chr01	6108091	(T/G)
24821	CadSNP416	*Desi*	Ca_Desi_Chr01	6108094	(C/T)
24822	CadSNP417	*Desi*	Ca_Desi_Chr01	6108141	(T/A)
24823	CadSNP418	*Desi*	Ca_Desi_Chr01	6108140	(A/G)
24824	CadSNP419	*Desi*	Ca_Desi_Chr01	6111677	(A/G)
24825	CadSNP420	*Desi*	Ca_Desi_Chr01	6111732	(T/G)
24826	CadSNP421	*Desi*	Ca_Desi_Chr01	6112469	(C/T)
24827	CadSNP422	*Desi*	Ca_Desi_Chr01	6112462	(G/A)
24828	CadSNP423	*Desi*	Ca_Desi_Chr01	6112461	(A/G)
24829	CadSNP424	*Desi*	Ca_Desi_Chr01	6112451	(A/G)
24830	CadSNP425	*Desi*	Ca_Desi_Chr01	6112443	(A/G)
24831	CadSNP426	*Desi*	Ca_Desi_Chr01	6112435	(C/A)
24832	CadSNP427	*Desi*	Ca_Desi_Chr01	6112434	(T/A)
24833	CadSNP428	*Desi*	Ca_Desi_Chr01	6112419	(T/G)
24834	CadSNP429	*Desi*	Ca_Desi_Chr01	6113487	(T/C)
24835	CadSNP430	*Desi*	Ca_Desi_Chr01	6113445	(T/C)
24836	CadSNP431	*Desi*	Ca_Desi_Chr01	6135902	(A/G)
24837	CadSNP432	*Desi*	Ca_Desi_Chr01	6141133	(A/C)
24838	CadSNP433	*Desi*	Ca_Desi_Chr01	6141126	(A/C)
24839	CadSNP434	*Desi*	Ca_Desi_Chr01	6149357	(C/A)
24840	CadSNP435	*Desi*	Ca_Desi_Chr01	6262953	(A/C)
24841	CadSNP436	*Desi*	Ca_Desi_Chr01	6262893	(T/A)
24842	CadSNP437	*Desi*	Ca_Desi_Chr01	6303938	(A/G)
24843	CadSNP438	*Desi*	Ca_Desi_Chr01	6303965	(T/G)
24844	CadSNP439	*Desi*	Ca_Desi_Chr01	6304541	(A/G)
24845	CadSNP440	*Desi*	Ca_Desi_Chr01	6313746	(T/A)
24846	CadSNP441	*Desi*	Ca_Desi_Chr01	6313759	(T/A)
24847	CadSNP442	*Desi*	Ca_Desi_Chr01	6318871	(G/T)
24848	CadSNP443	*Desi*	Ca_Desi_Chr01	6319012	(A/G)
24849	CadSNP444	*Desi*	Ca_Desi_Chr01	6319042	(A/C)
24850	CadSNP445	*Desi*	Ca_Desi_Chr01	6322174	(A/G)
24851	CadSNP446	*Desi*	Ca_Desi_Chr01	6322202	(G/T)

S.N.	SNP IDs	Cultivars	Chromosomes/scaffolds	Physical positions (bp)	SNPs
24852	CadSNP447	*Desi*	Ca_Desi_Chr01	6333247	(A/G)
24853	CadSNP448	*Desi*	Ca_Desi_Chr01	6333238	(C/T)
24854	CadSNP449	*Desi*	Ca_Desi_Chr01	6333418	(G/A)
24855	CadSNP450	*Desi*	Ca_Desi_Chr01	6341237	(C/T)
24856	CadSNP451	*Desi*	Ca_Desi_Chr01	6341213	(A/C)
24857	CadSNP452	*Desi*	Ca_Desi_Chr01	6351975	(T/C)
24858	CadSNP453	*Desi*	Ca_Desi_Chr01	6379951	(G/A)
24859	CadSNP454	*Desi*	Ca_Desi_Chr01	6399918	(C/T)
24860	CadSNP455	*Desi*	Ca_Desi_Chr01	6399989	(C/T)
24861	CadSNP456	*Desi*	Ca_Desi_Chr01	6400007	(C/T)
24862	CadSNP457	*Desi*	Ca_Desi_Chr01	6747779	(C/T)
24863	CadSNP458	*Desi*	Ca_Desi_Chr01	6750146	(A/G)
24864	CadSNP459	*Desi*	Ca_Desi_Chr01	6836227	(A/T)
24865	CadSNP460	*Desi*	Ca_Desi_Chr01	6858906	(T/A)
24866	CadSNP461	*Desi*	Ca_Desi_Chr01	6858922	(C/T)
24867	CadSNP462	*Desi*	Ca_Desi_Chr01	6858925	(C/T)
24868	CadSNP463	*Desi*	Ca_Desi_Chr01	6858952	(A/C)
24869	CadSNP464	*Desi*	Ca_Desi_Chr01	6858964	(A/C)
24870	CadSNP465	*Desi*	Ca_Desi_Chr01	6859408	(C/A)
24871	CadSNP466	*Desi*	Ca_Desi_Chr01	6859661	(C/A)
24872	CadSNP467	*Desi*	Ca_Desi_Chr01	6859634	(T/C)
24873	CadSNP468	*Desi*	Ca_Desi_Chr01	6859628	(G/A)
24874	CadSNP469	*Desi*	Ca_Desi_Chr01	6859622	(A/G)
24875	CadSNP470	*Desi*	Ca_Desi_Chr01	6861332	(T/A)
24876	CadSNP471	*Desi*	Ca_Desi_Chr01	6862834	(C/A)
24877	CadSNP472	*Desi*	Ca_Desi_Chr01	6862836	(T/G)
24878	CadSNP473	*Desi*	Ca_Desi_Chr01	6862866	(A/G)
24879	CadSNP474	*Desi*	Ca_Desi_Chr01	6862869	(C/T)
24880	CadSNP475	*Desi*	Ca_Desi_Chr01	6862872	(A/T)
24881	CadSNP476	*Desi*	Ca_Desi_Chr01	6862873	(A/C)
24882	CadSNP477	*Desi*	Ca_Desi_Chr01	6862881	(A/C)
24883	CadSNP478	*Desi*	Ca_Desi_Chr01	6862888	(T/A)
24884	CadSNP479	*Desi*	Ca_Desi_Chr01	6862889	(A/C)
24885	CadSNP480	*Desi*	Ca_Desi_Chr01	6862893	(T/C)
24886	CadSNP481	*Desi*	Ca_Desi_Chr01	7013189	(A/G)
24887	CadSNP482	*Desi*	Ca_Desi_Chr01	7142598	(C/T)
24888	CadSNP483	*Desi*	Ca_Desi_Chr01	7168613	(G/A)
24889	CadSNP484	*Desi*	Ca_Desi_Chr01	7274246	(C/T)

S.N.	SNP IDs	Cultivars	Chromosomes/scaffolds	Physical positions (bp)	SNPs
24890	CadSNP485	*Desi*	Ca_Desi_Chr01	7279148	(G/A)
24891	CadSNP486	*Desi*	Ca_Desi_Chr01	7279147	(G/A)
24892	CadSNP487	*Desi*	Ca_Desi_Chr01	7279116	(G/A)
24893	CadSNP488	*Desi*	Ca_Desi_Chr01	7279115	(T/C)
24894	CadSNP489	*Desi*	Ca_Desi_Chr01	7279106	(G/T)
24895	CadSNP490	*Desi*	Ca_Desi_Chr01	7279097	(G/A)
24896	CadSNP491	*Desi*	Ca_Desi_Chr01	7289926	(G/A)
24897	CadSNP492	*Desi*	Ca_Desi_Chr01	7289965	(G/A)
24898	CadSNP493	*Desi*	Ca_Desi_Chr01	7331882	(T/C)
24899	CadSNP494	*Desi*	Ca_Desi_Chr01	7331912	(C/G)
24900	CadSNP495	*Desi*	Ca_Desi_Chr01	7331936	(C/T)
24901	CadSNP496	*Desi*	Ca_Desi_Chr01	7331994	(G/A)
24902	CadSNP497	*Desi*	Ca_Desi_Chr01	7331987	(C/T)
24903	CadSNP498	*Desi*	Ca_Desi_Chr01	7383196	(A/C)
24904	CadSNP499	*Desi*	Ca_Desi_Chr01	7383206	(A/G)
24905	CadSNP500	*Desi*	Ca_Desi_Chr01	7383207	(G/A)
24906	CadSNP501	*Desi*	Ca_Desi_Chr01	7383208	(T/A)
24907	CadSNP502	*Desi*	Ca_Desi_Chr01	7383224	(A/G)
24908	CadSNP503	*Desi*	Ca_Desi_Chr01	7383225	(G/A)
24909	CadSNP504	*Desi*	Ca_Desi_Chr01	7383226	(C/A)
24910	CadSNP505	*Desi*	Ca_Desi_Chr01	7383229	(G/A)
24911	CadSNP506	*Desi*	Ca_Desi_Chr01	7383232	(G/A)
24912	CadSNP507	*Desi*	Ca_Desi_Chr01	7383255	(A/G)
24913	CadSNP508	*Desi*	Ca_Desi_Chr01	7383256	(G/A)
24914	CadSNP509	*Desi*	Ca_Desi_Chr01	7383259	(G/A)
24915	CadSNP510	*Desi*	Ca_Desi_Chr01	7383265	(T/G)
24916	CadSNP511	*Desi*	Ca_Desi_Chr01	7383271	(A/G)
24917	CadSNP512	*Desi*	Ca_Desi_Chr01	7383274	(C/T)
24918	CadSNP513	*Desi*	Ca_Desi_Chr01	7383281	(G/A)
24919	CadSNP514	*Desi*	Ca_Desi_Chr01	7383437	(T/C)
24920	CadSNP515	*Desi*	Ca_Desi_Chr01	7383436	(G/A)
24921	CadSNP516	*Desi*	Ca_Desi_Chr01	7383433	(T/A)
24922	CadSNP517	*Desi*	Ca_Desi_Chr01	7383406	(C/T)
24923	CadSNP518	*Desi*	Ca_Desi_Chr01	7383808	(A/T)
24924	CadSNP519	*Desi*	Ca_Desi_Chr01	7436170	(T/A)
24925	CadSNP520	*Desi*	Ca_Desi_Chr01	7436190	(T/G)
24926	CadSNP521	*Desi*	Ca_Desi_Chr01	7436196	(G/A)
24927	CadSNP522	*Desi*	Ca_Desi_Chr01	7436205	(T/G)

S.N.	SNP IDs	Cultivars	Chromosomes/scaffolds	Physical positions (bp)	SNPs
24928	CadSNP523	*Desi*	Ca_Desi_Chr01	7448000	(C/G)
24929	CadSNP524	*Desi*	Ca_Desi_Chr01	7489519	(T/A)
24930	CadSNP525	*Desi*	Ca_Desi_Chr01	7489492	(T/A)
24931	CadSNP526	*Desi*	Ca_Desi_Chr01	7489471	(T/A)
24932	CadSNP527	*Desi*	Ca_Desi_Chr01	7489452	(T/G)
24933	CadSNP528	*Desi*	Ca_Desi_Chr01	7489451	(A/T)
24934	CadSNP529	*Desi*	Ca_Desi_Chr01	7489447	(C/A)
24935	CadSNP530	*Desi*	Ca_Desi_Chr01	7606885	(C/G)
24936	CadSNP531	*Desi*	Ca_Desi_Chr01	7606933	(C/T)
24937	CadSNP532	*Desi*	Ca_Desi_Chr01	7632307	(A/G)
24938	CadSNP533	*Desi*	Ca_Desi_Chr01	7632356	(C/T)
24939	CadSNP534	*Desi*	Ca_Desi_Chr01	7668717	(G/A)
24940	CadSNP535	*Desi*	Ca_Desi_Chr01	7669506	(G/C)
24941	CadSNP536	*Desi*	Ca_Desi_Chr01	7669603	(G/C)
24942	CadSNP537	*Desi*	Ca_Desi_Chr01	7671799	(A/G)
24943	CadSNP538	*Desi*	Ca_Desi_Chr01	7671815	(G/A)
24944	CadSNP539	*Desi*	Ca_Desi_Chr01	7671819	(T/C)
24945	CadSNP540	*Desi*	Ca_Desi_Chr01	7671842	(A/T)
24946	CadSNP541	*Desi*	Ca_Desi_Chr01	7671878	(T/G)
24947	CadSNP542	*Desi*	Ca_Desi_Chr01	7671931	(C/A)
24948	CadSNP543	*Desi*	Ca_Desi_Chr01	7671947	(C/T)
24949	CadSNP544	*Desi*	Ca_Desi_Chr01	7671963	(C/T)
24950	CadSNP545	*Desi*	Ca_Desi_Chr01	7676039	(G/C)
24951	CadSNP546	*Desi*	Ca_Desi_Chr01	7680425	(A/G)
24952	CadSNP547	*Desi*	Ca_Desi_Chr01	7680457	(C/T)
24953	CadSNP548	*Desi*	Ca_Desi_Chr01	7680543	(C/T)
24954	CadSNP549	*Desi*	Ca_Desi_Chr01	7712801	(A/C)
24955	CadSNP550	*Desi*	Ca_Desi_Chr01	7712821	(C/T)
24956	CadSNP551	*Desi*	Ca_Desi_Chr01	7956977	(T/G)
24957	CadSNP552	*Desi*	Ca_Desi_Chr01	7957053	(G/A)
24958	CadSNP553	*Desi*	Ca_Desi_Chr01	8163297	(C/A)
24959	CadSNP554	*Desi*	Ca_Desi_Chr01	8173005	(T/C)
24960	CadSNP555	*Desi*	Ca_Desi_Chr01	8219717	(A/G)
24961	CadSNP556	*Desi*	Ca_Desi_Chr01	8220943	(G/A)
24962	CadSNP557	*Desi*	Ca_Desi_Chr01	8220952	(C/T)
24963	CadSNP558	*Desi*	Ca_Desi_Chr01	8221152	(G/A)
24964	CadSNP559	*Desi*	Ca_Desi_Chr01	8279866	(T/G)
24965	CadSNP560	*Desi*	Ca_Desi_Chr01	8280837	(C/A)

S.N.	SNP IDs	Cultivars	Chromosomes/scaffolds	Physical positions (bp)	SNPs
24966	CadSNP561	*Desi*	Ca_Desi_Chr01	8280838	(C/G)
24967	CadSNP562	*Desi*	Ca_Desi_Chr01	8297933	(A/G)
24968	CadSNP563	*Desi*	Ca_Desi_Chr01	8365636	(A/C)
24969	CadSNP564	*Desi*	Ca_Desi_Chr01	8390528	(C/T)
24970	CadSNP565	*Desi*	Ca_Desi_Chr01	8397872	(C/T)
24971	CadSNP566	*Desi*	Ca_Desi_Chr01	8398204	(G/T)
24972	CadSNP567	*Desi*	Ca_Desi_Chr01	8416775	(G/A)
24973	CadSNP568	*Desi*	Ca_Desi_Chr01	8422096	(G/T)
24974	CadSNP569	*Desi*	Ca_Desi_Chr01	8422113	(G/T)
24975	CadSNP570	*Desi*	Ca_Desi_Chr01	8426569	(G/A)
24976	CadSNP571	*Desi*	Ca_Desi_Chr01	8492439	(T/A)
24977	CadSNP572	*Desi*	Ca_Desi_Chr01	8492480	(A/T)
24978	CadSNP573	*Desi*	Ca_Desi_Chr01	8492496	(A/T)
24979	CadSNP574	*Desi*	Ca_Desi_Chr01	8492493	(A/C)
24980	CadSNP575	*Desi*	Ca_Desi_Chr01	8492492	(A/G)
24981	CadSNP576	*Desi*	Ca_Desi_Chr01	8506952	(G/A)
24982	CadSNP577	*Desi*	Ca_Desi_Chr01	8506982	(A/G)
24983	CadSNP578	*Desi*	Ca_Desi_Chr01	8561851	(G/A)
24984	CadSNP579	*Desi*	Ca_Desi_Chr01	8689520	(A/C)
24985	CadSNP580	*Desi*	Ca_Desi_Chr01	8689473	(C/T)
24986	CadSNP581	*Desi*	Ca_Desi_Chr01	8725597	(G/A)
24987	CadSNP582	*Desi*	Ca_Desi_Chr01	8725599	(C/A)
24988	CadSNP583	*Desi*	Ca_Desi_Chr01	8725654	(A/T)
24989	CadSNP584	*Desi*	Ca_Desi_Chr01	8772201	(A/G)
24990	CadSNP585	*Desi*	Ca_Desi_Chr01	8786857	(C/A)
24991	CadSNP586	*Desi*	Ca_Desi_Chr01	8871945	(C/T)
24992	CadSNP587	*Desi*	Ca_Desi_Chr01	8902125	(C/A)
24993	CadSNP588	*Desi*	Ca_Desi_Chr01	8952943	(A/G)
24994	CadSNP589	*Desi*	Ca_Desi_Chr01	8988064	(G/A)
24995	CadSNP590	*Desi*	Ca_Desi_Chr01	8988049	(G/A)
24996	CadSNP591	*Desi*	Ca_Desi_Chr01	9014610	(C/A)
24997	CadSNP592	*Desi*	Ca_Desi_Chr01	9022986	(G/A)
24998	CadSNP593	*Desi*	Ca_Desi_Chr01	9023240	(T/C)
24999	CadSNP594	*Desi*	Ca_Desi_Chr01	9023290	(G/A)
25000	CadSNP595	*Desi*	Ca_Desi_Chr01	9033783	(T/C)
25001	CadSNP596	*Desi*	Ca_Desi_Chr01	9089686	(T/C)
25002	CadSNP597	*Desi*	Ca_Desi_Chr01	9193835	(G/A)
25003	CadSNP598	*Desi*	Ca_Desi_Chr01	9217460	(C/T)

S.N.	SNP IDs	Cultivars	Chromosomes/scaffolds	Physical positions (bp)	SNPs
25004	CadSNP599	*Desi*	Ca_Desi_Chr01	9217685	(A/G)
25005	CadSNP600	*Desi*	Ca_Desi_Chr01	9217840	(G/T)
25006	CadSNP601	*Desi*	Ca_Desi_Chr01	9218827	(T/G)
25007	CadSNP602	*Desi*	Ca_Desi_Chr01	9218838	(A/C)
25008	CadSNP603	*Desi*	Ca_Desi_Chr01	9218853	(G/A)
25009	CadSNP604	*Desi*	Ca_Desi_Chr01	9218890	(C/A)
25010	CadSNP605	*Desi*	Ca_Desi_Chr01	9298126	(T/G)
25011	CadSNP606	*Desi*	Ca_Desi_Chr01	9298398	(A/G)
25012	CadSNP607	*Desi*	Ca_Desi_Chr01	9306517	(A/C)
25013	CadSNP608	*Desi*	Ca_Desi_Chr01	9308560	(T/C)
25014	CadSNP609	*Desi*	Ca_Desi_Chr01	9308653	(C/T)
25015	CadSNP610	*Desi*	Ca_Desi_Chr01	9354522	(G/A)
25016	CadSNP611	*Desi*	Ca_Desi_Chr01	9354518	(T/G)
25017	CadSNP612	*Desi*	Ca_Desi_Chr01	9361145	(G/T)
25018	CadSNP613	*Desi*	Ca_Desi_Chr01	9362408	(G/A)
25019	CadSNP614	*Desi*	Ca_Desi_Chr01	9761124	(A/G)
25020	CadSNP615	*Desi*	Ca_Desi_Chr01	9761183	(A/G)
25021	CadSNP616	*Desi*	Ca_Desi_Chr01	9761192	(T/G)
25022	CadSNP617	*Desi*	Ca_Desi_Chr01	9761256	(T/C)
25023	CadSNP618	*Desi*	Ca_Desi_Chr01	10000773	(T/C)
25024	CadSNP619	*Desi*	Ca_Desi_Chr01	10002060	(A/C)
25025	CadSNP620	*Desi*	Ca_Desi_Chr01	10012819	(T/C)
25026	CadSNP621	*Desi*	Ca_Desi_Chr01	10012821	(G/A)
25027	CadSNP622	*Desi*	Ca_Desi_Chr01	10039308	(T/A)
25028	CadSNP623	*Desi*	Ca_Desi_Chr01	10078804	(A/G)
25029	CadSNP624	*Desi*	Ca_Desi_Chr01	10106781	(G/C)
25030	CadSNP625	*Desi*	Ca_Desi_Chr01	10311695	(T/C)
25031	CadSNP626	*Desi*	Ca_Desi_Chr01	10322769	(C/T)
25032	CadSNP627	*Desi*	Ca_Desi_Chr01	10331572	(A/G)
25033	CadSNP628	*Desi*	Ca_Desi_Chr01	10331598	(G/A)
25034	CadSNP629	*Desi*	Ca_Desi_Chr01	10371032	(G/A)
25035	CadSNP630	*Desi*	Ca_Desi_Chr01	10441613	(G/A)
25036	CadSNP631	*Desi*	Ca_Desi_Chr01	10441719	(A/G)
25037	CadSNP632	*Desi*	Ca_Desi_Chr01	10441699	(G/A)
25038	CadSNP633	*Desi*	Ca_Desi_Chr01	10441697	(A/C)
25039	CadSNP634	*Desi*	Ca_Desi_Chr01	10441800	(C/A)
25040	CadSNP635	*Desi*	Ca_Desi_Chr01	10441806	(C/A)
25041	CadSNP636	*Desi*	Ca_Desi_Chr01	10441837	(G/A)

S.N.	SNP IDs	Cultivars	Chromosomes/scaffolds	Physical positions (bp)	SNPs
25042	CadSNP637	*Desi*	Ca_Desi_Chr01	10474199	(G/A)
25043	CadSNP638	*Desi*	Ca_Desi_Chr01	10530999	(T/C)
25044	CadSNP639	*Desi*	Ca_Desi_Chr01	10608390	(A/T)
25045	CadSNP640	*Desi*	Ca_Desi_Chr01	10707824	(G/T)
25046	CadSNP641	*Desi*	Ca_Desi_Chr01	10719893	(T/G)
25047	CadSNP642	*Desi*	Ca_Desi_Chr01	10719894	(T/A)
25048	CadSNP643	*Desi*	Ca_Desi_Chr01	10720003	(A/T)
25049	CadSNP644	*Desi*	Ca_Desi_Chr01	10723301	(C/T)
25050	CadSNP645	*Desi*	Ca_Desi_Chr01	10741069	(G/C)
25051	CadSNP646	*Desi*	Ca_Desi_Chr01	10741089	(A/G)
25052	CadSNP647	*Desi*	Ca_Desi_Chr01	10754555	(C/G)
25053	CadSNP648	*Desi*	Ca_Desi_Chr01	10763266	(T/C)
25054	CadSNP649	*Desi*	Ca_Desi_Chr01	10827427	(C/T)
25055	CadSNP650	*Desi*	Ca_Desi_Chr01	10876770	(A/G)
25056	CadSNP651	*Desi*	Ca_Desi_Chr01	10876828	(C/A)
25057	CadSNP652	*Desi*	Ca_Desi_Chr01	10943212	(T/C)
25058	CadSNP653	*Desi*	Ca_Desi_Chr01	10972557	(C/T)
25059	CadSNP654	*Desi*	Ca_Desi_Chr01	10972556	(G/A)
25060	CadSNP655	*Desi*	Ca_Desi_Chr01	11104634	(G/T)
25061	CadSNP656	*Desi*	Ca_Desi_Chr01	11104890	(C/T)
25062	CadSNP657	*Desi*	Ca_Desi_Chr01	11106063	(A/T)
25063	CadSNP658	*Desi*	Ca_Desi_Chr01	11106278	(C/T)
25064	CadSNP659	*Desi*	Ca_Desi_Chr01	11109156	(G/C)
25065	CadSNP660	*Desi*	Ca_Desi_Chr01	11109187	(A/T)
25066	CadSNP661	*Desi*	Ca_Desi_Chr01	11140259	(G/T)
25067	CadSNP662	*Desi*	Ca_Desi_Chr01	11140249	(A/C)
25068	CadSNP663	*Desi*	Ca_Desi_Chr01	11140319	(A/T)
25069	CadSNP664	*Desi*	Ca_Desi_Chr01	11140333	(G/A)
25070	CadSNP665	*Desi*	Ca_Desi_Chr01	11153637	(G/T)
25071	CadSNP666	*Desi*	Ca_Desi_Chr01	11188515	(T/C)
25072	CadSNP667	*Desi*	Ca_Desi_Chr01	11188519	(A/G)
25073	CadSNP668	*Desi*	Ca_Desi_Chr01	11203214	(A/G)
25074	CadSNP669	*Desi*	Ca_Desi_Chr01	11242051	(C/A)
25075	CadSNP670	*Desi*	Ca_Desi_Chr01	11242086	(A/T)
25076	CadSNP671	*Desi*	Ca_Desi_Chr01	11242117	(C/T)
25077	CadSNP672	*Desi*	Ca_Desi_Chr01	11255650	(A/C)
25078	CadSNP673	*Desi*	Ca_Desi_Chr01	11255645	(A/C)
25079	CadSNP674	*Desi*	Ca_Desi_Chr01	11258827	(A/T)

S.N.	SNP IDs	Cultivars	Chromosomes/scaffolds	Physical positions (bp)	SNPs
25080	CadSNP675	*Desi*	Ca_Desi_Chr01	11302818	(G/A)
25081	CadSNP676	*Desi*	Ca_Desi_Chr01	11302861	(G/A)
25082	CadSNP677	*Desi*	Ca_Desi_Chr01	11302870	(A/G)
25083	CadSNP678	*Desi*	Ca_Desi_Chr01	11305587	(T/G)
25084	CadSNP679	*Desi*	Ca_Desi_Chr01	11368294	(C/A)
25085	CadSNP680	*Desi*	Ca_Desi_Chr01	11411238	(A/G)
25086	CadSNP681	*Desi*	Ca_Desi_Chr01	11411433	(G/C)
25087	CadSNP682	*Desi*	Ca_Desi_Chr01	11447597	(C/T)
25088	CadSNP683	*Desi*	Ca_Desi_Chr01	11447611	(C/T)
25089	CadSNP684	*Desi*	Ca_Desi_Chr01	11447639	(T/C)
25090	CadSNP685	*Desi*	Ca_Desi_Chr01	11447647	(C/T)
25091	CadSNP686	*Desi*	Ca_Desi_Chr01	11447673	(T/C)
25092	CadSNP687	*Desi*	Ca_Desi_Chr01	11470235	(A/G)
25093	CadSNP688	*Desi*	Ca_Desi_Chr01	11479650	(A/T)
25094	CadSNP689	*Desi*	Ca_Desi_Chr01	11520563	(T/A)
25095	CadSNP690	*Desi*	Ca_Desi_Chr01	11520595	(T/C)
25096	CadSNP691	*Desi*	Ca_Desi_Chr01	11568882	(C/T)
25097	CadSNP692	*Desi*	Ca_Desi_Chr01	11569800	(A/G)
25098	CadSNP693	*Desi*	Ca_Desi_Chr01	11569811	(A/C)
25099	CadSNP694	*Desi*	Ca_Desi_Chr01	11586934	(G/T)
25100	CadSNP695	*Desi*	Ca_Desi_Chr01	11588193	(C/T)
25101	CadSNP696	*Desi*	Ca_Desi_Chr01	11588259	(G/A)
25102	CadSNP697	*Desi*	Ca_Desi_Chr01	11900706	(G/C)
25103	CadSNP698	*Desi*	Ca_Desi_Chr01	11906460	(A/C)
25104	CadSNP699	*Desi*	Ca_Desi_Chr01	11909644	(A/C)
25105	CadSNP700	*Desi*	Ca_Desi_Chr01	12045526	(G/A)
25106	CadSNP701	*Desi*	Ca_Desi_Chr01	12155983	(T/G)
25107	CadSNP702	*Desi*	Ca_Desi_Chr01	12159674	(A/C)
25108	CadSNP703	*Desi*	Ca_Desi_Chr01	12163294	(C/A)
25109	CadSNP704	*Desi*	Ca_Desi_Chr01	12171484	(A/G)
25110	CadSNP705	*Desi*	Ca_Desi_Chr01	12171505	(T/C)
25111	CadSNP706	*Desi*	Ca_Desi_Chr01	12191974	(C/T)
25112	CadSNP707	*Desi*	Ca_Desi_Chr01	12192017	(G/C)
25113	CadSNP708	*Desi*	Ca_Desi_Chr01	12192106	(A/C)
25114	CadSNP709	*Desi*	Ca_Desi_Chr01	12192068	(C/G)
25115	CadSNP710	*Desi*	Ca_Desi_Chr01	13563876	(T/G)
25116	CadSNP711	*Desi*	Ca_Desi_Chr01	13585102	(C/G)
25117	CadSNP712	*Desi*	Ca_Desi_Chr01	13585162	(T/A)

S.N.	SNP IDs	Cultivars	Chromosomes/scaffolds	Physical positions (bp)	SNPs
25118	CadSNP713	*Desi*	Ca_Desi_Chr01	13590335	(A/G)
25119	CadSNP714	*Desi*	Ca_Desi_Chr01	13602425	(C/A)
25120	CadSNP715	*Desi*	Ca_Desi_Chr01	13620048	(C/T)
25121	CadSNP716	*Desi*	Ca_Desi_Chr01	13641367	(C/T)
25122	CadSNP717	*Desi*	Ca_Desi_Chr01	13661302	(A/G)
25123	CadSNP718	*Desi*	Ca_Desi_Chr01	13661337	(G/A)
25124	CadSNP719	*Desi*	Ca_Desi_Chr01	13690923	(G/A)
25125	CadSNP720	*Desi*	Ca_Desi_Chr01	13737053	(G/A)
25126	CadSNP721	*Desi*	Ca_Desi_Chr01	13748421	(C/G)
25127	CadSNP722	*Desi*	Ca_Desi_Chr01	13766314	(G/A)
25128	CadSNP723	*Desi*	Ca_Desi_Chr01	13798808	(A/T)
25129	CadSNP724	*Desi*	Ca_Desi_Chr01	13834782	(G/C)
25130	CadSNP725	*Desi*	Ca_Desi_Chr01	13843833	(C/T)
25131	CadSNP726	*Desi*	Ca_Desi_Chr01	13843906	(G/A)
25132	CadSNP727	*Desi*	Ca_Desi_Chr01	13843980	(C/A)
25133	CadSNP728	*Desi*	Ca_Desi_Chr01	13843964	(C/G)
25134	CadSNP729	*Desi*	Ca_Desi_Chr01	13843940	(C/T)
25135	CadSNP730	*Desi*	Ca_Desi_Chr01	13844080	(C/T)
25136	CadSNP731	*Desi*	Ca_Desi_Chr01	13844081	(A/T)
25137	CadSNP732	*Desi*	Ca_Desi_Chr01	13844082	(A/C)
25138	CadSNP733	*Desi*	Ca_Desi_Chr01	13844150	(G/C)
25139	CadSNP734	*Desi*	Ca_Desi_Chr01	13852748	(A/G)
25140	CadSNP735	*Desi*	Ca_Desi_Chr01	13852733	(G/A)
25141	CadSNP736	*Desi*	Ca_Desi_Chr01	13852713	(C/T)
25142	CadSNP737	*Desi*	Ca_Desi_Chr01	13853257	(T/C)
25143	CadSNP738	*Desi*	Ca_Desi_Chr01	13853344	(T/C)
25144	CadSNP739	*Desi*	Ca_Desi_Chr01	13864949	(G/A)
25145	CadSNP740	*Desi*	Ca_Desi_Chr01	13927517	(T/C)
25146	CadSNP741	*Desi*	Ca_Desi_Chr01	13927520	(T/C)
25147	CadSNP742	*Desi*	Ca_Desi_Chr01	13927698	(T/G)
25148	CadSNP743	*Desi*	Ca_Desi_Chr01	13934729	(G/T)
25149	CadSNP744	*Desi*	Ca_Desi_Chr01	13935583	(G/A)
25150	CadSNP745	*Desi*	Ca_Desi_Chr01	13969163	(T/G)
25151	CadSNP746	*Desi*	Ca_Desi_Chr01	13969143	(A/C)
25152	CadSNP747	*Desi*	Ca_Desi_Chr01	14093206	(T/A)
25153	CadSNP748	*Desi*	Ca_Desi_Chr01	14113511	(G/C)
25154	CadSNP749	*Desi*	Ca_Desi_Chr01	14113485	(A/G)
25155	CadSNP750	*Desi*	Ca_Desi_Chr01	14113598	(C/T)

S.N.	SNP IDs	Cultivars	Chromosomes/scaffolds	Physical positions (bp)	SNPs
25156	CadSNP751	*Desi*	Ca_Desi_Chr01	14114903	(A/C)
25157	CadSNP752	*Desi*	Ca_Desi_Chr01	14128326	(T/G)
25158	CadSNP753	*Desi*	Ca_Desi_Chr01	14161553	(G/T)
25159	CadSNP754	*Desi*	Ca_Desi_Chr02	16503	(A/C)
25160	CadSNP755	*Desi*	Ca_Desi_Chr02	16504	(G/A)
25161	CadSNP756	*Desi*	Ca_Desi_Chr02	82708	(C/G)
25162	CadSNP757	*Desi*	Ca_Desi_Chr02	179194	(C/G)
25163	CadSNP758	*Desi*	Ca_Desi_Chr02	179173	(G/T)
25164	CadSNP759	*Desi*	Ca_Desi_Chr02	179260	(T/A)
25165	CadSNP760	*Desi*	Ca_Desi_Chr02	187800	(A/G)
25166	CadSNP761	*Desi*	Ca_Desi_Chr02	201869	(T/C)
25167	CadSNP762	*Desi*	Ca_Desi_Chr02	205617	(C/T)
25168	CadSNP763	*Desi*	Ca_Desi_Chr02	281905	(T/C)
25169	CadSNP764	*Desi*	Ca_Desi_Chr02	303118	(C/G)
25170	CadSNP765	*Desi*	Ca_Desi_Chr02	303138	(A/G)
25171	CadSNP766	*Desi*	Ca_Desi_Chr02	318397	(A/C)
25172	CadSNP767	*Desi*	Ca_Desi_Chr02	318414	(A/G)
25173	CadSNP768	*Desi*	Ca_Desi_Chr02	328850	(G/A)
25174	CadSNP769	*Desi*	Ca_Desi_Chr02	328828	(C/T)
25175	CadSNP770	*Desi*	Ca_Desi_Chr02	398016	(C/T)
25176	CadSNP771	*Desi*	Ca_Desi_Chr02	429417	(T/C)
25177	CadSNP772	*Desi*	Ca_Desi_Chr02	440499	(C/G)
25178	CadSNP773	*Desi*	Ca_Desi_Chr02	440520	(G/A)
25179	CadSNP774	*Desi*	Ca_Desi_Chr02	440592	(A/T)
25180	CadSNP775	*Desi*	Ca_Desi_Chr02	440595	(T/C)
25181	CadSNP776	*Desi*	Ca_Desi_Chr02	440600	(T/A)
25182	CadSNP777	*Desi*	Ca_Desi_Chr02	440607	(A/G)
25183	CadSNP778	*Desi*	Ca_Desi_Chr02	440754	(T/A)
25184	CadSNP779	*Desi*	Ca_Desi_Chr02	440724	(C/T)
25185	CadSNP780	*Desi*	Ca_Desi_Chr02	440718	(G/T)
25186	CadSNP781	*Desi*	Ca_Desi_Chr02	527661	(G/A)
25187	CadSNP782	*Desi*	Ca_Desi_Chr02	527635	(C/A)
25188	CadSNP783	*Desi*	Ca_Desi_Chr02	527634	(C/A)
25189	CadSNP784	*Desi*	Ca_Desi_Chr02	527633	(T/A)
25190	CadSNP785	*Desi*	Ca_Desi_Chr02	575365	(T/G)
25191	CadSNP786	*Desi*	Ca_Desi_Chr02	575518	(A/G)
25192	CadSNP787	*Desi*	Ca_Desi_Chr02	576524	(G/A)
25193	CadSNP788	*Desi*	Ca_Desi_Chr02	576624	(A/G)

S.N.	SNP IDs	Cultivars	Chromosomes/scaffolds	Physical positions (bp)	SNPs
25194	CadSNP789	*Desi*	Ca_Desi_Chr02	579705	(G/A)
25195	CadSNP790	*Desi*	Ca_Desi_Chr02	580263	(C/T)
25196	CadSNP791	*Desi*	Ca_Desi_Chr02	595836	(T/A)
25197	CadSNP792	*Desi*	Ca_Desi_Chr02	595837	(C/A)
25198	CadSNP793	*Desi*	Ca_Desi_Chr02	595842	(T/A)
25199	CadSNP794	*Desi*	Ca_Desi_Chr02	595845	(G/A)
25200	CadSNP795	*Desi*	Ca_Desi_Chr02	595850	(A/T)
25201	CadSNP796	*Desi*	Ca_Desi_Chr02	595851	(T/A)
25202	CadSNP797	*Desi*	Ca_Desi_Chr02	595853	(C/A)
25203	CadSNP798	*Desi*	Ca_Desi_Chr02	595863	(G/A)
25204	CadSNP799	*Desi*	Ca_Desi_Chr02	727262	(G/C)
25205	CadSNP800	*Desi*	Ca_Desi_Chr02	749831	(C/G)
25206	CadSNP801	*Desi*	Ca_Desi_Chr02	760148	(C/T)
25207	CadSNP802	*Desi*	Ca_Desi_Chr02	842935	(T/C)
25208	CadSNP803	*Desi*	Ca_Desi_Chr02	842908	(T/C)
25209	CadSNP804	*Desi*	Ca_Desi_Chr02	842943	(T/C)
25210	CadSNP805	*Desi*	Ca_Desi_Chr02	930040	(T/C)
25211	CadSNP806	*Desi*	Ca_Desi_Chr02	972909	(A/T)
25212	CadSNP807	*Desi*	Ca_Desi_Chr02	972918	(C/A)
25213	CadSNP808	*Desi*	Ca_Desi_Chr02	972921	(T/C)
25214	CadSNP809	*Desi*	Ca_Desi_Chr02	972930	(G/A)
25215	CadSNP810	*Desi*	Ca_Desi_Chr02	972933	(A/G)
25216	CadSNP811	*Desi*	Ca_Desi_Chr02	992292	(C/A)
25217	CadSNP812	*Desi*	Ca_Desi_Chr02	1019748	(G/A)
25218	CadSNP813	*Desi*	Ca_Desi_Chr02	1019708	(A/T)
25219	CadSNP814	*Desi*	Ca_Desi_Chr02	1019692	(G/T)
25220	CadSNP815	*Desi*	Ca_Desi_Chr02	1019793	(C/T)
25221	CadSNP816	*Desi*	Ca_Desi_Chr02	1048681	(G/T)
25222	CadSNP817	*Desi*	Ca_Desi_Chr02	1048657	(G/A)
25223	CadSNP818	*Desi*	Ca_Desi_Chr02	1066639	(A/C)
25224	CadSNP819	*Desi*	Ca_Desi_Chr02	1066693	(A/T)
25225	CadSNP820	*Desi*	Ca_Desi_Chr02	1066672	(T/A)
25226	CadSNP821	*Desi*	Ca_Desi_Chr02	1066647	(A/T)
25227	CadSNP822	*Desi*	Ca_Desi_Chr02	1081581	(T/C)
25228	CadSNP823	*Desi*	Ca_Desi_Chr02	1147386	(A/G)
25229	CadSNP824	*Desi*	Ca_Desi_Chr02	1147402	(T/C)
25230	CadSNP825	*Desi*	Ca_Desi_Chr02	1151458	(G/T)
25231	CadSNP826	*Desi*	Ca_Desi_Chr02	1205892	(A/C)

S.N.	SNP IDs	Cultivars	Chromosomes/scaffolds	Physical positions (bp)	SNPs
25232	CadSNP827	*Desi*	Ca_Desi_Chr02	1220563	(G/C)
25233	CadSNP828	*Desi*	Ca_Desi_Chr02	1367073	(C/A)
25234	CadSNP829	*Desi*	Ca_Desi_Chr02	1369594	(G/T)
25235	CadSNP830	*Desi*	Ca_Desi_Chr02	1369598	(G/T)
25236	CadSNP831	*Desi*	Ca_Desi_Chr02	1521985	(A/C)
25237	CadSNP832	*Desi*	Ca_Desi_Chr02	1521957	(A/C)
25238	CadSNP833	*Desi*	Ca_Desi_Chr02	1557630	(G/T)
25239	CadSNP834	*Desi*	Ca_Desi_Chr02	1565698	(G/A)
25240	CadSNP835	*Desi*	Ca_Desi_Chr02	1565686	(A/G)
25241	CadSNP836	*Desi*	Ca_Desi_Chr02	1565676	(C/T)
25242	CadSNP837	*Desi*	Ca_Desi_Chr02	1621762	(C/A)
25243	CadSNP838	*Desi*	Ca_Desi_Chr02	1846506	(G/T)
25244	CadSNP839	*Desi*	Ca_Desi_Chr02	1857220	(G/A)
25245	CadSNP840	*Desi*	Ca_Desi_Chr02	1857224	(A/G)
25246	CadSNP841	*Desi*	Ca_Desi_Chr02	1861385	(A/G)
25247	CadSNP842	*Desi*	Ca_Desi_Chr02	1861412	(G/A)
25248	CadSNP843	*Desi*	Ca_Desi_Chr02	1861525	(G/A)
25249	CadSNP844	*Desi*	Ca_Desi_Chr02	1861519	(C/T)
25250	CadSNP845	*Desi*	Ca_Desi_Chr02	1868000	(A/G)
25251	CadSNP846	*Desi*	Ca_Desi_Chr02	1883048	(A/G)
25252	CadSNP847	*Desi*	Ca_Desi_Chr02	1883019	(A/G)
25253	CadSNP848	*Desi*	Ca_Desi_Chr02	1894083	(A/T)
25254	CadSNP849	*Desi*	Ca_Desi_Chr02	1954750	(A/C)
25255	CadSNP850	*Desi*	Ca_Desi_Chr02	1958652	(A/C)
25256	CadSNP851	*Desi*	Ca_Desi_Chr02	1959776	(C/T)
25257	CadSNP852	*Desi*	Ca_Desi_Chr02	1996590	(T/C)
25258	CadSNP853	*Desi*	Ca_Desi_Chr02	1996699	(G/A)
25259	CadSNP854	*Desi*	Ca_Desi_Chr02	2100229	(C/T)
25260	CadSNP855	*Desi*	Ca_Desi_Chr02	2308597	(A/G)
25261	CadSNP856	*Desi*	Ca_Desi_Chr02	2308730	(G/A)
25262	CadSNP857	*Desi*	Ca_Desi_Chr02	2326287	(G/C)
25263	CadSNP858	*Desi*	Ca_Desi_Chr02	2344280	(G/A)
25264	CadSNP859	*Desi*	Ca_Desi_Chr02	2344333	(T/C)
25265	CadSNP860	*Desi*	Ca_Desi_Chr02	2361308	(G/A)
25266	CadSNP861	*Desi*	Ca_Desi_Chr02	2361249	(T/C)
25267	CadSNP862	*Desi*	Ca_Desi_Chr02	2363610	(C/G)
25268	CadSNP863	*Desi*	Ca_Desi_Chr02	2379453	(A/G)
25269	CadSNP864	*Desi*	Ca_Desi_Chr02	2406652	(A/G)

S.N.	SNP IDs	Cultivars	Chromosomes/scaffolds	Physical positions (bp)	SNPs
25270	CadSNP865	*Desi*	Ca_Desi_Chr02	2449541	(T/G)
25271	CadSNP866	*Desi*	Ca_Desi_Chr02	2449576	(T/A)
25272	CadSNP867	*Desi*	Ca_Desi_Chr02	2461994	(T/A)
25273	CadSNP868	*Desi*	Ca_Desi_Chr02	2488147	(A/G)
25274	CadSNP869	*Desi*	Ca_Desi_Chr02	2488151	(C/T)
25275	CadSNP870	*Desi*	Ca_Desi_Chr02	2488181	(T/G)
25276	CadSNP871	*Desi*	Ca_Desi_Chr02	2527417	(C/T)
25277	CadSNP872	*Desi*	Ca_Desi_Chr02	2527461	(C/T)
25278	CadSNP873	*Desi*	Ca_Desi_Chr02	2565668	(G/A)
25279	CadSNP874	*Desi*	Ca_Desi_Chr02	2574544	(T/C)
25280	CadSNP875	*Desi*	Ca_Desi_Chr02	2600740	(G/T)
25281	CadSNP876	*Desi*	Ca_Desi_Chr02	2600744	(G/T)
25282	CadSNP877	*Desi*	Ca_Desi_Chr02	2600751	(C/T)
25283	CadSNP878	*Desi*	Ca_Desi_Chr02	2607635	(G/C)
25284	CadSNP879	*Desi*	Ca_Desi_Chr02	2607620	(T/G)
25285	CadSNP880	*Desi*	Ca_Desi_Chr02	2607603	(T/G)
25286	CadSNP881	*Desi*	Ca_Desi_Chr02	2607737	(T/A)
25287	CadSNP882	*Desi*	Ca_Desi_Chr02	2607804	(A/C)
25288	CadSNP883	*Desi*	Ca_Desi_Chr02	2607820	(G/T)
25289	CadSNP884	*Desi*	Ca_Desi_Chr02	2607827	(T/G)
25290	CadSNP885	*Desi*	Ca_Desi_Chr02	2607828	(A/T)
25291	CadSNP886	*Desi*	Ca_Desi_Chr02	2607993	(A/T)
25292	CadSNP887	*Desi*	Ca_Desi_Chr02	2608058	(G/A)
25293	CadSNP888	*Desi*	Ca_Desi_Chr02	2608080	(C/T)
25294	CadSNP889	*Desi*	Ca_Desi_Chr02	2608756	(A/G)
25295	CadSNP890	*Desi*	Ca_Desi_Chr02	2704878	(C/T)
25296	CadSNP891	*Desi*	Ca_Desi_Chr02	2764423	(C/G)
25297	CadSNP892	*Desi*	Ca_Desi_Chr02	2764465	(A/G)
25298	CadSNP893	*Desi*	Ca_Desi_Chr02	2764476	(T/C)
25299	CadSNP894	*Desi*	Ca_Desi_Chr02	2764617	(C/T)
25300	CadSNP895	*Desi*	Ca_Desi_Chr02	2764833	(T/C)
25301	CadSNP896	*Desi*	Ca_Desi_Chr02	2849625	(G/A)
25302	CadSNP897	*Desi*	Ca_Desi_Chr02	2879030	(T/A)
25303	CadSNP898	*Desi*	Ca_Desi_Chr02	2890359	(G/A)
25304	CadSNP899	*Desi*	Ca_Desi_Chr02	2956401	(A/G)
25305	CadSNP900	*Desi*	Ca_Desi_Chr02	3068073	(T/A)
25306	CadSNP901	*Desi*	Ca_Desi_Chr02	3069887	(A/G)
25307	CadSNP902	*Desi*	Ca_Desi_Chr02	3069889	(T/A)

S.N.	SNP IDs	Cultivars	Chromosomes/scaffolds	Physical positions (bp)	SNPs
25308	CadSNP903	*Desi*	Ca_Desi_Chr02	3118713	(T/G)
25309	CadSNP904	*Desi*	Ca_Desi_Chr02	3331580	(G/T)
25310	CadSNP905	*Desi*	Ca_Desi_Chr02	3360033	(A/T)
25311	CadSNP906	*Desi*	Ca_Desi_Chr02	3589210	(C/T)
25312	CadSNP907	*Desi*	Ca_Desi_Chr02	3589227	(G/A)
25313	CadSNP908	*Desi*	Ca_Desi_Chr02	3589397	(G/A)
25314	CadSNP909	*Desi*	Ca_Desi_Chr02	3604220	(C/A)
25315	CadSNP910	*Desi*	Ca_Desi_Chr02	3604390	(G/A)
25316	CadSNP911	*Desi*	Ca_Desi_Chr02	3604373	(A/G)
25317	CadSNP912	*Desi*	Ca_Desi_Chr02	3604348	(G/A)
25318	CadSNP913	*Desi*	Ca_Desi_Chr02	3604347	(T/C)
25319	CadSNP914	*Desi*	Ca_Desi_Chr02	3604341	(G/A)
25320	CadSNP915	*Desi*	Ca_Desi_Chr02	3604339	(A/C)
25321	CadSNP916	*Desi*	Ca_Desi_Chr02	3604329	(G/A)
25322	CadSNP917	*Desi*	Ca_Desi_Chr02	3604402	(A/C)
25323	CadSNP918	*Desi*	Ca_Desi_Chr02	3604419	(T/C)
25324	CadSNP919	*Desi*	Ca_Desi_Chr02	3722112	(T/C)
25325	CadSNP920	*Desi*	Ca_Desi_Chr02	3722164	(T/A)
25326	CadSNP921	*Desi*	Ca_Desi_Chr02	3722146	(A/G)
25327	CadSNP922	*Desi*	Ca_Desi_Chr02	3750227	(C/A)
25328	CadSNP923	*Desi*	Ca_Desi_Chr02	3750343	(T/A)
25329	CadSNP924	*Desi*	Ca_Desi_Chr02	3750312	(A/G)
25330	CadSNP925	*Desi*	Ca_Desi_Chr02	3750295	(T/C)
25331	CadSNP926	*Desi*	Ca_Desi_Chr02	3756279	(C/A)
25332	CadSNP927	*Desi*	Ca_Desi_Chr02	3756269	(A/C)
25333	CadSNP928	*Desi*	Ca_Desi_Chr02	3756677	(T/C)
25334	CadSNP929	*Desi*	Ca_Desi_Chr02	3854174	(C/T)
25335	CadSNP930	*Desi*	Ca_Desi_Chr02	3854173	(A/T)
25336	CadSNP931	*Desi*	Ca_Desi_Chr02	3930203	(C/G)
25337	CadSNP932	*Desi*	Ca_Desi_Chr02	4041350	(C/T)
25338	CadSNP933	*Desi*	Ca_Desi_Chr02	4041319	(A/G)
25339	CadSNP934	*Desi*	Ca_Desi_Chr02	4058389	(G/A)
25340	CadSNP935	*Desi*	Ca_Desi_Chr02	4125467	(G/A)
25341	CadSNP936	*Desi*	Ca_Desi_Chr02	4185142	(A/G)
25342	CadSNP937	*Desi*	Ca_Desi_Chr02	4185428	(T/A)
25343	CadSNP938	*Desi*	Ca_Desi_Chr02	4185422	(T/C)
25344	CadSNP939	*Desi*	Ca_Desi_Chr02	4185408	(A/G)
25345	CadSNP940	*Desi*	Ca_Desi_Chr02	4185394	(A/T)

**S.N.**	**SNP IDs**	**Cultivars**	**Chromosomes/scaffolds**	**Physical positions (bp)**	**SNPs**
25346	CadSNP941	*Desi*	Ca_Desi_Chr02	4190614	(C/T)
25347	CadSNP942	*Desi*	Ca_Desi_Chr02	4190767	(T/A)
25348	CadSNP943	*Desi*	Ca_Desi_Chr02	4212810	(A/G)
25349	CadSNP944	*Desi*	Ca_Desi_Chr02	4238261	(G/A)
25350	CadSNP945	*Desi*	Ca_Desi_Chr02	4527225	(T/A)
25351	CadSNP946	*Desi*	Ca_Desi_Chr02	4527227	(A/G)
25352	CadSNP947	*Desi*	Ca_Desi_Chr02	4527234	(A/G)
25353	CadSNP948	*Desi*	Ca_Desi_Chr02	4528767	(A/T)
25354	CadSNP949	*Desi*	Ca_Desi_Chr02	4552112	(C/T)
25355	CadSNP950	*Desi*	Ca_Desi_Chr02	4727092	(G/C)
25356	CadSNP951	*Desi*	Ca_Desi_Chr02	5116790	(C/T)
25357	CadSNP952	*Desi*	Ca_Desi_Chr02	5116882	(G/A)
25358	CadSNP953	*Desi*	Ca_Desi_Chr02	5168628	(T/A)
25359	CadSNP954	*Desi*	Ca_Desi_Chr02	5168645	(C/A)
25360	CadSNP955	*Desi*	Ca_Desi_Chr02	5213222	(G/A)
25361	CadSNP956	*Desi*	Ca_Desi_Chr02	5223443	(G/A)
25362	CadSNP957	*Desi*	Ca_Desi_Chr02	5400370	(A/T)
25363	CadSNP958	*Desi*	Ca_Desi_Chr02	5400364	(C/T)
25364	CadSNP959	*Desi*	Ca_Desi_Chr02	5448589	(C/A)
25365	CadSNP960	*Desi*	Ca_Desi_Chr02	5462566	(C/G)
25366	CadSNP961	*Desi*	Ca_Desi_Chr02	5466959	(C/T)
25367	CadSNP962	*Desi*	Ca_Desi_Chr02	5466961	(A/G)
25368	CadSNP963	*Desi*	Ca_Desi_Chr02	5466969	(T/A)
25369	CadSNP964	*Desi*	Ca_Desi_Chr02	5466993	(C/T)
25370	CadSNP965	*Desi*	Ca_Desi_Chr02	5467020	(G/A)
25371	CadSNP966	*Desi*	Ca_Desi_Chr02	5472865	(A/G)
25372	CadSNP967	*Desi*	Ca_Desi_Chr02	5472802	(T/C)
25373	CadSNP968	*Desi*	Ca_Desi_Chr02	5503519	(G/A)
25374	CadSNP969	*Desi*	Ca_Desi_Chr02	5503511	(G/T)
25375	CadSNP970	*Desi*	Ca_Desi_Chr02	5503509	(A/G)
25376	CadSNP971	*Desi*	Ca_Desi_Chr02	5503507	(C/A)
25377	CadSNP972	*Desi*	Ca_Desi_Chr02	5503492	(G/A)
25378	CadSNP973	*Desi*	Ca_Desi_Chr02	5503490	(T/G)
25379	CadSNP974	*Desi*	Ca_Desi_Chr02	5503486	(G/A)
25380	CadSNP975	*Desi*	Ca_Desi_Chr02	5503637	(C/G)
25381	CadSNP976	*Desi*	Ca_Desi_Chr02	5503732	(G/C)
25382	CadSNP977	*Desi*	Ca_Desi_Chr02	5503727	(T/G)
25383	CadSNP978	*Desi*	Ca_Desi_Chr02	5513741	(T/C)

S.N.	SNP IDs	Cultivars	Chromosomes/scaffolds	Physical positions (bp)	SNPs
25384	CadSNP979	*Desi*	Ca_Desi_Chr02	5530121	(C/T)
25385	CadSNP980	*Desi*	Ca_Desi_Chr02	5548819	(G/A)
25386	CadSNP981	*Desi*	Ca_Desi_Chr02	5548807	(C/G)
25387	CadSNP982	*Desi*	Ca_Desi_Chr02	5549174	(G/A)
25388	CadSNP983	*Desi*	Ca_Desi_Chr02	5549164	(T/C)
25389	CadSNP984	*Desi*	Ca_Desi_Chr02	5549156	(T/A)
25390	CadSNP985	*Desi*	Ca_Desi_Chr02	5549150	(C/A)
25391	CadSNP986	*Desi*	Ca_Desi_Chr02	5549966	(A/T)
25392	CadSNP987	*Desi*	Ca_Desi_Chr02	5550007	(G/A)
25393	CadSNP988	*Desi*	Ca_Desi_Chr02	5550009	(A/C)
25394	CadSNP989	*Desi*	Ca_Desi_Chr02	5577178	(A/T)
25395	CadSNP990	*Desi*	Ca_Desi_Chr02	5632043	(A/G)
25396	CadSNP991	*Desi*	Ca_Desi_Chr02	5635080	(T/C)
25397	CadSNP992	*Desi*	Ca_Desi_Chr02	5635215	(A/C)
25398	CadSNP993	*Desi*	Ca_Desi_Chr02	5635178	(A/T)
25399	CadSNP994	*Desi*	Ca_Desi_Chr02	5648550	(A/G)
25400	CadSNP995	*Desi*	Ca_Desi_Chr02	5661404	(C/T)
25401	CadSNP996	*Desi*	Ca_Desi_Chr02	5661407	(A/G)
25402	CadSNP997	*Desi*	Ca_Desi_Chr02	5661416	(A/G)
25403	CadSNP998	*Desi*	Ca_Desi_Chr02	5661427	(A/G)
25404	CadSNP999	*Desi*	Ca_Desi_Chr02	5661431	(G/T)
25405	CadSNP1000	*Desi*	Ca_Desi_Chr02	5661464	(A/T)
25406	CadSNP1001	*Desi*	Ca_Desi_Chr02	5661470	(A/T)
25407	CadSNP1002	*Desi*	Ca_Desi_Chr02	5661482	(A/G)
25408	CadSNP1003	*Desi*	Ca_Desi_Chr02	5661485	(G/C)
25409	CadSNP1004	*Desi*	Ca_Desi_Chr02	5661500	(A/G)
25410	CadSNP1005	*Desi*	Ca_Desi_Chr02	5661498	(T/C)
25411	CadSNP1006	*Desi*	Ca_Desi_Chr02	5661494	(T/A)
25412	CadSNP1007	*Desi*	Ca_Desi_Chr02	5694128	(G/T)
25413	CadSNP1008	*Desi*	Ca_Desi_Chr02	5694127	(C/T)
25414	CadSNP1009	*Desi*	Ca_Desi_Chr02	5694118	(T/C)
25415	CadSNP1010	*Desi*	Ca_Desi_Chr02	5698170	(T/A)
25416	CadSNP1011	*Desi*	Ca_Desi_Chr02	5698197	(G/A)
25417	CadSNP1012	*Desi*	Ca_Desi_Chr02	5698994	(G/C)
25418	CadSNP1013	*Desi*	Ca_Desi_Chr02	5698976	(C/T)
25419	CadSNP1014	*Desi*	Ca_Desi_Chr02	5699101	(G/A)
25420	CadSNP1015	*Desi*	Ca_Desi_Chr02	5699314	(A/G)
25421	CadSNP1016	*Desi*	Ca_Desi_Chr02	5699361	(G/A)

S.N.	SNP IDs	Cultivars	Chromosomes/scaffolds	Physical positions (bp)	SNPs
25422	CadSNP1017	*Desi*	Ca_Desi_Chr02	5699391	(T/A)
25423	CadSNP1018	*Desi*	Ca_Desi_Chr02	5699393	(G/A)
25424	CadSNP1019	*Desi*	Ca_Desi_Chr02	5733756	(C/A)
25425	CadSNP1020	*Desi*	Ca_Desi_Chr02	5740309	(C/A)
25426	CadSNP1021	*Desi*	Ca_Desi_Chr02	5745142	(C/T)
25427	CadSNP1022	*Desi*	Ca_Desi_Chr02	5917902	(G/A)
25428	CadSNP1023	*Desi*	Ca_Desi_Chr02	5965393	(G/T)
25429	CadSNP1024	*Desi*	Ca_Desi_Chr02	5965391	(C/T)
25430	CadSNP1025	*Desi*	Ca_Desi_Chr02	5966192	(C/T)
25431	CadSNP1026	*Desi*	Ca_Desi_Chr02	5988875	(T/A)
25432	CadSNP1027	*Desi*	Ca_Desi_Chr02	6010840	(G/A)
25433	CadSNP1028	*Desi*	Ca_Desi_Chr02	6011026	(G/A)
25434	CadSNP1029	*Desi*	Ca_Desi_Chr02	6018033	(C/T)
25435	CadSNP1030	*Desi*	Ca_Desi_Chr02	6019934	(G/T)
25436	CadSNP1031	*Desi*	Ca_Desi_Chr02	6020224	(G/A)
25437	CadSNP1032	*Desi*	Ca_Desi_Chr02	6022635	(G/A)
25438	CadSNP1033	*Desi*	Ca_Desi_Chr02	6022627	(T/G)
25439	CadSNP1034	*Desi*	Ca_Desi_Chr02	6022619	(C/T)
25440	CadSNP1035	*Desi*	Ca_Desi_Chr02	6048386	(T/A)
25441	CadSNP1036	*Desi*	Ca_Desi_Chr02	6053043	(A/T)
25442	CadSNP1037	*Desi*	Ca_Desi_Chr02	6071359	(C/T)
25443	CadSNP1038	*Desi*	Ca_Desi_Chr02	6088304	(C/T)
25444	CadSNP1039	*Desi*	Ca_Desi_Chr02	6089048	(T/C)
25445	CadSNP1040	*Desi*	Ca_Desi_Chr02	6089257	(G/A)
25446	CadSNP1041	*Desi*	Ca_Desi_Chr02	6091962	(C/T)
25447	CadSNP1042	*Desi*	Ca_Desi_Chr02	6091970	(A/C)
25448	CadSNP1043	*Desi*	Ca_Desi_Chr02	6091973	(T/G)
25449	CadSNP1044	*Desi*	Ca_Desi_Chr02	6092094	(A/G)
25450	CadSNP1045	*Desi*	Ca_Desi_Chr02	6092092	(G/A)
25451	CadSNP1046	*Desi*	Ca_Desi_Chr02	6092158	(G/C)
25452	CadSNP1047	*Desi*	Ca_Desi_Chr02	6092265	(A/G)
25453	CadSNP1048	*Desi*	Ca_Desi_Chr02	6092376	(T/C)
25454	CadSNP1049	*Desi*	Ca_Desi_Chr02	6092347	(T/G)
25455	CadSNP1050	*Desi*	Ca_Desi_Chr02	6092444	(C/T)
25456	CadSNP1051	*Desi*	Ca_Desi_Chr02	6094379	(C/G)
25457	CadSNP1052	*Desi*	Ca_Desi_Chr02	6103902	(T/C)
25458	CadSNP1053	*Desi*	Ca_Desi_Chr02	6107446	(A/C)
25459	CadSNP1054	*Desi*	Ca_Desi_Chr02	6107432	(G/T)

S.N.	SNP IDs	Cultivars	Chromosomes/scaffolds	Physical positions (bp)	SNPs
25460	CadSNP1055	*Desi*	Ca_Desi_Chr02	6131587	(T/C)
25461	CadSNP1056	*Desi*	Ca_Desi_Chr02	6135210	(G/A)
25462	CadSNP1057	*Desi*	Ca_Desi_Chr02	6138794	(T/G)
25463	CadSNP1058	*Desi*	Ca_Desi_Chr02	6138861	(C/T)
25464	CadSNP1059	*Desi*	Ca_Desi_Chr02	6138912	(C/T)
25465	CadSNP1060	*Desi*	Ca_Desi_Chr02	6138975	(A/T)
25466	CadSNP1061	*Desi*	Ca_Desi_Chr02	6138976	(G/T)
25467	CadSNP1062	*Desi*	Ca_Desi_Chr02	6139045	(A/G)
25468	CadSNP1063	*Desi*	Ca_Desi_Chr02	6140423	(G/A)
25469	CadSNP1064	*Desi*	Ca_Desi_Chr02	6140387	(T/C)
25470	CadSNP1065	*Desi*	Ca_Desi_Chr02	6145109	(C/T)
25471	CadSNP1066	*Desi*	Ca_Desi_Chr02	6145149	(T/A)
25472	CadSNP1067	*Desi*	Ca_Desi_Chr02	6145148	(C/A)
25473	CadSNP1068	*Desi*	Ca_Desi_Chr02	6145235	(C/T)
25474	CadSNP1069	*Desi*	Ca_Desi_Chr02	6145253	(G/A)
25475	CadSNP1070	*Desi*	Ca_Desi_Chr02	6145263	(G/A)
25476	CadSNP1071	*Desi*	Ca_Desi_Chr02	6179369	(A/C)
25477	CadSNP1072	*Desi*	Ca_Desi_Chr02	6243184	(C/T)
25478	CadSNP1073	*Desi*	Ca_Desi_Chr02	6243219	(T/G)
25479	CadSNP1074	*Desi*	Ca_Desi_Chr02	6249860	(G/A)
25480	CadSNP1075	*Desi*	Ca_Desi_Chr02	6249876	(C/T)
25481	CadSNP1076	*Desi*	Ca_Desi_Chr02	6249888	(C/A)
25482	CadSNP1077	*Desi*	Ca_Desi_Chr02	6249889	(A/C)
25483	CadSNP1078	*Desi*	Ca_Desi_Chr02	6249975	(T/C)
25484	CadSNP1079	*Desi*	Ca_Desi_Chr02	6255732	(T/C)
25485	CadSNP1080	*Desi*	Ca_Desi_Chr02	6265666	(A/G)
25486	CadSNP1081	*Desi*	Ca_Desi_Chr02	6275717	(C/T)
25487	CadSNP1082	*Desi*	Ca_Desi_Chr02	6436332	(T/A)
25488	CadSNP1083	*Desi*	Ca_Desi_Chr02	6436365	(A/C)
25489	CadSNP1084	*Desi*	Ca_Desi_Chr02	6436390	(G/T)
25490	CadSNP1085	*Desi*	Ca_Desi_Chr02	6436396	(G/T)
25491	CadSNP1086	*Desi*	Ca_Desi_Chr02	6437062	(A/T)
25492	CadSNP1087	*Desi*	Ca_Desi_Chr02	6437145	(T/C)
25493	CadSNP1088	*Desi*	Ca_Desi_Chr02	6532962	(C/T)
25494	CadSNP1089	*Desi*	Ca_Desi_Chr02	6532976	(G/T)
25495	CadSNP1090	*Desi*	Ca_Desi_Chr02	6535217	(C/T)
25496	CadSNP1091	*Desi*	Ca_Desi_Chr02	6589542	(A/G)
25497	CadSNP1092	*Desi*	Ca_Desi_Chr02	6589551	(T/C)

S.N.	SNP IDs	Cultivars	Chromosomes/scaffolds	Physical positions (bp)	SNPs
25498	CadSNP1093	*Desi*	Ca_Desi_Chr02	6589573	(G/A)
25499	CadSNP1094	*Desi*	Ca_Desi_Chr02	6601251	(T/A)
25500	CadSNP1095	*Desi*	Ca_Desi_Chr02	6608684	(T/C)
25501	CadSNP1096	*Desi*	Ca_Desi_Chr02	6608737	(G/A)
25502	CadSNP1097	*Desi*	Ca_Desi_Chr02	6609027	(G/T)
25503	CadSNP1098	*Desi*	Ca_Desi_Chr02	6609053	(A/T)
25504	CadSNP1099	*Desi*	Ca_Desi_Chr02	6614650	(G/A)
25505	CadSNP1100	*Desi*	Ca_Desi_Chr02	6614735	(T/G)
25506	CadSNP1101	*Desi*	Ca_Desi_Chr02	6614685	(G/A)
25507	CadSNP1102	*Desi*	Ca_Desi_Chr02	6614760	(G/C)
25508	CadSNP1103	*Desi*	Ca_Desi_Chr02	6622281	(T/A)
25509	CadSNP1104	*Desi*	Ca_Desi_Chr02	6622600	(A/G)
25510	CadSNP1105	*Desi*	Ca_Desi_Chr02	6624856	(T/C)
25511	CadSNP1106	*Desi*	Ca_Desi_Chr02	6624907	(C/G)
25512	CadSNP1107	*Desi*	Ca_Desi_Chr02	6651178	(A/T)
25513	CadSNP1108	*Desi*	Ca_Desi_Chr02	6653902	(C/T)
25514	CadSNP1109	*Desi*	Ca_Desi_Chr02	6653887	(A/G)
25515	CadSNP1110	*Desi*	Ca_Desi_Chr02	6653879	(G/T)
25516	CadSNP1111	*Desi*	Ca_Desi_Chr02	6669823	(C/G)
25517	CadSNP1112	*Desi*	Ca_Desi_Chr02	6671859	(A/G)
25518	CadSNP1113	*Desi*	Ca_Desi_Chr02	6701392	(G/A)
25519	CadSNP1114	*Desi*	Ca_Desi_Chr02	6701394	(G/A)
25520	CadSNP1115	*Desi*	Ca_Desi_Chr02	6711702	(C/T)
25521	CadSNP1116	*Desi*	Ca_Desi_Chr02	6711747	(C/T)
25522	CadSNP1117	*Desi*	Ca_Desi_Chr02	6711829	(T/G)
25523	CadSNP1118	*Desi*	Ca_Desi_Chr02	6713000	(T/A)
25524	CadSNP1119	*Desi*	Ca_Desi_Chr02	6908990	(A/G)
25525	CadSNP1120	*Desi*	Ca_Desi_Chr02	6968294	(T/G)
25526	CadSNP1121	*Desi*	Ca_Desi_Chr02	6968382	(C/G)
25527	CadSNP1122	*Desi*	Ca_Desi_Chr02	6992535	(A/G)
25528	CadSNP1123	*Desi*	Ca_Desi_Chr02	7059796	(C/T)
25529	CadSNP1124	*Desi*	Ca_Desi_Chr02	7059783	(G/C)
25530	CadSNP1125	*Desi*	Ca_Desi_Chr02	7062006	(A/G)
25531	CadSNP1126	*Desi*	Ca_Desi_Chr02	7062059	(G/A)
25532	CadSNP1127	*Desi*	Ca_Desi_Chr02	7082429	(A/G)
25533	CadSNP1128	*Desi*	Ca_Desi_Chr02	7082437	(T/C)
25534	CadSNP1129	*Desi*	Ca_Desi_Chr02	7082452	(A/C)
25535	CadSNP1130	*Desi*	Ca_Desi_Chr02	7082486	(A/C)

S.N.	SNP IDs	Cultivars	Chromosomes/scaffolds	Physical positions (bp)	SNPs
25536	CadSNP1131	*Desi*	Ca_Desi_Chr02	7082872	(A/C)
25537	CadSNP1132	*Desi*	Ca_Desi_Chr02	7082948	(C/G)
25538	CadSNP1133	*Desi*	Ca_Desi_Chr02	7085006	(A/G)
25539	CadSNP1134	*Desi*	Ca_Desi_Chr02	7084987	(T/C)
25540	CadSNP1135	*Desi*	Ca_Desi_Chr02	7084976	(A/C)
25541	CadSNP1136	*Desi*	Ca_Desi_Chr02	7113287	(G/T)
25542	CadSNP1137	*Desi*	Ca_Desi_Chr02	7113358	(G/T)
25543	CadSNP1138	*Desi*	Ca_Desi_Chr02	7222282	(T/A)
25544	CadSNP1139	*Desi*	Ca_Desi_Chr02	7222474	(T/G)
25545	CadSNP1140	*Desi*	Ca_Desi_Chr02	7222511	(T/A)
25546	CadSNP1141	*Desi*	Ca_Desi_Chr02	7241676	(G/A)
25547	CadSNP1142	*Desi*	Ca_Desi_Chr02	7241824	(G/A)
25548	CadSNP1143	*Desi*	Ca_Desi_Chr02	7242108	(A/G)
25549	CadSNP1144	*Desi*	Ca_Desi_Chr02	7242124	(G/A)
25550	CadSNP1145	*Desi*	Ca_Desi_Chr02	7316884	(A/T)
25551	CadSNP1146	*Desi*	Ca_Desi_Chr02	7317863	(G/A)
25552	CadSNP1147	*Desi*	Ca_Desi_Chr02	7317845	(A/G)
25553	CadSNP1148	*Desi*	Ca_Desi_Chr02	7327612	(C/T)
25554	CadSNP1149	*Desi*	Ca_Desi_Chr02	7327751	(T/C)
25555	CadSNP1150	*Desi*	Ca_Desi_Chr02	7353195	(G/A)
25556	CadSNP1151	*Desi*	Ca_Desi_Chr02	7358767	(C/A)
25557	CadSNP1152	*Desi*	Ca_Desi_Chr02	7389166	(G/A)
25558	CadSNP1153	*Desi*	Ca_Desi_Chr02	7397445	(T/A)
25559	CadSNP1154	*Desi*	Ca_Desi_Chr02	7409824	(C/T)
25560	CadSNP1155	*Desi*	Ca_Desi_Chr02	7419315	(A/G)
25561	CadSNP1156	*Desi*	Ca_Desi_Chr02	7438626	(G/A)
25562	CadSNP1157	*Desi*	Ca_Desi_Chr02	7457010	(C/T)
25563	CadSNP1158	*Desi*	Ca_Desi_Chr02	7458342	(G/A)
25564	CadSNP1159	*Desi*	Ca_Desi_Chr02	7459382	(G/A)
25565	CadSNP1160	*Desi*	Ca_Desi_Chr02	7462051	(A/C)
25566	CadSNP1161	*Desi*	Ca_Desi_Chr02	7471326	(T/C)
25567	CadSNP1162	*Desi*	Ca_Desi_Chr02	7471357	(T/C)
25568	CadSNP1163	*Desi*	Ca_Desi_Chr02	7585941	(G/A)
25569	CadSNP1164	*Desi*	Ca_Desi_Chr02	7610221	(T/C)
25570	CadSNP1165	*Desi*	Ca_Desi_Chr02	7610503	(T/C)
25571	CadSNP1166	*Desi*	Ca_Desi_Chr02	7630044	(G/A)
25572	CadSNP1167	*Desi*	Ca_Desi_Chr02	7659346	(C/T)
25573	CadSNP1168	*Desi*	Ca_Desi_Chr02	7659312	(G/T)

S.N.	SNP IDs	Cultivars	Chromosomes/scaffolds	Physical positions (bp)	SNPs
25574	CadSNP1169	*Desi*	Ca_Desi_Chr02	7659401	(C/T)
25575	CadSNP1170	*Desi*	Ca_Desi_Chr02	7659625	(T/C)
25576	CadSNP1171	*Desi*	Ca_Desi_Chr02	7675697	(A/G)
25577	CadSNP1172	*Desi*	Ca_Desi_Chr02	7697803	(G/C)
25578	CadSNP1173	*Desi*	Ca_Desi_Chr02	7708782	(G/A)
25579	CadSNP1174	*Desi*	Ca_Desi_Chr02	7708761	(C/T)
25580	CadSNP1175	*Desi*	Ca_Desi_Chr02	7708760	(A/C)
25581	CadSNP1176	*Desi*	Ca_Desi_Chr02	7708749	(G/A)
25582	CadSNP1177	*Desi*	Ca_Desi_Chr02	7708765	(A/C)
25583	CadSNP1178	*Desi*	Ca_Desi_Chr02	7709532	(C/T)
25584	CadSNP1179	*Desi*	Ca_Desi_Chr02	7709745	(A/G)
25585	CadSNP1180	*Desi*	Ca_Desi_Chr02	7709743	(A/G)
25586	CadSNP1181	*Desi*	Ca_Desi_Chr02	7728470	(C/G)
25587	CadSNP1182	*Desi*	Ca_Desi_Chr02	7728474	(A/G)
25588	CadSNP1183	*Desi*	Ca_Desi_Chr02	7813999	(C/A)
25589	CadSNP1184	*Desi*	Ca_Desi_Chr02	7813998	(T/A)
25590	CadSNP1185	*Desi*	Ca_Desi_Chr02	7869084	(A/C)
25591	CadSNP1186	*Desi*	Ca_Desi_Chr02	7875419	(A/G)
25592	CadSNP1187	*Desi*	Ca_Desi_Chr02	7894116	(C/T)
25593	CadSNP1188	*Desi*	Ca_Desi_Chr02	7897881	(C/A)
25594	CadSNP1189	*Desi*	Ca_Desi_Chr02	7944911	(T/C)
25595	CadSNP1190	*Desi*	Ca_Desi_Chr02	7956738	(T/C)
25596	CadSNP1191	*Desi*	Ca_Desi_Chr02	7958321	(A/C)
25597	CadSNP1192	*Desi*	Ca_Desi_Chr02	7958494	(C/T)
25598	CadSNP1193	*Desi*	Ca_Desi_Chr02	7958522	(A/G)
25599	CadSNP1194	*Desi*	Ca_Desi_Chr02	7958529	(T/A)
25600	CadSNP1195	*Desi*	Ca_Desi_Chr02	7960966	(A/T)
25601	CadSNP1196	*Desi*	Ca_Desi_Chr02	8013247	(C/T)
25602	CadSNP1197	*Desi*	Ca_Desi_Chr02	8037211	(C/A)
25603	CadSNP1198	*Desi*	Ca_Desi_Chr02	8065988	(C/A)
25604	CadSNP1199	*Desi*	Ca_Desi_Chr02	8073314	(G/A)
25605	CadSNP1200	*Desi*	Ca_Desi_Chr02	8113745	(G/T)
25606	CadSNP1201	*Desi*	Ca_Desi_Chr02	8113738	(C/T)
25607	CadSNP1202	*Desi*	Ca_Desi_Chr02	8113723	(C/T)
25608	CadSNP1203	*Desi*	Ca_Desi_Chr02	8113708	(T/G)
25609	CadSNP1204	*Desi*	Ca_Desi_Chr02	8113696	(C/G)
25610	CadSNP1205	*Desi*	Ca_Desi_Chr02	8149881	(T/G)
25611	CadSNP1206	*Desi*	Ca_Desi_Chr02	8160645	(C/A)

S.N.	SNP IDs	Cultivars	Chromosomes/scaffolds	Physical positions (bp)	SNPs
25612	CadSNP1207	*Desi*	Ca_Desi_Chr02	8160750	(G/A)
25613	CadSNP1208	*Desi*	Ca_Desi_Chr02	8160737	(A/C)
25614	CadSNP1209	*Desi*	Ca_Desi_Chr02	8160733	(C/T)
25615	CadSNP1210	*Desi*	Ca_Desi_Chr02	8167177	(A/C)
25616	CadSNP1211	*Desi*	Ca_Desi_Chr02	8167702	(T/C)
25617	CadSNP1212	*Desi*	Ca_Desi_Chr02	8167708	(C/T)
25618	CadSNP1213	*Desi*	Ca_Desi_Chr02	8167709	(C/T)
25619	CadSNP1214	*Desi*	Ca_Desi_Chr02	8167714	(A/G)
25620	CadSNP1215	*Desi*	Ca_Desi_Chr02	8167719	(T/C)
25621	CadSNP1216	*Desi*	Ca_Desi_Chr02	8167720	(G/A)
25622	CadSNP1217	*Desi*	Ca_Desi_Chr02	8167721	(A/C)
25623	CadSNP1218	*Desi*	Ca_Desi_Chr02	8167724	(C/T)
25624	CadSNP1219	*Desi*	Ca_Desi_Chr02	8167729	(A/G)
25625	CadSNP1220	*Desi*	Ca_Desi_Chr02	8167735	(G/A)
25626	CadSNP1221	*Desi*	Ca_Desi_Chr02	8167740	(C/A)
25627	CadSNP1222	*Desi*	Ca_Desi_Chr02	8167744	(G/A)
25628	CadSNP1223	*Desi*	Ca_Desi_Chr02	8167753	(G/A)
25629	CadSNP1224	*Desi*	Ca_Desi_Chr02	8167754	(A/T)
25630	CadSNP1225	*Desi*	Ca_Desi_Chr02	8167759	(C/G)
25631	CadSNP1226	*Desi*	Ca_Desi_Chr02	8167760	(C/G)
25632	CadSNP1227	*Desi*	Ca_Desi_Chr02	8167764	(G/A)
25633	CadSNP1228	*Desi*	Ca_Desi_Chr02	8174448	(G/A)
25634	CadSNP1229	*Desi*	Ca_Desi_Chr02	8174458	(C/T)
25635	CadSNP1230	*Desi*	Ca_Desi_Chr02	8234099	(A/C)
25636	CadSNP1231	*Desi*	Ca_Desi_Chr02	8294583	(T/C)
25637	CadSNP1232	*Desi*	Ca_Desi_Chr02	8294721	(C/A)
25638	CadSNP1233	*Desi*	Ca_Desi_Chr02	8294715	(C/T)
25639	CadSNP1234	*Desi*	Ca_Desi_Chr02	8294709	(C/T)
25640	CadSNP1235	*Desi*	Ca_Desi_Chr02	8294706	(C/T)
25641	CadSNP1236	*Desi*	Ca_Desi_Chr02	8303613	(G/A)
25642	CadSNP1237	*Desi*	Ca_Desi_Chr02	8322723	(A/G)
25643	CadSNP1238	*Desi*	Ca_Desi_Chr02	8322740	(G/A)
25644	CadSNP1239	*Desi*	Ca_Desi_Chr02	8322763	(A/G)
25645	CadSNP1240	*Desi*	Ca_Desi_Chr02	8397884	(A/G)
25646	CadSNP1241	*Desi*	Ca_Desi_Chr02	8397955	(G/C)
25647	CadSNP1242	*Desi*	Ca_Desi_Chr02	8528763	(G/A)
25648	CadSNP1243	*Desi*	Ca_Desi_Chr02	8530649	(C/T)
25649	CadSNP1244	*Desi*	Ca_Desi_Chr02	9840097	(G/A)

S.N.	SNP IDs	Cultivars	Chromosomes/scaffolds	Physical positions (bp)	SNPs
25650	CadSNP1245	*Desi*	Ca_Desi_Chr02	9840141	(C/T)
25651	CadSNP1246	*Desi*	Ca_Desi_Chr02	9845051	(A/C)
25652	CadSNP1247	*Desi*	Ca_Desi_Chr02	9861790	(A/T)
25653	CadSNP1248	*Desi*	Ca_Desi_Chr02	9861771	(A/C)
25654	CadSNP1249	*Desi*	Ca_Desi_Chr02	9864788	(G/T)
25655	CadSNP1250	*Desi*	Ca_Desi_Chr02	9873103	(A/C)
25656	CadSNP1251	*Desi*	Ca_Desi_Chr02	9873154	(C/T)
25657	CadSNP1252	*Desi*	Ca_Desi_Chr02	9886040	(C/T)
25658	CadSNP1253	*Desi*	Ca_Desi_Chr02	10091173	(T/C)
25659	CadSNP1254	*Desi*	Ca_Desi_Chr02	10091208	(C/T)
25660	CadSNP1255	*Desi*	Ca_Desi_Chr02	10091494	(T/C)
25661	CadSNP1256	*Desi*	Ca_Desi_Chr02	10091528	(A/G)
25662	CadSNP1257	*Desi*	Ca_Desi_Chr02	10091558	(A/G)
25663	CadSNP1258	*Desi*	Ca_Desi_Chr02	10091566	(C/A)
25664	CadSNP1259	*Desi*	Ca_Desi_Chr02	10208337	(T/G)
25665	CadSNP1260	*Desi*	Ca_Desi_Chr02	10286615	(C/G)
25666	CadSNP1261	*Desi*	Ca_Desi_Chr02	10436482	(C/T)
25667	CadSNP1262	*Desi*	Ca_Desi_Chr02	10436528	(T/C)
25668	CadSNP1263	*Desi*	Ca_Desi_Chr02	10436644	(G/T)
25669	CadSNP1264	*Desi*	Ca_Desi_Chr02	10439843	(G/A)
25670	CadSNP1265	*Desi*	Ca_Desi_Chr02	10864591	(G/A)
25671	CadSNP1266	*Desi*	Ca_Desi_Chr02	10864593	(A/T)
25672	CadSNP1267	*Desi*	Ca_Desi_Chr02	10864609	(G/A)
25673	CadSNP1268	*Desi*	Ca_Desi_Chr02	10864621	(T/A)
25674	CadSNP1269	*Desi*	Ca_Desi_Chr02	10864624	(C/T)
25675	CadSNP1270	*Desi*	Ca_Desi_Chr02	10864631	(C/T)
25676	CadSNP1271	*Desi*	Ca_Desi_Chr02	10864664	(A/G)
25677	CadSNP1272	*Desi*	Ca_Desi_Chr02	10864728	(C/A)
25678	CadSNP1273	*Desi*	Ca_Desi_Chr02	10864722	(T/C)
25679	CadSNP1274	*Desi*	Ca_Desi_Chr02	10864699	(G/A)
25680	CadSNP1275	*Desi*	Ca_Desi_Chr02	10864698	(G/A)
25681	CadSNP1276	*Desi*	Ca_Desi_Chr02	11110650	(A/T)
25682	CadSNP1277	*Desi*	Ca_Desi_Chr02	11110651	(T/C)
25683	CadSNP1278	*Desi*	Ca_Desi_Chr02	11170269	(C/T)
25684	CadSNP1279	*Desi*	Ca_Desi_Chr02	11268822	(C/T)
25685	CadSNP1280	*Desi*	Ca_Desi_Chr02	11276069	(G/A)
25686	CadSNP1281	*Desi*	Ca_Desi_Chr02	11393742	(A/G)
25687	CadSNP1282	*Desi*	Ca_Desi_Chr02	11393800	(C/T)

S.N.	SNP IDs	Cultivars	Chromosomes/scaffolds	Physical positions (bp)	SNPs
25688	CadSNP1283	*Desi*	Ca_Desi_Chr02	11393824	(C/T)
25689	CadSNP1284	*Desi*	Ca_Desi_Chr02	11393816	(G/A)
25690	CadSNP1285	*Desi*	Ca_Desi_Chr02	11393862	(C/G)
25691	CadSNP1286	*Desi*	Ca_Desi_Chr02	11394075	(A/G)
25692	CadSNP1287	*Desi*	Ca_Desi_Chr02	11394066	(G/A)
25693	CadSNP1288	*Desi*	Ca_Desi_Chr02	11394036	(T/C)
25694	CadSNP1289	*Desi*	Ca_Desi_Chr02	11394024	(A/G)
25695	CadSNP1290	*Desi*	Ca_Desi_Chr02	11394153	(T/C)
25696	CadSNP1291	*Desi*	Ca_Desi_Chr02	11394247	(G/A)
25697	CadSNP1292	*Desi*	Ca_Desi_Chr02	11403900	(C/A)
25698	CadSNP1293	*Desi*	Ca_Desi_Chr02	11404024	(A/G)
25699	CadSNP1294	*Desi*	Ca_Desi_Chr02	11403986	(C/T)
25700	CadSNP1295	*Desi*	Ca_Desi_Chr02	11405477	(C/T)
25701	CadSNP1296	*Desi*	Ca_Desi_Chr02	11422984	(G/A)
25702	CadSNP1297	*Desi*	Ca_Desi_Chr02	11423000	(C/A)
25703	CadSNP1298	*Desi*	Ca_Desi_Chr02	11423014	(G/A)
25704	CadSNP1299	*Desi*	Ca_Desi_Chr02	11423022	(T/C)
25705	CadSNP1300	*Desi*	Ca_Desi_Chr02	11423023	(C/T)
25706	CadSNP1301	*Desi*	Ca_Desi_Chr02	11423041	(T/G)
25707	CadSNP1302	*Desi*	Ca_Desi_Chr02	11423053	(T/C)
25708	CadSNP1303	*Desi*	Ca_Desi_Chr02	11423165	(A/T)
25709	CadSNP1304	*Desi*	Ca_Desi_Chr02	11423145	(A/G)
25710	CadSNP1305	*Desi*	Ca_Desi_Chr02	11636237	(T/A)
25711	CadSNP1306	*Desi*	Ca_Desi_Chr02	11636243	(C/G)
25712	CadSNP1307	*Desi*	Ca_Desi_Chr02	11636275	(C/T)
25713	CadSNP1308	*Desi*	Ca_Desi_Chr02	11636378	(A/C)
25714	CadSNP1309	*Desi*	Ca_Desi_Chr02	11636495	(T/C)
25715	CadSNP1310	*Desi*	Ca_Desi_Chr02	11957822	(A/G)
25716	CadSNP1311	*Desi*	Ca_Desi_Chr02	11957812	(C/T)
25717	CadSNP1312	*Desi*	Ca_Desi_Chr02	11957805	(C/A)
25718	CadSNP1313	*Desi*	Ca_Desi_Chr02	11957800	(T/C)
25719	CadSNP1314	*Desi*	Ca_Desi_Chr02	11957789	(G/T)
25720	CadSNP1315	*Desi*	Ca_Desi_Chr02	11957784	(T/C)
25721	CadSNP1316	*Desi*	Ca_Desi_Chr02	11984016	(C/T)
25722	CadSNP1317	*Desi*	Ca_Desi_Chr02	11985665	(A/C)
25723	CadSNP1318	*Desi*	Ca_Desi_Chr02	11992316	(A/G)
25724	CadSNP1319	*Desi*	Ca_Desi_Chr02	12071159	(T/C)
25725	CadSNP1320	*Desi*	Ca_Desi_Chr02	12071213	(G/A)

S.N.	SNP IDs	Cultivars	Chromosomes/scaffolds	Physical positions (bp)	SNPs
25726	CadSNP1321	*Desi*	Ca_Desi_Chr02	12072110	(A/C)
25727	CadSNP1322	*Desi*	Ca_Desi_Chr02	12357679	(A/G)
25728	CadSNP1323	*Desi*	Ca_Desi_Chr02	12462629	(C/T)
25729	CadSNP1324	*Desi*	Ca_Desi_Chr02	12462654	(G/T)
25730	CadSNP1325	*Desi*	Ca_Desi_Chr02	12490110	(G/C)
25731	CadSNP1326	*Desi*	Ca_Desi_Chr02	12490080	(A/G)
25732	CadSNP1327	*Desi*	Ca_Desi_Chr02	12490043	(G/A)
25733	CadSNP1328	*Desi*	Ca_Desi_Chr02	12518521	(G/T)
25734	CadSNP1329	*Desi*	Ca_Desi_Chr02	12560789	(T/G)
25735	CadSNP1330	*Desi*	Ca_Desi_Chr02	12726887	(A/G)
25736	CadSNP1331	*Desi*	Ca_Desi_Chr02	12729502	(C/T)
25737	CadSNP1332	*Desi*	Ca_Desi_Chr02	12737222	(G/T)
25738	CadSNP1333	*Desi*	Ca_Desi_Chr02	12760068	(C/T)
25739	CadSNP1334	*Desi*	Ca_Desi_Chr02	12797455	(A/T)
25740	CadSNP1335	*Desi*	Ca_Desi_Chr02	12802226	(C/A)
25741	CadSNP1336	*Desi*	Ca_Desi_Chr02	12802223	(T/C)
25742	CadSNP1337	*Desi*	Ca_Desi_Chr02	12802202	(G/T)
25743	CadSNP1338	*Desi*	Ca_Desi_Chr02	12827722	(C/T)
25744	CadSNP1339	*Desi*	Ca_Desi_Chr02	12827705	(A/G)
25745	CadSNP1340	*Desi*	Ca_Desi_Chr02	12830428	(A/C)
25746	CadSNP1341	*Desi*	Ca_Desi_Chr02	12985136	(G/A)
25747	CadSNP1342	*Desi*	Ca_Desi_Chr02	13036994	(G/C)
25748	CadSNP1343	*Desi*	Ca_Desi_Chr02	13036951	(A/C)
25749	CadSNP1344	*Desi*	Ca_Desi_Chr02	13039200	(T/C)
25750	CadSNP1345	*Desi*	Ca_Desi_Chr02	13039869	(C/G)
25751	CadSNP1346	*Desi*	Ca_Desi_Chr02	13040155	(C/T)
25752	CadSNP1347	*Desi*	Ca_Desi_Chr02	13100484	(A/C)
25753	CadSNP1348	*Desi*	Ca_Desi_Chr02	13122853	(T/G)
25754	CadSNP1349	*Desi*	Ca_Desi_Chr02	13127113	(T/G)
25755	CadSNP1350	*Desi*	Ca_Desi_Chr02	13147204	(A/T)
25756	CadSNP1351	*Desi*	Ca_Desi_Chr02	13162836	(T/C)
25757	CadSNP1352	*Desi*	Ca_Desi_Chr02	13162848	(G/C)
25758	CadSNP1353	*Desi*	Ca_Desi_Chr02	13178463	(T/C)
25759	CadSNP1354	*Desi*	Ca_Desi_Chr02	13260498	(G/A)
25760	CadSNP1355	*Desi*	Ca_Desi_Chr02	13331702	(A/G)
25761	CadSNP1356	*Desi*	Ca_Desi_Chr02	13379964	(A/T)
25762	CadSNP1357	*Desi*	Ca_Desi_Chr02	13421370	(C/T)
25763	CadSNP1358	*Desi*	Ca_Desi_Chr02	13572362	(G/A)

S.N.	SNP IDs	Cultivars	Chromosomes/scaffolds	Physical positions (bp)	SNPs
25764	CadSNP1359	*Desi*	Ca_Desi_Chr02	13840468	(T/G)
25765	CadSNP1360	*Desi*	Ca_Desi_Chr02	13840749	(T/A)
25766	CadSNP1361	*Desi*	Ca_Desi_Chr02	13900392	(A/G)
25767	CadSNP1362	*Desi*	Ca_Desi_Chr02	13924598	(A/C)
25768	CadSNP1363	*Desi*	Ca_Desi_Chr02	13924579	(A/T)
25769	CadSNP1364	*Desi*	Ca_Desi_Chr02	14061466	(T/C)
25770	CadSNP1365	*Desi*	Ca_Desi_Chr02	14064995	(G/T)
25771	CadSNP1366	*Desi*	Ca_Desi_Chr02	14163542	(A/C)
25772	CadSNP1367	*Desi*	Ca_Desi_Chr02	14163647	(C/T)
25773	CadSNP1368	*Desi*	Ca_Desi_Chr02	14209016	(A/G)
25774	CadSNP1369	*Desi*	Ca_Desi_Chr02	14215276	(G/A)
25775	CadSNP1370	*Desi*	Ca_Desi_Chr02	14215293	(G/C)
25776	CadSNP1371	*Desi*	Ca_Desi_Chr02	14215344	(A/T)
25777	CadSNP1372	*Desi*	Ca_Desi_Chr02	14215466	(T/C)
25778	CadSNP1373	*Desi*	Ca_Desi_Chr02	14215453	(A/T)
25779	CadSNP1374	*Desi*	Ca_Desi_Chr02	14453533	(T/C)
25780	CadSNP1375	*Desi*	Ca_Desi_Chr02	14453917	(T/C)
25781	CadSNP1376	*Desi*	Ca_Desi_Chr02	14463390	(T/A)
25782	CadSNP1377	*Desi*	Ca_Desi_Chr02	14474102	(C/T)
25783	CadSNP1378	*Desi*	Ca_Desi_Chr02	14474157	(T/A)
25784	CadSNP1379	*Desi*	Ca_Desi_Chr02	14474167	(A/T)
25785	CadSNP1380	*Desi*	Ca_Desi_Chr02	14492206	(T/C)
25786	CadSNP1381	*Desi*	Ca_Desi_Chr02	14492207	(A/G)
25787	CadSNP1382	*Desi*	Ca_Desi_Chr02	14492297	(T/C)
25788	CadSNP1383	*Desi*	Ca_Desi_Chr02	14493784	(T/A)
25789	CadSNP1384	*Desi*	Ca_Desi_Chr02	14510462	(G/T)
25790	CadSNP1385	*Desi*	Ca_Desi_Chr02	14510458	(C/A)
25791	CadSNP1386	*Desi*	Ca_Desi_Chr02	14577347	(G/A)
25792	CadSNP1387	*Desi*	Ca_Desi_Chr02	14577488	(C/T)
25793	CadSNP1388	*Desi*	Ca_Desi_Chr02	14729007	(T/A)
25794	CadSNP1389	*Desi*	Ca_Desi_Chr02	14765296	(T/C)
25795	CadSNP1390	*Desi*	Ca_Desi_Chr02	14766612	(T/G)
25796	CadSNP1391	*Desi*	Ca_Desi_Chr02	14766747	(G/A)
25797	CadSNP1392	*Desi*	Ca_Desi_Chr02	14766780	(C/T)
25798	CadSNP1393	*Desi*	Ca_Desi_Chr02	14784192	(C/T)
25799	CadSNP1394	*Desi*	Ca_Desi_Chr02	14784195	(A/G)
25800	CadSNP1395	*Desi*	Ca_Desi_Chr02	14784208	(G/C)
25801	CadSNP1396	*Desi*	Ca_Desi_Chr02	14784243	(A/G)

S.N.	SNP IDs	Cultivars	Chromosomes/scaffolds	Physical positions (bp)	SNPs
25802	CadSNP1397	*Desi*	Ca_Desi_Chr02	14784247	(G/T)
25803	CadSNP1398	*Desi*	Ca_Desi_Chr02	14811203	(A/G)
25804	CadSNP1399	*Desi*	Ca_Desi_Chr02	14861116	(A/C)
25805	CadSNP1400	*Desi*	Ca_Desi_Chr02	14861159	(T/G)
25806	CadSNP1401	*Desi*	Ca_Desi_Chr02	14978631	(T/C)
25807	CadSNP1402	*Desi*	Ca_Desi_Chr02	14978653	(C/T)
25808	CadSNP1403	*Desi*	Ca_Desi_Chr02	15006255	(C/A)
25809	CadSNP1404	*Desi*	Ca_Desi_Chr02	15006256	(G/T)
25810	CadSNP1405	*Desi*	Ca_Desi_Chr02	15014922	(C/T)
25811	CadSNP1406	*Desi*	Ca_Desi_Chr02	15222856	(C/T)
25812	CadSNP1407	*Desi*	Ca_Desi_Chr02	15279307	(A/G)
25813	CadSNP1408	*Desi*	Ca_Desi_Chr02	15284031	(T/A)
25814	CadSNP1409	*Desi*	Ca_Desi_Chr02	15313773	(A/G)
25815	CadSNP1410	*Desi*	Ca_Desi_Chr02	15318170	(C/T)
25816	CadSNP1411	*Desi*	Ca_Desi_Chr02	15318202	(A/G)
25817	CadSNP1412	*Desi*	Ca_Desi_Chr02	15391794	(T/C)
25818	CadSNP1413	*Desi*	Ca_Desi_Chr02	15392614	(C/T)
25819	CadSNP1414	*Desi*	Ca_Desi_Chr02	15392855	(T/G)
25820	CadSNP1415	*Desi*	Ca_Desi_Chr02	15427877	(G/A)
25821	CadSNP1416	*Desi*	Ca_Desi_Chr02	15438733	(A/C)
25822	CadSNP1417	*Desi*	Ca_Desi_Chr02	15470642	(C/T)
25823	CadSNP1418	*Desi*	Ca_Desi_Chr02	15488586	(G/A)
25824	CadSNP1419	*Desi*	Ca_Desi_Chr02	15488553	(C/T)
25825	CadSNP1420	*Desi*	Ca_Desi_Chr02	15502293	(T/C)
25826	CadSNP1421	*Desi*	Ca_Desi_Chr02	15510668	(C/A)
25827	CadSNP1422	*Desi*	Ca_Desi_Chr02	15510675	(G/A)
25828	CadSNP1423	*Desi*	Ca_Desi_Chr02	15526715	(G/T)
25829	CadSNP1424	*Desi*	Ca_Desi_Chr02	15526673	(G/A)
25830	CadSNP1425	*Desi*	Ca_Desi_Chr02	15526670	(C/T)
25831	CadSNP1426	*Desi*	Ca_Desi_Chr02	15551741	(T/C)
25832	CadSNP1427	*Desi*	Ca_Desi_Chr02	15551733	(A/G)
25833	CadSNP1428	*Desi*	Ca_Desi_Chr02	15575093	(A/G)
25834	CadSNP1429	*Desi*	Ca_Desi_Chr02	15575141	(A/G)
25835	CadSNP1430	*Desi*	Ca_Desi_Chr02	15627570	(T/G)
25836	CadSNP1431	*Desi*	Ca_Desi_Chr02	15653215	(A/T)
25837	CadSNP1432	*Desi*	Ca_Desi_Chr02	15656951	(C/T)
25838	CadSNP1433	*Desi*	Ca_Desi_Chr02	15671766	(C/A)
25839	CadSNP1434	*Desi*	Ca_Desi_Chr02	15677639	(C/T)

S.N.	SNP IDs	Cultivars	Chromosomes/scaffolds	Physical positions (bp)	SNPs
25840	CadSNP1435	*Desi*	Ca_Desi_Chr02	15757574	(T/A)
25841	CadSNP1436	*Desi*	Ca_Desi_Chr02	15769880	(A/T)
25842	CadSNP1437	*Desi*	Ca_Desi_Chr02	15769883	(A/C)
25843	CadSNP1438	*Desi*	Ca_Desi_Chr02	15772497	(G/A)
25844	CadSNP1439	*Desi*	Ca_Desi_Chr02	15786439	(G/A)
25845	CadSNP1440	*Desi*	Ca_Desi_Chr02	15874395	(A/G)
25846	CadSNP1441	*Desi*	Ca_Desi_Chr02	15878454	(C/A)
25847	CadSNP1442	*Desi*	Ca_Desi_Chr02	15878469	(C/A)
25848	CadSNP1443	*Desi*	Ca_Desi_Chr02	15878472	(A/T)
25849	CadSNP1444	*Desi*	Ca_Desi_Chr02	15878509	(A/T)
25850	CadSNP1445	*Desi*	Ca_Desi_Chr02	15905306	(T/C)
25851	CadSNP1446	*Desi*	Ca_Desi_Chr02	15905388	(T/A)
25852	CadSNP1447	*Desi*	Ca_Desi_Chr02	15959857	(A/G)
25853	CadSNP1448	*Desi*	Ca_Desi_Chr02	15970026	(C/G)
25854	CadSNP1449	*Desi*	Ca_Desi_Chr02	15970055	(T/C)
25855	CadSNP1450	*Desi*	Ca_Desi_Chr02	15970076	(A/T)
25856	CadSNP1451	*Desi*	Ca_Desi_Chr02	15970077	(A/G)
25857	CadSNP1452	*Desi*	Ca_Desi_Chr02	15970117	(A/G)
25858	CadSNP1453	*Desi*	Ca_Desi_Chr02	15970114	(A/G)
25859	CadSNP1454	*Desi*	Ca_Desi_Chr02	16066784	(G/A)
25860	CadSNP1455	*Desi*	Ca_Desi_Chr02	16066788	(C/T)
25861	CadSNP1456	*Desi*	Ca_Desi_Chr02	16113340	(C/A)
25862	CadSNP1457	*Desi*	Ca_Desi_Chr02	16114802	(T/C)
25863	CadSNP1458	*Desi*	Ca_Desi_Chr02	16114821	(A/T)
25864	CadSNP1459	*Desi*	Ca_Desi_Chr02	16116122	(C/G)
25865	CadSNP1460	*Desi*	Ca_Desi_Chr02	16130330	(A/G)
25866	CadSNP1461	*Desi*	Ca_Desi_Chr02	16136692	(A/T)
25867	CadSNP1462	*Desi*	Ca_Desi_Chr02	16136703	(T/C)
25868	CadSNP1463	*Desi*	Ca_Desi_Chr02	16136908	(C/T)
25869	CadSNP1464	*Desi*	Ca_Desi_Chr02	16137005	(C/T)
25870	CadSNP1465	*Desi*	Ca_Desi_Chr02	16138793	(T/C)
25871	CadSNP1466	*Desi*	Ca_Desi_Chr02	16138943	(A/C)
25872	CadSNP1467	*Desi*	Ca_Desi_Chr02	16140958	(A/C)
25873	CadSNP1468	*Desi*	Ca_Desi_Chr02	16157597	(A/C)
25874	CadSNP1469	*Desi*	Ca_Desi_Chr02	16172626	(G/A)
25875	CadSNP1470	*Desi*	Ca_Desi_Chr02	16172603	(A/G)
25876	CadSNP1471	*Desi*	Ca_Desi_Chr02	16310713	(C/G)
25877	CadSNP1472	*Desi*	Ca_Desi_Chr02	16637277	(T/G)

S.N.	SNP IDs	Cultivars	Chromosomes/scaffolds	Physical positions (bp)	SNPs
25878	CadSNP1473	*Desi*	Ca_Desi_Chr02	16689981	(C/T)
25879	CadSNP1474	*Desi*	Ca_Desi_Chr02	16690001	(T/G)
25880	CadSNP1475	*Desi*	Ca_Desi_Chr02	16690008	(C/T)
25881	CadSNP1476	*Desi*	Ca_Desi_Chr02	16690011	(A/T)
25882	CadSNP1477	*Desi*	Ca_Desi_Chr02	16690097	(G/A)
25883	CadSNP1478	*Desi*	Ca_Desi_Chr02	16712209	(A/G)
25884	CadSNP1479	*Desi*	Ca_Desi_Chr02	16741302	(C/T)
25885	CadSNP1480	*Desi*	Ca_Desi_Chr02	16741251	(C/T)
25886	CadSNP1481	*Desi*	Ca_Desi_Chr02	16741238	(G/T)
25887	CadSNP1482	*Desi*	Ca_Desi_Chr02	16748554	(G/T)
25888	CadSNP1483	*Desi*	Ca_Desi_Chr02	16748556	(T/G)
25889	CadSNP1484	*Desi*	Ca_Desi_Chr02	16748700	(G/A)
25890	CadSNP1485	*Desi*	Ca_Desi_Chr02	16748679	(T/A)
25891	CadSNP1486	*Desi*	Ca_Desi_Chr02	16748726	(T/A)
25892	CadSNP1487	*Desi*	Ca_Desi_Chr02	16748758	(C/A)
25893	CadSNP1488	*Desi*	Ca_Desi_Chr02	16748779	(T/A)
25894	CadSNP1489	*Desi*	Ca_Desi_Chr02	16748803	(T/G)
25895	CadSNP1490	*Desi*	Ca_Desi_Chr02	16838445	(C/G)
25896	CadSNP1491	*Desi*	Ca_Desi_Chr02	16856357	(A/C)
25897	CadSNP1492	*Desi*	Ca_Desi_Chr02	16872692	(T/G)
25898	CadSNP1493	*Desi*	Ca_Desi_Chr02	16872691	(A/T)
25899	CadSNP1494	*Desi*	Ca_Desi_Chr02	16872685	(A/G)
25900	CadSNP1495	*Desi*	Ca_Desi_Chr02	16872684	(A/T)
25901	CadSNP1496	*Desi*	Ca_Desi_Chr02	16872680	(T/A)
25902	CadSNP1497	*Desi*	Ca_Desi_Chr02	16872674	(T/C)
25903	CadSNP1498	*Desi*	Ca_Desi_Chr02	16872671	(A/G)
25904	CadSNP1499	*Desi*	Ca_Desi_Chr02	16872665	(T/A)
25905	CadSNP1500	*Desi*	Ca_Desi_Chr02	16872659	(T/C)
25906	CadSNP1501	*Desi*	Ca_Desi_Chr02	16872657	(C/T)
25907	CadSNP1502	*Desi*	Ca_Desi_Chr02	16872653	(C/T)
25908	CadSNP1503	*Desi*	Ca_Desi_Chr02	16954914	(G/A)
25909	CadSNP1504	*Desi*	Ca_Desi_Chr02	17060800	(T/C)
25910	CadSNP1505	*Desi*	Ca_Desi_Chr02	17063464	(G/C)
25911	CadSNP1506	*Desi*	Ca_Desi_Chr02	17063468	(T/C)
25912	CadSNP1507	*Desi*	Ca_Desi_Chr02	17167481	(A/G)
25913	CadSNP1508	*Desi*	Ca_Desi_Chr02	17184026	(C/T)
25914	CadSNP1509	*Desi*	Ca_Desi_Chr02	17187208	(T/C)
25915	CadSNP1510	*Desi*	Ca_Desi_Chr02	17187245	(G/C)

S.N.	SNP IDs	Cultivars	Chromosomes/scaffolds	Physical positions (bp)	SNPs
25916	CadSNP1511	*Desi*	Ca_Desi_Chr02	17187980	(C/A)
25917	CadSNP1512	*Desi*	Ca_Desi_Chr03	44873	(T/G)
25918	CadSNP1513	*Desi*	Ca_Desi_Chr03	44877	(T/C)
25919	CadSNP1514	*Desi*	Ca_Desi_Chr03	45008	(C/T)
25920	CadSNP1515	*Desi*	Ca_Desi_Chr03	45009	(C/A)
25921	CadSNP1516	*Desi*	Ca_Desi_Chr03	45013	(G/A)
25922	CadSNP1517	*Desi*	Ca_Desi_Chr03	76445	(G/A)
25923	CadSNP1518	*Desi*	Ca_Desi_Chr03	80216	(A/C)
25924	CadSNP1519	*Desi*	Ca_Desi_Chr03	126485	(T/G)
25925	CadSNP1520	*Desi*	Ca_Desi_Chr03	126527	(C/T)
25926	CadSNP1521	*Desi*	Ca_Desi_Chr03	126619	(G/A)
25927	CadSNP1522	*Desi*	Ca_Desi_Chr03	335668	(T/C)
25928	CadSNP1523	*Desi*	Ca_Desi_Chr03	347892	(C/T)
25929	CadSNP1524	*Desi*	Ca_Desi_Chr03	347865	(C/A)
25930	CadSNP1525	*Desi*	Ca_Desi_Chr03	645024	(A/C)
25931	CadSNP1526	*Desi*	Ca_Desi_Chr03	669270	(C/T)
25932	CadSNP1527	*Desi*	Ca_Desi_Chr03	684601	(T/C)
25933	CadSNP1528	*Desi*	Ca_Desi_Chr03	684572	(G/A)
25934	CadSNP1529	*Desi*	Ca_Desi_Chr03	690339	(G/A)
25935	CadSNP1530	*Desi*	Ca_Desi_Chr03	690372	(G/C)
25936	CadSNP1531	*Desi*	Ca_Desi_Chr03	691472	(C/T)
25937	CadSNP1532	*Desi*	Ca_Desi_Chr03	693294	(G/A)
25938	CadSNP1533	*Desi*	Ca_Desi_Chr03	695497	(C/T)
25939	CadSNP1534	*Desi*	Ca_Desi_Chr03	695494	(T/A)
25940	CadSNP1535	*Desi*	Ca_Desi_Chr03	697967	(T/G)
25941	CadSNP1536	*Desi*	Ca_Desi_Chr03	698068	(G/A)
25942	CadSNP1537	*Desi*	Ca_Desi_Chr03	698170	(T/G)
25943	CadSNP1538	*Desi*	Ca_Desi_Chr03	753812	(T/G)
25944	CadSNP1539	*Desi*	Ca_Desi_Chr03	762935	(G/A)
25945	CadSNP1540	*Desi*	Ca_Desi_Chr03	768344	(G/A)
25946	CadSNP1541	*Desi*	Ca_Desi_Chr03	768325	(C/G)
25947	CadSNP1542	*Desi*	Ca_Desi_Chr03	768323	(A/T)
25948	CadSNP1543	*Desi*	Ca_Desi_Chr03	782934	(C/T)
25949	CadSNP1544	*Desi*	Ca_Desi_Chr03	786406	(A/G)
25950	CadSNP1545	*Desi*	Ca_Desi_Chr03	854698	(C/T)
25951	CadSNP1546	*Desi*	Ca_Desi_Chr03	954899	(T/C)
25952	CadSNP1547	*Desi*	Ca_Desi_Chr03	993205	(A/G)
25953	CadSNP1548	*Desi*	Ca_Desi_Chr03	993204	(G/A)

S.N.	SNP IDs	Cultivars	Chromosomes/scaffolds	Physical positions (bp)	SNPs
25954	CadSNP1549	*Desi*	Ca_Desi_Chr03	993159	(C/T)
25955	CadSNP1550	*Desi*	Ca_Desi_Chr03	1000520	(T/G)
25956	CadSNP1551	*Desi*	Ca_Desi_Chr03	1001326	(A/G)
25957	CadSNP1552	*Desi*	Ca_Desi_Chr03	1006408	(C/T)
25958	CadSNP1553	*Desi*	Ca_Desi_Chr03	1009680	(A/C)
25959	CadSNP1554	*Desi*	Ca_Desi_Chr03	1037187	(A/G)
25960	CadSNP1555	*Desi*	Ca_Desi_Chr03	1132867	(G/A)
25961	CadSNP1556	*Desi*	Ca_Desi_Chr03	1138482	(A/G)
25962	CadSNP1557	*Desi*	Ca_Desi_Chr03	1142176	(T/C)
25963	CadSNP1558	*Desi*	Ca_Desi_Chr03	1142293	(C/A)
25964	CadSNP1559	*Desi*	Ca_Desi_Chr03	1146163	(T/C)
25965	CadSNP1560	*Desi*	Ca_Desi_Chr03	1147189	(C/T)
25966	CadSNP1561	*Desi*	Ca_Desi_Chr03	1148157	(G/T)
25967	CadSNP1562	*Desi*	Ca_Desi_Chr03	1148176	(T/G)
25968	CadSNP1563	*Desi*	Ca_Desi_Chr03	1148257	(A/G)
25969	CadSNP1564	*Desi*	Ca_Desi_Chr03	1148353	(A/C)
25970	CadSNP1565	*Desi*	Ca_Desi_Chr03	1156273	(T/C)
25971	CadSNP1566	*Desi*	Ca_Desi_Chr03	1156347	(G/C)
25972	CadSNP1567	*Desi*	Ca_Desi_Chr03	1198705	(A/C)
25973	CadSNP1568	*Desi*	Ca_Desi_Chr03	1198729	(T/C)
25974	CadSNP1569	*Desi*	Ca_Desi_Chr03	1198799	(T/A)
25975	CadSNP1570	*Desi*	Ca_Desi_Chr03	1199241	(C/A)
25976	CadSNP1571	*Desi*	Ca_Desi_Chr03	1205701	(A/G)
25977	CadSNP1572	*Desi*	Ca_Desi_Chr03	1205850	(A/T)
25978	CadSNP1573	*Desi*	Ca_Desi_Chr03	1205847	(G/C)
25979	CadSNP1574	*Desi*	Ca_Desi_Chr03	1205826	(T/G)
25980	CadSNP1575	*Desi*	Ca_Desi_Chr03	1205824	(T/A)
25981	CadSNP1576	*Desi*	Ca_Desi_Chr03	1205811	(T/A)
25982	CadSNP1577	*Desi*	Ca_Desi_Chr03	1205789	(A/C)
25983	CadSNP1578	*Desi*	Ca_Desi_Chr03	1208905	(A/G)
25984	CadSNP1579	*Desi*	Ca_Desi_Chr03	1208869	(T/C)
25985	CadSNP1580	*Desi*	Ca_Desi_Chr03	1223420	(C/A)
25986	CadSNP1581	*Desi*	Ca_Desi_Chr03	1228195	(A/G)
25987	CadSNP1582	*Desi*	Ca_Desi_Chr03	1228218	(T/C)
25988	CadSNP1583	*Desi*	Ca_Desi_Chr03	1228231	(A/C)
25989	CadSNP1584	*Desi*	Ca_Desi_Chr03	1233409	(A/C)
25990	CadSNP1585	*Desi*	Ca_Desi_Chr03	1255515	(C/T)
25991	CadSNP1586	*Desi*	Ca_Desi_Chr03	1267770	(T/C)

S.N.	SNP IDs	Cultivars	Chromosomes/scaffolds	Physical positions (bp)	SNPs
25992	CadSNP1587	*Desi*	Ca_Desi_Chr03	1286463	(T/C)
25993	CadSNP1588	*Desi*	Ca_Desi_Chr03	1286513	(G/A)
25994	CadSNP1589	*Desi*	Ca_Desi_Chr03	1298429	(G/T)
25995	CadSNP1590	*Desi*	Ca_Desi_Chr03	1298524	(G/A)
25996	CadSNP1591	*Desi*	Ca_Desi_Chr03	1298497	(C/T)
25997	CadSNP1592	*Desi*	Ca_Desi_Chr03	1298488	(A/G)
25998	CadSNP1593	*Desi*	Ca_Desi_Chr03	1348788	(C/G)
25999	CadSNP1594	*Desi*	Ca_Desi_Chr03	1348911	(T/C)
26000	CadSNP1595	*Desi*	Ca_Desi_Chr03	1392395	(A/G)
26001	CadSNP1596	*Desi*	Ca_Desi_Chr03	1404434	(T/A)
26002	CadSNP1597	*Desi*	Ca_Desi_Chr03	1404499	(A/G)
26003	CadSNP1598	*Desi*	Ca_Desi_Chr03	1414321	(A/G)
26004	CadSNP1599	*Desi*	Ca_Desi_Chr03	1416925	(C/T)
26005	CadSNP1600	*Desi*	Ca_Desi_Chr03	1416904	(T/A)
26006	CadSNP1601	*Desi*	Ca_Desi_Chr03	1416895	(A/T)
26007	CadSNP1602	*Desi*	Ca_Desi_Chr03	1427742	(C/T)
26008	CadSNP1603	*Desi*	Ca_Desi_Chr03	1427752	(C/T)
26009	CadSNP1604	*Desi*	Ca_Desi_Chr03	1427760	(A/G)
26010	CadSNP1605	*Desi*	Ca_Desi_Chr03	1427788	(C/T)
26011	CadSNP1606	*Desi*	Ca_Desi_Chr03	1427793	(G/A)
26012	CadSNP1607	*Desi*	Ca_Desi_Chr03	1427796	(A/G)
26013	CadSNP1608	*Desi*	Ca_Desi_Chr03	1427799	(T/C)
26014	CadSNP1609	*Desi*	Ca_Desi_Chr03	1427811	(A/T)
26015	CadSNP1610	*Desi*	Ca_Desi_Chr03	1427822	(A/T)
26016	CadSNP1611	*Desi*	Ca_Desi_Chr03	1427904	(A/G)
26017	CadSNP1612	*Desi*	Ca_Desi_Chr03	1427901	(A/T)
26018	CadSNP1613	*Desi*	Ca_Desi_Chr03	1427892	(A/T)
26019	CadSNP1614	*Desi*	Ca_Desi_Chr03	1427874	(T/C)
26020	CadSNP1615	*Desi*	Ca_Desi_Chr03	1427865	(T/C)
26021	CadSNP1616	*Desi*	Ca_Desi_Chr03	1427862	(T/A)
26022	CadSNP1617	*Desi*	Ca_Desi_Chr03	1427850	(A/G)
26023	CadSNP1618	*Desi*	Ca_Desi_Chr03	1427826	(T/C)
26024	CadSNP1619	*Desi*	Ca_Desi_Chr03	1427905	(T/C)
26025	CadSNP1620	*Desi*	Ca_Desi_Chr03	1463047	(A/G)
26026	CadSNP1621	*Desi*	Ca_Desi_Chr03	1503173	(A/T)
26027	CadSNP1622	*Desi*	Ca_Desi_Chr03	1516868	(C/A)
26028	CadSNP1623	*Desi*	Ca_Desi_Chr03	1533891	(A/C)
26029	CadSNP1624	*Desi*	Ca_Desi_Chr03	1536663	(G/T)

S.N.	SNP IDs	Cultivars	Chromosomes/scaffolds	Physical positions (bp)	SNPs
26030	CadSNP1625	*Desi*	Ca_Desi_Chr03	1536675	(G/A)
26031	CadSNP1626	*Desi*	Ca_Desi_Chr03	1536708	(A/G)
26032	CadSNP1627	*Desi*	Ca_Desi_Chr03	1536748	(A/G)
26033	CadSNP1628	*Desi*	Ca_Desi_Chr03	1536750	(A/G)
26034	CadSNP1629	*Desi*	Ca_Desi_Chr03	1536753	(C/T)
26035	CadSNP1630	*Desi*	Ca_Desi_Chr03	1536804	(A/T)
26036	CadSNP1631	*Desi*	Ca_Desi_Chr03	1554464	(T/C)
26037	CadSNP1632	*Desi*	Ca_Desi_Chr03	1554518	(T/C)
26038	CadSNP1633	*Desi*	Ca_Desi_Chr03	1554578	(C/T)
26039	CadSNP1634	*Desi*	Ca_Desi_Chr03	1633031	(T/A)
26040	CadSNP1635	*Desi*	Ca_Desi_Chr03	1659975	(A/G)
26041	CadSNP1636	*Desi*	Ca_Desi_Chr03	1667821	(C/T)
26042	CadSNP1637	*Desi*	Ca_Desi_Chr03	1670337	(C/T)
26043	CadSNP1638	*Desi*	Ca_Desi_Chr03	1686934	(G/C)
26044	CadSNP1639	*Desi*	Ca_Desi_Chr03	1686974	(G/A)
26045	CadSNP1640	*Desi*	Ca_Desi_Chr03	2660813	(A/G)
26046	CadSNP1641	*Desi*	Ca_Desi_Chr03	2660782	(C/T)
26047	CadSNP1642	*Desi*	Ca_Desi_Chr03	2672499	(G/A)
26048	CadSNP1643	*Desi*	Ca_Desi_Chr03	2674284	(T/A)
26049	CadSNP1644	*Desi*	Ca_Desi_Chr03	2674302	(A/G)
26050	CadSNP1645	*Desi*	Ca_Desi_Chr03	2674439	(A/C)
26051	CadSNP1646	*Desi*	Ca_Desi_Chr03	2679323	(A/G)
26052	CadSNP1647	*Desi*	Ca_Desi_Chr03	2683637	(T/G)
26053	CadSNP1648	*Desi*	Ca_Desi_Chr03	2693535	(C/G)
26054	CadSNP1649	*Desi*	Ca_Desi_Chr03	2693537	(C/T)
26055	CadSNP1650	*Desi*	Ca_Desi_Chr03	2700887	(T/C)
26056	CadSNP1651	*Desi*	Ca_Desi_Chr03	2713239	(T/A)
26057	CadSNP1652	*Desi*	Ca_Desi_Chr03	2713248	(A/C)
26058	CadSNP1653	*Desi*	Ca_Desi_Chr03	2716549	(G/A)
26059	CadSNP1654	*Desi*	Ca_Desi_Chr03	2718128	(G/C)
26060	CadSNP1655	*Desi*	Ca_Desi_Chr03	2718129	(G/A)
26061	CadSNP1656	*Desi*	Ca_Desi_Chr03	2718182	(A/T)
26062	CadSNP1657	*Desi*	Ca_Desi_Chr03	2725750	(A/G)
26063	CadSNP1658	*Desi*	Ca_Desi_Chr03	2729400	(A/G)
26064	CadSNP1659	*Desi*	Ca_Desi_Chr03	2737288	(T/C)
26065	CadSNP1660	*Desi*	Ca_Desi_Chr03	2754108	(C/T)
26066	CadSNP1661	*Desi*	Ca_Desi_Chr03	2754162	(C/G)
26067	CadSNP1662	*Desi*	Ca_Desi_Chr03	2758011	(G/T)

S.N.	SNP IDs	Cultivars	Chromosomes/scaffolds	Physical positions (bp)	SNPs
26068	CadSNP1663	*Desi*	Ca_Desi_Chr03	2758003	(A/C)
26069	CadSNP1664	*Desi*	Ca_Desi_Chr03	2787196	(C/G)
26070	CadSNP1665	*Desi*	Ca_Desi_Chr03	2787283	(G/C)
26071	CadSNP1666	*Desi*	Ca_Desi_Chr03	2787268	(T/C)
26072	CadSNP1667	*Desi*	Ca_Desi_Chr03	2787355	(G/C)
26073	CadSNP1668	*Desi*	Ca_Desi_Chr03	2793058	(G/A)
26074	CadSNP1669	*Desi*	Ca_Desi_Chr03	2795523	(C/T)
26075	CadSNP1670	*Desi*	Ca_Desi_Chr03	2799304	(T/C)
26076	CadSNP1671	*Desi*	Ca_Desi_Chr03	2803750	(A/G)
26077	CadSNP1672	*Desi*	Ca_Desi_Chr03	2812122	(G/A)
26078	CadSNP1673	*Desi*	Ca_Desi_Chr03	2814757	(A/G)
26079	CadSNP1674	*Desi*	Ca_Desi_Chr03	2814908	(G/A)
26080	CadSNP1675	*Desi*	Ca_Desi_Chr03	2840621	(G/A)
26081	CadSNP1676	*Desi*	Ca_Desi_Chr03	2873563	(G/T)
26082	CadSNP1677	*Desi*	Ca_Desi_Chr03	2920219	(A/G)
26083	CadSNP1678	*Desi*	Ca_Desi_Chr03	2920379	(G/A)
26084	CadSNP1679	*Desi*	Ca_Desi_Chr03	2953036	(C/T)
26085	CadSNP1680	*Desi*	Ca_Desi_Chr03	2986236	(C/T)
26086	CadSNP1681	*Desi*	Ca_Desi_Chr03	2986233	(T/C)
26087	CadSNP1682	*Desi*	Ca_Desi_Chr03	2991036	(A/C)
26088	CadSNP1683	*Desi*	Ca_Desi_Chr03	3002455	(A/C)
26089	CadSNP1684	*Desi*	Ca_Desi_Chr03	3015825	(A/G)
26090	CadSNP1685	*Desi*	Ca_Desi_Chr03	3015816	(A/G)
26091	CadSNP1686	*Desi*	Ca_Desi_Chr03	3048193	(C/T)
26092	CadSNP1687	*Desi*	Ca_Desi_Chr03	3048229	(T/C)
26093	CadSNP1688	*Desi*	Ca_Desi_Chr03	3130784	(G/T)
26094	CadSNP1689	*Desi*	Ca_Desi_Chr03	3179253	(C/T)
26095	CadSNP1690	*Desi*	Ca_Desi_Chr03	3187918	(A/T)
26096	CadSNP1691	*Desi*	Ca_Desi_Chr03	3218964	(A/G)
26097	CadSNP1692	*Desi*	Ca_Desi_Chr03	3224433	(T/C)
26098	CadSNP1693	*Desi*	Ca_Desi_Chr03	3254955	(C/T)
26099	CadSNP1694	*Desi*	Ca_Desi_Chr03	3255060	(C/T)
26100	CadSNP1695	*Desi*	Ca_Desi_Chr03	3255029	(C/T)
26101	CadSNP1696	*Desi*	Ca_Desi_Chr03	3267987	(T/G)
26102	CadSNP1697	*Desi*	Ca_Desi_Chr03	3295800	(T/A)
26103	CadSNP1698	*Desi*	Ca_Desi_Chr03	3333129	(A/G)
26104	CadSNP1699	*Desi*	Ca_Desi_Chr03	3398702	(A/G)
26105	CadSNP1700	*Desi*	Ca_Desi_Chr03	3398708	(C/A)

S.N.	SNP IDs	Cultivars	Chromosomes/scaffolds	Physical positions (bp)	SNPs
26106	CadSNP1701	*Desi*	Ca_Desi_Chr03	3404200	(C/A)
26107	CadSNP1702	*Desi*	Ca_Desi_Chr03	3424969	(T/C)
26108	CadSNP1703	*Desi*	Ca_Desi_Chr03	3427256	(G/T)
26109	CadSNP1704	*Desi*	Ca_Desi_Chr03	3455564	(G/A)
26110	CadSNP1705	*Desi*	Ca_Desi_Chr03	3479333	(A/C)
26111	CadSNP1706	*Desi*	Ca_Desi_Chr03	3488455	(C/A)
26112	CadSNP1707	*Desi*	Ca_Desi_Chr03	3493823	(T/G)
26113	CadSNP1708	*Desi*	Ca_Desi_Chr03	3493826	(C/T)
26114	CadSNP1709	*Desi*	Ca_Desi_Chr03	3493840	(G/C)
26115	CadSNP1710	*Desi*	Ca_Desi_Chr03	3495518	(T/A)
26116	CadSNP1711	*Desi*	Ca_Desi_Chr03	3495509	(T/C)
26117	CadSNP1712	*Desi*	Ca_Desi_Chr03	3495557	(A/G)
26118	CadSNP1713	*Desi*	Ca_Desi_Chr03	3512869	(A/G)
26119	CadSNP1714	*Desi*	Ca_Desi_Chr03	3523403	(T/C)
26120	CadSNP1715	*Desi*	Ca_Desi_Chr03	3523411	(T/A)
26121	CadSNP1716	*Desi*	Ca_Desi_Chr03	3524914	(A/G)
26122	CadSNP1717	*Desi*	Ca_Desi_Chr03	3524855	(T/C)
26123	CadSNP1718	*Desi*	Ca_Desi_Chr03	3524842	(C/T)
26124	CadSNP1719	*Desi*	Ca_Desi_Chr03	3538547	(C/A)
26125	CadSNP1720	*Desi*	Ca_Desi_Chr03	3567295	(T/C)
26126	CadSNP1721	*Desi*	Ca_Desi_Chr03	3567298	(C/T)
26127	CadSNP1722	*Desi*	Ca_Desi_Chr03	3567301	(C/T)
26128	CadSNP1723	*Desi*	Ca_Desi_Chr03	3567305	(C/T)
26129	CadSNP1724	*Desi*	Ca_Desi_Chr03	3567444	(C/T)
26130	CadSNP1725	*Desi*	Ca_Desi_Chr03	3576516	(C/T)
26131	CadSNP1726	*Desi*	Ca_Desi_Chr03	3576517	(T/C)
26132	CadSNP1727	*Desi*	Ca_Desi_Chr03	3576686	(A/G)
26133	CadSNP1728	*Desi*	Ca_Desi_Chr03	3580883	(T/C)
26134	CadSNP1729	*Desi*	Ca_Desi_Chr03	3593781	(T/C)
26135	CadSNP1730	*Desi*	Ca_Desi_Chr03	3593776	(A/G)
26136	CadSNP1731	*Desi*	Ca_Desi_Chr03	3609578	(C/T)
26137	CadSNP1732	*Desi*	Ca_Desi_Chr03	3621996	(T/C)
26138	CadSNP1733	*Desi*	Ca_Desi_Chr03	3630551	(A/G)
26139	CadSNP1734	*Desi*	Ca_Desi_Chr03	3631362	(C/T)
26140	CadSNP1735	*Desi*	Ca_Desi_Chr03	3713712	(C/G)
26141	CadSNP1736	*Desi*	Ca_Desi_Chr03	3773822	(A/G)
26142	CadSNP1737	*Desi*	Ca_Desi_Chr03	3773865	(T/C)
26143	CadSNP1738	*Desi*	Ca_Desi_Chr03	3774007	(A/T)

S.N.	SNP IDs	Cultivars	Chromosomes/scaffolds	Physical positions (bp)	SNPs
26144	CadSNP1739	*Desi*	Ca_Desi_Chr03	3794458	(A/G)
26145	CadSNP1740	*Desi*	Ca_Desi_Chr03	3860790	(G/C)
26146	CadSNP1741	*Desi*	Ca_Desi_Chr03	3860844	(G/A)
26147	CadSNP1742	*Desi*	Ca_Desi_Chr03	3861051	(C/T)
26148	CadSNP1743	*Desi*	Ca_Desi_Chr03	3884489	(A/C)
26149	CadSNP1744	*Desi*	Ca_Desi_Chr03	3884665	(C/A)
26150	CadSNP1745	*Desi*	Ca_Desi_Chr03	3913855	(G/A)
26151	CadSNP1746	*Desi*	Ca_Desi_Chr03	3946342	(G/A)
26152	CadSNP1747	*Desi*	Ca_Desi_Chr03	3946457	(G/T)
26153	CadSNP1748	*Desi*	Ca_Desi_Chr03	3967388	(G/A)
26154	CadSNP1749	*Desi*	Ca_Desi_Chr03	3967371	(G/T)
26155	CadSNP1750	*Desi*	Ca_Desi_Chr03	3973351	(C/T)
26156	CadSNP1751	*Desi*	Ca_Desi_Chr03	3973376	(T/C)
26157	CadSNP1752	*Desi*	Ca_Desi_Chr03	3973381	(A/G)
26158	CadSNP1753	*Desi*	Ca_Desi_Chr03	4015979	(G/A)
26159	CadSNP1754	*Desi*	Ca_Desi_Chr03	4016158	(C/T)
26160	CadSNP1755	*Desi*	Ca_Desi_Chr03	4016176	(A/C)
26161	CadSNP1756	*Desi*	Ca_Desi_Chr03	4019445	(T/G)
26162	CadSNP1757	*Desi*	Ca_Desi_Chr03	4019505	(T/C)
26163	CadSNP1758	*Desi*	Ca_Desi_Chr03	4039042	(A/G)
26164	CadSNP1759	*Desi*	Ca_Desi_Chr03	4053024	(G/A)
26165	CadSNP1760	*Desi*	Ca_Desi_Chr03	4085844	(A/G)
26166	CadSNP1761	*Desi*	Ca_Desi_Chr03	4086443	(T/C)
26167	CadSNP1762	*Desi*	Ca_Desi_Chr03	4086479	(C/T)
26168	CadSNP1763	*Desi*	Ca_Desi_Chr03	4087924	(T/C)
26169	CadSNP1764	*Desi*	Ca_Desi_Chr03	4087934	(C/A)
26170	CadSNP1765	*Desi*	Ca_Desi_Chr03	4098735	(T/A)
26171	CadSNP1766	*Desi*	Ca_Desi_Chr03	4098784	(C/A)
26172	CadSNP1767	*Desi*	Ca_Desi_Chr03	4100611	(T/C)
26173	CadSNP1768	*Desi*	Ca_Desi_Chr03	4100608	(C/T)
26174	CadSNP1769	*Desi*	Ca_Desi_Chr03	4100572	(C/T)
26175	CadSNP1770	*Desi*	Ca_Desi_Chr03	4100651	(C/T)
26176	CadSNP1771	*Desi*	Ca_Desi_Chr03	4100710	(C/T)
26177	CadSNP1772	*Desi*	Ca_Desi_Chr03	4100757	(T/G)
26178	CadSNP1773	*Desi*	Ca_Desi_Chr03	4100741	(G/A)
26179	CadSNP1774	*Desi*	Ca_Desi_Chr03	4100725	(A/T)
26180	CadSNP1775	*Desi*	Ca_Desi_Chr03	4118888	(G/A)
26181	CadSNP1776	*Desi*	Ca_Desi_Chr03	4118883	(T/C)

S.N.	SNP IDs	Cultivars	Chromosomes/scaffolds	Physical positions (bp)	SNPs
26182	CadSNP1777	*Desi*	Ca_Desi_Chr03	4133177	(T/G)
26183	CadSNP1778	*Desi*	Ca_Desi_Chr03	4133180	(T/C)
26184	CadSNP1779	*Desi*	Ca_Desi_Chr03	4134312	(C/G)
26185	CadSNP1780	*Desi*	Ca_Desi_Chr03	4135452	(G/T)
26186	CadSNP1781	*Desi*	Ca_Desi_Chr03	4143474	(G/A)
26187	CadSNP1782	*Desi*	Ca_Desi_Chr03	4145509	(T/C)
26188	CadSNP1783	*Desi*	Ca_Desi_Chr03	4174824	(T/G)
26189	CadSNP1784	*Desi*	Ca_Desi_Chr03	4174837	(A/G)
26190	CadSNP1785	*Desi*	Ca_Desi_Chr03	4174863	(A/G)
26191	CadSNP1786	*Desi*	Ca_Desi_Chr03	4175622	(A/C)
26192	CadSNP1787	*Desi*	Ca_Desi_Chr03	4182312	(A/T)
26193	CadSNP1788	*Desi*	Ca_Desi_Chr03	4182322	(G/A)
26194	CadSNP1789	*Desi*	Ca_Desi_Chr03	4182356	(T/A)
26195	CadSNP1790	*Desi*	Ca_Desi_Chr03	4187139	(G/A)
26196	CadSNP1791	*Desi*	Ca_Desi_Chr03	4197023	(A/T)
26197	CadSNP1792	*Desi*	Ca_Desi_Chr03	4197024	(A/T)
26198	CadSNP1793	*Desi*	Ca_Desi_Chr03	4197306	(A/G)
26199	CadSNP1794	*Desi*	Ca_Desi_Chr03	4197307	(C/T)
26200	CadSNP1795	*Desi*	Ca_Desi_Chr03	4197439	(G/A)
26201	CadSNP1796	*Desi*	Ca_Desi_Chr03	4197390	(G/T)
26202	CadSNP1797	*Desi*	Ca_Desi_Chr03	4197482	(T/A)
26203	CadSNP1798	*Desi*	Ca_Desi_Chr03	4197487	(T/A)
26204	CadSNP1799	*Desi*	Ca_Desi_Chr03	4211457	(C/T)
26205	CadSNP1800	*Desi*	Ca_Desi_Chr03	4211455	(C/T)
26206	CadSNP1801	*Desi*	Ca_Desi_Chr03	4211441	(G/T)
26207	CadSNP1802	*Desi*	Ca_Desi_Chr03	4211431	(G/C)
26208	CadSNP1803	*Desi*	Ca_Desi_Chr03	4216738	(T/C)
26209	CadSNP1804	*Desi*	Ca_Desi_Chr03	4229802	(C/A)
26210	CadSNP1805	*Desi*	Ca_Desi_Chr03	4250045	(T/G)
26211	CadSNP1806	*Desi*	Ca_Desi_Chr03	4261374	(A/G)
26212	CadSNP1807	*Desi*	Ca_Desi_Chr03	4261410	(C/T)
26213	CadSNP1808	*Desi*	Ca_Desi_Chr03	4261422	(C/T)
26214	CadSNP1809	*Desi*	Ca_Desi_Chr03	4300046	(A/G)
26215	CadSNP1810	*Desi*	Ca_Desi_Chr03	4300111	(T/C)
26216	CadSNP1811	*Desi*	Ca_Desi_Chr03	4314473	(T/C)
26217	CadSNP1812	*Desi*	Ca_Desi_Chr03	4316822	(T/A)
26218	CadSNP1813	*Desi*	Ca_Desi_Chr03	4330756	(C/T)
26219	CadSNP1814	*Desi*	Ca_Desi_Chr03	4347515	(G/T)

S.N.	SNP IDs	Cultivars	Chromosomes/scaffolds	Physical positions (bp)	SNPs
26220	CadSNP1815	*Desi*	Ca_Desi_Chr03	4347520	(C/G)
26221	CadSNP1816	*Desi*	Ca_Desi_Chr03	4347579	(G/A)
26222	CadSNP1817	*Desi*	Ca_Desi_Chr03	4356878	(A/T)
26223	CadSNP1818	*Desi*	Ca_Desi_Chr03	4356995	(G/A)
26224	CadSNP1819	*Desi*	Ca_Desi_Chr03	4356981	(C/T)
26225	CadSNP1820	*Desi*	Ca_Desi_Chr03	4365790	(C/A)
26226	CadSNP1821	*Desi*	Ca_Desi_Chr03	4389997	(T/C)
26227	CadSNP1822	*Desi*	Ca_Desi_Chr03	4401074	(A/G)
26228	CadSNP1823	*Desi*	Ca_Desi_Chr03	4415458	(G/A)
26229	CadSNP1824	*Desi*	Ca_Desi_Chr03	4415548	(T/A)
26230	CadSNP1825	*Desi*	Ca_Desi_Chr03	4415704	(T/A)
26231	CadSNP1826	*Desi*	Ca_Desi_Chr03	4415709	(C/T)
26232	CadSNP1827	*Desi*	Ca_Desi_Chr03	4451536	(A/C)
26233	CadSNP1828	*Desi*	Ca_Desi_Chr03	4572951	(T/C)
26234	CadSNP1829	*Desi*	Ca_Desi_Chr03	4573221	(A/T)
26235	CadSNP1830	*Desi*	Ca_Desi_Chr03	4580231	(A/G)
26236	CadSNP1831	*Desi*	Ca_Desi_Chr03	4581060	(G/A)
26237	CadSNP1832	*Desi*	Ca_Desi_Chr03	4581079	(T/G)
26238	CadSNP1833	*Desi*	Ca_Desi_Chr03	4664491	(G/A)
26239	CadSNP1834	*Desi*	Ca_Desi_Chr03	4664461	(G/C)
26240	CadSNP1835	*Desi*	Ca_Desi_Chr03	4698954	(A/G)
26241	CadSNP1836	*Desi*	Ca_Desi_Chr03	4721302	(C/T)
26242	CadSNP1837	*Desi*	Ca_Desi_Chr03	4736130	(A/T)
26243	CadSNP1838	*Desi*	Ca_Desi_Chr03	4741278	(C/T)
26244	CadSNP1839	*Desi*	Ca_Desi_Chr03	4741284	(C/T)
26245	CadSNP1840	*Desi*	Ca_Desi_Chr03	4741395	(G/C)
26246	CadSNP1841	*Desi*	Ca_Desi_Chr03	4787269	(G/T)
26247	CadSNP1842	*Desi*	Ca_Desi_Chr03	4787368	(A/T)
26248	CadSNP1843	*Desi*	Ca_Desi_Chr03	4801104	(T/C)
26249	CadSNP1844	*Desi*	Ca_Desi_Chr03	4801073	(A/C)
26250	CadSNP1845	*Desi*	Ca_Desi_Chr03	5804745	(G/C)
26251	CadSNP1846	*Desi*	Ca_Desi_Chr03	5850077	(T/G)
26252	CadSNP1847	*Desi*	Ca_Desi_Chr03	5851070	(C/T)
26253	CadSNP1848	*Desi*	Ca_Desi_Chr03	5851142	(A/G)
26254	CadSNP1849	*Desi*	Ca_Desi_Chr03	5944499	(G/T)
26255	CadSNP1850	*Desi*	Ca_Desi_Chr03	5976239	(C/T)
26256	CadSNP1851	*Desi*	Ca_Desi_Chr03	5990527	(G/A)
26257	CadSNP1852	*Desi*	Ca_Desi_Chr03	5990581	(A/G)

S.N.	SNP IDs	Cultivars	Chromosomes/scaffolds	Physical positions (bp)	SNPs
26258	CadSNP1853	*Desi*	Ca_Desi_Chr03	5990565	(T/C)
26259	CadSNP1854	*Desi*	Ca_Desi_Chr03	6018129	(A/G)
26260	CadSNP1855	*Desi*	Ca_Desi_Chr03	6019858	(T/C)
26261	CadSNP1856	*Desi*	Ca_Desi_Chr03	6027089	(A/G)
26262	CadSNP1857	*Desi*	Ca_Desi_Chr03	6078359	(A/G)
26263	CadSNP1858	*Desi*	Ca_Desi_Chr03	6078356	(A/G)
26264	CadSNP1859	*Desi*	Ca_Desi_Chr03	6078513	(C/T)
26265	CadSNP1860	*Desi*	Ca_Desi_Chr03	6078557	(A/C)
26266	CadSNP1861	*Desi*	Ca_Desi_Chr03	6109024	(T/C)
26267	CadSNP1862	*Desi*	Ca_Desi_Chr03	6108969	(T/C)
26268	CadSNP1863	*Desi*	Ca_Desi_Chr03	6119240	(G/A)
26269	CadSNP1864	*Desi*	Ca_Desi_Chr03	6119374	(G/T)
26270	CadSNP1865	*Desi*	Ca_Desi_Chr03	6119459	(G/A)
26271	CadSNP1866	*Desi*	Ca_Desi_Chr03	6120706	(C/T)
26272	CadSNP1867	*Desi*	Ca_Desi_Chr03	6139595	(C/G)
26273	CadSNP1868	*Desi*	Ca_Desi_Chr03	6139652	(A/T)
26274	CadSNP1869	*Desi*	Ca_Desi_Chr03	6172165	(G/T)
26275	CadSNP1870	*Desi*	Ca_Desi_Chr03	6172170	(A/G)
26276	CadSNP1871	*Desi*	Ca_Desi_Chr03	6172185	(G/A)
26277	CadSNP1872	*Desi*	Ca_Desi_Chr03	6172225	(A/T)
26278	CadSNP1873	*Desi*	Ca_Desi_Chr03	6172249	(G/T)
26279	CadSNP1874	*Desi*	Ca_Desi_Chr03	6172800	(C/A)
26280	CadSNP1875	*Desi*	Ca_Desi_Chr03	6172834	(T/C)
26281	CadSNP1876	*Desi*	Ca_Desi_Chr03	6195683	(A/G)
26282	CadSNP1877	*Desi*	Ca_Desi_Chr03	6260072	(T/C)
26283	CadSNP1878	*Desi*	Ca_Desi_Chr03	6260171	(C/A)
26284	CadSNP1879	*Desi*	Ca_Desi_Chr03	6262005	(C/T)
26285	CadSNP1880	*Desi*	Ca_Desi_Chr03	6295042	(T/A)
26286	CadSNP1881	*Desi*	Ca_Desi_Chr03	6330548	(C/A)
26287	CadSNP1882	*Desi*	Ca_Desi_Chr03	6330729	(C/T)
26288	CadSNP1883	*Desi*	Ca_Desi_Chr03	6330673	(A/G)
26289	CadSNP1884	*Desi*	Ca_Desi_Chr03	6390861	(A/G)
26290	CadSNP1885	*Desi*	Ca_Desi_Chr03	6390842	(C/T)
26291	CadSNP1886	*Desi*	Ca_Desi_Chr03	6390835	(C/T)
26292	CadSNP1887	*Desi*	Ca_Desi_Chr03	6392638	(G/A)
26293	CadSNP1888	*Desi*	Ca_Desi_Chr03	6408930	(C/T)
26294	CadSNP1889	*Desi*	Ca_Desi_Chr03	6434624	(T/A)
26295	CadSNP1890	*Desi*	Ca_Desi_Chr03	6434627	(G/T)

S.N.	SNP IDs	Cultivars	Chromosomes/scaffolds	Physical positions (bp)	SNPs
26296	CadSNP1891	*Desi*	Ca_Desi_Chr03	6434644	(T/C)
26297	CadSNP1892	*Desi*	Ca_Desi_Chr03	6435914	(A/C)
26298	CadSNP1893	*Desi*	Ca_Desi_Chr03	6435907	(T/G)
26299	CadSNP1894	*Desi*	Ca_Desi_Chr03	6436024	(G/A)
26300	CadSNP1895	*Desi*	Ca_Desi_Chr03	6435997	(A/G)
26301	CadSNP1896	*Desi*	Ca_Desi_Chr03	6436085	(G/A)
26302	CadSNP1897	*Desi*	Ca_Desi_Chr03	6436108	(A/C)
26303	CadSNP1898	*Desi*	Ca_Desi_Chr03	6449814	(C/T)
26304	CadSNP1899	*Desi*	Ca_Desi_Chr03	6449896	(C/T)
26305	CadSNP1900	*Desi*	Ca_Desi_Chr03	6449881	(G/A)
26306	CadSNP1901	*Desi*	Ca_Desi_Chr03	6485692	(A/G)
26307	CadSNP1902	*Desi*	Ca_Desi_Chr03	6490909	(C/T)
26308	CadSNP1903	*Desi*	Ca_Desi_Chr03	6499799	(A/C)
26309	CadSNP1904	*Desi*	Ca_Desi_Chr03	6499971	(A/T)
26310	CadSNP1905	*Desi*	Ca_Desi_Chr03	6499977	(A/G)
26311	CadSNP1906	*Desi*	Ca_Desi_Chr03	6499989	(A/T)
26312	CadSNP1907	*Desi*	Ca_Desi_Chr03	6499995	(A/G)
26313	CadSNP1908	*Desi*	Ca_Desi_Chr03	6500010	(A/G)
26314	CadSNP1909	*Desi*	Ca_Desi_Chr03	6500013	(A/C)
26315	CadSNP1910	*Desi*	Ca_Desi_Chr03	6500019	(A/G)
26316	CadSNP1911	*Desi*	Ca_Desi_Chr03	6500028	(A/G)
26317	CadSNP1912	*Desi*	Ca_Desi_Chr03	6501197	(A/T)
26318	CadSNP1913	*Desi*	Ca_Desi_Chr03	6501233	(T/A)
26319	CadSNP1914	*Desi*	Ca_Desi_Chr03	6501239	(G/A)
26320	CadSNP1915	*Desi*	Ca_Desi_Chr03	6501241	(T/C)
26321	CadSNP1916	*Desi*	Ca_Desi_Chr03	6501247	(G/A)
26322	CadSNP1917	*Desi*	Ca_Desi_Chr03	6501251	(C/T)
26323	CadSNP1918	*Desi*	Ca_Desi_Chr03	6501259	(T/C)
26324	CadSNP1919	*Desi*	Ca_Desi_Chr03	6501274	(C/A)
26325	CadSNP1920	*Desi*	Ca_Desi_Chr03	6501442	(C/T)
26326	CadSNP1921	*Desi*	Ca_Desi_Chr03	6501440	(T/A)
26327	CadSNP1922	*Desi*	Ca_Desi_Chr03	6501431	(G/T)
26328	CadSNP1923	*Desi*	Ca_Desi_Chr03	6501428	(C/T)
26329	CadSNP1924	*Desi*	Ca_Desi_Chr03	6501410	(A/G)
26330	CadSNP1925	*Desi*	Ca_Desi_Chr03	6501383	(G/A)
26331	CadSNP1926	*Desi*	Ca_Desi_Chr03	6501376	(C/G)
26332	CadSNP1927	*Desi*	Ca_Desi_Chr03	6501371	(G/A)
26333	CadSNP1928	*Desi*	Ca_Desi_Chr03	6501368	(T/G)

S.N.	SNP IDs	Cultivars	Chromosomes/scaffolds	Physical positions (bp)	SNPs
26334	CadSNP1929	*Desi*	Ca_Desi_Chr03	6543102	(A/G)
26335	CadSNP1930	*Desi*	Ca_Desi_Chr03	6543089	(A/T)
26336	CadSNP1931	*Desi*	Ca_Desi_Chr03	6543414	(C/T)
26337	CadSNP1932	*Desi*	Ca_Desi_Chr03	6544021	(T/A)
26338	CadSNP1933	*Desi*	Ca_Desi_Chr03	6544048	(C/T)
26339	CadSNP1934	*Desi*	Ca_Desi_Chr03	6544056	(A/G)
26340	CadSNP1935	*Desi*	Ca_Desi_Chr03	6544127	(G/A)
26341	CadSNP1936	*Desi*	Ca_Desi_Chr03	6547513	(A/G)
26342	CadSNP1937	*Desi*	Ca_Desi_Chr03	6547617	(C/A)
26343	CadSNP1938	*Desi*	Ca_Desi_Chr03	6592195	(A/G)
26344	CadSNP1939	*Desi*	Ca_Desi_Chr03	6593914	(G/A)
26345	CadSNP1940	*Desi*	Ca_Desi_Chr03	6593992	(A/G)
26346	CadSNP1941	*Desi*	Ca_Desi_Chr03	6636057	(C/T)
26347	CadSNP1942	*Desi*	Ca_Desi_Chr03	6641323	(G/A)
26348	CadSNP1943	*Desi*	Ca_Desi_Chr03	6721053	(T/A)
26349	CadSNP1944	*Desi*	Ca_Desi_Chr03	6723774	(T/A)
26350	CadSNP1945	*Desi*	Ca_Desi_Chr03	6723852	(C/G)
26351	CadSNP1946	*Desi*	Ca_Desi_Chr03	6723839	(G/A)
26352	CadSNP1947	*Desi*	Ca_Desi_Chr03	6723814	(A/G)
26353	CadSNP1948	*Desi*	Ca_Desi_Chr03	6738215	(A/T)
26354	CadSNP1949	*Desi*	Ca_Desi_Chr03	6739004	(A/G)
26355	CadSNP1950	*Desi*	Ca_Desi_Chr03	6738992	(G/A)
26356	CadSNP1951	*Desi*	Ca_Desi_Chr03	6758389	(T/C)
26357	CadSNP1952	*Desi*	Ca_Desi_Chr03	6758514	(T/C)
26358	CadSNP1953	*Desi*	Ca_Desi_Chr03	6758461	(C/T)
26359	CadSNP1954	*Desi*	Ca_Desi_Chr03	6776135	(G/A)
26360	CadSNP1955	*Desi*	Ca_Desi_Chr03	6776192	(A/G)
26361	CadSNP1956	*Desi*	Ca_Desi_Chr03	6776469	(T/C)
26362	CadSNP1957	*Desi*	Ca_Desi_Chr03	6776529	(C/T)
26363	CadSNP1958	*Desi*	Ca_Desi_Chr03	6790404	(G/A)
26364	CadSNP1959	*Desi*	Ca_Desi_Chr03	6790441	(T/G)
26365	CadSNP1960	*Desi*	Ca_Desi_Chr03	6790700	(G/A)
26366	CadSNP1961	*Desi*	Ca_Desi_Chr03	6790766	(G/T)
26367	CadSNP1962	*Desi*	Ca_Desi_Chr03	6799069	(G/A)
26368	CadSNP1963	*Desi*	Ca_Desi_Chr03	6799079	(G/C)
26369	CadSNP1964	*Desi*	Ca_Desi_Chr03	6799080	(A/G)
26370	CadSNP1965	*Desi*	Ca_Desi_Chr03	6799092	(A/T)
26371	CadSNP1966	*Desi*	Ca_Desi_Chr03	6799102	(C/T)

S.N.	SNP IDs	Cultivars	Chromosomes/scaffolds	Physical positions (bp)	SNPs
26372	CadSNP1967	*Desi*	Ca_Desi_Chr03	6799103	(C/T)
26373	CadSNP1968	*Desi*	Ca_Desi_Chr03	6799104	(G/C)
26374	CadSNP1969	*Desi*	Ca_Desi_Chr03	6799108	(A/G)
26375	CadSNP1970	*Desi*	Ca_Desi_Chr03	6799122	(T/C)
26376	CadSNP1971	*Desi*	Ca_Desi_Chr03	6799206	(G/A)
26377	CadSNP1972	*Desi*	Ca_Desi_Chr03	6799167	(T/C)
26378	CadSNP1973	*Desi*	Ca_Desi_Chr03	6812141	(C/T)
26379	CadSNP1974	*Desi*	Ca_Desi_Chr03	6812138	(C/A)
26380	CadSNP1975	*Desi*	Ca_Desi_Chr03	6812125	(C/T)
26381	CadSNP1976	*Desi*	Ca_Desi_Chr03	6812111	(A/G)
26382	CadSNP1977	*Desi*	Ca_Desi_Chr03	6812104	(C/G)
26383	CadSNP1978	*Desi*	Ca_Desi_Chr03	6812091	(G/A)
26384	CadSNP1979	*Desi*	Ca_Desi_Chr03	6812090	(C/T)
26385	CadSNP1980	*Desi*	Ca_Desi_Chr03	6812089	(G/T)
26386	CadSNP1981	*Desi*	Ca_Desi_Chr03	6812085	(G/A)
26387	CadSNP1982	*Desi*	Ca_Desi_Chr03	6812195	(C/T)
26388	CadSNP1983	*Desi*	Ca_Desi_Chr03	6812249	(G/A)
26389	CadSNP1984	*Desi*	Ca_Desi_Chr03	6812306	(A/C)
26390	CadSNP1985	*Desi*	Ca_Desi_Chr03	6812334	(G/A)
26391	CadSNP1986	*Desi*	Ca_Desi_Chr03	6833787	(G/A)
26392	CadSNP1987	*Desi*	Ca_Desi_Chr03	6833812	(C/A)
26393	CadSNP1988	*Desi*	Ca_Desi_Chr03	6833831	(A/G)
26394	CadSNP1989	*Desi*	Ca_Desi_Chr03	6833840	(G/T)
26395	CadSNP1990	*Desi*	Ca_Desi_Chr03	7012818	(T/C)
26396	CadSNP1991	*Desi*	Ca_Desi_Chr03	7012976	(A/G)
26397	CadSNP1992	*Desi*	Ca_Desi_Chr03	7059071	(G/A)
26398	CadSNP1993	*Desi*	Ca_Desi_Chr03	7059090	(A/G)
26399	CadSNP1994	*Desi*	Ca_Desi_Chr03	7059208	(T/C)
26400	CadSNP1995	*Desi*	Ca_Desi_Chr03	7101708	(G/T)
26401	CadSNP1996	*Desi*	Ca_Desi_Chr03	7101702	(T/G)
26402	CadSNP1997	*Desi*	Ca_Desi_Chr03	7104055	(G/A)
26403	CadSNP1998	*Desi*	Ca_Desi_Chr03	7104519	(C/T)
26404	CadSNP1999	*Desi*	Ca_Desi_Chr03	7104579	(T/A)
26405	CadSNP2000	*Desi*	Ca_Desi_Chr03	7144144	(T/A)
26406	CadSNP2001	*Desi*	Ca_Desi_Chr03	7144200	(T/G)
26407	CadSNP2002	*Desi*	Ca_Desi_Chr03	7177530	(C/A)
26408	CadSNP2003	*Desi*	Ca_Desi_Chr03	7207213	(C/G)
26409	CadSNP2004	*Desi*	Ca_Desi_Chr03	7207239	(G/T)

S.N.	SNP IDs	Cultivars	Chromosomes/scaffolds	Physical positions (bp)	SNPs
26410	CadSNP2005	*Desi*	Ca_Desi_Chr03	7207240	(T/G)
26411	CadSNP2006	*Desi*	Ca_Desi_Chr03	7207270	(T/C)
26412	CadSNP2007	*Desi*	Ca_Desi_Chr03	7207273	(A/G)
26413	CadSNP2008	*Desi*	Ca_Desi_Chr03	7207388	(A/C)
26414	CadSNP2009	*Desi*	Ca_Desi_Chr03	7207370	(C/T)
26415	CadSNP2010	*Desi*	Ca_Desi_Chr03	7207355	(A/G)
26416	CadSNP2011	*Desi*	Ca_Desi_Chr03	7207306	(T/G)
26417	CadSNP2012	*Desi*	Ca_Desi_Chr03	7212795	(T/C)
26418	CadSNP2013	*Desi*	Ca_Desi_Chr03	7212796	(T/C)
26419	CadSNP2014	*Desi*	Ca_Desi_Chr03	7218359	(A/C)
26420	CadSNP2015	*Desi*	Ca_Desi_Chr03	7234381	(A/T)
26421	CadSNP2016	*Desi*	Ca_Desi_Chr03	7241614	(G/A)
26422	CadSNP2017	*Desi*	Ca_Desi_Chr03	7241639	(A/G)
26423	CadSNP2018	*Desi*	Ca_Desi_Chr03	7241675	(G/T)
26424	CadSNP2019	*Desi*	Ca_Desi_Chr03	8455273	(A/T)
26425	CadSNP2020	*Desi*	Ca_Desi_Chr03	8456386	(C/A)
26426	CadSNP2021	*Desi*	Ca_Desi_Chr03	8499689	(C/T)
26427	CadSNP2022	*Desi*	Ca_Desi_Chr03	8540339	(T/A)
26428	CadSNP2023	*Desi*	Ca_Desi_Chr03	8540535	(T/C)
26429	CadSNP2024	*Desi*	Ca_Desi_Chr03	8548425	(C/T)
26430	CadSNP2025	*Desi*	Ca_Desi_Chr03	8556886	(G/T)
26431	CadSNP2026	*Desi*	Ca_Desi_Chr03	8556931	(G/T)
26432	CadSNP2027	*Desi*	Ca_Desi_Chr03	8814590	(A/G)
26433	CadSNP2028	*Desi*	Ca_Desi_Chr03	8815626	(A/T)
26434	CadSNP2029	*Desi*	Ca_Desi_Chr03	8815663	(C/T)
26435	CadSNP2030	*Desi*	Ca_Desi_Chr03	8815664	(G/A)
26436	CadSNP2031	*Desi*	Ca_Desi_Chr03	8815694	(G/A)
26437	CadSNP2032	*Desi*	Ca_Desi_Chr03	8826351	(G/C)
26438	CadSNP2033	*Desi*	Ca_Desi_Chr03	8826641	(A/G)
26439	CadSNP2034	*Desi*	Ca_Desi_Chr03	8828383	(C/T)
26440	CadSNP2035	*Desi*	Ca_Desi_Chr03	8844120	(T/C)
26441	CadSNP2036	*Desi*	Ca_Desi_Chr03	8844165	(G/A)
26442	CadSNP2037	*Desi*	Ca_Desi_Chr03	8844171	(C/T)
26443	CadSNP2038	*Desi*	Ca_Desi_Chr03	8844245	(C/A)
26444	CadSNP2039	*Desi*	Ca_Desi_Chr03	8852584	(C/T)
26445	CadSNP2040	*Desi*	Ca_Desi_Chr03	8907433	(T/G)
26446	CadSNP2041	*Desi*	Ca_Desi_Chr03	8907468	(T/C)
26447	CadSNP2042	*Desi*	Ca_Desi_Chr03	8907459	(A/G)

S.N.	SNP IDs	Cultivars	Chromosomes/scaffolds	Physical positions (bp)	SNPs
26448	CadSNP2043	*Desi*	Ca_Desi_Chr03	8907661	(C/T)
26449	CadSNP2044	*Desi*	Ca_Desi_Chr03	8989825	(A/T)
26450	CadSNP2045	*Desi*	Ca_Desi_Chr03	9852607	(T/A)
26451	CadSNP2046	*Desi*	Ca_Desi_Chr03	9934450	(C/A)
26452	CadSNP2047	*Desi*	Ca_Desi_Chr03	9934613	(A/G)
26453	CadSNP2048	*Desi*	Ca_Desi_Chr03	9938011	(A/T)
26454	CadSNP2049	*Desi*	Ca_Desi_Chr03	10075249	(G/A)
26455	CadSNP2050	*Desi*	Ca_Desi_Chr03	10078325	(A/C)
26456	CadSNP2051	*Desi*	Ca_Desi_Chr03	10247487	(C/G)
26457	CadSNP2052	*Desi*	Ca_Desi_Chr03	10304204	(T/A)
26458	CadSNP2053	*Desi*	Ca_Desi_Chr03	10304224	(A/T)
26459	CadSNP2054	*Desi*	Ca_Desi_Chr03	10304244	(C/A)
26460	CadSNP2055	*Desi*	Ca_Desi_Chr03	10308956	(A/C)
26461	CadSNP2056	*Desi*	Ca_Desi_Chr03	10349421	(A/G)
26462	CadSNP2057	*Desi*	Ca_Desi_Chr03	10350151	(C/T)
26463	CadSNP2058	*Desi*	Ca_Desi_Chr03	10350133	(G/T)
26464	CadSNP2059	*Desi*	Ca_Desi_Chr03	10387956	(A/G)
26465	CadSNP2060	*Desi*	Ca_Desi_Chr03	10388061	(T/C)
26466	CadSNP2061	*Desi*	Ca_Desi_Chr03	10389534	(T/G)
26467	CadSNP2062	*Desi*	Ca_Desi_Chr03	10413004	(A/C)
26468	CadSNP2063	*Desi*	Ca_Desi_Chr03	10468011	(C/T)
26469	CadSNP2064	*Desi*	Ca_Desi_Chr03	10509285	(A/T)
26470	CadSNP2065	*Desi*	Ca_Desi_Chr03	10509304	(T/C)
26471	CadSNP2066	*Desi*	Ca_Desi_Chr03	10509352	(C/A)
26472	CadSNP2067	*Desi*	Ca_Desi_Chr03	10509908	(T/G)
26473	CadSNP2068	*Desi*	Ca_Desi_Chr03	10509938	(A/C)
26474	CadSNP2069	*Desi*	Ca_Desi_Chr03	10597811	(T/G)
26475	CadSNP2070	*Desi*	Ca_Desi_Chr03	10597847	(C/T)
26476	CadSNP2071	*Desi*	Ca_Desi_Chr03	10641927	(G/A)
26477	CadSNP2072	*Desi*	Ca_Desi_Chr03	10656917	(G/A)
26478	CadSNP2073	*Desi*	Ca_Desi_Chr03	10676655	(G/A)
26479	CadSNP2074	*Desi*	Ca_Desi_Chr03	10676659	(G/A)
26480	CadSNP2075	*Desi*	Ca_Desi_Chr03	10811691	(G/T)
26481	CadSNP2076	*Desi*	Ca_Desi_Chr03	10811721	(G/C)
26482	CadSNP2077	*Desi*	Ca_Desi_Chr03	10812216	(G/A)
26483	CadSNP2078	*Desi*	Ca_Desi_Chr03	10832501	(T/G)
26484	CadSNP2079	*Desi*	Ca_Desi_Chr03	10869140	(G/A)
26485	CadSNP2080	*Desi*	Ca_Desi_Chr03	10881083	(C/T)

S.N.	SNP IDs	Cultivars	Chromosomes/scaffolds	Physical positions (bp)	SNPs
26486	CadSNP2081	*Desi*	Ca_Desi_Chr03	10915073	(G/A)
26487	CadSNP2082	*Desi*	Ca_Desi_Chr03	11064391	(C/G)
26488	CadSNP2083	*Desi*	Ca_Desi_Chr03	11115658	(G/A)
26489	CadSNP2084	*Desi*	Ca_Desi_Chr03	11121805	(G/T)
26490	CadSNP2085	*Desi*	Ca_Desi_Chr03	11121776	(T/C)
26491	CadSNP2086	*Desi*	Ca_Desi_Chr03	11146489	(T/C)
26492	CadSNP2087	*Desi*	Ca_Desi_Chr03	11146442	(A/C)
26493	CadSNP2088	*Desi*	Ca_Desi_Chr03	11147829	(G/C)
26494	CadSNP2089	*Desi*	Ca_Desi_Chr03	11147853	(T/G)
26495	CadSNP2090	*Desi*	Ca_Desi_Chr03	11148357	(C/T)
26496	CadSNP2091	*Desi*	Ca_Desi_Chr03	11163988	(C/T)
26497	CadSNP2092	*Desi*	Ca_Desi_Chr03	11220397	(T/C)
26498	CadSNP2093	*Desi*	Ca_Desi_Chr03	11223968	(C/G)
26499	CadSNP2094	*Desi*	Ca_Desi_Chr03	11251354	(C/T)
26500	CadSNP2095	*Desi*	Ca_Desi_Chr03	11251333	(A/C)
26501	CadSNP2096	*Desi*	Ca_Desi_Chr03	11295835	(C/T)
26502	CadSNP2097	*Desi*	Ca_Desi_Chr03	11330443	(T/G)
26503	CadSNP2098	*Desi*	Ca_Desi_Chr03	11370420	(G/A)
26504	CadSNP2099	*Desi*	Ca_Desi_Chr03	11390003	(A/G)
26505	CadSNP2100	*Desi*	Ca_Desi_Chr03	11390004	(T/C)
26506	CadSNP2101	*Desi*	Ca_Desi_Chr03	11390007	(C/T)
26507	CadSNP2102	*Desi*	Ca_Desi_Chr03	11390016	(C/A)
26508	CadSNP2103	*Desi*	Ca_Desi_Chr03	11390049	(A/G)
26509	CadSNP2104	*Desi*	Ca_Desi_Chr03	11390057	(A/G)
26510	CadSNP2105	*Desi*	Ca_Desi_Chr03	11390970	(A/C)
26511	CadSNP2106	*Desi*	Ca_Desi_Chr03	11392062	(A/G)
26512	CadSNP2107	*Desi*	Ca_Desi_Chr03	11392061	(C/T)
26513	CadSNP2108	*Desi*	Ca_Desi_Chr03	11392059	(G/A)
26514	CadSNP2109	*Desi*	Ca_Desi_Chr03	11392056	(G/A)
26515	CadSNP2110	*Desi*	Ca_Desi_Chr03	11392049	(A/G)
26516	CadSNP2111	*Desi*	Ca_Desi_Chr03	11392045	(T/A)
26517	CadSNP2112	*Desi*	Ca_Desi_Chr03	11392037	(T/C)
26518	CadSNP2113	*Desi*	Ca_Desi_Chr03	11392024	(T/C)
26519	CadSNP2114	*Desi*	Ca_Desi_Chr03	11396406	(A/C)
26520	CadSNP2115	*Desi*	Ca_Desi_Chr03	11396407	(T/A)
26521	CadSNP2116	*Desi*	Ca_Desi_Chr03	11400666	(A/C)
26522	CadSNP2117	*Desi*	Ca_Desi_Chr03	11404484	(C/G)
26523	CadSNP2118	*Desi*	Ca_Desi_Chr03	11404536	(G/A)

S.N.	SNP IDs	Cultivars	Chromosomes/scaffolds	Physical positions (bp)	SNPs
26524	CadSNP2119	*Desi*	Ca_Desi_Chr03	11418718	(G/A)
26525	CadSNP2120	*Desi*	Ca_Desi_Chr03	11418716	(T/C)
26526	CadSNP2121	*Desi*	Ca_Desi_Chr03	11418710	(C/A)
26527	CadSNP2122	*Desi*	Ca_Desi_Chr03	11418707	(T/A)
26528	CadSNP2123	*Desi*	Ca_Desi_Chr03	11418699	(T/C)
26529	CadSNP2124	*Desi*	Ca_Desi_Chr03	11432450	(G/A)
26530	CadSNP2125	*Desi*	Ca_Desi_Chr03	11437660	(A/C)
26531	CadSNP2126	*Desi*	Ca_Desi_Chr03	11444564	(A/C)
26532	CadSNP2127	*Desi*	Ca_Desi_Chr03	11457594	(C/G)
26533	CadSNP2128	*Desi*	Ca_Desi_Chr03	11457591	(G/C)
26534	CadSNP2129	*Desi*	Ca_Desi_Chr03	11457652	(A/C)
26535	CadSNP2130	*Desi*	Ca_Desi_Chr03	11497356	(C/T)
26536	CadSNP2131	*Desi*	Ca_Desi_Chr03	11547118	(A/G)
26537	CadSNP2132	*Desi*	Ca_Desi_Chr03	11548485	(G/A)
26538	CadSNP2133	*Desi*	Ca_Desi_Chr03	11567306	(G/A)
26539	CadSNP2134	*Desi*	Ca_Desi_Chr03	11567364	(A/C)
26540	CadSNP2135	*Desi*	Ca_Desi_Chr03	11569456	(C/T)
26541	CadSNP2136	*Desi*	Ca_Desi_Chr03	11599239	(G/A)
26542	CadSNP2137	*Desi*	Ca_Desi_Chr03	11599240	(A/T)
26543	CadSNP2138	*Desi*	Ca_Desi_Chr03	11607367	(T/C)
26544	CadSNP2139	*Desi*	Ca_Desi_Chr03	11624139	(T/C)
26545	CadSNP2140	*Desi*	Ca_Desi_Chr03	11625131	(C/T)
26546	CadSNP2141	*Desi*	Ca_Desi_Chr03	11625124	(G/A)
26547	CadSNP2142	*Desi*	Ca_Desi_Chr03	11656953	(A/G)
26548	CadSNP2143	*Desi*	Ca_Desi_Chr03	11656922	(T/G)
26549	CadSNP2144	*Desi*	Ca_Desi_Chr03	11676785	(A/G)
26550	CadSNP2145	*Desi*	Ca_Desi_Chr03	11676764	(A/G)
26551	CadSNP2146	*Desi*	Ca_Desi_Chr03	11722037	(T/C)
26552	CadSNP2147	*Desi*	Ca_Desi_Chr03	11728702	(A/C)
26553	CadSNP2148	*Desi*	Ca_Desi_Chr03	11728698	(A/G)
26554	CadSNP2149	*Desi*	Ca_Desi_Chr03	11740570	(C/T)
26555	CadSNP2150	*Desi*	Ca_Desi_Chr03	11740579	(T/C)
26556	CadSNP2151	*Desi*	Ca_Desi_Chr03	11740600	(C/A)
26557	CadSNP2152	*Desi*	Ca_Desi_Chr03	11740672	(C/T)
26558	CadSNP2153	*Desi*	Ca_Desi_Chr03	11740669	(A/G)
26559	CadSNP2154	*Desi*	Ca_Desi_Chr03	11748297	(T/A)
26560	CadSNP2155	*Desi*	Ca_Desi_Chr03	11781845	(C/T)
26561	CadSNP2156	*Desi*	Ca_Desi_Chr03	11782814	(G/T)

S.N.	SNP IDs	Cultivars	Chromosomes/scaffolds	Physical positions (bp)	SNPs
26562	CadSNP2157	*Desi*	Ca_Desi_Chr03	11783341	(C/A)
26563	CadSNP2158	*Desi*	Ca_Desi_Chr03	11783336	(C/A)
26564	CadSNP2159	*Desi*	Ca_Desi_Chr03	11783335	(G/A)
26565	CadSNP2160	*Desi*	Ca_Desi_Chr03	11783334	(T/C)
26566	CadSNP2161	*Desi*	Ca_Desi_Chr03	11783331	(C/T)
26567	CadSNP2162	*Desi*	Ca_Desi_Chr03	11783328	(C/T)
26568	CadSNP2163	*Desi*	Ca_Desi_Chr03	11828957	(C/A)
26569	CadSNP2164	*Desi*	Ca_Desi_Chr03	11900779	(G/A)
26570	CadSNP2165	*Desi*	Ca_Desi_Chr03	11900840	(T/C)
26571	CadSNP2166	*Desi*	Ca_Desi_Chr03	11900841	(G/A)
26572	CadSNP2167	*Desi*	Ca_Desi_Chr03	11900930	(T/A)
26573	CadSNP2168	*Desi*	Ca_Desi_Chr03	11900889	(A/G)
26574	CadSNP2169	*Desi*	Ca_Desi_Chr03	11900887	(T/C)
26575	CadSNP2170	*Desi*	Ca_Desi_Chr03	11974197	(T/C)
26576	CadSNP2171	*Desi*	Ca_Desi_Chr03	11974244	(A/T)
26577	CadSNP2172	*Desi*	Ca_Desi_Chr03	11974241	(G/T)
26578	CadSNP2173	*Desi*	Ca_Desi_Chr03	11974238	(A/G)
26579	CadSNP2174	*Desi*	Ca_Desi_Chr03	12051872	(G/C)
26580	CadSNP2175	*Desi*	Ca_Desi_Chr03	12051878	(A/C)
26581	CadSNP2176	*Desi*	Ca_Desi_Chr03	12095970	(G/T)
26582	CadSNP2177	*Desi*	Ca_Desi_Chr03	12096007	(C/T)
26583	CadSNP2178	*Desi*	Ca_Desi_Chr03	12096018	(G/T)
26584	CadSNP2179	*Desi*	Ca_Desi_Chr03	12163332	(T/C)
26585	CadSNP2180	*Desi*	Ca_Desi_Chr03	12163323	(G/A)
26586	CadSNP2181	*Desi*	Ca_Desi_Chr03	12277447	(C/G)
26587	CadSNP2182	*Desi*	Ca_Desi_Chr03	12277580	(A/G)
26588	CadSNP2183	*Desi*	Ca_Desi_Chr03	12277558	(A/G)
26589	CadSNP2184	*Desi*	Ca_Desi_Chr03	12353499	(A/G)
26590	CadSNP2185	*Desi*	Ca_Desi_Chr03	12354035	(A/G)
26591	CadSNP2186	*Desi*	Ca_Desi_Chr03	12385670	(G/T)
26592	CadSNP2187	*Desi*	Ca_Desi_Chr03	12386707	(A/G)
26593	CadSNP2188	*Desi*	Ca_Desi_Chr03	12387498	(G/T)
26594	CadSNP2189	*Desi*	Ca_Desi_Chr03	12388427	(T/C)
26595	CadSNP2190	*Desi*	Ca_Desi_Chr03	12388434	(C/T)
26596	CadSNP2191	*Desi*	Ca_Desi_Chr03	12389294	(G/A)
26597	CadSNP2192	*Desi*	Ca_Desi_Chr03	12389297	(G/A)
26598	CadSNP2193	*Desi*	Ca_Desi_Chr03	12389303	(C/T)
26599	CadSNP2194	*Desi*	Ca_Desi_Chr03	12399041	(C/T)

S.N.	SNP IDs	Cultivars	Chromosomes/scaffolds	Physical positions (bp)	SNPs
26600	CadSNP2195	*Desi*	Ca_Desi_Chr03	12399107	(T/A)
26601	CadSNP2196	*Desi*	Ca_Desi_Chr03	12401439	(C/G)
26602	CadSNP2197	*Desi*	Ca_Desi_Chr03	12401650	(T/C)
26603	CadSNP2198	*Desi*	Ca_Desi_Chr03	12460531	(T/C)
26604	CadSNP2199	*Desi*	Ca_Desi_Chr03	12472072	(G/A)
26605	CadSNP2200	*Desi*	Ca_Desi_Chr03	12498074	(G/A)
26606	CadSNP2201	*Desi*	Ca_Desi_Chr03	12535870	(T/C)
26607	CadSNP2202	*Desi*	Ca_Desi_Chr03	12535887	(T/C)
26608	CadSNP2203	*Desi*	Ca_Desi_Chr03	12535894	(C/T)
26609	CadSNP2204	*Desi*	Ca_Desi_Chr03	12535919	(G/A)
26610	CadSNP2205	*Desi*	Ca_Desi_Chr03	12543791	(G/A)
26611	CadSNP2206	*Desi*	Ca_Desi_Chr03	12543837	(A/C)
26612	CadSNP2207	*Desi*	Ca_Desi_Chr03	12863764	(C/T)
26613	CadSNP2208	*Desi*	Ca_Desi_Chr03	12863779	(T/C)
26614	CadSNP2209	*Desi*	Ca_Desi_Chr03	12863789	(C/A)
26615	CadSNP2210	*Desi*	Ca_Desi_Chr03	12863810	(C/T)
26616	CadSNP2211	*Desi*	Ca_Desi_Chr03	12863812	(C/T)
26617	CadSNP2212	*Desi*	Ca_Desi_Chr03	12897333	(T/A)
26618	CadSNP2213	*Desi*	Ca_Desi_Chr03	12897345	(C/G)
26619	CadSNP2214	*Desi*	Ca_Desi_Chr03	12930500	(C/A)
26620	CadSNP2215	*Desi*	Ca_Desi_Chr03	12930626	(C/T)
26621	CadSNP2216	*Desi*	Ca_Desi_Chr03	12979506	(T/C)
26622	CadSNP2217	*Desi*	Ca_Desi_Chr03	12979551	(C/A)
26623	CadSNP2218	*Desi*	Ca_Desi_Chr03	13008395	(A/C)
26624	CadSNP2219	*Desi*	Ca_Desi_Chr03	13191097	(A/T)
26625	CadSNP2220	*Desi*	Ca_Desi_Chr03	13191072	(C/G)
26626	CadSNP2221	*Desi*	Ca_Desi_Chr03	13191049	(A/G)
26627	CadSNP2222	*Desi*	Ca_Desi_Chr03	13207036	(C/A)
26628	CadSNP2223	*Desi*	Ca_Desi_Chr03	13208922	(T/G)
26629	CadSNP2224	*Desi*	Ca_Desi_Chr03	13407432	(T/A)
26630	CadSNP2225	*Desi*	Ca_Desi_Chr03	13407459	(T/G)
26631	CadSNP2226	*Desi*	Ca_Desi_Chr03	13423127	(T/G)
26632	CadSNP2227	*Desi*	Ca_Desi_Chr03	13517775	(T/C)
26633	CadSNP2228	*Desi*	Ca_Desi_Chr03	13517888	(C/T)
26634	CadSNP2229	*Desi*	Ca_Desi_Chr03	13518296	(A/G)
26635	CadSNP2230	*Desi*	Ca_Desi_Chr03	13538576	(A/C)
26636	CadSNP2231	*Desi*	Ca_Desi_Chr03	13664273	(G/A)
26637	CadSNP2232	*Desi*	Ca_Desi_Chr03	13664267	(A/G)

**S.N.**	**SNP IDs**	**Cultivars**	**Chromosomes/scaffolds**	**Physical positions (bp)**	**SNPs**
26638	CadSNP2233	*Desi*	Ca_Desi_Chr03	13664213	(A/C)
26639	CadSNP2234	*Desi*	Ca_Desi_Chr03	13825261	(G/A)
26640	CadSNP2235	*Desi*	Ca_Desi_Chr03	13839597	(C/T)
26641	CadSNP2236	*Desi*	Ca_Desi_Chr03	13841817	(T/C)
26642	CadSNP2237	*Desi*	Ca_Desi_Chr03	13841895	(A/C)
26643	CadSNP2238	*Desi*	Ca_Desi_Chr03	13866595	(G/T)
26644	CadSNP2239	*Desi*	Ca_Desi_Chr03	13892242	(T/C)
26645	CadSNP2240	*Desi*	Ca_Desi_Chr03	13894638	(T/A)
26646	CadSNP2241	*Desi*	Ca_Desi_Chr03	13909204	(C/T)
26647	CadSNP2242	*Desi*	Ca_Desi_Chr03	13909208	(A/C)
26648	CadSNP2243	*Desi*	Ca_Desi_Chr03	13909226	(T/C)
26649	CadSNP2244	*Desi*	Ca_Desi_Chr03	13909264	(A/T)
26650	CadSNP2245	*Desi*	Ca_Desi_Chr03	13994137	(A/C)
26651	CadSNP2246	*Desi*	Ca_Desi_Chr03	14005265	(C/T)
26652	CadSNP2247	*Desi*	Ca_Desi_Chr03	14005543	(C/G)
26653	CadSNP2248	*Desi*	Ca_Desi_Chr03	14005497	(T/C)
26654	CadSNP2249	*Desi*	Ca_Desi_Chr03	14006868	(G/A)
26655	CadSNP2250	*Desi*	Ca_Desi_Chr03	14006814	(T/G)
26656	CadSNP2251	*Desi*	Ca_Desi_Chr03	14172661	(C/T)
26657	CadSNP2252	*Desi*	Ca_Desi_Chr03	14172702	(G/A)
26658	CadSNP2253	*Desi*	Ca_Desi_Chr03	14469822	(G/A)
26659	CadSNP2254	*Desi*	Ca_Desi_Chr03	14469811	(T/C)
26660	CadSNP2255	*Desi*	Ca_Desi_Chr03	14469806	(A/G)
26661	CadSNP2256	*Desi*	Ca_Desi_Chr03	14469801	(G/A)
26662	CadSNP2257	*Desi*	Ca_Desi_Chr03	14469790	(C/A)
26663	CadSNP2258	*Desi*	Ca_Desi_Chr03	14469745	(A/T)
26664	CadSNP2259	*Desi*	Ca_Desi_Chr03	14469761	(G/A)
26665	CadSNP2260	*Desi*	Ca_Desi_Chr03	14490484	(C/T)
26666	CadSNP2261	*Desi*	Ca_Desi_Chr03	14619725	(G/A)
26667	CadSNP2262	*Desi*	Ca_Desi_Chr03	14672190	(A/G)
26668	CadSNP2263	*Desi*	Ca_Desi_Chr03	14694727	(A/G)
26669	CadSNP2264	*Desi*	Ca_Desi_Chr03	14702839	(C/T)
26670	CadSNP2265	*Desi*	Ca_Desi_Chr03	14732794	(T/C)
26671	CadSNP2266	*Desi*	Ca_Desi_Chr03	14733079	(G/C)
26672	CadSNP2267	*Desi*	Ca_Desi_Chr03	14734975	(A/C)
26673	CadSNP2268	*Desi*	Ca_Desi_Chr03	14790196	(C/T)
26674	CadSNP2269	*Desi*	Ca_Desi_Chr03	14817852	(G/A)
26675	CadSNP2270	*Desi*	Ca_Desi_Chr03	14828680	(T/A)

S.N.	SNP IDs	Cultivars	Chromosomes/scaffolds	Physical positions (bp)	SNPs
26676	CadSNP2271	*Desi*	Ca_Desi_Chr03	14828628	(T/C)
26677	CadSNP2272	*Desi*	Ca_Desi_Chr03	14860205	(G/T)
26678	CadSNP2273	*Desi*	Ca_Desi_Chr03	14860342	(T/C)
26679	CadSNP2274	*Desi*	Ca_Desi_Chr03	14890519	(G/A)
26680	CadSNP2275	*Desi*	Ca_Desi_Chr03	14890526	(A/G)
26681	CadSNP2276	*Desi*	Ca_Desi_Chr03	14890671	(G/T)
26682	CadSNP2277	*Desi*	Ca_Desi_Chr03	14890670	(T/A)
26683	CadSNP2278	*Desi*	Ca_Desi_Chr03	14890662	(T/G)
26684	CadSNP2279	*Desi*	Ca_Desi_Chr03	14890652	(G/A)
26685	CadSNP2280	*Desi*	Ca_Desi_Chr03	14890643	(C/A)
26686	CadSNP2281	*Desi*	Ca_Desi_Chr03	14890606	(G/T)
26687	CadSNP2282	*Desi*	Ca_Desi_Chr03	14890599	(G/T)
26688	CadSNP2283	*Desi*	Ca_Desi_Chr03	14927251	(G/A)
26689	CadSNP2284	*Desi*	Ca_Desi_Chr03	15066955	(A/G)
26690	CadSNP2285	*Desi*	Ca_Desi_Chr03	15101360	(G/A)
26691	CadSNP2286	*Desi*	Ca_Desi_Chr03	15101362	(G/T)
26692	CadSNP2287	*Desi*	Ca_Desi_Chr03	15101368	(A/G)
26693	CadSNP2288	*Desi*	Ca_Desi_Chr03	15101369	(G/C)
26694	CadSNP2289	*Desi*	Ca_Desi_Chr03	15101397	(G/A)
26695	CadSNP2290	*Desi*	Ca_Desi_Chr03	15107917	(T/C)
26696	CadSNP2291	*Desi*	Ca_Desi_Chr03	15115080	(G/A)
26697	CadSNP2292	*Desi*	Ca_Desi_Chr03	15115083	(T/C)
26698	CadSNP2293	*Desi*	Ca_Desi_Chr03	15115094	(C/T)
26699	CadSNP2294	*Desi*	Ca_Desi_Chr03	15115099	(A/T)
26700	CadSNP2295	*Desi*	Ca_Desi_Chr03	15115111	(C/T)
26701	CadSNP2296	*Desi*	Ca_Desi_Chr03	15115114	(A/T)
26702	CadSNP2297	*Desi*	Ca_Desi_Chr03	15115115	(C/T)
26703	CadSNP2298	*Desi*	Ca_Desi_Chr03	15115122	(A/T)
26704	CadSNP2299	*Desi*	Ca_Desi_Chr03	15115126	(T/C)
26705	CadSNP2300	*Desi*	Ca_Desi_Chr03	15115128	(G/T)
26706	CadSNP2301	*Desi*	Ca_Desi_Chr03	15115141	(T/A)
26707	CadSNP2302	*Desi*	Ca_Desi_Chr03	15134512	(A/G)
26708	CadSNP2303	*Desi*	Ca_Desi_Chr03	15134527	(T/C)
26709	CadSNP2304	*Desi*	Ca_Desi_Chr03	15134699	(G/A)
26710	CadSNP2305	*Desi*	Ca_Desi_Chr03	15140391	(A/G)
26711	CadSNP2306	*Desi*	Ca_Desi_Chr03	15140451	(C/G)
26712	CadSNP2307	*Desi*	Ca_Desi_Chr03	15140454	(A/G)
26713	CadSNP2308	*Desi*	Ca_Desi_Chr03	15206796	(A/T)

S.N.	SNP IDs	Cultivars	Chromosomes/scaffolds	Physical positions (bp)	SNPs
26714	CadSNP2309	*Desi*	Ca_Desi_Chr03	15281781	(C/T)
26715	CadSNP2310	*Desi*	Ca_Desi_Chr03	15281745	(T/C)
26716	CadSNP2311	*Desi*	Ca_Desi_Chr03	15332475	(C/G)
26717	CadSNP2312	*Desi*	Ca_Desi_Chr03	15332490	(C/G)
26718	CadSNP2313	*Desi*	Ca_Desi_Chr03	15332493	(C/A)
26719	CadSNP2314	*Desi*	Ca_Desi_Chr03	15332539	(G/A)
26720	CadSNP2315	*Desi*	Ca_Desi_Chr03	15376304	(G/C)
26721	CadSNP2316	*Desi*	Ca_Desi_Chr03	15385992	(G/C)
26722	CadSNP2317	*Desi*	Ca_Desi_Chr03	15386093	(A/C)
26723	CadSNP2318	*Desi*	Ca_Desi_Chr03	15386074	(T/C)
26724	CadSNP2319	*Desi*	Ca_Desi_Chr03	15411640	(A/T)
26725	CadSNP2320	*Desi*	Ca_Desi_Chr03	15411677	(A/G)
26726	CadSNP2321	*Desi*	Ca_Desi_Chr03	15412922	(A/C)
26727	CadSNP2322	*Desi*	Ca_Desi_Chr03	15445856	(A/G)
26728	CadSNP2323	*Desi*	Ca_Desi_Chr03	15449309	(T/C)
26729	CadSNP2324	*Desi*	Ca_Desi_Chr03	15458600	(C/T)
26730	CadSNP2325	*Desi*	Ca_Desi_Chr03	15459712	(T/G)
26731	CadSNP2326	*Desi*	Ca_Desi_Chr03	15459709	(T/C)
26732	CadSNP2327	*Desi*	Ca_Desi_Chr03	15466277	(A/G)
26733	CadSNP2328	*Desi*	Ca_Desi_Chr03	15487859	(A/T)
26734	CadSNP2329	*Desi*	Ca_Desi_Chr03	15492126	(A/G)
26735	CadSNP2330	*Desi*	Ca_Desi_Chr03	15497431	(A/G)
26736	CadSNP2331	*Desi*	Ca_Desi_Chr03	15502488	(C/T)
26737	CadSNP2332	*Desi*	Ca_Desi_Chr03	15506011	(A/G)
26738	CadSNP2333	*Desi*	Ca_Desi_Chr03	15505991	(C/T)
26739	CadSNP2334	*Desi*	Ca_Desi_Chr03	15505987	(C/A)
26740	CadSNP2335	*Desi*	Ca_Desi_Chr03	15505983	(C/A)
26741	CadSNP2336	*Desi*	Ca_Desi_Chr03	15813048	(C/T)
26742	CadSNP2337	*Desi*	Ca_Desi_Chr03	15825478	(G/A)
26743	CadSNP2338	*Desi*	Ca_Desi_Chr03	15828104	(C/A)
26744	CadSNP2339	*Desi*	Ca_Desi_Chr03	15835391	(T/A)
26745	CadSNP2340	*Desi*	Ca_Desi_Chr03	15875198	(A/G)
26746	CadSNP2341	*Desi*	Ca_Desi_Chr03	15877087	(A/G)
26747	CadSNP2342	*Desi*	Ca_Desi_Chr03	15877222	(T/G)
26748	CadSNP2343	*Desi*	Ca_Desi_Chr03	15877185	(A/G)
26749	CadSNP2344	*Desi*	Ca_Desi_Chr03	15879088	(T/G)
26750	CadSNP2345	*Desi*	Ca_Desi_Chr03	15885833	(G/T)
26751	CadSNP2346	*Desi*	Ca_Desi_Chr03	15885849	(G/T)

S.N.	SNP IDs	Cultivars	Chromosomes/scaffolds	Physical positions (bp)	SNPs
26752	CadSNP2347	*Desi*	Ca_Desi_Chr03	15946686	(T/G)
26753	CadSNP2348	*Desi*	Ca_Desi_Chr03	15946765	(G/C)
26754	CadSNP2349	*Desi*	Ca_Desi_Chr03	15991427	(C/T)
26755	CadSNP2350	*Desi*	Ca_Desi_Chr03	15991589	(T/C)
26756	CadSNP2351	*Desi*	Ca_Desi_Chr03	16001126	(T/A)
26757	CadSNP2352	*Desi*	Ca_Desi_Chr03	16050465	(G/T)
26758	CadSNP2353	*Desi*	Ca_Desi_Chr03	16050429	(G/A)
26759	CadSNP2354	*Desi*	Ca_Desi_Chr03	16104825	(G/T)
26760	CadSNP2355	*Desi*	Ca_Desi_Chr03	16108691	(A/G)
26761	CadSNP2356	*Desi*	Ca_Desi_Chr03	16137168	(G/C)
26762	CadSNP2357	*Desi*	Ca_Desi_Chr03	16139116	(C/T)
26763	CadSNP2358	*Desi*	Ca_Desi_Chr03	16145409	(T/C)
26764	CadSNP2359	*Desi*	Ca_Desi_Chr03	16145494	(C/T)
26765	CadSNP2360	*Desi*	Ca_Desi_Chr03	16145469	(T/A)
26766	CadSNP2361	*Desi*	Ca_Desi_Chr03	16181589	(G/C)
26767	CadSNP2362	*Desi*	Ca_Desi_Chr03	16181578	(G/A)
26768	CadSNP2363	*Desi*	Ca_Desi_Chr03	16209416	(G/A)
26769	CadSNP2364	*Desi*	Ca_Desi_Chr03	16209498	(A/G)
26770	CadSNP2365	*Desi*	Ca_Desi_Chr03	16218534	(T/C)
26771	CadSNP2366	*Desi*	Ca_Desi_Chr03	16232554	(G/A)
26772	CadSNP2367	*Desi*	Ca_Desi_Chr03	16307552	(C/T)
26773	CadSNP2368	*Desi*	Ca_Desi_Chr03	16337251	(C/T)
26774	CadSNP2369	*Desi*	Ca_Desi_Chr03	16373373	(C/A)
26775	CadSNP2370	*Desi*	Ca_Desi_Chr03	16408351	(A/G)
26776	CadSNP2371	*Desi*	Ca_Desi_Chr03	16408411	(A/G)
26777	CadSNP2372	*Desi*	Ca_Desi_Chr03	16410820	(C/T)
26778	CadSNP2373	*Desi*	Ca_Desi_Chr03	16423933	(T/C)
26779	CadSNP2374	*Desi*	Ca_Desi_Chr03	16429296	(G/A)
26780	CadSNP2375	*Desi*	Ca_Desi_Chr03	16429290	(A/G)
26781	CadSNP2376	*Desi*	Ca_Desi_Chr03	16429260	(A/C)
26782	CadSNP2377	*Desi*	Ca_Desi_Chr03	16429257	(A/C)
26783	CadSNP2378	*Desi*	Ca_Desi_Chr03	16457210	(T/C)
26784	CadSNP2379	*Desi*	Ca_Desi_Chr03	16546902	(G/A)
26785	CadSNP2380	*Desi*	Ca_Desi_Chr03	16588500	(A/G)
26786	CadSNP2381	*Desi*	Ca_Desi_Chr03	16588533	(G/C)
26787	CadSNP2382	*Desi*	Ca_Desi_Chr03	16588555	(T/C)
26788	CadSNP2383	*Desi*	Ca_Desi_Chr03	16588661	(T/C)
26789	CadSNP2384	*Desi*	Ca_Desi_Chr03	16588648	(T/G)

S.N.	SNP IDs	Cultivars	Chromosomes/scaffolds	Physical positions (bp)	SNPs
26790	CadSNP2385	*Desi*	Ca_Desi_Chr03	16588642	(T/C)
26791	CadSNP2386	*Desi*	Ca_Desi_Chr03	16608969	(G/A)
26792	CadSNP2387	*Desi*	Ca_Desi_Chr03	16612390	(A/C)
26793	CadSNP2388	*Desi*	Ca_Desi_Chr03	16612523	(G/T)
26794	CadSNP2389	*Desi*	Ca_Desi_Chr03	16612522	(T/A)
26795	CadSNP2390	*Desi*	Ca_Desi_Chr03	16647962	(G/C)
26796	CadSNP2391	*Desi*	Ca_Desi_Chr03	16650388	(T/C)
26797	CadSNP2392	*Desi*	Ca_Desi_Chr03	16654383	(T/C)
26798	CadSNP2393	*Desi*	Ca_Desi_Chr03	16665981	(T/C)
26799	CadSNP2394	*Desi*	Ca_Desi_Chr03	16665928	(G/C)
26800	CadSNP2395	*Desi*	Ca_Desi_Chr03	16779590	(C/T)
26801	CadSNP2396	*Desi*	Ca_Desi_Chr03	16786527	(A/C)
26802	CadSNP2397	*Desi*	Ca_Desi_Chr03	16829269	(G/A)
26803	CadSNP2398	*Desi*	Ca_Desi_Chr03	16829284	(T/C)
26804	CadSNP2399	*Desi*	Ca_Desi_Chr03	16869021	(C/T)
26805	CadSNP2400	*Desi*	Ca_Desi_Chr03	16885534	(C/G)
26806	CadSNP2401	*Desi*	Ca_Desi_Chr03	16885685	(A/G)
26807	CadSNP2402	*Desi*	Ca_Desi_Chr03	16894386	(A/G)
26808	CadSNP2403	*Desi*	Ca_Desi_Chr03	16894455	(G/C)
26809	CadSNP2404	*Desi*	Ca_Desi_Chr03	16925661	(A/C)
26810	CadSNP2405	*Desi*	Ca_Desi_Chr03	16934270	(G/T)
26811	CadSNP2406	*Desi*	Ca_Desi_Chr03	16934316	(T/G)
26812	CadSNP2407	*Desi*	Ca_Desi_Chr03	16968147	(A/C)
26813	CadSNP2408	*Desi*	Ca_Desi_Chr03	16968093	(G/C)
26814	CadSNP2409	*Desi*	Ca_Desi_Chr03	17011107	(C/A)
26815	CadSNP2410	*Desi*	Ca_Desi_Chr03	17011108	(A/G)
26816	CadSNP2411	*Desi*	Ca_Desi_Chr03	17014193	(T/A)
26817	CadSNP2412	*Desi*	Ca_Desi_Chr03	17088351	(A/C)
26818	CadSNP2413	*Desi*	Ca_Desi_Chr03	17096881	(T/G)
26819	CadSNP2414	*Desi*	Ca_Desi_Chr03	17117877	(T/G)
26820	CadSNP2415	*Desi*	Ca_Desi_Chr03	17135055	(A/C)
26821	CadSNP2416	*Desi*	Ca_Desi_Chr03	17151326	(A/T)
26822	CadSNP2417	*Desi*	Ca_Desi_Chr03	17151439	(C/A)
26823	CadSNP2418	*Desi*	Ca_Desi_Chr03	17160362	(G/C)
26824	CadSNP2419	*Desi*	Ca_Desi_Chr03	17160394	(G/T)
26825	CadSNP2420	*Desi*	Ca_Desi_Chr03	17202494	(A/G)
26826	CadSNP2421	*Desi*	Ca_Desi_Chr03	17296670	(A/T)
26827	CadSNP2422	*Desi*	Ca_Desi_Chr03	17619289	(G/C)

S.N.	SNP IDs	Cultivars	Chromosomes/scaffolds	Physical positions (bp)	SNPs
26828	CadSNP2423	*Desi*	Ca_Desi_Chr03	17619292	(T/C)
26829	CadSNP2424	*Desi*	Ca_Desi_Chr03	17648928	(C/T)
26830	CadSNP2425	*Desi*	Ca_Desi_Chr03	17664224	(C/T)
26831	CadSNP2426	*Desi*	Ca_Desi_Chr03	17681284	(G/C)
26832	CadSNP2427	*Desi*	Ca_Desi_Chr03	17681331	(A/G)
26833	CadSNP2428	*Desi*	Ca_Desi_Chr03	17732900	(T/A)
26834	CadSNP2429	*Desi*	Ca_Desi_Chr03	17732871	(C/T)
26835	CadSNP2430	*Desi*	Ca_Desi_Chr03	17800447	(T/C)
26836	CadSNP2431	*Desi*	Ca_Desi_Chr03	17815074	(A/C)
26837	CadSNP2432	*Desi*	Ca_Desi_Chr03	17815081	(A/G)
26838	CadSNP2433	*Desi*	Ca_Desi_Chr03	17821525	(G/A)
26839	CadSNP2434	*Desi*	Ca_Desi_Chr03	17833336	(T/C)
26840	CadSNP2435	*Desi*	Ca_Desi_Chr03	17838091	(T/C)
26841	CadSNP2436	*Desi*	Ca_Desi_Chr03	17840051	(A/C)
26842	CadSNP2437	*Desi*	Ca_Desi_Chr03	17840040	(A/G)
26843	CadSNP2438	*Desi*	Ca_Desi_Chr03	17847712	(G/A)
26844	CadSNP2439	*Desi*	Ca_Desi_Chr03	17848961	(T/C)
26845	CadSNP2440	*Desi*	Ca_Desi_Chr03	17853430	(A/C)
26846	CadSNP2441	*Desi*	Ca_Desi_Chr03	17853418	(C/A)
26847	CadSNP2442	*Desi*	Ca_Desi_Chr03	17853412	(A/G)
[truncated: 549,151 more chars]
